# Duplex stem-loop-containing quadruplex motifs in the human genome: a combined genomic and structural study

**DOI:** 10.1093/nar/gkv355

**Published:** 2015-05-09

**Authors:** Kah Wai Lim, Piroon Jenjaroenpun, Zhen Jie Low, Zi Jian Khong, Yi Siang Ng, Vladimir Andreevich Kuznetsov, Anh Tuân Phan

**Affiliations:** 1 School of Physical and Mathematical Sciences, Nanyang Technological University, 637371, Singapore; 2 School of Biological Sciences, Nanyang Technological University, 637551, Singapore; 3 Department of Genome and Gene Expression Data Analysis, Bioinformatics Institute, 138671, Singapore

## Abstract

Duplex stem-loops and four-stranded G-quadruplexes have been implicated in (patho)biological processes. Overlap of stem-loop- and quadruplex-forming sequences could give rise to quadruplex–duplex hybrids (QDH), which combine features of both structural forms and could exhibit unique properties. Here, we present a combined genomic and structural study of stem-loop-containing quadruplex sequences (SLQS) in the human genome. Based on a maximum loop length of 20 nt, our survey identified 80 307 SLQS, embedded within 60 172 unique clusters. Our analysis suggested that these should cover close to half of total SLQS in the entire genome. Among these, 48 508 SLQS were strand-specifically located in genic/promoter regions, with the majority of genes displaying a low number of SLQS. Notably, genes containing abundant SLQS clusters were strongly associated with brain tissues. Enrichment analysis of SLQS-positive genes and mapping of SLQS onto transcriptional/mutagenesis hotspots and cancer-associated genes, provided a statistical framework supporting the biological involvements of SLQS. *In* *vitro* formation of diverse QDH by selective SLQS hits were successfully verified by nuclear magnetic resonance spectroscopy. Folding topologies of two SLQS were elucidated in detail. We also demonstrated that sequence changes at mutation/single-nucleotide polymorphism loci could affect the structural conformations adopted by SLQS. Thus, our predicted SLQS offer novel insights into the potential involvement of QDH in diverse (patho)biological processes and could represent novel regulatory signals.

## INTRODUCTION

DNA can adopt many non-canonical structural conformations, some of which have been shown to take part in normal cellular as well as pathobiological processes (1,2). One example is the G-quadruplex (G4) (3–5), a four-stranded helical complex built from the stacking of multiple G•G•G•G tetrads (6). G4 have been implicated in cellular processes (7) including recombination (8) and replication (9–12), and their formations were detected in ciliates (13) and human cells (14). There has been considerable interest in the development of chemical ligands specifically targeting these structures as an anticancer strategy (5,15), owing to the enrichment of G-rich sequences at the telomeres (16) and oncogenic promoters (17,18). For instance, targeting of genomic G4 was demonstrated against a G-rich fragment within nuclease hypersensitivity element III_1_ (NHE III_1_) of the c-*MYC* promoter by the porphyrin TMPyP4 (19), which led to down-regulation of c-*MYC* transcription. G4-forming sequences were also identified in other promoters including c-*KIT* (20,21), *KRAS* (22), *BCL2* (23), *RET* (24) and *hTERT* (25–28), prompting the view that these motifs could be involved in gene regulation at the transcriptional level (17,18).

**Figure 1.**
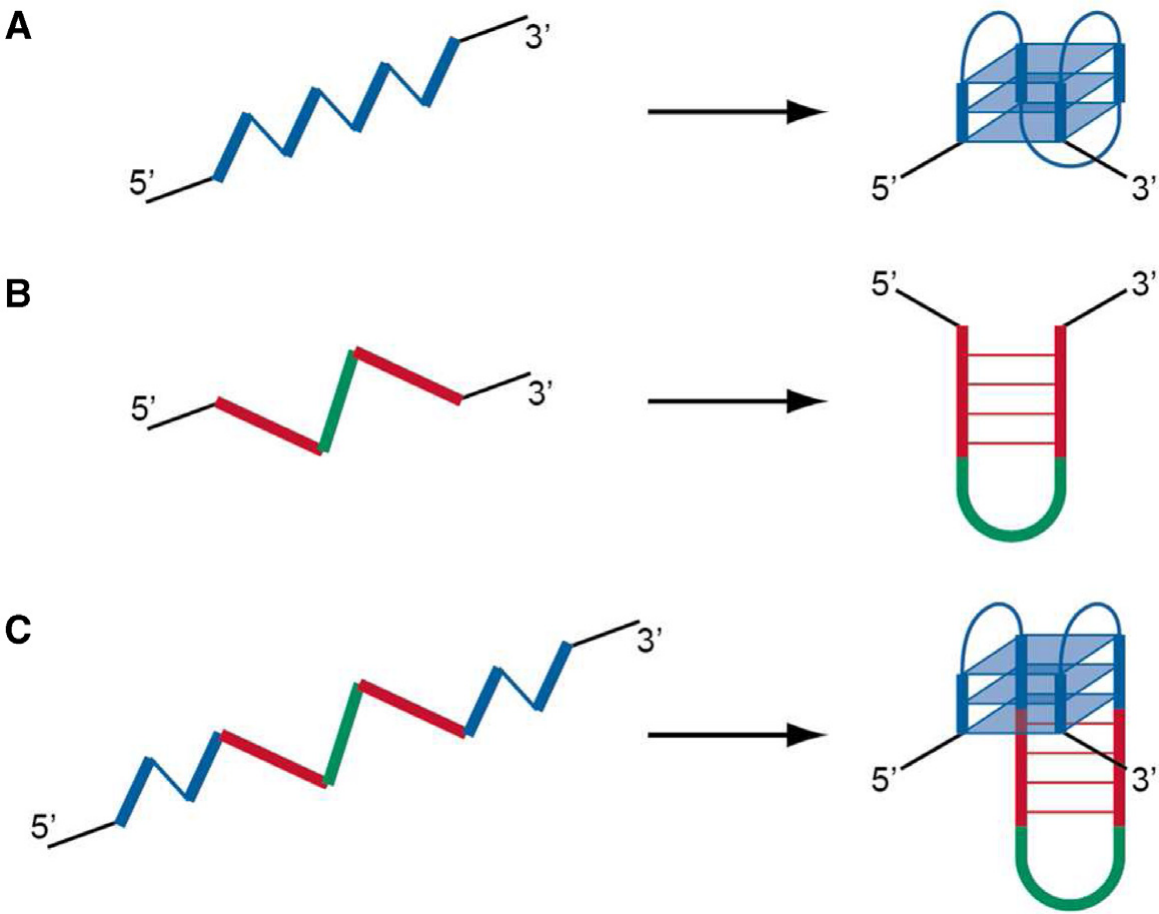
Formation of quadruplex, duplex stem-loop and stem-loop-containing quadruplex. Schematic diagrams illustrating the formation from a single DNA strand of (**A**) a G-quadruplex, (**B**) a duplex stem-loop and (**C**) a G-quadruplex containing a duplex stem-loop. G-tracts (thick lines) and quadruplex loops (thin lines) are coloured in blue, self-complementary tracts are coloured in red, while hairpin loops are coloured in green.

The G4 could exist in a diverse range of folding topologies, brought about by the relative orientations of the four strands constituting the core and the manner in which they are connected by linkers (known as loops). A typical intramolecular G4-forming sequence would consist of four G-tracts interspersed with three loops (Figure 1a), while exceptions have also been observed (21,29). Thermodynamic studies have concurred on the notion that shorter loops lead to more stable G4 structures (30–34). Based on these observations, various algorithms have been implemented in the identification of putative quadruplex sequences (PQS) in the human genome (35–40), mostly variants of the generic expression G_X1_N_L1_G_X2_N_L2_G_X3_N_L3_G_X4_, in which the G-tract (*X*) and loop length (*L*) were largely restricted to 2–5 and 7 nucleotides (nt), respectively. For instance, the *quadparser* algorithm (*X* ≥ 3, *L* = 1 – 7) identified >350 000 PQS in the human genome (36). On the other hand, G4 structures incorporating longer loops (*L* > 7) have also been investigated (41–43) and in these cases interactions involving the long loops could contribute towards stabilization of the structures.

Another non-canonical DNA structural conformation that has been extensively studied is the duplex stem-loop (SL) (or hairpin) motif (Figure 1b). These motifs are intricately involved in nucleic acid secondary structure formations and they were shown to be the major contributing factor of mutagenesis in certain diseases (1,2). Interspersing duplex SL motifs within quadruplex-forming motifs could lead to the generation of intramolecular quadruplex–duplex hybrids (QDH) (Figure 1c), which combine the structural traits of both conformations. The facile formation of such hybrid structures have been demonstrated (44) and they were shown to exhibit excellent stability (45,46). We have previously shown that such structures can arise in a diverse arrays of arrangement between the duplex and quadruplex segments (44). In addition, effects of various modifications at the quadruplex–duplex junction on the stability of these QDH structures revealed important considerations for the prediction of such motifs (46). In these structures, the SL motif plays a guiding role to bring remote G-tracts close together for the establishment of a G4 and simultaneously restricts the folding topology that can be adopted by the G4. The existence of sequence motifs with the potential to form intramolecular QDH in the human genome, which would necessitate a consideration of longer loops (*L* > 7) for PQS, could reveal as yet unknown biology of these motifs.

Here we developed a prediction model for the family of QDH-forming sequences, provided mapping of such QDH sequences onto the human genome and verified this model using nuclear magnetic resonance (NMR) spectroscopy. We performed a bioinformatics search to identify SL-containing quadruplex sequences (SLQS) in the human genome, a substantial number of which were found to reside within regulatory meaningful loci. Enrichment analysis revealed that SLQS display strand specificity and are preferentially distributed within specific genic and gene promoter regions, RNA polymerase II (Pol2) binding sites and other transcriptional regulatory DNA sites. In addition, these SLQS are enriched within specific functional classes of genes, and they occur at especially high frequencies in many hundreds of brain tissue-related and cancer-associated genes. NMR characterization on a selected list of human genome SLQS hits validated their adoption of diverse QDH-forming structures, with the folding topology of two particular sequences being elucidated in detail. We also explored the effects of sequence changes, which could arise at mutation/single-nucleotide polymorphism (SNP) loci, on the structural conformations adopted by these SLQS.

## MATERIALS AND METHODS

### Bioinformatics datasets

The following datasets associated with build *hg18* of the human genome were downloaded from the UCSC Genome Browser (47): the human genome sequence, RefGene annotation, chromatin accessibility regions (ENCODE Digital DNaseI Hypersensitivity Clusters), transcription factor binding sites (ENCODE Transcription Factor ChIP-seq) and RNA polymerase II binding sites (ENCODE Transcription Factor Binding Sites by ChIP-seq from Yale/UC-Davis/Harvard). Mutation data of high-grade serous ovarian carcinoma (HG-SOC) were acquired from The Cancer Genome Atlas (TCGA) Research Network (48). SNP data were obtained from dbSNP (49), of which a subset of sequence-validated SNPs (1000 Genomes) with minor allele frequency of >5% were used.

### Search algorithms

In-house Python scripts were developed to search for PQS_L20_, PQS_L7_ and SLQS from genomic data in the FASTA format. First, PQS_L20_ were identified based on the algorithm G_3–6_N_1–20_G_3–6_N_1–20_G_3–6_N_1–20_G_3–6_, in which G_3–6_ represents a G-tract comprising 3–6 successive guanines and N_1–20_ represents a loop comprising 1–20 nt (inclusive of guanines). PQS_L20_ for which all three loops are shorter or equal to 7 nt were sub-classified as PQS_L7_. For the remaining PQS_L20_, the long loops (*L* > 7) were extracted and screened for the existence of duplex stem-loop elements. During the extraction step, the loop sequences were extended both ways to include guanines in excess of three from the two flanking G-tracts (e.g. 2 G's will be added to the loop sequence from a flanking G-tract of G_5_). As such, the extracted loop sequences would range from 8 to 26 nt. Only for cases in which all of these long loops could form stable stem-loops (as determined using hybrid-ss-min module, applying default parameters, of the UNAFold package (50), version 3.8) with a base pair composition of ≥50% (with respect to the loop length) were the sequence classified as an SLQS.

### Analysis of the skewed frequency distribution of SLQS

The Kolmogorov–Waring (K–W) probability function was used to characterize and fit the distribution of the number of SLQS in a given gene of human genome (51,52). The function is described as:

(1) \begin{equation*} P(X = m) = p_m = p_0 \frac{{B(b + 1,m)}}{{B(a,m)}}\theta ^m , \end{equation*}

where *m* = 0, 1, 2, … and *b*, *a* and *θ* are the parameters of our model. $B(x)$ is the Beta function as previously described (51,52). In the case where *b* > *a* > 0, the probability of non-observed events is estimated by the formula $\mathop p\limits^{} _0 = (1 - \frac{a}{b})$. Equation (1) can be presented in the form of the following recursive formula for easy computational estimate of the model parameters:

(2) \begin{equation*} p_{m + 1} = \theta \frac{{(a + m)}}{{b + m + 1}}p_m \end{equation*}

In order to apply the probability function Equations (1) or (2) to the observed data, we assumed that the random variable *X* is restricted to sample size and the rarest events are non-observed. Thus, random variable *X* is doubly truncated, i.e. the range 1, 2, …, *J* ($J < \infty$). Using Equation (1), the probability distribution function of the resulting truncated distribution function is written as the following:

(3) \begin{equation*} p_m^T = p_m /(\sum\limits_{s = 1}^{s = J} {p_s )} = \frac{{p_m }}{{1 - p_0 - P_{J + 1} }} \end{equation*}

where $P_{J + 1} = \sum\nolimits_{s = J + 1}^\infty {p_s }$.

This probability distribution function corresponds to a typical situation in analysis of data having levels, *J*, where the occurrence values 0 and *J + 1*, *J + 2*, … are not detected. Details of the curve-fitting computational algorithm have been previously published (51).

### Gene ontology (GO) analysis

Gene ontology (GO) analysis was carried out separately using two systems. First, the database for annotation, visualization and integrated discovery (DAVID) bioinformatics resources (53) (version 6.7) was used to identify gene functional annotation terms that are significantly enriched in protein-coding genes encompassing at least one copy of SLQS. The list of gene symbols was supplied as the input and the output consists of a modified Fishers Exact *P*-value for each annotation term, which gives a measure of the enrichment within respective tissue categories. The annotation sets UniProt (UP_TISSUE) and CGAP_SAGE_QUARTILE were utilized. The false discovery rate (FDR) method is the default *P*-value adjustment method in this study. In this method, the *P*-values are first sorted and ranked. The smallest value gets rank 1, the second rank 2 and the largest gets rank N. Then, each *P*-value is multiplied by N and divided by its assigned rank to give the adjusted *P*-values.

Second, the MetaCore™ (GeneGo, St Joseph, MI) software was used to calculate the biological process enrichment statistics for genes encompassing SLQS in different gene segments, namely transcriptional hotspots and 5′-UTR. The list of genes (in terms of RefSeq IDs) was supplied as the input, and the output consists of a *P*-value for each GeneGo process term. A *P*-value (after Bonferroni correction) cut-off of 1 × 10^−5^ was exercised.

### DNA sample preparation

Unlabelled and site-specific labelled DNA oligonucleotides were chemically synthesized on an ABI 394 DNA/RNA synthesizer. The oligonucleotides were de-protected, purified, dialyzed successively against ∼20 mM KCl and against water, and prepared in a buffer containing 20 mM KCl and 20 mM potassium phosphate (pH 7.0).

### NMR spectroscopy

NMR experiments were performed on Bruker 600 and 700 MHz spectrometers at 25°C, unless otherwise specified. Resonances for guanine residues were assigned unambiguously by using site-specific low-enrichment ^15^N-labelling (54) and site-specific ^2^H labelling (55). Spectral assignments were assisted by NOESY, COSY, TOCSY and ^13^C-^1^H-HSQC, as previously described (56,57). All spectral analyses were performed using the program FELIX (Felix NMR, Inc.).

### Circular dichroism

Circular dichroism (CD) spectra were recorded on a JASCO-815 spectropolarimeter over the range of 220–320 nm using a 1-cm path length quartz cuvette with a reaction volume of 500 μl. For CD-melting experiments, cooling and heating were successively performed across the temperature range of 15–95°C over a total of 14 h. At intervals of 1°C, the full spectrum was recorded as an average of three scans, the spectrum of the buffer was subtracted and the data were zero-corrected at 320 nm. The molar ellipticity at 295 nm was extracted for melting analysis. Two baselines corresponding to the completely folded (low temperatures) and completely unfolded (high temperatures) states were manually drawn in order to determine the fractions of folded and unfolded species during the melting process. CD-melting experiments for G4ST02001786748 were carried out at strand concentrations of 2, 4, 20 and 100 μM.

## RESULTS

### *In silico* identification of stem-loop-containing quadruplex sequences (SLQS) in the human genome

A previous survey of PQS (based on the definition G_X ≥ 3_N_L = 1 – 7_G_X ≥ 3_N_L = 1 – 7_G_X ≥ 3_N_L = 1 – 7_G_X ≥ 3_) in the human genome revealed more than 350 000 candidates (36). It has further been shown that these motifs are highly overrepresented in the region immediately upstream of transcription start sites (18) (TSS) and are strongly associated with specific functional classes of genes (17). These observations corroborated the proposition of a regulatory role of PQS in gene transcription.

**Figure 2.**
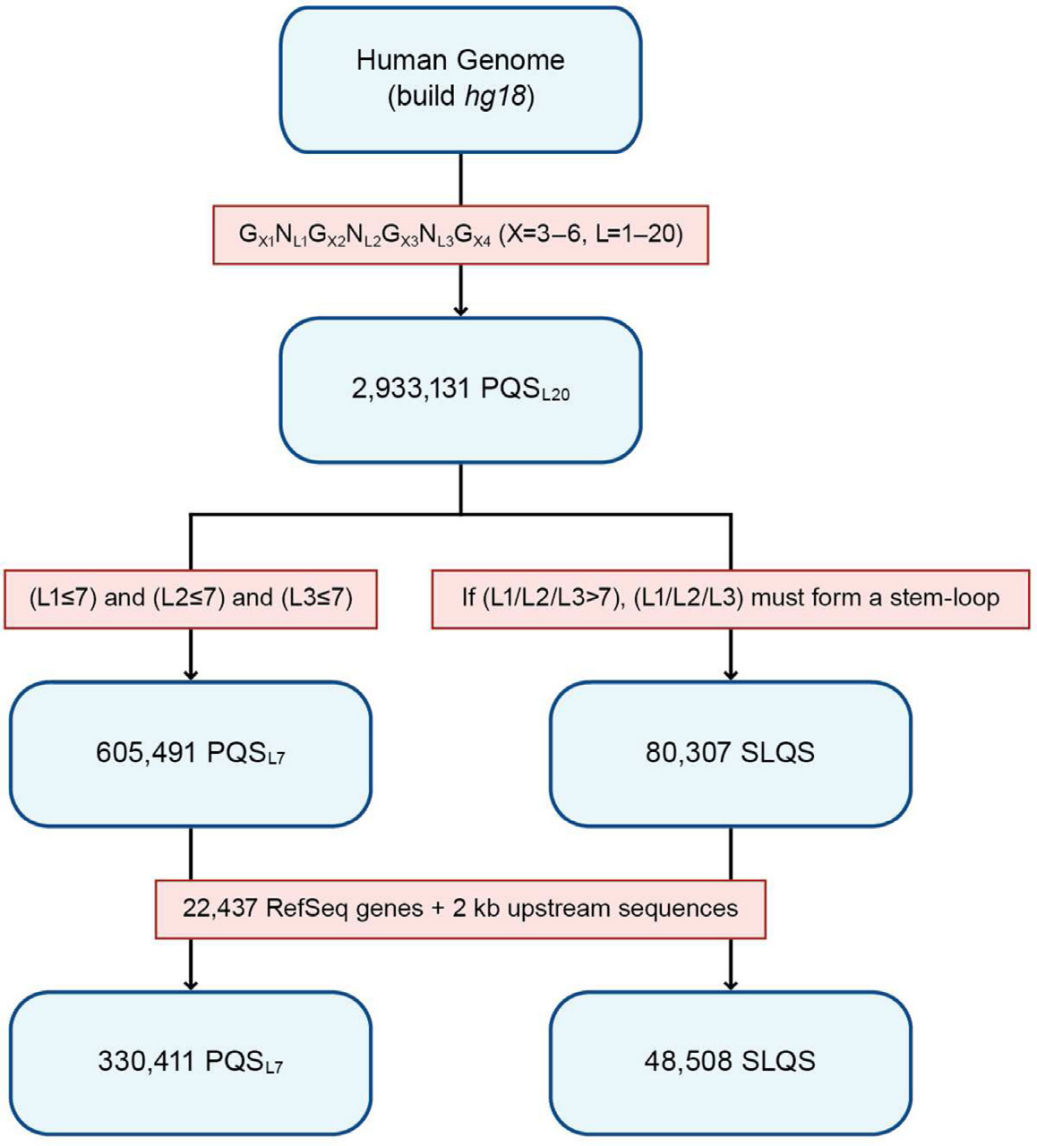
Flow chart of the search protocols used for the derivation of putative quadruplex sequences (PQS) and stem-loop-containing quadruplex sequences (SLQS) in the human genome. Sequence datasets are coloured in blue while search algorithms/selection criteria are coloured in red. Note that within each category, the sequence hits were permitted to overlap, wherein any two of them may share up to three identical G-tracts.

Here we sought to identify SLQS in the human genome, with a particular focus on genic (22 437 genes in the human RefSeq database (58)) and gene promoter (defined as the region ≤2 kb upstream of TSS) regions. First, the PQS parameters were altered (*X* = 3 – 6, *L* = 1 – 20; denoted as PQS_L20_) to accommodate longer loops that might harbour duplex SL elements. Based on this model, a total of 2 933 131 PQS_L20_ were identified (Figure 2). Note that these sequence hits were permitted to overlap, i.e. two PQS_L20_ can share up to three identical G-tracts. PQS_L20_ for which *L* ≤ 7 across all three loops were sub-classified as PQS_L7_, which would have the same loop length parameter as the *quadparser* algorithm (36). For all other PQS_L20_, in which any of L1, L2 or L3 is >7 nt in length, the long loops were subsequently screened for the existence of duplex SL elements using the UNAFold package (50) (see ‘Materials and Methods’ for details). For cases in which these long loops could form stable SL with a base pair composition of ≥50% (with respect to the loop length), the sequence was classified as a SLQS. Work flow of the screening process for PQS_L20_, PQS_L7_ and SLQS is outlined in Figure 2.

Based on our selection criteria, 605 491 PQS_L7_ (out of 360 438 unique clusters) and 80 307 SLQS (out of 60 172 unique clusters) were identified in the human genome (build *hg18*). The complete list of SLQS hits (each tagged with a unique SLQS ID), accompanied with gene annotations, is presented in Supplementary Dataset 1. Among these, 48 508 SLQS (out of 35 469 unique clusters) are spread across 12 315 RefSeq genes and/or their gene promoter regions (Table 1). A predominant number of these genic SLQS (92.8% or 45 029 out of 48 508 SLQS) comprise a single SL in the predicted structures, while a minority of these comprise two (6.7% or 3228 out of 48 508 SLQS) or three (0.5% or 251 out of 48 508) SL in the predicted structures.

**Table 1.** Distribution of PQS_L7_ and SLQS across various gene segments

	PQS_L7_			SLQS			Regulatory SLQS^a^		
Gene segment	Non-template	Template	Symbols^b^	Non-template	Template	Symbols^b^	Non-template	Template	Symbols^b^
Genic or Promoter^c^	166 964	173 924	19 099	27 355	23 116	12 315	7473	5963	6171
Genic	146 529	152 560	17 786	23 957	19 407	10 903	5578	3811	4648
Promoter^c^	25 781	25 924	11 801	4507	4487	4591	2568	2595	2866
Exon	12 276	18 857	9408	3090	4356	4141	1252	1320	1681
CDS^d^	2731	7026	4026	1061	2091	1936	286	404	510
Intron	136 399	136 129	16 638	21 589	15 805	9667	4589	2689	3675
5′-UTR^e^	3965	5249	4045	1226	1206	1529	814	740	1019
3′-UTR^f^	4770	5811	4065	672	913	1046	113	129	178

^a^SLQS mapped to gene regulatory loci (chromatin accessibility regions and transcription factor binding sites).

^b^Number of gene symbols for which the respective PQS_L7_/SLQS have been located within.

^c^2 kb upstream region of TSS.

^d^Coding DNA sequence.

^e^5′-untranslated region.

^f^3′-untranslated region.

**Figure 3.**
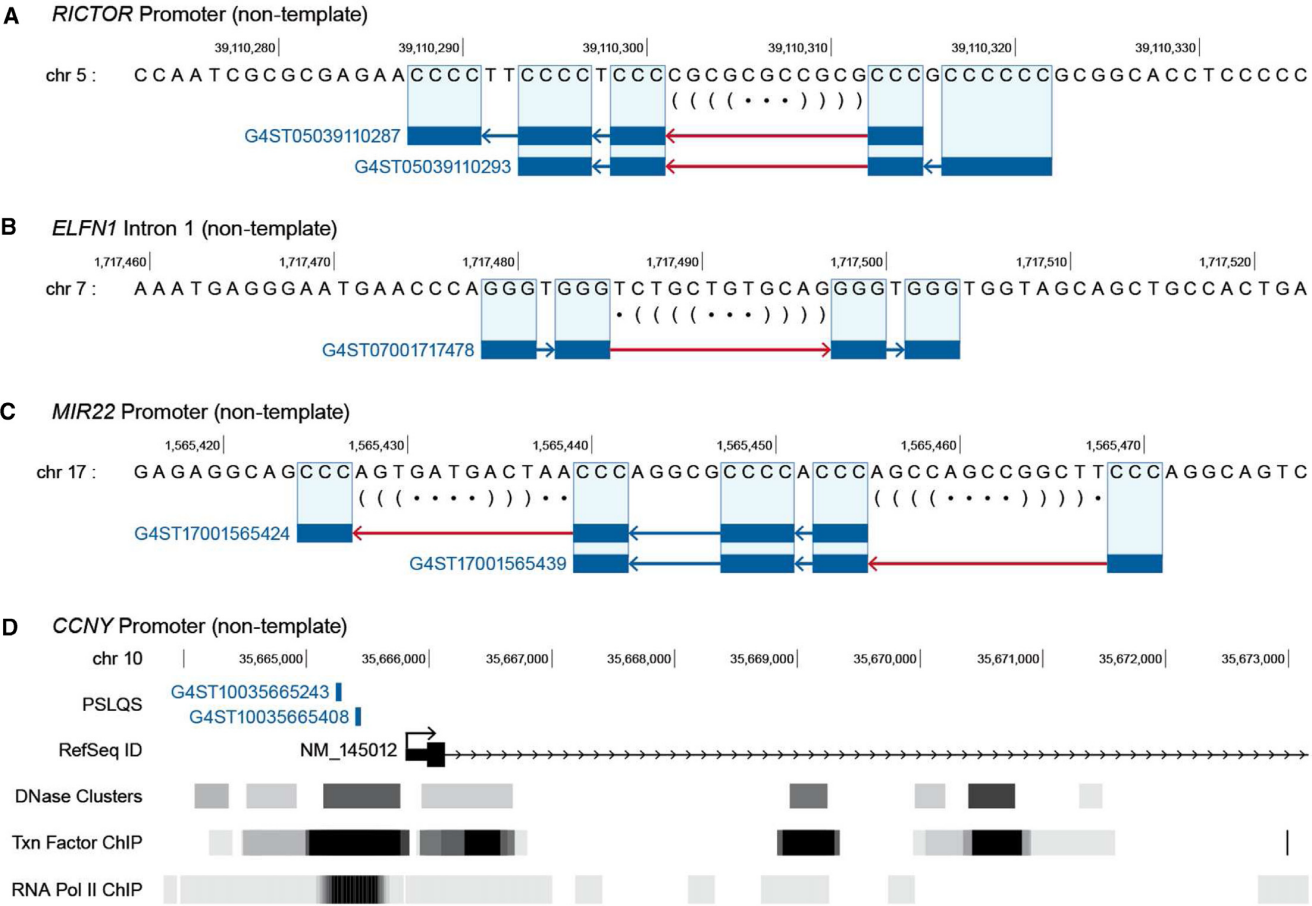
Localization of SLQS in genic and gene promoter regions of the human genome. (**A**–**C**) Examples of SLQS from *RICTOR* promoter (a), *ELFN1* Intron 1 (b) and *MIR22* promoter (c). In each panel, the strand placement, ID, and mapping (on a single-nucleotide scale) of the respective SLQS are displayed. G-tracts are shown as blue rectangles, short loops (≤7 nt) are shown as blue lines, while long loops capable of forming stable duplex stem-loops are shown as red lines. The predicted duplex stem-loops are outlined in dot-bracket notation; nucleotides involved in base pair formation are nested by matching pairs of brackets, whereas nucleotides not involved in base pair formation are marked by a dot. Only the reference genome set (build *hg18*) is shown, with SLQS located on the complementary strand (corresponding to C-tracts in the reference genome) running in the reverse direction. (**D**) Genome architecture in the vicinity of the transcription start sites (TSS) for *CCNY* gene (transcript NM_145012), which are mapped against open chromatin regions (DNase Clusters; ENCODE Digital DNaseI Hypersensitivity Clusters), transcription factor binding sites (Txn Factor ChIP; ENCODE Transcription Factor ChIP-seq) and RNA polymerase II binding sites (RNA Pol II ChIP; ENCODE Transcription Factor Binding Sites by ChIP-seq from Yale/UC-Davis/Harvard). Two SLQS motifs are located in the promoter region.

Examples of SLQS from the proximal promoter of the protein-coding gene *RICTOR* and the micro-RNA gene *MIR22*, and from the intron of gene *ELFN1*, are shown in Figure 3a–c on a single-nucleotide scale. In Figure 3d, genome architecture in the vicinity of the TSS for the *CCNY* gene is overlaid against open chromatin regions, transcription factor binding sites, and Pol2 binding sites. Co-localization of two SLQS within such transcriptional hotspots could give rise to the occurrence of single-stranded regions forming DNA secondary structures, and suggested the involvement of SLQS in Pol2-mediated transcriptional regulation.

Importantly, out of these 48 508 SLQS, 33 148 do not overlap with PQS_L7_ (i.e. they do not share any mutual G-tracts). Thus, these 33 148 SLQS would represent a novel set of potential regulatory genomic signals, distinct from the canonical PQS that have previously been reported in the literature (35,36). We note that 397 SLQS were spread across 285 genes for which PQS_L7_ were otherwise not found (gene list presented in Supplementary Dataset 2). On the other hand, SLQS that coincide with PQS_L7_ would extend the range of the putative quadruplex-forming regions, as in the case of the *RET* gene promoter (Supplementary Figure S1), which has been previously characterized (24).

**Figure 4.**
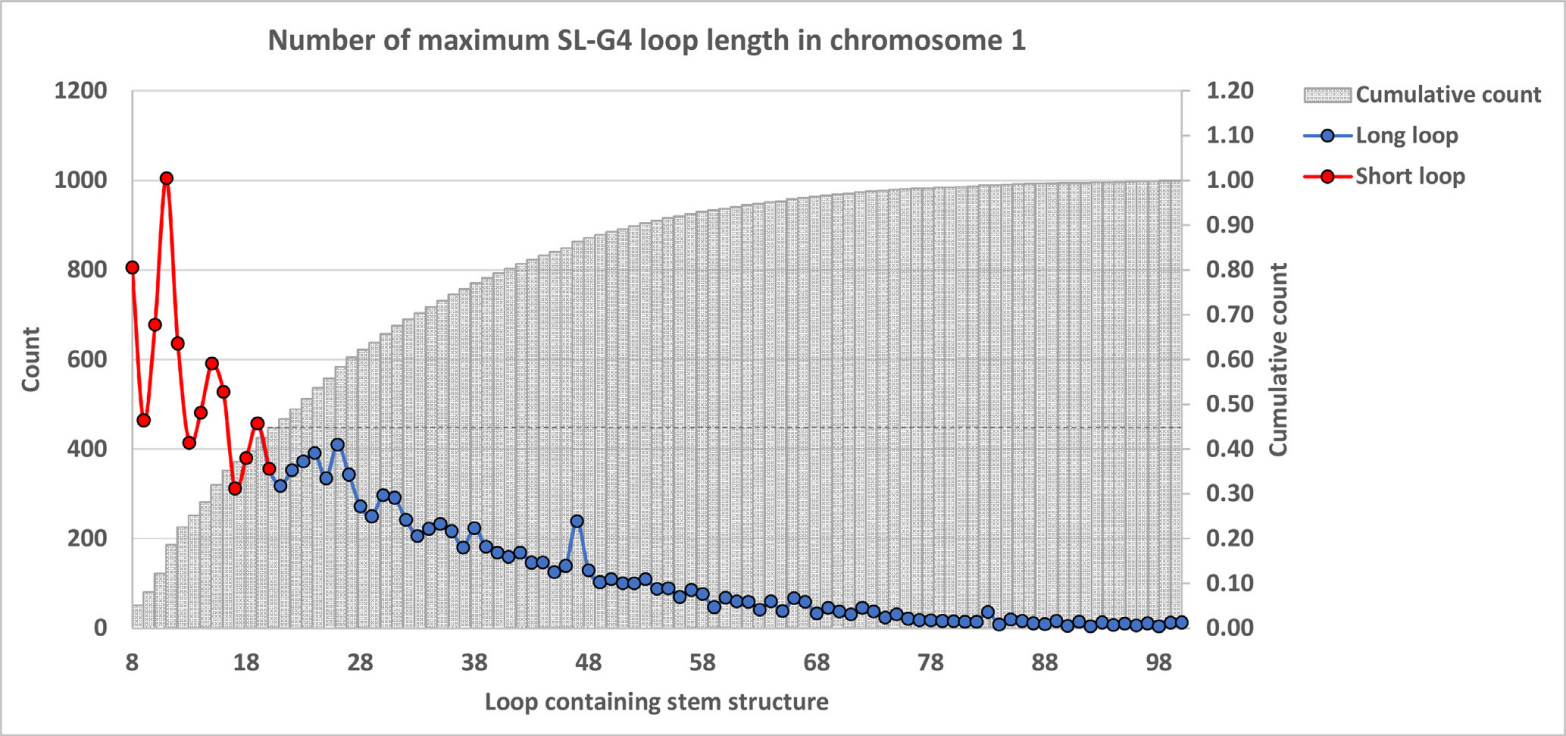
Distribution of SLQS in human Chromosome 1, according to the lengths of the stem-loops. Data in red represent counts of SLQS comprising short stem-loops (8–20 nt) while data in blue represent counts of SLQS comprising longer stem-loops (21–100 nt). Grey bars represent the cumulative frequency function of predicted SLQS in the given loop length interval (from 8–100 nt).

To probe the predominance of SLQS containing even longer SL (>20 nt in length), we examined the distribution of SLQS with SL length ranging from 8–100 nt across Chromosome 1 (which covers ∼15% of the entire human genome) as a representative sample (Figure 4). In this case, the number of SLQS generally decreased with increasing SL length. The cumulative curve showed that SLQS comprising SL length of 8–20 nt (our model) constitute ∼45% of the total SLQS population from Chromosome 1. Extrapolating from here, we expect that SLQS comprising SL length of 8–20 nt would provide a meaningful coverage of these motifs for the immediate study on the physical and biological features of potential QDH structures with short to medium SL in the human genome.

### Non-random distribution of SLQS in genic, gene promoter and Pol2 regions suggest a regulatory role for SLQS

**Figure 5.**
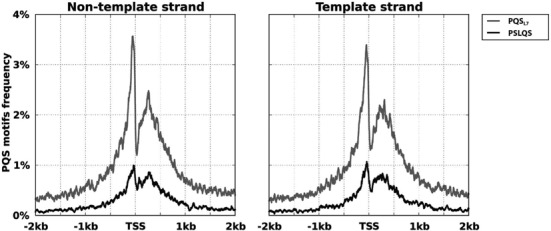
Distribution of putative quadruplex sequences (PQS) and SLQS in the vicinity of TSS of protein-coding genes. The frequencies of occurrence of PQS_L7_ and SLQS at each individual base position, normalized for the occurrences of the motifs across all positions, are plotted over the region 2 kb upstream/downstream of the TSS. The frequency plots of PQS_L7_ and SLQS on the non-template and template strands are shown on the left and right panels, respectively. TSS data were obtained from all protein-coding RefSeq transcripts using UCSC Genome Browser.

A detailed breakdown on the distribution of SLQS across different gene segments (as referenced against the RefGene annotation, UCSC Genome Browser (47)) in comparison with PQS_L7_ is presented in Table 1. SLQS showed a bi-modal enrichment pattern around the TSS (±500 nt) similar to that of PQS_L7_ (Figure 5), suggesting that SLQS could also be structurally and functionally important and exert an influence on transcriptional regulation.

Genes for which Pol2 binding regions are highly enriched with SLQS could exert a regulatory role. We found 13 914 SLQS located in the vicinity of Pol2 binding sites (at least 1-nt overlap between SLQS and Pol2 binding sites) from 6367 genes. Most of these genes were associated with one or two SLQS. A total of 297 genes were associated with more than 6 SLQS across their genic and/or gene promoter regions (Supplementary Dataset 3). Gene enrichment analysis revealed specific functional enrichment of these genes (Supplementary Dataset 4), e.g. ‘splice variants’ (*P* = 0.00021), ‘alternative splicing’ (*P* = 0.00018), ‘phosphoproteins’ (*P* = 3.7 × 10^−16^), ‘mutagenesis sites’ (*P* = 0.00016), ‘repressor’ (*P* = 0.00005), ‘activator’ (*P* = 0.000028), ‘proto-oncogene’ (*P* = 0.000054), ‘regulation of transcription from RNA polymerase II promoter’ (*P* = 1.85 × 10^−7^) and ‘transcription regulation’ (*p* < 2.86 × 10^−7^). In these cases, the adjusted *P*-values after Benjamini-Hochberg correction were used (see ‘Materials and Methods’ section). Genes with the highest number of SLQS (14–32 per gene) in Pol2 binding sites include *RNF213, BCOR, RNPC3, FAM38A, C14orf43, PIK3CD, KLF13, AHRR, ZMIZ1, UBE2E1, TP73, PREX1, MKNK2*.

### A probabilistic model for the distribution of SLQS count per gene

**Figure 6.**
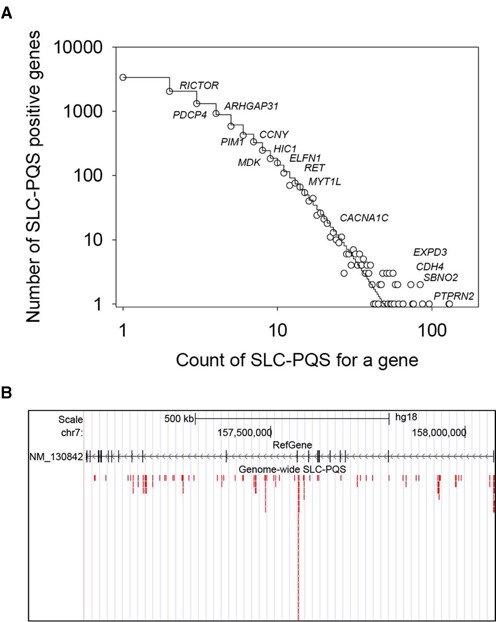
Distribution of SLQS at the population and single gene levels. (**A**) Distribution for the number of SLQS within protein-coding genes, according to the count of SLQS per gene. Selective gene symbols are marked at the positions corresponding to their respective count of SLQS. The skewed step function is the best-fitted Kolmogorov-Waring probability distribution function with parameters *θ* = 0.993183, *a* = 5.31136 and *b* = 8.27518. (**B**) Distribution of individual SLQS within the locus of *PTPRN2* gene.

A log–log plot of the distribution of SLQS (with respect to the count of SLQS per gene) for a protein-coding gene is presented in Figure 6a. The long right tail of the distribution is characteristic of observations for which few genes are highly populated whereas many other genes are less populated with SLQS. Out of 10 126 SLQS-containing protein-coding genes (based on gene classification in RefSeq database (58)), the vast majority encompass a single SLQS or a couple of SLQS. At the other extreme, a handful of genes exhibit an especially high count (>60) of SLQS (Supplementary Table S1), for instance *PTPRN2* (Figure 6b), *SBNO2*, *CDH4* and *EXD3*. The distribution of SLQS count in a given protein-coding gene locus can be approximated based on the K–W function (51,52) with best-fit parameters *θ* = 0.993183, *a* = 5.31136 and *b* = 8.27518. Such a function belongs to a family of skewed probability distributions, which were observed in many evolving and interactive (interconnecting) systems wherein the species birth-death processes are occurring and driving a system by evolution towards the complexity and self-organization function (51,52) (see ‘Materials and Methods’ section for detail). In this context, the Waring probabilistic function (a special case of K–W family at *θ* = 1) may help us better understand the roles of common and rare SLQS in gene functions and the origin of G-quadruplexes, stem-loop-forming sequences, and QDH, and their natural co-evolution in the genome.

### Gene ontology analysis of genes encompassing SLQS

To investigate if there is any evolutionary selection pressure for SLQS across specific biological classes of genes, GO analysis was carried out for the group of protein-coding genes encompassing at least one SLQS motif, using the database for annotation, visualization and integrated discovery (DAVID) bioinformatics resources (53) with all annotated genes as the background set. Strong tissue specificity was observed for these genes, specifically with regards to the brain and epithelium, as well as several cancer types and mental disorders (Supplementary Table S2). Among the 10 126 SLQS-containing protein-coding genes, 4541 are associated with the brain tissue (classified under the term ‘brain’ within UP_TISSUE annotation set of DAVID bioinformatics resources). This enrichment becomes more pronounced for the group of genes with high frequencies (>5) of SLQS (Supplementary Table S3). Similar enrichment of SLQS motifs across brain tissue related genes were observed when the GO analysis was performed at a transcriptome level (based on CGAP_SAGE_QUARTILE annotation set of DAVID bioinformatics resources) (Supplementary Table S4). In this case, more diverse and specific gene products were found including types of brain tissues, several cancer types and cartilage. GO analysis was repeated using PQS_L7_- and SLQS-containing genes as the background set (Supplementary Table S5), showing that *P*-values (with Benjamini correction) of GO terms using all human genes as the background set were more significant.

**Figure 7.**
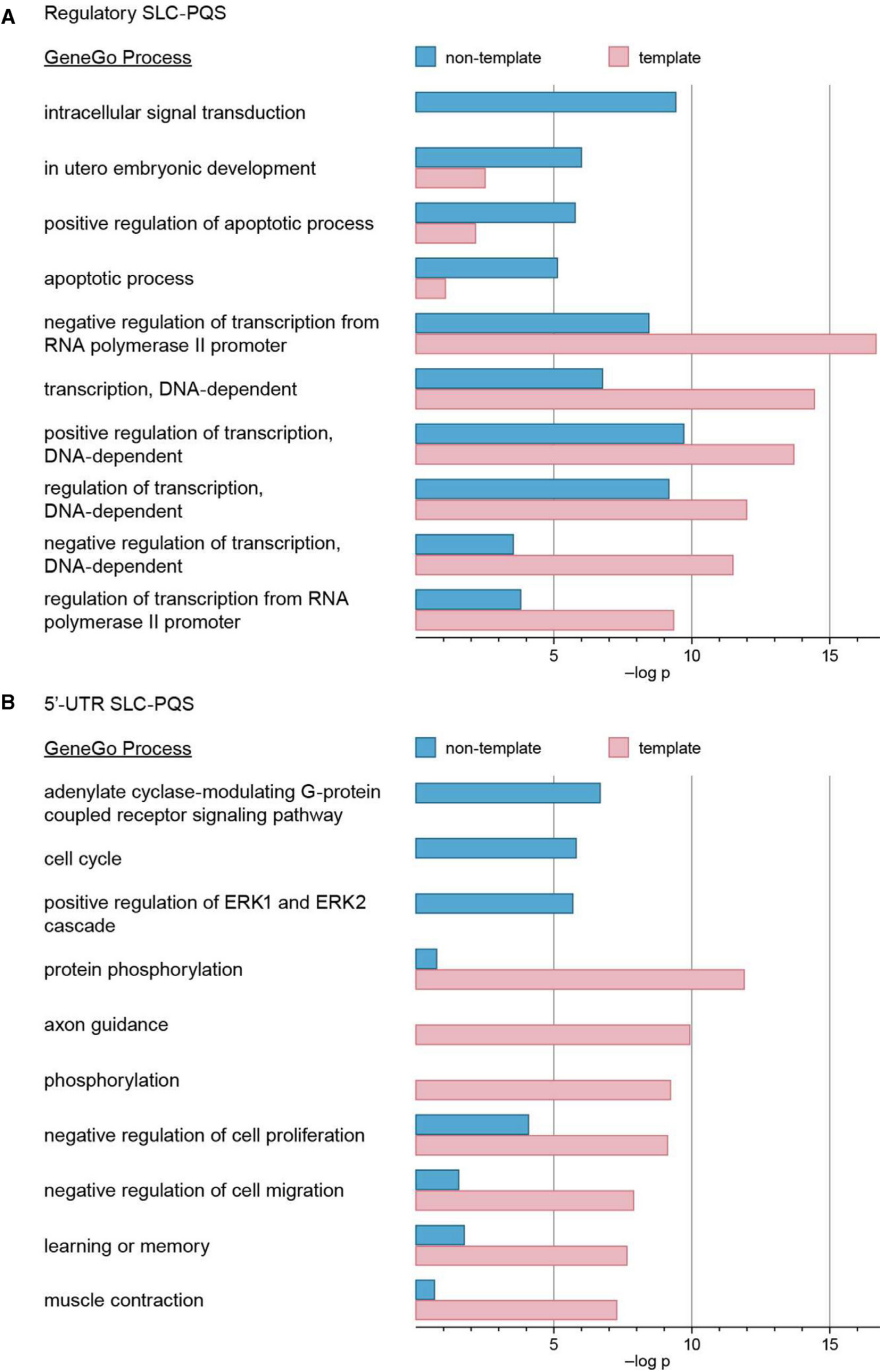
Gene ontology (GO) analysis of SLQS from different gene segments. Examples of GeneGo process categories (MetaCore^TM^) which are found to be significantly enriched with (**A**) promoter SLQS located at gene regulatory loci and (**B**) SLQS located at 5′-UTR, on either the template (pink) and/or non-template (blue) strands. The *P*-values are presented as negative logarithms.

Next, to explore the transcriptional regulatory potential of SLQS across different functional classes of genes, GO analysis was performed on 2866 gene symbols (represented by 4366 RefSeq IDs) for which SLQS located in promoter regions have been found to coincide with gene regulatory loci (defined as chromatin accessibility regions and transcription factor binding sites; Table 1), using the GeneGo MetaCore™ software. 19 GeneGo process categories were found to be enriched with regulatory SLQS (*P*-value < 1 × 10^−5^, after Bonferroni correction; Supplementary Table S6), ranging from ‘regulation of transcription’, ‘negative regulation of cell proliferation’ and ‘positive regulation of apoptotic process’, to ‘axon guidance’, ‘protein phosphorylation’ and ‘heart development’. These process categories overlap considerably with the GO categories previously reported for promoter PQS (18). The same GO analysis was performed on a control set of five replicas (each comprising 4366 RefSeq IDs) of randomly sampled genes from the pool of genes encompassing regulatory PQS_L7_, and showed that 18 of these GeneGo process categories were also enriched (*P* < 1 × 10^−5^, after Bonferroni correction, in at least one of the five replicas) with regulatory PQS_L7_, with ‘learning or memory’ being the sole exception (Supplementary Table S6). Interestingly, we note that the latter constitutes part of a larger group of genes related to brain tissue that corresponds to the long right tail in Figure 6a. GO analyses carried out separately for genes encompassing regulatory SLQS located either on the template or non-template strands suggested that SLQS found on different strands could regulate distinct functional classes of genes (Figure 7a and Supplementary Table S7). For instance, the GeneGo process categories ‘intracellular signal transduction’, ‘*in* *utero* embryonic development’ and ‘apoptotic process’ are enriched with regulatory SLQS located on the non-template strand, whereas the GeneGo process categories ‘regulation of transcription’, ‘nervous system development’ and ‘protein phosphorylation’ are enriched with regulatory SLQS located on the template strand.

The same procedures were repeated on 1529 gene symbols (represented by 2275 RefSeq IDs) for which SLQS have been localized to 5′-untranslated region (5′-UTR) (Table 1). 23 GeneGo process categories were found to be enriched with 5′-UTR SLQS (*P* < 1 × 10^−5^, after Bonferroni correction; Supplementary Table S8). Compared against the control (five replicas, each comprising 2275 RefSeq IDs), 10 of these GeneGo process categories were enriched with 5′-UTR SLQS but not 5′-UTR PQS_L7_ (Supplementary Table S8), including ‘response to drug’, ‘activation of adenylate cyclase’, ‘cell cycle’ and ‘angiogenesis’. Different preferences in strand placement were also observed for 5′-UTR SLQS from distinct functional classes of genes (Figure 7b and Supplementary Table S9). Notably, 3 GeneGo process categories are enriched with only 5′-UTR SLQS located on the non-template strand, namely ‘adenylate cyclase-modulating G-protein coupled receptor’, ‘positive regulation of ERK1 and ERK2 cascade’, and ‘cell cycle’, whereas diverse GeneGo process categories are enriched with 5′-UTR SLQS located on the template strand. We note that in the former case, QDH could be formed either on the non-template DNA or the transcribed RNA, and hence could possess additional translational regulatory potential, whereas in the latter case, QDH could only be formed on the template DNA.

### Mapping of SLQS to genomic loci of interest

Multiple annotation sets were overlaid across the sequence hits in order to isolate SLQS with potential functional and therapeutic implications (Figure 3d). A total of 8598 SLQS identified in the promoter regions were mapped against chromatin accessibility regions and transcription factor binding sites (annotations from UCSC Genome Browser (47)) to reveal 4852 SLQS within transcriptional regulatory regions. Cross-mapping and manual inspection of these sequences across various gene databases yielded numerous SLQS that are located within transcriptional active sites of cancer-associated genes (oncogenes and tumour suppressors) and genes involved in cell proliferation, apoptosis, signalling and epigenetic regulation, which could serve as prospective anticancer targets (15,59). Examples of these genes, among which include oncogene *PIM1*, proto-oncogene *RET*, members of RAS oncogene family (*RAB3D* and *RAB12*), neurite growth-promoting factor midkine (*MDK*) and cell cycle regulator cyclin Y (*CCNY*), are summarized in Table 2. On the other hand, 17 SLQS were mapped onto experimentally defined G-quadruplex-forming regions (determined through deep-sequencing of genomic DNA fragments, extracted from human breast adenocarcinoma cells, that are bound by G-quadruplex-specific antibody (60)), some of which are devoid of PQS_L7_ motifs (e.g. the segment of Intron 1 of *KISS1* gene where G4ST01202430640 is located; Supplementary Figure S2). These observations lent support to the formation of QDH in the context of double-stranded genomic DNA. The sequence hits were also cross-checked against mutation (The Cancer Genome Atlas (48) (TCGA)) and single-nucleotide polymorphism (SNP) (dbSNP (49), NCBI) databases to further identify SLQS that are situated across these genomic loci of potential interest/relevance (Supplementary Tables S10 and 11).

**Table 2.** Selective genes for which SLQS have been located within

Gene Symbol	Description	RefSeq ID	SLQS ID	Placement	Distance from TSS	Gene segment	Regulatory elements
*RICTOR*	*Homo sapiens* RPTOR independent companion of MTOR, complex 2, mRNA.	NM_152756	G4ST05039110287	non-template	−29	Promoter	Yes
*RAB3D*	*H. sapiens* RAB3D, member RAS oncogene family, mRNA.	NM_004283	G4ST19011311393	template	−49	Promoter	Yes
			G4ST19011311397	template	−53	Promoter	Yes
*RAB12*	*H. sapiens* RAB12, member RAS oncogene family, mRNA.	NM_001025300	G4ST18008599361	template	−58	Promoter	Yes
*CD24*	*H. sapiens* CD24 molecule, mRNA.	NM_013230	G4ST24019614192	non-template	−99	Promoter	Yes
*MYBL1*	*H. sapiens* v-myb myeloblastosis viral oncogene homologue (avian)-like 1, transcript variant 1, mRNA.	NM_001080416	G4ST08067688172	template	−138	Promoter	Yes
*NDRG2*	*H. sapiens* NDRG family member 2, transcript variant 3, mRNA.	NM_016250	G4ST14020563933	template	−158	Promoter	Yes
			G4ST14020563945	template	−170	Promoter	Yes
*PRKRIR*	*H. sapiens* protein-kinase, interferon-inducible double stranded RNA dependent inhibitor, repressor of P58 repressor, mRNA.	NM_004705	G4ST11075769784	non-template	−256	Promoter	Yes
*HIC1*	*H. sapiens* hypermethylated in cancer 1, transcript variant 1, mRNA.	NM_001098202	G4ST17001906034	template	−285	Promoter	Yes
		NM_001098202	G4ST17001906030	template	−289	Promoter	Yes
		NM_001098202	G4ST17001906024	template	−293	Promoter	Yes
*PDK1*	*H. sapiens* pyruvate dehydrogenase kinase, isozyme 1, nuclear gene encoding mitochondrial protein, mRNA.	NM_002610	G4ST02173128695	non-template	−298	Promoter	Yes
*IGF2BP2*	*H. sapiens* insulin-like growth factor 2 mRNA binding protein 2, transcript variant 1, mRNA.	NM_006548	G4ST03187025840	non-template	−319	Promoter	Yes
*CCNY*	*H. sapiens* cyclin Y, transcript variant 1, mRNA.	NM_145012	G4ST10035665408	non-template	−373	Promoter	Yes
*RET*	*H. sapiens* ret proto-oncogene, transcript variant 2, mRNA.	NM_020630	G4ST10042892416	template	−80	Promoter	Yes
			G4ST10042891022	template	−1472	Promoter	Yes
			G4ST10042891015	template	−1477	Promoter	Yes
*MDK*	*H. sapiens* midkine (neurite growth-promoting factor 2), transcript variant 2, mRNA.	NM_001012334	G4ST11046359281	template		5′-UTR	Yes
*PIM1*	*H. sapiens* pim-1 oncogene, mRNA.	NM_002648	G4ST06037246104	template		5′-UTR	Yes
*PDCD4*	*H. sapiens* programmed cell death 4 (neoplastic transformation inhibitor), transcript variant 1, mRNA.	NM_014456	G4ST10112621932	template		Intron 1	Yes
*ARHGAP31*	*H. sapiens* Rho GTPase activating protein 31, mRNA.	NM_020754	G4ST03120547683	template		Intron 1	Yes
*ELFN1*	*H. sapiens* extracellular leucine-rich repeat and fibronectin type III domain containing 1, mRNA.	NM_001128636	G4ST07001717478	non-template		Intron 1	
*MYO9B*	*H. sapiens* myosin IXB, transcript variant 2, mRNA.	NM_004145	G4ST19017166594	template		Exon 22	
*BMP8A*	*H. sapiens* bone morphogenetic protein 8a, mRNA.	NM_181809	G4ST01039729840	non-template	−28	Promoter	Yes
*MYT1L*	*H. sapiens* myelin transcription factor 1-like, mRNA.	NM_015025	G4ST02001786748	template		Intron 22	

### SLQS adopt diverse QDH structures

**Figure 8.**
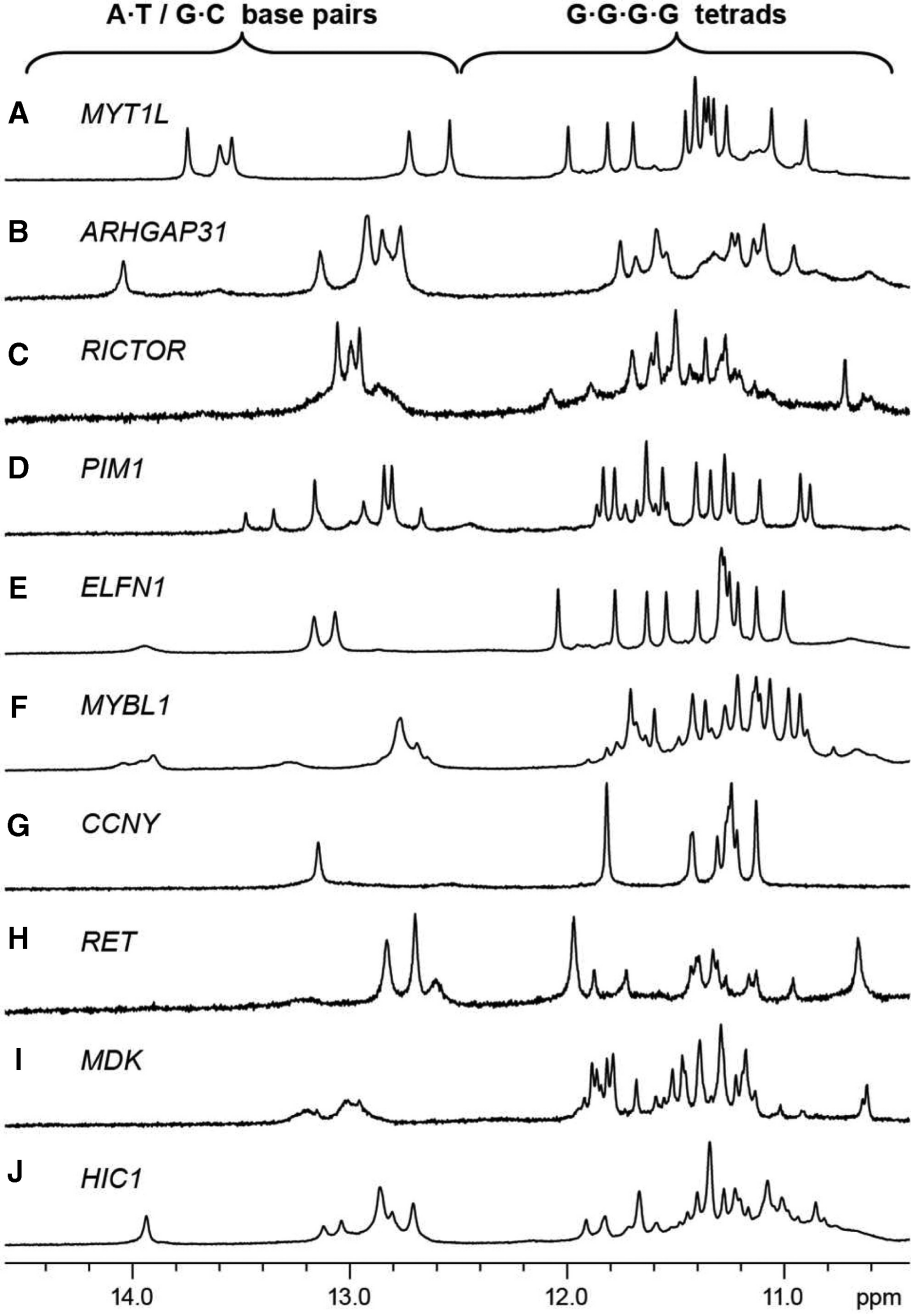
One-dimensional (1D) imino proton NMR spectra of SLQS identified across various genes in the human genome. Gene names for which the respective SLQS have been located within are indicated. Typical chemical shift ranges for imino protons participating in the formation of Watson–Crick base-pairs (A•T/G•C) and G-tetrads (G•G•G•G) are demarcated. (**A**) G4ST02001786748, (**B**) G4ST03120547683, (**C**) G4ST05039110287, (**D**) G4ST06037246104, (**E**) G4ST07001717478, (**F**) G4ST08067688172, (**G**) G4ST10035665408, (**H**) G4ST10042891022, (**I**) G4ST11046359281 and (**J**) G4ST17001906030.

To investigate the potential formation of QDH (44,46) by SLQS, we proceeded with the NMR characterization on a selected list of sequence hits (Table 3 and Supplementary Table S12). Imino protons (from thymine and guanine bases) of Watson–Crick base pairs typically resonate at 12.5–14.5 ppm while guanine imino protons of G-tetrads mostly resonate at 10.5–12.5 ppm. For the series of SLQS inspected, most of them displayed imino proton peaks in both Watson–Crick and G-tetrad regions (Figure 8 and Supplementary Figure S3), pointing to the coexistence of duplex and quadruplex elements and the likely formation of QDH. Many of these sequences exhibited multiple conformations, as shown by the number and intensity of duplex and tetrad imino proton peaks (e.g. G4ST05039110287 (*RICTOR* promoter), G4ST08067688172 (*MYBL1* promoter) and G4ST17001906030 (*HIC1* promoter); Figure 8c, f and j, respectively). In a few cases, the spectra showed the presence of a single major conformation. For instance, G4ST02001786748 (*MYT1L* Intron 22) and G4ST07001717478 (*ELFN1* Intron 1) displayed distinct sharp peaks amenable for detailed structural characterization (Figure 8a and e, respectively; see NMR data below). In other cases, weak/broadened (G4ST11046359281 (*MDK* 5′-UTR); Figure 8i) or fewer-than-expected (G4ST10035665408 (*CCNY* promoter); Figure 8g) duplex imino proton peaks suggested the absence of a stable stem-loop or the adoption of an alternative structure. Note the presence of both Watson–Crick duplex and G-tetrad imino proton peaks alone do not necessarily prove the adoption of QDH structures. Nevertheless, several observations could be made to reasonably suggest the adoption of such structures, for instance the matching peak intensity corresponding to G-tetrad and Watson–Crick imino protons of the major and minor species (Figure 8d). In two particular cases (Figure 8a and e), the detailed QDH folding topologies were fully determined (see below). The diverse sequence contexts of our predicted hits showed that a wide range of QDH with different topologies could be established.

**Table 3.** Representative SLQS oligonucleotides used for NMR study

SLQS ID	Sequence^a,b,c,d^	Gene symbol	Remark
G4ST02001786748	5′-A **GGG**AGAGGAGAGCTCT**GGG** TT **GGG** T **GGG**-3′	*MYT1L*	Single major conformation
G4ST03120547683	5′-GT **GGG** TGCAGTCAGAGCTGCT **GGG** AGT **GGG**TAGCCTCAGGCTA**GGG**-3′	*ARHGAP31*	Single major conformation
G4ST05039110287	5′-GC **GGG**CGCGGCGCGCG**GGG** A **GGGG** AA **GGGG** T-3′	*RICTOR*	Multiple conformations
G4ST06037246104	5′-GC **GGG** A **GGG**CGCGCCAGCG**GGG** TC **GGG** C-3′	*PIM1*	Two major conformations
G4ST07001717478	5′-CA **GGG** T **GGG** TCTGCTGTGCAG**GGG** T **GGG** T-3′	*ELFN1*	Single major conformation
G4ST08067688172	5′-GA **GGG** C **GGGG** CT **GGGG**AGCTGGAAGCT**GGG** A-3′	*MYBL1*	Multiple conformations
G4ST10035665408	5′-GA **GGG** C **GGG**CGCCGCTGGCGA **GGG** A **GGG** C-3′	*CCNY*	Single major conformation^c^
G4ST10042891022	5′-CA **GGG** AA **GGG**ACCTGATAGGTA **GGG** A **GGGG** C-3′	*RET*	Two major conformations^d^
G4ST11046359281	5′-CT **GGG**GCGGTTTCCGC**GGG** T **GGG** CA **GGG** A-3′	*MDK*	Multiple conformations^e^
G4ST17001906030	5′-GT **GGG** G **GGG** A **GGG**GGGAGCCACGCAGCTCCCA **GGGG** A-3′	*HIC1*	Multiple conformations

^a^G-tracts are in boldface.

^b^Self-complementary tracts are underlined.

^c^Structure may not correspond to a quadruplex–duplex hybrid.

^d^One conformation may not correspond to a quadruplex–duplex hybrid.

^e^Absence of stable duplex stem-loop in the major conformation.

### Sequence mutation study of SLQS

**Figure 9.**
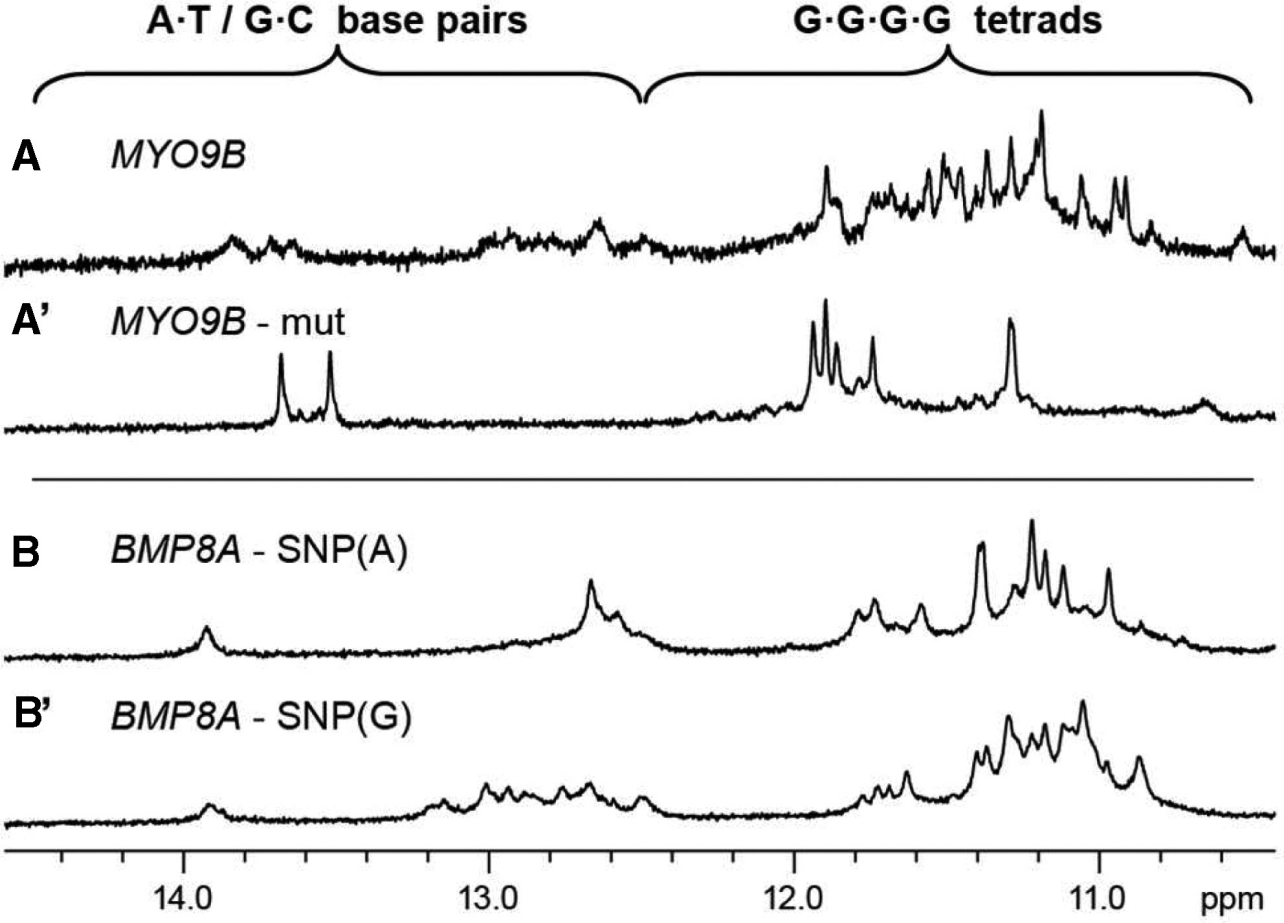
Modifications to nucleotide sequences affect the structural conformations adopted by SLQS. Shown here are the 1D imino proton NMR spectra of (**A**) G4ST19017166594 and (**A’**) its mutated counterpart G4ST19017166594del from the *MYO9B* gene, as well as two single-nucleotide polymorphs (**B**) G4ST01039729840A and (**B’**) G4ST01039729840G from the *BMP8A* gene.

Effects of nucleotide changes (arising from mutation or SNP) on the formation of QDH by SLQS were also examined. The *MYO9B* gene sequence G4ST19017166594 and its mutated counterpart G4ST19017166594del with a GAGAGT deletion (Table 4), which is observed in tumour cell lines (TCGA mutation database (48)), showed strikingly different imino proton NMR spectra (Figure 9a and a’), indicating their adoption of completely different folding topologies. Interestingly, the sequence context and spectral characteristics of G4ST19017166594del suggested that the structure might correspond to a G•C•G•C tetrad-containing quadruplex (61,62). Single-nucleotide changes, in the form of SNP, could also affect the proportions of different SLQS populations, as evidenced by two polymorphs of the *BMP8A* gene segment G4ST01039729840A and G4ST01039729840G (Table 4 and Figure 9b and b’).

**Table 4.** SLQS oligonucleotides used for mutational study

SLQS ID	Sequence^a,b,c,d^	Gene symbol	Remark
G4ST19017166594	5′-CT **GGGG** T **GGG**AGAGTCTTCTCT **GGG** CTT **GGGG** C-3′	*MYO9B*	Mutation site
G4ST19017166594del	5′-CT **GGGG** T **GG**– – – – – – CTTCTCT **GGG** CTT **GGGG** C-3′		
G4ST01039729840A	5′-CC **GGGGG** T **GGG** C **GGG** C(A)GCACAGACGGCTGAGCCG **GGG** C-3′	*BMP8A*	SNP locus
G4ST01039729840G	5′-CC **GGGGG** T **GGG** C **GGG**C(G)GCACAGACGGCTGAGCCG **GGG** C-3′		

^a^G-tracts are in boldface.

^b^Self-complementary tracts are underlined.

^c^Deletions are indicated by dash signs.

^d^The nucleotide positions that exhibit single-nucleotide polymorphism are bracketed.

### QDH folding topologies of two SLQS

**Figure 10.**
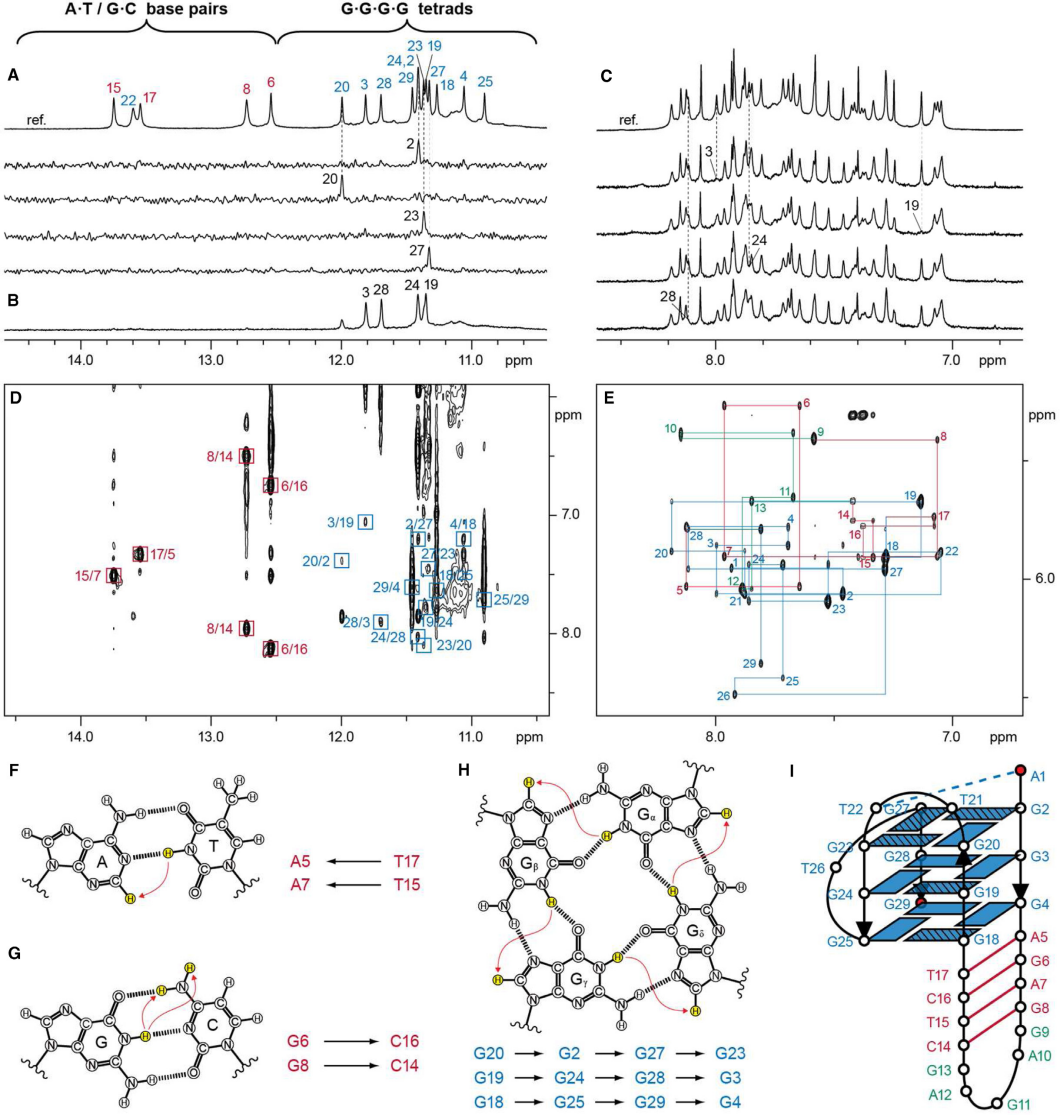
NMR structural characterization of the *MYT1L* gene sequence G4ST02001786748. (**A**) Assignments of G-tetrad imino protons through ^15^N-filtered spectra of samples, 2% ^15^N-labelled at the indicated positions. (**B**) 1D imino proton NMR spectrum after 2 h in D_2_O at 25°C. (**C**) Assignments of guanine H8 protons through site-specific ^2^H labelling at the indicated positions. (**D**) NOESY spectrum (mixing time, 200 ms) showing the cross-peaks that establish the alignment of the four Watson–Crick base pairs and the three G-tetrads. Cross-peaks between thymine imino proton and adenine H2 proton, and between guanine imino proton and cytosine amino protons, are framed in red and labelled with the residue number of thymine/guanine, followed by that of adenine/cytosine. Cross-peaks arising from imino-H8 connectivity around the three G-tetrads are framed in blue and labelled with the residue number of imino proton, followed by that of H8 proton. (**E**) NOESY spectrum (mixing time, 300 ms) showing the H8/H6-H1’ sequential connectivity of G4ST02001786748. Intraresidue H8/H6-H1’ NOE cross-peaks are labelled with residue numbers. (**F**) NOEs from thymine imino proton to adenine H2 proton that establish the A5•T17 and A7•T15 base pairs. (**G**) NOEs from guanine imino proton to cytosine amino protons that establish the G6•C16 and G8•C14 base pairs. (**H**) Cyclic guanine imino-H8 NOE connectivity patterns around a G_α_•G_β_•G_γ_•G_δ_ tetrad as indicated with arrows, with the connectivities observed for the G20•G2•G27•G23, G19•G24•G28•G3 and G18•G25•G29•G4 tetrads shown below. (**I**) Schematic diagram of the quadruplex–duplex hybrid structure adopted by G4ST02001786748. Quadruplex, duplex, and hairpin loop segments are coloured in blue, red and green, respectively. The 5′- and 3′-termini are shown as red circles. The non-canonical A1•T22 base pair is shown as a dotted line.

Detailed NMR structural characterization of the *MYT1L* gene sequence G4ST02001786748 (d[A **GGG** AGAGGAGAGCTCT **GGG** TT **GGG** T **GGG**]; tetrad guanine residues in bold, self-complementary tracts underlined) was performed. One-dimensional imino proton NMR spectrum of G4ST02001786748 indicated the adoption of a single predominant QDH structure; five major peaks at 12.5–13.8 ppm corresponded to the formation of five base pairs while twelve major peaks at 10.9–12.0 ppm corresponded to the establishment of three G-tetrads (Figure 10a). The same QDH conformation was adopted in varying cation types and conditions (Supplementary Figure S4). Concentration-independent melting profile of the CD spectra (melting temperature of ∼54°C at 40–50 mM K^+^; Supplementary Figure S5), as monitored at 295 nm, indicated the formation of an intramolecular structure by G4ST02001786748. Selective guanine imino (Figure 10a) and H8 (Figure 10c) protons of G4ST02001786748 were unambiguously assigned using site-specific low-enrichment ^15^N-labelling (54) and site-specific ^2^H-labelling (55) (Supplementary Table S13), respectively. Through-bond correlation experiments (COSY, TOCSY and ^13^C-^1^H-HSQC) facilitated the determination of the H8/H6-H1′ sequential connectivity of the oligonucleotide (Figure 10e). Guanine residues G2, G18, G19, G23 and G27 assume *syn* glycosidic conformation (as shown by the strong intensity of their intraresidue H8-H1′ NOE cross-peaks), whereas the other tetrad guanine residues adopt *anti* glycosidic conformation. Cyclic imino-H8 NOE connectivity patterns within the tetrads (Figure 10d and h) pointed to the formation of a (3+1) G-tetrad core (with three strands oriented in the same direction and one strand oriented in the opposite direction) consisting of three G-tetrad layers, G20•G2•G27•G23, G19•G24•G28•G3 and G18•G25•G29•G4, with the first tetrad arranged in the opposite hydrogen-bond directionality with respect to the latter two (Figure 10i). Glycosidic conformations of guanines around the first tetrad are *anti*•*syn*•*syn*•*syn* while those for the two other tetrads are *syn*•*anti*•*anti*•*anti*. The slower solvent exchange rate shown by imino protons of G3, G19, G24 and G28 (Figure 10b) is consistent with the central placement of G19•G24•G28•G3 (between the G20•G2•G27•G23 and G18•G25•G29•G4 tetrads) in the tetrad core. Signature imino-H2 (A•T base pair; Figure 10f) and imino-amino (G•C base pair; Figure 10g) NOE cross-peaks (Figure 10d) verified the establishment of the four Watson-Crick base pairs (A5•T17, G6•C16, A7•T15 and G8•C14) constituting the stem-loop, which extends directly from the wide groove of the tetrad core (Figure 10i). Continuous stacking between the quadruplex and duplex segments was supported by NOE cross-peaks between sugar and base protons of tetrad residues (G4 and G18) and the adjacent base pair (A5•T17). The duplex stem is closed off at the distal end by a five-nucleotide hairpin loop (G9-A10-G11-A12-G13). The second, edgewise, loop (T21–T22) of the quadruplex crosses a narrow groove, while the third loop (T26) traverses across a medium groove through the double-chain-reversal configuration. A non-canonical A1•T22 base pair capping the top of the tetrad core accounted for the imino proton peak at ∼13.6 ppm. Overall, the quadruplex segment of G4ST02001786748 bears a similar loop arrangement as a number of (3 + 1) G-quadruplexes previously observed under various sequence contexts (23,26,63,64). In particular, we note that G4ST02001786748 closely resembles the structure of Form 1 *hTERT* promoter G-quadruplex (26), with the GAG loop of the latter replaced by a duplex stem-loop.

**Figure 11.**
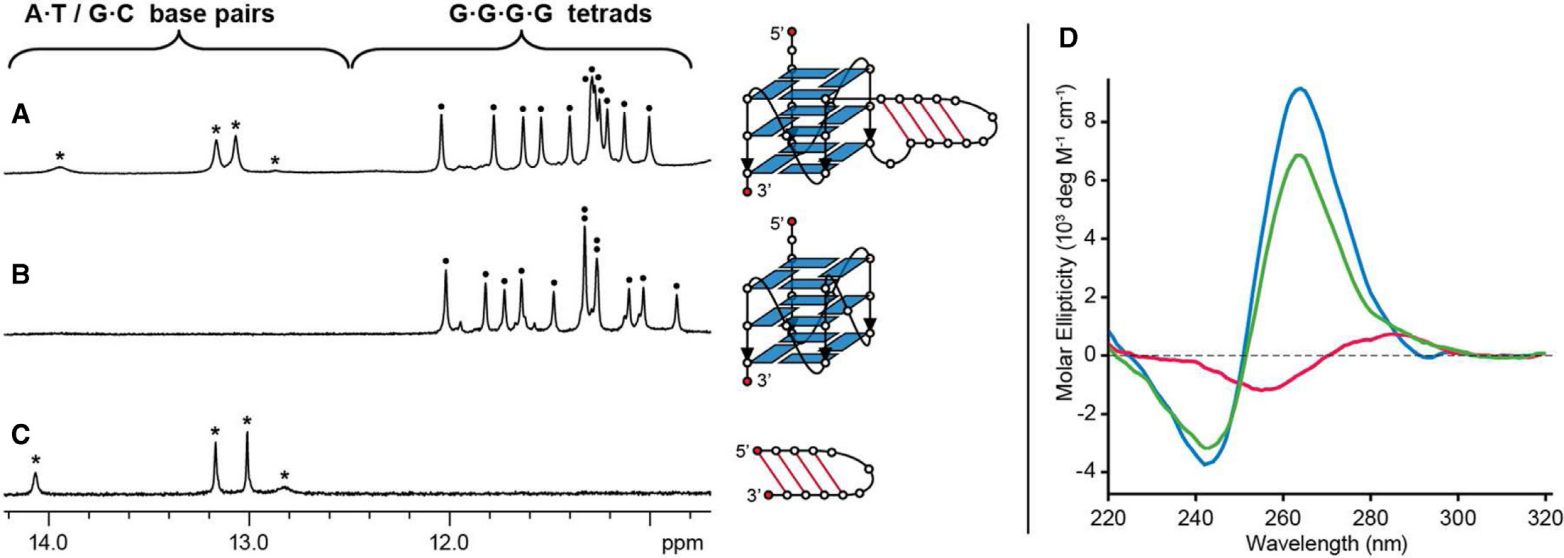
NMR and CD spectra of the *ELFN1* gene sequence G4ST07001717478. (**A**–**C**) NMR spectra of (a) G4ST07001717478 and its reference (b) quadruplex (G4ST07001717478_G4) and (c) duplex (G4ST07001717478_dx), with their respective schematic structures shown on the right. Quadruplex and duplex imino proton peaks are marked by circles and asterisks, respectively. (**D**) CD spectra of G4ST07001717478 (green), G4ST07001717478_G4 (blue) and G4ST07001717478_dx (red).

The *ELFN1* intron sequence G4ST07001717478 was next examined. Previously, it has been well established that the sequence motif GGGNGGG readily adopts a parallel-stranded arrangement of the two G-tracts through a double-chain-reversal configuration of the single-nucleotide loop (30,41,65,66). In the case of G4ST07001717478, the two GGGNGGG motifs flanking the middle duplex stem-containing loop would predispose the sequence towards adopting an all-parallel-stranded G-quadruplex core. Indeed, CD spectrum of G4ST07001717478 was similar to that of its reference quadruplex, G4ST07001717478_G4 (Supplementary Table S12), showing a positive peak near 260 nm and a negative peak near 240 nm (Figure 11d), characteristic of an all-parallel-stranded G-quadruplex (67,68). NMR imino proton peaks of G4ST07001717478 showed similar chemical shift patterns as G4ST07001717478_G4 and its reference duplex G4ST07001717478_dx (Supplementary Table S12) (Figure 11a–c). In the 2D NOESY spectra of G4ST07001717478, intensity of intraresidue H8-H1’ cross-peaks indicated that all guanine residues adopt an *anti* glycosidic conformation, consistent with its adoption of an all-parallel-stranded G-quadruplex. The same QDH conformation was adopted in varying cation types and concentrations (Supplementary Figure S6).

## DISCUSSION

### SLQS and PQS in the human genome

In this study, we explored the existence of SLQS in the human genome, which would otherwise have been overlooked by conventional PQS algorithms (generally complying with the form G_X1_N_L1_G_X2_N_L2_G_X3_N_L3_G_X4_) exercising an arbitrary loop-length cut-off of 7-nt (35,36) (e.g. *quadparser* algorithm and PQS_L7_). Based on a loop length of 8–20 nt, our prediction model identified 80 307 SLQS, 48 508 of which were located across genic and gene promoter regions. More than 68% (33 148 out of 48 508) of these SLQS do not overlap with PQS_L7_, representing a pool of genomic regions that could exert novel (previously uncharacterized) gene regulatory signals. Note that for practical considerations, here we limited the G-tract and initial loop length of SLQS to 3–6 nt and 20 nt, respectively, while stable QDH structures comprising only two G-tetrad layers (*X* = 2), or containing longer stem-loops (*L* > 20), may well exist. Nevertheless, we expect that our model would cover close to half of total SLQS population in the entire human genome.

### Biological implications of SLQS and QDH

In the context of double helical DNA, QDH formation by SLQS motifs could potentially arise when unwinding of the double helix occurs (e.g. during replication or transcription). For instance, single-stranded segments of genomic DNA may persist up to hundreds of nucleotides behind a DNA helicase, which could reasonably encompass the SLQS motifs examined in this study.

Bioinformatics analyses were performed on the distribution of SLQS across genic and gene promoter regions of the human genome. The 48 508 SLQS motifs were found to spread across a total of 12 315 genes. Distribution of these loci across protein-coding genes, according to the count of SLQS per gene, assumes the pattern of a skewed function exhibiting a high level of inequality, which can be approximated based on the K–W model (51,52). By the theory, such statistics were expected of an evolving, proliferating, death and selection in a population.

Strong tissue specificity with regards to the brain was observed for the group of genes encompassing SLQS motifs. Many of these brain tissue related genes exhibit high frequencies of SLQS motifs. Specifically, our study predicts important role of SLQS motifs in the biological function of the *PTPRN2* gene locus and its products. *PTPRN2* is the most SLQS-populated human gene, containing 129 SLQS motifs. PTPRN2 protein encoded by this gene is a member of the protein tyrosine phosphatase (PTP) family. PTPs are known signalling molecules that regulate a variety of cellular processes including cell growth, differentiation, mitotic cycle and oncogenic transformation. PTPRN2 protein was identified as a major autoantigen associated with insulin-dependent diabetes mellitus. Three alternatively spliced transcript variants of this gene, which encode distinct proteins, have been reported. *PTPRN2* is also one of the most R-loop-forming sequences (RLFS)-populated genes in the human genome, containing 140 RLFS (69). During transcription, the nascent RNA of RLFS could give rise to transcriptional RNA-DNA hybrid with the template DNA strand, leaving the non-template DNA strand unpaired. Such single-stranded DNA are G-rich and have the potential to form the R-loop (69). High frequencies of R-loops are often associated with DNA breaks, genome instability regions, cancer and mental diseases. According to the COSMIC database, *PTPRN2* copy number variations are observed in several cancers (for instance, in 46% of ovarian cancers) and in 73% of central neural system disorder tissues. It cannot be ruled out that SLQS motifs localized in RLFS loci are cooperative with RLFS in promoting R-loop formation and/or its stabilization, maintaining open chromatin and stalling RNA polymerase.

The SLQS motifs were found across numerous genomic loci of biological and medical interest. Out of 8598 SLQS identified in the gene promoter regions, more than 56% were mapped onto chromatin accessibility regions and transcription factor binding sites. Moreover, distributions of SLQS in TSS-proximal regions indicated their high enrichment, similar to PQS. Some of these could conceivably exert a regulatory function in transcription as that posited for promoter-associated PQS (17,18). Our GO analysis revealed that, in gene promoter regions, most of the GO categories (e.g. GeneGo process categories related to regulation of transcription, development, neurogenesis and kinase activity) of genes containing regulatory SLQS are consistent with the GO categories previously reported for promoter PQS (18). In contrast, in 5′-UTR regions, the GO categories (e.g. ‘response to drug’, ‘cell cycle’ and ‘angiogenesis’) of genes containing 5′-UTR SLQS show greater variations from those of genes containing 5′-UTR PQS. These observations suggest that there could be an association between the functional conservation of genes containing 5′-UTR SLQS and QDH formation. Interestingly, the GO category ‘cell cycle’ was found to be highly enriched in genes containing 5′-UTR SLQS on the non-template strand. For these sequences, QDH could either form on the non-template DNA or the transcribed RNA. Hence in addition to transcriptional regulation, QDH-forming sequences could affect the splicing and translation of genes that are involved in gene expression and cell cycle processes through the formation of 5′-UTR in nascent RNA, pre-mRNA or mRNA (70,71).

Recently, it has been shown that non-coding RNAs are produced in a pervasive manner (72) and their biological functions remain poorly understood. It could be worthwhile to explore if RNA QDH reside within these transcripts. Utilizing the present SLQS algorithm, we have identified 23 742 and 2429 SLQS with the potential to form RNA QDH within pre-mRNA and mature mRNA, respectively. We note that among these, 921 are localized to the 5′-UTR and could exhibit translational regulatory potential. As illustrated by the complex folding topology adopted by the aptameric RNA QDH targeting the fragile X mental retardation protein (FMRP) (73), folding principles governing their formations could deviate considerably from those that apply to the DNA counterparts. More of such structure will be needed before an algorithm can be developed to identify these RNA motifs with greater certainty.

Several SLQS were identified at mutation and SNP loci. These observations suggested the possible associations of SLQS with genome instability, genetic diseases and specific biological processes. Some of the SLQS were found to reside within experimentally defined G-quadruplex-forming regions (60), corroborating the formation of QDH in the context of genomic DNA. Further experimental studies will be required to shed light on the potential (patho)biological significance of these motifs.

### Structures of SLQS

Accurate identification and prediction of QDH-forming genomic sequences will be helpful towards the understanding of their potential biological functions. Towards this end, we carried out structural characterization on a selected list of SLQS using NMR. Among the sequence hits investigated include promoter-associated SLQS from cancer-associated genes such as *RAB3D*, *RAB12*, *HIC1*, *RET* and *CCNY*. Most of these SLQS displayed NMR signatures of both duplex and quadruplex elements, supporting their adoption of QDH structures. Structural polymorphism was observed in numerous cases, whereas a single predominant conformation was observed in a handful of other cases. These examples showcase the structural diversity that can arise from SLQS motifs. We determined the folding topology of the QDH adopted by two particular SLQS hits (G4ST02001786748 from Intron 22 of the *MYT1L* gene and G4ST07001717478 from Intron 1 of the *ELFN1* gene). Detailed understanding on the structures that can be adopted by various SLQS will aid in future drug design efforts targeting these non-canonical nucleic acid structures.

We have shown previously that QDH with three SL in three separate loops (44) and two or more SL in a single loop (74), can exist *in* *vitro*. In addition, G4 structures with more complex scaffold have been previously observed (21,29,75–81). In this study, two SLQS having different folding topologies and placement of duplex SL were examined. Taken together, these suggest that SLQS with yet more complex sequence contexts and drastically different features could exist.

## SUPPLEMENTARY DATA

Supplementary Data are available at NAR Online.

SUPPLEMENTARY DATA
