# Supplementary material for: Duplex stem-loop-containing quadruplex motifs in the human genome: a combined genomic and structural study
Source: Nucleic Acids Res. 2015 May 9;43(11):5630–46. doi: 10.1093/nar/gkv355 (PMC4477648; doi:10.1093/nar/gkv355)
Supplement: SUPPLEMENTARY DATA [file supp_43_11_5630__index.html]

Duplex stem-loop-containing quadruplex motifs in the human genome: a combined genomic and structural study — SUPPLEMENTARY DATA 

# Duplex stem-loop-containing quadruplex motifs in the human genome: a combined genomic and structural study

## SUPPLEMENTARY DATA

- SUPPLEMENTARY DATA
- SUPPLEMENTARY DATA
- SUPPLEMENTARY DATA
- SUPPLEMENTARY DATA
- SUPPLEMENTARY DATA
