# Supplementary material for: Duplex stem-loop-containing quadruplex motifs in the human genome: a combined genomic and structural study
Source: Nucleic Acids Res. 2015 May 9;43(11):5630–46. doi: 10.1093/nar/gkv355 (PMC4477648; doi:10.1093/nar/gkv355)
Supplement: SUPPLEMENTARY DATA [file supp_gkv355_nar-00300-f-2015-File002.pdf]

Duplex stem-loop-containing quadruplex motifs in the human genome: A combined genomic and structural study

Kah Wai Lim[1,2], Piroon Jenjaroenpun[3], Zhen Jie Low[1,2], Zi Jian Khong[1], Yi Siang Ng[1], Vladimir Andreevich Kuznetsov[3,*] & Anh Tuân Phan[1,*]

[1]School of Physical and Mathematical Sciences, Nanyang Technological University, Singapore 637371. [2]School of Biological Sciences, Nanyang Technological University, Singapore 637551. [3]Department of Genome and Gene Expression Data Analysis, Bioinformatics Institute, Singapore 138671.

Supplementary Information

Supplementary Table 1. The eleven highest SLQS-populated genes in the human genome.

Gene	SLQS Count	Gene Description
PTPRN2	129	protein tyrosine phosphatase, receptor type, N polypeptide 2, transcript variant 2, mRNA
SBNO2	96	strawberry notch homolog 2 (*Drosophila*), transcript variant 1, mRNA
CDH4	87	cadherin 4, type 1, R-cadherin (retinal), transcript variant 1, mRNA
EXD3	84	exonuclease 3'-5' domain containing 3, transcript variant 1, mRNA
SORCS2	84	sortilin-related VPS10 domain containing receptor 2, mRNA
KCNQ1	76	potassium voltage-gated channel, KQT-like subfamily, member 1, transcript variant 1, mRNA
RBFOX3	75	RNA binding protein, fox-1 homolog (*C. elegans*) 3, mRNA
LHPP	73	phospholysine phosphohistidine inorganic pyrophosphate phosphatase, transcript variant 1, mRNA
CAMTA1	73	calmodulin binding transcription activator 1, transcript variant 1, mRNA
MAD1L1	65	MAD1 mitotic arrest deficient-like 1 (yeast), transcript variant 2, mRNA
CTDP1	61	CTD (carboxy-terminal domain, RNA polymerase II, polypeptide A) phosphatase, subunit 1, transcript variant 3, mRNA

Supplementary Table 2. Gene ontology analysis of SLQS-containing brain associated genes across tissue subcategories and disease associations. 2,700 gene symbols were randomly chosen from the list of 4,541 brain tissue related genes (classified under the term "brain" within UP_TISSUE annotation set of the DAVID bioinformatics resources (1)) and subjected to gene ontology analysis by DAVID bioinformatics resources, of which 2,674 have been annotated by the system.

Term: Brain	Count	List Total	%	Pop Hits	Pop Total	Fold Enrichment	P-Value	Bonferroni[a]	Benjamini[b]
				UP_TISSUE					
Brain	2,674	2,674	100.00	7,789	18,201	2.34	<2.07E-24	<2.07E-24	<2.07E-24
Amygdala	186	2,674	6.96	605	18,201	2.09	2.07E-24	8.02E-22	4.01E-22
Epithelium	545	2,674	20.38	2,567	18,201	1.45	1.68E-22	6.50E-20	2.17E-20
Fetal brain	199	2,674	7.44	770	18,201	1.76	2.17E-16	8.59E-14	2.14E-14
Hippocampus	128	2,674	4.79	456	18,201	1.91	1.47E-13	5.69E-11	1.14E-11
Cerebellum	153	2,674	5.72	667	18,201	1.56	9.19E-09	3.56E-06	5.93E-07
Peripheral nervous system	24	2,674	0.90	56	18,201	2.92	1.44E-06	5.56E-04	7.95E-05
				GENETIC_ASSOCIATION_DB_DISEASE_CLASS					
PSYCH	145	493	5.42	557	3,049	1.61	3.75E-11	6.76E-10	6.76E-10
				GENETIC_ASSOCIATION_DB_DISEASE					
Schizophrenia	82	490	3.07	288	3,028	1.76	4.25E-08	7.85E-05	7.85E-05
Bipolar disorder	39	490	1.46	107	3,028	2.25	5.14E-07	9.49E-04	4.74E-04

[a]P-values after Bonferroni correction. [b]P-values after Benjamini correction.

Supplementary Table 3. Enrichment of SLQS motifs in brain tissue related genes. 4,541 SLQS-containing protein-coding genes are associated with the brain tissue (classified under the term "brain" within UP_TISSUE annotation set of the DAVID bioinformatics resources (1)). Sub-categorization of these genes according to the count of SLQS per genes showed that the enrichment is more pronounced within the group of genes with high frequencies (>5) of SLQS.

Category (for Unique Gene Symbols)	Number of Gene Symbols	List Total	% Gene Symbols	Fold Enrichment[a]	P-Value	Benjamini[b]
16-130 Events	216	317	68.14	1.59	4.08E-20	6.77E-18
11-15 Events	220	354	62.15	1.45	1.65E-13	2.82E-11
8-10 Events	333	551	60.44	1.41	1.66E-17	3.39E-15
6-7 Events	422	730	57.81	1.35	4.15E-17	1.02E-14
5 Events	283	546	51.83	1.21	1.22E-05	2.79E-03
4 Events	415	832	49.88	1.17	1.78E-05	4.45E-03
3 Events	610	1,221	49.96	1.17	1.25E-07	3.36E-05
2 Events	866	1,841	47.04	1.10	6.31E-05	2.13E-02
1 Event	1,218	2,658	45.82	1.07	3.52E-04	4.44E-02

[a]Expected population frequency (by chance) = 43% (7,789/18,201*100%). [b]P-values after Benjamini correction.

Supplementary Table 4. Gene ontology analysis of SLQS-containing brain tissue related genes based on CGAP_SAGE_QUARTILE annotation set of DAVID bioinformatics resources (1).

Term: Brain	Count	List Total	%	Pop Hits	Pop Total	Fold Enrichment	P-Value	Bonferroni[a]	Benjamini[b]
Count of Events						16-130			
656:brain_null_3rd	59	240	24.6	1,266	15,839	3.08	9.04E-15	3.12E-12	3.12E-12
430:brain_null_3rd	68	240	28.3	1,772	15,839	2.53	4.78E-13	1.66E-10	8.29E-11
133:brain_normal pediatric cortex_3rd	58	240	24.2	1,759	15,839	2.18	1.49E-08	5.18E-06	1.73E-06
10:brain_normal, cerebral cortex_3rd	53	240	22.1	1,578	15,839	2.22	4.44E-08	1.54E-05	3.85E-06
564:brain_Anaplastic GradeII Astrocytoma_3rd	48	240	20.0	1,425	15,839	2.22	2.25E-07	7.80E-05	1.56E-05
8:brain_normal whitematter, cerebral cortex_3rd	47	240	19.6	1,480	15,839	2.10	1.65E-06	5.72E-04	9.53E-05
Count of Events						11-15			
656:brain_null_3rd	53	283	18.7	1,266	15,839	2.34	9.66E-09	3.35E-06	1.68E-06
430:brain_null_3rd	71	283	25.1	1,772	15,839	2.24	7.12E-11	2.47E-08	2.47E-08
10:brain_normal, cerebral cortex_3rd	59	283	20.9	1,578	15,839	2.09	6.23E-08	2.16E-05	7.20E-06
Count of Events						8-10			
656:brain_null_3rd	85	455	18.7	1,266	15,839	2.34	2.08E-13	7.22E-11	2.41E-11
430:brain_null_3rd	107	455	23.5	1,772	15,839	2.10	6.67E-14	2.32E-11	1.16E-11
10:brain_normal, cerebral cortex_3rd	103	455	22.6	1,578	15,839	2.27	1.53E-15	5.39E-13	5.39E-13
8:brain_normal whitematter, cerebral cortex_3rd	80	455	17.6	1,480	15,839	1.88	3.77E-08	1.31E-05	3.27E-06
34:cerebellum_normal cerebellum_3rd	75	455	16.5	1,486	15,839	1.76	1.66E-06	5.74E-04	6.38E-05
425:colon_cell line derived from colorectal	63	455	13.9	1,108	15,839	1.98	2.77E-07	9.61E-05	1.92E-05
348:brain_astrocytoma grade II_3rd	78	455	17.1	1,519	15,839	1.79	4.77E-07	1.66E-04	2.76E-05
1368:cartilage_3rd	65	455	14.3	1,212	15,839	1.87	1.31E-06	4.54E-04	6.49E-05
53:brain_normal thalamus_3rd	87	455	19.1	1,810	15,839	1.67	1.38E-06	4.77E-04	5.97E-05
Count of Events						6-7			
656:brain_null_3rd	87	580	15.0	1,266	15,839	1.88	1.25E-08	4.33E-06	1.44E-06
564:brain_Anaplastic GradeII Astrocytoma_3rd	95	580	16.4	1,425	15,839	1.82	1.00E-08	3.47E-06	1.74E-06
8:brain_normal whitematter, cerebral cortex_3rd	99	580	17.1	1,480	15,839	1.83	3.73E-09	1.29E-06	1.29E-06
10:brain_normal, cerebral cortex_3rd	95	580	16.4	1,578	15,839	1.64	1.18E-06	4.08E-04	5.84E-05
1367:bone_3rd	91	580	15.7	1,399	15,839	1.78	7.15E-08	2.48E-05	6.20E-06
430:brain_null_3rd	107	580	18.5	1,772	15,839	1.65	1.68E-07	5.82E-05	1.16E-05
1368:cartilage_3rd	80	580	13.8	1,212	15,839	1.80	3.00E-07	1.04E-04	1.73E-05
Count of Events						5			
10:brain_normal, cerebral cortex_3rd	88	458	19.2	1,578	15,839	1.93	1.89E-09	6.57E-07	6.57E-07
7:brain_glioblastoma, cerebral cortex_3rd	84	458	18.3	1,616	15,839	1.80	1.15E-07	3.98E-05	1.99E-05

[a]P-values after Bonferroni correction. [b]P-values after Benjamini correction.

Supplementary Table 5. GO enrichment terms of genes containing SLQS at promoter region and regulatory elements with all human genes or PQS_{L7}-/SLQS-containing genes as background gene set. The GO analysis was performed using DAVID bioinformatics resources (1).

Term	List.Total	Background: human genes		Background: PQSL7 and/or SLQS containing genes	
		Pop.Hits	Benjamini	Pop.Hits	Benjamini
GO:0006357~regulation of transcription from RNA polymerase II promoter	1874	727	3.91E-06	700	1.69E-03
GO:0051173~positive regulation of nitrogen compound metabolic process	1874	644	1.56E-04	613	6.35E-03
GO:0051254~positive regulation of RNA metabolic process	1874	481	1.44E-04	462	6.60E-03
GO:0045892~negative regulation of transcription, DNA-dependent	1874	356	1.77E-04	343	7.10E-03
GO:0045893~positive regulation of transcription, DNA-dependent	1874	477	1.29E-04	459	7.27E-03
GO:0045935~positive regulation of nucleobase, nucleoside, nucleotide and nucleic acid metabolic process	1874	624	1.66E-04	596	8.20E-03
GO:0000122~negative regulation of transcription from RNA polymerase II promoter	1874	266	1.55E-04	259	8.32E-03
GO:0031327~negative regulation of cellular biosynthetic process	1874	561	2.51E-04	534	9.94E-03

Supplementary Table 6. GeneGo process categories enriched with regulatory SLQS.

GeneGo Process	P Value	Ratio	Control 1 P-Value	Control 2 P-Value	Control 3 P-Value	Control 4 P-Value	Control 5 P-Value
negative regulation of transcription from RNA polymerase II promoter	3.30E-21	194/753	1.31E-26	1.85E-14	1.48E-14	2.76E-15	8.03E-13
regulation of transcription, DNA-dependent	8.38E-19	521/3818	2.09E-21	6.23E-15	1.31E-17	5.78E-12	1.86E-15
positive regulation of transcription, DNA-dependent	9.69E-19	189/1383	3.48E-25	1.96E-16	8.85E-10	6.06E-17	9.63E-13
transcription, DNA-dependent	2.13E-17	488/2791	6.11E-19	6.57E-15	1.72E-13	2.65E-11	7.13E-15
negative regulation of cell proliferation	6.90E-14	144/802	4.80E-16	1.09E-14	3.71E-10	1.33E-12	7.75E-10
positive regulation of transcription from RNA polymerase II promoter	4.34E-12	212/1023	1.26E-24	8.93E-20	1.31E-13	4.39E-13	4.09E-16
regulation of transcription from RNA polymerase II promoter	1.42E-11	128/1758	3.44E-13	1.12E-08	9.60E-10	-	8.78E-09
negative regulation of transcription, DNA-dependent	2.68E-11	149/1135	2.89E-20	1.42E-18	6.77E-09	3.87E-13	2.11E-11
in utero embryonic development	8.23E-09	100/555	5.93E-08	-	-	-	-
positive regulation of apoptotic process	3.42E-08	106/726	3.25E-14	3.55E-09	1.61E-07	6.79E-07	6.19E-08
axon guidance	7.20E-08	103/490	1.07E-11	1.15E-13	1.89E-09	2.18E-07	1.54E-09
protein phosphorylation	7.73E-08	154/810	6.48E-07	3.85E-11	7.28E-09	9.64E-07	1.59E-08
phosphorylation	3.47E-07	161/1142	1.35E-08	5.27E-16	6.73E-12	7.06E-10	1.13E-13
learning or memory	9.76E-07	33/288	-	-	-	-	-
response to drug	1.09E-06	138/745	-	3.03E-06	-	-	-
heart development	1.90E-06	72/596	1.84E-14	4.21E-14	-	1.40E-06	1.80E-11
intracellular signal transduction	3.40E-06	92/1821	-	7.74E-07	9.70E-12	-	9.25E-09
neurotrophin TRK receptor signaling pathway	4.20E-06	83/343	2.17E-08	5.32E-11	3.02E-13	1.59E-07	2.20E-10
blood coagulation	5.94E-06	132/665	-	4.28E-07	1.00E-07	-	1.02E-12

Supplementary Table 7. GeneGo process categories enriched on the template and non-template strands of regulatory SLQS.

GeneGo Process	Total	Min(P-Value)	Both Strands P-Value	Both Strands In Data	Non-Template P-Value	Non-Template In Data	Template P-Value	Template In Data
intracellular signal transduction	1821	3.78E-10	3.40E-06	92	3.78E-10	70	1.13E+01	40
apoptotic process	1325	7.40E-06	2.08E-05	175	7.40E-06	112	8.48E-02	94
positive regulation of apoptotic process	726	3.42E-08	3.42E-08	106	1.69E-06	67	6.99E-03	56
in utero embryonic development	555	8.23E-09	8.23E-09	100	1.01E-06	63	3.07E-03	53
negative regulation of transcription from RNA polymerase II promoter	753	3.30E-21	3.30E-21	194	3.62E-09	105	2.10E-17	123
negative regulation of transcription, DNA-dependent	1135	3.22E-12	2.68E-11	149	2.93E-04	80	3.22E-12	100
transcription, DNA-dependent	2791	2.13E-17	2.13E-17	488	1.75E-07	267	3.65E-15	293
positive regulation of gene expression	1522	7.49E-07	4.55E-04	64	5.77E+00	31	7.49E-07	48
regulation of transcription from RNA polymerase II promoter	1758	1.42E-11	1.42E-11	128	1.59E-04	69	4.57E-10	82
positive regulation of transcription, DNA-dependent	1383	9.69E-19	9.69E-19	189	1.94E-10	109	1.98E-14	117
nervous system development	2539	1.50E-06	4.14E-05	123	6.48E-03	72	1.50E-06	82
regulation of transcription, DNA-dependent	3818	8.38E-19	8.38E-19	521	6.71E-10	293	1.02E-12	299
positive regulation of transcription from RNA polymerase II promoter	1023	4.34E-12	4.34E-12	212	4.53E-07	123	1.13E-09	129
protein phosphorylation	810	7.73E-08	7.73E-08	154	9.48E-04	87	4.95E-06	93

Supplementary Table 8. GeneGo process categories enriched with 5'-UTR SLQS.

GeneGo Process	P Value	Ratio	Control 1	Control 2	Control 3	Control 4	Control 5
			P-Value	P-Value	P-Value	P-Value	P-Value
protein phosphorylation	6.91E-13	117/810	2.51E-10	9.30E-10	3.56E-16	6.83E-12	6.30E-08
negative regulation of cell proliferation	1.56E-12	98/802	8.46E-08	5.08E-06	-	-	-
synaptic transmission	1.63E-11	95/826	1.02E-07	2.82E-07	7.07E-08	3.46E-06	1.20E-06
neurotrophin TRK receptor signaling pathway	1.40E-09	65/343	1.20E-12	2.47E-09	5.80E-13	5.11E-14	6.74E-08
heart development	3.66E-09	56/596	-	-	1.25E-06	5.50E-10	1.32E-08
axon guidance	4.82E-09	74/490	2.18E-11	3.28E-07	1.27E-08	1.80E-16	1.84E-10
phosphorylation	1.07E-08	112/1142	6.93E-09	5.92E-06	2.47E-11	1.21E-09	5.62E-07
response to drug	1.42E-08	98/745	-	-	-	-	-
activation of adenylate cyclase activity	4.09E-08	22/57	-	-	-	-	-
fibroblast growth factor receptor signaling pathway	1.20E-07	43/200	3.47E-08	2.28E-07	9.80E-10	1.60E-11	-
learning or memory	1.55E-07	26/288	4.35E-06	-	-	5.13E-06	-
cell cycle	2.90E-07	102/1493	-	-	-	-	-
peptidyl-serine phosphorylation	7.99E-07	27/107	-	9.05E-06	1.55E-06	-	-
angiogenesis	1.21E-06	53/389	-	-	-	-	-
intracellular signal transduction	1.68E-06	64/1821	-	-	-	-	-
negative regulation of cell migration	2.20E-06	27/199	-	-	-	-	-
muscle contraction	2.47E-06	34/287	-	-	-	-	-
energy reserve metabolic process	2.79E-06	35/222	-	-	-	-	6.41E-07
adenylate cyclase-activating G-protein coupled receptor signaling pathway	4.00E-06	22/96	-	-	-	-	-
positive regulation of transcription, DNA-dependent	4.90E-06	100/1383	7.85E-09	5.40E-11	1.56E-15	2.81E-09	7.43E-18
response to estradiol stimulus	6.06E-06	40/210	-	-	-	-	-
epidermal growth factor receptor signaling pathway	6.75E-06	44/232	1.50E-06	-	1.85E-09	2.03E-08	-
positive regulation of MAPK cascade	9.37E-06	29/465	-	-	-	-	-

Supplementary Table 9. GeneGo process categories enriched on the template and non-template strands of 5'-UTR SLQS.

GeneGo Process	Total	Min(P-Value)	Both Strands P-Value	Both Strands In Data	Non-Template P-Value	Non-Template In Data	Template P-Value	Template In Data
adenylate cyclase-modulating G-protein coupled receptor signalling pathway	184	2.07E-07	7.96E-05	22	2.07E-07	19	3.09E+00	9
positive regulation of ERK1 and ERK2 cascade	136	2.03E-06	5.67E-04	28	2.03E-06	23	4.40E+00	12
cell cycle	1,493	2.90E-07	2.90E-07	102	1.55E-06	64	2.89E+00	43
protein phosphorylation	810	6.91E-13	6.91E-13	117	1.76E-01	49	1.27E-12	76
axon guidance	490	1.16E-10	4.82E-09	74	5.48E+00	28	1.16E-10	52
phosphorylation	1,142	5.95E-10	1.07E-08	112	1.13E+01	44	5.95E-10	74
epidermal growth factor receptor signalling pathway	232	2.38E-07	6.75E-06	44	4.27E+00	18	2.38E-07	32
muscle contraction	287	5.27E-08	2.47E-06	34	2.12E-01	16	5.27E-08	26
energy reserve metabolic process	222	1.77E-07	2.79E-06	35	3.86E-01	16	1.77E-07	26
negative regulation of cell migration	199	1.30E-08	2.20E-06	27	2.82E-02	14	1.30E-08	22
angiogenesis	389	1.42E-07	1.21E-06	53	2.25E-01	25	1.42E-07	37
learning or memory	288	2.25E-08	1.55E-07	26	1.78E-02	13	2.25E-08	20
response to drug	745	1.42E-08	1.42E-08	98	5.21E-02	47	1.09E-07	61
negative regulation of cell proliferation	802	1.56E-12	1.56E-12	98	8.30E-05	50	7.64E-10	60
positive regulation of synapse assembly	33	8.07E-06	1.59E-04	13	1.47E-01	7	8.07E-06	11
adenylate cyclase-activating G-protein coupled receptor signalling pathway	96	4.00E-06	4.00E-06	22	1.45E-02	12	5.04E-06	16
heart development	596	3.66E-09	3.66E-09	56	4.23E-03	28	5.62E-06	33

Supplementary Table 10. Selective genes for which stem-loop-containing putative quadruplex sequences (SLQS) overlap with mutation loci.

SLQS ID	Gene Segment	Mutation Location	Variant Classification	Variant Type	Reference Allele	Tumor_Seq_Allele1	Tumor_Seq_Allele2	Mutation Status
G4ST07151491525	*MLL3* Exon 43	chr7:151491555-151491555	Missense Mutation	SNP	T	T	C	Somatic
G4ST19017166594	*MYO9B* Exon 22	chr19:17166612-17166617	In Frame Deletion	Deletion	ACTCTC	-	ACTCTC	Unknown

Supplementary Table 11. Selective genes for which stem-loop-containing putative quadruplex sequences (SLQS) overlap with SNP loci.

SLQS ID	Gene Segment	Regulatory Element	Reference SNP ID	SNP Location	Minor Allele Count	Ancestral Allele	RefSNP Alleles
G4ST19052451699	*CCDC9* Exon 1-Intron 1	Yes	rs2287689	chr19:52451713-52451713	T=0.498/1085	C	A/C
G4ST17054124462	*TEX14* Promoter & *RAD51C* Promoter	Yes	rs302873	chr17:54124480-54124480	C=0.452/984	C	C/G
G4ST03197538875	*TM4SF19* Intron 1	Yes	rs6782336	chr3:197538885-197538885	A=0.355/773	G	A/G
G4ST04077691855	*SHROOM3* Intron 1	-	rs62300892	chr4:77691876-77691876	A=0.225/489	G	A/G
G4ST18009752199	*RAB31* Intron 1	-	rs11663031	chr18:9752204-9752204	C=0.207/451	G	C/G
G4ST01039729840	*BMP8A* Promoter	Yes	rs1907066	chr1:39729855-39729855	A=0.118/257	G	A/G
G4ST19011311393	*RAB3D* Promoter	Yes	rs34938145	chr19:11311410-11311410	A=0.068/148	A	A/G

Supplementary Table 12. Additional DNA oligonucleotides used for NMR study.

SLQS ID	Sequence[a,b]	Gene Symbol
G4ST01000752784	5′-GA **GGGG** C **GGG** GCGCGTACGTGCA **GGG** A **GGGG** A-3′	*NCRNA00115 | LOC643837*
G4ST01006208469	5′-T **GGG** CACAGCGTG **GGG** A **GGG** CAGGAGACTG **GGG** A-3′	*ICMT*
G4ST03134602145	5′-T **GGG** A **GGG** GCTGCTGAAGGCAGC **GGG** TA **GGG**-3′	*BFSP2*
G4ST03187025840	5′-A **GGG** GCGCAGCGGCGC **GGGG** C **GGGG** A **GGG**-3′	*IGF2BP2*
G4ST11075769784	5′-GT **GGG** AAGGAGGCGGCAGCGTCTC **GGG** C **GGG** C **GGG** A-3′	*PRKRIR*
G4ST17076467975	5′-TA **GGG** TGCTTGGAAAGCG **GGG** AGA **GGG** A **GGG** T-3′	*RPTOR*
G4ST18008599361	5′-GA **GGG** GCCGGAGGC **GGG** C **GGG** T **GGG** C-3′	*RAB12*
G4ST19011311393	5′-TT **GGG** C **GGG** GCTTTCAGAGAGC **GGG** T **GGG** A-3′	*RAB3D*
G4ST23054492800	5′-GA **GGG** C **GGG** GACTCTTACTGGAGAGTT **GGG** T **GGGG** T-3′	*FGD1*
G4ST24019614192	5′-AA **GGG** CTTC **GGGG** A **GGG** CGCGGCCGCG **GGG** C-3′	*CD24*
G4ST07001717478_G4	5′-CA **GGG** T **GGG** TTTTTTTTTTTT **GGG** T **GGG** T-3′	
G4ST07001717478_dx	5′-TCTGCTGTGCAG-3′	

[a]G-tracts are in boldface. [b]Self-complementary tracts are underlined.

Supplementary Table 13. Site-specific labelled DNA sequences used for the *MYT1L* gene sequence G4ST02001786748[a,b,c,d].

Type	Sequence
^{15}N-labeled	5′-A ***GGG** AGAGGAGAGCTCT **GGG** TT **GGG** T **GGG**-3′
	5′-A **GGG** AGAGGAGAGCTCT **GG*G** TT **GGG** T **GGG**-3′
	5′-A **GGG** AGAGGAGAGCTCT **GGG** TT ***GGG** T **GGG**-3′
	5′-A **GGG** AGAGGAGAGCTCT **GGG** TT **GGG** T ***GGG**-3′
^{2}H-labeled	5′-A **G#GG** AGAGGAGAGCTCT **GGG** TT **GGG** T **GGG**-3′
	5′-A **GGG** AGAGGAGAGCTCT **G#GG** TT **GGG** T **GGG**-3′
	5′-A **GGG** AGAGGAGAGCTCT **GGG** TT **G#GG** T **GGG**-3′
	5′-A **GGG** AGAGGAGAGCTCT **GGG** TT **GGG** T **G#GG**-3′

[a]Individual nucleotides 2%-^{15}N-labeled are marked by asterisks (*G). [b]Individual nucleotides 2%-^{2}H-labeled at the H8 position are marked by hash signs (#G). [c]G-tracts are in boldface. [d]Self-complementary tracts are underlined.

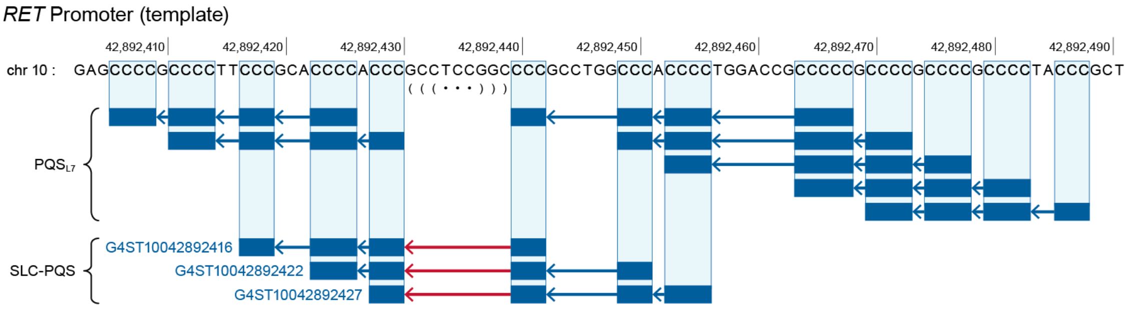

Supplementary Figure 1. Overlap of PQS_{L7} with SLQS extends the range of the putative quadruplex-forming regions. Shown here is the promoter region of the *RET* gene, which contains two stretches of PQS_{L7} separated by a duplex stem-loop motif (constituting several overlapping SLQS motifs). The strand placement, ID, and mapping (on a single-nucleotide scale) of the SLQS are displayed. G-tracts are shown as blue rectangles, short loops (≤7 nt) are shown as blue lines, while long loops capable of forming stable duplex stem-loops are shown as red lines. The predicted duplex stem-loops are outlined in dot-bracket notation; nucleotides involved in base pair formation are nested by matching pairs of brackets, whereas nucleotides not involved in base pair formation are marked by a dot. The PQS_{L7} and SLQS are located on the complementary strand of the reference genome set (*hg18*), corresponding to C-tracts in the reference genome.

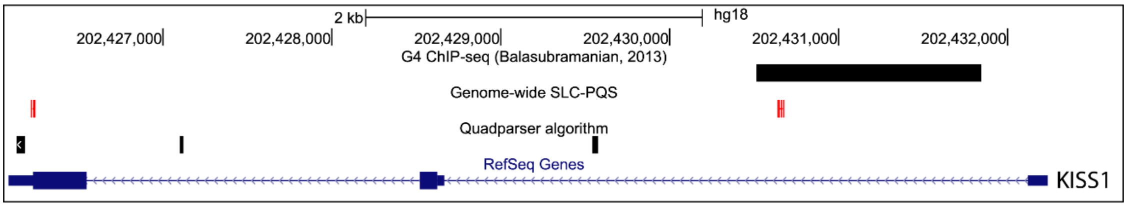

Supplementary Figure 2. Mapping of SLQS onto experimentally determined G-quadruplex-forming regions. Shown here is the segment of Intron 1 of *KISS1* gene where an SLQS motif (G4ST01202430640) is mapped onto an experimentally determined G-quadruplex-forming region (from human breast adenocarcinoma cells (2)) which is devoid of classical PQS (3,4).

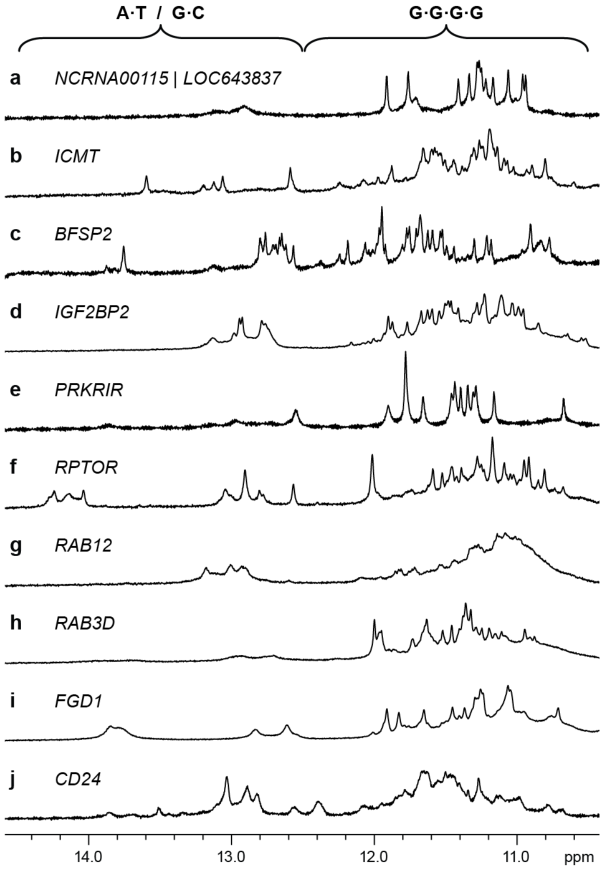

Supplementary Figure 3. Additional 1D imino proton NMR spectra of SLQS identified across various genes in the human genome. Gene names for which the respective SLQS have been located within are indicated. Typical chemical shift ranges for imino protons participating in the formation of Watson-Crick base-pairs (A•T/G•C) and G-tetrads (G•G•G•G) are demarcated. (**a**) G4ST01000752784, (**b**) G4ST01006208469, (**c**) G4ST03134602145, (**d**) G4ST03187025840, (**e**) G4ST11075769784, (**f**) G4ST17076467975, (**g**) G4ST18008599361, (**h**) G4ST19011311393, (**i**) G4ST23054492800, and (**j**) G4ST24019614192.

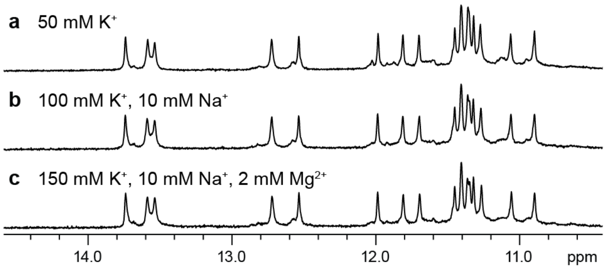

Supplementary Figure 4. 1D imino proton NMR spectra of G4ST02001786748 under different cation types and concentrations. (**a**) 50 mM K^+, (**b**) 100 mM K^+ and 10 mM Na^+, and (**c**) near-physiological ionic condition (150 mM K^+, 10 mM Na^+, and 2 mM Mg^{2+}).

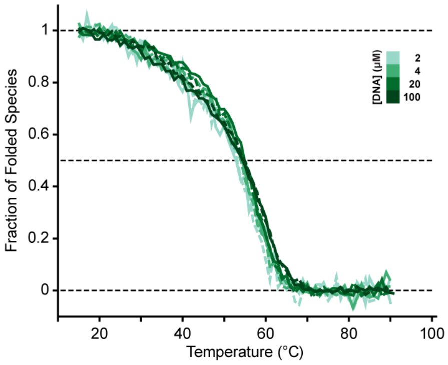

Supplementary Figure 5. Concentration-independent melting profile of the *MYT1L* gene sequence G4ST02001786748. Fractions of folded G-quadruplexes as a function of temperature at different concentrations of DNA strands (2-100 µM; color-coded on the top right corner), as monitored through the CD spectra at 295 nm.

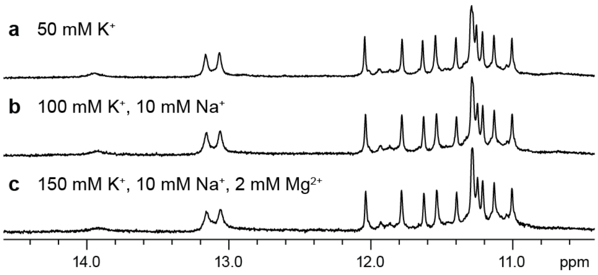

Supplementary Figure 6. 1D imino proton NMR spectra of G4ST07001717478 under different cation types and concentrations. (**a**) 50 mM K^+, (**b**) 100 mM K^+ and 10 mM Na^+, and (**c**) near-physiological ionic condition (150 mM K^+, 10 mM Na^+, and 2 mM Mg^{2+}).

REFERENCES

1. Huang, D.W., Sherman, B.T. and Lempicki, R.A. (2009) Systematic and integrative analysis of large gene lists using DAVID bioinformatics resources. *Nat. Protoc.*, **4**, 44–57.
2. Lam, E.Y.N., Beraldi, D., Tannahill, D. and Balasubramanian, S. (2013) G-quadruplex structures are stable and detectable in human genomic DNA. *Nat. Commun.*, **4**, 1796.
3. Todd, A.K., Johnston, M. and Neidle, S. (2005) Highly prevalent putative quadruplex sequence motifs in human DNA. *Nucleic Acids Res.*, **33**, 2901–2907.
4. Huppert, J.L. and Balasubramanian, S. (2005) Prevalence of quadruplexes in the human genome. *Nucleic Acids Res.*, **33**, 2908–2916.
