# Supplementary material for: Duplex stem-loop-containing quadruplex motifs in the human genome: a combined genomic and structural study
Source: Nucleic Acids Res. 2015 May 9;43(11):5630–46. doi: 10.1093/nar/gkv355 (PMC4477648; doi:10.1093/nar/gkv355)
Supplement: SUPPLEMENTARY DATA [file supp_gkv355_nar-00300-f-2015-File003.pdf]

Supplementary dataset 1

SLC-PQS ID(s)	SLC-PQS location	SLC-PQS strand	SLC-PQS sequence/DNA structure in Dot-bracket format	Gene Symbol	RefSeq ID(s)	Gene location	SLC-PQS Placement	SLC-PQS distance to TSS	Gene Segment(s)	Promoter	Genic	Exon	Intron	CDS	5'UTR	3'UTR	Chromatin accessibility region	Transcription factor binding region	Polymerase II biding region	PQS overlapped
G4ST01012419037	chr1:12419038-12419075	+	GGGAGCCTGGGGTGTGCAGCAGCAGGACTAGGGAGGGG<br>((.(((....)))..))..	VPS13D	NM_015378	chr1:12212700-12494685	non-template	38	Intron_65	--	Yes	--	Yes	--	--	--	Yes	Yes	Yes	--
G4ST01012419037	chr1:12419038-12419075	+	GGGAGCCTGGGGTGTGCAGCAGCAGGACTAGGGAGGGG<br>((.(((....)))..))..	VPS13D	NM_018156	chr1:12212700-12494685	non-template	38	Intron_64	--	Yes	--	Yes	--	--	--	Yes	Yes	Yes	--
G4ST01051544050	chr1:51544051-51544082	-	GGGGCTCACAGCTGGGGTGGGGGTGGTGTGGG<br>(((...)))..	TTC39A	NM_001080494	chr1:51525518-51560526	non-template	32	Intron_7	--	Yes	--	Yes	--	--	--	--	--	--	--
G4ST01051544050	chr1:51544051-51544082	-	GGGGCTCACAGCTGGGGTGGGGGTGGTGTGGG<br>(((...)))..	TTC39A	NM_001144832	chr1:51525518-51583373	non-template	32	Intron_7	--	Yes	--	Yes	--	--	--	--	--	--	--
G4ST01053755875	chr1:53755876-53755913	+	GGGCCTGGCAGATAAGCTCCCAGGGGTTGGGTGGGGGG<br>.((((.((.....)).))))	GLIS1	NM_147193	chr1:53744494-53972465	template	38	Intron_6	--	Yes	--	Yes	--	--	--	--	--	--	--
G4ST01110378974	chr1:110378975-110379013	+	GGGGGGCGAGGCTCGCACCGGGTCGGGCGGCGCCGGGGG<br>..((((...))))....	FAM40A	NM_033088	chr1:110378764-110398786	non-template	39	Intron_1.f	--	Yes	--	Yes	--	--	--	--	Yes	Yes	Yes
G4ST01000005796	chr1:5797-5836	+	GGGAGCTCCCTGGACTGGAGCCGGGAGGTGGGGAACAGGG<br>.(((((.......))))).	LOC100288778	NR_028269	chr1:4225-7502	template	40	Intron_4-Exon_5	--	Yes	Yes	Yes	--	--	--	--	--	--	--
G4ST01000005796	chr1:5797-5836	+	GGGAGCTCCCTGGACTGGAGCCGGGAGGTGGGGAACAGGG<br>.(((((.......))))).	WASH7P	NR_024540	chr1:4225-19233	template	40	Intron_8-Exon_9	--	Yes	Yes	Yes	--	--	--	--	--	--	--
G4ST01000008039	chr1:8040-8080	+	GGGCAGCAGGGCAGAGACTGGGCAGCCTCAGAGGCACGGGG<br>.((...)) ..((((...))))...	LOC100288778	NR_028269	chr1:4225-7502	template	-537	upstream	Yes	--	--	--	--	--	--	--	Yes	--	--
G4ST01000008039	chr1:8040-8080	+	GGGCAGCAGGGCAGAGACTGGGCAGCCTCAGAGGCACGGGG<br>.((...)) ..((((...))))...	WASH7P	NR_024540	chr1:4225-19233	template	41	Intron_3	--	Yes	--	Yes	--	--	--	--	Yes	--	--
G4ST01000008047	chr1:8048-8090	+	GGGCAGAGACTGGGCAGCCTCAGAGGCACGGGGAAATGGAGGG<br>.((...)) ..((((...))))...	LOC100288778	NR_028269	chr1:4225-7502	template	-545	upstream	Yes	--	--	--	--	--	--	--	Yes	--	--
G4ST01000008047	chr1:8048-8090	+	GGGCAGAGACTGGGCAGCCTCAGAGGCACGGGGAAATGGAGGG<br>.((...)) ..((((...))))...	WASH7P	NR_024540	chr1:4225-19233	template	43	Intron_3	--	Yes	--	Yes	--	--	--	--	Yes	--	--
G4ST01000018871	chr1:18872-18906	-	GGGAAGCCAAGGCTTGTGGGGCGCAGGGACCCGGG<br>((((....))))....	WASH7P	NR_024540	chr1:4225-19233	non-template	35	Intron_1.f	--	Yes	--	Yes	--	--	--	--	--	--	Yes
G4ST01000317496	chr1:317497-317534	-	GGGGAGCTTGGCTTAGGGAAGTTGTGGGCCTACCAGGG<br>.(((...)))..	LOC100132287	NR_028322	chr1:313755-318444	template	38	Exon_3.l	--	Yes	Yes	--	--	--	--	--	--	--	Yes
G4ST01000317496	chr1:317497-317534	-	GGGGAGCTTGGCTTAGGGAAGTTGTGGGCCTACCAGGG<br>.(((...)))..	LOC100132062	NR_028325	chr1:313755-318444	template	38	Exon_3.l	--	Yes	Yes	--	--	--	--	--	--	--	Yes
G4ST01000317496	chr1:317497-317534	-	GGGGAGCTTGGCTTAGGGAAGTTGTGGGCCTACCAGGG<br>.(((...)))..	LOC100133331	NR_028327	chr1:313755-318444	template	38	Exon_4.l	--	Yes	Yes	--	--	--	--	--	--	--	Yes
G4ST01000317506	chr1:317507-317540	-	GGGCCTGGGGAGCTTGGCTTAGGGAAGTTGTGGG<br>.(((...)))..	LOC100132287	NR_028322	chr1:313755-318444	template	34	Exon_3.l	--	Yes	Yes	--	--	--	--	--	--	--	Yes
G4ST01000317506	chr1:317507-317540	-	GGGCCTGGGGAGCTTGGCTTAGGGAAGTTGTGGG<br>.(((...)))..	LOC100132062	NR_028325	chr1:313755-318444	template	34	Exon_3.l	--	Yes	Yes	--	--	--	--	--	--	--	Yes
G4ST01000317506	chr1:317507-317540	-	GGGCCTGGGGAGCTTGGCTTAGGGAAGTTGTGGG<br>.(((...)))..	LOC100133331	NR_028327	chr1:313755-318444	template	34	Exon_4.l	--	Yes	Yes	--	--	--	--	--	--	--	Yes
G4ST01000651905	chr1:651906-651939	+	GGGCCTGGGGAGCTTGGCTTAGGGAAGTTGTGGG<br>.(((...)))..	LOC100133331	NR_028327	chr1:651002-655594	template	34	Exon_3.l	--	Yes	Yes	--	--	--	--	--	--	--	Yes
G4ST01000651911	chr1:651912-651949	+	GGGGAGCTTGGCTTAGGGAAGTTGTGGGCCTACCAGGG<br>.(((...)))..	LOC100133331	NR_028327	chr1:651002-655594	template	38	Exon_3.l	--	Yes	Yes	--	--	--	--	--	--	--	Yes
G4ST01000752784	chr1:752785-752813	+	GGGGCGGGGCGCGTACGTGCAGGGAGGGG<br>((((....)))).	NCRNA00115	NR_024321	chr1:751449-752765	template	-19	upstream	Yes	--	--	--	--	--	--	Yes	Yes	Yes	--
G4ST01000752784	chr1:752785-752813	+	GGGGCGGGGCGCGTACGTGCAGGGAGGGG<br>((((....)))).	LOC643837	NR_015368	chr1:752927-779603	non-template	-113	upstream	Yes	--	--	--	--	--	--	Yes	Yes	Yes	--
G4ST01000855088	chr1:855089-855115	+	GGGGCAGGGGAGGGCTGAGCCAGTGGG<br>(((...))).	SAMD11	NM_152486	chr1:850984-869824	non-template	27	Intron_2	--	Yes	--	Yes	--	--	--	--	--	--	Yes
G4ST01000862820	chr1:862821-862846	-	GGGAGGGGGTGGGCCAAGGCTGGGGG<br>(((....)))	SAMD11	NM_152486	chr1:850984-869824	template	26	Intron_5	--	Yes	--	Yes	--	--	--	--	--	--	Yes
G4ST01000863865	chr1:863866-863900	-	GGGCGGGCTGTGCAGGGTCACATGCCAGCGTGGGG<br>...((((....))))	SAMD11	NM_152486	chr1:850984-869824	template	35	Intron_5	--	Yes	--	Yes	--	--	--	Yes	Yes	Yes	--
G4ST01000865241	chr1:865242-865275	-	GGGGCCTGGGAGGCTGAGTGCAGCGGGCAGGGGG<br>..((((....))))	SAMD11	NM_152486	chr1:850984-869824	template	34	Intron_7	--	Yes	--	Yes	--	--	--	Yes	--	--	--
G4ST01000865248	chr1:865249-865283	-	GGGCGGCTGGGGCCTGGGAGGCTGAGTGCAGCGGG<br>..((((....))))	SAMD11	NM_152486	chr1:850984-869824	template	35	Intron_7	--	Yes	--	Yes	--	--	--	Yes	--	--	--
G4ST01000867739	chr1:867740-867769	+	GGGGAGGCGGGCGGGGCCGCGCGGCCCGGG<br>(((....)))..	SAMD11	NM_152486	chr1:850984-869824	non-template	30	Intron_10	--	Yes	--	Yes	--	--	--	Yes	Yes	Yes	--
G4ST01000867967	chr1:867968-868000	-	GGGGGGTCCGGAGCCCGGGGGCCCCTGGGGGGG<br>....((((...))))	SAMD11	NM_152486	chr1:850984-869824	template	33	Exon_11	--	Yes	Yes	--	Yes	--	--	Yes	Yes	Yes	Yes
G4ST01000867971	chr1:867972-868005	-	GGGGTGGGGGGTCCGGAGCCCGGGGGCCCCTGGG<br>....((((...))))	SAMD11	NM_152486	chr1:850984-869824	template	34	Exon_11	--	Yes	Yes	--	Yes	--	--	Yes	Yes	Yes	Yes
G4ST01000867979	chr1:867980-868010	-	GGGACGGGGTGGGGGGTCCGGAGCCCGGGGG<br>....((((...))))	SAMD11	NM_152486	chr1:850984-869824	template	31	Exon_11	--	Yes	Yes	--	Yes	--	--	Yes	Yes	Yes	Yes
G4ST01000868618	chr1:868619-868646	+	GGGTAAGGGGGGGCCCCAGTTCCTGGGG<br>...(((...)))	SAMD11	NM_152486	chr1:850984-869824	non-template	28	Exon_12-Intron_12	--	Yes	Yes	Yes	Yes	--	--	Yes	Yes	Yes	--
G4ST01000868624	chr1:868625-868651	+	GGGGGGGCCCCAGTTCCTGGGGCGGGG<br>...(((...)))	SAMD11	NM_152486	chr1:850984-869824	non-template	27	Intron_12	--	Yes	--	Yes	--	--	--	Yes	Yes	Yes	--
G4ST01000873701	chr1:873702-873734	-	GGGTGAGGCTGGCCTCCTGGGGAGGGGCCTGGG<br>.((((....))))....	NOC2L	NM_015658	chr1:869446-884542	non-template	33	Exon_13-Intron_13	--	Yes	Yes	Yes	Yes	--	--	--	--	--	--
G4ST01000874031	chr1:874032-874068	-	GGGGACACGCCAGGCTGTGTGGGGCCATGGGGCTGGG<br>.((((((...)).)))).	NOC2L	NM_015658	chr1:869446-884542	non-template	37	Intron_12	--	Yes	--	Yes	--	--	--	--	--	--	--
G4ST01000877535	chr1:877536-877570	+	GGGTGCTGGGCTGTCTCCAGACAGGGGCTTCGGGG<br>(((((....)))))	NOC2L	NM_015658	chr1:869446-884542	template	35	Intron_10	--	Yes	--	Yes	--	--	--	--	--	--	--
G4ST01000879577	chr1:879578-879614	-	GGGAGTTGGAGGCTGGGACTTCCGGGGACTCTTAGGG<br>(((.....)))	NOC2L	NM_015658	chr1:869446-884542	non-template	37	Intron_6	--	Yes	--	Yes	--	--	--	--	--	--	--
G4ST01000885312	chr1:885313-885345	+	GGGGGTCGGGGGAGCGGGGATGGCTTCCGGGGG<br>..(((...))).	NOC2L	NM_015658	chr1:869446-884542	template	-770	upstream	Yes	--	--	--	--	--	--	--	--	--	--
G4ST01000885312	chr1:885313-885345	+	GGGGGTCGGGGGAGCGGGGATGGCTTCCGGGGG<br>..(((...))).	KLHL17	NM_198317	chr1:885830-890962	non-template	-484	upstream	Yes	--	--	--	--	--	--	--	--	--	--
G4ST01000885319	chr1:885320-885355	+	GGGGGAGCGGGGATGGCTTCCGGGGGAGGAGGAGGG<br>..(((...))).	NOC2L	NM_015658	chr1:869446-884542	template	-777	upstream	Yes	--	--	--	--	--	--	--	--	--	--
G4ST01000885319	chr1:885320-885355	+	GGGGGAGCGGGGATGGCTTCCGGGGGAGGAGGAGGG<br>..(((...))).	KLHL17	NM_198317	chr1:885830-890962	non-template	-474	upstream	Yes	--	--	--	--	--	--	--	--	--	--
G4ST01000889088	chr1:889089-889113	+	GGGGAGGGCAGGTCCTGGGGCCGGG<br>(((...)))	KLHL17	NM_198317	chr1:885830-890962	non-template	25	Intron_8	--	Yes	--	Yes	--	--	--	--	--	--	--
G4ST01000892260	chr1:892261-892283	+	GGGAGAGAGCTGGGCGGGGCGGG<br>((....))	PLEKHN1	NM_001160184	chr1:891740-900347	non-template	23	Intron_2	--	Yes	--	Yes	--	--	--	Yes	Yes	Yes	Yes
G4ST01000892260	chr1:892261-892283	+	GGGAGAGAGCTGGGCGGGGCGGG<br>((....))	PLEKHN1	NM_032129	chr1:891740-900347	non-template	23	Intron_2	--	Yes	--	Yes	--	--	--	Yes	Yes	Yes	Yes
G4ST01000893183	chr1:893184-893213	+	GGGAAATGGGGCTGGGGGAGGAGCTCTGGG<br>.(((...))).	PLEKHN1	NM_001160184	chr1:891740-900347	non-template	30	Intron_2	--	Yes	--	Yes	--	--	--	--	Yes	--	--

G4ST01000893183	chr1:893184-893213	+	GGGAAATGGGGCTGGGGGAGGAGCTCTGGG<br>.(((...))).	PLEKHN1	NM_032129	chr1:891740-900347	non-template	30	Intron_2	--	Yes	--	Yes	--	--	--	--	Yes	--	--
G4ST01000896819	chr1:896820-896846	-	GGGTGAGAACCTCAGGGCGGGCAGGGG<br>.(((...))).	PLEKHN1	NM_001160184	chr1:891740-900347	template	27	Intron_8	--	Yes	--	Yes	--	--	--	--	--	--	--
G4ST01000896819	chr1:896820-896846	-	GGGTGAGAACCTCAGGGCGGGCAGGGG<br>.(((...))).	PLEKHN1	NM_032129	chr1:891740-900347	template	27	Intron_8	--	Yes	--	Yes	--	--	--	--	--	--	--
G4ST01000900522	chr1:900523-900553	-	GGGGGCTGCCTGGCTGGGTGCTGGGTGGGGG<br>.(((....))).	C1orf170	NR_027693	chr1:900442-907336	non-template	31	Exon_5.l	--	Yes	Yes	--	--	--	--	--	--	--	--
G4ST01000901945	chr1:901946-901979	-	GGGAGGCGGTTCCCGGGGTTGGTGGGGGTGGGGG<br>...(((...)))	C1orf170	NR_027693	chr1:900442-907336	non-template	34	Intron_3	--	Yes	--	Yes	--	--	--	--	--	--	--
G4ST01000901951	chr1:901952-901988	-	GGGGGGAGTGGGAGGCGGTTCCCGGGGTTGGTGGGGG<br>...(((...)))	C1orf170	NR_027693	chr1:900442-907336	non-template	37	Intron_3	--	Yes	--	Yes	--	--	--	--	--	--	--
G4ST01000901961	chr1:901962-901997	-	GGGGTTGGCGGGGGGAGTGGGAGGCGGTTCCCGGGG<br>...(((...)))	C1orf170	NR_027693	chr1:900442-907336	non-template	36	Intron_3	--	Yes	--	Yes	--	--	--	--	--	--	--
G4ST01000901976	chr1:901977-902011	-	GGGAGGCGGTTCCCGGGGTTGGCGGGGGGAGTGGG<br>...(((...)))	C1orf170	NR_027693	chr1:900442-907336	non-template	35	Intron_3	--	Yes	--	Yes	--	--	--	--	--	--	--
G4ST01000901982	chr1:901983-902020	-	GGGGGGAGTGGGAGGCGGTTCCCGGGGTTGGCGGGGGG<br>...(((...)))	C1orf170	NR_027693	chr1:900442-907336	non-template	38	Intron_3	--	Yes	--	Yes	--	--	--	--	--	--	--
G4ST01000901993	chr1:901994-902029	-	GGGGTTGGCGGGGGGAGTGGGAGGCGGTTCCCGGGG<br>...(((...)))	C1orf170	NR_027693	chr1:900442-907336	non-template	36	Intron_3	--	Yes	--	Yes	--	--	--	--	--	--	--
G4ST01000902008	chr1:902009-902043	-	GGGAGGCGGTTCCCGGGGTTGGCGGGGGGAGTGGG<br>...(((...)))	C1orf170	NR_027693	chr1:900442-907336	non-template	35	Intron_3	--	Yes	--	Yes	--	--	--	--	--	--	--
G4ST01000902014	chr1:902015-902052	-	GGGGGGAGTGGGAGGCGGTTCCCGGGGTTGGCGGGGGG<br>...(((...)))	C1orf170	NR_027693	chr1:900442-907336	non-template	38	Intron_3	--	Yes	--	Yes	--	--	--	--	--	--	--
G4ST01000902025	chr1:902026-902061	-	GGGGTTGGCGGGGGGAGTGGGAGGCGGTTCCCGGGG<br>...(((...)))	C1orf170	NR_027693	chr1:900442-907336	non-template	36	Intron_3	--	Yes	--	Yes	--	--	--	--	--	--	--
G4ST01000902040	chr1:902041-902075	-	GGGAGGCGGTTCCCGGGGTTGGCGGGGGGAGTGGG<br>...(((...)))	C1orf170	NR_027693	chr1:900442-907336	non-template	35	Intron_3	--	Yes	--	Yes	--	--	--	--	--	--	--
G4ST01000902046	chr1:902047-902084	-	GGGGGGAGTGGGAGGCGGTTCCCGGGGTTGGCGGGGGG<br>...(((...)))	C1orf170	NR_027693	chr1:900442-907336	non-template	38	Intron_3	--	Yes	--	Yes	--	--	--	--	--	--	--
G4ST01000902057	chr1:902058-902093	-	GGGGTTGGCGGGGGGAGTGGGAGGCGGTTCCCGGGG<br>...(((...)))	C1orf170	NR_027693	chr1:900442-907336	non-template	36	Intron_3	--	Yes	--	Yes	--	--	--	--	--	--	--
G4ST01000902072	chr1:902073-902107	-	GGGAGGCGGTTCCCGGGGTTGGCGGGGGGAGTGGG<br>...(((...)))	C1orf170	NR_027693	chr1:900442-907336	non-template	35	Intron_3	--	Yes	--	Yes	--	--	--	--	--	--	--
G4ST01000902078	chr1:902079-902116	-	GGGGGGAGTGGGAGGCGGTTCCCGGGGTTGGCGGGGGG<br>...(((...)))	C1orf170	NR_027693	chr1:900442-907336	non-template	38	Intron_3	--	Yes	--	Yes	--	--	--	--	--	--	--
G4ST01000902089	chr1:902090-902125	-	GGGGTTGGCGGGGGGAGTGGGAGGCGGTTCCCGGGG<br>...(((...)))	C1orf170	NR_027693	chr1:900442-907336	non-template	36	Intron_3	--	Yes	--	Yes	--	--	--	--	--	--	--
G4ST01000902104	chr1:902105-902139	-	GGGAGGCGGTTCCCGGGGTTGGCGGGGGGAGTGGG<br>...(((...)))	C1orf170	NR_027693	chr1:900442-907336	non-template	35	Intron_3	--	Yes	--	Yes	--	--	--	--	--	--	--
G4ST01000902110	chr1:902111-902148	-	GGGGGGAGTGGGAGGCGGTTCCCGGGGTTGGCGGGGGG<br>...(((...)))	C1orf170	NR_027693	chr1:900442-907336	non-template	38	Intron_3	--	Yes	--	Yes	--	--	--	--	--	--	--
G4ST01000902121	chr1:902122-902157	-	GGGGTTGGCGGGGGGAGTGGGAGGCGGTTCCCGGGG<br>...(((...)))	C1orf170	NR_027693	chr1:900442-907336	non-template	36	Intron_3	--	Yes	--	Yes	--	--	--	--	--	--	--
G4ST01000902136	chr1:902137-902171	-	GGGAGGCGGTTCCCGGGGTTGGCGGGGGGAGTGGG<br>...(((...)))	C1orf170	NR_027693	chr1:900442-907336	non-template	35	Intron_3	--	Yes	--	Yes	--	--	--	--	--	--	--
G4ST01000902142	chr1:902143-902180	-	GGGGGGAGTGGGAGGCGGTTCCCGGGGTTGGCGGGGGG<br>...(((...)))	C1orf170	NR_027693	chr1:900442-907336	non-template	38	Intron_3	--	Yes	--	Yes	--	--	--	--	--	--	--
G4ST01000902153	chr1:902154-902189	-	GGGGTTGGCGGGGGGAGTGGGAGGCGGTTCCCGGGG<br>...(((...)))	C1orf170	NR_027693	chr1:900442-907336	non-template	36	Intron_3	--	Yes	--	Yes	--	--	--	--	--	--	--
G4ST01000902168	chr1:902169-902203	-	GGGAGGCGGTTCCCGGGGTTGGCGGGGGGAGTGGG<br>...(((...)))	C1orf170	NR_027693	chr1:900442-907336	non-template	35	Intron_3	--	Yes	--	Yes	--	--	--	--	--	--	--
G4ST01000902174	chr1:902175-902212	-	GGGGGGAGTGGGAGGCGGTTCCCGGGGTTGGCGGGGGG<br>...(((...)))	C1orf170	NR_027693	chr1:900442-907336	non-template	38	Intron_3	--	Yes	--	Yes	--	--	--	--	--	--	--
G4ST01000902185	chr1:902186-902220	-	GGGGTTGCGGGGGGAGTGGGAGGCGGTTCCCGGGG<br>...(((...)))	C1orf170	NR_027693	chr1:900442-907336	non-template	35	Intron_3	--	Yes	--	Yes	--	--	--	--	--	--	--
G4ST01000902200	chr1:902201-902234	-	GGGAGGCGGTTCCCGGGGTTGCGGGGGGAGTGGG<br>...(((...)))	C1orf170	NR_027693	chr1:900442-907336	non-template	34	Intron_3	--	Yes	--	Yes	--	--	--	--	--	--	--
G4ST01000902206	chr1:902207-902243	-	GGGGGGAGTGGGAGGCGGTTCCCGGGGTTGCGGGGGG<br>...(((...)))	C1orf170	NR_027693	chr1:900442-907336	non-template	37	Intron_3	--	Yes	--	Yes	--	--	--	--	--	--	--
G4ST01000902216	chr1:902217-902251	-	GGGGTTGCGGGGGGAGTGGGAGGCGGTTCCCGGGG<br>...(((...)))	C1orf170	NR_027693	chr1:900442-907336	non-template	35	Intron_3	--	Yes	--	Yes	--	--	--	--	--	--	--
G4ST01000902231	chr1:902232-902265	-	GGGAGGCGGTTCCCGGGGTTGCGGGGGGAGTGGG<br>...(((...)))	C1orf170	NR_027693	chr1:900442-907336	non-template	34	Intron_3	--	Yes	--	Yes	--	--	--	--	--	--	--
G4ST01000902237	chr1:902238-902274	-	GGGGGGAGCGGGAGGCGGTTCCCGGGGTTGCGGGGGG<br>...(((...)))	C1orf170	NR_027693	chr1:900442-907336	non-template	37	Intron_3	--	Yes	--	Yes	--	--	--	--	--	--	--
G4ST01000902247	chr1:902248-902283	-	GGGGTTGGTGGGGGGAGCGGGAGGCGGTTCCCGGGG<br>...(((...)))	C1orf170	NR_027693	chr1:900442-907336	non-template	36	Intron_3	--	Yes	--	Yes	--	--	--	--	--	--	--
G4ST01000902262	chr1:902263-902297	-	GGGAGGCGGTTCCCGGGGTTGGTGGGGGGAGCGGG<br>...(((...)))	C1orf170	NR_027693	chr1:900442-907336	non-template	35	Intron_3	--	Yes	--	Yes	--	--	--	--	--	--	--
G4ST01000902268	chr1:902269-902306	-	GGGGGGAGCGGGAGGCGGTTCCCGGGGTTGGTGGGGGG<br>...(((...)))	C1orf170	NR_027693	chr1:900442-907336	non-template	38	Intron_3	--	Yes	--	Yes	--	--	--	--	--	--	--
G4ST01000902279	chr1:902280-902315	-	GGGGTTGGTGGGGGGAGCGGGAGGCGGTTCCCGGGG<br>...(((...)))	C1orf170	NR_027693	chr1:900442-907336	non-template	36	Intron_3	--	Yes	--	Yes	--	--	--	--	--	--	--
G4ST01000902294	chr1:902295-902329	-	GGGAGGCGGTTCCCGGGGTTGGTGGGGGGAGCGGG<br>...(((...)))	C1orf170	NR_027693	chr1:900442-907336	non-template	35	Intron_3	--	Yes	--	Yes	--	--	--	--	--	--	--
G4ST01000902300	chr1:902301-902338	-	GGGGGGAGCGGGAGGCGGTTCCCGGGGTTGGTGGGGGG<br>...(((...)))	C1orf170	NR_027693	chr1:900442-907336	non-template	38	Intron_3	--	Yes	--	Yes	--	--	--	--	--	--	--
G4ST01000902311	chr1:902312-902346	-	GGGGTTGCGGGGGGAGCGGGAGGCGGTTCCCGGGG<br>...(((...)))	C1orf170	NR_027693	chr1:900442-907336	non-template	35	Intron_3	--	Yes	--	Yes	--	--	--	--	--	--	--
G4ST01000902326	chr1:902327-902360	-	GGGAGGCGGTTCCCGGGGTTGCGGGGGGAGCGGG<br>...(((...)))	C1orf170	NR_027693	chr1:900442-907336	non-template	34	Intron_3	--	Yes	--	Yes	--	--	--	--	--	--	--
G4ST01000902332	chr1:902333-902369	-	GGGGGGAGCGGGAGGCGGTTCCCGGGGTTGCGGGGGG<br>...(((...)))	C1orf170	NR_027693	chr1:900442-907336	non-template	37	Intron_3	--	Yes	--	Yes	--	--	--	--	--	--	--
G4ST01000902342	chr1:902343-902378	-	GGGGTTGGTGGGGGGAGCGGGAGGCGGTTCCCGGGG<br>...(((...)))	C1orf170	NR_027693	chr1:900442-907336	non-template	36	Intron_3	--	Yes	--	Yes	--	--	--	--	--	--	--
G4ST01000902357	chr1:902358-902392	-	GGGAGGCGGTTCCCGGGGTTGGTGGGGGGAGCGGG<br>...(((...)))	C1orf170	NR_027693	chr1:900442-907336	non-template	35	Intron_3	--	Yes	--	Yes	--	--	--	--	--	--	--
G4ST01000902363	chr1:902364-902401	-	GGGGGGAGCGGGAGGCGGTTCCCGGGGTTGGTGGGGGG<br>...(((...)))	C1orf170	NR_027693	chr1:900442-907336	non-template	38	Intron_3	--	Yes	--	Yes	--	--	--	--	--	--	--
G4ST01000902374	chr1:902375-902410	-	GGGGTTGGTGGGGGGAGCGGGAGGCGGTTCCCGGGG<br>...(((...)))	C1orf170	NR_027693	chr1:900442-907336	non-template	36	Intron_3	--	Yes	--	Yes	--	--	--	--	--	--	--
G4ST01000902389	chr1:902390-902424	-	GGGAGGCGGTTCCCGGGGTTGGTGGGGGGAGCGGG<br>...(((...)))	C1orf170	NR_027693	chr1:900442-907336	non-template	35	Intron_3	--	Yes	--	Yes	--	--	--	--	--	--	--
G4ST01000902395	chr1:902396-902433	-	GGGGGGAGCGGGAGGCGGTTCCCGGGGTTGGTGGGGGG<br>...(((...)))	C1orf170	NR_027693	chr1:900442-907336	non-template	38	Intron_3	--	Yes	--	Yes	--	--	--	--	--	--	--
G4ST01000902406	chr1:902407-902442	-	GGGGTTGGTGGGGGGAGCGGGAGGCGGTTCCCGGGG<br>...(((...)))	C1orf170	NR_027693	chr1:900442-907336	non-template	36	Intron_3	--	Yes	--	Yes	--	--	--	--	--	--	--
G4ST01000902421	chr1:902422-902456	-	GGGAGGCGGTTCCCGGGGTTGGTGGGGGGAGCGGG<br>...(((...)))	C1orf170	NR_027693	chr1:900442-907336	non-template	35	Intron_3	--	Yes	--	Yes	--	--	--	--	--	--	--

G4ST01000902427	chr1:902428-902465	-	GGGGGGAGCGGGAGGCGGTTCCCGGGGTTGGTGGGGGG<br>...(((...)))	C1orf170	NR_027693	chr1:900442-907336	non-template	38	Intron_3	--	Yes	--	Yes	--	--	--	--	--	--	--
G4ST01000902438	chr1:902439-902474	-	GGGGTTGGTGGGGGGAGCGGGAGGCGGTTCCCGGGG<br>...(((...)))	C1orf170	NR_027693	chr1:900442-907336	non-template	36	Intron_3	--	Yes	--	Yes	--	--	--	--	--	--	--
G4ST01000902453	chr1:902454-902488	-	GGGAGGCGGTTCCCGGGGTTGGTGGGGGGAGCGGG<br>...(((...)))	C1orf170	NR_027693	chr1:900442-907336	non-template	35	Intron_3	--	Yes	--	Yes	--	--	--	--	--	--	--
G4ST01000902459	chr1:902460-902496	-	GGGGTGGTGGGAGGCGGTTCCCGGGGTTGGTGGGGGG<br>...(((...)))	C1orf170	NR_027693	chr1:900442-907336	non-template	37	Intron_3	--	Yes	--	Yes	--	--	--	--	--	--	--
G4ST01000902470	chr1:902471-902505	-	GGGGTTGGCGGGGTGGTGGGAGGCGGTTCCCGGGG<br>...(((...)))	C1orf170	NR_027693	chr1:900442-907336	non-template	35	Intron_3	--	Yes	--	Yes	--	--	--	--	--	--	--
G4ST01000902531	chr1:902532-902566	-	GGGGTTGCGGGGGGAGTGGGAGGCGGTTCCCGGGG<br>...(((...)))	C1orf170	NR_027693	chr1:900442-907336	non-template	35	Intron_3	--	Yes	--	Yes	--	--	--	--	--	--	--
G4ST01000902546	chr1:902547-902580	-	GGGAGGCGGTTCCCGGGGTTGCGGGGGGAGTGGG<br>...(((...)))	C1orf170	NR_027693	chr1:900442-907336	non-template	34	Intron_3	--	Yes	--	Yes	--	--	--	--	--	--	--
G4ST01000902552	chr1:902553-902589	-	GGGGGGAGCGGGAGGCGGTTCCCGGGGTTGCGGGGGG<br>...(((...)))	C1orf170	NR_027693	chr1:900442-907336	non-template	37	Intron_3	--	Yes	--	Yes	--	--	--	--	--	--	--
G4ST01000902562	chr1:902563-902598	-	GGGGTTGGTGGGGGGAGCGGGAGGCGGTTCCCGGGG<br>...(((...)))	C1orf170	NR_027693	chr1:900442-907336	non-template	36	Intron_3	--	Yes	--	Yes	--	--	--	--	--	--	--
G4ST01000902577	chr1:902578-902612	-	GGGAGGCGGTTCCCGGGGTTGGTGGGGGGAGCGGG<br>...(((...)))	C1orf170	NR_027693	chr1:900442-907336	non-template	35	Intron_3	--	Yes	--	Yes	--	--	--	--	--	--	--
G4ST01000902583	chr1:902584-902621	-	GGGGGGAGCGGGAGGCGGTTCCCGGGGTTGGTGGGGGG<br>...(((...)))	C1orf170	NR_027693	chr1:900442-907336	non-template	38	Intron_3	--	Yes	--	Yes	--	--	--	--	--	--	--
G4ST01000902594	chr1:902595-902630	-	GGGGTTGGTGGGGGGAGCGGGAGGCGGTTCCCGGGG<br>...(((...)))	C1orf170	NR_027693	chr1:900442-907336	non-template	36	Intron_3	--	Yes	--	Yes	--	--	--	--	--	--	--
G4ST01000902609	chr1:902610-902644	-	GGGAGGCGGTTCCCGGGGTTGGTGGGGGGAGCGGG<br>...(((...)))	C1orf170	NR_027693	chr1:900442-907336	non-template	35	Intron_3	--	Yes	--	Yes	--	--	--	--	--	--	--
G4ST01000902615	chr1:902616-902653	-	GGGGGGAGCGGGAGGCGGTTCCCGGGGTTGGTGGGGGG<br>...(((...)))	C1orf170	NR_027693	chr1:900442-907336	non-template	38	Intron_3	--	Yes	--	Yes	--	--	--	--	--	--	--
G4ST01000902626	chr1:902627-902662	-	GGGGTTGGTGGGGGGAGCGGGAGGCGGTTCCCGGGG<br>...(((...)))	C1orf170	NR_027693	chr1:900442-907336	non-template	36	Intron_3	--	Yes	--	Yes	--	--	--	--	--	--	--
G4ST01000902641	chr1:902642-902676	-	GGGAGGCGGTTCCCGGGGTTGGTGGGGGGAGCGGG<br>...(((...)))	C1orf170	NR_027693	chr1:900442-907336	non-template	35	Intron_3	--	Yes	--	Yes	--	--	--	--	--	--	--
G4ST01000902647	chr1:902648-902685	-	GGGGGGAGCGGGAGGCGGTTCCCGGGGTTGGTGGGGGG<br>...(((...)))	C1orf170	NR_027693	chr1:900442-907336	non-template	38	Intron_3	--	Yes	--	Yes	--	--	--	--	--	--	--
G4ST01000902658	chr1:902659-902694	-	GGGGTTGGTGGGGGGAGCGGGAGGCGGTTCCCGGGG<br>...(((...)))	C1orf170	NR_027693	chr1:900442-907336	non-template	36	Intron_3	--	Yes	--	Yes	--	--	--	--	--	--	--
G4ST01000902673	chr1:902674-902708	-	GGGAGGCGGTTCCCGGGGTTGGTGGGGGGAGCGGG<br>...(((...)))	C1orf170	NR_027693	chr1:900442-907336	non-template	35	Intron_3	--	Yes	--	Yes	--	--	--	--	--	--	--
G4ST01000902679	chr1:902680-902717	-	GGGGGGAGCGGGAGGCGGTTCCCGGGGTTGGTGGGGGG<br>...(((...)))	C1orf170	NR_027693	chr1:900442-907336	non-template	38	Intron_3	--	Yes	--	Yes	--	--	--	--	--	--	--
G4ST01000902690	chr1:902691-902726	-	GGGGTTGGTGGGGGGAGCGGGAGGCGGTTCCCGGGG<br>...(((...)))	C1orf170	NR_027693	chr1:900442-907336	non-template	36	Intron_3	--	Yes	--	Yes	--	--	--	--	--	--	--
G4ST01000902705	chr1:902706-902740	-	GGGAGGCGGTTCCCGGGGTTGGTGGGGGGAGCGGG<br>...(((...)))	C1orf170	NR_027693	chr1:900442-907336	non-template	35	Intron_3	--	Yes	--	Yes	--	--	--	--	--	--	--
G4ST01000902711	chr1:902712-902749	-	GGGGGGAGCGGGAGGCGGTTCCCGGGGTTGGTGGGGGG<br>...(((...)))	C1orf170	NR_027693	chr1:900442-907336	non-template	38	Intron_3	--	Yes	--	Yes	--	--	--	--	--	--	--
G4ST01000902722	chr1:902723-902758	-	GGGGTTGGTGGGGGGAGCGGGAGGCGGTTCCCGGGG<br>...(((...)))	C1orf170	NR_027693	chr1:900442-907336	non-template	36	Intron_3	--	Yes	--	Yes	--	--	--	--	--	--	--
G4ST01000902737	chr1:902738-902772	-	GGGAGGCGGTTCCCGGGGTTGGTGGGGGGAGCGGG<br>...(((...)))	C1orf170	NR_027693	chr1:900442-907336	non-template	35	Intron_3	--	Yes	--	Yes	--	--	--	--	--	--	--
G4ST01000902743	chr1:902744-902781	-	GGGGGGAGCGGGAGGCGGTTCCCGGGGTTGGTGGGGGG<br>...(((...)))	C1orf170	NR_027693	chr1:900442-907336	non-template	38	Intron_3	--	Yes	--	Yes	--	--	--	--	--	--	--
G4ST01000902754	chr1:902755-902790	-	GGGGTTGGTGGGGGGAGCGGGAGGCGGTTCCCGGGG<br>...(((...)))	C1orf170	NR_027693	chr1:900442-907336	non-template	36	Intron_3	--	Yes	--	Yes	--	--	--	--	--	--	--
G4ST01000902769	chr1:902770-902804	-	GGGAGGCGGTTCCCGGGGTTGGTGGGGGGAGCGGG<br>...(((...)))	C1orf170	NR_027693	chr1:900442-907336	non-template	35	Intron_3	--	Yes	--	Yes	--	--	--	--	--	--	--
G4ST01000902775	chr1:902776-902813	-	GGGGGGAGCGGGAGGCGGTTCCCGGGGTTGGTGGGGGG<br>...(((...)))	C1orf170	NR_027693	chr1:900442-907336	non-template	38	Intron_3	--	Yes	--	Yes	--	--	--	--	--	--	--
G4ST01000902786	chr1:902787-902822	-	GGGGTTGGTGGGGGGAGCGGGAGGCGGTTCCCGGGG<br>...(((...)))	C1orf170	NR_027693	chr1:900442-907336	non-template	36	Intron_3	--	Yes	--	Yes	--	--	--	--	--	--	--
G4ST01000902801	chr1:902802-902836	-	GGGAGGCGGTTCCCGGGGTTGGTGGGGGGAGCGGG<br>...(((...)))	C1orf170	NR_027693	chr1:900442-907336	non-template	35	Intron_3	--	Yes	--	Yes	--	--	--	--	--	--	--
G4ST01000902807	chr1:902808-902845	-	GGGGGGAGCGGGAGGCGGTTCCCGGGGTTGGTGGGGGG<br>...(((...)))	C1orf170	NR_027693	chr1:900442-907336	non-template	38	Intron_3	--	Yes	--	Yes	--	--	--	--	--	--	--
G4ST01000902818	chr1:902819-902854	-	GGGGTTGGTGGGGGGAGCGGGAGGCGGTTCCCGGGG<br>...(((...)))	C1orf170	NR_027693	chr1:900442-907336	non-template	36	Intron_3	--	Yes	--	Yes	--	--	--	--	--	--	--
G4ST01000902833	chr1:902834-902868	-	GGGAGGCGGTTCCCGGGGTTGGTGGGGGGAGCGGG<br>...(((...)))	C1orf170	NR_027693	chr1:900442-907336	non-template	35	Intron_3	--	Yes	--	Yes	--	--	--	--	--	--	--
G4ST01000902839	chr1:902840-902877	-	GGGGGGAGCGGGAGGCGGTTCCCGGGGTTGGTGGGGGG<br>...(((...)))	C1orf170	NR_027693	chr1:900442-907336	non-template	38	Intron_3	--	Yes	--	Yes	--	--	--	--	--	--	--
G4ST01000902850	chr1:902851-902886	-	GGGGTTGGTGGGGGGAGCGGGAGGCGGTTCCCGGGG<br>...(((...)))	C1orf170	NR_027693	chr1:900442-907336	non-template	36	Intron_3	--	Yes	--	Yes	--	--	--	--	--	--	--
G4ST01000902865	chr1:902866-902900	-	GGGAGGCGGTTCCCGGGGTTGGTGGGGGGAGCGGG<br>...(((...)))	C1orf170	NR_027693	chr1:900442-907336	non-template	35	Intron_3	--	Yes	--	Yes	--	--	--	--	--	--	--
G4ST01000902871	chr1:902872-902909	-	GGGGGGAGCGGGAGGCGGTTCCCGGGGTTGGTGGGGGG<br>...(((...)))	C1orf170	NR_027693	chr1:900442-907336	non-template	38	Intron_3	--	Yes	--	Yes	--	--	--	--	--	--	--
G4ST01000902882	chr1:902883-902918	-	GGGGTTGGTGGGGGGAGCGGGAGGCGGTTCCCGGGG<br>...(((...)))	C1orf170	NR_027693	chr1:900442-907336	non-template	36	Intron_3	--	Yes	--	Yes	--	--	--	--	--	--	--
G4ST01000902897	chr1:902898-902932	-	GGGAGGCGGTTCCCGGGGTTGGTGGGGGGAGCGGG<br>...(((...)))	C1orf170	NR_027693	chr1:900442-907336	non-template	35	Intron_3	--	Yes	--	Yes	--	--	--	--	--	--	--
G4ST01000902903	chr1:902904-902941	-	GGGGGGAGCGGGAGGCGGTTCCCGGGGTTGGTGGGGGG<br>...(((...)))	C1orf170	NR_027693	chr1:900442-907336	non-template	38	Intron_3	--	Yes	--	Yes	--	--	--	--	--	--	--
G4ST01000902914	chr1:902915-902949	-	GGGGTTGCGGGGGGAGCGGGAGGCGGTTCCCGGGG<br>...(((...)))	C1orf170	NR_027693	chr1:900442-907336	non-template	35	Intron_3	--	Yes	--	Yes	--	--	--	--	--	--	--
G4ST01000902975	chr1:902976-903008	-	GGGGTTGGTGGGGGTGGGAGGCGGTTCCCGGGG<br>...(((...)))	C1orf170	NR_027693	chr1:900442-907336	non-template	33	Intron_3	--	Yes	--	Yes	--	--	--	--	--	--	--
G4ST01000902990	chr1:902991-903022	-	GGGAGGCGGTTCCCGGGGTTGGTGGGGGTGGG<br>...(((...)))	C1orf170	NR_027693	chr1:900442-907336	non-template	32	Intron_3	--	Yes	--	Yes	--	--	--	--	--	--	--
G4ST01000902994	chr1:902995-903031	-	GGGGGGATTGGGAGGCGGTTCCCGGGGTTGGTGGGGG<br>...(((...)))	C1orf170	NR_027693	chr1:900442-907336	non-template	37	Intron_3	--	Yes	--	Yes	--	--	--	--	--	--	--
G4ST01000903004	chr1:903005-903037	-	GGGGTTGGGGGGATTGGGAGGCGGTTCCCGGGG<br>...(((...)))	C1orf170	NR_027693	chr1:900442-907336	non-template	33	Intron_3	--	Yes	--	Yes	--	--	--	--	--	--	--
G4ST01000903048	chr1:903049-903080	-	GGGAGGCGGTTCCCGGGGTTGGTGGGGGTGGG<br>...(((...)))	C1orf170	NR_027693	chr1:900442-907336	non-template	32	Intron_3	--	Yes	--	Yes	--	--	--	--	--	--	--
G4ST01000903052	chr1:903053-903089	-	GGGGGGATTGGGAGGCGGTTCCCGGGGTTGGTGGGGG<br>...(((...)))	C1orf170	NR_027693	chr1:900442-907336	non-template	37	Intron_3	--	Yes	--	Yes	--	--	--	--	--	--	--
G4ST01000903062	chr1:903063-903095	-	GGGGTTGGGGGGATTGGGAGGCGGTTCCCGGGG<br>...(((...)))	C1orf170	NR_027693	chr1:900442-907336	non-template	33	Intron_3	--	Yes	--	Yes	--	--	--	--	--	--	--
G4ST01000903106	chr1:903107-903138	-	GGGAGGCGGTTCCCGGGGTTGGGGGTGGTGGG<br>...(((...)))	C1orf170	NR_027693	chr1:900442-907336	non-template	32	Intron_3	--	Yes	--	Yes	--	--	--	--	--	--	--

G4ST01000903113	chr1:903114-903147	-	GGGGGGAGTGGGAGGCGGTTCCCGGGGTTGGGGG<br>...(((...)))	C1orf170	NR_027693	chr1:900442-907336	non-template	34	Intron_3	--	Yes	--	Yes	--	--	--	--	--	--	--
G4ST01000903120	chr1:903121-903155	-	GGGGTTGCGGGGGGAGTGGGAGGCGGTTCCCGGGG<br>...(((...)))	C1orf170	NR_027693	chr1:900442-907336	non-template	35	Intron_3	--	Yes	--	Yes	--	--	--	--	--	--	--
G4ST01000903135	chr1:903136-903169	-	GGGAGGCGGTTCCCGGGGTTGCGGGGGGAGTGGG<br>...(((...)))	C1orf170	NR_027693	chr1:900442-907336	non-template	34	Intron_3	--	Yes	--	Yes	--	--	--	--	--	--	--
G4ST01000903141	chr1:903142-903177	-	GGGGTGGTGGGAGGCGGTTCCCGGGGTTGCGGGGGG<br>...(((...)))	C1orf170	NR_027693	chr1:900442-907336	non-template	36	Intron_3	--	Yes	--	Yes	--	--	--	--	--	--	--
G4ST01000903151	chr1:903152-903185	-	GGGGTTGCGGGGTGGTGGGAGGCGGTTCCCGGGG<br>...(((...)))	C1orf170	NR_027693	chr1:900442-907336	non-template	34	Intron_3	--	Yes	--	Yes	--	--	--	--	--	--	--
G4ST01000903211	chr1:903212-903244	-	GGGGTTGGGGGGATTGGGAGGCGGTTCCCGGGG<br>...(((...)))	C1orf170	NR_027693	chr1:900442-907336	non-template	33	Intron_3	--	Yes	--	Yes	--	--	--	--	--	--	--
G4ST01000903226	chr1:903227-903258	-	GGGAGGCGGTTCCTGGGGTTGGGGGGATTGGG<br>(((.....))).	C1orf170	NR_027693	chr1:900442-907336	non-template	32	Intron_3	--	Yes	--	Yes	--	--	--	--	--	--	Yes
G4ST01000903232	chr1:903233-903264	-	GGGGTTGGGAGGCGGTTCCTGGGGTTGGGGGG<br>(((.....))).	C1orf170	NR_027693	chr1:900442-907336	non-template	32	Intron_3	--	Yes	--	Yes	--	--	--	--	--	--	Yes
G4ST01000903240	chr1:903241-903268	-	GGGGGGGGTTGGGAGGCGGTTCCTGGGG<br>(((.....))).	C1orf170	NR_027693	chr1:900442-907336	non-template	28	Intron_3	--	Yes	--	Yes	--	--	--	--	--	--	Yes
G4ST01000903260	chr1:903261-903288	-	GGGAGGCGGTTCCTGGGGTTGGGGGGGG<br>(((.....))).	C1orf170	NR_027693	chr1:900442-907336	non-template	28	Intron_3	--	Yes	--	Yes	--	--	--	--	Yes	--	Yes
G4ST01000903265	chr1:903266-903294	-	GGGGTTGGGAGGCGGTTCCTGGGGTTGGG<br>(((.....))).	C1orf170	NR_027693	chr1:900442-907336	non-template	29	Intron_3	--	Yes	--	Yes	--	--	--	--	Yes	--	Yes
G4ST01000903270	chr1:903271-903298	-	GGGGGGGGTTGGGAGGCGGTTCCTGGGG<br>(((.....))).	C1orf170	NR_027693	chr1:900442-907336	non-template	28	Intron_3	--	Yes	--	Yes	--	--	--	--	Yes	--	Yes
G4ST01000903290	chr1:903291-903320	-	GGGGCAGGCAGTTCCTGGGGTTGGGGGGGG<br>.((((.....))))	C1orf170	NR_027693	chr1:900442-907336	non-template	30	Intron_3	--	Yes	--	Yes	--	--	--	Yes	Yes	--	Yes
G4ST01000903390	chr1:903391-903424	-	GGGTGGGGCAGGCGGTTCCTGGGGTTGGAGGGGG<br>.((((.....))))	C1orf170	NR_027693	chr1:900442-907336	non-template	34	Intron_3	--	Yes	--	Yes	--	--	--	Yes	Yes	--	--
G4ST01000903400	chr1:903401-903430	-	GGGTTAGGGTGGGGCAGGCGGTTCCTGGGG<br>.((((.....))))	C1orf170	NR_027693	chr1:900442-907336	non-template	30	Intron_3	--	Yes	--	Yes	--	--	--	Yes	Yes	--	--
G4ST01000903837	chr1:903838-903869	+	GGGCGGGAACCCTGCCAGAGGCTGGGGGAGGG<br>...((.(((...))).))	C1orf170	NR_027693	chr1:900442-907336	template	32	Intron_3	--	Yes	--	Yes	--	--	--	--	--	--	--
G4ST01000904669	chr1:904670-904705	+	GGGCCCCAGTTGCTGTCTGGGGGGCTGAGGGCCGGG<br>.(((((.......)))))	C1orf170	NR_027693	chr1:900442-907336	template	36	Exon_3	--	Yes	Yes	--	--	--	--	Yes	--	--	--
G4ST01000944924	chr1:944925-944958	-	GGGGCTCCGGGCGGCGCGGAGCCGGGCGGCAGGG<br>.(((.....)))	AGRN	NM_198576	chr1:945366-981355	template	-407	upstream	Yes	--	--	--	--	--	--	Yes	Yes	Yes	--
G4ST01000945625	chr1:945626-945663	-	GGGGCGGCAGGCGTCGCGAGGGGAGGCCGGGGTCCGGG<br>(((((....)))))...	AGRN	NM_198576	chr1:945366-981355	template	38	Intron_1.f	--	Yes	--	Yes	--	--	--	Yes	Yes	--	Yes
G4ST01000945631	chr1:945632-945675	-	GGGGGTCCCGGCGGGGCGGCAGGCGTCGCGAGGGGAGGCCGGGG<br>(((((....)))))...	AGRN	NM_198576	chr1:945366-981355	template	44	Intron_1.f	--	Yes	--	Yes	--	--	--	--	Yes	--	Yes
G4ST01000945640	chr1:945641-945680	-	GGGGCGGGGGTCCCGGCGGGGCGGCAGGCGTCGCGAGGGG<br>(((((....)))))...	AGRN	NM_198576	chr1:945366-981355	template	40	Intron_1.f	--	Yes	--	Yes	--	--	--	--	Yes	--	Yes
G4ST01000948678	chr1:948679-948720	+	GGGTCCAGGGCCTCAGTGCAGCACAGAGGAGGGTCCCCTGGG<br>((((.(((...))).)))).	AGRN	NM_198576	chr1:945366-981355	non-template	42	Intron_2	--	Yes	--	Yes	--	--	--	--	--	--	--
G4ST01000952987	chr1:952988-953032	+	GGGGGCAGCAGGGCAGTCTGGGAGTCAGACCAGGCCTGGTCAGGG<br>.....(((((....))))).	AGRN	NM_198576	chr1:945366-981355	non-template	45	Intron_2	--	Yes	--	Yes	--	--	--	--	--	--	--
G4ST01000952997	chr1:952998-953036	+	GGGCAGTCTGGGAGTCAGACCAGGCCTGGTCAGGGTGGG<br>.....(((((....))))).	AGRN	NM_198576	chr1:945366-981355	non-template	39	Intron_2	--	Yes	--	Yes	--	--	--	--	--	--	--
G4ST01000953006	chr1:953007-953044	+	GGGAGTCAGACCAGGCCTGGTCAGGGTGGGAGCGGGGG<br>.....(((((....))))).	AGRN	NM_198576	chr1:945366-981355	non-template	38	Intron_2	--	Yes	--	Yes	--	--	--	--	--	--	--
G4ST01000958671	chr1:958672-958706	-	GGGCCGGGCGGGTCCGCGCCCCGCACCCGCGGGGG<br>.(((((.........)))))	AGRN	NM_198576	chr1:945366-981355	template	35	Intron_2	--	Yes	--	Yes	--	--	--	Yes	Yes	Yes	--
G4ST01000958694	chr1:958695-958720	-	GGGGAGCAGGAGCTGGGCCGGGCGGG<br>.(((....)))	AGRN	NM_198576	chr1:945366-981355	template	26	Intron_2	--	Yes	--	Yes	--	--	--	Yes	Yes	Yes	--
G4ST01000961027	chr1:961028-961063	+	GGGGAGGGGCCTGGAGGGCGGCAGTCTGTCCCTGGG<br>.((((...))))...	AGRN	NM_198576	chr1:945366-981355	non-template	36	Intron_3	--	Yes	--	Yes	--	--	--	--	Yes	--	--
G4ST01000962180	chr1:962181-962216	+	GGGGACACGGGTTGCAGGGGCCTGCAACAGGCTGGG<br>(((((...))))).	AGRN	NM_198576	chr1:945366-981355	non-template	36	Intron_3	--	Yes	--	Yes	--	--	--	--	--	--	--
G4ST01000962817	chr1:962818-962850	+	GGGAGAGTGGGTGGGGAGGCCGAGTGGCCGGGG<br>..((((....)))).	AGRN	NM_198576	chr1:945366-981355	non-template	33	Intron_3	--	Yes	--	Yes	--	--	--	--	--	--	--
G4ST01000962825	chr1:962826-962855	+	GGGTGGGGAGGCCGAGTGGCCGGGGAGGGG<br>..((((....)))).	AGRN	NM_198576	chr1:945366-981355	non-template	30	Intron_3	--	Yes	--	Yes	--	--	--	--	--	--	--
G4ST01000964041	chr1:964042-964079	+	GGGAGTCCCAGGGCCTTGGACAAGGCAGGGCCAAAGGG<br>((((....))))..	AGRN	NM_198576	chr1:945366-981355	non-template	38	Intron_3	--	Yes	--	Yes	--	--	--	Yes	Yes	--	--
G4ST01000964051	chr1:964052-964085	+	GGGCCTTGGACAAGGCAGGGCCAAAGGGAGAGGG<br>((((....))))..	AGRN	NM_198576	chr1:945366-981355	non-template	34	Intron_3	--	Yes	--	Yes	--	--	--	Yes	Yes	--	--
G4ST01000965878	chr1:965879-965905	-	GGGGCGGGTAGGGGAGAAAGCTCAGGG<br>(((....))).	AGRN	NM_198576	chr1:945366-981355	template	27	Intron_3	--	Yes	--	Yes	--	--	--	--	Yes	Yes	--
G4ST01000966984	chr1:966985-967016	+	GGGTCGAGGTGGGCGGCTCCCCCGGGGGAGGG<br>(((.....))).	AGRN	NM_198576	chr1:945366-981355	non-template	32	Intron_6	--	Yes	--	Yes	--	--	--	--	--	--	--
G4ST01000967031	chr1:967032-967063	+	GGGTCGAGGTGGGCGGCTCCCCCGGGGGAGGG<br>(((.....))).	AGRN	NM_198576	chr1:945366-981355	non-template	32	Intron_6	--	Yes	--	Yes	--	--	--	--	--	--	--
G4ST01000967178	chr1:967179-967215	-	GGGCTCCGGGCACTGGTCTAGGACGCAGGGGTGAGGG<br>..((((((...))).)))	AGRN	NM_198576	chr1:945366-981355	template	37	Intron_6-Exon_7	--	Yes	Yes	Yes	Yes	--	--	--	--	--	--
G4ST01000969127	chr1:969128-969157	-	GGGGCCGGGCGGGGGGTGCTCACACCGGGG<br>.((((....)))).	AGRN	NM_198576	chr1:945366-981355	template	30	Exon_10	--	Yes	Yes	--	Yes	--	--	--	--	--	--
G4ST01000971962	chr1:971963-972003	+	GGGGGTGGCTCTGGGGGTGAGCAGGGATCAAGGACTTGGGG<br>..(((....)))	AGRN	NM_198576	chr1:945366-981355	non-template	41	Exon_18-Intron_18	--	Yes	Yes	Yes	Yes	--	--	--	--	--	--
G4ST01000971974	chr1:971975-972007	+	GGGGGTGAGCAGGGATCAAGGACTTGGGGTGGG<br>..(((....)))	AGRN	NM_198576	chr1:945366-981355	non-template	33	Exon_18-Intron_18	--	Yes	Yes	Yes	Yes	--	--	--	--	--	--
G4ST01000971985	chr1:971986-972011	+	GGGATCAAGGACTTGGGGTGGGTGGG<br>..(((....)))	AGRN	NM_198576	chr1:945366-981355	non-template	26	Intron_18	--	Yes	--	Yes	--	--	--	--	--	--	--
G4ST01000973647	chr1:973648-973674	+	GGGTGGGGGCAAGGATTGGGGGTGGGG<br>..(((...))).	AGRN	NM_198576	chr1:945366-981355	non-template	27	Intron_23	--	Yes	--	Yes	--	--	--	--	--	--	--
G4ST01000975170	chr1:975171-975207	-	GGGCGCCGCCCCATGGCAGGGGTTGGGCTGGCAGGGG<br>.((((.....))))..	AGRN	NM_198576	chr1:945366-981355	template	37	Exon_27	--	Yes	Yes	--	Yes	--	--	--	Yes	--	--
G4ST01000981226	chr1:981227-981264	-	GGGTCCTGCTCCTAGGAGGACTGGGCTGGGGCTGGGGG<br>.(((.((...)).)))...	AGRN	NM_198576	chr1:945366-981355	template	38	Exon_36.l	--	Yes	Yes	--	--	--	Yes	--	--	--	Yes
G4ST01001017866	chr1:1017867-1017899	+	GGGGTCCTGGGACAAGGGCTGTGAGCAGCAGGG<br>(((....)))..	C1orf159	NM_017891	chr1:1007061-1041599	template	33	Intron_1.f	--	Yes	--	Yes	--	--	--	--	--	--	--
G4ST01001025663	chr1:1025664-1025696	-	GGGTAAGGCTGTGCCTGGGTGGGCACAGCGGGG<br>..((((...))))	C1orf159	NM_017891	chr1:1007061-1041599	non-template	33	Intron_1.f	--	Yes	--	Yes	--	--	--	--	--	--	--
G4ST01001025673	chr1:1025674-1025706	-	GGGATGCTGAGGGTAAGGCTGTGCCTGGGTGGG<br>..((((...))))	C1orf159	NM_017891	chr1:1007061-1041599	non-template	33	Intron_1.f	--	Yes	--	Yes	--	--	--	--	--	--	--
G4ST01001034611	chr1:1034612-1034649	+	GGGGGGCAGGGAGGAATGGGGAGTTAGTGTTTAATGGG<br>..((((.....))))	C1orf159	NM_017891	chr1:1007061-1041599	template	38	Intron_1.f	--	Yes	--	Yes	--	--	--	--	--	--	Yes
G4ST01001041495	chr1:1041496-1041528	+	GGGGCGCCGGGGCGGGGCGCGAAGCGCCCTGGG<br>((((...))))...	C1orf159	NM_017891	chr1:1007061-1041599	template	33	Exon_1.f	--	Yes	Yes	--	--	Yes	--	Yes	Yes	Yes	Yes
G4ST01001092284	chr1:1092285-1092325	-	GGGTTGCATGGGACTCGCTGGGAAGCTCAGTAGCTGGGGGG<br>.((((....))))...	MIR200A	NR_029834	chr1:1093106-1093195	template	-780	upstream	Yes	--	--	--	--	--	--	--	--	--	--

G4ST01001092284	chr1:1092285-1092325	-	GGGTTGCATGGGACTCGCTGGGAAGCTCAGTAGCTGGGGGG<br>.((((....)))).. .	MIR429	NR_029957	chr1:1094248-1094330	template	-1922	upstream	Yes	--	--	--	--	--	--	--	--	--	--
G4ST01001092284	chr1:1092285-1092325	-	GGGTTGCATGGGACTCGCTGGGAAGCTCAGTAGCTGGGGGG<br>.((((....)))).. .	MIR200B	NR_029639	chr1:1092347-1092441	template	-21	upstream	Yes	--	--	--	--	--	--	--	--	--	--
G4ST01001093415	chr1:1093416-1093452	+	GGGGAGGGGCCGGCCTCTGCCGGGGGTGCCCCCGGGG<br>.(((((....)))))	MIR429	NR_029957	chr1:1094248-1094330	non-template	-795	upstream	Yes	--	--	--	--	--	--	--	--	--	--
G4ST01001098896	chr1:1098897-1098924	+	GGGCCGGGCGGAGGGGCAGCAACTGGGG<br>.(((...)))	TTLL10	NM_001130045	chr1:1099149-1123176	non-template	-224	upstream	Yes	--	--	--	--	--	--	--	--	--	Yes
G4ST01001105156	chr1:1105157-1105193	+	GGGCCAGGGGGATTGTCCAGGGTATTGGAGAAATGGG<br>(((...))). .(((.....)))	TTLL10	NM_153254	chr1:1104940-1111106	non-template	37	Intron_1.f	--	Yes	--	Yes	--	--	--	--	--	--	--
G4ST01001105156	chr1:1105157-1105193	+	GGGCCAGGGGGATTGTCCAGGGTATTGGAGAAATGGG<br>(((...))). .(((.....)))	TTLL10	NM_001130045	chr1:1099149-1123176	non-template	37	Intron_5	--	Yes	--	Yes	--	--	--	--	--	--	--
G4ST01001105162	chr1:1105163-1105198	+	GGGGGATTGTCCAGGGTATTGGAGAAATGGGCTGGG<br>(((...))). .(((.....)))	TTLL10	NM_153254	chr1:1104940-1111106	non-template	36	Intron_1.f	--	Yes	--	Yes	--	--	--	--	--	--	--
G4ST01001105162	chr1:1105163-1105198	+	GGGGGATTGTCCAGGGTATTGGAGAAATGGGCTGGG<br>(((...))). .(((.....)))	TTLL10	NM_001130045	chr1:1099149-1123176	non-template	36	Intron_5	--	Yes	--	Yes	--	--	--	--	--	--	--
G4ST01001105635	chr1:1105636-1105668	-	GGGCGGCCAGCGGAGGCCGGGCTGGGCCCCGGG<br>.((((......))))	TTLL10	NM_153254	chr1:1104940-1111106	template	33	Intron_2	--	Yes	--	Yes	--	--	--	--	--	--	--
G4ST01001105635	chr1:1105636-1105668	-	GGGCGGCCAGCGGAGGCCGGGCTGGGCCCCGGG<br>.((((......))))	TTLL10	NM_001130045	chr1:1099149-1123176	template	33	Intron_6	--	Yes	--	Yes	--	--	--	--	--	--	--
G4ST01001105642	chr1:1105643-1105677	-	GGGGATGGCGGGCGGCCAGCGGAGGCCGGGCTGGG<br>.((((......))))	TTLL10	NM_153254	chr1:1104940-1111106	template	35	Intron_2	--	Yes	--	Yes	--	--	--	--	--	--	--
G4ST01001105642	chr1:1105643-1105677	-	GGGGATGGCGGGCGGCCAGCGGAGGCCGGGCTGGG<br>.((((......))))	TTLL10	NM_001130045	chr1:1099149-1123176	template	35	Intron_6	--	Yes	--	Yes	--	--	--	--	--	--	--
G4ST01001105647	chr1:1105648-1105683	-	GGGGTTGGGGATGGCGGGCGGCCAGCGGAGGCCGGG<br>.((((......))))	TTLL10	NM_153254	chr1:1104940-1111106	template	36	Intron_2	--	Yes	--	Yes	--	--	--	--	--	--	--
G4ST01001105647	chr1:1105648-1105683	-	GGGGTTGGGGATGGCGGGCGGCCAGCGGAGGCCGGG<br>.((((......))))	TTLL10	NM_001130045	chr1:1099149-1123176	template	36	Intron_6	--	Yes	--	Yes	--	--	--	--	--	--	--
G4ST01001106219	chr1:1106220-1106257	-	GGGGCTGGAGCCAGCCTGGGAGCCTGGGTGGCCAGGGG<br>((((....))))..	TTLL10	NM_153254	chr1:1104940-1111106	template	38	Intron_4	--	Yes	--	Yes	--	--	--	--	--	--	Yes
G4ST01001106219	chr1:1106220-1106257	-	GGGGCTGGAGCCAGCCTGGGAGCCTGGGTGGCCAGGGG<br>((((....))))..	TTLL10	NM_001130045	chr1:1099149-1123176	template	38	Intron_8	--	Yes	--	Yes	--	--	--	--	--	--	Yes
G4ST01001106229	chr1:1106230-1106263	-	GGGGCAGGGGCTGGAGCCAGCCTGGGAGCCTGGG<br>((((....))))..	TTLL10	NM_153254	chr1:1104940-1111106	template	34	Intron_4	--	Yes	--	Yes	--	--	--	--	--	--	Yes
G4ST01001106229	chr1:1106230-1106263	-	GGGGCAGGGGCTGGAGCCAGCCTGGGAGCCTGGG<br>((((....))))..	TTLL10	NM_001130045	chr1:1099149-1123176	template	34	Intron_8	--	Yes	--	Yes	--	--	--	--	--	--	Yes
G4ST01001106237	chr1:1106238-1106268	-	GGGCAGGGGCAGGGGCTGGAGCCAGCCTGGG<br>((((....))))..	TTLL10	NM_153254	chr1:1104940-1111106	template	31	Intron_4	--	Yes	--	Yes	--	--	--	--	--	--	Yes
G4ST01001106237	chr1:1106238-1106268	-	GGGCAGGGGCAGGGGCTGGAGCCAGCCTGGG<br>((((....))))..	TTLL10	NM_001130045	chr1:1099149-1123176	template	31	Intron_8	--	Yes	--	Yes	--	--	--	--	--	--	Yes
G4ST01001108064	chr1:1108065-1108097	+	GGGGCGTGGCTGGGTTGGGGGCGAGGCCAGGGG<br>(((...)))..	TTLL10	NM_153254	chr1:1104940-1111106	non-template	33	Intron_6	--	Yes	--	Yes	--	--	--	Yes	--	--	--
G4ST01001108064	chr1:1108065-1108097	+	GGGGCGTGGCTGGGTTGGGGGCGAGGCCAGGGG<br>(((...)))..	TTLL10	NM_001130045	chr1:1099149-1123176	non-template	33	Intron_10	--	Yes	--	Yes	--	--	--	Yes	--	--	--
G4ST01001108567	chr1:1108568-1108598	-	GGGCCGGGGAGTAGGGGGGCTGGCAGCCGGG<br>.((((...))))	TTLL10	NM_153254	chr1:1104940-1111106	template	31	Intron_7	--	Yes	--	Yes	--	--	--	--	--	--	--
G4ST01001108567	chr1:1108568-1108598	-	GGGCCGGGGAGTAGGGGGGCTGGCAGCCGGG<br>.((((...))))	TTLL10	NM_001130045	chr1:1099149-1123176	template	31	Intron_11	--	Yes	--	Yes	--	--	--	--	--	--	--
G4ST01001108990	chr1:1108991-1109029	-	GGGGGCATTTCTGGGCCTGAATATGTCAGGGGAATTGGG<br>.((((.....))))	TTLL10	NM_153254	chr1:1104940-1111106	template	39	Intron_7	--	Yes	--	Yes	--	--	--	--	--	--	--
G4ST01001108990	chr1:1108991-1109029	-	GGGGGCATTTCTGGGCCTGAATATGTCAGGGGAATTGGG<br>.((((.....))))	TTLL10	NM_001130045	chr1:1099149-1123176	template	39	Intron_11	--	Yes	--	Yes	--	--	--	--	--	--	--
G4ST01001108997	chr1:1108998-1109035	-	GGGAAAGGGGGCATTTCTGGGCCTGAATATGTCAGGGG<br>.((((.....))))	TTLL10	NM_153254	chr1:1104940-1111106	template	38	Intron_7	--	Yes	--	Yes	--	--	--	--	--	--	--
G4ST01001108997	chr1:1108998-1109035	-	GGGAAAGGGGGCATTTCTGGGCCTGAATATGTCAGGGG<br>.((((.....))))	TTLL10	NM_001130045	chr1:1099149-1123176	template	38	Intron_11	--	Yes	--	Yes	--	--	--	--	--	--	--
G4ST01001109691	chr1:1109692-1109724	+	GGGGCCCTGTGGGCAGAGATTTCTGGGGACGGG<br>((((....))))	TTLL10	NM_153254	chr1:1104940-1111106	non-template	33	Intron_8.l	--	Yes	--	Yes	--	--	--	--	--	--	--
G4ST01001109691	chr1:1109692-1109724	+	GGGGCCCTGTGGGCAGAGATTTCTGGGGACGGG<br>((((....))))	TTLL10	NM_001130045	chr1:1099149-1123176	non-template	33	Intron_12	--	Yes	--	Yes	--	--	--	--	--	--	--
G4ST01001109701	chr1:1109702-1109734	+	GGGCAGAGATTTCTGGGGACGGGTCTTCATGGG<br>((((....))))	TTLL10	NM_153254	chr1:1104940-1111106	non-template	33	Intron_8.l	--	Yes	--	Yes	--	--	--	--	--	--	--
G4ST01001109701	chr1:1109702-1109734	+	GGGCAGAGATTTCTGGGGACGGGTCTTCATGGG<br>((((....))))	TTLL10	NM_001130045	chr1:1099149-1123176	non-template	33	Intron_12	--	Yes	--	Yes	--	--	--	--	--	--	--
G4ST01001122560	chr1:1122561-1122604	-	GGGACCCAGGTGAGCTGAGGTGTGGGGAAGGTTGGGGCAGGGGG<br>((((((.....))).)))...	TTLL10	NM_001130045	chr1:1099149-1123176	template	44	Intron_15.l	--	Yes	--	Yes	--	--	--	--	--	--	Yes
G4ST01001122567	chr1:1122568-1122609	-	GGGGTGGGACCCAGGTGAGCTGAGGTGTGGGGAAGGTTGGGG<br>((((((.....))).)))...	TTLL10	NM_001130045	chr1:1099149-1123176	template	42	Intron_15.l	--	Yes	--	Yes	--	--	--	--	--	--	Yes
G4ST01001122577	chr1:1122578-1122617	-	GGGTGTGAGGGGTGGGACCCAGGTGAGCTGAGGTGTGGGG<br>((((((.....))).)))...	TTLL10	NM_001130045	chr1:1099149-1123176	template	40	Intron_15.l	--	Yes	--	Yes	--	--	--	--	--	--	Yes
G4ST01001123112	chr1:1123113-1123147	-	GGGCAGGCAGCACGGCTGGGGCGCGGGGCGCGGGG<br>....((((...))))	TTLL10	NM_001130045	chr1:1099149-1123176	template	35	Exon_16.l	--	Yes	Yes	--	--	--	Yes	--	--	--	Yes
G4ST01001132395	chr1:1132396-1132431	+	GGGGCTTGGGAGGGGCACCAGGAGGAGCTGGTGGGG<br>.((((((......))))))	TNFRSF18	NM_148901	chr1:1128751-1131952	template	-443	upstream	Yes	--	--	--	--	--	--	--	Yes	--	--
G4ST01001132395	chr1:1132396-1132431	+	GGGGCTTGGGAGGGGCACCAGGAGGAGCTGGTGGGG<br>.((((((......))))))	TNFRSF18	NM_148902	chr1:1128751-1131952	template	-443	upstream	Yes	--	--	--	--	--	--	--	Yes	--	--
G4ST01001132395	chr1:1132396-1132431	+	GGGGCTTGGGAGGGGCACCAGGAGGAGCTGGTGGGG<br>.((((((......))))))	TNFRSF18	NM_004195	chr1:1128751-1131952	template	-443	upstream	Yes	--	--	--	--	--	--	--	Yes	--	--
G4ST01001132643	chr1:1132644-1132683	-	GGGCTGGGCAATCCAGGGAGGCTTCCTGGTGGAGGCAGGG<br>..((((((....))))))).	TNFRSF18	NM_148901	chr1:1128751-1131952	non-template	-691	upstream	Yes	--	--	--	--	--	--	--	Yes	--	--
G4ST01001132643	chr1:1132644-1132683	-	GGGCTGGGCAATCCAGGGAGGCTTCCTGGTGGAGGCAGGG<br>..((((((....))))))).	TNFRSF18	NM_148902	chr1:1128751-1131952	non-template	-691	upstream	Yes	--	--	--	--	--	--	--	Yes	--	--
G4ST01001132643	chr1:1132644-1132683	-	GGGCTGGGCAATCCAGGGAGGCTTCCTGGTGGAGGCAGGG<br>..((((((....))))))).	TNFRSF18	NM_004195	chr1:1128751-1131952	non-template	-691	upstream	Yes	--	--	--	--	--	--	--	Yes	--	--
G4ST01001137732	chr1:1137733-1137776	-	GGGGGCAGTTTTGGGGGAAGGGGAGGTGCTGGTGGCCCTGGGGG<br>.(((.((.....)))))..	TNFRSF4	NM_003327	chr1:1136569-1139411	non-template	44	Intron_4	--	Yes	--	Yes	--	--	--	--	--	--	Yes
G4ST01001137989	chr1:1137990-1138022	+	GGGTGCTGGGGACATGGGGCAGAGCCTGTGGGG<br>.(((...)))..	TNFRSF4	NM_003327	chr1:1136569-1139411	template	33	Intron_3	--	Yes	--	Yes	--	--	--	--	Yes	--	--
G4ST01001138709	chr1:1138710-1138741	+	GGGCCCCCGGAGGGAGAGGGGGTGCCCCTGGG<br>((...)).	TNFRSF4	NM_003327	chr1:1136569-1139411	template	32	Intron_2	--	Yes	--	Yes	--	--	--	Yes	Yes	--	--
G4ST01001139125	chr1:1139126-1139162	-	GGGCCAGCCAGGAGGCTGGTCCTGGGGAGGGGGCGGG<br>((((((....)))))).... .	TNFRSF4	NM_003327	chr1:1136569-1139411	non-template	37	Intron_1.f	--	Yes	--	Yes	--	--	--	--	Yes	--	Yes
G4ST01001139129	chr1:1139130-1139167	-	GGGATGGGCCAGCCAGGAGGCTGGTCCTGGGGAGGGGG<br>((((((....)))))).... .	TNFRSF4	NM_003327	chr1:1136569-1139411	non-template	38	Intron_1.f	--	Yes	--	Yes	--	--	--	--	Yes	--	Yes
G4ST01001139135	chr1:1139136-1139173	-	GGGGCAGGGATGGGCCAGCCAGGAGGCTGGTCCTGGGG<br>((((((....)))))).... .	TNFRSF4	NM_003327	chr1:1136569-1139411	non-template	38	Intron_1.f	--	Yes	--	Yes	--	--	--	--	Yes	--	Yes
G4ST01001143918	chr1:1143919-1143953	-	GGGCATCGTCGGGAGGTCGGGGCAGGTCCTGCGGG<br>((((...))))	SDF4	NM_016547	chr1:1142151-1157310	non-template	35	Intron_5	--	Yes	--	Yes	--	--	--	--	--	--	Yes
G4ST01001143918	chr1:1143919-1143953	-	GGGCATCGTCGGGAGGTCGGGGCAGGTCCTGCGGG<br>((((...))))	SDF4	NM_016176	chr1:1142151-1157310	non-template	35	Intron_5	--	Yes	--	Yes	--	--	--	--	--	--	Yes

G4ST01001144503	chr1:1144504-1144544	-	GGGCTGTGTGGGGGGTCCAGGCCATGTCCTGGGGGTCTGGG<br>....(((((......)))))	SDF4	NM_016547	chr1:1142151-1157310	non-template	41	Intron_4	--	Yes	--	Yes	--	--	--	--	--	--	--
G4ST01001144503	chr1:1144504-1144544	-	GGGCTGTGTGGGGGGTCCAGGCCATGTCCTGGGGGTCTGGG<br>....(((((......)))))	SDF4	NM_016176	chr1:1142151-1157310	non-template	41	Intron_4	--	Yes	--	Yes	--	--	--	--	--	--	--
G4ST01001147788	chr1:1147789-1147836	-	GGGGAGAGGGCACAGCCTGTGCCTGAGGGCCTGGCCCTGAGGCCTGGG<br>((((...))))..... ...((((....)))).	SDF4	NM_016547	chr1:1142151-1157310	non-template	48	Intron_4	--	Yes	--	Yes	--	--	--	--	--	--	--
G4ST01001147788	chr1:1147789-1147836	-	GGGGAGAGGGCACAGCCTGTGCCTGAGGGCCTGGCCCTGAGGCCTGGG<br>((((...))))..... ...((((....)))).	SDF4	NM_016176	chr1:1142151-1157310	non-template	48	Intron_4	--	Yes	--	Yes	--	--	--	--	--	--	--
G4ST01001148962	chr1:1148963-1148999	-	GGGCAGACAGGCCCTGTCTGAGGGACGGGCGGTGGGG<br>(((((((...))))))).	SDF4	NM_016547	chr1:1142151-1157310	non-template	37	Intron_3	--	Yes	--	Yes	--	--	--	--	--	--	--
G4ST01001148962	chr1:1148963-1148999	-	GGGCAGACAGGCCCTGTCTGAGGGACGGGCGGTGGGG<br>(((((((...))))))).	SDF4	NM_016176	chr1:1142151-1157310	non-template	37	Intron_3	--	Yes	--	Yes	--	--	--	--	--	--	--
G4ST01001157496	chr1:1157497-1157555	+	GGGCCGCCGGCCCGGCGCGGGCGCCATGAAGCTGCTGCGGCGGGCGTGGCGGCGGCGGG<br>.(((((...))))). .(((.....((....))))) (((....)))..	B3GALT6	NM_080605	chr1:1157492-1160283	non-template	59	Exon_1.u	--	Yes	Yes	--	Yes	Yes	--	--	Yes	Yes	--
G4ST01001157496	chr1:1157497-1157555	+	GGGCCGCCGGCCCGGCGCGGGCGCCATGAAGCTGCTGCGGCGGGCGTGGCGGCGGCGGG<br>.(((((...))))). .(((.....((....))))) (((....)))..	SDF4	NM_016547	chr1:1142151-1157310	template	-186	upstream	Yes	--	--	--	--	--	--	--	Yes	Yes	--
G4ST01001157496	chr1:1157497-1157555	+	GGGCCGCCGGCCCGGCGCGGGCGCCATGAAGCTGCTGCGGCGGGCGTGGCGGCGGCGGG<br>.(((((...))))). .(((.....((....))))) (((....)))..	SDF4	NM_016176	chr1:1142151-1157310	template	-186	upstream	Yes	--	--	--	--	--	--	--	Yes	Yes	--
G4ST01001169155	chr1:1169156-1169184	-	GGGAGCTTAGAGCGGGGTGTTGGGTGGGG<br>.(((...))).	FAM132A	NM_001014980	chr1:1167696-1171965	non-template	29	Intron_4	--	Yes	--	Yes	--	--	--	--	Yes	--	--
G4ST01001170786	chr1:1170787-1170830	-	GGGCCAGTCCCCCTGGAAGGGAGGGGGCACCCCACTGCCCAGGG<br>((((.....)))).. ((((......))))..	FAM132A	NM_001014980	chr1:1167696-1171965	non-template	44	Intron_1.f	--	Yes	--	Yes	--	--	--	--	--	--	--
G4ST01001171696	chr1:1171697-1171735	-	GGGCCCTCGCGGGGCAGCTTCGGAGCTGGGGCTCTGGGG<br>.(((((....)))))	FAM132A	NM_001014980	chr1:1167696-1171965	non-template	39	Intron_1.f	--	Yes	--	Yes	--	--	--	--	--	--	--
G4ST01001171704	chr1:1171705-1171746	-	GGGACGCACGTGGGCCCTCGCGGGGCAGCTTCGGAGCTGGGG<br>((....)) .(((((....)))))	FAM132A	NM_001014980	chr1:1167696-1171965	non-template	42	Intron_1.f	--	Yes	--	Yes	--	--	--	--	--	--	--
G4ST01001179997	chr1:1179998-1180037	-	GGGAACCTCGGGGCCCAGGGGTGGAGCTGGCGTCCGCGGG<br>(((((.......)))))	UBE2J2	NM_194315	chr1:1179155-1199097	non-template	40	Exon_8.l	--	Yes	Yes	--	--	--	Yes	Yes	--	--	Yes
G4ST01001179997	chr1:1179998-1180037	-	GGGAACCTCGGGGCCCAGGGGTGGAGCTGGCGTCCGCGGG<br>(((((.......)))))	UBE2J2	NM_194457	chr1:1179155-1199097	non-template	40	Exon_6.l	--	Yes	Yes	--	--	--	Yes	Yes	--	--	Yes
G4ST01001179997	chr1:1179998-1180037	-	GGGAACCTCGGGGCCCAGGGGTGGAGCTGGCGTCCGCGGG<br>(((((.......)))))	UBE2J2	NM_194458	chr1:1179155-1199097	non-template	40	Exon_7.l	--	Yes	Yes	--	--	--	Yes	Yes	--	--	Yes
G4ST01001179997	chr1:1179998-1180037	-	GGGAACCTCGGGGCCCAGGGGTGGAGCTGGCGTCCGCGGG<br>(((((.......)))))	UBE2J2	NM_058167	chr1:1179155-1199097	non-template	40	Exon_7.l	--	Yes	Yes	--	--	--	Yes	Yes	--	--	Yes
G4ST01001181063	chr1:1181064-1181093	-	GGGCCGGGGGCTGGGCCTGTCCCACAGGGG<br>.((((...))))	UBE2J2	NM_194315	chr1:1179155-1199097	non-template	30	Intron_7.l	--	Yes	--	Yes	--	--	--	--	--	--	--
G4ST01001181063	chr1:1181064-1181093	-	GGGCCGGGGGCTGGGCCTGTCCCACAGGGG<br>.((((...))))	UBE2J2	NM_194457	chr1:1179155-1199097	non-template	30	Intron_5.l	--	Yes	--	Yes	--	--	--	--	--	--	--
G4ST01001181063	chr1:1181064-1181093	-	GGGCCGGGGGCTGGGCCTGTCCCACAGGGG<br>.((((...))))	UBE2J2	NM_194458	chr1:1179155-1199097	non-template	30	Intron_6.l	--	Yes	--	Yes	--	--	--	--	--	--	--
G4ST01001181063	chr1:1181064-1181093	-	GGGCCGGGGGCTGGGCCTGTCCCACAGGGG<br>.((((...))))	UBE2J2	NM_058167	chr1:1179155-1199097	non-template	30	Intron_6.l	--	Yes	--	Yes	--	--	--	--	--	--	--
G4ST01001182640	chr1:1182641-1182665	-	GGGAGCCGAGCTGAGGGGGGGGGGG<br>(((...)))..	UBE2J2	NM_194315	chr1:1179155-1199097	non-template	25	Intron_4	--	Yes	--	Yes	--	--	--	--	--	--	Yes
G4ST01001182640	chr1:1182641-1182665	-	GGGAGCCGAGCTGAGGGGGGGGGGG<br>(((...)))..	UBE2J2	NM_194457	chr1:1179155-1199097	non-template	25	Intron_2	--	Yes	--	Yes	--	--	--	--	--	--	Yes
G4ST01001182640	chr1:1182641-1182665	-	GGGAGCCGAGCTGAGGGGGGGGGGG<br>(((...)))..	UBE2J2	NM_194458	chr1:1179155-1199097	non-template	25	Intron_3	--	Yes	--	Yes	--	--	--	--	--	--	Yes
G4ST01001182640	chr1:1182641-1182665	-	GGGAGCCGAGCTGAGGGGGGGGGGG<br>(((...)))..	UBE2J2	NM_058167	chr1:1179155-1199097	non-template	25	Intron_3	--	Yes	--	Yes	--	--	--	--	--	--	Yes
G4ST01001192774	chr1:1192775-1192817	-	GGGGCTGGAGAGAGTCTCTGGTGTGGGGTCCTTAGGGGAGGGG<br>.(((((((...)))))))....	UBE2J2	NM_194315	chr1:1179155-1199097	non-template	43	Intron_2	--	Yes	--	Yes	--	--	--	--	--	--	--
G4ST01001192774	chr1:1192775-1192817	-	GGGGCTGGAGAGAGTCTCTGGTGTGGGGTCCTTAGGGGAGGGG<br>.(((((((...)))))))....	UBE2J2	NM_194457	chr1:1179155-1199097	non-template	43	Intron_1.f	--	Yes	--	Yes	--	--	--	--	--	--	--
G4ST01001192774	chr1:1192775-1192817	-	GGGGCTGGAGAGAGTCTCTGGTGTGGGGTCCTTAGGGGAGGGG<br>.(((((((...)))))))....	UBE2J2	NM_194458	chr1:1179155-1199097	non-template	43	Intron_2	--	Yes	--	Yes	--	--	--	--	--	--	--
G4ST01001192774	chr1:1192775-1192817	-	GGGGCTGGAGAGAGTCTCTGGTGTGGGGTCCTTAGGGGAGGGG<br>.(((((((...)))))))....	UBE2J2	NM_058167	chr1:1179155-1199097	non-template	43	Intron_2	--	Yes	--	Yes	--	--	--	--	--	--	--
G4ST01001192835	chr1:1192836-1192881	-	GGGGCTGGAGAGAATCTCCGGTGTGGGGCCCTTGAGGGGGGCGGGG<br>.(((((((...)))))))....	UBE2J2	NM_194315	chr1:1179155-1199097	non-template	46	Intron_2	--	Yes	--	Yes	--	--	--	--	--	--	--
G4ST01001192835	chr1:1192836-1192881	-	GGGGCTGGAGAGAATCTCCGGTGTGGGGCCCTTGAGGGGGGCGGGG<br>.(((((((...)))))))....	UBE2J2	NM_194457	chr1:1179155-1199097	non-template	46	Intron_1.f	--	Yes	--	Yes	--	--	--	--	--	--	--
G4ST01001192835	chr1:1192836-1192881	-	GGGGCTGGAGAGAATCTCCGGTGTGGGGCCCTTGAGGGGGGCGGGG<br>.(((((((...)))))))....	UBE2J2	NM_194458	chr1:1179155-1199097	non-template	46	Intron_2	--	Yes	--	Yes	--	--	--	--	--	--	--
G4ST01001192835	chr1:1192836-1192881	-	GGGGCTGGAGAGAATCTCCGGTGTGGGGCCCTTGAGGGGGGCGGGG<br>.(((((((...)))))))....	UBE2J2	NM_058167	chr1:1179155-1199097	non-template	46	Intron_2	--	Yes	--	Yes	--	--	--	--	--	--	--
G4ST01001199292	chr1:1199293-1199325	-	GGGGAGGGAAGCGCGCGGCTTCGGGGTCTGGGG<br>((((.....))))..	SCNN1D	NR_037668	chr1:1200501-1217272	template	-1175	upstream	Yes	--	--	--	--	--	--	--	Yes	Yes	--
G4ST01001199292	chr1:1199293-1199325	-	GGGGAGGGAAGCGCGCGGCTTCGGGGTCTGGGG<br>((((.....))))..	SCNN1D	NM_001130413	chr1:1200501-1217272	template	-1175	upstream	Yes	--	--	--	--	--	--	--	Yes	Yes	--
G4ST01001199292	chr1:1199293-1199325	-	GGGGAGGGAAGCGCGCGGCTTCGGGGTCTGGGG<br>((((.....))))..	UBE2J2	NM_194315	chr1:1179155-1199097	non-template	-195	upstream	Yes	--	--	--	--	--	--	--	Yes	Yes	--
G4ST01001199292	chr1:1199293-1199325	-	GGGGAGGGAAGCGCGCGGCTTCGGGGTCTGGGG<br>((((.....))))..	UBE2J2	NM_194457	chr1:1179155-1199097	non-template	-195	upstream	Yes	--	--	--	--	--	--	--	Yes	Yes	--
G4ST01001199292	chr1:1199293-1199325	-	GGGGAGGGAAGCGCGCGGCTTCGGGGTCTGGGG<br>((((.....))))..	UBE2J2	NM_194458	chr1:1179155-1199097	non-template	-195	upstream	Yes	--	--	--	--	--	--	--	Yes	Yes	--
G4ST01001199292	chr1:1199293-1199325	-	GGGGAGGGAAGCGCGCGGCTTCGGGGTCTGGGG<br>((((.....))))..	UBE2J2	NM_058167	chr1:1179155-1199097	non-template	-195	upstream	Yes	--	--	--	--	--	--	--	Yes	Yes	--
G4ST01001199299	chr1:1199300-1199339	-	GGGCCGTGGCACATGGGGAGGGAAGCGCGCGGCTTCGGGG<br>.((((...)))) ((((.....))))..	SCNN1D	NR_037668	chr1:1200501-1217272	template	-1161	upstream	Yes	--	--	--	--	--	--	--	Yes	Yes	--
G4ST01001199299	chr1:1199300-1199339	-	GGGCCGTGGCACATGGGGAGGGAAGCGCGCGGCTTCGGGG<br>.((((...)))) ((((.....))))..	SCNN1D	NM_001130413	chr1:1200501-1217272	template	-1161	upstream	Yes	--	--	--	--	--	--	--	Yes	Yes	--
G4ST01001199299	chr1:1199300-1199339	-	GGGCCGTGGCACATGGGGAGGGAAGCGCGCGGCTTCGGGG<br>.((((...)))) ((((.....))))..	UBE2J2	NM_194315	chr1:1179155-1199097	non-template	-202	upstream	Yes	--	--	--	--	--	--	--	Yes	Yes	--
G4ST01001199299	chr1:1199300-1199339	-	GGGCCGTGGCACATGGGGAGGGAAGCGCGCGGCTTCGGGG<br>.((((...)))) ((((.....))))..	UBE2J2	NM_194457	chr1:1179155-1199097	non-template	-202	upstream	Yes	--	--	--	--	--	--	--	Yes	Yes	--
G4ST01001199299	chr1:1199300-1199339	-	GGGCCGTGGCACATGGGGAGGGAAGCGCGCGGCTTCGGGG<br>.((((...)))) ((((.....))))..	UBE2J2	NM_194458	chr1:1179155-1199097	non-template	-202	upstream	Yes	--	--	--	--	--	--	--	Yes	Yes	--
G4ST01001199299	chr1:1199300-1199339	-	GGGCCGTGGCACATGGGGAGGGAAGCGCGCGGCTTCGGGG<br>.((((...)))) ((((.....))))..	UBE2J2	NM_058167	chr1:1179155-1199097	non-template	-202	upstream	Yes	--	--	--	--	--	--	--	Yes	Yes	--
G4ST01001199482	chr1:1199483-1199520	+	GGGCCCCTGGAGGCTCAGTGGAGGGTTGGGCTCCAGGG<br>((.(((......))).)).	SCNN1D	NR_037668	chr1:1200501-1217272	non-template	-980	upstream	Yes	--	--	--	--	--	--	Yes	Yes	Yes	--
G4ST01001199482	chr1:1199483-1199520	+	GGGCCCCTGGAGGCTCAGTGGAGGGTTGGGCTCCAGGG<br>((.(((......))).)).	SCNN1D	NM_001130413	chr1:1200501-1217272	non-template	-980	upstream	Yes	--	--	--	--	--	--	Yes	Yes	Yes	--

G4ST01001199482	chr1:1199483-1199520	+	GGGCCCCTGGAGGCTCAGTGGAGGGTTGGGCTCCAGGG<br>((.(((......))).)).	UBE2J2	NM_194315	chr1:1179155-1199097	template	-385	upstream	Yes	--	--	--	--	--	--	Yes	Yes	Yes	--
G4ST01001199482	chr1:1199483-1199520	+	GGGCCCCTGGAGGCTCAGTGGAGGGTTGGGCTCCAGGG<br>((.(((......))).)).	UBE2J2	NM_194457	chr1:1179155-1199097	template	-385	upstream	Yes	--	--	--	--	--	--	Yes	Yes	Yes	--
G4ST01001199482	chr1:1199483-1199520	+	GGGCCCCTGGAGGCTCAGTGGAGGGTTGGGCTCCAGGG<br>((.(((......))).)).	UBE2J2	NM_194458	chr1:1179155-1199097	template	-385	upstream	Yes	--	--	--	--	--	--	Yes	Yes	Yes	--
G4ST01001199482	chr1:1199483-1199520	+	GGGCCCCTGGAGGCTCAGTGGAGGGTTGGGCTCCAGGG<br>((.(((......))).)).	UBE2J2	NM_058167	chr1:1179155-1199097	template	-385	upstream	Yes	--	--	--	--	--	--	Yes	Yes	Yes	--
G4ST01001207602	chr1:1207603-1207642	-	GGGTCCCTGGGCTTGGCTGGGGGAGCCCAGCCCCACAGGG<br>((.((...)).))...	SCNN1D	NR_037668	chr1:1200501-1217272	template	40	Intron_6	--	Yes	--	Yes	--	--	--	Yes	Yes	--	--
G4ST01001207602	chr1:1207603-1207642	-	GGGTCCCTGGGCTTGGCTGGGGGAGCCCAGCCCCACAGGG<br>((.((...)).))...	SCNN1D	NM_001130413	chr1:1200501-1217272	template	40	Intron_6	--	Yes	--	Yes	--	--	--	Yes	Yes	--	--
G4ST01001211802	chr1:1211803-1211829	+	GGGAAGGTGGGAGGGGCTGCGAGCGGG<br>(((...)))	SCNN1D	NR_037668	chr1:1200501-1217272	non-template	27	Intron_8	--	Yes	--	Yes	--	--	--	--	--	--	--
G4ST01001211802	chr1:1211803-1211829	+	GGGAAGGTGGGAGGGGCTGCGAGCGGG<br>(((...)))	SCNN1D	NM_001130413	chr1:1200501-1217272	non-template	27	Intron_9	--	Yes	--	Yes	--	--	--	--	--	--	--
G4ST01001211810	chr1:1211811-1211834	+	GGGAGGGGCTGCGAGCGGGCTGGG<br>(((...)))	SCNN1D	NR_037668	chr1:1200501-1217272	non-template	24	Intron_8	--	Yes	--	Yes	--	--	--	--	--	--	--
G4ST01001211810	chr1:1211811-1211834	+	GGGAGGGGCTGCGAGCGGGCTGGG<br>(((...)))	SCNN1D	NM_001130413	chr1:1200501-1217272	non-template	24	Intron_9	--	Yes	--	Yes	--	--	--	--	--	--	--
G4ST01001211814	chr1:1211815-1211842	+	GGGGCTGCGAGCGGGCTGGGCTCCGGGG<br>(((...)))	SCNN1D	NR_037668	chr1:1200501-1217272	non-template	28	Intron_8	--	Yes	--	Yes	--	--	--	--	--	--	--
G4ST01001211814	chr1:1211815-1211842	+	GGGGCTGCGAGCGGGCTGGGCTCCGGGG<br>(((...)))	SCNN1D	NM_001130413	chr1:1200501-1217272	non-template	28	Intron_9	--	Yes	--	Yes	--	--	--	--	--	--	--
G4ST01001217881	chr1:1217882-1217924	+	GGGGGAGGCCCTGCCTCCCTGCCGGGCACCTTTGGGGAAGGGG<br>((((((...))))))).....	ACAP3	NM_030649	chr1:1217627-1233132	template	43	Exon_24.l	--	Yes	Yes	--	--	--	Yes	--	--	--	--
G4ST01001219209	chr1:1219210-1219241	+	GGGGCGGGGCGGGGCGAGTGTGGTCGCGGGGG<br>((((......))))..	ACAP3	NM_030649	chr1:1217627-1233132	template	32	Intron_22	--	Yes	--	Yes	--	--	--	Yes	Yes	Yes	Yes
G4ST01001221563	chr1:1221564-1221592	-	GGGGGTGGGGGTTGGGCCGGAGCTGGGGG<br>(((....)))	ACAP3	NM_030649	chr1:1217627-1233132	non-template	29	Intron_15	--	Yes	--	Yes	--	--	--	--	--	--	Yes
G4ST01001224995	chr1:1224996-1225028	+	GGGTGTGCCGGGCATGGGGTGAGGACGCAGGGG<br>(((....)))..	ACAP3	NM_030649	chr1:1217627-1233132	template	33	Intron_9	--	Yes	--	Yes	--	--	--	--	Yes	--	Yes
G4ST01001232894	chr1:1232895-1232922	-	GGGCCGGGCCGCGGCCGCCGGGGAGGGG<br>...(((...)))	PUSL1	NM_153339	chr1:1233857-1236920	template	-934	upstream	Yes	--	--	--	--	--	--	Yes	Yes	Yes	--
G4ST01001232894	chr1:1232895-1232922	-	GGGCCGGGCCGCGGCCGCCGGGGAGGGG<br>...(((...)))	ACAP3	NM_030649	chr1:1217627-1233132	non-template	28	Intron_1.f	--	Yes	--	Yes	--	--	--	Yes	Yes	Yes	--
G4ST01001232899	chr1:1232900-1232937	-	GGGGAGGTCAGCGCCGGGCCGGGCCGCGGCCGCCGGGG<br>..(((....))) ...(((...)))	PUSL1	NM_153339	chr1:1233857-1236920	template	-919	upstream	Yes	--	--	--	--	--	--	Yes	Yes	Yes	--
G4ST01001232899	chr1:1232900-1232937	-	GGGGAGGTCAGCGCCGGGCCGGGCCGCGGCCGCCGGGG<br>..(((....))) ...(((...)))	ACAP3	NM_030649	chr1:1217627-1233132	non-template	38	Intron_1.f	--	Yes	--	Yes	--	--	--	Yes	Yes	Yes	--
G4ST01001232914	chr1:1232915-1232942	-	GGGGTGGGGAGGTCAGCGCCGGGCCGGG<br>..(((....)))	PUSL1	NM_153339	chr1:1233857-1236920	template	-914	upstream	Yes	--	--	--	--	--	--	Yes	Yes	Yes	--
G4ST01001232914	chr1:1232915-1232942	-	GGGGTGGGGAGGTCAGCGCCGGGCCGGG<br>..(((....)))	ACAP3	NM_030649	chr1:1217627-1233132	non-template	28	Intron_1.f	--	Yes	--	Yes	--	--	--	Yes	Yes	Yes	--
G4ST01001233135	chr1:1233136-1233163	-	GGGCGGGCGCCGAGGCGGCGCGGGCGGG<br>(((((...))))).	PUSL1	NM_153339	chr1:1233857-1236920	template	-693	upstream	Yes	--	--	--	--	--	--	--	Yes	Yes	--
G4ST01001233135	chr1:1233136-1233163	-	GGGCGGGCGCCGAGGCGGCGCGGGCGGG<br>(((((...))))).	ACAP3	NM_030649	chr1:1217627-1233132	non-template	-3	upstream	Yes	--	--	--	--	--	--	--	Yes	Yes	--
G4ST01001233139	chr1:1233140-1233168	-	GGGGCGGGCGGGCGCCGAGGCGGCGCGGG<br>(((((...))))).	PUSL1	NM_153339	chr1:1233857-1236920	template	-688	upstream	Yes	--	--	--	--	--	--	--	Yes	Yes	--
G4ST01001233139	chr1:1233140-1233168	-	GGGGCGGGCGGGCGCCGAGGCGGCGCGGG<br>(((((...))))).	ACAP3	NM_030649	chr1:1217627-1233132	non-template	-7	upstream	Yes	--	--	--	--	--	--	--	Yes	Yes	--
G4ST01001233399	chr1:1233400-1233435	+	GGGGACAGTCGGTGGAAATGTGGGGCGCGGGGAGGG<br>.(((.((....))..))).	PUSL1	NM_153339	chr1:1233857-1236920	non-template	-421	upstream	Yes	--	--	--	--	--	--	Yes	Yes	Yes	Yes
G4ST01001233399	chr1:1233400-1233435	+	GGGGACAGTCGGTGGAAATGTGGGGCGCGGGGAGGG<br>.(((.((....))..))).	ACAP3	NM_030649	chr1:1217627-1233132	template	-267	upstream	Yes	--	--	--	--	--	--	Yes	Yes	Yes	Yes
G4ST01001233986	chr1:1233987-1234016	+	GGGCGCCACGTGGGCCCGGGCGGAGGCGGG<br>((...)).	PUSL1	NM_153339	chr1:1233857-1236920	non-template	30	Intron_1.f	--	Yes	--	Yes	--	--	--	Yes	Yes	Yes	Yes
G4ST01001233986	chr1:1233987-1234016	+	GGGCGCCACGTGGGCCCGGGCGGAGGCGGG<br>((...)).	ACAP3	NM_030649	chr1:1217627-1233132	template	-854	upstream	Yes	--	--	--	--	--	--	Yes	Yes	Yes	Yes
G4ST01001244139	chr1:1244140-1244173	-	GGGGCTGGAGGGGAGATGGGCCAGCTCTGGAGGG<br>(((....))).	CPSF3L	NM_017871	chr1:1236828-1249909	non-template	34	Intron_4	--	Yes	--	Yes	--	--	--	--	Yes	Yes	Yes
G4ST01001249255	chr1:1249256-1249290	+	GGGCTGAGGGTCTGGGGGACTGGAGCGCTGGGGGG<br>...((((....)))).	GLTPD1	NM_001029885	chr1:1250006-1254139	non-template	-715	upstream	Yes	--	--	--	--	--	--	--	--	--	Yes
G4ST01001249255	chr1:1249256-1249290	+	GGGCTGAGGGTCTGGGGGACTGGAGCGCTGGGGGG<br>...((((....)))).	CPSF3L	NM_017871	chr1:1236828-1249909	template	35	Intron_1.f	--	Yes	--	Yes	--	--	--	--	--	--	Yes
G4ST01001249262	chr1:1249263-1249298	+	GGGTCTGGGGGACTGGAGCGCTGGGGGGCTGGGGGG<br>...((((....)))).	GLTPD1	NM_001029885	chr1:1250006-1254139	non-template	-707	upstream	Yes	--	--	--	--	--	--	--	--	--	Yes
G4ST01001249262	chr1:1249263-1249298	+	GGGTCTGGGGGACTGGAGCGCTGGGGGGCTGGGGGG<br>...((((....)))).	CPSF3L	NM_017871	chr1:1236828-1249909	template	36	Intron_1.f	--	Yes	--	Yes	--	--	--	--	--	--	Yes
G4ST01001249268	chr1:1249269-1249305	+	GGGGGACTGGAGCGCTGGGGGGCTGGGGGGCTGAGGG<br>...((((....)))).	GLTPD1	NM_001029885	chr1:1250006-1254139	non-template	-700	upstream	Yes	--	--	--	--	--	--	--	--	--	Yes
G4ST01001249268	chr1:1249269-1249305	+	GGGGGACTGGAGCGCTGGGGGGCTGGGGGGCTGAGGG<br>...((((....)))).	CPSF3L	NM_017871	chr1:1236828-1249909	template	37	Intron_1.f	--	Yes	--	Yes	--	--	--	--	--	--	Yes
G4ST01001249310	chr1:1249311-1249344	+	GGGGCTGAGGGGCTGGGAGGCTGAGAGGCTGGGG<br>((.((...)).)).	GLTPD1	NM_001029885	chr1:1250006-1254139	non-template	-661	upstream	Yes	--	--	--	--	--	--	--	--	--	Yes
G4ST01001249310	chr1:1249311-1249344	+	GGGGCTGAGGGGCTGGGAGGCTGAGAGGCTGGGG<br>((.((...)).)).	CPSF3L	NM_017871	chr1:1236828-1249909	template	34	Intron_1.f	--	Yes	--	Yes	--	--	--	--	--	--	Yes
G4ST01001249318	chr1:1249319-1249354	+	GGGGCTGGGAGGCTGAGAGGCTGGGGAGCTGGGGGG<br>((.((...)).)).	GLTPD1	NM_001029885	chr1:1250006-1254139	non-template	-651	upstream	Yes	--	--	--	--	--	--	--	--	--	Yes
G4ST01001249318	chr1:1249319-1249354	+	GGGGCTGGGAGGCTGAGAGGCTGGGGAGCTGGGGGG<br>((.((...)).)).	CPSF3L	NM_017871	chr1:1236828-1249909	template	36	Intron_1.f	--	Yes	--	Yes	--	--	--	--	--	--	Yes
G4ST01001249324	chr1:1249325-1249361	+	GGGAGGCTGAGAGGCTGGGGAGCTGGGGGGCTGAGGG<br>((.((...)).)).	GLTPD1	NM_001029885	chr1:1250006-1254139	non-template	-644	upstream	Yes	--	--	--	--	--	--	--	--	--	Yes
G4ST01001249324	chr1:1249325-1249361	+	GGGAGGCTGAGAGGCTGGGGAGCTGGGGGGCTGAGGG<br>((.((...)).)).	CPSF3L	NM_017871	chr1:1236828-1249909	template	37	Intron_1.f	--	Yes	--	Yes	--	--	--	--	--	--	Yes
G4ST01001249404	chr1:1249405-1249440	+	GGGGGGCTGAGGGGCTGGGAGGCTGAGAGGCTGGGG<br>((.((...)).)).	GLTPD1	NM_001029885	chr1:1250006-1254139	non-template	-565	upstream	Yes	--	--	--	--	--	--	--	Yes	--	Yes
G4ST01001249404	chr1:1249405-1249440	+	GGGGGGCTGAGGGGCTGGGAGGCTGAGAGGCTGGGG<br>((.((...)).)).	CPSF3L	NM_017871	chr1:1236828-1249909	template	36	Intron_1.f	--	Yes	--	Yes	--	--	--	--	Yes	--	Yes
G4ST01001249414	chr1:1249415-1249450	+	GGGGCTGGGAGGCTGAGAGGCTGGGGAGCTGGGGGG<br>((.((...)).)).	GLTPD1	NM_001029885	chr1:1250006-1254139	non-template	-555	upstream	Yes	--	--	--	--	--	--	--	Yes	--	Yes
G4ST01001249414	chr1:1249415-1249450	+	GGGGCTGGGAGGCTGAGAGGCTGGGGAGCTGGGGGG<br>((.((...)).)).	CPSF3L	NM_017871	chr1:1236828-1249909	template	36	Intron_1.f	--	Yes	--	Yes	--	--	--	--	Yes	--	Yes
G4ST01001249420	chr1:1249421-1249457	+	GGGAGGCTGAGAGGCTGGGGAGCTGGGGGGCTGAGGG<br>((.((...)).)).	GLTPD1	NM_001029885	chr1:1250006-1254139	non-template	-548	upstream	Yes	--	--	--	--	--	--	--	Yes	--	Yes
G4ST01001249420	chr1:1249421-1249457	+	GGGAGGCTGAGAGGCTGGGGAGCTGGGGGGCTGAGGG<br>((.((...)).)).	CPSF3L	NM_017871	chr1:1236828-1249909	template	37	Intron_1.f	--	Yes	--	Yes	--	--	--	--	Yes	--	Yes
G4ST01001250235	chr1:1250236-1250268	-	GGGGCGGAGGCCCGGGGTGGTGTTGGGGAGGGG<br>.(((....)))	GLTPD1	NM_001029885	chr1:1250006-1254139	template	33	Exon_1.f	--	Yes	Yes	--	--	Yes	--	Yes	Yes	Yes	--

G4ST01001250235	chr1:1250236-1250268	-	GGGGCGGAGGCCCGGGGTGGTGTTGGGGAGGGG<br>.(((....)))	CPSF3L	NM_017871	chr1:1236828-1249909	non-template	-326	upstream	Yes	--	--	--	--	--	--	Yes	Yes	Yes	--
G4ST01001252397	chr1:1252398-1252444	-	GGGGCAGCCCGCCTGGGGTGCAGGGGACGCTGCCCAGCCTGCGGGGG<br>.(((.....))) ..(((.((...))..))).	GLTPD1	NM_001029885	chr1:1250006-1254139	template	47	Intron_2.l	--	Yes	--	Yes	--	--	--	--	--	--	--
G4ST01001256799	chr1:1256800-1256829	+	GGGTCGGGGTCAGGGTGACCAGGTCTGGGG<br>.(((...)))..	TAS1R3	NM_152228	chr1:1256589-1259707	non-template	30	Intron_1.f	--	Yes	--	Yes	--	--	--	Yes	Yes	--	Yes
G4ST01001261479	chr1:1261480-1261511	-	GGGGGGGCCACCCGGGGGACCCCCTGTCCGGG<br>((((.....))))	DVL1	NM_004421	chr1:1260521-1274355	non-template	32	Exon_15.l	--	Yes	Yes	--	Yes	--	--	--	--	--	--
G4ST01001263266	chr1:1263267-1263301	+	GGGTCCTGGTAGGCAGGCGGGAAGCAGGGTGGGGG<br>.((((.....)))).	DVL1	NM_004421	chr1:1260521-1274355	template	35	Exon_14	--	Yes	Yes	--	Yes	--	--	--	--	--	Yes
G4ST01001263316	chr1:1263317-1263358	+	GGGGTAGCCCTGACCCAGAGGCCAGGGGGCAGCCGGGTGGGG<br>.....((((.((....)).))))	DVL1	NM_004421	chr1:1260521-1274355	template	42	Exon_14	--	Yes	Yes	--	Yes	--	--	--	--	--	Yes
G4ST01001267520	chr1:1267521-1267559	-	GGGGGTTGCTGGCCAACAGGGGAGGGAGGCCTGCCTGGG<br>.((((.....)))).. (((....)))	DVL1	NM_004421	chr1:1260521-1274355	non-template	39	Intron_3	--	Yes	--	Yes	--	--	--	--	--	--	--
G4ST01001273798	chr1:1273799-1273835	-	GGGAGCGCCTGCCGTTGGTGCGGGCTGGGGTCAGGGG<br>.(((((.......)))))	DVL1	NM_004421	chr1:1260521-1274355	non-template	37	Intron_1.f	--	Yes	--	Yes	--	--	--	Yes	Yes	--	--
G4ST01001273805	chr1:1273806-1273845	-	GGGGCTGGCCGGGAGCGCCTGCCGTTGGTGCGGGCTGGGG<br>.(((((.......)))))	DVL1	NM_004421	chr1:1260521-1274355	non-template	40	Intron_1.f	--	Yes	--	Yes	--	--	--	Yes	Yes	--	--
G4ST01001274449	chr1:1274450-1274480	-	GGGGGCGGCGGCCGGGGCCTGGGCGGTCGGG<br>(((....))).	DVL1	NM_004421	chr1:1260521-1274355	non-template	-94	upstream	Yes	--	--	--	--	--	--	--	Yes	Yes	Yes
G4ST01001274457	chr1:1274458-1274487	-	GGGAGCCGGGGGCGGCGGCCGGGGCCTGGG<br>(((....))).	DVL1	NM_004421	chr1:1260521-1274355	non-template	-102	upstream	Yes	--	--	--	--	--	--	--	Yes	Yes	Yes
G4ST01001274761	chr1:1274762-1274789	-	GGGCCCGCGCGCGGGGGCGGGGCCGGGG<br>.(((....)))	DVL1	NM_004421	chr1:1260521-1274355	non-template	-406	upstream	Yes	--	--	--	--	--	--	Yes	Yes	Yes	Yes
G4ST01001279381	chr1:1279382-1279416	+	GGGTGGGGTGAAGGGGGCCCTGGTCCCCAGGAGGG<br>...(((((...))))).	MXRA8	NM_032348	chr1:1277934-1283778	template	35	Intron_7	--	Yes	--	Yes	--	--	--	--	--	Yes	--
G4ST01001279385	chr1:1279386-1279421	+	GGGGTGAAGGGGGCCCTGGTCCCCAGGAGGGCCGGG<br>...(((((...))))).	MXRA8	NM_032348	chr1:1277934-1283778	template	36	Intron_7	--	Yes	--	Yes	--	--	--	--	--	Yes	--
G4ST01001279393	chr1:1279394-1279428	+	GGGGGCCCTGGTCCCCAGGAGGGCCGGGAGGGGGG<br>...(((((...))))).	MXRA8	NM_032348	chr1:1277934-1283778	template	35	Intron_7	--	Yes	--	Yes	--	--	--	--	--	Yes	--
G4ST01001280381	chr1:1280382-1280427	+	GGGGGTGGCCGGGGCTGCGGAGGGGGCGCGGTCAGCGGCGCGCGGG<br>.(((((........)))))	MXRA8	NM_032348	chr1:1277934-1283778	template	46	Intron_4-Exon_5	--	Yes	Yes	Yes	Yes	--	--	Yes	--	--	--
G4ST01001280391	chr1:1280392-1280433	+	GGGGCTGCGGAGGGGGCGCGGTCAGCGGCGCGCGGGCCGGGG<br>.(((((........)))))	MXRA8	NM_032348	chr1:1277934-1283778	template	42	Intron_4-Exon_5	--	Yes	Yes	Yes	Yes	--	--	--	--	--	--
G4ST01001282626	chr1:1282627-1282662	-	GGGGTCTGTGGGGACACAGGGGGTGGTCACCAGGGG<br>(((....)))..	MXRA8	NM_032348	chr1:1277934-1283778	non-template	36	Intron_1.f	--	Yes	--	Yes	--	--	--	Yes	--	--	Yes
G4ST01001324063	chr1:1324064-1324099	+	GGGATCCAGCGCACTGGCGGGCGGGGAGGCCCCGGG<br>..((((....)))).	CCNL2	NM_001039577	chr1:1317531-1324581	template	36	Intron_1.f	--	Yes	--	Yes	--	--	--	Yes	--	--	Yes
G4ST01001324063	chr1:1324064-1324099	+	GGGATCCAGCGCACTGGCGGGCGGGGAGGCCCCGGG<br>..((((....)))).	CCNL2	NM_030937	chr1:1310954-1324581	template	36	Intron_1.f	--	Yes	--	Yes	--	--	--	Yes	--	--	Yes
G4ST01001324063	chr1:1324064-1324099	+	GGGATCCAGCGCACTGGCGGGCGGGGAGGCCCCGGG<br>..((((....)))).	LOC148413	NR_015434	chr1:1324773-1327289	non-template	-673	upstream	Yes	--	--	--	--	--	--	Yes	--	--	Yes
G4ST01001332048	chr1:1332049-1332086	+	GGGGCTGAGGGTGGCCGGGCGGCTGCACACTAGCTGGG<br>..((((.....)))).	MRPL20	NM_017971	chr1:1327139-1332556	template	38	Intron_2	--	Yes	--	Yes	--	--	--	Yes	Yes	Yes	--
G4ST01001332814	chr1:1332815-1332846	+	GGGTCGGGGGCCCCGCGGCCGGGGAGCTCGGG<br>((((....)))).	MRPL20	NM_017971	chr1:1327139-1332556	template	-258	upstream	Yes	--	--	--	--	--	--	--	Yes	Yes	--
G4ST01001332819	chr1:1332820-1332857	+	GGGGGCCCCGCGGCCGGGGAGCTCGGGTGCGGCGCGGG<br>((((....)))). .((...))	MRPL20	NM_017971	chr1:1327139-1332556	template	-263	upstream	Yes	--	--	--	--	--	--	--	Yes	Yes	--
G4ST01001332834	chr1:1332835-1332862	+	GGGGAGCTCGGGTGCGGCGCGGGCGGGG<br>.((...))	MRPL20	NM_017971	chr1:1327139-1332556	template	-278	upstream	Yes	--	--	--	--	--	--	--	Yes	Yes	--
G4ST01001332843	chr1:1332844-1332867	+	GGGTGCGGCGCGGGCGGGGAGGGG<br>.((...))	MRPL20	NM_017971	chr1:1327139-1332556	template	-287	upstream	Yes	--	--	--	--	--	--	--	Yes	Yes	--
G4ST01001332854	chr1:1332855-1332884	+	GGGCGGGGAGGGGCAGGCCGCCCCCTGGGG<br>.((((......))))	MRPL20	NM_017971	chr1:1327139-1332556	template	-298	upstream	Yes	--	--	--	--	--	--	--	Yes	Yes	--
G4ST01001350842	chr1:1350843-1350879	-	GGGGACCTGGGATGCGCGTGAGCCGCAGGGTGTGGGG<br>.(((((....).))))	TMEM88B	NM_001146685	chr1:1351371-1353030	template	-491	upstream	Yes	--	--	--	--	--	--	--	--	--	--
G4ST01001352756	chr1:1352757-1352783	-	GGGCCGTCAGTGGGGGGCGGGGCGGGG<br>(((....))).	TMEM88B	NM_001146685	chr1:1351371-1353030	template	27	Intron_1.u	--	Yes	--	Yes	--	--	--	--	--	--	Yes
G4ST01001352761	chr1:1352762-1352787	-	GGGCGGGCCGTCAGTGGGGGGCGGGG<br>(((....))).	TMEM88B	NM_001146685	chr1:1351371-1353030	template	26	Intron_1.u	--	Yes	--	Yes	--	--	--	--	--	--	Yes
G4ST01001352766	chr1:1352767-1352794	-	GGGGGCCGGGCGGGCCGTCAGTGGGGGG<br>(((....))).	TMEM88B	NM_001146685	chr1:1351371-1353030	template	28	Intron_1.u	--	Yes	--	Yes	--	--	--	--	--	--	Yes
G4ST01001364610	chr1:1364611-1364641	+	GGGCCGGGGGCTTCGGGCCCGGAGTCGGGGG<br>.(((....)))	VWA1	NM_199121	chr1:1360766-1368125	non-template	31	Exon_3.l	--	Yes	Yes	--	--	--	Yes	--	--	--	--
G4ST01001364610	chr1:1364611-1364641	+	GGGCCGGGGGCTTCGGGCCCGGAGTCGGGGG<br>.(((....)))	VWA1	NM_022834	chr1:1360766-1368125	non-template	31	Exon_3.l	--	Yes	Yes	--	Yes	--	--	--	--	--	--
G4ST01001364615	chr1:1364616-1364646	+	GGGGGCTTCGGGCCCGGAGTCGGGGGCTGGG<br>.(((....)))	VWA1	NM_199121	chr1:1360766-1368125	non-template	31	Exon_3.l	--	Yes	Yes	--	--	--	Yes	--	--	--	--
G4ST01001364615	chr1:1364616-1364646	+	GGGGGCTTCGGGCCCGGAGTCGGGGGCTGGG<br>.(((....)))	VWA1	NM_022834	chr1:1360766-1368125	non-template	31	Exon_3.l	--	Yes	Yes	--	Yes	--	--	--	--	--	--
G4ST01001365129	chr1:1365130-1365169	-	GGGCCGGGGTCGCGCGGAGTCCGCGTGGGGAAAGGCCGGG<br>..(((((((...)))))))	VWA1	NM_199121	chr1:1360766-1368125	template	40	Exon_3.l	--	Yes	Yes	--	--	--	Yes	--	--	--	--
G4ST01001365129	chr1:1365130-1365169	-	GGGCCGGGGTCGCGCGGAGTCCGCGTGGGGAAAGGCCGGG<br>..(((((((...)))))))	VWA1	NM_022834	chr1:1360766-1368125	template	40	Exon_3.l	--	Yes	Yes	--	--	--	Yes	--	--	--	--
G4ST01001365139	chr1:1365140-1365175	-	GGGAGAGGGCCGGGGTCGCGCGGAGTCCGCGTGGGG<br>..(((((((...)))))))	VWA1	NM_199121	chr1:1360766-1368125	template	36	Exon_3.l	--	Yes	Yes	--	--	--	Yes	--	--	--	--
G4ST01001365139	chr1:1365140-1365175	-	GGGAGAGGGCCGGGGTCGCGCGGAGTCCGCGTGGGG<br>..(((((((...)))))))	VWA1	NM_022834	chr1:1360766-1368125	template	36	Exon_3.l	--	Yes	Yes	--	--	--	Yes	--	--	--	--
G4ST01001367138	chr1:1367139-1367181	-	GGGGAGGCAGAGCCTCTGCCAGACGGGTATGTGGGGAGCAGGG<br>..((((((...))))))....	VWA1	NM_199121	chr1:1360766-1368125	template	43	Exon_3.l	--	Yes	Yes	--	--	--	Yes	--	Yes	--	--
G4ST01001367138	chr1:1367139-1367181	-	GGGGAGGCAGAGCCTCTGCCAGACGGGTATGTGGGGAGCAGGG<br>..((((((...))))))....	VWA1	NM_022834	chr1:1360766-1368125	template	43	Exon_3.l	--	Yes	Yes	--	--	--	Yes	--	Yes	--	--
G4ST01001367145	chr1:1367146-1367188	-	GGGCAGAGGGGAGGCAGAGCCTCTGCCAGACGGGTATGTGGGG<br>..((((((...))))))....	VWA1	NM_199121	chr1:1360766-1368125	template	43	Exon_3.l	--	Yes	Yes	--	--	--	Yes	--	Yes	--	--
G4ST01001367145	chr1:1367146-1367188	-	GGGCAGAGGGGAGGCAGAGCCTCTGCCAGACGGGTATGTGGGG<br>..((((((...))))))....	VWA1	NM_022834	chr1:1360766-1368125	template	43	Exon_3.l	--	Yes	Yes	--	--	--	Yes	--	Yes	--	--
G4ST01001381999	chr1:1382000-1382037	-	GGGTGCCCGGGCTGTGCCCTGCGCGGGGTCACTGTGGG<br>..((((...)))).	ATAD3C	NM_001039211	chr1:1374932-1395401	template	38	Intron_7	--	Yes	--	Yes	--	--	--	--	--	--	--
G4ST01001384478	chr1:1384479-1384507	+	GGGAGCCCCTCGGGTCCTGGGCCCCCGGG<br>((...)).	ATAD3C	NM_001039211	chr1:1374932-1395401	non-template	29	Intron_9	--	Yes	--	Yes	--	--	--	--	--	--	Yes
G4ST01001396833	chr1:1396834-1396868	-	GGGCGGAGGGCGGGGGGCGTGGTGAAGCGTCTGGG<br>.((((((......))))).	ATAD3B	NM_031921	chr1:1397027-1421445	template	-158	upstream	Yes	--	--	--	--	--	--	Yes	Yes	Yes	Yes
G4ST01001412318	chr1:1412319-1412356	-	GGGTGCCCGGGCTGTGCCCTGCGCGGGGTCACTGTGGG<br>..((((...)))).	ATAD3B	NM_031921	chr1:1397027-1421445	template	38	Intron_11	--	Yes	--	Yes	--	--	--	--	--	--	--
G4ST01001436790	chr1:1436791-1436833	-	GGGGCCAGCGAGCTCCTCTTGCGGGGCGTGGACGGGGCCGGGG<br>....(((((.....))))).	ATAD3A	NM_001170535	chr1:1437386-1459930	template	-552	upstream	Yes	--	--	--	--	--	--	--	Yes	Yes	Yes
G4ST01001436790	chr1:1436791-1436833	-	GGGGCCAGCGAGCTCCTCTTGCGGGGCGTGGACGGGGCCGGGG<br>....(((((.....))))).	ATAD3A	NM_018188	chr1:1437386-1459930	template	-552	upstream	Yes	--	--	--	--	--	--	--	Yes	Yes	Yes
G4ST01001436790	chr1:1436791-1436833	-	GGGGCCAGCGAGCTCCTCTTGCGGGGCGTGGACGGGGCCGGGG<br>....(((((.....))))).	ATAD3A	NM_001170536	chr1:1437773-1459930	template	-939	upstream	Yes	--	--	--	--	--	--	--	Yes	Yes	Yes

G4ST01001447828	chr1:1447829-1447863	+	GGGATTCGGGGCCGGGAATTCGCGTTCCTGTGGGG<br>....((((....))))	ATAD3A	NM_001170535	chr1:1437386-1459930	non-template	35	Intron_7	--	Yes	--	Yes	--	--	--	--	--	--	--
G4ST01001447828	chr1:1447829-1447863	+	GGGATTCGGGGCCGGGAATTCGCGTTCCTGTGGGG<br>....((((....))))	ATAD3A	NM_018188	chr1:1437386-1459930	non-template	35	Intron_7	--	Yes	--	Yes	--	--	--	--	--	--	--
G4ST01001447828	chr1:1447829-1447863	+	GGGATTCGGGGCCGGGAATTCGCGTTCCTGTGGGG<br>....((((....))))	ATAD3A	NM_001170536	chr1:1437773-1459930	non-template	35	Intron_7	--	Yes	--	Yes	--	--	--	--	--	--	--
G4ST01001450047	chr1:1450048-1450085	-	GGGTGCCCGGGCTGTGCCCTGCGCGGGGTCACTGTGGG<br>..((((...)))).	ATAD3A	NM_001170535	chr1:1437386-1459930	template	38	Intron_11	--	Yes	--	Yes	--	--	--	--	--	--	--
G4ST01001450047	chr1:1450048-1450085	-	GGGTGCCCGGGCTGTGCCCTGCGCGGGGTCACTGTGGG<br>..((((...)))).	ATAD3A	NM_018188	chr1:1437386-1459930	template	38	Intron_11	--	Yes	--	Yes	--	--	--	--	--	--	--
G4ST01001450047	chr1:1450048-1450085	-	GGGTGCCCGGGCTGTGCCCTGCGCGGGGTCACTGTGGG<br>..((((...)))).	ATAD3A	NM_001170536	chr1:1437773-1459930	template	38	Intron_11	--	Yes	--	Yes	--	--	--	--	--	--	--
G4ST01001451778	chr1:1451779-1451807	+	GGGAGCCCCTCGGGTCCTGGGCCCCCGGG<br>((...)).	ATAD3A	NM_001170535	chr1:1437386-1459930	non-template	29	Intron_13	--	Yes	--	Yes	--	--	--	--	--	--	Yes
G4ST01001451778	chr1:1451779-1451807	+	GGGAGCCCCTCGGGTCCTGGGCCCCCGGG<br>((...)).	ATAD3A	NM_018188	chr1:1437386-1459930	non-template	29	Intron_13	--	Yes	--	Yes	--	--	--	--	--	--	Yes
G4ST01001451778	chr1:1451779-1451807	+	GGGAGCCCCTCGGGTCCTGGGCCCCCGGG<br>((...)).	ATAD3A	NM_001170536	chr1:1437773-1459930	non-template	29	Intron_13	--	Yes	--	Yes	--	--	--	--	--	--	Yes
G4ST01001457915	chr1:1457916-1457944	+	GGGCCCTGGGTGGGGTGCAGCCACTCGGG<br>(((....)))..	ATAD3A	NM_001170535	chr1:1437386-1459930	non-template	29	Intron_15.l	--	Yes	--	Yes	--	--	--	--	--	--	--
G4ST01001457915	chr1:1457916-1457944	+	GGGCCCTGGGTGGGGTGCAGCCACTCGGG<br>(((....)))..	ATAD3A	NM_018188	chr1:1437386-1459930	non-template	29	Intron_15.l	--	Yes	--	Yes	--	--	--	--	--	--	--
G4ST01001457915	chr1:1457916-1457944	+	GGGCCCTGGGTGGGGTGCAGCCACTCGGG<br>(((....)))..	ATAD3A	NM_001170536	chr1:1437773-1459930	non-template	29	Intron_15.l	--	Yes	--	Yes	--	--	--	--	--	--	--
G4ST01001460251	chr1:1460252-1460294	-	GGGGGGCAGGTGGGGGCAGGGGCGGCGAGGCGCGCGGCCCGGG<br>((.(((.....))).))..	C1orf70	NM_001114748	chr1:1460021-1465603	non-template	43	Exon_4.l	--	Yes	Yes	--	--	--	Yes	--	--	--	Yes
G4ST01001465346	chr1:1465347-1465375	+	GGGGAGGGGAGCGGAGGCCGGGGTGGGGG<br>...(((...)))	C1orf70	NM_001114748	chr1:1460021-1465603	template	29	Intron_1.f	--	Yes	--	Yes	--	--	--	--	--	--	--
G4ST01001466025	chr1:1466026-1466061	-	GGGGCGGGACGCGAAGCCGAGGCTCCCGGGGAGGGG<br>...((.((((....)))..))	C1orf70	NM_001114748	chr1:1460021-1465603	non-template	-422	upstream	Yes	--	--	--	--	--	--	Yes	--	--	--
G4ST01001468190	chr1:1468191-1468215	-	GGGAGCCAGGCGGGCGGGCGCTGGG<br>.((...))	SSU72	NM_014188	chr1:1466916-1500125	non-template	25	Intron_4.l	--	Yes	--	Yes	--	--	--	--	--	--	--
G4ST01001541304	chr1:1541305-1541336	+	GGGCGGGACGGAAACGTCCGGGCAAGTTGGGG<br>(((....)))..	MIB2	NR_033183	chr1:1540658-1555853	non-template	32	Intron_1.f	--	Yes	--	Yes	--	--	--	Yes	Yes	Yes	--
G4ST01001541304	chr1:1541305-1541336	+	GGGCGGGACGGAAACGTCCGGGCAAGTTGGGG<br>(((....)))..	MIB2	NM_080875	chr1:1540658-1555853	non-template	32	Intron_1.f	--	Yes	--	Yes	--	--	--	Yes	Yes	Yes	--
G4ST01001541304	chr1:1541305-1541336	+	GGGCGGGACGGAAACGTCCGGGCAAGTTGGGG<br>(((....)))..	MIB2	NM_001170689	chr1:1541108-1555853	non-template	32	Exon_1.f	--	Yes	Yes	--	--	Yes	--	Yes	Yes	Yes	--
G4ST01001541304	chr1:1541305-1541336	+	GGGCGGGACGGAAACGTCCGGGCAAGTTGGGG<br>(((....)))..	MIB2	NM_001170688	chr1:1540658-1555853	non-template	32	Intron_1.f	--	Yes	--	Yes	--	--	--	Yes	Yes	Yes	--
G4ST01001541304	chr1:1541305-1541336	+	GGGCGGGACGGAAACGTCCGGGCAAGTTGGGG<br>(((....)))..	MIB2	NM_001170686	chr1:1540658-1555853	non-template	32	Intron_1.f	--	Yes	--	Yes	--	--	--	Yes	Yes	Yes	--
G4ST01001541304	chr1:1541305-1541336	+	GGGCGGGACGGAAACGTCCGGGCAAGTTGGGG<br>(((....)))..	MIB2	NM_001170687	chr1:1540658-1555853	non-template	32	Intron_1.f	--	Yes	--	Yes	--	--	--	Yes	Yes	Yes	--
G4ST01001548429	chr1:1548430-1548473	-	GGGCGCCGTGCCAGCCTGGCGCGGGAACTGCAGGGTGGTCTGGG<br>....((((((...))))))	MIB2	NR_033183	chr1:1540658-1555853	template	44	Intron_2	--	Yes	--	Yes	--	--	--	--	--	--	Yes
G4ST01001548429	chr1:1548430-1548473	-	GGGCGCCGTGCCAGCCTGGCGCGGGAACTGCAGGGTGGTCTGGG<br>....((((((...))))))	MIB2	NM_080875	chr1:1540658-1555853	template	44	Intron_2	--	Yes	--	Yes	--	--	--	--	--	--	Yes
G4ST01001548429	chr1:1548430-1548473	-	GGGCGCCGTGCCAGCCTGGCGCGGGAACTGCAGGGTGGTCTGGG<br>....((((((...))))))	MIB2	NM_001170689	chr1:1541108-1555853	template	44	Intron_2	--	Yes	--	Yes	--	--	--	--	--	--	Yes
G4ST01001548429	chr1:1548430-1548473	-	GGGCGCCGTGCCAGCCTGGCGCGGGAACTGCAGGGTGGTCTGGG<br>....((((((...))))))	MIB2	NM_001170688	chr1:1540658-1555853	template	44	Intron_2	--	Yes	--	Yes	--	--	--	--	--	--	Yes
G4ST01001548429	chr1:1548430-1548473	-	GGGCGCCGTGCCAGCCTGGCGCGGGAACTGCAGGGTGGTCTGGG<br>....((((((...))))))	MIB2	NM_001170686	chr1:1540658-1555853	template	44	Intron_2	--	Yes	--	Yes	--	--	--	--	--	--	Yes
G4ST01001548429	chr1:1548430-1548473	-	GGGCGCCGTGCCAGCCTGGCGCGGGAACTGCAGGGTGGTCTGGG<br>....((((((...))))))	MIB2	NM_001170687	chr1:1540658-1555853	template	44	Intron_2	--	Yes	--	Yes	--	--	--	--	--	--	Yes
G4ST01001548438	chr1:1548439-1548482	-	GGGAAAGGCGGGCGCCGTGCCAGCCTGGCGCGGGAACTGCAGGG<br>....((((((...))))))	MIB2	NR_033183	chr1:1540658-1555853	template	44	Intron_2	--	Yes	--	Yes	--	--	--	--	--	--	Yes
G4ST01001548438	chr1:1548439-1548482	-	GGGAAAGGCGGGCGCCGTGCCAGCCTGGCGCGGGAACTGCAGGG<br>....((((((...))))))	MIB2	NM_080875	chr1:1540658-1555853	template	44	Intron_2	--	Yes	--	Yes	--	--	--	--	--	--	Yes
G4ST01001548438	chr1:1548439-1548482	-	GGGAAAGGCGGGCGCCGTGCCAGCCTGGCGCGGGAACTGCAGGG<br>....((((((...))))))	MIB2	NM_001170689	chr1:1541108-1555853	template	44	Intron_2	--	Yes	--	Yes	--	--	--	--	--	--	Yes
G4ST01001548438	chr1:1548439-1548482	-	GGGAAAGGCGGGCGCCGTGCCAGCCTGGCGCGGGAACTGCAGGG<br>....((((((...))))))	MIB2	NM_001170688	chr1:1540658-1555853	template	44	Intron_2	--	Yes	--	Yes	--	--	--	--	--	--	Yes
G4ST01001548438	chr1:1548439-1548482	-	GGGAAAGGCGGGCGCCGTGCCAGCCTGGCGCGGGAACTGCAGGG<br>....((((((...))))))	MIB2	NM_001170686	chr1:1540658-1555853	template	44	Intron_2	--	Yes	--	Yes	--	--	--	--	--	--	Yes
G4ST01001548438	chr1:1548439-1548482	-	GGGAAAGGCGGGCGCCGTGCCAGCCTGGCGCGGGAACTGCAGGG<br>....((((((...))))))	MIB2	NM_001170687	chr1:1540658-1555853	template	44	Intron_2	--	Yes	--	Yes	--	--	--	--	--	--	Yes
G4ST01001548999	chr1:1549000-1549030	-	GGGGTGCCGGACGCCTGGGGGTGGGGTGGGG<br>...((((....))))	MIB2	NR_033183	chr1:1540658-1555853	template	31	Intron_3-Exon_4	--	Yes	Yes	Yes	--	--	--	--	--	--	Yes
G4ST01001548999	chr1:1549000-1549030	-	GGGGTGCCGGACGCCTGGGGGTGGGGTGGGG<br>...((((....))))	MIB2	NM_080875	chr1:1540658-1555853	template	31	Intron_3-Exon_4	--	Yes	Yes	Yes	Yes	--	--	--	--	--	Yes
G4ST01001548999	chr1:1549000-1549030	-	GGGGTGCCGGACGCCTGGGGGTGGGGTGGGG<br>...((((....))))	MIB2	NM_001170689	chr1:1541108-1555853	template	31	Intron_3-Exon_4	--	Yes	Yes	Yes	Yes	--	--	--	--	--	Yes
G4ST01001548999	chr1:1549000-1549030	-	GGGGTGCCGGACGCCTGGGGGTGGGGTGGGG<br>...((((....))))	MIB2	NM_001170688	chr1:1540658-1555853	template	31	Intron_3-Exon_4	--	Yes	Yes	Yes	Yes	--	--	--	--	--	Yes
G4ST01001548999	chr1:1549000-1549030	-	GGGGTGCCGGACGCCTGGGGGTGGGGTGGGG<br>...((((....))))	MIB2	NM_001170686	chr1:1540658-1555853	template	31	Intron_3-Exon_4	--	Yes	Yes	Yes	Yes	--	--	--	--	--	Yes
G4ST01001548999	chr1:1549000-1549030	-	GGGGTGCCGGACGCCTGGGGGTGGGGTGGGG<br>...((((....))))	MIB2	NM_001170687	chr1:1540658-1555853	template	31	Intron_3-Exon_4	--	Yes	Yes	Yes	Yes	--	--	--	--	--	Yes
G4ST01001551178	chr1:1551179-1551200	+	GGGCGGGAGGGAGGCGGCTGGG<br>((....))	MIB2	NR_033183	chr1:1540658-1555853	non-template	22	Intron_8	--	Yes	--	Yes	--	--	--	--	--	--	--
G4ST01001551178	chr1:1551179-1551200	+	GGGCGGGAGGGAGGCGGCTGGG<br>((....))	MIB2	NM_080875	chr1:1540658-1555853	non-template	22	Intron_8	--	Yes	--	Yes	--	--	--	--	--	--	--
G4ST01001551178	chr1:1551179-1551200	+	GGGCGGGAGGGAGGCGGCTGGG<br>((....))	MIB2	NM_001170689	chr1:1541108-1555853	non-template	22	Intron_7	--	Yes	--	Yes	--	--	--	--	--	--	--
G4ST01001551178	chr1:1551179-1551200	+	GGGCGGGAGGGAGGCGGCTGGG<br>((....))	MIB2	NM_001170688	chr1:1540658-1555853	non-template	22	Intron_7	--	Yes	--	Yes	--	--	--	--	--	--	--
G4ST01001551178	chr1:1551179-1551200	+	GGGCGGGAGGGAGGCGGCTGGG<br>((....))	MIB2	NM_001170686	chr1:1540658-1555853	non-template	22	Intron_8	--	Yes	--	Yes	--	--	--	--	--	--	--
G4ST01001551178	chr1:1551179-1551200	+	GGGCGGGAGGGAGGCGGCTGGG<br>((....))	MIB2	NM_001170687	chr1:1540658-1555853	non-template	22	Intron_8	--	Yes	--	Yes	--	--	--	--	--	--	--
G4ST01001556224	chr1:1556225-1556255	+	GGGTCTGGGGGCCGGGCGCACGCAGGCGGGG<br>(((......)))	MMP23A	NR_002946	chr1:1558022-1559890	non-template	-1766	upstream	Yes	--	--	--	--	--	--	--	--	--	--
G4ST01001556224	chr1:1556225-1556255	+	GGGTCTGGGGGCCGGGCGCACGCAGGCGGGG<br>(((......)))	MMP23B	NM_006983	chr1:1557423-1559893	non-template	-1167	upstream	Yes	--	--	--	--	--	--	--	--	--	--
G4ST01001557625	chr1:1557626-1557652	+	GGGGGGGAGGCCAGGTGCCGGGTTGGG<br>.(((.....)))	MMP23A	NR_002946	chr1:1558022-1559890	non-template	-369	upstream	Yes	--	--	--	--	--	--	--	--	--	--
G4ST01001557625	chr1:1557626-1557652	+	GGGGGGGAGGCCAGGTGCCGGGTTGGG<br>.(((.....)))	MMP23B	NM_006983	chr1:1557423-1559893	non-template	27	Intron_1.f	--	Yes	--	Yes	--	--	--	--	--	--	--

G4ST01001557629	chr1:1557630-1557656	+	GGGAGGCCAGGTGCCGGGTTGGGGGGG<br>.(((.....)))	MMP23A	NR_002946	chr1:1558022-1559890	non-template	-365	upstream	Yes	--	--	--	--	--	--	--	--	--	--
G4ST01001557629	chr1:1557630-1557656	+	GGGAGGCCAGGTGCCGGGTTGGGGGGG<br>.(((.....)))	MMP23B	NM_006983	chr1:1557423-1559893	non-template	27	Intron_1.f	--	Yes	--	Yes	--	--	--	--	--	--	--
G4ST01001558385	chr1:1558386-1558416	-	GGGGCTGAGAGGCCGGGCGCGCAGGGCCGGG<br>(((....))).	MMP23A	NR_002946	chr1:1558022-1559890	template	31	Intron_1.f	--	Yes	--	Yes	--	--	--	--	--	--	Yes
G4ST01001558385	chr1:1558386-1558416	-	GGGGCTGAGAGGCCGGGCGCGCAGGGCCGGG<br>(((....))).	MMP23B	NM_006983	chr1:1557423-1559893	template	31	Intron_3	--	Yes	--	Yes	--	--	--	--	--	--	Yes
G4ST01001558390	chr1:1558391-1558427	-	GGGGGGAGCACGGGGCTGAGAGGCCGGGCGCGCAGGG<br>(((....))).	MMP23A	NR_002946	chr1:1558022-1559890	template	37	Intron_1.f	--	Yes	--	Yes	--	--	--	--	--	--	Yes
G4ST01001558390	chr1:1558391-1558427	-	GGGGGGAGCACGGGGCTGAGAGGCCGGGCGCGCAGGG<br>(((....))).	MMP23B	NM_006983	chr1:1557423-1559893	template	37	Intron_3	--	Yes	--	Yes	--	--	--	--	--	--	Yes
G4ST01001558952	chr1:1558953-1558985	-	GGGCCCCGGGGGTCAGCGCCTGGGAGCCCCGGG<br>(((....))).	MMP23A	NR_002946	chr1:1558022-1559890	template	33	Intron_2	--	Yes	--	Yes	--	--	--	--	--	--	Yes
G4ST01001558952	chr1:1558953-1558985	-	GGGCCCCGGGGGTCAGCGCCTGGGAGCCCCGGG<br>(((....))).	MMP23B	NM_006983	chr1:1557423-1559893	template	33	Intron_4	--	Yes	--	Yes	--	--	--	--	--	--	Yes
G4ST01001559447	chr1:1559448-1559484	-	GGGGGGCGGTGGGGTGGTGGCCACCGTGGGGAAGGGG<br>((((...))))....	MMP23A	NR_002946	chr1:1558022-1559890	template	37	Exon_5	--	Yes	Yes	--	--	--	--	--	--	--	--
G4ST01001559447	chr1:1559448-1559484	-	GGGGGGCGGTGGGGTGGTGGCCACCGTGGGGAAGGGG<br>((((...))))....	MMP23B	NM_006983	chr1:1557423-1559893	template	37	Exon_7	--	Yes	Yes	--	Yes	--	--	--	--	--	--
G4ST01001565553	chr1:1565554-1565592	+	GGGCAGGGGAGTCGGGCACATAGTCGCCCTCTGTCAGGG<br>.(((.((.....)).)))..	CDK11B	NM_033492	chr1:1560963-1645635	template	39	Exon_11	--	Yes	Yes	--	Yes	--	--	--	--	--	Yes
G4ST01001565553	chr1:1565554-1565592	+	GGGCAGGGGAGTCGGGCACATAGTCGCCCTCTGTCAGGG<br>.(((.((.....)).)))..	CDK11B	NM_033489	chr1:1560963-1645635	template	39	Exon_12	--	Yes	Yes	--	Yes	--	--	--	--	--	Yes
G4ST01001565553	chr1:1565554-1565592	+	GGGCAGGGGAGTCGGGCACATAGTCGCCCTCTGTCAGGG<br>.(((.((.....)).)))..	CDK11B	NM_033488	chr1:1560963-1645635	template	39	Exon_12	--	Yes	Yes	--	Yes	--	--	--	--	--	Yes
G4ST01001565553	chr1:1565554-1565592	+	GGGCAGGGGAGTCGGGCACATAGTCGCCCTCTGTCAGGG<br>.(((.((.....)).)))..	CDK11B	NM_033487	chr1:1560963-1645635	template	39	Exon_9	--	Yes	Yes	--	Yes	--	--	--	--	--	Yes
G4ST01001565553	chr1:1565554-1565592	+	GGGCAGGGGAGTCGGGCACATAGTCGCCCTCTGTCAGGG<br>.(((.((.....)).)))..	CDK11B	NM_033486	chr1:1560963-1645635	template	39	Exon_11	--	Yes	Yes	--	Yes	--	--	--	--	--	Yes
G4ST01001565553	chr1:1565554-1565592	+	GGGCAGGGGAGTCGGGCACATAGTCGCCCTCTGTCAGGG<br>.(((.((.....)).)))..	CDK11B	NM_033493	chr1:1560963-1645635	template	39	Exon_11	--	Yes	Yes	--	Yes	--	--	--	--	--	Yes
G4ST01001586582	chr1:1586583-1586613	-	GGGAGGGGACAGAGGGCCCTGGAAGGAAGGG<br>.(((...)))..	SLC35E2B	NM_001110781	chr1:1582802-1614103	non-template	31	Exon_9.l	--	Yes	Yes	--	--	--	Yes	--	--	--	Yes
G4ST01001586582	chr1:1586583-1586613	-	GGGAGGGGACAGAGGGCCCTGGAAGGAAGGG<br>.(((...)))..	CDK11B	NM_033492	chr1:1560963-1645635	non-template	31	Intron_6	--	Yes	--	Yes	--	--	--	--	--	--	Yes
G4ST01001586582	chr1:1586583-1586613	-	GGGAGGGGACAGAGGGCCCTGGAAGGAAGGG<br>.(((...)))..	CDK11B	NM_033489	chr1:1560963-1645635	non-template	31	Intron_7	--	Yes	--	Yes	--	--	--	--	--	--	Yes
G4ST01001586582	chr1:1586583-1586613	-	GGGAGGGGACAGAGGGCCCTGGAAGGAAGGG<br>.(((...)))..	CDK11B	NM_033488	chr1:1560963-1645635	non-template	31	Intron_7	--	Yes	--	Yes	--	--	--	--	--	--	Yes
G4ST01001586582	chr1:1586583-1586613	-	GGGAGGGGACAGAGGGCCCTGGAAGGAAGGG<br>.(((...)))..	CDK11B	NM_033487	chr1:1560963-1645635	non-template	31	Intron_5	--	Yes	--	Yes	--	--	--	--	--	--	Yes
G4ST01001586582	chr1:1586583-1586613	-	GGGAGGGGACAGAGGGCCCTGGAAGGAAGGG<br>.(((...)))..	CDK11B	NM_033486	chr1:1560963-1645635	non-template	31	Intron_6	--	Yes	--	Yes	--	--	--	--	--	--	Yes
G4ST01001586582	chr1:1586583-1586613	-	GGGAGGGGACAGAGGGCCCTGGAAGGAAGGG<br>.(((...)))..	CDK11B	NM_033493	chr1:1560963-1645635	non-template	31	Intron_6	--	Yes	--	Yes	--	--	--	--	--	--	Yes
G4ST01001613057	chr1:1613058-1613098	-	GGGCACAGGGCGTCCCTCCACGGGGTGCCTTTGGCGTTGGG<br>(((......))) .((((...))))..	SLC35E2B	NM_001110781	chr1:1582802-1614103	non-template	41	Intron_1.f	--	Yes	--	Yes	--	--	--	--	--	--	--
G4ST01001613057	chr1:1613058-1613098	-	GGGCACAGGGCGTCCCTCCACGGGGTGCCTTTGGCGTTGGG<br>(((......))) .((((...))))..	CDK11B	NM_033492	chr1:1560963-1645635	non-template	41	Intron_6	--	Yes	--	Yes	--	--	--	--	--	--	--
G4ST01001613057	chr1:1613058-1613098	-	GGGCACAGGGCGTCCCTCCACGGGGTGCCTTTGGCGTTGGG<br>(((......))) .((((...))))..	CDK11B	NM_033489	chr1:1560963-1645635	non-template	41	Intron_7	--	Yes	--	Yes	--	--	--	--	--	--	--
G4ST01001613057	chr1:1613058-1613098	-	GGGCACAGGGCGTCCCTCCACGGGGTGCCTTTGGCGTTGGG<br>(((......))) .((((...))))..	CDK11B	NM_033488	chr1:1560963-1645635	non-template	41	Intron_7	--	Yes	--	Yes	--	--	--	--	--	--	--
G4ST01001613057	chr1:1613058-1613098	-	GGGCACAGGGCGTCCCTCCACGGGGTGCCTTTGGCGTTGGG<br>(((......))) .((((...))))..	CDK11B	NM_033487	chr1:1560963-1645635	non-template	41	Intron_5	--	Yes	--	Yes	--	--	--	--	--	--	--
G4ST01001613057	chr1:1613058-1613098	-	GGGCACAGGGCGTCCCTCCACGGGGTGCCTTTGGCGTTGGG<br>(((......))) .((((...))))..	CDK11B	NM_033486	chr1:1560963-1645635	non-template	41	Intron_6	--	Yes	--	Yes	--	--	--	--	--	--	--
G4ST01001613057	chr1:1613058-1613098	-	GGGCACAGGGCGTCCCTCCACGGGGTGCCTTTGGCGTTGGG<br>(((......))) .((((...))))..	CDK11B	NM_033493	chr1:1560963-1645635	non-template	41	Intron_6	--	Yes	--	Yes	--	--	--	--	--	--	--
G4ST01001613073	chr1:1613074-1613104	-	GGGGGAGGGCACAGGGCGTCCCTCCACGGGG<br>(((......)))	SLC35E2B	NM_001110781	chr1:1582802-1614103	non-template	31	Intron_1.f	--	Yes	--	Yes	--	--	--	--	--	--	--
G4ST01001613073	chr1:1613074-1613104	-	GGGGGAGGGCACAGGGCGTCCCTCCACGGGG<br>(((......)))	CDK11B	NM_033492	chr1:1560963-1645635	non-template	31	Intron_6	--	Yes	--	Yes	--	--	--	--	--	--	--
G4ST01001613073	chr1:1613074-1613104	-	GGGGGAGGGCACAGGGCGTCCCTCCACGGGG<br>(((......)))	CDK11B	NM_033489	chr1:1560963-1645635	non-template	31	Intron_7	--	Yes	--	Yes	--	--	--	--	--	--	--
G4ST01001613073	chr1:1613074-1613104	-	GGGGGAGGGCACAGGGCGTCCCTCCACGGGG<br>(((......)))	CDK11B	NM_033488	chr1:1560963-1645635	non-template	31	Intron_7	--	Yes	--	Yes	--	--	--	--	--	--	--
G4ST01001613073	chr1:1613074-1613104	-	GGGGGAGGGCACAGGGCGTCCCTCCACGGGG<br>(((......)))	CDK11B	NM_033487	chr1:1560963-1645635	non-template	31	Intron_5	--	Yes	--	Yes	--	--	--	--	--	--	--
G4ST01001613073	chr1:1613074-1613104	-	GGGGGAGGGCACAGGGCGTCCCTCCACGGGG<br>(((......)))	CDK11B	NM_033486	chr1:1560963-1645635	non-template	31	Intron_6	--	Yes	--	Yes	--	--	--	--	--	--	--
G4ST01001613073	chr1:1613074-1613104	-	GGGGGAGGGCACAGGGCGTCCCTCCACGGGG<br>(((......)))	CDK11B	NM_033493	chr1:1560963-1645635	non-template	31	Intron_6	--	Yes	--	Yes	--	--	--	--	--	--	--
G4ST01001614698	chr1:1614699-1614744	+	GGGAAAGCTGCTCTGACAGCTGGGCCCGGAGCTGCGGGGGGCGGGG<br>..(((((......))))) ((((......))))	SLC35E2B	NM_001110781	chr1:1582802-1614103	template	-595	upstream	Yes	--	--	--	--	--	--	--	--	--	--
G4ST01001614698	chr1:1614699-1614744	+	GGGAAAGCTGCTCTGACAGCTGGGCCCGGAGCTGCGGGGGGCGGGG<br>..(((((......))))) ((((......))))	CDK11B	NM_033492	chr1:1560963-1645635	template	46	Intron_6	--	Yes	--	Yes	--	--	--	--	--	--	--
G4ST01001614698	chr1:1614699-1614744	+	GGGAAAGCTGCTCTGACAGCTGGGCCCGGAGCTGCGGGGGGCGGGG<br>..(((((......))))) ((((......))))	CDK11B	NM_033489	chr1:1560963-1645635	template	46	Intron_7	--	Yes	--	Yes	--	--	--	--	--	--	--
G4ST01001614698	chr1:1614699-1614744	+	GGGAAAGCTGCTCTGACAGCTGGGCCCGGAGCTGCGGGGGGCGGGG<br>..(((((......))))) ((((......))))	CDK11B	NM_033488	chr1:1560963-1645635	template	46	Intron_7	--	Yes	--	Yes	--	--	--	--	--	--	--
G4ST01001614698	chr1:1614699-1614744	+	GGGAAAGCTGCTCTGACAGCTGGGCCCGGAGCTGCGGGGGGCGGGG<br>..(((((......))))) ((((......))))	CDK11B	NM_033487	chr1:1560963-1645635	template	46	Intron_5	--	Yes	--	Yes	--	--	--	--	--	--	--
G4ST01001614698	chr1:1614699-1614744	+	GGGAAAGCTGCTCTGACAGCTGGGCCCGGAGCTGCGGGGGGCGGGG<br>..(((((......))))) ((((......))))	CDK11B	NM_033486	chr1:1560963-1645635	template	46	Intron_6	--	Yes	--	Yes	--	--	--	--	--	--	--
G4ST01001614698	chr1:1614699-1614744	+	GGGAAAGCTGCTCTGACAGCTGGGCCCGGAGCTGCGGGGGGCGGGG<br>..(((((......))))) ((((......))))	CDK11B	NM_033493	chr1:1560963-1645635	template	46	Intron_6	--	Yes	--	Yes	--	--	--	--	--	--	--
G4ST01001614719	chr1:1614720-1614757	+	GGGCCCGGAGCTGCGGGGGGCGGGGCCGCCGCGCGGGG<br>((((......)))) ..(((...)))	SLC35E2B	NM_001110781	chr1:1582802-1614103	template	-616	upstream	Yes	--	--	--	--	--	--	--	--	--	--
G4ST01001614719	chr1:1614720-1614757	+	GGGCCCGGAGCTGCGGGGGGCGGGGCCGCCGCGCGGGG<br>((((......)))) ..(((...)))	CDK11B	NM_033492	chr1:1560963-1645635	template	38	Intron_6	--	Yes	--	Yes	--	--	--	--	--	--	--
G4ST01001614719	chr1:1614720-1614757	+	GGGCCCGGAGCTGCGGGGGGCGGGGCCGCCGCGCGGGG<br>((((......)))) ..(((...)))	CDK11B	NM_033489	chr1:1560963-1645635	template	38	Intron_7	--	Yes	--	Yes	--	--	--	--	--	--	--
G4ST01001614719	chr1:1614720-1614757	+	GGGCCCGGAGCTGCGGGGGGCGGGGCCGCCGCGCGGGG<br>((((......)))) ..(((...)))	CDK11B	NM_033488	chr1:1560963-1645635	template	38	Intron_7	--	Yes	--	Yes	--	--	--	--	--	--	--
G4ST01001614719	chr1:1614720-1614757	+	GGGCCCGGAGCTGCGGGGGGCGGGGCCGCCGCGCGGGG<br>((((......)))) ..(((...)))	CDK11B	NM_033487	chr1:1560963-1645635	template	38	Intron_5	--	Yes	--	Yes	--	--	--	--	--	--	--
G4ST01001614719	chr1:1614720-1614757	+	GGGCCCGGAGCTGCGGGGGGCGGGGCCGCCGCGCGGGG<br>((((......)))) ..(((...)))	CDK11B	NM_033486	chr1:1560963-1645635	template	38	Intron_6	--	Yes	--	Yes	--	--	--	--	--	--	--

G4ST01001614719	chr1:1614720-1614757	+	GGGCCCGGAGCTGCGGGGGGCGGGGCCGCCGCGCGGGG<br>((((......)))) ..(((...)))	CDK11B	NM_033493	chr1:1560963-1645635	template	38	Intron_6	--	Yes	--	Yes	--	--	--	--	--	--	--
G4ST01001614733	chr1:1614734-1614775	+	GGGGGGCGGGGCCGCCGCGCGGGGTGAGGACTCGCCTCAGGG<br>..(((...))) ..((((.....)))).	SLC35E2B	NM_001110781	chr1:1582802-1614103	template	-630	upstream	Yes	--	--	--	--	--	--	--	--	--	--
G4ST01001614733	chr1:1614734-1614775	+	GGGGGGCGGGGCCGCCGCGCGGGGTGAGGACTCGCCTCAGGG<br>..(((...))) ..((((.....)))).	CDK11B	NM_033492	chr1:1560963-1645635	template	42	Intron_6	--	Yes	--	Yes	--	--	--	--	--	--	--
G4ST01001614733	chr1:1614734-1614775	+	GGGGGGCGGGGCCGCCGCGCGGGGTGAGGACTCGCCTCAGGG<br>..(((...))) ..((((.....)))).	CDK11B	NM_033489	chr1:1560963-1645635	template	42	Intron_7	--	Yes	--	Yes	--	--	--	--	--	--	--
G4ST01001614733	chr1:1614734-1614775	+	GGGGGGCGGGGCCGCCGCGCGGGGTGAGGACTCGCCTCAGGG<br>..(((...))) ..((((.....)))).	CDK11B	NM_033488	chr1:1560963-1645635	template	42	Intron_7	--	Yes	--	Yes	--	--	--	--	--	--	--
G4ST01001614733	chr1:1614734-1614775	+	GGGGGGCGGGGCCGCCGCGCGGGGTGAGGACTCGCCTCAGGG<br>..(((...))) ..((((.....)))).	CDK11B	NM_033487	chr1:1560963-1645635	template	42	Intron_5	--	Yes	--	Yes	--	--	--	--	--	--	--
G4ST01001614733	chr1:1614734-1614775	+	GGGGGGCGGGGCCGCCGCGCGGGGTGAGGACTCGCCTCAGGG<br>..(((...))) ..((((.....)))).	CDK11B	NM_033486	chr1:1560963-1645635	template	42	Intron_6	--	Yes	--	Yes	--	--	--	--	--	--	--
G4ST01001614733	chr1:1614734-1614775	+	GGGGGGCGGGGCCGCCGCGCGGGGTGAGGACTCGCCTCAGGG<br>..(((...))) ..((((.....)))).	CDK11B	NM_033493	chr1:1560963-1645635	template	42	Intron_6	--	Yes	--	Yes	--	--	--	--	--	--	--
G4ST01001619434	chr1:1619435-1619471	+	GGGCCTTGTTAGGACAACAGGGACGGTGCGGGGTGGG<br>...((((.....))))	MMP23A	NR_002946	chr1:1621238-1623107	non-template	-1766	upstream	Yes	--	--	--	--	--	--	--	--	--	Yes
G4ST01001619434	chr1:1619435-1619471	+	GGGCCTTGTTAGGACAACAGGGACGGTGCGGGGTGGG<br>...((((.....))))	CDK11B	NM_033492	chr1:1560963-1645635	template	37	Intron_6	--	Yes	--	Yes	--	--	--	--	--	--	Yes
G4ST01001619434	chr1:1619435-1619471	+	GGGCCTTGTTAGGACAACAGGGACGGTGCGGGGTGGG<br>...((((.....))))	CDK11B	NM_033489	chr1:1560963-1645635	template	37	Intron_7	--	Yes	--	Yes	--	--	--	--	--	--	Yes
G4ST01001619434	chr1:1619435-1619471	+	GGGCCTTGTTAGGACAACAGGGACGGTGCGGGGTGGG<br>...((((.....))))	CDK11B	NM_033488	chr1:1560963-1645635	template	37	Intron_7	--	Yes	--	Yes	--	--	--	--	--	--	Yes
G4ST01001619434	chr1:1619435-1619471	+	GGGCCTTGTTAGGACAACAGGGACGGTGCGGGGTGGG<br>...((((.....))))	CDK11B	NM_033487	chr1:1560963-1645635	template	37	Intron_5	--	Yes	--	Yes	--	--	--	--	--	--	Yes
G4ST01001619434	chr1:1619435-1619471	+	GGGCCTTGTTAGGACAACAGGGACGGTGCGGGGTGGG<br>...((((.....))))	CDK11B	NM_033486	chr1:1560963-1645635	template	37	Intron_6	--	Yes	--	Yes	--	--	--	--	--	--	Yes
G4ST01001619434	chr1:1619435-1619471	+	GGGCCTTGTTAGGACAACAGGGACGGTGCGGGGTGGG<br>...((((.....))))	CDK11B	NM_033493	chr1:1560963-1645635	template	37	Intron_6	--	Yes	--	Yes	--	--	--	--	--	--	Yes
G4ST01001619573	chr1:1619574-1619623	-	GGGGCCAGCAGGGCTGGAAGAGCCCCTCCATCCGGGCTGCCTAACAGGGG<br>..(((.(((...)))..))) (((.....)))	MMP23A	NR_002946	chr1:1621238-1623107	template	-1614	upstream	Yes	--	--	--	--	--	--	--	--	--	--
G4ST01001619573	chr1:1619574-1619623	-	GGGGCCAGCAGGGCTGGAAGAGCCCCTCCATCCGGGCTGCCTAACAGGGG<br>..(((.(((...)))..))) (((.....)))	CDK11B	NM_033492	chr1:1560963-1645635	non-template	50	Intron_6	--	Yes	--	Yes	--	--	--	--	--	--	--
G4ST01001619573	chr1:1619574-1619623	-	GGGGCCAGCAGGGCTGGAAGAGCCCCTCCATCCGGGCTGCCTAACAGGGG<br>..(((.(((...)))..))) (((.....)))	CDK11B	NM_033489	chr1:1560963-1645635	non-template	50	Intron_7	--	Yes	--	Yes	--	--	--	--	--	--	--
G4ST01001619573	chr1:1619574-1619623	-	GGGGCCAGCAGGGCTGGAAGAGCCCCTCCATCCGGGCTGCCTAACAGGGG<br>..(((.(((...)))..))) (((.....)))	CDK11B	NM_033488	chr1:1560963-1645635	non-template	50	Intron_7	--	Yes	--	Yes	--	--	--	--	--	--	--
G4ST01001619573	chr1:1619574-1619623	-	GGGGCCAGCAGGGCTGGAAGAGCCCCTCCATCCGGGCTGCCTAACAGGGG<br>..(((.(((...)))..))) (((.....)))	CDK11B	NM_033487	chr1:1560963-1645635	non-template	50	Intron_5	--	Yes	--	Yes	--	--	--	--	--	--	--
G4ST01001619573	chr1:1619574-1619623	-	GGGGCCAGCAGGGCTGGAAGAGCCCCTCCATCCGGGCTGCCTAACAGGGG<br>..(((.(((...)))..))) (((.....)))	CDK11B	NM_033486	chr1:1560963-1645635	non-template	50	Intron_6	--	Yes	--	Yes	--	--	--	--	--	--	--
G4ST01001619573	chr1:1619574-1619623	-	GGGGCCAGCAGGGCTGGAAGAGCCCCTCCATCCGGGCTGCCTAACAGGGG<br>..(((.(((...)))..))) (((.....)))	CDK11B	NM_033493	chr1:1560963-1645635	non-template	50	Intron_6	--	Yes	--	Yes	--	--	--	--	--	--	--
G4ST01001621601	chr1:1621602-1621632	-	GGGGCTGAGAGGCCGGGCGCGCAGGGCCGGG<br>(((....))).	MMP23A	NR_002946	chr1:1621238-1623107	template	31	Intron_1.f	--	Yes	--	Yes	--	--	--	--	--	--	Yes
G4ST01001621601	chr1:1621602-1621632	-	GGGGCTGAGAGGCCGGGCGCGCAGGGCCGGG<br>(((....))).	CDK11B	NM_033492	chr1:1560963-1645635	non-template	31	Intron_6	--	Yes	--	Yes	--	--	--	--	--	--	Yes
G4ST01001621601	chr1:1621602-1621632	-	GGGGCTGAGAGGCCGGGCGCGCAGGGCCGGG<br>(((....))).	CDK11B	NM_033489	chr1:1560963-1645635	non-template	31	Intron_7	--	Yes	--	Yes	--	--	--	--	--	--	Yes
G4ST01001621601	chr1:1621602-1621632	-	GGGGCTGAGAGGCCGGGCGCGCAGGGCCGGG<br>(((....))).	CDK11B	NM_033488	chr1:1560963-1645635	non-template	31	Intron_7	--	Yes	--	Yes	--	--	--	--	--	--	Yes
G4ST01001621601	chr1:1621602-1621632	-	GGGGCTGAGAGGCCGGGCGCGCAGGGCCGGG<br>(((....))).	CDK11B	NM_033487	chr1:1560963-1645635	non-template	31	Intron_5	--	Yes	--	Yes	--	--	--	--	--	--	Yes
G4ST01001621601	chr1:1621602-1621632	-	GGGGCTGAGAGGCCGGGCGCGCAGGGCCGGG<br>(((....))).	CDK11B	NM_033486	chr1:1560963-1645635	non-template	31	Intron_6	--	Yes	--	Yes	--	--	--	--	--	--	Yes
G4ST01001621601	chr1:1621602-1621632	-	GGGGCTGAGAGGCCGGGCGCGCAGGGCCGGG<br>(((....))).	CDK11B	NM_033493	chr1:1560963-1645635	non-template	31	Intron_6	--	Yes	--	Yes	--	--	--	--	--	--	Yes
G4ST01001621606	chr1:1621607-1621643	-	GGGGGGAGCACGGGGCTGAGAGGCCGGGCGCGCAGGG<br>(((....))).	MMP23A	NR_002946	chr1:1621238-1623107	template	37	Intron_1.f	--	Yes	--	Yes	--	--	--	--	--	--	Yes
G4ST01001621606	chr1:1621607-1621643	-	GGGGGGAGCACGGGGCTGAGAGGCCGGGCGCGCAGGG<br>(((....))).	CDK11B	NM_033492	chr1:1560963-1645635	non-template	37	Intron_6	--	Yes	--	Yes	--	--	--	--	--	--	Yes
G4ST01001621606	chr1:1621607-1621643	-	GGGGGGAGCACGGGGCTGAGAGGCCGGGCGCGCAGGG<br>(((....))).	CDK11B	NM_033489	chr1:1560963-1645635	non-template	37	Intron_7	--	Yes	--	Yes	--	--	--	--	--	--	Yes
G4ST01001621606	chr1:1621607-1621643	-	GGGGGGAGCACGGGGCTGAGAGGCCGGGCGCGCAGGG<br>(((....))).	CDK11B	NM_033488	chr1:1560963-1645635	non-template	37	Intron_7	--	Yes	--	Yes	--	--	--	--	--	--	Yes
G4ST01001621606	chr1:1621607-1621643	-	GGGGGGAGCACGGGGCTGAGAGGCCGGGCGCGCAGGG<br>(((....))).	CDK11B	NM_033487	chr1:1560963-1645635	non-template	37	Intron_5	--	Yes	--	Yes	--	--	--	--	--	--	Yes
G4ST01001621606	chr1:1621607-1621643	-	GGGGGGAGCACGGGGCTGAGAGGCCGGGCGCGCAGGG<br>(((....))).	CDK11B	NM_033486	chr1:1560963-1645635	non-template	37	Intron_6	--	Yes	--	Yes	--	--	--	--	--	--	Yes
G4ST01001621606	chr1:1621607-1621643	-	GGGGGGAGCACGGGGCTGAGAGGCCGGGCGCGCAGGG<br>(((....))).	CDK11B	NM_033493	chr1:1560963-1645635	non-template	37	Intron_6	--	Yes	--	Yes	--	--	--	--	--	--	Yes
G4ST01001622168	chr1:1622169-1622201	-	GGGCCCCGGGGGTCAGCGCCTGGGAGCCCCGGG<br>(((....))).	MMP23A	NR_002946	chr1:1621238-1623107	template	33	Intron_2	--	Yes	--	Yes	--	--	--	--	--	--	Yes
G4ST01001622168	chr1:1622169-1622201	-	GGGCCCCGGGGGTCAGCGCCTGGGAGCCCCGGG<br>(((....))).	CDK11B	NM_033492	chr1:1560963-1645635	non-template	33	Intron_6	--	Yes	--	Yes	--	--	--	--	--	--	Yes
G4ST01001622168	chr1:1622169-1622201	-	GGGCCCCGGGGGTCAGCGCCTGGGAGCCCCGGG<br>(((....))).	CDK11B	NM_033489	chr1:1560963-1645635	non-template	33	Intron_7	--	Yes	--	Yes	--	--	--	--	--	--	Yes
G4ST01001622168	chr1:1622169-1622201	-	GGGCCCCGGGGGTCAGCGCCTGGGAGCCCCGGG<br>(((....))).	CDK11B	NM_033488	chr1:1560963-1645635	non-template	33	Intron_7	--	Yes	--	Yes	--	--	--	--	--	--	Yes
G4ST01001622168	chr1:1622169-1622201	-	GGGCCCCGGGGGTCAGCGCCTGGGAGCCCCGGG<br>(((....))).	CDK11B	NM_033487	chr1:1560963-1645635	non-template	33	Intron_5	--	Yes	--	Yes	--	--	--	--	--	--	Yes
G4ST01001622168	chr1:1622169-1622201	-	GGGCCCCGGGGGTCAGCGCCTGGGAGCCCCGGG<br>(((....))).	CDK11B	NM_033486	chr1:1560963-1645635	non-template	33	Intron_6	--	Yes	--	Yes	--	--	--	--	--	--	Yes
G4ST01001622168	chr1:1622169-1622201	-	GGGCCCCGGGGGTCAGCGCCTGGGAGCCCCGGG<br>(((....))).	CDK11B	NM_033493	chr1:1560963-1645635	non-template	33	Intron_6	--	Yes	--	Yes	--	--	--	--	--	--	Yes
G4ST01001622663	chr1:1622664-1622700	-	GGGGGGCGGTGGGGTGGTGGCCACCGTGGGGAAGGGG<br>((((...))))....	MMP23A	NR_002946	chr1:1621238-1623107	template	37	Exon_5	--	Yes	Yes	--	--	--	--	--	--	--	--
G4ST01001622663	chr1:1622664-1622700	-	GGGGGGCGGTGGGGTGGTGGCCACCGTGGGGAAGGGG<br>((((...))))....	CDK11B	NM_033492	chr1:1560963-1645635	non-template	37	Intron_6	--	Yes	--	Yes	--	--	--	--	--	--	--
G4ST01001622663	chr1:1622664-1622700	-	GGGGGGCGGTGGGGTGGTGGCCACCGTGGGGAAGGGG<br>((((...))))....	CDK11B	NM_033489	chr1:1560963-1645635	non-template	37	Intron_7	--	Yes	--	Yes	--	--	--	--	--	--	--
G4ST01001622663	chr1:1622664-1622700	-	GGGGGGCGGTGGGGTGGTGGCCACCGTGGGGAAGGGG<br>((((...))))....	CDK11B	NM_033488	chr1:1560963-1645635	non-template	37	Intron_7	--	Yes	--	Yes	--	--	--	--	--	--	--
G4ST01001622663	chr1:1622664-1622700	-	GGGGGGCGGTGGGGTGGTGGCCACCGTGGGGAAGGGG<br>((((...))))....	CDK11B	NM_033487	chr1:1560963-1645635	non-template	37	Intron_5	--	Yes	--	Yes	--	--	--	--	--	--	--
G4ST01001622663	chr1:1622664-1622700	-	GGGGGGCGGTGGGGTGGTGGCCACCGTGGGGAAGGGG<br>((((...))))....	CDK11B	NM_033486	chr1:1560963-1645635	non-template	37	Intron_6	--	Yes	--	Yes	--	--	--	--	--	--	--
G4ST01001622663	chr1:1622664-1622700	-	GGGGGGCGGTGGGGTGGTGGCCACCGTGGGGAAGGGG<br>((((...))))....	CDK11B	NM_033493	chr1:1560963-1645635	non-template	37	Intron_6	--	Yes	--	Yes	--	--	--	--	--	--	--

G4ST01001623796	chr1:1623797-1623831	+	GGGCTGGTCTGGACGAGTGGGTTGGGGCAAGAGGG<br>((.((....)).)).	CDK11B	NM_033492	chr1:1560963-1645635	template	35	Intron_6	--	Yes	--	Yes	--	--	--	--	--	--	--
G4ST01001623796	chr1:1623797-1623831	+	GGGCTGGTCTGGACGAGTGGGTTGGGGCAAGAGGG<br>((.((....)).)).	CDK11B	NM_033489	chr1:1560963-1645635	template	35	Intron_7	--	Yes	--	Yes	--	--	--	--	--	--	--
G4ST01001623796	chr1:1623797-1623831	+	GGGCTGGTCTGGACGAGTGGGTTGGGGCAAGAGGG<br>((.((....)).)).	CDK11B	NM_033488	chr1:1560963-1645635	template	35	Intron_7	--	Yes	--	Yes	--	--	--	--	--	--	--
G4ST01001623796	chr1:1623797-1623831	+	GGGCTGGTCTGGACGAGTGGGTTGGGGCAAGAGGG<br>((.((....)).)).	CDK11B	NM_033487	chr1:1560963-1645635	template	35	Intron_5	--	Yes	--	Yes	--	--	--	--	--	--	--
G4ST01001623796	chr1:1623797-1623831	+	GGGCTGGTCTGGACGAGTGGGTTGGGGCAAGAGGG<br>((.((....)).)).	CDK11B	NM_033486	chr1:1560963-1645635	template	35	Intron_6	--	Yes	--	Yes	--	--	--	--	--	--	--
G4ST01001623796	chr1:1623797-1623831	+	GGGCTGGTCTGGACGAGTGGGTTGGGGCAAGAGGG<br>((.((....)).)).	CDK11B	NM_033493	chr1:1560963-1645635	template	35	Intron_6	--	Yes	--	Yes	--	--	--	--	--	--	--
G4ST01001628760	chr1:1628761-1628799	+	GGGCAGGGGAGTCGGGCACATAGTCGCCCTCTGTCAGGG<br>.(((.((.....)).)))..	CDK11A	NM_033529	chr1:1624030-1645651	template	39	Exon_11	--	Yes	Yes	--	Yes	--	--	--	--	--	Yes
G4ST01001628760	chr1:1628761-1628799	+	GGGCAGGGGAGTCGGGCACATAGTCGCCCTCTGTCAGGG<br>.(((.((.....)).)))..	CDK11A	NM_024011	chr1:1624030-1645651	template	39	Exon_11	--	Yes	Yes	--	Yes	--	--	--	--	--	Yes
G4ST01001628760	chr1:1628761-1628799	+	GGGCAGGGGAGTCGGGCACATAGTCGCCCTCTGTCAGGG<br>.(((.((.....)).)))..	CDK11B	NM_033492	chr1:1560963-1645635	template	39	Intron_6	--	Yes	--	Yes	--	--	--	--	--	--	Yes
G4ST01001628760	chr1:1628761-1628799	+	GGGCAGGGGAGTCGGGCACATAGTCGCCCTCTGTCAGGG<br>.(((.((.....)).)))..	CDK11B	NM_033489	chr1:1560963-1645635	template	39	Intron_7	--	Yes	--	Yes	--	--	--	--	--	--	Yes
G4ST01001628760	chr1:1628761-1628799	+	GGGCAGGGGAGTCGGGCACATAGTCGCCCTCTGTCAGGG<br>.(((.((.....)).)))..	CDK11B	NM_033488	chr1:1560963-1645635	template	39	Intron_7	--	Yes	--	Yes	--	--	--	--	--	--	Yes
G4ST01001628760	chr1:1628761-1628799	+	GGGCAGGGGAGTCGGGCACATAGTCGCCCTCTGTCAGGG<br>.(((.((.....)).)))..	CDK11B	NM_033487	chr1:1560963-1645635	template	39	Intron_5	--	Yes	--	Yes	--	--	--	--	--	--	Yes
G4ST01001628760	chr1:1628761-1628799	+	GGGCAGGGGAGTCGGGCACATAGTCGCCCTCTGTCAGGG<br>.(((.((.....)).)))..	CDK11B	NM_033486	chr1:1560963-1645635	template	39	Intron_6	--	Yes	--	Yes	--	--	--	--	--	--	Yes
G4ST01001628760	chr1:1628761-1628799	+	GGGCAGGGGAGTCGGGCACATAGTCGCCCTCTGTCAGGG<br>.(((.((.....)).)))..	CDK11B	NM_033493	chr1:1560963-1645635	template	39	Intron_6	--	Yes	--	Yes	--	--	--	--	--	--	Yes
G4ST01001666332	chr1:1666333-1666373	-	GGGCACAGGGCGTCCCTCCACGGGGTGCCTTTGGCGTTGGG<br>(((......))) .((((...))))..	SLC35E2	NM_001199787	chr1:1646137-1667298	non-template	41	Intron_1.f	--	Yes	--	Yes	--	--	--	--	--	--	--
G4ST01001666332	chr1:1666333-1666373	-	GGGCACAGGGCGTCCCTCCACGGGGTGCCTTTGGCGTTGGG<br>(((......))) .((((...))))..	SLC35E2	NM_182838	chr1:1648684-1667298	non-template	41	Intron_1.f	--	Yes	--	Yes	--	--	--	--	--	--	--
G4ST01001666348	chr1:1666349-1666379	-	GGGGGAGGGCACAGGGCGTCCCTCCACGGGG<br>(((......)))	SLC35E2	NM_001199787	chr1:1646137-1667298	non-template	31	Intron_1.f	--	Yes	--	Yes	--	--	--	--	--	--	--
G4ST01001666348	chr1:1666349-1666379	-	GGGGGAGGGCACAGGGCGTCCCTCCACGGGG<br>(((......)))	SLC35E2	NM_182838	chr1:1648684-1667298	non-template	31	Intron_1.f	--	Yes	--	Yes	--	--	--	--	--	--	--
G4ST01001667964	chr1:1667965-1668010	+	GGGAAAGCTGCTCTGACAGCTGGGCCCGGAGCTGCGGGGGGCGGGG<br>..(((((......))))) ((((......))))	SLC35E2	NM_001199787	chr1:1646137-1667298	template	-666	upstream	Yes	--	--	--	--	--	--	--	--	--	--
G4ST01001667964	chr1:1667965-1668010	+	GGGAAAGCTGCTCTGACAGCTGGGCCCGGAGCTGCGGGGGGCGGGG<br>..(((((......))))) ((((......))))	SLC35E2	NM_182838	chr1:1648684-1667298	template	-666	upstream	Yes	--	--	--	--	--	--	--	--	--	--
G4ST01001667985	chr1:1667986-1668023	+	GGGCCCGGAGCTGCGGGGGGCGGGGCCGCCGCGCGGGG<br>((((......)))) ..(((...)))	SLC35E2	NM_001199787	chr1:1646137-1667298	template	-687	upstream	Yes	--	--	--	--	--	--	--	--	--	--
G4ST01001667985	chr1:1667986-1668023	+	GGGCCCGGAGCTGCGGGGGGCGGGGCCGCCGCGCGGGG<br>((((......)))) ..(((...)))	SLC35E2	NM_182838	chr1:1648684-1667298	template	-687	upstream	Yes	--	--	--	--	--	--	--	--	--	--
G4ST01001667999	chr1:1668000-1668041	+	GGGGGGCGGGGCCGCCGCGCGGGGTGAGGACTCGCCTCAGGG<br>..(((...))) ..((((.....)))).	SLC35E2	NM_001199787	chr1:1646137-1667298	template	-701	upstream	Yes	--	--	--	--	--	--	--	--	--	--
G4ST01001667999	chr1:1668000-1668041	+	GGGGGGCGGGGCCGCCGCGCGGGGTGAGGACTCGCCTCAGGG<br>..(((...))) ..((((.....)))).	SLC35E2	NM_182838	chr1:1648684-1667298	template	-701	upstream	Yes	--	--	--	--	--	--	--	--	--	--
G4ST01001677047	chr1:1677048-1677072	+	GGGCGGGTGGGAGGCAGCCTTGGGG<br>(((...)))..	NADK	NM_001198995	chr1:1672531-1679941	template	25	Intron_4	--	Yes	--	Yes	--	--	--	--	--	--	--
G4ST01001677047	chr1:1677048-1677072	+	GGGCGGGTGGGAGGCAGCCTTGGGG<br>(((...)))..	NADK	NM_023018	chr1:1672531-1699769	template	25	Intron_6	--	Yes	--	Yes	--	--	--	--	--	--	--
G4ST01001677047	chr1:1677048-1677072	+	GGGCGGGTGGGAGGCAGCCTTGGGG<br>(((...)))..	NADK	NM_001198993	chr1:1672531-1700157	template	25	Intron_6	--	Yes	--	Yes	--	--	--	--	--	--	--
G4ST01001677047	chr1:1677048-1677072	+	GGGCGGGTGGGAGGCAGCCTTGGGG<br>(((...)))..	NADK	NM_001198994	chr1:1672531-1701368	template	25	Intron_8	--	Yes	--	Yes	--	--	--	--	--	--	--
G4ST01001681305	chr1:1681306-1681335	+	GGGGCCGGGAGGGCAGTGGAGCACTCTGGG<br>.((((...))))..	NADK	NM_001198995	chr1:1672531-1679941	template	-1364	upstream	Yes	--	--	--	--	--	--	Yes	Yes	--	--
G4ST01001681305	chr1:1681306-1681335	+	GGGGCCGGGAGGGCAGTGGAGCACTCTGGG<br>.((((...))))..	NADK	NM_023018	chr1:1672531-1699769	template	30	Intron_3	--	Yes	--	Yes	--	--	--	Yes	Yes	--	--
G4ST01001681305	chr1:1681306-1681335	+	GGGGCCGGGAGGGCAGTGGAGCACTCTGGG<br>.((((...))))..	NADK	NM_001198993	chr1:1672531-1700157	template	30	Intron_3	--	Yes	--	Yes	--	--	--	Yes	Yes	--	--
G4ST01001681305	chr1:1681306-1681335	+	GGGGCCGGGAGGGCAGTGGAGCACTCTGGG<br>.((((...))))..	NADK	NM_001198994	chr1:1672531-1701368	template	30	Intron_3	--	Yes	--	Yes	--	--	--	Yes	Yes	--	--
G4ST01001699368	chr1:1699369-1699402	-	GGGCGAGGCTCGGCCTTGGGGACGGGTCTGAGGG<br>..((((...))))..	NADK	NM_023018	chr1:1672531-1699769	non-template	34	Intron_1.f	--	Yes	--	Yes	--	--	--	Yes	Yes	Yes	--
G4ST01001699368	chr1:1699369-1699402	-	GGGCGAGGCTCGGCCTTGGGGACGGGTCTGAGGG<br>..((((...))))..	NADK	NM_001198993	chr1:1672531-1700157	non-template	34	Intron_1.f	--	Yes	--	Yes	--	--	--	Yes	Yes	Yes	--
G4ST01001699368	chr1:1699369-1699402	-	GGGCGAGGCTCGGCCTTGGGGACGGGTCTGAGGG<br>..((((...))))..	NADK	NM_001198994	chr1:1672531-1701368	non-template	34	Intron_1.f	--	Yes	--	Yes	--	--	--	Yes	Yes	Yes	--
G4ST01001734093	chr1:1734094-1734124	-	GGGTGGGGTGGGCGCCAGATCCTTGCATGGG<br>.((.((...)).))..	GNB1	NM_002074	chr1:1706585-1812386	non-template	31	Intron_5	--	Yes	--	Yes	--	--	--	--	--	--	Yes
G4ST01001774391	chr1:1774392-1774424	-	GGGAGTGGAGCACAGGGGCAGGAGGGGAAAGGG<br>.(((...)))..	GNB1	NM_002074	chr1:1706585-1812386	non-template	33	Intron_1.f	--	Yes	--	Yes	--	--	--	--	--	--	Yes
G4ST01001790979	chr1:1790980-1791005	-	GGGAGGGCAGTTTGAGGGTAGTTGGG<br>((...)).	GNB1	NM_002074	chr1:1706585-1812386	non-template	26	Intron_1.f	--	Yes	--	Yes	--	--	--	Yes	Yes	--	--
G4ST01001790987	chr1:1790988-1791011	-	GGGGTTGGGAGGGCAGTTTGAGGG<br>((...)).	GNB1	NM_002074	chr1:1706585-1812386	non-template	24	Intron_1.f	--	Yes	--	Yes	--	--	--	Yes	Yes	--	--
G4ST01001811928	chr1:1811929-1811959	-	GGGGTCCGAGGTTCGGGGTGCAGGGTCGGGG<br>...(((...)))	GNB1	NM_002074	chr1:1706585-1812386	non-template	31	Intron_1.f	--	Yes	--	Yes	--	--	--	Yes	Yes	Yes	Yes
G4ST01001811934	chr1:1811935-1811966	-	GGGGGCTGGGGTCCGAGGTTCGGGGTGCAGGG<br>...(((...)))	GNB1	NM_002074	chr1:1706585-1812386	non-template	32	Intron_1.f	--	Yes	--	Yes	--	--	--	Yes	Yes	Yes	Yes
G4ST01001811941	chr1:1811942-1811971	-	GGGCCGGGGGCTGGGGTCCGAGGTTCGGGG<br>...(((...)))	GNB1	NM_002074	chr1:1706585-1812386	non-template	30	Intron_1.f	--	Yes	--	Yes	--	--	--	Yes	Yes	Yes	Yes
G4ST01001811961	chr1:1811962-1811989	-	GGGCGGCGTCCGAAGGGGGGGCCGGGGG<br>(((...)))..	GNB1	NM_002074	chr1:1706585-1812386	non-template	28	Intron_1.f	--	Yes	--	Yes	--	--	--	Yes	Yes	Yes	Yes
G4ST01001811968	chr1:1811969-1811998	-	GGGTGCGGCGGGCGGCGTCCGAAGGGGGGG<br>(((...)))..	GNB1	NM_002074	chr1:1706585-1812386	non-template	30	Intron_1.f	--	Yes	--	Yes	--	--	--	Yes	--	Yes	Yes
G4ST01001836080	chr1:1836081-1836116	+	GGGTGGGGCAGGGAGGAGCTGGACCCTGGCTTTGGG<br>...(((((.....)))))..	CALML6	NM_138705	chr1:1836126-1838593	non-template	-9	upstream	Yes	--	--	--	--	--	--	--	--	--	--
G4ST01001836963	chr1:1836964-1837003	-	GGGTGGTGACACCAGGGCTGCTGTGGGCAGAGTGCTGGGG<br>.(((...))). (((....)))	CALML6	NM_138705	chr1:1836126-1838593	template	40	Intron_1.f-Exon_2	--	Yes	Yes	Yes	Yes	--	--	--	--	--	--
G4ST01001837348	chr1:1837349-1837380	+	GGGCTGGAAGCCTTTCCCGGGCCAGGGCTGGG<br>..((((....)))).	CALML6	NM_138705	chr1:1836126-1838593	non-template	32	Intron_2	--	Yes	--	Yes	--	--	--	--	--	--	--
G4ST01001838975	chr1:1838976-1839012	+	GGGGCCTCTTCCAGGCTGAGGGCCTGCTGGGGCTGGG<br>((((.....))))...	TMEM52	NM_178545	chr1:1838889-1840600	template	37	Exon_5.l	--	Yes	Yes	--	--	--	Yes	--	--	--	Yes
G4ST01001839087	chr1:1839088-1839121	+	GGGGCCACAGCAATGTGTGGGACGGTGGGGTGGG<br>..((((....)))).	TMEM52	NM_178545	chr1:1838889-1840600	template	34	Exon_5.l	--	Yes	Yes	--	--	--	Yes	--	--	--	Yes
G4ST01001881429	chr1:1881430-1881456	-	GGGCAGGGGGCCAGGCCCGGGATGGGG<br>(((...))).	KIAA1751	NM_001080484	chr1:1874612-1925136	non-template	27	Intron_14	--	Yes	--	Yes	--	--	--	Yes	Yes	Yes	--

G4ST01001881650	chr1:1881651-1881687	+	GGGCACAGGTGCCAGGCCTGGGACCGGGGCTCTAGGG<br>...((((.....))))	KIAA1751	NM_001080484	chr1:1874612-1925136	template	37	Intron_14	--	Yes	--	Yes	--	--	--	--	Yes	Yes	--
G4ST01001885580	chr1:1885581-1885604	-	GGGATGTAGCAGGGACAGGGTGGG<br>.((...))	KIAA1751	NM_001080484	chr1:1874612-1925136	non-template	24	Intron_13	--	Yes	--	Yes	--	--	--	--	--	--	--
G4ST01001885584	chr1:1885585-1885610	-	GGGACAGGGATGTAGCAGGGACAGGG<br>.((...))	KIAA1751	NM_001080484	chr1:1874612-1925136	non-template	26	Intron_13	--	Yes	--	Yes	--	--	--	--	--	--	--
G4ST01001886911	chr1:1886912-1886951	-	GGGAAGCCAGGGAGGAGGCTGCCCGGCTGCCGGGCATGGG<br>....(((.((...)).)))	KIAA1751	NM_001080484	chr1:1874612-1925136	non-template	40	Intron_12	--	Yes	--	Yes	--	--	--	--	--	--	--
G4ST01001889636	chr1:1889637-1889676	+	GGGCAGCAACACTGCTGGGGGTGACGCAGGGTCTGGTGGG<br>(((((....))))).	KIAA1751	NM_001080484	chr1:1874612-1925136	template	40	Intron_11	--	Yes	--	Yes	--	--	--	--	--	--	--
G4ST01001894391	chr1:1894392-1894433	-	GGGACACGAGCTCCAGCCGTGCAGGGCTTGGGGGCAGATGGG<br>.((((.((....))))))..	KIAA1751	NM_001080484	chr1:1874612-1925136	non-template	42	Intron_7	--	Yes	--	Yes	--	--	--	--	--	--	--
G4ST01001895254	chr1:1895255-1895291	+	GGGGGAGAGGGCGGAGGGGCTGGCAGAAGCTGCTGGG<br>....((((...)))).	KIAA1751	NM_001080484	chr1:1874612-1925136	template	37	Intron_7	--	Yes	--	Yes	--	--	--	--	--	--	--
G4ST01001895262	chr1:1895263-1895296	+	GGGCGGAGGGGCTGGCAGAAGCTGCTGGGAAGGG<br>....((((...)))).	KIAA1751	NM_001080484	chr1:1874612-1925136	template	34	Intron_7	--	Yes	--	Yes	--	--	--	--	--	--	--
G4ST01001898577	chr1:1898578-1898608	+	GGGAAAGATTGGGATTTTGAGAATGGGAGGG<br>(((.....)))	KIAA1751	NM_001080484	chr1:1874612-1925136	template	31	Intron_6	--	Yes	--	Yes	--	--	--	--	--	--	--
G4ST01001908639	chr1:1908640-1908671	+	GGGCACGTGGTGGGAGGTCTGGGGCCCACGGG<br>.((...))	KIAA1751	NM_001080484	chr1:1874612-1925136	template	32	Intron_4	--	Yes	--	Yes	--	--	--	--	--	--	Yes
G4ST01001940796	chr1:1940797-1940827	+	GGGCGGGGTCCGCGCGGCGCGGGGTCGGGGG<br>...((((...))))	GABRD	NM_000815	chr1:1940628-1952052	non-template	31	Intron_1.f	--	Yes	--	Yes	--	--	--	Yes	Yes	--	--
G4ST01001940800	chr1:1940801-1940837	+	GGGGTCCGCGCGGCGCGGGGTCGGGGGCGGTGGGGGG<br>...((((...))))	GABRD	NM_000815	chr1:1940628-1952052	non-template	37	Intron_1.f	--	Yes	--	Yes	--	--	--	Yes	Yes	--	--
G4ST01001944048	chr1:1944049-1944080	+	GGGCCACTCCTGGGGGTCCCGTGGGCGCGGGG<br>(((....)))	GABRD	NM_000815	chr1:1940628-1952052	non-template	32	Intron_1.f	--	Yes	--	Yes	--	--	--	--	--	--	Yes
G4ST01001970957	chr1:1970958-1970994	+	GGGATGGGCTGATCAGGGAAAACCGTATTTTCAGGGG<br>((((.....))))...	PRKCZ	NM_002744	chr1:1971769-2106694	non-template	-774	upstream	Yes	--	--	--	--	--	--	Yes	Yes	Yes	--
G4ST01001971721	chr1:1971722-1971751	-	GGGGCGGGACCGTCCGGCGGGGGCGGGGGG<br>.(((.....)))	PRKCZ	NM_002744	chr1:1971769-2106694	template	-17	upstream	Yes	--	--	--	--	--	--	--	Yes	Yes	Yes
G4ST01001971728	chr1:1971729-1971762	-	GGGGGGCGCGCGGGGCGGGACCGTCCGGCGGGGG<br>.(((.....)))	PRKCZ	NM_002744	chr1:1971769-2106694	template	-6	upstream	Yes	--	--	--	--	--	--	--	Yes	Yes	Yes
G4ST01001972083	chr1:1972084-1972118	+	GGGAGAGAGGGAAGGGGCGGCGAGGTCGCTGCGGG<br>((((((....))))))	PRKCZ	NM_002744	chr1:1971769-2106694	non-template	35	Intron_1.f	--	Yes	--	Yes	--	--	--	Yes	Yes	--	--
G4ST01001972091	chr1:1972092-1972125	+	GGGAAGGGGCGGCGAGGTCGCTGCGGGCCCGGGG<br>((((((....))))))	PRKCZ	NM_002744	chr1:1971769-2106694	non-template	34	Intron_1.f	--	Yes	--	Yes	--	--	--	--	Yes	--	--
G4ST01001972096	chr1:1972097-1972135	+	GGGGCGGCGAGGTCGCTGCGGGCCCGGGGCTGTCGCGGG<br>((((((....))))))	PRKCZ	NM_002744	chr1:1971769-2106694	non-template	39	Intron_1.f	--	Yes	--	Yes	--	--	--	--	Yes	--	--
G4ST01001972643	chr1:1972644-1972689	+	GGGGCAGCGCTGCTGGGGGTGGCGGGGGCTGCACCAGGTGGCCGGG<br>.((((...)))). ((((((.....))))))	PRKCZ	NM_002744	chr1:1971769-2106694	non-template	46	Intron_1.f	--	Yes	--	Yes	--	--	--	Yes	--	--	--
G4ST01001989224	chr1:1989225-1989265	+	GGGGTGGCATCCGCTGGCACCTGGGCTGACCTGGGACGGGG<br>(((.((.....)).)))..	PRKCZ	NM_002744	chr1:1971769-2106694	non-template	41	Intron_4	--	Yes	--	Yes	--	--	--	--	--	--	--
G4ST01001989494	chr1:1989495-1989522	+	GGGAGAGCATCGCTAGGGAGGGGTGGGG<br>..(((...))).	PRKCZ	NM_002744	chr1:1971769-2106694	non-template	28	Intron_4	--	Yes	--	Yes	--	--	--	--	--	--	--
G4ST01002002657	chr1:2002658-2002693	+	GGGGGTGGGTGCTGACGGGTCTCTGGCAGGAGGGGG<br>.((((....)))).	PRKCZ	NM_002744	chr1:1971769-2106694	non-template	36	Intron_4	--	Yes	--	Yes	--	--	--	--	--	--	Yes
G4ST01002002657	chr1:2002658-2002693	+	GGGGGTGGGTGCTGACGGGTCTCTGGCAGGAGGGGG<br>.((((....)))).	PRKCZ	NM_001033581	chr1:1994946-2106694	non-template	36	Intron_1.f	--	Yes	--	Yes	--	--	--	--	--	--	Yes
G4ST01002002663	chr1:2002664-2002705	+	GGGTGCTGACGGGTCTCTGGCAGGAGGGGGCAGGCTGCAGGG<br>.((((....)))). .(((...))).	PRKCZ	NM_002744	chr1:1971769-2106694	non-template	42	Intron_4	--	Yes	--	Yes	--	--	--	--	--	--	Yes
G4ST01002002663	chr1:2002664-2002705	+	GGGTGCTGACGGGTCTCTGGCAGGAGGGGGCAGGCTGCAGGG<br>.((((....)))). .(((...))).	PRKCZ	NM_001033581	chr1:1994946-2106694	non-template	42	Intron_1.f	--	Yes	--	Yes	--	--	--	--	--	--	Yes
G4ST01002002673	chr1:2002674-2002714	+	GGGTCTCTGGCAGGAGGGGGCAGGCTGCAGGGAGCAGGGGG<br>.((((....)))). .(((...))).	PRKCZ	NM_002744	chr1:1971769-2106694	non-template	41	Intron_4	--	Yes	--	Yes	--	--	--	--	--	--	Yes
G4ST01002002673	chr1:2002674-2002714	+	GGGTCTCTGGCAGGAGGGGGCAGGCTGCAGGGAGCAGGGGG<br>.((((....)))). .(((...))).	PRKCZ	NM_001033581	chr1:1994946-2106694	non-template	41	Intron_1.f	--	Yes	--	Yes	--	--	--	--	--	--	Yes
G4ST01002009367	chr1:2009368-2009406	+	GGGTGGGGGCCTCGGGCTCGGATGCTGCCTGAGTTGGGG<br>(((((.......)))))...	PRKCZ	NM_002744	chr1:1971769-2106694	non-template	39	Intron_4	--	Yes	--	Yes	--	--	--	--	--	--	--
G4ST01002009367	chr1:2009368-2009406	+	GGGTGGGGGCCTCGGGCTCGGATGCTGCCTGAGTTGGGG<br>(((((.......)))))...	PRKCZ	NM_001033581	chr1:1994946-2106694	non-template	39	Intron_1.f	--	Yes	--	Yes	--	--	--	--	--	--	--
G4ST01002013098	chr1:2013099-2013140	+	GGGAGCAGCAGGGGGCGTCCGAGGGCCCTGACCCCAGGCGGG<br>.((((....)))).	PRKCZ	NM_002744	chr1:1971769-2106694	non-template	42	Intron_4	--	Yes	--	Yes	--	--	--	Yes	Yes	--	--
G4ST01002013098	chr1:2013099-2013140	+	GGGAGCAGCAGGGGGCGTCCGAGGGCCCTGACCCCAGGCGGG<br>.((((....)))).	PRKCZ	NM_001033581	chr1:1994946-2106694	non-template	42	Intron_1.f	--	Yes	--	Yes	--	--	--	Yes	Yes	--	--
G4ST01002016319	chr1:2016320-2016360	-	GGGGCCTGGGGTGTGGCGGAGCAGCACATGGGGGGCTGGGG<br>((((.((...)).)))).....	PRKCZ	NM_002744	chr1:1971769-2106694	template	41	Intron_4	--	Yes	--	Yes	--	--	--	--	--	--	--
G4ST01002016319	chr1:2016320-2016360	-	GGGGCCTGGGGTGTGGCGGAGCAGCACATGGGGGGCTGGGG<br>((((.((...)).)))).....	PRKCZ	NM_001033581	chr1:1994946-2106694	template	41	Intron_1.f	--	Yes	--	Yes	--	--	--	--	--	--	--
G4ST01002035279	chr1:2035280-2035309	+	GGGCTCCACGGGGCTGGCTCTGGGGGAGGG<br>.(((....)))	PRKCZ	NM_002744	chr1:1971769-2106694	non-template	30	Intron_4	--	Yes	--	Yes	--	--	--	--	--	--	--
G4ST01002035279	chr1:2035280-2035309	+	GGGCTCCACGGGGCTGGCTCTGGGGGAGGG<br>.(((....)))	PRKCZ	NM_001033581	chr1:1994946-2106694	non-template	30	Intron_1.f	--	Yes	--	Yes	--	--	--	--	--	--	--
G4ST01002035279	chr1:2035280-2035309	+	GGGCTCCACGGGGCTGGCTCTGGGGGAGGG<br>.(((....)))	PRKCZ	NM_001033582	chr1:2026015-2106694	non-template	30	Intron_1.f	--	Yes	--	Yes	--	--	--	--	--	--	--
G4ST01002035288	chr1:2035289-2035317	+	GGGGCTGGCTCTGGGGGAGGGAGAGCGGG<br>.(((....)))	PRKCZ	NM_002744	chr1:1971769-2106694	non-template	29	Intron_4	--	Yes	--	Yes	--	--	--	--	--	--	--
G4ST01002035288	chr1:2035289-2035317	+	GGGGCTGGCTCTGGGGGAGGGAGAGCGGG<br>.(((....)))	PRKCZ	NM_001033581	chr1:1994946-2106694	non-template	29	Intron_1.f	--	Yes	--	Yes	--	--	--	--	--	--	--
G4ST01002035288	chr1:2035289-2035317	+	GGGGCTGGCTCTGGGGGAGGGAGAGCGGG<br>.(((....)))	PRKCZ	NM_001033582	chr1:2026015-2106694	non-template	29	Intron_1.f	--	Yes	--	Yes	--	--	--	--	--	--	--
G4ST01002047595	chr1:2047596-2047639	+	GGGCTGGTCTGCTGACCTGGGCTGCAGGGGCTGCCCGGCTGGGG<br>..((((....)))). (((....)))..	PRKCZ	NM_002744	chr1:1971769-2106694	non-template	44	Intron_4	--	Yes	--	Yes	--	--	--	--	--	--	--
G4ST01002047595	chr1:2047596-2047639	+	GGGCTGGTCTGCTGACCTGGGCTGCAGGGGCTGCCCGGCTGGGG<br>..((((....)))). (((....)))..	PRKCZ	NM_001033581	chr1:1994946-2106694	non-template	44	Intron_1.f	--	Yes	--	Yes	--	--	--	--	--	--	--
G4ST01002047595	chr1:2047596-2047639	+	GGGCTGGTCTGCTGACCTGGGCTGCAGGGGCTGCCCGGCTGGGG<br>..((((....)))). (((....)))..	PRKCZ	NM_001033582	chr1:2026015-2106694	non-template	44	Intron_1.f	--	Yes	--	Yes	--	--	--	--	--	--	--
G4ST01002047755	chr1:2047756-2047797	-	GGGTCGGCGGGGCAGGGTCCCCAGCCCAAGGCGGCTGGGGGG<br>..(((((((......)))))))	PRKCZ	NM_002744	chr1:1971769-2106694	template	42	Intron_4	--	Yes	--	Yes	--	--	--	--	Yes	--	Yes
G4ST01002047755	chr1:2047756-2047797	-	GGGTCGGCGGGGCAGGGTCCCCAGCCCAAGGCGGCTGGGGGG<br>..(((((((......)))))))	PRKCZ	NM_001033581	chr1:1994946-2106694	template	42	Intron_1.f	--	Yes	--	Yes	--	--	--	--	Yes	--	Yes
G4ST01002047755	chr1:2047756-2047797	-	GGGTCGGCGGGGCAGGGTCCCCAGCCCAAGGCGGCTGGGGGG<br>..(((((((......)))))))	PRKCZ	NM_001033582	chr1:2026015-2106694	template	42	Intron_1.f	--	Yes	--	Yes	--	--	--	--	Yes	--	Yes
G4ST01002048402	chr1:2048403-2048440	+	GGGGAAGGGGGAGGTTGTTGCCTCAGTGGGAGCCTGGG<br>.(((((....)))))...	PRKCZ	NM_002744	chr1:1971769-2106694	non-template	38	Intron_4	--	Yes	--	Yes	--	--	--	Yes	--	--	--
G4ST01002048402	chr1:2048403-2048440	+	GGGGAAGGGGGAGGTTGTTGCCTCAGTGGGAGCCTGGG<br>.(((((....)))))...	PRKCZ	NM_001033581	chr1:1994946-2106694	non-template	38	Intron_1.f	--	Yes	--	Yes	--	--	--	Yes	--	--	--
G4ST01002048402	chr1:2048403-2048440	+	GGGGAAGGGGGAGGTTGTTGCCTCAGTGGGAGCCTGGG<br>.(((((....)))))...	PRKCZ	NM_001033582	chr1:2026015-2106694	non-template	38	Intron_1.f	--	Yes	--	Yes	--	--	--	Yes	--	--	--
G4ST01002066243	chr1:2066244-2066283	+	GGGCCCTGCTGCAGCGTGCAGTGGGTGTGTCTGGGGAGGG<br>.....(((((...))))).	PRKCZ	NM_002744	chr1:1971769-2106694	non-template	40	Intron_6	--	Yes	--	Yes	--	--	--	--	--	--	--

G4ST01002066243	chr1:2066244-2066283	+	GGGCCCTGCTGCAGCGTGCAGTGGGTGTGTCTGGGGAGGG<br>.....(((((...))))).	PRKCZ	NM_001033581	chr1:1994946-2106694	non-template	40	Intron_3	--	Yes	--	Yes	--	--	--	--	--	--	--
G4ST01002066243	chr1:2066244-2066283	+	GGGCCCTGCTGCAGCGTGCAGTGGGTGTGTCTGGGGAGGG<br>.....(((((...))))).	PRKCZ	NM_001033582	chr1:2026015-2106694	non-template	40	Intron_3	--	Yes	--	Yes	--	--	--	--	--	--	--
G4ST01002090714	chr1:2090715-2090751	+	GGGAGGGTTCGGGCCCACGAAGGCCGCTTCTGTGGGG<br>..(((((((....)))).)))	PRKCZ	NM_002744	chr1:1971769-2106694	non-template	37	Intron_10	--	Yes	--	Yes	--	--	--	--	--	--	--
G4ST01002090714	chr1:2090715-2090751	+	GGGAGGGTTCGGGCCCACGAAGGCCGCTTCTGTGGGG<br>..(((((((....)))).)))	PRKCZ	NM_001033581	chr1:1994946-2106694	non-template	37	Intron_7	--	Yes	--	Yes	--	--	--	--	--	--	--
G4ST01002090714	chr1:2090715-2090751	+	GGGAGGGTTCGGGCCCACGAAGGCCGCTTCTGTGGGG<br>..(((((((....)))).)))	PRKCZ	NM_001033582	chr1:2026015-2106694	non-template	37	Intron_7	--	Yes	--	Yes	--	--	--	--	--	--	--
G4ST01002102090	chr1:2102091-2102130	-	GGGCTGGGCCCAGGAGGTAGTGCCCTGGGGGCAGCATGGG<br>.(((((.........)))))	PRKCZ	NM_002744	chr1:1971769-2106694	template	40	Intron_16	--	Yes	--	Yes	--	--	--	--	Yes	Yes	--
G4ST01002102090	chr1:2102091-2102130	-	GGGCTGGGCCCAGGAGGTAGTGCCCTGGGGGCAGCATGGG<br>.(((((.........)))))	PRKCZ	NM_001033581	chr1:1994946-2106694	template	40	Intron_13	--	Yes	--	Yes	--	--	--	--	Yes	Yes	--
G4ST01002102090	chr1:2102091-2102130	-	GGGCTGGGCCCAGGAGGTAGTGCCCTGGGGGCAGCATGGG<br>.(((((.........)))))	PRKCZ	NM_001033582	chr1:2026015-2106694	template	40	Intron_13	--	Yes	--	Yes	--	--	--	--	Yes	Yes	--
G4ST01002113125	chr1:2113126-2113158	-	GGGTGGATGGGAAGCTGGGCACCTGGTGAGGGG<br>(((...)))..	C1orf86	NM_001146310	chr1:2105759-2129032	non-template	33	Intron_7.l	--	Yes	--	Yes	--	--	--	--	--	--	Yes
G4ST01002113125	chr1:2113126-2113158	-	GGGTGGATGGGAAGCTGGGCACCTGGTGAGGGG<br>(((...)))..	LOC100128003	NR_024445	chr1:2110342-2114884	non-template	33	Exon_1.u	--	Yes	Yes	--	--	--	--	--	--	--	Yes
G4ST01002113125	chr1:2113126-2113158	-	GGGTGGATGGGAAGCTGGGCACCTGGTGAGGGG<br>(((...)))..	C1orf86	NM_182533	chr1:2110848-2116074	non-template	33	Intron_3.l	--	Yes	--	Yes	--	--	--	--	--	--	Yes
G4ST01002115235	chr1:2115236-2115265	-	GGGGTTTCCAGGGCGGGAGCCATCGCTGGG<br>(((....)))	C1orf86	NM_001146310	chr1:2105759-2129032	non-template	30	Intron_6	--	Yes	--	Yes	--	--	--	Yes	Yes	Yes	--
G4ST01002115235	chr1:2115236-2115265	-	GGGGTTTCCAGGGCGGGAGCCATCGCTGGG<br>(((....)))	LOC100128003	NR_024445	chr1:2110342-2114884	non-template	-351	upstream	Yes	--	--	--	--	--	--	Yes	Yes	Yes	--
G4ST01002115235	chr1:2115236-2115265	-	GGGGTTTCCAGGGCGGGAGCCATCGCTGGG<br>(((....)))	C1orf86	NM_182533	chr1:2110848-2116074	non-template	30	Intron_2	--	Yes	--	Yes	--	--	--	Yes	Yes	Yes	--
G4ST01002115822	chr1:2115823-2115857	+	GGGCGCTGAATGGCGGCGCGGGGTGGGGCGACGGG<br>(((((.....)))))..	C1orf86	NM_001146310	chr1:2105759-2129032	template	35	Intron_6	--	Yes	--	Yes	--	--	--	--	Yes	Yes	Yes
G4ST01002115822	chr1:2115823-2115857	+	GGGCGCTGAATGGCGGCGCGGGGTGGGGCGACGGG<br>(((((.....)))))..	LOC100128003	NR_024445	chr1:2110342-2114884	template	-938	upstream	Yes	--	--	--	--	--	--	--	Yes	Yes	Yes
G4ST01002115822	chr1:2115823-2115857	+	GGGCGCTGAATGGCGGCGCGGGGTGGGGCGACGGG<br>(((((.....)))))..	C1orf86	NM_182533	chr1:2110848-2116074	template	35	Intron_1.f	--	Yes	--	Yes	--	--	--	--	Yes	Yes	Yes
G4ST01002116701	chr1:2116702-2116738	-	GGGAGAGGCAAGTGGCCTGGGCCCATGGGGCACTGGG<br>..((((.....))))	C1orf86	NM_001146310	chr1:2105759-2129032	non-template	37	Intron_6	--	Yes	--	Yes	--	--	--	Yes	Yes	Yes	--
G4ST01002116701	chr1:2116702-2116738	-	GGGAGAGGCAAGTGGCCTGGGCCCATGGGGCACTGGG<br>..((((.....))))	LOC100128003	NR_024445	chr1:2110342-2114884	non-template	-1817	upstream	Yes	--	--	--	--	--	--	Yes	Yes	Yes	--
G4ST01002116701	chr1:2116702-2116738	-	GGGAGAGGCAAGTGGCCTGGGCCCATGGGGCACTGGG<br>..((((.....))))	C1orf86	NM_182533	chr1:2110848-2116074	non-template	-627	upstream	Yes	--	--	--	--	--	--	Yes	Yes	Yes	--
G4ST01002116708	chr1:2116709-2116745	-	GGGCCAAGGGAGAGGCAAGTGGCCTGGGCCCATGGGG<br>..((((.....))))	C1orf86	NM_001146310	chr1:2105759-2129032	non-template	37	Intron_6	--	Yes	--	Yes	--	--	--	Yes	Yes	Yes	--
G4ST01002116708	chr1:2116709-2116745	-	GGGCCAAGGGAGAGGCAAGTGGCCTGGGCCCATGGGG<br>..((((.....))))	LOC100128003	NR_024445	chr1:2110342-2114884	non-template	-1824	upstream	Yes	--	--	--	--	--	--	Yes	Yes	Yes	--
G4ST01002116708	chr1:2116709-2116745	-	GGGCCAAGGGAGAGGCAAGTGGCCTGGGCCCATGGGG<br>..((((.....))))	C1orf86	NM_182533	chr1:2110848-2116074	non-template	-634	upstream	Yes	--	--	--	--	--	--	Yes	Yes	Yes	--
G4ST01002116717	chr1:2116718-2116755	-	GGGCCCAGCAGGGCCAAGGGAGAGGCAAGTGGCCTGGG<br>..((((.....))))	C1orf86	NM_001146310	chr1:2105759-2129032	non-template	38	Intron_6	--	Yes	--	Yes	--	--	--	Yes	Yes	Yes	--
G4ST01002116717	chr1:2116718-2116755	-	GGGCCCAGCAGGGCCAAGGGAGAGGCAAGTGGCCTGGG<br>..((((.....))))	LOC100128003	NR_024445	chr1:2110342-2114884	non-template	-1833	upstream	Yes	--	--	--	--	--	--	Yes	Yes	Yes	--
G4ST01002116717	chr1:2116718-2116755	-	GGGCCCAGCAGGGCCAAGGGAGAGGCAAGTGGCCTGGG<br>..((((.....))))	C1orf86	NM_182533	chr1:2110848-2116074	non-template	-643	upstream	Yes	--	--	--	--	--	--	Yes	Yes	Yes	--
G4ST01002119184	chr1:2119185-2119215	-	GGGAAGCTGTCAGGCGGGAGGGGATTGGGGG<br>..(((....)))	C1orf86	NM_001146310	chr1:2105759-2129032	non-template	31	Intron_6	--	Yes	--	Yes	--	--	--	Yes	Yes	--	--
G4ST01002121753	chr1:2121754-2121796	+	GGGGTTGGGGGTGGAGCAGCTGCACGCCAGGGAATGCCGGGGG<br>((((..((....)).)))).	C1orf86	NM_001146310	chr1:2105759-2129032	template	43	Intron_5	--	Yes	--	Yes	--	--	--	--	--	--	Yes
G4ST01002121759	chr1:2121760-2121803	+	GGGGGTGGAGCAGCTGCACGCCAGGGAATGCCGGGGGCCCGGGG<br>((((..((....)).)))).	C1orf86	NM_001146310	chr1:2105759-2129032	template	44	Intron_5	--	Yes	--	Yes	--	--	--	--	--	--	Yes
G4ST01002125882	chr1:2125883-2125920	-	GGGTTCTCCGGCTCCCGGGGGCTCGGCGGCGGGCGGGG<br>....((((...)))) .(((....)))	C1orf86	NM_001146310	chr1:2105759-2129032	non-template	38	Intron_3	--	Yes	--	Yes	--	--	--	Yes	Yes	Yes	--
G4ST01002126157	chr1:2126158-2126185	-	GGGGCGTGGGAGGTGCCCGGGAGGAGGG<br>.((...))	C1orf86	NM_001146310	chr1:2105759-2129032	non-template	28	Intron_3	--	Yes	--	Yes	--	--	--	Yes	Yes	Yes	Yes
G4ST01002126164	chr1:2126165-2126189	-	GGGCGGGGCGTGGGAGGTGCCCGGG<br>.((...))	C1orf86	NM_001146310	chr1:2105759-2129032	non-template	25	Intron_3	--	Yes	--	Yes	--	--	--	Yes	Yes	Yes	Yes
G4ST01002126251	chr1:2126252-2126283	-	GGGGCGCGTGGCGCCCGGGGGGCGGGGCGGGG<br>((((...)))).... .	C1orf86	NM_001146310	chr1:2105759-2129032	non-template	32	Intron_3	--	Yes	--	Yes	--	--	--	Yes	--	Yes	Yes
G4ST01002126256	chr1:2126257-2126288	-	GGGGCGGGGCGCGTGGCGCCCGGGGGGCGGGG<br>((((...)))).... .	C1orf86	NM_001146310	chr1:2105759-2129032	non-template	32	Intron_3	--	Yes	--	Yes	--	--	--	Yes	--	Yes	Yes
G4ST01002126261	chr1:2126262-2126297	-	GGGGCTTGAGGGGCGGGGCGCGTGGCGCCCGGGGGG<br>((((...)))).... .	C1orf86	NM_001146310	chr1:2105759-2129032	non-template	36	Intron_3	--	Yes	--	Yes	--	--	--	Yes	--	Yes	Yes
G4ST01002148169	chr1:2148170-2148202	-	GGGACGCAATGGTGTGTTTGGGGAGGGGCAGGG<br>(((((....)))))...	SKI	NM_003036	chr1:2149994-2231512	template	-1791	upstream	Yes	--	--	--	--	--	--	Yes	Yes	Yes	--
G4ST01002148174	chr1:2148175-2148209	-	GGGCGCAGGGACGCAATGGTGTGTTTGGGGAGGGG<br>(((((....)))))...	SKI	NM_003036	chr1:2149994-2231512	template	-1784	upstream	Yes	--	--	--	--	--	--	Yes	Yes	Yes	--
G4ST01002148561	chr1:2148562-2148588	+	GGGCGGGGCGGGGCCGGCGCGGCCGGG<br>((((...)))).	SKI	NM_003036	chr1:2149994-2231512	non-template	-1405	upstream	Yes	--	--	--	--	--	--	Yes	Yes	Yes	Yes
G4ST01002149472	chr1:2149473-2149497	+	GGGCGGGCCGGGGGCCGGCGGCGGG<br>.(((...)))	SKI	NM_003036	chr1:2149994-2231512	non-template	-496	upstream	Yes	--	--	--	--	--	--	--	Yes	Yes	Yes
G4ST01002149522	chr1:2149523-2149553	+	GGGACGAGCCGGGCGGGCGCCTCGCGGTGGG<br>(((...)))..	SKI	NM_003036	chr1:2149994-2231512	non-template	-440	upstream	Yes	--	--	--	--	--	--	--	Yes	Yes	--
G4ST01002149532	chr1:2149533-2149563	+	GGGCGGGCGCCTCGCGGTGGGAGGAGCGGGG<br>(((...)))..	SKI	NM_003036	chr1:2149994-2231512	non-template	-430	upstream	Yes	--	--	--	--	--	--	--	Yes	Yes	--
G4ST01002149791	chr1:2149792-2149825	+	GGGGGGCCCGGGCGGCGGCGGGCGCGGCGCGGGG<br>(((...)))	SKI	NM_003036	chr1:2149994-2231512	non-template	-168	upstream	Yes	--	--	--	--	--	--	--	--	Yes	Yes
G4ST01002166341	chr1:2166342-2166389	-	GGGGAGTGAGTGTCTCATGGGGACAAAGGCTGAGCCTGGGAAGGTGGG<br>..(((((...))))). .....((((...))))	SKI	NM_003036	chr1:2149994-2231512	template	48	Intron_1.f	--	Yes	--	Yes	--	--	--	--	--	--	--
G4ST01002167941	chr1:2167942-2167983	+	GGGCGGGGGCTCTGCCGGGACTGGTGGTGTTCATGGAGAGGG<br>.((.(((.....)))..)).	SKI	NM_003036	chr1:2149994-2231512	non-template	42	Intron_1.f	--	Yes	--	Yes	--	--	--	Yes	--	--	--
G4ST01002192159	chr1:2192160-2192193	-	GGGACAGGGCCAGGTGCCCTGGGGCAGGAGAGGG<br>.((((...))))	SKI	NM_003036	chr1:2149994-2231512	template	34	Intron_1.f	--	Yes	--	Yes	--	--	--	--	Yes	--	--
G4ST01002192169	chr1:2192170-2192203	-	GGGGTGGCCCGGGACAGGGCCAGGTGCCCTGGGG<br>.((((...))))	SKI	NM_003036	chr1:2149994-2231512	template	34	Intron_1.f	--	Yes	--	Yes	--	--	--	--	Yes	--	--
G4ST01002192184	chr1:2192185-2192221	-	GGGCAGCTGGTCCAGCCTGGGGTGGCCCGGGACAGGG<br>..((((...))))...	SKI	NM_003036	chr1:2149994-2231512	template	37	Intron_1.f	--	Yes	--	Yes	--	--	--	--	Yes	--	--
G4ST01002194132	chr1:2194133-2194159	-	GGGCGAGGCTCGGGGCGTGGGAGGGGG<br>(((...)))	SKI	NM_003036	chr1:2149994-2231512	template	27	Intron_1.f	--	Yes	--	Yes	--	--	--	Yes	--	--	Yes
G4ST01002194138	chr1:2194139-2194163	-	GGGAGGGCGAGGCTCGGGGCGTGGG<br>(((...)))	SKI	NM_003036	chr1:2149994-2231512	template	25	Intron_1.f	--	Yes	--	Yes	--	--	--	Yes	--	--	Yes
G4ST01002194144	chr1:2194145-2194170	-	GGGGCGTGGGAGGGCGAGGCTCGGGG<br>(((...)))	SKI	NM_003036	chr1:2149994-2231512	template	26	Intron_1.f	--	Yes	--	Yes	--	--	--	Yes	--	--	Yes

G4ST01002211141	chr1:2211142-2211175	+	GGGCAGCACCTGGCTGGGCTGACTGGGCAGAGGG<br>.(((.....)))	SKI	NM_003036	chr1:2149994-2231512	non-template	34	Intron_1.f	--	Yes	--	Yes	--	--	--	Yes	--	--	Yes
G4ST01002211165	chr1:2211166-2211195	+	GGGCAGAGGGAAGGGGTGGCCTGGCCAGGG<br>..(((...))).	SKI	NM_003036	chr1:2149994-2231512	non-template	30	Intron_1.f	--	Yes	--	Yes	--	--	--	Yes	--	--	Yes
G4ST01002211172	chr1:2211173-2211206	+	GGGAAGGGGTGGCCTGGCCAGGGTGAGCATGGGG<br>..(((...))).	SKI	NM_003036	chr1:2149994-2231512	non-template	34	Intron_1.f	--	Yes	--	Yes	--	--	--	Yes	--	--	Yes
G4ST01002214427	chr1:2214428-2214468	+	GGGCTCGGGCCTGAGCGGGGCAGGCCAGTGTGAGCTGGGGG<br>.....(((((....)))))	SKI	NM_003036	chr1:2149994-2231512	non-template	41	Intron_1.f	--	Yes	--	Yes	--	--	--	--	--	--	--
G4ST01002214433	chr1:2214434-2214474	+	GGGCCTGAGCGGGGCAGGCCAGTGTGAGCTGGGGGTTGGGG<br>.....(((((....)))))	SKI	NM_003036	chr1:2149994-2231512	non-template	41	Intron_1.f	--	Yes	--	Yes	--	--	--	--	--	--	--
G4ST01002216045	chr1:2216046-2216085	-	GGGGGCCTGAGGGCCCTGGGAGAGCAGGTGGCTCTCGGGG<br>(((((.....)))))..	SKI	NM_003036	chr1:2149994-2231512	template	40	Intron_1.f	--	Yes	--	Yes	--	--	--	--	--	--	--
G4ST01002221355	chr1:2221356-2221383	-	GGGCAGGGCTGGGCTGAGGAATAGCGGG<br>(((.....))).	SKI	NM_003036	chr1:2149994-2231512	template	28	Intron_1.f	--	Yes	--	Yes	--	--	--	--	--	--	Yes
G4ST01002223732	chr1:2223733-2223770	-	GGGGTGGTCGCGGGGAGGGGACACCGTGTCCCTGTGGG<br>((((...))))....	SKI	NM_003036	chr1:2149994-2231512	template	38	Intron_1.f	--	Yes	--	Yes	--	--	--	--	--	--	--
G4ST01002225890	chr1:2225891-2225924	+	GGGGCGAGTGGTGCTGGGAGGTCCAGGGCACGGG<br>(((.....))).	SKI	NM_003036	chr1:2149994-2231512	non-template	34	Intron_5	--	Yes	--	Yes	--	--	--	--	--	--	--
G4ST01002250207	chr1:2250208-2250247	-	GGGCTGCAGATGCCTGCGGGTGCAGAGGGGCCTCCCTGGG<br>..((((....))))	MORN1	NM_024848	chr1:2242556-2312853	non-template	40	Intron_12	--	Yes	--	Yes	--	--	--	--	--	--	Yes
G4ST01002254829	chr1:2254830-2254871	+	GGGGGACCCGAGGGAGGTCTCGGACCTCAGGGGTCTCCTGGG<br>(((((...)))))...	MORN1	NM_024848	chr1:2242556-2312853	template	42	Intron_12	--	Yes	--	Yes	--	--	--	--	--	--	--
G4ST01002254840	chr1:2254841-2254880	+	GGGAGGTCTCGGACCTCAGGGGTCTCCTGGGACTCCAGGG<br>(((((...)))))...	MORN1	NM_024848	chr1:2242556-2312853	template	40	Intron_12	--	Yes	--	Yes	--	--	--	--	--	--	--
G4ST01002263835	chr1:2263836-2263873	+	GGGTGGGGCCTGGAGAAGTCAGCGGGAACACGCGGGGG<br>..((((.....)))).	MORN1	NM_024848	chr1:2242556-2312853	template	38	Intron_10	--	Yes	--	Yes	--	--	--	--	--	--	--
G4ST01002268310	chr1:2268311-2268340	+	GGGAGATAGGGAGCTTCTTGGGACATGGGG<br>((...)).	MORN1	NM_024848	chr1:2242556-2312853	template	30	Intron_10	--	Yes	--	Yes	--	--	--	--	--	--	Yes
G4ST01002268318	chr1:2268319-2268349	+	GGGAGCTTCTTGGGACATGGGGACTCTTGGG<br>((...)).	MORN1	NM_024848	chr1:2242556-2312853	template	31	Intron_10	--	Yes	--	Yes	--	--	--	--	--	--	Yes
G4ST01002271943	chr1:2271944-2271977	+	GGGCATGGGGGGCAGGGCTGGAGGTGCTCCTGGG<br>..((((...)))).	LOC100129534	NR_024489	chr1:2271714-2273960	template	34	Exon_1.u	--	Yes	Yes	--	--	--	--	--	--	--	--
G4ST01002271943	chr1:2271944-2271977	+	GGGCATGGGGGGCAGGGCTGGAGGTGCTCCTGGG<br>..((((...)))).	MORN1	NM_024848	chr1:2242556-2312853	template	34	Intron_10	--	Yes	--	Yes	--	--	--	--	--	--	--
G4ST01002278200	chr1:2278201-2278245	+	GGGGGGCAGAGTTGGGGGCTGGGGGGTTCTGCCGGAGCACCTGGG<br>.(((.((.....)).))).	MORN1	NM_024848	chr1:2242556-2312853	template	45	Intron_10	--	Yes	--	Yes	--	--	--	--	--	--	--
G4ST01002280521	chr1:2280522-2280560	-	GGGGGGAGCCCATCCCCCCGGGTCAAGGTGGGAGATGGG<br>((((.....))))....	MORN1	NM_024848	chr1:2242556-2312853	non-template	39	Intron_8	--	Yes	--	Yes	--	--	--	--	--	--	--
G4ST01002303360	chr1:2303361-2303397	+	GGGGACCTGTGGGACAGAACAGCCTCTGGGGACAGGG<br>.((((......))))	MORN1	NM_024848	chr1:2242556-2312853	template	37	Intron_6	--	Yes	--	Yes	--	--	--	--	Yes	--	Yes
G4ST01002303370	chr1:2303371-2303407	+	GGGACAGAACAGCCTCTGGGGACAGGGCTAGGCTGGG<br>.((((......))))	MORN1	NM_024848	chr1:2242556-2312853	template	37	Intron_6	--	Yes	--	Yes	--	--	--	--	Yes	--	Yes
G4ST01002303865	chr1:2303866-2303900	+	GGGAAGGGGCGGGCAGAAGAGAATTCTGCCCAGGG<br>(((((.....)))))....	MORN1	NM_024848	chr1:2242556-2312853	template	35	Intron_6	--	Yes	--	Yes	--	--	--	--	Yes	--	Yes
G4ST01002320311	chr1:2320312-2320349	+	GGGTGGGGCTCTGGGCCAGCATCGCTCAGGTGCCCGGG<br>...(((((.....)))))..	RER1	NM_007033	chr1:2313074-2326743	non-template	38	Intron_3	--	Yes	--	Yes	--	--	--	--	--	Yes	--
G4ST01002323828	chr1:2323829-2323856	+	GGGGAGCGCAGCTGTGGGAGCGGGCGGG<br>.(((...)))..	RER1	NM_007033	chr1:2313074-2326743	non-template	28	Intron_6.l	--	Yes	--	Yes	--	--	--	--	--	Yes	--
G4ST01002327976	chr1:2327977-2328010	-	GGGGGCCACACATGGGGGCTGGGGGTGTCCTGGG<br>..(((....)))	PEX10	NM_153818	chr1:2326101-2333870	non-template	34	Intron_4	--	Yes	--	Yes	--	--	--	--	Yes	Yes	--
G4ST01002327976	chr1:2327977-2328010	-	GGGGGCCACACATGGGGGCTGGGGGTGTCCTGGG<br>..(((....)))	PEX10	NM_002617	chr1:2326101-2333870	non-template	34	Intron_4	--	Yes	--	Yes	--	--	--	--	Yes	Yes	--
G4ST01002329294	chr1:2329295-2329328	+	GGGGCAGACTGCTGGGCCAGGCAGGGGCGAGGGG<br>(((...))).	PEX10	NM_153818	chr1:2326101-2333870	template	34	Intron_3	--	Yes	--	Yes	--	--	--	--	--	--	Yes
G4ST01002329294	chr1:2329295-2329328	+	GGGGCAGACTGCTGGGCCAGGCAGGGGCGAGGGG<br>(((...))).	PEX10	NM_002617	chr1:2326101-2333870	template	34	Intron_3	--	Yes	--	Yes	--	--	--	--	--	--	Yes
G4ST01002330429	chr1:2330430-2330473	-	GGGGACAGCTAGGGAGAAAGGGGAAAGAGCAGAGACTCTTGGGG<br>..(((((......))))).	PEX10	NM_153818	chr1:2326101-2333870	non-template	44	Intron_2	--	Yes	--	Yes	--	--	--	--	--	Yes	Yes
G4ST01002330429	chr1:2330430-2330473	-	GGGGACAGCTAGGGAGAAAGGGGAAAGAGCAGAGACTCTTGGGG<br>..(((((......))))).	PEX10	NM_002617	chr1:2326101-2333870	non-template	44	Intron_2	--	Yes	--	Yes	--	--	--	--	--	Yes	Yes
G4ST01002334299	chr1:2334300-2334335	-	GGGCGCGAGGGCGGGACCGCCGCGGCCGGCGCTGGG<br>..(((((....)))))..	PEX10	NM_153818	chr1:2326101-2333870	non-template	-429	upstream	Yes	--	--	--	--	--	--	Yes	Yes	Yes	--
G4ST01002334299	chr1:2334300-2334335	-	GGGCGCGAGGGCGGGACCGCCGCGGCCGGCGCTGGG<br>..(((((....)))))..	PEX10	NM_002617	chr1:2326101-2333870	non-template	-429	upstream	Yes	--	--	--	--	--	--	Yes	Yes	Yes	--
G4ST01002395625	chr1:2395626-2395665	+	GGGGAGTGGGCGGTCCCTTTGGACCTCAGGGCAGGCAGGG<br>.(((((....)))))....	PLCH2	NM_014638	chr1:2397614-2426824	non-template	-1948	upstream	Yes	--	--	--	--	--	--	--	--	--	--
G4ST01002397709	chr1:2397710-2397744	+	GGGCCTGGGCACAGCCCTGTGGGGGCTTCGGAGGG<br>((((....)))).	PLCH2	NM_014638	chr1:2397614-2426824	non-template	35	Exon_1.f	--	Yes	Yes	--	--	Yes	--	--	--	--	--
G4ST01002400417	chr1:2400418-2400456	+	GGGGAGAGACATGGCAGTCCTCAAGGGCTGGGATCAGGG<br>(((.(((......))))))..	PLCH2	NM_014638	chr1:2397614-2426824	non-template	39	Intron_2	--	Yes	--	Yes	--	--	--	--	--	--	--
G4ST01002400966	chr1:2400967-2401001	+	GGGGCTGCCAGCAGGGGGACGCTGGGCCCCCGGGG<br>.(((....))).	PLCH2	NM_014638	chr1:2397614-2426824	non-template	35	Intron_2	--	Yes	--	Yes	--	--	--	--	--	--	--
G4ST01002411255	chr1:2411256-2411286	+	GGGAGGCATCCGGGAGAGAGGGACATTGGGG<br>.((...))	PLCH2	NM_014638	chr1:2397614-2426824	non-template	31	Intron_10	--	Yes	--	Yes	--	--	--	--	--	--	--
G4ST01002411266	chr1:2411267-2411309	+	GGGAGAGAGGGACATTGGGGCAGATTCGCACAGCGGCCTGGGG<br>.(((..((((...)))).)))	PLCH2	NM_014638	chr1:2397614-2426824	non-template	43	Intron_10	--	Yes	--	Yes	--	--	--	--	--	--	--
G4ST01002411274	chr1:2411275-2411318	+	GGGACATTGGGGCAGATTCGCACAGCGGCCTGGGGCCTGGGGGG<br>.(((..((((...)))).)))	PLCH2	NM_014638	chr1:2397614-2426824	non-template	44	Intron_10	--	Yes	--	Yes	--	--	--	--	--	--	--
G4ST01002411490	chr1:2411491-2411519	-	GGGTGGGCCAGGTGCTGGTGGGAATGGGG<br>((((...)))).	PLCH2	NM_014638	chr1:2397614-2426824	template	29	Intron_10	--	Yes	--	Yes	--	--	--	--	--	--	--
G4ST01002411497	chr1:2411498-2411525	-	GGGCCTGGGTGGGCCAGGTGCTGGTGGG<br>((((...)))).	PLCH2	NM_014638	chr1:2397614-2426824	template	28	Intron_10	--	Yes	--	Yes	--	--	--	--	--	--	--
G4ST01002416284	chr1:2416285-2416313	-	GGGGCACTGGGAGCAGGGAGGACACTGGG<br>((....))	PLCH2	NM_014638	chr1:2397614-2426824	template	29	Intron_12	--	Yes	--	Yes	--	--	--	--	--	--	Yes
G4ST01002418001	chr1:2418002-2418033	+	GGGTGAGTGGCTCGGGGACCTGGGGCCACGGG<br>.(((...))).	PLCH2	NM_014638	chr1:2397614-2426824	non-template	32	Exon_14-Intron_14	--	Yes	Yes	Yes	Yes	--	--	--	--	--	--
G4ST01002420304	chr1:2420305-2420342	+	GGGCCGGGGGCTCCAGGCTGGAGCGGTGCTGGGCCGGG<br>.((((((...))))))....	PLCH2	NM_014638	chr1:2397614-2426824	non-template	38	Intron_18	--	Yes	--	Yes	--	--	--	--	--	--	--
G4ST01002422738	chr1:2422739-2422777	+	GGGCTCAGGGGGCAGCAAGCCAGCTGCGGGCCTTGGGGG<br>.(((((......)))))	PLCH2	NM_014638	chr1:2397614-2426824	non-template	39	Intron_20	--	Yes	--	Yes	--	--	--	Yes	--	--	--
G4ST01002426523	chr1:2426524-2426559	+	GGGAACCTGGGCGGCTCTGGAGGCCCAGGGCAGGGG<br>.((((.....))))..	PLCH2	NM_014638	chr1:2397614-2426824	non-template	36	Exon_22.l	--	Yes	Yes	--	--	--	Yes	--	--	--	--
G4ST01002426531	chr1:2426532-2426563	+	GGGCGGCTCTGGAGGCCCAGGGCAGGGGTGGG<br>.((((.....))))..	PLCH2	NM_014638	chr1:2397614-2426824	non-template	32	Exon_22.l	--	Yes	Yes	--	--	--	Yes	--	--	--	--
G4ST01002431538	chr1:2431539-2431568	+	GGGCTGGGTGAGGGTGCTGTGCCCAGCGGG<br>.((((....))))	PANK4	NM_018216	chr1:2429835-2447895	template	30	Intron_16	--	Yes	--	Yes	--	--	--	--	--	--	--
G4ST01002439719	chr1:2439720-2439762	-	GGGCCTGTGCTCCAGGCCAGGGCGGCCCAGGGTGGAGGTGGGG<br>((((.....))))....	PANK4	NM_018216	chr1:2429835-2447895	non-template	43	Intron_8	--	Yes	--	Yes	--	--	--	--	--	--	Yes
G4ST01002453299	chr1:2453300-2453336	+	GGGAGGCAGTGACACTGGGGGCTGGGACTGCTGGGGG<br>...((((...)))).	HES5	NM_001010926	chr1:2450044-2451544	template	-1755	upstream	Yes	--	--	--	--	--	--	--	--	--	--

G4ST01002453315 chr1:2453316-2453357	+	GGGGGCTGGGACTGCTGGGGGCTCATCTCTGAGCAGGTGGGG<br>.(((((....))))).....	HES5	NM_001010926	chr1:2450044-2451544	template	-1771	upstream	Yes	--	--	--	--	--	--	--	--	--	--
G4ST01002474282 chr1:2474283-2474319	+	GGGCTGGAACCCAGGTGGGGGATGCCTGGGTGGTGGG<br>((((...))))....	LOC115110	NR_026927	chr1:2471219-2474144	template	-138	upstream	Yes	--	--	--	--	--	--	--	--	--	Yes
G4ST01002479375 chr1:2479376-2479411	+	GGGGTCTGAAGGGCCCTCTGGGCTGGCCCAGGAGGG<br>(((...)))..	TNFRSF14	NM_003820	chr1:2479151-2486613	template	36	Exon_8.l	--	Yes	Yes	--	--	--	Yes	--	--	Yes	--
G4ST01002479385 chr1:2479386-2479416	+	GGGCCCTCTGGGCTGGCCCAGGAGGGCTGGG<br>(((...)))..	TNFRSF14	NM_003820	chr1:2479151-2486613	template	31	Exon_8.l	--	Yes	Yes	--	--	--	Yes	--	--	Yes	--
G4ST01002481742 chr1:2481743-2481774	-	GGGATGGCTGCCCCAGGGAGGGGGCGGTGGGG<br>.(((.....)))	TNFRSF14	NM_003820	chr1:2479151-2486613	non-template	32	Intron_5	--	Yes	--	Yes	--	--	--	--	--	Yes	--
G4ST01002481750 chr1:2481751-2481785	-	GGGATGGAGCAGGGATGGCTGCCCCAGGGAGGGGG<br>.((...)) .(((.....)))	TNFRSF14	NM_003820	chr1:2479151-2486613	non-template	35	Intron_5	--	Yes	--	Yes	--	--	--	--	--	Yes	--
G4ST01002483361 chr1:2483362-2483394	+	GGGACGAGGGCTTGCAGGAAGGTCCTGGGAGGG<br>.....((((...))))	TNFRSF14	NM_003820	chr1:2479151-2486613	template	33	Intron_3	--	Yes	--	Yes	--	--	--	--	--	Yes	--
G4ST01002507430 chr1:2507431-2507459	-	GGGGGTCCCAGCAGTGGGGGGCGGGGGGG<br>...((((....))))	C1orf93	NR_036637	chr1:2507759-2512768	template	-299	upstream	Yes	--	--	--	--	--	--	--	Yes	Yes	Yes
G4ST01002507430 chr1:2507431-2507459	-	GGGGGTCCCAGCAGTGGGGGGCGGGGGGG<br>...((((....))))	C1orf93	NR_036638	chr1:2507759-2512768	template	-299	upstream	Yes	--	--	--	--	--	--	--	Yes	Yes	Yes
G4ST01002507430 chr1:2507431-2507459	-	GGGGGTCCCAGCAGTGGGGGGCGGGGGGG<br>...((((....))))	C1orf93	NM_001195736	chr1:2508049-2512768	template	-589	upstream	Yes	--	--	--	--	--	--	--	Yes	Yes	Yes
G4ST01002507430 chr1:2507431-2507459	-	GGGGGTCCCAGCAGTGGGGGGCGGGGGGG<br>...((((....))))	C1orf93	NM_001195737	chr1:2508049-2512768	template	-589	upstream	Yes	--	--	--	--	--	--	--	Yes	Yes	Yes
G4ST01002507430 chr1:2507431-2507459	-	GGGGGTCCCAGCAGTGGGGGGCGGGGGGG<br>...((((....))))	C1orf93	NM_001195738	chr1:2508049-2512768	template	-589	upstream	Yes	--	--	--	--	--	--	--	Yes	Yes	Yes
G4ST01002507430 chr1:2507431-2507459	-	GGGGGTCCCAGCAGTGGGGGGCGGGGGGG<br>...((((....))))	C1orf93	NM_001195740	chr1:2508049-2512768	template	-589	upstream	Yes	--	--	--	--	--	--	--	Yes	Yes	Yes
G4ST01002507430 chr1:2507431-2507459	-	GGGGGTCCCAGCAGTGGGGGGCGGGGGGG<br>...((((....))))	C1orf93	NM_001195741	chr1:2508049-2512768	template	-589	upstream	Yes	--	--	--	--	--	--	--	Yes	Yes	Yes
G4ST01002507430 chr1:2507431-2507459	-	GGGGGTCCCAGCAGTGGGGGGCGGGGGGG<br>...((((....))))	C1orf93	NM_152371	chr1:2508049-2512768	template	-589	upstream	Yes	--	--	--	--	--	--	--	Yes	Yes	Yes
G4ST01002507434 chr1:2507435-2507464	-	GGGGCGGGGGTCCCAGCAGTGGGGGGCGGG<br>...((((....))))	C1orf93	NR_036637	chr1:2507759-2512768	template	-294	upstream	Yes	--	--	--	--	--	--	--	Yes	Yes	Yes
G4ST01002507434 chr1:2507435-2507464	-	GGGGCGGGGGTCCCAGCAGTGGGGGGCGGG<br>...((((....))))	C1orf93	NR_036638	chr1:2507759-2512768	template	-294	upstream	Yes	--	--	--	--	--	--	--	Yes	Yes	Yes
G4ST01002507434 chr1:2507435-2507464	-	GGGGCGGGGGTCCCAGCAGTGGGGGGCGGG<br>...((((....))))	C1orf93	NM_001195736	chr1:2508049-2512768	template	-584	upstream	Yes	--	--	--	--	--	--	--	Yes	Yes	Yes
G4ST01002507434 chr1:2507435-2507464	-	GGGGCGGGGGTCCCAGCAGTGGGGGGCGGG<br>...((((....))))	C1orf93	NM_001195737	chr1:2508049-2512768	template	-584	upstream	Yes	--	--	--	--	--	--	--	Yes	Yes	Yes
G4ST01002507434 chr1:2507435-2507464	-	GGGGCGGGGGTCCCAGCAGTGGGGGGCGGG<br>...((((....))))	C1orf93	NM_001195738	chr1:2508049-2512768	template	-584	upstream	Yes	--	--	--	--	--	--	--	Yes	Yes	Yes
G4ST01002507434 chr1:2507435-2507464	-	GGGGCGGGGGTCCCAGCAGTGGGGGGCGGG<br>...((((....))))	C1orf93	NM_001195740	chr1:2508049-2512768	template	-584	upstream	Yes	--	--	--	--	--	--	--	Yes	Yes	Yes
G4ST01002507434 chr1:2507435-2507464	-	GGGGCGGGGGTCCCAGCAGTGGGGGGCGGG<br>...((((....))))	C1orf93	NM_001195741	chr1:2508049-2512768	template	-584	upstream	Yes	--	--	--	--	--	--	--	Yes	Yes	Yes
G4ST01002507434 chr1:2507435-2507464	-	GGGGCGGGGGTCCCAGCAGTGGGGGGCGGG<br>...((((....))))	C1orf93	NM_152371	chr1:2508049-2512768	template	-584	upstream	Yes	--	--	--	--	--	--	--	Yes	Yes	Yes
G4ST01002508070 chr1:2508071-2508104	+	GGGGCGGGGCTTGGGGCTGGATCTATGAGCCGGG<br>...((.((...)).))	C1orf93	NR_036637	chr1:2507759-2512768	non-template	34	Intron_1.f	--	Yes	--	Yes	--	--	--	Yes	Yes	Yes	Yes
G4ST01002508070 chr1:2508071-2508104	+	GGGGCGGGGCTTGGGGCTGGATCTATGAGCCGGG<br>...((.((...)).))	C1orf93	NR_036638	chr1:2507759-2512768	non-template	34	Intron_1.f	--	Yes	--	Yes	--	--	--	Yes	Yes	Yes	Yes
G4ST01002508070 chr1:2508071-2508104	+	GGGGCGGGGCTTGGGGCTGGATCTATGAGCCGGG<br>...((.((...)).))	C1orf93	NM_001195736	chr1:2508049-2512768	non-template	34	Exon_1.f	--	Yes	Yes	--	Yes	Yes	--	Yes	Yes	Yes	Yes
G4ST01002508070 chr1:2508071-2508104	+	GGGGCGGGGCTTGGGGCTGGATCTATGAGCCGGG<br>...((.((...)).))	C1orf93	NM_001195737	chr1:2508049-2512768	non-template	34	Exon_1.f	--	Yes	Yes	--	Yes	Yes	--	Yes	Yes	Yes	Yes
G4ST01002508070 chr1:2508071-2508104	+	GGGGCGGGGCTTGGGGCTGGATCTATGAGCCGGG<br>...((.((...)).))	C1orf93	NM_001195738	chr1:2508049-2512768	non-template	34	Exon_1.f	--	Yes	Yes	--	Yes	Yes	--	Yes	Yes	Yes	Yes
G4ST01002508070 chr1:2508071-2508104	+	GGGGCGGGGCTTGGGGCTGGATCTATGAGCCGGG<br>...((.((...)).))	C1orf93	NM_001195740	chr1:2508049-2512768	non-template	34	Exon_1.f	--	Yes	Yes	--	Yes	Yes	--	Yes	Yes	Yes	Yes
G4ST01002508070 chr1:2508071-2508104	+	GGGGCGGGGCTTGGGGCTGGATCTATGAGCCGGG<br>...((.((...)).))	C1orf93	NM_001195741	chr1:2508049-2512768	non-template	34	Exon_1.f	--	Yes	Yes	--	Yes	Yes	--	Yes	Yes	Yes	Yes
G4ST01002508070 chr1:2508071-2508104	+	GGGGCGGGGCTTGGGGCTGGATCTATGAGCCGGG<br>...((.((...)).))	C1orf93	NM_152371	chr1:2508049-2512768	non-template	34	Exon_1.f	--	Yes	Yes	--	Yes	Yes	--	Yes	Yes	Yes	Yes
G4ST01002508075 chr1:2508076-2508111	+	GGGGCTTGGGGCTGGATCTATGAGCCGGGAGCGGGG<br>...((.((...)).))	C1orf93	NR_036637	chr1:2507759-2512768	non-template	36	Intron_1.f	--	Yes	--	Yes	--	--	--	Yes	Yes	Yes	Yes
G4ST01002508075 chr1:2508076-2508111	+	GGGGCTTGGGGCTGGATCTATGAGCCGGGAGCGGGG<br>...((.((...)).))	C1orf93	NR_036638	chr1:2507759-2512768	non-template	36	Intron_1.f	--	Yes	--	Yes	--	--	--	Yes	Yes	Yes	Yes
G4ST01002508075 chr1:2508076-2508111	+	GGGGCTTGGGGCTGGATCTATGAGCCGGGAGCGGGG<br>...((.((...)).))	C1orf93	NM_001195736	chr1:2508049-2512768	non-template	36	Exon_1.f	--	Yes	Yes	--	Yes	Yes	--	Yes	Yes	Yes	Yes
G4ST01002508075 chr1:2508076-2508111	+	GGGGCTTGGGGCTGGATCTATGAGCCGGGAGCGGGG<br>...((.((...)).))	C1orf93	NM_001195737	chr1:2508049-2512768	non-template	36	Exon_1.f	--	Yes	Yes	--	Yes	Yes	--	Yes	Yes	Yes	Yes
G4ST01002508075 chr1:2508076-2508111	+	GGGGCTTGGGGCTGGATCTATGAGCCGGGAGCGGGG<br>...((.((...)).))	C1orf93	NM_001195738	chr1:2508049-2512768	non-template	36	Exon_1.f	--	Yes	Yes	--	Yes	Yes	--	Yes	Yes	Yes	Yes
G4ST01002508075 chr1:2508076-2508111	+	GGGGCTTGGGGCTGGATCTATGAGCCGGGAGCGGGG<br>...((.((...)).))	C1orf93	NM_001195740	chr1:2508049-2512768	non-template	36	Exon_1.f	--	Yes	Yes	--	Yes	Yes	--	Yes	Yes	Yes	Yes
G4ST01002508075 chr1:2508076-2508111	+	GGGGCTTGGGGCTGGATCTATGAGCCGGGAGCGGGG<br>...((.((...)).))	C1orf93	NM_001195741	chr1:2508049-2512768	non-template	36	Exon_1.f	--	Yes	Yes	--	Yes	Yes	--	Yes	Yes	Yes	Yes
G4ST01002508075 chr1:2508076-2508111	+	GGGGCTTGGGGCTGGATCTATGAGCCGGGAGCGGGG<br>...((.((...)).))	C1orf93	NM_152371	chr1:2508049-2512768	non-template	36	Exon_1.f	--	Yes	Yes	--	Yes	Yes	--	Yes	Yes	Yes	Yes
G4ST01002508909 chr1:2508910-2508936	+	GGGGGTGGGCTGAGCACGGAGGGTGGG<br>(((....))).	C1orf93	NR_036637	chr1:2507759-2512768	non-template	27	Intron_2	--	Yes	--	Yes	--	--	--	Yes	--	--	--
G4ST01002508909 chr1:2508910-2508936	+	GGGGGTGGGCTGAGCACGGAGGGTGGG<br>(((....))).	C1orf93	NR_036638	chr1:2507759-2512768	non-template	27	Intron_2	--	Yes	--	Yes	--	--	--	Yes	--	--	--
G4ST01002508909 chr1:2508910-2508936	+	GGGGGTGGGCTGAGCACGGAGGGTGGG<br>(((....))).	C1orf93	NM_001195736	chr1:2508049-2512768	non-template	27	Intron_2	--	Yes	--	Yes	--	--	--	Yes	--	--	--
G4ST01002508909 chr1:2508910-2508936	+	GGGGGTGGGCTGAGCACGGAGGGTGGG<br>(((....))).	C1orf93	NM_001195737	chr1:2508049-2512768	non-template	27	Intron_2	--	Yes	--	Yes	--	--	--	Yes	--	--	--
G4ST01002508909 chr1:2508910-2508936	+	GGGGGTGGGCTGAGCACGGAGGGTGGG<br>(((....))).	C1orf93	NM_001195738	chr1:2508049-2512768	non-template	27	Intron_2	--	Yes	--	Yes	--	--	--	Yes	--	--	--
G4ST01002508909 chr1:2508910-2508936	+	GGGGGTGGGCTGAGCACGGAGGGTGGG<br>(((....))).	C1orf93	NM_001195740	chr1:2508049-2512768	non-template	27	Intron_2	--	Yes	--	Yes	--	--	--	Yes	--	--	--
G4ST01002508909 chr1:2508910-2508936	+	GGGGGTGGGCTGAGCACGGAGGGTGGG<br>(((....))).	C1orf93	NM_001195741	chr1:2508049-2512768	non-template	27	Intron_2	--	Yes	--	Yes	--	--	--	Yes	--	--	--
G4ST01002508909 chr1:2508910-2508936	+	GGGGGTGGGCTGAGCACGGAGGGTGGG<br>(((....))).	C1orf93	NM_152371	chr1:2508049-2512768	non-template	27	Intron_2	--	Yes	--	Yes	--	--	--	Yes	--	--	--
G4ST01002512251 chr1:2512252-2512282	+	GGGTGGGCGTGGGCCGCACAGCGCGGCAGGG<br>((((.....))))..	C1orf93	NR_036637	chr1:2507759-2512768	non-template	31	Exon_7.l	--	Yes	Yes	--	--	--	--	--	Yes	--	--
G4ST01002512251 chr1:2512252-2512282	+	GGGTGGGCGTGGGCCGCACAGCGCGGCAGGG<br>((((.....))))..	C1orf93	NR_036638	chr1:2507759-2512768	non-template	31	Exon_7.l	--	Yes	Yes	--	--	--	--	--	Yes	--	--
G4ST01002512251 chr1:2512252-2512282	+	GGGTGGGCGTGGGCCGCACAGCGCGGCAGGG<br>((((.....))))..	C1orf93	NM_001195736	chr1:2508049-2512768	non-template	31	Exon_7.l	--	Yes	Yes	--	--	--	Yes	--	Yes	--	--

G4ST01002512251	chr1:2512252-2512282	+	GGGTGGGCGTGGGCCGCACAGCGCGGCAGGG<br>((((.....))))..	C1orf93	NM_001195737	chr1:2508049-2512768	non-template	31	Exon_7.l	--	Yes	Yes	--	--	--	Yes	--	Yes	--	--
G4ST01002512251	chr1:2512252-2512282	+	GGGTGGGCGTGGGCCGCACAGCGCGGCAGGG<br>((((.....))))..	C1orf93	NM_001195738	chr1:2508049-2512768	non-template	31	Exon_7.l	--	Yes	Yes	--	--	--	Yes	--	Yes	--	--
G4ST01002512251	chr1:2512252-2512282	+	GGGTGGGCGTGGGCCGCACAGCGCGGCAGGG<br>((((.....))))..	C1orf93	NM_001195740	chr1:2508049-2512768	non-template	31	Exon_6.l	--	Yes	Yes	--	--	--	Yes	--	Yes	--	--
G4ST01002512251	chr1:2512252-2512282	+	GGGTGGGCGTGGGCCGCACAGCGCGGCAGGG<br>((((.....))))..	C1orf93	NM_001195741	chr1:2508049-2512768	non-template	31	Exon_6.l	--	Yes	Yes	--	--	--	Yes	--	Yes	--	--
G4ST01002512251	chr1:2512252-2512282	+	GGGTGGGCGTGGGCCGCACAGCGCGGCAGGG<br>((((.....))))..	C1orf93	NM_152371	chr1:2508049-2512768	non-template	31	Exon_7.l	--	Yes	Yes	--	--	--	Yes	--	Yes	--	--
G4ST01002512251	chr1:2512252-2512282	+	GGGTGGGCGTGGGCCGCACAGCGCGGCAGGG<br>((((.....))))..	MMEL1	NM_033467	chr1:2511941-2554341	template	31	Exon_24.l	--	Yes	Yes	--	--	--	Yes	--	Yes	--	--
G4ST01002513861	chr1:2513862-2513894	-	GGGGAGGGGGTGCCAGTGCATCAGGGGAGGGGG<br>(((((....)))))..	MMEL1	NM_033467	chr1:2511941-2554341	non-template	33	Intron_21	--	Yes	--	Yes	--	--	--	--	--	--	--
G4ST01002514074	chr1:2514075-2514112	+	GGGCCAGGGCTGGGGCTCCGGTACCCCCGCAGCCTGGG<br>(((.(((.....))).)))..	MMEL1	NM_033467	chr1:2511941-2554341	template	38	Intron_20	--	Yes	--	Yes	--	--	--	Yes	--	--	--
G4ST01002524534	chr1:2524535-2524566	-	GGGTGCCCAAGTCAGTGGGGGGAGGGCAAGGG<br>..((((......))))	MMEL1	NM_033467	chr1:2511941-2554341	non-template	32	Intron_11	--	Yes	--	Yes	--	--	--	--	--	--	Yes
G4ST01002527957	chr1:2527958-2527999	+	GGGGAGCCCTGGGGCCAGGGCCTGGCCGGAGCCTGGCTGGGG<br>((.(((....))).))....	MMEL1	NM_033467	chr1:2511941-2554341	template	42	Intron_7	--	Yes	--	Yes	--	--	--	--	--	--	--
G4ST01002549967	chr1:2549968-2550002	+	GGGGCCGGCGTGGGGGCGGGGGGAGTGTTCCCGGG<br>((((....))))	MMEL1	NM_033467	chr1:2511941-2554341	template	35	Intron_2	--	Yes	--	Yes	--	--	--	--	--	--	Yes
G4ST01002549978	chr1:2549979-2550013	+	GGGGGCGGGGGGAGTGTTCCCGGGACAGCCGGGGG<br>((((....))))	MMEL1	NM_033467	chr1:2511941-2554341	template	35	Intron_2	--	Yes	--	Yes	--	--	--	--	--	--	Yes
G4ST01002549984	chr1:2549985-2550020	+	GGGGGGAGTGTTCCCGGGACAGCCGGGGGCGGGGGG<br>((((....))))	MMEL1	NM_033467	chr1:2511941-2554341	template	36	Intron_2	--	Yes	--	Yes	--	--	--	--	--	--	Yes
G4ST01002549999	chr1:2550000-2550032	+	GGGACAGCCGGGGGCGGGGGGAGTGTTCCCGGG<br>((((....))))	MMEL1	NM_033467	chr1:2511941-2554341	template	33	Intron_2	--	Yes	--	Yes	--	--	--	--	--	--	--
G4ST01002550385	chr1:2550386-2550420	+	GGGAGTCGGGGCCGGGGCAGAGCTTTCTGGAAGGG<br>.((((....))))....	MMEL1	NM_033467	chr1:2511941-2554341	template	35	Intron_2	--	Yes	--	Yes	--	--	--	--	--	--	--
G4ST01002550392	chr1:2550393-2550428	+	GGGGCCGGGGCAGAGCTTTCTGGAAGGGACCGCGGG<br>.((((....))))....	MMEL1	NM_033467	chr1:2511941-2554341	template	36	Intron_2	--	Yes	--	Yes	--	--	--	--	--	--	--
G4ST01002553396	chr1:2553397-2553421	-	GGGAGGCCTGCCTGGGTGGGTGGGG<br>(((....)))	MMEL1	NM_033467	chr1:2511941-2554341	non-template	25	Intron_1.f	--	Yes	--	Yes	--	--	--	--	--	--	Yes
G4ST01002554682	chr1:2554683-2554720	+	GGGTTCTGGGGATCTTGCCAAGGTGCAAGGGGAAGGGG<br>...(((((......)))))	MMEL1	NM_033467	chr1:2511941-2554341	template	-341	upstream	Yes	--	--	--	--	--	--	--	--	--	--
G4ST01002973231	chr1:2973232-2973269	+	GGGGCAAAGGAGGCCTCTGCTGGGCTGCAGGGTGAGGG<br>(((.(((...))).))).	FLJ42875	NR_015440	chr1:2966041-2974149	template	38	Intron_1.f	--	Yes	--	Yes	--	--	--	--	--	--	Yes
G4ST01002973231	chr1:2973232-2973269	+	GGGGCAAAGGAGGCCTCTGCTGGGCTGCAGGGTGAGGG<br>(((.(((...))).))).	FLJ42875	NR_024371	chr1:2970496-2974149	template	38	Intron_1.u	--	Yes	--	Yes	--	--	--	--	--	--	Yes
G4ST01002974943	chr1:2974944-2974984	+	GGGGGGCGGTGGGGCCCGGAGGGGGCTTCGGAGGAGCCGGG<br>((((((...))))))	FLJ42875	NR_015440	chr1:2966041-2974149	template	-794	upstream	Yes	--	--	--	--	--	--	--	--	--	--
G4ST01002974943	chr1:2974944-2974984	+	GGGGGGCGGTGGGGCCCGGAGGGGGCTTCGGAGGAGCCGGG<br>((((((...))))))	FLJ42875	NR_024371	chr1:2970496-2974149	template	-794	upstream	Yes	--	--	--	--	--	--	--	--	--	--
G4ST01002974943	chr1:2974944-2974984	+	GGGGGGCGGTGGGGCCCGGAGGGGGCTTCGGAGGAGCCGGG<br>((((((...))))))	PRDM16	NM_199454	chr1:2975602-3345045	non-template	-617	upstream	Yes	--	--	--	--	--	--	--	--	--	--
G4ST01002974943	chr1:2974944-2974984	+	GGGGGGCGGTGGGGCCCGGAGGGGGCTTCGGAGGAGCCGGG<br>((((((...))))))	PRDM16	NM_022114	chr1:2975602-3345045	non-template	-617	upstream	Yes	--	--	--	--	--	--	--	--	--	--
G4ST01002975993	chr1:2975994-2976030	+	GGGCCCGGGAACCCGAGGCGCGCGGTGGGGGCCGGGG<br>.(((((.....)).)))..	FLJ42875	NR_015440	chr1:2966041-2974149	template	-1844	upstream	Yes	--	--	--	--	--	--	--	--	--	Yes
G4ST01002975993	chr1:2975994-2976030	+	GGGCCCGGGAACCCGAGGCGCGCGGTGGGGGCCGGGG<br>.(((((.....)).)))..	FLJ42875	NR_024371	chr1:2970496-2974149	template	-1844	upstream	Yes	--	--	--	--	--	--	--	--	--	Yes
G4ST01002975993	chr1:2975994-2976030	+	GGGCCCGGGAACCCGAGGCGCGCGGTGGGGGCCGGGG<br>.(((((.....)).)))..	PRDM16	NM_199454	chr1:2975602-3345045	non-template	37	Intron_1.f	--	Yes	--	Yes	--	--	--	--	--	--	Yes
G4ST01002975993	chr1:2975994-2976030	+	GGGCCCGGGAACCCGAGGCGCGCGGTGGGGGCCGGGG<br>.(((((.....)).)))..	PRDM16	NM_022114	chr1:2975602-3345045	non-template	37	Intron_1.f	--	Yes	--	Yes	--	--	--	--	--	--	Yes
G4ST01002975999	chr1:2976000-2976037	+	GGGAACCCGAGGCGCGCGGTGGGGGCCGGGGAGGGGGG<br>.(((((.....)).)))..	FLJ42875	NR_015440	chr1:2966041-2974149	template	-1850	upstream	Yes	--	--	--	--	--	--	--	--	--	Yes
G4ST01002975999	chr1:2976000-2976037	+	GGGAACCCGAGGCGCGCGGTGGGGGCCGGGGAGGGGGG<br>.(((((.....)).)))..	FLJ42875	NR_024371	chr1:2970496-2974149	template	-1850	upstream	Yes	--	--	--	--	--	--	--	--	--	Yes
G4ST01002975999	chr1:2976000-2976037	+	GGGAACCCGAGGCGCGCGGTGGGGGCCGGGGAGGGGGG<br>.(((((.....)).)))..	PRDM16	NM_199454	chr1:2975602-3345045	non-template	38	Intron_1.f	--	Yes	--	Yes	--	--	--	--	--	--	Yes
G4ST01002975999	chr1:2976000-2976037	+	GGGAACCCGAGGCGCGCGGTGGGGGCCGGGGAGGGGGG<br>.(((((.....)).)))..	PRDM16	NM_022114	chr1:2975602-3345045	non-template	38	Intron_1.f	--	Yes	--	Yes	--	--	--	--	--	--	Yes
G4ST01002976502	chr1:2976503-2976525	-	GGGTCGGGAGGTGGCCGGGTGGG<br>.((...))	PRDM16	NM_199454	chr1:2975602-3345045	template	23	Intron_1.f	--	Yes	--	Yes	--	--	--	Yes	--	--	--
G4ST01002976502	chr1:2976503-2976525	-	GGGTCGGGAGGTGGCCGGGTGGG<br>.((...))	PRDM16	NM_022114	chr1:2975602-3345045	template	23	Intron_1.f	--	Yes	--	Yes	--	--	--	Yes	--	--	--
G4ST01002980381	chr1:2980382-2980407	-	GGGGCGCGGGTCGGGATGTCGCAGGG<br>.((...))	PRDM16	NM_199454	chr1:2975602-3345045	template	26	Intron_1.f	--	Yes	--	Yes	--	--	--	--	--	--	--
G4ST01002980381	chr1:2980382-2980407	-	GGGGCGCGGGTCGGGATGTCGCAGGG<br>.((...))	PRDM16	NM_022114	chr1:2975602-3345045	template	26	Intron_1.f	--	Yes	--	Yes	--	--	--	--	--	--	--
G4ST01002988201	chr1:2988202-2988235	+	GGGGGACCTTCCATGGGCAGCAGGGCAGCAAGGG<br>(((...)))..	PRDM16	NM_199454	chr1:2975602-3345045	non-template	34	Intron_1.f	--	Yes	--	Yes	--	--	--	--	--	--	--
G4ST01002988201	chr1:2988202-2988235	+	GGGGGACCTTCCATGGGCAGCAGGGCAGCAAGGG<br>(((...)))..	PRDM16	NM_022114	chr1:2975602-3345045	non-template	34	Intron_1.f	--	Yes	--	Yes	--	--	--	--	--	--	--
G4ST01003012499	chr1:3012500-3012542	-	GGGGAGCTGGGCTGGCATCCTTTGAGGCCGAGGGTGAGACGGG<br>.((((.((....)).)))).	PRDM16	NM_199454	chr1:2975602-3345045	template	43	Intron_1.f	--	Yes	--	Yes	--	--	--	--	--	--	--
G4ST01003012499	chr1:3012500-3012542	-	GGGGAGCTGGGCTGGCATCCTTTGAGGCCGAGGGTGAGACGGG<br>.((((.((....)).)))).	PRDM16	NM_022114	chr1:2975602-3345045	template	43	Intron_1.f	--	Yes	--	Yes	--	--	--	--	--	--	--
G4ST01003023157	chr1:3023158-3023190	-	GGGGTGAGGGCTTCAGGGCTGCAAGAGCAAGGG<br>.(((....))).	PRDM16	NM_199454	chr1:2975602-3345045	template	33	Intron_1.f	--	Yes	--	Yes	--	--	--	--	--	--	--
G4ST01003023157	chr1:3023158-3023190	-	GGGGTGAGGGCTTCAGGGCTGCAAGAGCAAGGG<br>.(((....))).	PRDM16	NM_022114	chr1:2975602-3345045	template	33	Intron_1.f	--	Yes	--	Yes	--	--	--	--	--	--	--
G4ST01003042214	chr1:3042215-3042247	-	GGGGAAGGGACGGACGGTCTGGGGACGGCTGGG<br>.((((...))))	PRDM16	NM_199454	chr1:2975602-3345045	template	33	Intron_1.f	--	Yes	--	Yes	--	--	--	--	--	--	--
G4ST01003042214	chr1:3042215-3042247	-	GGGGAAGGGACGGACGGTCTGGGGACGGCTGGG<br>.((((...))))	PRDM16	NM_022114	chr1:2975602-3345045	template	33	Intron_1.f	--	Yes	--	Yes	--	--	--	--	--	--	--
G4ST01003042223	chr1:3042224-3042256	-	GGGAGCCACGGGGAAGGGACGGACGGTCTGGGG<br>.((((...))))	PRDM16	NM_199454	chr1:2975602-3345045	template	33	Intron_1.f	--	Yes	--	Yes	--	--	--	--	--	--	--
G4ST01003042223	chr1:3042224-3042256	-	GGGAGCCACGGGGAAGGGACGGACGGTCTGGGG<br>.((((...))))	PRDM16	NM_022114	chr1:2975602-3345045	template	33	Intron_1.f	--	Yes	--	Yes	--	--	--	--	--	--	--
G4ST01003055406	chr1:3055407-3055443	-	GGGGTGGCCAAAGCCGGAGGGCCCCTGGGATGGGGGG<br>.((((....))))..	PRDM16	NM_199454	chr1:2975602-3345045	template	37	Intron_1.f	--	Yes	--	Yes	--	--	--	--	--	--	--
G4ST01003055406	chr1:3055407-3055443	-	GGGGTGGCCAAAGCCGGAGGGCCCCTGGGATGGGGGG<br>.((((....))))..	PRDM16	NM_022114	chr1:2975602-3345045	template	37	Intron_1.f	--	Yes	--	Yes	--	--	--	--	--	--	--
G4ST01003056209	chr1:3056210-3056237	+	GGGAGGGAGGCTCCTGGGCTGGCTGGGG<br>((....))	PRDM16	NM_199454	chr1:2975602-3345045	non-template	28	Intron_1.f	--	Yes	--	Yes	--	--	--	--	--	--	--
G4ST01003056209	chr1:3056210-3056237	+	GGGAGGGAGGCTCCTGGGCTGGCTGGGG<br>((....))	PRDM16	NM_022114	chr1:2975602-3345045	non-template	28	Intron_1.f	--	Yes	--	Yes	--	--	--	--	--	--	--

G4ST01003066587	chr1:3066588-3066618	+	GGGTGGGCCCTGTGGGGACCAGCCCCTGGGG<br>...(((...)))	PRDM16	NM_199454	chr1:2975602-3345045	non-template	31	Intron_1.f	--	Yes	--	Yes	--	--	--	--	--	--	--
G4ST01003066587	chr1:3066588-3066618	+	GGGTGGGCCCTGTGGGGACCAGCCCCTGGGG<br>...(((...)))	PRDM16	NM_022114	chr1:2975602-3345045	non-template	31	Intron_1.f	--	Yes	--	Yes	--	--	--	--	--	--	--
G4ST01003066953	chr1:3066954-3066979	+	GGGGATGGGACAGGGAATGGCTTGGG<br>((....))	PRDM16	NM_199454	chr1:2975602-3345045	non-template	26	Intron_1.f	--	Yes	--	Yes	--	--	--	--	--	--	--
G4ST01003066953	chr1:3066954-3066979	+	GGGGATGGGACAGGGAATGGCTTGGG<br>((....))	PRDM16	NM_022114	chr1:2975602-3345045	non-template	26	Intron_1.f	--	Yes	--	Yes	--	--	--	--	--	--	--
G4ST01003076197	chr1:3076198-3076234	-	GGGCCCGAGGCTCGGGGGGAACCAGGGGCTGCGGGGG<br>(((((...)))))	PRDM16	NM_199454	chr1:2975602-3345045	template	37	Intron_1.f	--	Yes	--	Yes	--	--	--	--	--	--	--
G4ST01003076197	chr1:3076198-3076234	-	GGGCCCGAGGCTCGGGGGGAACCAGGGGCTGCGGGGG<br>(((((...)))))	PRDM16	NM_022114	chr1:2975602-3345045	template	37	Intron_1.f	--	Yes	--	Yes	--	--	--	--	--	--	--
G4ST01003076206	chr1:3076207-3076241	-	GGGGAGAGGGCCCGAGGCTCGGGGGGAACCAGGGG<br>(((((...)))))	PRDM16	NM_199454	chr1:2975602-3345045	template	35	Intron_1.f	--	Yes	--	Yes	--	--	--	--	--	--	--
G4ST01003076206	chr1:3076207-3076241	-	GGGGAGAGGGCCCGAGGCTCGGGGGGAACCAGGGG<br>(((((...)))))	PRDM16	NM_022114	chr1:2975602-3345045	template	35	Intron_1.f	--	Yes	--	Yes	--	--	--	--	--	--	--
G4ST01003108849	chr1:3108850-3108881	+	GGGCTTTGCTGAGAGGCAGGGATGGGTCAGGG<br>...((((....))))	PRDM16	NM_199454	chr1:2975602-3345045	non-template	32	Intron_2	--	Yes	--	Yes	--	--	--	--	--	--	--
G4ST01003108849	chr1:3108850-3108881	+	GGGCTTTGCTGAGAGGCAGGGATGGGTCAGGG<br>...((((....))))	PRDM16	NM_022114	chr1:2975602-3345045	non-template	32	Intron_2	--	Yes	--	Yes	--	--	--	--	--	--	--
G4ST01003118795	chr1:3118796-3118827	+	GGGCGGTGGGGGCAGGGCTGCCTGAGCGGGGG<br>((((....))))	PRDM16	NM_199454	chr1:2975602-3345045	non-template	32	Intron_2	--	Yes	--	Yes	--	--	--	--	--	--	--
G4ST01003118795	chr1:3118796-3118827	+	GGGCGGTGGGGGCAGGGCTGCCTGAGCGGGGG<br>((((....))))	PRDM16	NM_022114	chr1:2975602-3345045	non-template	32	Intron_2	--	Yes	--	Yes	--	--	--	--	--	--	--
G4ST01003135760	chr1:3135761-3135794	-	GGGGAGGGGCCCCCGGGGCACCTGCAGGTGTGGG<br>.((((....)))).	PRDM16	NM_199454	chr1:2975602-3345045	template	34	Intron_2	--	Yes	--	Yes	--	--	--	--	--	--	Yes
G4ST01003135760	chr1:3135761-3135794	-	GGGGAGGGGCCCCCGGGGCACCTGCAGGTGTGGG<br>.((((....)))).	PRDM16	NM_022114	chr1:2975602-3345045	template	34	Intron_2	--	Yes	--	Yes	--	--	--	--	--	--	Yes
G4ST01003141050	chr1:3141051-3141086	-	GGGTGGAGGGTGGGGACTGGCACAGTCACGGTGGGG<br>(((((...)))))......	PRDM16	NM_199454	chr1:2975602-3345045	template	36	Intron_2	--	Yes	--	Yes	--	--	--	--	--	--	--
G4ST01003141050	chr1:3141051-3141086	-	GGGTGGAGGGTGGGGACTGGCACAGTCACGGTGGGG<br>(((((...)))))......	PRDM16	NM_022114	chr1:2975602-3345045	template	36	Intron_2	--	Yes	--	Yes	--	--	--	--	--	--	--
G4ST01003141071	chr1:3141072-3141107	-	GGGAACCAGGCCCTGGCCTGTGGGTGGAGGGTGGGG<br>...(((((....))))).	PRDM16	NM_199454	chr1:2975602-3345045	template	36	Intron_2	--	Yes	--	Yes	--	--	--	--	--	--	--
G4ST01003141071	chr1:3141072-3141107	-	GGGAACCAGGCCCTGGCCTGTGGGTGGAGGGTGGGG<br>...(((((....))))).	PRDM16	NM_022114	chr1:2975602-3345045	template	36	Intron_2	--	Yes	--	Yes	--	--	--	--	--	--	--
G4ST01003145921	chr1:3145922-3145957	-	GGGGGGTCAGGGTTGTCTATGCAGGGCACCCAGGGG<br>.(((....)))	PRDM16	NM_199454	chr1:2975602-3345045	template	36	Intron_2	--	Yes	--	Yes	--	--	--	--	--	--	--
G4ST01003145921	chr1:3145922-3145957	-	GGGGGGTCAGGGTTGTCTATGCAGGGCACCCAGGGG<br>.(((....)))	PRDM16	NM_022114	chr1:2975602-3345045	template	36	Intron_2	--	Yes	--	Yes	--	--	--	--	--	--	--
G4ST01003145931	chr1:3145932-3145967	-	GGGTGGAGTCGGGGGGTCAGGGTTGTCTATGCAGGG<br>.(((....)))	PRDM16	NM_199454	chr1:2975602-3345045	template	36	Intron_2	--	Yes	--	Yes	--	--	--	--	--	--	--
G4ST01003145931	chr1:3145932-3145967	-	GGGTGGAGTCGGGGGGTCAGGGTTGTCTATGCAGGG<br>.(((....)))	PRDM16	NM_022114	chr1:2975602-3345045	template	36	Intron_2	--	Yes	--	Yes	--	--	--	--	--	--	--
G4ST01003156471	chr1:3156472-3156507	-	GGGCTGGTCCCCAGGGGCGGATGAGGGTCCCCAGGG<br>((((...))))	PRDM16	NM_199454	chr1:2975602-3345045	template	36	Intron_3	--	Yes	--	Yes	--	--	--	--	Yes	--	--
G4ST01003156471	chr1:3156472-3156507	-	GGGCTGGTCCCCAGGGGCGGATGAGGGTCCCCAGGG<br>((((...))))	PRDM16	NM_022114	chr1:2975602-3345045	template	36	Intron_3	--	Yes	--	Yes	--	--	--	--	Yes	--	--
G4ST01003169529	chr1:3169530-3169562	-	GGGGTGGGAGGGGCCGCCAGGCAGGCTGCAGGG<br>((.(((.....))).)).	PRDM16	NM_199454	chr1:2975602-3345045	template	33	Intron_3	--	Yes	--	Yes	--	--	--	--	--	--	--
G4ST01003169529	chr1:3169530-3169562	-	GGGGTGGGAGGGGCCGCCAGGCAGGCTGCAGGG<br>((.(((.....))).)).	PRDM16	NM_022114	chr1:2975602-3345045	template	33	Intron_3	--	Yes	--	Yes	--	--	--	--	--	--	--
G4ST01003175117	chr1:3175118-3175158	-	GGGGTTAGGGGTAAGAAGCCCTTCTTCAGGGAACTCTGGGG<br>..(((((....)))))..	PRDM16	NM_199454	chr1:2975602-3345045	template	41	Intron_3	--	Yes	--	Yes	--	--	--	--	--	--	--
G4ST01003175117	chr1:3175118-3175158	-	GGGGTTAGGGGTAAGAAGCCCTTCTTCAGGGAACTCTGGGG<br>..(((((....)))))..	PRDM16	NM_022114	chr1:2975602-3345045	template	41	Intron_3	--	Yes	--	Yes	--	--	--	--	--	--	--
G4ST01003184419	chr1:3184420-3184454	+	GGGACCCTCTCATCTGAAAAGGAGGGAGGGCAGGG<br>..(((.((....))..))).	PRDM16	NM_199454	chr1:2975602-3345045	non-template	35	Intron_3	--	Yes	--	Yes	--	--	--	--	Yes	--	Yes
G4ST01003184419	chr1:3184420-3184454	+	GGGACCCTCTCATCTGAAAAGGAGGGAGGGCAGGG<br>..(((.((....))..))).	PRDM16	NM_022114	chr1:2975602-3345045	non-template	35	Intron_3	--	Yes	--	Yes	--	--	--	--	Yes	--	Yes
G4ST01003190484	chr1:3190485-3190522	+	GGGGGCCATCCTCCCATGGCCTGGGCCTGGGGCAGGGG<br>((((((......)))))).	PRDM16	NM_199454	chr1:2975602-3345045	non-template	38	Intron_3	--	Yes	--	Yes	--	--	--	Yes	Yes	--	--
G4ST01003190484	chr1:3190485-3190522	+	GGGGGCCATCCTCCCATGGCCTGGGCCTGGGGCAGGGG<br>((((((......)))))).	PRDM16	NM_022114	chr1:2975602-3345045	non-template	38	Intron_3	--	Yes	--	Yes	--	--	--	Yes	Yes	--	--
G4ST01003196296	chr1:3196297-3196330	-	GGGAGTGGGGTCTGGTGACCGGGCAGGAGTGGGG<br>(((....))).	PRDM16	NM_199454	chr1:2975602-3345045	template	34	Intron_3	--	Yes	--	Yes	--	--	--	--	--	--	--
G4ST01003196296	chr1:3196297-3196330	-	GGGAGTGGGGTCTGGTGACCGGGCAGGAGTGGGG<br>(((....))).	PRDM16	NM_022114	chr1:2975602-3345045	template	34	Intron_3	--	Yes	--	Yes	--	--	--	--	--	--	--
G4ST01003203047	chr1:3203048-3203084	+	GGGCAGGGTGGGCACATGCCAGAGGTTGCCATGGGGG<br>..((((.((.....)).)))).	PRDM16	NM_199454	chr1:2975602-3345045	non-template	37	Intron_3	--	Yes	--	Yes	--	--	--	--	--	--	--
G4ST01003203047	chr1:3203048-3203084	+	GGGCAGGGTGGGCACATGCCAGAGGTTGCCATGGGGG<br>..((((.((.....)).)))).	PRDM16	NM_022114	chr1:2975602-3345045	non-template	37	Intron_3	--	Yes	--	Yes	--	--	--	--	--	--	--
G4ST01003204184	chr1:3204185-3204220	+	GGGAGTCAGGGAAGGGCTGCCCGATGGCAGGAAGGG<br>(((((....)))))...	PRDM16	NM_199454	chr1:2975602-3345045	non-template	36	Intron_3	--	Yes	--	Yes	--	--	--	--	--	--	Yes
G4ST01003204184	chr1:3204185-3204220	+	GGGAGTCAGGGAAGGGCTGCCCGATGGCAGGAAGGG<br>(((((....)))))...	PRDM16	NM_022114	chr1:2975602-3345045	non-template	36	Intron_3	--	Yes	--	Yes	--	--	--	--	--	--	Yes
G4ST01003215469	chr1:3215470-3215502	-	GGGACAGGAACTGGGGGATACCAGGGGCTAGGG<br>.(((...))).	PRDM16	NM_199454	chr1:2975602-3345045	template	33	Intron_3	--	Yes	--	Yes	--	--	--	--	--	--	--
G4ST01003215469	chr1:3215470-3215502	-	GGGACAGGAACTGGGGGATACCAGGGGCTAGGG<br>.(((...))).	PRDM16	NM_022114	chr1:2975602-3345045	template	33	Intron_3	--	Yes	--	Yes	--	--	--	--	--	--	--
G4ST01003215475	chr1:3215476-3215511	-	GGGTCCACAGGGACAGGAACTGGGGGATACCAGGGG<br>.(((...))).	PRDM16	NM_199454	chr1:2975602-3345045	template	36	Intron_3	--	Yes	--	Yes	--	--	--	--	--	--	--
G4ST01003215475	chr1:3215476-3215511	-	GGGTCCACAGGGACAGGAACTGGGGGATACCAGGGG<br>.(((...))).	PRDM16	NM_022114	chr1:2975602-3345045	template	36	Intron_3	--	Yes	--	Yes	--	--	--	--	--	--	--
G4ST01003246641	chr1:3246642-3246682	-	GGGGTCTGCTTGGGGATTTAGGGGGTACAGTGTGCAAAGGG<br>.((((...))))...	PRDM16	NM_199454	chr1:2975602-3345045	template	41	Intron_3	--	Yes	--	Yes	--	--	--	--	--	--	Yes
G4ST01003246641	chr1:3246642-3246682	-	GGGGTCTGCTTGGGGATTTAGGGGGTACAGTGTGCAAAGGG<br>.((((...))))...	PRDM16	NM_022114	chr1:2975602-3345045	template	41	Intron_3	--	Yes	--	Yes	--	--	--	--	--	--	Yes
G4ST01003252647	chr1:3252648-3252685	-	GGGTACGTCGCTCACCGCCGTGGGGCAGGGCCAACGGG<br>.(((.((.....)).))).	PRDM16	NM_199454	chr1:2975602-3345045	template	38	Intron_3	--	Yes	--	Yes	--	--	--	--	--	--	--
G4ST01003252647	chr1:3252648-3252685	-	GGGTACGTCGCTCACCGCCGTGGGGCAGGGCCAACGGG<br>.(((.((.....)).))).	PRDM16	NM_022114	chr1:2975602-3345045	template	38	Intron_3	--	Yes	--	Yes	--	--	--	--	--	--	--
G4ST01003252655	chr1:3252656-3252691	-	GGGGAAGGGTACGTCGCTCACCGCCGTGGGGCAGGG<br>.(((.((.....)).))).	PRDM16	NM_199454	chr1:2975602-3345045	template	36	Intron_3	--	Yes	--	Yes	--	--	--	--	--	--	--
G4ST01003252655	chr1:3252656-3252691	-	GGGGAAGGGTACGTCGCTCACCGCCGTGGGGCAGGG<br>.(((.((.....)).))).	PRDM16	NM_022114	chr1:2975602-3345045	template	36	Intron_3	--	Yes	--	Yes	--	--	--	--	--	--	--
G4ST01003255216	chr1:3255217-3255256	-	GGGAAGCTCTGAGCTCTTTGGGAGAGTGGGTGGAGTTGGG<br>.((((...))))....	PRDM16	NM_199454	chr1:2975602-3345045	template	40	Intron_3	--	Yes	--	Yes	--	--	--	Yes	--	--	--
G4ST01003255216	chr1:3255217-3255256	-	GGGAAGCTCTGAGCTCTTTGGGAGAGTGGGTGGAGTTGGG<br>.((((...))))....	PRDM16	NM_022114	chr1:2975602-3345045	template	40	Intron_3	--	Yes	--	Yes	--	--	--	Yes	--	--	--

G4ST01003258318	chr1:3258319-3258356	+	GGGAGAGCTGGGGGCGGGGAGGCACTGATGCCTGAGGG<br>.(((((....)))))..	PRDM16	NM_199454	chr1:2975602-3345045	non-template	38	Intron_3	--	Yes	--	Yes	--	--	--	--	--	--	--
G4ST01003258318	chr1:3258319-3258356	+	GGGAGAGCTGGGGGCGGGGAGGCACTGATGCCTGAGGG<br>.(((((....)))))..	PRDM16	NM_022114	chr1:2975602-3345045	non-template	38	Intron_3	--	Yes	--	Yes	--	--	--	--	--	--	--
G4ST01003258327	chr1:3258328-3258364	+	GGGGGCGGGGAGGCACTGATGCCTGAGGGTAGCTGGG<br>.(((((....)))))..	PRDM16	NM_199454	chr1:2975602-3345045	non-template	37	Intron_3	--	Yes	--	Yes	--	--	--	--	--	--	--
G4ST01003258327	chr1:3258328-3258364	+	GGGGGCGGGGAGGCACTGATGCCTGAGGGTAGCTGGG<br>.(((((....)))))..	PRDM16	NM_022114	chr1:2975602-3345045	non-template	37	Intron_3	--	Yes	--	Yes	--	--	--	--	--	--	--
G4ST01003259543	chr1:3259544-3259576	-	GGGACGTCCCTGCGTGGGAGTCTGGGCTGGGGG<br>((((....))))	PRDM16	NM_199454	chr1:2975602-3345045	template	33	Intron_3	--	Yes	--	Yes	--	--	--	Yes	--	--	--
G4ST01003259543	chr1:3259544-3259576	-	GGGACGTCCCTGCGTGGGAGTCTGGGCTGGGGG<br>((((....))))	PRDM16	NM_022114	chr1:2975602-3345045	template	33	Intron_3	--	Yes	--	Yes	--	--	--	Yes	--	--	--
G4ST01003267230	chr1:3267231-3267260	-	GGGGGTCCTGCTGGGAGCCAGGCGGGTGGG<br>.((...))	PRDM16	NM_199454	chr1:2975602-3345045	template	30	Intron_3	--	Yes	--	Yes	--	--	--	--	--	--	--
G4ST01003267230	chr1:3267231-3267260	-	GGGGGTCCTGCTGGGAGCCAGGCGGGTGGG<br>.((...))	PRDM16	NM_022114	chr1:2975602-3345045	template	30	Intron_3	--	Yes	--	Yes	--	--	--	--	--	--	--
G4ST01003267234	chr1:3267235-3267272	-	GGGCCTGAGTCAGGGGGTCCTGCTGGGAGCCAGGCGGG<br>((((...)))) .((...))	PRDM16	NM_199454	chr1:2975602-3345045	template	38	Intron_3	--	Yes	--	Yes	--	--	--	--	--	--	--
G4ST01003267234	chr1:3267235-3267272	-	GGGCCTGAGTCAGGGGGTCCTGCTGGGAGCCAGGCGGG<br>((((...)))) .((...))	PRDM16	NM_022114	chr1:2975602-3345045	template	38	Intron_3	--	Yes	--	Yes	--	--	--	--	--	--	--
G4ST01003270053	chr1:3270054-3270094	-	GGGGGCTTGTTTCCAAGGGGGCCTTATTGGGGGGCAATGGG<br>..((((....)))).	PRDM16	NM_199454	chr1:2975602-3345045	template	41	Intron_3	--	Yes	--	Yes	--	--	--	--	--	--	--
G4ST01003270053	chr1:3270054-3270094	-	GGGGGCTTGTTTCCAAGGGGGCCTTATTGGGGGGCAATGGG<br>..((((....)))).	PRDM16	NM_022114	chr1:2975602-3345045	template	41	Intron_3	--	Yes	--	Yes	--	--	--	--	--	--	--
G4ST01003274932	chr1:3274933-3274970	-	GGGAGCGGCTGGGCTTGGGGGAGACACTGTCTTTGGGG<br>..((((...))))....	PRDM16	NM_199454	chr1:2975602-3345045	template	38	Intron_3	--	Yes	--	Yes	--	--	--	--	--	--	Yes
G4ST01003274932	chr1:3274933-3274970	-	GGGAGCGGCTGGGCTTGGGGGAGACACTGTCTTTGGGG<br>..((((...))))....	PRDM16	NM_022114	chr1:2975602-3345045	template	38	Intron_3	--	Yes	--	Yes	--	--	--	--	--	--	Yes
G4ST01003280072	chr1:3280073-3280114	+	GGGACCTGGAGCTTCTCTCCTGGGGGTGCTGGGCAAGCGGGG<br>.((.((((.....)))).))	PRDM16	NM_199454	chr1:2975602-3345045	non-template	42	Intron_3	--	Yes	--	Yes	--	--	--	--	--	--	Yes
G4ST01003280072	chr1:3280073-3280114	+	GGGACCTGGAGCTTCTCTCCTGGGGGTGCTGGGCAAGCGGGG<br>.((.((((.....)))).))	PRDM16	NM_022114	chr1:2975602-3345045	non-template	42	Intron_3	--	Yes	--	Yes	--	--	--	--	--	--	Yes
G4ST01003296222	chr1:3296223-3296260	+	GGGACTTTCGGGGCAATCAGATTGCAAGGGAGATAGGG<br>((((.....))))..	PRDM16	NM_199454	chr1:2975602-3345045	non-template	38	Intron_4	--	Yes	--	Yes	--	--	--	Yes	--	--	--
G4ST01003296222	chr1:3296223-3296260	+	GGGACTTTCGGGGCAATCAGATTGCAAGGGAGATAGGG<br>((((.....))))..	PRDM16	NM_022114	chr1:2975602-3345045	non-template	38	Intron_4	--	Yes	--	Yes	--	--	--	Yes	--	--	--
G4ST01003301749	chr1:3301750-3301789	-	GGGCTCCCAGGGATTGAGGGGACGTGGCCATCCTTGTGGG<br>.(((.((....)).)))	PRDM16	NM_199454	chr1:2975602-3345045	template	40	Intron_4	--	Yes	--	Yes	--	--	--	--	--	--	Yes
G4ST01003301749	chr1:3301750-3301789	-	GGGCTCCCAGGGATTGAGGGGACGTGGCCATCCTTGTGGG<br>.(((.((....)).)))	PRDM16	NM_022114	chr1:2975602-3345045	template	40	Intron_4	--	Yes	--	Yes	--	--	--	--	--	--	Yes
G4ST01003329551	chr1:3329552-3329592	+	GGGACGAAGTGGGATGGGGATATGCCTCGGCCAGGCGTGGG<br>...((((((.....))))))	PRDM16	NM_199454	chr1:2975602-3345045	non-template	41	Intron_12	--	Yes	--	Yes	--	--	--	--	Yes	--	--
G4ST01003329551	chr1:3329552-3329592	+	GGGACGAAGTGGGATGGGGATATGCCTCGGCCAGGCGTGGG<br>...((((((.....))))))	PRDM16	NM_022114	chr1:2975602-3345045	non-template	41	Intron_12	--	Yes	--	Yes	--	--	--	--	Yes	--	--
G4ST01003330033	chr1:3330034-3330065	+	GGGCCACCGGCACCGGTCGGGGCGGGGTGGGG<br>..((((....))))..	PRDM16	NM_199454	chr1:2975602-3345045	non-template	32	Intron_12	--	Yes	--	Yes	--	--	--	Yes	Yes	--	--
G4ST01003330033	chr1:3330034-3330065	+	GGGCCACCGGCACCGGTCGGGGCGGGGTGGGG<br>..((((....))))..	PRDM16	NM_022114	chr1:2975602-3345045	non-template	32	Intron_12	--	Yes	--	Yes	--	--	--	Yes	Yes	--	--
G4ST01003333239	chr1:3333240-3333271	+	GGGGACAGGGCAGGACGTGCCTGGGGACAGGG<br>((((.....))))	PRDM16	NM_199454	chr1:2975602-3345045	non-template	32	Intron_14	--	Yes	--	Yes	--	--	--	--	Yes	--	--
G4ST01003333239	chr1:3333240-3333271	+	GGGGACAGGGCAGGACGTGCCTGGGGACAGGG<br>((((.....))))	PRDM16	NM_022114	chr1:2975602-3345045	non-template	32	Intron_14	--	Yes	--	Yes	--	--	--	--	Yes	--	--
G4ST01003338866	chr1:3338867-3338894	-	GGGAGGGAGACGAAGGCGGCTGGGCGGG<br>((.((....)).))	PRDM16	NM_199454	chr1:2975602-3345045	template	28	Intron_16.l	--	Yes	--	Yes	--	--	--	--	--	--	--
G4ST01003338866	chr1:3338867-3338894	-	GGGAGGGAGACGAAGGCGGCTGGGCGGG<br>((.((....)).))	PRDM16	NM_022114	chr1:2975602-3345045	template	28	Intron_16.l	--	Yes	--	Yes	--	--	--	--	--	--	--
G4ST01003342343	chr1:3342344-3342378	+	GGGGCCATGGGGTGGCCCCGCCGGGGCAGCGGGGG<br>..(((...))).	PRDM16	NM_199454	chr1:2975602-3345045	non-template	35	Exon_17.l	--	Yes	Yes	--	--	--	Yes	--	--	--	--
G4ST01003342343	chr1:3342344-3342378	+	GGGGCCATGGGGTGGCCCCGCCGGGGCAGCGGGGG<br>..(((...))).	PRDM16	NM_022114	chr1:2975602-3345045	non-template	35	Exon_17.l	--	Yes	Yes	--	--	--	Yes	--	--	--	--
G4ST01003343031	chr1:3343032-3343060	-	GGGAGGGCGAGGCTCGCTGGGGCCTGGGG<br>(((...)))...	PRDM16	NM_199454	chr1:2975602-3345045	template	29	Exon_17.l	--	Yes	Yes	--	--	--	Yes	--	--	--	--
G4ST01003343031	chr1:3343032-3343060	-	GGGAGGGCGAGGCTCGCTGGGGCCTGGGG<br>(((...)))...	PRDM16	NM_022114	chr1:2975602-3345045	template	29	Exon_17.l	--	Yes	Yes	--	--	--	Yes	--	--	--	--
G4ST01003343243	chr1:3343244-3343273	+	GGGTCAGGGCTGCAAGCCAGGGGTCCAGGG<br>(((.....)))	PRDM16	NM_199454	chr1:2975602-3345045	non-template	30	Exon_17.l	--	Yes	Yes	--	--	--	Yes	--	--	--	--
G4ST01003343243	chr1:3343244-3343273	+	GGGTCAGGGCTGCAAGCCAGGGGTCCAGGG<br>(((.....)))	PRDM16	NM_022114	chr1:2975602-3345045	non-template	30	Exon_17.l	--	Yes	Yes	--	--	--	Yes	--	--	--	--
G4ST01003369415	chr1:3369416-3369449	+	GGGGTAGGGGGGTGGACACCCGCGGGGGCAGGGG<br>((((....))))....	ARHGEF16	NM_014448	chr1:3361007-3387537	non-template	34	Intron_1.f	--	Yes	--	Yes	--	--	--	--	--	--	--
G4ST01003374091	chr1:3374092-3374115	+	GGGAGACAGCTGGGATGGGAGGGG<br>((....))	ARHGEF16	NM_014448	chr1:3361007-3387537	non-template	24	Intron_4	--	Yes	--	Yes	--	--	--	--	--	--	Yes
G4ST01003376515	chr1:3376516-3376543	+	GGGGTGGGGTGGGGTGTGTGTGCGTGGG<br>.(((....))).	ARHGEF16	NM_014448	chr1:3361007-3387537	non-template	28	Intron_6	--	Yes	--	Yes	--	--	--	--	--	--	--
G4ST01003376717	chr1:3376718-3376752	+	GGGCAGGGGTGTCTGCACGGGTGTGTCTGCACGGG<br>(((....))) .(((....)))	ARHGEF16	NM_014448	chr1:3361007-3387537	non-template	35	Intron_6	--	Yes	--	Yes	--	--	--	--	Yes	--	--
G4ST01003377396	chr1:3377397-3377430	+	GGGGTGTGTGTGCGTGGGCAGGGGTGTGCGTGGG<br>.(((....))).	ARHGEF16	NM_014448	chr1:3361007-3387537	non-template	34	Intron_6	--	Yes	--	Yes	--	--	--	--	Yes	--	--
G4ST01003379303	chr1:3379304-3379330	-	GGGATGCTGGACAGCAGGGAGGGCGGG<br>.((((....))))	ARHGEF16	NM_014448	chr1:3361007-3387537	template	27	Intron_6	--	Yes	--	Yes	--	--	--	--	--	--	Yes
G4ST01003379703	chr1:3379704-3379739	-	GGGCTGGGAGCAGGAATGTGCTTTGGGAGACCAGGG<br>((((......))))..	ARHGEF16	NM_014448	chr1:3361007-3387537	template	36	Intron_7	--	Yes	--	Yes	--	--	--	--	--	--	--
G4ST01003382272	chr1:3382273-3382320	+	GGGCAGGCACTGTGGGCAGCCAGAGGCTGGCTGGGCAGGTGTCCTGGG<br>(((...))). .((((((...)))))) .(((...)))	ARHGEF16	NM_014448	chr1:3361007-3387537	non-template	48	Intron_9	--	Yes	--	Yes	--	--	--	--	Yes	--	--
G4ST01003382285	chr1:3382286-3382331	+	GGGCAGCCAGAGGCTGGCTGGGCAGGTGTCCTGGGCACACTGTGGG<br>.((((((...)))))) .(((...))) .((...))	ARHGEF16	NM_014448	chr1:3361007-3387537	non-template	46	Intron_9	--	Yes	--	Yes	--	--	--	--	Yes	--	--
G4ST01003382304	chr1:3382305-3382340	+	GGGCAGGTGTCCTGGGCACACTGTGGGCCGTGTGGG<br>.(((...))) .((...))	ARHGEF16	NM_014448	chr1:3361007-3387537	non-template	36	Intron_9	--	Yes	--	Yes	--	--	--	--	Yes	--	--
G4ST01003383681	chr1:3383682-3383707	-	GGGGGTGGCAGTACAGGGTGGGGGGG<br>.(((....))).	ARHGEF16	NM_014448	chr1:3361007-3387537	template	26	Intron_10	--	Yes	--	Yes	--	--	--	--	--	--	Yes
G4ST01003396235	chr1:3396236-3396265	+	GGGCCCCGGGTGCACTGGGAGGTCCCTGGG<br>((....))	MEGF6	NM_001409	chr1:3394366-3517919	template	30	Exon_37.l	--	Yes	Yes	--	--	--	Yes	--	--	--	--
G4ST01003397674	chr1:3397675-3397708	-	GGGCAGTGTGTTGCTGGGCGTGGGGAGTCCTGGG<br>.(((.....)))	MEGF6	NM_001409	chr1:3394366-3517919	non-template	34	Intron_35	--	Yes	--	Yes	--	--	--	--	Yes	--	--
G4ST01003397683	chr1:3397684-3397718	-	GGGGCCCCATGGGCAGTGTGTTGCTGGGCGTGGGG<br>.(((.....)))	MEGF6	NM_001409	chr1:3394366-3517919	non-template	35	Intron_35	--	Yes	--	Yes	--	--	--	--	Yes	--	--
G4ST01003402668	chr1:3402669-3402702	-	GGGACCCCAGCAGAGCTGGCGGGGTGGGCGGGGG<br>...(((((...)))))..	MEGF6	NM_001409	chr1:3394366-3517919	non-template	34	Intron_29	--	Yes	--	Yes	--	--	--	Yes	--	--	--
G4ST01003408842	chr1:3408843-3408872	-	GGGGCTCCCAGCTGGGTGGAGGGGAGTGGG<br>(((...))).	MEGF6	NM_001409	chr1:3394366-3517919	non-template	30	Intron_17	--	Yes	--	Yes	--	--	--	--	Yes	--	--

G4ST01003408965	chr1:3408966-3408998	+	GGGGAGGGCAGGGGGCCTCCTGGAGGCCTAGGG<br>((((((...))))))..	MEGF6	NM_001409	chr1:3394366-3517919	template	33	Intron_17	--	Yes	--	Yes	--	--	--	--	Yes	--	Yes
G4ST01003409189	chr1:3409190-3409227	-	GGGCAGGCGTGGGAGGGCAGAGGAAAGCTCTGCCAGGG<br>(((((.....)))))...	MEGF6	NM_001409	chr1:3394366-3517919	non-template	38	Intron_17	--	Yes	--	Yes	--	--	--	--	Yes	--	--
G4ST01003417645	chr1:3417646-3417684	+	GGGAGTCAGGGTCTGGGGTGTGACTTCCAGGACACTGGG<br>((((..((...)).)))).	MEGF6	NM_001409	chr1:3394366-3517919	template	39	Intron_9	--	Yes	--	Yes	--	--	--	--	Yes	--	--
G4ST01003419326	chr1:3419327-3419355	-	GGGCCAAGCAGCCTTGGGGAGGGGTCGGG<br>.((((....))))	MEGF6	NM_001409	chr1:3394366-3517919	non-template	29	Intron_7	--	Yes	--	Yes	--	--	--	--	--	--	--
G4ST01003421327	chr1:3421328-3421362	-	GGGCTAAGAAGGGTGGTGGGAGGAGCTGGCCTGGG<br>(((......)))	MEGF6	NM_001409	chr1:3394366-3517919	non-template	35	Intron_6	--	Yes	--	Yes	--	--	--	--	--	--	--
G4ST01003424065	chr1:3424066-3424102	-	GGGTAGGGGTGGCTGGGCTGTGTGTTTTGCAGCTGGG<br>(((((.....)))))..	MEGF6	NM_001409	chr1:3394366-3517919	non-template	37	Intron_5	--	Yes	--	Yes	--	--	--	--	Yes	--	--
G4ST01003426385	chr1:3426386-3426421	+	GGGGGACACAGGGAGGGGATGGCCAGGCCACCAGGG<br>..((((...))))...	MEGF6	NM_001409	chr1:3394366-3517919	template	36	Intron_5	--	Yes	--	Yes	--	--	--	--	--	--	--
G4ST01003427078	chr1:3427079-3427116	+	GGGCTGGGGAACGGGGACCCACACTCTCACAGTGTGGG<br>.....(((((.....)))))	MEGF6	NM_001409	chr1:3394366-3517919	template	38	Intron_5	--	Yes	--	Yes	--	--	--	--	--	--	Yes
G4ST01003429967	chr1:3429968-3429997	-	GGGTGTGGGCTGTGGCAGGTAGGGTAGGGG<br>(((...)))...	MEGF6	NM_001409	chr1:3394366-3517919	non-template	30	Intron_5	--	Yes	--	Yes	--	--	--	--	--	--	--
G4ST01003430733	chr1:3430734-3430767	-	GGGACCTGGGTCTGGGCCCCCGGCACCTGGGGGG<br>..((((.....))))	MEGF6	NM_001409	chr1:3394366-3517919	non-template	34	Intron_4	--	Yes	--	Yes	--	--	--	--	--	--	Yes
G4ST01003430757	chr1:3430758-3430791	-	GGGTGGTGCCCTCGAGGCACGGGAGGGACCTGGG<br>..(((((.....)))))	MEGF6	NM_001409	chr1:3394366-3517919	non-template	34	Intron_4	--	Yes	--	Yes	--	--	--	--	--	--	Yes
G4ST01003430764	chr1:3430765-3430800	-	GGGGCTCATGGGTGGTGCCCTCGAGGCACGGGAGGG<br>..(((((.....)))))	MEGF6	NM_001409	chr1:3394366-3517919	non-template	36	Intron_4	--	Yes	--	Yes	--	--	--	--	--	--	Yes
G4ST01003430768	chr1:3430769-3430806	-	GGGTGAGGGGCTCATGGGTGGTGCCCTCGAGGCACGGG<br>..(((((.....)))))	MEGF6	NM_001409	chr1:3394366-3517919	non-template	38	Intron_4	--	Yes	--	Yes	--	--	--	--	--	--	Yes
G4ST01003433884	chr1:3433885-3433918	-	GGGAGGCAGCCTCAGGGCTTAGGGACCCTCGGGG<br>(((...)))..	MEGF6	NM_001409	chr1:3394366-3517919	non-template	34	Intron_4	--	Yes	--	Yes	--	--	--	--	--	--	--
G4ST01003443495	chr1:3443496-3443527	+	GGGCTGGGTGTGGGAGCCAGAGAGGCTGTGGG<br>((((.....))))..	MEGF6	NM_001409	chr1:3394366-3517919	template	32	Intron_4	--	Yes	--	Yes	--	--	--	--	--	--	Yes
G4ST01003443500	chr1:3443501-3443538	+	GGGTGTGGGAGCCAGAGAGGCTGTGGGCATAGTGGGGG<br>((((.....))))..	MEGF6	NM_001409	chr1:3394366-3517919	template	38	Intron_4	--	Yes	--	Yes	--	--	--	--	--	--	Yes
G4ST01003448615	chr1:3448616-3448648	+	GGGAAGGCAGCCCTCGGGCCTGGATGGGTGGGG<br>.(((....))).	MEGF6	NM_001409	chr1:3394366-3517919	template	33	Intron_4	--	Yes	--	Yes	--	--	--	Yes	Yes	--	--
G4ST01003449754	chr1:3449755-3449783	-	GGGTTCGGGCTGCTCAGAGCAGGGAAGGG<br>..(((...))).	MEGF6	NM_001409	chr1:3394366-3517919	non-template	29	Intron_4	--	Yes	--	Yes	--	--	--	--	Yes	Yes	--
G4ST01003453163	chr1:3453164-3453199	+	GGGAAGGAGGGTGCCAGGGAGGCCACCATGGCGGGG<br>..((((...)))).	MEGF6	NM_001409	chr1:3394366-3517919	template	36	Intron_4	--	Yes	--	Yes	--	--	--	--	--	--	Yes
G4ST01003453298	chr1:3453299-3453326	+	GGGCTGGGAAGGGGGCTGCCAGGCGGGG<br>.(((....))).	MEGF6	NM_001409	chr1:3394366-3517919	template	28	Intron_4	--	Yes	--	Yes	--	--	--	--	--	--	Yes
G4ST01003453303	chr1:3453304-3453336	+	GGGAAGGGGGCTGCCAGGCGGGGTGGCAGTGGG<br>.(((....))).	MEGF6	NM_001409	chr1:3394366-3517919	template	33	Intron_4	--	Yes	--	Yes	--	--	--	--	--	--	Yes
G4ST01003453308	chr1:3453309-3453341	+	GGGGGCTGCCAGGCGGGGTGGCAGTGGGCTGGG<br>.(((....))).	MEGF6	NM_001409	chr1:3394366-3517919	template	33	Intron_4	--	Yes	--	Yes	--	--	--	--	--	--	Yes
G4ST01003453333	chr1:3453334-3453361	+	GGGCTGGGAAGGGGGCTGCCAGGCGGGG<br>.(((....))).	MEGF6	NM_001409	chr1:3394366-3517919	template	28	Intron_4	--	Yes	--	Yes	--	--	--	--	--	--	Yes
G4ST01003453338	chr1:3453339-3453371	+	GGGAAGGGGGCTGCCAGGCGGGGTGGCAGTGGG<br>.(((....))).	MEGF6	NM_001409	chr1:3394366-3517919	template	33	Intron_4	--	Yes	--	Yes	--	--	--	--	--	--	Yes
G4ST01003453343	chr1:3453344-3453376	+	GGGGGCTGCCAGGCGGGGTGGCAGTGGGCTGGG<br>.(((....))).	MEGF6	NM_001409	chr1:3394366-3517919	template	33	Intron_4	--	Yes	--	Yes	--	--	--	--	--	--	Yes
G4ST01003454135	chr1:3454136-3454166	-	GGGCATGGGCTCTGGGTCCTGGCAGGCTGGG<br>.(((...)))..	MEGF6	NM_001409	chr1:3394366-3517919	non-template	31	Intron_4	--	Yes	--	Yes	--	--	--	--	Yes	Yes	Yes
G4ST01003458523	chr1:3458524-3458555	-	GGGGAGCCACCGCTGGGGTCCTGAGGGTTGGG<br>.(((....))).	MEGF6	NM_001409	chr1:3394366-3517919	non-template	32	Intron_4	--	Yes	--	Yes	--	--	--	--	--	--	--
G4ST01003459665	chr1:3459666-3459698	-	GGGGGGAGCTGGGCAGGCTGGGCCCCTGGTGGG<br>((...)).	MEGF6	NM_001409	chr1:3394366-3517919	non-template	33	Intron_4	--	Yes	--	Yes	--	--	--	--	--	--	Yes
G4ST01003461425	chr1:3461426-3461450	-	GGGAGAGGGCCGGGCAGGACCTGGG<br>.((...))	MEGF6	NM_001409	chr1:3394366-3517919	non-template	25	Intron_4	--	Yes	--	Yes	--	--	--	--	Yes	--	--
G4ST01003473925	chr1:3473926-3473960	-	GGGGGGGGCATGTCCATGCTGGGGGTGCAGGAGGG<br>((((....))))...	MEGF6	NM_001409	chr1:3394366-3517919	non-template	35	Intron_4	--	Yes	--	Yes	--	--	--	--	--	--	--
G4ST01003473935	chr1:3473936-3473970	-	GGGAGAGCCTGGGGGGGGCATGTCCATGCTGGGGG<br>((((....))))...	MEGF6	NM_001409	chr1:3394366-3517919	non-template	35	Intron_4	--	Yes	--	Yes	--	--	--	--	--	--	--
G4ST01003474349	chr1:3474350-3474380	-	GGGTCCAGGGTGGCGGGCAGCAGCCTGGGGG<br>(((....))).	MEGF6	NM_001409	chr1:3394366-3517919	non-template	31	Intron_4	--	Yes	--	Yes	--	--	--	--	--	--	Yes
G4ST01003477124	chr1:3477125-3477151	-	GGGCCTGGCACAGGGGCAGGGCAGGGG<br>.(((...)))	MEGF6	NM_001409	chr1:3394366-3517919	non-template	27	Intron_4	--	Yes	--	Yes	--	--	--	Yes	Yes	--	--
G4ST01003488267	chr1:3488268-3488306	-	GGGCTCCAGGAGAGGGGCCCTGAGAACAGGAGGGTGGGG<br>(((.....))) ..((((....)))).	MEGF6	NM_001409	chr1:3394366-3517919	non-template	39	Intron_3	--	Yes	--	Yes	--	--	--	--	--	--	--
G4ST01003489224	chr1:3489225-3489261	-	GGGGCGCTGTGGGCTCTGATTTGGAGACGGGTTAGGG<br>((((.....))))..	MEGF6	NM_001409	chr1:3394366-3517919	non-template	37	Intron_3	--	Yes	--	Yes	--	--	--	--	--	--	--
G4ST01003489230	chr1:3489231-3489273	-	GGGGGCAAGAGCGGGGCGCTGTGGGCTCTGATTTGGAGACGGG<br>((((.....))))..	MEGF6	NM_001409	chr1:3394366-3517919	non-template	43	Intron_3	--	Yes	--	Yes	--	--	--	--	--	--	--
G4ST01003502322	chr1:3502323-3502351	+	GGGACCGGGTGGGCTGTGCCAGGCAGGGG<br>((((.....))))	MEGF6	NM_001409	chr1:3394366-3517919	template	29	Intron_2	--	Yes	--	Yes	--	--	--	--	--	--	--
G4ST01003502759	chr1:3502760-3502795	-	GGGGAGGGGGACGTCATGTCCTCTGGGTAGCTGGGG<br>((((.....))))...	MEGF6	NM_001409	chr1:3394366-3517919	non-template	36	Intron_2	--	Yes	--	Yes	--	--	--	--	--	--	--
G4ST01003509403	chr1:3509404-3509439	-	GGGGACATCTCTGAAGCGAGGTGGGGAGGGCTGGGG<br>..((((((......))))))	MEGF6	NM_001409	chr1:3394366-3517919	non-template	36	Intron_1.f	--	Yes	--	Yes	--	--	--	--	--	--	--
G4ST01003510341	chr1:3510342-3510373	-	GGGGGTGGGCTGAAGTCAGAGGGGACACAGGG<br>(((....)))..	MEGF6	NM_001409	chr1:3394366-3517919	non-template	32	Intron_1.f	--	Yes	--	Yes	--	--	--	--	--	--	--
G4ST01003512367	chr1:3512368-3512396	-	GGGGTTGGGGAGGGATCTTCATGAAGGGG<br>..(((....)))	MEGF6	NM_001409	chr1:3394366-3517919	non-template	29	Intron_1.f	--	Yes	--	Yes	--	--	--	--	--	--	Yes
G4ST01003515623	chr1:3515624-3515651	-	GGGCTCTGGTACGGGGGTGGGGCAAGGG<br>..(((...)))	MEGF6	NM_001409	chr1:3394366-3517919	non-template	28	Intron_1.f	--	Yes	--	Yes	--	--	--	Yes	--	Yes	--
G4ST01003519155	chr1:3519156-3519182	+	GGGAGCCCTGGCTGGGAGGGGCTTGGG<br>(((....)))	MEGF6	NM_001409	chr1:3394366-3517919	template	-1236	upstream	Yes	--	--	--	--	--	--	--	--	--	Yes
G4ST01003531138	chr1:3531139-3531173	-	GGGTGTCGCGGAGCGGCTGGGAAGGAGGGGCTGGG<br>.(((((...))))).	TPRG1L	NM_182752	chr1:3531416-3536554	template	-242	upstream	Yes	--	--	--	--	--	--	--	Yes	Yes	--
G4ST01003531469	chr1:3531470-3531512	+	GGGTCGGGGTCGGTAAGGGGTGCGGCAATGCTGCAACTGCGGG<br>.((((((...)))))).....	TPRG1L	NM_182752	chr1:3531416-3536554	non-template	43	Exon_1.f	--	Yes	Yes	--	Yes	Yes	--	--	Yes	Yes	--
G4ST01003538191	chr1:3538192-3538232	+	GGGGTGGGGCGGCTGCACCCTGCAGTCTGGGTTCAGTTGGG<br>..(((((((...))))))).	WDR8	NM_017818	chr1:3537191-3556531	template	41	Intron_10	--	Yes	--	Yes	--	--	--	--	--	--	--
G4ST01003538196	chr1:3538197-3538242	+	GGGGCGGCTGCACCCTGCAGTCTGGGTTCAGTTGGGCCCTGTAGGG<br>..(((((((...))))))).	WDR8	NM_017818	chr1:3537191-3556531	template	46	Intron_10	--	Yes	--	Yes	--	--	--	--	--	--	--
G4ST01003563636	chr1:3563637-3563674	+	GGGCAGGGTCGGGTTCCCTCGGAGGCTCTGAGAGGGGG<br>....((((((...))))))...	TP73	NM_005427	chr1:3558989-3642625	non-template	38	Intron_1.f	--	Yes	--	Yes	--	--	--	--	Yes	Yes	--
G4ST01003563636	chr1:3563637-3563674	+	GGGCAGGGTCGGGTTCCCTCGGAGGCTCTGAGAGGGGG<br>....((((((...))))))...	TP73	NM_001204184	chr1:3558989-3642625	non-template	38	Intron_1.f	--	Yes	--	Yes	--	--	--	--	Yes	Yes	--
G4ST01003563636	chr1:3563637-3563674	+	GGGCAGGGTCGGGTTCCCTCGGAGGCTCTGAGAGGGGG<br>....((((((...))))))...	TP73	NM_001204185	chr1:3558989-3642625	non-template	38	Intron_1.f	--	Yes	--	Yes	--	--	--	--	Yes	Yes	--

G4ST01003563636	chr1:3563637-3563674	+	GGGCAGGGTCGGGTTCCCTCGGAGGCTCTGAGAGGGGG<br>....((((((...))))))...	TP73	NM_001204186	chr1:3558989-3642625	non-template	38	Intron_1.f	--	Yes	--	Yes	--	--	--	--	Yes	Yes	--
G4ST01003563636	chr1:3563637-3563674	+	GGGCAGGGTCGGGTTCCCTCGGAGGCTCTGAGAGGGGG<br>....((((((...))))))...	TP73	NM_001204187	chr1:3558989-3642625	non-template	38	Intron_1.f	--	Yes	--	Yes	--	--	--	--	Yes	Yes	--
G4ST01003563636	chr1:3563637-3563674	+	GGGCAGGGTCGGGTTCCCTCGGAGGCTCTGAGAGGGGG<br>....((((((...))))))...	TP73	NM_001204188	chr1:3558989-3642625	non-template	38	Intron_1.f	--	Yes	--	Yes	--	--	--	--	Yes	Yes	--
G4ST01003570810	chr1:3570811-3570841	+	GGGTTGGGGGTGGCACCTGGGGAAGGCTGGG<br>(((...)))..	TP73	NM_005427	chr1:3558989-3642625	non-template	31	Intron_1.f	--	Yes	--	Yes	--	--	--	Yes	Yes	Yes	--
G4ST01003570810	chr1:3570811-3570841	+	GGGTTGGGGGTGGCACCTGGGGAAGGCTGGG<br>(((...)))..	TP73	NM_001204184	chr1:3558989-3642625	non-template	31	Intron_1.f	--	Yes	--	Yes	--	--	--	Yes	Yes	Yes	--
G4ST01003570810	chr1:3570811-3570841	+	GGGTTGGGGGTGGCACCTGGGGAAGGCTGGG<br>(((...)))..	TP73	NM_001204185	chr1:3558989-3642625	non-template	31	Intron_1.f	--	Yes	--	Yes	--	--	--	Yes	Yes	Yes	--
G4ST01003570810	chr1:3570811-3570841	+	GGGTTGGGGGTGGCACCTGGGGAAGGCTGGG<br>(((...)))..	TP73	NM_001204186	chr1:3558989-3642625	non-template	31	Intron_1.f	--	Yes	--	Yes	--	--	--	Yes	Yes	Yes	--
G4ST01003570810	chr1:3570811-3570841	+	GGGTTGGGGGTGGCACCTGGGGAAGGCTGGG<br>(((...)))..	TP73	NM_001204187	chr1:3558989-3642625	non-template	31	Intron_1.f	--	Yes	--	Yes	--	--	--	Yes	Yes	Yes	--
G4ST01003570810	chr1:3570811-3570841	+	GGGTTGGGGGTGGCACCTGGGGAAGGCTGGG<br>(((...)))..	TP73	NM_001204188	chr1:3558989-3642625	non-template	31	Intron_1.f	--	Yes	--	Yes	--	--	--	Yes	Yes	Yes	--
G4ST01003570815	chr1:3570816-3570850	+	GGGGGTGGCACCTGGGGAAGGCTGGGCTGAGGGGG<br>(((...)))..	TP73	NM_005427	chr1:3558989-3642625	non-template	35	Intron_1.f	--	Yes	--	Yes	--	--	--	Yes	Yes	Yes	--
G4ST01003570815	chr1:3570816-3570850	+	GGGGGTGGCACCTGGGGAAGGCTGGGCTGAGGGGG<br>(((...)))..	TP73	NM_001204184	chr1:3558989-3642625	non-template	35	Intron_1.f	--	Yes	--	Yes	--	--	--	Yes	Yes	Yes	--
G4ST01003570815	chr1:3570816-3570850	+	GGGGGTGGCACCTGGGGAAGGCTGGGCTGAGGGGG<br>(((...)))..	TP73	NM_001204185	chr1:3558989-3642625	non-template	35	Intron_1.f	--	Yes	--	Yes	--	--	--	Yes	Yes	Yes	--
G4ST01003570815	chr1:3570816-3570850	+	GGGGGTGGCACCTGGGGAAGGCTGGGCTGAGGGGG<br>(((...)))..	TP73	NM_001204186	chr1:3558989-3642625	non-template	35	Intron_1.f	--	Yes	--	Yes	--	--	--	Yes	Yes	Yes	--
G4ST01003570815	chr1:3570816-3570850	+	GGGGGTGGCACCTGGGGAAGGCTGGGCTGAGGGGG<br>(((...)))..	TP73	NM_001204187	chr1:3558989-3642625	non-template	35	Intron_1.f	--	Yes	--	Yes	--	--	--	Yes	Yes	Yes	--
G4ST01003570815	chr1:3570816-3570850	+	GGGGGTGGCACCTGGGGAAGGCTGGGCTGAGGGGG<br>(((...)))..	TP73	NM_001204188	chr1:3558989-3642625	non-template	35	Intron_1.f	--	Yes	--	Yes	--	--	--	Yes	Yes	Yes	--
G4ST01003577616	chr1:3577617-3577656	+	GGGATGGCAGGGGTGGGCCTGTCCCTGGAGAGGCGTGGGG<br>(((.((....)).)))....	TP73	NM_005427	chr1:3558989-3642625	non-template	40	Intron_1.f	--	Yes	--	Yes	--	--	--	--	--	Yes	--
G4ST01003577616	chr1:3577617-3577656	+	GGGATGGCAGGGGTGGGCCTGTCCCTGGAGAGGCGTGGGG<br>(((.((....)).)))....	TP73	NM_001204184	chr1:3558989-3642625	non-template	40	Intron_1.f	--	Yes	--	Yes	--	--	--	--	--	Yes	--
G4ST01003577616	chr1:3577617-3577656	+	GGGATGGCAGGGGTGGGCCTGTCCCTGGAGAGGCGTGGGG<br>(((.((....)).)))....	TP73	NM_001204185	chr1:3558989-3642625	non-template	40	Intron_1.f	--	Yes	--	Yes	--	--	--	--	--	Yes	--
G4ST01003577616	chr1:3577617-3577656	+	GGGATGGCAGGGGTGGGCCTGTCCCTGGAGAGGCGTGGGG<br>(((.((....)).)))....	TP73	NM_001204186	chr1:3558989-3642625	non-template	40	Intron_1.f	--	Yes	--	Yes	--	--	--	--	--	Yes	--
G4ST01003577616	chr1:3577617-3577656	+	GGGATGGCAGGGGTGGGCCTGTCCCTGGAGAGGCGTGGGG<br>(((.((....)).)))....	TP73	NM_001204187	chr1:3558989-3642625	non-template	40	Intron_1.f	--	Yes	--	Yes	--	--	--	--	--	Yes	--
G4ST01003577616	chr1:3577617-3577656	+	GGGATGGCAGGGGTGGGCCTGTCCCTGGAGAGGCGTGGGG<br>(((.((....)).)))....	TP73	NM_001204188	chr1:3558989-3642625	non-template	40	Intron_1.f	--	Yes	--	Yes	--	--	--	--	--	Yes	--
G4ST01003577625	chr1:3577626-3577662	+	GGGGTGGGCCTGTCCCTGGAGAGGCGTGGGGTGTGGG<br>(((.((....)).)))....	TP73	NM_005427	chr1:3558989-3642625	non-template	37	Intron_1.f	--	Yes	--	Yes	--	--	--	--	--	Yes	--
G4ST01003577625	chr1:3577626-3577662	+	GGGGTGGGCCTGTCCCTGGAGAGGCGTGGGGTGTGGG<br>(((.((....)).)))....	TP73	NM_001204184	chr1:3558989-3642625	non-template	37	Intron_1.f	--	Yes	--	Yes	--	--	--	--	--	Yes	--
G4ST01003577625	chr1:3577626-3577662	+	GGGGTGGGCCTGTCCCTGGAGAGGCGTGGGGTGTGGG<br>(((.((....)).)))....	TP73	NM_001204185	chr1:3558989-3642625	non-template	37	Intron_1.f	--	Yes	--	Yes	--	--	--	--	--	Yes	--
G4ST01003577625	chr1:3577626-3577662	+	GGGGTGGGCCTGTCCCTGGAGAGGCGTGGGGTGTGGG<br>(((.((....)).)))....	TP73	NM_001204186	chr1:3558989-3642625	non-template	37	Intron_1.f	--	Yes	--	Yes	--	--	--	--	--	Yes	--
G4ST01003577625	chr1:3577626-3577662	+	GGGGTGGGCCTGTCCCTGGAGAGGCGTGGGGTGTGGG<br>(((.((....)).)))....	TP73	NM_001204187	chr1:3558989-3642625	non-template	37	Intron_1.f	--	Yes	--	Yes	--	--	--	--	--	Yes	--
G4ST01003577625	chr1:3577626-3577662	+	GGGGTGGGCCTGTCCCTGGAGAGGCGTGGGGTGTGGG<br>(((.((....)).)))....	TP73	NM_001204188	chr1:3558989-3642625	non-template	37	Intron_1.f	--	Yes	--	Yes	--	--	--	--	--	Yes	--
G4ST01003577630	chr1:3577631-3577669	+	GGGCCTGTCCCTGGAGAGGCGTGGGGTGTGGGAGCTGGG<br>(((.((....)).)))....	TP73	NM_005427	chr1:3558989-3642625	non-template	39	Intron_1.f	--	Yes	--	Yes	--	--	--	--	--	Yes	--
G4ST01003577630	chr1:3577631-3577669	+	GGGCCTGTCCCTGGAGAGGCGTGGGGTGTGGGAGCTGGG<br>(((.((....)).)))....	TP73	NM_001204184	chr1:3558989-3642625	non-template	39	Intron_1.f	--	Yes	--	Yes	--	--	--	--	--	Yes	--
G4ST01003577630	chr1:3577631-3577669	+	GGGCCTGTCCCTGGAGAGGCGTGGGGTGTGGGAGCTGGG<br>(((.((....)).)))....	TP73	NM_001204185	chr1:3558989-3642625	non-template	39	Intron_1.f	--	Yes	--	Yes	--	--	--	--	--	Yes	--
G4ST01003577630	chr1:3577631-3577669	+	GGGCCTGTCCCTGGAGAGGCGTGGGGTGTGGGAGCTGGG<br>(((.((....)).)))....	TP73	NM_001204186	chr1:3558989-3642625	non-template	39	Intron_1.f	--	Yes	--	Yes	--	--	--	--	--	Yes	--
G4ST01003577630	chr1:3577631-3577669	+	GGGCCTGTCCCTGGAGAGGCGTGGGGTGTGGGAGCTGGG<br>(((.((....)).)))....	TP73	NM_001204187	chr1:3558989-3642625	non-template	39	Intron_1.f	--	Yes	--	Yes	--	--	--	--	--	Yes	--
G4ST01003577630	chr1:3577631-3577669	+	GGGCCTGTCCCTGGAGAGGCGTGGGGTGTGGGAGCTGGG<br>(((.((....)).)))....	TP73	NM_001204188	chr1:3558989-3642625	non-template	39	Intron_1.f	--	Yes	--	Yes	--	--	--	--	--	Yes	--
G4ST01003580697	chr1:3580698-3580726	-	GGGCCGTGGCCCACGGGAAGCGGGAGGGG<br>..(((...)))	TP73	NM_005427	chr1:3558989-3642625	template	29	Intron_1.f	--	Yes	--	Yes	--	--	--	Yes	Yes	Yes	Yes
G4ST01003580697	chr1:3580698-3580726	-	GGGCCGTGGCCCACGGGAAGCGGGAGGGG<br>..(((...)))	TP73	NM_001204184	chr1:3558989-3642625	template	29	Intron_1.f	--	Yes	--	Yes	--	--	--	Yes	Yes	Yes	Yes
G4ST01003580697	chr1:3580698-3580726	-	GGGCCGTGGCCCACGGGAAGCGGGAGGGG<br>..(((...)))	TP73	NM_001204185	chr1:3558989-3642625	template	29	Intron_1.f	--	Yes	--	Yes	--	--	--	Yes	Yes	Yes	Yes
G4ST01003580697	chr1:3580698-3580726	-	GGGCCGTGGCCCACGGGAAGCGGGAGGGG<br>..(((...)))	TP73	NM_001204186	chr1:3558989-3642625	template	29	Intron_1.f	--	Yes	--	Yes	--	--	--	Yes	Yes	Yes	Yes
G4ST01003580697	chr1:3580698-3580726	-	GGGCCGTGGCCCACGGGAAGCGGGAGGGG<br>..(((...)))	TP73	NM_001204187	chr1:3558989-3642625	template	29	Intron_1.f	--	Yes	--	Yes	--	--	--	Yes	Yes	Yes	Yes
G4ST01003580697	chr1:3580698-3580726	-	GGGCCGTGGCCCACGGGAAGCGGGAGGGG<br>..(((...)))	TP73	NM_001204188	chr1:3558989-3642625	template	29	Intron_1.f	--	Yes	--	Yes	--	--	--	Yes	Yes	Yes	Yes
G4ST01003581420	chr1:3581421-3581457	-	GGGCCATTGTGGGAGGAGACACCTGGGCCATTGTGGG<br>(((.....)))	TP73	NM_005427	chr1:3558989-3642625	template	37	Intron_1.f	--	Yes	--	Yes	--	--	--	Yes	--	--	--
G4ST01003581420	chr1:3581421-3581457	-	GGGCCATTGTGGGAGGAGACACCTGGGCCATTGTGGG<br>(((.....)))	TP73	NM_001204184	chr1:3558989-3642625	template	37	Intron_1.f	--	Yes	--	Yes	--	--	--	Yes	--	--	--
G4ST01003581420	chr1:3581421-3581457	-	GGGCCATTGTGGGAGGAGACACCTGGGCCATTGTGGG<br>(((.....)))	TP73	NM_001204185	chr1:3558989-3642625	template	37	Intron_1.f	--	Yes	--	Yes	--	--	--	Yes	--	--	--
G4ST01003581420	chr1:3581421-3581457	-	GGGCCATTGTGGGAGGAGACACCTGGGCCATTGTGGG<br>(((.....)))	TP73	NM_001204186	chr1:3558989-3642625	template	37	Intron_1.f	--	Yes	--	Yes	--	--	--	Yes	--	--	--
G4ST01003581420	chr1:3581421-3581457	-	GGGCCATTGTGGGAGGAGACACCTGGGCCATTGTGGG<br>(((.....)))	TP73	NM_001204187	chr1:3558989-3642625	template	37	Intron_1.f	--	Yes	--	Yes	--	--	--	Yes	--	--	--
G4ST01003581420	chr1:3581421-3581457	-	GGGCCATTGTGGGAGGAGACACCTGGGCCATTGTGGG<br>(((.....)))	TP73	NM_001204188	chr1:3558989-3642625	template	37	Intron_1.f	--	Yes	--	Yes	--	--	--	Yes	--	--	--
G4ST01003581430	chr1:3581431-3581471	-	GGGAGGAGACACCTGGGCCATTGTGGGAGGAGACACCTGGG<br>(((.....))) (((.....)))	TP73	NM_005427	chr1:3558989-3642625	template	41	Intron_1.f	--	Yes	--	Yes	--	--	--	--	--	--	--
G4ST01003581430	chr1:3581431-3581471	-	GGGAGGAGACACCTGGGCCATTGTGGGAGGAGACACCTGGG<br>(((.....))) (((.....)))	TP73	NM_001204184	chr1:3558989-3642625	template	41	Intron_1.f	--	Yes	--	Yes	--	--	--	--	--	--	--
G4ST01003581430	chr1:3581431-3581471	-	GGGAGGAGACACCTGGGCCATTGTGGGAGGAGACACCTGGG<br>(((.....))) (((.....)))	TP73	NM_001204185	chr1:3558989-3642625	template	41	Intron_1.f	--	Yes	--	Yes	--	--	--	--	--	--	--
G4ST01003581430	chr1:3581431-3581471	-	GGGAGGAGACACCTGGGCCATTGTGGGAGGAGACACCTGGG<br>(((.....))) (((.....)))	TP73	NM_001204186	chr1:3558989-3642625	template	41	Intron_1.f	--	Yes	--	Yes	--	--	--	--	--	--	--
G4ST01003581430	chr1:3581431-3581471	-	GGGAGGAGACACCTGGGCCATTGTGGGAGGAGACACCTGGG<br>(((.....))) (((.....)))	TP73	NM_001204187	chr1:3558989-3642625	template	41	Intron_1.f	--	Yes	--	Yes	--	--	--	--	--	--	--

G4ST01003581430	chr1:3581431-3581471	-	GGGAGGAGACACCTGGGCCATTGTGGGAGGAGACACCTGGG<br>(((.....))) (((.....)))	TP73	NM_001204188	chr1:3558989-3642625	template	41	Intron_1.f	--	Yes	--	Yes	--	--	--	--	--	--	--
G4ST01003581444	chr1:3581445-3581481	-	GGGCCATTGTGGGAGGAGACACCTGGGCCATTGTGGG<br>(((.....)))	TP73	NM_005427	chr1:3558989-3642625	template	37	Intron_1.f	--	Yes	--	Yes	--	--	--	--	--	--	--
G4ST01003581444	chr1:3581445-3581481	-	GGGCCATTGTGGGAGGAGACACCTGGGCCATTGTGGG<br>(((.....)))	TP73	NM_001204184	chr1:3558989-3642625	template	37	Intron_1.f	--	Yes	--	Yes	--	--	--	--	--	--	--
G4ST01003581444	chr1:3581445-3581481	-	GGGCCATTGTGGGAGGAGACACCTGGGCCATTGTGGG<br>(((.....)))	TP73	NM_001204185	chr1:3558989-3642625	template	37	Intron_1.f	--	Yes	--	Yes	--	--	--	--	--	--	--
G4ST01003581444	chr1:3581445-3581481	-	GGGCCATTGTGGGAGGAGACACCTGGGCCATTGTGGG<br>(((.....)))	TP73	NM_001204186	chr1:3558989-3642625	template	37	Intron_1.f	--	Yes	--	Yes	--	--	--	--	--	--	--
G4ST01003581444	chr1:3581445-3581481	-	GGGCCATTGTGGGAGGAGACACCTGGGCCATTGTGGG<br>(((.....)))	TP73	NM_001204187	chr1:3558989-3642625	template	37	Intron_1.f	--	Yes	--	Yes	--	--	--	--	--	--	--
G4ST01003581444	chr1:3581445-3581481	-	GGGCCATTGTGGGAGGAGACACCTGGGCCATTGTGGG<br>(((.....)))	TP73	NM_001204188	chr1:3558989-3642625	template	37	Intron_1.f	--	Yes	--	Yes	--	--	--	--	--	--	--
G4ST01003581454	chr1:3581455-3581495	-	GGGAGGAGACACCTGGGCCATTGTGGGAGGAGACACCTGGG<br>(((.....))) (((.....)))	TP73	NM_005427	chr1:3558989-3642625	template	41	Intron_1.f	--	Yes	--	Yes	--	--	--	--	--	--	--
G4ST01003581454	chr1:3581455-3581495	-	GGGAGGAGACACCTGGGCCATTGTGGGAGGAGACACCTGGG<br>(((.....))) (((.....)))	TP73	NM_001204184	chr1:3558989-3642625	template	41	Intron_1.f	--	Yes	--	Yes	--	--	--	--	--	--	--
G4ST01003581454	chr1:3581455-3581495	-	GGGAGGAGACACCTGGGCCATTGTGGGAGGAGACACCTGGG<br>(((.....))) (((.....)))	TP73	NM_001204185	chr1:3558989-3642625	template	41	Intron_1.f	--	Yes	--	Yes	--	--	--	--	--	--	--
G4ST01003581454	chr1:3581455-3581495	-	GGGAGGAGACACCTGGGCCATTGTGGGAGGAGACACCTGGG<br>(((.....))) (((.....)))	TP73	NM_001204186	chr1:3558989-3642625	template	41	Intron_1.f	--	Yes	--	Yes	--	--	--	--	--	--	--
G4ST01003581454	chr1:3581455-3581495	-	GGGAGGAGACACCTGGGCCATTGTGGGAGGAGACACCTGGG<br>(((.....))) (((.....)))	TP73	NM_001204187	chr1:3558989-3642625	template	41	Intron_1.f	--	Yes	--	Yes	--	--	--	--	--	--	--
G4ST01003581454	chr1:3581455-3581495	-	GGGAGGAGACACCTGGGCCATTGTGGGAGGAGACACCTGGG<br>(((.....))) (((.....)))	TP73	NM_001204188	chr1:3558989-3642625	template	41	Intron_1.f	--	Yes	--	Yes	--	--	--	--	--	--	--
G4ST01003581468	chr1:3581469-3581505	-	GGGCCATTGTGGGAGGAGACACCTGGGCCATTGTGGG<br>(((.....)))	TP73	NM_005427	chr1:3558989-3642625	template	37	Intron_1.f	--	Yes	--	Yes	--	--	--	--	--	--	--
G4ST01003581468	chr1:3581469-3581505	-	GGGCCATTGTGGGAGGAGACACCTGGGCCATTGTGGG<br>(((.....)))	TP73	NM_001204184	chr1:3558989-3642625	template	37	Intron_1.f	--	Yes	--	Yes	--	--	--	--	--	--	--
G4ST01003581468	chr1:3581469-3581505	-	GGGCCATTGTGGGAGGAGACACCTGGGCCATTGTGGG<br>(((.....)))	TP73	NM_001204185	chr1:3558989-3642625	template	37	Intron_1.f	--	Yes	--	Yes	--	--	--	--	--	--	--
G4ST01003581468	chr1:3581469-3581505	-	GGGCCATTGTGGGAGGAGACACCTGGGCCATTGTGGG<br>(((.....)))	TP73	NM_001204186	chr1:3558989-3642625	template	37	Intron_1.f	--	Yes	--	Yes	--	--	--	--	--	--	--
G4ST01003581468	chr1:3581469-3581505	-	GGGCCATTGTGGGAGGAGACACCTGGGCCATTGTGGG<br>(((.....)))	TP73	NM_001204187	chr1:3558989-3642625	template	37	Intron_1.f	--	Yes	--	Yes	--	--	--	--	--	--	--
G4ST01003581468	chr1:3581469-3581505	-	GGGCCATTGTGGGAGGAGACACCTGGGCCATTGTGGG<br>(((.....)))	TP73	NM_001204188	chr1:3558989-3642625	template	37	Intron_1.f	--	Yes	--	Yes	--	--	--	--	--	--	--
G4ST01003581478	chr1:3581479-3581519	-	GGGAGGAGACACCTGGGCCATTGTGGGAGGAGACACCTGGG<br>(((.....))) (((.....)))	TP73	NM_005427	chr1:3558989-3642625	template	41	Intron_1.f	--	Yes	--	Yes	--	--	--	--	--	--	--
G4ST01003581478	chr1:3581479-3581519	-	GGGAGGAGACACCTGGGCCATTGTGGGAGGAGACACCTGGG<br>(((.....))) (((.....)))	TP73	NM_001204184	chr1:3558989-3642625	template	41	Intron_1.f	--	Yes	--	Yes	--	--	--	--	--	--	--
G4ST01003581478	chr1:3581479-3581519	-	GGGAGGAGACACCTGGGCCATTGTGGGAGGAGACACCTGGG<br>(((.....))) (((.....)))	TP73	NM_001204185	chr1:3558989-3642625	template	41	Intron_1.f	--	Yes	--	Yes	--	--	--	--	--	--	--
G4ST01003581478	chr1:3581479-3581519	-	GGGAGGAGACACCTGGGCCATTGTGGGAGGAGACACCTGGG<br>(((.....))) (((.....)))	TP73	NM_001204186	chr1:3558989-3642625	template	41	Intron_1.f	--	Yes	--	Yes	--	--	--	--	--	--	--
G4ST01003581478	chr1:3581479-3581519	-	GGGAGGAGACACCTGGGCCATTGTGGGAGGAGACACCTGGG<br>(((.....))) (((.....)))	TP73	NM_001204187	chr1:3558989-3642625	template	41	Intron_1.f	--	Yes	--	Yes	--	--	--	--	--	--	--
G4ST01003581478	chr1:3581479-3581519	-	GGGAGGAGACACCTGGGCCATTGTGGGAGGAGACACCTGGG<br>(((.....))) (((.....)))	TP73	NM_001204188	chr1:3558989-3642625	template	41	Intron_1.f	--	Yes	--	Yes	--	--	--	--	--	--	--
G4ST01003581492	chr1:3581493-3581529	-	GGGCCATTGTGGGAGGAGACACCTGGGCCATTGTGGG<br>(((.....)))	TP73	NM_005427	chr1:3558989-3642625	template	37	Intron_1.f	--	Yes	--	Yes	--	--	--	--	--	--	--
G4ST01003581492	chr1:3581493-3581529	-	GGGCCATTGTGGGAGGAGACACCTGGGCCATTGTGGG<br>(((.....)))	TP73	NM_001204184	chr1:3558989-3642625	template	37	Intron_1.f	--	Yes	--	Yes	--	--	--	--	--	--	--
G4ST01003581492	chr1:3581493-3581529	-	GGGCCATTGTGGGAGGAGACACCTGGGCCATTGTGGG<br>(((.....)))	TP73	NM_001204185	chr1:3558989-3642625	template	37	Intron_1.f	--	Yes	--	Yes	--	--	--	--	--	--	--
G4ST01003581492	chr1:3581493-3581529	-	GGGCCATTGTGGGAGGAGACACCTGGGCCATTGTGGG<br>(((.....)))	TP73	NM_001204186	chr1:3558989-3642625	template	37	Intron_1.f	--	Yes	--	Yes	--	--	--	--	--	--	--
G4ST01003581492	chr1:3581493-3581529	-	GGGCCATTGTGGGAGGAGACACCTGGGCCATTGTGGG<br>(((.....)))	TP73	NM_001204187	chr1:3558989-3642625	template	37	Intron_1.f	--	Yes	--	Yes	--	--	--	--	--	--	--
G4ST01003581492	chr1:3581493-3581529	-	GGGCCATTGTGGGAGGAGACACCTGGGCCATTGTGGG<br>(((.....)))	TP73	NM_001204188	chr1:3558989-3642625	template	37	Intron_1.f	--	Yes	--	Yes	--	--	--	--	--	--	--
G4ST01003581502	chr1:3581503-3581543	-	GGGAGGAGACACCTGGGCCATTGTGGGAGGAGACACCTGGG<br>(((.....))) (((.....)))	TP73	NM_005427	chr1:3558989-3642625	template	41	Intron_1.f	--	Yes	--	Yes	--	--	--	--	--	--	--
G4ST01003581502	chr1:3581503-3581543	-	GGGAGGAGACACCTGGGCCATTGTGGGAGGAGACACCTGGG<br>(((.....))) (((.....)))	TP73	NM_001204184	chr1:3558989-3642625	template	41	Intron_1.f	--	Yes	--	Yes	--	--	--	--	--	--	--
G4ST01003581502	chr1:3581503-3581543	-	GGGAGGAGACACCTGGGCCATTGTGGGAGGAGACACCTGGG<br>(((.....))) (((.....)))	TP73	NM_001204185	chr1:3558989-3642625	template	41	Intron_1.f	--	Yes	--	Yes	--	--	--	--	--	--	--
G4ST01003581502	chr1:3581503-3581543	-	GGGAGGAGACACCTGGGCCATTGTGGGAGGAGACACCTGGG<br>(((.....))) (((.....)))	TP73	NM_001204186	chr1:3558989-3642625	template	41	Intron_1.f	--	Yes	--	Yes	--	--	--	--	--	--	--
G4ST01003581502	chr1:3581503-3581543	-	GGGAGGAGACACCTGGGCCATTGTGGGAGGAGACACCTGGG<br>(((.....))) (((.....)))	TP73	NM_001204187	chr1:3558989-3642625	template	41	Intron_1.f	--	Yes	--	Yes	--	--	--	--	--	--	--
G4ST01003581502	chr1:3581503-3581543	-	GGGAGGAGACACCTGGGCCATTGTGGGAGGAGACACCTGGG<br>(((.....))) (((.....)))	TP73	NM_001204188	chr1:3558989-3642625	template	41	Intron_1.f	--	Yes	--	Yes	--	--	--	--	--	--	--
G4ST01003581516	chr1:3581517-3581553	-	GGGCCATTGTGGGAGGAGACACCTGGGCCATTGTGGG<br>(((.....)))	TP73	NM_005427	chr1:3558989-3642625	template	37	Intron_1.f	--	Yes	--	Yes	--	--	--	--	--	--	--
G4ST01003581516	chr1:3581517-3581553	-	GGGCCATTGTGGGAGGAGACACCTGGGCCATTGTGGG<br>(((.....)))	TP73	NM_001204184	chr1:3558989-3642625	template	37	Intron_1.f	--	Yes	--	Yes	--	--	--	--	--	--	--
G4ST01003581516	chr1:3581517-3581553	-	GGGCCATTGTGGGAGGAGACACCTGGGCCATTGTGGG<br>(((.....)))	TP73	NM_001204185	chr1:3558989-3642625	template	37	Intron_1.f	--	Yes	--	Yes	--	--	--	--	--	--	--
G4ST01003581516	chr1:3581517-3581553	-	GGGCCATTGTGGGAGGAGACACCTGGGCCATTGTGGG<br>(((.....)))	TP73	NM_001204186	chr1:3558989-3642625	template	37	Intron_1.f	--	Yes	--	Yes	--	--	--	--	--	--	--
G4ST01003581516	chr1:3581517-3581553	-	GGGCCATTGTGGGAGGAGACACCTGGGCCATTGTGGG<br>(((.....)))	TP73	NM_001204187	chr1:3558989-3642625	template	37	Intron_1.f	--	Yes	--	Yes	--	--	--	--	--	--	--
G4ST01003581516	chr1:3581517-3581553	-	GGGCCATTGTGGGAGGAGACACCTGGGCCATTGTGGG<br>(((.....)))	TP73	NM_001204188	chr1:3558989-3642625	template	37	Intron_1.f	--	Yes	--	Yes	--	--	--	--	--	--	--
G4ST01003581526	chr1:3581527-3581567	-	GGGAGGAGACACCTGGGCCATTGTGGGAGGAGACACCTGGG<br>(((.....))) (((.....)))	TP73	NM_005427	chr1:3558989-3642625	template	41	Intron_1.f	--	Yes	--	Yes	--	--	--	--	--	--	--
G4ST01003581526	chr1:3581527-3581567	-	GGGAGGAGACACCTGGGCCATTGTGGGAGGAGACACCTGGG<br>(((.....))) (((.....)))	TP73	NM_001204184	chr1:3558989-3642625	template	41	Intron_1.f	--	Yes	--	Yes	--	--	--	--	--	--	--
G4ST01003581526	chr1:3581527-3581567	-	GGGAGGAGACACCTGGGCCATTGTGGGAGGAGACACCTGGG<br>(((.....))) (((.....)))	TP73	NM_001204185	chr1:3558989-3642625	template	41	Intron_1.f	--	Yes	--	Yes	--	--	--	--	--	--	--
G4ST01003581526	chr1:3581527-3581567	-	GGGAGGAGACACCTGGGCCATTGTGGGAGGAGACACCTGGG<br>(((.....))) (((.....)))	TP73	NM_001204186	chr1:3558989-3642625	template	41	Intron_1.f	--	Yes	--	Yes	--	--	--	--	--	--	--
G4ST01003581526	chr1:3581527-3581567	-	GGGAGGAGACACCTGGGCCATTGTGGGAGGAGACACCTGGG<br>(((.....))) (((.....)))	TP73	NM_001204187	chr1:3558989-3642625	template	41	Intron_1.f	--	Yes	--	Yes	--	--	--	--	--	--	--
G4ST01003581526	chr1:3581527-3581567	-	GGGAGGAGACACCTGGGCCATTGTGGGAGGAGACACCTGGG<br>(((.....))) (((.....)))	TP73	NM_001204188	chr1:3558989-3642625	template	41	Intron_1.f	--	Yes	--	Yes	--	--	--	--	--	--	--
G4ST01003581540	chr1:3581541-3581577	-	GGGCCATTGTGGGAGGAGACACCTGGGCCATTGTGGG<br>(((.....)))	TP73	NM_005427	chr1:3558989-3642625	template	37	Intron_1.f	--	Yes	--	Yes	--	--	--	--	--	--	--

G4ST01003581540	chr1:3581541-3581577	-	GGGCCATTGTGGGAGGAGACACCTGGGCCATTGTGGG<br>(((.....)))	TP73	NM_001204184	chr1:3558989-3642625	template	37	Intron_1.f	--	Yes	--	Yes	--	--	--	--	--	--	--
G4ST01003581540	chr1:3581541-3581577	-	GGGCCATTGTGGGAGGAGACACCTGGGCCATTGTGGG<br>(((.....)))	TP73	NM_001204185	chr1:3558989-3642625	template	37	Intron_1.f	--	Yes	--	Yes	--	--	--	--	--	--	--
G4ST01003581540	chr1:3581541-3581577	-	GGGCCATTGTGGGAGGAGACACCTGGGCCATTGTGGG<br>(((.....)))	TP73	NM_001204186	chr1:3558989-3642625	template	37	Intron_1.f	--	Yes	--	Yes	--	--	--	--	--	--	--
G4ST01003581540	chr1:3581541-3581577	-	GGGCCATTGTGGGAGGAGACACCTGGGCCATTGTGGG<br>(((.....)))	TP73	NM_001204187	chr1:3558989-3642625	template	37	Intron_1.f	--	Yes	--	Yes	--	--	--	--	--	--	--
G4ST01003581540	chr1:3581541-3581577	-	GGGCCATTGTGGGAGGAGACACCTGGGCCATTGTGGG<br>(((.....)))	TP73	NM_001204188	chr1:3558989-3642625	template	37	Intron_1.f	--	Yes	--	Yes	--	--	--	--	--	--	--
G4ST01003581550	chr1:3581551-3581591	-	GGGAGGAGACACCTGGGCCATTGTGGGAGGAGACACCTGGG<br>(((.....))) (((.....)))	TP73	NM_005427	chr1:3558989-3642625	template	41	Intron_1.f	--	Yes	--	Yes	--	--	--	--	--	--	--
G4ST01003581550	chr1:3581551-3581591	-	GGGAGGAGACACCTGGGCCATTGTGGGAGGAGACACCTGGG<br>(((.....))) (((.....)))	TP73	NM_001204184	chr1:3558989-3642625	template	41	Intron_1.f	--	Yes	--	Yes	--	--	--	--	--	--	--
G4ST01003581550	chr1:3581551-3581591	-	GGGAGGAGACACCTGGGCCATTGTGGGAGGAGACACCTGGG<br>(((.....))) (((.....)))	TP73	NM_001204185	chr1:3558989-3642625	template	41	Intron_1.f	--	Yes	--	Yes	--	--	--	--	--	--	--
G4ST01003581550	chr1:3581551-3581591	-	GGGAGGAGACACCTGGGCCATTGTGGGAGGAGACACCTGGG<br>(((.....))) (((.....)))	TP73	NM_001204186	chr1:3558989-3642625	template	41	Intron_1.f	--	Yes	--	Yes	--	--	--	--	--	--	--
G4ST01003581550	chr1:3581551-3581591	-	GGGAGGAGACACCTGGGCCATTGTGGGAGGAGACACCTGGG<br>(((.....))) (((.....)))	TP73	NM_001204187	chr1:3558989-3642625	template	41	Intron_1.f	--	Yes	--	Yes	--	--	--	--	--	--	--
G4ST01003581550	chr1:3581551-3581591	-	GGGAGGAGACACCTGGGCCATTGTGGGAGGAGACACCTGGG<br>(((.....))) (((.....)))	TP73	NM_001204188	chr1:3558989-3642625	template	41	Intron_1.f	--	Yes	--	Yes	--	--	--	--	--	--	--
G4ST01003581564	chr1:3581565-3581601	-	GGGCCATTGTGGGAGGAGACACCTGGGCCATTGTGGG<br>(((.....)))	TP73	NM_005427	chr1:3558989-3642625	template	37	Intron_1.f	--	Yes	--	Yes	--	--	--	--	--	--	--
G4ST01003581564	chr1:3581565-3581601	-	GGGCCATTGTGGGAGGAGACACCTGGGCCATTGTGGG<br>(((.....)))	TP73	NM_001204184	chr1:3558989-3642625	template	37	Intron_1.f	--	Yes	--	Yes	--	--	--	--	--	--	--
G4ST01003581564	chr1:3581565-3581601	-	GGGCCATTGTGGGAGGAGACACCTGGGCCATTGTGGG<br>(((.....)))	TP73	NM_001204185	chr1:3558989-3642625	template	37	Intron_1.f	--	Yes	--	Yes	--	--	--	--	--	--	--
G4ST01003581564	chr1:3581565-3581601	-	GGGCCATTGTGGGAGGAGACACCTGGGCCATTGTGGG<br>(((.....)))	TP73	NM_001204186	chr1:3558989-3642625	template	37	Intron_1.f	--	Yes	--	Yes	--	--	--	--	--	--	--
G4ST01003581564	chr1:3581565-3581601	-	GGGCCATTGTGGGAGGAGACACCTGGGCCATTGTGGG<br>(((.....)))	TP73	NM_001204187	chr1:3558989-3642625	template	37	Intron_1.f	--	Yes	--	Yes	--	--	--	--	--	--	--
G4ST01003581564	chr1:3581565-3581601	-	GGGCCATTGTGGGAGGAGACACCTGGGCCATTGTGGG<br>(((.....)))	TP73	NM_001204188	chr1:3558989-3642625	template	37	Intron_1.f	--	Yes	--	Yes	--	--	--	--	--	--	--
G4ST01003581574	chr1:3581575-3581615	-	GGGAGGAGACACCTGGGCCATTGTGGGAGGAGACACCTGGG<br>(((.....))) (((.....)))	TP73	NM_005427	chr1:3558989-3642625	template	41	Intron_1.f	--	Yes	--	Yes	--	--	--	--	--	--	--
G4ST01003581574	chr1:3581575-3581615	-	GGGAGGAGACACCTGGGCCATTGTGGGAGGAGACACCTGGG<br>(((.....))) (((.....)))	TP73	NM_001204184	chr1:3558989-3642625	template	41	Intron_1.f	--	Yes	--	Yes	--	--	--	--	--	--	--
G4ST01003581574	chr1:3581575-3581615	-	GGGAGGAGACACCTGGGCCATTGTGGGAGGAGACACCTGGG<br>(((.....))) (((.....)))	TP73	NM_001204185	chr1:3558989-3642625	template	41	Intron_1.f	--	Yes	--	Yes	--	--	--	--	--	--	--
G4ST01003581574	chr1:3581575-3581615	-	GGGAGGAGACACCTGGGCCATTGTGGGAGGAGACACCTGGG<br>(((.....))) (((.....)))	TP73	NM_001204186	chr1:3558989-3642625	template	41	Intron_1.f	--	Yes	--	Yes	--	--	--	--	--	--	--
G4ST01003581574	chr1:3581575-3581615	-	GGGAGGAGACACCTGGGCCATTGTGGGAGGAGACACCTGGG<br>(((.....))) (((.....)))	TP73	NM_001204187	chr1:3558989-3642625	template	41	Intron_1.f	--	Yes	--	Yes	--	--	--	--	--	--	--
G4ST01003581574	chr1:3581575-3581615	-	GGGAGGAGACACCTGGGCCATTGTGGGAGGAGACACCTGGG<br>(((.....))) (((.....)))	TP73	NM_001204188	chr1:3558989-3642625	template	41	Intron_1.f	--	Yes	--	Yes	--	--	--	--	--	--	--
G4ST01003581588	chr1:3581589-3581625	-	GGGCCATTGTGGGAGGAGACACCTGGGCCATTGTGGG<br>(((.....)))	TP73	NM_005427	chr1:3558989-3642625	template	37	Intron_1.f	--	Yes	--	Yes	--	--	--	--	--	--	--
G4ST01003581588	chr1:3581589-3581625	-	GGGCCATTGTGGGAGGAGACACCTGGGCCATTGTGGG<br>(((.....)))	TP73	NM_001204184	chr1:3558989-3642625	template	37	Intron_1.f	--	Yes	--	Yes	--	--	--	--	--	--	--
G4ST01003581588	chr1:3581589-3581625	-	GGGCCATTGTGGGAGGAGACACCTGGGCCATTGTGGG<br>(((.....)))	TP73	NM_001204185	chr1:3558989-3642625	template	37	Intron_1.f	--	Yes	--	Yes	--	--	--	--	--	--	--
G4ST01003581588	chr1:3581589-3581625	-	GGGCCATTGTGGGAGGAGACACCTGGGCCATTGTGGG<br>(((.....)))	TP73	NM_001204186	chr1:3558989-3642625	template	37	Intron_1.f	--	Yes	--	Yes	--	--	--	--	--	--	--
G4ST01003581588	chr1:3581589-3581625	-	GGGCCATTGTGGGAGGAGACACCTGGGCCATTGTGGG<br>(((.....)))	TP73	NM_001204187	chr1:3558989-3642625	template	37	Intron_1.f	--	Yes	--	Yes	--	--	--	--	--	--	--
G4ST01003581588	chr1:3581589-3581625	-	GGGCCATTGTGGGAGGAGACACCTGGGCCATTGTGGG<br>(((.....)))	TP73	NM_001204188	chr1:3558989-3642625	template	37	Intron_1.f	--	Yes	--	Yes	--	--	--	--	--	--	--
G4ST01003581598	chr1:3581599-3581639	-	GGGAGGAGACACCTGGGCCATTGTGGGAGGAGACACCTGGG<br>(((.....))) (((.....)))	TP73	NM_005427	chr1:3558989-3642625	template	41	Intron_1.f	--	Yes	--	Yes	--	--	--	--	--	--	--
G4ST01003581598	chr1:3581599-3581639	-	GGGAGGAGACACCTGGGCCATTGTGGGAGGAGACACCTGGG<br>(((.....))) (((.....)))	TP73	NM_001204184	chr1:3558989-3642625	template	41	Intron_1.f	--	Yes	--	Yes	--	--	--	--	--	--	--
G4ST01003581598	chr1:3581599-3581639	-	GGGAGGAGACACCTGGGCCATTGTGGGAGGAGACACCTGGG<br>(((.....))) (((.....)))	TP73	NM_001204185	chr1:3558989-3642625	template	41	Intron_1.f	--	Yes	--	Yes	--	--	--	--	--	--	--
G4ST01003581598	chr1:3581599-3581639	-	GGGAGGAGACACCTGGGCCATTGTGGGAGGAGACACCTGGG<br>(((.....))) (((.....)))	TP73	NM_001204186	chr1:3558989-3642625	template	41	Intron_1.f	--	Yes	--	Yes	--	--	--	--	--	--	--
G4ST01003581598	chr1:3581599-3581639	-	GGGAGGAGACACCTGGGCCATTGTGGGAGGAGACACCTGGG<br>(((.....))) (((.....)))	TP73	NM_001204187	chr1:3558989-3642625	template	41	Intron_1.f	--	Yes	--	Yes	--	--	--	--	--	--	--
G4ST01003581598	chr1:3581599-3581639	-	GGGAGGAGACACCTGGGCCATTGTGGGAGGAGACACCTGGG<br>(((.....))) (((.....)))	TP73	NM_001204188	chr1:3558989-3642625	template	41	Intron_1.f	--	Yes	--	Yes	--	--	--	--	--	--	--
G4ST01003581612	chr1:3581613-3581649	-	GGGCCATTGTGGGAGGAGACACCTGGGCCATTGTGGG<br>(((.....)))	TP73	NM_005427	chr1:3558989-3642625	template	37	Intron_1.f	--	Yes	--	Yes	--	--	--	--	--	--	--
G4ST01003581612	chr1:3581613-3581649	-	GGGCCATTGTGGGAGGAGACACCTGGGCCATTGTGGG<br>(((.....)))	TP73	NM_001204184	chr1:3558989-3642625	template	37	Intron_1.f	--	Yes	--	Yes	--	--	--	--	--	--	--
G4ST01003581612	chr1:3581613-3581649	-	GGGCCATTGTGGGAGGAGACACCTGGGCCATTGTGGG<br>(((.....)))	TP73	NM_001204185	chr1:3558989-3642625	template	37	Intron_1.f	--	Yes	--	Yes	--	--	--	--	--	--	--
G4ST01003581612	chr1:3581613-3581649	-	GGGCCATTGTGGGAGGAGACACCTGGGCCATTGTGGG<br>(((.....)))	TP73	NM_001204186	chr1:3558989-3642625	template	37	Intron_1.f	--	Yes	--	Yes	--	--	--	--	--	--	--
G4ST01003581612	chr1:3581613-3581649	-	GGGCCATTGTGGGAGGAGACACCTGGGCCATTGTGGG<br>(((.....)))	TP73	NM_001204187	chr1:3558989-3642625	template	37	Intron_1.f	--	Yes	--	Yes	--	--	--	--	--	--	--
G4ST01003581612	chr1:3581613-3581649	-	GGGCCATTGTGGGAGGAGACACCTGGGCCATTGTGGG<br>(((.....)))	TP73	NM_001204188	chr1:3558989-3642625	template	37	Intron_1.f	--	Yes	--	Yes	--	--	--	--	--	--	--
G4ST01003581622	chr1:3581623-3581663	-	GGGAGGAGACACCTGGGCCATTGTGGGAGGAGACACCTGGG<br>(((.....))) (((.....)))	TP73	NM_005427	chr1:3558989-3642625	template	41	Intron_1.f	--	Yes	--	Yes	--	--	--	--	--	--	--
G4ST01003581622	chr1:3581623-3581663	-	GGGAGGAGACACCTGGGCCATTGTGGGAGGAGACACCTGGG<br>(((.....))) (((.....)))	TP73	NM_001204184	chr1:3558989-3642625	template	41	Intron_1.f	--	Yes	--	Yes	--	--	--	--	--	--	--
G4ST01003581622	chr1:3581623-3581663	-	GGGAGGAGACACCTGGGCCATTGTGGGAGGAGACACCTGGG<br>(((.....))) (((.....)))	TP73	NM_001204185	chr1:3558989-3642625	template	41	Intron_1.f	--	Yes	--	Yes	--	--	--	--	--	--	--
G4ST01003581622	chr1:3581623-3581663	-	GGGAGGAGACACCTGGGCCATTGTGGGAGGAGACACCTGGG<br>(((.....))) (((.....)))	TP73	NM_001204186	chr1:3558989-3642625	template	41	Intron_1.f	--	Yes	--	Yes	--	--	--	--	--	--	--
G4ST01003581622	chr1:3581623-3581663	-	GGGAGGAGACACCTGGGCCATTGTGGGAGGAGACACCTGGG<br>(((.....))) (((.....)))	TP73	NM_001204187	chr1:3558989-3642625	template	41	Intron_1.f	--	Yes	--	Yes	--	--	--	--	--	--	--
G4ST01003581622	chr1:3581623-3581663	-	GGGAGGAGACACCTGGGCCATTGTGGGAGGAGACACCTGGG<br>(((.....))) (((.....)))	TP73	NM_001204188	chr1:3558989-3642625	template	41	Intron_1.f	--	Yes	--	Yes	--	--	--	--	--	--	--
G4ST01003581636	chr1:3581637-3581673	-	GGGCCATTGTGGGAGGAGACACCTGGGCCATTGTGGG<br>(((.....)))	TP73	NM_005427	chr1:3558989-3642625	template	37	Intron_1.f	--	Yes	--	Yes	--	--	--	--	--	--	--
G4ST01003581636	chr1:3581637-3581673	-	GGGCCATTGTGGGAGGAGACACCTGGGCCATTGTGGG<br>(((.....)))	TP73	NM_001204184	chr1:3558989-3642625	template	37	Intron_1.f	--	Yes	--	Yes	--	--	--	--	--	--	--
G4ST01003581636	chr1:3581637-3581673	-	GGGCCATTGTGGGAGGAGACACCTGGGCCATTGTGGG<br>(((.....)))	TP73	NM_001204185	chr1:3558989-3642625	template	37	Intron_1.f	--	Yes	--	Yes	--	--	--	--	--	--	--

G4ST01003581636	chr1:3581637-3581673	-	GGGCCATTGTGGGAGGAGACACCTGGGCCATTGTGGG<br>(((.....)))	TP73	NM_001204186	chr1:3558989-3642625	template	37	Intron_1.f	--	Yes	--	Yes	--	--	--	--	--	--	--
G4ST01003581636	chr1:3581637-3581673	-	GGGCCATTGTGGGAGGAGACACCTGGGCCATTGTGGG<br>(((.....)))	TP73	NM_001204187	chr1:3558989-3642625	template	37	Intron_1.f	--	Yes	--	Yes	--	--	--	--	--	--	--
G4ST01003581636	chr1:3581637-3581673	-	GGGCCATTGTGGGAGGAGACACCTGGGCCATTGTGGG<br>(((.....)))	TP73	NM_001204188	chr1:3558989-3642625	template	37	Intron_1.f	--	Yes	--	Yes	--	--	--	--	--	--	--
G4ST01003581646	chr1:3581647-3581687	-	GGGAGGAGACACCTGGGCCATTGTGGGAGGAGACACCTGGG<br>(((.....))) (((.....)))	TP73	NM_005427	chr1:3558989-3642625	template	41	Intron_1.f	--	Yes	--	Yes	--	--	--	Yes	--	--	--
G4ST01003581646	chr1:3581647-3581687	-	GGGAGGAGACACCTGGGCCATTGTGGGAGGAGACACCTGGG<br>(((.....))) (((.....)))	TP73	NM_001204184	chr1:3558989-3642625	template	41	Intron_1.f	--	Yes	--	Yes	--	--	--	Yes	--	--	--
G4ST01003581646	chr1:3581647-3581687	-	GGGAGGAGACACCTGGGCCATTGTGGGAGGAGACACCTGGG<br>(((.....))) (((.....)))	TP73	NM_001204185	chr1:3558989-3642625	template	41	Intron_1.f	--	Yes	--	Yes	--	--	--	Yes	--	--	--
G4ST01003581646	chr1:3581647-3581687	-	GGGAGGAGACACCTGGGCCATTGTGGGAGGAGACACCTGGG<br>(((.....))) (((.....)))	TP73	NM_001204186	chr1:3558989-3642625	template	41	Intron_1.f	--	Yes	--	Yes	--	--	--	Yes	--	--	--
G4ST01003581646	chr1:3581647-3581687	-	GGGAGGAGACACCTGGGCCATTGTGGGAGGAGACACCTGGG<br>(((.....))) (((.....)))	TP73	NM_001204187	chr1:3558989-3642625	template	41	Intron_1.f	--	Yes	--	Yes	--	--	--	Yes	--	--	--
G4ST01003581646	chr1:3581647-3581687	-	GGGAGGAGACACCTGGGCCATTGTGGGAGGAGACACCTGGG<br>(((.....))) (((.....)))	TP73	NM_001204188	chr1:3558989-3642625	template	41	Intron_1.f	--	Yes	--	Yes	--	--	--	Yes	--	--	--
G4ST01003581660	chr1:3581661-3581697	-	GGGCCATTGTGGGAGGAGACACCTGGGCCATTGTGGG<br>(((.....)))	TP73	NM_005427	chr1:3558989-3642625	template	37	Intron_1.f	--	Yes	--	Yes	--	--	--	Yes	--	--	--
G4ST01003581660	chr1:3581661-3581697	-	GGGCCATTGTGGGAGGAGACACCTGGGCCATTGTGGG<br>(((.....)))	TP73	NM_001204184	chr1:3558989-3642625	template	37	Intron_1.f	--	Yes	--	Yes	--	--	--	Yes	--	--	--
G4ST01003581660	chr1:3581661-3581697	-	GGGCCATTGTGGGAGGAGACACCTGGGCCATTGTGGG<br>(((.....)))	TP73	NM_001204185	chr1:3558989-3642625	template	37	Intron_1.f	--	Yes	--	Yes	--	--	--	Yes	--	--	--
G4ST01003581660	chr1:3581661-3581697	-	GGGCCATTGTGGGAGGAGACACCTGGGCCATTGTGGG<br>(((.....)))	TP73	NM_001204186	chr1:3558989-3642625	template	37	Intron_1.f	--	Yes	--	Yes	--	--	--	Yes	--	--	--
G4ST01003581660	chr1:3581661-3581697	-	GGGCCATTGTGGGAGGAGACACCTGGGCCATTGTGGG<br>(((.....)))	TP73	NM_001204187	chr1:3558989-3642625	template	37	Intron_1.f	--	Yes	--	Yes	--	--	--	Yes	--	--	--
G4ST01003581660	chr1:3581661-3581697	-	GGGCCATTGTGGGAGGAGACACCTGGGCCATTGTGGG<br>(((.....)))	TP73	NM_001204188	chr1:3558989-3642625	template	37	Intron_1.f	--	Yes	--	Yes	--	--	--	Yes	--	--	--
G4ST01003581670	chr1:3581671-3581711	-	GGGAGGAGACACCTGGGCCATTGTGGGAGGAGACACCTGGG<br>(((.....))) (((.....)))	TP73	NM_005427	chr1:3558989-3642625	template	41	Intron_1.f	--	Yes	--	Yes	--	--	--	Yes	--	--	--
G4ST01003581670	chr1:3581671-3581711	-	GGGAGGAGACACCTGGGCCATTGTGGGAGGAGACACCTGGG<br>(((.....))) (((.....)))	TP73	NM_001204184	chr1:3558989-3642625	template	41	Intron_1.f	--	Yes	--	Yes	--	--	--	Yes	--	--	--
G4ST01003581670	chr1:3581671-3581711	-	GGGAGGAGACACCTGGGCCATTGTGGGAGGAGACACCTGGG<br>(((.....))) (((.....)))	TP73	NM_001204185	chr1:3558989-3642625	template	41	Intron_1.f	--	Yes	--	Yes	--	--	--	Yes	--	--	--
G4ST01003581670	chr1:3581671-3581711	-	GGGAGGAGACACCTGGGCCATTGTGGGAGGAGACACCTGGG<br>(((.....))) (((.....)))	TP73	NM_001204186	chr1:3558989-3642625	template	41	Intron_1.f	--	Yes	--	Yes	--	--	--	Yes	--	--	--
G4ST01003581670	chr1:3581671-3581711	-	GGGAGGAGACACCTGGGCCATTGTGGGAGGAGACACCTGGG<br>(((.....))) (((.....)))	TP73	NM_001204187	chr1:3558989-3642625	template	41	Intron_1.f	--	Yes	--	Yes	--	--	--	Yes	--	--	--
G4ST01003581670	chr1:3581671-3581711	-	GGGAGGAGACACCTGGGCCATTGTGGGAGGAGACACCTGGG<br>(((.....))) (((.....)))	TP73	NM_001204188	chr1:3558989-3642625	template	41	Intron_1.f	--	Yes	--	Yes	--	--	--	Yes	--	--	--
G4ST01003581684	chr1:3581685-3581721	-	GGGCCATTGTGGGAGGAGACACCTGGGCCATTGTGGG<br>(((.....)))	TP73	NM_005427	chr1:3558989-3642625	template	37	Intron_1.f	--	Yes	--	Yes	--	--	--	Yes	--	--	--
G4ST01003581684	chr1:3581685-3581721	-	GGGCCATTGTGGGAGGAGACACCTGGGCCATTGTGGG<br>(((.....)))	TP73	NM_001204184	chr1:3558989-3642625	template	37	Intron_1.f	--	Yes	--	Yes	--	--	--	Yes	--	--	--
G4ST01003581684	chr1:3581685-3581721	-	GGGCCATTGTGGGAGGAGACACCTGGGCCATTGTGGG<br>(((.....)))	TP73	NM_001204185	chr1:3558989-3642625	template	37	Intron_1.f	--	Yes	--	Yes	--	--	--	Yes	--	--	--
G4ST01003581684	chr1:3581685-3581721	-	GGGCCATTGTGGGAGGAGACACCTGGGCCATTGTGGG<br>(((.....)))	TP73	NM_001204186	chr1:3558989-3642625	template	37	Intron_1.f	--	Yes	--	Yes	--	--	--	Yes	--	--	--
G4ST01003581684	chr1:3581685-3581721	-	GGGCCATTGTGGGAGGAGACACCTGGGCCATTGTGGG<br>(((.....)))	TP73	NM_001204187	chr1:3558989-3642625	template	37	Intron_1.f	--	Yes	--	Yes	--	--	--	Yes	--	--	--
G4ST01003581684	chr1:3581685-3581721	-	GGGCCATTGTGGGAGGAGACACCTGGGCCATTGTGGG<br>(((.....)))	TP73	NM_001204188	chr1:3558989-3642625	template	37	Intron_1.f	--	Yes	--	Yes	--	--	--	Yes	--	--	--
G4ST01003581694	chr1:3581695-3581735	-	GGGAGGAGACACCTGGGCCATTGTGGGAGGAGACACCTGGG<br>(((.....))) (((.....)))	TP73	NM_005427	chr1:3558989-3642625	template	41	Intron_1.f	--	Yes	--	Yes	--	--	--	Yes	--	--	--
G4ST01003581694	chr1:3581695-3581735	-	GGGAGGAGACACCTGGGCCATTGTGGGAGGAGACACCTGGG<br>(((.....))) (((.....)))	TP73	NM_001204184	chr1:3558989-3642625	template	41	Intron_1.f	--	Yes	--	Yes	--	--	--	Yes	--	--	--
G4ST01003581694	chr1:3581695-3581735	-	GGGAGGAGACACCTGGGCCATTGTGGGAGGAGACACCTGGG<br>(((.....))) (((.....)))	TP73	NM_001204185	chr1:3558989-3642625	template	41	Intron_1.f	--	Yes	--	Yes	--	--	--	Yes	--	--	--
G4ST01003581694	chr1:3581695-3581735	-	GGGAGGAGACACCTGGGCCATTGTGGGAGGAGACACCTGGG<br>(((.....))) (((.....)))	TP73	NM_001204186	chr1:3558989-3642625	template	41	Intron_1.f	--	Yes	--	Yes	--	--	--	Yes	--	--	--
G4ST01003581694	chr1:3581695-3581735	-	GGGAGGAGACACCTGGGCCATTGTGGGAGGAGACACCTGGG<br>(((.....))) (((.....)))	TP73	NM_001204187	chr1:3558989-3642625	template	41	Intron_1.f	--	Yes	--	Yes	--	--	--	Yes	--	--	--
G4ST01003581694	chr1:3581695-3581735	-	GGGAGGAGACACCTGGGCCATTGTGGGAGGAGACACCTGGG<br>(((.....))) (((.....)))	TP73	NM_001204188	chr1:3558989-3642625	template	41	Intron_1.f	--	Yes	--	Yes	--	--	--	Yes	--	--	--
G4ST01003581708	chr1:3581709-3581745	-	GGGCCATTGTGGGAGGAGACACCTGGGCCATTGTGGG<br>(((.....)))	TP73	NM_005427	chr1:3558989-3642625	template	37	Intron_1.f	--	Yes	--	Yes	--	--	--	Yes	--	--	--
G4ST01003581708	chr1:3581709-3581745	-	GGGCCATTGTGGGAGGAGACACCTGGGCCATTGTGGG<br>(((.....)))	TP73	NM_001204184	chr1:3558989-3642625	template	37	Intron_1.f	--	Yes	--	Yes	--	--	--	Yes	--	--	--
G4ST01003581708	chr1:3581709-3581745	-	GGGCCATTGTGGGAGGAGACACCTGGGCCATTGTGGG<br>(((.....)))	TP73	NM_001204185	chr1:3558989-3642625	template	37	Intron_1.f	--	Yes	--	Yes	--	--	--	Yes	--	--	--
G4ST01003581708	chr1:3581709-3581745	-	GGGCCATTGTGGGAGGAGACACCTGGGCCATTGTGGG<br>(((.....)))	TP73	NM_001204186	chr1:3558989-3642625	template	37	Intron_1.f	--	Yes	--	Yes	--	--	--	Yes	--	--	--
G4ST01003581708	chr1:3581709-3581745	-	GGGCCATTGTGGGAGGAGACACCTGGGCCATTGTGGG<br>(((.....)))	TP73	NM_001204187	chr1:3558989-3642625	template	37	Intron_1.f	--	Yes	--	Yes	--	--	--	Yes	--	--	--
G4ST01003581708	chr1:3581709-3581745	-	GGGCCATTGTGGGAGGAGACACCTGGGCCATTGTGGG<br>(((.....)))	TP73	NM_001204188	chr1:3558989-3642625	template	37	Intron_1.f	--	Yes	--	Yes	--	--	--	Yes	--	--	--
G4ST01003581718	chr1:3581719-3581759	-	GGGAGGAGACACCTGGGCCATTGTGGGAGGAGACACCTGGG<br>(((.....))) (((.....)))	TP73	NM_005427	chr1:3558989-3642625	template	41	Intron_1.f	--	Yes	--	Yes	--	--	--	Yes	--	--	--
G4ST01003581718	chr1:3581719-3581759	-	GGGAGGAGACACCTGGGCCATTGTGGGAGGAGACACCTGGG<br>(((.....))) (((.....)))	TP73	NM_001204184	chr1:3558989-3642625	template	41	Intron_1.f	--	Yes	--	Yes	--	--	--	Yes	--	--	--
G4ST01003581718	chr1:3581719-3581759	-	GGGAGGAGACACCTGGGCCATTGTGGGAGGAGACACCTGGG<br>(((.....))) (((.....)))	TP73	NM_001204185	chr1:3558989-3642625	template	41	Intron_1.f	--	Yes	--	Yes	--	--	--	Yes	--	--	--
G4ST01003581718	chr1:3581719-3581759	-	GGGAGGAGACACCTGGGCCATTGTGGGAGGAGACACCTGGG<br>(((.....))) (((.....)))	TP73	NM_001204186	chr1:3558989-3642625	template	41	Intron_1.f	--	Yes	--	Yes	--	--	--	Yes	--	--	--
G4ST01003581718	chr1:3581719-3581759	-	GGGAGGAGACACCTGGGCCATTGTGGGAGGAGACACCTGGG<br>(((.....))) (((.....)))	TP73	NM_001204187	chr1:3558989-3642625	template	41	Intron_1.f	--	Yes	--	Yes	--	--	--	Yes	--	--	--
G4ST01003581718	chr1:3581719-3581759	-	GGGAGGAGACACCTGGGCCATTGTGGGAGGAGACACCTGGG<br>(((.....))) (((.....)))	TP73	NM_001204188	chr1:3558989-3642625	template	41	Intron_1.f	--	Yes	--	Yes	--	--	--	Yes	--	--	--
G4ST01003581732	chr1:3581733-3581770	-	GGGGCCATTGTGGGAGGAGACACCTGGGCCATTGTGGG<br>(((.....)))	TP73	NM_005427	chr1:3558989-3642625	template	38	Intron_1.f	--	Yes	--	Yes	--	--	--	Yes	--	--	--
G4ST01003581732	chr1:3581733-3581770	-	GGGGCCATTGTGGGAGGAGACACCTGGGCCATTGTGGG<br>(((.....)))	TP73	NM_001204184	chr1:3558989-3642625	template	38	Intron_1.f	--	Yes	--	Yes	--	--	--	Yes	--	--	--
G4ST01003581732	chr1:3581733-3581770	-	GGGGCCATTGTGGGAGGAGACACCTGGGCCATTGTGGG<br>(((.....)))	TP73	NM_001204185	chr1:3558989-3642625	template	38	Intron_1.f	--	Yes	--	Yes	--	--	--	Yes	--	--	--
G4ST01003581732	chr1:3581733-3581770	-	GGGGCCATTGTGGGAGGAGACACCTGGGCCATTGTGGG<br>(((.....)))	TP73	NM_001204186	chr1:3558989-3642625	template	38	Intron_1.f	--	Yes	--	Yes	--	--	--	Yes	--	--	--
G4ST01003581732	chr1:3581733-3581770	-	GGGGCCATTGTGGGAGGAGACACCTGGGCCATTGTGGG<br>(((.....)))	TP73	NM_001204187	chr1:3558989-3642625	template	38	Intron_1.f	--	Yes	--	Yes	--	--	--	Yes	--	--	--

G4ST01003581732	chr1:3581733-3581770	-	GGGGCCATTGTGGGAGGAGACACCTGGGCCATTGTGGG<br>(((.....)))	TP73	NM_001204188	chr1:3558989-3642625	template	38	Intron_1.f	--	Yes	--	Yes	--	--	--	Yes	--	--	--
G4ST01003603092	chr1:3603093-3603123	+	GGGCAGGGCCCAACTGGGAATGGCTCATGGG<br>.(((...)))	TP73	NM_005427	chr1:3558989-3642625	non-template	31	Intron_3	--	Yes	--	Yes	--	--	--	--	--	--	--
G4ST01003603092	chr1:3603093-3603123	+	GGGCAGGGCCCAACTGGGAATGGCTCATGGG<br>.(((...)))	TP73	NM_001126242	chr1:3597096-3642625	non-template	31	Intron_1.f	--	Yes	--	Yes	--	--	--	--	--	--	--
G4ST01003603092	chr1:3603093-3603123	+	GGGCAGGGCCCAACTGGGAATGGCTCATGGG<br>.(((...)))	TP73	NM_001204192	chr1:3604470-3642625	non-template	-1346	upstream	Yes	--	--	--	--	--	--	--	--	--	--
G4ST01003603092	chr1:3603093-3603123	+	GGGCAGGGCCCAACTGGGAATGGCTCATGGG<br>.(((...)))	TP73	NM_001126241	chr1:3597096-3642625	non-template	31	Intron_1.f	--	Yes	--	Yes	--	--	--	--	--	--	--
G4ST01003603092	chr1:3603093-3603123	+	GGGCAGGGCCCAACTGGGAATGGCTCATGGG<br>.(((...)))	TP73	NM_001126240	chr1:3597096-3642625	non-template	31	Intron_1.f	--	Yes	--	Yes	--	--	--	--	--	--	--
G4ST01003603092	chr1:3603093-3603123	+	GGGCAGGGCCCAACTGGGAATGGCTCATGGG<br>.(((...)))	TP73	NM_001204184	chr1:3558989-3642625	non-template	31	Intron_3	--	Yes	--	Yes	--	--	--	--	--	--	--
G4ST01003603092	chr1:3603093-3603123	+	GGGCAGGGCCCAACTGGGAATGGCTCATGGG<br>.(((...)))	TP73	NM_001204185	chr1:3558989-3642625	non-template	31	Intron_3	--	Yes	--	Yes	--	--	--	--	--	--	--
G4ST01003603092	chr1:3603093-3603123	+	GGGCAGGGCCCAACTGGGAATGGCTCATGGG<br>.(((...)))	TP73	NM_001204186	chr1:3558989-3642625	non-template	31	Intron_3	--	Yes	--	Yes	--	--	--	--	--	--	--
G4ST01003603092	chr1:3603093-3603123	+	GGGCAGGGCCCAACTGGGAATGGCTCATGGG<br>.(((...)))	TP73	NM_001204187	chr1:3558989-3642625	non-template	31	Intron_3	--	Yes	--	Yes	--	--	--	--	--	--	--
G4ST01003603092	chr1:3603093-3603123	+	GGGCAGGGCCCAACTGGGAATGGCTCATGGG<br>.(((...)))	TP73	NM_001204188	chr1:3558989-3642625	non-template	31	Intron_3	--	Yes	--	Yes	--	--	--	--	--	--	--
G4ST01003603092	chr1:3603093-3603123	+	GGGCAGGGCCCAACTGGGAATGGCTCATGGG<br>.(((...)))	TP73	NM_001204189	chr1:3597096-3642625	non-template	31	Intron_1.f	--	Yes	--	Yes	--	--	--	--	--	--	--
G4ST01003603092	chr1:3603093-3603123	+	GGGCAGGGCCCAACTGGGAATGGCTCATGGG<br>.(((...)))	TP73	NM_001204190	chr1:3597096-3642625	non-template	31	Intron_1.f	--	Yes	--	Yes	--	--	--	--	--	--	--
G4ST01003603092	chr1:3603093-3603123	+	GGGCAGGGCCCAACTGGGAATGGCTCATGGG<br>.(((...)))	TP73	NM_001204191	chr1:3597096-3642625	non-template	31	Intron_1.f	--	Yes	--	Yes	--	--	--	--	--	--	--
G4ST01003604644	chr1:3604645-3604674	+	GGGTTGGGCTCTGGGGTGCTGGCAGCAGGG<br>..(((...))).	TP73	NM_005427	chr1:3558989-3642625	non-template	30	Intron_3	--	Yes	--	Yes	--	--	--	--	--	--	--
G4ST01003604644	chr1:3604645-3604674	+	GGGTTGGGCTCTGGGGTGCTGGCAGCAGGG<br>..(((...))).	TP73	NM_001126242	chr1:3597096-3642625	non-template	30	Intron_1.f	--	Yes	--	Yes	--	--	--	--	--	--	--
G4ST01003604644	chr1:3604645-3604674	+	GGGTTGGGCTCTGGGGTGCTGGCAGCAGGG<br>..(((...))).	TP73	NM_001204192	chr1:3604470-3642625	non-template	30	Intron_1.f	--	Yes	--	Yes	--	--	--	--	--	--	--
G4ST01003604644	chr1:3604645-3604674	+	GGGTTGGGCTCTGGGGTGCTGGCAGCAGGG<br>..(((...))).	TP73	NM_001126241	chr1:3597096-3642625	non-template	30	Intron_1.f	--	Yes	--	Yes	--	--	--	--	--	--	--
G4ST01003604644	chr1:3604645-3604674	+	GGGTTGGGCTCTGGGGTGCTGGCAGCAGGG<br>..(((...))).	TP73	NM_001126240	chr1:3597096-3642625	non-template	30	Intron_1.f	--	Yes	--	Yes	--	--	--	--	--	--	--
G4ST01003604644	chr1:3604645-3604674	+	GGGTTGGGCTCTGGGGTGCTGGCAGCAGGG<br>..(((...))).	TP73	NM_001204184	chr1:3558989-3642625	non-template	30	Intron_3	--	Yes	--	Yes	--	--	--	--	--	--	--
G4ST01003604644	chr1:3604645-3604674	+	GGGTTGGGCTCTGGGGTGCTGGCAGCAGGG<br>..(((...))).	TP73	NM_001204185	chr1:3558989-3642625	non-template	30	Intron_3	--	Yes	--	Yes	--	--	--	--	--	--	--
G4ST01003604644	chr1:3604645-3604674	+	GGGTTGGGCTCTGGGGTGCTGGCAGCAGGG<br>..(((...))).	TP73	NM_001204186	chr1:3558989-3642625	non-template	30	Intron_3	--	Yes	--	Yes	--	--	--	--	--	--	--
G4ST01003604644	chr1:3604645-3604674	+	GGGTTGGGCTCTGGGGTGCTGGCAGCAGGG<br>..(((...))).	TP73	NM_001204187	chr1:3558989-3642625	non-template	30	Intron_3	--	Yes	--	Yes	--	--	--	--	--	--	--
G4ST01003604644	chr1:3604645-3604674	+	GGGTTGGGCTCTGGGGTGCTGGCAGCAGGG<br>..(((...))).	TP73	NM_001204188	chr1:3558989-3642625	non-template	30	Intron_3	--	Yes	--	Yes	--	--	--	--	--	--	--
G4ST01003604644	chr1:3604645-3604674	+	GGGTTGGGCTCTGGGGTGCTGGCAGCAGGG<br>..(((...))).	TP73	NM_001204189	chr1:3597096-3642625	non-template	30	Intron_1.f	--	Yes	--	Yes	--	--	--	--	--	--	--
G4ST01003604644	chr1:3604645-3604674	+	GGGTTGGGCTCTGGGGTGCTGGCAGCAGGG<br>..(((...))).	TP73	NM_001204190	chr1:3597096-3642625	non-template	30	Intron_1.f	--	Yes	--	Yes	--	--	--	--	--	--	--
G4ST01003604644	chr1:3604645-3604674	+	GGGTTGGGCTCTGGGGTGCTGGCAGCAGGG<br>..(((...))).	TP73	NM_001204191	chr1:3597096-3642625	non-template	30	Intron_1.f	--	Yes	--	Yes	--	--	--	--	--	--	--
G4ST01003604962	chr1:3604963-3604991	+	GGGGAGGTGGGCTTTGAGCGGGATCCGGG<br>((...)).	TP73	NM_005427	chr1:3558989-3642625	non-template	29	Intron_3	--	Yes	--	Yes	--	--	--	--	--	--	--
G4ST01003604962	chr1:3604963-3604991	+	GGGGAGGTGGGCTTTGAGCGGGATCCGGG<br>((...)).	TP73	NM_001126242	chr1:3597096-3642625	non-template	29	Intron_1.f	--	Yes	--	Yes	--	--	--	--	--	--	--
G4ST01003604962	chr1:3604963-3604991	+	GGGGAGGTGGGCTTTGAGCGGGATCCGGG<br>((...)).	TP73	NM_001204192	chr1:3604470-3642625	non-template	29	Intron_1.f	--	Yes	--	Yes	--	--	--	--	--	--	--
G4ST01003604962	chr1:3604963-3604991	+	GGGGAGGTGGGCTTTGAGCGGGATCCGGG<br>((...)).	TP73	NM_001126241	chr1:3597096-3642625	non-template	29	Intron_1.f	--	Yes	--	Yes	--	--	--	--	--	--	--
G4ST01003604962	chr1:3604963-3604991	+	GGGGAGGTGGGCTTTGAGCGGGATCCGGG<br>((...)).	TP73	NM_001126240	chr1:3597096-3642625	non-template	29	Intron_1.f	--	Yes	--	Yes	--	--	--	--	--	--	--
G4ST01003604962	chr1:3604963-3604991	+	GGGGAGGTGGGCTTTGAGCGGGATCCGGG<br>((...)).	TP73	NM_001204184	chr1:3558989-3642625	non-template	29	Intron_3	--	Yes	--	Yes	--	--	--	--	--	--	--
G4ST01003604962	chr1:3604963-3604991	+	GGGGAGGTGGGCTTTGAGCGGGATCCGGG<br>((...)).	TP73	NM_001204185	chr1:3558989-3642625	non-template	29	Intron_3	--	Yes	--	Yes	--	--	--	--	--	--	--
G4ST01003604962	chr1:3604963-3604991	+	GGGGAGGTGGGCTTTGAGCGGGATCCGGG<br>((...)).	TP73	NM_001204186	chr1:3558989-3642625	non-template	29	Intron_3	--	Yes	--	Yes	--	--	--	--	--	--	--
G4ST01003604962	chr1:3604963-3604991	+	GGGGAGGTGGGCTTTGAGCGGGATCCGGG<br>((...)).	TP73	NM_001204187	chr1:3558989-3642625	non-template	29	Intron_3	--	Yes	--	Yes	--	--	--	--	--	--	--
G4ST01003604962	chr1:3604963-3604991	+	GGGGAGGTGGGCTTTGAGCGGGATCCGGG<br>((...)).	TP73	NM_001204188	chr1:3558989-3642625	non-template	29	Intron_3	--	Yes	--	Yes	--	--	--	--	--	--	--
G4ST01003604962	chr1:3604963-3604991	+	GGGGAGGTGGGCTTTGAGCGGGATCCGGG<br>((...)).	TP73	NM_001204189	chr1:3597096-3642625	non-template	29	Intron_1.f	--	Yes	--	Yes	--	--	--	--	--	--	--
G4ST01003604962	chr1:3604963-3604991	+	GGGGAGGTGGGCTTTGAGCGGGATCCGGG<br>((...)).	TP73	NM_001204190	chr1:3597096-3642625	non-template	29	Intron_1.f	--	Yes	--	Yes	--	--	--	--	--	--	--
G4ST01003604962	chr1:3604963-3604991	+	GGGGAGGTGGGCTTTGAGCGGGATCCGGG<br>((...)).	TP73	NM_001204191	chr1:3597096-3642625	non-template	29	Intron_1.f	--	Yes	--	Yes	--	--	--	--	--	--	--
G4ST01003604970	chr1:3604971-3605001	+	GGGCTTTGAGCGGGATCCGGGAGGCAGGGGG<br>((...)).	TP73	NM_005427	chr1:3558989-3642625	non-template	31	Intron_3	--	Yes	--	Yes	--	--	--	--	--	--	--
G4ST01003604970	chr1:3604971-3605001	+	GGGCTTTGAGCGGGATCCGGGAGGCAGGGGG<br>((...)).	TP73	NM_001126242	chr1:3597096-3642625	non-template	31	Intron_1.f	--	Yes	--	Yes	--	--	--	--	--	--	--
G4ST01003604970	chr1:3604971-3605001	+	GGGCTTTGAGCGGGATCCGGGAGGCAGGGGG<br>((...)).	TP73	NM_001204192	chr1:3604470-3642625	non-template	31	Intron_1.f	--	Yes	--	Yes	--	--	--	--	--	--	--
G4ST01003604970	chr1:3604971-3605001	+	GGGCTTTGAGCGGGATCCGGGAGGCAGGGGG<br>((...)).	TP73	NM_001126241	chr1:3597096-3642625	non-template	31	Intron_1.f	--	Yes	--	Yes	--	--	--	--	--	--	--
G4ST01003604970	chr1:3604971-3605001	+	GGGCTTTGAGCGGGATCCGGGAGGCAGGGGG<br>((...)).	TP73	NM_001126240	chr1:3597096-3642625	non-template	31	Intron_1.f	--	Yes	--	Yes	--	--	--	--	--	--	--
G4ST01003604970	chr1:3604971-3605001	+	GGGCTTTGAGCGGGATCCGGGAGGCAGGGGG<br>((...)).	TP73	NM_001204184	chr1:3558989-3642625	non-template	31	Intron_3	--	Yes	--	Yes	--	--	--	--	--	--	--
G4ST01003604970	chr1:3604971-3605001	+	GGGCTTTGAGCGGGATCCGGGAGGCAGGGGG<br>((...)).	TP73	NM_001204185	chr1:3558989-3642625	non-template	31	Intron_3	--	Yes	--	Yes	--	--	--	--	--	--	--
G4ST01003604970	chr1:3604971-3605001	+	GGGCTTTGAGCGGGATCCGGGAGGCAGGGGG<br>((...)).	TP73	NM_001204186	chr1:3558989-3642625	non-template	31	Intron_3	--	Yes	--	Yes	--	--	--	--	--	--	--
G4ST01003604970	chr1:3604971-3605001	+	GGGCTTTGAGCGGGATCCGGGAGGCAGGGGG<br>((...)).	TP73	NM_001204187	chr1:3558989-3642625	non-template	31	Intron_3	--	Yes	--	Yes	--	--	--	--	--	--	--
G4ST01003604970	chr1:3604971-3605001	+	GGGCTTTGAGCGGGATCCGGGAGGCAGGGGG<br>((...)).	TP73	NM_001204188	chr1:3558989-3642625	non-template	31	Intron_3	--	Yes	--	Yes	--	--	--	--	--	--	--

G4ST01003604970	chr1:3604971-3605001	+	GGGCTTTGAGCGGGATCCGGGAGGCAGGGGG<br>((...)).	TP73	NM_001204189	chr1:3597096-3642625	non-template	31	Intron_1.f	--	Yes	--	Yes	--	--	--	--	--	--	--
G4ST01003604970	chr1:3604971-3605001	+	GGGCTTTGAGCGGGATCCGGGAGGCAGGGGG<br>((...)).	TP73	NM_001204190	chr1:3597096-3642625	non-template	31	Intron_1.f	--	Yes	--	Yes	--	--	--	--	--	--	--
G4ST01003604970	chr1:3604971-3605001	+	GGGCTTTGAGCGGGATCCGGGAGGCAGGGGG<br>((...)).	TP73	NM_001204191	chr1:3597096-3642625	non-template	31	Intron_1.f	--	Yes	--	Yes	--	--	--	--	--	--	--
G4ST01003604981	chr1:3604982-3605014	+	GGGATCCGGGAGGCAGGGGGCAGAAGCTGAGGG<br>..(((...))).	TP73	NM_005427	chr1:3558989-3642625	non-template	33	Intron_3	--	Yes	--	Yes	--	--	--	--	--	--	--
G4ST01003604981	chr1:3604982-3605014	+	GGGATCCGGGAGGCAGGGGGCAGAAGCTGAGGG<br>..(((...))).	TP73	NM_001126242	chr1:3597096-3642625	non-template	33	Intron_1.f	--	Yes	--	Yes	--	--	--	--	--	--	--
G4ST01003604981	chr1:3604982-3605014	+	GGGATCCGGGAGGCAGGGGGCAGAAGCTGAGGG<br>..(((...))).	TP73	NM_001204192	chr1:3604470-3642625	non-template	33	Intron_1.f	--	Yes	--	Yes	--	--	--	--	--	--	--
G4ST01003604981	chr1:3604982-3605014	+	GGGATCCGGGAGGCAGGGGGCAGAAGCTGAGGG<br>..(((...))).	TP73	NM_001126241	chr1:3597096-3642625	non-template	33	Intron_1.f	--	Yes	--	Yes	--	--	--	--	--	--	--
G4ST01003604981	chr1:3604982-3605014	+	GGGATCCGGGAGGCAGGGGGCAGAAGCTGAGGG<br>..(((...))).	TP73	NM_001126240	chr1:3597096-3642625	non-template	33	Intron_1.f	--	Yes	--	Yes	--	--	--	--	--	--	--
G4ST01003604981	chr1:3604982-3605014	+	GGGATCCGGGAGGCAGGGGGCAGAAGCTGAGGG<br>..(((...))).	TP73	NM_001204184	chr1:3558989-3642625	non-template	33	Intron_3	--	Yes	--	Yes	--	--	--	--	--	--	--
G4ST01003604981	chr1:3604982-3605014	+	GGGATCCGGGAGGCAGGGGGCAGAAGCTGAGGG<br>..(((...))).	TP73	NM_001204185	chr1:3558989-3642625	non-template	33	Intron_3	--	Yes	--	Yes	--	--	--	--	--	--	--
G4ST01003604981	chr1:3604982-3605014	+	GGGATCCGGGAGGCAGGGGGCAGAAGCTGAGGG<br>..(((...))).	TP73	NM_001204186	chr1:3558989-3642625	non-template	33	Intron_3	--	Yes	--	Yes	--	--	--	--	--	--	--
G4ST01003604981	chr1:3604982-3605014	+	GGGATCCGGGAGGCAGGGGGCAGAAGCTGAGGG<br>..(((...))).	TP73	NM_001204187	chr1:3558989-3642625	non-template	33	Intron_3	--	Yes	--	Yes	--	--	--	--	--	--	--
G4ST01003604981	chr1:3604982-3605014	+	GGGATCCGGGAGGCAGGGGGCAGAAGCTGAGGG<br>..(((...))).	TP73	NM_001204188	chr1:3558989-3642625	non-template	33	Intron_3	--	Yes	--	Yes	--	--	--	--	--	--	--
G4ST01003604981	chr1:3604982-3605014	+	GGGATCCGGGAGGCAGGGGGCAGAAGCTGAGGG<br>..(((...))).	TP73	NM_001204189	chr1:3597096-3642625	non-template	33	Intron_1.f	--	Yes	--	Yes	--	--	--	--	--	--	--
G4ST01003604981	chr1:3604982-3605014	+	GGGATCCGGGAGGCAGGGGGCAGAAGCTGAGGG<br>..(((...))).	TP73	NM_001204190	chr1:3597096-3642625	non-template	33	Intron_1.f	--	Yes	--	Yes	--	--	--	--	--	--	--
G4ST01003604981	chr1:3604982-3605014	+	GGGATCCGGGAGGCAGGGGGCAGAAGCTGAGGG<br>..(((...))).	TP73	NM_001204191	chr1:3597096-3642625	non-template	33	Intron_1.f	--	Yes	--	Yes	--	--	--	--	--	--	--
G4ST01003607632	chr1:3607633-3607673	-	GGGCACAGGGACGGATGGGGAAGAGCAGGTGCTCTCGGGGG<br>..(((((....)))))...	TP73	NM_005427	chr1:3558989-3642625	template	41	Intron_3	--	Yes	--	Yes	--	--	--	--	--	--	--
G4ST01003607632	chr1:3607633-3607673	-	GGGCACAGGGACGGATGGGGAAGAGCAGGTGCTCTCGGGGG<br>..(((((....)))))...	TP73	NM_001126242	chr1:3597096-3642625	template	41	Intron_1.f	--	Yes	--	Yes	--	--	--	--	--	--	--
G4ST01003607632	chr1:3607633-3607673	-	GGGCACAGGGACGGATGGGGAAGAGCAGGTGCTCTCGGGGG<br>..(((((....)))))...	TP73	NM_001204192	chr1:3604470-3642625	template	41	Intron_1.f	--	Yes	--	Yes	--	--	--	--	--	--	--
G4ST01003607632	chr1:3607633-3607673	-	GGGCACAGGGACGGATGGGGAAGAGCAGGTGCTCTCGGGGG<br>..(((((....)))))...	TP73	NM_001126241	chr1:3597096-3642625	template	41	Intron_1.f	--	Yes	--	Yes	--	--	--	--	--	--	--
G4ST01003607632	chr1:3607633-3607673	-	GGGCACAGGGACGGATGGGGAAGAGCAGGTGCTCTCGGGGG<br>..(((((....)))))...	TP73	NM_001126240	chr1:3597096-3642625	template	41	Intron_1.f	--	Yes	--	Yes	--	--	--	--	--	--	--
G4ST01003607632	chr1:3607633-3607673	-	GGGCACAGGGACGGATGGGGAAGAGCAGGTGCTCTCGGGGG<br>..(((((....)))))...	TP73	NM_001204184	chr1:3558989-3642625	template	41	Intron_3	--	Yes	--	Yes	--	--	--	--	--	--	--
G4ST01003607632	chr1:3607633-3607673	-	GGGCACAGGGACGGATGGGGAAGAGCAGGTGCTCTCGGGGG<br>..(((((....)))))...	TP73	NM_001204185	chr1:3558989-3642625	template	41	Intron_3	--	Yes	--	Yes	--	--	--	--	--	--	--
G4ST01003607632	chr1:3607633-3607673	-	GGGCACAGGGACGGATGGGGAAGAGCAGGTGCTCTCGGGGG<br>..(((((....)))))...	TP73	NM_001204186	chr1:3558989-3642625	template	41	Intron_3	--	Yes	--	Yes	--	--	--	--	--	--	--
G4ST01003607632	chr1:3607633-3607673	-	GGGCACAGGGACGGATGGGGAAGAGCAGGTGCTCTCGGGGG<br>..(((((....)))))...	TP73	NM_001204187	chr1:3558989-3642625	template	41	Intron_3	--	Yes	--	Yes	--	--	--	--	--	--	--
G4ST01003607632	chr1:3607633-3607673	-	GGGCACAGGGACGGATGGGGAAGAGCAGGTGCTCTCGGGGG<br>..(((((....)))))...	TP73	NM_001204188	chr1:3558989-3642625	template	41	Intron_3	--	Yes	--	Yes	--	--	--	--	--	--	--
G4ST01003607632	chr1:3607633-3607673	-	GGGCACAGGGACGGATGGGGAAGAGCAGGTGCTCTCGGGGG<br>..(((((....)))))...	TP73	NM_001204189	chr1:3597096-3642625	template	41	Intron_1.f	--	Yes	--	Yes	--	--	--	--	--	--	--
G4ST01003607632	chr1:3607633-3607673	-	GGGCACAGGGACGGATGGGGAAGAGCAGGTGCTCTCGGGGG<br>..(((((....)))))...	TP73	NM_001204190	chr1:3597096-3642625	template	41	Intron_1.f	--	Yes	--	Yes	--	--	--	--	--	--	--
G4ST01003607632	chr1:3607633-3607673	-	GGGCACAGGGACGGATGGGGAAGAGCAGGTGCTCTCGGGGG<br>..(((((....)))))...	TP73	NM_001204191	chr1:3597096-3642625	template	41	Intron_1.f	--	Yes	--	Yes	--	--	--	--	--	--	--
G4ST01003608972	chr1:3608973-3609011	+	GGGAGGAGAGATGAGGCCTCCTGGGTGGGTGCGTGGGGG<br>(((((..........)))))	TP73	NM_005427	chr1:3558989-3642625	non-template	39	Intron_3	--	Yes	--	Yes	--	--	--	--	--	--	--
G4ST01003608972	chr1:3608973-3609011	+	GGGAGGAGAGATGAGGCCTCCTGGGTGGGTGCGTGGGGG<br>(((((..........)))))	TP73	NM_001126242	chr1:3597096-3642625	non-template	39	Intron_1.f	--	Yes	--	Yes	--	--	--	--	--	--	--
G4ST01003608972	chr1:3608973-3609011	+	GGGAGGAGAGATGAGGCCTCCTGGGTGGGTGCGTGGGGG<br>(((((..........)))))	TP73	NM_001204192	chr1:3604470-3642625	non-template	39	Intron_1.f	--	Yes	--	Yes	--	--	--	--	--	--	--
G4ST01003608972	chr1:3608973-3609011	+	GGGAGGAGAGATGAGGCCTCCTGGGTGGGTGCGTGGGGG<br>(((((..........)))))	TP73	NM_001126241	chr1:3597096-3642625	non-template	39	Intron_1.f	--	Yes	--	Yes	--	--	--	--	--	--	--
G4ST01003608972	chr1:3608973-3609011	+	GGGAGGAGAGATGAGGCCTCCTGGGTGGGTGCGTGGGGG<br>(((((..........)))))	TP73	NM_001126240	chr1:3597096-3642625	non-template	39	Intron_1.f	--	Yes	--	Yes	--	--	--	--	--	--	--
G4ST01003608972	chr1:3608973-3609011	+	GGGAGGAGAGATGAGGCCTCCTGGGTGGGTGCGTGGGGG<br>(((((..........)))))	TP73	NM_001204184	chr1:3558989-3642625	non-template	39	Intron_3	--	Yes	--	Yes	--	--	--	--	--	--	--
G4ST01003608972	chr1:3608973-3609011	+	GGGAGGAGAGATGAGGCCTCCTGGGTGGGTGCGTGGGGG<br>(((((..........)))))	TP73	NM_001204185	chr1:3558989-3642625	non-template	39	Intron_3	--	Yes	--	Yes	--	--	--	--	--	--	--
G4ST01003608972	chr1:3608973-3609011	+	GGGAGGAGAGATGAGGCCTCCTGGGTGGGTGCGTGGGGG<br>(((((..........)))))	TP73	NM_001204186	chr1:3558989-3642625	non-template	39	Intron_3	--	Yes	--	Yes	--	--	--	--	--	--	--
G4ST01003608972	chr1:3608973-3609011	+	GGGAGGAGAGATGAGGCCTCCTGGGTGGGTGCGTGGGGG<br>(((((..........)))))	TP73	NM_001204187	chr1:3558989-3642625	non-template	39	Intron_3	--	Yes	--	Yes	--	--	--	--	--	--	--
G4ST01003608972	chr1:3608973-3609011	+	GGGAGGAGAGATGAGGCCTCCTGGGTGGGTGCGTGGGGG<br>(((((..........)))))	TP73	NM_001204188	chr1:3558989-3642625	non-template	39	Intron_3	--	Yes	--	Yes	--	--	--	--	--	--	--
G4ST01003608972	chr1:3608973-3609011	+	GGGAGGAGAGATGAGGCCTCCTGGGTGGGTGCGTGGGGG<br>(((((..........)))))	TP73	NM_001204189	chr1:3597096-3642625	non-template	39	Intron_1.f	--	Yes	--	Yes	--	--	--	--	--	--	--
G4ST01003608972	chr1:3608973-3609011	+	GGGAGGAGAGATGAGGCCTCCTGGGTGGGTGCGTGGGGG<br>(((((..........)))))	TP73	NM_001204190	chr1:3597096-3642625	non-template	39	Intron_1.f	--	Yes	--	Yes	--	--	--	--	--	--	--
G4ST01003608972	chr1:3608973-3609011	+	GGGAGGAGAGATGAGGCCTCCTGGGTGGGTGCGTGGGGG<br>(((((..........)))))	TP73	NM_001204191	chr1:3597096-3642625	non-template	39	Intron_1.f	--	Yes	--	Yes	--	--	--	--	--	--	--
G4ST01003610150	chr1:3610151-3610176	+	GGGATGGAGCAGGGATGGGAGTCGGG<br>.((...))	TP73	NM_005427	chr1:3558989-3642625	non-template	26	Intron_3	--	Yes	--	Yes	--	--	--	--	Yes	Yes	Yes
G4ST01003610150	chr1:3610151-3610176	+	GGGATGGAGCAGGGATGGGAGTCGGG<br>.((...))	TP73	NM_001126242	chr1:3597096-3642625	non-template	26	Intron_1.f	--	Yes	--	Yes	--	--	--	--	Yes	Yes	Yes
G4ST01003610150	chr1:3610151-3610176	+	GGGATGGAGCAGGGATGGGAGTCGGG<br>.((...))	TP73	NM_001204192	chr1:3604470-3642625	non-template	26	Intron_1.f	--	Yes	--	Yes	--	--	--	--	Yes	Yes	Yes
G4ST01003610150	chr1:3610151-3610176	+	GGGATGGAGCAGGGATGGGAGTCGGG<br>.((...))	TP73	NM_001126241	chr1:3597096-3642625	non-template	26	Intron_1.f	--	Yes	--	Yes	--	--	--	--	Yes	Yes	Yes
G4ST01003610150	chr1:3610151-3610176	+	GGGATGGAGCAGGGATGGGAGTCGGG<br>.((...))	TP73	NM_001126240	chr1:3597096-3642625	non-template	26	Intron_1.f	--	Yes	--	Yes	--	--	--	--	Yes	Yes	Yes
G4ST01003610150	chr1:3610151-3610176	+	GGGATGGAGCAGGGATGGGAGTCGGG<br>.((...))	TP73	NM_001204184	chr1:3558989-3642625	non-template	26	Intron_3	--	Yes	--	Yes	--	--	--	--	Yes	Yes	Yes
G4ST01003610150	chr1:3610151-3610176	+	GGGATGGAGCAGGGATGGGAGTCGGG<br>.((...))	TP73	NM_001204185	chr1:3558989-3642625	non-template	26	Intron_3	--	Yes	--	Yes	--	--	--	--	Yes	Yes	Yes
G4ST01003610150	chr1:3610151-3610176	+	GGGATGGAGCAGGGATGGGAGTCGGG<br>.((...))	TP73	NM_001204186	chr1:3558989-3642625	non-template	26	Intron_3	--	Yes	--	Yes	--	--	--	--	Yes	Yes	Yes

G4ST01003610150	chr1:3610151-3610176	+	GGGATGGAGCAGGGATGGGAGTCGGG<br>.((...))	TP73	NM_001204187	chr1:3558989-3642625	non-template	26	Intron_3	--	Yes	--	Yes	--	--	--	--	Yes	Yes	Yes
G4ST01003610150	chr1:3610151-3610176	+	GGGATGGAGCAGGGATGGGAGTCGGG<br>.((...))	TP73	NM_001204188	chr1:3558989-3642625	non-template	26	Intron_3	--	Yes	--	Yes	--	--	--	--	Yes	Yes	Yes
G4ST01003610150	chr1:3610151-3610176	+	GGGATGGAGCAGGGATGGGAGTCGGG<br>.((...))	TP73	NM_001204189	chr1:3597096-3642625	non-template	26	Intron_1.f	--	Yes	--	Yes	--	--	--	--	Yes	Yes	Yes
G4ST01003610150	chr1:3610151-3610176	+	GGGATGGAGCAGGGATGGGAGTCGGG<br>.((...))	TP73	NM_001204190	chr1:3597096-3642625	non-template	26	Intron_1.f	--	Yes	--	Yes	--	--	--	--	Yes	Yes	Yes
G4ST01003610150	chr1:3610151-3610176	+	GGGATGGAGCAGGGATGGGAGTCGGG<br>.((...))	TP73	NM_001204191	chr1:3597096-3642625	non-template	26	Intron_1.f	--	Yes	--	Yes	--	--	--	--	Yes	Yes	Yes
G4ST01003611142	chr1:3611143-3611181	+	GGGCCTCGGTGAGGAGTCTGGGAGCCGGGGACTCTGGGG<br>((((...)))).....	TP73	NM_005427	chr1:3558989-3642625	non-template	39	Intron_3	--	Yes	--	Yes	--	--	--	--	--	Yes	Yes
G4ST01003611142	chr1:3611143-3611181	+	GGGCCTCGGTGAGGAGTCTGGGAGCCGGGGACTCTGGGG<br>((((...)))).....	TP73	NM_001126242	chr1:3597096-3642625	non-template	39	Intron_1.f	--	Yes	--	Yes	--	--	--	--	--	Yes	Yes
G4ST01003611142	chr1:3611143-3611181	+	GGGCCTCGGTGAGGAGTCTGGGAGCCGGGGACTCTGGGG<br>((((...)))).....	TP73	NM_001204192	chr1:3604470-3642625	non-template	39	Intron_1.f	--	Yes	--	Yes	--	--	--	--	--	Yes	Yes
G4ST01003611142	chr1:3611143-3611181	+	GGGCCTCGGTGAGGAGTCTGGGAGCCGGGGACTCTGGGG<br>((((...)))).....	TP73	NM_001126241	chr1:3597096-3642625	non-template	39	Intron_1.f	--	Yes	--	Yes	--	--	--	--	--	Yes	Yes
G4ST01003611142	chr1:3611143-3611181	+	GGGCCTCGGTGAGGAGTCTGGGAGCCGGGGACTCTGGGG<br>((((...)))).....	TP73	NM_001126240	chr1:3597096-3642625	non-template	39	Intron_1.f	--	Yes	--	Yes	--	--	--	--	--	Yes	Yes
G4ST01003611142	chr1:3611143-3611181	+	GGGCCTCGGTGAGGAGTCTGGGAGCCGGGGACTCTGGGG<br>((((...)))).....	TP73	NM_001204184	chr1:3558989-3642625	non-template	39	Intron_3	--	Yes	--	Yes	--	--	--	--	--	Yes	Yes
G4ST01003611142	chr1:3611143-3611181	+	GGGCCTCGGTGAGGAGTCTGGGAGCCGGGGACTCTGGGG<br>((((...)))).....	TP73	NM_001204185	chr1:3558989-3642625	non-template	39	Intron_3	--	Yes	--	Yes	--	--	--	--	--	Yes	Yes
G4ST01003611142	chr1:3611143-3611181	+	GGGCCTCGGTGAGGAGTCTGGGAGCCGGGGACTCTGGGG<br>((((...)))).....	TP73	NM_001204186	chr1:3558989-3642625	non-template	39	Intron_3	--	Yes	--	Yes	--	--	--	--	--	Yes	Yes
G4ST01003611142	chr1:3611143-3611181	+	GGGCCTCGGTGAGGAGTCTGGGAGCCGGGGACTCTGGGG<br>((((...)))).....	TP73	NM_001204187	chr1:3558989-3642625	non-template	39	Intron_3	--	Yes	--	Yes	--	--	--	--	--	Yes	Yes
G4ST01003611142	chr1:3611143-3611181	+	GGGCCTCGGTGAGGAGTCTGGGAGCCGGGGACTCTGGGG<br>((((...)))).....	TP73	NM_001204188	chr1:3558989-3642625	non-template	39	Intron_3	--	Yes	--	Yes	--	--	--	--	--	Yes	Yes
G4ST01003611142	chr1:3611143-3611181	+	GGGCCTCGGTGAGGAGTCTGGGAGCCGGGGACTCTGGGG<br>((((...)))).....	TP73	NM_001204189	chr1:3597096-3642625	non-template	39	Intron_1.f	--	Yes	--	Yes	--	--	--	--	--	Yes	Yes
G4ST01003611142	chr1:3611143-3611181	+	GGGCCTCGGTGAGGAGTCTGGGAGCCGGGGACTCTGGGG<br>((((...)))).....	TP73	NM_001204190	chr1:3597096-3642625	non-template	39	Intron_1.f	--	Yes	--	Yes	--	--	--	--	--	Yes	Yes
G4ST01003611142	chr1:3611143-3611181	+	GGGCCTCGGTGAGGAGTCTGGGAGCCGGGGACTCTGGGG<br>((((...)))).....	TP73	NM_001204191	chr1:3597096-3642625	non-template	39	Intron_1.f	--	Yes	--	Yes	--	--	--	--	--	Yes	Yes
G4ST01003614034	chr1:3614035-3614072	-	GGGTGGGCACGCTGGCGGCGTGCTCTGGGGTGTAGGGG<br>(((((.....))))).....	TP73	NM_005427	chr1:3558989-3642625	template	38	Exon_4	--	Yes	Yes	--	Yes	--	--	Yes	Yes	--	--
G4ST01003614034	chr1:3614035-3614072	-	GGGTGGGCACGCTGGCGGCGTGCTCTGGGGTGTAGGGG<br>(((((.....))))).....	TP73	NM_001126242	chr1:3597096-3642625	template	38	Exon_2	--	Yes	Yes	--	Yes	--	--	Yes	Yes	--	--
G4ST01003614034	chr1:3614035-3614072	-	GGGTGGGCACGCTGGCGGCGTGCTCTGGGGTGTAGGGG<br>(((((.....))))).....	TP73	NM_001204192	chr1:3604470-3642625	template	38	Exon_2	--	Yes	Yes	--	Yes	--	--	Yes	Yes	--	--
G4ST01003614034	chr1:3614035-3614072	-	GGGTGGGCACGCTGGCGGCGTGCTCTGGGGTGTAGGGG<br>(((((.....))))).....	TP73	NM_001126241	chr1:3597096-3642625	template	38	Exon_2	--	Yes	Yes	--	Yes	--	--	Yes	Yes	--	--
G4ST01003614034	chr1:3614035-3614072	-	GGGTGGGCACGCTGGCGGCGTGCTCTGGGGTGTAGGGG<br>(((((.....))))).....	TP73	NM_001126240	chr1:3597096-3642625	template	38	Exon_2	--	Yes	Yes	--	Yes	--	--	Yes	Yes	--	--
G4ST01003614034	chr1:3614035-3614072	-	GGGTGGGCACGCTGGCGGCGTGCTCTGGGGTGTAGGGG<br>(((((.....))))).....	TP73	NM_001204184	chr1:3558989-3642625	template	38	Exon_4	--	Yes	Yes	--	Yes	--	--	Yes	Yes	--	--
G4ST01003614034	chr1:3614035-3614072	-	GGGTGGGCACGCTGGCGGCGTGCTCTGGGGTGTAGGGG<br>(((((.....))))).....	TP73	NM_001204185	chr1:3558989-3642625	template	38	Exon_4	--	Yes	Yes	--	Yes	--	--	Yes	Yes	--	--
G4ST01003614034	chr1:3614035-3614072	-	GGGTGGGCACGCTGGCGGCGTGCTCTGGGGTGTAGGGG<br>(((((.....))))).....	TP73	NM_001204186	chr1:3558989-3642625	template	38	Exon_4	--	Yes	Yes	--	Yes	--	--	Yes	Yes	--	--
G4ST01003614034	chr1:3614035-3614072	-	GGGTGGGCACGCTGGCGGCGTGCTCTGGGGTGTAGGGG<br>(((((.....))))).....	TP73	NM_001204187	chr1:3558989-3642625	template	38	Exon_4	--	Yes	Yes	--	Yes	--	--	Yes	Yes	--	--
G4ST01003614034	chr1:3614035-3614072	-	GGGTGGGCACGCTGGCGGCGTGCTCTGGGGTGTAGGGG<br>(((((.....))))).....	TP73	NM_001204188	chr1:3558989-3642625	template	38	Exon_4	--	Yes	Yes	--	Yes	--	--	Yes	Yes	--	--
G4ST01003614034	chr1:3614035-3614072	-	GGGTGGGCACGCTGGCGGCGTGCTCTGGGGTGTAGGGG<br>(((((.....))))).....	TP73	NM_001204189	chr1:3597096-3642625	template	38	Exon_2	--	Yes	Yes	--	Yes	--	--	Yes	Yes	--	--
G4ST01003614034	chr1:3614035-3614072	-	GGGTGGGCACGCTGGCGGCGTGCTCTGGGGTGTAGGGG<br>(((((.....))))).....	TP73	NM_001204190	chr1:3597096-3642625	template	38	Exon_2	--	Yes	Yes	--	Yes	--	--	Yes	Yes	--	--
G4ST01003614034	chr1:3614035-3614072	-	GGGTGGGCACGCTGGCGGCGTGCTCTGGGGTGTAGGGG<br>(((((.....))))).....	TP73	NM_001204191	chr1:3597096-3642625	template	38	Exon_2	--	Yes	Yes	--	Yes	--	--	Yes	Yes	--	--
G4ST01003614042	chr1:3614043-3614080	-	GGGCGAGTGGGTGGGCACGCTGGCGGCGTGCTCTGGGG<br>(((((.....))))).....	TP73	NM_005427	chr1:3558989-3642625	template	38	Exon_4	--	Yes	Yes	--	Yes	--	--	Yes	Yes	--	--
G4ST01003614042	chr1:3614043-3614080	-	GGGCGAGTGGGTGGGCACGCTGGCGGCGTGCTCTGGGG<br>(((((.....))))).....	TP73	NM_001126242	chr1:3597096-3642625	template	38	Exon_2	--	Yes	Yes	--	Yes	--	--	Yes	Yes	--	--
G4ST01003614042	chr1:3614043-3614080	-	GGGCGAGTGGGTGGGCACGCTGGCGGCGTGCTCTGGGG<br>(((((.....))))).....	TP73	NM_001204192	chr1:3604470-3642625	template	38	Exon_2	--	Yes	Yes	--	Yes	--	--	Yes	Yes	--	--
G4ST01003614042	chr1:3614043-3614080	-	GGGCGAGTGGGTGGGCACGCTGGCGGCGTGCTCTGGGG<br>(((((.....))))).....	TP73	NM_001126241	chr1:3597096-3642625	template	38	Exon_2	--	Yes	Yes	--	Yes	--	--	Yes	Yes	--	--
G4ST01003614042	chr1:3614043-3614080	-	GGGCGAGTGGGTGGGCACGCTGGCGGCGTGCTCTGGGG<br>(((((.....))))).....	TP73	NM_001126240	chr1:3597096-3642625	template	38	Exon_2	--	Yes	Yes	--	Yes	--	--	Yes	Yes	--	--
G4ST01003614042	chr1:3614043-3614080	-	GGGCGAGTGGGTGGGCACGCTGGCGGCGTGCTCTGGGG<br>(((((.....))))).....	TP73	NM_001204184	chr1:3558989-3642625	template	38	Exon_4	--	Yes	Yes	--	Yes	--	--	Yes	Yes	--	--
G4ST01003614042	chr1:3614043-3614080	-	GGGCGAGTGGGTGGGCACGCTGGCGGCGTGCTCTGGGG<br>(((((.....))))).....	TP73	NM_001204185	chr1:3558989-3642625	template	38	Exon_4	--	Yes	Yes	--	Yes	--	--	Yes	Yes	--	--
G4ST01003614042	chr1:3614043-3614080	-	GGGCGAGTGGGTGGGCACGCTGGCGGCGTGCTCTGGGG<br>(((((.....))))).....	TP73	NM_001204186	chr1:3558989-3642625	template	38	Exon_4	--	Yes	Yes	--	Yes	--	--	Yes	Yes	--	--
G4ST01003614042	chr1:3614043-3614080	-	GGGCGAGTGGGTGGGCACGCTGGCGGCGTGCTCTGGGG<br>(((((.....))))).....	TP73	NM_001204187	chr1:3558989-3642625	template	38	Exon_4	--	Yes	Yes	--	Yes	--	--	Yes	Yes	--	--
G4ST01003614042	chr1:3614043-3614080	-	GGGCGAGTGGGTGGGCACGCTGGCGGCGTGCTCTGGGG<br>(((((.....))))).....	TP73	NM_001204188	chr1:3558989-3642625	template	38	Exon_4	--	Yes	Yes	--	Yes	--	--	Yes	Yes	--	--
G4ST01003614042	chr1:3614043-3614080	-	GGGCGAGTGGGTGGGCACGCTGGCGGCGTGCTCTGGGG<br>(((((.....))))).....	TP73	NM_001204189	chr1:3597096-3642625	template	38	Exon_2	--	Yes	Yes	--	Yes	--	--	Yes	Yes	--	--
G4ST01003614042	chr1:3614043-3614080	-	GGGCGAGTGGGTGGGCACGCTGGCGGCGTGCTCTGGGG<br>(((((.....))))).....	TP73	NM_001204190	chr1:3597096-3642625	template	38	Exon_2	--	Yes	Yes	--	Yes	--	--	Yes	Yes	--	--
G4ST01003614042	chr1:3614043-3614080	-	GGGCGAGTGGGTGGGCACGCTGGCGGCGTGCTCTGGGG<br>(((((.....))))).....	TP73	NM_001204191	chr1:3597096-3642625	template	38	Exon_2	--	Yes	Yes	--	Yes	--	--	Yes	Yes	--	--
G4ST01003615291	chr1:3615292-3615338	-	GGGGGCCAGCACTGAGCTGTGCCCGGGGAGGCGGGGCCTCGTGGGGG<br>(((((((.....)))).)))..	TP73	NM_005427	chr1:3558989-3642625	template	47	Intron_4	--	Yes	--	Yes	--	--	--	--	--	--	Yes
G4ST01003615291	chr1:3615292-3615338	-	GGGGGCCAGCACTGAGCTGTGCCCGGGGAGGCGGGGCCTCGTGGGGG<br>(((((((.....)))).)))..	TP73	NM_001126242	chr1:3597096-3642625	template	47	Intron_2	--	Yes	--	Yes	--	--	--	--	--	--	Yes
G4ST01003615291	chr1:3615292-3615338	-	GGGGGCCAGCACTGAGCTGTGCCCGGGGAGGCGGGGCCTCGTGGGGG<br>(((((((.....)))).)))..	TP73	NM_001204192	chr1:3604470-3642625	template	47	Intron_2	--	Yes	--	Yes	--	--	--	--	--	--	Yes
G4ST01003615291	chr1:3615292-3615338	-	GGGGGCCAGCACTGAGCTGTGCCCGGGGAGGCGGGGCCTCGTGGGGG<br>(((((((.....)))).)))..	TP73	NM_001126241	chr1:3597096-3642625	template	47	Intron_2	--	Yes	--	Yes	--	--	--	--	--	--	Yes
G4ST01003615291	chr1:3615292-3615338	-	GGGGGCCAGCACTGAGCTGTGCCCGGGGAGGCGGGGCCTCGTGGGGG<br>(((((((.....)))).)))..	TP73	NM_001126240	chr1:3597096-3642625	template	47	Intron_2	--	Yes	--	Yes	--	--	--	--	--	--	Yes
G4ST01003615291	chr1:3615292-3615338	-	GGGGGCCAGCACTGAGCTGTGCCCGGGGAGGCGGGGCCTCGTGGGGG<br>(((((((.....)))).)))..	TP73	NM_001204184	chr1:3558989-3642625	template	47	Intron_4	--	Yes	--	Yes	--	--	--	--	--	--	Yes

G4ST01003615291	chr1:3615292-3615338	-	GGGGGCCAGCACTGAGCTGTGCCCGGGGAGGCGGGGCCTCGTGGGGG<br>(((((((.....)))).)))..	TP73	NM_001204185	chr1:3558989-3642625	template	47	Intron_4	--	Yes	--	Yes	--	--	--	--	--	--	Yes
G4ST01003615291	chr1:3615292-3615338	-	GGGGGCCAGCACTGAGCTGTGCCCGGGGAGGCGGGGCCTCGTGGGGG<br>(((((((.....)))).)))..	TP73	NM_001204186	chr1:3558989-3642625	template	47	Intron_4	--	Yes	--	Yes	--	--	--	--	--	--	Yes
G4ST01003615291	chr1:3615292-3615338	-	GGGGGCCAGCACTGAGCTGTGCCCGGGGAGGCGGGGCCTCGTGGGGG<br>(((((((.....)))).)))..	TP73	NM_001204187	chr1:3558989-3642625	template	47	Intron_4	--	Yes	--	Yes	--	--	--	--	--	--	Yes
G4ST01003615291	chr1:3615292-3615338	-	GGGGGCCAGCACTGAGCTGTGCCCGGGGAGGCGGGGCCTCGTGGGGG<br>(((((((.....)))).)))..	TP73	NM_001204188	chr1:3558989-3642625	template	47	Intron_4	--	Yes	--	Yes	--	--	--	--	--	--	Yes
G4ST01003615291	chr1:3615292-3615338	-	GGGGGCCAGCACTGAGCTGTGCCCGGGGAGGCGGGGCCTCGTGGGGG<br>(((((((.....)))).)))..	TP73	NM_001204189	chr1:3597096-3642625	template	47	Intron_2	--	Yes	--	Yes	--	--	--	--	--	--	Yes
G4ST01003615291	chr1:3615292-3615338	-	GGGGGCCAGCACTGAGCTGTGCCCGGGGAGGCGGGGCCTCGTGGGGG<br>(((((((.....)))).)))..	TP73	NM_001204190	chr1:3597096-3642625	template	47	Intron_2	--	Yes	--	Yes	--	--	--	--	--	--	Yes
G4ST01003615291	chr1:3615292-3615338	-	GGGGGCCAGCACTGAGCTGTGCCCGGGGAGGCGGGGCCTCGTGGGGG<br>(((((((.....)))).)))..	TP73	NM_001204191	chr1:3597096-3642625	template	47	Intron_2	--	Yes	--	Yes	--	--	--	--	--	--	Yes
G4ST01003630442	chr1:3630443-3630471	+	GGGCACCACGTGGGTGGGGGGTGCAGGGG<br>.((...))	TP73	NM_005427	chr1:3558989-3642625	non-template	29	Intron_6	--	Yes	--	Yes	--	--	--	--	--	Yes	Yes
G4ST01003630442	chr1:3630443-3630471	+	GGGCACCACGTGGGTGGGGGGTGCAGGGG<br>.((...))	TP73	NM_001126242	chr1:3597096-3642625	non-template	29	Intron_4	--	Yes	--	Yes	--	--	--	--	--	Yes	Yes
G4ST01003630442	chr1:3630443-3630471	+	GGGCACCACGTGGGTGGGGGGTGCAGGGG<br>.((...))	TP73	NM_001204192	chr1:3604470-3642625	non-template	29	Intron_4	--	Yes	--	Yes	--	--	--	--	--	Yes	Yes
G4ST01003630442	chr1:3630443-3630471	+	GGGCACCACGTGGGTGGGGGGTGCAGGGG<br>.((...))	TP73	NM_001126241	chr1:3597096-3642625	non-template	29	Intron_4	--	Yes	--	Yes	--	--	--	--	--	Yes	Yes
G4ST01003630442	chr1:3630443-3630471	+	GGGCACCACGTGGGTGGGGGGTGCAGGGG<br>.((...))	TP73	NM_001126240	chr1:3597096-3642625	non-template	29	Intron_4	--	Yes	--	Yes	--	--	--	--	--	Yes	Yes
G4ST01003630442	chr1:3630443-3630471	+	GGGCACCACGTGGGTGGGGGGTGCAGGGG<br>.((...))	TP73	NM_001204184	chr1:3558989-3642625	non-template	29	Intron_6	--	Yes	--	Yes	--	--	--	--	--	Yes	Yes
G4ST01003630442	chr1:3630443-3630471	+	GGGCACCACGTGGGTGGGGGGTGCAGGGG<br>.((...))	TP73	NM_001204185	chr1:3558989-3642625	non-template	29	Intron_6	--	Yes	--	Yes	--	--	--	--	--	Yes	Yes
G4ST01003630442	chr1:3630443-3630471	+	GGGCACCACGTGGGTGGGGGGTGCAGGGG<br>.((...))	TP73	NM_001204186	chr1:3558989-3642625	non-template	29	Intron_6	--	Yes	--	Yes	--	--	--	--	--	Yes	Yes
G4ST01003630442	chr1:3630443-3630471	+	GGGCACCACGTGGGTGGGGGGTGCAGGGG<br>.((...))	TP73	NM_001204187	chr1:3558989-3642625	non-template	29	Intron_6	--	Yes	--	Yes	--	--	--	--	--	Yes	Yes
G4ST01003630442	chr1:3630443-3630471	+	GGGCACCACGTGGGTGGGGGGTGCAGGGG<br>.((...))	TP73	NM_001204188	chr1:3558989-3642625	non-template	29	Intron_6	--	Yes	--	Yes	--	--	--	--	--	Yes	Yes
G4ST01003630442	chr1:3630443-3630471	+	GGGCACCACGTGGGTGGGGGGTGCAGGGG<br>.((...))	TP73	NM_001204189	chr1:3597096-3642625	non-template	29	Intron_4	--	Yes	--	Yes	--	--	--	--	--	Yes	Yes
G4ST01003630442	chr1:3630443-3630471	+	GGGCACCACGTGGGTGGGGGGTGCAGGGG<br>.((...))	TP73	NM_001204190	chr1:3597096-3642625	non-template	29	Intron_4	--	Yes	--	Yes	--	--	--	--	--	Yes	Yes
G4ST01003630442	chr1:3630443-3630471	+	GGGCACCACGTGGGTGGGGGGTGCAGGGG<br>.((...))	TP73	NM_001204191	chr1:3597096-3642625	non-template	29	Intron_4	--	Yes	--	Yes	--	--	--	--	--	Yes	Yes
G4ST01003633980	chr1:3633981-3634007	+	GGGTGGGGAAGGTGGGCAGGTTGAGGG<br>((...)).	TP73	NM_005427	chr1:3558989-3642625	non-template	27	Intron_7	--	Yes	--	Yes	--	--	--	Yes	Yes	Yes	--
G4ST01003633980	chr1:3633981-3634007	+	GGGTGGGGAAGGTGGGCAGGTTGAGGG<br>((...)).	TP73	NM_001126242	chr1:3597096-3642625	non-template	27	Intron_5	--	Yes	--	Yes	--	--	--	Yes	Yes	Yes	--
G4ST01003633980	chr1:3633981-3634007	+	GGGTGGGGAAGGTGGGCAGGTTGAGGG<br>((...)).	TP73	NM_001204192	chr1:3604470-3642625	non-template	27	Intron_5	--	Yes	--	Yes	--	--	--	Yes	Yes	Yes	--
G4ST01003633980	chr1:3633981-3634007	+	GGGTGGGGAAGGTGGGCAGGTTGAGGG<br>((...)).	TP73	NM_001126241	chr1:3597096-3642625	non-template	27	Intron_5	--	Yes	--	Yes	--	--	--	Yes	Yes	Yes	--
G4ST01003633980	chr1:3633981-3634007	+	GGGTGGGGAAGGTGGGCAGGTTGAGGG<br>((...)).	TP73	NM_001126240	chr1:3597096-3642625	non-template	27	Intron_5	--	Yes	--	Yes	--	--	--	Yes	Yes	Yes	--
G4ST01003633980	chr1:3633981-3634007	+	GGGTGGGGAAGGTGGGCAGGTTGAGGG<br>((...)).	TP73	NM_001204184	chr1:3558989-3642625	non-template	27	Intron_7	--	Yes	--	Yes	--	--	--	Yes	Yes	Yes	--
G4ST01003633980	chr1:3633981-3634007	+	GGGTGGGGAAGGTGGGCAGGTTGAGGG<br>((...)).	TP73	NM_001204185	chr1:3558989-3642625	non-template	27	Intron_7	--	Yes	--	Yes	--	--	--	Yes	Yes	Yes	--
G4ST01003633980	chr1:3633981-3634007	+	GGGTGGGGAAGGTGGGCAGGTTGAGGG<br>((...)).	TP73	NM_001204186	chr1:3558989-3642625	non-template	27	Intron_7	--	Yes	--	Yes	--	--	--	Yes	Yes	Yes	--
G4ST01003633980	chr1:3633981-3634007	+	GGGTGGGGAAGGTGGGCAGGTTGAGGG<br>((...)).	TP73	NM_001204187	chr1:3558989-3642625	non-template	27	Intron_7	--	Yes	--	Yes	--	--	--	Yes	Yes	Yes	--
G4ST01003633980	chr1:3633981-3634007	+	GGGTGGGGAAGGTGGGCAGGTTGAGGG<br>((...)).	TP73	NM_001204188	chr1:3558989-3642625	non-template	27	Intron_7	--	Yes	--	Yes	--	--	--	Yes	Yes	Yes	--
G4ST01003633980	chr1:3633981-3634007	+	GGGTGGGGAAGGTGGGCAGGTTGAGGG<br>((...)).	TP73	NM_001204189	chr1:3597096-3642625	non-template	27	Intron_5	--	Yes	--	Yes	--	--	--	Yes	Yes	Yes	--
G4ST01003633980	chr1:3633981-3634007	+	GGGTGGGGAAGGTGGGCAGGTTGAGGG<br>((...)).	TP73	NM_001204190	chr1:3597096-3642625	non-template	27	Intron_5	--	Yes	--	Yes	--	--	--	Yes	Yes	Yes	--
G4ST01003633980	chr1:3633981-3634007	+	GGGTGGGGAAGGTGGGCAGGTTGAGGG<br>((...)).	TP73	NM_001204191	chr1:3597096-3642625	non-template	27	Intron_5	--	Yes	--	Yes	--	--	--	Yes	Yes	Yes	--
G4ST01003633984	chr1:3633985-3634011	+	GGGGAAGGTGGGCAGGTTGAGGGTGGG<br>((...)).	TP73	NM_005427	chr1:3558989-3642625	non-template	27	Intron_7	--	Yes	--	Yes	--	--	--	Yes	Yes	Yes	--
G4ST01003633984	chr1:3633985-3634011	+	GGGGAAGGTGGGCAGGTTGAGGGTGGG<br>((...)).	TP73	NM_001126242	chr1:3597096-3642625	non-template	27	Intron_5	--	Yes	--	Yes	--	--	--	Yes	Yes	Yes	--
G4ST01003633984	chr1:3633985-3634011	+	GGGGAAGGTGGGCAGGTTGAGGGTGGG<br>((...)).	TP73	NM_001204192	chr1:3604470-3642625	non-template	27	Intron_5	--	Yes	--	Yes	--	--	--	Yes	Yes	Yes	--
G4ST01003633984	chr1:3633985-3634011	+	GGGGAAGGTGGGCAGGTTGAGGGTGGG<br>((...)).	TP73	NM_001126241	chr1:3597096-3642625	non-template	27	Intron_5	--	Yes	--	Yes	--	--	--	Yes	Yes	Yes	--
G4ST01003633984	chr1:3633985-3634011	+	GGGGAAGGTGGGCAGGTTGAGGGTGGG<br>((...)).	TP73	NM_001126240	chr1:3597096-3642625	non-template	27	Intron_5	--	Yes	--	Yes	--	--	--	Yes	Yes	Yes	--
G4ST01003633984	chr1:3633985-3634011	+	GGGGAAGGTGGGCAGGTTGAGGGTGGG<br>((...)).	TP73	NM_001204184	chr1:3558989-3642625	non-template	27	Intron_7	--	Yes	--	Yes	--	--	--	Yes	Yes	Yes	--
G4ST01003633984	chr1:3633985-3634011	+	GGGGAAGGTGGGCAGGTTGAGGGTGGG<br>((...)).	TP73	NM_001204185	chr1:3558989-3642625	non-template	27	Intron_7	--	Yes	--	Yes	--	--	--	Yes	Yes	Yes	--
G4ST01003633984	chr1:3633985-3634011	+	GGGGAAGGTGGGCAGGTTGAGGGTGGG<br>((...)).	TP73	NM_001204186	chr1:3558989-3642625	non-template	27	Intron_7	--	Yes	--	Yes	--	--	--	Yes	Yes	Yes	--
G4ST01003633984	chr1:3633985-3634011	+	GGGGAAGGTGGGCAGGTTGAGGGTGGG<br>((...)).	TP73	NM_001204187	chr1:3558989-3642625	non-template	27	Intron_7	--	Yes	--	Yes	--	--	--	Yes	Yes	Yes	--
G4ST01003633984	chr1:3633985-3634011	+	GGGGAAGGTGGGCAGGTTGAGGGTGGG<br>((...)).	TP73	NM_001204188	chr1:3558989-3642625	non-template	27	Intron_7	--	Yes	--	Yes	--	--	--	Yes	Yes	Yes	--
G4ST01003633984	chr1:3633985-3634011	+	GGGGAAGGTGGGCAGGTTGAGGGTGGG<br>((...)).	TP73	NM_001204189	chr1:3597096-3642625	non-template	27	Intron_5	--	Yes	--	Yes	--	--	--	Yes	Yes	Yes	--
G4ST01003633984	chr1:3633985-3634011	+	GGGGAAGGTGGGCAGGTTGAGGGTGGG<br>((...)).	TP73	NM_001204190	chr1:3597096-3642625	non-template	27	Intron_5	--	Yes	--	Yes	--	--	--	Yes	Yes	Yes	--
G4ST01003633984	chr1:3633985-3634011	+	GGGGAAGGTGGGCAGGTTGAGGGTGGG<br>((...)).	TP73	NM_001204191	chr1:3597096-3642625	non-template	27	Intron_5	--	Yes	--	Yes	--	--	--	Yes	Yes	Yes	--
G4ST01003633993	chr1:3633994-3634016	+	GGGCAGGTTGAGGGTGGGCAGGG<br>((...)).	TP73	NM_005427	chr1:3558989-3642625	non-template	23	Intron_7	--	Yes	--	Yes	--	--	--	--	Yes	Yes	--
G4ST01003633993	chr1:3633994-3634016	+	GGGCAGGTTGAGGGTGGGCAGGG<br>((...)).	TP73	NM_001126242	chr1:3597096-3642625	non-template	23	Intron_5	--	Yes	--	Yes	--	--	--	--	Yes	Yes	--
G4ST01003633993	chr1:3633994-3634016	+	GGGCAGGTTGAGGGTGGGCAGGG<br>((...)).	TP73	NM_001204192	chr1:3604470-3642625	non-template	23	Intron_5	--	Yes	--	Yes	--	--	--	--	Yes	Yes	--
G4ST01003633993	chr1:3633994-3634016	+	GGGCAGGTTGAGGGTGGGCAGGG<br>((...)).	TP73	NM_001126241	chr1:3597096-3642625	non-template	23	Intron_5	--	Yes	--	Yes	--	--	--	--	Yes	Yes	--

G4ST01003633993	chr1:3633994-3634016	+	GGGCAGGTTGAGGGTGGGCAGGG<br>((...)).	TP73	NM_001126240	chr1:3597096-3642625	non-template	23	Intron_5	--	Yes	--	Yes	--	--	--	--	Yes	Yes	--
G4ST01003633993	chr1:3633994-3634016	+	GGGCAGGTTGAGGGTGGGCAGGG<br>((...)).	TP73	NM_001204184	chr1:3558989-3642625	non-template	23	Intron_7	--	Yes	--	Yes	--	--	--	--	Yes	Yes	--
G4ST01003633993	chr1:3633994-3634016	+	GGGCAGGTTGAGGGTGGGCAGGG<br>((...)).	TP73	NM_001204185	chr1:3558989-3642625	non-template	23	Intron_7	--	Yes	--	Yes	--	--	--	--	Yes	Yes	--
G4ST01003633993	chr1:3633994-3634016	+	GGGCAGGTTGAGGGTGGGCAGGG<br>((...)).	TP73	NM_001204186	chr1:3558989-3642625	non-template	23	Intron_7	--	Yes	--	Yes	--	--	--	--	Yes	Yes	--
G4ST01003633993	chr1:3633994-3634016	+	GGGCAGGTTGAGGGTGGGCAGGG<br>((...)).	TP73	NM_001204187	chr1:3558989-3642625	non-template	23	Intron_7	--	Yes	--	Yes	--	--	--	--	Yes	Yes	--
G4ST01003633993	chr1:3633994-3634016	+	GGGCAGGTTGAGGGTGGGCAGGG<br>((...)).	TP73	NM_001204188	chr1:3558989-3642625	non-template	23	Intron_7	--	Yes	--	Yes	--	--	--	--	Yes	Yes	--
G4ST01003633993	chr1:3633994-3634016	+	GGGCAGGTTGAGGGTGGGCAGGG<br>((...)).	TP73	NM_001204189	chr1:3597096-3642625	non-template	23	Intron_5	--	Yes	--	Yes	--	--	--	--	Yes	Yes	--
G4ST01003633993	chr1:3633994-3634016	+	GGGCAGGTTGAGGGTGGGCAGGG<br>((...)).	TP73	NM_001204190	chr1:3597096-3642625	non-template	23	Intron_5	--	Yes	--	Yes	--	--	--	--	Yes	Yes	--
G4ST01003633993	chr1:3633994-3634016	+	GGGCAGGTTGAGGGTGGGCAGGG<br>((...)).	TP73	NM_001204191	chr1:3597096-3642625	non-template	23	Intron_5	--	Yes	--	Yes	--	--	--	--	Yes	Yes	--
G4ST01003634291	chr1:3634292-3634324	-	GGGAGGAGTCTGGGCGTGGGGGGCCGTTCAGGG<br>((....))	TP73	NM_005427	chr1:3558989-3642625	template	33	Intron_8	--	Yes	--	Yes	--	--	--	--	--	--	--
G4ST01003634291	chr1:3634292-3634324	-	GGGAGGAGTCTGGGCGTGGGGGGCCGTTCAGGG<br>((....))	TP73	NM_001126242	chr1:3597096-3642625	template	33	Intron_6	--	Yes	--	Yes	--	--	--	--	--	--	--
G4ST01003634291	chr1:3634292-3634324	-	GGGAGGAGTCTGGGCGTGGGGGGCCGTTCAGGG<br>((....))	TP73	NM_001204192	chr1:3604470-3642625	template	33	Intron_6	--	Yes	--	Yes	--	--	--	--	--	--	--
G4ST01003634291	chr1:3634292-3634324	-	GGGAGGAGTCTGGGCGTGGGGGGCCGTTCAGGG<br>((....))	TP73	NM_001126241	chr1:3597096-3642625	template	33	Intron_6	--	Yes	--	Yes	--	--	--	--	--	--	--
G4ST01003634291	chr1:3634292-3634324	-	GGGAGGAGTCTGGGCGTGGGGGGCCGTTCAGGG<br>((....))	TP73	NM_001126240	chr1:3597096-3642625	template	33	Intron_6	--	Yes	--	Yes	--	--	--	--	--	--	--
G4ST01003634291	chr1:3634292-3634324	-	GGGAGGAGTCTGGGCGTGGGGGGCCGTTCAGGG<br>((....))	TP73	NM_001204184	chr1:3558989-3642625	template	33	Intron_8	--	Yes	--	Yes	--	--	--	--	--	--	--
G4ST01003634291	chr1:3634292-3634324	-	GGGAGGAGTCTGGGCGTGGGGGGCCGTTCAGGG<br>((....))	TP73	NM_001204185	chr1:3558989-3642625	template	33	Intron_8	--	Yes	--	Yes	--	--	--	--	--	--	--
G4ST01003634291	chr1:3634292-3634324	-	GGGAGGAGTCTGGGCGTGGGGGGCCGTTCAGGG<br>((....))	TP73	NM_001204186	chr1:3558989-3642625	template	33	Intron_8	--	Yes	--	Yes	--	--	--	--	--	--	--
G4ST01003634291	chr1:3634292-3634324	-	GGGAGGAGTCTGGGCGTGGGGGGCCGTTCAGGG<br>((....))	TP73	NM_001204187	chr1:3558989-3642625	template	33	Intron_8	--	Yes	--	Yes	--	--	--	--	--	--	--
G4ST01003634291	chr1:3634292-3634324	-	GGGAGGAGTCTGGGCGTGGGGGGCCGTTCAGGG<br>((....))	TP73	NM_001204188	chr1:3558989-3642625	template	33	Intron_8	--	Yes	--	Yes	--	--	--	--	--	--	--
G4ST01003634291	chr1:3634292-3634324	-	GGGAGGAGTCTGGGCGTGGGGGGCCGTTCAGGG<br>((....))	TP73	NM_001204189	chr1:3597096-3642625	template	33	Intron_6	--	Yes	--	Yes	--	--	--	--	--	--	--
G4ST01003634291	chr1:3634292-3634324	-	GGGAGGAGTCTGGGCGTGGGGGGCCGTTCAGGG<br>((....))	TP73	NM_001204190	chr1:3597096-3642625	template	33	Intron_6	--	Yes	--	Yes	--	--	--	--	--	--	--
G4ST01003634291	chr1:3634292-3634324	-	GGGAGGAGTCTGGGCGTGGGGGGCCGTTCAGGG<br>((....))	TP73	NM_001204191	chr1:3597096-3642625	template	33	Intron_6	--	Yes	--	Yes	--	--	--	--	--	--	--
G4ST01003640802	chr1:3640803-3640838	+	GGGCAGGGAACCGGAGCCCCTGGGGGGCCTCACGGG<br>..((((.....)))).	TP73	NM_005427	chr1:3558989-3642625	non-template	36	Exon_14.l	--	Yes	Yes	--	--	--	Yes	--	Yes	Yes	--
G4ST01003640802	chr1:3640803-3640838	+	GGGCAGGGAACCGGAGCCCCTGGGGGGCCTCACGGG<br>..((((.....)))).	TP73	NM_001126242	chr1:3597096-3642625	non-template	36	Exon_11.l	--	Yes	Yes	--	--	--	Yes	--	Yes	Yes	--
G4ST01003640802	chr1:3640803-3640838	+	GGGCAGGGAACCGGAGCCCCTGGGGGGCCTCACGGG<br>..((((.....)))).	TP73	NM_001204192	chr1:3604470-3642625	non-template	36	Exon_12.l	--	Yes	Yes	--	--	--	Yes	--	Yes	Yes	--
G4ST01003640802	chr1:3640803-3640838	+	GGGCAGGGAACCGGAGCCCCTGGGGGGCCTCACGGG<br>..((((.....)))).	TP73	NM_001126241	chr1:3597096-3642625	non-template	36	Exon_11.l	--	Yes	Yes	--	--	--	Yes	--	Yes	Yes	--
G4ST01003640802	chr1:3640803-3640838	+	GGGCAGGGAACCGGAGCCCCTGGGGGGCCTCACGGG<br>..((((.....)))).	TP73	NM_001126240	chr1:3597096-3642625	non-template	36	Exon_12.l	--	Yes	Yes	--	--	--	Yes	--	Yes	Yes	--
G4ST01003640802	chr1:3640803-3640838	+	GGGCAGGGAACCGGAGCCCCTGGGGGGCCTCACGGG<br>..((((.....)))).	TP73	NM_001204184	chr1:3558989-3642625	non-template	36	Exon_13.l	--	Yes	Yes	--	--	--	Yes	--	Yes	Yes	--
G4ST01003640802	chr1:3640803-3640838	+	GGGCAGGGAACCGGAGCCCCTGGGGGGCCTCACGGG<br>..((((.....)))).	TP73	NM_001204185	chr1:3558989-3642625	non-template	36	Exon_13.l	--	Yes	Yes	--	--	--	Yes	--	Yes	Yes	--
G4ST01003640802	chr1:3640803-3640838	+	GGGCAGGGAACCGGAGCCCCTGGGGGGCCTCACGGG<br>..((((.....)))).	TP73	NM_001204186	chr1:3558989-3642625	non-template	36	Exon_11.l	--	Yes	Yes	--	--	--	Yes	--	Yes	Yes	--
G4ST01003640802	chr1:3640803-3640838	+	GGGCAGGGAACCGGAGCCCCTGGGGGGCCTCACGGG<br>..((((.....)))).	TP73	NM_001204187	chr1:3558989-3642625	non-template	36	Exon_12.l	--	Yes	Yes	--	--	--	Yes	--	Yes	Yes	--
G4ST01003640802	chr1:3640803-3640838	+	GGGCAGGGAACCGGAGCCCCTGGGGGGCCTCACGGG<br>..((((.....)))).	TP73	NM_001204188	chr1:3558989-3642625	non-template	36	Exon_12.l	--	Yes	Yes	--	--	--	Yes	--	Yes	Yes	--
G4ST01003640802	chr1:3640803-3640838	+	GGGCAGGGAACCGGAGCCCCTGGGGGGCCTCACGGG<br>..((((.....)))).	TP73	NM_001204189	chr1:3597096-3642625	non-template	36	Exon_9.l	--	Yes	Yes	--	--	--	Yes	--	Yes	Yes	--
G4ST01003640802	chr1:3640803-3640838	+	GGGCAGGGAACCGGAGCCCCTGGGGGGCCTCACGGG<br>..((((.....)))).	TP73	NM_001204190	chr1:3597096-3642625	non-template	36	Exon_10.l	--	Yes	Yes	--	--	--	Yes	--	Yes	Yes	--
G4ST01003640802	chr1:3640803-3640838	+	GGGCAGGGAACCGGAGCCCCTGGGGGGCCTCACGGG<br>..((((.....)))).	TP73	NM_001204191	chr1:3597096-3642625	non-template	36	Exon_10.l	--	Yes	Yes	--	--	--	Yes	--	Yes	Yes	--
G4ST01003643324	chr1:3643325-3643357	-	GGGGACAGGTAGGAGCCTAGGGAAAGGGGTGGG<br>...((((....)))).	KIAA0495	NR_033712	chr1:3642408-3653195	non-template	33	Exon_6.l	--	Yes	Yes	--	--	--	--	--	--	Yes	Yes
G4ST01003643324	chr1:3643325-3643357	-	GGGGACAGGTAGGAGCCTAGGGAAAGGGGTGGG<br>...((((....)))).	KIAA0495	NR_033710	chr1:3642408-3653797	non-template	33	Exon_6.l	--	Yes	Yes	--	--	--	--	--	--	Yes	Yes
G4ST01003643324	chr1:3643325-3643357	-	GGGGACAGGTAGGAGCCTAGGGAAAGGGGTGGG<br>...((((....)))).	KIAA0495	NR_033709	chr1:3642408-3653797	non-template	33	Exon_5.l	--	Yes	Yes	--	--	--	--	--	--	Yes	Yes
G4ST01003643324	chr1:3643325-3643357	-	GGGGACAGGTAGGAGCCTAGGGAAAGGGGTGGG<br>...((((....)))).	KIAA0495	NR_033711	chr1:3642408-3653797	non-template	33	Exon_4.l	--	Yes	Yes	--	--	--	--	--	--	Yes	Yes
G4ST01003643324	chr1:3643325-3643357	-	GGGGACAGGTAGGAGCCTAGGGAAAGGGGTGGG<br>...((((....)))).	KIAA0495	NR_033708	chr1:3642408-3653797	non-template	33	Exon_5.l	--	Yes	Yes	--	--	--	--	--	--	Yes	Yes
G4ST01003652202	chr1:3652203-3652243	-	GGGGTGGCTGGCCACCTTGGCCTGGGCATCTTTGGGGTGGG<br>......(((((...))))).	KIAA0495	NR_033712	chr1:3642408-3653195	non-template	41	Exon_2	--	Yes	Yes	--	--	--	--	--	--	--	Yes
G4ST01003652202	chr1:3652203-3652243	-	GGGGTGGCTGGCCACCTTGGCCTGGGCATCTTTGGGGTGGG<br>......(((((...))))).	KIAA0495	NR_033710	chr1:3642408-3653797	non-template	41	Exon_1.f	--	Yes	Yes	--	--	--	--	--	--	--	Yes
G4ST01003652202	chr1:3652203-3652243	-	GGGGTGGCTGGCCACCTTGGCCTGGGCATCTTTGGGGTGGG<br>......(((((...))))).	KIAA0495	NR_033709	chr1:3642408-3653797	non-template	41	Exon_1.f	--	Yes	Yes	--	--	--	--	--	--	--	Yes
G4ST01003652202	chr1:3652203-3652243	-	GGGGTGGCTGGCCACCTTGGCCTGGGCATCTTTGGGGTGGG<br>......(((((...))))).	KIAA0495	NR_033711	chr1:3642408-3653797	non-template	41	Exon_1.f	--	Yes	Yes	--	--	--	--	--	--	--	Yes
G4ST01003652202	chr1:3652203-3652243	-	GGGGTGGCTGGCCACCTTGGCCTGGGCATCTTTGGGGTGGG<br>......(((((...))))).	KIAA0495	NR_033708	chr1:3642408-3653797	non-template	41	Exon_1.f	--	Yes	Yes	--	--	--	--	--	--	--	Yes
G4ST01003660818	chr1:3660819-3660857	+	GGGAGCCAGGGCAGCAATGAAGCTGGGGGTGGAGGGGGG<br>((((......)))).	CCDC27	NM_152492	chr1:3658825-3678069	non-template	39	Intron_2	--	Yes	--	Yes	--	--	--	--	--	--	--
G4ST01003723939	chr1:3723940-3723964	-	GGGACAGGAGTGGGCTGGGCTGGGG<br>((....))	KIAA0562	NM_014704	chr1:3718505-3763657	non-template	25	Intron_20	--	Yes	--	Yes	--	--	--	--	--	--	--
G4ST01003731315	chr1:3731316-3731348	+	GGGAAGGGGGCAGTGGCACTGTGGGAAGGGGGG<br>..((((...)))).	KIAA0562	NM_014704	chr1:3718505-3763657	template	33	Intron_18	--	Yes	--	Yes	--	--	--	--	--	--	--
G4ST01003731320	chr1:3731321-3731363	+	GGGGGCAGTGGCACTGTGGGAAGGGGGGCAGTGGCACTGTGGG<br>..((((...)))). ...((((...)))).	KIAA0562	NM_014704	chr1:3718505-3763657	template	43	Intron_18	--	Yes	--	Yes	--	--	--	--	--	--	--
G4ST01003731337	chr1:3731338-3731370	+	GGGAAGGGGGGCAGTGGCACTGTGGGAAGGGGG<br>...((((...)))).	KIAA0562	NM_014704	chr1:3718505-3763657	template	33	Intron_18	--	Yes	--	Yes	--	--	--	--	--	--	--

G4ST01003731342	chr1:3731343-3731385	+	GGGGGGCAGTGGCACTGTGGGAAGGGGGCAGTGGCACTGTGGG<br>...((((...)))). ..((((...)))).	KIAA0562	NM_014704	chr1:3718505-3763657	template	43	Intron_18	--	Yes	--	Yes	--	--	--	--	--	--	--
G4ST01003731360	chr1:3731361-3731392	+	GGGAAGGGGGCAGTGGCACTGTGGGAAGGGGG<br>..((((...)))).	KIAA0562	NM_014704	chr1:3718505-3763657	template	32	Intron_18	--	Yes	--	Yes	--	--	--	--	--	--	--
G4ST01003731365	chr1:3731366-3731408	+	GGGGGCAGTGGCACTGTGGGAAGGGGGACAGTGGCACTGTGGG<br>..((((...)))). ..(((((...)))))	KIAA0562	NM_014704	chr1:3718505-3763657	template	43	Intron_18	--	Yes	--	Yes	--	--	--	--	--	--	--
G4ST01003731382	chr1:3731383-3731415	+	GGGAAGGGGGACAGTGGCACTGTGGGAAGGGGG<br>..(((((...)))))	KIAA0562	NM_014704	chr1:3718505-3763657	template	33	Intron_18	--	Yes	--	Yes	--	--	--	--	--	--	--
G4ST01003731387	chr1:3731388-3731430	+	GGGGGACAGTGGCACTGTGGGAAGGGGGCAGTGGCACTGTGGG<br>..(((((...))))) ..((((...)))).	KIAA0562	NM_014704	chr1:3718505-3763657	template	43	Intron_18	--	Yes	--	Yes	--	--	--	--	--	--	--
G4ST01003731405	chr1:3731406-3731437	+	GGGAAGGGGGCAGTGGCACTGTGGGAAGGGGG<br>..((((...)))).	KIAA0562	NM_014704	chr1:3718505-3763657	template	32	Intron_18	--	Yes	--	Yes	--	--	--	--	--	--	--
G4ST01003731410	chr1:3731411-3731452	+	GGGGGCAGTGGCACTGTGGGAAGGGGGCAGTGGCACTGTGGG<br>..((((...)))). ..((((...)))).	KIAA0562	NM_014704	chr1:3718505-3763657	template	42	Intron_18	--	Yes	--	Yes	--	--	--	--	--	--	--
G4ST01003731427	chr1:3731428-3731460	+	GGGAAGGGGGGCAGTGGCACTGTGGGAAGGGGG<br>..((((...)))).	KIAA0562	NM_014704	chr1:3718505-3763657	template	33	Intron_18	--	Yes	--	Yes	--	--	--	--	--	--	--
G4ST01003731432	chr1:3731433-3731475	+	GGGGGCAGTGGCACTGTGGGAAGGGGGGCAGTGGCACTGTGGG<br>..((((...)))). ...((((...)))).	KIAA0562	NM_014704	chr1:3718505-3763657	template	43	Intron_18	--	Yes	--	Yes	--	--	--	--	--	--	--
G4ST01003731449	chr1:3731450-3731482	+	GGGAAGGGGGGCAGTGGCACTGTGGGAAGGGGG<br>...((((...)))).	KIAA0562	NM_014704	chr1:3718505-3763657	template	33	Intron_18	--	Yes	--	Yes	--	--	--	--	--	--	--
G4ST01003731454	chr1:3731455-3731497	+	GGGGGGCAGTGGCACTGTGGGAAGGGGGCAGTGGCACTGTGGG<br>...((((...)))). ..((((...)))).	KIAA0562	NM_014704	chr1:3718505-3763657	template	43	Intron_18	--	Yes	--	Yes	--	--	--	--	--	--	--
G4ST01003731472	chr1:3731473-3731502	+	GGGAAGGGGGCAGTGGCACTGTGGGAAGGG<br>..((((...)))).	KIAA0562	NM_014704	chr1:3718505-3763657	template	30	Intron_18	--	Yes	--	Yes	--	--	--	--	--	--	--
G4ST01003731477	chr1:3731478-3731519	+	GGGGGCAGTGGCACTGTGGGAAGGGAGCAGTGGCACTGTGGG<br>..((((...)))). ..((((...)))).	KIAA0562	NM_014704	chr1:3718505-3763657	template	42	Intron_18	--	Yes	--	Yes	--	--	--	--	--	--	--
G4ST01003731494	chr1:3731495-3731527	+	GGGAAGGGAGCAGTGGCACTGTGGGAAGGGGGG<br>..((((...)))).	KIAA0562	NM_014704	chr1:3718505-3763657	template	33	Intron_18	--	Yes	--	Yes	--	--	--	--	--	--	--
G4ST01003731499	chr1:3731500-3731542	+	GGGAGCAGTGGCACTGTGGGAAGGGGGGCAGTGGCACTGTGGG<br>..((((...)))). ...((((...)))).	KIAA0562	NM_014704	chr1:3718505-3763657	template	43	Intron_18	--	Yes	--	Yes	--	--	--	--	--	--	--
G4ST01003731516	chr1:3731517-3731549	+	GGGAAGGGGGGCAGTGGCACTGTGGGAAGGGGG<br>...((((...)))).	KIAA0562	NM_014704	chr1:3718505-3763657	template	33	Intron_18	--	Yes	--	Yes	--	--	--	--	--	--	--
G4ST01003731521	chr1:3731522-3731564	+	GGGGGGCAGTGGCACTGTGGGAAGGGGGCAGTGGCACTGTGGG<br>...((((...)))). ..((((...)))).	KIAA0562	NM_014704	chr1:3718505-3763657	template	43	Intron_18	--	Yes	--	Yes	--	--	--	--	--	--	--
G4ST01003731539	chr1:3731540-3731571	+	GGGAAGGGGGCAGTGGCACTGTGGGAAGGGGG<br>..((((...)))).	KIAA0562	NM_014704	chr1:3718505-3763657	template	32	Intron_18	--	Yes	--	Yes	--	--	--	--	--	--	--
G4ST01003731544	chr1:3731545-3731586	+	GGGGGCAGTGGCACTGTGGGAAGGGGGCAGTGGCACTGTGGG<br>..((((...)))). ..((((...)))).	KIAA0562	NM_014704	chr1:3718505-3763657	template	42	Intron_18	--	Yes	--	Yes	--	--	--	--	--	--	--
G4ST01003731561	chr1:3731562-3731593	+	GGGAAGGGGGCAGTGGCACTGTGGGAAGGGGG<br>..((((...)))).	KIAA0562	NM_014704	chr1:3718505-3763657	template	32	Intron_18	--	Yes	--	Yes	--	--	--	--	--	--	--
G4ST01003758672	chr1:3758673-3758700	-	GGGGCTTGGGCTGGGAGGCAGCCTCGGG<br>(((...))).	KIAA0562	NM_014704	chr1:3718505-3763657	non-template	28	Intron_2	--	Yes	--	Yes	--	--	--	--	--	--	Yes
G4ST01003806551	chr1:3806552-3806586	-	GGGTCCCGGCAGCAGCTGCGCCGGGCAGGGCGGGG<br>....(((.((....)))))	LOC100133612	NR_024455	chr1:3806828-3821871	template	-241	upstream	Yes	--	--	--	--	--	--	Yes	Yes	Yes	Yes
G4ST01003806551	chr1:3806552-3806586	-	GGGTCCCGGCAGCAGCTGCGCCGGGCAGGGCGGGG<br>....(((.((....)))))	C1orf174	NM_207356	chr1:3795557-3806717	non-template	35	Intron_1.f	--	Yes	--	Yes	--	--	--	Yes	Yes	Yes	Yes
G4ST01003806556	chr1:3806557-3806591	-	GGGCTGGGTCCCGGCAGCAGCTGCGCCGGGCAGGG<br>....(((.((....)))))	LOC100133612	NR_024455	chr1:3806828-3821871	template	-236	upstream	Yes	--	--	--	--	--	--	Yes	Yes	Yes	Yes
G4ST01003806556	chr1:3806557-3806591	-	GGGCTGGGTCCCGGCAGCAGCTGCGCCGGGCAGGG<br>....(((.((....)))))	C1orf174	NM_207356	chr1:3795557-3806717	non-template	35	Intron_1.f	--	Yes	--	Yes	--	--	--	Yes	Yes	Yes	Yes
G4ST01003807610	chr1:3807611-3807646	+	GGGCGGGAGGGGGCCTTAGTGGAGGCCGTCGGTGGG<br>((((((....)))))))......	LOC100133612	NR_024455	chr1:3806828-3821871	non-template	36	Intron_1.f	--	Yes	--	Yes	--	--	--	--	Yes	Yes	--
G4ST01003807610	chr1:3807611-3807646	+	GGGCGGGAGGGGGCCTTAGTGGAGGCCGTCGGTGGG<br>((((((....)))))))......	C1orf174	NM_207356	chr1:3795557-3806717	template	-893	upstream	Yes	--	--	--	--	--	--	--	Yes	Yes	--
G4ST01003810632	chr1:3810633-3810670	-	GGGGCCTGCATCCTACAGGATGAGGGAGGGAGAGGGGG<br>.....(((((....))))).	LOC100133612	NR_024455	chr1:3806828-3821871	template	38	Intron_2	--	Yes	--	Yes	--	--	--	--	Yes	--	--
G4ST01003810640	chr1:3810641-3810680	-	GGGCCTGAAAGGGGCCTGCATCCTACAGGATGAGGGAGGG<br>.....(((((....))))).	LOC100133612	NR_024455	chr1:3806828-3821871	template	40	Intron_2	--	Yes	--	Yes	--	--	--	--	Yes	--	--
G4ST01003810644	chr1:3810645-3810684	-	GGGTGGGCCTGAAAGGGGCCTGCATCCTACAGGATGAGGG<br>.....(((((....))))).	LOC100133612	NR_024455	chr1:3806828-3821871	template	40	Intron_2	--	Yes	--	Yes	--	--	--	--	Yes	--	--
G4ST01004614419	chr1:4614420-4614457	+	GGGGCTCGAGCTCCCGTTCGAGGGGCGGGGAAGGCGGG<br>.(((((((....)))))))	AJAP1	NM_018836	chr1:4614965-4743711	non-template	-507	upstream	Yes	--	--	--	--	--	--	Yes	--	--	--
G4ST01004614419	chr1:4614420-4614457	+	GGGGCTCGAGCTCCCGTTCGAGGGGCGGGGAAGGCGGG<br>.(((((((....)))))))	AJAP1	NM_001042478	chr1:4614965-4737714	non-template	-507	upstream	Yes	--	--	--	--	--	--	Yes	--	--	--
G4ST01004614539	chr1:4614540-4614586	+	GGGAGGCGGCTGAGCAGCGCGGGCGGCTCTGCGGCGGGCGCGGTGGG<br>..(((.((....))))) ..(((....)))	AJAP1	NM_018836	chr1:4614965-4743711	non-template	-378	upstream	Yes	--	--	--	--	--	--	Yes	--	--	Yes
G4ST01004614539	chr1:4614540-4614586	+	GGGAGGCGGCTGAGCAGCGCGGGCGGCTCTGCGGCGGGCGCGGTGGG<br>..(((.((....))))) ..(((....)))	AJAP1	NM_001042478	chr1:4614965-4737714	non-template	-378	upstream	Yes	--	--	--	--	--	--	Yes	--	--	Yes
G4ST01004614559	chr1:4614560-4614592	+	GGGCGGCTCTGCGGCGGGCGCGGTGGGCGCGGG<br>..(((....)))	AJAP1	NM_018836	chr1:4614965-4743711	non-template	-372	upstream	Yes	--	--	--	--	--	--	--	--	--	Yes
G4ST01004614559	chr1:4614560-4614592	+	GGGCGGCTCTGCGGCGGGCGCGGTGGGCGCGGG<br>..(((....)))	AJAP1	NM_001042478	chr1:4614965-4737714	non-template	-372	upstream	Yes	--	--	--	--	--	--	--	--	--	Yes
G4ST01004631372	chr1:4631373-4631400	-	GGGCTCTAAGTGGGCTGGGGACAAAGGG<br>((...)).	AJAP1	NM_018836	chr1:4614965-4743711	template	28	Intron_1.f	--	Yes	--	Yes	--	--	--	--	--	--	--
G4ST01004631372	chr1:4631373-4631400	-	GGGCTCTAAGTGGGCTGGGGACAAAGGG<br>((...)).	AJAP1	NM_001042478	chr1:4614965-4737714	template	28	Intron_1.f	--	Yes	--	Yes	--	--	--	--	--	--	--
G4ST01004631380	chr1:4631381-4631407	-	GGGCCCTGGGCTCTAAGTGGGCTGGGG<br>((...)).	AJAP1	NM_018836	chr1:4614965-4743711	template	27	Intron_1.f	--	Yes	--	Yes	--	--	--	--	--	--	--
G4ST01004631380	chr1:4631381-4631407	-	GGGCCCTGGGCTCTAAGTGGGCTGGGG<br>((...)).	AJAP1	NM_001042478	chr1:4614965-4737714	template	27	Intron_1.f	--	Yes	--	Yes	--	--	--	--	--	--	--
G4ST01004632081	chr1:4632082-4632112	+	GGGAGGAGGGAGCGGTCGGGAGTCACTTGGG<br>((...)).	AJAP1	NM_018836	chr1:4614965-4743711	non-template	31	Intron_1.f	--	Yes	--	Yes	--	--	--	--	--	--	--
G4ST01004632081	chr1:4632082-4632112	+	GGGAGGAGGGAGCGGTCGGGAGTCACTTGGG<br>((...)).	AJAP1	NM_001042478	chr1:4614965-4737714	non-template	31	Intron_1.f	--	Yes	--	Yes	--	--	--	--	--	--	--
G4ST01004634956	chr1:4634957-4634999	+	GGGCAGTGGTCAGAGCCATCAGGGCTGGAGAGCGGTGGGGGGG<br>..(((((....))))).. (((.....))).	AJAP1	NM_018836	chr1:4614965-4743711	non-template	43	Intron_1.f	--	Yes	--	Yes	--	--	--	--	--	--	--
G4ST01004634956	chr1:4634957-4634999	+	GGGCAGTGGTCAGAGCCATCAGGGCTGGAGAGCGGTGGGGGGG<br>..(((((....))))).. (((.....))).	AJAP1	NM_001042478	chr1:4614965-4737714	non-template	43	Intron_1.f	--	Yes	--	Yes	--	--	--	--	--	--	--
G4ST01004653286	chr1:4653287-4653318	+	GGGCTTCCTGGAGGCAGGGGGCGGGGGAGGG<br>...((((....))))	AJAP1	NM_018836	chr1:4614965-4743711	non-template	32	Intron_1.f	--	Yes	--	Yes	--	--	--	--	--	--	--
G4ST01004653286	chr1:4653287-4653318	+	GGGCTTCCTGGAGGCAGGGGGCGGGGGAGGG<br>...((((....))))	AJAP1	NM_001042478	chr1:4614965-4737714	non-template	32	Intron_1.f	--	Yes	--	Yes	--	--	--	--	--	--	--
G4ST01004664221	chr1:4664222-4664251	-	GGGCACCGTTGTGGGGTGGGGGGAGGGGGG<br>(((....)))	AJAP1	NM_018836	chr1:4614965-4743711	template	30	Intron_1.f	--	Yes	--	Yes	--	--	--	--	--	--	--
G4ST01004664221	chr1:4664222-4664251	-	GGGCACCGTTGTGGGGTGGGGGGAGGGGGG<br>(((....)))	AJAP1	NM_001042478	chr1:4614965-4737714	template	30	Intron_1.f	--	Yes	--	Yes	--	--	--	--	--	--	--
G4ST01004670865	chr1:4670866-4670903	-	GGGCGGGGCTGCCTCTCCACGCAGCGGGGCTGCAGGGG<br>(((((........))))).	AJAP1	NM_018836	chr1:4614965-4743711	template	38	Intron_1.f	--	Yes	--	Yes	--	--	--	Yes	Yes	--	--
G4ST01004670865	chr1:4670866-4670903	-	GGGCGGGGCTGCCTCTCCACGCAGCGGGGCTGCAGGGG<br>(((((........))))).	AJAP1	NM_001042478	chr1:4614965-4737714	template	38	Intron_1.f	--	Yes	--	Yes	--	--	--	Yes	Yes	--	--

G4ST01004672091	chr1:4672092-4672129	-	GGGCACGAGGGCGGCCGCCTGGTCCCGGGGCCTGTGGG<br>.((((....)))). ..	AJAP1	NM_018836	chr1:4614965-4743711	template	38	Exon_2	--	Yes	Yes	--	Yes	--	--	--	--	--	--
G4ST01004672091	chr1:4672092-4672129	-	GGGCACGAGGGCGGCCGCCTGGTCCCGGGGCCTGTGGG<br>.((((....)))). ..	AJAP1	NM_001042478	chr1:4614965-4737714	template	38	Exon_2	--	Yes	Yes	--	Yes	--	--	--	--	--	--
G4ST01004672099	chr1:4672100-4672152	-	GGGGGCTTGGCCAGTCCTGCCTTGGGCACGAGGGCGGCCGCCTGGTCCCGGGG<br>(((..((.....)).))).. .((((....)))). ..	AJAP1	NM_018836	chr1:4614965-4743711	template	53	Exon_2	--	Yes	Yes	--	Yes	--	--	--	--	--	--
G4ST01004672099	chr1:4672100-4672152	-	GGGGGCTTGGCCAGTCCTGCCTTGGGCACGAGGGCGGCCGCCTGGTCCCGGGG<br>(((..((.....)).))).. .((((....)))). ..	AJAP1	NM_001042478	chr1:4614965-4737714	template	53	Exon_2	--	Yes	Yes	--	Yes	--	--	--	--	--	--
G4ST01004689181	chr1:4689182-4689214	+	GGGGAGCACAGGGCAGGGCCCGAGTCTCGAGGG<br>..(((...))).	AJAP1	NM_018836	chr1:4614965-4743711	non-template	33	Intron_2	--	Yes	--	Yes	--	--	--	--	--	--	--
G4ST01004689181	chr1:4689182-4689214	+	GGGGAGCACAGGGCAGGGCCCGAGTCTCGAGGG<br>..(((...))).	AJAP1	NM_001042478	chr1:4614965-4737714	non-template	33	Intron_2	--	Yes	--	Yes	--	--	--	--	--	--	--
G4ST01004696269	chr1:4696270-4696301	-	GGGAAAGGGAGGGCTGCGCACAGCGCAGCGGG<br>((((((...)))))).	AJAP1	NM_018836	chr1:4614965-4743711	template	32	Intron_2	--	Yes	--	Yes	--	--	--	Yes	--	--	--
G4ST01004696269	chr1:4696270-4696301	-	GGGAAAGGGAGGGCTGCGCACAGCGCAGCGGG<br>((((((...)))))).	AJAP1	NM_001042478	chr1:4614965-4737714	template	32	Intron_2	--	Yes	--	Yes	--	--	--	Yes	--	--	--
G4ST01004704357	chr1:4704358-4704395	+	GGGGAGAGGCCTCGGGGGAGGATGTGGGCCTGGCTGGG<br>(((....)))..	AJAP1	NM_018836	chr1:4614965-4743711	non-template	38	Intron_2	--	Yes	--	Yes	--	--	--	--	Yes	--	--
G4ST01004704357	chr1:4704358-4704395	+	GGGGAGAGGCCTCGGGGGAGGATGTGGGCCTGGCTGGG<br>(((....)))..	AJAP1	NM_001042478	chr1:4614965-4737714	non-template	38	Intron_2	--	Yes	--	Yes	--	--	--	--	Yes	--	--
G4ST01005850136	chr1:5850137-5850167	+	GGGCCGCGGGAGCTGGGATTTTTTAGAAGGG<br>.(((....)))	NPHP4	NM_015102	chr1:5845457-5975120	template	31	Intron_24	--	Yes	--	Yes	--	--	--	--	--	--	--
G4ST01005872215	chr1:5872216-5872252	-	GGGAGGCAGAGCCGGGTCCCGCTGGGCATGTGTGGGG<br>.(((...)))	NPHP4	NM_015102	chr1:5845457-5975120	non-template	37	Intron_17	--	Yes	--	Yes	--	--	--	--	--	--	--
G4ST01005892615	chr1:5892616-5892666	-	GGGGCTTCCTCCTGCTGAGGTGCAGGGGCTGAGAGCCCTGGGTCTGCTGGG<br>((..((((.....)))).)).. (((...)))...	NPHP4	NM_015102	chr1:5845457-5975120	non-template	51	Intron_11	--	Yes	--	Yes	--	--	--	--	Yes	Yes	--
G4ST01005987584	chr1:5987585-5987620	+	GGGTGGAGTGGGGGGATGTGGGAGCTGAGCTGAGGG<br>(((...)))..	KCNAB2	NM_001199861	chr1:5974945-6083840	non-template	36	Intron_1.f	--	Yes	--	Yes	--	--	--	--	--	--	Yes
G4ST01005987593	chr1:5987594-5987627	+	GGGGGGATGTGGGAGCTGAGCTGAGGGATGAGGG<br>(((...)))..	KCNAB2	NM_001199861	chr1:5974945-6083840	non-template	34	Intron_1.f	--	Yes	--	Yes	--	--	--	--	--	--	Yes
G4ST01005987642	chr1:5987643-5987678	+	GGGAGCTGAGCTAAGGGGTGAGGGTGGAGTAGGGGG<br>(((...)))...	KCNAB2	NM_001199861	chr1:5974945-6083840	non-template	36	Intron_1.f	--	Yes	--	Yes	--	--	--	--	--	--	Yes
G4ST01005987742	chr1:5987743-5987779	+	GGGTAGAGTGGGGGGATGTGGGAGCTGAGCTGAGGGG<br>(((...)))...	KCNAB2	NM_001199861	chr1:5974945-6083840	non-template	37	Intron_1.f	--	Yes	--	Yes	--	--	--	--	--	--	Yes
G4ST01005987751	chr1:5987752-5987785	+	GGGGGGATGTGGGAGCTGAGCTGAGGGGTGAGGG<br>(((...)))...	KCNAB2	NM_001199861	chr1:5974945-6083840	non-template	34	Intron_1.f	--	Yes	--	Yes	--	--	--	--	--	--	Yes
G4ST01005987761	chr1:5987762-5987797	+	GGGAGCTGAGCTGAGGGGTGAGGGTGCAGTGGGGGG<br>(((...)))...	KCNAB2	NM_001199861	chr1:5974945-6083840	non-template	36	Intron_1.f	--	Yes	--	Yes	--	--	--	--	--	--	Yes
G4ST01005987782	chr1:5987783-5987819	+	GGGTGCAGTGGGGGGACGTGGGAGCTGAGCTGAGGGG<br>(((...)))...	KCNAB2	NM_001199861	chr1:5974945-6083840	non-template	37	Intron_1.f	--	Yes	--	Yes	--	--	--	--	--	--	Yes
G4ST01005987791	chr1:5987792-5987825	+	GGGGGGACGTGGGAGCTGAGCTGAGGGGTGAGGG<br>(((...)))...	KCNAB2	NM_001199861	chr1:5974945-6083840	non-template	34	Intron_1.f	--	Yes	--	Yes	--	--	--	--	--	--	Yes
G4ST01005987801	chr1:5987802-5987837	+	GGGAGCTGAGCTGAGGGGTGAGGGTAGAGTGGGGGG<br>(((...)))...	KCNAB2	NM_001199861	chr1:5974945-6083840	non-template	36	Intron_1.f	--	Yes	--	Yes	--	--	--	--	--	--	Yes
G4ST01005987822	chr1:5987823-5987859	+	GGGTAGAGTGGGGGGATGTGGGAGCTGAGCTGAGGGG<br>(((...)))...	KCNAB2	NM_001199861	chr1:5974945-6083840	non-template	37	Intron_1.f	--	Yes	--	Yes	--	--	--	--	--	--	Yes
G4ST01005987831	chr1:5987832-5987865	+	GGGGGGATGTGGGAGCTGAGCTGAGGGGTGAGGG<br>(((...)))...	KCNAB2	NM_001199861	chr1:5974945-6083840	non-template	34	Intron_1.f	--	Yes	--	Yes	--	--	--	--	--	--	Yes
G4ST01005987841	chr1:5987842-5987877	+	GGGAGCTGAGCTGAGGGGTGAGGGTGGAGTAGGGGG<br>(((...)))...	KCNAB2	NM_001199861	chr1:5974945-6083840	non-template	36	Intron_1.f	--	Yes	--	Yes	--	--	--	--	--	--	Yes
G4ST01005988021	chr1:5988022-5988057	+	GGGTGAACTGGGGGATGTGGGAGCTGAGCTGAGGGG<br>(((...)))...	KCNAB2	NM_001199861	chr1:5974945-6083840	non-template	36	Intron_1.f	--	Yes	--	Yes	--	--	--	--	--	--	--
G4ST01005988030	chr1:5988031-5988063	+	GGGGGATGTGGGAGCTGAGCTGAGGGGTGGGGG<br>(((...)))...	KCNAB2	NM_001199861	chr1:5974945-6083840	non-template	33	Intron_1.f	--	Yes	--	Yes	--	--	--	--	--	--	--
G4ST01005988540	chr1:5988541-5988574	-	GGGGGGATGTGGGAGCTGAGCTGAGGGGTGAGGG<br>(((...)))...	KCNAB2	NM_001199861	chr1:5974945-6083840	template	34	Intron_1.f	--	Yes	--	Yes	--	--	--	--	--	--	Yes
G4ST01005988546	chr1:5988547-5988583	-	GGGTGGAGTGGGGGGATGTGGGAGCTGAGCTGAGGGG<br>(((...)))...	KCNAB2	NM_001199861	chr1:5974945-6083840	template	37	Intron_1.f	--	Yes	--	Yes	--	--	--	--	--	--	Yes
G4ST01005988649	chr1:5988650-5988682	-	GGGAGCTGAGCTGAGGGGTGAGGGTGGAGTGGG<br>(((...)))...	KCNAB2	NM_001199861	chr1:5974945-6083840	template	33	Intron_1.f	--	Yes	--	Yes	--	--	--	--	--	--	Yes
G4ST01005988658	chr1:5988659-5988692	-	GGGGGGATGTGGGAGCTGAGCTGAGGGGTGAGGG<br>(((...)))...	KCNAB2	NM_001199861	chr1:5974945-6083840	template	34	Intron_1.f	--	Yes	--	Yes	--	--	--	--	--	--	Yes
G4ST01005988664	chr1:5988665-5988701	-	GGGTGGAGTGGGGGGATGTGGGAGCTGAGCTGAGGGG<br>(((...)))...	KCNAB2	NM_001199861	chr1:5974945-6083840	template	37	Intron_1.f	--	Yes	--	Yes	--	--	--	--	--	--	Yes
G4ST01005988728	chr1:5988729-5988761	-	GGGAGCTGAGCTGAGGGGTGAGGGTGGAGTGGG<br>(((...)))...	KCNAB2	NM_001199861	chr1:5974945-6083840	template	33	Intron_1.f	--	Yes	--	Yes	--	--	--	--	--	--	Yes
G4ST01005988737	chr1:5988738-5988771	-	GGGGGGACGTGGGAGCTGAGCTGAGGGGTGAGGG<br>(((...)))...	KCNAB2	NM_001199861	chr1:5974945-6083840	template	34	Intron_1.f	--	Yes	--	Yes	--	--	--	--	--	--	Yes
G4ST01005988743	chr1:5988744-5988780	-	GGGTGGAGTGGGGGGACGTGGGAGCTGAGCTGAGGGG<br>(((...)))...	KCNAB2	NM_001199861	chr1:5974945-6083840	template	37	Intron_1.f	--	Yes	--	Yes	--	--	--	--	--	--	Yes
G4ST01005988765	chr1:5988766-5988801	-	GGGAGCTGAGCTGAGGGGTGAGGGTGGAGTGGGGGG<br>(((...)))...	KCNAB2	NM_001199861	chr1:5974945-6083840	template	36	Intron_1.f	--	Yes	--	Yes	--	--	--	--	--	--	Yes
G4ST01005988777	chr1:5988778-5988811	-	GGGGGGATGTGGGAGCTGAGCTGAGGGGTGAGGG<br>(((...)))...	KCNAB2	NM_001199861	chr1:5974945-6083840	template	34	Intron_1.f	--	Yes	--	Yes	--	--	--	--	--	--	Yes
G4ST01005988783	chr1:5988784-5988820	-	GGGTGGAGTGGGGGGATGTGGGAGCTGAGCTGAGGGG<br>(((...)))...	KCNAB2	NM_001199861	chr1:5974945-6083840	template	37	Intron_1.f	--	Yes	--	Yes	--	--	--	--	--	--	Yes
G4ST01005988886	chr1:5988887-5988919	-	GGGAGCTGAGCTGAGGGGTGAGGGTGGAGTGGG<br>(((...)))...	KCNAB2	NM_001199861	chr1:5974945-6083840	template	33	Intron_1.f	--	Yes	--	Yes	--	--	--	--	--	--	Yes
G4ST01005988895	chr1:5988896-5988929	-	GGGGGGATGTGGGAGCTGAGCTGAGGGGTGAGGG<br>(((...)))...	KCNAB2	NM_001199861	chr1:5974945-6083840	template	34	Intron_1.f	--	Yes	--	Yes	--	--	--	--	--	--	Yes
G4ST01005988901	chr1:5988902-5988938	-	GGGTGGAGTGGGGGGATGTGGGAGCTGAGCTGAGGGG<br>(((...)))...	KCNAB2	NM_001199861	chr1:5974945-6083840	template	37	Intron_1.f	--	Yes	--	Yes	--	--	--	--	--	--	Yes
G4ST01005988965	chr1:5988966-5988998	-	GGGAGCTGAGCTGAGGGGTGAGGGTGGAGTGGG<br>(((...)))...	KCNAB2	NM_001199861	chr1:5974945-6083840	template	33	Intron_1.f	--	Yes	--	Yes	--	--	--	--	--	--	Yes
G4ST01005988974	chr1:5988975-5989008	-	GGGGGGACGTGGGAGCTGAGCTGAGGGGTGAGGG<br>(((...)))...	KCNAB2	NM_001199861	chr1:5974945-6083840	template	34	Intron_1.f	--	Yes	--	Yes	--	--	--	--	--	--	Yes
G4ST01005988980	chr1:5988981-5989019	-	GGGGGTGGAGTGGGGGGACGTGGGAGCTGAGCTGAGGGG<br>(((...)))...	KCNAB2	NM_001199861	chr1:5974945-6083840	template	39	Intron_1.f	--	Yes	--	Yes	--	--	--	--	--	--	Yes
G4ST01005989002	chr1:5989003-5989038	-	GGGAGCTGAGCTGAGGGGTGGGGGTGGAGTGGGGGG<br>(((...)))...	KCNAB2	NM_001199861	chr1:5974945-6083840	template	36	Intron_1.f	--	Yes	--	Yes	--	--	--	--	--	--	Yes
G4ST01005989014	chr1:5989015-5989048	-	GGGGGGATGTGGGAGCTGAGCTGAGGGGTGGGGG<br>(((...)))...	KCNAB2	NM_001199861	chr1:5974945-6083840	template	34	Intron_1.f	--	Yes	--	Yes	--	--	--	--	--	--	Yes
G4ST01005989020	chr1:5989021-5989057	-	GGGTGGAGTGGGGGGATGTGGGAGCTGAGCTGAGGGG<br>(((...)))...	KCNAB2	NM_001199861	chr1:5974945-6083840	template	37	Intron_1.f	--	Yes	--	Yes	--	--	--	--	--	--	Yes
G4ST01005989084	chr1:5989085-5989117	-	GGGAGCTGAGCTGAGGGGTGAGGGTGGAGTGGG<br>(((...)))...	KCNAB2	NM_001199861	chr1:5974945-6083840	template	33	Intron_1.f	--	Yes	--	Yes	--	--	--	--	--	--	Yes
G4ST01005989093	chr1:5989094-5989127	-	GGGGGGACGTGGGAGCTGAGCTGAGGGGTGAGGG<br>(((...)))...	KCNAB2	NM_001199861	chr1:5974945-6083840	template	34	Intron_1.f	--	Yes	--	Yes	--	--	--	--	--	--	Yes

G4ST01005989099	chr1:5989100-5989136	-	GGGTGCAGTGGGGGGACGTGGGAGCTGAGCTGAGGGG<br>(((...)))...	KCNAB2	NM_001199861	chr1:5974945-6083840	template	37	Intron_1.f	--	Yes	--	Yes	--	--	--	--	--	--	Yes
G4ST01005989159	chr1:5989160-5989190	-	GGGTGATGTGGGAGCTGAGCTGAGGGGTGGG<br>(((...)))...	KCNAB2	NM_001199861	chr1:5974945-6083840	template	31	Intron_1.f	--	Yes	--	Yes	--	--	--	--	--	--	Yes
G4ST01005989163	chr1:5989164-5989200	-	GGGGAGGAGTGGGTGATGTGGGAGCTGAGCTGAGGGG<br>(((...)))...	KCNAB2	NM_001199861	chr1:5974945-6083840	template	37	Intron_1.f	--	Yes	--	Yes	--	--	--	--	--	--	Yes
G4ST01005989187	chr1:5989188-5989220	-	GGGAGCTGAGCTGAGGGGTAGGGGAGGAGTGGG<br>(((...)))...	KCNAB2	NM_001199861	chr1:5974945-6083840	template	33	Intron_1.f	--	Yes	--	Yes	--	--	--	--	--	--	Yes
G4ST01005989196	chr1:5989197-5989230	-	GGGGGGATGTGGGAGCTGAGCTGAGGGGTAGGGG<br>(((...)))...	KCNAB2	NM_001199861	chr1:5974945-6083840	template	34	Intron_1.f	--	Yes	--	Yes	--	--	--	--	--	--	Yes
G4ST01005989202	chr1:5989203-5989239	-	GGGTGAAGTGGGGGGATGTGGGAGCTGAGCTGAGGGG<br>(((...)))...	KCNAB2	NM_001199861	chr1:5974945-6083840	template	37	Intron_1.f	--	Yes	--	Yes	--	--	--	--	--	Yes	Yes
G4ST01005989224	chr1:5989225-5989260	-	GGGAGCTGAGCTGAGGGGTGAGGGTGAAGTGGGGGG<br>(((...)))...	KCNAB2	NM_001199861	chr1:5974945-6083840	template	36	Intron_1.f	--	Yes	--	Yes	--	--	--	--	--	Yes	Yes
G4ST01005989236	chr1:5989237-5989270	-	GGGGGGATGTGGGAGCTGAGCTGAGGGGTGAGGG<br>(((...)))...	KCNAB2	NM_001199861	chr1:5974945-6083840	template	34	Intron_1.f	--	Yes	--	Yes	--	--	--	--	--	Yes	Yes
G4ST01005989242	chr1:5989243-5989279	-	GGGTGGAGTGGGGGGATGTGGGAGCTGAGCTGAGGGG<br>(((...)))...	KCNAB2	NM_001199861	chr1:5974945-6083840	template	37	Intron_1.f	--	Yes	--	Yes	--	--	--	--	--	Yes	Yes
G4ST01005989304	chr1:5989305-5989340	-	GGGAGCTGAGCTGAGGGGTGGAGGGTGAAGTGGGGG<br>(((...)))...	KCNAB2	NM_001199861	chr1:5974945-6083840	template	36	Intron_1.f	--	Yes	--	Yes	--	--	--	--	--	Yes	Yes
G4ST01005989315	chr1:5989316-5989349	-	GGGGGATGTGGGAGCTGAGCTGAGGGGTGGAGGG<br>(((...)))...	KCNAB2	NM_001199861	chr1:5974945-6083840	template	34	Intron_1.f	--	Yes	--	Yes	--	--	--	--	--	Yes	Yes
G4ST01005989322	chr1:5989323-5989358	-	GGGTGAAGTGGGGGATGTGGGAGCTGAGCTGAGGGG<br>(((...)))...	KCNAB2	NM_001199861	chr1:5974945-6083840	template	36	Intron_1.f	--	Yes	--	Yes	--	--	--	--	--	Yes	Yes
G4ST01006032147	chr1:6032148-6032173	-	GGGCATGGCTCCCGGGGGTGGGGGGG<br>..(((...))).	KCNAB2	NM_001199861	chr1:5974945-6083840	template	26	Intron_3	--	Yes	--	Yes	--	--	--	--	--	Yes	--
G4ST01006032147	chr1:6032148-6032173	-	GGGCATGGCTCCCGGGGGTGGGGGGG<br>..(((...))).	KCNAB2	NM_001199863	chr1:6028761-6083840	template	26	Intron_1.f	--	Yes	--	Yes	--	--	--	--	--	Yes	--
G4ST01006032147	chr1:6032148-6032173	-	GGGCATGGCTCCCGGGGGTGGGGGGG<br>..(((...))).	KCNAB2	NM_001199860	chr1:6016935-6083840	template	26	Intron_3	--	Yes	--	Yes	--	--	--	--	--	Yes	--
G4ST01006032147	chr1:6032148-6032173	-	GGGCATGGCTCCCGGGGGTGGGGGGG<br>..(((...))).	KCNAB2	NM_172130	chr1:6008660-6083840	template	26	Intron_2	--	Yes	--	Yes	--	--	--	--	--	Yes	--
G4ST01006032147	chr1:6032148-6032173	-	GGGCATGGCTCCCGGGGGTGGGGGGG<br>..(((...))).	KCNAB2	NM_001199862	chr1:6028568-6083840	template	26	Intron_1.f	--	Yes	--	Yes	--	--	--	--	--	Yes	--
G4ST01006032147	chr1:6032148-6032173	-	GGGCATGGCTCCCGGGGGTGGGGGGG<br>..(((...))).	KCNAB2	NM_003636	chr1:6008660-6083840	template	26	Intron_3	--	Yes	--	Yes	--	--	--	--	--	Yes	--
G4ST01006032778	chr1:6032779-6032806	-	GGGAAGGAGCTGGGAGGGCTATCCTGGG<br>.((...))	KCNAB2	NM_001199861	chr1:5974945-6083840	template	28	Intron_3	--	Yes	--	Yes	--	--	--	--	Yes	--	--
G4ST01006032778	chr1:6032779-6032806	-	GGGAAGGAGCTGGGAGGGCTATCCTGGG<br>.((...))	KCNAB2	NM_001199863	chr1:6028761-6083840	template	28	Intron_1.f	--	Yes	--	Yes	--	--	--	--	Yes	--	--
G4ST01006032778	chr1:6032779-6032806	-	GGGAAGGAGCTGGGAGGGCTATCCTGGG<br>.((...))	KCNAB2	NM_001199860	chr1:6016935-6083840	template	28	Intron_3	--	Yes	--	Yes	--	--	--	--	Yes	--	--
G4ST01006032778	chr1:6032779-6032806	-	GGGAAGGAGCTGGGAGGGCTATCCTGGG<br>.((...))	KCNAB2	NM_172130	chr1:6008660-6083840	template	28	Intron_2	--	Yes	--	Yes	--	--	--	--	Yes	--	--
G4ST01006032778	chr1:6032779-6032806	-	GGGAAGGAGCTGGGAGGGCTATCCTGGG<br>.((...))	KCNAB2	NM_001199862	chr1:6028568-6083840	template	28	Intron_1.f	--	Yes	--	Yes	--	--	--	--	Yes	--	--
G4ST01006032778	chr1:6032779-6032806	-	GGGAAGGAGCTGGGAGGGCTATCCTGGG<br>.((...))	KCNAB2	NM_003636	chr1:6008660-6083840	template	28	Intron_3	--	Yes	--	Yes	--	--	--	--	Yes	--	--
G4ST01006049036	chr1:6049037-6049065	+	GGGCAGTTTGTGCTGAGGGCCGGGAGGGG<br>((((....)))).	KCNAB2	NM_001199861	chr1:5974945-6083840	non-template	29	Intron_3	--	Yes	--	Yes	--	--	--	--	--	--	--
G4ST01006049036	chr1:6049037-6049065	+	GGGCAGTTTGTGCTGAGGGCCGGGAGGGG<br>((((....)))).	KCNAB2	NM_001199863	chr1:6028761-6083840	non-template	29	Intron_1.f	--	Yes	--	Yes	--	--	--	--	--	--	--
G4ST01006049036	chr1:6049037-6049065	+	GGGCAGTTTGTGCTGAGGGCCGGGAGGGG<br>((((....)))).	KCNAB2	NM_001199860	chr1:6016935-6083840	non-template	29	Intron_3	--	Yes	--	Yes	--	--	--	--	--	--	--
G4ST01006049036	chr1:6049037-6049065	+	GGGCAGTTTGTGCTGAGGGCCGGGAGGGG<br>((((....)))).	KCNAB2	NM_172130	chr1:6008660-6083840	non-template	29	Intron_2	--	Yes	--	Yes	--	--	--	--	--	--	--
G4ST01006049036	chr1:6049037-6049065	+	GGGCAGTTTGTGCTGAGGGCCGGGAGGGG<br>((((....)))).	KCNAB2	NM_001199862	chr1:6028568-6083840	non-template	29	Intron_2	--	Yes	--	Yes	--	--	--	--	--	--	--
G4ST01006049036	chr1:6049037-6049065	+	GGGCAGTTTGTGCTGAGGGCCGGGAGGGG<br>((((....)))).	KCNAB2	NM_003636	chr1:6008660-6083840	non-template	29	Intron_3	--	Yes	--	Yes	--	--	--	--	--	--	--
G4ST01006054553	chr1:6054554-6054590	+	GGGCTCCCGGGAGGATCCGGGGACAGGATGCCTGGGG<br>..((((...))))	KCNAB2	NM_001199861	chr1:5974945-6083840	non-template	37	Intron_3	--	Yes	--	Yes	--	--	--	Yes	--	--	--
G4ST01006054553	chr1:6054554-6054590	+	GGGCTCCCGGGAGGATCCGGGGACAGGATGCCTGGGG<br>..((((...))))	KCNAB2	NM_001199863	chr1:6028761-6083840	non-template	37	Intron_1.f	--	Yes	--	Yes	--	--	--	Yes	--	--	--
G4ST01006054553	chr1:6054554-6054590	+	GGGCTCCCGGGAGGATCCGGGGACAGGATGCCTGGGG<br>..((((...))))	KCNAB2	NM_001199860	chr1:6016935-6083840	non-template	37	Intron_3	--	Yes	--	Yes	--	--	--	Yes	--	--	--
G4ST01006054553	chr1:6054554-6054590	+	GGGCTCCCGGGAGGATCCGGGGACAGGATGCCTGGGG<br>..((((...))))	KCNAB2	NM_172130	chr1:6008660-6083840	non-template	37	Intron_2	--	Yes	--	Yes	--	--	--	Yes	--	--	--
G4ST01006054553	chr1:6054554-6054590	+	GGGCTCCCGGGAGGATCCGGGGACAGGATGCCTGGGG<br>..((((...))))	KCNAB2	NM_001199862	chr1:6028568-6083840	non-template	37	Intron_2	--	Yes	--	Yes	--	--	--	Yes	--	--	--
G4ST01006054553	chr1:6054554-6054590	+	GGGCTCCCGGGAGGATCCGGGGACAGGATGCCTGGGG<br>..((((...))))	KCNAB2	NM_003636	chr1:6008660-6083840	non-template	37	Intron_3	--	Yes	--	Yes	--	--	--	Yes	--	--	--
G4ST01006061624	chr1:6061625-6061654	+	GGGTGGGCACCGTGGAGGTGGGGAGACGGG<br>((((.....))))	KCNAB2	NM_001199861	chr1:5974945-6083840	non-template	30	Intron_5	--	Yes	--	Yes	--	--	--	--	--	--	--
G4ST01006061624	chr1:6061625-6061654	+	GGGTGGGCACCGTGGAGGTGGGGAGACGGG<br>((((.....))))	KCNAB2	NM_001199863	chr1:6028761-6083840	non-template	30	Intron_3	--	Yes	--	Yes	--	--	--	--	--	--	--
G4ST01006061624	chr1:6061625-6061654	+	GGGTGGGCACCGTGGAGGTGGGGAGACGGG<br>((((.....))))	KCNAB2	NM_001199860	chr1:6016935-6083840	non-template	30	Intron_5	--	Yes	--	Yes	--	--	--	--	--	--	--
G4ST01006061624	chr1:6061625-6061654	+	GGGTGGGCACCGTGGAGGTGGGGAGACGGG<br>((((.....))))	KCNAB2	NM_172130	chr1:6008660-6083840	non-template	30	Intron_4	--	Yes	--	Yes	--	--	--	--	--	--	--
G4ST01006061624	chr1:6061625-6061654	+	GGGTGGGCACCGTGGAGGTGGGGAGACGGG<br>((((.....))))	KCNAB2	NM_001199862	chr1:6028568-6083840	non-template	30	Intron_4	--	Yes	--	Yes	--	--	--	--	--	--	--
G4ST01006061624	chr1:6061625-6061654	+	GGGTGGGCACCGTGGAGGTGGGGAGACGGG<br>((((.....))))	KCNAB2	NM_003636	chr1:6008660-6083840	non-template	30	Intron_5	--	Yes	--	Yes	--	--	--	--	--	--	--
G4ST01006083555	chr1:6083556-6083600	-	GGGACCTGGGATCTGAGCACAAAGCTGAGTGGGAAACAGTGGGGG<br>..((.(((.....))).)).	KCNAB2	NM_001199861	chr1:5974945-6083840	template	45	Exon_16.l	--	Yes	Yes	--	--	--	Yes	--	--	Yes	--
G4ST01006083555	chr1:6083556-6083600	-	GGGACCTGGGATCTGAGCACAAAGCTGAGTGGGAAACAGTGGGGG<br>..((.(((.....))).)).	KCNAB2	NM_001199863	chr1:6028761-6083840	template	45	Exon_14.l	--	Yes	Yes	--	--	--	Yes	--	--	Yes	--
G4ST01006083555	chr1:6083556-6083600	-	GGGACCTGGGATCTGAGCACAAAGCTGAGTGGGAAACAGTGGGGG<br>..((.(((.....))).)).	KCNAB2	NM_001199860	chr1:6016935-6083840	template	45	Exon_16.l	--	Yes	Yes	--	--	--	Yes	--	--	Yes	--
G4ST01006083555	chr1:6083556-6083600	-	GGGACCTGGGATCTGAGCACAAAGCTGAGTGGGAAACAGTGGGGG<br>..((.(((.....))).)).	KCNAB2	NM_172130	chr1:6008660-6083840	template	45	Exon_15.l	--	Yes	Yes	--	--	--	Yes	--	--	Yes	--
G4ST01006083555	chr1:6083556-6083600	-	GGGACCTGGGATCTGAGCACAAAGCTGAGTGGGAAACAGTGGGGG<br>..((.(((.....))).)).	KCNAB2	NM_001199862	chr1:6028568-6083840	template	45	Exon_16.l	--	Yes	Yes	--	--	--	Yes	--	--	Yes	--
G4ST01006083555	chr1:6083556-6083600	-	GGGACCTGGGATCTGAGCACAAAGCTGAGTGGGAAACAGTGGGGG<br>..((.(((.....))).)).	KCNAB2	NM_003636	chr1:6008660-6083840	template	45	Exon_16.l	--	Yes	Yes	--	--	--	Yes	--	--	Yes	--
G4ST01006085908	chr1:6085909-6085950	-	GGGCACCCAGGAAAGCCTGGCCAGGGCTCCTTTGGGCCTGGG<br>...(((((....)))))...	CHD5	NM_015557	chr1:6084434-6162781	non-template	42	Exon_42.l	--	Yes	Yes	--	--	--	Yes	--	--	--	--
G4ST01006091565	chr1:6091566-6091596	+	GGGATGGAGGGATGAAGGGCTGTGCCAGGGG<br>(((...)))	CHD5	NM_015557	chr1:6084434-6162781	template	31	Intron_38	--	Yes	--	Yes	--	--	--	Yes	Yes	--	--

G4ST01006093218	chr1:6093219-6093251	+	GGGGCCGTAGGGGGAGGCAGCTCAGGGAGGGGG<br>.(((....))).	CHD5	NM_015557	chr1:6084434-6162781	template	33	Intron_36	--	Yes	--	Yes	--	--	--	--	--	--	Yes
G4ST01006093227	chr1:6093228-6093256	+	GGGGGAGGCAGCTCAGGGAGGGGGAGGGG<br>.(((....))).	CHD5	NM_015557	chr1:6084434-6162781	template	29	Intron_36	--	Yes	--	Yes	--	--	--	--	--	--	Yes
G4ST01006118770	chr1:6118771-6118803	+	GGGGGATGGGGCGGTGGGCTCCGGCCACCGGGG<br>...(((...)))	CHD5	NM_015557	chr1:6084434-6162781	template	33	Intron_17	--	Yes	--	Yes	--	--	--	--	--	--	--
G4ST01006129117	chr1:6129118-6129151	-	GGGGCTAGAGGAGCTGGGTAGGGAGATGCAGGGG<br>(((.....))).	CHD5	NM_015557	chr1:6084434-6162781	non-template	34	Intron_10	--	Yes	--	Yes	--	--	--	--	--	--	--
G4ST01006129128	chr1:6129129-6129156	-	GGGCTGGGGCTAGAGGAGCTGGGTAGGG<br>(((.....))).	CHD5	NM_015557	chr1:6084434-6162781	non-template	28	Intron_10	--	Yes	--	Yes	--	--	--	--	--	--	--
G4ST01006129133	chr1:6129134-6129163	-	GGGACCTGGGCTGGGGCTAGAGGAGCTGGG<br>(((.....))).	CHD5	NM_015557	chr1:6084434-6162781	non-template	30	Intron_10	--	Yes	--	Yes	--	--	--	--	--	--	--
G4ST01006129147	chr1:6129148-6129181	-	GGGAGGATTGTCCTGCCTGGGACCTGGGCTGGGG<br>((((...))))....	CHD5	NM_015557	chr1:6084434-6162781	non-template	34	Intron_10	--	Yes	--	Yes	--	--	--	--	--	--	--
G4ST01006131401	chr1:6131402-6131442	+	GGGGCGGGGCCTTTTGAGGAGGGCAGGCCTTCCCCTGGGGG<br>.(((....))). ((((......)))).	CHD5	NM_015557	chr1:6084434-6162781	template	41	Intron_9	--	Yes	--	Yes	--	--	--	Yes	Yes	Yes	--
G4ST01006131406	chr1:6131407-6131447	+	GGGGCCTTTTGAGGAGGGCAGGCCTTCCCCTGGGGGCGGGG<br>.(((....))). ((((......)))).	CHD5	NM_015557	chr1:6084434-6162781	template	41	Intron_9	--	Yes	--	Yes	--	--	--	Yes	Yes	Yes	--
G4ST01006131456	chr1:6131457-6131484	+	GGGGTGGGGCGGGGCGTCCGGCGCGGGG<br>(((.....))).	CHD5	NM_015557	chr1:6084434-6162781	template	28	Intron_9	--	Yes	--	Yes	--	--	--	Yes	Yes	Yes	--
G4ST01006131461	chr1:6131462-6131488	+	GGGGCGGGGCGTCCGGCGCGGGGCGGG<br>(((.....))).	CHD5	NM_015557	chr1:6084434-6162781	template	27	Intron_9	--	Yes	--	Yes	--	--	--	Yes	Yes	Yes	--
G4ST01006137689	chr1:6137690-6137722	-	GGGGCGTTTTGGGGGTGGGCTGTGGTCAGTGGG<br>(((....))).	CHD5	NM_015557	chr1:6084434-6162781	non-template	33	Intron_4	--	Yes	--	Yes	--	--	--	Yes	--	--	--
G4ST01006147811	chr1:6147812-6147843	-	GGGGGCCAGGGGGTCTTTGAGACCTGGGAGGG<br>(((((...))))).	CHD5	NM_015557	chr1:6084434-6162781	non-template	32	Intron_2	--	Yes	--	Yes	--	--	--	--	--	--	--
G4ST01006147815	chr1:6147816-6147850	-	GGGGGCTGGGGGCCAGGGGGTCTTTGAGACCTGGG<br>(((((...))))).	CHD5	NM_015557	chr1:6084434-6162781	non-template	35	Intron_2	--	Yes	--	Yes	--	--	--	--	--	--	--
G4ST01006152029	chr1:6152030-6152060	+	GGGGGCCCTGGCCGGGCAGGCTCGGGAAGGG<br>(((....)))	CHD5	NM_015557	chr1:6084434-6162781	template	31	Intron_1.f	--	Yes	--	Yes	--	--	--	--	--	--	--
G4ST01006155288	chr1:6155289-6155317	-	GGGCTGTTTCAGTGGGAGGGTGCTGGGGG<br>(((...))).	CHD5	NM_015557	chr1:6084434-6162781	non-template	29	Intron_1.f	--	Yes	--	Yes	--	--	--	Yes	Yes	--	--
G4ST01006162492	chr1:6162493-6162528	-	GGGCGGAGCGCGGGCGGCGGGGGCGGCGCGGCCGGG<br>((...)). (((......)))	CHD5	NM_015557	chr1:6084434-6162781	non-template	36	Intron_1.f	--	Yes	--	Yes	--	--	--	--	--	--	--
G4ST01006162505	chr1:6162506-6162535	-	GGGCCCAGGGCGGAGCGCGGGCGGCGGGGG<br>((...)).	CHD5	NM_015557	chr1:6084434-6162781	non-template	30	Intron_1.f	--	Yes	--	Yes	--	--	--	--	--	--	--
G4ST01006162514	chr1:6162515-6162540	-	GGGCCGGGCCCAGGGCGGAGCGCGGG<br>((...)).	CHD5	NM_015557	chr1:6084434-6162781	non-template	26	Intron_1.f	--	Yes	--	Yes	--	--	--	--	--	--	--
G4ST01006198874	chr1:6198875-6198905	+	GGGGGCTTCTGGGTGGGGACAACGTCCTGGG<br>(((...)))..	RNF207	NM_207396	chr1:6188776-6203946	non-template	31	Intron_16	--	Yes	--	Yes	--	--	--	--	--	--	Yes
G4ST01006208462	chr1:6208463-6208490	-	GGGGAGGGCAGGAGACTGGGGAAGTGGG<br>(((....)))	ICMT	NM_012405	chr1:6203840-6218631	non-template	28	Intron_4.l	--	Yes	--	Yes	--	--	--	--	--	Yes	--
G4ST01006208469	chr1:6208470-6208501	-	GGGCACAGCGTGGGGAGGGCAGGAGACTGGGG<br>(((...))) (((....)))	ICMT	NM_012405	chr1:6203840-6218631	non-template	32	Intron_4.l	--	Yes	--	Yes	--	--	--	--	--	--	--
G4ST01006225092	chr1:6225093-6225123	-	GGGCGGGCGGCGCGGCCGGGCACAAAAGGGG<br>.(((...)))	HES3	NM_001024598	chr1:6226839-6228225	template	-1715	upstream	Yes	--	--	--	--	--	--	Yes	Yes	--	--
G4ST01006225103	chr1:6225104-6225128	-	GGGGCGGGCGGGCGGCGCGGCCGGG<br>.(((...)))	HES3	NM_001024598	chr1:6226839-6228225	template	-1710	upstream	Yes	--	--	--	--	--	--	Yes	--	--	--
G4ST01006226543	chr1:6226544-6226577	+	GGGGGTGGGCATGGCAGGGGTCCTCAGACTGGGG<br>(((....)))..	HES3	NM_001024598	chr1:6226839-6228225	template	-261	upstream	Yes	--	--	--	--	--	--	--	--	--	Yes
G4ST01006231206	chr1:6231207-6231241	+	GGGTTCCCAAGGGGGCAGGGGGCACTGAGAGTGGG<br>...(((...)))	GPR153	NM_207370	chr1:6229993-6243622	template	35	Exon_6.l	--	Yes	Yes	--	--	--	Yes	--	--	--	--
G4ST01006231216	chr1:6231217-6231258	+	GGGGGCAGGGGGCACTGAGAGTGGGAGGCCTCAGGCCTAGGG<br>...(((...))) (((((...))))).	GPR153	NM_207370	chr1:6229993-6243622	template	42	Exon_6.l	--	Yes	Yes	--	--	--	Yes	--	--	--	--
G4ST01006231875	chr1:6231876-6231915	+	GGGAGGGGTGGCGCATGTCTGCGCGCGGGGCGGAGGCGGG<br>...(((((....)))))...	GPR153	NM_207370	chr1:6229993-6243622	template	40	Exon_6.l	--	Yes	Yes	--	--	--	Yes	Yes	Yes	Yes	--
G4ST01006232340	chr1:6232341-6232369	+	GGGGGCCGGGCCCGGGGCGGCGGCGCGGG<br>(((....)))	GPR153	NM_207370	chr1:6229993-6243622	template	29	Exon_6.l	--	Yes	Yes	--	Yes	--	--	Yes	--	--	--
G4ST01006232347	chr1:6232348-6232380	+	GGGCCCGGGGCGGCGGCGCGGGCTGCCGGGGGG<br>(((....)))	GPR153	NM_207370	chr1:6229993-6243622	template	33	Exon_6.l	--	Yes	Yes	--	Yes	--	--	Yes	--	--	--
G4ST01006232353	chr1:6232354-6232391	+	GGGGCGGCGGCGCGGGCTGCCGGGGGGCGAGTCGCGGG<br>(((....))) ..(((...)))	GPR153	NM_207370	chr1:6229993-6243622	template	38	Exon_6.l	--	Yes	Yes	--	Yes	--	--	Yes	--	--	--
G4ST01006232814	chr1:6232815-6232850	+	GGGCTGTGTCAGACAGCTGGGGTCGGTTGGGGAGGG<br>((((.....))))...	GPR153	NM_207370	chr1:6229993-6243622	template	36	Intron_5.l	--	Yes	--	Yes	--	--	--	Yes	--	--	--
G4ST01006234335	chr1:6234336-6234383	+	GGGAGGCCCCGCCTTCCGGGAGCTACCTGGCTACTGGGCAGCACTGGG<br>((((...))))... ((((....))))...	GPR153	NM_207370	chr1:6229993-6243622	template	48	Intron_3	--	Yes	--	Yes	--	--	--	--	--	--	--
G4ST01006250023	chr1:6250024-6250068	+	GGGTGTCCTGGGGGGCTGGTGGGAATGGCCCGTCCAAGGCCAGGG<br>..(((((.......)))))	ACOT7	NM_181865	chr1:6246919-6343351	template	45	Intron_8.l	--	Yes	--	Yes	--	--	--	--	--	--	--
G4ST01006250023	chr1:6250024-6250068	+	GGGTGTCCTGGGGGGCTGGTGGGAATGGCCCGTCCAAGGCCAGGG<br>..(((((.......)))))	ACOT7	NM_181864	chr1:6246919-6368470	template	45	Intron_8.l	--	Yes	--	Yes	--	--	--	--	--	--	--
G4ST01006250023	chr1:6250024-6250068	+	GGGTGTCCTGGGGGGCTGGTGGGAATGGCCCGTCCAAGGCCAGGG<br>..(((((.......)))))	ACOT7	NM_181866	chr1:6246919-6341591	template	45	Intron_8.l	--	Yes	--	Yes	--	--	--	--	--	--	--
G4ST01006250023	chr1:6250024-6250068	+	GGGTGTCCTGGGGGGCTGGTGGGAATGGCCCGTCCAAGGCCAGGG<br>..(((((.......)))))	ACOT7	NM_007274	chr1:6246919-6376413	template	45	Intron_8.l	--	Yes	--	Yes	--	--	--	--	--	--	--
G4ST01006257802	chr1:6257803-6257836	-	GGGACTGGAGGGGCAGGGGAGGCTTCCTCTTGGG<br>((((...))))..	ACOT7	NM_181865	chr1:6246919-6343351	non-template	34	Intron_8.l	--	Yes	--	Yes	--	--	--	--	--	--	Yes
G4ST01006257802	chr1:6257803-6257836	-	GGGACTGGAGGGGCAGGGGAGGCTTCCTCTTGGG<br>((((...))))..	ACOT7	NM_181864	chr1:6246919-6368470	non-template	34	Intron_8.l	--	Yes	--	Yes	--	--	--	--	--	--	Yes
G4ST01006257802	chr1:6257803-6257836	-	GGGACTGGAGGGGCAGGGGAGGCTTCCTCTTGGG<br>((((...))))..	ACOT7	NM_181866	chr1:6246919-6341591	non-template	34	Intron_8.l	--	Yes	--	Yes	--	--	--	--	--	--	Yes
G4ST01006257802	chr1:6257803-6257836	-	GGGACTGGAGGGGCAGGGGAGGCTTCCTCTTGGG<br>((((...))))..	ACOT7	NM_007274	chr1:6246919-6376413	non-template	34	Intron_8.l	--	Yes	--	Yes	--	--	--	--	--	--	Yes
G4ST01006259585	chr1:6259586-6259618	-	GGGCCAGCCCTGGGGGAAGGGGCTGCAGAGGGG<br>(((....)))	ACOT7	NM_181865	chr1:6246919-6343351	non-template	33	Intron_8.l	--	Yes	--	Yes	--	--	--	--	--	--	Yes
G4ST01006259585	chr1:6259586-6259618	-	GGGCCAGCCCTGGGGGAAGGGGCTGCAGAGGGG<br>(((....)))	ACOT7	NM_181864	chr1:6246919-6368470	non-template	33	Intron_8.l	--	Yes	--	Yes	--	--	--	--	--	--	Yes
G4ST01006259585	chr1:6259586-6259618	-	GGGCCAGCCCTGGGGGAAGGGGCTGCAGAGGGG<br>(((....)))	ACOT7	NM_181866	chr1:6246919-6341591	non-template	33	Intron_8.l	--	Yes	--	Yes	--	--	--	--	--	--	Yes
G4ST01006259585	chr1:6259586-6259618	-	GGGCCAGCCCTGGGGGAAGGGGCTGCAGAGGGG<br>(((....)))	ACOT7	NM_007274	chr1:6246919-6376413	non-template	33	Intron_8.l	--	Yes	--	Yes	--	--	--	--	--	--	Yes
G4ST01006259939	chr1:6259940-6259974	+	GGGGGCAGCCGGGAGAGGGAACCGTCATTGGTGGG<br>.((((....))))	ACOT7	NM_181865	chr1:6246919-6343351	template	35	Intron_8.l	--	Yes	--	Yes	--	--	--	--	--	--	--
G4ST01006259939	chr1:6259940-6259974	+	GGGGGCAGCCGGGAGAGGGAACCGTCATTGGTGGG<br>.((((....))))	ACOT7	NM_181864	chr1:6246919-6368470	template	35	Intron_8.l	--	Yes	--	Yes	--	--	--	--	--	--	--
G4ST01006259939	chr1:6259940-6259974	+	GGGGGCAGCCGGGAGAGGGAACCGTCATTGGTGGG<br>.((((....))))	ACOT7	NM_181866	chr1:6246919-6341591	template	35	Intron_8.l	--	Yes	--	Yes	--	--	--	--	--	--	--
G4ST01006259939	chr1:6259940-6259974	+	GGGGGCAGCCGGGAGAGGGAACCGTCATTGGTGGG<br>.((((....))))	ACOT7	NM_007274	chr1:6246919-6376413	template	35	Intron_8.l	--	Yes	--	Yes	--	--	--	--	--	--	--
G4ST01006261052	chr1:6261053-6261090	+	GGGGCCCCATGGGGACGGGGCAGTAAGAGCTGTTCGGG<br>.((((....))))...	ACOT7	NM_181865	chr1:6246919-6343351	template	38	Intron_8.l	--	Yes	--	Yes	--	--	--	Yes	--	--	--

G4ST01006261052	chr1:6261053-6261090	+	GGGGCCCCATGGGGACGGGGCAGTAAGAGCTGTTCGGG<br>.((((....)))).. .	ACOT7	NM_181864	chr1:6246919-6368470	template	38	Intron_8.l	--	Yes	--	Yes	--	--	--	Yes	--	--	--
G4ST01006261052	chr1:6261053-6261090	+	GGGGCCCCATGGGGACGGGGCAGTAAGAGCTGTTCGGG<br>.((((....)))).. .	ACOT7	NM_181866	chr1:6246919-6341591	template	38	Intron_8.l	--	Yes	--	Yes	--	--	--	Yes	--	--	--
G4ST01006261052	chr1:6261053-6261090	+	GGGGCCCCATGGGGACGGGGCAGTAAGAGCTGTTCGGG<br>.((((....)))).. .	ACOT7	NM_007274	chr1:6246919-6376413	template	38	Intron_8.l	--	Yes	--	Yes	--	--	--	Yes	--	--	--
G4ST01006266847	chr1:6266848-6266879	+	GGGAAGGGACTGTCCCAGGGGACCTCGGGGGG<br>.(((...)))	ACOT7	NM_181865	chr1:6246919-6343351	template	32	Intron_7	--	Yes	--	Yes	--	--	--	--	--	--	--
G4ST01006266847	chr1:6266848-6266879	+	GGGAAGGGACTGTCCCAGGGGACCTCGGGGGG<br>.(((...)))	ACOT7	NM_181864	chr1:6246919-6368470	template	32	Intron_7	--	Yes	--	Yes	--	--	--	--	--	--	--
G4ST01006266847	chr1:6266848-6266879	+	GGGAAGGGACTGTCCCAGGGGACCTCGGGGGG<br>.(((...)))	ACOT7	NM_181866	chr1:6246919-6341591	template	32	Intron_7	--	Yes	--	Yes	--	--	--	--	--	--	--
G4ST01006266847	chr1:6266848-6266879	+	GGGAAGGGACTGTCCCAGGGGACCTCGGGGGG<br>.(((...)))	ACOT7	NM_007274	chr1:6246919-6376413	template	32	Intron_7	--	Yes	--	Yes	--	--	--	--	--	--	--
G4ST01006272651	chr1:6272652-6272681	+	GGGAAGATTGTTCTGGGGTACCCGGGTGGG<br>.(((....))).	ACOT7	NM_181865	chr1:6246919-6343351	template	30	Intron_7	--	Yes	--	Yes	--	--	--	--	--	--	--
G4ST01006272651	chr1:6272652-6272681	+	GGGAAGATTGTTCTGGGGTACCCGGGTGGG<br>.(((....))).	ACOT7	NM_181864	chr1:6246919-6368470	template	30	Intron_7	--	Yes	--	Yes	--	--	--	--	--	--	--
G4ST01006272651	chr1:6272652-6272681	+	GGGAAGATTGTTCTGGGGTACCCGGGTGGG<br>.(((....))).	ACOT7	NM_181866	chr1:6246919-6341591	template	30	Intron_7	--	Yes	--	Yes	--	--	--	--	--	--	--
G4ST01006272651	chr1:6272652-6272681	+	GGGAAGATTGTTCTGGGGTACCCGGGTGGG<br>.(((....))).	ACOT7	NM_007274	chr1:6246919-6376413	template	30	Intron_7	--	Yes	--	Yes	--	--	--	--	--	--	--
G4ST01006340159	chr1:6340160-6340203	+	GGGTGGGACAGAGGTGTCTGGGCAGACTGTCCCCAGTCATAGGG<br>(((....))).. ..(((((....)))))...	ACOT7	NM_181865	chr1:6246919-6343351	template	44	Intron_1.f	--	Yes	--	Yes	--	--	--	Yes	Yes	--	--
G4ST01006340159	chr1:6340160-6340203	+	GGGTGGGACAGAGGTGTCTGGGCAGACTGTCCCCAGTCATAGGG<br>(((....))).. ..(((((....)))))...	ACOT7	NM_181864	chr1:6246919-6368470	template	44	Intron_1.f	--	Yes	--	Yes	--	--	--	Yes	Yes	--	--
G4ST01006340159	chr1:6340160-6340203	+	GGGTGGGACAGAGGTGTCTGGGCAGACTGTCCCCAGTCATAGGG<br>(((....))).. ..(((((....)))))...	ACOT7	NM_181866	chr1:6246919-6341591	template	44	Intron_1.f	--	Yes	--	Yes	--	--	--	Yes	Yes	--	--
G4ST01006340159	chr1:6340160-6340203	+	GGGTGGGACAGAGGTGTCTGGGCAGACTGTCCCCAGTCATAGGG<br>(((....))).. ..(((((....)))))...	ACOT7	NM_007274	chr1:6246919-6376413	template	44	Intron_1.f	--	Yes	--	Yes	--	--	--	Yes	Yes	--	--
G4ST01006342993	chr1:6342994-6343024	-	GGGCCCCGGCAGGCTGCCTGGGTGGGGAGGG<br>....((((...)))).	ACOT7	NM_181865	chr1:6246919-6343351	non-template	31	Intron_1.f	--	Yes	--	Yes	--	--	--	--	--	--	Yes
G4ST01006342993	chr1:6342994-6343024	-	GGGCCCCGGCAGGCTGCCTGGGTGGGGAGGG<br>....((((...)))).	ACOT7	NM_181864	chr1:6246919-6368470	non-template	31	Intron_1.f	--	Yes	--	Yes	--	--	--	--	--	--	Yes
G4ST01006342993	chr1:6342994-6343024	-	GGGCCCCGGCAGGCTGCCTGGGTGGGGAGGG<br>....((((...)))).	ACOT7	NM_181866	chr1:6246919-6341591	non-template	-1402	upstream	Yes	--	--	--	--	--	--	--	--	--	Yes
G4ST01006342993	chr1:6342994-6343024	-	GGGCCCCGGCAGGCTGCCTGGGTGGGGAGGG<br>....((((...)))).	ACOT7	NM_007274	chr1:6246919-6376413	non-template	31	Intron_1.f	--	Yes	--	Yes	--	--	--	--	--	--	Yes
G4ST01006342997	chr1:6342998-6343030	-	GGGCCTGGGCCCCGGCAGGCTGCCTGGGTGGGG<br>....((((...)))).	ACOT7	NM_181865	chr1:6246919-6343351	non-template	33	Intron_1.f	--	Yes	--	Yes	--	--	--	--	--	--	Yes
G4ST01006342997	chr1:6342998-6343030	-	GGGCCTGGGCCCCGGCAGGCTGCCTGGGTGGGG<br>....((((...)))).	ACOT7	NM_181864	chr1:6246919-6368470	non-template	33	Intron_1.f	--	Yes	--	Yes	--	--	--	--	--	--	Yes
G4ST01006342997	chr1:6342998-6343030	-	GGGCCTGGGCCCCGGCAGGCTGCCTGGGTGGGG<br>....((((...)))).	ACOT7	NM_181866	chr1:6246919-6341591	non-template	-1406	upstream	Yes	--	--	--	--	--	--	--	--	--	Yes
G4ST01006342997	chr1:6342998-6343030	-	GGGCCTGGGCCCCGGCAGGCTGCCTGGGTGGGG<br>....((((...)))).	ACOT7	NM_007274	chr1:6246919-6376413	non-template	33	Intron_1.f	--	Yes	--	Yes	--	--	--	--	--	--	Yes
G4ST01006351149	chr1:6351150-6351193	-	GGGGAAGCAGCATGATTGGGCATGGAGGTGCGGGCAGAGAGGGG<br>.((.((...)).)) (((....))).	ACOT7	NM_181864	chr1:6246919-6368470	non-template	44	Intron_1.f	--	Yes	--	Yes	--	--	--	--	--	--	--
G4ST01006351149	chr1:6351150-6351193	-	GGGGAAGCAGCATGATTGGGCATGGAGGTGCGGGCAGAGAGGGG<br>.((.((...)).)) (((....))).	ACOT7	NM_007274	chr1:6246919-6376413	non-template	44	Intron_1.f	--	Yes	--	Yes	--	--	--	--	--	--	--
G4ST01006351402	chr1:6351403-6351446	+	GGGGGCGGTGCAGTCAGCTGCCCGGGGAATGCAGGGCACAGGGG<br>..(((.((((....)))))))	ACOT7	NM_181864	chr1:6246919-6368470	template	44	Intron_1.f	--	Yes	--	Yes	--	--	--	--	--	--	--
G4ST01006351402	chr1:6351403-6351446	+	GGGGGCGGTGCAGTCAGCTGCCCGGGGAATGCAGGGCACAGGGG<br>..(((.((((....)))))))	ACOT7	NM_007274	chr1:6246919-6376413	template	44	Intron_1.f	--	Yes	--	Yes	--	--	--	--	--	--	--
G4ST01006361431	chr1:6361432-6361462	+	GGGCAGGGGCTGGTGTTCAGCAAGGGGAGGG<br>((((.....)))).. .	ACOT7	NM_181864	chr1:6246919-6368470	template	31	Intron_1.f	--	Yes	--	Yes	--	--	--	--	--	--	--
G4ST01006361431	chr1:6361432-6361462	+	GGGCAGGGGCTGGTGTTCAGCAAGGGGAGGG<br>((((.....)))).. .	ACOT7	NM_007274	chr1:6246919-6376413	template	31	Intron_1.f	--	Yes	--	Yes	--	--	--	--	--	--	--
G4ST01006402190	chr1:6402191-6402220	-	GGGGCGCGGAGCGGGGGCAGGGGGACGGGG<br>.(((...))).	HES2	NM_019089	chr1:6397881-6402566	non-template	30	Intron_2	--	Yes	--	Yes	--	--	--	--	--	--	Yes
G4ST01006402196	chr1:6402197-6402227	-	GGGTCCTGGGGCGCGGAGCGGGGGCAGGGGG<br>.(((...))).	HES2	NM_019089	chr1:6397881-6402566	non-template	31	Intron_2	--	Yes	--	Yes	--	--	--	--	--	--	Yes
G4ST01006402203	chr1:6402204-6402234	-	GGGCAGAGGGTCCTGGGGCGCGGAGCGGGGG<br>.(((...))).	HES2	NM_019089	chr1:6397881-6402566	non-template	31	Intron_2	--	Yes	--	Yes	--	--	--	--	--	--	Yes
G4ST01006402224	chr1:6402225-6402258	-	GGGAGGTGAGCGCGCCCGGGCGGAGGGCAGAGGG<br>.((((....)))).	HES2	NM_019089	chr1:6397881-6402566	non-template	34	Exon_2-Intron_2	--	Yes	Yes	Yes	Yes	--	--	Yes	--	--	Yes
G4ST01006402231	chr1:6402232-6402263	-	GGGCCGGGAGGTGAGCGCGCCCGGGCGGAGGG<br>.((((....)))).	HES2	NM_019089	chr1:6397881-6402566	non-template	32	Exon_2-Intron_2	--	Yes	Yes	Yes	Yes	--	--	Yes	--	--	Yes
G4ST01006406406	chr1:6406407-6406445	+	GGGGCTGGGGCGGAGTGAAACCTTCAGCCCGGGGTGGGG<br>.(((..(((.....)))..)))	ESPN	NM_031475	chr1:6407435-6443591	non-template	-989	upstream	Yes	--	--	--	--	--	--	Yes	Yes	--	--
G4ST01006407469	chr1:6407470-6407505	-	GGGGATCTGCGGGCCGGGAGGCGGAGCGGCTGTGGG<br>((.((...)).))..	ESPN	NM_031475	chr1:6407435-6443591	template	36	Exon_1.f	--	Yes	Yes	--	--	Yes	--	Yes	Yes	Yes	--
G4ST01006408698	chr1:6408699-6408738	+	GGGATTTGGATGAAATTCTGGGTGTGGGGGCAGCCATGGG<br>((((.....)))).. .	ESPN	NM_031475	chr1:6407435-6443591	non-template	40	Intron_1.f	--	Yes	--	Yes	--	--	--	--	--	--	Yes
G4ST01006412650	chr1:6412651-6412684	+	GGGCTCACGCAGGAGGGGCAGCTGGGAATTGGGG<br>(((......)))	MIR4252	NR_036218	chr1:6412481-6412543	template	-107	upstream	Yes	--	--	--	--	--	--	--	Yes	--	Yes
G4ST01006412650	chr1:6412651-6412684	+	GGGCTCACGCAGGAGGGGCAGCTGGGAATTGGGG<br>(((......)))	ESPN	NM_031475	chr1:6407435-6443591	non-template	34	Intron_2	--	Yes	--	Yes	--	--	--	--	Yes	--	Yes
G4ST01006426386	chr1:6426387-6426427	+	GGGGGCCTGTGACACTTCTTAGGCAGGGAGGGGGCATGGGG<br>.(((((.((....)).)))))).	ESPN	NM_031475	chr1:6407435-6443591	non-template	41	Intron_5	--	Yes	--	Yes	--	--	--	--	--	--	--
G4ST01006429844	chr1:6429845-6429871	-	GGGAGGGGCGCACACAGTGGGGAGGGG<br>...(((...)))	ESPN	NM_031475	chr1:6407435-6443591	template	27	Intron_7	--	Yes	--	Yes	--	--	--	--	Yes	--	--
G4ST01006433838	chr1:6433839-6433874	+	GGGAGAGGGCCAGAGGGAGGCTCCACCCCAGCCGGG<br>.((((.......))))	ESPN	NM_031475	chr1:6407435-6443591	non-template	36	Intron_8	--	Yes	--	Yes	--	--	--	Yes	Yes	--	--
G4ST01006434404	chr1:6434405-6434439	-	GGGCCAGGGAAGCCGGGTCGCAGGCTCCTGCTGGG<br>..((((....)))).	ESPN	NM_031475	chr1:6407435-6443591	template	35	Intron_9	--	Yes	--	Yes	--	--	--	--	--	--	--
G4ST01006439394	chr1:6439395-6439443	+	GGGAAGATGGCAACCATGGGGTGAAGGTCCTTCACAGGGGAGCCAGGGG<br>...((((...)))). ((((((...))))))..	ESPN	NM_031475	chr1:6407435-6443591	non-template	49	Intron_10	--	Yes	--	Yes	--	--	--	--	--	--	--
G4ST01006443144	chr1:6443145-6443179	-	GGGGTGCGGGGGAGGGGGCTGCATCCTGGAGGGGG<br>..((.((...)).)).	ESPN	NM_031475	chr1:6407435-6443591	template	35	Exon_13.l	--	Yes	Yes	--	--	--	Yes	Yes	Yes	--	--
G4ST01006444372	chr1:6444373-6444415	+	GGGGACTCTGGCGAGAGTGTGGGCGCAGCAGCGGGGCCTGGGG<br>.(((((....))))).. (((....)))	TNFRSF25	NM_148965	chr1:6443801-6448842	template	43	Intron_9.l-Exon_10.l	--	Yes	Yes	Yes	Yes	--	--	--	Yes	--	--
G4ST01006444372	chr1:6444373-6444415	+	GGGGACTCTGGCGAGAGTGTGGGCGCAGCAGCGGGGCCTGGGG<br>.(((((....))))).. (((....)))	TNFRSF25	NM_003790	chr1:6443801-6448842	template	43	Intron_9.l-Exon_10.l	--	Yes	Yes	Yes	Yes	--	--	--	Yes	--	--
G4ST01006444372	chr1:6444373-6444415	+	GGGGACTCTGGCGAGAGTGTGGGCGCAGCAGCGGGGCCTGGGG<br>.(((((....))))).. (((....)))	TNFRSF25	NM_148970	chr1:6443801-6448842	template	43	Intron_4.l-Exon_5.l	--	Yes	Yes	Yes	Yes	--	--	--	Yes	--	--
G4ST01006444372	chr1:6444373-6444415	+	GGGGACTCTGGCGAGAGTGTGGGCGCAGCAGCGGGGCCTGGGG<br>.(((((....))))).. (((....)))	TNFRSF25	NM_148967	chr1:6443801-6448842	template	43	Intron_8.l-Exon_9.l	--	Yes	Yes	Yes	Yes	--	--	--	Yes	--	--
G4ST01006444372	chr1:6444373-6444415	+	GGGGACTCTGGCGAGAGTGTGGGCGCAGCAGCGGGGCCTGGGG<br>.(((((....))))).. (((....)))	TNFRSF25	NM_148966	chr1:6443801-6448842	template	43	Intron_8.l-Exon_9.l	--	Yes	Yes	Yes	Yes	--	--	--	Yes	--	--

G4ST01006444392	chr1:6444393-6444420	+	GGGCGCAGCAGCGGGGCCTGGGGCAGGG<br>(((....)))	TNFRSF25	NM_148965	chr1:6443801-6448842	template	28	Intron_9.I-Exon_10.I	--	Yes	Yes	Yes	Yes	--	--	--	Yes	--	--
G4ST01006444392	chr1:6444393-6444420	+	GGGCGCAGCAGCGGGGCCTGGGGCAGGG<br>(((....)))	TNFRSF25	NM_003790	chr1:6443801-6448842	template	28	Intron_9.I-Exon_10.I	--	Yes	Yes	Yes	Yes	--	--	--	Yes	--	--
G4ST01006444392	chr1:6444393-6444420	+	GGGCGCAGCAGCGGGGCCTGGGGCAGGG<br>(((....)))	TNFRSF25	NM_148970	chr1:6443801-6448842	template	28	Intron_4.I-Exon_5.I	--	Yes	Yes	Yes	Yes	--	--	--	Yes	--	--
G4ST01006444392	chr1:6444393-6444420	+	GGGCGCAGCAGCGGGGCCTGGGGCAGGG<br>(((....)))	TNFRSF25	NM_148967	chr1:6443801-6448842	template	28	Intron_8.I-Exon_9.I	--	Yes	Yes	Yes	Yes	--	--	--	Yes	--	--
G4ST01006444392	chr1:6444393-6444420	+	GGGCGCAGCAGCGGGGCCTGGGGCAGGG<br>(((....)))	TNFRSF25	NM_148966	chr1:6443801-6448842	template	28	Intron_8.I-Exon_9.I	--	Yes	Yes	Yes	Yes	--	--	--	Yes	--	--
G4ST01006447130	chr1:6447131-6447163	+	GGGACAGGAGTGGGTCAGGCAGGGAGAAGGGGG<br>((....))	TNFRSF25	NM_148965	chr1:6443801-6448842	template	33	Intron_4	--	Yes	--	Yes	--	--	--	--	Yes	--	Yes
G4ST01006447130	chr1:6447131-6447163	+	GGGACAGGAGTGGGTCAGGCAGGGAGAAGGGGG<br>((....))	TNFRSF25	NM_003790	chr1:6443801-6448842	template	33	Intron_4	--	Yes	--	Yes	--	--	--	--	Yes	--	Yes
G4ST01006447130	chr1:6447131-6447163	+	GGGACAGGAGTGGGTCAGGCAGGGAGAAGGGGG<br>((....))	TNFRSF25	NM_148970	chr1:6443801-6448842	template	33	Intron_2	--	Yes	--	Yes	--	--	--	--	Yes	--	Yes
G4ST01006447130	chr1:6447131-6447163	+	GGGACAGGAGTGGGTCAGGCAGGGAGAAGGGGG<br>((....))	TNFRSF25	NM_001039664	chr1:6446758-6448842	template	33	Intron_4.I	--	Yes	--	Yes	--	--	--	--	Yes	--	Yes
G4ST01006447130	chr1:6447131-6447163	+	GGGACAGGAGTGGGTCAGGCAGGGAGAAGGGGG<br>((....))	TNFRSF25	NM_148967	chr1:6443801-6448842	template	33	Intron_3	--	Yes	--	Yes	--	--	--	--	Yes	--	Yes
G4ST01006447130	chr1:6447131-6447163	+	GGGACAGGAGTGGGTCAGGCAGGGAGAAGGGGG<br>((....))	TNFRSF25	NM_148966	chr1:6443801-6448842	template	33	Intron_4	--	Yes	--	Yes	--	--	--	--	Yes	--	Yes
G4ST01006449040	chr1:6449041-6449066	-	GGGTTGGGGCGAGAGTCGGGGTGGGG<br>.(((...)))	TNFRSF25	NM_148965	chr1:6443801-6448842	non-template	-198	upstream	Yes	--	--	--	--	--	--	Yes	Yes	--	--
G4ST01006449040	chr1:6449041-6449066	-	GGGTTGGGGCGAGAGTCGGGGTGGGG<br>.(((...)))	TNFRSF25	NM_003790	chr1:6443801-6448842	non-template	-198	upstream	Yes	--	--	--	--	--	--	Yes	Yes	--	--
G4ST01006449040	chr1:6449041-6449066	-	GGGTTGGGGCGAGAGTCGGGGTGGGG<br>.(((...)))	TNFRSF25	NM_148970	chr1:6443801-6448842	non-template	-198	upstream	Yes	--	--	--	--	--	--	Yes	Yes	--	--
G4ST01006449040	chr1:6449041-6449066	-	GGGTTGGGGCGAGAGTCGGGGTGGGG<br>.(((...)))	TNFRSF25	NM_001039664	chr1:6446758-6448842	non-template	-198	upstream	Yes	--	--	--	--	--	--	Yes	Yes	--	--
G4ST01006449040	chr1:6449041-6449066	-	GGGTTGGGGCGAGAGTCGGGGTGGGG<br>.(((...)))	TNFRSF25	NM_148967	chr1:6443801-6448842	non-template	-198	upstream	Yes	--	--	--	--	--	--	Yes	Yes	--	--
G4ST01006449040	chr1:6449041-6449066	-	GGGTTGGGGCGAGAGTCGGGGTGGGG<br>.(((...)))	TNFRSF25	NM_148966	chr1:6443801-6448842	non-template	-198	upstream	Yes	--	--	--	--	--	--	Yes	Yes	--	--
G4ST01006449040	chr1:6449041-6449066	-	GGGTTGGGGCGAGAGTCGGGGTGGGG<br>.(((...)))	PLEKHG5	NM_198681	chr1:6448739-6502656	non-template	26	Exon_22.I	--	Yes	Yes	--	--	--	Yes	Yes	Yes	--	--
G4ST01006449040	chr1:6449041-6449066	-	GGGTTGGGGCGAGAGTCGGGGTGGGG<br>.(((...)))	PLEKHG5	NM_001042664	chr1:6448739-6473227	non-template	26	Exon_21.I	--	Yes	Yes	--	--	--	Yes	Yes	Yes	--	--
G4ST01006449040	chr1:6449041-6449066	-	GGGTTGGGGCGAGAGTCGGGGTGGGG<br>.(((...)))	PLEKHG5	NM_020631	chr1:6448739-6474347	non-template	26	Exon_21.I	--	Yes	Yes	--	--	--	Yes	Yes	Yes	--	--
G4ST01006449040	chr1:6449041-6449066	-	GGGTTGGGGCGAGAGTCGGGGTGGGG<br>.(((...)))	PLEKHG5	NM_001042663	chr1:6448739-6480071	non-template	26	Exon_22.I	--	Yes	Yes	--	--	--	Yes	Yes	Yes	--	--
G4ST01006449040	chr1:6449041-6449066	-	GGGTTGGGGCGAGAGTCGGGGTGGGG<br>.(((...)))	PLEKHG5	NM_001042665	chr1:6448739-6468601	non-template	26	Exon_21.I	--	Yes	Yes	--	--	--	Yes	Yes	Yes	--	--
G4ST01006452823	chr1:6452824-6452862	+	GGGGCCCAGGAGGTCCCTAGGAAGGGCAGGGCACAGGGG<br>.((.(((.....))).))..	PLEKHG5	NM_198681	chr1:6448739-6502656	template	39	Intron_17	--	Yes	--	Yes	--	--	--	--	--	--	Yes
G4ST01006452823	chr1:6452824-6452862	+	GGGGCCCAGGAGGTCCCTAGGAAGGGCAGGGCACAGGGG<br>.((.(((.....))).))..	PLEKHG5	NM_001042664	chr1:6448739-6473227	template	39	Intron_16	--	Yes	--	Yes	--	--	--	--	--	--	Yes
G4ST01006452823	chr1:6452824-6452862	+	GGGGCCCAGGAGGTCCCTAGGAAGGGCAGGGCACAGGGG<br>.((.(((.....))).))..	PLEKHG5	NM_020631	chr1:6448739-6474347	template	39	Intron_16	--	Yes	--	Yes	--	--	--	--	--	--	Yes
G4ST01006452823	chr1:6452824-6452862	+	GGGGCCCAGGAGGTCCCTAGGAAGGGCAGGGCACAGGGG<br>.((.(((.....))).))..	PLEKHG5	NM_001042663	chr1:6448739-6480071	template	39	Intron_17	--	Yes	--	Yes	--	--	--	--	--	--	Yes
G4ST01006452823	chr1:6452824-6452862	+	GGGGCCCAGGAGGTCCCTAGGAAGGGCAGGGCACAGGGG<br>.((.(((.....))).))..	PLEKHG5	NM_001042665	chr1:6448739-6468601	template	39	Intron_16	--	Yes	--	Yes	--	--	--	--	--	--	Yes
G4ST01006454502	chr1:6454503-6454533	-	GGGGAGCCTGAGGCTGGGTGGGGCCCTAGGG<br>.((((...))))	PLEKHG5	NM_198681	chr1:6448739-6502656	non-template	31	Intron_11	--	Yes	--	Yes	--	--	--	--	--	--	--
G4ST01006454502	chr1:6454503-6454533	-	GGGGAGCCTGAGGCTGGGTGGGGCCCTAGGG<br>.((((...))))	PLEKHG5	NM_001042664	chr1:6448739-6473227	non-template	31	Intron_10	--	Yes	--	Yes	--	--	--	--	--	--	--
G4ST01006454502	chr1:6454503-6454533	-	GGGGAGCCTGAGGCTGGGTGGGGCCCTAGGG<br>.((((...))))	PLEKHG5	NM_020631	chr1:6448739-6474347	non-template	31	Intron_10	--	Yes	--	Yes	--	--	--	--	--	--	--
G4ST01006454502	chr1:6454503-6454533	-	GGGGAGCCTGAGGCTGGGTGGGGCCCTAGGG<br>.((((...))))	PLEKHG5	NM_001042663	chr1:6448739-6480071	non-template	31	Intron_11	--	Yes	--	Yes	--	--	--	--	--	--	--
G4ST01006454502	chr1:6454503-6454533	-	GGGGAGCCTGAGGCTGGGTGGGGCCCTAGGG<br>.((((...))))	PLEKHG5	NM_001042665	chr1:6448739-6468601	non-template	31	Intron_10	--	Yes	--	Yes	--	--	--	--	--	--	--
G4ST01006458627	chr1:6458628-6458671	+	GGGGCTGGTGCGCGGCGGGCATGACCGGGTGGACACGTTCCGGG<br>((((.....)))) .(((.....)))	PLEKHG5	NM_198681	chr1:6448739-6502656	template	44	Exon_4	--	Yes	Yes	--	Yes	--	--	--	--	--	--
G4ST01006458627	chr1:6458628-6458671	+	GGGGCTGGTGCGCGGCGGGCATGACCGGGTGGACACGTTCCGGG<br>((((.....)))) .(((.....)))	PLEKHG5	NM_001042664	chr1:6448739-6473227	template	44	Exon_3	--	Yes	Yes	--	Yes	--	--	--	--	--	--
G4ST01006458627	chr1:6458628-6458671	+	GGGGCTGGTGCGCGGCGGGCATGACCGGGTGGACACGTTCCGGG<br>((((.....)))) .(((.....)))	PLEKHG5	NM_020631	chr1:6448739-6474347	template	44	Exon_3	--	Yes	Yes	--	Yes	--	--	--	--	--	--
G4ST01006458627	chr1:6458628-6458671	+	GGGGCTGGTGCGCGGCGGGCATGACCGGGTGGACACGTTCCGGG<br>((((.....)))) .(((.....)))	PLEKHG5	NM_001042663	chr1:6448739-6480071	template	44	Exon_4	--	Yes	Yes	--	Yes	--	--	--	--	--	--
G4ST01006458627	chr1:6458628-6458671	+	GGGGCTGGTGCGCGGCGGGCATGACCGGGTGGACACGTTCCGGG<br>((((.....)))) .(((.....)))	PLEKHG5	NM_001042665	chr1:6448739-6468601	template	44	Exon_3	--	Yes	Yes	--	Yes	--	--	--	--	--	--
G4ST01006466856	chr1:6466857-6466884	-	GGGATCCAGGGTGGGGTGCGTCATAGGG<br>(((...))).	PLEKHG5	NM_198681	chr1:6448739-6502656	non-template	28	Intron_2	--	Yes	--	Yes	--	--	--	--	--	--	--
G4ST01006466856	chr1:6466857-6466884	-	GGGATCCAGGGTGGGGTGCGTCATAGGG<br>(((...))).	PLEKHG5	NM_001042664	chr1:6448739-6473227	non-template	28	Intron_1.f	--	Yes	--	Yes	--	--	--	--	--	--	--
G4ST01006466856	chr1:6466857-6466884	-	GGGATCCAGGGTGGGGTGCGTCATAGGG<br>(((...))).	PLEKHG5	NM_020631	chr1:6448739-6474347	non-template	28	Intron_1.f	--	Yes	--	Yes	--	--	--	--	--	--	--
G4ST01006466856	chr1:6466857-6466884	-	GGGATCCAGGGTGGGGTGCGTCATAGGG<br>(((...))).	PLEKHG5	NM_001042663	chr1:6448739-6480071	non-template	28	Intron_2	--	Yes	--	Yes	--	--	--	--	--	--	--
G4ST01006466856	chr1:6466857-6466884	-	GGGATCCAGGGTGGGGTGCGTCATAGGG<br>(((...))).	PLEKHG5	NM_001042665	chr1:6448739-6468601	non-template	28	Intron_1.f	--	Yes	--	Yes	--	--	--	--	--	--	--
G4ST01006468394	chr1:6468395-6468430	-	GGGCGGAGGCGGGGCTGTGGGGGTCCCCCGGCCGGG<br>((((....))))	PLEKHG5	NM_198681	chr1:6448739-6502656	non-template	36	Intron_2	--	Yes	--	Yes	--	--	--	--	--	--	Yes
G4ST01006468394	chr1:6468395-6468430	-	GGGCGGAGGCGGGGCTGTGGGGGTCCCCCGGCCGGG<br>((((....))))	PLEKHG5	NM_001042664	chr1:6448739-6473227	non-template	36	Intron_1.f	--	Yes	--	Yes	--	--	--	--	--	--	Yes
G4ST01006468394	chr1:6468395-6468430	-	GGGCGGAGGCGGGGCTGTGGGGGTCCCCCGGCCGGG<br>((((....))))	PLEKHG5	NM_020631	chr1:6448739-6474347	non-template	36	Intron_1.f	--	Yes	--	Yes	--	--	--	--	--	--	Yes
G4ST01006468394	chr1:6468395-6468430	-	GGGCGGAGGCGGGGCTGTGGGGGTCCCCCGGCCGGG<br>((((....))))	PLEKHG5	NM_001042663	chr1:6448739-6480071	non-template	36	Intron_2	--	Yes	--	Yes	--	--	--	--	--	--	Yes
G4ST01006468394	chr1:6468395-6468430	-	GGGCGGAGGCGGGGCTGTGGGGGTCCCCCGGCCGGG<br>((((....))))	PLEKHG5	NM_001042665	chr1:6448739-6468601	non-template	36	Intron_1.f	--	Yes	--	Yes	--	--	--	--	--	--	Yes
G4ST01006474895	chr1:6474896-6474930	-	GGGCCACAGGGGACATTGGGCACCACATGTGAGGG<br>(((.....))).	PLEKHG5	NM_198681	chr1:6448739-6502656	non-template	35	Intron_2	--	Yes	--	Yes	--	--	--	--	Yes	--	--
G4ST01006474895	chr1:6474896-6474930	-	GGGCCACAGGGGACATTGGGCACCACATGTGAGGG<br>(((.....))).	PLEKHG5	NM_001042664	chr1:6448739-6473227	non-template	-1668	upstream	Yes	--	--	--	--	--	--	--	Yes	--	--
G4ST01006474895	chr1:6474896-6474930	-	GGGCCACAGGGGACATTGGGCACCACATGTGAGGG<br>(((.....))).	PLEKHG5	NM_020631	chr1:6448739-6474347	non-template	-548	upstream	Yes	--	--	--	--	--	--	--	Yes	--	--

G4ST01006474895	chr1:6474896-6474930	-	GGGCCACAGGGGACATTGGGCACCACATGTGAGGG<br>(((.....))).	PLEKHG5	NM_001042663	chr1:6448739-6480071	non-template	35	Intron_2	--	Yes	--	Yes	--	--	--	--	Yes	--	--
G4ST01006476688	chr1:6476689-6476724	-	GGGGAGGGTGAGGTGGGAGGATCATTTGAGCCTGGG<br>(((.((....)).)))	PLEKHG5	NM_198681	chr1:6448739-6502656	non-template	36	Intron_2	--	Yes	--	Yes	--	--	--	--	--	--	--
G4ST01006476688	chr1:6476689-6476724	-	GGGGAGGGTGAGGTGGGAGGATCATTTGAGCCTGGG<br>(((.((....)).)))	PLEKHG5	NM_001042663	chr1:6448739-6480071	non-template	36	Intron_2	--	Yes	--	Yes	--	--	--	--	--	--	--
G4ST01006479096	chr1:6479097-6479136	-	GGGAGGCTGGAGAGCCTGGGGACCCTCGGGGGCAGAGGGG<br>(((((....))))).	PLEKHG5	NM_198681	chr1:6448739-6502656	non-template	40	Intron_2	--	Yes	--	Yes	--	--	--	--	--	--	Yes
G4ST01006479096	chr1:6479097-6479136	-	GGGAGGCTGGAGAGCCTGGGGACCCTCGGGGGCAGAGGGG<br>(((((....))))).	PLEKHG5	NM_001042663	chr1:6448739-6480071	non-template	40	Intron_2	--	Yes	--	Yes	--	--	--	--	--	--	Yes
G4ST01006481220	chr1:6481221-6481254	+	GGGGGCCTGGTGGGCAGGGTAGCCCTTTGGGGGG<br>...(((...)))	PLEKHG5	NM_198681	chr1:6448739-6502656	template	34	Intron_1.f	--	Yes	--	Yes	--	--	--	--	--	--	--
G4ST01006481220	chr1:6481221-6481254	+	GGGGGCCTGGTGGGCAGGGTAGCCCTTTGGGGGG<br>...(((...)))	PLEKHG5	NM_001042663	chr1:6448739-6480071	template	-1149	upstream	Yes	--	--	--	--	--	--	--	--	--	--
G4ST01006485449	chr1:6485450-6485486	-	GGGTGGGTGAGCCTGGGCACACGGCTGTCTGTGTGGG<br>.((((((....))))))	PLEKHG5	NM_198681	chr1:6448739-6502656	non-template	37	Intron_1.f	--	Yes	--	Yes	--	--	--	--	--	--	--
G4ST01006508952	chr1:6508953-6508982	-	GGGACTGCGAGTGGGCTCTGGGAACGGGGG<br>(((...)))	NOL9	NM_024654	chr1:6503994-6537245	non-template	30	Intron_11.l	--	Yes	--	Yes	--	--	--	--	Yes	--	--
G4ST01006536864	chr1:6536865-6536892	+	GGGTTCGGAGGCCGGGGTCGGGCTAGGG<br>..(((...)))	NOL9	NM_024654	chr1:6503994-6537245	template	28	Exon_1.f	--	Yes	Yes	--	Yes	--	--	Yes	Yes	Yes	Yes
G4ST01006536864	chr1:6536865-6536892	+	GGGTTCGGAGGCCGGGGTCGGGCTAGGG<br>..(((...)))	TAS1R1	NM_138697	chr1:6537925-6562404	non-template	-1032	upstream	Yes	--	--	--	--	--	--	Yes	Yes	Yes	Yes
G4ST01006536864	chr1:6536865-6536892	+	GGGTTCGGAGGCCGGGGTCGGGCTAGGG<br>..(((...)))	TAS1R1	NM_177540	chr1:6537925-6562404	non-template	-1032	upstream	Yes	--	--	--	--	--	--	Yes	Yes	Yes	Yes
G4ST01006554728	chr1:6554729-6554759	-	GGGGCAGGGAGTCAGTGCTCAGGGTAGAGGG<br>(((....)))..	TAS1R1	NM_138697	chr1:6537925-6562404	template	31	Intron_2	--	Yes	--	Yes	--	--	--	--	--	--	--
G4ST01006554728	chr1:6554729-6554759	-	GGGGCAGGGAGTCAGTGCTCAGGGTAGAGGG<br>(((....)))..	TAS1R1	NM_177540	chr1:6537925-6562404	template	31	Intron_2	--	Yes	--	Yes	--	--	--	--	--	--	--
G4ST01006554735	chr1:6554736-6554764	-	GGGAAGGGGCAGGGAGTCAGTGCTCAGGG<br>(((....)))..	TAS1R1	NM_138697	chr1:6537925-6562404	template	29	Intron_2	--	Yes	--	Yes	--	--	--	--	--	--	--
G4ST01006554735	chr1:6554736-6554764	-	GGGAAGGGGCAGGGAGTCAGTGCTCAGGG<br>(((....)))..	TAS1R1	NM_177540	chr1:6537925-6562404	template	29	Intron_2	--	Yes	--	Yes	--	--	--	--	--	--	--
G4ST01006557099	chr1:6557100-6557125	-	GGGGCAGGTTCTGGGGGAGGGGTGGG<br>.(((...))).	TAS1R1	NM_138697	chr1:6537925-6562404	template	26	Intron_2	--	Yes	--	Yes	--	--	--	--	--	--	--
G4ST01006557099	chr1:6557100-6557125	-	GGGGCAGGTTCTGGGGGAGGGGTGGG<br>.(((...))).	TAS1R1	NM_177540	chr1:6537925-6562404	template	26	Intron_2	--	Yes	--	Yes	--	--	--	--	--	--	--
G4ST01006562767	chr1:6562768-6562800	+	GGGAGGACCTGCCTGGGTGAGGAGGGCGCGGGG<br>(((.....)))	ZBTB48	NM_005341	chr1:6562650-6571927	non-template	33	Exon_1.f-Intron_1.f	--	Yes	Yes	Yes	--	Yes	--	--	Yes	Yes	Yes
G4ST01006571859	chr1:6571860-6571896	-	GGGGTGGGGCCAAGTGGGCTCTGGTGGCCAGAATGGG<br>..((((...))))...	ZBTB48	NM_005341	chr1:6562650-6571927	template	37	Exon_11.l	--	Yes	Yes	--	--	--	Yes	Yes	Yes	Yes	--
G4ST01006582130	chr1:6582131-6582164	-	GGGAGGGTGCAGGTGGGTGACTCACCTGAGTGGG<br>..((((....))))	KLHL21	NM_014851	chr1:6573371-6585516	non-template	34	Intron_1.f	--	Yes	--	Yes	--	--	--	Yes	Yes	--	--
G4ST01006584408	chr1:6584409-6584437	-	GGGCCAGGGGCTGGGCAGGCTCTCTGGGG<br>(((.....)))	KLHL21	NM_014851	chr1:6573371-6585516	non-template	29	Intron_1.f	--	Yes	--	Yes	--	--	--	Yes	Yes	Yes	Yes
G4ST01006586276	chr1:6586277-6586303	-	GGGAGGCCCGGGAGCCCCTAGGGCGGG<br>((...)).	KLHL21	NM_014851	chr1:6573371-6585516	non-template	-760	upstream	Yes	--	--	--	--	--	--	Yes	Yes	Yes	--
G4ST01006596103	chr1:6596104-6596149	-	GGGGGAGGTGCGGGGGCCCGCGCCGGCTCGGCCGCGGGGGGAGGGG<br>..((((((((((...))).)))))))	PHF13	NM_153812	chr1:6596343-6606680	template	-193	upstream	Yes	--	--	--	--	--	--	Yes	Yes	Yes	--
G4ST01006597391	chr1:6597392-6597430	-	GGGGCGGAGCCAGCAAAGCCCGCGGGCGGGGGGCGGGGG<br>((((.((.......))))))	PHF13	NM_153812	chr1:6596343-6606680	template	39	Intron_1.f	--	Yes	--	Yes	--	--	--	Yes	--	--	--
G4ST01006597517	chr1:6597518-6597549	+	GGGGTCGGGGGTGCGGGTCCGGCGACCGCGGG<br>..(((...))).	PHF13	NM_153812	chr1:6596343-6606680	non-template	32	Intron_1.f	--	Yes	--	Yes	--	--	--	Yes	Yes	--	Yes
G4ST01006597523	chr1:6597524-6597553	+	GGGGGTGCGGGTCCGGCGACCGCGGGCGGG<br>..(((...))).	PHF13	NM_153812	chr1:6596343-6606680	non-template	30	Intron_1.f	--	Yes	--	Yes	--	--	--	Yes	Yes	--	Yes
G4ST01006597531	chr1:6597532-6597559	+	GGGTCCGGCGACCGCGGGCGGGTGGGGG<br>..(((...))).	PHF13	NM_153812	chr1:6596343-6606680	non-template	28	Intron_1.f	--	Yes	--	Yes	--	--	--	Yes	Yes	--	Yes
G4ST01006597711	chr1:6597712-6597738	+	GGGGGGCGGCTGCCGCGGGAGGGCGGG<br>..((((...))))	PHF13	NM_153812	chr1:6596343-6606680	non-template	27	Intron_1.f	--	Yes	--	Yes	--	--	--	--	--	--	Yes
G4ST01006612148	chr1:6612149-6612187	-	GGGGGTTTGAGGGTCCAGAAGGGCTTCCTGAAGGAAGGG<br>.((((....))))	THAP3	NM_001195753	chr1:6607512-6616229	template	39	Intron_3	--	Yes	--	Yes	--	--	--	--	--	--	--
G4ST01006612148	chr1:6612149-6612187	-	GGGGGTTTGAGGGTCCAGAAGGGCTTCCTGAAGGAAGGG<br>.((((....))))	THAP3	NM_001195752	chr1:6607797-6616229	template	39	Intron_2	--	Yes	--	Yes	--	--	--	--	--	--	--
G4ST01006612148	chr1:6612149-6612187	-	GGGGGTTTGAGGGTCCAGAAGGGCTTCCTGAAGGAAGGG<br>.((((....))))	THAP3	NM_138350	chr1:6607797-6618233	template	39	Intron_2	--	Yes	--	Yes	--	--	--	--	--	--	--
G4ST01006768542	chr1:6768543-6768575	-	GGGAGCGGCGGGGATGCCCGGTCCGGGTGGGGG<br>(((.....))).	CAMTA1	NM_001195563	chr1:6767971-6854694	template	33	Intron_1.f	--	Yes	--	Yes	--	--	--	Yes	Yes	Yes	--
G4ST01006768542	chr1:6768543-6768575	-	GGGAGCGGCGGGGATGCCCGGTCCGGGTGGGGG<br>(((.....))).	CAMTA1	NM_015215	chr1:6767971-7752353	template	33	Intron_1.f	--	Yes	--	Yes	--	--	--	Yes	Yes	Yes	--
G4ST01006768548	chr1:6768549-6768581	-	GGGCTCGGGAGCGGCGGGGATGCCCGGTCCGGG<br>(((.....))).	CAMTA1	NM_001195563	chr1:6767971-6854694	template	33	Intron_1.f	--	Yes	--	Yes	--	--	--	Yes	Yes	Yes	--
G4ST01006768548	chr1:6768549-6768581	-	GGGCTCGGGAGCGGCGGGGATGCCCGGTCCGGG<br>(((.....))).	CAMTA1	NM_015215	chr1:6767971-7752353	template	33	Intron_1.f	--	Yes	--	Yes	--	--	--	Yes	Yes	Yes	--
G4ST01006768609	chr1:6768610-6768655	+	GGGTCCACGGTGGGGGCCGCGGGGCGGAAAGTTTATCCGCCTCGGG<br>.(((...))) (((((.......)))))...	CAMTA1	NM_001195563	chr1:6767971-6854694	non-template	46	Intron_1.f	--	Yes	--	Yes	--	--	--	--	Yes	Yes	--
G4ST01006768609	chr1:6768610-6768655	+	GGGTCCACGGTGGGGGCCGCGGGGCGGAAAGTTTATCCGCCTCGGG<br>.(((...))) (((((.......)))))...	CAMTA1	NM_015215	chr1:6767971-7752353	non-template	46	Intron_1.f	--	Yes	--	Yes	--	--	--	--	Yes	Yes	--
G4ST01006888176	chr1:6888177-6888205	-	GGGAAGCGGGGCTGGTGGCAGGGGTGGGG<br>.(((....)))	CAMTA1	NM_015215	chr1:6767971-7752353	template	29	Intron_3	--	Yes	--	Yes	--	--	--	--	--	--	--
G4ST01006890557	chr1:6890558-6890585	-	GGGCTGGGAGGCCTCTGGGTTTTGTGGG<br>((....))	CAMTA1	NM_015215	chr1:6767971-7752353	template	28	Intron_3	--	Yes	--	Yes	--	--	--	--	--	--	--
G4ST01006969618	chr1:6969619-6969654	-	GGGGGCGGAGGGGCTGGGGAACCTGGCCCTAGGGGG<br>...((((....))))	CAMTA1	NM_015215	chr1:6767971-7752353	template	36	Intron_3	--	Yes	--	Yes	--	--	--	--	--	--	Yes
G4ST01007027070	chr1:7027071-7027104	+	GGGGACCCCGGGAAAGCAGGGCAGTGCCACTGGG<br>.(((...)))	CAMTA1	NM_015215	chr1:6767971-7752353	non-template	34	Intron_3	--	Yes	--	Yes	--	--	--	--	--	--	--
G4ST01007035218	chr1:7035219-7035260	-	GGGGAAAGGGGCCCGATGCAGAAGGCACGTGGGCGAGGAGGG<br>...((.(((.....))))).	CAMTA1	NM_015215	chr1:6767971-7752353	template	42	Intron_3	--	Yes	--	Yes	--	--	--	--	--	--	--
G4ST01007066982	chr1:7066983-7067012	+	GGGCTGCTCCAGGGGAAGCTGTGGGAGGGG<br>(((...)))	CAMTA1	NM_015215	chr1:6767971-7752353	non-template	30	Intron_3	--	Yes	--	Yes	--	--	--	Yes	--	--	--
G4ST01007070122	chr1:7070123-7070156	+	GGGTCAGTGGGGAGCAGGGAGCTGTCAGCCTGGG<br>.(((...)))..	CAMTA1	NM_015215	chr1:6767971-7752353	non-template	34	Intron_3	--	Yes	--	Yes	--	--	--	--	--	--	--
G4ST01007070130	chr1:7070131-7070161	+	GGGGAGCAGGGAGCTGTCAGCCTGGGCTGGG<br>.(((...)))..	CAMTA1	NM_015215	chr1:6767971-7752353	non-template	31	Intron_3	--	Yes	--	Yes	--	--	--	--	--	--	--
G4ST01007080497	chr1:7080498-7080531	+	GGGAGGGAGAAATGGGGAGTTAGTGTTTAATGGG<br>..((((.....))))	CAMTA1	NM_015215	chr1:6767971-7752353	non-template	34	Intron_4	--	Yes	--	Yes	--	--	--	--	--	--	Yes
G4ST01007080501	chr1:7080502-7080550	+	GGGAGAAATGGGGAGTTAGTGTTTAATGGGTACAGCGTTTCAGCTGGGG<br>..((((.....)))) ..((((......))))	CAMTA1	NM_015215	chr1:6767971-7752353	non-template	49	Intron_4	--	Yes	--	Yes	--	--	--	--	--	--	Yes
G4ST01007080510	chr1:7080511-7080558	+	GGGGAGTTAGTGTTTAATGGGTACAGCGTTTCAGCTGGGGAAGATGGG<br>..((((.....)))) ..((((......))))	CAMTA1	NM_015215	chr1:6767971-7752353	non-template	48	Intron_4	--	Yes	--	Yes	--	--	--	--	--	--	Yes
G4ST01007082454	chr1:7082455-7082484	+	GGGCGAGGGGCTGGGAATGTCAGCATTGGG<br>((((....))))	CAMTA1	NM_015215	chr1:6767971-7752353	non-template	30	Intron_4	--	Yes	--	Yes	--	--	--	--	--	--	--

G4ST01007106270	chr1:7106271-7106314	-	GGGCTCAGAGGGGCCAAGGTACTACCCAGGCTGGGTGCCCAGGG<br>(((..(((...)))..))).	CAMTA1	NM_015215	chr1:6767971-7752353	template	44	Intron_4	--	Yes	--	Yes	--	--	--	--	--	--	--
G4ST01007157378	chr1:7157379-7157420	+	GGGGGGCCTGGGAATTGGGAGGAGTTAGAGCTGCTTCTAGGG<br>((.(((....))).)))....	CAMTA1	NM_015215	chr1:6767971-7752353	non-template	42	Intron_4	--	Yes	--	Yes	--	--	--	--	--	--	--
G4ST01007159755	chr1:7159756-7159782	-	GGGAGGAGCCTCCATGGGTGGGAGGGG<br>.(((...)))..	CAMTA1	NM_015215	chr1:6767971-7752353	template	27	Intron_4	--	Yes	--	Yes	--	--	--	Yes	--	--	--
G4ST01007171096	chr1:7171097-7171127	-	GGGGAGAGGGAGGGAGTTACTGTTTAATGGG<br>.((((.....))))	CAMTA1	NM_015215	chr1:6767971-7752353	template	31	Intron_4	--	Yes	--	Yes	--	--	--	--	--	--	Yes
G4ST01007201203	chr1:7201204-7201238	-	GGGGCAGCATTGGCAAATGTGCAGGGGAGGGAGGG<br>(((.((((....)))))))..	CAMTA1	NM_015215	chr1:6767971-7752353	template	35	Intron_4	--	Yes	--	Yes	--	--	--	--	Yes	--	--
G4ST01007208173	chr1:7208174-7208209	+	GGGGTTGTGGGTGGGGTGTTGCTGAGAACACCTGGG<br>(((((......)))))..	CAMTA1	NM_015215	chr1:6767971-7752353	non-template	36	Intron_4	--	Yes	--	Yes	--	--	--	--	--	--	--
G4ST01007208181	chr1:7208182-7208213	+	GGGTGGGGTGTTGCTGAGAACACCTGGGAGGG<br>(((((......)))))..	CAMTA1	NM_015215	chr1:6767971-7752353	non-template	32	Intron_4	--	Yes	--	Yes	--	--	--	--	--	--	--
G4ST01007210479	chr1:7210480-7210507	-	GGGTGAGGAGTCCTGGGGAGGGGAGGGG<br>..(((...))).	CAMTA1	NM_015215	chr1:6767971-7752353	template	28	Intron_4	--	Yes	--	Yes	--	--	--	--	--	--	--
G4ST01007210484	chr1:7210485-7210515	-	GGGGCCCTGGGTGAGGAGTCCTGGGGAGGGG<br>..(((...))).	CAMTA1	NM_015215	chr1:6767971-7752353	template	31	Intron_4	--	Yes	--	Yes	--	--	--	--	--	--	--
G4ST01007263000	chr1:7263001-7263039	-	GGGACAGGAGGGTCTTACCCTATAAGCAGGGAGCTTGGG<br>.((((.....))))..	CAMTA1	NM_015215	chr1:6767971-7752353	template	39	Intron_5	--	Yes	--	Yes	--	--	--	--	Yes	--	--
G4ST01007318787	chr1:7318788-7318827	+	GGGGAGGGATGCATGGGGACAGTGTTGTTCACAGTGTGGG<br>.(((.(((.....))).)))	CAMTA1	NM_015215	chr1:6767971-7752353	non-template	40	Intron_5	--	Yes	--	Yes	--	--	--	--	Yes	--	Yes
G4ST01007332590	chr1:7332591-7332623	+	GGGAACTTCTGGCAGAAGGGAAGAGGGATGGGG<br>...((((...))))	CAMTA1	NM_015215	chr1:6767971-7752353	non-template	33	Intron_5	--	Yes	--	Yes	--	--	--	--	--	--	--
G4ST01007334915	chr1:7334916-7334950	+	GGGAACCTGGGCTTCCTGGGTCCTTTCCAAAGGGG<br>..(((....)))	CAMTA1	NM_015215	chr1:6767971-7752353	non-template	35	Intron_5	--	Yes	--	Yes	--	--	--	--	--	--	--
G4ST01007370136	chr1:7370137-7370175	-	GGGGTCTAGGGCAGCTGGGACCCTGCTTGCTGGCAGGGG<br>...(((((....)))))	CAMTA1	NM_015215	chr1:6767971-7752353	template	39	Intron_5	--	Yes	--	Yes	--	--	--	Yes	--	--	--
G4ST01007386805	chr1:7386806-7386838	-	GGGACAAGAGTGTGGGTGGTGTCGGGAGTGGGG<br>(((....)))	CAMTA1	NM_015215	chr1:6767971-7752353	template	33	Intron_5	--	Yes	--	Yes	--	--	--	Yes	--	--	--
G4ST01007386812	chr1:7386813-7386848	-	GGGCCATGGAGGGACAAGAGTGTGGGTGGTGTCGGG<br>(((....)))	CAMTA1	NM_015215	chr1:6767971-7752353	template	36	Intron_5	--	Yes	--	Yes	--	--	--	--	--	--	--
G4ST01007391182	chr1:7391183-7391215	+	GGGACAGGGTAGGGAGGAGGAAGCCACCCTGGG<br>.((.((...)).))..	CAMTA1	NM_015215	chr1:6767971-7752353	non-template	33	Intron_5	--	Yes	--	Yes	--	--	--	--	Yes	--	--
G4ST01007419183	chr1:7419184-7419217	+	GGGCCCCAGGGAACCTGAGGTGGGAGGAGCAGGG<br>.(((...)))	CAMTA1	NM_015215	chr1:6767971-7752353	non-template	34	Intron_5	--	Yes	--	Yes	--	--	--	--	--	--	--
G4ST01007420831	chr1:7420832-7420867	+	GGGCTGCCACCTCCAGGCAGCTGGGCAGGGCCTGGG<br>(((((.......)))))..	CAMTA1	NM_015215	chr1:6767971-7752353	non-template	36	Intron_5	--	Yes	--	Yes	--	--	--	--	--	--	--
G4ST01007421758	chr1:7421759-7421795	-	GGGAGCAGCAGGGCCTGGAGGGGCCCTCGGCCCTGGG<br>(((...)))...	CAMTA1	NM_015215	chr1:6767971-7752353	template	37	Intron_5	--	Yes	--	Yes	--	--	--	--	--	--	--
G4ST01007426300	chr1:7426301-7426337	+	GGGGTAGAGGGAGGCCCAGAGCCTGGGGCATTCTGGG<br>((((.....)))).	CAMTA1	NM_015215	chr1:6767971-7752353	non-template	37	Intron_5	--	Yes	--	Yes	--	--	--	--	--	--	--
G4ST01007428036	chr1:7428037-7428082	+	GGGAGCTGTGGTCTGAGCTGGGTCTGAAGGGCTGGTCAGCCGGGGG<br>((((........)))) ((((....))))	CAMTA1	NM_015215	chr1:6767971-7752353	non-template	46	Intron_5	--	Yes	--	Yes	--	--	--	--	--	--	--
G4ST01007437375	chr1:7437376-7437409	+	GGGCGGGAGGCCTCAGTCAGCCTGGGTCTGAGGG<br>((((........))))	CAMTA1	NM_015215	chr1:6767971-7752353	non-template	34	Intron_5	--	Yes	--	Yes	--	--	--	--	--	--	--
G4ST01007437700	chr1:7437701-7437736	+	GGGCCCTGGGGGCATCCAGGGCCGGCCTGGCTGGGG<br>.((((...))))	CAMTA1	NM_015215	chr1:6767971-7752353	non-template	36	Intron_5	--	Yes	--	Yes	--	--	--	Yes	--	--	--
G4ST01007443914	chr1:7443915-7443949	-	GGGGCCACGATCTGGGGGAGTCAGGGCTTCAAGGG<br>.(((.....)))	CAMTA1	NM_015215	chr1:6767971-7752353	template	35	Intron_5	--	Yes	--	Yes	--	--	--	--	--	--	--
G4ST01007443923	chr1:7443924-7443958	-	GGGACCCCAGGGGCCACGATCTGGGGGAGTCAGGG<br>.(((.....)))	CAMTA1	NM_015215	chr1:6767971-7752353	template	35	Intron_5	--	Yes	--	Yes	--	--	--	--	--	--	--
G4ST01007443931	chr1:7443932-7443966	-	GGGAGCTTGGGACCCCAGGGGCCACGATCTGGGGG<br>.(((.....)))	CAMTA1	NM_015215	chr1:6767971-7752353	template	35	Intron_5	--	Yes	--	Yes	--	--	--	--	--	--	--
G4ST01007445741	chr1:7445742-7445775	+	GGGGCCGGGGGTTCATGTGAGCAGGGACACGGGG<br>.((((....)))).	CAMTA1	NM_015215	chr1:6767971-7752353	non-template	34	Intron_5	--	Yes	--	Yes	--	--	--	--	--	--	--
G4ST01007445747	chr1:7445748-7445779	+	GGGGGTTCATGTGAGCAGGGACACGGGGAGGG<br>.((((....)))).	CAMTA1	NM_015215	chr1:6767971-7752353	non-template	32	Intron_5	--	Yes	--	Yes	--	--	--	--	--	--	--
G4ST01007463754	chr1:7463755-7463785	-	GGGTAGGAGGGAGCCGTGGCTTAGGGTTGGG<br>(((....)))..	CAMTA1	NM_015215	chr1:6767971-7752353	template	31	Intron_6	--	Yes	--	Yes	--	--	--	--	--	--	--
G4ST01007468285	chr1:7468286-7468323	-	GGGGAAGGGGCAGAGTCATTGTGACTCAGTGGGAGGGG<br>...((((((...))))))....	CAMTA1	NM_015215	chr1:6767971-7752353	template	38	Intron_6	--	Yes	--	Yes	--	--	--	Yes	--	--	--
G4ST01007487099	chr1:7487100-7487134	+	GGGAGGCCTTCTCAGCCTCGGGCAGGGAGTGTGGG<br>((((.......)))).	CAMTA1	NM_015215	chr1:6767971-7752353	non-template	35	Intron_6	--	Yes	--	Yes	--	--	--	--	--	--	--
G4ST01007527591	chr1:7527592-7527624	-	GGGGCCATGTGGGAGATGTCTGGGTTATGAGGG<br>((....))	CAMTA1	NM_015215	chr1:6767971-7752353	template	33	Intron_6	--	Yes	--	Yes	--	--	--	--	--	--	--
G4ST01007527600	chr1:7527601-7527630	-	GGGGGTGGGGCCATGTGGGAGATGTCTGGG<br>((....))	CAMTA1	NM_015215	chr1:6767971-7752353	template	30	Intron_6	--	Yes	--	Yes	--	--	--	--	--	--	--
G4ST01007562007	chr1:7562008-7562038	+	GGGCCCTGGGCATGGCAGCGTGGGCTTGGGG<br>.(((....)))	CAMTA1	NM_015215	chr1:6767971-7752353	non-template	31	Intron_6	--	Yes	--	Yes	--	--	--	--	--	--	--
G4ST01007575160	chr1:7575161-7575202	+	GGGCCAGCACCCTCTGGGGGTCCTCTGATGACCAGGGCAGGG<br>((((......)))) ((((.......)))).	CAMTA1	NM_015215	chr1:6767971-7752353	non-template	42	Intron_6	--	Yes	--	Yes	--	--	--	--	--	--	Yes
G4ST01007575175	chr1:7575176-7575212	+	GGGGGTCCTCTGATGACCAGGGCAGGGACTCTTAGGG<br>((((.......)))).	CAMTA1	NM_015215	chr1:6767971-7752353	non-template	37	Intron_6	--	Yes	--	Yes	--	--	--	--	--	--	Yes
G4ST01007577271	chr1:7577272-7577306	+	GGGTGGTGACTGCACCAGGGCAGAGCAGGGTGGGG<br>.((((....)))).	CAMTA1	NM_015215	chr1:6767971-7752353	non-template	35	Intron_6	--	Yes	--	Yes	--	--	--	--	--	--	--
G4ST01007613695	chr1:7613696-7613730	-	GGGGTGTGCAGGGGAGTGGGGAACAGCCTTCAGGG<br>(((.....))).	CAMTA1	NM_015215	chr1:6767971-7752353	template	35	Intron_6	--	Yes	--	Yes	--	--	--	--	--	--	--
G4ST01007616883	chr1:7616884-7616919	+	GGGGGTATATGGGAGGATTAGGCTTCCTGGGGTGGG<br>((((.......)))).	CAMTA1	NM_015215	chr1:6767971-7752353	non-template	36	Intron_6	--	Yes	--	Yes	--	--	--	--	--	--	--
G4ST01007616934	chr1:7616935-7616968	+	GGGTGATCGACTGGCCGTTGAGGGAGAGGGAGGG<br>...(((((.....)))))	CAMTA1	NM_015215	chr1:6767971-7752353	non-template	34	Intron_6	--	Yes	--	Yes	--	--	--	--	--	--	--
G4ST01007618771	chr1:7618772-7618810	-	GGGGGTGGGGGGTCCCTTCCCGGGAGCCTCCATGCTGGG<br>(((......))) (((......)))	CAMTA1	NM_015215	chr1:6767971-7752353	template	39	Intron_6	--	Yes	--	Yes	--	--	--	--	--	--	--
G4ST01007619826	chr1:7619827-7619864	-	GGGCCAGCCTGGGACCCGTGGGAGGCCAAGGTGGCGGG<br>..((((...))))	CAMTA1	NM_015215	chr1:6767971-7752353	template	38	Intron_6	--	Yes	--	Yes	--	--	--	Yes	--	--	--
G4ST01007631124	chr1:7631125-7631158	+	GGGGGGATCAGCCCCGGGTGGGAGAGAGAGGGGG<br>(((.....))).	CAMTA1	NM_015215	chr1:6767971-7752353	non-template	34	Intron_7	--	Yes	--	Yes	--	--	--	--	--	--	--
G4ST01007631973	chr1:7631974-7632015	+	GGGGCCTGGGAGTCATGGGGGAGGCAGCGATAGCGCTGGGGG<br>.....(((((....))))).	CAMTA1	NM_015215	chr1:6767971-7752353	non-template	42	Intron_7	--	Yes	--	Yes	--	--	--	--	Yes	--	--
G4ST01007634475	chr1:7634476-7634516	-	GGGAGACCGGGTGGCAGCACCGTGCTGGCCTGGGGTCGGGG<br>.(((((((...)))).)))..	CAMTA1	NM_015215	chr1:6767971-7752353	template	41	Intron_7	--	Yes	--	Yes	--	--	--	--	Yes	--	--
G4ST01007635148	chr1:7635149-7635195	-	GGGCTGTCTCAGAGGGGCCAGGCCTTGCTGGCTGGGGCCTTAGTGGG<br>(((...))).. (((((......)))))..	CAMTA1	NM_015215	chr1:6767971-7752353	template	47	Intron_7	--	Yes	--	Yes	--	--	--	--	--	--	--
G4ST01007644315	chr1:7644316-7644355	-	GGGGGGCTGTCAGAGCCCGTGGGTGCAGAGGGGACCTGGG<br>(((((.....)))))..	CAMTA1	NM_015215	chr1:6767971-7752353	template	40	Intron_7	--	Yes	--	Yes	--	--	--	--	--	--	Yes
G4ST01007655530	chr1:7655531-7655568	-	GGGCCTTCTGGAGCCAGGCAGGTGGGGGCTGGGAGGGG<br>(((.(((....)))..)))...	CAMTA1	NM_015215	chr1:6767971-7752353	template	38	Intron_10	--	Yes	--	Yes	--	--	--	Yes	--	--	Yes
G4ST01007663766	chr1:7663767-7663800	-	GGGGAAGGGAGGGGAAGACCCCTAGGTCTGGGGG<br>..(((((....)))))..	CAMTA1	NM_015215	chr1:6767971-7752353	template	34	Intron_11	--	Yes	--	Yes	--	--	--	Yes	Yes	--	Yes

G4ST01007686721	chr1:7686722-7686751	-	GGGGGCCAGGCTGGAGGGAGGGGGCCGGGG<br>..(((...))).	CAMTA1	NM_015215	chr1:6767971-7752353	template	30	Intron_11	--	Yes	--	Yes	--	--	--	Yes	Yes	Yes	--
G4ST01007686727	chr1:7686728-7686761	-	GGGGAGGCGTGGGGGCCAGGCTGGAGGGAGGGGG<br>..(((...))).	CAMTA1	NM_015215	chr1:6767971-7752353	template	34	Intron_11	--	Yes	--	Yes	--	--	--	Yes	Yes	Yes	--
G4ST01007686912	chr1:7686913-7686938	+	GGGCGGGCGCGGGGCGGCCCCGCGGG<br>(((....)))	CAMTA1	NM_015215	chr1:6767971-7752353	non-template	26	Intron_11	--	Yes	--	Yes	--	--	--	--	Yes	Yes	--
G4ST01007686916	chr1:7686917-7686944	+	GGGCGCGGGGCGGCCCCGCGGGAAGGGG<br>(((....)))	CAMTA1	NM_015215	chr1:6767971-7752353	non-template	28	Intron_11	--	Yes	--	Yes	--	--	--	--	Yes	Yes	--
G4ST01007686922	chr1:7686923-7686950	+	GGGGCGGCCCCGCGGGAAGGGGAACGGG<br>(((....)))	CAMTA1	NM_015215	chr1:6767971-7752353	non-template	28	Intron_11	--	Yes	--	Yes	--	--	--	--	Yes	Yes	--
G4ST01007686960	chr1:7686961-7686993	+	GGGGCTCCGGGGGCCGTCGGCCCGGGCAGCGGG<br>((((...)))).	CAMTA1	NM_015215	chr1:6767971-7752353	non-template	33	Intron_11	--	Yes	--	Yes	--	--	--	--	Yes	Yes	--
G4ST01007688137	chr1:7688138-7688169	+	GGGCGGGGACGAGCGTGTCGGGCGCGCGCGGG<br>((((....))))	CAMTA1	NM_015215	chr1:6767971-7752353	non-template	32	Intron_11	--	Yes	--	Yes	--	--	--	--	--	--	Yes
G4ST01007688141	chr1:7688142-7688174	+	GGGGACGAGCGTGTCGGGCGCGCGCGGGCGGGG<br>((((....))))	CAMTA1	NM_015215	chr1:6767971-7752353	non-template	33	Intron_11	--	Yes	--	Yes	--	--	--	--	--	--	Yes
G4ST01007766289	chr1:7766290-7766323	-	GGGCGACGGTGTCGAGGGCGGGGGCATTAGAGGG<br>((((...)))).	PER3	NM_016831	chr1:7767350-7827824	template	-1026	upstream	Yes	--	--	--	--	--	--	Yes	Yes	Yes	Yes
G4ST01007766299	chr1:7766300-7766327	-	GGGGGGGCGACGGTGTCGAGGGCGGGGG<br>((((...)))).	PER3	NM_016831	chr1:7767350-7827824	template	-1022	upstream	Yes	--	--	--	--	--	--	Yes	Yes	Yes	Yes
G4ST01007766305	chr1:7766306-7766332	-	GGGGCGGGGGGGCGACGGTGTCGAGGG<br>((((...)))).	PER3	NM_016831	chr1:7767350-7827824	template	-1017	upstream	Yes	--	--	--	--	--	--	Yes	Yes	Yes	Yes
G4ST01007766746	chr1:7766747-7766785	-	GGGGCCCCCGGGGCGCGTTACATAACGCGCGGGCTCGGG<br>(((((((....)))))))	PER3	NM_016831	chr1:7767350-7827824	template	-564	upstream	Yes	--	--	--	--	--	--	Yes	Yes	Yes	Yes
G4ST01007766752	chr1:7766753-7766791	-	GGGGGCGGGGCCCCCGGGGCGCGTTACATAACGCGCGGG<br>(((((((....)))))))	PER3	NM_016831	chr1:7767350-7827824	template	-558	upstream	Yes	--	--	--	--	--	--	Yes	Yes	Yes	Yes
G4ST01007766970	chr1:7766971-7767011	+	GGGCGGCCCCGGGGGCGAGCGGCTGTGCGCGGGGCCAAGGG<br>..((.(((....))).))	PER3	NM_016831	chr1:7767350-7827824	non-template	-338	upstream	Yes	--	--	--	--	--	--	--	Yes	Yes	--
G4ST01007766980	chr1:7766981-7767017	+	GGGGGCGAGCGGCTGTGCGCGGGGCCAAGGGCGGGGG<br>..((.(((....))).))	PER3	NM_016831	chr1:7767350-7827824	non-template	-332	upstream	Yes	--	--	--	--	--	--	--	Yes	Yes	--
G4ST01007961955	chr1:7961956-7961992	-	GGGGAGGCTATGCATGGCCAGGGGCGGGGGCCTGGGG<br>..(((((....)))))..	PARK7	NM_007262	chr1:7944301-7967929	template	37	Intron_6.I	--	Yes	--	Yes	--	--	--	--	--	--	--
G4ST01007961955	chr1:7961956-7961992	-	GGGGAGGCTATGCATGGCCAGGGGCGGGGGCCTGGGG<br>..(((((....)))))..	PARK7	NM_001123377	chr1:7944301-7967929	template	37	Intron_6.I	--	Yes	--	Yes	--	--	--	--	--	--	--
G4ST01008009586	chr1:8009587-8009629	+	GGGACGATGAGGGACAGTGAGGGACTGTGTAGGACAGTGAGGG<br>(((((.....)))))..	ERRFI1	NM_018948	chr1:7994366-8008980	template	-606	upstream	Yes	--	--	--	--	--	--	Yes	Yes	Yes	Yes
G4ST01008009596	chr1:8009597-8009639	+	GGGACAGTGAGGGACTGTGTAGGACAGTGAGGGACAGTGTGGG<br>(((((.....)))))..	ERRFI1	NM_018948	chr1:7994366-8008980	template	-616	upstream	Yes	--	--	--	--	--	--	Yes	Yes	Yes	Yes
G4ST01008009606	chr1:8009607-8009649	+	GGGACTGTGTAGGACAGTGAGGGACAGTGTGGGACGGTGAGGG<br>(((((.....)))))..	ERRFI1	NM_018948	chr1:7994366-8008980	template	-626	upstream	Yes	--	--	--	--	--	--	Yes	Yes	Yes	Yes
G4ST01008318040	chr1:8318041-8318073	+	GGGGGTGCGGGGCTCTGATGAGGGGTTTGTGGG<br>.(((....)))	SLC45A1	NM_001080397	chr1:8306977-8326814	non-template	33	Intron_4	--	Yes	--	Yes	--	--	--	--	--	--	--
G4ST01008321806	chr1:8321807-8321842	-	GGGGCTGTGACACCAAAGGGGCTCAGGGACTGAGGG<br>.((.((....)).))	SLC45A1	NM_001080397	chr1:8306977-8326814	template	36	Intron_6	--	Yes	--	Yes	--	--	--	--	--	--	--
G4ST01008321814	chr1:8321815-8321850	-	GGGGTCTTGGGGCTGTGACACCAAAGGGGCTCAGGG<br>.((.((....)).))	SLC45A1	NM_001080397	chr1:8306977-8326814	template	36	Intron_6	--	Yes	--	Yes	--	--	--	--	--	--	--
G4ST01008337977	chr1:8337978-8338016	+	GGGGGAGGAGCCTGGCCCTGGGCACACGGGGAGGTTGGG<br>..(((.((...)))))	RERE	NM_012102	chr1:8335051-8800286	template	39	Intron_23.I	--	Yes	--	Yes	--	--	--	--	--	--	Yes
G4ST01008337977	chr1:8337978-8338016	+	GGGGGAGGAGCCTGGCCCTGGGCACACGGGGAGGTTGGG<br>..(((.((...)))))	RERE	NM_001042681	chr1:8335051-8800286	template	39	Intron_22.I	--	Yes	--	Yes	--	--	--	--	--	--	Yes
G4ST01008337977	chr1:8337978-8338016	+	GGGGGAGGAGCCTGGCCCTGGGCACACGGGGAGGTTGGG<br>..(((.((...)))))	RERE	NM_001042682	chr1:8335051-8406334	template	39	Intron_12.I	--	Yes	--	Yes	--	--	--	--	--	--	Yes
G4ST01008341093	chr1:8341094-8341127	+	GGGTGGGGCCCGGCGGTCGGGGGGATGGTGAGGG<br>.((((.....))))	RERE	NM_012102	chr1:8335051-8800286	template	34	Exon_21	--	Yes	Yes	--	Yes	--	--	--	--	--	--
G4ST01008341093	chr1:8341094-8341127	+	GGGTGGGGCCCGGCGGTCGGGGGGATGGTGAGGG<br>.((((.....))))	RERE	NM_001042681	chr1:8335051-8800286	template	34	Exon_20	--	Yes	Yes	--	Yes	--	--	--	--	--	--
G4ST01008341093	chr1:8341094-8341127	+	GGGTGGGGCCCGGCGGTCGGGGGGATGGTGAGGG<br>.((((.....))))	RERE	NM_001042682	chr1:8335051-8406334	template	34	Exon_10	--	Yes	Yes	--	Yes	--	--	--	--	--	--
G4ST01008343305	chr1:8343306-8343346	+	GGGGAGGGTGCTTGTGGGCCTGTGGCGCCGGCAGCTGGGGG<br>((.((.((....)).)).))	RERE	NM_012102	chr1:8335051-8800286	template	41	Exon_19	--	Yes	Yes	--	Yes	--	--	--	--	--	--
G4ST01008343305	chr1:8343306-8343346	+	GGGGAGGGTGCTTGTGGGCCTGTGGCGCCGGCAGCTGGGGG<br>((.((.((....)).)).))	RERE	NM_001042681	chr1:8335051-8800286	template	41	Exon_18	--	Yes	Yes	--	Yes	--	--	--	--	--	--
G4ST01008343305	chr1:8343306-8343346	+	GGGGAGGGTGCTTGTGGGCCTGTGGCGCCGGCAGCTGGGGG<br>((.((.((....)).)).))	RERE	NM_001042682	chr1:8335051-8406334	template	41	Exon_8	--	Yes	Yes	--	Yes	--	--	--	--	--	--
G4ST01008343310	chr1:8343311-8343351	+	GGGTGCTTGTGGGCCTGTGGCGCCGGCAGCTGGGGGATGGG<br>((.((.((....)).)).))	RERE	NM_012102	chr1:8335051-8800286	template	41	Exon_19	--	Yes	Yes	--	Yes	--	--	--	--	--	--
G4ST01008343310	chr1:8343311-8343351	+	GGGTGCTTGTGGGCCTGTGGCGCCGGCAGCTGGGGGATGGG<br>((.((.((....)).)).))	RERE	NM_001042681	chr1:8335051-8800286	template	41	Exon_18	--	Yes	Yes	--	Yes	--	--	--	--	--	--
G4ST01008343310	chr1:8343311-8343351	+	GGGTGCTTGTGGGCCTGTGGCGCCGGCAGCTGGGGGATGGG<br>((.((.((....)).)).))	RERE	NM_001042682	chr1:8335051-8406334	template	41	Exon_8	--	Yes	Yes	--	Yes	--	--	--	--	--	--
G4ST01008343569	chr1:8343570-8343604	+	GGGGCCCAGCCTGCAGGCTGTGAGGGCCGGGTGGG<br>...(((((....)))))...	RERE	NM_012102	chr1:8335051-8800286	template	35	Exon_19	--	Yes	Yes	--	Yes	--	--	--	--	Yes	--
G4ST01008343569	chr1:8343570-8343604	+	GGGGCCCAGCCTGCAGGCTGTGAGGGCCGGGTGGG<br>...(((((....)))))...	RERE	NM_001042681	chr1:8335051-8800286	template	35	Exon_18	--	Yes	Yes	--	Yes	--	--	--	--	Yes	--
G4ST01008343569	chr1:8343570-8343604	+	GGGGCCCAGCCTGCAGGCTGTGAGGGCCGGGTGGG<br>...(((((....)))))...	RERE	NM_001042682	chr1:8335051-8406334	template	35	Exon_8	--	Yes	Yes	--	Yes	--	--	--	--	Yes	--
G4ST01008343921	chr1:8343922-8343968	+	GGGAGCCTGCAAGGCTGGGGGCTGGGCCTGCAGCATCTGCTGCTGGG<br>(((((...))))).. ...(((((....))))).	RERE	NM_012102	chr1:8335051-8800286	template	47	Exon_19	--	Yes	Yes	--	Yes	--	--	--	--	Yes	--
G4ST01008343921	chr1:8343922-8343968	+	GGGAGCCTGCAAGGCTGGGGGCTGGGCCTGCAGCATCTGCTGCTGGG<br>(((((...))))).. ...(((((....))))).	RERE	NM_001042681	chr1:8335051-8800286	template	47	Exon_18	--	Yes	Yes	--	Yes	--	--	--	--	Yes	--
G4ST01008343921	chr1:8343922-8343968	+	GGGAGCCTGCAAGGCTGGGGGCTGGGCCTGCAGCATCTGCTGCTGGG<br>(((((...))))).. ...(((((....))))).	RERE	NM_001042682	chr1:8335051-8406334	template	47	Exon_8	--	Yes	Yes	--	Yes	--	--	--	--	Yes	--
G4ST01008354377	chr1:8354378-8354410	+	GGGCAATCTGGGCTGGAGCAACAGGGGCCGGGG<br>(((......)))	RERE	NM_012102	chr1:8335051-8800286	template	33	Intron_13	--	Yes	--	Yes	--	--	--	--	--	--	--
G4ST01008354377	chr1:8354378-8354410	+	GGGCAATCTGGGCTGGAGCAACAGGGGCCGGGG<br>(((......)))	RERE	NM_001042681	chr1:8335051-8800286	template	33	Intron_12	--	Yes	--	Yes	--	--	--	--	--	--	--
G4ST01008354377	chr1:8354378-8354410	+	GGGCAATCTGGGCTGGAGCAACAGGGGCCGGGG<br>(((......)))	RERE	NM_001042682	chr1:8335051-8406334	template	33	Intron_2	--	Yes	--	Yes	--	--	--	--	--	--	--
G4ST01008358976	chr1:8358977-8359010	-	GGGTGCTGGGATGGGGGCTCTACCCTGAGCTGGG<br>.((((......)))).	RERE	NM_012102	chr1:8335051-8800286	non-template	34	Intron_13	--	Yes	--	Yes	--	--	--	--	--	--	--
G4ST01008358976	chr1:8358977-8359010	-	GGGTGCTGGGATGGGGGCTCTACCCTGAGCTGGG<br>.((((......)))).	RERE	NM_001042681	chr1:8335051-8800286	non-template	34	Intron_12	--	Yes	--	Yes	--	--	--	--	--	--	--
G4ST01008358976	chr1:8358977-8359010	-	GGGTGCTGGGATGGGGGCTCTACCCTGAGCTGGG<br>.((((......)))).	RERE	NM_001042682	chr1:8335051-8406334	non-template	34	Intron_2	--	Yes	--	Yes	--	--	--	--	--	--	--
G4ST01008406365	chr1:8406366-8406400	+	GGGGCCGCGCGGCGCGGGGCCCGGGGGGCGCGGGG<br>..((((...))))	RERE	NM_012102	chr1:8335051-8800286	template	35	Intron_12	--	Yes	--	Yes	--	--	--	Yes	Yes	Yes	Yes
G4ST01008406365	chr1:8406366-8406400	+	GGGGCCGCGCGGCGCGGGGCCCGGGGGGCGCGGGG<br>..((((...))))	RERE	NM_001042681	chr1:8335051-8800286	template	35	Intron_11	--	Yes	--	Yes	--	--	--	Yes	Yes	Yes	Yes
G4ST01008406365	chr1:8406366-8406400	+	GGGGCCGCGCGGCGCGGGGCCCGGGGGGCGCGGGG<br>..((((...))))	RERE	NM_001042682	chr1:8335051-8406334	template	-31	upstream	Yes	--	--	--	--	--	--	Yes	Yes	Yes	Yes

G4ST01008408212	chr1:8408213-8408252	+	GGGAGGTGGGTTACAGGGGACAGTCTACACAGACTTGGGG<br>...(((((....)))))..	RERE	NM_012102	chr1:8335051-8800286	template	40	Intron_12	--	Yes	--	Yes	--	--	--	--	--	--	--
G4ST01008408212	chr1:8408213-8408252	+	GGGAGGTGGGTTACAGGGGACAGTCTACACAGACTTGGGG<br>...(((((....)))))..	RERE	NM_001042681	chr1:8335051-8800286	template	40	Intron_11	--	Yes	--	Yes	--	--	--	--	--	--	--
G4ST01008408212	chr1:8408213-8408252	+	GGGAGGTGGGTTACAGGGGACAGTCTACACAGACTTGGGG<br>...(((((....)))))..	RERE	NM_001042682	chr1:8335051-8406334	template	-1878	upstream	Yes	--	--	--	--	--	--	--	--	--	--
G4ST01008408219	chr1:8408220-8408256	+	GGGTTACAGGGGACAGTCTACACAGACTTGGGGCGGG<br>...(((((....)))))..	RERE	NM_012102	chr1:8335051-8800286	template	37	Intron_12	--	Yes	--	Yes	--	--	--	--	--	--	--
G4ST01008408219	chr1:8408220-8408256	+	GGGTTACAGGGGACAGTCTACACAGACTTGGGGCGGG<br>...(((((....)))))..	RERE	NM_001042681	chr1:8335051-8800286	template	37	Intron_11	--	Yes	--	Yes	--	--	--	--	--	--	--
G4ST01008408219	chr1:8408220-8408256	+	GGGTTACAGGGGACAGTCTACACAGACTTGGGGCGGG<br>...(((((....)))))..	RERE	NM_001042682	chr1:8335051-8406334	template	-1885	upstream	Yes	--	--	--	--	--	--	--	--	--	--
G4ST01008516260	chr1:8516261-8516294	-	GGGAAACCCACCTGTTTAGGGTTGGGATTCTGGG<br>((((......)))).	RERE	NM_012102	chr1:8335051-8800286	non-template	34	Intron_8	--	Yes	--	Yes	--	--	--	--	--	--	--
G4ST01008516260	chr1:8516261-8516294	-	GGGAAACCCACCTGTTTAGGGTTGGGATTCTGGG<br>((((......)))).	RERE	NM_001042681	chr1:8335051-8800286	non-template	34	Intron_7	--	Yes	--	Yes	--	--	--	--	--	--	--
G4ST01008516268	chr1:8516269-8516305	-	GGGGAGTAACAGGGAAACCCACCTGTTTAGGGTTGGG<br>((((......)))).	RERE	NM_012102	chr1:8335051-8800286	non-template	37	Intron_8	--	Yes	--	Yes	--	--	--	--	--	--	--
G4ST01008516268	chr1:8516269-8516305	-	GGGGAGTAACAGGGAAACCCACCTGTTTAGGGTTGGG<br>((((......)))).	RERE	NM_001042681	chr1:8335051-8800286	non-template	37	Intron_7	--	Yes	--	Yes	--	--	--	--	--	--	--
G4ST01008547711	chr1:8547712-8547748	+	GGGCAGTGGGGGGAATGGGGAGCTAATGCTCAAAGGG<br>((((....))))...	RERE	NM_012102	chr1:8335051-8800286	template	37	Intron_5	--	Yes	--	Yes	--	--	--	--	--	--	--
G4ST01008547711	chr1:8547712-8547748	+	GGGCAGTGGGGGGAATGGGGAGCTAATGCTCAAAGGG<br>((((....))))...	RERE	NM_001042681	chr1:8335051-8800286	template	37	Intron_4	--	Yes	--	Yes	--	--	--	--	--	--	--
G4ST01008799909	chr1:8799910-8799942	+	GGGGCGCAGGGCCGAGAGGGGCGGCCCGCGGGG<br>(((...))).	RERE	NM_012102	chr1:8335051-8800286	template	33	Exon_1.f	--	Yes	Yes	--	--	Yes	--	Yes	Yes	Yes	Yes
G4ST01008799909	chr1:8799910-8799942	+	GGGGCGCAGGGCCGAGAGGGGCGGCCCGCGGGG<br>(((...))).	RERE	NM_001042681	chr1:8335051-8800286	template	33	Exon_1.f	--	Yes	Yes	--	--	Yes	--	Yes	Yes	Yes	Yes
G4ST01008854490	chr1:8854491-8854523	+	GGGGCTGGAAGCAGGGAGGCCATGGGCTGTGGG<br>(((...))).	ENO1	NM_001201483	chr1:8843646-8854222	template	-268	upstream	Yes	--	--	--	--	--	--	Yes	Yes	Yes	--
G4ST01008854490	chr1:8854491-8854523	+	GGGGCTGGAAGCAGGGAGGCCATGGGCTGTGGG<br>(((...))).	ENO1	NM_001428	chr1:8843646-8861738	template	33	Intron_3	--	Yes	--	Yes	--	--	--	Yes	Yes	Yes	--
G4ST01008989698	chr1:8989699-8989751	+	GGGTGCCAGACCTGAGTCTGGGCCCCCCTGGGAGCCACAGATGATCAGTGGGG<br>....((((....)))) ...(((.((...)).)))	SLC2A7	NM_207420	chr1:8985946-9008991	template	53	Intron_10	--	Yes	--	Yes	--	--	--	--	--	--	--
G4ST01009007589	chr1:9007590-9007619	-	GGGCACAAGGTGGGCACAAGGTGGGCCGGG<br>.((...)) .((...))	SLC2A7	NM_207420	chr1:8985946-9008991	non-template	30	Intron_2	--	Yes	--	Yes	--	--	--	--	--	--	--
G4ST01009022366	chr1:9022367-9022397	-	GGGGCGCAGGTCCTGGGGCGCCGGGCGTGGG<br>...(((...)))	SLC2A5	NR_024180	chr1:9019592-9071097	non-template	31	Intron_9	--	Yes	--	Yes	--	--	--	--	--	--	Yes
G4ST01009022366	chr1:9022367-9022397	-	GGGGCGCAGGTCCTGGGGCGCCGGGCGTGGG<br>...(((...)))	SLC2A5	NM_003039	chr1:9019592-9052474	non-template	31	Intron_7	--	Yes	--	Yes	--	--	--	--	--	--	Yes
G4ST01009022372	chr1:9022373-9022406	-	GGGGGGCGCGGGGCGCAGGTCCTGGGGCGCCGGG<br>...(((...)))	SLC2A5	NR_024180	chr1:9019592-9071097	non-template	34	Intron_9	--	Yes	--	Yes	--	--	--	--	--	--	Yes
G4ST01009022372	chr1:9022373-9022406	-	GGGGGGCGCGGGGCGCAGGTCCTGGGGCGCCGGG<br>...(((...)))	SLC2A5	NM_003039	chr1:9019592-9052474	non-template	34	Intron_7	--	Yes	--	Yes	--	--	--	--	--	--	Yes
G4ST01009022379	chr1:9022380-9022412	-	GGGCTCGGGGGGCGCGGGGCGCAGGTCCTGGGG<br>...(((...)))	SLC2A5	NR_024180	chr1:9019592-9071097	non-template	33	Intron_9	--	Yes	--	Yes	--	--	--	--	--	--	Yes
G4ST01009022379	chr1:9022380-9022412	-	GGGCTCGGGGGGCGCGGGGCGCAGGTCCTGGGG<br>...(((...)))	SLC2A5	NM_003039	chr1:9019592-9052474	non-template	33	Intron_7	--	Yes	--	Yes	--	--	--	--	--	--	Yes
G4ST01009054218	chr1:9054219-9054246	-	GGGCTGGGCCGTCAAGGCGGGGACTGGG<br>.(((....)))	SLC2A5	NR_024180	chr1:9019592-9071097	non-template	28	Intron_2	--	Yes	--	Yes	--	--	--	Yes	--	--	--
G4ST01009054218	chr1:9054219-9054246	-	GGGCTGGGCCGTCAAGGCGGGGACTGGG<br>.(((....)))	SLC2A5	NM_003039	chr1:9019592-9052474	non-template	-1744	upstream	Yes	--	--	--	--	--	--	Yes	--	--	--
G4ST01009054218	chr1:9054219-9054246	-	GGGCTGGGCCGTCAAGGCGGGGACTGGG<br>.(((....)))	SLC2A5	NM_001135585	chr1:9024014-9052474	non-template	-1744	upstream	Yes	--	--	--	--	--	--	Yes	--	--	--
G4ST01009054508	chr1:9054509-9054537	-	GGGCTGAGGCAGAGGGGTCCTGGGCAGGG<br>(((...)))..	SLC2A5	NR_024180	chr1:9019592-9071097	non-template	29	Intron_2	--	Yes	--	Yes	--	--	--	--	Yes	--	--
G4ST01009054513	chr1:9054514-9054546	-	GGGGGATCCGGGCTGAGGCAGAGGGGTCCTGGG<br>(((...)))..	SLC2A5	NR_024180	chr1:9019592-9071097	non-template	33	Intron_2	--	Yes	--	Yes	--	--	--	--	Yes	--	--
G4ST01009054613	chr1:9054614-9054642	-	GGGGGGGATCTGGGCTGAGGCAGAGGGGG<br>(((...)))...	SLC2A5	NR_024180	chr1:9019592-9071097	non-template	29	Intron_2	--	Yes	--	Yes	--	--	--	--	--	--	--
G4ST01009093618	chr1:9093619-9093653	-	GGGCCTGTGGGGGCACAGGGCTGCTGCAGACAGGG<br>(((...)))...	GPR157	NM_024980	chr1:9087063-9111816	non-template	35	Intron_2	--	Yes	--	Yes	--	--	--	Yes	Yes	--	--
G4ST01009111224	chr1:9111225-9111259	+	GGGCAAGACCGGGAGGGGTCGGCCTGTGTCGGGGG<br>..((((....)))).	GPR157	NM_024980	chr1:9087063-9111816	template	35	Intron_1.f	--	Yes	--	Yes	--	--	--	--	Yes	Yes	Yes
G4ST01009111875	chr1:9111876-9111904	-	GGGGCGCGGCGCGTGGGCGGGGCTCAGGG<br>.(((...))).	GPR157	NM_024980	chr1:9087063-9111816	non-template	-59	upstream	Yes	--	--	--	--	--	--	Yes	Yes	Yes	Yes
G4ST01009111882	chr1:9111883-9111909	-	GGGGTGGGGCGCGGCGCGTGGGCGGGG<br>.(((...))).	GPR157	NM_024980	chr1:9087063-9111816	non-template	-66	upstream	Yes	--	--	--	--	--	--	Yes	Yes	Yes	Yes
G4ST01009111887	chr1:9111888-9111917	-	GGGGTACCGGGGTGGGGCGCGGCGCGTGGG<br>.(((...))).	GPR157	NM_024980	chr1:9087063-9111816	non-template	-71	upstream	Yes	--	--	--	--	--	--	Yes	Yes	Yes	Yes
G4ST01009111900	chr1:9111901-9111931	-	GGGGCTTAAAGGCCGGGGTACCGGGGTGGGG<br>(((....)))..	GPR157	NM_024980	chr1:9087063-9111816	non-template	-84	upstream	Yes	--	--	--	--	--	--	Yes	Yes	Yes	--
G4ST01009111990	chr1:9111991-9112022	-	GGGCATGTGGGCCGGGGGACGTGTGTCCGGGG<br>((((....)))).	GPR157	NM_024980	chr1:9087063-9111816	non-template	-174	upstream	Yes	--	--	--	--	--	--	Yes	Yes	Yes	Yes
G4ST01009112087	chr1:9112088-9112120	-	GGGCCCTGTGGACAGGGGATCGGGGCGCGTGGG<br>..(((....)))	GPR157	NM_024980	chr1:9087063-9111816	non-template	-271	upstream	Yes	--	--	--	--	--	--	Yes	Yes	Yes	--
G4ST01009230822	chr1:9230823-9230849	-	GGGCACGCACTGCGGGGGCTGGGGGGG<br>..(((...))).	H6PD	NM_004285	chr1:9217450-9253981	template	27	Intron_3	--	Yes	--	Yes	--	--	--	--	--	--	--
G4ST01009276255	chr1:9276256-9276292	-	GGGAGAAGCCGCGGCGGCTCCGGGGCGGCGGGGAGGG<br>...(((((...)))))...	SPSB1	NM_025106	chr1:9275528-9352177	template	37	Intron_1.f	--	Yes	--	Yes	--	--	--	Yes	Yes	Yes	--
G4ST01009287739	chr1:9287740-9287774	-	GGGACTCTCCTGAGGGGGCAGATGGGCCACAAGGG<br>.(((....))).	SPSB1	NM_025106	chr1:9275528-9352177	template	35	Intron_1.f	--	Yes	--	Yes	--	--	--	--	--	--	Yes
G4ST01009287748	chr1:9287749-9287783	-	GGGAGGAGAGGGACTCTCCTGAGGGGGCAGATGGG<br>.(((....))).	SPSB1	NM_025106	chr1:9275528-9352177	template	35	Intron_1.f	--	Yes	--	Yes	--	--	--	--	--	--	Yes
G4ST01009299202	chr1:9299203-9299232	+	GGGGGGGGCTGATCGAAGCGGGTTCAGGGG<br>(((......)))	SPSB1	NM_025106	chr1:9275528-9352177	non-template	30	Intron_1.f	--	Yes	--	Yes	--	--	--	--	Yes	--	--
G4ST01009299206	chr1:9299207-9299254	+	GGGGCTGATCGAAGCGGGTTCAGGGGCTGCCATTTACGAAAGGCTGGG<br>(((......))) ...(((.((....)).))).	SPSB1	NM_025106	chr1:9275528-9352177	non-template	48	Intron_1.f	--	Yes	--	Yes	--	--	--	--	Yes	--	--
G4ST01009311849	chr1:9311850-9311890	+	GGGGCCCGAGCACAGCCGGTTGAGGGTTTGTGGGTGGGGGG<br>..(((.((...)))))....	SPSB1	NM_025106	chr1:9275528-9352177	non-template	41	Intron_1.f	--	Yes	--	Yes	--	--	--	--	--	--	--
G4ST01009312083	chr1:9312084-9312116	+	GGGAGTAGGGAAGAGAGCCTTTGGGAAGATGGG<br>(((.....))).	SPSB1	NM_025106	chr1:9275528-9352177	non-template	33	Intron_1.f	--	Yes	--	Yes	--	--	--	--	--	--	--
G4ST01009345330	chr1:9345331-9345368	+	GGGGAACAGTGAGACAGTTGTGGGTTGGGGCTCTGGGG<br>.(((.((...)).))))..	SPSB1	NM_025106	chr1:9275528-9352177	non-template	38	Intron_2.l	--	Yes	--	Yes	--	--	--	Yes	Yes	--	--
G4ST01009350958	chr1:9350959-9350992	-	GGGCACGTGTGCACATGTGCCCAGGGAGGGCGGG<br>((((((....))))))....	SPSB1	NM_025106	chr1:9275528-9352177	template	34	Exon_3.l	--	Yes	Yes	--	--	--	Yes	--	--	--	--
G4ST01009538677	chr1:9538678-9538709	+	GGGCAGGAAAGTTGGGGGTGGGTACAGGAGGG<br>(((.....))).	SLC25A33	NM_032315	chr1:9522115-9565418	non-template	32	Intron_2	--	Yes	--	Yes	--	--	--	--	Yes	--	--
G4ST01009571434	chr1:9571435-9571464	-	GGGGGCGCGGGCCGCAGGCGGGACGCGGGG<br>((...)).	TMEM201	NM_001010866	chr1:9571519-9587607	template	-54	upstream	Yes	--	--	--	--	--	--	--	Yes	Yes	--

G4ST01009571434	chr1:9571435-9571464	-	GGGGGCGCGGGCCGCAGGCGGGACGCGGGG<br>((...)).	TMEM201	NM_001130924	chr1:9571519-9597522	template	-54	upstream	Yes	--	--	--	--	--	--	--	Yes	Yes	--
G4ST01009584107	chr1:9584108-9584138	+	GGGGCCAGGGCCAGGAGTTGGCGGGCAGGGG<br>(((.....))).	TMEM201	NM_001010866	chr1:9571519-9587607	non-template	31	Intron_5.l	--	Yes	--	Yes	--	--	--	--	--	--	--
G4ST01009584107	chr1:9584108-9584138	+	GGGGCCAGGGCCAGGAGTTGGCGGGCAGGGG<br>(((.....))).	TMEM201	NM_001130924	chr1:9571519-9597522	non-template	31	Intron_5	--	Yes	--	Yes	--	--	--	--	--	--	--
G4ST01009585036	chr1:9585037-9585066	-	GGGCCAGGACCTGGGGCTGTAGGGTGGGGG<br>.(((...)))	TMEM201	NM_001010866	chr1:9571519-9587607	template	30	Exon_6.l	--	Yes	Yes	--	--	--	Yes	--	--	--	Yes
G4ST01009585036	chr1:9585037-9585066	-	GGGCCAGGACCTGGGGCTGTAGGGTGGGGG<br>.(((...)))	TMEM201	NM_001130924	chr1:9571519-9597522	template	30	Intron_6	--	Yes	--	Yes	--	--	--	--	--	--	Yes
G4ST01009585042	chr1:9585043-9585072	-	GGGACTGGGCCAGGACCTGGGGCTGTAGGG<br>.(((...)))	TMEM201	NM_001010866	chr1:9571519-9587607	template	30	Exon_6.l	--	Yes	Yes	--	--	--	Yes	--	--	--	Yes
G4ST01009585042	chr1:9585043-9585072	-	GGGACTGGGCCAGGACCTGGGGCTGTAGGG<br>.(((...)))	TMEM201	NM_001130924	chr1:9571519-9597522	template	30	Intron_6	--	Yes	--	Yes	--	--	--	--	--	--	Yes
G4ST01009587323	chr1:9587324-9587356	-	GGGGTACGGGGGGCCGGCTTGGCCTGGGTGGGG<br>.((((.....)))).	TMEM201	NM_001010866	chr1:9571519-9587607	template	33	Exon_6.l	--	Yes	Yes	--	--	--	Yes	--	--	--	--
G4ST01009587323	chr1:9587324-9587356	-	GGGGTACGGGGGGCCGGCTTGGCCTGGGTGGGG<br>.((((.....)))).	TMEM201	NM_001130924	chr1:9571519-9597522	template	33	Intron_6	--	Yes	--	Yes	--	--	--	--	--	--	--
G4ST01009590498	chr1:9590499-9590524	+	GGGTACATAGTGTAGGGAGGGCCGGG<br>.(((...))).	TMEM201	NM_001130924	chr1:9571519-9597522	non-template	26	Intron_7	--	Yes	--	Yes	--	--	--	--	--	--	--
G4ST01009591584	chr1:9591585-9591615	+	GGGTGGGTCGGGGGCAGGAGTTCCTGCAGGG<br>.(((((....))))).	TMEM201	NM_001130924	chr1:9571519-9597522	non-template	31	Intron_7	--	Yes	--	Yes	--	--	--	--	--	--	--
G4ST01009593313	chr1:9593314-9593347	-	GGGCGTGGTGGGGGCCTCTCCAGGGGGTGACGGG<br>..(((....)))	TMEM201	NM_001130924	chr1:9571519-9597522	template	34	Exon_9	--	Yes	Yes	--	Yes	--	--	--	--	--	--
G4ST01009634351	chr1:9634352-9634385	-	GGGCGCGAGTGAGCCTCGAGGGAGGGAGGCGGGG<br>..((((.....)))).	PIK3CD	NM_005026	chr1:9634377-9711759	template	9	upstream-Exon_1.f	Yes	Yes	Yes	--	--	Yes	--	Yes	Yes	Yes	Yes
G4ST01009635334	chr1:9635335-9635374	+	GGGGAACTGGGACGACCTTTCGTGGGCACCAGGATGGGGG<br>((((....)))) ..(((...)))	C1orf200	NR_027045	chr1:9635255-9637231	template	40	Exon_2.l	--	Yes	Yes	--	--	--	--	Yes	Yes	Yes	Yes
G4ST01009635334	chr1:9635335-9635374	+	GGGGAACTGGGACGACCTTTCGTGGGCACCAGGATGGGGG<br>((((....)))) ..(((...)))	PIK3CD	NM_005026	chr1:9634377-9711759	non-template	40	Intron_1.f	--	Yes	--	Yes	--	--	--	Yes	Yes	Yes	Yes
G4ST01009635342	chr1:9635343-9635384	+	GGGACGACCTTTCGTGGGCACCAGGATGGGGGCGTTGCAGGG<br>((((....)))) ..(((...)))	C1orf200	NR_027045	chr1:9635255-9637231	template	42	Exon_2.l	--	Yes	Yes	--	--	--	--	Yes	Yes	Yes	Yes
G4ST01009635342	chr1:9635343-9635384	+	GGGACGACCTTTCGTGGGCACCAGGATGGGGGCGTTGCAGGG<br>((((....)))) ..(((...)))	PIK3CD	NM_005026	chr1:9634377-9711759	non-template	42	Intron_1.f	--	Yes	--	Yes	--	--	--	Yes	Yes	Yes	Yes
G4ST01009635357	chr1:9635358-9635389	+	GGGCACCAGGATGGGGGCGTTGCAGGGCGGGG<br>..(((...)))	C1orf200	NR_027045	chr1:9635255-9637231	template	32	Exon_2.l	--	Yes	Yes	--	--	--	--	Yes	Yes	Yes	Yes
G4ST01009635357	chr1:9635358-9635389	+	GGGCACCAGGATGGGGGCGTTGCAGGGCGGGG<br>..(((...)))	PIK3CD	NM_005026	chr1:9634377-9711759	non-template	32	Intron_1.f	--	Yes	--	Yes	--	--	--	Yes	Yes	Yes	Yes
G4ST01009670257	chr1:9670258-9670284	-	GGGAGAGGGCGGAGCCCGGGCCGGGGG<br>.((...))	PIK3CD	NM_005026	chr1:9634377-9711759	template	27	Intron_1.f	--	Yes	--	Yes	--	--	--	Yes	Yes	Yes	--
G4ST01009670284	chr1:9670285-9670319	+	GGGATCTGTGAAAGCACAGCCAGGGTGCGGGCGGG<br>..(((((....)))))...	PIK3CD	NM_005026	chr1:9634377-9711759	non-template	35	Intron_1.f	--	Yes	--	Yes	--	--	--	Yes	Yes	Yes	--
G4ST01009670306	chr1:9670307-9670341	+	GGGTGCGGGCGGGTGAGTGCCCACCTGGTGCAGGG<br>...(((((.....))))).	PIK3CD	NM_005026	chr1:9634377-9711759	non-template	35	Intron_1.f	--	Yes	--	Yes	--	--	--	Yes	Yes	Yes	--
G4ST01009672081	chr1:9672082-9672112	-	GGGGCGGGGCCGGGGCCGGCGGCGGCGCGGG<br>((((....))))..	PIK3CD	NM_005026	chr1:9634377-9711759	template	31	Intron_1.f	--	Yes	--	Yes	--	--	--	Yes	Yes	Yes	Yes
G4ST01009672407	chr1:9672408-9672435	-	GGGACGCCCGGCGGGGCTGGGGGCGGGG<br>.(((...)))	PIK3CD	NM_005026	chr1:9634377-9711759	template	28	Intron_1.f	--	Yes	--	Yes	--	--	--	--	--	Yes	Yes
G4ST01009672412	chr1:9672413-9672440	-	GGGGTGGGACGCCCGGCGGGGCTGGGGG<br>.(((...)))	PIK3CD	NM_005026	chr1:9634377-9711759	template	28	Intron_1.f	--	Yes	--	Yes	--	--	--	--	--	Yes	Yes
G4ST01009672419	chr1:9672420-9672444	-	GGGCGGGGTGGGACGCCCGGCGGGG<br>.(((...)))	PIK3CD	NM_005026	chr1:9634377-9711759	template	25	Intron_1.f	--	Yes	--	Yes	--	--	--	--	--	Yes	Yes
G4ST01009674223	chr1:9674224-9674254	+	GGGGGAACGGGCCAAGTGTGTGGGGGAAGGG<br>(((......)))	PIK3CD	NM_005026	chr1:9634377-9711759	non-template	31	Intron_2	--	Yes	--	Yes	--	--	--	--	Yes	--	--
G4ST01009698963	chr1:9698964-9698998	-	GGGTAGGGTCTGGGGAGTTCACAAATGACACTGGG<br>.((((((....))).)))	PIK3CD	NM_005026	chr1:9634377-9711759	template	35	Intron_5	--	Yes	--	Yes	--	--	--	Yes	Yes	Yes	--
G4ST01009699904	chr1:9699905-9699933	+	GGGCTGGGGGCCTGTGGGACTGCCGTGGG<br>((....))	PIK3CD	NM_005026	chr1:9634377-9711759	non-template	29	Intron_7	--	Yes	--	Yes	--	--	--	Yes	Yes	Yes	--
G4ST01009699909	chr1:9699910-9699940	+	GGGGGCCTGTGGGACTGCCGTGGGTGGGGGG<br>((....))	PIK3CD	NM_005026	chr1:9634377-9711759	non-template	31	Intron_7	--	Yes	--	Yes	--	--	--	Yes	Yes	Yes	--
G4ST01009707340	chr1:9707341-9707370	+	GGGCAAGGGCAGGTGTCCTTGGGGAAGGGG<br>.(((...)))..	PIK3CD	NM_005026	chr1:9634377-9711759	non-template	30	Intron_22	--	Yes	--	Yes	--	--	--	--	--	Yes	--
G4ST01009750844	chr1:9750845-9750883	+	GGGGGCGGGGTTGTCCTGGACGGCACCATGGGGGCGGGG<br>((((((...))))))).......	CLSTN1	NM_014944	chr1:9711666-9807137	template	39	Intron_2	--	Yes	--	Yes	--	--	--	Yes	--	--	--
G4ST01009750844	chr1:9750845-9750883	+	GGGGGCGGGGTTGTCCTGGACGGCACCATGGGGGCGGGG<br>((((((...))))))).......	CLSTN1	NM_001009566	chr1:9711666-9807137	template	39	Intron_2	--	Yes	--	Yes	--	--	--	Yes	--	--	--
G4ST01009770793	chr1:9770794-9770828	+	GGGAGGGTGAGGTGGGAGGATCACCTGAGCCTGGG<br>(((.((....)).)))	CLSTN1	NM_014944	chr1:9711666-9807137	template	35	Intron_1.f	--	Yes	--	Yes	--	--	--	--	--	--	--
G4ST01009770793	chr1:9770794-9770828	+	GGGAGGGTGAGGTGGGAGGATCACCTGAGCCTGGG<br>(((.((....)).)))	CLSTN1	NM_001009566	chr1:9711666-9807137	template	35	Intron_1.f	--	Yes	--	Yes	--	--	--	--	--	--	--
G4ST01009806443	chr1:9806444-9806485	+	GGGGCTCTAGGGGCCTGGGGCTAGCTGCTCCGCGGCGCGGGG<br>....(((((...)))))...	CLSTN1	NM_014944	chr1:9711666-9807137	template	42	Exon_1.f	--	Yes	Yes	--	--	Yes	--	Yes	Yes	Yes	--
G4ST01009806443	chr1:9806444-9806485	+	GGGGCTCTAGGGGCCTGGGGCTAGCTGCTCCGCGGCGCGGGG<br>....(((((...)))))...	CLSTN1	NM_001009566	chr1:9711666-9807137	template	42	Exon_1.f	--	Yes	Yes	--	--	Yes	--	Yes	Yes	Yes	--
G4ST01009806452	chr1:9806453-9806496	+	GGGGCCTGGGGCTAGCTGCTCCGCGGCGCGGGGAGCTCCGGGGG<br>....(((((...)))))...	CLSTN1	NM_014944	chr1:9711666-9807137	template	44	Exon_1.f	--	Yes	Yes	--	--	Yes	--	Yes	Yes	Yes	--
G4ST01009806452	chr1:9806453-9806496	+	GGGGCCTGGGGCTAGCTGCTCCGCGGCGCGGGGAGCTCCGGGGG<br>....(((((...)))))...	CLSTN1	NM_001009566	chr1:9711666-9807137	template	44	Exon_1.f	--	Yes	Yes	--	--	Yes	--	Yes	Yes	Yes	--
G4ST01009834875	chr1:9834876-9834917	+	GGGAGGACGCACCCAGCTGTCGGGAGGGCCAGAGATGGAGGG<br>..(((((.....)).))) (((....))).	CTNNBIP1	NM_020248	chr1:9830921-9892903	template	42	Intron_5.l	--	Yes	--	Yes	--	--	--	--	--	--	--
G4ST01009834875	chr1:9834876-9834917	+	GGGAGGACGCACCCAGCTGTCGGGAGGGCCAGAGATGGAGGG<br>..(((((.....)).))) (((....))).	CTNNBIP1	NM_001012329	chr1:9830921-9892903	template	42	Intron_4.l	--	Yes	--	Yes	--	--	--	--	--	--	--
G4ST01009844656	chr1:9844657-9844687	-	GGGTGGGAACAGGGCAAGATGGATTTGGGGG<br>((((.....)))).	CTNNBIP1	NM_020248	chr1:9830921-9892903	non-template	31	Intron_5.l	--	Yes	--	Yes	--	--	--	--	--	--	Yes
G4ST01009844656	chr1:9844657-9844687	-	GGGTGGGAACAGGGCAAGATGGATTTGGGGG<br>((((.....)))).	CTNNBIP1	NM_001012329	chr1:9830921-9892903	non-template	31	Intron_4.l	--	Yes	--	Yes	--	--	--	--	--	--	Yes
G4ST01009867596	chr1:9867597-9867629	+	GGGGACAGGGCCTGTGTCACAAGGGCAGCTGGG<br>..(((...))).	CTNNBIP1	NM_020248	chr1:9830921-9892903	template	33	Intron_1.f	--	Yes	--	Yes	--	--	--	--	--	--	--
G4ST01009867596	chr1:9867597-9867629	+	GGGGACAGGGCCTGTGTCACAAGGGCAGCTGGG<br>..(((...))).	CTNNBIP1	NM_001012329	chr1:9830921-9892903	template	33	Intron_1.f	--	Yes	--	Yes	--	--	--	--	--	--	--
G4ST01009867603	chr1:9867604-9867636	+	GGGCCTGTGTCACAAGGGCAGCTGGGAGCTGGG<br>..(((...))).	CTNNBIP1	NM_020248	chr1:9830921-9892903	template	33	Intron_1.f	--	Yes	--	Yes	--	--	--	--	--	--	--
G4ST01009867603	chr1:9867604-9867636	+	GGGCCTGTGTCACAAGGGCAGCTGGGAGCTGGG<br>..(((...))).	CTNNBIP1	NM_001012329	chr1:9830921-9892903	template	33	Intron_1.f	--	Yes	--	Yes	--	--	--	--	--	--	--
G4ST01010293687	chr1:10293688-10293717	-	GGGCATGGAATGCAGGGAGCAAGGGCTGGG<br>(((...)))..	KIF1B	NM_015074	chr1:10193351-10364248	template	30	Intron_20	--	Yes	--	Yes	--	--	--	--	--	--	--
G4ST01010416025	chr1:10416026-10416058	+	GGGGCTGCCAGGGGCTTGCAGCAGAGGGATGGG<br>(((...)))...	APITD1	NM_199294	chr1:10412746-10425459	non-template	33	Intron_1.f	--	Yes	--	Yes	--	--	--	--	--	--	--
G4ST01010416025	chr1:10416026-10416058	+	GGGGCTGCCAGGGGCTTGCAGCAGAGGGATGGG<br>(((...)))...	APITD1	NR_036462	chr1:10413391-10425459	non-template	33	Intron_1.f	--	Yes	--	Yes	--	--	--	--	--	--	--

G4ST01010416025	chr1:10416026-10416058	+	GGGGCTGCCAGGGGCTTGCAGCAGAGGGATGGG<br>(((...)))...	APITD1-CORT	NR_037186	chr1:10413391-10434647	non-template	33	Intron_1.f	--	Yes	--	Yes	--	--	--	--	--	--	--
G4ST01010416025	chr1:10416026-10416058	+	GGGGCTGCCAGGGGCTTGCAGCAGAGGGATGGG<br>(((...)))...	APITD1-CORT	NR_037187	chr1:10413391-10434647	non-template	33	Intron_1.f	--	Yes	--	Yes	--	--	--	--	--	--	--
G4ST01010416025	chr1:10416026-10416058	+	GGGGCTGCCAGGGGCTTGCAGCAGAGGGATGGG<br>(((...)))...	APITD1-CORT	NM_198544	chr1:10412746-10434647	non-template	33	Intron_1.f	--	Yes	--	Yes	--	--	--	--	--	--	--
G4ST01010416025	chr1:10416026-10416058	+	GGGGCTGCCAGGGGCTTGCAGCAGAGGGATGGG<br>(((...)))...	APITD1-CORT	NM_199006	chr1:10412746-10434647	non-template	33	Intron_1.f	--	Yes	--	Yes	--	--	--	--	--	--	--
G4ST01010518955	chr1:10518956-10518995	+	GGGCTGTGCAGCACGGCCTACAGGGGGACTGGGGCTGGGG<br>((((((......))..)))).	PEX14	NM_004565	chr1:10457590-10613405	non-template	40	Intron_3	--	Yes	--	Yes	--	--	--	--	--	--	--
G4ST01010612191	chr1:10612192-10612228	-	GGGATCTGCCAGGAGGGGATCTTCGGGGCTGATGGGG<br>.(((....))).	PEX14	NM_004565	chr1:10457590-10613405	template	37	Exon_9.l	--	Yes	Yes	--	Yes	--	--	--	--	--	Yes
G4ST01010621252	chr1:10621253-10621286	+	GGGGCCGAATCGGCCGCGGGGAGGGGCGCCGGGG<br>((((...))))....	CASZ1	NM_001079843	chr1:10619253-10779294	template	34	Exon_21.l	--	Yes	Yes	--	--	--	Yes	--	--	--	--
G4ST01010632573	chr1:10632574-10632607	+	GGGAGGGGGTCATTGCGAGGATCCAAGGGAAGGG<br>.....(((.((...)))))	CASZ1	NM_001079843	chr1:10619253-10779294	template	34	Intron_13	--	Yes	--	Yes	--	--	--	Yes	--	--	Yes
G4ST01010632573	chr1:10632574-10632607	+	GGGAGGGGGTCATTGCGAGGATCCAAGGGAAGGG<br>.....(((.((...)))))	CASZ1	NM_017766	chr1:10629857-10779294	template	34	Intron_13	--	Yes	--	Yes	--	--	--	Yes	--	--	Yes
G4ST01010632577	chr1:10632578-10632613	+	GGGGGTCATTGCGAGGATCCAAGGGAAGGGAGGGGG<br>.....(((.((...)))))	CASZ1	NM_001079843	chr1:10619253-10779294	template	36	Intron_13	--	Yes	--	Yes	--	--	--	Yes	--	--	Yes
G4ST01010632577	chr1:10632578-10632613	+	GGGGGTCATTGCGAGGATCCAAGGGAAGGGAGGGGG<br>.....(((.((...)))))	CASZ1	NM_017766	chr1:10629857-10779294	template	36	Intron_13	--	Yes	--	Yes	--	--	--	Yes	--	--	Yes
G4ST01010633939	chr1:10633940-10633973	+	GGGCCTCCTGGAGGAGAAGGGCGGGAGGCTGGGG<br>.((((....))))..	CASZ1	NM_001079843	chr1:10619253-10779294	template	34	Intron_11	--	Yes	--	Yes	--	--	--	--	Yes	--	Yes
G4ST01010633939	chr1:10633940-10633973	+	GGGCCTCCTGGAGGAGAAGGGCGGGAGGCTGGGG<br>.((((....))))..	CASZ1	NM_017766	chr1:10629857-10779294	template	34	Intron_11	--	Yes	--	Yes	--	--	--	--	Yes	--	Yes
G4ST01010635716	chr1:10635717-10635753	+	GGGAGGGGGGACCTGCAGTGGCCCCAGGGATGCAGGG<br>(((.((......)).))).	CASZ1	NM_001079843	chr1:10619253-10779294	template	37	Intron_11	--	Yes	--	Yes	--	--	--	--	--	--	--
G4ST01010635716	chr1:10635717-10635753	+	GGGAGGGGGGACCTGCAGTGGCCCCAGGGATGCAGGG<br>(((.((......)).))).	CASZ1	NM_017766	chr1:10629857-10779294	template	37	Intron_11	--	Yes	--	Yes	--	--	--	--	--	--	--
G4ST01010638076	chr1:10638077-10638104	-	GGGCAGCGTCTGGTGGGCCAGGGTGGGG<br>(((...)))..	CASZ1	NM_001079843	chr1:10619253-10779294	non-template	28	Intron_9	--	Yes	--	Yes	--	--	--	--	--	--	--
G4ST01010638076	chr1:10638077-10638104	-	GGGCAGCGTCTGGTGGGCCAGGGTGGGG<br>(((...)))..	CASZ1	NM_017766	chr1:10629857-10779294	non-template	28	Intron_9	--	Yes	--	Yes	--	--	--	--	--	--	--
G4ST01010638216	chr1:10638217-10638244	+	GGGAGTGGGGGGCTCAGAGCCGGGAGGG<br>.((((...))))	CASZ1	NM_001079843	chr1:10619253-10779294	template	28	Intron_9	--	Yes	--	Yes	--	--	--	--	--	--	--
G4ST01010638216	chr1:10638217-10638244	+	GGGAGTGGGGGGCTCAGAGCCGGGAGGG<br>.((((...))))	CASZ1	NM_017766	chr1:10629857-10779294	template	28	Intron_9	--	Yes	--	Yes	--	--	--	--	--	--	--
G4ST01010638222	chr1:10638223-10638250	+	GGGGGGCTCAGAGCCGGGAGGGCCTGGG<br>.((((...))))	CASZ1	NM_001079843	chr1:10619253-10779294	template	28	Intron_9	--	Yes	--	Yes	--	--	--	--	--	--	--
G4ST01010638222	chr1:10638223-10638250	+	GGGGGGCTCAGAGCCGGGAGGGCCTGGG<br>.((((...))))	CASZ1	NM_017766	chr1:10629857-10779294	template	28	Intron_9	--	Yes	--	Yes	--	--	--	--	--	--	--
G4ST01010639407	chr1:10639408-10639441	-	GGGGCTGGGGCTGGGACTGTCACCCACAGCAGGG<br>.((((.....))))..	CASZ1	NM_001079843	chr1:10619253-10779294	non-template	34	Intron_7	--	Yes	--	Yes	--	--	--	--	--	--	Yes
G4ST01010639407	chr1:10639408-10639441	-	GGGGCTGGGGCTGGGACTGTCACCCACAGCAGGG<br>.((((.....))))..	CASZ1	NM_017766	chr1:10629857-10779294	non-template	34	Intron_7	--	Yes	--	Yes	--	--	--	--	--	--	Yes
G4ST01010642487	chr1:10642488-10642515	+	GGGGTGCGGGAGCCAGGCTGGGGGTGGG<br>(((...)))..	CASZ1	NM_001079843	chr1:10619253-10779294	template	28	Exon_6	--	Yes	Yes	--	Yes	--	--	--	--	--	--
G4ST01010642487	chr1:10642488-10642515	+	GGGGTGCGGGAGCCAGGCTGGGGGTGGG<br>(((...)))..	CASZ1	NM_017766	chr1:10629857-10779294	template	28	Exon_6	--	Yes	Yes	--	Yes	--	--	--	--	--	--
G4ST01010643215	chr1:10643216-10643251	+	GGGAGTGGGAGGGACAGGAGGTCCCCCCACTGGGGG<br>.(((..((.....)).))).	CASZ1	NM_001079843	chr1:10619253-10779294	template	36	Intron_5	--	Yes	--	Yes	--	--	--	--	--	--	Yes
G4ST01010643215	chr1:10643216-10643251	+	GGGAGTGGGAGGGACAGGAGGTCCCCCCACTGGGGG<br>.(((..((.....)).))).	CASZ1	NM_017766	chr1:10629857-10779294	template	36	Intron_5	--	Yes	--	Yes	--	--	--	--	--	--	Yes
G4ST01010647177	chr1:10647178-10647204	+	GGGAGGGCCAGGGACAGCGGTGTAGGG<br>(((....))).	CASZ1	NM_001079843	chr1:10619253-10779294	template	27	Intron_5	--	Yes	--	Yes	--	--	--	--	--	--	--
G4ST01010647177	chr1:10647178-10647204	+	GGGAGGGCCAGGGACAGCGGTGTAGGG<br>(((....))).	CASZ1	NM_017766	chr1:10629857-10779294	template	27	Intron_5	--	Yes	--	Yes	--	--	--	--	--	--	--
G4ST01010647533	chr1:10647534-10647565	-	GGGGAGGGGCACCCCAGGTGAGAAGGGAAGGG<br>.((((...))))....	CASZ1	NM_001079843	chr1:10619253-10779294	non-template	32	Intron_5	--	Yes	--	Yes	--	--	--	--	--	--	--
G4ST01010647533	chr1:10647534-10647565	-	GGGGAGGGGCACCCCAGGTGAGAAGGGAAGGG<br>.((((...))))....	CASZ1	NM_017766	chr1:10629857-10779294	non-template	32	Intron_5	--	Yes	--	Yes	--	--	--	--	--	--	--
G4ST01010654734	chr1:10654735-10654768	-	GGGTGAGCCGGGGAGGCCTCTCGGGCAAGCAGGG<br>(((....)))	CASZ1	NM_001079843	chr1:10619253-10779294	non-template	34	Intron_4	--	Yes	--	Yes	--	--	--	--	--	--	--
G4ST01010654734	chr1:10654735-10654768	-	GGGTGAGCCGGGGAGGCCTCTCGGGCAAGCAGGG<br>(((....)))	CASZ1	NM_017766	chr1:10629857-10779294	non-template	34	Intron_4	--	Yes	--	Yes	--	--	--	--	--	--	--
G4ST01010654743	chr1:10654744-10654772	-	GGGCGGGTGAGCCGGGGAGGCCTCTCGGG<br>(((....)))	CASZ1	NM_001079843	chr1:10619253-10779294	non-template	29	Intron_4	--	Yes	--	Yes	--	--	--	--	--	--	--
G4ST01010654743	chr1:10654744-10654772	-	GGGCGGGTGAGCCGGGGAGGCCTCTCGGG<br>(((....)))	CASZ1	NM_017766	chr1:10629857-10779294	non-template	29	Intron_4	--	Yes	--	Yes	--	--	--	--	--	--	--
G4ST01010659928	chr1:10659929-10659961	+	GGGGGCAGGGGAAGGCACCTTCAGGGTCAGGGG<br>((((....)))).	CASZ1	NM_001079843	chr1:10619253-10779294	template	33	Intron_4	--	Yes	--	Yes	--	--	--	--	--	--	--
G4ST01010659928	chr1:10659929-10659961	+	GGGGGCAGGGGAAGGCACCTTCAGGGTCAGGGG<br>((((....)))).	CASZ1	NM_017766	chr1:10629857-10779294	template	33	Intron_4	--	Yes	--	Yes	--	--	--	--	--	--	--
G4ST01010660701	chr1:10660702-10660738	+	GGGGAGACAGGGCAGGCGCTCTCTGGGGATGAGAGGG<br>((((.....))))	CASZ1	NM_001079843	chr1:10619253-10779294	template	37	Intron_4	--	Yes	--	Yes	--	--	--	--	--	--	--
G4ST01010660701	chr1:10660702-10660738	+	GGGGAGACAGGGCAGGCGCTCTCTGGGGATGAGAGGG<br>((((.....))))	CASZ1	NM_017766	chr1:10629857-10779294	template	37	Intron_4	--	Yes	--	Yes	--	--	--	--	--	--	--
G4ST01010661626	chr1:10661627-10661656	-	GGGCTGGCTCACAGCGGGCGGGCAGACGGG<br>(((.....))).	CASZ1	NM_001079843	chr1:10619253-10779294	non-template	30	Intron_4	--	Yes	--	Yes	--	--	--	--	--	--	--
G4ST01010661626	chr1:10661627-10661656	-	GGGCTGGCTCACAGCGGGCGGGCAGACGGG<br>(((.....))).	CASZ1	NM_017766	chr1:10629857-10779294	non-template	30	Intron_4	--	Yes	--	Yes	--	--	--	--	--	--	--
G4ST01010661634	chr1:10661635-10661661	-	GGGGTGGGCTGGCTCACAGCGGGCGGG<br>(((.....))).	CASZ1	NM_001079843	chr1:10619253-10779294	non-template	27	Intron_4	--	Yes	--	Yes	--	--	--	--	--	--	--
G4ST01010661634	chr1:10661635-10661661	-	GGGGTGGGCTGGCTCACAGCGGGCGGG<br>(((.....))).	CASZ1	NM_017766	chr1:10629857-10779294	non-template	27	Intron_4	--	Yes	--	Yes	--	--	--	--	--	--	--
G4ST01010664425	chr1:10664426-10664463	+	GGGGCTCCAGGAACCTTTCCTGGAGGGGTGGGGTGGGG<br>.((((((((.....))))))))	CASZ1	NM_001079843	chr1:10619253-10779294	template	38	Intron_4	--	Yes	--	Yes	--	--	--	Yes	--	--	Yes
G4ST01010664425	chr1:10664426-10664463	+	GGGGCTCCAGGAACCTTTCCTGGAGGGGTGGGGTGGGG<br>.((((((((.....))))))))	CASZ1	NM_017766	chr1:10629857-10779294	template	38	Intron_4	--	Yes	--	Yes	--	--	--	Yes	--	--	Yes
G4ST01010668363	chr1:10668364-10668403	-	GGGGGAGGCTGGGGTCTTCCTGTTGCCAGGAAGGGTGGGG<br>...((((((....))))))	CASZ1	NM_001079843	chr1:10619253-10779294	non-template	40	Intron_4	--	Yes	--	Yes	--	--	--	--	Yes	--	--
G4ST01010668363	chr1:10668364-10668403	-	GGGGGAGGCTGGGGTCTTCCTGTTGCCAGGAAGGGTGGGG<br>...((((((....))))))	CASZ1	NM_017766	chr1:10629857-10779294	non-template	40	Intron_4	--	Yes	--	Yes	--	--	--	--	Yes	--	--
G4ST01010669120	chr1:10669121-10669161	+	GGGGACCAAGGGCTGGGGACAGTTCTAAGCCGAGGACTGGG<br>...((((((......))))))	CASZ1	NM_001079843	chr1:10619253-10779294	template	41	Intron_4	--	Yes	--	Yes	--	--	--	Yes	Yes	--	--
G4ST01010669120	chr1:10669121-10669161	+	GGGGACCAAGGGCTGGGGACAGTTCTAAGCCGAGGACTGGG<br>...((((((......))))))	CASZ1	NM_017766	chr1:10629857-10779294	template	41	Intron_4	--	Yes	--	Yes	--	--	--	Yes	Yes	--	--
G4ST01010669129	chr1:10669130-10669169	+	GGGCTGGGGACAGTTCTAAGCCGAGGACTGGGTCTTGGGG<br>...((((((......))))))	CASZ1	NM_001079843	chr1:10619253-10779294	template	40	Intron_4	--	Yes	--	Yes	--	--	--	Yes	Yes	--	--

G4ST01010669129	chr1:10669130-10669169	+	GGGCTGGGGACAGTTCTAAGCCGAGGACTGGGTCTTGGGG<br>...((((((......))))))	CASZ1	NM_017766	chr1:10629857-10779294	template	40	Intron_4	--	Yes	--	Yes	--	--	--	Yes	Yes	--	--
G4ST01010669134	chr1:10669135-10669178	+	GGGGACAGTTCTAAGCCGAGGACTGGGTCTTGGGGACACTCGGG<br>...((((((......))))))	CASZ1	NM_001079843	chr1:10619253-10779294	template	44	Intron_4	--	Yes	--	Yes	--	--	--	Yes	Yes	--	--
G4ST01010669134	chr1:10669135-10669178	+	GGGGACAGTTCTAAGCCGAGGACTGGGTCTTGGGGACACTCGGG<br>...((((((......))))))	CASZ1	NM_017766	chr1:10629857-10779294	template	44	Intron_4	--	Yes	--	Yes	--	--	--	Yes	Yes	--	--
G4ST01010676633	chr1:10676634-10676666	-	GGGCCGCTCCTGCGGGGCGGGGGACAAGCAGGG<br>.(((....)))	CASZ1	NM_001079843	chr1:10619253-10779294	non-template	33	Intron_3	--	Yes	--	Yes	--	--	--	Yes	Yes	Yes	Yes
G4ST01010676633	chr1:10676634-10676666	-	GGGCCGCTCCTGCGGGGCGGGGGACAAGCAGGG<br>.(((....)))	CASZ1	NM_017766	chr1:10629857-10779294	non-template	33	Intron_3	--	Yes	--	Yes	--	--	--	Yes	Yes	Yes	Yes
G4ST01010676643	chr1:10676644-10676671	-	GGGACGGGCCGCTCCTGCGGGGCGGGGG<br>.(((....)))	CASZ1	NM_001079843	chr1:10619253-10779294	non-template	28	Intron_3	--	Yes	--	Yes	--	--	--	Yes	Yes	Yes	Yes
G4ST01010676643	chr1:10676644-10676671	-	GGGACGGGCCGCTCCTGCGGGGCGGGGG<br>.(((....)))	CASZ1	NM_017766	chr1:10629857-10779294	non-template	28	Intron_3	--	Yes	--	Yes	--	--	--	Yes	Yes	Yes	Yes
G4ST01010678231	chr1:10678232-10678266	-	GGGGACAGGCCTGGAGACGGCGTGGGGCGGGGGGG<br>..((.(((.(....)))).))	CASZ1	NM_001079843	chr1:10619253-10779294	non-template	35	Intron_3	--	Yes	--	Yes	--	--	--	Yes	--	--	Yes
G4ST01010678231	chr1:10678232-10678266	-	GGGGACAGGCCTGGAGACGGCGTGGGGCGGGGGGG<br>..((.(((.(....)))).))	CASZ1	NM_017766	chr1:10629857-10779294	non-template	35	Intron_3	--	Yes	--	Yes	--	--	--	Yes	--	--	Yes
G4ST01010687827	chr1:10687828-10687865	-	GGGCAGTGTGGGGCAGTGGGGCACCTGTGGTGGCTGGG<br>.((((...))))...	CASZ1	NM_001079843	chr1:10619253-10779294	non-template	38	Intron_3	--	Yes	--	Yes	--	--	--	--	--	--	--
G4ST01010687827	chr1:10687828-10687865	-	GGGCAGTGTGGGGCAGTGGGGCACCTGTGGTGGCTGGG<br>.((((...))))...	CASZ1	NM_017766	chr1:10629857-10779294	non-template	38	Intron_3	--	Yes	--	Yes	--	--	--	--	--	--	--
G4ST01010699897	chr1:10699898-10699932	-	GGGGAGCTGCAGAGGCTGGGGGTGGGGTGAGTGGG<br>.((((.....))))..	CASZ1	NM_001079843	chr1:10619253-10779294	non-template	35	Intron_2	--	Yes	--	Yes	--	--	--	Yes	Yes	--	Yes
G4ST01010699897	chr1:10699898-10699932	-	GGGGAGCTGCAGAGGCTGGGGGTGGGGTGAGTGGG<br>.((((.....))))..	CASZ1	NM_017766	chr1:10629857-10779294	non-template	35	Intron_2	--	Yes	--	Yes	--	--	--	Yes	Yes	--	Yes
G4ST01010699905	chr1:10699906-10699940	-	GGGGAACTGGGGAGCTGCAGAGGCTGGGGGTGGGG<br>.((((.....))))..	CASZ1	NM_001079843	chr1:10619253-10779294	non-template	35	Intron_2	--	Yes	--	Yes	--	--	--	Yes	Yes	--	Yes
G4ST01010699905	chr1:10699906-10699940	-	GGGGAACTGGGGAGCTGCAGAGGCTGGGGGTGGGG<br>.((((.....))))..	CASZ1	NM_017766	chr1:10629857-10779294	non-template	35	Intron_2	--	Yes	--	Yes	--	--	--	Yes	Yes	--	Yes
G4ST01010699910	chr1:10699911-10699949	-	GGGAAGTTTGGGGAACTGGGGAGCTGCAGAGGCTGGGGG<br>.((((.....))))..	CASZ1	NM_001079843	chr1:10619253-10779294	non-template	39	Intron_2	--	Yes	--	Yes	--	--	--	Yes	Yes	--	Yes
G4ST01010699910	chr1:10699911-10699949	-	GGGAAGTTTGGGGAACTGGGGAGCTGCAGAGGCTGGGGG<br>.((((.....))))..	CASZ1	NM_017766	chr1:10629857-10779294	non-template	39	Intron_2	--	Yes	--	Yes	--	--	--	Yes	Yes	--	Yes
G4ST01010707357	chr1:10707358-10707396	+	GGGCACTGCCCAGCCAGAGGCAGGGGCAAGGGCCCTGGG<br>..(((((........)))))	CASZ1	NM_001079843	chr1:10619253-10779294	template	39	Intron_2	--	Yes	--	Yes	--	--	--	--	--	--	Yes
G4ST01010707357	chr1:10707358-10707396	+	GGGCACTGCCCAGCCAGAGGCAGGGGCAAGGGCCCTGGG<br>..(((((........)))))	CASZ1	NM_017766	chr1:10629857-10779294	template	39	Intron_2	--	Yes	--	Yes	--	--	--	--	--	--	Yes
G4ST01010709859	chr1:10709860-10709896	+	GGGAAGCTGGGAGACCAGGGCGTCTTGCAGGCAGGGG<br>.((((...))))..	CASZ1	NM_001079843	chr1:10619253-10779294	template	37	Intron_2	--	Yes	--	Yes	--	--	--	--	--	--	--
G4ST01010709859	chr1:10709860-10709896	+	GGGAAGCTGGGAGACCAGGGCGTCTTGCAGGCAGGGG<br>.((((...))))..	CASZ1	NM_017766	chr1:10629857-10779294	template	37	Intron_2	--	Yes	--	Yes	--	--	--	--	--	--	--
G4ST01010719521	chr1:10719522-10719558	+	GGGTGGGAGGTGCCCGGGCCTGCAGGTGCTCCTGGGG<br>.((...)) ....((((....))))	CASZ1	NM_001079843	chr1:10619253-10779294	template	37	Intron_2	--	Yes	--	Yes	--	--	--	--	--	--	--
G4ST01010719521	chr1:10719522-10719558	+	GGGTGGGAGGTGCCCGGGCCTGCAGGTGCTCCTGGGG<br>.((...)) ....((((....))))	CASZ1	NM_017766	chr1:10629857-10779294	template	37	Intron_2	--	Yes	--	Yes	--	--	--	--	--	--	--
G4ST01010719525	chr1:10719526-10719565	+	GGGAGGTGCCCGGGCCTGCAGGTGCTCCTGGGGCCGTGGG<br>.((...)) ....((((....))))	CASZ1	NM_001079843	chr1:10619253-10779294	template	40	Intron_2	--	Yes	--	Yes	--	--	--	--	--	--	--
G4ST01010719525	chr1:10719526-10719565	+	GGGAGGTGCCCGGGCCTGCAGGTGCTCCTGGGGCCGTGGG<br>.((...)) ....((((....))))	CASZ1	NM_017766	chr1:10629857-10779294	template	40	Intron_2	--	Yes	--	Yes	--	--	--	--	--	--	--
G4ST01010719536	chr1:10719537-10719570	+	GGGCCTGCAGGTGCTCCTGGGGCCGTGGGCTGGG<br>....((((....))))	CASZ1	NM_001079843	chr1:10619253-10779294	template	34	Intron_2	--	Yes	--	Yes	--	--	--	--	--	--	--
G4ST01010719536	chr1:10719537-10719570	+	GGGCCTGCAGGTGCTCCTGGGGCCGTGGGCTGGG<br>....((((....))))	CASZ1	NM_017766	chr1:10629857-10779294	template	34	Intron_2	--	Yes	--	Yes	--	--	--	--	--	--	--
G4ST01010720245	chr1:10720246-10720283	+	GGGTGCCCAAGGGAGTTACATGGCTGTAAGGGTTGGGG<br>..(((((....)))))	CASZ1	NM_001079843	chr1:10619253-10779294	template	38	Intron_2	--	Yes	--	Yes	--	--	--	--	--	--	Yes
G4ST01010720245	chr1:10720246-10720283	+	GGGTGCCCAAGGGAGTTACATGGCTGTAAGGGTTGGGG<br>..(((((....)))))	CASZ1	NM_017766	chr1:10629857-10779294	template	38	Intron_2	--	Yes	--	Yes	--	--	--	--	--	--	Yes
G4ST01010720255	chr1:10720256-10720291	+	GGGAGTTACATGGCTGTAAGGGTTGGGGTCTGTGGG<br>..(((((....)))))	CASZ1	NM_001079843	chr1:10619253-10779294	template	36	Intron_2	--	Yes	--	Yes	--	--	--	--	--	--	Yes
G4ST01010720255	chr1:10720256-10720291	+	GGGAGTTACATGGCTGTAAGGGTTGGGGTCTGTGGG<br>..(((((....)))))	CASZ1	NM_017766	chr1:10629857-10779294	template	36	Intron_2	--	Yes	--	Yes	--	--	--	--	--	--	Yes
G4ST01010720511	chr1:10720512-10720545	+	GGGAAGGCTGGGCACAGCCCTGTGGGGCGCTGGG<br>((((....))))	CASZ1	NM_001079843	chr1:10619253-10779294	template	34	Intron_2	--	Yes	--	Yes	--	--	--	--	--	--	--
G4ST01010720511	chr1:10720512-10720545	+	GGGAAGGCTGGGCACAGCCCTGTGGGGCGCTGGG<br>((((....))))	CASZ1	NM_017766	chr1:10629857-10779294	template	34	Intron_2	--	Yes	--	Yes	--	--	--	--	--	--	--
G4ST01010720885	chr1:10720886-10720926	+	GGGAACAGGGCCGCCTGGGCCTTCCAGGTGAGGAAGGCGGG<br>((((((......)))))).	CASZ1	NM_001079843	chr1:10619253-10779294	template	41	Intron_2	--	Yes	--	Yes	--	--	--	--	--	--	--
G4ST01010720885	chr1:10720886-10720926	+	GGGAACAGGGCCGCCTGGGCCTTCCAGGTGAGGAAGGCGGG<br>((((((......)))))).	CASZ1	NM_017766	chr1:10629857-10779294	template	41	Intron_2	--	Yes	--	Yes	--	--	--	--	--	--	--
G4ST01010720892	chr1:10720893-10720933	+	GGGCCGCCTGGGCCTTCCAGGTGAGGAAGGCGGGAATGGGG<br>((((((......)))))).	CASZ1	NM_001079843	chr1:10619253-10779294	template	41	Intron_2	--	Yes	--	Yes	--	--	--	--	--	--	--
G4ST01010720892	chr1:10720893-10720933	+	GGGCCGCCTGGGCCTTCCAGGTGAGGAAGGCGGGAATGGGG<br>((((((......)))))).	CASZ1	NM_017766	chr1:10629857-10779294	template	41	Intron_2	--	Yes	--	Yes	--	--	--	--	--	--	--
G4ST01010722143	chr1:10722144-10722186	-	GGGGGGCGGAGGGCTCAGGGAAAGGTCTGGAAGCCTGAGTGGG<br>..(((.((...))))))....	CASZ1	NM_001079843	chr1:10619253-10779294	non-template	43	Intron_2	--	Yes	--	Yes	--	--	--	--	--	--	Yes
G4ST01010722143	chr1:10722144-10722186	-	GGGGGGCGGAGGGCTCAGGGAAAGGTCTGGAAGCCTGAGTGGG<br>..(((.((...))))))....	CASZ1	NM_017766	chr1:10629857-10779294	non-template	43	Intron_2	--	Yes	--	Yes	--	--	--	--	--	--	Yes
G4ST01010729872	chr1:10729873-10729906	+	GGGGCTGGGAAGCAAAGGGGAAGCCGTCCTTGGG<br>.(((.....)))	CASZ1	NM_001079843	chr1:10619253-10779294	template	34	Intron_2	--	Yes	--	Yes	--	--	--	--	--	--	Yes
G4ST01010729872	chr1:10729873-10729906	+	GGGGCTGGGAAGCAAAGGGGAAGCCGTCCTTGGG<br>.(((.....)))	CASZ1	NM_017766	chr1:10629857-10779294	template	34	Intron_2	--	Yes	--	Yes	--	--	--	--	--	--	Yes
G4ST01010729878	chr1:10729879-10729915	+	GGGAAGCAAAGGGGAAGCCGTCCTTGGGAAGGTGGGG<br>.(((.....)))	CASZ1	NM_001079843	chr1:10619253-10779294	template	37	Intron_2	--	Yes	--	Yes	--	--	--	--	--	--	Yes
G4ST01010729878	chr1:10729879-10729915	+	GGGAAGCAAAGGGGAAGCCGTCCTTGGGAAGGTGGGG<br>.(((.....)))	CASZ1	NM_017766	chr1:10629857-10779294	template	37	Intron_2	--	Yes	--	Yes	--	--	--	--	--	--	Yes
G4ST01010734413	chr1:10734414-10734453	+	GGGCAGCCCAGGGGGCCAGGGCAGAGGAGGCCTCCCAGGG<br>..((((...))))...	CASZ1	NM_001079843	chr1:10619253-10779294	template	40	Intron_2	--	Yes	--	Yes	--	--	--	--	--	--	Yes
G4ST01010734413	chr1:10734414-10734453	+	GGGCAGCCCAGGGGGCCAGGGCAGAGGAGGCCTCCCAGGG<br>..((((...))))...	CASZ1	NM_017766	chr1:10629857-10779294	template	40	Intron_2	--	Yes	--	Yes	--	--	--	--	--	--	Yes
G4ST01010735993	chr1:10735994-10736018	-	GGGTGAGGGAGGAGGCTGGGCAGGG<br>((....))	CASZ1	NM_001079843	chr1:10619253-10779294	non-template	25	Intron_2	--	Yes	--	Yes	--	--	--	Yes	--	--	--
G4ST01010735993	chr1:10735994-10736018	-	GGGTGAGGGAGGAGGCTGGGCAGGG<br>((....))	CASZ1	NM_017766	chr1:10629857-10779294	non-template	25	Intron_2	--	Yes	--	Yes	--	--	--	Yes	--	--	--
G4ST01010737166	chr1:10737167-10737209	-	GGGGGCAGGACTGAGGAAGCCTGGGGTCCAGTAGGGTCAGGGG<br>..((((.((.....))))))	CASZ1	NM_001079843	chr1:10619253-10779294	non-template	43	Intron_2	--	Yes	--	Yes	--	--	--	--	--	--	--
G4ST01010737166	chr1:10737167-10737209	-	GGGGGCAGGACTGAGGAAGCCTGGGGTCCAGTAGGGTCAGGGG<br>..((((.((.....))))))	CASZ1	NM_017766	chr1:10629857-10779294	non-template	43	Intron_2	--	Yes	--	Yes	--	--	--	--	--	--	--
G4ST01010738681	chr1:10738682-10738711	-	GGGCCGGCCCTGGGGGAGGGTGTGTCCGGG<br>(((....)))	CASZ1	NM_001079843	chr1:10619253-10779294	non-template	30	Intron_2	--	Yes	--	Yes	--	--	--	Yes	Yes	Yes	Yes

G4ST01010738681	chr1:10738682-10738711	-	GGGCCGGCCCTGGGGGAGGGTGTGTCCGGG<br>(((....)))	CASZ1	NM_017766	chr1:10629857-10779294	non-template	30	Intron_2	--	Yes	--	Yes	--	--	--	Yes	Yes	Yes	Yes
G4ST01010753347	chr1:10753348-10753372	-	GGGGTGGGGCTGGGAGGAAGCTGGG<br>((....))	CASZ1	NM_001079843	chr1:10619253-10779294	non-template	25	Intron_1.f	--	Yes	--	Yes	--	--	--	--	--	--	--
G4ST01010753347	chr1:10753348-10753372	-	GGGGTGGGGCTGGGAGGAAGCTGGG<br>((....))	CASZ1	NM_017766	chr1:10629857-10779294	non-template	25	Intron_1.f	--	Yes	--	Yes	--	--	--	--	--	--	--
G4ST01010758973	chr1:10758974-10759000	-	GGGGGCTTGGGAAGGGCAGAGCCTGGG<br>.((...))	CASZ1	NM_001079843	chr1:10619253-10779294	non-template	27	Intron_1.f	--	Yes	--	Yes	--	--	--	--	--	--	--
G4ST01010758973	chr1:10758974-10759000	-	GGGGGCTTGGGAAGGGCAGAGCCTGGG<br>.((...))	CASZ1	NM_017766	chr1:10629857-10779294	non-template	27	Intron_1.f	--	Yes	--	Yes	--	--	--	--	--	--	--
G4ST01010765381	chr1:10765382-10765412	+	GGGAAGGGGAGTGGGCCTGGTCACCTGGGGG<br>((.((...)).))	CASZ1	NM_001079843	chr1:10619253-10779294	template	31	Intron_1.f	--	Yes	--	Yes	--	--	--	--	--	--	--
G4ST01010765381	chr1:10765382-10765412	+	GGGAAGGGGAGTGGGCCTGGTCACCTGGGGG<br>((.((...)).))	CASZ1	NM_017766	chr1:10629857-10779294	template	31	Intron_1.f	--	Yes	--	Yes	--	--	--	--	--	--	--
G4ST01010765386	chr1:10765387-10765418	+	GGGGAGTGGGCCTGGTCACCTGGGGGAGGGGG<br>((.((...)).))	CASZ1	NM_001079843	chr1:10619253-10779294	template	32	Intron_1.f	--	Yes	--	Yes	--	--	--	--	--	--	--
G4ST01010765386	chr1:10765387-10765418	+	GGGGAGTGGGCCTGGTCACCTGGGGGAGGGGG<br>((.((...)).))	CASZ1	NM_017766	chr1:10629857-10779294	template	32	Intron_1.f	--	Yes	--	Yes	--	--	--	--	--	--	--
G4ST01010765393	chr1:10765394-10765423	+	GGGCCTGGTCACCTGGGGGAGGGGGCTGGG<br>((.((...)).))	CASZ1	NM_001079843	chr1:10619253-10779294	template	30	Intron_1.f	--	Yes	--	Yes	--	--	--	--	--	--	--
G4ST01010765393	chr1:10765394-10765423	+	GGGCCTGGTCACCTGGGGGAGGGGGCTGGG<br>((.((...)).))	CASZ1	NM_017766	chr1:10629857-10779294	template	30	Intron_1.f	--	Yes	--	Yes	--	--	--	--	--	--	--
G4ST01010769458	chr1:10769459-10769506	-	GGGAACTTGGGGGTGAATTTGGGGGCTGCCTTTCAGGCAGAATGGGGG<br>..((((((...))))))....	CASZ1	NM_001079843	chr1:10619253-10779294	non-template	48	Intron_1.f	--	Yes	--	Yes	--	--	--	--	--	--	--
G4ST01010769458	chr1:10769459-10769506	-	GGGAACTTGGGGGTGAATTTGGGGGCTGCCTTTCAGGCAGAATGGGGG<br>..((((((...))))))....	CASZ1	NM_017766	chr1:10629857-10779294	non-template	48	Intron_1.f	--	Yes	--	Yes	--	--	--	--	--	--	--
G4ST01010769990	chr1:10769991-10770019	-	GGGGATCGGGGGACGGGGTGAGACACGGG<br>(((...)))	CASZ1	NM_001079843	chr1:10619253-10779294	non-template	29	Intron_1.f	--	Yes	--	Yes	--	--	--	--	--	--	--
G4ST01010769990	chr1:10769991-10770019	-	GGGGATCGGGGGACGGGGTGAGACACGGG<br>(((...)))	CASZ1	NM_017766	chr1:10629857-10779294	non-template	29	Intron_1.f	--	Yes	--	Yes	--	--	--	--	--	--	--
G4ST01010777536	chr1:10777537-10777568	-	GGGGCCGCTGGCGGGGGCTGGCCGGGCGCGGG<br>.(((....)))	CASZ1	NM_001079843	chr1:10619253-10779294	non-template	32	Intron_1.f	--	Yes	--	Yes	--	--	--	Yes	--	--	Yes
G4ST01010777536	chr1:10777537-10777568	-	GGGGCCGCTGGCGGGGGCTGGCCGGGCGCGGG<br>.(((....)))	CASZ1	NM_017766	chr1:10629857-10779294	non-template	32	Intron_1.f	--	Yes	--	Yes	--	--	--	Yes	--	--	Yes
G4ST01010777594	chr1:10777595-10777626	-	GGGTCTGGAAGGGGAGGGGGCGGCCGCCGGGG<br>(((....))).	CASZ1	NM_001079843	chr1:10619253-10779294	non-template	32	Intron_1.f	--	Yes	--	Yes	--	--	--	--	--	--	Yes
G4ST01010777594	chr1:10777595-10777626	-	GGGTCTGGAAGGGGAGGGGGCGGCCGCCGGGG<br>(((....))).	CASZ1	NM_017766	chr1:10629857-10779294	non-template	32	Intron_1.f	--	Yes	--	Yes	--	--	--	--	--	--	Yes
G4ST01010778896	chr1:10778897-10778934	-	GGGGAATGCCCGGCGCGCGGCTCCGGGGGAGGGGAGGG<br>......((((.((...)).))))	CASZ1	NM_001079843	chr1:10619253-10779294	non-template	38	Intron_1.f	--	Yes	--	Yes	--	--	--	Yes	--	--	--
G4ST01010778896	chr1:10778897-10778934	-	GGGGAATGCCCGGCGCGCGGCTCCGGGGGAGGGGAGGG<br>......((((.((...)).))))	CASZ1	NM_017766	chr1:10629857-10779294	non-template	38	Intron_1.f	--	Yes	--	Yes	--	--	--	Yes	--	--	--
G4ST01010779144	chr1:10779145-10779177	+	GGGAGGCCCGCGGAGCTGGGGGACGTGGGGGGG<br>...((.((...)).))	CASZ1	NM_001079843	chr1:10619253-10779294	template	33	Intron_1.f	--	Yes	--	Yes	--	--	--	Yes	Yes	Yes	--
G4ST01010779144	chr1:10779145-10779177	+	GGGAGGCCCGCGGAGCTGGGGGACGTGGGGGGG<br>...((.((...)).))	CASZ1	NM_017766	chr1:10629857-10779294	template	33	Intron_1.f	--	Yes	--	Yes	--	--	--	Yes	Yes	Yes	--
G4ST01010779161	chr1:10779162-10779190	+	GGGGGACGTGGGGGGGCCGCGGCGGAGGG<br>(((...))).	CASZ1	NM_001079843	chr1:10619253-10779294	template	29	Intron_1.f	--	Yes	--	Yes	--	--	--	--	Yes	Yes	--
G4ST01010779161	chr1:10779162-10779190	+	GGGGGACGTGGGGGGGCCGCGGCGGAGGG<br>(((...))).	CASZ1	NM_017766	chr1:10629857-10779294	template	29	Intron_1.f	--	Yes	--	Yes	--	--	--	--	Yes	Yes	--
G4ST01010929336	chr1:10929337-10929376	+	GGGTCACTGAGGGTCACTGGGGCTGTCCAAGCCAGCGGGG<br>((((.......)))).	C1orf127	NM_001170754	chr1:10929117-10964681	template	40	Exon_13.l	--	Yes	Yes	--	--	--	Yes	--	--	--	Yes
G4ST01010953929	chr1:10953930-10953961	-	GGGGCAGGATGGGCAGAGGCTGGGGTCAGGGG<br>(((...)))	C1orf127	NM_001170754	chr1:10929117-10964681	non-template	32	Intron_2	--	Yes	--	Yes	--	--	--	--	--	--	--
G4ST01011027686	chr1:11027687-11027714	-	GGGACGGGCCAGTGCTCTGGGGCGTGGG<br>.(((....)))	MASP2	NM_139208	chr1:11027442-11029883	non-template	28	Intron_4.l	--	Yes	--	Yes	--	--	--	--	--	--	--
G4ST01011027686	chr1:11027687-11027714	-	GGGACGGGCCAGTGCTCTGGGGCGTGGG<br>.(((....)))	MASP2	NM_006610	chr1:11009167-11029883	non-template	28	Intron_4	--	Yes	--	Yes	--	--	--	--	--	--	--
G4ST01011027889	chr1:11027890-11027929	+	GGGGAAGCAGGGCTGAGAAGGGGAGCTGGAGGCAGCAGGG<br>..((((....)))).	MASP2	NM_139208	chr1:11027442-11029883	template	40	Intron_4.l	--	Yes	--	Yes	--	--	--	--	--	--	--
G4ST01011027889	chr1:11027890-11027929	+	GGGGAAGCAGGGCTGAGAAGGGGAGCTGGAGGCAGCAGGG<br>..((((....)))).	MASP2	NM_006610	chr1:11009167-11029883	template	40	Intron_4	--	Yes	--	Yes	--	--	--	--	--	--	--
G4ST01011029427	chr1:11029428-11029466	-	GGGACACCTGGGAGCAGGGGCCAGGCTTGGCCAGGAGGG<br>.((.(((...))).)).	MASP2	NM_139208	chr1:11027442-11029883	non-template	39	Intron_2	--	Yes	--	Yes	--	--	--	--	Yes	--	Yes
G4ST01011029427	chr1:11029428-11029466	-	GGGACACCTGGGAGCAGGGGCCAGGCTTGGCCAGGAGGG<br>.((.(((...))).)).	MASP2	NM_006610	chr1:11009167-11029883	non-template	39	Intron_2	--	Yes	--	Yes	--	--	--	--	Yes	--	Yes
G4ST01011041583	chr1:11041584-11041616	-	GGGGTCAGGGGGTCTGCTTCAGGGGCCTGGGGG<br>...(((...)))	SRM	NM_003132	chr1:11037236-11042678	non-template	33	Intron_2	--	Yes	--	Yes	--	--	--	Yes	--	--	--
G4ST01011041591	chr1:11041592-11041624	-	GGGTCCGAGGGGTCAGGGGGTCTGCTTCAGGGG<br>...(((...)))	SRM	NM_003132	chr1:11037236-11042678	non-template	33	Intron_2	--	Yes	--	Yes	--	--	--	Yes	--	--	--
G4ST01011042548	chr1:11042549-11042581	+	GGGGCCGGAGGCGGCGGGGCCGTCGGGGCCGGG<br>((((....)))).	SRM	NM_003132	chr1:11037236-11042678	template	33	Exon_1.f	--	Yes	Yes	--	Yes	--	--	Yes	Yes	Yes	--
G4ST01011042693	chr1:11042694-11042722	+	GGGCGCGGGGCGGGGCCTGCCGGCGGGGG<br>(((....)))...	SRM	NM_003132	chr1:11037236-11042678	template	-15	upstream	Yes	--	--	--	--	--	--	Yes	Yes	Yes	--
G4ST01011096496	chr1:11096497-11096532	+	GGGAGGGACCTCATGGGAGGTGATTAGATCATGGGG<br>..((((.....)))).	MTOR	NM_004958	chr1:11089175-11245195	template	36	Intron_53	--	Yes	--	Yes	--	--	--	--	--	--	--
G4ST01011244853	chr1:11244854-11244887	+	GGGCGGACGTACCGCGGGCCGAAGGGTCCAGGGG<br>(((.....))).	MTOR	NM_004958	chr1:11089175-11245195	template	34	Intron_1.f	--	Yes	--	Yes	--	--	--	--	Yes	--	Yes
G4ST01011255553	chr1:11255554-11255580	-	GGGGACGGGCCAGGAGCTGGGGCCGGG<br>.(((...)))	UBIAD1	NM_013319	chr1:11255842-11271078	template	-261	upstream	Yes	--	--	--	--	--	--	Yes	Yes	Yes	--
G4ST01011255558	chr1:11255559-11255586	-	GGGAGCGGGGACGGGCCAGGAGCTGGGG<br>.(((...)))	UBIAD1	NM_013319	chr1:11255842-11271078	template	-255	upstream	Yes	--	--	--	--	--	--	Yes	Yes	Yes	--
G4ST01011255710	chr1:11255711-11255741	-	GGGGGCCTGGTCAGAGGCGGGGCGGGCAGGG<br>.((((......)))).	UBIAD1	NM_013319	chr1:11255842-11271078	template	-100	upstream	Yes	--	--	--	--	--	--	Yes	Yes	Yes	Yes
G4ST01011255715	chr1:11255716-11255748	-	GGGGGGCGGGGGCCTGGTCAGAGGCGGGGCGGG<br>.((((......)))).	UBIAD1	NM_013319	chr1:11255842-11271078	template	-93	upstream	Yes	--	--	--	--	--	--	Yes	Yes	Yes	Yes
G4ST01011255719	chr1:11255720-11255753	-	GGGGTGGGGGGCGGGGGCCTGGTCAGAGGCGGGG<br>.((((......)))).	UBIAD1	NM_013319	chr1:11255842-11271078	template	-88	upstream	Yes	--	--	--	--	--	--	Yes	Yes	Yes	Yes
G4ST01011269643	chr1:11269644-11269683	-	GGGTAGGTGTGGGGAGCATCAGAAGATGCAATAGGGAGGG<br>..(((((....)))))....	UBIAD1	NM_013319	chr1:11255842-11271078	template	40	Exon_2.l	--	Yes	Yes	--	--	--	Yes	Yes	--	--	--
G4ST01011461380	chr1:11461381-11461408	+	GGGAGCGCGGGCCCGGGAGAGACTTGGG<br>((...)).	PTCHD2	NM_020780	chr1:11461882-11520227	non-template	-473	upstream	Yes	--	--	--	--	--	--	Yes	Yes	--	--
G4ST01011462650	chr1:11462651-11462684	+	GGGCAGCCGGGCAGTGGCGGCGCTGAGGGTCGGG<br>(((((....))))).	PTCHD2	NM_020780	chr1:11461882-11520227	non-template	34	Intron_1.f	--	Yes	--	Yes	--	--	--	--	Yes	--	--
G4ST01011482697	chr1:11482698-11482725	-	GGGCCTGGCAGGAGAGGGCAGGGCTGGG<br>(((...)))...	PTCHD2	NM_020780	chr1:11461882-11520227	template	28	Intron_1.f	--	Yes	--	Yes	--	--	--	Yes	--	--	--
G4ST01011503942	chr1:11503943-11503970	+	GGGAAGGGGTGGGGTTAACCAGGCTGGG<br>(((.....))).	PTCHD2	NM_020780	chr1:11461882-11520227	non-template	28	Intron_10	--	Yes	--	Yes	--	--	--	--	--	--	Yes
G4ST01011504025	chr1:11504026-11504053	+	GGGGTGGGGTTAGCCAGGCTGGGAGGGG<br>...(((...)))	PTCHD2	NM_020780	chr1:11461882-11520227	non-template	28	Intron_10	--	Yes	--	Yes	--	--	--	--	--	--	Yes

G4ST01011504030	chr1:11504031-11504062	+	GGGGTTAGCCAGGCTGGGAGGGGAGAGGAGGG<br>...(((...)))	PTCHD2	NM_020780	chr1:11461882-11520227	non-template	32	Intron_10	--	Yes	--	Yes	--	--	--	--	--	--	Yes
G4ST01011504049	chr1:11504050-11504082	+	GGGGAGAGGAGGGAAGGGGTTAGCCAGGCTGGG<br>...(((...)))	PTCHD2	NM_020780	chr1:11461882-11520227	non-template	33	Intron_10	--	Yes	--	Yes	--	--	--	--	--	--	Yes
G4ST01011504059	chr1:11504060-11504087	+	GGGAAGGGGTTAGCCAGGCTGGGAGGGG<br>...(((...)))	PTCHD2	NM_020780	chr1:11461882-11520227	non-template	28	Intron_10	--	Yes	--	Yes	--	--	--	--	--	--	Yes
G4ST01011504064	chr1:11504065-11504096	+	GGGGTTAGCCAGGCTGGGAGGGGAGAGGAGGG<br>...(((...)))	PTCHD2	NM_020780	chr1:11461882-11520227	non-template	32	Intron_10	--	Yes	--	Yes	--	--	--	--	--	--	Yes
G4ST01011504132	chr1:11504133-11504160	+	GGGAAAGGGTGGGGTTAGCCAGGCTGGG<br>...(((...)))	PTCHD2	NM_020780	chr1:11461882-11520227	non-template	28	Intron_10	--	Yes	--	Yes	--	--	--	Yes	--	--	Yes
G4ST01011504138	chr1:11504139-11504165	+	GGGTGGGGTTAGCCAGGCTGGGAGGGG<br>...(((...)))	PTCHD2	NM_020780	chr1:11461882-11520227	non-template	27	Intron_10	--	Yes	--	Yes	--	--	--	Yes	--	--	Yes
G4ST01011504248	chr1:11504249-11504276	+	GGGAAGGGGTGGGGTTAGCCAGGCTGGG<br>...(((...)))	PTCHD2	NM_020780	chr1:11461882-11520227	non-template	28	Intron_10	--	Yes	--	Yes	--	--	--	Yes	--	--	Yes
G4ST01011504253	chr1:11504254-11504281	+	GGGGTGGGGTTAGCCAGGCTGGGAGGGG<br>...(((...)))	PTCHD2	NM_020780	chr1:11461882-11520227	non-template	28	Intron_10	--	Yes	--	Yes	--	--	--	Yes	--	--	Yes
G4ST01011504258	chr1:11504259-11504290	+	GGGGTTAGCCAGGCTGGGAGGGGAGAGGAGGG<br>...(((...)))	PTCHD2	NM_020780	chr1:11461882-11520227	non-template	32	Intron_10	--	Yes	--	Yes	--	--	--	Yes	--	--	Yes
G4ST01011504277	chr1:11504278-11504310	+	GGGGAGAGGAGGGAAGGGGTTAGCCAGGCTGGG<br>...(((...)))	PTCHD2	NM_020780	chr1:11461882-11520227	non-template	33	Intron_10	--	Yes	--	Yes	--	--	--	Yes	--	--	Yes
G4ST01011504287	chr1:11504288-11504315	+	GGGAAGGGGTTAGCCAGGCTGGGAGGGG<br>...(((...)))	PTCHD2	NM_020780	chr1:11461882-11520227	non-template	28	Intron_10	--	Yes	--	Yes	--	--	--	Yes	--	--	Yes
G4ST01011506503	chr1:11506504-11506533	-	GGGCCCGATGGGCAGAGGGAAGAGGCTGGG<br>.((...))	PTCHD2	NM_020780	chr1:11461882-11520227	template	30	Intron_10	--	Yes	--	Yes	--	--	--	--	--	--	--
G4ST01011507995	chr1:11507996-11508030	+	GGGTGGTGGGGGGCTCTCAGGGCCACACTGGTGGG<br>(((...))).	PTCHD2	NM_020780	chr1:11461882-11520227	non-template	35	Intron_12	--	Yes	--	Yes	--	--	--	--	--	--	--
G4ST01011635819	chr1:11635820-11635858	-	GGGGAGCATCAGGTGTCCGATGGGGCCAGGGCCGGAGGG<br>...((((.((...))))))	FBXO44	NM_183413	chr1:11637501-11645971	template	-1642	upstream	Yes	--	--	--	--	--	--	--	Yes	Yes	Yes
G4ST01011635819	chr1:11635820-11635858	-	GGGGAGCATCAGGTGTCCGATGGGGCCAGGGCCGGAGGG<br>...((((.((...))))))	FBXO44	NM_183412	chr1:11637019-11645971	template	-1160	upstream	Yes	--	--	--	--	--	--	--	Yes	Yes	Yes
G4ST01011635819	chr1:11635820-11635858	-	GGGGAGCATCAGGTGTCCGATGGGGCCAGGGCCGGAGGG<br>...((((.((...))))))	FBXO44	NM_033182	chr1:11637501-11645971	template	-1642	upstream	Yes	--	--	--	--	--	--	--	Yes	Yes	Yes
G4ST01011635819	chr1:11635820-11635858	-	GGGGAGCATCAGGTGTCCGATGGGGCCAGGGCCGGAGGG<br>...((((.((...))))))	FBXO44	NM_001014765	chr1:11637501-11645971	template	-1642	upstream	Yes	--	--	--	--	--	--	--	Yes	Yes	Yes
G4ST01011635819	chr1:11635820-11635858	-	GGGGAGCATCAGGTGTCCGATGGGGCCAGGGCCGGAGGG<br>...((((.((...))))))	FBXO2	NM_012168	chr1:11631005-11637475	non-template	39	Intron_1.f	--	Yes	--	Yes	--	--	--	--	Yes	Yes	Yes
G4ST01011636166	chr1:11636167-11636197	+	GGGCTGGAGGCGGGGGAATGGGGGGTGAGGG<br>(((....)))	FBXO44	NM_183413	chr1:11637501-11645971	non-template	-1303	upstream	Yes	--	--	--	--	--	--	Yes	Yes	Yes	Yes
G4ST01011636166	chr1:11636167-11636197	+	GGGCTGGAGGCGGGGGAATGGGGGGTGAGGG<br>(((....)))	FBXO44	NM_183412	chr1:11637019-11645971	non-template	-821	upstream	Yes	--	--	--	--	--	--	Yes	Yes	Yes	Yes
G4ST01011636166	chr1:11636167-11636197	+	GGGCTGGAGGCGGGGGAATGGGGGGTGAGGG<br>(((....)))	FBXO44	NM_033182	chr1:11637501-11645971	non-template	-1303	upstream	Yes	--	--	--	--	--	--	Yes	Yes	Yes	Yes
G4ST01011636166	chr1:11636167-11636197	+	GGGCTGGAGGCGGGGGAATGGGGGGTGAGGG<br>(((....)))	FBXO44	NM_001014765	chr1:11637501-11645971	non-template	-1303	upstream	Yes	--	--	--	--	--	--	Yes	Yes	Yes	Yes
G4ST01011636166	chr1:11636167-11636197	+	GGGCTGGAGGCGGGGGAATGGGGGGTGAGGG<br>(((....)))	FBXO2	NM_012168	chr1:11631005-11637475	template	31	Intron_1.f	--	Yes	--	Yes	--	--	--	Yes	Yes	Yes	Yes
G4ST01011637117	chr1:11637118-11637142	-	GGGGCCAGAGGCGGGGCGGGGCGGG<br>(((...))).	FBXO44	NM_183413	chr1:11637501-11645971	template	-358	upstream	Yes	--	--	--	--	--	--	Yes	Yes	Yes	Yes
G4ST01011637117	chr1:11637118-11637142	-	GGGGCCAGAGGCGGGGCGGGGCGGG<br>(((...))).	FBXO44	NM_183412	chr1:11637019-11645971	template	25	Exon_1.f	--	Yes	Yes	--	--	Yes	--	Yes	Yes	Yes	Yes
G4ST01011637117	chr1:11637118-11637142	-	GGGGCCAGAGGCGGGGCGGGGCGGG<br>(((...))).	FBXO44	NM_033182	chr1:11637501-11645971	template	-358	upstream	Yes	--	--	--	--	--	--	Yes	Yes	Yes	Yes
G4ST01011637117	chr1:11637118-11637142	-	GGGGCCAGAGGCGGGGCGGGGCGGG<br>(((...))).	FBXO44	NM_001014765	chr1:11637501-11645971	template	-358	upstream	Yes	--	--	--	--	--	--	Yes	Yes	Yes	Yes
G4ST01011637117	chr1:11637118-11637142	-	GGGGCCAGAGGCGGGGCGGGGCGGG<br>(((...))).	FBXO2	NM_012168	chr1:11631005-11637475	non-template	25	Exon_1.f	--	Yes	Yes	--	--	Yes	--	Yes	Yes	Yes	Yes
G4ST01011637121	chr1:11637122-11637148	-	GGGCGCGGGGCCAGAGGCGGGGCGGGG<br>(((...))).	FBXO44	NM_183413	chr1:11637501-11645971	template	-352	upstream	Yes	--	--	--	--	--	--	Yes	Yes	Yes	Yes
G4ST01011637121	chr1:11637122-11637148	-	GGGCGCGGGGCCAGAGGCGGGGCGGGG<br>(((...))).	FBXO44	NM_183412	chr1:11637019-11645971	template	27	Exon_1.f	--	Yes	Yes	--	--	Yes	--	Yes	Yes	Yes	Yes
G4ST01011637121	chr1:11637122-11637148	-	GGGCGCGGGGCCAGAGGCGGGGCGGGG<br>(((...))).	FBXO44	NM_033182	chr1:11637501-11645971	template	-352	upstream	Yes	--	--	--	--	--	--	Yes	Yes	Yes	Yes
G4ST01011637121	chr1:11637122-11637148	-	GGGCGCGGGGCCAGAGGCGGGGCGGGG<br>(((...))).	FBXO44	NM_001014765	chr1:11637501-11645971	template	-352	upstream	Yes	--	--	--	--	--	--	Yes	Yes	Yes	Yes
G4ST01011637121	chr1:11637122-11637148	-	GGGCGCGGGGCCAGAGGCGGGGCGGGG<br>(((...))).	FBXO2	NM_012168	chr1:11631005-11637475	non-template	27	Exon_1.f	--	Yes	Yes	--	--	Yes	--	Yes	Yes	Yes	Yes
G4ST01011637126	chr1:11637127-11637157	-	GGGGCGGCCGGGCGCGGGGCCAGAGGCGGGG<br>(((...))).	FBXO44	NM_183413	chr1:11637501-11645971	template	-343	upstream	Yes	--	--	--	--	--	--	Yes	Yes	Yes	Yes
G4ST01011637126	chr1:11637127-11637157	-	GGGGCGGCCGGGCGCGGGGCCAGAGGCGGGG<br>(((...))).	FBXO44	NM_183412	chr1:11637019-11645971	template	31	Exon_1.f	--	Yes	Yes	--	--	Yes	--	Yes	Yes	Yes	Yes
G4ST01011637126	chr1:11637127-11637157	-	GGGGCGGCCGGGCGCGGGGCCAGAGGCGGGG<br>(((...))).	FBXO44	NM_033182	chr1:11637501-11645971	template	-343	upstream	Yes	--	--	--	--	--	--	Yes	Yes	Yes	Yes
G4ST01011637126	chr1:11637127-11637157	-	GGGGCGGCCGGGCGCGGGGCCAGAGGCGGGG<br>(((...))).	FBXO44	NM_001014765	chr1:11637501-11645971	template	-343	upstream	Yes	--	--	--	--	--	--	Yes	Yes	Yes	Yes
G4ST01011637126	chr1:11637127-11637157	-	GGGGCGGCCGGGCGCGGGGCCAGAGGCGGGG<br>(((...))).	FBXO2	NM_012168	chr1:11631005-11637475	non-template	31	Exon_1.f	--	Yes	Yes	--	--	Yes	--	Yes	Yes	Yes	Yes
G4ST01011637145	chr1:11637146-11637184	-	GGGCCTCGAGCAGACTGCTGGGACTGCGGGGCGGCCGGG<br>.....((((...))))	FBXO44	NM_183413	chr1:11637501-11645971	template	-316	upstream	Yes	--	--	--	--	--	--	Yes	Yes	Yes	Yes
G4ST01011637145	chr1:11637146-11637184	-	GGGCCTCGAGCAGACTGCTGGGACTGCGGGGCGGCCGGG<br>.....((((...))))	FBXO44	NM_183412	chr1:11637019-11645971	template	39	Exon_1.f	--	Yes	Yes	--	--	Yes	--	Yes	Yes	Yes	Yes
G4ST01011637145	chr1:11637146-11637184	-	GGGCCTCGAGCAGACTGCTGGGACTGCGGGGCGGCCGGG<br>.....((((...))))	FBXO44	NM_033182	chr1:11637501-11645971	template	-316	upstream	Yes	--	--	--	--	--	--	Yes	Yes	Yes	Yes
G4ST01011637145	chr1:11637146-11637184	-	GGGCCTCGAGCAGACTGCTGGGACTGCGGGGCGGCCGGG<br>.....((((...))))	FBXO44	NM_001014765	chr1:11637501-11645971	template	-316	upstream	Yes	--	--	--	--	--	--	Yes	Yes	Yes	Yes
G4ST01011637145	chr1:11637146-11637184	-	GGGCCTCGAGCAGACTGCTGGGACTGCGGGGCGGCCGGG<br>.....((((...))))	FBXO2	NM_012168	chr1:11631005-11637475	non-template	39	Exon_1.f	--	Yes	Yes	--	--	Yes	--	Yes	Yes	Yes	Yes
G4ST01011637870	chr1:11637871-11637907	+	GGGGCTGGCGGAGCCCAGCCGCGGGGCTGGGGGTGGG<br>((((((...).)))))....	FBXO44	NM_183413	chr1:11637501-11645971	non-template	37	Intron_1.f	--	Yes	--	Yes	--	--	--	--	Yes	--	Yes
G4ST01011637870	chr1:11637871-11637907	+	GGGGCTGGCGGAGCCCAGCCGCGGGGCTGGGGGTGGG<br>((((((...).)))))....	FBXO44	NM_183412	chr1:11637019-11645971	non-template	37	Intron_1.f	--	Yes	--	Yes	--	--	--	--	Yes	--	Yes
G4ST01011637870	chr1:11637871-11637907	+	GGGGCTGGCGGAGCCCAGCCGCGGGGCTGGGGGTGGG<br>((((((...).)))))....	FBXO44	NM_033182	chr1:11637501-11645971	non-template	37	Intron_1.f	--	Yes	--	Yes	--	--	--	--	Yes	--	Yes
G4ST01011637870	chr1:11637871-11637907	+	GGGGCTGGCGGAGCCCAGCCGCGGGGCTGGGGGTGGG<br>((((((...).)))))....	FBXO44	NM_001014765	chr1:11637501-11645971	non-template	37	Intron_1.f	--	Yes	--	Yes	--	--	--	--	Yes	--	Yes
G4ST01011637870	chr1:11637871-11637907	+	GGGGCTGGCGGAGCCCAGCCGCGGGGCTGGGGGTGGG<br>((((((...).)))))....	FBXO2	NM_012168	chr1:11631005-11637475	template	-395	upstream	Yes	--	--	--	--	--	--	--	Yes	--	Yes
G4ST01011652961	chr1:11652962-11652996	-	GGGCCTCCATTAGGTGGGCCTGGTGGGTGCAGGGG<br>(((.....))).	FBXO6	NM_018438	chr1:11646737-11656996	template	35	Intron_2	--	Yes	--	Yes	--	--	--	--	--	--	--
G4ST01011652969	chr1:11652970-11653000	-	GGGTGGGCCTCCATTAGGTGGGCCTGGTGGG<br>(((.....))).	FBXO6	NM_018438	chr1:11646737-11656996	template	31	Intron_2	--	Yes	--	Yes	--	--	--	--	--	--	--

G4ST01011655750	chr1:11655751-11655790	+	GGGCCACAGTGGGGCATAGGGAGCGCTGAAGCCAGCTGGG<br>...((((....)))).	FBXO6	NM_018438	chr1:11646737-11656996	non-template	40	Intron_4	--	Yes	--	Yes	--	--	--	--	--	Yes	--
G4ST01011655851	chr1:11655852-11655881	-	GGGCTGGCCCCAGGGGTGGGGGCAGGTGGG<br>(((....)))	FBXO6	NM_018438	chr1:11646737-11656996	template	30	Intron_4	--	Yes	--	Yes	--	--	--	--	--	Yes	--
G4ST01011658432	chr1:11658433-11658475	-	GGGGGTGCTGCTGAGCCAGCAAGGGAAGTGGGAAGAGGCTGGG<br>..(((((......))))). .((...))	MAD2L2	NM_006341	chr1:11657124-11663858	non-template	43	Intron_6	--	Yes	--	Yes	--	--	--	--	--	--	--
G4ST01011658432	chr1:11658433-11658475	-	GGGGGTGCTGCTGAGCCAGCAAGGGAAGTGGGAAGAGGCTGGG<br>..(((((......))))). .((...))	MAD2L2	NM_001127325	chr1:11657124-11674265	non-template	43	Intron_6	--	Yes	--	Yes	--	--	--	--	--	--	--
G4ST01011674232	chr1:11674233-11674274	-	GGGCTCGACTGCCCCCAGCCGAGGGGCAGCCCCGGGGCCGGG<br>((((.(((....))).))))	C1orf187	NM_198545	chr1:11674368-11702923	template	-93	upstream	Yes	--	--	--	--	--	--	Yes	Yes	--	--
G4ST01011674232	chr1:11674233-11674274	-	GGGCTCGACTGCCCCCAGCCGAGGGGCAGCCCCGGGGCCGGG<br>((((.(((....))).))))	MAD2L2	NM_001127325	chr1:11657124-11674265	non-template	33	upstream-Exon_1.f	Yes	Yes	Yes	--	--	Yes	--	Yes	Yes	--	--
G4ST01011674237	chr1:11674238-11674281	-	GGGTGGCGGGCTCGACTGCCCCCAGCCGAGGGGCAGCCCCGGGG<br>((((.(((....))).))))	C1orf187	NM_198545	chr1:11674368-11702923	template	-86	upstream	Yes	--	--	--	--	--	--	Yes	Yes	--	--
G4ST01011674237	chr1:11674238-11674281	-	GGGTGGCGGGCTCGACTGCCCCCAGCCGAGGGGCAGCCCCGGGG<br>((((.(((....))).))))	MAD2L2	NM_001127325	chr1:11657124-11674265	non-template	28	upstream-Exon_1.f	Yes	Yes	Yes	--	--	Yes	--	Yes	Yes	--	--
G4ST01011674248	chr1:11674249-11674289	-	GGGGCGGCGGGTGGCGGGCTCGACTGCCCCCAGCCGAGGGG<br>((((.(((....))).))))	C1orf187	NM_198545	chr1:11674368-11702923	template	-78	upstream	Yes	--	--	--	--	--	--	Yes	Yes	--	--
G4ST01011674248	chr1:11674249-11674289	-	GGGGCGGCGGGTGGCGGGCTCGACTGCCCCCAGCCGAGGGG<br>((((.(((....))).))))	MAD2L2	NM_001127325	chr1:11657124-11674265	non-template	17	upstream-Exon_1.f	Yes	Yes	Yes	--	--	Yes	--	Yes	Yes	--	--
G4ST01011675052	chr1:11675053-11675099	-	GGGCACAGATGGGACGGCGCGGGGCCACCGGCGGTGCTGGTTCCGGG<br>...((((((...))))))...	C1orf187	NM_198545	chr1:11674368-11702923	template	47	Intron_1.f	--	Yes	--	Yes	--	--	--	--	Yes	--	--
G4ST01011675052	chr1:11675053-11675099	-	GGGCACAGATGGGACGGCGCGGGGCCACCGGCGGTGCTGGTTCCGGG<br>...((((((...))))))...	MAD2L2	NM_001127325	chr1:11657124-11674265	non-template	-787	upstream	Yes	--	--	--	--	--	--	--	Yes	--	--
G4ST01011723845	chr1:11723846-11723884	-	GGGGCTTCTCTGAGGAAGGGATGGGGGAGGCTGAGGGGG<br>..((((....))))	AGTRAP	NM_020350	chr1:11718729-11733415	template	39	Intron_1.f	--	Yes	--	Yes	--	--	--	--	Yes	--	Yes
G4ST01011723845	chr1:11723846-11723884	-	GGGGCTTCTCTGAGGAAGGGATGGGGGAGGCTGAGGGGG<br>..((((....))))	AGTRAP	NM_001040196	chr1:11718729-11733415	template	39	Intron_1.f	--	Yes	--	Yes	--	--	--	--	Yes	--	Yes
G4ST01011723845	chr1:11723846-11723884	-	GGGGCTTCTCTGAGGAAGGGATGGGGGAGGCTGAGGGGG<br>..((((....))))	AGTRAP	NM_001040197	chr1:11718729-11733415	template	39	Intron_1.f	--	Yes	--	Yes	--	--	--	--	Yes	--	Yes
G4ST01011723845	chr1:11723846-11723884	-	GGGGCTTCTCTGAGGAAGGGATGGGGGAGGCTGAGGGGG<br>..((((....))))	AGTRAP	NM_001040195	chr1:11718729-11733415	template	39	Intron_1.f	--	Yes	--	Yes	--	--	--	--	Yes	--	Yes
G4ST01011723845	chr1:11723846-11723884	-	GGGGCTTCTCTGAGGAAGGGATGGGGGAGGCTGAGGGGG<br>..((((....))))	AGTRAP	NM_001040194	chr1:11718729-11733415	template	39	Intron_1.f	--	Yes	--	Yes	--	--	--	--	Yes	--	Yes
G4ST01011723857	chr1:11723858-11723894	-	GGGGTGGTCAGGGGCTTCTCTGAGGAAGGGATGGGGG<br>..((((....))))	AGTRAP	NM_020350	chr1:11718729-11733415	template	37	Intron_1.f	--	Yes	--	Yes	--	--	--	--	Yes	--	Yes
G4ST01011723857	chr1:11723858-11723894	-	GGGGTGGTCAGGGGCTTCTCTGAGGAAGGGATGGGGG<br>..((((....))))	AGTRAP	NM_001040196	chr1:11718729-11733415	template	37	Intron_1.f	--	Yes	--	Yes	--	--	--	--	Yes	--	Yes
G4ST01011723857	chr1:11723858-11723894	-	GGGGTGGTCAGGGGCTTCTCTGAGGAAGGGATGGGGG<br>..((((....))))	AGTRAP	NM_001040197	chr1:11718729-11733415	template	37	Intron_1.f	--	Yes	--	Yes	--	--	--	--	Yes	--	Yes
G4ST01011723857	chr1:11723858-11723894	-	GGGGTGGTCAGGGGCTTCTCTGAGGAAGGGATGGGGG<br>..((((....))))	AGTRAP	NM_001040195	chr1:11718729-11733415	template	37	Intron_1.f	--	Yes	--	Yes	--	--	--	--	Yes	--	Yes
G4ST01011723857	chr1:11723858-11723894	-	GGGGTGGTCAGGGGCTTCTCTGAGGAAGGGATGGGGG<br>..((((....))))	AGTRAP	NM_001040194	chr1:11718729-11733415	template	37	Intron_1.f	--	Yes	--	Yes	--	--	--	--	Yes	--	Yes
G4ST01011723864	chr1:11723865-11723899	-	GGGTTGGGGTGGTCAGGGGCTTCTCTGAGGAAGGG<br>..((((....))))	AGTRAP	NM_020350	chr1:11718729-11733415	template	35	Intron_1.f	--	Yes	--	Yes	--	--	--	--	Yes	--	Yes
G4ST01011723864	chr1:11723865-11723899	-	GGGTTGGGGTGGTCAGGGGCTTCTCTGAGGAAGGG<br>..((((....))))	AGTRAP	NM_001040196	chr1:11718729-11733415	template	35	Intron_1.f	--	Yes	--	Yes	--	--	--	--	Yes	--	Yes
G4ST01011723864	chr1:11723865-11723899	-	GGGTTGGGGTGGTCAGGGGCTTCTCTGAGGAAGGG<br>..((((....))))	AGTRAP	NM_001040197	chr1:11718729-11733415	template	35	Intron_1.f	--	Yes	--	Yes	--	--	--	--	Yes	--	Yes
G4ST01011723864	chr1:11723865-11723899	-	GGGTTGGGGTGGTCAGGGGCTTCTCTGAGGAAGGG<br>..((((....))))	AGTRAP	NM_001040195	chr1:11718729-11733415	template	35	Intron_1.f	--	Yes	--	Yes	--	--	--	--	Yes	--	Yes
G4ST01011723864	chr1:11723865-11723899	-	GGGTTGGGGTGGTCAGGGGCTTCTCTGAGGAAGGG<br>..((((....))))	AGTRAP	NM_001040194	chr1:11718729-11733415	template	35	Intron_1.f	--	Yes	--	Yes	--	--	--	--	Yes	--	Yes
G4ST01011770342	chr1:11770343-11770388	+	GGGCGGCAGGCAGCCGGGAGAGGCTGGAGGCCTGGGGAGATGGGGG<br>.(((.....))) ..((((.....)))).	MTHFR	NM_005957	chr1:11768374-11788747	template	46	Exon_12.l	--	Yes	Yes	--	--	--	Yes	Yes	Yes	Yes	--
G4ST01011770357	chr1:11770358-11770393	+	GGGAGAGGCTGGAGGCCTGGGGAGATGGGGGCAGGG<br>..((((.....)))).	MTHFR	NM_005957	chr1:11768374-11788747	template	36	Exon_12.l	--	Yes	Yes	--	--	--	Yes	Yes	Yes	Yes	--
G4ST01011773974	chr1:11773975-11774003	+	GGGGATGTGGGGTGGGGGTAAGACCTGGG<br>(((...))).	MTHFR	NM_005957	chr1:11768374-11788747	template	29	Intron_10	--	Yes	--	Yes	--	--	--	--	Yes	Yes	--
G4ST01011817420	chr1:11817421-11817451	+	GGGGACACGGGCACTGTGGGACTGCAGTGGG<br>((....))	CLCN6	NM_001286	chr1:11788794-11825788	non-template	31	Intron_17	--	Yes	--	Yes	--	--	--	--	--	--	Yes
G4ST01011817428	chr1:11817429-11817456	+	GGGCACTGTGGGACTGCAGTGGGCAGGG<br>((....))	CLCN6	NM_001286	chr1:11788794-11825788	non-template	28	Intron_17	--	Yes	--	Yes	--	--	--	--	--	--	Yes
G4ST01011823098	chr1:11823099-11823134	-	GGGCCTGTGCCAACACTGGAGGGTGGGAACACAGGG<br>((.(((....))).)).	NPPA-AS1	NR_037806	chr1:11822963-11830260	template	36	Exon_1.f	--	Yes	Yes	--	--	--	--	--	--	--	--
G4ST01011823098	chr1:11823099-11823134	-	GGGCCTGTGCCAACACTGGAGGGTGGGAACACAGGG<br>((.(((....))).)).	CLCN6	NM_001286	chr1:11788794-11825788	template	36	Exon_23.l	--	Yes	Yes	--	--	--	Yes	--	--	--	--
G4ST01011823107	chr1:11823108-11823139	-	GGGGTGGGCCTGTGCCAACACTGGAGGGTGGG<br>((.(((....))).)).	NPPA-AS1	NR_037806	chr1:11822963-11830260	template	32	Exon_1.f	--	Yes	Yes	--	--	--	--	--	--	--	--
G4ST01011823107	chr1:11823108-11823139	-	GGGGTGGGCCTGTGCCAACACTGGAGGGTGGG<br>((.(((....))).)).	CLCN6	NM_001286	chr1:11788794-11825788	template	32	Exon_23.l	--	Yes	Yes	--	--	--	Yes	--	--	--	--
G4ST01011830589	chr1:11830590-11830630	-	GGGTCTGGGGGGCTGTTGGGGCTGCCCAAGGCAGAGAGGGG<br>.(((((...)))))....	NPPA	NM_006172	chr1:11828354-11830427	non-template	-162	upstream	Yes	--	--	--	--	--	--	--	--	--	--
G4ST01011832356	chr1:11832357-11832395	-	GGGGCATCTGAAAGATGGGGAATGGGGGTTTAATTGGGG<br>.(((((...)))))	NPPA	NM_006172	chr1:11828354-11830427	non-template	-1929	upstream	Yes	--	--	--	--	--	--	--	Yes	--	--
G4ST01011908882	chr1:11908883-11908913	+	GGGCCGCCAGGCGCGGGCAAGGGTGCGAGGG<br>.(((...))).	KIAA2013	NM_138346	chr1:11902232-11909072	template	31	Exon_1.f	--	Yes	Yes	--	--	Yes	--	--	Yes	Yes	--
G4ST01011908896	chr1:11908897-11908924	+	GGGCAAGGGTGCGAGGGCGGCCGCCGGG<br>.((...))	KIAA2013	NM_138346	chr1:11902232-11909072	template	28	Exon_1.f	--	Yes	Yes	--	--	Yes	--	--	Yes	Yes	--
G4ST01011921231	chr1:11921232-11921270	-	GGGAAAGGGAAGAGCTGGCCTCTTTGGGGGCTGCTGGGG<br>(((((.....)))))...	PLOD1	NM_000302	chr1:11917311-11958186	template	39	Intron_1.f	--	Yes	--	Yes	--	--	--	--	--	--	--
G4ST01011947593	chr1:11947594-11947628	+	GGGGGCCGTCGGGCACGAGGGCTGGTCCTCAGGGG<br>(((.....)))	PLOD1	NM_000302	chr1:11917311-11958186	non-template	35	Intron_13	--	Yes	--	Yes	--	--	--	--	--	--	--
G4ST01011947603	chr1:11947604-11947637	+	GGGCACGAGGGCTGGTCCTCAGGGGTCTGCAGGG<br>(((.....)))	PLOD1	NM_000302	chr1:11917311-11958186	non-template	34	Intron_13	--	Yes	--	Yes	--	--	--	--	--	--	--
G4ST01011953458	chr1:11953459-11953494	+	GGGTGGGCAAGCCTGGGGCATAGCCAGGATGCGGGG<br>((((.......)))).	PLOD1	NM_000302	chr1:11917311-11958186	non-template	36	Exon_17-Intron_17	--	Yes	Yes	Yes	Yes	--	--	--	--	--	--
G4ST01011953462	chr1:11953463-11953503	+	GGGCAAGCCTGGGGCATAGCCAGGATGCGGGGACAGTTGGG<br>((((.......)))).	PLOD1	NM_000302	chr1:11917311-11958186	non-template	41	Intron_17	--	Yes	--	Yes	--	--	--	--	--	--	--
G4ST01011953472	chr1:11953473-11953508	+	GGGGCATAGCCAGGATGCGGGGACAGTTGGGTGGGG<br>((((.......)))).	PLOD1	NM_000302	chr1:11917311-11958186	non-template	36	Intron_17	--	Yes	--	Yes	--	--	--	--	--	--	--
G4ST01011953891	chr1:11953892-11953925	+	GGGGTGGGTGGGGGCTGGAGAGTCTGGCAAGGGG<br>.((((((...))))))...	PLOD1	NM_000302	chr1:11917311-11958186	non-template	34	Intron_17	--	Yes	--	Yes	--	--	--	--	--	--	Yes
G4ST01011953896	chr1:11953897-11953930	+	GGGTGGGGGCTGGAGAGTCTGGCAAGGGGAGGGG<br>.((((((...))))))...	PLOD1	NM_000302	chr1:11917311-11958186	non-template	34	Intron_17	--	Yes	--	Yes	--	--	--	--	--	--	Yes
G4ST01011953900	chr1:11953901-11953936	+	GGGGGCTGGAGAGTCTGGCAAGGGGAGGGGCTGGGG<br>.((((((...))))))...	PLOD1	NM_000302	chr1:11917311-11958186	non-template	36	Intron_17	--	Yes	--	Yes	--	--	--	--	--	--	Yes
G4ST01011955774	chr1:11955775-11955802	+	GGGTCTGTGGGAGGGCTTCCTGGAAGGG<br>.(((...)))	PLOD1	NM_000302	chr1:11917311-11958186	non-template	28	Intron_18.l	--	Yes	--	Yes	--	--	--	Yes	--	--	--

G4ST01011963306	chr1:11963307-11963337	+	GGGCCACGGGACCTGGGACTGGTCGGTGGGG<br>(((....))).	MFN2	NM_001127660	chr1:11962825-11996159	non-template	31	Intron_1.f	--	Yes	--	Yes	--	--	--	Yes	Yes	Yes	Yes
G4ST01011963306	chr1:11963307-11963337	+	GGGCCACGGGACCTGGGACTGGTCGGTGGGG<br>(((....))).	MFN2	NM_014874	chr1:11962825-11996159	non-template	31	Intron_1.f	--	Yes	--	Yes	--	--	--	Yes	Yes	Yes	Yes
G4ST01011974230	chr1:11974231-11974263	+	GGGTGGCAAGAGGCCAACAGGGCCAGGGCAGGG<br>((((.....))))...	MFN2	NM_001127660	chr1:11962825-11996159	non-template	33	Intron_2	--	Yes	--	Yes	--	--	--	--	--	--	Yes
G4ST01011974230	chr1:11974231-11974263	+	GGGTGGCAAGAGGCCAACAGGGCCAGGGCAGGG<br>((((.....))))...	MFN2	NM_014874	chr1:11962825-11996159	non-template	33	Intron_3	--	Yes	--	Yes	--	--	--	--	--	--	Yes
G4ST01012005093	chr1:12005094-12005134	+	GGGAGAGCGGGACATCTGCTGATGGGAGGAGCTTCCTTGGG<br>..(((....))) ((((...)))).	MIIP	NM_021933	chr1:12002099-12014693	non-template	41	Intron_3	--	Yes	--	Yes	--	--	--	--	--	--	--
G4ST01012070139	chr1:12070140-12070169	-	GGGACGGAGGGGAGGGCATCCCACATGGGG<br>(((.....)))	TNFRSF8	NM_001243	chr1:12046021-12126851	template	30	Intron_2	--	Yes	--	Yes	--	--	--	--	Yes	--	--
G4ST01012087709	chr1:12087710-12087747	+	GGGGTATGGGCCACAGAACCTGTCTGGGTGGAGTTGGG<br>..((((...))))..	TNFRSF8	NM_001243	chr1:12046021-12126851	non-template	38	Intron_4	--	Yes	--	Yes	--	--	--	--	--	Yes	--
G4ST01012106085	chr1:12106086-12106116	+	GGGGATGCCTGGGAGGCAGCTGGGCTGGGGG<br>((....))	TNFRSF8	NM_001243	chr1:12046021-12126851	non-template	31	Intron_9	--	Yes	--	Yes	--	--	--	--	--	Yes	Yes
G4ST01012106095	chr1:12106096-12106127	+	GGGAGGCAGCTGGGCTGGGGGTGGAGGTGGGG<br>((....))	TNFRSF8	NM_001243	chr1:12046021-12126851	non-template	32	Intron_9	--	Yes	--	Yes	--	--	--	--	--	Yes	Yes
G4ST01012148976	chr1:12148977-12149012	+	GGGAGGCCAGAGGCGGGAAGATCACTTGAGGGTGGG<br>..(((...))) (((....)))..	TNFRSF1B	NM_001066	chr1:12149647-12191864	non-template	-634	upstream	Yes	--	--	--	--	--	--	--	--	--	--
G4ST01012149361	chr1:12149362-12149390	+	GGGAGCTGGGAGGGCAGGTGGAGGCCGGG<br>..(((....)))	TNFRSF1B	NM_001066	chr1:12149647-12191864	non-template	-256	upstream	Yes	--	--	--	--	--	--	--	Yes	Yes	--
G4ST01012149368	chr1:12149369-12149405	+	GGGAGGGCAGGTGGAGGCCGGGCAGGTGGAGGCCGGG<br>..(((....))) ..(((....)))	TNFRSF1B	NM_001066	chr1:12149647-12191864	non-template	-241	upstream	Yes	--	--	--	--	--	--	--	Yes	Yes	--
G4ST01012149515	chr1:12149516-12149555	+	GGGTCGGAGGCCCAGCAGCGTCTGGGGAGGGGCGTGGGGG<br>.((((.((......)).))))	TNFRSF1B	NM_001066	chr1:12149647-12191864	non-template	-91	upstream	Yes	--	--	--	--	--	--	Yes	Yes	Yes	--
G4ST01012150100	chr1:12150101-12150132	+	GGGGCATTTGGGGCTGAGCAGCGGGTCCTGGG<br>(((....)))	TNFRSF1B	NM_001066	chr1:12149647-12191864	non-template	32	Intron_1.f	--	Yes	--	Yes	--	--	--	Yes	Yes	Yes	--
G4ST01012174018	chr1:12174019-12174058	+	GGGGAGGAGCGAAACTGCTGGGAAGAGCGGGACTGCGGGG<br>.((.((.....)).))	TNFRSF1B	NM_001066	chr1:12149647-12191864	non-template	40	Intron_3	--	Yes	--	Yes	--	--	--	--	--	--	Yes
G4ST01012186731	chr1:12186732-12186761	-	GGGGAGAGGGGAGGGCAGACATGGTCAGGG<br>..(((...))).	TNFRSF1B	NM_001066	chr1:12149647-12191864	template	30	Intron_9.l	--	Yes	--	Yes	--	--	--	--	Yes	--	Yes
G4ST01012213501	chr1:12213502-12213539	+	GGGTGGTGGGGTCTCTGTCCCCTGGAGGGGGCTGAGGG<br>..(((((.....))))).	VPS13D	NM_015378	chr1:12212700-12494685	non-template	38	Intron_1.f	--	Yes	--	Yes	--	--	--	Yes	Yes	--	--
G4ST01012213501	chr1:12213502-12213539	+	GGGTGGTGGGGTCTCTGTCCCCTGGAGGGGGCTGAGGG<br>..(((((.....))))).	VPS13D	NM_018156	chr1:12212700-12494685	non-template	38	Intron_1.f	--	Yes	--	Yes	--	--	--	Yes	Yes	--	--
G4ST01012460726	chr1:12460727-12460760	+	GGGGCCTATATATAGGGGCCCAGGGCCTGCTGGG<br>..(((....)))	VPS13D	NM_015378	chr1:12212700-12494685	non-template	34	Intron_67	--	Yes	--	Yes	--	--	--	--	Yes	Yes	Yes
G4ST01012460726	chr1:12460727-12460760	+	GGGGCCTATATATAGGGGCCCAGGGCCTGCTGGG<br>..(((....)))	VPS13D	NM_018156	chr1:12212700-12494685	non-template	34	Intron_66	--	Yes	--	Yes	--	--	--	--	Yes	Yes	Yes
G4ST01012555307	chr1:12555308-12555338	-	GGGGCTGGGAAAGAACACTCTGGGTCAGGGG<br>..(((....)))	DHRS3	NM_004753	chr1:12550526-12600407	non-template	31	Intron_5.l	--	Yes	--	Yes	--	--	--	Yes	Yes	--	--
G4ST01012560589	chr1:12560590-12560618	-	GGGGGTGTAACCTGGGAGGGAGGCGAGGG<br>(((...))).	DHRS3	NM_004753	chr1:12550526-12600407	non-template	29	Intron_4	--	Yes	--	Yes	--	--	--	--	--	--	--
G4ST01012560598	chr1:12560599-12560623	-	GGGCTGGGGGTGTAACCTGGGAGGG<br>(((...))).	DHRS3	NM_004753	chr1:12550526-12600407	non-template	25	Intron_4	--	Yes	--	Yes	--	--	--	--	--	--	--
G4ST01012570433	chr1:12570434-12570467	-	GGGGGCTGGGGGACAAGGGGTTGGACCCAAAGGG<br>.(((....))).	DHRS3	NM_004753	chr1:12550526-12600407	non-template	34	Intron_1.f	--	Yes	--	Yes	--	--	--	--	Yes	--	--
G4ST01012578055	chr1:12578056-12578089	+	GGGGATCGGGCTGGGATCCCAAGCGGCGGATGGG<br>((((........))))	DHRS3	NM_004753	chr1:12550526-12600407	template	34	Intron_1.f	--	Yes	--	Yes	--	--	--	Yes	--	--	Yes
G4ST01012578062	chr1:12578063-12578096	+	GGGCTGGGATCCCAAGCGGCGGATGGGAAAGGGG<br>((((........))))	DHRS3	NM_004753	chr1:12550526-12600407	template	34	Intron_1.f	--	Yes	--	Yes	--	--	--	Yes	--	--	Yes
G4ST01012578067	chr1:12578068-12578106	+	GGGATCCCAAGCGGCGGATGGGAAAGGGGTGGAAAGGGG<br>((((........))))	DHRS3	NM_004753	chr1:12550526-12600407	template	39	Intron_1.f	--	Yes	--	Yes	--	--	--	Yes	--	--	Yes
G4ST01012578803	chr1:12578804-12578843	-	GGGGTGCTCGAGGGGAGCGAGGCCGCTCCAGGGAGGTGGG<br>(((((....)))))..	DHRS3	NM_004753	chr1:12550526-12600407	non-template	40	Intron_1.f	--	Yes	--	Yes	--	--	--	Yes	Yes	--	--
G4ST01012641336	chr1:12641337-12641374	-	GGGCAGCATCAGCGCTGGGGGTGGAAACGGGTGCAGGG<br>((((......)))).	AADACL4	NM_001013630	chr1:12627153-12649684	template	38	Intron_2	--	Yes	--	Yes	--	--	--	Yes	--	--	Yes
G4ST01012641343	chr1:12641344-12641379	-	GGGCAGGGCAGCATCAGCGCTGGGGGTGGAAACGGG<br>((((......)))).	AADACL4	NM_001013630	chr1:12627153-12649684	template	36	Intron_2	--	Yes	--	Yes	--	--	--	Yes	--	--	Yes
G4ST01012641353	chr1:12641354-12641386	-	GGGTGCAGGGCAGGGCAGCATCAGCGCTGGGGG<br>((((......)))).	AADACL4	NM_001013630	chr1:12627153-12649684	template	33	Intron_2	--	Yes	--	Yes	--	--	--	Yes	--	--	Yes
G4ST01012641371	chr1:12641372-12641400	-	GGGGGTGGAGACCAGGGTGCAGGGCAGGG<br>(((....))).	AADACL4	NM_001013630	chr1:12627153-12649684	template	29	Intron_2	--	Yes	--	Yes	--	--	--	Yes	--	--	--
G4ST01012898366	chr1:12898367-12898404	-	GGGCAGGATGGGACCGGGTGTTCTAGTAGAACCCTGGG<br>.(((((...)))))...	PRAMEF8	NM_001012276	chr1:12899037-12903155	template	-632	upstream	Yes	--	--	--	--	--	--	--	--	--	--
G4ST01012898366	chr1:12898367-12898404	-	GGGCAGGATGGGACCGGGTGTTCTAGTAGAACCCTGGG<br>.(((((...)))))...	PRAMEF7	NM_001012277	chr1:12900100-12902820	template	-1695	upstream	Yes	--	--	--	--	--	--	--	--	--	--
G4ST01012959570	chr1:12959571-12959609	+	GGGCAGAGGGATGACCTGGGGTAGAAGCTACAGAGAGGG<br>((((...))))....	PRAMEF22	NM_001100631	chr1:12958130-12960968	non-template	39	Intron_2.l	--	Yes	--	Yes	--	--	--	--	--	--	--
G4ST01012959577	chr1:12959578-12959622	+	GGGATGACCTGGGGTAGAAGCTACAGAGAGGGACATCGTGTAGGG<br>((((...)))).... (((...))).	PRAMEF22	NM_001100631	chr1:12958130-12960968	non-template	45	Intron_2.l	--	Yes	--	Yes	--	--	--	--	--	--	--
G4ST01013202765	chr1:13202766-13202810	-	GGGATGACCTGGGGTAGAAGCTACAGAGAGGGACATCGTGTAGGG<br>((((...)))).... (((...))).	PRAMEF3	NM_001013692	chr1:13200783-13204279	non-template	45	Intron_2.l	--	Yes	--	Yes	--	--	--	--	--	--	--
G4ST01013202765	chr1:13202766-13202810	-	GGGATGACCTGGGGTAGAAGCTACAGAGAGGGACATCGTGTAGGG<br>((((...)))).... (((...))).	PRAMEF22	NM_001100631	chr1:13201420-13204258	non-template	45	Intron_2.l	--	Yes	--	Yes	--	--	--	--	--	--	--
G4ST01013202778	chr1:13202779-13202817	-	GGGCAGAGGGATGACCTGGGGTAGAAGCTACAGAGAGGG<br>((((...))))....	PRAMEF3	NM_001013692	chr1:13200783-13204279	non-template	39	Intron_2.l	--	Yes	--	Yes	--	--	--	--	--	--	--
G4ST01013202778	chr1:13202779-13202817	-	GGGCAGAGGGATGACCTGGGGTAGAAGCTACAGAGAGGG<br>((((...))))....	PRAMEF22	NM_001100631	chr1:13201420-13204258	non-template	39	Intron_2.l	--	Yes	--	Yes	--	--	--	--	--	--	--
G4ST01013263984	chr1:13263985-13264022	+	GGGCAGGATGGGACCGGGTGTTCTAGTAGAACCCTGGG<br>.(((((...)))))...	PRAMEF8	NM_001012276	chr1:13259234-13263352	template	-632	upstream	Yes	--	--	--	--	--	--	--	--	--	--
G4ST01013484769	chr1:13484770-13484807	+	GGGCAGGATGGGACCGGGTGTTCTAGTAGAACCCTGGG<br>.(((((...)))))...	PRAMEF7	NM_001012277	chr1:13480354-13483074	template	-1695	upstream	Yes	--	--	--	--	--	--	--	--	--	--
G4ST01013484769	chr1:13484770-13484807	+	GGGCAGGATGGGACCGGGTGTTCTAGTAGAACCCTGGG<br>.(((((...)))))...	PRAMEF8	NM_001012276	chr1:13480019-13484137	template	-632	upstream	Yes	--	--	--	--	--	--	--	--	--	--
G4ST01013694522	chr1:13694523-13694556	+	GGGGGAGGGAGGGAAGAGGAGTGACTGCTCTGGG<br>.((((.((...)).))))	LRRC38	NM_001010847	chr1:13674032-13712829	template	34	Intron_1.u	--	Yes	--	Yes	--	--	--	--	--	--	Yes
G4ST01013708999	chr1:13709000-13709030	-	GGGCCTGGGGGTAGGGGGCTGAGGCCTGGGG<br>(((....)))..	LRRC38	NM_001010847	chr1:13674032-13712829	non-template	31	Intron_1.u	--	Yes	--	Yes	--	--	--	--	--	--	--
G4ST01013949022	chr1:13949023-13949062	+	GGGCCGCGTCGGTCGCGGAGTCGGGGTGTGGGAGTGGGGG<br>(((((.....)))))....	PRDM2	NM_001135610	chr1:13899322-14024161	non-template	40	Intron_6	--	Yes	--	Yes	--	--	--	Yes	Yes	Yes	--
G4ST01013949022	chr1:13949023-13949062	+	GGGCCGCGTCGGTCGCGGAGTCGGGGTGTGGGAGTGGGGG<br>(((((.....)))))....	PRDM2	NM_012231	chr1:13903937-14024161	non-template	40	Intron_6	--	Yes	--	Yes	--	--	--	Yes	Yes	Yes	--
G4ST01013949022	chr1:13949023-13949062	+	GGGCCGCGTCGGTCGCGGAGTCGGGGTGTGGGAGTGGGGG<br>(((((.....)))))....	PRDM2	NM_015866	chr1:13903937-13987161	non-template	40	Intron_6	--	Yes	--	Yes	--	--	--	Yes	Yes	Yes	--
G4ST01013949022	chr1:13949023-13949062	+	GGGCCGCGTCGGTCGCGGAGTCGGGGTGTGGGAGTGGGGG<br>(((((.....)))))....	PRDM2	NM_001007257	chr1:13948463-13987161	non-template	40	Intron_1.f	--	Yes	--	Yes	--	--	--	Yes	Yes	Yes	--
G4ST01014004029	chr1:14004030-14004058	+	GGGGACTGTGTCAGGGAGTGGGGCATGGG<br>(((...))).	PRDM2	NM_001135610	chr1:13899322-14024161	non-template	29	Intron_6	--	Yes	--	Yes	--	--	--	--	--	--	--

G4ST01014004029	chr1:14004030-14004058	+	GGGGACTGTGTCAGGGAGTGGGGCATGGG<br>(((...))).	PRDM2	NM_012231	chr1:13903937-14024161	non-template	29	Intron_8	--	Yes	--	Yes	--	--	--	--	--	--	--
G4ST01014005016	chr1:14005017-14005043	-	GGGTGGAGGGAAAGGAGTTTGGGAGGG<br>(((....)))	PRDM2	NM_001135610	chr1:13899322-14024161	template	27	Intron_6	--	Yes	--	Yes	--	--	--	--	--	--	--
G4ST01014005016	chr1:14005017-14005043	-	GGGTGGAGGGAAAGGAGTTTGGGAGGG<br>(((....)))	PRDM2	NM_012231	chr1:13903937-14024161	template	27	Intron_8	--	Yes	--	Yes	--	--	--	--	--	--	--
G4ST01014009183	chr1:14009184-14009221	+	GGGGGTTAGGGCTTCAGCATATGAATGGGGTGCAGGGG<br>.((((.....))))..	PRDM2	NM_001135610	chr1:13899322-14024161	non-template	38	Intron_6	--	Yes	--	Yes	--	--	--	Yes	--	--	--
G4ST01014009183	chr1:14009184-14009221	+	GGGGGTTAGGGCTTCAGCATATGAATGGGGTGCAGGGG<br>.((((.....))))..	PRDM2	NM_012231	chr1:13903937-14024161	non-template	38	Intron_8	--	Yes	--	Yes	--	--	--	Yes	--	--	--
G4ST01014015077	chr1:14015078-14015111	+	GGGTGTTGGGGCCTGGCAGGAGGGGAGAGCAGGG<br>.(((...)))..	PRDM2	NM_001135610	chr1:13899322-14024161	non-template	34	Intron_6	--	Yes	--	Yes	--	--	--	--	--	--	--
G4ST01014015077	chr1:14015078-14015111	+	GGGTGTTGGGGCCTGGCAGGAGGGGAGAGCAGGG<br>.(((...)))..	PRDM2	NM_012231	chr1:13903937-14024161	non-template	34	Intron_8	--	Yes	--	Yes	--	--	--	--	--	--	--
G4ST01014020090	chr1:14020091-14020123	-	GGGGCTGGAAAGGGCTGTTTGCGGGGGAGTGGG<br>(((....)))	PRDM2	NM_001135610	chr1:13899322-14024161	template	33	Intron_7.l	--	Yes	--	Yes	--	--	--	--	--	--	--
G4ST01014020090	chr1:14020091-14020123	-	GGGGCTGGAAAGGGCTGTTTGCGGGGGAGTGGG<br>(((....)))	PRDM2	NM_012231	chr1:13903937-14024161	template	33	Intron_9.l	--	Yes	--	Yes	--	--	--	--	--	--	--
G4ST01014797243	chr1:14797244-14797280	+	GGGGTGGGCAGCGCAGGTCTGGCCTGCGGGTGGGGGG<br>....(((((.....)))))	KAZN	NM_015209	chr1:14797800-15267238	non-template	-519	upstream	Yes	--	--	--	--	--	--	Yes	--	Yes	--
G4ST01014797243	chr1:14797244-14797280	+	GGGGTGGGCAGCGCAGGTCTGGCCTGCGGGTGGGGGG<br>....(((((.....)))))	KAZN	NM_201628	chr1:14797800-15317131	non-template	-519	upstream	Yes	--	--	--	--	--	--	Yes	--	Yes	--
G4ST01014797394	chr1:14797395-14797436	-	GGGGGCCTCAGAGGGGAGCCGGGGTGGATCTCCATGGAAGGG<br>((((....))))....	KAZN	NM_015209	chr1:14797800-15267238	template	-363	upstream	Yes	--	--	--	--	--	--	--	--	Yes	--
G4ST01014797394	chr1:14797395-14797436	-	GGGGGCCTCAGAGGGGAGCCGGGGTGGATCTCCATGGAAGGG<br>((((....))))....	KAZN	NM_201628	chr1:14797800-15317131	template	-363	upstream	Yes	--	--	--	--	--	--	--	--	Yes	--
G4ST01014797412	chr1:14797413-14797449	-	GGGTGCTTGGAAAGGGGGCCTCAGAGGGGAGCCGGGG<br>..(((...))).	KAZN	NM_015209	chr1:14797800-15267238	template	-350	upstream	Yes	--	--	--	--	--	--	--	--	Yes	--
G4ST01014797412	chr1:14797413-14797449	-	GGGTGCTTGGAAAGGGGGCCTCAGAGGGGAGCCGGGG<br>..(((...))).	KAZN	NM_201628	chr1:14797800-15317131	template	-350	upstream	Yes	--	--	--	--	--	--	--	--	Yes	--
G4ST01014842394	chr1:14842395-14842428	-	GGGGTCTACTTGAAGGTGGAGGGTGGGAGGAGGG<br>..(((((....))))).	KAZN	NM_015209	chr1:14797800-15267238	template	34	Intron_1.f	--	Yes	--	Yes	--	--	--	--	--	--	--
G4ST01014842394	chr1:14842395-14842428	-	GGGGTCTACTTGAAGGTGGAGGGTGGGAGGAGGG<br>..(((((....))))).	KAZN	NM_201628	chr1:14797800-15317131	template	34	Intron_1.f	--	Yes	--	Yes	--	--	--	--	--	--	--
G4ST01014859188	chr1:14859189-14859216	+	GGGCCTGCTTCAGTGGGGCTGGGAGGGG<br>.(((...)))..	KAZN	NM_015209	chr1:14797800-15267238	non-template	28	Intron_1.f	--	Yes	--	Yes	--	--	--	Yes	--	--	Yes
G4ST01014859188	chr1:14859189-14859216	+	GGGCCTGCTTCAGTGGGGCTGGGAGGGG<br>.(((...)))..	KAZN	NM_201628	chr1:14797800-15317131	non-template	28	Intron_1.f	--	Yes	--	Yes	--	--	--	Yes	--	--	Yes
G4ST01014869157	chr1:14869158-14869184	-	GGGAAGGGAAGGGAGAAGACTCTCGGG<br>(((....))).	KAZN	NM_015209	chr1:14797800-15267238	template	27	Intron_1.f	--	Yes	--	Yes	--	--	--	--	--	--	Yes
G4ST01014869157	chr1:14869158-14869184	-	GGGAAGGGAAGGGAGAAGACTCTCGGG<br>(((....))).	KAZN	NM_201628	chr1:14797800-15317131	template	27	Intron_1.f	--	Yes	--	Yes	--	--	--	--	--	--	Yes
G4ST01014916084	chr1:14916085-14916117	-	GGGAGCCTCCCAGCTGGGCCTTGAAGGGCAGGG<br>(((......)))	KAZN	NM_015209	chr1:14797800-15267238	template	33	Intron_1.f	--	Yes	--	Yes	--	--	--	--	--	--	--
G4ST01014916084	chr1:14916085-14916117	-	GGGAGCCTCCCAGCTGGGCCTTGAAGGGCAGGG<br>(((......)))	KAZN	NM_201628	chr1:14797800-15317131	template	33	Intron_1.f	--	Yes	--	Yes	--	--	--	--	--	--	--
G4ST01014916089	chr1:14916090-14916123	-	GGGCAAGGGAGCCTCCCAGCTGGGCCTTGAAGGG<br>(((......)))	KAZN	NM_015209	chr1:14797800-15267238	template	34	Intron_1.f	--	Yes	--	Yes	--	--	--	--	--	--	--
G4ST01014916089	chr1:14916090-14916123	-	GGGCAAGGGAGCCTCCCAGCTGGGCCTTGAAGGG<br>(((......)))	KAZN	NM_201628	chr1:14797800-15317131	template	34	Intron_1.f	--	Yes	--	Yes	--	--	--	--	--	--	--
G4ST01014921838	chr1:14921839-14921872	-	GGGGCTGGGGAAGGGAATCTAGGTGTGGATAGGG<br>.((((......)))).	KAZN	NM_015209	chr1:14797800-15267238	template	34	Intron_1.f	--	Yes	--	Yes	--	--	--	--	--	--	--
G4ST01014921838	chr1:14921839-14921872	-	GGGGCTGGGGAAGGGAATCTAGGTGTGGATAGGG<br>.((((......)))).	KAZN	NM_201628	chr1:14797800-15317131	template	34	Intron_1.f	--	Yes	--	Yes	--	--	--	--	--	--	--
G4ST01014928600	chr1:14928601-14928637	+	GGGAATGCAAGGGAAGAGCAGGGAATTCCAGAATGGG<br>.(((....)))	KAZN	NM_015209	chr1:14797800-15267238	non-template	37	Intron_1.f	--	Yes	--	Yes	--	--	--	--	--	--	--
G4ST01014928600	chr1:14928601-14928637	+	GGGAATGCAAGGGAAGAGCAGGGAATTCCAGAATGGG<br>.(((....)))	KAZN	NM_201628	chr1:14797800-15317131	non-template	37	Intron_1.f	--	Yes	--	Yes	--	--	--	--	--	--	--
G4ST01014953243	chr1:14953244-14953271	+	GGGTGGGTTGGGGTGGACAGCTGCAGGG<br>((((....)))).	KAZN	NM_015209	chr1:14797800-15267238	non-template	28	Intron_1.f	--	Yes	--	Yes	--	--	--	--	--	--	--
G4ST01014953243	chr1:14953244-14953271	+	GGGTGGGTTGGGGTGGACAGCTGCAGGG<br>((((....)))).	KAZN	NM_201628	chr1:14797800-15317131	non-template	28	Intron_1.f	--	Yes	--	Yes	--	--	--	--	--	--	--
G4ST01014966028	chr1:14966029-14966068	+	GGGGAAAATAAGGGCTGGAGGGACCAAATCAATGGTGGGG<br>((((......)))).	KAZN	NM_015209	chr1:14797800-15267238	non-template	40	Intron_1.f	--	Yes	--	Yes	--	--	--	--	--	Yes	--
G4ST01014966028	chr1:14966029-14966068	+	GGGGAAAATAAGGGCTGGAGGGACCAAATCAATGGTGGGG<br>((((......)))).	KAZN	NM_201628	chr1:14797800-15317131	non-template	40	Intron_1.f	--	Yes	--	Yes	--	--	--	--	--	Yes	--
G4ST01014966039	chr1:14966040-14966074	+	GGGCTGGAGGGACCAAATCAATGGTGGGGAGTGGG<br>((((......)))).	KAZN	NM_015209	chr1:14797800-15267238	non-template	35	Intron_1.f	--	Yes	--	Yes	--	--	--	--	--	Yes	--
G4ST01014966039	chr1:14966040-14966074	+	GGGCTGGAGGGACCAAATCAATGGTGGGGAGTGGG<br>((((......)))).	KAZN	NM_201628	chr1:14797800-15317131	non-template	35	Intron_1.f	--	Yes	--	Yes	--	--	--	--	--	Yes	--
G4ST01014999961	chr1:14999962-14999993	+	GGGAGGGGAAGTGGGGAGTGACTGCTCGTGGG<br>((((....))))..	KAZN	NM_015209	chr1:14797800-15267238	non-template	32	Intron_1.f	--	Yes	--	Yes	--	--	--	Yes	Yes	--	Yes
G4ST01014999961	chr1:14999962-14999993	+	GGGAGGGGAAGTGGGGAGTGACTGCTCGTGGG<br>((((....))))..	KAZN	NM_201628	chr1:14797800-15317131	non-template	32	Intron_1.f	--	Yes	--	Yes	--	--	--	Yes	Yes	--	Yes
G4ST01014999965	chr1:14999966-14999999	+	GGGGAAGTGGGGAGTGACTGCTCGTGGGTAGGGG<br>((((....))))..	KAZN	NM_015209	chr1:14797800-15267238	non-template	34	Intron_1.f	--	Yes	--	Yes	--	--	--	Yes	Yes	--	Yes
G4ST01014999965	chr1:14999966-14999999	+	GGGGAAGTGGGGAGTGACTGCTCGTGGGTAGGGG<br>((((....))))..	KAZN	NM_201628	chr1:14797800-15317131	non-template	34	Intron_1.f	--	Yes	--	Yes	--	--	--	Yes	Yes	--	Yes
G4ST01015093533	chr1:15093534-15093573	+	GGGTCCCTGGGAAGACTGAATGTGTTAGGGACGTCAAGGG<br>((.((.....)).)).	KAZN	NM_015209	chr1:14797800-15267238	non-template	40	Intron_1.f	--	Yes	--	Yes	--	--	--	--	--	--	--
G4ST01015093533	chr1:15093534-15093573	+	GGGTCCCTGGGAAGACTGAATGTGTTAGGGACGTCAAGGG<br>((.((.....)).)).	KAZN	NM_201628	chr1:14797800-15317131	non-template	40	Intron_1.f	--	Yes	--	Yes	--	--	--	--	--	--	--
G4ST01015123077	chr1:15123078-15123121	+	GGGGCGACGCGGCGGCGCTCCCCGGGCACTGGGGCCAGTCTGGG<br>(((.((...)).)))....	KAZN	NM_015209	chr1:14797800-15267238	non-template	44	Intron_1.f	--	Yes	--	Yes	--	--	--	Yes	Yes	Yes	--
G4ST01015123077	chr1:15123078-15123121	+	GGGGCGACGCGGCGGCGCTCCCCGGGCACTGGGGCCAGTCTGGG<br>(((.((...)).)))....	KAZN	NM_201628	chr1:14797800-15317131	non-template	44	Intron_1.f	--	Yes	--	Yes	--	--	--	Yes	Yes	Yes	--
G4ST01015123077	chr1:15123078-15123121	+	GGGGCGACGCGGCGGCGCTCCCCGGGCACTGGGGCCAGTCTGGG<br>(((.((...)).)))....	KAZN	NM_001018000	chr1:15123212-15267238	non-template	-90	upstream	Yes	--	--	--	--	--	--	Yes	Yes	Yes	--
G4ST01015123100	chr1:15123101-15123134	+	GGGCACTGGGGCCAGTCTGGGCGCCGCGGCGGGG<br>(((....)))	KAZN	NM_015209	chr1:14797800-15267238	non-template	34	Intron_1.f	--	Yes	--	Yes	--	--	--	Yes	Yes	Yes	Yes
G4ST01015123100	chr1:15123101-15123134	+	GGGCACTGGGGCCAGTCTGGGCGCCGCGGCGGGG<br>(((....)))	KAZN	NM_201628	chr1:14797800-15317131	non-template	34	Intron_1.f	--	Yes	--	Yes	--	--	--	Yes	Yes	Yes	Yes
G4ST01015123100	chr1:15123101-15123134	+	GGGCACTGGGGCCAGTCTGGGCGCCGCGGCGGGG<br>(((....)))	KAZN	NM_001018000	chr1:15123212-15267238	non-template	-77	upstream	Yes	--	--	--	--	--	--	Yes	Yes	Yes	Yes
G4ST01015123107	chr1:15123108-15123140	+	GGGGCCAGTCTGGGCGCCGCGGCGGGGCGGGGG<br>(((....)))	KAZN	NM_015209	chr1:14797800-15267238	non-template	33	Intron_1.f	--	Yes	--	Yes	--	--	--	Yes	Yes	Yes	Yes
G4ST01015123107	chr1:15123108-15123140	+	GGGGCCAGTCTGGGCGCCGCGGCGGGGCGGGGG<br>(((....)))	KAZN	NM_201628	chr1:14797800-15317131	non-template	33	Intron_1.f	--	Yes	--	Yes	--	--	--	Yes	Yes	Yes	Yes
G4ST01015123107	chr1:15123108-15123140	+	GGGGCCAGTCTGGGCGCCGCGGCGGGGCGGGGG<br>(((....)))	KAZN	NM_001018000	chr1:15123212-15267238	non-template	-71	upstream	Yes	--	--	--	--	--	--	Yes	Yes	Yes	Yes

G4ST01015123118	chr1:15123119-15123145	+	GGGCGCCGCGGCGGGGCGGGGGCGGGG<br>(((....)))	KAZN	NM_015209	chr1:14797800-15267238	non-template	27	Intron_1.f	--	Yes	--	Yes	--	--	--	Yes	Yes	Yes	Yes
G4ST01015123118	chr1:15123119-15123145	+	GGGCGCCGCGGCGGGGCGGGGGCGGGG<br>(((....)))	KAZN	NM_201628	chr1:14797800-15317131	non-template	27	Intron_1.f	--	Yes	--	Yes	--	--	--	Yes	Yes	Yes	Yes
G4ST01015123118	chr1:15123119-15123145	+	GGGCGCCGCGGCGGGGCGGGGGCGGGG<br>(((....)))	KAZN	NM_001018000	chr1:15123212-15267238	non-template	-66	upstream	Yes	--	--	--	--	--	--	Yes	Yes	Yes	Yes
G4ST01015123713	chr1:15123714-15123746	+	GGGGCCGGGCGCGCGAGGGCGCTCGGAGCCGGG<br>.(((...))).	KAZN	NM_015209	chr1:14797800-15267238	non-template	33	Intron_1.f	--	Yes	--	Yes	--	--	--	Yes	Yes	Yes	--
G4ST01015123713	chr1:15123714-15123746	+	GGGGCCGGGCGCGCGAGGGCGCTCGGAGCCGGG<br>.(((...))).	KAZN	NM_201628	chr1:14797800-15317131	non-template	33	Intron_1.f	--	Yes	--	Yes	--	--	--	Yes	Yes	Yes	--
G4ST01015123713	chr1:15123714-15123746	+	GGGGCCGGGCGCGCGAGGGCGCTCGGAGCCGGG<br>.(((...))).	KAZN	NM_001018000	chr1:15123212-15267238	non-template	33	Intron_1.f	--	Yes	--	Yes	--	--	--	Yes	Yes	Yes	--
G4ST01015123719	chr1:15123720-15123752	+	GGGCGCGCGAGGGCGCTCGGAGCCGGGATCGGG<br>.(((...))).	KAZN	NM_015209	chr1:14797800-15267238	non-template	33	Intron_1.f	--	Yes	--	Yes	--	--	--	Yes	Yes	Yes	--
G4ST01015123719	chr1:15123720-15123752	+	GGGCGCGCGAGGGCGCTCGGAGCCGGGATCGGG<br>.(((...))).	KAZN	NM_201628	chr1:14797800-15317131	non-template	33	Intron_1.f	--	Yes	--	Yes	--	--	--	Yes	Yes	Yes	--
G4ST01015123719	chr1:15123720-15123752	+	GGGCGCGCGAGGGCGCTCGGAGCCGGGATCGGG<br>.(((...))).	KAZN	NM_001018000	chr1:15123212-15267238	non-template	33	Intron_1.f	--	Yes	--	Yes	--	--	--	Yes	Yes	Yes	--
G4ST01015135062	chr1:15135063-15135096	-	GGGCAGGGGGGCTTGGAGGCTCCAGGGAAATGGG<br>.....((((...))))	KAZN	NM_015209	chr1:14797800-15267238	template	34	Intron_1.f	--	Yes	--	Yes	--	--	--	--	--	--	--
G4ST01015135062	chr1:15135063-15135096	-	GGGCAGGGGGGCTTGGAGGCTCCAGGGAAATGGG<br>.....((((...))))	KAZN	NM_201628	chr1:14797800-15317131	template	34	Intron_1.f	--	Yes	--	Yes	--	--	--	--	--	--	--
G4ST01015135062	chr1:15135063-15135096	-	GGGCAGGGGGGCTTGGAGGCTCCAGGGAAATGGG<br>.....((((...))))	KAZN	NM_001018001	chr1:15128883-15267238	template	34	Intron_1.f	--	Yes	--	Yes	--	--	--	--	--	--	--
G4ST01015135062	chr1:15135063-15135096	-	GGGCAGGGGGGCTTGGAGGCTCCAGGGAAATGGG<br>.....((((...))))	KAZN	NM_001018000	chr1:15123212-15267238	template	34	Intron_1.f	--	Yes	--	Yes	--	--	--	--	--	--	--
G4ST01015147861	chr1:15147862-15147890	-	GGGCTGACTTGGGATGGGAGCTTACTGGG<br>((....))	KAZN	NM_015209	chr1:14797800-15267238	template	29	Intron_1.f	--	Yes	--	Yes	--	--	--	Yes	--	--	--
G4ST01015147861	chr1:15147862-15147890	-	GGGCTGACTTGGGATGGGAGCTTACTGGG<br>((....))	KAZN	NM_201628	chr1:14797800-15317131	template	29	Intron_1.f	--	Yes	--	Yes	--	--	--	Yes	--	--	--
G4ST01015147861	chr1:15147862-15147890	-	GGGCTGACTTGGGATGGGAGCTTACTGGG<br>((....))	KAZN	NM_001017999	chr1:15145002-15267238	template	29	Intron_1.f	--	Yes	--	Yes	--	--	--	Yes	--	--	--
G4ST01015147861	chr1:15147862-15147890	-	GGGCTGACTTGGGATGGGAGCTTACTGGG<br>((....))	KAZN	NM_001018001	chr1:15128883-15267238	template	29	Intron_1.f	--	Yes	--	Yes	--	--	--	Yes	--	--	--
G4ST01015147861	chr1:15147862-15147890	-	GGGCTGACTTGGGATGGGAGCTTACTGGG<br>((....))	KAZN	NM_001018000	chr1:15123212-15267238	template	29	Intron_1.f	--	Yes	--	Yes	--	--	--	Yes	--	--	--
G4ST01015157001	chr1:15157002-15157025	+	GGGAGGGCAGGGAGGTGCCCTGGG<br>(((...)))	KAZN	NM_015209	chr1:14797800-15267238	non-template	24	Intron_1.f	--	Yes	--	Yes	--	--	--	--	--	--	--
G4ST01015157001	chr1:15157002-15157025	+	GGGAGGGCAGGGAGGTGCCCTGGG<br>(((...)))	KAZN	NM_201628	chr1:14797800-15317131	non-template	24	Intron_1.f	--	Yes	--	Yes	--	--	--	--	--	--	--
G4ST01015157001	chr1:15157002-15157025	+	GGGAGGGCAGGGAGGTGCCCTGGG<br>(((...)))	KAZN	NM_001017999	chr1:15145002-15267238	non-template	24	Intron_1.f	--	Yes	--	Yes	--	--	--	--	--	--	--
G4ST01015157001	chr1:15157002-15157025	+	GGGAGGGCAGGGAGGTGCCCTGGG<br>(((...)))	KAZN	NM_001018001	chr1:15128883-15267238	non-template	24	Intron_1.f	--	Yes	--	Yes	--	--	--	--	--	--	--
G4ST01015157001	chr1:15157002-15157025	+	GGGAGGGCAGGGAGGTGCCCTGGG<br>(((...)))	KAZN	NM_001018000	chr1:15123212-15267238	non-template	24	Intron_1.f	--	Yes	--	Yes	--	--	--	--	--	--	--
G4ST01015158538	chr1:15158539-15158569	-	GGGGAGGGGACAAGGGCAGCCCTGGCTGGGG<br>((((....))))	KAZN	NM_015209	chr1:14797800-15267238	template	31	Intron_1.f	--	Yes	--	Yes	--	--	--	--	--	--	Yes
G4ST01015158538	chr1:15158539-15158569	-	GGGGAGGGGACAAGGGCAGCCCTGGCTGGGG<br>((((....))))	KAZN	NM_201628	chr1:14797800-15317131	template	31	Intron_1.f	--	Yes	--	Yes	--	--	--	--	--	--	Yes
G4ST01015158538	chr1:15158539-15158569	-	GGGGAGGGGACAAGGGCAGCCCTGGCTGGGG<br>((((....))))	KAZN	NM_001017999	chr1:15145002-15267238	template	31	Intron_1.f	--	Yes	--	Yes	--	--	--	--	--	--	Yes
G4ST01015158538	chr1:15158539-15158569	-	GGGGAGGGGACAAGGGCAGCCCTGGCTGGGG<br>((((....))))	KAZN	NM_001018001	chr1:15128883-15267238	template	31	Intron_1.f	--	Yes	--	Yes	--	--	--	--	--	--	Yes
G4ST01015158538	chr1:15158539-15158569	-	GGGGAGGGGACAAGGGCAGCCCTGGCTGGGG<br>((((....))))	KAZN	NM_001018000	chr1:15123212-15267238	template	31	Intron_1.f	--	Yes	--	Yes	--	--	--	--	--	--	Yes
G4ST01015198561	chr1:15198562-15198595	-	GGGGCGGGGGGAGGGGGGCAGGCAATGCCTGGGG<br>...(((((...)))))	KAZN	NM_015209	chr1:14797800-15267238	template	34	Intron_2	--	Yes	--	Yes	--	--	--	Yes	--	--	--
G4ST01015198561	chr1:15198562-15198595	-	GGGGCGGGGGGAGGGGGGCAGGCAATGCCTGGGG<br>...(((((...)))))	KAZN	NM_201628	chr1:14797800-15317131	template	34	Intron_2	--	Yes	--	Yes	--	--	--	Yes	--	--	--
G4ST01015198561	chr1:15198562-15198595	-	GGGGCGGGGGGAGGGGGGCAGGCAATGCCTGGGG<br>...(((((...)))))	KAZN	NM_001017999	chr1:15145002-15267238	template	34	Intron_2	--	Yes	--	Yes	--	--	--	Yes	--	--	--
G4ST01015198561	chr1:15198562-15198595	-	GGGGCGGGGGGAGGGGGGCAGGCAATGCCTGGGG<br>...(((((...)))))	KAZN	NM_001018001	chr1:15128883-15267238	template	34	Intron_2	--	Yes	--	Yes	--	--	--	Yes	--	--	--
G4ST01015198561	chr1:15198562-15198595	-	GGGGCGGGGGGAGGGGGGCAGGCAATGCCTGGGG<br>...(((((...)))))	KAZN	NM_001018000	chr1:15123212-15267238	template	34	Intron_2	--	Yes	--	Yes	--	--	--	Yes	--	--	--
G4ST01015199137	chr1:15199138-15199173	+	GGGAAGATGATCTGGATTGTCTGGGTGGGCCCTGGG<br>.(((((((....)))))))	KAZN	NM_015209	chr1:14797800-15267238	non-template	36	Intron_2	--	Yes	--	Yes	--	--	--	--	--	--	Yes
G4ST01015199137	chr1:15199138-15199173	+	GGGAAGATGATCTGGATTGTCTGGGTGGGCCCTGGG<br>.(((((((....)))))))	KAZN	NM_201628	chr1:14797800-15317131	non-template	36	Intron_2	--	Yes	--	Yes	--	--	--	--	--	--	Yes
G4ST01015199137	chr1:15199138-15199173	+	GGGAAGATGATCTGGATTGTCTGGGTGGGCCCTGGG<br>.(((((((....)))))))	KAZN	NM_001017999	chr1:15145002-15267238	non-template	36	Intron_2	--	Yes	--	Yes	--	--	--	--	--	--	Yes
G4ST01015199137	chr1:15199138-15199173	+	GGGAAGATGATCTGGATTGTCTGGGTGGGCCCTGGG<br>.(((((((....)))))))	KAZN	NM_001018001	chr1:15128883-15267238	non-template	36	Intron_2	--	Yes	--	Yes	--	--	--	--	--	--	Yes
G4ST01015199137	chr1:15199138-15199173	+	GGGAAGATGATCTGGATTGTCTGGGTGGGCCCTGGG<br>.(((((((....)))))))	KAZN	NM_001018000	chr1:15123212-15267238	non-template	36	Intron_2	--	Yes	--	Yes	--	--	--	--	--	--	Yes
G4ST01015208132	chr1:15208133-15208167	-	GGGAAGGGGGCTGCTGGCTCACCCCAGCGGGCGGG<br>....(((((......)))))	KAZN	NM_015209	chr1:14797800-15267238	template	35	Intron_2	--	Yes	--	Yes	--	--	--	--	--	--	--
G4ST01015208132	chr1:15208133-15208167	-	GGGAAGGGGGCTGCTGGCTCACCCCAGCGGGCGGG<br>....(((((......)))))	KAZN	NM_201628	chr1:14797800-15317131	template	35	Intron_2	--	Yes	--	Yes	--	--	--	--	--	--	--
G4ST01015208132	chr1:15208133-15208167	-	GGGAAGGGGGCTGCTGGCTCACCCCAGCGGGCGGG<br>....(((((......)))))	KAZN	NM_001017999	chr1:15145002-15267238	template	35	Intron_2	--	Yes	--	Yes	--	--	--	--	--	--	--
G4ST01015208132	chr1:15208133-15208167	-	GGGAAGGGGGCTGCTGGCTCACCCCAGCGGGCGGG<br>....(((((......)))))	KAZN	NM_001018001	chr1:15128883-15267238	template	35	Intron_2	--	Yes	--	Yes	--	--	--	--	--	--	--
G4ST01015208132	chr1:15208133-15208167	-	GGGAAGGGGGCTGCTGGCTCACCCCAGCGGGCGGG<br>....(((((......)))))	KAZN	NM_001018000	chr1:15123212-15267238	template	35	Intron_2	--	Yes	--	Yes	--	--	--	--	--	--	--
G4ST01015217896	chr1:15217897-15217922	-	GGGAGGGGAGGGCAGCTGCTGGGGGG<br>(((...)))..	KAZN	NM_015209	chr1:14797800-15267238	template	26	Intron_2	--	Yes	--	Yes	--	--	--	--	--	--	--
G4ST01015217896	chr1:15217897-15217922	-	GGGAGGGGAGGGCAGCTGCTGGGGGG<br>(((...)))..	KAZN	NM_201628	chr1:14797800-15317131	template	26	Intron_2	--	Yes	--	Yes	--	--	--	--	--	--	--
G4ST01015217896	chr1:15217897-15217922	-	GGGAGGGGAGGGCAGCTGCTGGGGGG<br>(((...)))..	KAZN	NM_001017999	chr1:15145002-15267238	template	26	Intron_2	--	Yes	--	Yes	--	--	--	--	--	--	--
G4ST01015217896	chr1:15217897-15217922	-	GGGAGGGGAGGGCAGCTGCTGGGGGG<br>(((...)))..	KAZN	NM_001018001	chr1:15128883-15267238	template	26	Intron_2	--	Yes	--	Yes	--	--	--	--	--	--	--
G4ST01015217896	chr1:15217897-15217922	-	GGGAGGGGAGGGCAGCTGCTGGGGGG<br>(((...)))..	KAZN	NM_001018000	chr1:15123212-15267238	template	26	Intron_2	--	Yes	--	Yes	--	--	--	--	--	--	--
G4ST01015248533	chr1:15248534-15248574	-	GGGGGGCTGGGGTGGAGGGGGTGCTGCTGGCATCTGGTGGG<br>(((((.....)))))....	KAZN	NM_015209	chr1:14797800-15267238	template	41	Intron_4	--	Yes	--	Yes	--	--	--	--	--	--	--
G4ST01015248533	chr1:15248534-15248574	-	GGGGGGCTGGGGTGGAGGGGGTGCTGCTGGCATCTGGTGGG<br>(((((.....)))))....	KAZN	NM_201628	chr1:14797800-15317131	template	41	Intron_4	--	Yes	--	Yes	--	--	--	--	--	--	--

G4ST01015248533	chr1:15248534-15248574	-	GGGGGGCTGGGGTGGAGGGGGTGCTGCTGGCATCTGGTGGG<br>(((((.....)))))....	KAZN	NM_001017999	chr1:15145002-15267238	template	41	Intron_4	--	Yes	--	Yes	--	--	--	--	--	--	--
G4ST01015248533	chr1:15248534-15248574	-	GGGGGGCTGGGGTGGAGGGGGTGCTGCTGGCATCTGGTGGG<br>(((((.....)))))....	KAZN	NM_001018001	chr1:15128883-15267238	template	41	Intron_4	--	Yes	--	Yes	--	--	--	--	--	--	--
G4ST01015248533	chr1:15248534-15248574	-	GGGGGGCTGGGGTGGAGGGGGTGCTGCTGGCATCTGGTGGG<br>(((((.....)))))....	KAZN	NM_001018000	chr1:15123212-15267238	template	41	Intron_4	--	Yes	--	Yes	--	--	--	--	--	--	--
G4ST01015300852	chr1:15300853-15300883	+	GGGAGGTGAGGACCAGGGCACAGGGTGGGGG<br>.(((....))).	KAZN	NM_201628	chr1:14797800-15317131	non-template	31	Exon_11-Intron_11	--	Yes	Yes	Yes	Yes	--	--	--	--	--	--
G4ST01015302422	chr1:15302423-15302463	-	GGGCCCGGCGCTCCTGGAGGGCCTTGTGGGGAGACAGTGGG<br>.((((.....)))).	KAZN	NM_201628	chr1:14797800-15317131	template	41	Intron_11-Exon_12	--	Yes	Yes	Yes	Yes	--	--	--	Yes	--	--
G4ST01015352802	chr1:15352803-15352829	-	GGGAGCGCCGGCGTGCGGGAGGGAGGG<br>.((((....))))	TMEM51	NM_001136216	chr1:15351615-15419561	template	27	Intron_1.f	--	Yes	--	Yes	--	--	--	Yes	Yes	Yes	Yes
G4ST01015352802	chr1:15352803-15352829	-	GGGAGCGCCGGCGTGCGGGAGGGAGGG<br>.((((....))))	TMEM51	NM_001136217	chr1:15351615-15419561	template	27	Intron_1.f	--	Yes	--	Yes	--	--	--	Yes	Yes	Yes	Yes
G4ST01015352802	chr1:15352803-15352829	-	GGGAGCGCCGGCGTGCGGGAGGGAGGG<br>.((((....))))	TMEM51	NM_001136218	chr1:15352816-15419561	template	14	upstream-Exon_1.f	Yes	Yes	Yes	--	--	Yes	--	Yes	Yes	Yes	Yes
G4ST01015352802	chr1:15352803-15352829	-	GGGAGCGCCGGCGTGCGGGAGGGAGGG<br>.((((....))))	TMEM51	NM_018022	chr1:15352816-15419561	template	14	upstream-Exon_1.f	Yes	Yes	Yes	--	--	Yes	--	Yes	Yes	Yes	Yes
G4ST01015352802	chr1:15352803-15352829	-	GGGAGCGCCGGCGTGCGGGAGGGAGGG<br>.((((....))))	C1orf126	NR_027136	chr1:15310898-15351547	non-template	-1255	upstream	Yes	--	--	--	--	--	--	Yes	Yes	Yes	Yes
G4ST01015405212	chr1:15405213-15405256	+	GGGGGCCGAGGTGGGAGGATTGCTTAAGCCTGGGAGGTCAAGGG<br>(((.((....)).)))	TMEM51	NM_001136216	chr1:15351615-15419561	non-template	44	Intron_1.f	--	Yes	--	Yes	--	--	--	--	Yes	--	--
G4ST01015405212	chr1:15405213-15405256	+	GGGGGCCGAGGTGGGAGGATTGCTTAAGCCTGGGAGGTCAAGGG<br>(((.((....)).)))	TMEM51	NM_001136217	chr1:15351615-15419561	non-template	44	Intron_1.f	--	Yes	--	Yes	--	--	--	--	Yes	--	--
G4ST01015405212	chr1:15405213-15405256	+	GGGGGCCGAGGTGGGAGGATTGCTTAAGCCTGGGAGGTCAAGGG<br>(((.((....)).)))	TMEM51	NM_001136218	chr1:15352816-15419561	non-template	44	Intron_1.f	--	Yes	--	Yes	--	--	--	--	Yes	--	--
G4ST01015405212	chr1:15405213-15405256	+	GGGGGCCGAGGTGGGAGGATTGCTTAAGCCTGGGAGGTCAAGGG<br>(((.((....)).)))	TMEM51	NM_018022	chr1:15352816-15419561	non-template	44	Intron_1.f	--	Yes	--	Yes	--	--	--	--	Yes	--	--
G4ST01015508909	chr1:15508910-15508936	+	GGGAGGGGCTGTCGGGAGGCCCCTGGG<br>((....))	FHAD1	NM_052929	chr1:15446355-15597209	non-template	27	Intron_7	--	Yes	--	Yes	--	--	--	--	--	--	--
G4ST01015509467	chr1:15509468-15509504	+	GGGCAGGGCTGCTCTGGGCACCAGGCATCCCAGGGGG<br>..((.((....)).))	FHAD1	NM_052929	chr1:15446355-15597209	non-template	37	Intron_7	--	Yes	--	Yes	--	--	--	--	--	--	--
G4ST01015524953	chr1:15524954-15524982	-	GGGCCTGGGGTCACTTGGCTGGGGAAGGG<br>(((....)))..	FHAD1	NM_052929	chr1:15446355-15597209	template	29	Intron_11	--	Yes	--	Yes	--	--	--	--	--	--	--
G4ST01015526084	chr1:15526085-15526129	-	GGGGGCAACAGTGTCAGGGGGTCAGCAGGGGCCGGCAAGTGGGGG<br>((((....)))).... .(((.....)))	FHAD1	NM_052929	chr1:15446355-15597209	template	45	Intron_11	--	Yes	--	Yes	--	--	--	--	Yes	--	--
G4ST01015536636	chr1:15536637-15536675	+	GGGGTGGGGTGAGTAGGGGTGCCCCTGGCATCCAGTGGG<br>(((((...)))))).....	FHAD1	NM_052929	chr1:15446355-15597209	non-template	39	Intron_14	--	Yes	--	Yes	--	--	--	--	Yes	--	--
G4ST01015545095	chr1:15545096-15545136	-	GGGAGCGCAGGGGAGGGCGGAGGACTGATACTCCTCCGGGG<br>(((((((.......)))))))	FHAD1	NM_052929	chr1:15446355-15597209	template	41	Intron_17	--	Yes	--	Yes	--	--	--	Yes	Yes	Yes	--
G4ST01015576433	chr1:15576434-15576468	-	GGGGAAGGCCTGTCTTGGGAGCGGGTCAGGCTGGG<br>.((((....))))	FHAD1	NM_052929	chr1:15446355-15597209	template	35	Intron_27	--	Yes	--	Yes	--	--	--	Yes	--	--	--
G4ST01015586427	chr1:15586428-15586456	+	GGGATGAGAATGGGATGGGGGTTTGGGGG<br>((....))	FHAD1	NM_052929	chr1:15446355-15597209	non-template	29	Intron_30.l	--	Yes	--	Yes	--	--	--	--	--	--	--
G4ST01015587306	chr1:15587307-15587343	-	GGGAAGGGAAGGAGGGAGGTGTGCAGTGCTCCTTGGG<br>(((.((.....)).))).	FHAD1	NM_052929	chr1:15446355-15597209	template	37	Intron_30.l	--	Yes	--	Yes	--	--	--	Yes	--	--	Yes
G4ST01015608385	chr1:15608386-15608418	-	GGGAGATGGGACTGGGTTTCACCCCTGAAAGGG<br>(((((....)))))	EFHD2	NM_024329	chr1:15608978-15629426	template	-559	upstream	Yes	--	--	--	--	--	--	Yes	Yes	--	--
G4ST01015625259	chr1:15625260-15625307	+	GGGCCTGTTACAGCAGGCACCAGGGAGGGTTCTGAGATCCTCTCGGGG<br>(((((....)))))..... ....((((...)))).	EFHD2	NM_024329	chr1:15608978-15629426	non-template	48	Intron_2	--	Yes	--	Yes	--	--	--	--	Yes	--	--
G4ST01015628618	chr1:15628619-15628650	+	GGGTCTCGGGGCTCTGGAGAGAGTGGGGCGGG<br>.((((....))))..	EFHD2	NM_024329	chr1:15608978-15629426	non-template	32	Exon_4.l	--	Yes	Yes	--	--	--	Yes	--	Yes	Yes	--
G4ST01015662610	chr1:15662611-15662645	+	GGGAGCCAGGAGCCCCCAGGCCTGGGAGGGAAGGG<br>.(((.((.....)).)))..	CELA2A	NM_033440	chr1:15655810-15671173	non-template	35	Intron_5	--	Yes	--	Yes	--	--	--	Yes	Yes	--	--
G4ST01015682488	chr1:15682489-15682523	+	GGGAGCCAGGAGCCCCCAGGCCTGGGAGGGAAGGG<br>.(((.((.....)).)))..	CELA2B	NM_015849	chr1:15675183-15690482	non-template	35	Intron_5	--	Yes	--	Yes	--	--	--	--	--	--	--
G4ST01015704835	chr1:15704836-15704862	-	GGGAGAAGCCCTCTGGGAGAGGGAGGG<br>(((.....)))	CASP9	NM_032996	chr1:15691356-15723872	non-template	27	Intron_5	--	Yes	--	Yes	--	--	--	--	--	--	--
G4ST01015704835	chr1:15704836-15704862	-	GGGAGAAGCCCTCTGGGAGAGGGAGGG<br>(((.....)))	CASP9	NM_001229	chr1:15691356-15723527	non-template	27	Intron_5	--	Yes	--	Yes	--	--	--	--	--	--	--
G4ST01015717113	chr1:15717114-15717163	-	GGGCATGACACGTAGTCATTTGGGACTCACTATGAGTACAGCTGGGTGGG<br>.(((((.....))))).. (((((...)))))......	CASP9	NM_032996	chr1:15691356-15723872	non-template	50	Intron_2	--	Yes	--	Yes	--	--	--	--	--	--	--
G4ST01015717113	chr1:15717114-15717163	-	GGGCATGACACGTAGTCATTTGGGACTCACTATGAGTACAGCTGGGTGGG<br>.(((((.....))))).. (((((...)))))......	CASP9	NM_001229	chr1:15691356-15723527	non-template	50	Intron_2	--	Yes	--	Yes	--	--	--	--	--	--	--
G4ST01015717117	chr1:15717118-15717167	-	GGGTGGGCATGACACGTAGTCATTTGGGACTCACTATGAGTACAGCTGGG<br>.(((((.....))))).. (((((...)))))......	CASP9	NM_032996	chr1:15691356-15723872	non-template	50	Intron_2	--	Yes	--	Yes	--	--	--	--	--	--	--
G4ST01015717117	chr1:15717118-15717167	-	GGGTGGGCATGACACGTAGTCATTTGGGACTCACTATGAGTACAGCTGGG<br>.(((((.....))))).. (((((...)))))......	CASP9	NM_001229	chr1:15691356-15723527	non-template	50	Intron_2	--	Yes	--	Yes	--	--	--	--	--	--	--
G4ST01015723357	chr1:15723358-15723384	-	GGGGGCAGGCCCTGGGGCGGGGGCGGG<br>..(((...)))	CASP9	NM_032996	chr1:15691356-15723872	non-template	27	Intron_1.f	--	Yes	--	Yes	--	--	--	Yes	Yes	Yes	Yes
G4ST01015723357	chr1:15723358-15723384	-	GGGGGCAGGCCCTGGGGCGGGGGCGGG<br>..(((...)))	CASP9	NM_001229	chr1:15691356-15723527	non-template	27	Exon_1.f	--	Yes	Yes	--	--	Yes	--	Yes	Yes	Yes	Yes
G4ST01015723361	chr1:15723362-15723397	-	GGGGCCGTGACGCGGGGGCAGGCCCTGGGGCGGGGG<br>.(((.....))) ..(((...)))	CASP9	NM_032996	chr1:15691356-15723872	non-template	36	Intron_1.f	--	Yes	--	Yes	--	--	--	Yes	Yes	Yes	Yes
G4ST01015723361	chr1:15723362-15723397	-	GGGGCCGTGACGCGGGGGCAGGCCCTGGGGCGGGGG<br>.(((.....))) ..(((...)))	CASP9	NM_001229	chr1:15691356-15723527	non-template	36	Exon_1.f	--	Yes	Yes	--	--	Yes	--	Yes	Yes	Yes	Yes
G4ST01015723640	chr1:15723641-15723680	-	GGGGCGGTGACGTGAGGTCAGTGCGGGGATACGGGGCGGG<br>(((.((((.....)))).))).	CASP9	NM_032996	chr1:15691356-15723872	non-template	40	Exon_1.f-Intron_1.f	--	Yes	Yes	Yes	--	Yes	--	Yes	Yes	Yes	Yes
G4ST01015723640	chr1:15723641-15723680	-	GGGGCGGTGACGTGAGGTCAGTGCGGGGATACGGGGCGGG<br>(((.((((.....)))).))).	CASP9	NM_001229	chr1:15691356-15723527	non-template	-113	upstream	Yes	--	--	--	--	--	--	Yes	Yes	Yes	Yes
G4ST01015723644	chr1:15723645-15723686	-	GGGGGCGGGGCGGTGACGTGAGGTCAGTGCGGGGATACGGGG<br>(((.((((.....)))).))).	CASP9	NM_032996	chr1:15691356-15723872	non-template	42	Exon_1.f-Intron_1.f	--	Yes	Yes	Yes	--	Yes	--	Yes	Yes	Yes	Yes
G4ST01015723644	chr1:15723645-15723686	-	GGGGGCGGGGCGGTGACGTGAGGTCAGTGCGGGGATACGGGG<br>(((.((((.....)))).))).	CASP9	NM_001229	chr1:15691356-15723527	non-template	-117	upstream	Yes	--	--	--	--	--	--	Yes	Yes	Yes	Yes
G4ST01015745292	chr1:15745293-15745333	+	GGGAGGATCACTTGATCCTGGGAGGTTTGGGAGGTTGAGGG<br>((((((....))))))	DNAJC16	NM_015291	chr1:15725939-15770815	non-template	41	Intron_5	--	Yes	--	Yes	--	--	--	--	--	--	--
G4ST01015777732	chr1:15777733-15777763	-	GGGCCATGGGAAGTCTATCGCTGGGCGGGGG<br>.(((.....)))	AGMAT	NM_024758	chr1:15771739-15784192	non-template	31	Intron_4	--	Yes	--	Yes	--	--	--	--	Yes	--	--
G4ST01015883770	chr1:15883771-15883798	-	GGGGAAGGGCGGGGGGCCCCGGCCCGGG<br>((((....))))	PLEKHM2	NM_015164	chr1:15883414-15933851	template	28	Intron_1.f	--	Yes	--	Yes	--	--	--	Yes	Yes	Yes	Yes
G4ST01015883977	chr1:15883978-15884032	-	GGGGTTCCTTCAGGAGCTCCCGGGGGCGCCGCTGCCCCAGTGGGGGACCGGAGGG<br>(((((....)))))...... ....((((((...))))))	PLEKHM2	NM_015164	chr1:15883414-15933851	template	55	Intron_1.f	--	Yes	--	Yes	--	--	--	Yes	Yes	Yes	--
G4ST01015937610	chr1:15937611-15937632	+	GGGAGGGCACGACGTGGGTGGG<br>.((...))	SLC25A34	NM_207348	chr1:15935396-15940471	non-template	22	Intron_3	--	Yes	--	Yes	--	--	--	--	--	--	--
G4ST01015944007	chr1:15944008-15944047	+	GGGCACAGGGAAGGGGTGAGGCTGGCCAGGTTCGAAGGGG<br>.((((.((....)).))))....	TMEM82	NM_001013641	chr1:15941504-15947064	non-template	40	Intron_4	--	Yes	--	Yes	--	--	--	--	--	--	--
G4ST01015956912	chr1:15956913-15956950	-	GGGGAGGCAGAGCCTGGACTCTGGGGCCCGGGATGGGG<br>....((((((......)))))	FBLIM1	NM_017556	chr1:15957842-15985671	template	-891	upstream	Yes	--	--	--	--	--	--	Yes	Yes	--	Yes
G4ST01015956918	chr1:15956919-15956954	-	GGGAGGGGAGGCAGAGCCTGGACTCTGGGGCCCGGG<br>....(((((......)))))	FBLIM1	NM_017556	chr1:15957842-15985671	template	-887	upstream	Yes	--	--	--	--	--	--	Yes	Yes	--	Yes

G4ST01015956924	chr1:15956925-15956960	-	GGGAGCGGGAGGGGAGGCAGAGCCTGGACTCTGGGG<br>....(((((......)))))	FBLIM1	NM_017556	chr1:15957842-15985671	template	-881	upstream	Yes	--	--	--	--	--	--	Yes	Yes	--	Yes
G4ST01015957708	chr1:15957709-15957747	+	GGGCAGGTTGGGGGGCAGGTAGGGGCCGCTTCCGCGGGG<br>..(((....)))	FBLIM1	NM_017556	chr1:15957842-15985671	non-template	-94	upstream	Yes	--	--	--	--	--	--	Yes	--	--	Yes
G4ST01015957717	chr1:15957718-15957751	+	GGGGGGCAGGTAGGGGCCGCTTCCGCGGGGCGGG<br>..(((....)))	FBLIM1	NM_017556	chr1:15957842-15985671	non-template	-90	upstream	Yes	--	--	--	--	--	--	Yes	--	--	Yes
G4ST01015960230	chr1:15960231-15960265	-	GGGCTGGTCCCCAGGGGCTGCTGTGGGGCTTCGGG<br>((((...))))	FBLIM1	NM_017556	chr1:15957842-15985671	template	35	Intron_1.f	--	Yes	--	Yes	--	--	--	Yes	Yes	--	--
G4ST01015963123	chr1:15963124-15963158	-	GGGTCCTGGTTCAGGCCTGGGCAGTGGGCAGGGGG<br>.((((...))))...	FBLIM1	NM_001024215	chr1:15963581-15974302	template	-422	upstream	Yes	--	--	--	--	--	--	--	--	--	Yes
G4ST01015963123	chr1:15963124-15963158	-	GGGTCCTGGTTCAGGCCTGGGCAGTGGGCAGGGGG<br>.((((...))))...	FBLIM1	NM_017556	chr1:15957842-15985671	template	35	Intron_1.f	--	Yes	--	Yes	--	--	--	--	--	--	Yes
G4ST01015963123	chr1:15963124-15963158	-	GGGTCCTGGTTCAGGCCTGGGCAGTGGGCAGGGGG<br>.((((...))))...	FBLIM1	NM_001024216	chr1:15964046-15985671	template	-887	upstream	Yes	--	--	--	--	--	--	--	--	--	Yes
G4ST01015964520	chr1:15964521-15964553	+	GGGTTATTGGGATTATGGGAAGAGGATCTTGGG<br>((((...))))	FBLIM1	NM_001024215	chr1:15963581-15974302	non-template	33	Intron_2	--	Yes	--	Yes	--	--	--	--	--	--	--
G4ST01015964520	chr1:15964521-15964553	+	GGGTTATTGGGATTATGGGAAGAGGATCTTGGG<br>((((...))))	FBLIM1	NM_017556	chr1:15957842-15985671	non-template	33	Intron_3	--	Yes	--	Yes	--	--	--	--	--	--	--
G4ST01015964520	chr1:15964521-15964553	+	GGGTTATTGGGATTATGGGAAGAGGATCTTGGG<br>((((...))))	FBLIM1	NM_001024216	chr1:15964046-15985671	non-template	33	Intron_1.f	--	Yes	--	Yes	--	--	--	--	--	--	--
G4ST01015964528	chr1:15964529-15964561	+	GGGATTATGGGAAGAGGATCTTGGGAGTGTGGG<br>((((...))))	FBLIM1	NM_001024215	chr1:15963581-15974302	non-template	33	Intron_2	--	Yes	--	Yes	--	--	--	--	--	--	--
G4ST01015964528	chr1:15964529-15964561	+	GGGATTATGGGAAGAGGATCTTGGGAGTGTGGG<br>((((...))))	FBLIM1	NM_017556	chr1:15957842-15985671	non-template	33	Intron_3	--	Yes	--	Yes	--	--	--	--	--	--	--
G4ST01015964528	chr1:15964529-15964561	+	GGGATTATGGGAAGAGGATCTTGGGAGTGTGGG<br>((((...))))	FBLIM1	NM_001024216	chr1:15964046-15985671	non-template	33	Intron_1.f	--	Yes	--	Yes	--	--	--	--	--	--	--
G4ST01016151035	chr1:16151036-16151064	-	GGGTGGGGTGGGGCAGGCTGCCTGGTGGG<br>.((((...))))..	ZBTB17	NM_003443	chr1:16140951-16175214	non-template	29	Intron_2	--	Yes	--	Yes	--	--	--	Yes	Yes	--	--
G4ST01016174850	chr1:16174851-16174885	-	GGGGGGCGTCGCCGGGCGTGCTGGCGGGGAGTGGG<br>(((....)))	ZBTB17	NM_003443	chr1:16140951-16175214	non-template	35	Intron_1.f	--	Yes	--	Yes	--	--	--	--	Yes	Yes	Yes
G4ST01016174856	chr1:16174857-16174890	-	GGGGCGGGGGGCGTCGCCGGGCGTGCTGGCGGGG<br>(((....)))	ZBTB17	NM_003443	chr1:16140951-16175214	non-template	34	Intron_1.f	--	Yes	--	Yes	--	--	--	--	Yes	Yes	Yes
G4ST01016231257	chr1:16231258-16231290	-	GGGACTCTGGCAAGTAGAGAGGGAGAGGGAGGG<br>.(((((.....))))).	CLCNKA	NM_004070	chr1:16221073-16233132	template	33	Intron_16-Exon_17	--	Yes	Yes	Yes	Yes	--	--	--	--	--	--
G4ST01016231257	chr1:16231258-16231290	-	GGGACTCTGGCAAGTAGAGAGGGAGAGGGAGGG<br>.(((((.....))))).	CLCNKA	NM_001042704	chr1:16221073-16233132	template	33	Intron_16-Exon_17	--	Yes	Yes	Yes	Yes	--	--	--	--	--	--
G4ST01016243260	chr1:16243261-16243288	+	GGGGAAGGGGCTCTGAGCAGGGCTTGGG<br>(((...))).	CLCNKB	NM_000085	chr1:16242834-16256390	non-template	28	Intron_1.f	--	Yes	--	Yes	--	--	--	--	--	--	--
G4ST01016249078	chr1:16249079-16249113	-	GGGCTAGGATGGGCTTTAGGGGCTCAGGTGGCGGG<br>(((.....)))	CLCNKB	NM_001165945	chr1:16247871-16256390	template	35	Intron_3	--	Yes	--	Yes	--	--	--	--	--	--	Yes
G4ST01016249078	chr1:16249079-16249113	-	GGGCTAGGATGGGCTTTAGGGGCTCAGGTGGCGGG<br>(((.....)))	CLCNKB	NM_000085	chr1:16242834-16256390	template	35	Intron_10	--	Yes	--	Yes	--	--	--	--	--	--	Yes
G4ST01016257645	chr1:16257646-16257683	+	GGGCTGGGGGGCTGCGGGATGCCAGCGGTGCTGGGGGG<br>...(((((...))))).	FAM131C	NM_182623	chr1:16256851-16272714	template	38	Exon_7.l	--	Yes	Yes	--	Yes	--	--	--	--	--	--
G4ST01016323651	chr1:16323652-16323681	-	GGGGGCCCCGCCAGGGCTGGGGACAGAGGG<br>(((...))).	EPHA2	NM_004431	chr1:16323419-16355169	non-template	30	Exon_17.l	--	Yes	Yes	--	--	--	Yes	--	--	--	--
G4ST01016325281	chr1:16325282-16325328	-	GGGAGCATAAGTGATTTCATGAGGGGACGGAGGGGCATGGAAGGGGG<br>..(((((....))..)))..	EPHA2	NM_004431	chr1:16323419-16355169	non-template	47	Intron_16.l	--	Yes	--	Yes	--	--	--	--	--	--	--
G4ST01016326536	chr1:16326537-16326575	+	GGGACCCCAGGGGACCAGGCAGCTGGTCCCCTGGGAGGG<br>((((((....))))))....	EPHA2	NM_004431	chr1:16323419-16355169	template	39	Intron_16.l	--	Yes	--	Yes	--	--	--	--	--	--	--
G4ST01016338362	chr1:16338363-16338402	+	GGGCTGTCCAGGGCTTGGGCAGCTGCTGAAGTGGCCAGGG<br>..(((((....)))))..	EPHA2	NM_004431	chr1:16323419-16355169	template	40	Intron_3	--	Yes	--	Yes	--	--	--	Yes	Yes	--	--
G4ST01016338372	chr1:16338373-16338406	+	GGGCTTGGGCAGCTGCTGAAGTGGCCAGGGTGGG<br>..(((((....)))))..	EPHA2	NM_004431	chr1:16323419-16355169	template	34	Intron_3	--	Yes	--	Yes	--	--	--	Yes	Yes	--	--
G4ST01016344753	chr1:16344754-16344787	-	GGGGATGGCAGGGTCTGTTGCAGGGGCTTGGGGG<br>.(((...)))	EPHA2	NM_004431	chr1:16323419-16355169	non-template	34	Intron_3	--	Yes	--	Yes	--	--	--	Yes	Yes	Yes	--
G4ST01016401149	chr1:16401150-16401181	-	GGGGGTAGGGCTGGCATCCAGGGGGTTCTGGG<br>((((...)))).	ARHGEF19	NM_153213	chr1:16397186-16411691	non-template	32	Intron_13	--	Yes	--	Yes	--	--	--	--	--	--	--
G4ST01016401302	chr1:16401303-16401337	-	GGGGCAGAGGGCAGGGCTGGAGGCCAGAGTAGGGG<br>((((...))))....	ARHGEF19	NM_153213	chr1:16397186-16411691	non-template	35	Intron_13	--	Yes	--	Yes	--	--	--	--	--	--	--
G4ST01016401400	chr1:16401401-16401438	+	GGGGTGCCCGCCATGGGGACGGGGTGCCCATCATGGGG<br>(((.....))). (((.....))).	ARHGEF19	NM_153213	chr1:16397186-16411691	template	38	Intron_13	--	Yes	--	Yes	--	--	--	--	--	--	--
G4ST01016401414	chr1:16401415-16401444	+	GGGGACGGGGTGCCCATCATGGGGACGGGG<br>(((.....))).	ARHGEF19	NM_153213	chr1:16397186-16411691	template	30	Intron_13	--	Yes	--	Yes	--	--	--	--	--	--	--
G4ST01016401420	chr1:16401421-16401458	+	GGGGTGCCCATCATGGGGACGGGGTGCCTGTCATGGGG<br>(((.....))). (((.....))).	ARHGEF19	NM_153213	chr1:16397186-16411691	template	38	Intron_13	--	Yes	--	Yes	--	--	--	--	--	--	--
G4ST01016401434	chr1:16401435-16401464	+	GGGGACGGGGTGCCTGTCATGGGGAGGGGG<br>(((.....))).	ARHGEF19	NM_153213	chr1:16397186-16411691	template	30	Intron_13	--	Yes	--	Yes	--	--	--	--	--	--	--
G4ST01016405783	chr1:16405784-16405812	-	GGGTGCGGGTAGGGGGCGGAGCCAGCGGG<br>(((...)))...	ARHGEF19	NM_153213	chr1:16397186-16411691	non-template	29	Intron_6	--	Yes	--	Yes	--	--	--	Yes	Yes	Yes	Yes
G4ST01016406002	chr1:16406003-16406049	-	GGGACTGCAAGCTGCAGGAGGTGGGCGCGCGCGGGGACCCGGCGGGG<br>.(((((...)))))....	ARHGEF19	NM_153213	chr1:16397186-16411691	non-template	47	Exon_6-Intron_6	--	Yes	Yes	Yes	Yes	--	--	Yes	Yes	Yes	Yes
G4ST01016436102	chr1:16436103-16436132	-	GGGGAGGGCTAGGTAGCCCTGGGAGTCGGG<br>..(((....)))	RSG1	NM_030907	chr1:16430769-16436246	non-template	30	Exon_1.f	--	Yes	Yes	--	--	Yes	--	--	Yes	Yes	--
G4ST01016450083	chr1:16450084-16450108	+	GGGCAGGCCCTGGGCGAGGGATGGG<br>.((...))	FBXO42	NM_018994	chr1:16449146-16551535	template	25	Exon_10.l	--	Yes	Yes	--	Yes	--	--	--	--	--	--
G4ST01016566635	chr1:16566636-16566673	-	GGGGGGTCAGGAAAGGAGGCCCCGGGTGGCGGGTGGGG<br>(((((.........))))).	C1orf144	NM_001114600	chr1:16566170-16597227	template	38	Intron_1.f	--	Yes	--	Yes	--	--	--	Yes	Yes	Yes	Yes
G4ST01016566635	chr1:16566636-16566673	-	GGGGGGTCAGGAAAGGAGGCCCCGGGTGGCGGGTGGGG<br>(((((.........))))).	C1orf144	NM_015609	chr1:16566170-16597227	template	38	Intron_1.f	--	Yes	--	Yes	--	--	--	Yes	Yes	Yes	Yes
G4ST01016566640	chr1:16566641-16566679	-	GGGCGCGGGGGGTCAGGAAAGGAGGCCCCGGGTGGCGGG<br>(((((.........))))).	C1orf144	NM_001114600	chr1:16566170-16597227	template	39	Intron_1.f	--	Yes	--	Yes	--	--	--	Yes	Yes	Yes	Yes
G4ST01016566640	chr1:16566641-16566679	-	GGGCGCGGGGGGTCAGGAAAGGAGGCCCCGGGTGGCGGG<br>(((((.........))))).	C1orf144	NM_015609	chr1:16566170-16597227	template	39	Intron_1.f	--	Yes	--	Yes	--	--	--	Yes	Yes	Yes	Yes
G4ST01016630245	chr1:16630246-16630272	+	GGGGAGGAAGCCTGGGCAGGGGAAGGG<br>.(((...)))	SPATA21	NM_198546	chr1:16597725-16636506	template	27	Intron_3	--	Yes	--	Yes	--	--	--	--	--	--	--
G4ST01016631620	chr1:16631621-16631652	-	GGGAGGTGGGCACAGAGGCTGGGGTCTGTGGG<br>..(((...)))	SPATA21	NM_198546	chr1:16597725-16636506	non-template	32	Intron_2	--	Yes	--	Yes	--	--	--	--	--	--	--
G4ST01016650127	chr1:16650128-16650157	-	GGGGTTACTCAGGGCAGTTACTGGGAGGGG<br>.((...))	NECAP2	NM_018090	chr1:16639754-16659171	template	30	Intron_5	--	Yes	--	Yes	--	--	--	--	Yes	--	--
G4ST01016650127	chr1:16650128-16650157	-	GGGGTTACTCAGGGCAGTTACTGGGAGGGG<br>.((...))	NECAP2	NM_001145278	chr1:16639754-16659171	template	30	Intron_5	--	Yes	--	Yes	--	--	--	--	Yes	--	--
G4ST01016650127	chr1:16650128-16650157	-	GGGGTTACTCAGGGCAGTTACTGGGAGGGG<br>.((...))	NECAP2	NM_001145277	chr1:16639754-16659171	template	30	Intron_5	--	Yes	--	Yes	--	--	--	--	Yes	--	--
G4ST01016683184	chr1:16683185-16683223	-	GGGGCCAGGGTCCGGGGGAGGAGTCCGAGCGCCTTGGGG<br>..(((.((....)).)))..	CROCCP3	NR_023386	chr1:16666518-16691783	non-template	39	Intron_9	--	Yes	--	Yes	--	--	--	--	--	--	Yes
G4ST01016843539	chr1:16843540-16843578	-	GGGGCCAGGCGGGGGCGACTTGGGGGAGCCATCCTTGGG<br>(((....)))..	MST1P2	NR_027504	chr1:16844656-16849502	template	-1077	upstream	Yes	--	--	--	--	--	--	Yes	Yes	Yes	--
G4ST01016849041	chr1:16849042-16849075	+	GGGCCCTGGGCCAGCCCTGGAAGGGTATGGGGGG<br>(((....)))..	MST1P2	NR_027504	chr1:16844656-16849502	non-template	34	Intron_13.l	--	Yes	--	Yes	--	--	--	--	--	--	--

G4ST01016892421	chr1:16892422-16892460	+	GGGCCCAGGGTGTCCAGGGGCTCCCAGTCGGCCAGCGGG<br>(((.((....))..)))	ESPNP	NR_026567	chr1:16890300-16919239	template	39	Intron_10.l	--	Yes	--	Yes	--	--	--	Yes	--	--	--
G4ST01016892428	chr1:16892429-16892467	+	GGGTGTCCAGGGGCTCCCAGTCGGCCAGCGGGCCGTGGG<br>(((.((....))..)))	ESPNP	NR_026567	chr1:16890300-16919239	template	39	Intron_10.l	--	Yes	--	Yes	--	--	--	Yes	--	--	--
G4ST01016895816	chr1:16895817-16895851	+	GGGCCAGGGAAGCCGGGTCGCAGGCTCCTGCTGGG<br>..((((....)))).	ESPNP	NR_026567	chr1:16890300-16919239	template	35	Intron_9	--	Yes	--	Yes	--	--	--	Yes	Yes	--	--
G4ST01016896382	chr1:16896383-16896417	-	GGGAGAGGGCCAGAGGGAGGCTCCACCCAGCCGGG<br>.((((......))))	ESPNP	NR_026567	chr1:16890300-16919239	non-template	35	Intron_8	--	Yes	--	Yes	--	--	--	Yes	Yes	Yes	--
G4ST01016906855	chr1:16906856-16906888	-	GGGGCGGAGGACCGTGGGCGGGGCCTGCAGGGG<br>.(((....))).	ESPNP	NR_026567	chr1:16890300-16919239	non-template	33	Intron_2	--	Yes	--	Yes	--	--	--	Yes	Yes	--	--
G4ST01016916086	chr1:16916087-16916129	+	GGGCCCCAGGACCCTCCTGGGAGGGTGGCCACTGGCCTTGGGG<br>....((((...)))) .((((...))))...	ESPNP	NR_026567	chr1:16890300-16919239	template	43	Intron_1.f	--	Yes	--	Yes	--	--	--	--	--	--	--
G4ST01016956555	chr1:16956556-16956589	-	GGGCCCTGGGCCAGCCCTGGAAGGGTATGGGGGG<br>(((....)))..	MST1P9	NR_002729	chr1:16953988-16963562	non-template	34	Intron_14.l	--	Yes	--	Yes	--	--	--	--	--	--	--
G4ST01016962609	chr1:16962610-16962634	-	GGGGAGGGCAAGGGCTCACAGAGGG<br>((...)).	MST1P9	NR_002729	chr1:16953988-16963562	non-template	25	Intron_1.f	--	Yes	--	Yes	--	--	--	--	--	--	--
G4ST01017124207	chr1:17124208-17124239	+	GGGCCTCCGGGTGGCCGGGAGGTGGCCCTGGG<br>(((....)))	CROCC	NM_014675	chr1:17121032-17172061	non-template	32	Intron_3	--	Yes	--	Yes	--	--	--	--	--	--	--
G4ST01017129160	chr1:17129161-17129197	-	GGGGAGCTGATGGTGAGCCCCAGGGGTTGGGGCGGGG<br>...(((..((....)).)))	CROCC	NM_014675	chr1:17121032-17172061	template	37	Intron_4	--	Yes	--	Yes	--	--	--	--	--	--	--
G4ST01017129165	chr1:17129166-17129203	-	GGGGATGGGGAGCTGATGGTGAGCCCCAGGGGTTGGGG<br>...(((..((....)).)))	CROCC	NM_014675	chr1:17121032-17172061	template	38	Intron_4	--	Yes	--	Yes	--	--	--	--	--	--	--
G4ST01017154291	chr1:17154292-17154320	+	GGGATGGGGCAGGCTCCCTGGGTCCAGGG<br>..(((...)))	CROCC	NM_014675	chr1:17121032-17172061	non-template	29	Intron_23	--	Yes	--	Yes	--	--	--	--	--	--	--
G4ST01017154296	chr1:17154297-17154328	+	GGGGCAGGCTCCCTGGGTCCAGGGATCTGGGG<br>..(((...)))	CROCC	NM_014675	chr1:17121032-17172061	non-template	32	Intron_23	--	Yes	--	Yes	--	--	--	--	--	--	--
G4ST01017157696	chr1:17157697-17157738	+	GGGCCGGGAGCTGGCGGAGCTGCAGGGCCGCCTGGCGCTGGG<br>.((..((...)).)). .(((...)))..	CROCC	NM_014675	chr1:17121032-17172061	non-template	42	Exon_26	--	Yes	Yes	--	Yes	--	--	--	--	--	--
G4ST01017157701	chr1:17157702-17157746	+	GGGAGCTGGCGGAGCTGCAGGGCCGCCTGGCGCTGGGCGAGCGGG<br>.((..((...)).)). .(((...)))..	CROCC	NM_014675	chr1:17121032-17172061	non-template	45	Exon_26	--	Yes	Yes	--	Yes	--	--	--	--	--	--
G4ST01017160216	chr1:17160217-17160251	+	GGGCAGTGCCGCGCGCAGGGAAGGGGGGAGGTGGG<br>..((((...)))).	CROCC	NM_014675	chr1:17121032-17172061	non-template	35	Intron_27	--	Yes	--	Yes	--	--	--	Yes	Yes	Yes	Yes
G4ST01017160357	chr1:17160358-17160384	-	GGGAGGGGTCTCGGTGAGGGGACGGGG<br>..(((...)))	CROCC	NM_014675	chr1:17121032-17172061	template	27	Intron_27	--	Yes	--	Yes	--	--	--	--	Yes	--	--
G4ST01017165655	chr1:17165656-17165688	+	GGGCAGGAGCTGAGGGCCAGCGGGGCGACGGGG<br>(((...))).	CROCC	NM_014675	chr1:17121032-17172061	non-template	33	Intron_30	--	Yes	--	Yes	--	--	--	--	Yes	--	--
G4ST01017177704	chr1:17177705-17177738	-	GGGTGTGGGTGAGGACAGAATGGCCTGGGCTGGG<br>..(((.((...)).)))	MFAP2	NM_001135248	chr1:17173586-17179760	non-template	34	Intron_1.f	--	Yes	--	Yes	--	--	--	--	Yes	--	--
G4ST01017177704	chr1:17177705-17177738	-	GGGTGTGGGTGAGGACAGAATGGCCTGGGCTGGG<br>..(((.((...)).)))	MFAP2	NM_001135247	chr1:17173586-17180668	non-template	34	Intron_1.f	--	Yes	--	Yes	--	--	--	--	Yes	--	--
G4ST01017177704	chr1:17177705-17177738	-	GGGTGTGGGTGAGGACAGAATGGCCTGGGCTGGG<br>..(((.((...)).)))	MFAP2	NM_017459	chr1:17173586-17180668	non-template	34	Intron_1.f	--	Yes	--	Yes	--	--	--	--	Yes	--	--
G4ST01017177704	chr1:17177705-17177738	-	GGGTGTGGGTGAGGACAGAATGGCCTGGGCTGGG<br>..(((.((...)).)))	MFAP2	NM_002403	chr1:17173586-17179760	non-template	34	Intron_1.f	--	Yes	--	Yes	--	--	--	--	Yes	--	--
G4ST01017179004	chr1:17179005-17179043	+	GGGGTTTTGGGGCGTGCGGGAGGCCAGGACCTGCCTGGG<br>((((........))))	MFAP2	NM_001135248	chr1:17173586-17179760	template	39	Intron_1.f	--	Yes	--	Yes	--	--	--	Yes	--	--	--
G4ST01017179004	chr1:17179005-17179043	+	GGGGTTTTGGGGCGTGCGGGAGGCCAGGACCTGCCTGGG<br>((((........))))	MFAP2	NM_001135247	chr1:17173586-17180668	template	39	Intron_1.f	--	Yes	--	Yes	--	--	--	Yes	--	--	--
G4ST01017179004	chr1:17179005-17179043	+	GGGGTTTTGGGGCGTGCGGGAGGCCAGGACCTGCCTGGG<br>((((........))))	MFAP2	NM_017459	chr1:17173586-17180668	template	39	Intron_1.f	--	Yes	--	Yes	--	--	--	Yes	--	--	--
G4ST01017179004	chr1:17179005-17179043	+	GGGGTTTTGGGGCGTGCGGGAGGCCAGGACCTGCCTGGG<br>((((........))))	MFAP2	NM_002403	chr1:17173586-17179760	template	39	Intron_1.f	--	Yes	--	Yes	--	--	--	Yes	--	--	--
G4ST01017179435	chr1:17179436-17179465	-	GGGAAGGGCGGGGAGCCCGAGTTTGGGGGG<br>...((((....))))	MFAP2	NM_001135248	chr1:17173586-17179760	non-template	30	Intron_1.f	--	Yes	--	Yes	--	--	--	--	Yes	--	--
G4ST01017179435	chr1:17179436-17179465	-	GGGAAGGGCGGGGAGCCCGAGTTTGGGGGG<br>...((((....))))	MFAP2	NM_001135247	chr1:17173586-17180668	non-template	30	Intron_1.f	--	Yes	--	Yes	--	--	--	--	Yes	--	--
G4ST01017179435	chr1:17179436-17179465	-	GGGAAGGGCGGGGAGCCCGAGTTTGGGGGG<br>...((((....))))	MFAP2	NM_017459	chr1:17173586-17180668	non-template	30	Intron_1.f	--	Yes	--	Yes	--	--	--	--	Yes	--	--
G4ST01017179435	chr1:17179436-17179465	-	GGGAAGGGCGGGGAGCCCGAGTTTGGGGGG<br>...((((....))))	MFAP2	NM_002403	chr1:17173586-17179760	non-template	30	Intron_1.f	--	Yes	--	Yes	--	--	--	--	Yes	--	--
G4ST01017180533	chr1:17180534-17180561	-	GGGCTAAGGGCAGGGGAGGAAGCCTGGG<br>.(((...)))	MFAP2	NM_001135248	chr1:17173586-17179760	non-template	-773	upstream	Yes	--	--	--	--	--	--	--	--	--	--
G4ST01017180533	chr1:17180534-17180561	-	GGGCTAAGGGCAGGGGAGGAAGCCTGGG<br>.(((...)))	MFAP2	NM_001135247	chr1:17173586-17180668	non-template	28	Intron_1.f	--	Yes	--	Yes	--	--	--	--	--	--	--
G4ST01017180533	chr1:17180534-17180561	-	GGGCTAAGGGCAGGGGAGGAAGCCTGGG<br>.(((...)))	MFAP2	NM_017459	chr1:17173586-17180668	non-template	28	Intron_1.f	--	Yes	--	Yes	--	--	--	--	--	--	--
G4ST01017180533	chr1:17180534-17180561	-	GGGCTAAGGGCAGGGGAGGAAGCCTGGG<br>.(((...)))	MFAP2	NM_002403	chr1:17173586-17179760	non-template	-773	upstream	Yes	--	--	--	--	--	--	--	--	--	--
G4ST01017182504	chr1:17182505-17182542	-	GGGGCAAGATGACAGTCTCATCGGGAAGAGGGACAGGG<br>....(((((.....)))))	MFAP2	NM_001135247	chr1:17173586-17180668	non-template	-1836	upstream	Yes	--	--	--	--	--	--	--	--	--	--
G4ST01017182504	chr1:17182505-17182542	-	GGGGCAAGATGACAGTCTCATCGGGAAGAGGGACAGGG<br>....(((((.....)))))	MFAP2	NM_017459	chr1:17173586-17180668	non-template	-1836	upstream	Yes	--	--	--	--	--	--	--	--	--	--
G4ST01017185282	chr1:17185283-17185323	+	GGGGTGCCCGTGGGCCTGCACTACCTCAGGGGGCCGGCGGG<br>((((........))))	ATP13A2	NM_001141974	chr1:17185040-17211010	template	41	Exon_27.l	--	Yes	Yes	--	Yes	--	--	--	--	--	--
G4ST01017185282	chr1:17185283-17185323	+	GGGGTGCCCGTGGGCCTGCACTACCTCAGGGGGCCGGCGGG<br>((((........))))	ATP13A2	NM_022089	chr1:17185040-17211010	template	41	Exon_29.l	--	Yes	Yes	--	Yes	--	Yes	--	--	--	--
G4ST01017185282	chr1:17185283-17185323	+	GGGGTGCCCGTGGGCCTGCACTACCTCAGGGGGCCGGCGGG<br>((((........))))	ATP13A2	NM_001141973	chr1:17185040-17211010	template	41	Exon_29.l	--	Yes	Yes	--	Yes	--	Yes	--	--	--	--
G4ST01017185822	chr1:17185823-17185850	-	GGGAGGGAGGAGCCCTGGGGTGCTGGGG<br>(((...))).	ATP13A2	NM_001141974	chr1:17185040-17211010	non-template	28	Intron_26.l	--	Yes	--	Yes	--	--	--	--	--	--	--
G4ST01017185822	chr1:17185823-17185850	-	GGGAGGGAGGAGCCCTGGGGTGCTGGGG<br>(((...))).	ATP13A2	NM_022089	chr1:17185040-17211010	non-template	28	Intron_27	--	Yes	--	Yes	--	--	--	--	--	--	--
G4ST01017185822	chr1:17185823-17185850	-	GGGAGGGAGGAGCCCTGGGGTGCTGGGG<br>(((...))).	ATP13A2	NM_001141973	chr1:17185040-17211010	non-template	28	Intron_27	--	Yes	--	Yes	--	--	--	--	--	--	--
G4ST01017195407	chr1:17195408-17195433	+	GGGATTTGGGACCCAGGTGGGGGGGG<br>((....))	ATP13A2	NM_001141974	chr1:17185040-17211010	template	26	Intron_13	--	Yes	--	Yes	--	--	--	--	--	--	Yes
G4ST01017195407	chr1:17195408-17195433	+	GGGATTTGGGACCCAGGTGGGGGGGG<br>((....))	ATP13A2	NM_022089	chr1:17185040-17211010	template	26	Intron_13	--	Yes	--	Yes	--	--	--	--	--	--	Yes
G4ST01017195407	chr1:17195408-17195433	+	GGGATTTGGGACCCAGGTGGGGGGGG<br>((....))	ATP13A2	NM_001141973	chr1:17185040-17211010	template	26	Intron_13	--	Yes	--	Yes	--	--	--	--	--	--	Yes
G4ST01017195414	chr1:17195415-17195440	+	GGGACCCAGGTGGGGGGGGCTATGGG<br>((....))	ATP13A2	NM_001141974	chr1:17185040-17211010	template	26	Intron_13	--	Yes	--	Yes	--	--	--	--	--	--	Yes
G4ST01017195414	chr1:17195415-17195440	+	GGGACCCAGGTGGGGGGGGCTATGGG<br>((....))	ATP13A2	NM_022089	chr1:17185040-17211010	template	26	Intron_13	--	Yes	--	Yes	--	--	--	--	--	--	Yes
G4ST01017195414	chr1:17195415-17195440	+	GGGACCCAGGTGGGGGGGGCTATGGG<br>((....))	ATP13A2	NM_001141973	chr1:17185040-17211010	template	26	Intron_13	--	Yes	--	Yes	--	--	--	--	--	--	Yes
G4ST01017210893	chr1:17210894-17210930	+	GGGCGGGGGCGCGGTCCGGACGGCCCGGGGCGAGGGG<br>....(((.((....)).)))	ATP13A2	NM_001141974	chr1:17185040-17211010	template	37	Exon_1.f	--	Yes	Yes	--	--	Yes	--	--	Yes	Yes	Yes
G4ST01017210893	chr1:17210894-17210930	+	GGGCGGGGGCGCGGTCCGGACGGCCCGGGGCGAGGGG<br>....(((.((....)).)))	ATP13A2	NM_022089	chr1:17185040-17211010	template	37	Exon_1.f	--	Yes	Yes	--	--	Yes	--	--	Yes	Yes	Yes

G4ST01017210893	chr1:17210894-17210930	+	GGGCGGGGGCGCGGTCCGGACGGCCCGGGGCGAGGGG<br>....(((.((....)).)))	ATP13A2	NM_001141973	chr1:17185040-17211010	template	37	Exon_1.f	--	Yes	Yes	--	--	Yes	--	--	Yes	Yes	Yes
G4ST01017210897	chr1:17210898-17210937	+	GGGGGCGCGGTCCGGACGGCCCGGGGCGAGGGGCGCTGGG<br>....(((.((....)).)))	ATP13A2	NM_001141974	chr1:17185040-17211010	template	40	Exon_1.f	--	Yes	Yes	--	--	Yes	--	--	Yes	Yes	Yes
G4ST01017210897	chr1:17210898-17210937	+	GGGGGCGCGGTCCGGACGGCCCGGGGCGAGGGGCGCTGGG<br>....(((.((....)).)))	ATP13A2	NM_022089	chr1:17185040-17211010	template	40	Exon_1.f	--	Yes	Yes	--	--	Yes	--	--	Yes	Yes	Yes
G4ST01017210897	chr1:17210898-17210937	+	GGGGGCGCGGTCCGGACGGCCCGGGGCGAGGGGCGCTGGG<br>....(((.((....)).)))	ATP13A2	NM_001141973	chr1:17185040-17211010	template	40	Exon_1.f	--	Yes	Yes	--	--	Yes	--	--	Yes	Yes	Yes
G4ST01017211043	chr1:17211044-17211072	-	GGGCGCTGCGAGGGCCGCAGAGGGCCGGG<br>((...)).	ATP13A2	NM_001141974	chr1:17185040-17211010	non-template	-33	upstream	Yes	--	--	--	--	--	--	Yes	Yes	Yes	Yes
G4ST01017211043	chr1:17211044-17211072	-	GGGCGCTGCGAGGGCCGCAGAGGGCCGGG<br>((...)).	ATP13A2	NM_022089	chr1:17185040-17211010	non-template	-33	upstream	Yes	--	--	--	--	--	--	Yes	Yes	Yes	Yes
G4ST01017211043	chr1:17211044-17211072	-	GGGCGCTGCGAGGGCCGCAGAGGGCCGGG<br>((...)).	ATP13A2	NM_001141973	chr1:17185040-17211010	non-template	-33	upstream	Yes	--	--	--	--	--	--	Yes	Yes	Yes	Yes
G4ST01017219194	chr1:17219195-17219221	+	GGGAGGTGGCTGGGTGGAGTGGGAGGG<br>((....))	SDHB	NM_003000	chr1:17217812-17253252	template	27	Intron_7.l	--	Yes	--	Yes	--	--	--	--	--	--	--
G4ST01017284953	chr1:17284954-17284982	+	GGGTGGGCCTGAGAGGCTAGGGTCTGGGG<br>(((...)))...	PADI2	NM_007365	chr1:17265843-17318535	template	29	Intron_7	--	Yes	--	Yes	--	--	--	--	--	--	--
G4ST01017306811	chr1:17306812-17306861	+	GGGGCTATGGGCAAAGACTACTGGTCTTGGGCTCTGGCAGGAGAATTGGG<br>..(((((.....))))) ((((....))))....	PADI2	NM_007365	chr1:17265843-17318535	template	50	Intron_1.f	--	Yes	--	Yes	--	--	--	--	--	--	--
G4ST01017429292	chr1:17429293-17429335	+	GGGAGCTGGGGGCAGCTCGGGAAGCCTGCAGCCTGGCTTGGGG<br>(((((........))))).	PADI1	NM_013358	chr1:17404208-17445088	non-template	43	Intron_9	--	Yes	--	Yes	--	--	--	--	Yes	--	--
G4ST01017429299	chr1:17429300-17429341	+	GGGGGCAGCTCGGGAAGCCTGCAGCCTGGCTTGGGGAAAGGG<br>(((((........))))).	PADI1	NM_013358	chr1:17404208-17445088	non-template	42	Intron_9	--	Yes	--	Yes	--	--	--	--	Yes	--	--
G4ST01017453171	chr1:17453172-17453210	+	GGGTATACGGGGCTGCCACTTTCCAGTGGTGGGGTGGGG<br>....(((((....)))))..	PADI3	NM_016233	chr1:17448180-17483314	non-template	39	Intron_1.f	--	Yes	--	Yes	--	--	--	--	--	--	--
G4ST01017453179	chr1:17453180-17453215	+	GGGGCTGCCACTTTCCAGTGGTGGGGTGGGGCAGGG<br>....(((((....)))))..	PADI3	NM_016233	chr1:17448180-17483314	non-template	36	Intron_1.f	--	Yes	--	Yes	--	--	--	--	--	--	--
G4ST01017455502	chr1:17455503-17455530	-	GGGGAAGGGAGGGGCTGCAAGCCGAGGG<br>(((...)))...	PADI3	NM_016233	chr1:17448180-17483314	template	28	Intron_1.f	--	Yes	--	Yes	--	--	--	--	--	--	--
G4ST01017470088	chr1:17470089-17470126	-	GGGGCTCAGGATGGAGCCGGGGCCTGGGGGCCAGAGGG<br>((((......))))..	PADI3	NM_016233	chr1:17448180-17483314	template	38	Intron_8	--	Yes	--	Yes	--	--	--	--	--	--	--
G4ST01017470096	chr1:17470097-17470133	-	GGGAAGAGGGGCTCAGGATGGAGCCGGGGCCTGGGGG<br>((((......))))..	PADI3	NM_016233	chr1:17448180-17483314	template	37	Intron_8	--	Yes	--	Yes	--	--	--	--	--	--	--
G4ST01017471458	chr1:17471459-17471490	+	GGGTTTGGGGCAGGACTCTGGGGCACGATGGG<br>.(((....)))	PADI3	NM_016233	chr1:17448180-17483314	non-template	32	Intron_9	--	Yes	--	Yes	--	--	--	--	--	--	--
G4ST01017471464	chr1:17471465-17471495	+	GGGGCAGGACTCTGGGGCACGATGGGCTGGG<br>.(((....)))	PADI3	NM_016233	chr1:17448180-17483314	non-template	31	Intron_9	--	Yes	--	Yes	--	--	--	--	--	--	--
G4ST01017519592	chr1:17519593-17519625	+	GGGTAAAGGGTGGGCAATTCCCTGAACTGAGGG<br>((.(((...))).)).	PADI4	NM_012387	chr1:17507277-17563082	non-template	33	Intron_1.f	--	Yes	--	Yes	--	--	--	--	--	--	--
G4ST01017535953	chr1:17535954-17535986	-	GGGAAATGACTGCATTTCGGGAGGGATACAGGG<br>(((((....))))).	PADI4	NM_012387	chr1:17507277-17563082	template	33	Intron_4	--	Yes	--	Yes	--	--	--	--	--	--	--
G4ST01017541331	chr1:17541332-17541370	-	GGGGGTAAGGGGAAGGAGGGGCTGTCACCGCTGCCAGGG<br>((.((....)).))..	PADI4	NM_012387	chr1:17507277-17563082	template	39	Intron_7	--	Yes	--	Yes	--	--	--	--	--	--	Yes
G4ST01017545260	chr1:17545261-17545297	+	GGGCCAAGAGACACTTGGGGGACCCGGGCTCCTGGGG<br>(((((.....)))))	PADI4	NM_012387	chr1:17507277-17563082	non-template	37	Intron_9	--	Yes	--	Yes	--	--	--	--	--	--	--
G4ST01017554134	chr1:17554135-17554173	+	GGGGCACGGGAGGAATAGGAACTGTTAGGGTGACCAGGG<br>...(((((...))))).	PADI4	NM_012387	chr1:17507277-17563082	non-template	39	Intron_11	--	Yes	--	Yes	--	--	--	--	--	--	--
G4ST01017558299	chr1:17558300-17558334	-	GGGGGACTGATGGCAGTCGGGGGTGGGGGTGGGGG<br>.(((((....)))))..	PADI4	NM_012387	chr1:17507277-17563082	template	35	Intron_14	--	Yes	--	Yes	--	--	--	Yes	--	--	Yes
G4ST01017585171	chr1:17585172-17585204	+	GGGCAGCTCTGTGAGGACTGAGGGCAGGGAGGG<br>(((.(((....)))))).	PADI6	NM_207421	chr1:17571328-17600782	non-template	33	Intron_6	--	Yes	--	Yes	--	--	--	--	--	--	--
G4ST01017631775	chr1:17631776-17631817	+	GGGGAGGGAGTTTCCTGGGTGACCTGGACATTTGGTCAAGGG<br>((....)) (((((.........))))).	RCC2	NM_001136204	chr1:17605838-17637644	template	42	Intron_1.f	--	Yes	--	Yes	--	--	--	--	Yes	Yes	--
G4ST01017631775	chr1:17631776-17631817	+	GGGGAGGGAGTTTCCTGGGTGACCTGGACATTTGGTCAAGGG<br>((....)) (((((.........))))).	RCC2	NM_018715	chr1:17605838-17638807	template	42	Intron_2	--	Yes	--	Yes	--	--	--	--	Yes	Yes	--
G4ST01017636991	chr1:17636992-17637030	+	GGGAGCTGTGGGGGCGGGGCCATGACCCCCTCGTGCGGG<br>..(((((.....))))).	RCC2	NM_001136204	chr1:17605838-17637644	template	39	Intron_1.f	--	Yes	--	Yes	--	--	--	Yes	Yes	Yes	Yes
G4ST01017636991	chr1:17636992-17637030	+	GGGAGCTGTGGGGGCGGGGCCATGACCCCCTCGTGCGGG<br>..(((((.....))))).	RCC2	NM_018715	chr1:17605838-17638807	template	39	Intron_2	--	Yes	--	Yes	--	--	--	Yes	Yes	Yes	Yes
G4ST01017738612	chr1:17738613-17738645	+	GGGCTGCCGGGACCGCTGGGGGCTCGCAGCGGG<br>.(((...)))	ARHGEF10L	NM_018125	chr1:17738917-17896957	non-template	-271	upstream	Yes	--	--	--	--	--	--	--	Yes	--	Yes
G4ST01017738620	chr1:17738621-17738652	+	GGGACCGCTGGGGGCTCGCAGCGGGTCGGGGG<br>.(((...)))	ARHGEF10L	NM_018125	chr1:17738917-17896957	non-template	-264	upstream	Yes	--	--	--	--	--	--	--	Yes	--	Yes
G4ST01017739100	chr1:17739101-17739141	+	GGGAGGCGCTCCAGGCGCCAGGGTCCTCCGGGTGTCCTGGG<br>.(((((.....))))).	ARHGEF10L	NM_018125	chr1:17738917-17896957	non-template	41	Intron_1.f	--	Yes	--	Yes	--	--	--	Yes	Yes	Yes	--
G4ST01017746991	chr1:17746992-17747024	+	GGGTTCTGGGAGTAGACGCTGGGAGTAGAGGGG<br>(((....)))	ARHGEF10L	NM_018125	chr1:17738917-17896957	non-template	33	Intron_1.f	--	Yes	--	Yes	--	--	--	--	--	--	--
G4ST01017749935	chr1:17749936-17749968	+	GGGCAGGCAGCCTGGGAGCTAGGGGAAGATGGG<br>.(((...)))	ARHGEF10L	NM_018125	chr1:17738917-17896957	non-template	33	Intron_1.f	--	Yes	--	Yes	--	--	--	Yes	--	--	--
G4ST01017755186	chr1:17755187-17755220	+	GGGAGCATGGGGGGGGCCTGGAAACACAGGGGGG<br>.((((......)))).	ARHGEF10L	NM_018125	chr1:17738917-17896957	non-template	34	Intron_1.f	--	Yes	--	Yes	--	--	--	--	--	--	Yes
G4ST01017755194	chr1:17755195-17755226	+	GGGGGGGGCCTGGAAACACAGGGGGGCCTGGG<br>.((((......)))).	ARHGEF10L	NM_018125	chr1:17738917-17896957	non-template	32	Intron_1.f	--	Yes	--	Yes	--	--	--	--	--	--	Yes
G4ST01017755198	chr1:17755199-17755239	+	GGGGCCTGGAAACACAGGGGGGCCTGGGAGCATATGGGGGG<br>.((((......)))).	ARHGEF10L	NM_018125	chr1:17738917-17896957	non-template	41	Intron_1.f	--	Yes	--	Yes	--	--	--	--	--	--	Yes
G4ST01017781289	chr1:17781290-17781322	-	GGGGCGGGATGGGGCAAGGCCAGGCATGCTGGG<br>(((..((...)).))).	ARHGEF10L	NM_001011722	chr1:17779635-17896957	template	33	Intron_1.f	--	Yes	--	Yes	--	--	--	Yes	--	--	--
G4ST01017781289	chr1:17781290-17781322	-	GGGGCGGGATGGGGCAAGGCCAGGCATGCTGGG<br>(((..((...)).))).	ARHGEF10L	NM_018125	chr1:17738917-17896957	template	33	Intron_2	--	Yes	--	Yes	--	--	--	Yes	--	--	--
G4ST01017796455	chr1:17796456-17796484	+	GGGAGAGGCTGCTCCGGGTGGGTACAGGG<br>.(((....))).	ARHGEF10L	NM_001011722	chr1:17779635-17896957	non-template	29	Intron_3	--	Yes	--	Yes	--	--	--	Yes	--	--	Yes
G4ST01017796455	chr1:17796456-17796484	+	GGGAGAGGCTGCTCCGGGTGGGTACAGGG<br>.(((....))).	ARHGEF10L	NM_018125	chr1:17738917-17896957	non-template	29	Intron_4	--	Yes	--	Yes	--	--	--	Yes	--	--	Yes
G4ST01017801086	chr1:17801087-17801120	-	GGGCAAAAGTGGGCTGAGGGCCAGGCTGCTGGGG<br>.(((....)))	ARHGEF10L	NM_001011722	chr1:17779635-17896957	template	34	Intron_3	--	Yes	--	Yes	--	--	--	--	--	--	Yes
G4ST01017801086	chr1:17801087-17801120	-	GGGCAAAAGTGGGCTGAGGGCCAGGCTGCTGGGG<br>.(((....)))	ARHGEF10L	NM_018125	chr1:17738917-17896957	template	34	Intron_4	--	Yes	--	Yes	--	--	--	--	--	--	Yes
G4ST01017802378	chr1:17802379-17802406	+	GGGAGGGCGCCTTGGAGGGGGTGGGGGG<br>..(((...)))	ARHGEF10L	NM_001011722	chr1:17779635-17896957	non-template	28	Intron_4	--	Yes	--	Yes	--	--	--	Yes	Yes	--	--
G4ST01017802378	chr1:17802379-17802406	+	GGGAGGGCGCCTTGGAGGGGGTGGGGGG<br>..(((...)))	ARHGEF10L	NM_018125	chr1:17738917-17896957	non-template	28	Intron_5	--	Yes	--	Yes	--	--	--	Yes	Yes	--	--
G4ST01017802382	chr1:17802383-17802411	+	GGGCGCCTTGGAGGGGGTGGGGGGCGGGG<br>..(((...)))	ARHGEF10L	NM_001011722	chr1:17779635-17896957	non-template	29	Intron_4	--	Yes	--	Yes	--	--	--	Yes	Yes	--	--
G4ST01017802382	chr1:17802383-17802411	+	GGGCGCCTTGGAGGGGGTGGGGGGCGGGG<br>..(((...)))	ARHGEF10L	NM_018125	chr1:17738917-17896957	non-template	29	Intron_5	--	Yes	--	Yes	--	--	--	Yes	Yes	--	--
G4ST01017802438	chr1:17802439-17802470	+	GGGCAGCTGGCGGAGCTAAGGGCTGGGCTGGG<br>.((((.....))))..	ARHGEF10L	NM_001011722	chr1:17779635-17896957	non-template	32	Intron_4	--	Yes	--	Yes	--	--	--	Yes	Yes	--	--
G4ST01017802438	chr1:17802439-17802470	+	GGGCAGCTGGCGGAGCTAAGGGCTGGGCTGGG<br>.((((.....))))..	ARHGEF10L	NM_018125	chr1:17738917-17896957	non-template	32	Intron_5	--	Yes	--	Yes	--	--	--	Yes	Yes	--	--

G4ST01017809284	chr1:17809285-17809312	+	GGGACTCTGATTCAGGGGTTGGGTGGGG<br>...(((...)))	ARHGEF10L	NM_001011722	chr1:17779635-17896957	non-template	28	Intron_6	--	Yes	--	Yes	--	--	--	--	--	--	--
G4ST01017809284	chr1:17809285-17809312	+	GGGACTCTGATTCAGGGGTTGGGTGGGG<br>...(((...)))	ARHGEF10L	NM_018125	chr1:17738917-17896957	non-template	28	Intron_7	--	Yes	--	Yes	--	--	--	--	--	--	--
G4ST01017822059	chr1:17822060-17822093	-	GGGGGTTGCGGTAGTCCTGCCGGGGAGGGCGGGG<br>.....(((((....)))))	ARHGEF10L	NM_001011722	chr1:17779635-17896957	template	34	Intron_9-Exon_10	--	Yes	Yes	Yes	Yes	--	--	--	--	--	--
G4ST01017822059	chr1:17822060-17822093	-	GGGGGTTGCGGTAGTCCTGCCGGGGAGGGCGGGG<br>.....(((((....)))))	ARHGEF10L	NM_018125	chr1:17738917-17896957	template	34	Intron_11-Exon_12	--	Yes	Yes	Yes	Yes	--	--	--	--	--	--
G4ST01017825176	chr1:17825177-17825207	+	GGGCTGGGAGGTGCCTCCTGGGGTAGGAGGG<br>(((.....))).	ARHGEF10L	NM_001011722	chr1:17779635-17896957	non-template	31	Intron_12	--	Yes	--	Yes	--	--	--	--	--	--	--
G4ST01017825176	chr1:17825177-17825207	+	GGGCTGGGAGGTGCCTCCTGGGGTAGGAGGG<br>(((.....))).	ARHGEF10L	NM_018125	chr1:17738917-17896957	non-template	31	Intron_14	--	Yes	--	Yes	--	--	--	--	--	--	--
G4ST01017825381	chr1:17825382-17825418	+	GGGAGTGAAGGCACAGGGAATCCCGGGACCCGCAGGG<br>.(((....))).	ARHGEF10L	NM_001011722	chr1:17779635-17896957	non-template	37	Intron_12	--	Yes	--	Yes	--	--	--	--	--	--	--
G4ST01017825381	chr1:17825382-17825418	+	GGGAGTGAAGGCACAGGGAATCCCGGGACCCGCAGGG<br>.(((....))).	ARHGEF10L	NM_018125	chr1:17738917-17896957	non-template	37	Intron_14	--	Yes	--	Yes	--	--	--	--	--	--	--
G4ST01017825827	chr1:17825828-17825858	-	GGGGAGGGAGAATCGGGAGTTGTTGAATGGG<br>.(((....)))	ARHGEF10L	NM_001011722	chr1:17779635-17896957	template	31	Intron_12	--	Yes	--	Yes	--	--	--	--	--	--	--
G4ST01017825827	chr1:17825828-17825858	-	GGGGAGGGAGAATCGGGAGTTGTTGAATGGG<br>.(((....)))	ARHGEF10L	NM_018125	chr1:17738917-17896957	template	31	Intron_14	--	Yes	--	Yes	--	--	--	--	--	--	--
G4ST01017843489	chr1:17843490-17843524	+	GGGATGGGTCTCCTCTCCAGGAGTAGGGCCGAGGG<br>.(((((....)))))..	ARHGEF10L	NM_001011722	chr1:17779635-17896957	non-template	35	Intron_19	--	Yes	--	Yes	--	--	--	--	Yes	--	--
G4ST01017843489	chr1:17843490-17843524	+	GGGATGGGTCTCCTCTCCAGGAGTAGGGCCGAGGG<br>.(((((....)))))..	ARHGEF10L	NM_018125	chr1:17738917-17896957	non-template	35	Intron_21	--	Yes	--	Yes	--	--	--	--	Yes	--	--
G4ST01017893070	chr1:17893071-17893114	-	GGGAGAGGCTGGGAGTGCAGGGGGCAGGAGGCTTTCTGAGGGGG<br>..(((((....)))))...	ARHGEF10L	NM_001011722	chr1:17779635-17896957	template	44	Intron_25	--	Yes	--	Yes	--	--	--	--	--	--	Yes
G4ST01017893070	chr1:17893071-17893114	-	GGGAGAGGCTGGGAGTGCAGGGGGCAGGAGGCTTTCTGAGGGGG<br>..(((((....)))))...	ARHGEF10L	NM_018125	chr1:17738917-17896957	template	44	Intron_27	--	Yes	--	Yes	--	--	--	--	--	--	Yes
G4ST01017997432	chr1:17997433-17997472	+	GGGGAGGGGCCAGGAGGGCAGAAGACCCACTGCTGCTGGG<br>(((.((.....)).)))..	ACTL8	NM_030812	chr1:17954395-18026145	non-template	40	Intron_1.f	--	Yes	--	Yes	--	--	--	--	--	--	--
G4ST01017997437	chr1:17997438-17997476	+	GGGGCCAGGAGGGCAGAAGACCCACTGCTGCTGGGAGGG<br>(((.((.....)).)))..	ACTL8	NM_030812	chr1:17954395-18026145	non-template	39	Intron_1.f	--	Yes	--	Yes	--	--	--	--	--	--	--
G4ST01018310325	chr1:18310326-18310354	-	GGGGCGGGGCCACGCTGGAGGGGCATGGG<br>.(((...)))..	IGSF21	NM_032880	chr1:18306827-18577564	template	29	Intron_1.f	--	Yes	--	Yes	--	--	--	Yes	--	--	--
G4ST01018354816	chr1:18354817-18354843	+	GGGGAGCCGGGAAGGGCTGGAAGAGGG<br>((...)).	IGSF21	NM_032880	chr1:18306827-18577564	non-template	27	Intron_1.f	--	Yes	--	Yes	--	--	--	--	--	--	--
G4ST01018355250	chr1:18355251-18355284	+	GGGCATCCAGGGCTGGGGCTGGCACTTCCAGGGG<br>.((((.....))))	IGSF21	NM_032880	chr1:18306827-18577564	non-template	34	Intron_1.f	--	Yes	--	Yes	--	--	--	--	--	--	Yes
G4ST01018392171	chr1:18392172-18392200	+	GGGAAGGGGGTGAGGGCTGTGTCCAGGGG<br>(((....)))	IGSF21	NM_032880	chr1:18306827-18577564	non-template	29	Intron_1.f	--	Yes	--	Yes	--	--	--	--	--	--	--
G4ST01018407246	chr1:18407247-18407280	+	GGGAGGAAGGGAAGGGGCCCAGAGGCCTGGTGGG<br>..((((....)))).	IGSF21	NM_032880	chr1:18306827-18577564	non-template	34	Intron_1.f	--	Yes	--	Yes	--	--	--	--	--	--	Yes
G4ST01018429841	chr1:18429842-18429883	+	GGGGGCTCCAGGAGTCAAGGTCAGGGCAGGGCTTCCAGAGGG<br>(((((...)))))....... ((...)).	IGSF21	NM_032880	chr1:18306827-18577564	non-template	42	Intron_2	--	Yes	--	Yes	--	--	--	--	--	--	--
G4ST01018429864	chr1:18429865-18429889	+	GGGCAGGGCTTCCAGAGGGAATGGG<br>((...)).	IGSF21	NM_032880	chr1:18306827-18577564	non-template	25	Intron_2	--	Yes	--	Yes	--	--	--	--	--	--	--
G4ST01018429985	chr1:18429986-18430017	-	GGGGGGCAGGCTGCGGGGAGAGGGAGATGGGG<br>..(((...))).	IGSF21	NM_032880	chr1:18306827-18577564	template	32	Intron_2	--	Yes	--	Yes	--	--	--	Yes	--	--	--
G4ST01018491078	chr1:18491079-18491108	+	GGGGGTGGCGGGCCGACAGCGGGGGAAGGG<br>(((....)))	IGSF21	NM_032880	chr1:18306827-18577564	non-template	30	Intron_3	--	Yes	--	Yes	--	--	--	--	--	--	Yes
G4ST01018491087	chr1:18491088-18491115	+	GGGCCGACAGCGGGGGAAGGGCGGAGGG<br>(((....)))	IGSF21	NM_032880	chr1:18306827-18577564	non-template	28	Intron_3	--	Yes	--	Yes	--	--	--	--	--	--	Yes
G4ST01018497330	chr1:18497331-18497361	+	GGGTAGAGAGGGTGGGTTGGAAGTGCCAGGG<br>.(((.....)))	IGSF21	NM_032880	chr1:18306827-18577564	non-template	31	Intron_3	--	Yes	--	Yes	--	--	--	--	--	--	--
G4ST01018524756	chr1:18524757-18524797	-	GGGGTGGGGTCCCTTACAGGAGACCCCAGGGGGGTTCTGGG<br>..((((....((.....))))))	IGSF21	NM_032880	chr1:18306827-18577564	template	41	Intron_3	--	Yes	--	Yes	--	--	--	--	--	--	--
G4ST01018538389	chr1:18538390-18538418	-	GGGTCAAGGGGAGGGAAATTTCCTTTGGG<br>(((.....)))	IGSF21	NM_032880	chr1:18306827-18577564	template	29	Intron_4	--	Yes	--	Yes	--	--	--	Yes	Yes	--	--
G4ST01018544169	chr1:18544170-18544207	-	GGGCTTGGGAATGGGCTTGTCCAGGCTTTTGCTTGGGG<br>......(((((....)))))	IGSF21	NM_032880	chr1:18306827-18577564	template	38	Intron_4	--	Yes	--	Yes	--	--	--	--	Yes	--	Yes
G4ST01018552421	chr1:18552422-18552451	+	GGGCAGGGATTGGAGGGAAGGATGCCTGGG<br>.(((...)))	IGSF21	NM_032880	chr1:18306827-18577564	non-template	30	Intron_4	--	Yes	--	Yes	--	--	--	--	--	--	--
G4ST01018573652	chr1:18573653-18573688	-	GGGCTTGCACCAAGGAGAGGGAATCGCTGGGAAGGG<br>((((...))))....	IGSF21	NM_032880	chr1:18306827-18577564	template	36	Intron_6	--	Yes	--	Yes	--	--	--	--	--	--	Yes
G4ST01018573657	chr1:18573658-18573698	-	GGGAAGAGCAGGGCTTGCACCAAGGAGAGGGAATCGCTGGG<br>((((...))))....	IGSF21	NM_032880	chr1:18306827-18577564	template	41	Intron_6	--	Yes	--	Yes	--	--	--	--	--	--	Yes
G4ST01018679955	chr1:18679956-18679989	-	GGGTCCTGCTGAGCGGAGGGGAGGGAGTTCTGGG<br>..((((...))))..	KLHDC7A	NM_152375	chr1:18680011-18685067	template	-21	upstream	Yes	--	--	--	--	--	--	Yes	Yes	Yes	--
G4ST01018846111	chr1:18846112-18846142	+	GGGCTGGAGGGGGAGGGGCGACCCGCGCGGG<br>(((.....)))	PAX7	NM_013945	chr1:18830087-18935219	non-template	31	Intron_4	--	Yes	--	Yes	--	--	--	Yes	--	--	Yes
G4ST01018846111	chr1:18846112-18846142	+	GGGCTGGAGGGGGAGGGGCGACCCGCGCGGG<br>(((.....)))	PAX7	NM_001135254	chr1:18830087-18947947	non-template	31	Intron_4	--	Yes	--	Yes	--	--	--	Yes	--	--	Yes
G4ST01018846111	chr1:18846112-18846142	+	GGGCTGGAGGGGGAGGGGCGACCCGCGCGGG<br>(((.....)))	PAX7	NM_002584	chr1:18830087-18935219	non-template	31	Intron_4	--	Yes	--	Yes	--	--	--	Yes	--	--	Yes
G4ST01018846119	chr1:18846120-18846148	+	GGGGGAGGGGCGACCCGCGCGGGAAGGGG<br>(((.....)))	PAX7	NM_013945	chr1:18830087-18935219	non-template	29	Intron_4	--	Yes	--	Yes	--	--	--	Yes	--	--	Yes
G4ST01018846119	chr1:18846120-18846148	+	GGGGGAGGGGCGACCCGCGCGGGAAGGGG<br>(((.....)))	PAX7	NM_001135254	chr1:18830087-18947947	non-template	29	Intron_4	--	Yes	--	Yes	--	--	--	Yes	--	--	Yes
G4ST01018846119	chr1:18846120-18846148	+	GGGGGAGGGGCGACCCGCGCGGGAAGGGG<br>(((.....)))	PAX7	NM_002584	chr1:18830087-18935219	non-template	29	Intron_4	--	Yes	--	Yes	--	--	--	Yes	--	--	Yes
G4ST01018846125	chr1:18846126-18846154	+	GGGGCGACCCGCGCGGGAAGGGGCGGGGG<br>(((.....)))	PAX7	NM_013945	chr1:18830087-18935219	non-template	29	Intron_4	--	Yes	--	Yes	--	--	--	Yes	--	--	Yes
G4ST01018846125	chr1:18846126-18846154	+	GGGGCGACCCGCGCGGGAAGGGGCGGGGG<br>(((.....)))	PAX7	NM_001135254	chr1:18830087-18947947	non-template	29	Intron_4	--	Yes	--	Yes	--	--	--	Yes	--	--	Yes
G4ST01018846125	chr1:18846126-18846154	+	GGGGCGACCCGCGCGGGAAGGGGCGGGGG<br>(((.....)))	PAX7	NM_002584	chr1:18830087-18935219	non-template	29	Intron_4	--	Yes	--	Yes	--	--	--	Yes	--	--	Yes
G4ST01018848601	chr1:18848602-18848635	-	GGGGTTTATGAAGAACGGGGGAGGGGGATGGGGG<br>((((.....))))..	PAX7	NM_013945	chr1:18830087-18935219	template	34	Intron_4	--	Yes	--	Yes	--	--	--	--	--	--	--
G4ST01018848601	chr1:18848602-18848635	-	GGGGTTTATGAAGAACGGGGGAGGGGGATGGGGG<br>((((.....))))..	PAX7	NM_001135254	chr1:18830087-18947947	template	34	Intron_4	--	Yes	--	Yes	--	--	--	--	--	--	--
G4ST01018848601	chr1:18848602-18848635	-	GGGGTTTATGAAGAACGGGGGAGGGGGATGGGGG<br>((((.....))))..	PAX7	NM_002584	chr1:18830087-18935219	template	34	Intron_4	--	Yes	--	Yes	--	--	--	--	--	--	--
G4ST01018865685	chr1:18865686-18865714	+	GGGCAAAGGCTTTGGGGTGGGGGAAAGGG<br>(((.....)))	PAX7	NM_013945	chr1:18830087-18935219	non-template	29	Intron_4	--	Yes	--	Yes	--	--	--	--	--	--	Yes
G4ST01018865685	chr1:18865686-18865714	+	GGGCAAAGGCTTTGGGGTGGGGGAAAGGG<br>(((.....)))	PAX7	NM_001135254	chr1:18830087-18947947	non-template	29	Intron_4	--	Yes	--	Yes	--	--	--	--	--	--	Yes
G4ST01018865685	chr1:18865686-18865714	+	GGGCAAAGGCTTTGGGGTGGGGGAAAGGG<br>(((.....)))	PAX7	NM_002584	chr1:18830087-18935219	non-template	29	Intron_4	--	Yes	--	Yes	--	--	--	--	--	--	Yes
G4ST01018878399	chr1:18878400-18878434	+	GGGCTCCAGCACTGAGGAGGGGTGAGGGCGTGGGG<br>((((........))))	PAX7	NM_013945	chr1:18830087-18935219	non-template	35	Intron_4	--	Yes	--	Yes	--	--	--	--	--	--	Yes

G4ST01018878399	chr1:18878400-18878434	+	GGGCTCCAGCACTGAGGAGGGGTGAGGGCGTGGGG<br>((((........))))	PAX7	NM_001135254	chr1:18830087-18947947	non-template	35	Intron_4	--	Yes	--	Yes	--	--	--	--	--	--	Yes
G4ST01018878399	chr1:18878400-18878434	+	GGGCTCCAGCACTGAGGAGGGGTGAGGGCGTGGGG<br>((((........))))	PAX7	NM_002584	chr1:18830087-18935219	non-template	35	Intron_4	--	Yes	--	Yes	--	--	--	--	--	--	Yes
G4ST01018891091	chr1:18891092-18891134	+	GGGATGGCCAAGGTCAGTGGGTTTAATGGGACCCCTCGGGGGG<br>.((((....))))..	PAX7	NM_013945	chr1:18830087-18935219	non-template	43	Intron_5	--	Yes	--	Yes	--	--	--	--	--	--	--
G4ST01018891091	chr1:18891092-18891134	+	GGGATGGCCAAGGTCAGTGGGTTTAATGGGACCCCTCGGGGGG<br>.((((....))))..	PAX7	NM_001135254	chr1:18830087-18947947	non-template	43	Intron_5	--	Yes	--	Yes	--	--	--	--	--	--	--
G4ST01018891091	chr1:18891092-18891134	+	GGGATGGCCAAGGTCAGTGGGTTTAATGGGACCCCTCGGGGGG<br>.((((....))))..	PAX7	NM_002584	chr1:18830087-18935219	non-template	43	Intron_5	--	Yes	--	Yes	--	--	--	--	--	--	--
G4ST01018910999	chr1:18911000-18911029	-	GGGGCAGGGGAGCAGTCTCTGGGAGCTGGG<br>(((....))).	PAX7	NM_013945	chr1:18830087-18935219	template	30	Intron_7.l	--	Yes	--	Yes	--	--	--	--	--	--	--
G4ST01018910999	chr1:18911000-18911029	-	GGGGCAGGGGAGCAGTCTCTGGGAGCTGGG<br>(((....))).	PAX7	NM_001135254	chr1:18830087-18947947	template	30	Intron_7	--	Yes	--	Yes	--	--	--	--	--	--	--
G4ST01018910999	chr1:18911000-18911029	-	GGGGCAGGGGAGCAGTCTCTGGGAGCTGGG<br>(((....))).	PAX7	NM_002584	chr1:18830087-18935219	template	30	Intron_7.l	--	Yes	--	Yes	--	--	--	--	--	--	--
G4ST01018911006	chr1:18911007-18911033	-	GGGTGGGGCAGGGGAGCAGTCTCTGGG<br>(((....))).	PAX7	NM_013945	chr1:18830087-18935219	template	27	Intron_7.l	--	Yes	--	Yes	--	--	--	--	--	--	--
G4ST01018911006	chr1:18911007-18911033	-	GGGTGGGGCAGGGGAGCAGTCTCTGGG<br>(((....))).	PAX7	NM_001135254	chr1:18830087-18947947	template	27	Intron_7	--	Yes	--	Yes	--	--	--	--	--	--	--
G4ST01018911006	chr1:18911007-18911033	-	GGGTGGGGCAGGGGAGCAGTCTCTGGG<br>(((....))).	PAX7	NM_002584	chr1:18830087-18935219	template	27	Intron_7.l	--	Yes	--	Yes	--	--	--	--	--	--	--
G4ST01018916026	chr1:18916027-18916058	+	GGGCGGACCCAAGTCCAGGGCGGGGGTTGGGG<br>.((((....)))).	PAX7	NM_013945	chr1:18830087-18935219	non-template	32	Intron_7.l	--	Yes	--	Yes	--	--	--	--	--	--	--
G4ST01018916026	chr1:18916027-18916058	+	GGGCGGACCCAAGTCCAGGGCGGGGGTTGGGG<br>.((((....)))).	PAX7	NM_001135254	chr1:18830087-18947947	non-template	32	Intron_7	--	Yes	--	Yes	--	--	--	--	--	--	--
G4ST01018916026	chr1:18916027-18916058	+	GGGCGGACCCAAGTCCAGGGCGGGGGTTGGGG<br>.((((....)))).	PAX7	NM_002584	chr1:18830087-18935219	non-template	32	Intron_7.l	--	Yes	--	Yes	--	--	--	--	--	--	--
G4ST01018924885	chr1:18924886-18924914	-	GGGTCCAAGGGCTCAGGGCCCCTGGTGGG<br>((...)).	PAX7	NM_013945	chr1:18830087-18935219	template	29	Intron_7.l	--	Yes	--	Yes	--	--	--	--	--	--	--
G4ST01018924885	chr1:18924886-18924914	-	GGGTCCAAGGGCTCAGGGCCCCTGGTGGG<br>((...)).	PAX7	NM_001135254	chr1:18830087-18947947	template	29	Intron_7	--	Yes	--	Yes	--	--	--	--	--	--	--
G4ST01018924885	chr1:18924886-18924914	-	GGGTCCAAGGGCTCAGGGCCCCTGGTGGG<br>((...)).	PAX7	NM_002584	chr1:18830087-18935219	template	29	Intron_7.l	--	Yes	--	Yes	--	--	--	--	--	--	--
G4ST01018931997	chr1:18931998-18932022	-	GGGGGCTGGGAGACTACTGGGAGGG<br>((....))	PAX7	NM_013945	chr1:18830087-18935219	template	25	Intron_7.l	--	Yes	--	Yes	--	--	--	--	--	--	--
G4ST01018931997	chr1:18931998-18932022	-	GGGGGCTGGGAGACTACTGGGAGGG<br>((....))	PAX7	NM_001135254	chr1:18830087-18947947	template	25	Intron_7	--	Yes	--	Yes	--	--	--	--	--	--	--
G4ST01018931997	chr1:18931998-18932022	-	GGGGGCTGGGAGACTACTGGGAGGG<br>((....))	PAX7	NM_002584	chr1:18830087-18935219	template	25	Intron_7.l	--	Yes	--	Yes	--	--	--	--	--	--	--
G4ST01018936337	chr1:18936338-18936371	-	GGGCTGGGGGAGAGGACACCCTCAGGGATGGGGG<br>...((((....)))).	PAX7	NM_001135254	chr1:18830087-18947947	template	34	Intron_8.l	--	Yes	--	Yes	--	--	--	--	--	--	--
G4ST01018936344	chr1:18936345-18936376	-	GGGCTGGGCTGGGGGAGAGGACACCCTCAGGG<br>...((((....)))).	PAX7	NM_001135254	chr1:18830087-18947947	template	32	Intron_8.l	--	Yes	--	Yes	--	--	--	--	--	--	--
G4ST01019058482	chr1:19058483-19058520	-	GGGGTGGGAGGCCAGGCCTGGTCCTGTGGGTCCTTGGG<br>((((((....))).)))..	TAS1R2	NM_152232	chr1:19038680-19058742	non-template	38	Intron_1.f	--	Yes	--	Yes	--	--	--	--	--	--	--
G4ST01019058490	chr1:19058491-19058526	-	GGGGATGGGGTGGGAGGCCAGGCCTGGTCCTGTGGG<br>((((((....))).)))..	TAS1R2	NM_152232	chr1:19038680-19058742	non-template	36	Intron_1.f	--	Yes	--	Yes	--	--	--	--	--	--	--
G4ST01019070670	chr1:19070671-19070701	-	GGGTGCTGGTGCCAGCTGGGCTGGGTGTGGG<br>.((((....)))).	ALDH4A1	NM_003748	chr1:19070511-19101880	non-template	31	Exon_15.l	--	Yes	Yes	--	--	--	Yes	--	--	--	--
G4ST01019070670	chr1:19070671-19070701	-	GGGTGCTGGTGCCAGCTGGGCTGGGTGTGGG<br>.((((....)))).	ALDH4A1	NM_001161504	chr1:19070511-19089972	non-template	31	Exon_15.l	--	Yes	Yes	--	--	--	Yes	--	--	--	--
G4ST01019070670	chr1:19070671-19070701	-	GGGTGCTGGTGCCAGCTGGGCTGGGTGTGGG<br>.((((....)))).	ALDH4A1	NM_170726	chr1:19070511-19101880	non-template	31	Exon_16.l	--	Yes	Yes	--	--	--	Yes	--	--	--	--
G4ST01019070676	chr1:19070677-19070707	-	GGGGGTGGGTGCTGGTGCCAGCTGGGCTGGG<br>.((((....)))).	ALDH4A1	NM_003748	chr1:19070511-19101880	non-template	31	Exon_15.l	--	Yes	Yes	--	--	--	Yes	--	--	--	--
G4ST01019070676	chr1:19070677-19070707	-	GGGGGTGGGTGCTGGTGCCAGCTGGGCTGGG<br>.((((....)))).	ALDH4A1	NM_001161504	chr1:19070511-19089972	non-template	31	Exon_15.l	--	Yes	Yes	--	--	--	Yes	--	--	--	--
G4ST01019070676	chr1:19070677-19070707	-	GGGGGTGGGTGCTGGTGCCAGCTGGGCTGGG<br>.((((....)))).	ALDH4A1	NM_170726	chr1:19070511-19101880	non-template	31	Exon_16.l	--	Yes	Yes	--	--	--	Yes	--	--	--	--
G4ST01019075555	chr1:19075556-19075587	+	GGGCAGGGAGGAGGGAGGTCTAAGAGGCTGGG<br>..((((...)))).	ALDH4A1	NM_003748	chr1:19070511-19101880	template	32	Intron_11	--	Yes	--	Yes	--	--	--	--	--	--	Yes
G4ST01019075555	chr1:19075556-19075587	+	GGGCAGGGAGGAGGGAGGTCTAAGAGGCTGGG<br>..((((...)))).	ALDH4A1	NM_001161504	chr1:19070511-19089972	template	32	Intron_11	--	Yes	--	Yes	--	--	--	--	--	--	Yes
G4ST01019075555	chr1:19075556-19075587	+	GGGCAGGGAGGAGGGAGGTCTAAGAGGCTGGG<br>..((((...)))).	ALDH4A1	NM_170726	chr1:19070511-19101880	template	32	Intron_11	--	Yes	--	Yes	--	--	--	--	--	--	Yes
G4ST01019081094	chr1:19081095-19081131	-	GGGCCAGACGCCTTGGAGGAGTGGGGTCGGGTAGGGG<br>....((.(((...))).)).	ALDH4A1	NM_003748	chr1:19070511-19101880	non-template	37	Intron_7	--	Yes	--	Yes	--	--	--	--	--	--	--
G4ST01019081094	chr1:19081095-19081131	-	GGGCCAGACGCCTTGGAGGAGTGGGGTCGGGTAGGGG<br>....((.(((...))).)).	ALDH4A1	NM_001161504	chr1:19070511-19089972	non-template	37	Intron_7	--	Yes	--	Yes	--	--	--	--	--	--	--
G4ST01019081094	chr1:19081095-19081131	-	GGGCCAGACGCCTTGGAGGAGTGGGGTCGGGTAGGGG<br>....((.(((...))).)).	ALDH4A1	NM_170726	chr1:19070511-19101880	non-template	37	Intron_7	--	Yes	--	Yes	--	--	--	--	--	--	--
G4ST01019084714	chr1:19084715-19084752	+	GGGAGAGGGAGAGGTTGGGGACTGCCACCTGCAGCGGG<br>..((((.....)))).	ALDH4A1	NM_003748	chr1:19070511-19101880	template	38	Intron_4	--	Yes	--	Yes	--	--	--	--	--	--	--
G4ST01019084714	chr1:19084715-19084752	+	GGGAGAGGGAGAGGTTGGGGACTGCCACCTGCAGCGGG<br>..((((.....)))).	ALDH4A1	NM_001161504	chr1:19070511-19089972	template	38	Intron_4	--	Yes	--	Yes	--	--	--	--	--	--	--
G4ST01019084714	chr1:19084715-19084752	+	GGGAGAGGGAGAGGTTGGGGACTGCCACCTGCAGCGGG<br>..((((.....)))).	ALDH4A1	NM_170726	chr1:19070511-19101880	template	38	Intron_4	--	Yes	--	Yes	--	--	--	--	--	--	--
G4ST01019089055	chr1:19089056-19089087	-	GGGAGGGTGGGAGGCACCCGAGAGGTGCAGGG<br>..(((((.....))))).	ALDH4A1	NM_003748	chr1:19070511-19101880	non-template	32	Intron_2	--	Yes	--	Yes	--	--	--	--	--	--	--
G4ST01019089055	chr1:19089056-19089087	-	GGGAGGGTGGGAGGCACCCGAGAGGTGCAGGG<br>..(((((.....))))).	ALDH4A1	NM_001161504	chr1:19070511-19089972	non-template	32	Intron_2	--	Yes	--	Yes	--	--	--	--	--	--	--
G4ST01019089055	chr1:19089056-19089087	-	GGGAGGGTGGGAGGCACCCGAGAGGTGCAGGG<br>..(((((.....))))).	ALDH4A1	NM_170726	chr1:19070511-19101880	non-template	32	Intron_2	--	Yes	--	Yes	--	--	--	--	--	--	--
G4ST01019092200	chr1:19092201-19092228	-	GGGACCTGGGCTCCAAAGAGGGGCTGGG<br>(((....)))	ALDH4A1	NM_003748	chr1:19070511-19101880	non-template	28	Intron_1.f	--	Yes	--	Yes	--	--	--	--	--	--	--
G4ST01019092200	chr1:19092201-19092228	-	GGGACCTGGGCTCCAAAGAGGGGCTGGG<br>(((....)))	ALDH4A1	NM_170726	chr1:19070511-19101880	non-template	28	Intron_1.f	--	Yes	--	Yes	--	--	--	--	--	--	--
G4ST01019101491	chr1:19101492-19101530	-	GGGGCCCGGGGCGGCCCGAGCGCGCGCCTGGGGCCCGGG<br>..(((.((....)).))))..	ALDH4A1	NM_003748	chr1:19070511-19101880	non-template	39	Intron_1.f	--	Yes	--	Yes	--	--	--	Yes	Yes	Yes	--
G4ST01019101491	chr1:19101492-19101530	-	GGGGCCCGGGGCGGCCCGAGCGCGCGCCTGGGGCCCGGG<br>..(((.((....)).))))..	ALDH4A1	NM_170726	chr1:19070511-19101880	non-template	39	Intron_1.f	--	Yes	--	Yes	--	--	--	Yes	Yes	Yes	--
G4ST01019105208	chr1:19105209-19105244	-	GGGAGTGAGGACTTGGGGCAGGTGGGTAGGAGAGGG<br>(((....)))..	IFFO2	NM_001136265	chr1:19103361-19155413	non-template	36	Exon_9.l	--	Yes	Yes	--	--	--	Yes	--	--	--	--
G4ST01019117301	chr1:19117302-19117337	+	GGGTCAGGTAGTGCTACCTCTCGGGGAAGGGGAGGG<br>..((((((....))))))....	IFFO2	NM_001136265	chr1:19103361-19155413	template	36	Intron_4	--	Yes	--	Yes	--	--	--	--	--	--	Yes
G4ST01019121720	chr1:19121721-19121753	+	GGGCTCCCGGGCCCGGACCTGGAGGGCAAGGGG<br>.(((....))).	IFFO2	NM_001136265	chr1:19103361-19155413	template	33	Intron_1.f	--	Yes	--	Yes	--	--	--	Yes	Yes	--	--
G4ST01019121728	chr1:19121729-19121758	+	GGGCCCGGACCTGGAGGGCAAGGGGCTGGG<br>.(((....))).	IFFO2	NM_001136265	chr1:19103361-19155413	template	30	Intron_1.f	--	Yes	--	Yes	--	--	--	Yes	Yes	--	--

G4ST01019122314	chr1:19122315-19122348	-	GGGGAATGGGGAGTGTCGGGGAGGTGCCCCTGGG<br>.(((....)))	IFFO2	NM_001136265	chr1:19103361-19155413	non-template	34	Intron_1.f	--	Yes	--	Yes	--	--	--	Yes	Yes	--	Yes
G4ST01019130299	chr1:19130300-19130340	-	GGGAGGAGGGCACTGCCAAGCATGCTGCTTGGGATTATGGG<br>.......(((((...)))))	IFFO2	NM_001136265	chr1:19103361-19155413	non-template	41	Intron_1.f	--	Yes	--	Yes	--	--	--	--	--	--	--
G4ST01019130912	chr1:19130913-19130952	-	GGGGCATTTTCTAAGTGATGTGCTGGGGGTGTGGGCAGGG<br>((((..((.....)).))))...	IFFO2	NM_001136265	chr1:19103361-19155413	non-template	40	Intron_1.f	--	Yes	--	Yes	--	--	--	Yes	Yes	--	Yes
G4ST01019132036	chr1:19132037-19132065	-	GGGAGATATTCTGGGCTGCCGGGCCTGGG<br>(((...)))	IFFO2	NM_001136265	chr1:19103361-19155413	non-template	29	Intron_1.f	--	Yes	--	Yes	--	--	--	--	--	--	--
G4ST01019132042	chr1:19132043-19132072	-	GGGCTGTGGGAGATATTCTGGGCTGCCGGG<br>(((...)))	IFFO2	NM_001136265	chr1:19103361-19155413	non-template	30	Intron_1.f	--	Yes	--	Yes	--	--	--	--	--	--	--
G4ST01019137419	chr1:19137420-19137446	+	GGGAAGGCTGGGAGGGAGGCTACTGGG<br>((....))	IFFO2	NM_001136265	chr1:19103361-19155413	template	27	Intron_1.f	--	Yes	--	Yes	--	--	--	--	--	--	--
G4ST01019137428	chr1:19137429-19137450	+	GGGAGGGAGGCTACTGGGAGGG<br>((....))	IFFO2	NM_001136265	chr1:19103361-19155413	template	22	Intron_1.f	--	Yes	--	Yes	--	--	--	--	--	--	--
G4ST01019155300	chr1:19155301-19155332	-	GGGGGCGGCGGCGGCGGCGGGGCAGGGCCGGG<br>..((.((....)).))	IFFO2	NM_001136265	chr1:19103361-19155413	non-template	32	Exon_1.f	--	Yes	Yes	--	Yes	--	--	--	Yes	--	--
G4ST01019155305	chr1:19155306-19155347	-	GGGGGCTGCCCTGGCGGGGGCGGCGGCGGCGGCGGGGCAGGG<br>..((((....)))) ..((.((....)).))	IFFO2	NM_001136265	chr1:19103361-19155413	non-template	42	Exon_1.f	--	Yes	Yes	--	Yes	--	--	--	Yes	--	--
G4ST01019321876	chr1:19321877-19321902	-	GGGACCTGGTAGGGCAGGGAGGGGGG<br>((...)).	UBR4	NM_020765	chr1:19273587-19409333	non-template	26	Intron_66	--	Yes	--	Yes	--	--	--	Yes	--	Yes	--
G4ST01019321883	chr1:19321884-19321907	-	GGGCTGGGACCTGGTAGGGCAGGG<br>((...)).	UBR4	NM_020765	chr1:19273587-19409333	non-template	24	Intron_66	--	Yes	--	Yes	--	--	--	Yes	--	Yes	--
G4ST01019439111	chr1:19439112-19439144	-	GGGCAGAGGTTGTGAGCCTGGGATGGGTCAGGG<br>...((((.....))))	KIAA0090	NM_015047	chr1:19417171-19450633	non-template	33	Intron_7	--	Yes	--	Yes	--	--	--	--	--	--	--
G4ST01019472820	chr1:19472821-19472848	+	GGGATGGGGTGGGGGACGAAGCCGTGGG<br>..(((....)))	AKR7L	NM_001145289	chr1:19465063-19473155	template	28	Intron_1.f	--	Yes	--	Yes	--	--	--	--	--	--	--
G4ST01019472820	chr1:19472821-19472848	+	GGGATGGGGTGGGGGACGAAGCCGTGGG<br>..(((....)))	AKR7L	NM_201252	chr1:19465063-19473155	template	28	Intron_1.f	--	Yes	--	Yes	--	--	--	--	--	--	--
G4ST01019483208	chr1:19483209-19483250	+	GGGAAGGGGGGACGGTGGCACAGGTGTCAGGGCCACATTGGG<br>..((((.((...))..)))).	AKR7A3	NM_012067	chr1:19481644-19487867	template	42	Intron_5	--	Yes	--	Yes	--	--	--	--	--	--	--
G4ST01019548908	chr1:19548909-19548943	-	GGGGCGGGCAAGGGGGCTGTGGAAATACGGCCGGG<br>(((((((....)))))))	CAPZB	NM_004930	chr1:19537854-19684653	non-template	35	Intron_6	--	Yes	--	Yes	--	--	--	--	--	--	--
G4ST01019558531	chr1:19558532-19558573	-	GGGGCTGGCCGCAGCCTGCCTGAGGGAGCCCTGGGCAGGGGG<br>((.(((....))).))....	CAPZB	NM_004930	chr1:19537854-19684653	non-template	42	Intron_4	--	Yes	--	Yes	--	--	--	--	--	--	Yes
G4ST01019569327	chr1:19569328-19569370	-	GGGTGGTCTGGGGATCCCGGGCACAGCCCTGTACTGTGATGGG<br>(((((.......)))))..	CAPZB	NM_004930	chr1:19537854-19684653	non-template	43	Intron_4	--	Yes	--	Yes	--	--	--	--	--	--	--
G4ST01019650349	chr1:19650350-19650384	+	GGGTCTGGGGACTGGAGTCATGGGACAAAAAGGGG<br>(((....)))..	CAPZB	NM_004930	chr1:19537854-19684653	template	35	Intron_1.f	--	Yes	--	Yes	--	--	--	Yes	Yes	--	--
G4ST01019653406	chr1:19653407-19653450	-	GGGGTAACATAGGGGACCTGATGGGACAAACTTGCTCTTTGGGG<br>.((((.......))))	CAPZB	NM_004930	chr1:19537854-19684653	non-template	44	Intron_1.f	--	Yes	--	Yes	--	--	--	--	--	--	--
G4ST01019684155	chr1:19684156-19684194	+	GGGGGAGGAAGGGGGCCCACGCCAGGGTCACCGAGGGGG<br>(((....))).	CAPZB	NM_004930	chr1:19537854-19684653	template	39	Intron_1.f	--	Yes	--	Yes	--	--	--	Yes	Yes	Yes	--
G4ST01019684385	chr1:19684386-19684414	-	GGGGGAGGGGCGGGGTCCCGGACCCTGGG<br>(((...)))...	CAPZB	NM_004930	chr1:19537854-19684653	non-template	29	Intron_1.f	--	Yes	--	Yes	--	--	--	Yes	Yes	Yes	Yes
G4ST01019684784	chr1:19684785-19684815	+	GGGGTGCGGGGGCGGGGCCTGCGAGGCGGGG<br>((((...)))).	CAPZB	NM_004930	chr1:19537854-19684653	template	-131	upstream	Yes	--	--	--	--	--	--	Yes	Yes	Yes	Yes
G4ST01019684791	chr1:19684792-19684820	+	GGGGGCGGGGCCTGCGAGGCGGGGCGGGG<br>((((...)))).	CAPZB	NM_004930	chr1:19537854-19684653	template	-138	upstream	Yes	--	--	--	--	--	--	Yes	Yes	Yes	Yes
G4ST01019814743	chr1:19814744-19814787	-	GGGGGCGGTGGGGGGCTGAGGTGGGAGGATCGCTTGAGCCTGGG<br>(((.((....)).)))	C1orf151	NM_001204083	chr1:19796058-19828902	template	44	Intron_1.f	--	Yes	--	Yes	--	--	--	--	--	--	--
G4ST01019814743	chr1:19814744-19814787	-	GGGGGCGGTGGGGGGCTGAGGTGGGAGGATCGCTTGAGCCTGGG<br>(((.((....)).)))	C1orf151	NM_001204082	chr1:19796058-19828902	template	44	Intron_1.f	--	Yes	--	Yes	--	--	--	--	--	--	--
G4ST01019814743	chr1:19814744-19814787	-	GGGGGCGGTGGGGGGCTGAGGTGGGAGGATCGCTTGAGCCTGGG<br>(((.((....)).)))	C1orf151	NR_033757	chr1:19796058-19828902	template	44	Intron_2	--	Yes	--	Yes	--	--	--	--	--	--	--
G4ST01019814743	chr1:19814744-19814787	-	GGGGGCGGTGGGGGGCTGAGGTGGGAGGATCGCTTGAGCCTGGG<br>(((.((....)).)))	C1orf151	NR_033758	chr1:19796058-19828902	template	44	Intron_1.f	--	Yes	--	Yes	--	--	--	--	--	--	--
G4ST01019814743	chr1:19814744-19814787	-	GGGGGCGGTGGGGGGCTGAGGTGGGAGGATCGCTTGAGCCTGGG<br>(((.((....)).)))	C1orf15-NBL1	NM_001204088	chr1:19796058-19857536	template	44	Intron_2	--	Yes	--	Yes	--	--	--	--	--	--	--
G4ST01019814743	chr1:19814744-19814787	-	GGGGGCGGTGGGGGGCTGAGGTGGGAGGATCGCTTGAGCCTGGG<br>(((.((....)).)))	C1orf15-NBL1	NM_001204089	chr1:19796058-19857536	template	44	Intron_1.f	--	Yes	--	Yes	--	--	--	--	--	--	--
G4ST01019814743	chr1:19814744-19814787	-	GGGGGCGGTGGGGGGCTGAGGTGGGAGGATCGCTTGAGCCTGGG<br>(((.((....)).)))	C1orf151	NM_001032363	chr1:19796058-19828902	template	44	Intron_1.f	--	Yes	--	Yes	--	--	--	--	--	--	--
G4ST01019843681	chr1:19843682-19843727	+	GGGGGACCTGGCGGGCGCCTCCGGACGCCGGCGCTGGGGACGTGGG<br>.((..((((...)))).))..	NBL1	NM_182744	chr1:19842310-19857536	non-template	46	Intron_1.f	--	Yes	--	Yes	--	--	--	--	Yes	Yes	--
G4ST01019843681	chr1:19843682-19843727	+	GGGGGACCTGGCGGGCGCCTCCGGACGCCGGCGCTGGGGACGTGGG<br>.((..((((...)))).))..	C1orf15-NBL1	NM_001204088	chr1:19796058-19857536	non-template	46	Intron_2	--	Yes	--	Yes	--	--	--	--	Yes	Yes	--
G4ST01019843681	chr1:19843682-19843727	+	GGGGGACCTGGCGGGCGCCTCCGGACGCCGGCGCTGGGGACGTGGG<br>.((..((((...)))).))..	C1orf15-NBL1	NM_001204089	chr1:19796058-19857536	non-template	46	Intron_1.f	--	Yes	--	Yes	--	--	--	--	Yes	Yes	--
G4ST01019843681	chr1:19843682-19843727	+	GGGGGACCTGGCGGGCGCCTCCGGACGCCGGCGCTGGGGACGTGGG<br>.((..((((...)))).))..	NBL1	NM_001204085	chr1:19844453-19857536	non-template	-725	upstream	Yes	--	--	--	--	--	--	--	Yes	Yes	--
G4ST01019843681	chr1:19843682-19843727	+	GGGGGACCTGGCGGGCGCCTCCGGACGCCGGCGCTGGGGACGTGGG<br>.((..((((...)))).))..	NBL1	NM_001204084	chr1:19842795-19857536	non-template	46	Intron_1.f	--	Yes	--	Yes	--	--	--	--	Yes	Yes	--
G4ST01019843681	chr1:19843682-19843727	+	GGGGGACCTGGCGGGCGCCTCCGGACGCCGGCGCTGGGGACGTGGG<br>.((..((((...)))).))..	NBL1	NM_005380	chr1:19843394-19857536	non-template	46	Intron_1.f	--	Yes	--	Yes	--	--	--	--	Yes	Yes	--
G4ST01019843681	chr1:19843682-19843727	+	GGGGGACCTGGCGGGCGCCTCCGGACGCCGGCGCTGGGGACGTGGG<br>.((..((((...)))).))..	NBL1	NM_001204086	chr1:19844847-19857536	non-template	-1119	upstream	Yes	--	--	--	--	--	--	--	Yes	Yes	--
G4ST01019855372	chr1:19855373-19855403	+	GGGTGAGCCTCAGCTGGGCTGGGCTTTGGGG<br>..(((....)))	NBL1	NM_182744	chr1:19842310-19857536	non-template	31	Intron_3.l	--	Yes	--	Yes	--	--	--	--	Yes	--	Yes
G4ST01019855372	chr1:19855373-19855403	+	GGGTGAGCCTCAGCTGGGCTGGGCTTTGGGG<br>..(((....)))	C1orf15-NBL1	NM_001204088	chr1:19796058-19857536	non-template	31	Intron_4.l	--	Yes	--	Yes	--	--	--	--	Yes	--	Yes
G4ST01019855372	chr1:19855373-19855403	+	GGGTGAGCCTCAGCTGGGCTGGGCTTTGGGG<br>..(((....)))	C1orf15-NBL1	NM_001204089	chr1:19796058-19857536	non-template	31	Intron_3.l	--	Yes	--	Yes	--	--	--	--	Yes	--	Yes
G4ST01019855372	chr1:19855373-19855403	+	GGGTGAGCCTCAGCTGGGCTGGGCTTTGGGG<br>..(((....)))	NBL1	NM_001204085	chr1:19844453-19857536	non-template	31	Intron_3.l	--	Yes	--	Yes	--	--	--	--	Yes	--	Yes
G4ST01019855372	chr1:19855373-19855403	+	GGGTGAGCCTCAGCTGGGCTGGGCTTTGGGG<br>..(((....)))	NBL1	NM_001204084	chr1:19842795-19857536	non-template	31	Intron_3.l	--	Yes	--	Yes	--	--	--	--	Yes	--	Yes
G4ST01019855372	chr1:19855373-19855403	+	GGGTGAGCCTCAGCTGGGCTGGGCTTTGGGG<br>..(((....)))	NBL1	NM_005380	chr1:19843394-19857536	non-template	31	Intron_3.l	--	Yes	--	Yes	--	--	--	--	Yes	--	Yes
G4ST01019855372	chr1:19855373-19855403	+	GGGTGAGCCTCAGCTGGGCTGGGCTTTGGGG<br>..(((....)))	NBL1	NM_001204086	chr1:19844847-19857536	non-template	31	Intron_3.l	--	Yes	--	Yes	--	--	--	--	Yes	--	Yes
G4ST01019863764	chr1:19863765-19863801	+	GGGGCATCTGAGGGTGTGGGGGCTGCCACAGCCCGGG<br>(((((...))))).	HTR6	NM_000871	chr1:19864367-19878642	non-template	-565	upstream	Yes	--	--	--	--	--	--	Yes	--	Yes	Yes
G4ST01019989025	chr1:19989026-19989063	+	GGGAGGGTGAGGTGGGAGAATCACTTGAGTCTTGGGGG<br>(((.((....)).)))...	TMCO4	NM_181719	chr1:19881293-19998997	template	38	Intron_2	--	Yes	--	Yes	--	--	--	--	--	--	--
G4ST01020081821	chr1:20081822-20081852	+	GGGCCGGGAGCGAGGGAGGCGGCGCCGGGGG<br>.(((...)))..	OTUD3	NM_015207	chr1:20081475-20112024	non-template	31	Intron_1.f	--	Yes	--	Yes	--	--	--	Yes	Yes	Yes	Yes
G4ST01020081826	chr1:20081827-20081862	+	GGGAGCGAGGGAGGCGGCGCCGGGGGACGCGCCGGG<br>.(((...)))..	OTUD3	NM_015207	chr1:20081475-20112024	non-template	36	Intron_1.f	--	Yes	--	Yes	--	--	--	Yes	Yes	Yes	Yes
G4ST01020121509	chr1:20121510-20121553	+	GGGACCTGTGAGCCAGGCTGGGCTGGACCAGGGGCTGCTCGGGG<br>.((((.....)))).. (((...)))	PLA2G2E	NM_014589	chr1:20119387-20122697	template	44	Intron_2	--	Yes	--	Yes	--	--	--	--	--	--	--

G4ST01020121605	chr1:20121606-20121640	-	GGGAGGATCCTGGGAGAAGGAGGGAAGCCTGGGGG<br>((....))	PLA2G2E	NM_014589	chr1:20119387-20122697	non-template	35	Intron_2	--	Yes	--	Yes	--	--	--	--	--	--	--
G4ST01020121616	chr1:20121617-20121650	-	GGGGGATCCTGGGAGGATCCTGGGAGAAGGAGGG<br>((....))	PLA2G2E	NM_014589	chr1:20119387-20122697	non-template	34	Intron_2	--	Yes	--	Yes	--	--	--	--	--	--	--
G4ST01020273676	chr1:20273677-20273701	-	GGGAGGTGGGCACTGAGTGGGTGGG<br>.((...))	PLA2G5	NM_000929	chr1:20269288-20290981	template	25	Intron_1.f	--	Yes	--	Yes	--	--	--	--	--	--	--
G4ST01020283830	chr1:20283831-20283857	+	GGGTGGGATCTGAATGGGCCACGGGGG<br>((....))	PLA2G5	NM_000929	chr1:20269288-20290981	non-template	27	Intron_1.f	--	Yes	--	Yes	--	--	--	--	--	--	--
G4ST01020337188	chr1:20337189-20337224	-	GGGGAGAATTGAGATACTGGGAGGGGATGGTAAGGG<br>.((.((....)).))	PLA2G2F	NM_022819	chr1:20338410-20349466	template	-1185	upstream	Yes	--	--	--	--	--	--	--	--	--	--
G4ST01020345774	chr1:20345775-20345822	+	GGGAAGGAGTCTAGTGGAGACTGGGGTGCACTTGGGACCCCTTGGGGG<br>....(((((.....))))).	PLA2G2F	NM_022819	chr1:20338410-20349466	non-template	48	Intron_4.l	--	Yes	--	Yes	--	--	--	--	--	--	--
G4ST01020369955	chr1:20369956-20369988	-	GGGCAGTGGTGGGAGGTGCCAGCCAGGGCTGGG<br>.(((....))).	PLA2G2C	NM_001105572	chr1:20363071-20374274	non-template	33	Intron_2.l	--	Yes	--	Yes	--	--	--	--	--	--	--
G4ST01020386554	chr1:20386555-20386589	-	GGGCGGTGGGGGAAACAGGTGTTTGGGGATCTGGG<br>..((((....)))).	UBXN10	NM_152376	chr1:20385165-20392529	template	35	Intron_1.u	--	Yes	--	Yes	--	--	--	--	Yes	Yes	--
G4ST01020386561	chr1:20386562-20386594	-	GGGATGGGCGGTGGGGGAAACAGGTGTTTGGGG<br>..((((....)))).	UBXN10	NM_152376	chr1:20385165-20392529	template	33	Intron_1.u	--	Yes	--	Yes	--	--	--	--	Yes	Yes	--
G4ST01020494174	chr1:20494175-20494206	+	GGGGTGGGGAAAGGCTTCAGGGAGGAGGTGGG<br>(((....))).	VWA5B1	NM_001039500	chr1:20489999-20553974	non-template	32	Intron_1.f	--	Yes	--	Yes	--	--	--	--	--	--	--
G4ST01020494179	chr1:20494180-20494215	+	GGGGAAAGGCTTCAGGGAGGAGGTGGGTTTGAAGGG<br>(((....))).	VWA5B1	NM_001039500	chr1:20489999-20553974	non-template	36	Intron_1.f	--	Yes	--	Yes	--	--	--	--	--	--	--
G4ST01020508729	chr1:20508730-20508764	+	GGGAACCCATTGGTGGGTAGAAGAGGGTAGTGGGG<br>.(((....)))	VWA5B1	NM_001039500	chr1:20489999-20553974	non-template	35	Intron_1.f	--	Yes	--	Yes	--	--	--	--	--	--	--
G4ST01020529121	chr1:20529122-20529148	+	GGGCAGGGATGAAATTGGGAGCATGGG<br>((...)).	VWA5B1	NM_001039500	chr1:20489999-20553974	non-template	27	Intron_9	--	Yes	--	Yes	--	--	--	Yes	Yes	--	--
G4ST01020551748	chr1:20551749-20551791	+	GGGAGACTGGCCCTGCCACATCTGGGGCCCCTCAGGGCTTGGG<br>(((.((((...))))..))).	VWA5B1	NM_001039500	chr1:20489999-20553974	non-template	43	Intron_21.l	--	Yes	--	Yes	--	--	--	--	--	--	--
G4ST01020552951	chr1:20552952-20552985	-	GGGGTCTTGGACTCAGACGGGCGGGTGGTGAGGG<br>((((.......))))	VWA5B1	NM_001039500	chr1:20489999-20553974	template	34	Exon_22.l	--	Yes	Yes	--	Yes	--	--	--	--	--	--
G4ST01020552960	chr1:20552961-20552993	-	GGGGACTCGGGGTCTTGGACTCAGACGGGCGGG<br>((((.......))))	VWA5B1	NM_001039500	chr1:20489999-20553974	template	33	Exon_22.l	--	Yes	Yes	--	Yes	--	--	--	--	--	--
G4ST01020552964	chr1:20552965-20553012	-	GGGGAGCTGGTGCACAGCTGGGGACTCGGGGTCTTGGACTCAGACGGG<br>.(((((.....))))). ((((.......))))	VWA5B1	NM_001039500	chr1:20489999-20553974	template	48	Exon_22.l	--	Yes	Yes	--	Yes	--	--	--	--	--	--
G4ST01020565118	chr1:20565119-20565156	-	GGGTGCTCGTTGGAGGAAGGAGGGGTGAGGGGAGGGGG<br>..(((.((.....)).)))	LOC339505	NR_033887	chr1:20558881-20627874	non-template	38	Intron_10	--	Yes	--	Yes	--	--	--	Yes	--	--	--
G4ST01020565124	chr1:20565125-20565161	-	GGGGAGGGTGCTCGTTGGAGGAAGGAGGGGTGAGGGG<br>..(((.((.....)).)))	LOC339505	NR_033887	chr1:20558881-20627874	non-template	37	Intron_10	--	Yes	--	Yes	--	--	--	Yes	--	--	--
G4ST01020565131	chr1:20565132-20565167	-	GGGGTTGGGGAGGGTGCTCGTTGGAGGAAGGAGGGG<br>..(((.((.....)).)))	LOC339505	NR_033887	chr1:20558881-20627874	non-template	36	Intron_10	--	Yes	--	Yes	--	--	--	Yes	--	--	--
G4ST01020565758	chr1:20565759-20565800	-	GGGCGGGGGACCCAGCGGGGTCGCGGCGCGGCGTCCGCGGGG<br>..(((((((...)).)))))	LOC339505	NR_033887	chr1:20558881-20627874	non-template	42	Intron_10	--	Yes	--	Yes	--	--	--	--	--	--	Yes
G4ST01020569206	chr1:20569207-20569244	-	GGGGGCCCTTGGCCAGCATGGGCCTGGGGCTGCTTGGG<br>((((...)))).... .	LOC339505	NR_033887	chr1:20558881-20627874	non-template	38	Intron_10	--	Yes	--	Yes	--	--	--	--	--	--	Yes
G4ST01020578723	chr1:20578724-20578754	+	GGGAACTGGGGAAGCCAAAGCTGGGTGAGGG<br>..(((....)))	LOC339505	NR_033887	chr1:20558881-20627874	template	31	Intron_6	--	Yes	--	Yes	--	--	--	--	--	--	--
G4ST01020601043	chr1:20601044-20601076	+	GGGCAGGGCACAGAGGGAGATCACCATCTTGGG<br>((((....)))).	LOC339505	NR_033887	chr1:20558881-20627874	template	33	Intron_3	--	Yes	--	Yes	--	--	--	--	--	--	--
G4ST01020684521	chr1:20684522-20684559	+	GGGTCAGCGGCGCTGGGGCCGGGGGCGGCCGGCCGGGG<br>.((((...)))) ..(((....)))	CAMK2N1	NM_018584	chr1:20681471-20685315	template	38	Exon_1.f	--	Yes	Yes	--	--	Yes	--	--	Yes	Yes	--
G4ST01020684909	chr1:20684910-20684948	+	GGGAGCTGCGAGCGGCGGGGCTGCGAGGGGCGGAGGGGG<br>.((((....)))).	CAMK2N1	NM_018584	chr1:20681471-20685315	template	39	Exon_1.f	--	Yes	Yes	--	--	Yes	--	Yes	Yes	Yes	Yes
G4ST01020685861	chr1:20685862-20685890	+	GGGCCTCCAGGAGGGGGAGGGGGAGAGGG<br>((((...))))	CAMK2N1	NM_018584	chr1:20681471-20685315	template	-546	upstream	Yes	--	--	--	--	--	--	Yes	Yes	Yes	Yes
G4ST01020707266	chr1:20707267-20707302	+	GGGGCAAGGGGTTGTAGAGGGAACCGGAAGGTGGGG<br>.(((....))).	MUL1	NM_024544	chr1:20698528-20707261	template	-5	upstream	Yes	--	--	--	--	--	--	Yes	Yes	Yes	--
G4ST01020707273	chr1:20707274-20707311	+	GGGGTTGTAGAGGGAACCGGAAGGTGGGGTTGCCCGGG<br>.(((....))).	MUL1	NM_024544	chr1:20698528-20707261	template	-12	upstream	Yes	--	--	--	--	--	--	Yes	Yes	Yes	--
G4ST01020753508	chr1:20753509-20753538	+	GGGGAGAATTGGGGCCGGGCCGGCGGTGGG<br>((...)).	FAM43B	NM_207334	chr1:20751519-20754100	non-template	30	Exon_1.u	--	Yes	Yes	--	--	--	Yes	Yes	--	--	--
G4ST01020786531	chr1:20786532-20786558	+	GGGCTGGATGCAGTGGGAGGGGAGGGG<br>(((....))).	CDA	NM_001785	chr1:20788031-20817987	non-template	-1472	upstream	Yes	--	--	--	--	--	--	Yes	Yes	--	Yes
G4ST01020799397	chr1:20799398-20799427	+	GGGCGGGGCTGGGAGAGTCACTAGGCTGGG<br>..((((....))))	CDA	NM_001785	chr1:20788031-20817987	non-template	30	Intron_1.f	--	Yes	--	Yes	--	--	--	Yes	--	--	--
G4ST01020883584	chr1:20883585-20883621	+	GGGCACTGGAGGCCAGGGGGCTGGGGGCTTGGCTGGG<br>..((((...)))).	KIF17	NM_001122819	chr1:20863094-20916904	template	37	Intron_10	--	Yes	--	Yes	--	--	--	--	--	--	Yes
G4ST01020883584	chr1:20883585-20883621	+	GGGCACTGGAGGCCAGGGGGCTGGGGGCTTGGCTGGG<br>..((((...)))).	KIF17	NM_020816	chr1:20863094-20916904	template	37	Intron_10	--	Yes	--	Yes	--	--	--	--	--	--	Yes
G4ST01020887728	chr1:20887729-20887767	-	GGGCTGGCCACTCCAGTGAGGGCTCCGAGGGCACCCGGG<br>((((.....))))...	KIF17	NM_001122819	chr1:20863094-20916904	non-template	39	Intron_7	--	Yes	--	Yes	--	--	--	--	--	--	--
G4ST01020887728	chr1:20887729-20887767	-	GGGCTGGCCACTCCAGTGAGGGCTCCGAGGGCACCCGGG<br>((((.....))))...	KIF17	NM_020816	chr1:20863094-20916904	non-template	39	Intron_7	--	Yes	--	Yes	--	--	--	--	--	--	--
G4ST01020896211	chr1:20896212-20896244	-	GGGCTGTGGGTACCTGGGGCCCAGCCTCTGGGG<br>...(((...)))	KIF17	NM_001122819	chr1:20863094-20916904	non-template	33	Intron_6	--	Yes	--	Yes	--	--	--	--	Yes	--	--
G4ST01020896211	chr1:20896212-20896244	-	GGGCTGTGGGTACCTGGGGCCCAGCCTCTGGGG<br>...(((...)))	KIF17	NM_020816	chr1:20863094-20916904	non-template	33	Intron_6	--	Yes	--	Yes	--	--	--	--	Yes	--	--
G4ST01020916882	chr1:20916883-20916912	+	GGGGGGCGGGGCCTTGAGGCAGGGGCGGGG<br>(((....)))..	KIF17	NM_001122819	chr1:20863094-20916904	template	22	upstream-Exon_1.f	Yes	Yes	Yes	--	--	Yes	--	Yes	Yes	Yes	Yes
G4ST01020916882	chr1:20916883-20916912	+	GGGGGGCGGGGCCTTGAGGCAGGGGCGGGG<br>(((....)))..	KIF17	NM_020816	chr1:20863094-20916904	template	22	upstream-Exon_1.f	Yes	Yes	Yes	--	--	Yes	--	Yes	Yes	Yes	Yes
G4ST01020916889	chr1:20916890-20916925	+	GGGGCCTTGAGGCAGGGGCGGGGCCGCGGCGGGGGG<br>(((....)))..	KIF17	NM_001122819	chr1:20863094-20916904	template	15	upstream-Exon_1.f	Yes	Yes	Yes	--	--	Yes	--	Yes	Yes	Yes	Yes
G4ST01020916889	chr1:20916890-20916925	+	GGGGCCTTGAGGCAGGGGCGGGGCCGCGGCGGGGGG<br>(((....)))..	KIF17	NM_020816	chr1:20863094-20916904	template	15	upstream-Exon_1.f	Yes	Yes	Yes	--	--	Yes	--	Yes	Yes	Yes	Yes
G4ST01020917078	chr1:20917079-20917110	-	GGGCAGGCGGCTGGGGCGGCTCCGGGCTCGGG<br>(((....)))	KIF17	NM_001122819	chr1:20863094-20916904	non-template	-174	upstream	Yes	--	--	--	--	--	--	Yes	Yes	Yes	--
G4ST01020917078	chr1:20917079-20917110	-	GGGCAGGCGGCTGGGGCGGCTCCGGGCTCGGG<br>(((....)))	KIF17	NM_020816	chr1:20863094-20916904	non-template	-174	upstream	Yes	--	--	--	--	--	--	Yes	Yes	Yes	--
G4ST01020917171	chr1:20917172-20917208	-	GGGCGAAGCTCGCGGGCCAGGCGGGGCGTTCCCGGGG<br>(((...))).	KIF17	NM_001122819	chr1:20863094-20916904	non-template	-267	upstream	Yes	--	--	--	--	--	--	Yes	Yes	Yes	--
G4ST01020917171	chr1:20917172-20917208	-	GGGCGAAGCTCGCGGGCCAGGCGGGGCGTTCCCGGGG<br>(((...))).	KIF17	NM_020816	chr1:20863094-20916904	non-template	-267	upstream	Yes	--	--	--	--	--	--	Yes	Yes	Yes	--
G4ST01020917182	chr1:20917183-20917219	-	GGGGATCACTCGGGCGAAGCTCGCGGGCCAGGCGGGG<br>(((...))).	KIF17	NM_001122819	chr1:20863094-20916904	non-template	-278	upstream	Yes	--	--	--	--	--	--	Yes	Yes	Yes	--
G4ST01020917182	chr1:20917183-20917219	-	GGGGATCACTCGGGCGAAGCTCGCGGGCCAGGCGGGG<br>(((...))).	KIF17	NM_020816	chr1:20863094-20916904	non-template	-278	upstream	Yes	--	--	--	--	--	--	Yes	Yes	Yes	--
G4ST01020922854	chr1:20922855-20922890	-	GGGTGATGAGGCCTGCGGCCGCAGGGAATGGGAGGG<br>...((.((((...)))).))	SH2D5	NM_001103160	chr1:20918812-20931720	non-template	36	Intron_6	--	Yes	--	Yes	--	--	--	--	--	--	--
G4ST01020922854	chr1:20922855-20922890	-	GGGTGATGAGGCCTGCGGCCGCAGGGAATGGGAGGG<br>...((.((((...)))).))	SH2D5	NM_001103161	chr1:20918812-20931720	non-template	36	Intron_7	--	Yes	--	Yes	--	--	--	--	--	--	--

G4ST01020925228	chr1:20925229-20925252	-	GGGCGGGCCTGGAAGGAGGGAGGG<br>(((...))).	SH2D5	NM_001103160	chr1:20918812-20931720	non-template	24	Intron_3	--	Yes	--	Yes	--	--	--	Yes	--	--	--
G4ST01020925228	chr1:20925229-20925252	-	GGGCGGGCCTGGAAGGAGGGAGGG<br>(((...))).	SH2D5	NM_001103161	chr1:20918812-20931720	non-template	24	Intron_4	--	Yes	--	Yes	--	--	--	Yes	--	--	--
G4ST01020930805	chr1:20930806-20930842	+	GGGGCGGGCGCGGGGGTGCGAAGCGCTCCAGAGTGGG<br>((.(((...))).)).....	SH2D5	NM_001103160	chr1:20918812-20931720	template	37	Intron_1.f	--	Yes	--	Yes	--	--	--	Yes	--	--	--
G4ST01020930805	chr1:20930806-20930842	+	GGGGCGGGCGCGGGGGTGCGAAGCGCTCCAGAGTGGG<br>((.(((...))).)).....	SH2D5	NM_001103161	chr1:20918812-20931720	template	37	Intron_1.f	--	Yes	--	Yes	--	--	--	Yes	--	--	--
G4ST01020931100	chr1:20931101-20931135	-	GGGAGACGCGGGGGTCGCGGGCCGGAGCGGCCGGG<br>(((...)))...	SH2D5	NM_001103160	chr1:20918812-20931720	non-template	35	Intron_1.f	--	Yes	--	Yes	--	--	--	--	Yes	--	--
G4ST01020931100	chr1:20931101-20931135	-	GGGAGACGCGGGGGTCGCGGGCCGGAGCGGCCGGG<br>(((...)))...	SH2D5	NM_001103161	chr1:20918812-20931720	non-template	35	Intron_1.f	--	Yes	--	Yes	--	--	--	--	Yes	--	--
G4ST01020931114	chr1:20931115-20931149	-	GGGGCTGCGGCGGCGGGAGACGCGGGGGTCGCGGG<br>(((.....)))	SH2D5	NM_001103160	chr1:20918812-20931720	non-template	35	Intron_1.f	--	Yes	--	Yes	--	--	--	--	Yes	--	--
G4ST01020931114	chr1:20931115-20931149	-	GGGGCTGCGGCGGCGGGAGACGCGGGGGTCGCGGG<br>(((.....)))	SH2D5	NM_001103161	chr1:20918812-20931720	non-template	35	Intron_1.f	--	Yes	--	Yes	--	--	--	--	Yes	--	--
G4ST01020932121	chr1:20932122-20932157	+	GGGCCTGAGGGCCCCTGGAGTTCCAGGGGAGGCGGG<br>...((((....))))	SH2D5	NM_001103160	chr1:20918812-20931720	template	-401	upstream	Yes	--	--	--	--	--	--	Yes	Yes	--	--
G4ST01020932121	chr1:20932122-20932157	+	GGGCCTGAGGGCCCCTGGAGTTCCAGGGGAGGCGGG<br>...((((....))))	SH2D5	NM_001103161	chr1:20918812-20931720	template	-401	upstream	Yes	--	--	--	--	--	--	Yes	Yes	--	--
G4ST01020986458	chr1:20986459-20986499	+	GGGGGCGCGCGGGGACGGGCGGACGCCGGCGGAGCGCGGGG<br>....(((..((...)))))	HP1BP3	NM_016287	chr1:20941758-20985768	template	-690	upstream	Yes	--	--	--	--	--	--	Yes	Yes	Yes	Yes
G4ST01021015403	chr1:21015404-21015439	+	GGGGGGGGGGGGGGTCGGCCAGCCGCCCCGTCCGGG<br>(((.(((....))))))....	EIF4G3	NM_003760	chr1:21005372-21310463	template	36	Intron_29	--	Yes	--	Yes	--	--	--	--	--	--	Yes
G4ST01021015403	chr1:21015404-21015439	+	GGGGGGGGGGGGGGTCGGCCAGCCGCCCCGTCCGGG<br>(((.(((....))))))....	EIF4G3	NM_001198801	chr1:21005372-21375968	template	36	Intron_32	--	Yes	--	Yes	--	--	--	--	--	--	Yes
G4ST01021015403	chr1:21015404-21015439	+	GGGGGGGGGGGGGGTCGGCCAGCCGCCCCGTCCGGG<br>(((.(((....))))))....	EIF4G3	NM_001198802	chr1:21005372-21375968	template	36	Intron_32	--	Yes	--	Yes	--	--	--	--	--	--	Yes
G4ST01021015407	chr1:21015408-21015443	+	GGGGGGGGGGTCGGCCAGCCGCCCCGTCCGGGAGGG<br>(((.(((....))))))....	EIF4G3	NM_003760	chr1:21005372-21310463	template	36	Intron_29	--	Yes	--	Yes	--	--	--	--	--	--	Yes
G4ST01021015407	chr1:21015408-21015443	+	GGGGGGGGGGTCGGCCAGCCGCCCCGTCCGGGAGGG<br>(((.(((....))))))....	EIF4G3	NM_001198801	chr1:21005372-21375968	template	36	Intron_32	--	Yes	--	Yes	--	--	--	--	--	--	Yes
G4ST01021015407	chr1:21015408-21015443	+	GGGGGGGGGGTCGGCCAGCCGCCCCGTCCGGGAGGG<br>(((.(((....))))))....	EIF4G3	NM_001198802	chr1:21005372-21375968	template	36	Intron_32	--	Yes	--	Yes	--	--	--	--	--	--	Yes
G4ST01021015411	chr1:21015412-21015450	+	GGGGGGTCGGCCAGCCGCCCCGTCCGGGAGGGAGGTGGG<br>(((.(((....))))))....	EIF4G3	NM_003760	chr1:21005372-21310463	template	39	Intron_29	--	Yes	--	Yes	--	--	--	--	--	--	Yes
G4ST01021015411	chr1:21015412-21015450	+	GGGGGGTCGGCCAGCCGCCCCGTCCGGGAGGGAGGTGGG<br>(((.(((....))))))....	EIF4G3	NM_001198801	chr1:21005372-21375968	template	39	Intron_32	--	Yes	--	Yes	--	--	--	--	--	--	Yes
G4ST01021015411	chr1:21015412-21015450	+	GGGGGGTCGGCCAGCCGCCCCGTCCGGGAGGGAGGTGGG<br>(((.(((....))))))....	EIF4G3	NM_001198802	chr1:21005372-21375968	template	39	Intron_32	--	Yes	--	Yes	--	--	--	--	--	--	Yes
G4ST01021435211	chr1:21435212-21435242	-	GGGATGGGAGTCAGGGAAGACTTCCCTTGGG<br>(((......)))	ECE1	NM_001397	chr1:21416327-21489569	non-template	31	Intron_12	--	Yes	--	Yes	--	--	--	--	--	--	--
G4ST01021435211	chr1:21435212-21435242	-	GGGATGGGAGTCAGGGAAGACTTCCCTTGGG<br>(((......)))	ECE1	NM_001113349	chr1:21416327-21489353	non-template	31	Intron_11	--	Yes	--	Yes	--	--	--	--	--	--	--
G4ST01021435211	chr1:21435212-21435242	-	GGGATGGGAGTCAGGGAAGACTTCCCTTGGG<br>(((......)))	ECE1	NM_001113347	chr1:21416327-21478770	non-template	31	Intron_10	--	Yes	--	Yes	--	--	--	--	--	--	--
G4ST01021435211	chr1:21435212-21435242	-	GGGATGGGAGTCAGGGAAGACTTCCCTTGGG<br>(((......)))	ECE1	NM_001113348	chr1:21416327-21544621	non-template	31	Intron_12	--	Yes	--	Yes	--	--	--	--	--	--	--
G4ST01021441736	chr1:21441737-21441776	-	GGGGCACAGGGAGGCCTCAAGAGAGGCAAGGGCAAGTGGG<br>..(((((....)))))..	ECE1	NM_001397	chr1:21416327-21489569	non-template	40	Intron_10	--	Yes	--	Yes	--	--	--	--	--	--	--
G4ST01021441736	chr1:21441737-21441776	-	GGGGCACAGGGAGGCCTCAAGAGAGGCAAGGGCAAGTGGG<br>..(((((....)))))..	ECE1	NM_001113349	chr1:21416327-21489353	non-template	40	Intron_9	--	Yes	--	Yes	--	--	--	--	--	--	--
G4ST01021441736	chr1:21441737-21441776	-	GGGGCACAGGGAGGCCTCAAGAGAGGCAAGGGCAAGTGGG<br>..(((((....)))))..	ECE1	NM_001113347	chr1:21416327-21478770	non-template	40	Intron_8	--	Yes	--	Yes	--	--	--	--	--	--	--
G4ST01021441736	chr1:21441737-21441776	-	GGGGCACAGGGAGGCCTCAAGAGAGGCAAGGGCAAGTGGG<br>..(((((....)))))..	ECE1	NM_001113348	chr1:21416327-21544621	non-template	40	Intron_10	--	Yes	--	Yes	--	--	--	--	--	--	--
G4ST01021478172	chr1:21478173-21478209	-	GGGCGAGGGGGGCTTCCCTGCTTAAGCCGGGGCAGGG<br>.(((((........))))).	ECE1	NM_001397	chr1:21416327-21489569	non-template	37	Intron_3	--	Yes	--	Yes	--	--	--	--	--	Yes	--
G4ST01021478172	chr1:21478173-21478209	-	GGGCGAGGGGGGCTTCCCTGCTTAAGCCGGGGCAGGG<br>.(((((........))))).	ECE1	NM_001113349	chr1:21416327-21489353	non-template	37	Intron_2	--	Yes	--	Yes	--	--	--	--	--	Yes	--
G4ST01021478172	chr1:21478173-21478209	-	GGGCGAGGGGGGCTTCCCTGCTTAAGCCGGGGCAGGG<br>.(((((........))))).	ECE1	NM_001113347	chr1:21416327-21478770	non-template	37	Intron_1.f	--	Yes	--	Yes	--	--	--	--	--	Yes	--
G4ST01021478172	chr1:21478173-21478209	-	GGGCGAGGGGGGCTTCCCTGCTTAAGCCGGGGCAGGG<br>.(((((........))))).	ECE1	NM_001113348	chr1:21416327-21544621	non-template	37	Intron_3	--	Yes	--	Yes	--	--	--	--	--	Yes	--
G4ST01021483910	chr1:21483911-21483940	+	GGGAGTGGTCTGGGCAAGGGTCTGGCAGGG<br>((....))	ECE1	NM_001397	chr1:21416327-21489569	template	30	Intron_2	--	Yes	--	Yes	--	--	--	--	--	--	--
G4ST01021483910	chr1:21483911-21483940	+	GGGAGTGGTCTGGGCAAGGGTCTGGCAGGG<br>((....))	ECE1	NM_001113349	chr1:21416327-21489353	template	30	Intron_1.f	--	Yes	--	Yes	--	--	--	--	--	--	--
G4ST01021483910	chr1:21483911-21483940	+	GGGAGTGGTCTGGGCAAGGGTCTGGCAGGG<br>((....))	ECE1	NM_001113348	chr1:21416327-21544621	template	30	Intron_2	--	Yes	--	Yes	--	--	--	--	--	--	--
G4ST01021488494	chr1:21488495-21488526	+	GGGACGGGGGTGGGGCCCTCCCCAGGAGTGGG<br>...((((...)))).	ECE1	NM_001397	chr1:21416327-21489569	template	32	Intron_2	--	Yes	--	Yes	--	--	--	Yes	Yes	--	--
G4ST01021488494	chr1:21488495-21488526	+	GGGACGGGGGTGGGGCCCTCCCCAGGAGTGGG<br>...((((...)))).	ECE1	NM_001113349	chr1:21416327-21489353	template	32	Intron_1.f	--	Yes	--	Yes	--	--	--	Yes	Yes	--	--
G4ST01021488494	chr1:21488495-21488526	+	GGGACGGGGGTGGGGCCCTCCCCAGGAGTGGG<br>...((((...)))).	ECE1	NM_001113348	chr1:21416327-21544621	template	32	Intron_2	--	Yes	--	Yes	--	--	--	Yes	Yes	--	--
G4ST01021488499	chr1:21488500-21488534	+	GGGGGTGGGGCCCTCCCCAGGAGTGGGCAGATGGG<br>...((((...)))).	ECE1	NM_001397	chr1:21416327-21489569	template	35	Intron_2	--	Yes	--	Yes	--	--	--	Yes	Yes	--	--
G4ST01021488499	chr1:21488500-21488534	+	GGGGGTGGGGCCCTCCCCAGGAGTGGGCAGATGGG<br>...((((...)))).	ECE1	NM_001113349	chr1:21416327-21489353	template	35	Intron_1.f	--	Yes	--	Yes	--	--	--	Yes	Yes	--	--
G4ST01021488499	chr1:21488500-21488534	+	GGGGGTGGGGCCCTCCCCAGGAGTGGGCAGATGGG<br>...((((...)))).	ECE1	NM_001113348	chr1:21416327-21544621	template	35	Intron_2	--	Yes	--	Yes	--	--	--	Yes	Yes	--	--
G4ST01021488918	chr1:21488919-21488953	+	GGGAGGCTCTAGGGATGGCGGGGGCAGGTGAGGGG<br>((...)).	ECE1	NM_001397	chr1:21416327-21489569	template	35	Intron_2	--	Yes	--	Yes	--	--	--	Yes	Yes	Yes	--
G4ST01021488918	chr1:21488919-21488953	+	GGGAGGCTCTAGGGATGGCGGGGGCAGGTGAGGGG<br>((...)).	ECE1	NM_001113349	chr1:21416327-21489353	template	35	Intron_1.f	--	Yes	--	Yes	--	--	--	Yes	Yes	Yes	--
G4ST01021488918	chr1:21488919-21488953	+	GGGAGGCTCTAGGGATGGCGGGGGCAGGTGAGGGG<br>((...)).	ECE1	NM_001113348	chr1:21416327-21544621	template	35	Intron_2	--	Yes	--	Yes	--	--	--	Yes	Yes	Yes	--
G4ST01021489305	chr1:21489306-21489342	-	GGGGCGGGGCGCGGCGGCGGCGGCGCCAGGGTCGGGG<br>..(((.((....)).)))..	ECE1	NM_001397	chr1:21416327-21489569	non-template	37	Intron_1.f	--	Yes	--	Yes	--	--	--	Yes	Yes	Yes	--
G4ST01021489305	chr1:21489306-21489342	-	GGGGCGGGGCGCGGCGGCGGCGGCGCCAGGGTCGGGG<br>..(((.((....)).)))..	ECE1	NM_001113349	chr1:21416327-21489353	non-template	37	Exon_1.f	--	Yes	Yes	--	--	Yes	--	Yes	Yes	Yes	--
G4ST01021489305	chr1:21489306-21489342	-	GGGGCGGGGCGCGGCGGCGGCGGCGCCAGGGTCGGGG<br>..(((.((....)).)))..	ECE1	NM_001113348	chr1:21416327-21544621	non-template	37	Intron_1.f	--	Yes	--	Yes	--	--	--	Yes	Yes	Yes	--
G4ST01021489486	chr1:21489487-21489525	-	GGGCAGCGGGACCAGGCGTCTGGGGCACAGCATGCGGGG<br>..(((....))) (((.....))).	ECE1	NM_001397	chr1:21416327-21489569	non-template	39	Exon_1.f	--	Yes	Yes	--	Yes	Yes	--	Yes	Yes	Yes	--
G4ST01021489486	chr1:21489487-21489525	-	GGGCAGCGGGACCAGGCGTCTGGGGCACAGCATGCGGGG<br>..(((....))) (((.....))).	ECE1	NM_001113349	chr1:21416327-21489353	non-template	-133	upstream	Yes	--	--	--	--	--	--	Yes	Yes	Yes	--
G4ST01021489486	chr1:21489487-21489525	-	GGGCAGCGGGACCAGGCGTCTGGGGCACAGCATGCGGGG<br>..(((....))) (((.....))).	ECE1	NM_001113348	chr1:21416327-21544621	non-template	39	Intron_1.f	--	Yes	--	Yes	--	--	--	Yes	Yes	Yes	--

G4ST01021496021	chr1:21496022-21496059	+	GGGCAGGGGCTGGGGAGGCCTCAAGGTGAGACTCAGGG<br>(((..((((...)))).))).	ECE1	NM_001113348	chr1:21416327-21544621	template	38	Intron_1.f	--	Yes	--	Yes	--	--	--	--	--	--	--
G4ST01021526160	chr1:21526161-21526195	-	GGGAGTGGGGTGCTTTGAAGCATCTACAGGGAGGG<br>(((((....)))))....	ECE1	NM_001113348	chr1:21416327-21544621	non-template	35	Intron_1.f	--	Yes	--	Yes	--	--	--	Yes	Yes	--	--
G4ST01021535093	chr1:21535094-21535128	+	GGGGGCAGGGGCAGGGCTACAGAGTGAATCTGGGG<br>...((((.....))))	ECE1	NM_001113348	chr1:21416327-21544621	template	35	Intron_1.f	--	Yes	--	Yes	--	--	--	Yes	Yes	--	--
G4ST01021544627	chr1:21544628-21544659	+	GGGGCGGGGCCGCCGCGGGGCCAATGGCAGGG<br>(((...))).	ECE1	NM_001113348	chr1:21416327-21544621	template	-6	upstream	Yes	--	--	--	--	--	--	Yes	Yes	Yes	--
G4ST01021544632	chr1:21544633-21544667	+	GGGGCCGCCGCGGGGCCAATGGCAGGGCGCGGGGG<br>(((...))).	ECE1	NM_001113348	chr1:21416327-21544621	template	-11	upstream	Yes	--	--	--	--	--	--	Yes	Yes	Yes	--
G4ST01021544643	chr1:21544644-21544673	+	GGGGCCAATGGCAGGGCGCGGGGGCCTGGG<br>(((...))).	ECE1	NM_001113348	chr1:21416327-21544621	template	-22	upstream	Yes	--	--	--	--	--	--	Yes	Yes	Yes	--
G4ST01021638925	chr1:21638926-21638973	+	GGGGCGGTTCAAGTGGCCGGGGAAATCAGCGATTTGGAGGGTCGAGGG<br>.((((.......)))) .(((((...)))))...	NBPF3	NM_032264	chr1:21639218-21683980	non-template	-244	upstream	Yes	--	--	--	--	--	--	--	Yes	Yes	--
G4ST01021708621	chr1:21708622-21708661	-	GGGGATGGACCACGTCCGCTGGGGCCCTGGGGAACCAGGG<br>..(((((...)))))...	ALPL	NM_001177520	chr1:21708445-21777492	template	40	Intron_1.f	--	Yes	--	Yes	--	--	--	Yes	Yes	--	Yes
G4ST01021708621	chr1:21708622-21708661	-	GGGGATGGACCACGTCCGCTGGGGCCCTGGGGAACCAGGG<br>..(((((...)))))...	ALPL	NM_001127501	chr1:21708445-21777492	template	40	Intron_1.f	--	Yes	--	Yes	--	--	--	Yes	Yes	--	Yes
G4ST01021708621	chr1:21708622-21708661	-	GGGGATGGACCACGTCCGCTGGGGCCCTGGGGAACCAGGG<br>..(((((...)))))...	ALPL	NM_000478	chr1:21708445-21777492	template	40	Intron_1.f	--	Yes	--	Yes	--	--	--	Yes	Yes	--	Yes
G4ST01021709019	chr1:21709020-21709058	-	GGGACGCCTGGGAGGGCGCCAGGCCACGAGGCCTTGGGG<br>....(((((....)))))..	ALPL	NM_001177520	chr1:21708445-21777492	template	39	Intron_1.f	--	Yes	--	Yes	--	--	--	--	--	--	Yes
G4ST01021709019	chr1:21709020-21709058	-	GGGACGCCTGGGAGGGCGCCAGGCCACGAGGCCTTGGGG<br>....(((((....)))))..	ALPL	NM_001127501	chr1:21708445-21777492	template	39	Intron_1.f	--	Yes	--	Yes	--	--	--	--	--	--	Yes
G4ST01021709019	chr1:21709020-21709058	-	GGGACGCCTGGGAGGGCGCCAGGCCACGAGGCCTTGGGG<br>....(((((....)))))..	ALPL	NM_000478	chr1:21708445-21777492	template	39	Intron_1.f	--	Yes	--	Yes	--	--	--	--	--	--	Yes
G4ST01021741285	chr1:21741286-21741322	-	GGGGCTGGGGGGCACCTGCATAGCTGCCTAGGGAGGG<br>.((((.((....)).))))..	ALPL	NM_001177520	chr1:21708445-21777492	template	37	Intron_1.f	--	Yes	--	Yes	--	--	--	--	--	--	--
G4ST01021741285	chr1:21741286-21741322	-	GGGGCTGGGGGGCACCTGCATAGCTGCCTAGGGAGGG<br>.((((.((....)).))))..	ALPL	NM_001127501	chr1:21708445-21777492	template	37	Intron_1.f	--	Yes	--	Yes	--	--	--	--	--	--	--
G4ST01021741285	chr1:21741286-21741322	-	GGGGCTGGGGGGCACCTGCATAGCTGCCTAGGGAGGG<br>.((((.((....)).))))..	ALPL	NM_000478	chr1:21708445-21777492	template	37	Intron_1.f	--	Yes	--	Yes	--	--	--	--	--	--	--
G4ST01021764278	chr1:21764279-21764315	-	GGGAAAAGGGCAGACGTGGGTCTCTACTGTGGAGGGG<br>.((((.....))))	ALPL	NM_001177520	chr1:21708445-21777492	template	37	Intron_4	--	Yes	--	Yes	--	--	--	--	--	--	--
G4ST01021764278	chr1:21764279-21764315	-	GGGAAAAGGGCAGACGTGGGTCTCTACTGTGGAGGGG<br>.((((.....))))	ALPL	NM_001127501	chr1:21708445-21777492	template	37	Intron_5	--	Yes	--	Yes	--	--	--	--	--	--	--
G4ST01021764278	chr1:21764279-21764315	-	GGGAAAAGGGCAGACGTGGGTCTCTACTGTGGAGGGG<br>.((((.....))))	ALPL	NM_000478	chr1:21708445-21777492	template	37	Intron_6	--	Yes	--	Yes	--	--	--	--	--	--	--
G4ST01021795919	chr1:21795920-21795949	-	GGGGGAGTGAGCACTGGGTTGGGAGCCGGG<br>..(((....)))	RAP1GAP	NM_001145658	chr1:21795295-21850935	non-template	30	Exon_25.l	--	Yes	Yes	--	--	--	Yes	--	--	--	--
G4ST01021795919	chr1:21795920-21795949	-	GGGGGAGTGAGCACTGGGTTGGGAGCCGGG<br>..(((....)))	RAP1GAP	NM_002885	chr1:21795295-21868443	non-template	30	Exon_25.l	--	Yes	Yes	--	--	--	Yes	--	--	--	--
G4ST01021795919	chr1:21795920-21795949	-	GGGGGAGTGAGCACTGGGTTGGGAGCCGGG<br>..(((....)))	RAP1GAP	NM_001145657	chr1:21795295-21868443	non-template	30	Exon_27.l	--	Yes	Yes	--	--	--	Yes	--	--	--	--
G4ST01021798731	chr1:21798732-21798765	-	GGGAGTGAGGGCCTGGCCCAGGAGGGGCTCGGGG<br>((((...))))..	RAP1GAP	NM_001145658	chr1:21795295-21850935	non-template	34	Intron_20	--	Yes	--	Yes	--	--	--	--	--	--	Yes
G4ST01021798731	chr1:21798732-21798765	-	GGGAGTGAGGGCCTGGCCCAGGAGGGGCTCGGGG<br>((((...))))..	RAP1GAP	NM_002885	chr1:21795295-21868443	non-template	34	Intron_20	--	Yes	--	Yes	--	--	--	--	--	--	Yes
G4ST01021798731	chr1:21798732-21798765	-	GGGAGTGAGGGCCTGGCCCAGGAGGGGCTCGGGG<br>((((...))))..	RAP1GAP	NM_001145657	chr1:21795295-21868443	non-template	34	Intron_22	--	Yes	--	Yes	--	--	--	--	--	--	Yes
G4ST01021798738	chr1:21798739-21798771	-	GGGCAAGGGAGTGAGGGCCTGGCCCAGGAGGGG<br>((((...))))..	RAP1GAP	NM_001145658	chr1:21795295-21850935	non-template	33	Intron_20	--	Yes	--	Yes	--	--	--	--	--	--	Yes
G4ST01021798738	chr1:21798739-21798771	-	GGGCAAGGGAGTGAGGGCCTGGCCCAGGAGGGG<br>((((...))))..	RAP1GAP	NM_002885	chr1:21795295-21868443	non-template	33	Intron_20	--	Yes	--	Yes	--	--	--	--	--	--	Yes
G4ST01021798738	chr1:21798739-21798771	-	GGGCAAGGGAGTGAGGGCCTGGCCCAGGAGGGG<br>((((...))))..	RAP1GAP	NM_001145657	chr1:21795295-21868443	non-template	33	Intron_22	--	Yes	--	Yes	--	--	--	--	--	--	Yes
G4ST01021800546	chr1:21800547-21800584	+	GGGCACCTCTGTGCTCCAGAGCCGGGCGCGGGGCTGGG<br>...(((((.....)))))..	RAP1GAP	NM_001145658	chr1:21795295-21850935	template	38	Intron_20	--	Yes	--	Yes	--	--	--	--	--	--	--
G4ST01021800546	chr1:21800547-21800584	+	GGGCACCTCTGTGCTCCAGAGCCGGGCGCGGGGCTGGG<br>...(((((.....)))))..	RAP1GAP	NM_002885	chr1:21795295-21868443	template	38	Intron_20	--	Yes	--	Yes	--	--	--	--	--	--	--
G4ST01021800546	chr1:21800547-21800584	+	GGGCACCTCTGTGCTCCAGAGCCGGGCGCGGGGCTGGG<br>...(((((.....)))))..	RAP1GAP	NM_001145657	chr1:21795295-21868443	template	38	Intron_22	--	Yes	--	Yes	--	--	--	--	--	--	--
G4ST01021800569	chr1:21800570-21800606	+	GGGCGCGGGGCTGGGCGTGCACAAAGTGCTCGCTGGG<br>((.((((...)))).))..	RAP1GAP	NM_001145658	chr1:21795295-21850935	template	37	Intron_20	--	Yes	--	Yes	--	--	--	--	--	--	--
G4ST01021800569	chr1:21800570-21800606	+	GGGCGCGGGGCTGGGCGTGCACAAAGTGCTCGCTGGG<br>((.((((...)))).))..	RAP1GAP	NM_002885	chr1:21795295-21868443	template	37	Intron_20	--	Yes	--	Yes	--	--	--	--	--	--	--
G4ST01021800569	chr1:21800570-21800606	+	GGGCGCGGGGCTGGGCGTGCACAAAGTGCTCGCTGGG<br>((.((((...)))).))..	RAP1GAP	NM_001145657	chr1:21795295-21868443	template	37	Intron_22	--	Yes	--	Yes	--	--	--	--	--	--	--
G4ST01021804904	chr1:21804905-21804932	+	GGGGAGGGCCAAGGAGCTTGGGAAGGGG<br>..(((...)))	RAP1GAP	NM_001145658	chr1:21795295-21850935	template	28	Intron_18	--	Yes	--	Yes	--	--	--	--	--	--	--
G4ST01021804904	chr1:21804905-21804932	+	GGGGAGGGCCAAGGAGCTTGGGAAGGGG<br>..(((...)))	RAP1GAP	NM_002885	chr1:21795295-21868443	template	28	Intron_18	--	Yes	--	Yes	--	--	--	--	--	--	--
G4ST01021804904	chr1:21804905-21804932	+	GGGGAGGGCCAAGGAGCTTGGGAAGGGG<br>..(((...)))	RAP1GAP	NM_001145657	chr1:21795295-21868443	template	28	Intron_19	--	Yes	--	Yes	--	--	--	--	--	--	--
G4ST01021807668	chr1:21807669-21807702	-	GGGACCTGTCAGGTGGGGGGGTGGACCTGGGGGG<br>((((...))))	RAP1GAP	NM_001145658	chr1:21795295-21850935	non-template	34	Intron_16	--	Yes	--	Yes	--	--	--	--	Yes	Yes	Yes
G4ST01021807668	chr1:21807669-21807702	-	GGGACCTGTCAGGTGGGGGGGTGGACCTGGGGGG<br>((((...))))	RAP1GAP	NM_002885	chr1:21795295-21868443	non-template	34	Intron_16	--	Yes	--	Yes	--	--	--	--	Yes	Yes	Yes
G4ST01021807668	chr1:21807669-21807702	-	GGGACCTGTCAGGTGGGGGGGTGGACCTGGGGGG<br>((((...))))	RAP1GAP	NM_001145657	chr1:21795295-21868443	non-template	34	Intron_17	--	Yes	--	Yes	--	--	--	--	Yes	Yes	Yes
G4ST01021807681	chr1:21807682-21807712	-	GGGACACCTTGGGACCTGTCAGGTGGGGGGG<br>((((...))))	RAP1GAP	NM_001145658	chr1:21795295-21850935	non-template	31	Intron_16	--	Yes	--	Yes	--	--	--	--	Yes	Yes	Yes
G4ST01021807681	chr1:21807682-21807712	-	GGGACACCTTGGGACCTGTCAGGTGGGGGGG<br>((((...))))	RAP1GAP	NM_002885	chr1:21795295-21868443	non-template	31	Intron_16	--	Yes	--	Yes	--	--	--	--	Yes	Yes	Yes
G4ST01021807681	chr1:21807682-21807712	-	GGGACACCTTGGGACCTGTCAGGTGGGGGGG<br>((((...))))	RAP1GAP	NM_001145657	chr1:21795295-21868443	non-template	31	Intron_17	--	Yes	--	Yes	--	--	--	--	Yes	Yes	Yes
G4ST01021816672	chr1:21816673-21816714	+	GGGGTATGAGGGGACAGGCCAGAGCCTGTGTGGGCCCCAGGG<br>.((((((....))))))..	RAP1GAP	NM_001145658	chr1:21795295-21850935	template	42	Intron_6	--	Yes	--	Yes	--	--	--	--	--	--	--
G4ST01021816672	chr1:21816673-21816714	+	GGGGTATGAGGGGACAGGCCAGAGCCTGTGTGGGCCCCAGGG<br>.((((((....))))))..	RAP1GAP	NM_002885	chr1:21795295-21868443	template	42	Intron_6	--	Yes	--	Yes	--	--	--	--	--	--	--
G4ST01021816672	chr1:21816673-21816714	+	GGGGTATGAGGGGACAGGCCAGAGCCTGTGTGGGCCCCAGGG<br>.((((((....))))))..	RAP1GAP	NM_001145657	chr1:21795295-21868443	template	42	Intron_7	--	Yes	--	Yes	--	--	--	--	--	--	--
G4ST01021817630	chr1:21817631-21817661	-	GGGGAGGGAGTGATAGGGAGGCAGGCCTGGG<br>(((....)))	RAP1GAP	NM_001145658	chr1:21795295-21850935	non-template	31	Intron_5	--	Yes	--	Yes	--	--	--	--	--	--	--
G4ST01021817630	chr1:21817631-21817661	-	GGGGAGGGAGTGATAGGGAGGCAGGCCTGGG<br>(((....)))	RAP1GAP	NM_002885	chr1:21795295-21868443	non-template	31	Intron_5	--	Yes	--	Yes	--	--	--	--	--	--	--
G4ST01021817630	chr1:21817631-21817661	-	GGGGAGGGAGTGATAGGGAGGCAGGCCTGGG<br>(((....)))	RAP1GAP	NM_001145657	chr1:21795295-21868443	non-template	31	Intron_6	--	Yes	--	Yes	--	--	--	--	--	--	--
G4ST01021818339	chr1:21818340-21818375	+	GGGGCTGGAGGCTGGCAGCCTCAGGGAGGGCTGGGG<br>....((((((...))))))).	RAP1GAP	NM_001145658	chr1:21795295-21850935	template	36	Intron_4	--	Yes	--	Yes	--	--	--	Yes	Yes	--	Yes

G4ST01021818339	chr1:21818340-21818375	+	GGGGCTGGAGGCTGGCAGCCTCAGGGAGGGCTGGGG<br>....((((((...)))))).	RAP1GAP	NM_002885	chr1:21795295-21868443	template	36	Intron_4	--	Yes	--	Yes	--	--	--	Yes	Yes	--	Yes
G4ST01021818339	chr1:21818340-21818375	+	GGGGCTGGAGGCTGGCAGCCTCAGGGAGGGCTGGGG<br>....((((((...)))))).	RAP1GAP	NM_001145657	chr1:21795295-21868443	template	36	Intron_5	--	Yes	--	Yes	--	--	--	Yes	Yes	--	Yes
G4ST01021819031	chr1:21819032-21819068	-	GGGGAGCCACAGAGCTGGCTGGGCTGGGGCTGGGGGG<br>.(((((......)))))	RAP1GAP	NM_001145658	chr1:21795295-21850935	non-template	37	Intron_4	--	Yes	--	Yes	--	--	--	--	--	--	--
G4ST01021819031	chr1:21819032-21819068	-	GGGGAGCCACAGAGCTGGCTGGGCTGGGGCTGGGGGG<br>.(((((......)))))	RAP1GAP	NM_002885	chr1:21795295-21868443	non-template	37	Intron_4	--	Yes	--	Yes	--	--	--	--	--	--	--
G4ST01021819031	chr1:21819032-21819068	-	GGGGAGCCACAGAGCTGGCTGGGCTGGGGCTGGGGGG<br>.(((((......)))))	RAP1GAP	NM_001145657	chr1:21795295-21868443	non-template	37	Intron_5	--	Yes	--	Yes	--	--	--	--	--	--	--
G4ST01021821634	chr1:21821635-21821670	+	GGGCTCGCCCCACGGCGGGGCCCGGAGGGGGCGGGG<br>..((((....))))	RAP1GAP	NM_001145658	chr1:21795295-21850935	template	36	Intron_3	--	Yes	--	Yes	--	--	--	Yes	--	Yes	--
G4ST01021821634	chr1:21821635-21821670	+	GGGCTCGCCCCACGGCGGGGCCCGGAGGGGGCGGGG<br>..((((....))))	RAP1GAP	NM_002885	chr1:21795295-21868443	template	36	Intron_3	--	Yes	--	Yes	--	--	--	Yes	--	Yes	--
G4ST01021821634	chr1:21821635-21821670	+	GGGCTCGCCCCACGGCGGGGCCCGGAGGGGGCGGGG<br>..((((....))))	RAP1GAP	NM_001145657	chr1:21795295-21868443	template	36	Intron_4	--	Yes	--	Yes	--	--	--	Yes	--	Yes	--
G4ST01021824014	chr1:21824015-21824052	+	GGGCTCCTCCTGAGATGCTCAGGGGAAGAGGGACTGGG<br>......(((((...)))))	RAP1GAP	NM_001145658	chr1:21795295-21850935	template	38	Intron_3	--	Yes	--	Yes	--	--	--	Yes	Yes	--	--
G4ST01021824014	chr1:21824015-21824052	+	GGGCTCCTCCTGAGATGCTCAGGGGAAGAGGGACTGGG<br>......(((((...)))))	RAP1GAP	NM_002885	chr1:21795295-21868443	template	38	Intron_3	--	Yes	--	Yes	--	--	--	Yes	Yes	--	--
G4ST01021824014	chr1:21824015-21824052	+	GGGCTCCTCCTGAGATGCTCAGGGGAAGAGGGACTGGG<br>......(((((...)))))	RAP1GAP	NM_001145657	chr1:21795295-21868443	template	38	Intron_4	--	Yes	--	Yes	--	--	--	Yes	Yes	--	--
G4ST01021830941	chr1:21830942-21830972	-	GGGACTGAGGGCCTGGGCTGAGGACAGTGGG<br>(((....))).	RAP1GAP	NM_001145658	chr1:21795295-21850935	non-template	31	Intron_2	--	Yes	--	Yes	--	--	--	--	Yes	--	--
G4ST01021830941	chr1:21830942-21830972	-	GGGACTGAGGGCCTGGGCTGAGGACAGTGGG<br>(((....))).	RAP1GAP	NM_002885	chr1:21795295-21868443	non-template	31	Intron_2	--	Yes	--	Yes	--	--	--	--	Yes	--	--
G4ST01021830941	chr1:21830942-21830972	-	GGGACTGAGGGCCTGGGCTGAGGACAGTGGG<br>(((....))).	RAP1GAP	NM_001145657	chr1:21795295-21868443	non-template	31	Intron_3	--	Yes	--	Yes	--	--	--	--	Yes	--	--
G4ST01021831267	chr1:21831268-21831295	+	GGGAAAGGGTGAAGCAAGGGTGTGGGGG<br>((...)).	RAP1GAP	NM_001145658	chr1:21795295-21850935	template	28	Intron_2	--	Yes	--	Yes	--	--	--	Yes	Yes	--	--
G4ST01021831267	chr1:21831268-21831295	+	GGGAAAGGGTGAAGCAAGGGTGTGGGGG<br>((...)).	RAP1GAP	NM_002885	chr1:21795295-21868443	template	28	Intron_2	--	Yes	--	Yes	--	--	--	Yes	Yes	--	--
G4ST01021831267	chr1:21831268-21831295	+	GGGAAAGGGTGAAGCAAGGGTGTGGGGG<br>((...)).	RAP1GAP	NM_001145657	chr1:21795295-21868443	template	28	Intron_3	--	Yes	--	Yes	--	--	--	Yes	Yes	--	--
G4ST01021831273	chr1:21831274-21831300	+	GGGTGAAGCAAGGGTGTGGGGGAAGGG<br>((...)).	RAP1GAP	NM_001145658	chr1:21795295-21850935	template	27	Intron_2	--	Yes	--	Yes	--	--	--	Yes	Yes	--	--
G4ST01021831273	chr1:21831274-21831300	+	GGGTGAAGCAAGGGTGTGGGGGAAGGG<br>((...)).	RAP1GAP	NM_002885	chr1:21795295-21868443	template	27	Intron_2	--	Yes	--	Yes	--	--	--	Yes	Yes	--	--
G4ST01021831273	chr1:21831274-21831300	+	GGGTGAAGCAAGGGTGTGGGGGAAGGG<br>((...)).	RAP1GAP	NM_001145657	chr1:21795295-21868443	template	27	Intron_3	--	Yes	--	Yes	--	--	--	Yes	Yes	--	--
G4ST01021832234	chr1:21832235-21832273	+	GGGCACCAGAAGTGTGGTGGGGCTGTCCTGGGTGGTGGG<br>(((((......)))))	RAP1GAP	NM_001145658	chr1:21795295-21850935	template	39	Intron_2	--	Yes	--	Yes	--	--	--	--	Yes	--	--
G4ST01021832234	chr1:21832235-21832273	+	GGGCACCAGAAGTGTGGTGGGGCTGTCCTGGGTGGTGGG<br>(((((......)))))	RAP1GAP	NM_002885	chr1:21795295-21868443	template	39	Intron_2	--	Yes	--	Yes	--	--	--	--	Yes	--	--
G4ST01021832234	chr1:21832235-21832273	+	GGGCACCAGAAGTGTGGTGGGGCTGTCCTGGGTGGTGGG<br>(((((......)))))	RAP1GAP	NM_001145657	chr1:21795295-21868443	template	39	Intron_3	--	Yes	--	Yes	--	--	--	--	Yes	--	--
G4ST01021840491	chr1:21840492-21840528	-	GGGTATCAGGGAGGGGCCTCTCTGAGGCCTGAATGGG<br>(((((...))))))......	RAP1GAP	NM_001145658	chr1:21795295-21850935	non-template	37	Intron_2	--	Yes	--	Yes	--	--	--	--	--	--	--
G4ST01021840491	chr1:21840492-21840528	-	GGGTATCAGGGAGGGGCCTCTCTGAGGCCTGAATGGG<br>(((((...))))))......	RAP1GAP	NM_002885	chr1:21795295-21868443	non-template	37	Intron_2	--	Yes	--	Yes	--	--	--	--	--	--	--
G4ST01021840491	chr1:21840492-21840528	-	GGGTATCAGGGAGGGGCCTCTCTGAGGCCTGAATGGG<br>(((((...))))))......	RAP1GAP	NM_001145657	chr1:21795295-21868443	non-template	37	Intron_3	--	Yes	--	Yes	--	--	--	--	--	--	--
G4ST01021850988	chr1:21850989-21851026	-	GGGGCGCGGACGTATCCGAGCTGGGGCGGGGGCGGGGG<br>((.((((....)))).))..	RAP1GAP	NM_001145658	chr1:21795295-21850935	non-template	-53	upstream	Yes	--	--	--	--	--	--	Yes	Yes	Yes	Yes
G4ST01021850988	chr1:21850989-21851026	-	GGGGCGCGGACGTATCCGAGCTGGGGCGGGGGCGGGGG<br>((.((((....)))).))..	RAP1GAP	NM_002885	chr1:21795295-21868443	non-template	38	Intron_1.f	--	Yes	--	Yes	--	--	--	Yes	Yes	Yes	Yes
G4ST01021850988	chr1:21850989-21851026	-	GGGGCGCGGACGTATCCGAGCTGGGGCGGGGGCGGGGG<br>((.((((....)))).))..	RAP1GAP	NM_001145657	chr1:21795295-21868443	non-template	38	Intron_2	--	Yes	--	Yes	--	--	--	Yes	Yes	Yes	Yes
G4ST01021851179	chr1:21851180-21851209	-	GGGGGGCGGCGGCCGGGAAGGGGGTGGGGG<br>.(((....)))	RAP1GAP	NM_001145658	chr1:21795295-21850935	non-template	-244	upstream	Yes	--	--	--	--	--	--	--	--	--	Yes
G4ST01021851179	chr1:21851180-21851209	-	GGGGGGCGGCGGCCGGGAAGGGGGTGGGGG<br>.(((....)))	RAP1GAP	NM_002885	chr1:21795295-21868443	non-template	30	Intron_1.f	--	Yes	--	Yes	--	--	--	--	--	--	Yes
G4ST01021851179	chr1:21851180-21851209	-	GGGGGGCGGCGGCCGGGAAGGGGGTGGGGG<br>.(((....)))	RAP1GAP	NM_001145657	chr1:21795295-21868443	non-template	30	Intron_2	--	Yes	--	Yes	--	--	--	--	--	--	Yes
G4ST01021851885	chr1:21851886-21851917	-	GGGCTGGAGGGCAGTGTGCTCTGGGGTCAGGG<br>(((......)))	RAP1GAP	NM_001145658	chr1:21795295-21850935	non-template	-950	upstream	Yes	--	--	--	--	--	--	--	--	--	Yes
G4ST01021851885	chr1:21851886-21851917	-	GGGCTGGAGGGCAGTGTGCTCTGGGGTCAGGG<br>(((......)))	RAP1GAP	NM_002885	chr1:21795295-21868443	non-template	32	Intron_1.f	--	Yes	--	Yes	--	--	--	--	--	--	Yes
G4ST01021851885	chr1:21851886-21851917	-	GGGCTGGAGGGCAGTGTGCTCTGGGGTCAGGG<br>(((......)))	RAP1GAP	NM_001145657	chr1:21795295-21868443	non-template	32	Intron_2	--	Yes	--	Yes	--	--	--	--	--	--	Yes
G4ST01021851891	chr1:21851892-21851924	-	GGGTGTTGGGCTGGAGGGCAGTGTGCTCTGGGG<br>(((......)))	RAP1GAP	NM_001145658	chr1:21795295-21850935	non-template	-956	upstream	Yes	--	--	--	--	--	--	--	--	--	Yes
G4ST01021851891	chr1:21851892-21851924	-	GGGTGTTGGGCTGGAGGGCAGTGTGCTCTGGGG<br>(((......)))	RAP1GAP	NM_002885	chr1:21795295-21868443	non-template	33	Intron_1.f	--	Yes	--	Yes	--	--	--	--	--	--	Yes
G4ST01021851891	chr1:21851892-21851924	-	GGGTGTTGGGCTGGAGGGCAGTGTGCTCTGGGG<br>(((......)))	RAP1GAP	NM_001145657	chr1:21795295-21868443	non-template	33	Intron_2	--	Yes	--	Yes	--	--	--	--	--	--	Yes
G4ST01021863559	chr1:21863560-21863594	-	GGGCAAGGGCTGGGGGTGGCAATGTGGACACTGGG<br>.(((.((...))..))).	RAP1GAP	NM_002885	chr1:21795295-21868443	non-template	35	Intron_1.f	--	Yes	--	Yes	--	--	--	--	--	--	--
G4ST01021863559	chr1:21863560-21863594	-	GGGCAAGGGCTGGGGGTGGCAATGTGGACACTGGG<br>.(((.((...))..))).	RAP1GAP	NM_001145657	chr1:21795295-21868443	non-template	35	Intron_2	--	Yes	--	Yes	--	--	--	--	--	--	--
G4ST01021982313	chr1:21982314-21982348	-	GGGTTAGGGGCGCGGGAGGCGCAGAGGCTGCTGGG<br>....((((...)))).	USP48	NM_001032730	chr1:21926926-21982275	non-template	-38	upstream	Yes	--	--	--	--	--	--	Yes	Yes	Yes	--
G4ST01021982313	chr1:21982314-21982348	-	GGGTTAGGGGCGCGGGAGGCGCAGAGGCTGCTGGG<br>....((((...)))).	USP48	NM_032236	chr1:21877379-21982275	non-template	-38	upstream	Yes	--	--	--	--	--	--	Yes	Yes	Yes	--
G4ST01022013745	chr1:22013746-22013778	-	GGGGACCCCAGGGGCCGGGGCGCCCCCGGCGGG<br>..(((...)))	LDLRAD2	NM_001013693	chr1:22011345-22024301	template	33	Exon_2	--	Yes	Yes	--	Yes	--	--	Yes	Yes	Yes	--
G4ST01022022846	chr1:22022847-22022874	-	GGGAGGGGACTCTGTCGTGGGGCCGGGG<br>(((...)))...	HSPG2	NM_005529	chr1:22021324-22136337	non-template	28	Intron_95	--	Yes	--	Yes	--	--	--	--	--	Yes	--
G4ST01022022846	chr1:22022847-22022874	-	GGGAGGGGACTCTGTCGTGGGGCCGGGG<br>(((...)))...	LDLRAD2	NM_001013693	chr1:22011345-22024301	template	28	Exon_5.l	--	Yes	Yes	--	--	--	Yes	--	--	Yes	--
G4ST01022030915	chr1:22030916-22030950	+	GGGGGTTTGTAAGAAGGGCTAGGGGCCCAGTTGGG<br>..(((....)))	HSPG2	NM_005529	chr1:22021324-22136337	template	35	Intron_81	--	Yes	--	Yes	--	--	--	--	--	Yes	Yes
G4ST01022037778	chr1:22037779-22037815	+	GGGGAGATGAGGGCATTCCAGGTGGGGTTATGGAGGG<br>(((.....)))	HSPG2	NM_005529	chr1:22021324-22136337	template	37	Intron_74	--	Yes	--	Yes	--	--	--	--	--	Yes	--
G4ST01022043554	chr1:22043555-22043584	-	GGGGCCATTGCCTGGGGGCGGGGCTGGGGG<br>.(((.....)))	HSPG2	NM_005529	chr1:22021324-22136337	non-template	30	Intron_64	--	Yes	--	Yes	--	--	--	--	--	Yes	--
G4ST01022071916	chr1:22071917-22071954	-	GGGCAGGGGCTGCCCGGGGCTGCTGGAAGTGGCCAGGG<br>((((((...))))))..	HSPG2	NM_005529	chr1:22021324-22136337	non-template	38	Intron_31	--	Yes	--	Yes	--	--	--	--	--	--	Yes
G4ST01022074662	chr1:22074663-22074696	-	GGGGCAGGAGGACCAGCATGGGCTTGGGGAGGGG<br>((.((....)).))..	HSPG2	NM_005529	chr1:22021324-22136337	non-template	34	Intron_25	--	Yes	--	Yes	--	--	--	Yes	--	--	--

G4ST01022075501	chr1:22075502-22075528	+	GGGCTGGGATGGGGGCCGTGGCCTGGG<br>(((....))).	HSPG2	NM_005529	chr1:22021324-22136337	template	27	Intron_22	--	Yes	--	Yes	--	--	--	--	--	--	--
G4ST01022083759	chr1:22083760-22083797	+	GGGCGGCATGGGCGGGGGCTGCAGCCGGCTGTGGTGGG<br>..((((((....)))))).	HSPG2	NM_005529	chr1:22021324-22136337	template	38	Intron_12	--	Yes	--	Yes	--	--	--	--	--	--	Yes
G4ST01022083800	chr1:22083801-22083835	-	GGGCAGGTGTCACCGGGTGTGGGGGCAGAGCCGGG<br>..(((...)))	HSPG2	NM_005529	chr1:22021324-22136337	non-template	35	Intron_12	--	Yes	--	Yes	--	--	--	--	--	--	--
G4ST01022091384	chr1:22091385-22091414	-	GGGGCTGAGGCCAGGGGCTTGGGTGGGGGG<br>.(((....)))	HSPG2	NM_005529	chr1:22021324-22136337	non-template	30	Intron_3	--	Yes	--	Yes	--	--	--	--	--	--	--
G4ST01022091391	chr1:22091392-22091421	-	GGGCCGTGGGGCTGAGGCCAGGGGCTTGGG<br>.(((....)))	HSPG2	NM_005529	chr1:22021324-22136337	non-template	30	Intron_3	--	Yes	--	Yes	--	--	--	--	--	--	--
G4ST01022092241	chr1:22092242-22092272	-	GGGACCCAGGGCTGGAGTTCGGGGGCCTGGG<br>(((.....)))	HSPG2	NM_005529	chr1:22021324-22136337	non-template	31	Intron_3	--	Yes	--	Yes	--	--	--	--	--	--	Yes
G4ST01022092247	chr1:22092248-22092282	-	GGGACCCACAGGGACCCAGGGCTGGAGTTCGGGGG<br>(((.....)))	HSPG2	NM_005529	chr1:22021324-22136337	non-template	35	Intron_3	--	Yes	--	Yes	--	--	--	--	--	--	Yes
G4ST01022092349	chr1:22092350-22092391	-	GGGGTGTTGACGGGCAGAGATGGGTGCAGCAGCCCTGCAGGG<br>.((((.....)))).	HSPG2	NM_005529	chr1:22021324-22136337	non-template	42	Intron_3	--	Yes	--	Yes	--	--	--	--	--	--	--
G4ST01022093257	chr1:22093258-22093299	+	GGGACGGGTCTTGACACACAGGGGGCTGGAGCTGAGCCAGGG<br>.((((.....)))) ((((.......)))).	HSPG2	NM_005529	chr1:22021324-22136337	template	42	Intron_3	--	Yes	--	Yes	--	--	--	--	--	--	--
G4ST01022093633	chr1:22093634-22093674	-	GGGGGAAGAGGTGGGGAACAGGGCACAGCAGCTGTAGGGGG<br>.((((...))))...	HSPG2	NM_005529	chr1:22021324-22136337	non-template	41	Intron_3	--	Yes	--	Yes	--	--	--	Yes	Yes	--	--
G4ST01022093711	chr1:22093712-22093764	+	GGGCAGGTGGGCTGGCCAGGACTGCCCAGCTGGGGCAGAGCTGCTGCAGTGGG<br>((((.((....)).))))... ((((.....))))...	HSPG2	NM_005529	chr1:22021324-22136337	template	53	Intron_3	--	Yes	--	Yes	--	--	--	Yes	Yes	--	--
G4ST01022097993	chr1:22097994-22098029	+	GGGAGCAGGGAGCTGGTCAGCGGGTGGCGGCGGGGG<br>.(((....)))	HSPG2	NM_005529	chr1:22021324-22136337	template	36	Intron_1.f	--	Yes	--	Yes	--	--	--	Yes	--	Yes	--
G4ST01022098000	chr1:22098001-22098039	+	GGGAGCTGGTCAGCGGGTGGCGGCGGGGGTGGCCTTGGG<br>.(((....)))	HSPG2	NM_005529	chr1:22021324-22136337	template	39	Intron_1.f	--	Yes	--	Yes	--	--	--	Yes	--	Yes	--
G4ST01022110419	chr1:22110420-22110457	+	GGGCCTGGCCCAAGCAGGGGGAGAATGGGCAGGCTGGG<br>((((.......))))	HSPG2	NM_005529	chr1:22021324-22136337	template	38	Intron_1.f	--	Yes	--	Yes	--	--	--	Yes	Yes	Yes	--
G4ST01022130262	chr1:22130263-22130302	-	GGGGTGTGGCTCGTGGCTGCTGGGGTGGCCGGGTCGGGGG<br>..(((((.....)))))..	HSPG2	NM_005529	chr1:22021324-22136337	non-template	40	Intron_1.f	--	Yes	--	Yes	--	--	--	Yes	Yes	Yes	--
G4ST01022136563	chr1:22136564-22136599	-	GGGCCGGGCCGCGGCCCGCGCGGAGGGGGCTGGGGG<br>(((((.....)))))...	HSPG2	NM_005529	chr1:22021324-22136337	non-template	-226	upstream	Yes	--	--	--	--	--	--	Yes	Yes	Yes	Yes
G4ST01022136570	chr1:22136571-22136607	-	GGGGAGCCGGGCCGGGCCGCGGCCCGCGCGGAGGGGG<br>(((((.....)))))...	HSPG2	NM_005529	chr1:22021324-22136337	non-template	-233	upstream	Yes	--	--	--	--	--	--	Yes	Yes	Yes	Yes
G4ST01022251984	chr1:22251985-22252014	+	GGGCGGGCGCGGGTCCGCGTTCTGTGGGGG<br>.((((.....))))	CDC42	NM_001791	chr1:22251707-22292023	non-template	30	Intron_1.f	--	Yes	--	Yes	--	--	--	--	Yes	Yes	--
G4ST01022251984	chr1:22251985-22252014	+	GGGCGGGCGCGGGTCCGCGTTCTGTGGGGG<br>.((((.....))))	CDC42	NM_044472	chr1:22251707-22289883	non-template	30	Intron_1.f	--	Yes	--	Yes	--	--	--	--	Yes	Yes	--
G4ST01022251984	chr1:22251985-22252014	+	GGGCGGGCGCGGGTCCGCGTTCTGTGGGGG<br>.((((.....))))	CDC42	NM_001039802	chr1:22251707-22292023	non-template	30	Intron_1.f	--	Yes	--	Yes	--	--	--	--	Yes	Yes	--
G4ST01022251988	chr1:22251989-22252022	+	GGGCGCGGGTCCGCGTTCTGTGGGGGAGCCGGGG<br>.((((.....))))	CDC42	NM_001791	chr1:22251707-22292023	non-template	34	Intron_1.f	--	Yes	--	Yes	--	--	--	--	Yes	Yes	--
G4ST01022251988	chr1:22251989-22252022	+	GGGCGCGGGTCCGCGTTCTGTGGGGGAGCCGGGG<br>.((((.....))))	CDC42	NM_044472	chr1:22251707-22289883	non-template	34	Intron_1.f	--	Yes	--	Yes	--	--	--	--	Yes	Yes	--
G4ST01022251988	chr1:22251989-22252022	+	GGGCGCGGGTCCGCGTTCTGTGGGGGAGCCGGGG<br>.((((.....))))	CDC42	NM_001039802	chr1:22251707-22292023	non-template	34	Intron_1.f	--	Yes	--	Yes	--	--	--	--	Yes	Yes	--
G4ST01022251994	chr1:22251995-22252028	+	GGGTCCGCGTTCTGTGGGGGAGCCGGGGCGGGGG<br>.((((.....))))	CDC42	NM_001791	chr1:22251707-22292023	non-template	34	Intron_1.f	--	Yes	--	Yes	--	--	--	--	Yes	Yes	--
G4ST01022251994	chr1:22251995-22252028	+	GGGTCCGCGTTCTGTGGGGGAGCCGGGGCGGGGG<br>.((((.....))))	CDC42	NM_044472	chr1:22251707-22289883	non-template	34	Intron_1.f	--	Yes	--	Yes	--	--	--	--	Yes	Yes	--
G4ST01022251994	chr1:22251995-22252028	+	GGGTCCGCGTTCTGTGGGGGAGCCGGGGCGGGGG<br>.((((.....))))	CDC42	NM_001039802	chr1:22251707-22292023	non-template	34	Intron_1.f	--	Yes	--	Yes	--	--	--	--	Yes	Yes	--
G4ST01022252348	chr1:22252349-22252386	+	GGGCGGGGGAGGGGAGTGGCGTTGCGGCCGCGGCGGGG<br>......((((((....))))))	CDC42	NM_001791	chr1:22251707-22292023	non-template	38	Intron_1.f	--	Yes	--	Yes	--	--	--	Yes	Yes	Yes	Yes
G4ST01022252348	chr1:22252349-22252386	+	GGGCGGGGGAGGGGAGTGGCGTTGCGGCCGCGGCGGGG<br>......((((((....))))))	CDC42	NM_044472	chr1:22251707-22289883	non-template	38	Intron_1.f	--	Yes	--	Yes	--	--	--	Yes	Yes	Yes	Yes
G4ST01022252348	chr1:22252349-22252386	+	GGGCGGGGGAGGGGAGTGGCGTTGCGGCCGCGGCGGGG<br>......((((((....))))))	CDC42	NM_001039802	chr1:22251707-22292023	non-template	38	Intron_1.f	--	Yes	--	Yes	--	--	--	Yes	Yes	Yes	Yes
G4ST01022319607	chr1:22319608-22319643	+	GGGTGGGGGAGAGGGGCCATCAGGAGATGGCAAGGG<br>((((((....))))))..	WNT4	NM_030761	chr1:22316385-22342106	template	36	Intron_4.l	--	Yes	--	Yes	--	--	--	--	--	Yes	Yes
G4ST01022319611	chr1:22319612-22319653	+	GGGGGAGAGGGGCCATCAGGAGATGGCAAGGGAAGGCAGGGG<br>((((((....))))))..	WNT4	NM_030761	chr1:22316385-22342106	template	42	Intron_4.l	--	Yes	--	Yes	--	--	--	--	--	Yes	Yes
G4ST01022319619	chr1:22319620-22319657	+	GGGGCCATCAGGAGATGGCAAGGGAAGGCAGGGGAGGG<br>((((((....))))))..	WNT4	NM_030761	chr1:22316385-22342106	template	38	Intron_4.l	--	Yes	--	Yes	--	--	--	--	--	Yes	Yes
G4ST01022334650	chr1:22334651-22334685	+	GGGGTGCTGGGGACAGATGTCTGGGTGGTTACGGG<br>(((....))).	WNT4	NM_030761	chr1:22316385-22342106	template	35	Intron_1.f	--	Yes	--	Yes	--	--	--	--	--	--	--
G4ST01022338592	chr1:22338593-22338629	-	GGGGTTCTAGCAGAATCCCACGGGTGAGGGGAGGGGG<br>(((((...)))))....	WNT4	NM_030761	chr1:22316385-22342106	non-template	37	Intron_1.f	--	Yes	--	Yes	--	--	--	--	--	--	--
G4ST01022338598	chr1:22338599-22338637	-	GGGGTAGCGGGGTTCTAGCAGAATCCCACGGGTGAGGGG<br>(((((...)))))....	WNT4	NM_030761	chr1:22316385-22342106	non-template	39	Intron_1.f	--	Yes	--	Yes	--	--	--	--	--	--	--
G4ST01022342083	chr1:22342084-22342110	+	GGGCGGGCCGGGGCGCGCGCGGCGGGG<br>.(((.....)))	WNT4	NM_030761	chr1:22316385-22342106	template	23	upstream-Exon_1.f	Yes	Yes	Yes	--	--	Yes	--	--	Yes	--	Yes
G4ST01022342749	chr1:22342750-22342777	-	GGGGGCCAGGAGGCAGGGAGGGGCCGGG<br>.(((....))).	WNT4	NM_030761	chr1:22316385-22342106	non-template	-643	upstream	Yes	--	--	--	--	--	--	--	Yes	--	Yes
G4ST01022342754	chr1:22342755-22342791	-	GGGCCGCAGGACGCGGGGGCCAGGAGGCAGGGAGGGG<br>((((.....)))) .(((....))).	WNT4	NM_030761	chr1:22316385-22342106	non-template	-648	upstream	Yes	--	--	--	--	--	--	--	Yes	--	Yes
G4ST01022342759	chr1:22342760-22342797	-	GGGGCTGGGCCGCAGGACGCGGGGGCCAGGAGGCAGGG<br>((((.....)))) .(((....))).	WNT4	NM_030761	chr1:22316385-22342106	non-template	-653	upstream	Yes	--	--	--	--	--	--	--	Yes	--	Yes
G4ST01022342772	chr1:22342773-22342801	-	GGGAGGGGCTGGGCCGCAGGACGCGGGGG<br>((((.....))))	WNT4	NM_030761	chr1:22316385-22342106	non-template	-666	upstream	Yes	--	--	--	--	--	--	--	Yes	--	--
G4ST01022342788	chr1:22342789-22342816	-	GGGAGGTGAGCGCCAGGGAGGGGCTGGG<br>.(((....))).	WNT4	NM_030761	chr1:22316385-22342106	non-template	-682	upstream	Yes	--	--	--	--	--	--	--	Yes	--	--
G4ST01022762698	chr1:22762699-22762724	-	GGGGGCCATGGCCGGGCCGGGCCGGG<br>(((....)))	EPHA8	NM_020526	chr1:22762591-22802674	template	26	Exon_1.f	--	Yes	Yes	--	Yes	Yes	--	--	--	Yes	Yes
G4ST01022762698	chr1:22762699-22762724	-	GGGGGCCATGGCCGGGCCGGGCCGGG<br>(((....)))	EPHA8	NM_001006943	chr1:22762591-22788699	template	26	Exon_1.f	--	Yes	Yes	--	Yes	Yes	--	--	--	Yes	Yes
G4ST01022762703	chr1:22762704-22762728	-	GGGCGGGGGCCATGGCCGGGCCGGG<br>(((....)))	EPHA8	NM_020526	chr1:22762591-22802674	template	25	Exon_1.f	--	Yes	Yes	--	Yes	Yes	--	--	--	Yes	Yes
G4ST01022762703	chr1:22762704-22762728	-	GGGCGGGGGCCATGGCCGGGCCGGG<br>(((....)))	EPHA8	NM_001006943	chr1:22762591-22788699	template	25	Exon_1.f	--	Yes	Yes	--	Yes	Yes	--	--	--	Yes	Yes
G4ST01022796655	chr1:22796656-22796691	+	GGGGTCTGGCAAGCCCAGGGGGTCCAAGGGCCTGGG<br>..((((.....)))).	EPHA8	NM_020526	chr1:22762591-22802674	non-template	36	Intron_10	--	Yes	--	Yes	--	--	--	--	--	--	Yes
G4ST01022797320	chr1:22797321-22797348	+	GGGCTTGGGGAGGGAGGTCCAGTCTGGG<br>(((.....)))	EPHA8	NM_020526	chr1:22762591-22802674	non-template	28	Intron_12	--	Yes	--	Yes	--	--	--	--	--	--	--
G4ST01022797326	chr1:22797327-22797355	+	GGGGAGGGAGGTCCAGTCTGGGTGCTGGG<br>(((.....)))	EPHA8	NM_020526	chr1:22762591-22802674	non-template	29	Intron_12	--	Yes	--	Yes	--	--	--	--	--	--	--
G4ST01022800562	chr1:22800563-22800593	+	GGGTGGGGCTGGGGCCCCATGCGTGTGGGGG<br>...((((....))))	EPHA8	NM_020526	chr1:22762591-22802674	non-template	31	Intron_16.l	--	Yes	--	Yes	--	--	--	--	--	--	--
G4ST01022800566	chr1:22800567-22800598	+	GGGGCTGGGGCCCCATGCGTGTGGGGGCAGGG<br>...((((....))))	EPHA8	NM_020526	chr1:22762591-22802674	non-template	32	Intron_16.l	--	Yes	--	Yes	--	--	--	--	--	--	--

G4ST01022800572	chr1:22800573-22800604	+	GGGGCCCCATGCGTGTGGGGGCAGGGGGGGGG<br>...((((....))))	EPHA8	NM_020526	chr1:22762591-22802674	non-template	32	Intron_16.l	--	Yes	--	Yes	--	--	--	--	--	--	--
G4ST01022800660	chr1:22800661-22800695	-	GGGTGCTGCTGGCCAGCTGGGCCTGGGAGGCTGGG<br>.((((.....)))).	EPHA8	NM_020526	chr1:22762591-22802674	template	35	Intron_16.l	--	Yes	--	Yes	--	--	--	--	--	--	--
G4ST01022802250	chr1:22802251-22802282	-	GGGATAGCTGTGCTGGCTATGGGGAGGGGGGG<br>((((((.....)))))).	EPHA8	NM_020526	chr1:22762591-22802674	template	32	Exon_17.l	--	Yes	Yes	--	--	--	Yes	--	--	--	Yes
G4ST01022840990	chr1:22840991-22841019	+	GGGGAAGGGCCCTGGGGCTGAGCCAGGGG<br>.(((...)))	C1QC	NM_001114101	chr1:22842705-22847190	non-template	-1685	upstream	Yes	--	--	--	--	--	--	--	Yes	--	--
G4ST01022840990	chr1:22840991-22841019	+	GGGGAAGGGCCCTGGGGCTGAGCCAGGGG<br>.(((...)))	C1QC	NM_172369	chr1:22842705-22847190	non-template	-1685	upstream	Yes	--	--	--	--	--	--	--	Yes	--	--
G4ST01022846388	chr1:22846389-22846428	-	GGGGCCGGGCACCCCTGGCATCCCAGGGGGTCCCATGGGG<br>....(((((....)))))	C1QC	NM_001114101	chr1:22842705-22847190	template	40	Exon_3.l	--	Yes	Yes	--	Yes	--	--	--	--	--	--
G4ST01022846388	chr1:22846389-22846428	-	GGGGCCGGGCACCCCTGGCATCCCAGGGGGTCCCATGGGG<br>....(((((....)))))	C1QC	NM_172369	chr1:22842705-22847190	template	40	Exon_3.l	--	Yes	Yes	--	Yes	--	--	--	--	--	--
G4ST01022909493	chr1:22909494-22909524	-	GGGCGGGGGAAGGGGCTTCCCAAAGCGGGGG<br>((((....))))..	EPHB2	NM_017449	chr1:22909918-23114410	template	-393	upstream	Yes	--	--	--	--	--	--	Yes	Yes	Yes	Yes
G4ST01022909493	chr1:22909494-22909524	-	GGGCGGGGGAAGGGGCTTCCCAAAGCGGGGG<br>((((....))))..	EPHB2	NM_004442	chr1:22909918-23114410	template	-393	upstream	Yes	--	--	--	--	--	--	Yes	Yes	Yes	Yes
G4ST01022910224	chr1:22910225-22910279	+	GGGGGCCGGCGCCGGGCGCAGGTGGCCGCGGGGAGGTGTGTCCGGCCGCGCCGGG<br>(((....))) (((.((...))))) ..((((((......))))))	EPHB2	NM_017449	chr1:22909918-23114410	non-template	55	Intron_1.f	--	Yes	--	Yes	--	--	--	Yes	--	--	Yes
G4ST01022910224	chr1:22910225-22910279	+	GGGGGCCGGCGCCGGGCGCAGGTGGCCGCGGGGAGGTGTGTCCGGCCGCGCCGGG<br>(((....))) (((.((...))))) ..((((((......))))))	EPHB2	NM_004442	chr1:22909918-23114410	non-template	55	Intron_1.f	--	Yes	--	Yes	--	--	--	Yes	--	--	Yes
G4ST01022910237	chr1:22910238-22910288	+	GGGCGCAGGTGGCCGCGGGGAGGTGTGTCCGGCCGCGCCGGGAGGAGCGGG<br>(((.((...))))) ..((((((......))))))	EPHB2	NM_017449	chr1:22909918-23114410	non-template	51	Intron_1.f	--	Yes	--	Yes	--	--	--	Yes	--	--	Yes
G4ST01022910237	chr1:22910238-22910288	+	GGGCGCAGGTGGCCGCGGGGAGGTGTGTCCGGCCGCGCCGGGAGGAGCGGG<br>(((.((...))))) ..((((((......))))))	EPHB2	NM_004442	chr1:22909918-23114410	non-template	51	Intron_1.f	--	Yes	--	Yes	--	--	--	Yes	--	--	Yes
G4ST01022910253	chr1:22910254-22910297	+	GGGGAGGTGTGTCCGGCCGCGCCGGGAGGAGCGGGCCCCGCGGG<br>..((((((......))))))	EPHB2	NM_017449	chr1:22909918-23114410	non-template	44	Intron_1.f	--	Yes	--	Yes	--	--	--	Yes	--	--	Yes
G4ST01022910253	chr1:22910254-22910297	+	GGGGAGGTGTGTCCGGCCGCGCCGGGAGGAGCGGGCCCCGCGGG<br>..((((((......))))))	EPHB2	NM_004442	chr1:22909918-23114410	non-template	44	Intron_1.f	--	Yes	--	Yes	--	--	--	Yes	--	--	Yes
G4ST01022910472	chr1:22910473-22910507	+	GGGTGGAGGGGCGCGGGCCCGGCCGTGGCGGGGGG<br>((((.......))))	EPHB2	NM_017449	chr1:22909918-23114410	non-template	35	Intron_1.f	--	Yes	--	Yes	--	--	--	Yes	--	--	--
G4ST01022910472	chr1:22910473-22910507	+	GGGTGGAGGGGCGCGGGCCCGGCCGTGGCGGGGGG<br>((((.......))))	EPHB2	NM_004442	chr1:22909918-23114410	non-template	35	Intron_1.f	--	Yes	--	Yes	--	--	--	Yes	--	--	--
G4ST01022910479	chr1:22910480-22910511	+	GGGGCGCGGGCCCGGCCGTGGCGGGGGGCGGG<br>((((.......))))	EPHB2	NM_017449	chr1:22909918-23114410	non-template	32	Intron_1.f	--	Yes	--	Yes	--	--	--	Yes	--	--	--
G4ST01022910479	chr1:22910480-22910511	+	GGGGCGCGGGCCCGGCCGTGGCGGGGGGCGGG<br>((((.......))))	EPHB2	NM_004442	chr1:22909918-23114410	non-template	32	Intron_1.f	--	Yes	--	Yes	--	--	--	Yes	--	--	--
G4ST01022910486	chr1:22910487-22910516	+	GGGCCCGGCCGTGGCGGGGGGCGGGGGGGG<br>((((.......))))	EPHB2	NM_017449	chr1:22909918-23114410	non-template	30	Intron_1.f	--	Yes	--	Yes	--	--	--	Yes	--	--	--
G4ST01022910486	chr1:22910487-22910516	+	GGGCCCGGCCGTGGCGGGGGGCGGGGGGGG<br>((((.......))))	EPHB2	NM_004442	chr1:22909918-23114410	non-template	30	Intron_1.f	--	Yes	--	Yes	--	--	--	Yes	--	--	--
G4ST01022910501	chr1:22910502-22910535	+	GGGGGGCGGGGGGGGCGCCGGCGCTCGGTCGGGG<br>.(((((.....))).))	EPHB2	NM_017449	chr1:22909918-23114410	non-template	34	Intron_1.f	--	Yes	--	Yes	--	--	--	Yes	--	--	--
G4ST01022910501	chr1:22910502-22910535	+	GGGGGGCGGGGGGGGCGCCGGCGCTCGGTCGGGG<br>.(((((.....))).))	EPHB2	NM_004442	chr1:22909918-23114410	non-template	34	Intron_1.f	--	Yes	--	Yes	--	--	--	Yes	--	--	--
G4ST01022965698	chr1:22965699-22965733	+	GGGGGGAGGAGAGGGCTAGCAGGGAAGGAGTTGGG<br>((....))	EPHB2	NM_017449	chr1:22909918-23114410	non-template	35	Intron_1.f	--	Yes	--	Yes	--	--	--	--	--	--	--
G4ST01022965698	chr1:22965699-22965733	+	GGGGGGAGGAGAGGGCTAGCAGGGAAGGAGTTGGG<br>((....))	EPHB2	NM_004442	chr1:22909918-23114410	non-template	35	Intron_1.f	--	Yes	--	Yes	--	--	--	--	--	--	--
G4ST01022966759	chr1:22966760-22966792	-	GGGGGAAGAGGGGCACCCTGGGCCAGTGGTGGG<br>((...)).	EPHB2	NM_017449	chr1:22909918-23114410	template	33	Intron_1.f	--	Yes	--	Yes	--	--	--	--	--	--	--
G4ST01022966759	chr1:22966760-22966792	-	GGGGGAAGAGGGGCACCCTGGGCCAGTGGTGGG<br>((...)).	EPHB2	NM_004442	chr1:22909918-23114410	template	33	Intron_1.f	--	Yes	--	Yes	--	--	--	--	--	--	--
G4ST01022974817	chr1:22974818-22974859	+	GGGTACGGTAGGGATAGGGCCAAAGTCACTCCCATGGCTGGG<br>....(((((......)))))	EPHB2	NM_017449	chr1:22909918-23114410	non-template	42	Intron_1.f	--	Yes	--	Yes	--	--	--	--	--	--	Yes
G4ST01022974817	chr1:22974818-22974859	+	GGGTACGGTAGGGATAGGGCCAAAGTCACTCCCATGGCTGGG<br>....(((((......)))))	EPHB2	NM_004442	chr1:22909918-23114410	non-template	42	Intron_1.f	--	Yes	--	Yes	--	--	--	--	--	--	Yes
G4ST01022996719	chr1:22996720-22996756	-	GGGACCCCAGACTGGAGGAGGCAGGGTGGGAGCAGGG<br>.(((((...))).))....	EPHB2	NM_017449	chr1:22909918-23114410	template	37	Intron_3	--	Yes	--	Yes	--	--	--	--	--	--	--
G4ST01022996719	chr1:22996720-22996756	-	GGGACCCCAGACTGGAGGAGGCAGGGTGGGAGCAGGG<br>.(((((...))).))....	EPHB2	NM_004442	chr1:22909918-23114410	template	37	Intron_3	--	Yes	--	Yes	--	--	--	--	--	--	--
G4ST01023038828	chr1:23038829-23038869	+	GGGCCACCAACCCTGGTGAATGGGCAAAGTTGGGACTTGGG<br>.(((((....)))))...	EPHB2	NM_017449	chr1:22909918-23114410	non-template	41	Intron_3	--	Yes	--	Yes	--	--	--	--	--	--	--
G4ST01023038828	chr1:23038829-23038869	+	GGGCCACCAACCCTGGTGAATGGGCAAAGTTGGGACTTGGG<br>.(((((....)))))...	EPHB2	NM_004442	chr1:22909918-23114410	non-template	41	Intron_3	--	Yes	--	Yes	--	--	--	--	--	--	--
G4ST01023051645	chr1:23051646-23051681	+	GGGAGGCCAGACCTGGGGCTGGGGGCAATGCGGGGG<br>(((.....))).	EPHB2	NM_017449	chr1:22909918-23114410	non-template	36	Intron_3	--	Yes	--	Yes	--	--	--	--	--	--	Yes
G4ST01023051645	chr1:23051646-23051681	+	GGGAGGCCAGACCTGGGGCTGGGGGCAATGCGGGGG<br>(((.....))).	EPHB2	NM_004442	chr1:22909918-23114410	non-template	36	Intron_3	--	Yes	--	Yes	--	--	--	--	--	--	Yes
G4ST01023081325	chr1:23081326-23081354	-	GGGGCACTGTGGGATCAGAGAGGGAAGGG<br>.((...))	EPHB2	NM_017449	chr1:22909918-23114410	template	29	Intron_5	--	Yes	--	Yes	--	--	--	--	--	--	--
G4ST01023081325	chr1:23081326-23081354	-	GGGGCACTGTGGGATCAGAGAGGGAAGGG<br>.((...))	EPHB2	NM_004442	chr1:22909918-23114410	template	29	Intron_5	--	Yes	--	Yes	--	--	--	--	--	--	--
G4ST01023109421	chr1:23109422-23109459	-	GGGACAATGCAGGAGTGCAGTGGGGTTGGGTGGATGGG<br>.((.((((....)))).))	EPHB2	NM_017449	chr1:22909918-23114410	template	38	Intron_13	--	Yes	--	Yes	--	--	--	--	--	--	Yes
G4ST01023109421	chr1:23109422-23109459	-	GGGACAATGCAGGAGTGCAGTGGGGTTGGGTGGATGGG<br>.((.((((....)))).))	EPHB2	NM_004442	chr1:22909918-23114410	template	38	Intron_13	--	Yes	--	Yes	--	--	--	--	--	--	Yes
G4ST01023280091	chr1:23280092-23280130	+	GGGTGACTGAAGACAGTCCTGGGCTTGGAGGGGCCTGGG<br>.(((((....)))))..	KDM1A	NM_015013	chr1:23218528-23282771	non-template	39	Intron_16	--	Yes	--	Yes	--	--	--	--	Yes	--	--
G4ST01023280091	chr1:23280092-23280130	+	GGGTGACTGAAGACAGTCCTGGGCTTGGAGGGGCCTGGG<br>.(((((....)))))..	KDM1A	NM_001009999	chr1:23218528-23282771	non-template	39	Intron_18	--	Yes	--	Yes	--	--	--	--	Yes	--	--
G4ST01023307034	chr1:23307035-23307085	-	GGGTAGCTCAGAATGAGTGGGAAATGGGTTCTCGTCATAGCAGTGACTGGG<br>...((((...)))). .....(((((....))))).	LUZP1	NM_001142546	chr1:23283103-23367938	non-template	51	Intron_2	--	Yes	--	Yes	--	--	--	--	--	--	--
G4ST01023307034	chr1:23307035-23307085	-	GGGTAGCTCAGAATGAGTGGGAAATGGGTTCTCGTCATAGCAGTGACTGGG<br>...((((...)))). .....(((((....))))).	LUZP1	NM_033631	chr1:23283103-23367938	non-template	51	Intron_3	--	Yes	--	Yes	--	--	--	--	--	--	--
G4ST01023367793	chr1:23367794-23367819	-	GGGAGCGCGGCGCGGGGAGGGGAGGG<br>..(((...)))	LUZP1	NM_001142546	chr1:23283103-23367938	non-template	26	Intron_1.f	--	Yes	--	Yes	--	--	--	Yes	Yes	Yes	Yes
G4ST01023367793	chr1:23367794-23367819	-	GGGAGCGCGGCGCGGGGAGGGGAGGG<br>..(((...)))	LUZP1	NM_033631	chr1:23283103-23367938	non-template	26	Intron_1.f	--	Yes	--	Yes	--	--	--	Yes	Yes	Yes	Yes
G4ST01023543821	chr1:23543822-23543857	-	GGGAGGACTCGGGGGCTAAGGGCGGCCGGTGGCGGG<br>..(((...)))	HNRNPR	NM_001102397	chr1:23508863-23543440	non-template	-381	upstream	Yes	--	--	--	--	--	--	--	Yes	Yes	Yes
G4ST01023543821	chr1:23543822-23543857	-	GGGAGGACTCGGGGGCTAAGGGCGGCCGGTGGCGGG<br>..(((...)))	HNRNPR	NM_001102398	chr1:23508863-23543440	non-template	-381	upstream	Yes	--	--	--	--	--	--	--	Yes	Yes	Yes
G4ST01023543821	chr1:23543822-23543857	-	GGGAGGACTCGGGGGCTAAGGGCGGCCGGTGGCGGG<br>..(((...)))	HNRNPR	NM_001102399	chr1:23508863-23543440	non-template	-381	upstream	Yes	--	--	--	--	--	--	--	Yes	Yes	Yes

G4ST01023543821	chr1:23543822-23543857	-	GGGAGGACTCGGGGGCTAAGGGCGGCCGGTGGCGGG<br>..(((...)))	HNRNPR	NM_005826	chr1:23508863-23543440	non-template	-381	upstream	Yes	--	--	--	--	--	--	--	Yes	Yes	Yes
G4ST01023618115	chr1:23618116-23618149	-	GGGGGTGGGAAGCCTTGGGACAGTGCCTGGGGGG<br>.(((...)))..	TCEA3	NM_003196	chr1:23580142-23623848	non-template	34	Intron_2	--	Yes	--	Yes	--	--	--	--	--	--	Yes
G4ST01023623536	chr1:23623537-23623575	-	GGGGACGTACTGGCGGGGCGCCATGGGGGCGCGTACGGG<br>..(((....)))	TCEA3	NM_003196	chr1:23580142-23623848	non-template	39	Intron_1.f	--	Yes	--	Yes	--	--	--	--	Yes	--	Yes
G4ST01023623546	chr1:23623547-23623580	-	GGGGTGGGGACGTACTGGCGGGGCGCCATGGGGG<br>..(((....)))	TCEA3	NM_003196	chr1:23580142-23623848	non-template	34	Intron_1.f	--	Yes	--	Yes	--	--	--	--	Yes	--	Yes
G4ST01023633497	chr1:23633498-23633530	+	GGGGATCAAAGTCAGGGGAAAAAGTGGGAGGGG<br>(((....)))..	ASAP3	NM_017707	chr1:23627643-23683337	template	33	Intron_18	--	Yes	--	Yes	--	--	--	--	--	--	Yes
G4ST01023633497	chr1:23633498-23633530	+	GGGGATCAAAGTCAGGGGAAAAAGTGGGAGGGG<br>(((....)))..	ASAP3	NM_001143778	chr1:23627643-23683337	template	33	Intron_17	--	Yes	--	Yes	--	--	--	--	--	--	Yes
G4ST01023635880	chr1:23635881-23635908	-	GGGTGGGACCTGGCCGAGGTGGGCTGGG<br>((((.....))))	ASAP3	NM_017707	chr1:23627643-23683337	non-template	28	Intron_15	--	Yes	--	Yes	--	--	--	Yes	--	--	--
G4ST01023635880	chr1:23635881-23635908	-	GGGTGGGACCTGGCCGAGGTGGGCTGGG<br>((((.....))))	ASAP3	NM_001143778	chr1:23627643-23683337	non-template	28	Intron_14	--	Yes	--	Yes	--	--	--	Yes	--	--	--
G4ST01023658445	chr1:23658446-23658480	-	GGGCAGTTTTCAGAAAATGGGTGTCATGGGATGGG<br>..((((.....))))	ASAP3	NM_017707	chr1:23627643-23683337	non-template	35	Intron_1.f	--	Yes	--	Yes	--	--	--	Yes	Yes	--	--
G4ST01023658445	chr1:23658446-23658480	-	GGGCAGTTTTCAGAAAATGGGTGTCATGGGATGGG<br>..((((.....))))	ASAP3	NM_001143778	chr1:23627643-23683337	non-template	35	Intron_1.f	--	Yes	--	Yes	--	--	--	Yes	Yes	--	--
G4ST01023658450	chr1:23658451-23658484	-	GGGAGGGCAGTTTTCAGAAAATGGGTGTCATGGG<br>..((((.....))))	ASAP3	NM_017707	chr1:23627643-23683337	non-template	34	Intron_1.f	--	Yes	--	Yes	--	--	--	Yes	Yes	--	--
G4ST01023658450	chr1:23658451-23658484	-	GGGAGGGCAGTTTTCAGAAAATGGGTGTCATGGG<br>..((((.....))))	ASAP3	NM_001143778	chr1:23627643-23683337	non-template	34	Intron_1.f	--	Yes	--	Yes	--	--	--	Yes	Yes	--	--
G4ST01023678039	chr1:23678040-23678073	-	GGGAGGGGGCGCTTCTCAGAGAAGAGGGGCAGGG<br>....(((((...)))))..	ASAP3	NM_017707	chr1:23627643-23683337	non-template	34	Intron_1.f	--	Yes	--	Yes	--	--	--	--	Yes	Yes	--
G4ST01023678039	chr1:23678040-23678073	-	GGGAGGGGGCGCTTCTCAGAGAAGAGGGGCAGGG<br>....(((((...)))))..	ASAP3	NM_001143778	chr1:23627643-23683337	non-template	34	Intron_1.f	--	Yes	--	Yes	--	--	--	--	Yes	Yes	--
G4ST01023683042	chr1:23683043-23683068	-	GGGAGGGGTCGGGGGTCGGCGCCGGG<br>(((....)))	ASAP3	NM_017707	chr1:23627643-23683337	non-template	26	Intron_1.f	--	Yes	--	Yes	--	--	--	--	Yes	Yes	Yes
G4ST01023683042	chr1:23683043-23683068	-	GGGAGGGGTCGGGGGTCGGCGCCGGG<br>(((....)))	ASAP3	NM_001143778	chr1:23627643-23683337	non-template	26	Intron_1.f	--	Yes	--	Yes	--	--	--	--	Yes	Yes	Yes
G4ST01023727789	chr1:23727790-23727829	+	GGGAGGGAGGTACAGACAGATGTACTTGGGCTCCTCTGGG<br>..(((((......)))))..	E2F2	NM_004091	chr1:23705507-23730299	template	40	Intron_1.f	--	Yes	--	Yes	--	--	--	Yes	Yes	Yes	--
G4ST01023727793	chr1:23727794-23727833	+	GGGAGGTACAGACAGATGTACTTGGGCTCCTCTGGGAGGG<br>..(((((......)))))..	E2F2	NM_004091	chr1:23705507-23730299	template	40	Intron_1.f	--	Yes	--	Yes	--	--	--	Yes	Yes	Yes	--
G4ST01023990213	chr1:23990214-23990261	+	GGGTTCCCGGGACTCGAACGGAAGTTCCGGCGGGGGCGGCCGAGGGGG<br>...((..((((...)))).)).	LYPLA2	NM_007260	chr1:23990233-23994616	non-template	29	upstream-Exon_1.f	Yes	Yes	Yes	--	--	Yes	--	Yes	Yes	Yes	--
G4ST01023990353	chr1:23990354-23990385	+	GGGCGTGTAGGGGGAGGTCGGCCTCGGGAGGG<br>.((((....))))	LYPLA2	NM_007260	chr1:23990233-23994616	non-template	32	Intron_1.f	--	Yes	--	Yes	--	--	--	--	Yes	Yes	--
G4ST01023991158	chr1:23991159-23991189	+	GGGGACATGGGCAGCCCCCTGGGGGTCTGGG<br>(((....))).	LYPLA2	NM_007260	chr1:23990233-23994616	non-template	31	Intron_1.f	--	Yes	--	Yes	--	--	--	--	Yes	--	--
G4ST01023991166	chr1:23991167-23991199	+	GGGCAGCCCCCTGGGGGTCTGGGAATGGATGGG<br>(((....))).	LYPLA2	NM_007260	chr1:23990233-23994616	non-template	33	Intron_1.f	--	Yes	--	Yes	--	--	--	--	Yes	--	--
G4ST01023999353	chr1:23999354-23999387	-	GGGGTCCGCGCCAGGCGCGGGGCGGGGGCGGGGG<br>...(((((...)))))	GALE	NM_001127621	chr1:23994676-23998647	non-template	-706	upstream	Yes	--	--	--	--	--	--	Yes	Yes	Yes	--
G4ST01023999353	chr1:23999354-23999387	-	GGGGTCCGCGCCAGGCGCGGGGCGGGGGCGGGGG<br>...(((((...)))))	GALE	NM_000403	chr1:23994676-23999621	non-template	34	Intron_1.f	--	Yes	--	Yes	--	--	--	Yes	Yes	Yes	--
G4ST01023999353	chr1:23999354-23999387	-	GGGGTCCGCGCCAGGCGCGGGGCGGGGGCGGGGG<br>...(((((...)))))	GALE	NM_001008216	chr1:23994676-23999881	non-template	34	Intron_1.f	--	Yes	--	Yes	--	--	--	Yes	Yes	Yes	--
G4ST01024004028	chr1:24004029-24004063	-	GGGATAGTGGGAGGTGACTGACCTGGGAAGATGGG<br>((((.....))))	HMGCL	NM_000191	chr1:24000954-24024536	non-template	35	Intron_7	--	Yes	--	Yes	--	--	--	--	--	--	--
G4ST01024004028	chr1:24004029-24004063	-	GGGATAGTGGGAGGTGACTGACCTGGGAAGATGGG<br>((((.....))))	HMGCL	NM_001166059	chr1:24000954-24024536	non-template	35	Intron_5	--	Yes	--	Yes	--	--	--	--	--	--	--
G4ST01024016898	chr1:24016899-24016938	+	GGGCAGAGATGACTAACCCTCTGGGGAGTATGGGATGGGG<br>(((((..........)))))	HMGCL	NM_000191	chr1:24000954-24024536	template	40	Intron_2	--	Yes	--	Yes	--	--	--	--	Yes	--	--
G4ST01024016898	chr1:24016899-24016938	+	GGGCAGAGATGACTAACCCTCTGGGGAGTATGGGATGGGG<br>(((((..........)))))	HMGCL	NM_001166059	chr1:24000954-24024536	template	40	Intron_2	--	Yes	--	Yes	--	--	--	--	Yes	--	--
G4ST01024179007	chr1:24179008-24179044	+	GGGAGAAAGGGGCTGCAAACTGCGGAGGGGGAGGGGG<br>.(((((...)))))...	SRSF10	NM_054016	chr1:24168160-24179540	template	37	Intron_1.f	--	Yes	--	Yes	--	--	--	Yes	Yes	Yes	--
G4ST01024179007	chr1:24179008-24179044	+	GGGAGAAAGGGGCTGCAAACTGCGGAGGGGGAGGGGG<br>.(((((...)))))...	SRSF10	NM_001191005	chr1:24168160-24179540	template	37	Intron_1.f	--	Yes	--	Yes	--	--	--	Yes	Yes	Yes	--
G4ST01024179007	chr1:24179008-24179044	+	GGGAGAAAGGGGCTGCAAACTGCGGAGGGGGAGGGGG<br>.(((((...)))))...	SRSF10	NM_001191007	chr1:24165524-24179540	template	37	Intron_1.f	--	Yes	--	Yes	--	--	--	Yes	Yes	Yes	--
G4ST01024179007	chr1:24179008-24179044	+	GGGAGAAAGGGGCTGCAAACTGCGGAGGGGGAGGGGG<br>.(((((...)))))...	SRSF10	NR_034035	chr1:24165524-24179540	template	37	Intron_1.f	--	Yes	--	Yes	--	--	--	Yes	Yes	Yes	--
G4ST01024179007	chr1:24179008-24179044	+	GGGAGAAAGGGGCTGCAAACTGCGGAGGGGGAGGGGG<br>.(((((...)))))...	SRSF10	NM_001191009	chr1:24165524-24179540	template	37	Intron_1.f	--	Yes	--	Yes	--	--	--	Yes	Yes	Yes	--
G4ST01024179007	chr1:24179008-24179044	+	GGGAGAAAGGGGCTGCAAACTGCGGAGGGGGAGGGGG<br>.(((((...)))))...	SRSF10	NM_006625	chr1:24165524-24179540	template	37	Intron_1.f	--	Yes	--	Yes	--	--	--	Yes	Yes	Yes	--
G4ST01024179007	chr1:24179008-24179044	+	GGGAGAAAGGGGCTGCAAACTGCGGAGGGGGAGGGGG<br>.(((((...)))))...	SRSF10	NM_001191006	chr1:24165524-24179540	template	37	Intron_1.f	--	Yes	--	Yes	--	--	--	Yes	Yes	Yes	--
G4ST01024261863	chr1:24261864-24261894	-	GGGCAGCAGCTGGGAACTGGGGGAGAAAGGG<br>.((...))	MYOM3	NM_152372	chr1:24255118-24311252	non-template	31	Intron_32	--	Yes	--	Yes	--	--	--	--	--	--	Yes
G4ST01024263113	chr1:24263114-24263156	-	GGGTGACGGTAGGAGCTGGCGATGGGTCAGGGGCTGTGGAGGG<br>...((.(((...))).))..	MYOM3	NM_152372	chr1:24255118-24311252	non-template	43	Exon_30-Intron_30	--	Yes	Yes	Yes	Yes	--	--	--	--	--	--
G4ST01024281452	chr1:24281453-24281485	-	GGGATGGGGCCTGAGCAGCTCTGGGGGGTGGGG<br>.((.(((...))).)).	MYOM3	NM_152372	chr1:24255118-24311252	non-template	33	Intron_17	--	Yes	--	Yes	--	--	--	--	--	--	--
G4ST01024288246	chr1:24288247-24288284	-	GGGGCCAAGCACTTCTTGGTGGGACTATGTTGGGAGGG<br>.(((((.....))))). ((...)).	MYOM3	NM_152372	chr1:24255118-24311252	non-template	38	Intron_14	--	Yes	--	Yes	--	--	--	Yes	--	--	--
G4ST01024290053	chr1:24290054-24290094	-	GGGGGGGCCTGTCTCCAACAGGTGCCAGGGCGAGTCTGGGG<br>(((((.....))))).....	MYOM3	NM_152372	chr1:24255118-24311252	non-template	41	Intron_11-Exon_12	--	Yes	Yes	Yes	Yes	--	--	--	--	--	Yes
G4ST01024290064	chr1:24290065-24290104	-	GGGGCTGAAAGGGGGGGCCTGTCTCCAACAGGTGCCAGGG<br>(((((.....))))).....	MYOM3	NM_152372	chr1:24255118-24311252	non-template	40	Intron_11-Exon_12	--	Yes	Yes	Yes	Yes	--	--	--	--	--	Yes
G4ST01024292692	chr1:24292693-24292729	+	GGGGACTCAGAAGTGGAGTCCAGGGCTCAGGGGCGGG<br>(((((.......)))))..	MYOM3	NM_152372	chr1:24255118-24311252	template	37	Intron_9	--	Yes	--	Yes	--	--	--	--	--	--	--
G4ST01024297127	chr1:24297128-24297165	+	GGGCTCCATGGGAAACACAAGTCCCCTGTGTGGGTGGG<br>..(((((.......)))))	MYOM3	NM_152372	chr1:24255118-24311252	template	38	Intron_6	--	Yes	--	Yes	--	--	--	--	--	--	--
G4ST01024307501	chr1:24307502-24307531	+	GGGGGCCCTGGGAACAGGGCAGTGGCTGGG<br>.((...))	MYOM3	NM_152372	chr1:24255118-24311252	template	30	Intron_2	--	Yes	--	Yes	--	--	--	--	Yes	--	--
G4ST01024307510	chr1:24307511-24307536	+	GGGAACAGGGCAGTGGCTGGGCGGGG<br>.((...))	MYOM3	NM_152372	chr1:24255118-24311252	template	26	Intron_2	--	Yes	--	Yes	--	--	--	--	Yes	--	--
G4ST01024358069	chr1:24358070-24358098	-	GGGGAGGGGTTGAGAGTAAGGGAGTGGGG<br>.(((....)))	IL28RA	NM_170743	chr1:24353234-24386352	non-template	29	Intron_6.l	--	Yes	--	Yes	--	--	--	--	--	--	--
G4ST01024358069	chr1:24358070-24358098	-	GGGGAGGGGTTGAGAGTAAGGGAGTGGGG<br>.(((....)))	IL28RA	NM_173064	chr1:24353234-24386352	non-template	29	Intron_6.l	--	Yes	--	Yes	--	--	--	--	--	--	--
G4ST01024358069	chr1:24358070-24358098	-	GGGGAGGGGTTGAGAGTAAGGGAGTGGGG<br>.(((....)))	IL28RA	NM_173065	chr1:24353234-24386352	non-template	29	Intron_5.l	--	Yes	--	Yes	--	--	--	--	--	--	--
G4ST01024405480	chr1:24405481-24405517	-	GGGGTAGGAACTGCCTGTGCCTGGGGCTGGGGAAGGG<br>..(((.((.....)).))).	LOC284632	NR_027087	chr1:24399317-24410767	template	37	Intron_2.l	--	Yes	--	Yes	--	--	--	--	--	--	Yes

G4ST01024405485	chr1:24405486-24405523	-	GGGGAAGGGGTAGGAACTGCCTGTGCCTGGGGCTGGGG<br>..(((.((.....)).))).	LOC284632	NR_027087	chr1:24399317-24410767	template	38	Intron_2.l	--	Yes	--	Yes	--	--	--	--	--	--	Yes
G4ST01024517069	chr1:24517070-24517100	-	GGGGTCAGGGCTCTGGAGAGGGGGTACTGGG<br>(((....))).	GRHL3	NM_001195010	chr1:24518643-24554395	template	-1542	upstream	Yes	--	--	--	--	--	--	--	--	--	--
G4ST01024517069	chr1:24517070-24517100	-	GGGGTCAGGGCTCTGGAGAGGGGGTACTGGG<br>(((....))).	GRHL3	NM_198174	chr1:24518399-24563557	template	-1298	upstream	Yes	--	--	--	--	--	--	--	--	--	--
G4ST01024517069	chr1:24517070-24517100	-	GGGGTCAGGGCTCTGGAGAGGGGGTACTGGG<br>(((....))).	GRHL3	NM_198173	chr1:24518399-24554395	template	-1298	upstream	Yes	--	--	--	--	--	--	--	--	--	--
G4ST01024517076	chr1:24517077-24517105	-	GGGGTGGGGTCAGGGCTCTGGAGAGGGGG<br>(((....))).	GRHL3	NM_001195010	chr1:24518643-24554395	template	-1537	upstream	Yes	--	--	--	--	--	--	--	--	--	--
G4ST01024517076	chr1:24517077-24517105	-	GGGGTGGGGTCAGGGCTCTGGAGAGGGGG<br>(((....))).	GRHL3	NM_198174	chr1:24518399-24563557	template	-1293	upstream	Yes	--	--	--	--	--	--	--	--	--	--
G4ST01024517076	chr1:24517077-24517105	-	GGGGTGGGGTCAGGGCTCTGGAGAGGGGG<br>(((....))).	GRHL3	NM_198173	chr1:24518399-24554395	template	-1293	upstream	Yes	--	--	--	--	--	--	--	--	--	--
G4ST01024538850	chr1:24538851-24538875	+	GGGCAGTGGCTGGGATGGGACTGGG<br>.((...))	GRHL3	NM_001195010	chr1:24518643-24554395	non-template	25	Intron_8	--	Yes	--	Yes	--	--	--	--	--	--	--
G4ST01024538850	chr1:24538851-24538875	+	GGGCAGTGGCTGGGATGGGACTGGG<br>.((...))	GRHL3	NM_198174	chr1:24518399-24563557	non-template	25	Intron_8	--	Yes	--	Yes	--	--	--	--	--	--	--
G4ST01024538850	chr1:24538851-24538875	+	GGGCAGTGGCTGGGATGGGACTGGG<br>.((...))	GRHL3	NM_021180	chr1:24522117-24554395	non-template	25	Intron_8	--	Yes	--	Yes	--	--	--	--	--	--	--
G4ST01024538850	chr1:24538851-24538875	+	GGGCAGTGGCTGGGATGGGACTGGG<br>.((...))	GRHL3	NM_198173	chr1:24518399-24554395	non-template	25	Intron_8	--	Yes	--	Yes	--	--	--	--	--	--	--
G4ST01024550660	chr1:24550661-24550702	-	GGGAGGCCTGCCACAGGTGGGGCTGGAGCCAGGGGGCATGGG<br>...((((...)))).. .(((....))).	GRHL3	NM_001195010	chr1:24518643-24554395	template	42	Intron_15.l	--	Yes	--	Yes	--	--	--	Yes	Yes	--	--
G4ST01024550660	chr1:24550661-24550702	-	GGGAGGCCTGCCACAGGTGGGGCTGGAGCCAGGGGGCATGGG<br>...((((...)))).. .(((....))).	GRHL3	NM_198174	chr1:24518399-24563557	template	42	Intron_15.l	--	Yes	--	Yes	--	--	--	Yes	Yes	--	--
G4ST01024550660	chr1:24550661-24550702	-	GGGAGGCCTGCCACAGGTGGGGCTGGAGCCAGGGGGCATGGG<br>...((((...)))).. .(((....))).	GRHL3	NM_021180	chr1:24522117-24554395	template	42	Intron_15.l	--	Yes	--	Yes	--	--	--	Yes	Yes	--	--
G4ST01024550660	chr1:24550661-24550702	-	GGGAGGCCTGCCACAGGTGGGGCTGGAGCCAGGGGGCATGGG<br>...((((...)))).. .(((....))).	GRHL3	NM_198173	chr1:24518399-24554395	template	42	Intron_15.l	--	Yes	--	Yes	--	--	--	Yes	Yes	--	--
G4ST01024553215	chr1:24553216-24553258	+	GGGGGCCAGCAGGCCTGGGTTGGGTTGTGGCTCGGCCACTGGG<br>((((....)))). ..(((((...))))).	GRHL3	NM_001195010	chr1:24518643-24554395	non-template	43	Intron_15.l	--	Yes	--	Yes	--	--	--	--	--	--	--
G4ST01024553215	chr1:24553216-24553258	+	GGGGGCCAGCAGGCCTGGGTTGGGTTGTGGCTCGGCCACTGGG<br>((((....)))). ..(((((...))))).	GRHL3	NM_198174	chr1:24518399-24563557	non-template	43	Intron_15.l	--	Yes	--	Yes	--	--	--	--	--	--	--
G4ST01024553215	chr1:24553216-24553258	+	GGGGGCCAGCAGGCCTGGGTTGGGTTGTGGCTCGGCCACTGGG<br>((((....)))). ..(((((...))))).	GRHL3	NM_021180	chr1:24522117-24554395	non-template	43	Intron_15.l	--	Yes	--	Yes	--	--	--	--	--	--	--
G4ST01024553215	chr1:24553216-24553258	+	GGGGGCCAGCAGGCCTGGGTTGGGTTGTGGCTCGGCCACTGGG<br>((((....)))). ..(((((...))))).	GRHL3	NM_198173	chr1:24518399-24554395	non-template	43	Intron_15.l	--	Yes	--	Yes	--	--	--	--	--	--	--
G4ST01024755147	chr1:24755148-24755178	+	GGGGGCGGGATCCCAGGGTGGCAGCGCCGGG<br>.(((...)))	C1orf130	NM_001010980	chr1:24755154-24808405	non-template	25	upstream-Exon_1.f	Yes	Yes	Yes	--	--	Yes	--	Yes	Yes	--	Yes
G4ST01024755153	chr1:24755154-24755189	+	GGGATCCCAGGGTGGCAGCGCCGGGCGGCGGCGGGG<br>.(((...)))	C1orf130	NM_001010980	chr1:24755154-24808405	non-template	36	Exon_1.f	--	Yes	Yes	--	--	Yes	--	Yes	Yes	--	Yes
G4ST01024791131	chr1:24791132-24791162	+	GGGCAGGGACTGGGGAGGCCAATGCCTGGGG<br>.((((....)))).	C1orf130	NM_001010980	chr1:24755154-24808405	non-template	31	Intron_1.f	--	Yes	--	Yes	--	--	--	Yes	--	--	--
G4ST01024840776	chr1:24840777-24840803	+	GGGAGCCAGGGAAGACCCTGGGAAGGG<br>.((...))	SRRM1	NM_005839	chr1:24842181-24872359	non-template	-1377	upstream	Yes	--	--	--	--	--	--	Yes	Yes	Yes	--
G4ST01025003620	chr1:25003621-25003649	+	GGGTGGGTGGGGGGATGTGTGTACGTGGG<br>...((((....))))	CLIC4	NM_013943	chr1:24944347-25043402	non-template	29	Intron_2	--	Yes	--	Yes	--	--	--	--	--	--	--
G4ST01025003624	chr1:25003625-25003653	+	GGGTGGGGGGATGTGTGTACGTGGGTGGG<br>...((((....))))	CLIC4	NM_013943	chr1:24944347-25043402	non-template	29	Intron_2	--	Yes	--	Yes	--	--	--	--	--	--	--
G4ST01025003628	chr1:25003629-25003658	+	GGGGGGATGTGTGTACGTGGGTGGGTGGGG<br>...((((....))))	CLIC4	NM_013943	chr1:24944347-25043402	non-template	30	Intron_2	--	Yes	--	Yes	--	--	--	--	--	--	--
G4ST01025032872	chr1:25032873-25032914	-	GGGGGGGGGGTCGGCCAGCCGCCCCGGCCGGGAGGTGAGGGG<br>(((.(((....)))))).....	CLIC4	NM_013943	chr1:24944347-25043402	template	42	Intron_4	--	Yes	--	Yes	--	--	--	--	--	--	Yes
G4ST01025032882	chr1:25032883-25032918	-	GGGGGGGGGGGGGGTCGGCCAGCCGCCCCGGCCGGG<br>(((.(((....)))))).....	CLIC4	NM_013943	chr1:24944347-25043402	template	36	Intron_4	--	Yes	--	Yes	--	--	--	--	--	--	Yes
G4ST01025103481	chr1:25103482-25103516	+	GGGGACAGCAGTCTGGGTGGCGGGGGCCTTCTGGG<br>(((....))).	RUNX3	NM_004350	chr1:25098589-25129357	template	35	Intron_4.l	--	Yes	--	Yes	--	--	--	--	--	Yes	Yes
G4ST01025103481	chr1:25103482-25103516	+	GGGGACAGCAGTCTGGGTGGCGGGGGCCTTCTGGG<br>(((....))).	RUNX3	NM_001031680	chr1:25098589-25164088	template	35	Intron_5.l	--	Yes	--	Yes	--	--	--	--	--	Yes	Yes
G4ST01025104102	chr1:25104103-25104133	+	GGGCGGCTGCGGCTGGGGGCTGGGGCTGGGG<br>((((....)))).	RUNX3	NM_004350	chr1:25098589-25129357	template	31	Intron_4.l	--	Yes	--	Yes	--	--	--	--	Yes	Yes	Yes
G4ST01025104102	chr1:25104103-25104133	+	GGGCGGCTGCGGCTGGGGGCTGGGGCTGGGG<br>((((....)))).	RUNX3	NM_001031680	chr1:25098589-25164088	template	31	Intron_5.l	--	Yes	--	Yes	--	--	--	--	Yes	Yes	Yes
G4ST01025104234	chr1:25104235-25104269	+	GGGGGATCCCCAGGAGGGAGTCCGAGGGCAGAGGG<br>...(((...)))	RUNX3	NM_004350	chr1:25098589-25129357	template	35	Intron_4.l	--	Yes	--	Yes	--	--	--	Yes	Yes	Yes	Yes
G4ST01025104234	chr1:25104235-25104269	+	GGGGGATCCCCAGGAGGGAGTCCGAGGGCAGAGGG<br>...(((...)))	RUNX3	NM_001031680	chr1:25098589-25164088	template	35	Intron_5.l	--	Yes	--	Yes	--	--	--	Yes	Yes	Yes	Yes
G4ST01025105501	chr1:25105502-25105538	-	GGGCTAAGTGGGGTGCCAAGGGTGCTTGGTGGCCGGG<br>.(((....))).	RUNX3	NM_004350	chr1:25098589-25129357	non-template	37	Intron_4.l	--	Yes	--	Yes	--	--	--	--	--	Yes	--
G4ST01025105501	chr1:25105502-25105538	-	GGGCTAAGTGGGGTGCCAAGGGTGCTTGGTGGCCGGG<br>.(((....))).	RUNX3	NM_001031680	chr1:25098589-25164088	non-template	37	Intron_5.l	--	Yes	--	Yes	--	--	--	--	--	Yes	--
G4ST01025113616	chr1:25113617-25113657	+	GGGGGCCAGCGGGCAGGGAGGCGAGCGGTTCCCTCCCAGGG<br>.((.(((.......))))).	RUNX3	NM_004350	chr1:25098589-25129357	template	41	Intron_3	--	Yes	--	Yes	--	--	--	Yes	Yes	Yes	Yes
G4ST01025113616	chr1:25113617-25113657	+	GGGGGCCAGCGGGCAGGGAGGCGAGCGGTTCCCTCCCAGGG<br>.((.(((.......))))).	RUNX3	NM_001031680	chr1:25098589-25164088	template	41	Intron_4	--	Yes	--	Yes	--	--	--	Yes	Yes	Yes	Yes
G4ST01025120901	chr1:25120902-25120931	-	GGGCCCGGGGCCCTGTGGCAGGGCACTGGG<br>(((....))).	RUNX3	NM_004350	chr1:25098589-25129357	non-template	30	Intron_2	--	Yes	--	Yes	--	--	--	--	--	Yes	--
G4ST01025120901	chr1:25120902-25120931	-	GGGCCCGGGGCCCTGTGGCAGGGCACTGGG<br>(((....))).	RUNX3	NM_001031680	chr1:25098589-25164088	non-template	30	Intron_3	--	Yes	--	Yes	--	--	--	--	--	Yes	--
G4ST01025124176	chr1:25124177-25124209	-	GGGACACTTTGGGAGTTAAGACTGGGGGTTGGG<br>(((....)))..	RUNX3	NM_004350	chr1:25098589-25129357	non-template	33	Intron_2	--	Yes	--	Yes	--	--	--	--	Yes	Yes	--
G4ST01025124176	chr1:25124177-25124209	-	GGGACACTTTGGGAGTTAAGACTGGGGGTTGGG<br>(((....)))..	RUNX3	NM_001031680	chr1:25098589-25164088	non-template	33	Intron_3	--	Yes	--	Yes	--	--	--	--	Yes	Yes	--
G4ST01025127912	chr1:25127913-25127943	-	GGGCAGGGCAGGAAGGGCCGGCGGCTGGGGG<br>(((.....)))	RUNX3	NM_004350	chr1:25098589-25129357	non-template	31	Intron_1.f	--	Yes	--	Yes	--	--	--	Yes	Yes	Yes	--
G4ST01025127912	chr1:25127913-25127943	-	GGGCAGGGCAGGAAGGGCCGGCGGCTGGGGG<br>(((.....)))	RUNX3	NM_001031680	chr1:25098589-25164088	non-template	31	Intron_2	--	Yes	--	Yes	--	--	--	Yes	Yes	Yes	--
G4ST01025129277	chr1:25129278-25129307	-	GGGGCGGCCGCGGGCGAGCTTCGGGGCGGG<br>(((...)))	RUNX3	NM_004350	chr1:25098589-25129357	non-template	30	Exon_1.f	--	Yes	Yes	--	--	Yes	--	--	Yes	Yes	--
G4ST01025129277	chr1:25129278-25129307	-	GGGGCGGCCGCGGGCGAGCTTCGGGGCGGG<br>(((...)))	RUNX3	NM_001031680	chr1:25098589-25164088	non-template	30	Intron_1.f	--	Yes	--	Yes	--	--	--	--	Yes	Yes	--
G4ST01025142305	chr1:25142306-25142344	+	GGGGGAGGCCAGGGCTTTGGGAGCATGCTGGCAGCAGGG<br>....((((...))))	RUNX3	NM_001031680	chr1:25098589-25164088	template	39	Intron_1.f	--	Yes	--	Yes	--	--	--	--	--	Yes	--
G4ST01025142316	chr1:25142317-25142354	+	GGGCTTTGGGAGCATGCTGGCAGCAGGGTGGAGGGGGG<br>....((((...))))	RUNX3	NM_001031680	chr1:25098589-25164088	template	38	Intron_1.f	--	Yes	--	Yes	--	--	--	--	--	Yes	--
G4ST01025159985	chr1:25159986-25160031	-	GGGCACAGGGGCCCCCTGGGGTCTACCCCCAGGCAGGACAAGGGGG<br>((((.((....))..))))....	RUNX3	NM_001031680	chr1:25098589-25164088	non-template	46	Intron_1.f	--	Yes	--	Yes	--	--	--	--	--	Yes	--
G4ST01025164572	chr1:25164573-25164605	-	GGGCTCTGGGCACTCGGTGAGGGGCCCGCGGGG<br>(((...)))..	RUNX3	NM_001031680	chr1:25098589-25164088	non-template	-484	upstream	Yes	--	--	--	--	--	--	--	Yes	Yes	--

G4ST01025479084	chr1:25479085-25479131	+	GGGTGGAAATGGGCCTGGAGCCCAGGAGAAGTGGGAGGATGAGGGGG<br>(((((...)))))......	RHD	NM_001127691	chr1:25471568-25529523	non-template	47	Intron_1.f	--	Yes	--	Yes	--	--	--	--	Yes	Yes	--
G4ST01025479084	chr1:25479085-25479131	+	GGGTGGAAATGGGCCTGGAGCCCAGGAGAAGTGGGAGGATGAGGGGG<br>(((((...)))))......	RHD	NM_016124	chr1:25471568-25529523	non-template	47	Intron_1.f	--	Yes	--	Yes	--	--	--	--	Yes	Yes	--
G4ST01025479094	chr1:25479095-25479138	+	GGGCCTGGAGCCCAGGAGAAGTGGGAGGATGAGGGGGCAGGGGG<br>(((((...)))))......	RHD	NM_001127691	chr1:25471568-25529523	non-template	44	Intron_1.f	--	Yes	--	Yes	--	--	--	--	Yes	Yes	--
G4ST01025479094	chr1:25479095-25479138	+	GGGCCTGGAGCCCAGGAGAAGTGGGAGGATGAGGGGGCAGGGGG<br>(((((...)))))......	RHD	NM_016124	chr1:25471568-25529523	non-template	44	Intron_1.f	--	Yes	--	Yes	--	--	--	--	Yes	Yes	--
G4ST01025482951	chr1:25482952-25482981	-	GGGCAGGGAGGGGCCCAGCAAAGCGGAGGG<br>..((.((...)))).	RHD	NM_001127691	chr1:25471568-25529523	template	30	Intron_1.f	--	Yes	--	Yes	--	--	--	--	--	--	--
G4ST01025482951	chr1:25482952-25482981	-	GGGCAGGGAGGGGCCCAGCAAAGCGGAGGG<br>..((.((...)))).	RHD	NM_016124	chr1:25471568-25529523	template	30	Intron_1.f	--	Yes	--	Yes	--	--	--	--	--	--	--
G4ST01025489891	chr1:25489892-25489932	+	GGGACCTGGGAGAAAAGGGCCAAAAGCTCCATTTGGTGGGG<br>(((((.......)))))..	RHD	NM_001127691	chr1:25471568-25529523	non-template	41	Intron_3	--	Yes	--	Yes	--	--	--	--	--	--	Yes
G4ST01025489891	chr1:25489892-25489932	+	GGGACCTGGGAGAAAAGGGCCAAAAGCTCCATTTGGTGGGG<br>(((((.......)))))..	RHD	NM_016124	chr1:25471568-25529523	non-template	41	Intron_3	--	Yes	--	Yes	--	--	--	--	--	--	Yes
G4ST01025489898	chr1:25489899-25489941	+	GGGAGAAAAGGGCCAAAAGCTCCATTTGGTGGGGTTTCCAGGG<br>(((((.......)))))..	RHD	NM_001127691	chr1:25471568-25529523	non-template	43	Intron_3	--	Yes	--	Yes	--	--	--	--	--	--	Yes
G4ST01025489898	chr1:25489899-25489941	+	GGGAGAAAAGGGCCAAAAGCTCCATTTGGTGGGGTTTCCAGGG<br>(((((.......)))))..	RHD	NM_016124	chr1:25471568-25529523	non-template	43	Intron_3	--	Yes	--	Yes	--	--	--	--	--	--	Yes
G4ST01025498579	chr1:25498580-25498605	-	GGGTGTAACAAGGGCAGGGTGGAGGG<br>((...)).	RHD	NM_001127691	chr1:25471568-25529523	template	26	Intron_3	--	Yes	--	Yes	--	--	--	--	--	--	--
G4ST01025498579	chr1:25498580-25498605	-	GGGTGTAACAAGGGCAGGGTGGAGGG<br>((...)).	RHD	NM_016124	chr1:25471568-25529523	template	26	Intron_3	--	Yes	--	Yes	--	--	--	--	--	--	--
G4ST01025499861	chr1:25499862-25499888	+	GGGCAGAGCCTGCTGGGTTGGGCTGGG<br>(((...)))..	RHD	NM_001127691	chr1:25471568-25529523	non-template	27	Intron_3	--	Yes	--	Yes	--	--	--	Yes	--	--	--
G4ST01025499861	chr1:25499862-25499888	+	GGGCAGAGCCTGCTGGGTTGGGCTGGG<br>(((...)))..	RHD	NM_016124	chr1:25471568-25529523	non-template	27	Intron_3	--	Yes	--	Yes	--	--	--	Yes	--	--	--
G4ST01025537769	chr1:25537770-25537805	-	GGGGGATTTTCCAGGGATCCTGGGGTGTCCCGGGGG<br>(((...))).	TMEM50A	NM_014313	chr1:25537376-25561439	template	36	Intron_1.f	--	Yes	--	Yes	--	--	--	--	Yes	Yes	Yes
G4ST01025537780	chr1:25537781-25537813	-	GGGGGATCGGGGGATTTTCCAGGGATCCTGGGG<br>(((...))).	TMEM50A	NM_014313	chr1:25537376-25561439	template	33	Intron_1.f	--	Yes	--	Yes	--	--	--	--	Yes	Yes	Yes
G4ST01025601601	chr1:25601602-25601644	-	GGGAGAAAAGGGCCAAAAGCTCCATTTGGTGGGGCTTCCGGGG<br>(((((.......)))))..	RHCE	NM_020485	chr1:25561327-25619950	non-template	43	Intron_3	--	Yes	--	Yes	--	--	--	--	--	--	Yes
G4ST01025601601	chr1:25601602-25601644	-	GGGAGAAAAGGGCCAAAAGCTCCATTTGGTGGGGCTTCCGGGG<br>(((((.......)))))..	RHCE	NM_138618	chr1:25561327-25619950	non-template	43	Intron_3	--	Yes	--	Yes	--	--	--	--	--	--	Yes
G4ST01025601601	chr1:25601602-25601644	-	GGGAGAAAAGGGCCAAAAGCTCCATTTGGTGGGGCTTCCGGGG<br>(((((.......)))))..	RHCE	NM_138617	chr1:25561327-25619950	non-template	43	Intron_3	--	Yes	--	Yes	--	--	--	--	--	--	Yes
G4ST01025601601	chr1:25601602-25601644	-	GGGAGAAAAGGGCCAAAAGCTCCATTTGGTGGGGCTTCCGGGG<br>(((((.......)))))..	RHCE	NM_138616	chr1:25561327-25619950	non-template	43	Intron_3	--	Yes	--	Yes	--	--	--	--	--	--	Yes
G4ST01025601610	chr1:25601611-25601651	-	GGGACCTGGGAGAAAAGGGCCAAAAGCTCCATTTGGTGGGG<br>(((((.......)))))..	RHCE	NM_020485	chr1:25561327-25619950	non-template	41	Intron_3	--	Yes	--	Yes	--	--	--	--	--	--	Yes
G4ST01025601610	chr1:25601611-25601651	-	GGGACCTGGGAGAAAAGGGCCAAAAGCTCCATTTGGTGGGG<br>(((((.......)))))..	RHCE	NM_138618	chr1:25561327-25619950	non-template	41	Intron_3	--	Yes	--	Yes	--	--	--	--	--	--	Yes
G4ST01025601610	chr1:25601611-25601651	-	GGGACCTGGGAGAAAAGGGCCAAAAGCTCCATTTGGTGGGG<br>(((((.......)))))..	RHCE	NM_138617	chr1:25561327-25619950	non-template	41	Intron_3	--	Yes	--	Yes	--	--	--	--	--	--	Yes
G4ST01025601610	chr1:25601611-25601651	-	GGGACCTGGGAGAAAAGGGCCAAAAGCTCCATTTGGTGGGG<br>(((((.......)))))..	RHCE	NM_138616	chr1:25561327-25619950	non-template	41	Intron_3	--	Yes	--	Yes	--	--	--	--	--	--	Yes
G4ST01025608616	chr1:25608617-25608646	+	GGGCAGGGAGGGGCCCAGCAAAGCGGAGGG<br>..((.((...)))).	RHCE	NM_020485	chr1:25561327-25619950	template	30	Intron_1.f	--	Yes	--	Yes	--	--	--	--	--	--	--
G4ST01025608616	chr1:25608617-25608646	+	GGGCAGGGAGGGGCCCAGCAAAGCGGAGGG<br>..((.((...)))).	RHCE	NM_138618	chr1:25561327-25619950	template	30	Intron_1.f	--	Yes	--	Yes	--	--	--	--	--	--	--
G4ST01025608616	chr1:25608617-25608646	+	GGGCAGGGAGGGGCCCAGCAAAGCGGAGGG<br>..((.((...)))).	RHCE	NM_138617	chr1:25561327-25619950	template	30	Intron_1.f	--	Yes	--	Yes	--	--	--	--	--	--	--
G4ST01025608616	chr1:25608617-25608646	+	GGGCAGGGAGGGGCCCAGCAAAGCGGAGGG<br>..((.((...)))).	RHCE	NM_138616	chr1:25561327-25619950	template	30	Intron_1.f	--	Yes	--	Yes	--	--	--	--	--	--	--
G4ST01025629102	chr1:25629103-25629136	-	GGGCCGGACCGGGAGCCTGCTCGGCGGGTCCGGG<br>.(((.....)))	TMEM57	NM_018202	chr1:25629975-25699285	template	-838	upstream	Yes	--	--	--	--	--	--	--	Yes	Yes	Yes
G4ST01025629108	chr1:25629109-25629141	-	GGGCCGGGCCGGACCGGGAGCCTGCTCGGCGGG<br>.(((.....)))	TMEM57	NM_018202	chr1:25629975-25699285	template	-833	upstream	Yes	--	--	--	--	--	--	--	Yes	Yes	Yes
G4ST01025630290	chr1:25630291-25630341	-	GGGGTTACATAACGGGGCCAGGCCCCTCGGAGCTGGAGGGGGACCGCAGGG<br>(((....))). .((((.((....))..))))...	TMEM57	NM_018202	chr1:25629975-25699285	template	51	Intron_1.f	--	Yes	--	Yes	--	--	--	Yes	Yes	Yes	--
G4ST01025630444	chr1:25630445-25630470	+	GGGTCGGCCCGGCCGGGCGGGCAGGG<br>..(((...)))	TMEM57	NM_018202	chr1:25629975-25699285	non-template	26	Intron_1.f	--	Yes	--	Yes	--	--	--	Yes	Yes	Yes	Yes
G4ST01025761592	chr1:25761593-25761632	+	GGGATGGAGCTGCAGAAGCTGCAGGGAGGGAGCCAGGGGG<br>.((.((((.....)))).))	LDLRAP1	NM_015627	chr1:25742663-25767964	non-template	40	Intron_4	--	Yes	--	Yes	--	--	--	--	Yes	--	--
G4ST01025765585	chr1:25765586-25765611	-	GGGGTGGGGAACAAGCTGTGGGAGGG<br>..(((...)))	LDLRAP1	NM_015627	chr1:25742663-25767964	template	26	Intron_8.l	--	Yes	--	Yes	--	--	--	--	--	--	Yes
G4ST01025765589	chr1:25765590-25765617	-	GGGGGTGGGGTGGGGAACAAGCTGTGGG<br>..(((...)))	LDLRAP1	NM_015627	chr1:25742663-25767964	template	28	Intron_8.l	--	Yes	--	Yes	--	--	--	--	--	--	Yes
G4ST01025815682	chr1:25815683-25815706	+	GGGGAGGGCGGGGCTTGGAGCGGG<br>(((...)))	MAN1C1	NM_020379	chr1:25816546-25983845	non-template	-839	upstream	Yes	--	--	--	--	--	--	Yes	Yes	Yes	--
G4ST01025817092	chr1:25817093-25817126	-	GGGTTGGGAGGCGGCTCCTGCTCGCGGGCCGGGG<br>..(((((....)).)))	MAN1C1	NM_020379	chr1:25816546-25983845	template	34	Exon_1.f	--	Yes	Yes	--	Yes	--	--	Yes	--	Yes	--
G4ST01025817098	chr1:25817099-25817133	-	GGGGGCCGGGTTGGGAGGCGGCTCCTGCTCGCGGG<br>..(((((....)).)))	MAN1C1	NM_020379	chr1:25816546-25983845	template	35	Exon_1.f	--	Yes	Yes	--	Yes	--	--	Yes	--	Yes	--
G4ST01025817118	chr1:25817119-25817147	-	GGGGCCGGCGCGGCGGGGGCCGGGTTGGG<br>((((...))))..	MAN1C1	NM_020379	chr1:25816546-25983845	template	29	Exon_1.f	--	Yes	Yes	--	Yes	--	--	--	--	--	--
G4ST01025817209	chr1:25817210-25817248	-	GGGCCGCCGTGGCCTCCTCGCGGGGTCCAGTGGGGCGGG<br>.(((.(.((...)).))))	MAN1C1	NM_020379	chr1:25816546-25983845	template	39	Exon_1.f	--	Yes	Yes	--	Yes	--	--	Yes	--	--	--
G4ST01025818330	chr1:25818331-25818359	+	GGGGGCCGTGGGCAAGCTGTGGGCCAGGG<br>((...)).	MAN1C1	NM_020379	chr1:25816546-25983845	non-template	29	Intron_1.f	--	Yes	--	Yes	--	--	--	Yes	Yes	--	--
G4ST01025907303	chr1:25907304-25907335	+	GGGAAGGGGTGGGCAATTCCCTGAACTGAGGG<br>((.(((...))).)).	MAN1C1	NM_020379	chr1:25816546-25983845	non-template	32	Intron_2	--	Yes	--	Yes	--	--	--	Yes	--	--	--
G4ST01025910742	chr1:25910743-25910777	-	GGGGCAGGCTTGCAGCACGCAGGGGAGGGGCGGGG<br>((..(((...)))..))..	MAN1C1	NM_020379	chr1:25816546-25983845	template	35	Intron_2	--	Yes	--	Yes	--	--	--	Yes	Yes	--	Yes
G4ST01025910747	chr1:25910748-25910784	-	GGGGCCCGGGGCAGGCTTGCAGCACGCAGGGGAGGGG<br>((..(((...)))..))..	MAN1C1	NM_020379	chr1:25816546-25983845	template	37	Intron_2	--	Yes	--	Yes	--	--	--	Yes	Yes	--	Yes
G4ST01025910752	chr1:25910753-25910795	-	GGGGAGGAAGTGGGGCCCGGGGCAGGCTTGCAGCACGCAGGGG<br>((..(((...)))..))..	MAN1C1	NM_020379	chr1:25816546-25983845	template	43	Intron_2	--	Yes	--	Yes	--	--	--	Yes	Yes	--	Yes
G4ST01025968684	chr1:25968685-25968716	-	GGGATGGGAGGCTTGGGCTGTGCCAGGAAGGG<br>(((...)))...	MAN1C1	NM_020379	chr1:25816546-25983845	template	32	Intron_7	--	Yes	--	Yes	--	--	--	Yes	--	--	Yes
G4ST01025999318	chr1:25999319-25999356	-	GGGGCCGGGGCTGGGCGGCCCGCGTTGGCCCGGCCGGG<br>.((((.((....))..))))	SEPN1	NM_020451	chr1:25999254-26017300	template	38	Exon_1.f	--	Yes	Yes	--	Yes	--	--	Yes	Yes	Yes	Yes
G4ST01025999318	chr1:25999319-25999356	-	GGGGCCGGGGCTGGGCGGCCCGCGTTGGCCCGGCCGGG<br>.((((.((....))..))))	SEPN1	NM_206926	chr1:25999254-26017300	template	38	Exon_1.f	--	Yes	Yes	--	Yes	--	--	Yes	Yes	Yes	Yes
G4ST01026011971	chr1:26011972-26012006	+	GGGCCTCAGGGACTGCCCATGGTAGGGGCGTGGGG<br>.(((((...)))))	SEPN1	NM_020451	chr1:25999254-26017300	non-template	35	Intron_10	--	Yes	--	Yes	--	--	--	--	--	--	Yes
G4ST01026011971	chr1:26011972-26012006	+	GGGCCTCAGGGACTGCCCATGGTAGGGGCGTGGGG<br>.(((((...)))))	SEPN1	NM_206926	chr1:25999254-26017300	non-template	35	Intron_9	--	Yes	--	Yes	--	--	--	--	--	--	Yes

G4ST01026011979	chr1:26011980-26012012	+	GGGACTGCCCATGGTAGGGGCGTGGGGTGAGGG<br>.(((((...)))))	SEPN1	NM_020451	chr1:25999254-26017300	non-template	33	Intron_10	--	Yes	--	Yes	--	--	--	--	--	--	Yes
G4ST01026011979	chr1:26011980-26012012	+	GGGACTGCCCATGGTAGGGGCGTGGGGTGAGGG<br>.(((((...)))))	SEPN1	NM_206926	chr1:25999254-26017300	non-template	33	Intron_9	--	Yes	--	Yes	--	--	--	--	--	--	Yes
G4ST01026013241	chr1:26013242-26013288	-	GGGCCCTGTGGGGTCTGAGTGGGGGTGCCTACCACGGTGCCATTGGG<br>((((((......))))))...	SEPN1	NM_020451	chr1:25999254-26017300	template	47	Exon_12-Intron_12.l	--	Yes	Yes	Yes	Yes	--	--	--	--	--	--
G4ST01026013241	chr1:26013242-26013288	-	GGGCCCTGTGGGGTCTGAGTGGGGGTGCCTACCACGGTGCCATTGGG<br>((((((......))))))...	SEPN1	NM_206926	chr1:25999254-26017300	template	47	Exon_11-Intron_11.l	--	Yes	Yes	Yes	Yes	--	--	--	--	--	--
G4ST01026020083	chr1:26020084-26020124	+	GGGCCCAGTGTTGGGGGTTGAGGGCCAGTGTGGCTGGAGGG<br>.(((....))) ((((.....)))).	FAM54B	NM_001099625	chr1:26018984-26032020	non-template	41	Intron_1.f	--	Yes	--	Yes	--	--	--	Yes	Yes	Yes	--
G4ST01026020083	chr1:26020084-26020124	+	GGGCCCAGTGTTGGGGGTTGAGGGCCAGTGTGGCTGGAGGG<br>.(((....))) ((((.....)))).	FAM54B	NM_001099627	chr1:26018984-26032020	non-template	41	Intron_1.f	--	Yes	--	Yes	--	--	--	Yes	Yes	Yes	--
G4ST01026020083	chr1:26020084-26020124	+	GGGCCCAGTGTTGGGGGTTGAGGGCCAGTGTGGCTGGAGGG<br>.(((....))) ((((.....)))).	LOC646471	NR_024498	chr1:26019032-26022684	template	41	Exon_1.u	--	Yes	Yes	--	--	--	--	Yes	Yes	Yes	--
G4ST01026020083	chr1:26020084-26020124	+	GGGCCCAGTGTTGGGGGTTGAGGGCCAGTGTGGCTGGAGGG<br>.(((....))) ((((.....)))).	FAM54B	NM_019557	chr1:26019271-26032020	non-template	41	Intron_1.f	--	Yes	--	Yes	--	--	--	Yes	Yes	Yes	--
G4ST01026020083	chr1:26020084-26020124	+	GGGCCCAGTGTTGGGGGTTGAGGGCCAGTGTGGCTGGAGGG<br>.(((....))) ((((.....)))).	FAM54B	NM_001099626	chr1:26019906-26032020	non-template	41	Intron_1.f	--	Yes	--	Yes	--	--	--	Yes	Yes	Yes	--
G4ST01026020095	chr1:26020096-26020132	+	GGGGGTTGAGGGCCAGTGTGGCTGGAGGGCAAGGGGG<br>((((.....)))).	FAM54B	NM_001099625	chr1:26018984-26032020	non-template	37	Intron_1.f	--	Yes	--	Yes	--	--	--	Yes	Yes	Yes	--
G4ST01026020095	chr1:26020096-26020132	+	GGGGGTTGAGGGCCAGTGTGGCTGGAGGGCAAGGGGG<br>((((.....)))).	FAM54B	NM_001099627	chr1:26018984-26032020	non-template	37	Intron_1.f	--	Yes	--	Yes	--	--	--	Yes	Yes	Yes	--
G4ST01026020095	chr1:26020096-26020132	+	GGGGGTTGAGGGCCAGTGTGGCTGGAGGGCAAGGGGG<br>((((.....)))).	LOC646471	NR_024498	chr1:26019032-26022684	template	37	Exon_1.u	--	Yes	Yes	--	--	--	--	Yes	Yes	Yes	--
G4ST01026020095	chr1:26020096-26020132	+	GGGGGTTGAGGGCCAGTGTGGCTGGAGGGCAAGGGGG<br>((((.....)))).	FAM54B	NM_019557	chr1:26019271-26032020	non-template	37	Intron_1.f	--	Yes	--	Yes	--	--	--	Yes	Yes	Yes	--
G4ST01026020095	chr1:26020096-26020132	+	GGGGGTTGAGGGCCAGTGTGGCTGGAGGGCAAGGGGG<br>((((.....)))).	FAM54B	NM_001099626	chr1:26019906-26032020	non-template	37	Intron_1.f	--	Yes	--	Yes	--	--	--	Yes	Yes	Yes	--
G4ST01026053446	chr1:26053447-26053476	-	GGGAGGGAGGGAGTATATTTTGCATATGGG<br>...((((.....))))	C1orf135	NM_024037	chr1:26033084-26058435	non-template	30	Intron_1.f	--	Yes	--	Yes	--	--	--	--	--	--	--
G4ST01026062904	chr1:26062905-26062937	+	GGGCCATGGCCATGGCTGTGGGCCTGGGCAGGG<br>((((....))))....	PAQR7	NM_178422	chr1:26060562-26070331	template	33	Exon_2.l	--	Yes	Yes	--	Yes	Yes	--	--	--	--	Yes
G4ST01026105691	chr1:26105692-26105731	+	GGGGGTTGCAGGGCGCCTGGGGAGCATCCCCAGGTGTGGG<br>...((((....)))).	MIR3917	NR_037481	chr1:26105440-26105532	template	-159	upstream	Yes	--	--	--	--	--	--	--	Yes	Yes	--
G4ST01026105691	chr1:26105692-26105731	+	GGGGGTTGCAGGGCGCCTGGGGAGCATCCCCAGGTGTGGG<br>...((((....)))).	STMN1	NM_005563	chr1:26099194-26105580	template	-111	upstream	Yes	--	--	--	--	--	--	--	Yes	Yes	--
G4ST01026105691	chr1:26105692-26105731	+	GGGGGTTGCAGGGCGCCTGGGGAGCATCCCCAGGTGTGGG<br>...((((....)))).	STMN1	NM_203401	chr1:26099194-26105231	template	-460	upstream	Yes	--	--	--	--	--	--	--	Yes	Yes	--
G4ST01026105691	chr1:26105692-26105731	+	GGGGGTTGCAGGGCGCCTGGGGAGCATCCCCAGGTGTGGG<br>...((((....)))).	STMN1	NM_203399	chr1:26099194-26105955	template	40	Intron_1.f	--	Yes	--	Yes	--	--	--	--	Yes	Yes	--
G4ST01026105691	chr1:26105692-26105731	+	GGGGGTTGCAGGGCGCCTGGGGAGCATCCCCAGGTGTGGG<br>...((((....)))).	STMN1	NM_001145454	chr1:26083264-26105580	template	-111	upstream	Yes	--	--	--	--	--	--	--	Yes	Yes	--
G4ST01026105701	chr1:26105702-26105737	+	GGGCGCCTGGGGAGCATCCCCAGGTGTGGGCCTGGG<br>...((((....)))).	MIR3917	NR_037481	chr1:26105440-26105532	template	-169	upstream	Yes	--	--	--	--	--	--	--	Yes	Yes	--
G4ST01026105701	chr1:26105702-26105737	+	GGGCGCCTGGGGAGCATCCCCAGGTGTGGGCCTGGG<br>...((((....)))).	STMN1	NM_005563	chr1:26099194-26105580	template	-121	upstream	Yes	--	--	--	--	--	--	--	Yes	Yes	--
G4ST01026105701	chr1:26105702-26105737	+	GGGCGCCTGGGGAGCATCCCCAGGTGTGGGCCTGGG<br>...((((....)))).	STMN1	NM_203401	chr1:26099194-26105231	template	-470	upstream	Yes	--	--	--	--	--	--	--	Yes	Yes	--
G4ST01026105701	chr1:26105702-26105737	+	GGGCGCCTGGGGAGCATCCCCAGGTGTGGGCCTGGG<br>...((((....)))).	STMN1	NM_203399	chr1:26099194-26105955	template	36	Intron_1.f	--	Yes	--	Yes	--	--	--	--	Yes	Yes	--
G4ST01026105701	chr1:26105702-26105737	+	GGGCGCCTGGGGAGCATCCCCAGGTGTGGGCCTGGG<br>...((((....)))).	STMN1	NM_001145454	chr1:26083264-26105580	template	-121	upstream	Yes	--	--	--	--	--	--	--	Yes	Yes	--
G4ST01026105709	chr1:26105710-26105747	+	GGGGAGCATCCCCAGGTGTGGGCCTGGGACTAGGGGGG<br>...((((....)))).	MIR3917	NR_037481	chr1:26105440-26105532	template	-177	upstream	Yes	--	--	--	--	--	--	--	Yes	Yes	--
G4ST01026105709	chr1:26105710-26105747	+	GGGGAGCATCCCCAGGTGTGGGCCTGGGACTAGGGGGG<br>...((((....)))).	STMN1	NM_005563	chr1:26099194-26105580	template	-129	upstream	Yes	--	--	--	--	--	--	--	Yes	Yes	--
G4ST01026105709	chr1:26105710-26105747	+	GGGGAGCATCCCCAGGTGTGGGCCTGGGACTAGGGGGG<br>...((((....)))).	STMN1	NM_203401	chr1:26099194-26105231	template	-478	upstream	Yes	--	--	--	--	--	--	--	Yes	Yes	--
G4ST01026105709	chr1:26105710-26105747	+	GGGGAGCATCCCCAGGTGTGGGCCTGGGACTAGGGGGG<br>...((((....)))).	STMN1	NM_203399	chr1:26099194-26105955	template	38	Intron_1.f	--	Yes	--	Yes	--	--	--	--	Yes	Yes	--
G4ST01026105709	chr1:26105710-26105747	+	GGGGAGCATCCCCAGGTGTGGGCCTGGGACTAGGGGGG<br>...((((....)))).	STMN1	NM_001145454	chr1:26083264-26105580	template	-129	upstream	Yes	--	--	--	--	--	--	--	Yes	Yes	--
G4ST01026231611	chr1:26231612-26231650	+	GGGGGAGGGCACCCATGGGGTGTTTTATGAATACTTGGG<br>(((((.....)))))..	EXTL1	NM_004455	chr1:26220858-26235541	non-template	39	Intron_7	--	Yes	--	Yes	--	--	--	--	--	--	Yes
G4ST01026234989	chr1:26234990-26235026	-	GGGACTAGGAACCCTAGGAATGGGTGGCGGGGTGGGG<br>.(((((...)))))....	EXTL1	NM_004455	chr1:26220858-26235541	template	37	Exon_11.l	--	Yes	Yes	--	--	--	Yes	Yes	Yes	Yes	Yes
G4ST01026244631	chr1:26244632-26244662	+	GGGGCGGGGCTGCCTGGGGCAAAGTGCTGGG<br>(((...))).	SLC30A2	NM_001004434	chr1:26237101-26245191	template	31	Intron_1.f	--	Yes	--	Yes	--	--	--	Yes	--	--	Yes
G4ST01026244631	chr1:26244632-26244662	+	GGGGCGGGGCTGCCTGGGGCAAAGTGCTGGG<br>(((...))).	SLC30A2	NM_032513	chr1:26237101-26245191	template	31	Intron_1.f	--	Yes	--	Yes	--	--	--	Yes	--	--	Yes
G4ST01026244636	chr1:26244637-26244667	+	GGGGCTGCCTGGGGCAAAGTGCTGGGCGGGG<br>(((...))).	SLC30A2	NM_001004434	chr1:26237101-26245191	template	31	Intron_1.f	--	Yes	--	Yes	--	--	--	Yes	--	--	Yes
G4ST01026244636	chr1:26244637-26244667	+	GGGGCTGCCTGGGGCAAAGTGCTGGGCGGGG<br>(((...))).	SLC30A2	NM_032513	chr1:26237101-26245191	template	31	Intron_1.f	--	Yes	--	Yes	--	--	--	Yes	--	--	Yes
G4ST01026244646	chr1:26244647-26244671	+	GGGGCAAAGTGCTGGGCGGGGCGGG<br>(((...))).	SLC30A2	NM_001004434	chr1:26237101-26245191	template	25	Intron_1.f	--	Yes	--	Yes	--	--	--	Yes	--	--	Yes
G4ST01026244646	chr1:26244647-26244671	+	GGGGCAAAGTGCTGGGCGGGGCGGG<br>(((...))).	SLC30A2	NM_032513	chr1:26237101-26245191	template	25	Intron_1.f	--	Yes	--	Yes	--	--	--	Yes	--	--	Yes
G4ST01026310515	chr1:26310516-26310544	+	GGGTAGAGGGAGGGGAGGCCGCGGCCGGG<br>..(((....)))	PDIK1L	NR_026685	chr1:26310243-26324613	non-template	29	Intron_1.f	--	Yes	--	Yes	--	--	--	--	Yes	Yes	Yes
G4ST01026310515	chr1:26310516-26310544	+	GGGTAGAGGGAGGGGAGGCCGCGGCCGGG<br>..(((....)))	PDIK1L	NM_152835	chr1:26310855-26324613	non-template	-310	upstream	Yes	--	--	--	--	--	--	--	Yes	Yes	Yes
G4ST01026310515	chr1:26310516-26310544	+	GGGTAGAGGGAGGGGAGGCCGCGGCCGGG<br>..(((....)))	PDIK1L	NR_026686	chr1:26310855-26324613	non-template	-310	upstream	Yes	--	--	--	--	--	--	--	Yes	Yes	Yes
G4ST01026360379	chr1:26360380-26360414	-	GGGGGTCCGTCCTCTGGACGGGGTGGAGGGAGGGG<br>....(((((...)))))	GRRP1	NM_024869	chr1:26358098-26361706	template	35	Exon_2.l	--	Yes	Yes	--	Yes	--	--	--	--	--	--
G4ST01026383465	chr1:26383466-26383497	-	GGGCCAAGGGAGGCAGAGTCTGTCAGGGAGGG<br>.(((((...))))).	CNKSR1	NR_023345	chr1:26376569-26388962	template	32	Intron_11-Exon_12	--	Yes	Yes	Yes	--	--	--	--	--	--	Yes
G4ST01026383465	chr1:26383466-26383497	-	GGGCCAAGGGAGGCAGAGTCTGTCAGGGAGGG<br>.(((((...))))).	CNKSR1	NM_006314	chr1:26376568-26388962	template	32	Intron_11-Exon_12	--	Yes	Yes	Yes	Yes	--	--	--	--	--	Yes
G4ST01026383469	chr1:26383470-26383505	-	GGGGCTCAGGGCCAAGGGAGGCAGAGTCTGTCAGGG<br>.(((((...))))).	CNKSR1	NR_023345	chr1:26376569-26388962	template	36	Intron_11-Exon_12	--	Yes	Yes	Yes	--	--	--	--	--	--	Yes
G4ST01026383469	chr1:26383470-26383505	-	GGGGCTCAGGGCCAAGGGAGGCAGAGTCTGTCAGGG<br>.(((((...))))).	CNKSR1	NM_006314	chr1:26376568-26388962	template	36	Intron_11-Exon_12	--	Yes	Yes	Yes	Yes	--	--	--	--	--	Yes
G4ST01026479555	chr1:26479556-26479590	-	GGGGAGGGGAGGGCAGGCTGCGGTCGTGGTCAGGG<br>..((((((....)))))).	SH3BGRL3	NM_031286	chr1:26478800-26480600	template	35	Intron_1.f	--	Yes	--	Yes	--	--	--	Yes	Yes	Yes	Yes
G4ST01026484027	chr1:26484028-26484062	-	GGGTTAGTGCCGCACTGAGGGCGTGTCCGGGAGGG<br>..((((...))))..	UBXN11	NM_145345	chr1:26481360-26517343	non-template	35	Intron_10	--	Yes	--	Yes	--	--	--	Yes	Yes	Yes	--
G4ST01026484027	chr1:26484028-26484062	-	GGGTTAGTGCCGCACTGAGGGCGTGTCCGGGAGGG<br>..((((...))))..	UBXN11	NM_183008	chr1:26481360-26505782	non-template	35	Intron_11	--	Yes	--	Yes	--	--	--	Yes	Yes	Yes	--

G4ST01026484027	chr1:26484028-26484062	-	GGGTTAGTGCCGCACTGAGGGCGTGTCCGGGAGGG<br>..((((...))))..	UBXN11	NM_001077262	chr1:26481360-26505782	non-template	35	Intron_6	--	Yes	--	Yes	--	--	--	Yes	Yes	Yes	--
G4ST01026485162	chr1:26485163-26485195	-	GGGAGGGAGCCACAGAGGCTAGGGCTGCTAGGG<br>((((.....)))).	UBXN11	NM_145345	chr1:26481360-26517343	non-template	33	Intron_8	--	Yes	--	Yes	--	--	--	Yes	Yes	--	--
G4ST01026485162	chr1:26485163-26485195	-	GGGAGGGAGCCACAGAGGCTAGGGCTGCTAGGG<br>((((.....)))).	UBXN11	NM_183008	chr1:26481360-26505782	non-template	33	Intron_9	--	Yes	--	Yes	--	--	--	Yes	Yes	--	--
G4ST01026485162	chr1:26485163-26485195	-	GGGAGGGAGCCACAGAGGCTAGGGCTGCTAGGG<br>((((.....)))).	UBXN11	NM_001077262	chr1:26481360-26505782	non-template	33	Intron_4	--	Yes	--	Yes	--	--	--	Yes	Yes	--	--
G4ST01026488677	chr1:26488678-26488706	-	GGGCGTGGGCATCGCCATGATGGGCAGGG<br>.(((.....)))	UBXN11	NM_145345	chr1:26481360-26517343	non-template	29	Intron_8	--	Yes	--	Yes	--	--	--	Yes	Yes	--	--
G4ST01026488677	chr1:26488678-26488706	-	GGGCGTGGGCATCGCCATGATGGGCAGGG<br>.(((.....)))	UBXN11	NM_183008	chr1:26481360-26505782	non-template	29	Intron_9	--	Yes	--	Yes	--	--	--	Yes	Yes	--	--
G4ST01026488677	chr1:26488678-26488706	-	GGGCGTGGGCATCGCCATGATGGGCAGGG<br>.(((.....)))	UBXN11	NM_001077262	chr1:26481360-26505782	non-template	29	Intron_4	--	Yes	--	Yes	--	--	--	Yes	Yes	--	--
G4ST01026488849	chr1:26488850-26488902	-	GGGCGGCACCGCGTGAGCTGCCGGGGTGCCCTGGGCACTTGTGCAGTGAGGGG<br>(((((..((....))))))) ((((.....))))..	UBXN11	NM_145345	chr1:26481360-26517343	non-template	53	Intron_8	--	Yes	--	Yes	--	--	--	Yes	Yes	Yes	--
G4ST01026488849	chr1:26488850-26488902	-	GGGCGGCACCGCGTGAGCTGCCGGGGTGCCCTGGGCACTTGTGCAGTGAGGGG<br>(((((..((....))))))) ((((.....))))..	UBXN11	NM_183008	chr1:26481360-26505782	non-template	53	Intron_9	--	Yes	--	Yes	--	--	--	Yes	Yes	Yes	--
G4ST01026488849	chr1:26488850-26488902	-	GGGCGGCACCGCGTGAGCTGCCGGGGTGCCCTGGGCACTTGTGCAGTGAGGGG<br>(((((..((....))))))) ((((.....))))..	UBXN11	NM_001077262	chr1:26481360-26505782	non-template	53	Intron_4	--	Yes	--	Yes	--	--	--	Yes	Yes	Yes	--
G4ST01026490845	chr1:26490846-26490876	-	GGGTGGGTGTCGTGGGGAGAGTTCTCAAGGG<br>(((....)))..	UBXN11	NM_145345	chr1:26481360-26517343	non-template	31	Intron_8	--	Yes	--	Yes	--	--	--	--	--	--	--
G4ST01026490845	chr1:26490846-26490876	-	GGGTGGGTGTCGTGGGGAGAGTTCTCAAGGG<br>(((....)))..	UBXN11	NM_183008	chr1:26481360-26505782	non-template	31	Intron_9	--	Yes	--	Yes	--	--	--	--	--	--	--
G4ST01026490845	chr1:26490846-26490876	-	GGGTGGGTGTCGTGGGGAGAGTTCTCAAGGG<br>(((....)))..	UBXN11	NM_001077262	chr1:26481360-26505782	non-template	31	Intron_4	--	Yes	--	Yes	--	--	--	--	--	--	--
G4ST01026496952	chr1:26496953-26496986	-	GGGCTGGGGAGGCCAGGAGTTGGACTAGGGTGGG<br>.((.(((.....))).)).	UBXN11	NM_145345	chr1:26481360-26517343	non-template	34	Intron_5	--	Yes	--	Yes	--	--	--	--	Yes	--	--
G4ST01026496952	chr1:26496953-26496986	-	GGGCTGGGGAGGCCAGGAGTTGGACTAGGGTGGG<br>.((.(((.....))).)).	UBXN11	NM_183008	chr1:26481360-26505782	non-template	34	Intron_6	--	Yes	--	Yes	--	--	--	--	Yes	--	--
G4ST01026496952	chr1:26496953-26496986	-	GGGCTGGGGAGGCCAGGAGTTGGACTAGGGTGGG<br>.((.(((.....))).)).	UBXN11	NM_001077262	chr1:26481360-26505782	non-template	34	Intron_4	--	Yes	--	Yes	--	--	--	--	Yes	--	--
G4ST01026496956	chr1:26496957-26496992	-	GGGACTGGGCTGGGGAGGCCAGGAGTTGGACTAGGG<br>.((.(((.....))).)).	UBXN11	NM_145345	chr1:26481360-26517343	non-template	36	Intron_5	--	Yes	--	Yes	--	--	--	--	Yes	--	--
G4ST01026496956	chr1:26496957-26496992	-	GGGACTGGGCTGGGGAGGCCAGGAGTTGGACTAGGG<br>.((.(((.....))).)).	UBXN11	NM_183008	chr1:26481360-26505782	non-template	36	Intron_6	--	Yes	--	Yes	--	--	--	--	Yes	--	--
G4ST01026496956	chr1:26496957-26496992	-	GGGACTGGGCTGGGGAGGCCAGGAGTTGGACTAGGG<br>.((.(((.....))).)).	UBXN11	NM_001077262	chr1:26481360-26505782	non-template	36	Intron_4	--	Yes	--	Yes	--	--	--	--	Yes	--	--
G4ST01026499791	chr1:26499792-26499828	+	GGGGCTGGGGCTGGGCCTCGGCCTCCTGAGCTAAGGG<br>.(((((....)))))....	UBXN11	NM_145345	chr1:26481360-26517343	template	37	Intron_4	--	Yes	--	Yes	--	--	--	--	--	--	Yes
G4ST01026499791	chr1:26499792-26499828	+	GGGGCTGGGGCTGGGCCTCGGCCTCCTGAGCTAAGGG<br>.(((((....)))))....	UBXN11	NM_183008	chr1:26481360-26505782	template	37	Intron_5	--	Yes	--	Yes	--	--	--	--	--	--	Yes
G4ST01026499791	chr1:26499792-26499828	+	GGGGCTGGGGCTGGGCCTCGGCCTCCTGAGCTAAGGG<br>.(((((....)))))....	UBXN11	NM_001077262	chr1:26481360-26505782	template	37	Intron_4	--	Yes	--	Yes	--	--	--	--	--	--	Yes
G4ST01026499797	chr1:26499798-26499833	+	GGGGCTGGGCCTCGGCCTCCTGAGCTAAGGGTGGGG<br>.(((((....)))))....	UBXN11	NM_145345	chr1:26481360-26517343	template	36	Intron_4	--	Yes	--	Yes	--	--	--	--	--	--	Yes
G4ST01026499797	chr1:26499798-26499833	+	GGGGCTGGGCCTCGGCCTCCTGAGCTAAGGGTGGGG<br>.(((((....)))))....	UBXN11	NM_183008	chr1:26481360-26505782	template	36	Intron_5	--	Yes	--	Yes	--	--	--	--	--	--	Yes
G4ST01026499797	chr1:26499798-26499833	+	GGGGCTGGGCCTCGGCCTCCTGAGCTAAGGGTGGGG<br>.(((((....)))))....	UBXN11	NM_001077262	chr1:26481360-26505782	template	36	Intron_4	--	Yes	--	Yes	--	--	--	--	--	--	Yes
G4ST01026514587	chr1:26514588-26514621	+	GGGTGAGGAGAGACCTCTGGGTGAAAGGGCAGGG<br>.((((.....)))).	UBXN11	NM_145345	chr1:26481360-26517343	template	34	Intron_1.f	--	Yes	--	Yes	--	--	--	Yes	Yes	--	Yes
G4ST01026527525	chr1:26527526-26527567	+	GGGATTCCTCTGCCTGGAATAGGGTTCCCAGGGCGGAGAGGG<br>(((((.......))))).	AIM1L	NM_001039775	chr1:26520937-26553208	template	42	Intron_16	--	Yes	--	Yes	--	--	--	--	--	--	Yes
G4ST01026535527	chr1:26535528-26535557	-	GGGGGCGGGGCCGAGGGCGCGGCCGCGGGG<br>(((....)))	AIM1L	NM_001039775	chr1:26520937-26553208	non-template	30	Intron_12	--	Yes	--	Yes	--	--	--	Yes	Yes	Yes	--
G4ST01026535540	chr1:26535541-26535569	-	GGGGCCTTAGAAGGGGGCGGGGCCGAGGG<br>.(((....)))	AIM1L	NM_001039775	chr1:26520937-26553208	non-template	29	Intron_12	--	Yes	--	Yes	--	--	--	Yes	Yes	Yes	--
G4ST01026535547	chr1:26535548-26535574	-	GGGGCGGGGCCTTAGAAGGGGGCGGGG<br>.(((....)))	AIM1L	NM_001039775	chr1:26520937-26553208	non-template	27	Intron_12	--	Yes	--	Yes	--	--	--	Yes	Yes	Yes	--
G4ST01026535552	chr1:26535553-26535582	-	GGGCGCTTGGGGCGGGGCCTTAGAAGGGGG<br>.(((....)))	AIM1L	NM_001039775	chr1:26520937-26553208	non-template	30	Intron_12	--	Yes	--	Yes	--	--	--	Yes	Yes	Yes	--
G4ST01026536949	chr1:26536950-26536990	-	GGGGGCCAGAAGCACGGTCTGGGGGGATGGGACCTGATGGG<br>..(((((.......))))).	AIM1L	NM_001039775	chr1:26520937-26553208	non-template	41	Intron_8	--	Yes	--	Yes	--	--	--	--	--	--	Yes
G4ST01026536959	chr1:26536960-26536997	-	GGGCACTGGGGGCCAGAAGCACGGTCTGGGGGGATGGG<br>..(((((.......))))).	AIM1L	NM_001039775	chr1:26520937-26553208	non-template	38	Intron_8	--	Yes	--	Yes	--	--	--	--	--	--	Yes
G4ST01026542774	chr1:26542775-26542808	-	GGGTGGGCAAGGGAGGTGCCTGGCCTCACCTGGG<br>(((((........)))))	AIM1L	NM_001039775	chr1:26520937-26553208	non-template	34	Intron_2	--	Yes	--	Yes	--	--	--	--	--	--	--
G4ST01026609268	chr1:26609269-26609309	+	GGGTCAGCTCAGAGGCCTGAGGGGGAAGAGAGGGGGAGGGG<br>....(((((....))))).	LIN28A	NM_024674	chr1:26609856-26628806	non-template	-546	upstream	Yes	--	--	--	--	--	--	Yes	--	--	Yes
G4ST01026611375	chr1:26611376-26611414	+	GGGGGTGAAATTCAGTGTCGCAGGGCAGGGGGTGAAGGG<br>.((((.(.....).)))).	LIN28A	NM_024674	chr1:26609856-26628806	non-template	39	Intron_2	--	Yes	--	Yes	--	--	--	Yes	--	--	--
G4ST01026672191	chr1:26672192-26672229	-	GGGAGAGGGAGGCGCGCGCAGCCCGGGGAACTCGTGGG<br>...((.((...)).))	HMGN2	NM_005517	chr1:26671489-26675720	template	38	Intron_1.f	--	Yes	--	Yes	--	--	--	Yes	Yes	Yes	--
G4ST01026672201	chr1:26672202-26672233	-	GGGCGGGAGAGGGAGGCGCGCGCAGCCCGGGG<br>...((.((...)).))	HMGN2	NM_005517	chr1:26671489-26675720	template	32	Intron_1.f	--	Yes	--	Yes	--	--	--	Yes	Yes	Yes	--
G4ST01026728740	chr1:26728741-26728772	+	GGGAATGCCGCGCCGCGGGGGCTGGGGCGGGG<br>....((((...))))	RPS6KA1	NM_002953	chr1:26728836-26774107	non-template	-63	upstream	Yes	--	--	--	--	--	--	Yes	Yes	Yes	--
G4ST01026730709	chr1:26730710-26730751	+	GGGGAAGACCAGCGGACTTCCTGGGATTGGTGGGGCAGGGGG<br>((((.((...)).))))..	RPS6KA1	NM_002953	chr1:26728836-26774107	non-template	42	Intron_1.f	--	Yes	--	Yes	--	--	--	--	Yes	--	--
G4ST01026731521	chr1:26731522-26731549	+	GGGAGAGAGGCTCAGGGATGGGGAAGGG<br>.(((...))).	RPS6KA1	NM_002953	chr1:26728836-26774107	non-template	28	Intron_1.f	--	Yes	--	Yes	--	--	--	--	--	--	--
G4ST01026734378	chr1:26734379-26734416	+	GGGGAGCTGTTCCAGGGGCAGGGAGATTATGTAATGGG<br>...(((...))) ..((((....)))	RPS6KA1	NM_002953	chr1:26728836-26774107	non-template	38	Intron_1.f	--	Yes	--	Yes	--	--	--	--	Yes	--	--
G4ST01026738909	chr1:26738910-26738949	-	GGGGTCCAGCTCCGGAGATAGCTGGGGGAGGGGGCCTGGG<br>..((((((........))))))	RPS6KA1	NM_002953	chr1:26728836-26774107	template	40	Intron_2	--	Yes	--	Yes	--	--	--	Yes	--	--	--
G4ST01026743179	chr1:26743180-26743209	-	GGGCAGGGATCCCCAGGAAGGGGTTAAGGG<br>.(((...)))..	RPS6KA1	NM_001006665	chr1:26744930-26774107	template	-1720	upstream	Yes	--	--	--	--	--	--	Yes	--	Yes	--
G4ST01026743179	chr1:26743180-26743209	-	GGGCAGGGATCCCCAGGAAGGGGTTAAGGG<br>.(((...)))..	RPS6KA1	NM_002953	chr1:26728836-26774107	template	30	Intron_2	--	Yes	--	Yes	--	--	--	Yes	--	Yes	--
G4ST01026752686	chr1:26752687-26752714	+	GGGCAATCTGAGGGATGGGTGAGTGGGG<br>((...)).	MIR1976	NR_031740	chr1:26753620-26753671	non-template	-905	upstream	Yes	--	--	--	--	--	--	--	--	--	Yes
G4ST01026752686	chr1:26752687-26752714	+	GGGCAATCTGAGGGATGGGTGAGTGGGG<br>((...)).	RPS6KA1	NM_001006665	chr1:26744930-26774107	non-template	28	Intron_6	--	Yes	--	Yes	--	--	--	--	--	--	Yes
G4ST01026752686	chr1:26752687-26752714	+	GGGCAATCTGAGGGATGGGTGAGTGGGG<br>((...)).	RPS6KA1	NM_002953	chr1:26728836-26774107	non-template	28	Intron_7	--	Yes	--	Yes	--	--	--	--	--	--	Yes
G4ST01026753209	chr1:26753210-26753245	-	GGGTGATGGTGTTACCACAGGGCTGTGGGAGTTGGG<br>...((((...))))..	MIR1976	NR_031740	chr1:26753620-26753671	template	-374	upstream	Yes	--	--	--	--	--	--	Yes	--	--	--

G4ST01026753209	chr1:26753210-26753245	-	GGGTGATGGTGTTACCACAGGGCTGTGGGAGTTGGG<br>...((((...))))..	RPS6KA1	NM_001006665	chr1:26744930-26774107	template	36	Intron_6	--	Yes	--	Yes	--	--	--	Yes	--	--	--
G4ST01026753209	chr1:26753210-26753245	-	GGGTGATGGTGTTACCACAGGGCTGTGGGAGTTGGG<br>...((((...))))..	RPS6KA1	NM_002953	chr1:26728836-26774107	template	36	Intron_7	--	Yes	--	Yes	--	--	--	Yes	--	--	--
G4ST01026753567	chr1:26753568-26753606	+	GGGGGTGATGCCTTCTGGCCTCTGGGCACGGGGGTTGGG<br>...((.(((....))).)).	MIR1976	NR_031740	chr1:26753620-26753671	non-template	-13	upstream	Yes	--	--	--	--	--	--	Yes	--	--	--
G4ST01026753567	chr1:26753568-26753606	+	GGGGGTGATGCCTTCTGGCCTCTGGGCACGGGGGTTGGG<br>...((.(((....))).)).	RPS6KA1	NM_001006665	chr1:26744930-26774107	non-template	39	Intron_7	--	Yes	--	Yes	--	--	--	Yes	--	--	--
G4ST01026753567	chr1:26753568-26753606	+	GGGGGTGATGCCTTCTGGCCTCTGGGCACGGGGGTTGGG<br>...((.(((....))).)).	RPS6KA1	NM_002953	chr1:26728836-26774107	non-template	39	Intron_8	--	Yes	--	Yes	--	--	--	Yes	--	--	--
G4ST01026894976	chr1:26894977-26895013	-	GGGCTGGAGGGCGCGCGGCAGCAGCCGGGGCGCCGGG<br>....((((....))))	ARID1A	NM_006015	chr1:26895109-26981188	template	-95	upstream	Yes	--	--	--	--	--	--	Yes	Yes	Yes	Yes
G4ST01026894976	chr1:26894977-26895013	-	GGGCTGGAGGGCGCGCGGCAGCAGCCGGGGCGCCGGG<br>....((((....))))	ARID1A	NM_139135	chr1:26895109-26981188	template	-95	upstream	Yes	--	--	--	--	--	--	Yes	Yes	Yes	Yes
G4ST01026894983	chr1:26894984-26895017	-	GGGCGGGCTGGAGGGCGCGCGGCAGCAGCCGGGG<br>....((((....))))	ARID1A	NM_006015	chr1:26895109-26981188	template	-91	upstream	Yes	--	--	--	--	--	--	Yes	Yes	Yes	Yes
G4ST01026894983	chr1:26894984-26895017	-	GGGCGGGCTGGAGGGCGCGCGGCAGCAGCCGGGG<br>....((((....))))	ARID1A	NM_139135	chr1:26895109-26981188	template	-91	upstream	Yes	--	--	--	--	--	--	Yes	Yes	Yes	Yes
G4ST01026896397	chr1:26896398-26896438	+	GGGGCTACCAGGGCTACCCCGGGGGCGACTACAGTGGCGGG<br>.((.((....)).))	ARID1A	NM_006015	chr1:26895109-26981188	non-template	41	Exon_1.f	--	Yes	Yes	--	Yes	--	--	--	Yes	Yes	--
G4ST01026896397	chr1:26896398-26896438	+	GGGGCTACCAGGGCTACCCCGGGGGCGACTACAGTGGCGGG<br>.((.((....)).))	ARID1A	NM_139135	chr1:26895109-26981188	non-template	41	Exon_1.f	--	Yes	Yes	--	Yes	--	--	--	Yes	Yes	--
G4ST01026896874	chr1:26896875-26896911	+	GGGGTCTTGATGGGGGTGCGGGGCAGAGTGTGCGGGG<br>(((.....))).	ARID1A	NM_006015	chr1:26895109-26981188	non-template	37	Intron_1.f	--	Yes	--	Yes	--	--	--	Yes	Yes	Yes	Yes
G4ST01026896874	chr1:26896875-26896911	+	GGGGTCTTGATGGGGGTGCGGGGCAGAGTGTGCGGGG<br>(((.....))).	ARID1A	NM_139135	chr1:26895109-26981188	non-template	37	Intron_1.f	--	Yes	--	Yes	--	--	--	Yes	Yes	Yes	Yes
G4ST01026896885	chr1:26896886-26896916	+	GGGGGTGCGGGGCAGAGTGTGCGGGGAAGGG<br>(((.....))).	ARID1A	NM_006015	chr1:26895109-26981188	non-template	31	Intron_1.f	--	Yes	--	Yes	--	--	--	Yes	Yes	Yes	Yes
G4ST01026896885	chr1:26896886-26896916	+	GGGGGTGCGGGGCAGAGTGTGCGGGGAAGGG<br>(((.....))).	ARID1A	NM_139135	chr1:26895109-26981188	non-template	31	Intron_1.f	--	Yes	--	Yes	--	--	--	Yes	Yes	Yes	Yes
G4ST01026896893	chr1:26896894-26896928	+	GGGGCAGAGTGTGCGGGGAAGGGCCTGGCCGGGGG<br>(((.....))).	ARID1A	NM_006015	chr1:26895109-26981188	non-template	35	Intron_1.f	--	Yes	--	Yes	--	--	--	Yes	Yes	Yes	Yes
G4ST01026896893	chr1:26896894-26896928	+	GGGGCAGAGTGTGCGGGGAAGGGCCTGGCCGGGGG<br>(((.....))).	ARID1A	NM_139135	chr1:26895109-26981188	non-template	35	Intron_1.f	--	Yes	--	Yes	--	--	--	Yes	Yes	Yes	Yes
G4ST01026979257	chr1:26979258-26979292	-	GGGTGGAAAAGGGGTCCTGGGCTTCAGCTGAAGGG<br>.(((....)))	ARID1A	NM_006015	chr1:26895109-26981188	template	35	Exon_20.l	--	Yes	Yes	--	Yes	--	--	--	--	--	Yes
G4ST01026979257	chr1:26979258-26979292	-	GGGTGGAAAAGGGGTCCTGGGCTTCAGCTGAAGGG<br>.(((....)))	ARID1A	NM_139135	chr1:26895109-26981188	template	35	Exon_20.l	--	Yes	Yes	--	Yes	--	--	--	--	--	Yes
G4ST01027026934	chr1:27026935-27026962	-	GGGAGCCGGCTGGGTGGAGGCGGGCGGG<br>((....))	ZDHHC18	NM_032283	chr1:27025788-27054798	template	28	Intron_1.f	--	Yes	--	Yes	--	--	--	Yes	--	--	Yes
G4ST01027026938	chr1:27026939-27026969	-	GGGGCCTGGGAGCCGGCTGGGTGGAGGCGGG<br>((....))	ZDHHC18	NM_032283	chr1:27025788-27054798	template	31	Intron_1.f	--	Yes	--	Yes	--	--	--	Yes	--	--	Yes
G4ST01027026948	chr1:27026949-27026978	-	GGGGGGCCAGGGGCCTGGGAGCCGGCTGGG<br>((....))	ZDHHC18	NM_032283	chr1:27025788-27054798	template	30	Intron_1.f	--	Yes	--	Yes	--	--	--	Yes	--	--	Yes
G4ST01027032346	chr1:27032347-27032381	+	GGGAGGGTGTGTGATTTTACATAGGGTGGCCAGGG<br>.(((((....))))).	ZDHHC18	NM_032283	chr1:27025788-27054798	non-template	35	Intron_2	--	Yes	--	Yes	--	--	--	--	--	Yes	--
G4ST01027040662	chr1:27040663-27040697	+	GGGGAGCCAAAAGCTGGGACTCTGGGGCCTGTGGG<br>.(((.....)))	ZDHHC18	NM_032283	chr1:27025788-27054798	non-template	35	Intron_2	--	Yes	--	Yes	--	--	--	--	--	--	--
G4ST01027091420	chr1:27091421-27091458	+	GGGAAGAGGGAAACCAGGGCATAGCTGAAAGGCTTGGG<br>...((((....)))).	GPN2	NM_018066	chr1:27078460-27089456	template	-1964	upstream	Yes	--	--	--	--	--	--	--	--	--	--
G4ST01027091420	chr1:27091421-27091458	+	GGGAAGAGGGAAACCAGGGCATAGCTGAAAGGCTTGGG<br>...((((....)))).	GPATCH3	NM_022078	chr1:27089566-27099549	template	38	Intron_6.l	--	Yes	--	Yes	--	--	--	--	--	--	--
G4ST01027091427	chr1:27091428-27091467	+	GGGAAACCAGGGCATAGCTGAAAGGCTTGGGCCCCCAGGG<br>...((((....)))).	GPN2	NM_018066	chr1:27078460-27089456	template	-1971	upstream	Yes	--	--	--	--	--	--	--	--	--	--
G4ST01027091427	chr1:27091428-27091467	+	GGGAAACCAGGGCATAGCTGAAAGGCTTGGGCCCCCAGGG<br>...((((....)))).	GPATCH3	NM_022078	chr1:27089566-27099549	template	40	Intron_6.l	--	Yes	--	Yes	--	--	--	--	--	--	--
G4ST01027111050	chr1:27111051-27111084	+	GGGTCAGGCGGCCTTGGGCTGCTGGGCACCAGGG<br>..(((...))).	NR0B2	NM_021969	chr1:27110562-27113154	template	34	Exon_2.l	--	Yes	Yes	--	Yes	--	--	--	Yes	--	--
G4ST01027113561	chr1:27113562-27113591	+	GGGAAGGGAGCCAGGACCCTGGGGGCTGGG<br>..((((....))))	NR0B2	NM_021969	chr1:27110562-27113154	template	-407	upstream	Yes	--	--	--	--	--	--	Yes	Yes	Yes	--
G4ST01027150913	chr1:27150914-27150942	+	GGGGCTGGGGAGGTGGCCCTCGGGTAGGG<br>((((....))))	C1orf172	NM_152365	chr1:27148634-27159488	template	29	Exon_2	--	Yes	Yes	--	Yes	--	--	--	--	--	--
G4ST01027150919	chr1:27150920-27150952	+	GGGGAGGTGGCCCTCGGGTAGGGATACACAGGG<br>((((....))))	C1orf172	NM_152365	chr1:27148634-27159488	template	33	Exon_2	--	Yes	Yes	--	Yes	--	--	--	--	--	--
G4ST01027299238	chr1:27299239-27299278	+	GGGGTGGGGGCTGTGGGCCCAGCTGCCATGCGGTAGGGGG<br>.....(((((....))))).	SLC9A1	NM_003047	chr1:27297887-27354038	template	40	Exon_12.l	--	Yes	Yes	--	--	--	Yes	--	--	--	Yes
G4ST01027299243	chr1:27299244-27299283	+	GGGGGCTGTGGGCCCAGCTGCCATGCGGTAGGGGGAGGGG<br>.....(((((....))))).	SLC9A1	NM_003047	chr1:27297887-27354038	template	40	Exon_12.l	--	Yes	Yes	--	--	--	Yes	--	--	--	Yes
G4ST01027299252	chr1:27299253-27299288	+	GGGCCCAGCTGCCATGCGGTAGGGGGAGGGGCAGGG<br>.....(((((....))))).	SLC9A1	NM_003047	chr1:27297887-27354038	template	36	Exon_12.l	--	Yes	Yes	--	--	--	Yes	--	--	--	Yes
G4ST01027304925	chr1:27304926-27304957	-	GGGCCAGGGCAGGGCAGGCCCTTGCCTTGGGG<br>.((((....))))..	SLC9A1	NM_003047	chr1:27297887-27354038	non-template	32	Intron_5	--	Yes	--	Yes	--	--	--	--	--	--	--
G4ST01027308728	chr1:27308729-27308760	-	GGGCGGGGTGCTTGTGGGCGTGGTCTACGGGG<br>(((.....)))	SLC9A1	NM_003047	chr1:27297887-27354038	non-template	32	Exon_3	--	Yes	Yes	--	Yes	--	--	--	--	--	--
G4ST01027349844	chr1:27349845-27349883	-	GGGTAGACTCTTGCAGAGACTGGGGTGGGAGGAGCAGGG<br>.((.((((...)))).)).	SLC9A1	NM_003047	chr1:27297887-27354038	non-template	39	Intron_1.f	--	Yes	--	Yes	--	--	--	--	--	--	Yes
G4ST01027506404	chr1:27506405-27506441	+	GGGGAGAGGGGTGGGTCTCCTGAGCCACTCAGATGGG<br>....(((((...)))))..	WDTC1	NM_015023	chr1:27433594-27507697	non-template	37	Exon_16.l	--	Yes	Yes	--	--	--	Yes	Yes	Yes	--	--
G4ST01027531230	chr1:27531231-27531264	+	GGGGCGGGCCAGGCCTTCTGGGGCACAGGAGGGG<br>.(((.....)))	TMEM222	NM_032125	chr1:27521223-27535478	non-template	34	Intron_3	--	Yes	--	Yes	--	--	--	--	--	--	--
G4ST01027531230	chr1:27531231-27531264	+	GGGGCGGGCCAGGCCTTCTGGGGCACAGGAGGGG<br>.(((.....)))	TMEM222	NR_037576	chr1:27521223-27535478	non-template	34	Intron_4	--	Yes	--	Yes	--	--	--	--	--	--	--
G4ST01027531230	chr1:27531231-27531264	+	GGGGCGGGCCAGGCCTTCTGGGGCACAGGAGGGG<br>.(((.....)))	TMEM222	NR_037577	chr1:27521223-27535478	non-template	34	Intron_3	--	Yes	--	Yes	--	--	--	--	--	--	--
G4ST01027531230	chr1:27531231-27531264	+	GGGGCGGGCCAGGCCTTCTGGGGCACAGGAGGGG<br>.(((.....)))	TMEM222	NR_037580	chr1:27521223-27535478	non-template	34	Intron_4	--	Yes	--	Yes	--	--	--	--	--	--	--
G4ST01027533379	chr1:27533380-27533412	-	GGGAGAGCAGCCTCTGGGCAGTGTGTGGGTGGG<br>((((....)))) ((...)).	TMEM222	NM_032125	chr1:27521223-27535478	template	33	Intron_5.l	--	Yes	--	Yes	--	--	--	--	--	--	--
G4ST01027533379	chr1:27533380-27533412	-	GGGAGAGCAGCCTCTGGGCAGTGTGTGGGTGGG<br>((((....)))) ((...)).	TMEM222	NR_037576	chr1:27521223-27535478	template	33	Intron_6.l	--	Yes	--	Yes	--	--	--	--	--	--	--
G4ST01027533379	chr1:27533380-27533412	-	GGGAGAGCAGCCTCTGGGCAGTGTGTGGGTGGG<br>((((....)))) ((...)).	TMEM222	NR_037577	chr1:27521223-27535478	template	33	Intron_5	--	Yes	--	Yes	--	--	--	--	--	--	--
G4ST01027533379	chr1:27533380-27533412	-	GGGAGAGCAGCCTCTGGGCAGTGTGTGGGTGGG<br>((((....)))) ((...)).	TMEM222	NR_037580	chr1:27521223-27535478	template	33	Intron_6	--	Yes	--	Yes	--	--	--	--	--	--	--
G4ST01027534724	chr1:27534725-27534760	-	GGGGAGGAGGGGACAGGGTGCTTCTGAGAGGCAGGG<br>.(((((...))))).	TMEM222	NM_032125	chr1:27521223-27535478	template	36	Exon_6.l	--	Yes	Yes	--	--	--	Yes	--	--	--	--
G4ST01027534724	chr1:27534725-27534760	-	GGGGAGGAGGGGACAGGGTGCTTCTGAGAGGCAGGG<br>.(((((...))))).	TMEM222	NR_037576	chr1:27521223-27535478	template	36	Exon_7.l	--	Yes	Yes	--	--	--	--	--	--	--	--
G4ST01027534724	chr1:27534725-27534760	-	GGGGAGGAGGGGACAGGGTGCTTCTGAGAGGCAGGG<br>.(((((...))))).	TMEM222	NR_037577	chr1:27521223-27535478	template	36	Exon_7.l	--	Yes	Yes	--	--	--	--	--	--	--	--

G4ST01027534724	chr1:27534725-27534760	-	GGGGAGGAGGGGACAGGGTGCTTCTGAGAGGCAGGG<br>.(((((...))))).	TMEM222	NR_037580	chr1:27521223-27535478	template	36	Exon_8.l	--	Yes	Yes	--	--	--	--	--	--	--	--
G4ST01027542462	chr1:27542463-27542493	+	GGGCAGGGCTGGCGGCGGGGGCATCCCTGGG<br>(((....)))	SYTL1	NM_001193308	chr1:27541070-27553010	non-template	31	Intron_1.f	--	Yes	--	Yes	--	--	--	Yes	Yes	Yes	--
G4ST01027542462	chr1:27542463-27542493	+	GGGCAGGGCTGGCGGCGGGGGCATCCCTGGG<br>(((....)))	SYTL1	NM_032872	chr1:27541070-27553010	non-template	31	Intron_1.f	--	Yes	--	Yes	--	--	--	Yes	Yes	Yes	--
G4ST01027542467	chr1:27542468-27542510	+	GGGCTGGCGGCGGGGGCATCCCTGGGTTGGAGGCCTCCGTGGG<br>(((....))) .((((....)))).	SYTL1	NM_001193308	chr1:27541070-27553010	non-template	43	Intron_1.f	--	Yes	--	Yes	--	--	--	Yes	Yes	Yes	--
G4ST01027542467	chr1:27542468-27542510	+	GGGCTGGCGGCGGGGGCATCCCTGGGTTGGAGGCCTCCGTGGG<br>(((....))) .((((....)))).	SYTL1	NM_032872	chr1:27541070-27553010	non-template	43	Intron_1.f	--	Yes	--	Yes	--	--	--	Yes	Yes	Yes	--
G4ST01027542478	chr1:27542479-27542516	+	GGGGGCATCCCTGGGTTGGAGGCCTCCGTGGGAGTGGG<br>.((((....)))).	SYTL1	NM_001193308	chr1:27541070-27553010	non-template	38	Intron_1.f	--	Yes	--	Yes	--	--	--	Yes	Yes	Yes	--
G4ST01027542478	chr1:27542479-27542516	+	GGGGGCATCCCTGGGTTGGAGGCCTCCGTGGGAGTGGG<br>.((((....)))).	SYTL1	NM_032872	chr1:27541070-27553010	non-template	38	Intron_1.f	--	Yes	--	Yes	--	--	--	Yes	Yes	Yes	--
G4ST01027543969	chr1:27543970-27544001	+	GGGCATGGGTCCGGGTCTGTGTCTGCAGAGGG<br>.((((....)))).	SYTL1	NM_001193308	chr1:27541070-27553010	non-template	32	Intron_1.f	--	Yes	--	Yes	--	--	--	--	--	--	--
G4ST01027543969	chr1:27543970-27544001	+	GGGCATGGGTCCGGGTCTGTGTCTGCAGAGGG<br>.((((....)))).	SYTL1	NM_032872	chr1:27541070-27553010	non-template	32	Intron_1.f	--	Yes	--	Yes	--	--	--	--	--	--	--
G4ST01027559495	chr1:27559496-27559524	-	GGGGCCAGGCTGGCGTGGGATGGGCGGGG<br>((((...))))..	MAP3K6	NM_004672	chr1:27554257-27565924	non-template	29	Intron_15	--	Yes	--	Yes	--	--	--	Yes	--	--	Yes
G4ST01027559500	chr1:27559501-27559529	-	GGGGCGGGGCCAGGCTGGCGTGGGATGGG<br>((((...))))..	MAP3K6	NM_004672	chr1:27554257-27565924	non-template	29	Intron_15	--	Yes	--	Yes	--	--	--	Yes	--	--	Yes
G4ST01027559505	chr1:27559506-27559537	-	GGGTCCTTGGGGCGGGGCCAGGCTGGCGTGGG<br>((((...))))..	MAP3K6	NM_004672	chr1:27554257-27565924	non-template	32	Intron_15	--	Yes	--	Yes	--	--	--	Yes	--	--	Yes
G4ST01027559842	chr1:27559843-27559875	-	GGGCAAGGGCACGTATGGGGTGGTGTACGCGGG<br>(((.....)))	MAP3K6	NM_004672	chr1:27554257-27565924	non-template	33	Exon_15	--	Yes	Yes	--	Yes	--	--	Yes	Yes	--	--
G4ST01027559855	chr1:27559856-27559893	-	GGGCGAGCGGCTGGTGCTGGGCAAGGGCACGTATGGGG<br>..((((.....))))	MAP3K6	NM_004672	chr1:27554257-27565924	non-template	38	Exon_15	--	Yes	Yes	--	Yes	--	--	Yes	Yes	--	--
G4ST01027563224	chr1:27563225-27563261	-	GGGTGCTCCAGGAGGGGGCTGGTGAGGGTATGGTGGG<br>..(((...))).	MAP3K6	NM_004672	chr1:27554257-27565924	non-template	37	Intron_4	--	Yes	--	Yes	--	--	--	--	--	--	Yes
G4ST01027563233	chr1:27563234-27563265	-	GGGAGGGTGCTCCAGGAGGGGGCTGGTGAGGG<br>..(((...))).	MAP3K6	NM_004672	chr1:27554257-27565924	non-template	32	Intron_4	--	Yes	--	Yes	--	--	--	--	--	--	Yes
G4ST01027565677	chr1:27565678-27565720	+	GGGGGCGCTCAGGCGCGGGGCGCCGCGCACTGGGAACCGGGGG<br>.((((....)))). (((...)))...	MAP3K6	NM_004672	chr1:27554257-27565924	template	43	Exon_1.f	--	Yes	Yes	--	--	Yes	--	Yes	Yes	Yes	--
G4ST01027565693	chr1:27565694-27565725	+	GGGGCGCCGCGCACTGGGAACCGGGGGCGGGG<br>(((...)))...	MAP3K6	NM_004672	chr1:27554257-27565924	template	32	Exon_1.f	--	Yes	Yes	--	--	Yes	--	Yes	Yes	Yes	--
G4ST01027582324	chr1:27582325-27582355	+	GGGGTGGGGGTGGCCGGGGGCGGTGGCCGGG<br>(((....)))	CD164L2	NM_207397	chr1:27578183-27582392	template	31	Exon_1.f	--	Yes	Yes	--	--	Yes	--	Yes	Yes	Yes	Yes
G4ST01027582329	chr1:27582330-27582375	+	GGGGGTGGCCGGGGGCGGTGGCCGGGATCGGTGGACAGCTGCCGGG<br>(((....))) ...(((((....)))))	CD164L2	NM_207397	chr1:27578183-27582392	template	46	Exon_1.f	--	Yes	Yes	--	--	Yes	--	Yes	Yes	Yes	Yes
G4ST01027591513	chr1:27591514-27591555	+	GGGCGCCGCGTCAAGGACAGCGCGGGCAGACTCGGGAGGGGG<br>...((((((...))).))).	GPR3	NM_005281	chr1:27591739-27594904	non-template	-183	upstream	Yes	--	--	--	--	--	--	Yes	Yes	Yes	Yes
G4ST01027733506	chr1:27733507-27733535	-	GGGGGGGAGGGAGAAGAACCTCTTGCGGG<br>..((((...))))..	AHDC1	NM_001029882	chr1:27733343-27802730	non-template	29	Exon_7.l	--	Yes	Yes	--	--	--	Yes	--	--	--	--
G4ST01027746312	chr1:27746313-27746342	-	GGGCGCAGTGGGCAGAGGCCTGGGGGTGGG<br>(((....))).	AHDC1	NM_001029882	chr1:27733343-27802730	non-template	30	Intron_6.l	--	Yes	--	Yes	--	--	--	--	--	--	Yes
G4ST01027746316	chr1:27746317-27746350	-	GGGGATGTGGGCGCAGTGGGCAGAGGCCTGGGGG<br>(((....))).	AHDC1	NM_001029882	chr1:27733343-27802730	non-template	34	Intron_6.l	--	Yes	--	Yes	--	--	--	--	--	--	Yes
G4ST01027747052	chr1:27747053-27747074	+	GGGTGGGTGGGCCAGCGGTGGG<br>((...)).	AHDC1	NM_001029882	chr1:27733343-27802730	template	22	Exon_6	--	Yes	Yes	--	Yes	--	--	--	--	--	--
G4ST01027747056	chr1:27747057-27747097	+	GGGTGGGCCAGCGGTGGGAAATGCTTGCCATCAAGCTCGGG<br>((...)). ....(((((....)))))..	AHDC1	NM_001029882	chr1:27733343-27802730	template	41	Exon_6	--	Yes	Yes	--	Yes	--	--	--	--	--	--
G4ST01027747060	chr1:27747061-27747112	+	GGGCCAGCGGTGGGAAATGCTTGCCATCAAGCTCGGGAGCACCCAGGCTGGG<br>((...)). ....(((((....))))).. (((......)))	AHDC1	NM_001029882	chr1:27733343-27802730	template	52	Exon_6	--	Yes	Yes	--	Yes	--	--	--	--	--	--
G4ST01027755271	chr1:27755272-27755305	-	GGGGTGGTCACAGCCACAGCCGGGGAGGGTGGGG<br>(((((....))))).....	AHDC1	NM_001029882	chr1:27733343-27802730	non-template	34	Intron_3	--	Yes	--	Yes	--	--	--	Yes	Yes	--	Yes
G4ST01027755276	chr1:27755277-27755310	-	GGGGTGGGGTGGTCACAGCCACAGCCGGGGAGGG<br>(((((....))))).....	AHDC1	NM_001029882	chr1:27733343-27802730	non-template	34	Intron_3	--	Yes	--	Yes	--	--	--	Yes	Yes	--	Yes
G4ST01027755280	chr1:27755281-27755316	-	GGGGAAGGGGTGGGGTGGTCACAGCCACAGCCGGGG<br>(((((....))))).....	AHDC1	NM_001029882	chr1:27733343-27802730	non-template	36	Intron_3	--	Yes	--	Yes	--	--	--	Yes	Yes	--	Yes
G4ST01027756054	chr1:27756055-27756086	-	GGGCCCTGATGGCCCCAGGGGGCGGGGCAGGG<br>.((((.......))))	AHDC1	NM_001029882	chr1:27733343-27802730	non-template	32	Intron_3	--	Yes	--	Yes	--	--	--	Yes	Yes	--	--
G4ST01027756059	chr1:27756060-27756090	-	GGGGGGGCCCTGATGGCCCCAGGGGGCGGGG<br>.((((.......))))	AHDC1	NM_001029882	chr1:27733343-27802730	non-template	31	Intron_3	--	Yes	--	Yes	--	--	--	Yes	Yes	--	--
G4ST01027766822	chr1:27766823-27766873	-	GGGCTTTTGCAAGCCAAAGGGGCAGTTCTGGGCTACAAGGAGTATTGTGGG<br>..((((.....)))). ..((((......))))	AHDC1	NM_001029882	chr1:27733343-27802730	non-template	51	Intron_1.f	--	Yes	--	Yes	--	--	--	--	--	--	--
G4ST01027766841	chr1:27766842-27766877	-	GGGAGGGCTTTTGCAAGCCAAAGGGGCAGTTCTGGG<br>..((((.....)))).	AHDC1	NM_001029882	chr1:27733343-27802730	non-template	36	Intron_1.f	--	Yes	--	Yes	--	--	--	--	--	--	--
G4ST01027766851	chr1:27766852-27766886	-	GGGGGAGCTGGGAGGGCTTTTGCAAGCCAAAGGGG<br>..((((.....)))).	AHDC1	NM_001029882	chr1:27733343-27802730	non-template	35	Intron_1.f	--	Yes	--	Yes	--	--	--	--	--	--	--
G4ST01027767850	chr1:27767851-27767876	+	GGGAGCCTGCGGCGGGCCGGGCCGGG<br>.(((...)))	AHDC1	NM_001029882	chr1:27733343-27802730	template	26	Intron_1.f	--	Yes	--	Yes	--	--	--	--	Yes	--	Yes
G4ST01027769204	chr1:27769205-27769239	-	GGGAGGGTAGGGAAAGGTTGGCATGCCCAGCTGGG<br>....(((((.....))))).	AHDC1	NM_001029882	chr1:27733343-27802730	non-template	35	Intron_1.f	--	Yes	--	Yes	--	--	--	--	Yes	--	--
G4ST01027774388	chr1:27774389-27774423	+	GGGCGGAGAGGGAAACCAGGCGGCTGGGGGAGGGG<br>...((((....))))	AHDC1	NM_001029882	chr1:27733343-27802730	template	35	Intron_1.f	--	Yes	--	Yes	--	--	--	Yes	Yes	--	--
G4ST01027777038	chr1:27777039-27777066	-	GGGAGAACATCTGGGCTGTGGGCTGGGG<br>(((...)))	AHDC1	NM_001029882	chr1:27733343-27802730	non-template	28	Intron_1.f	--	Yes	--	Yes	--	--	--	Yes	--	--	Yes
G4ST01027782309	chr1:27782310-27782343	-	GGGAGGGCTGGTGGCCTCAAGGACATAGGGAGGG<br>...(((.((....)).))).	AHDC1	NM_001029882	chr1:27733343-27802730	non-template	34	Intron_1.f	--	Yes	--	Yes	--	--	--	--	--	--	--
G4ST01027789323	chr1:27789324-27789355	-	GGGACCCAGGCATCCTGGCGGGTGAGGGCGGG<br>..(((((...))))).	AHDC1	NM_001029882	chr1:27733343-27802730	non-template	32	Intron_1.f	--	Yes	--	Yes	--	--	--	Yes	Yes	--	--
G4ST01027792796	chr1:27792797-27792831	-	GGGGGGATAGCAGGGATTGGTGAGATAGGGAGGGG<br>(((.....))).	AHDC1	NM_001029882	chr1:27733343-27802730	non-template	35	Intron_1.f	--	Yes	--	Yes	--	--	--	--	--	--	--
G4ST01027797539	chr1:27797540-27797580	+	GGGATCCAGGAGGGAGGGGCCAGGAGAGGCCAGGCCTTGGG<br>.((...)) (((.((.....)).)))...	AHDC1	NM_001029882	chr1:27733343-27802730	template	41	Intron_1.f	--	Yes	--	Yes	--	--	--	Yes	Yes	--	Yes
G4ST01027797550	chr1:27797551-27797591	+	GGGAGGGGCCAGGAGAGGCCAGGCCTTGGGCGAAGGAGGGG<br>(((.((.....)).)))...	AHDC1	NM_001029882	chr1:27733343-27802730	template	41	Intron_1.f	--	Yes	--	Yes	--	--	--	Yes	Yes	--	Yes
G4ST01027797554	chr1:27797555-27797596	+	GGGGCCAGGAGAGGCCAGGCCTTGGGCGAAGGAGGGGCTGGG<br>(((.((.....)).)))...	AHDC1	NM_001029882	chr1:27733343-27802730	template	42	Intron_1.f	--	Yes	--	Yes	--	--	--	Yes	Yes	--	Yes
G4ST01027798463	chr1:27798464-27798501	-	GGGGGAGGCGCCGCTGCTGCCGGGAGAGAGAGGGAGGG<br>...(((((....)).)))	AHDC1	NM_001029882	chr1:27733343-27802730	non-template	38	Intron_1.f	--	Yes	--	Yes	--	--	--	Yes	Yes	--	--
G4ST01027798467	chr1:27798468-27798506	-	GGGGAGGGGGAGGCGCCGCTGCTGCCGGGAGAGAGAGGG<br>...(((((....)).)))	AHDC1	NM_001029882	chr1:27733343-27802730	non-template	39	Intron_1.f	--	Yes	--	Yes	--	--	--	Yes	Yes	--	--
G4ST01027798546	chr1:27798547-27798584	-	GGGGGCCTCCTGGGAGTGGAGGGGGCCGGAGCCTGGGG<br>(((....)))..	AHDC1	NM_001029882	chr1:27733343-27802730	non-template	38	Intron_1.f	--	Yes	--	Yes	--	--	--	Yes	Yes	--	--
G4ST01027800930	chr1:27800931-27800957	-	GGGCGGGGGCATGTGCCCGGGCCTGGG<br>(((....))).	AHDC1	NM_001029882	chr1:27733343-27802730	non-template	27	Intron_1.f	--	Yes	--	Yes	--	--	--	Yes	Yes	--	--
G4ST01027801303	chr1:27801304-27801334	-	GGGGGCCAGGGCCAGATGTGGAGGGGTGGGG<br>(((....)))..	AHDC1	NM_001029882	chr1:27733343-27802730	non-template	31	Intron_1.f	--	Yes	--	Yes	--	--	--	Yes	Yes	--	--

G4ST01027814332	chr1:27814333-27814369	-	GGGCCGAGTGAGACCACTAGGGAGCTGGGGAGGGGGG<br>...((((....)))).	FGR	NM_005248	chr1:27811388-27834314	non-template	37	Intron_9	--	Yes	--	Yes	--	--	--	Yes	--	Yes	--
G4ST01027814332	chr1:27814333-27814369	-	GGGCCGAGTGAGACCACTAGGGAGCTGGGGAGGGGGG<br>...((((....)))).	FGR	NM_001042729	chr1:27811388-27825693	non-template	37	Intron_9	--	Yes	--	Yes	--	--	--	Yes	--	Yes	--
G4ST01027814332	chr1:27814333-27814369	-	GGGCCGAGTGAGACCACTAGGGAGCTGGGGAGGGGGG<br>...((((....)))).	FGR	NM_001042747	chr1:27811388-27825338	non-template	37	Intron_9	--	Yes	--	Yes	--	--	--	Yes	--	Yes	--
G4ST01027814339	chr1:27814340-27814376	-	GGGAGCCGGGCCGAGTGAGACCACTAGGGAGCTGGGG<br>...((((....)))).	FGR	NM_005248	chr1:27811388-27834314	non-template	37	Intron_9	--	Yes	--	Yes	--	--	--	Yes	--	Yes	--
G4ST01027814339	chr1:27814340-27814376	-	GGGAGCCGGGCCGAGTGAGACCACTAGGGAGCTGGGG<br>...((((....)))).	FGR	NM_001042729	chr1:27811388-27825693	non-template	37	Intron_9	--	Yes	--	Yes	--	--	--	Yes	--	Yes	--
G4ST01027814339	chr1:27814340-27814376	-	GGGAGCCGGGCCGAGTGAGACCACTAGGGAGCTGGGG<br>...((((....)))).	FGR	NM_001042747	chr1:27811388-27825338	non-template	37	Intron_9	--	Yes	--	Yes	--	--	--	Yes	--	Yes	--
G4ST01027814347	chr1:27814348-27814386	-	GGGCTGAGGTGGGAGCCGGGCCGAGTGAGACCACTAGGG<br>...((((....)))).	FGR	NM_005248	chr1:27811388-27834314	non-template	39	Intron_9	--	Yes	--	Yes	--	--	--	Yes	--	Yes	--
G4ST01027814347	chr1:27814348-27814386	-	GGGCTGAGGTGGGAGCCGGGCCGAGTGAGACCACTAGGG<br>...((((....)))).	FGR	NM_001042729	chr1:27811388-27825693	non-template	39	Intron_9	--	Yes	--	Yes	--	--	--	Yes	--	Yes	--
G4ST01027814347	chr1:27814348-27814386	-	GGGCTGAGGTGGGAGCCGGGCCGAGTGAGACCACTAGGG<br>...((((....)))).	FGR	NM_001042747	chr1:27811388-27825338	non-template	39	Intron_9	--	Yes	--	Yes	--	--	--	Yes	--	Yes	--
G4ST01028071616	chr1:28071617-28071639	-	GGGGCGGGCGGGCGGTCCCCGGG<br>.((...))	C1orf38	NM_004848	chr1:28071642-28085780	template	-2	upstream	Yes	--	--	--	--	--	--	Yes	Yes	Yes	Yes
G4ST01028071616	chr1:28071617-28071639	-	GGGGCGGGCGGGCGGTCCCCGGG<br>.((...))	C1orf38	NM_001105556	chr1:28071642-28085780	template	-2	upstream	Yes	--	--	--	--	--	--	Yes	Yes	Yes	Yes
G4ST01028071616	chr1:28071617-28071639	-	GGGGCGGGCGGGCGGTCCCCGGG<br>.((...))	C1orf38	NM_001039477	chr1:28071642-28085780	template	-2	upstream	Yes	--	--	--	--	--	--	Yes	Yes	Yes	Yes
G4ST01028071627	chr1:28071628-28071652	-	GGGCTCAGACTGAGGGGCGGGCGGG<br>((((...))))	C1orf38	NM_004848	chr1:28071642-28085780	template	11	upstream-Exon_1.f	Yes	Yes	Yes	--	--	Yes	--	Yes	Yes	Yes	--
G4ST01028071627	chr1:28071628-28071652	-	GGGCTCAGACTGAGGGGCGGGCGGG<br>((((...))))	C1orf38	NM_001105556	chr1:28071642-28085780	template	11	upstream-Exon_1.f	Yes	Yes	Yes	--	--	Yes	--	Yes	Yes	Yes	--
G4ST01028071627	chr1:28071628-28071652	-	GGGCTCAGACTGAGGGGCGGGCGGG<br>((((...))))	C1orf38	NM_001039477	chr1:28071642-28085780	template	11	upstream-Exon_1.f	Yes	Yes	Yes	--	--	Yes	--	Yes	Yes	Yes	--
G4ST01028113400	chr1:28113401-28113430	-	GGGCGGGAGCGGAGCTGGGGCGTTTGGGGG<br>(((...))).	RPA2	NM_002946	chr1:28090636-28113823	non-template	30	Intron_1.f	--	Yes	--	Yes	--	--	--	--	Yes	Yes	--
G4ST01028113410	chr1:28113411-28113440	-	GGGAGAGGAAGGGCGGGAGCGGAGCTGGGG<br>(((...))).	RPA2	NM_002946	chr1:28090636-28113823	non-template	30	Intron_1.f	--	Yes	--	Yes	--	--	--	--	Yes	Yes	--
G4ST01028140908	chr1:28140909-28140945	+	GGGTGGTGGGTTGGGCTGAGCTGACAAGGTCGCTGGG<br>.(((.((....)).)))..	SMPDL3B	NM_014474	chr1:28134091-28158250	non-template	37	Intron_1.f	--	Yes	--	Yes	--	--	--	Yes	--	--	--
G4ST01028140908	chr1:28140909-28140945	+	GGGTGGTGGGTTGGGCTGAGCTGACAAGGTCGCTGGG<br>.(((.((....)).)))..	SMPDL3B	NM_001009568	chr1:28134091-28155578	non-template	37	Intron_1.f	--	Yes	--	Yes	--	--	--	Yes	--	--	--
G4ST01028153679	chr1:28153680-28153709	-	GGGTGGGTGGGTGAGTTTGTGCCAAGCGGG<br>...(((((...)))))	SMPDL3B	NM_014474	chr1:28134091-28158250	template	30	Intron_5	--	Yes	--	Yes	--	--	--	--	--	--	Yes
G4ST01028153679	chr1:28153680-28153709	-	GGGTGGGTGGGTGAGTTTGTGCCAAGCGGG<br>...(((((...)))))	SMPDL3B	NM_001009568	chr1:28134091-28155578	template	30	Intron_5	--	Yes	--	Yes	--	--	--	--	--	--	Yes
G4ST01028159953	chr1:28159954-28159991	+	GGGCAGGGCCTTGGGAAGTCACTGACCTCTCAGTGGGG<br>....((((((....))))))	XKR8	NM_018053	chr1:28159091-28167191	non-template	38	Intron_1.f	--	Yes	--	Yes	--	--	--	--	--	--	--
G4ST01028161191	chr1:28161192-28161237	-	GGGGTCCTGTGGGCAAGAGGACATTTGCCTCTTGGGCACTTCTGGG<br>.((((((.......))))))	XKR8	NM_018053	chr1:28159091-28167191	template	46	Intron_1.f	--	Yes	--	Yes	--	--	--	Yes	--	--	--
G4ST01028374408	chr1:28374409-28374438	-	GGGTCCTGGGCCTCAGGTGGGCATCCGGGG<br>((...)).	PTAFR	NM_001164723	chr1:28346264-28375771	non-template	30	Intron_1.f	--	Yes	--	Yes	--	--	--	Yes	Yes	Yes	--
G4ST01028374408	chr1:28374409-28374438	-	GGGTCCTGGGCCTCAGGTGGGCATCCGGGG<br>((...)).	PTAFR	NM_000952	chr1:28346264-28376042	non-template	30	Intron_1.u	--	Yes	--	Yes	--	--	--	Yes	Yes	Yes	--
G4ST01028374408	chr1:28374409-28374438	-	GGGTCCTGGGCCTCAGGTGGGCATCCGGGG<br>((...)).	PTAFR	NM_001164722	chr1:28346264-28393024	non-template	30	Intron_1.u	--	Yes	--	Yes	--	--	--	Yes	Yes	Yes	--
G4ST01028374408	chr1:28374409-28374438	-	GGGTCCTGGGCCTCAGGTGGGCATCCGGGG<br>((...)).	PTAFR	NM_001164721	chr1:28346264-28393034	non-template	30	Intron_2.l	--	Yes	--	Yes	--	--	--	Yes	Yes	Yes	--
G4ST01028665801	chr1:28665802-28665831	-	GGGGGAGGGATAGGGGGCTGCTTGGCAGGG<br>.(((....))).	PHACTR4	NM_023923	chr1:28637248-28699468	template	30	Exon_5	--	Yes	Yes	--	Yes	--	--	--	--	--	--
G4ST01028665801	chr1:28665802-28665831	-	GGGGGAGGGATAGGGGGCTGCTTGGCAGGG<br>.(((....))).	PHACTR4	NM_001048183	chr1:28568680-28699468	template	30	Exon_6	--	Yes	Yes	--	Yes	--	--	--	--	--	--
G4ST01028718585	chr1:28718586-28718611	-	GGGGGACTGGGTGGGTTGCCCAAGGG<br>((....))	RCC1	NR_030725	chr1:28705042-28738295	template	26	Intron_4	--	Yes	--	Yes	--	--	--	--	Yes	--	--
G4ST01028718585	chr1:28718586-28718611	-	GGGGGACTGGGTGGGTTGCCCAAGGG<br>((....))	RCC1	NR_030726	chr1:28705042-28738295	template	26	Intron_4	--	Yes	--	Yes	--	--	--	--	Yes	--	--
G4ST01028718585	chr1:28718586-28718611	-	GGGGGACTGGGTGGGTTGCCCAAGGG<br>((....))	RCC1	NM_001048199	chr1:28705042-28738295	template	26	Intron_3	--	Yes	--	Yes	--	--	--	--	Yes	--	--
G4ST01028718585	chr1:28718586-28718611	-	GGGGGACTGGGTGGGTTGCCCAAGGG<br>((....))	RCC1	NM_001048195	chr1:28717332-28738295	template	26	Intron_1.f	--	Yes	--	Yes	--	--	--	--	Yes	--	--
G4ST01028718585	chr1:28718586-28718611	-	GGGGGACTGGGTGGGTTGCCCAAGGG<br>((....))	RCC1	NM_001048194	chr1:28717332-28738295	template	26	Intron_1.f	--	Yes	--	Yes	--	--	--	--	Yes	--	--
G4ST01028718585	chr1:28718586-28718611	-	GGGGGACTGGGTGGGTTGCCCAAGGG<br>((....))	RCC1	NM_001269	chr1:28717332-28738295	template	26	Intron_1.f	--	Yes	--	Yes	--	--	--	--	Yes	--	--
G4ST01028752143	chr1:28752144-28752179	-	GGGGCTCTGCGGGCGGGGCTCGAAGGCCCGCGCGGG<br>((.((......)).))	TRNAU1AP	NR_003109	chr1:28752116-28777644	template	36	Exon_1.f	--	Yes	Yes	--	--	--	--	Yes	Yes	Yes	Yes
G4ST01028752143	chr1:28752144-28752179	-	GGGGCTCTGCGGGCGGGGCTCGAAGGCCCGCGCGGG<br>((.((......)).))	TRNAU1AP	NM_017846	chr1:28752116-28777644	template	36	Exon_1.f	--	Yes	Yes	--	--	Yes	--	Yes	Yes	Yes	Yes
G4ST01028752439	chr1:28752440-28752472	+	GGGTTCGTGGGAGGGAAAGGTGGTGCTTTGGGG<br>((((......)))).	TRNAU1AP	NR_003109	chr1:28752116-28777644	non-template	33	Intron_1.f	--	Yes	--	Yes	--	--	--	--	Yes	Yes	--
G4ST01028752439	chr1:28752440-28752472	+	GGGTTCGTGGGAGGGAAAGGTGGTGCTTTGGGG<br>((((......)))).	TRNAU1AP	NM_017846	chr1:28752116-28777644	non-template	33	Intron_1.f	--	Yes	--	Yes	--	--	--	--	Yes	Yes	--
G4ST01028752656	chr1:28752657-28752692	+	GGGCTGAACGGGAGGGGCTCTGGAACCCGGAGTGGG<br>.((((((...)))))).	TRNAU1AP	NR_003109	chr1:28752116-28777644	non-template	36	Intron_1.f	--	Yes	--	Yes	--	--	--	Yes	Yes	Yes	--
G4ST01028752656	chr1:28752657-28752692	+	GGGCTGAACGGGAGGGGCTCTGGAACCCGGAGTGGG<br>.((((((...)))))).	TRNAU1AP	NM_017846	chr1:28752116-28777644	non-template	36	Intron_1.f	--	Yes	--	Yes	--	--	--	Yes	Yes	Yes	--
G4ST01028780612	chr1:28780613-28780648	-	GGGGGTTTGGGAACCGGTCTCCTGGGGGATGCGGGG<br>..((((....))))	SNHG12	NR_024127	chr1:28777637-28780953	non-template	36	Intron_1.f	--	Yes	--	Yes	--	--	--	Yes	Yes	Yes	--
G4ST01028780612	chr1:28780613-28780648	-	GGGGGTTTGGGAACCGGTCTCCTGGGGGATGCGGGG<br>..((((....))))	SNORA61	NR_002987	chr1:28778863-28778992	non-template	-1620	upstream	Yes	--	--	--	--	--	--	Yes	Yes	Yes	--
G4ST01028780612	chr1:28780613-28780648	-	GGGGGTTTGGGAACCGGTCTCCTGGGGGATGCGGGG<br>..((((....))))	SNORA44	NR_002976	chr1:28779480-28779611	non-template	-1001	upstream	Yes	--	--	--	--	--	--	Yes	Yes	Yes	--
G4ST01028780612	chr1:28780613-28780648	-	GGGGGTTTGGGAACCGGTCTCCTGGGGGATGCGGGG<br>..((((....))))	SNORA16A	NR_003035	chr1:28780019-28780152	non-template	-460	upstream	Yes	--	--	--	--	--	--	Yes	Yes	Yes	--
G4ST01028780620	chr1:28780621-28780653	-	GGGGTGGGGGTTTGGGAACCGGTCTCCTGGGGG<br>..((((....))))	SNHG12	NR_024127	chr1:28777637-28780953	non-template	33	Intron_1.f	--	Yes	--	Yes	--	--	--	Yes	Yes	Yes	--
G4ST01028780620	chr1:28780621-28780653	-	GGGGTGGGGGTTTGGGAACCGGTCTCCTGGGGG<br>..((((....))))	SNORA61	NR_002987	chr1:28778863-28778992	non-template	-1628	upstream	Yes	--	--	--	--	--	--	Yes	Yes	Yes	--
G4ST01028780620	chr1:28780621-28780653	-	GGGGTGGGGGTTTGGGAACCGGTCTCCTGGGGG<br>..((((....))))	SNORA44	NR_002976	chr1:28779480-28779611	non-template	-1009	upstream	Yes	--	--	--	--	--	--	Yes	Yes	Yes	--
G4ST01028780620	chr1:28780621-28780653	-	GGGGTGGGGGTTTGGGAACCGGTCTCCTGGGGG<br>..((((....))))	SNORA16A	NR_003035	chr1:28780019-28780152	non-template	-468	upstream	Yes	--	--	--	--	--	--	Yes	Yes	Yes	--
G4ST01028791551	chr1:28791552-28791588	+	GGGGCGGGGTCGGGGCGCGGACAAAACCGCCGCGGGG<br>.(((((.........)))))	RAB42	NM_152304	chr1:28791299-28793675	non-template	37	Intron_1.u	--	Yes	--	Yes	--	--	--	Yes	Yes	Yes	Yes

G4ST01028791551	chr1:28791552-28791588	+	GGGGCGGGGTCGGGGCGCGGACAAAACCGCCGCGGGG<br>.(((((.........)))))	RAB42	NM_001193532	chr1:28791594-28793675	non-template	-5	upstream	Yes	--	--	--	--	--	--	Yes	Yes	Yes	Yes
G4ST01028791556	chr1:28791557-28791596	+	GGGGTCGGGGCGCGGACAAAACCGCCGCGGGGCGGCGGGG<br>.(((((.........)))))	RAB42	NM_152304	chr1:28791299-28793675	non-template	40	Intron_1.u	--	Yes	--	Yes	--	--	--	Yes	Yes	Yes	Yes
G4ST01028791556	chr1:28791557-28791596	+	GGGGTCGGGGCGCGGACAAAACCGCCGCGGGGCGGCGGGG<br>.(((((.........)))))	RAB42	NM_001193532	chr1:28791594-28793675	non-template	3	upstream-Exon_1.f	Yes	Yes	Yes	--	--	Yes	--	Yes	Yes	Yes	Yes
G4ST01028841562	chr1:28841563-28841596	-	GGGCAGTGAAGACTGGGGCTTCTTTGGGGACGGG<br>((((....))))	TAF12	NM_001135218	chr1:28802196-28842191	non-template	34	Intron_1.f	--	Yes	--	Yes	--	--	--	Yes	Yes	--	Yes
G4ST01028841562	chr1:28841563-28841596	-	GGGCAGTGAAGACTGGGGCTTCTTTGGGGACGGG<br>((((....))))	TAF12	NM_005644	chr1:28802196-28842191	non-template	34	Intron_1.f	--	Yes	--	Yes	--	--	--	Yes	Yes	--	Yes
G4ST01028867990	chr1:28867991-28868026	+	GGGCGGGCGCGGGGGCCGCGCGGCGGCCGGCGAGGG<br>((((((...)))))).....	GMEB1	NM_006582	chr1:28867827-28914702	non-template	36	Intron_1.f	--	Yes	--	Yes	--	--	--	Yes	Yes	Yes	Yes
G4ST01028867990	chr1:28867991-28868026	+	GGGCGGGCGCGGGGGCCGCGCGGCGGCCGGCGAGGG<br>((((((...)))))).....	GMEB1	NM_024482	chr1:28867827-28914702	non-template	36	Intron_1.f	--	Yes	--	Yes	--	--	--	Yes	Yes	Yes	Yes
G4ST01028867994	chr1:28867995-28868030	+	GGGCGCGGGGGCCGCGCGGCGGCCGGCGAGGGCGGG<br>((((((...)))))).....	GMEB1	NM_006582	chr1:28867827-28914702	non-template	36	Intron_1.f	--	Yes	--	Yes	--	--	--	Yes	Yes	Yes	Yes
G4ST01028867994	chr1:28867995-28868030	+	GGGCGCGGGGGCCGCGCGGCGGCCGGCGAGGGCGGG<br>((((((...)))))).....	GMEB1	NM_024482	chr1:28867827-28914702	non-template	36	Intron_1.f	--	Yes	--	Yes	--	--	--	Yes	Yes	Yes	Yes
G4ST01028868000	chr1:28868001-28868034	+	GGGGGCCGCGCGGCGGCCGGCGAGGGCGGGCGGG<br>((((((...)))))).....	GMEB1	NM_006582	chr1:28867827-28914702	non-template	34	Intron_1.f	--	Yes	--	Yes	--	--	--	Yes	Yes	Yes	Yes
G4ST01028868000	chr1:28868001-28868034	+	GGGGGCCGCGCGGCGGCCGGCGAGGGCGGGCGGG<br>((((((...)))))).....	GMEB1	NM_024482	chr1:28867827-28914702	non-template	34	Intron_1.f	--	Yes	--	Yes	--	--	--	Yes	Yes	Yes	Yes
G4ST01029011291	chr1:29011292-29011322	+	GGGCGGACGCCGGGGGCTGGGCCGGTGCGGG<br>(((...))).	OPRD1	NM_000911	chr1:29011241-29062795	non-template	31	Exon_1.f	--	Yes	Yes	--	--	Yes	--	Yes	Yes	--	--
G4ST01029015385	chr1:29015386-29015418	+	GGGGGATTTGGGAGCCCATGATGGGGGCAGGGG<br>...(((...)))	OPRD1	NM_000911	chr1:29011241-29062795	non-template	33	Intron_1.f	--	Yes	--	Yes	--	--	--	--	--	--	--
G4ST01029015394	chr1:29015395-29015422	+	GGGAGCCCATGATGGGGGCAGGGGAGGG<br>...(((...)))	OPRD1	NM_000911	chr1:29011241-29062795	non-template	28	Intron_1.f	--	Yes	--	Yes	--	--	--	--	--	--	--
G4ST01029015969	chr1:29015970-29016014	-	GGGCCAGGCGGGAGGAAGAGGGGGTCACCTGCCACAGGTGAGGGG<br>...((((((...))))))..	OPRD1	NM_000911	chr1:29011241-29062795	template	45	Intron_1.f	--	Yes	--	Yes	--	--	--	--	--	--	--
G4ST01029055754	chr1:29055755-29055786	-	GGGCCTGGGCAGGGCAACAGGTAGCCTGGGGG<br>...((((...)))).	OPRD1	NM_000911	chr1:29011241-29062795	template	32	Intron_1.f	--	Yes	--	Yes	--	--	--	--	--	--	--
G4ST01029086326	chr1:29086327-29086360	+	GGGGGGCGACCCTCGGTCCCCGGGAGGGACGGGG<br>(((.((((...)))))))	EPB41	NM_001166007	chr1:29086190-29319145	non-template	34	Intron_1.f	--	Yes	--	Yes	--	--	--	Yes	Yes	Yes	Yes
G4ST01029086326	chr1:29086327-29086360	+	GGGGGGCGACCCTCGGTCCCCGGGAGGGACGGGG<br>(((.((((...)))))))	EPB41	NM_203343	chr1:29086190-29319145	non-template	34	Intron_1.f	--	Yes	--	Yes	--	--	--	Yes	Yes	Yes	Yes
G4ST01029086326	chr1:29086327-29086360	+	GGGGGGCGACCCTCGGTCCCCGGGAGGGACGGGG<br>(((.((((...)))))))	EPB41	NM_203342	chr1:29086190-29319145	non-template	34	Intron_1.f	--	Yes	--	Yes	--	--	--	Yes	Yes	Yes	Yes
G4ST01029086326	chr1:29086327-29086360	+	GGGGGGCGACCCTCGGTCCCCGGGAGGGACGGGG<br>(((.((((...)))))))	EPB41	NM_001166005	chr1:29086190-29319145	non-template	34	Intron_1.f	--	Yes	--	Yes	--	--	--	Yes	Yes	Yes	Yes
G4ST01029086326	chr1:29086327-29086360	+	GGGGGGCGACCCTCGGTCCCCGGGAGGGACGGGG<br>(((.((((...)))))))	EPB41	NM_004437	chr1:29086190-29319145	non-template	34	Intron_1.f	--	Yes	--	Yes	--	--	--	Yes	Yes	Yes	Yes
G4ST01029090124	chr1:29090125-29090169	+	GGGGCAGAGGGAGCAACCTCCAGGTCAGCCAGGGCCCACTAGGGG<br>.((.(((....)))..))..	EPB41	NM_001166007	chr1:29086190-29319145	non-template	45	Intron_1.f	--	Yes	--	Yes	--	--	--	Yes	Yes	--	--
G4ST01029090124	chr1:29090125-29090169	+	GGGGCAGAGGGAGCAACCTCCAGGTCAGCCAGGGCCCACTAGGGG<br>.((.(((....)))..))..	EPB41	NM_203343	chr1:29086190-29319145	non-template	45	Intron_1.f	--	Yes	--	Yes	--	--	--	Yes	Yes	--	--
G4ST01029090124	chr1:29090125-29090169	+	GGGGCAGAGGGAGCAACCTCCAGGTCAGCCAGGGCCCACTAGGGG<br>.((.(((....)))..))..	EPB41	NM_203342	chr1:29086190-29319145	non-template	45	Intron_1.f	--	Yes	--	Yes	--	--	--	Yes	Yes	--	--
G4ST01029090124	chr1:29090125-29090169	+	GGGGCAGAGGGAGCAACCTCCAGGTCAGCCAGGGCCCACTAGGGG<br>.((.(((....)))..))..	EPB41	NM_001166005	chr1:29086190-29319145	non-template	45	Intron_1.f	--	Yes	--	Yes	--	--	--	Yes	Yes	--	--
G4ST01029090124	chr1:29090125-29090169	+	GGGGCAGAGGGAGCAACCTCCAGGTCAGCCAGGGCCCACTAGGGG<br>.((.(((....)))..))..	EPB41	NM_004437	chr1:29086190-29319145	non-template	45	Intron_1.f	--	Yes	--	Yes	--	--	--	Yes	Yes	--	--
G4ST01029097805	chr1:29097806-29097842	-	GGGCAAGGGGCAGGAGGGGAGCCAGCTTGGCAGTGGG<br>..((((...))))...	EPB41	NM_001166007	chr1:29086190-29319145	template	37	Intron_1.f	--	Yes	--	Yes	--	--	--	Yes	--	--	--
G4ST01029097805	chr1:29097806-29097842	-	GGGCAAGGGGCAGGAGGGGAGCCAGCTTGGCAGTGGG<br>..((((...))))...	EPB41	NM_203343	chr1:29086190-29319145	template	37	Intron_1.f	--	Yes	--	Yes	--	--	--	Yes	--	--	--
G4ST01029097805	chr1:29097806-29097842	-	GGGCAAGGGGCAGGAGGGGAGCCAGCTTGGCAGTGGG<br>..((((...))))...	EPB41	NM_203342	chr1:29086190-29319145	template	37	Intron_1.f	--	Yes	--	Yes	--	--	--	Yes	--	--	--
G4ST01029097805	chr1:29097806-29097842	-	GGGCAAGGGGCAGGAGGGGAGCCAGCTTGGCAGTGGG<br>..((((...))))...	EPB41	NM_001166005	chr1:29086190-29319145	template	37	Intron_1.f	--	Yes	--	Yes	--	--	--	Yes	--	--	--
G4ST01029097805	chr1:29097806-29097842	-	GGGCAAGGGGCAGGAGGGGAGCCAGCTTGGCAGTGGG<br>..((((...))))...	EPB41	NM_004437	chr1:29086190-29319145	template	37	Intron_1.f	--	Yes	--	Yes	--	--	--	Yes	--	--	--
G4ST01029113547	chr1:29113548-29113578	-	GGGCCGCGCCGGAGGGAAGGAGGGGGCCGGG<br>(((...))).	EPB41	NM_001166006	chr1:29113675-29264320	template	-96	upstream	Yes	--	--	--	--	--	--	Yes	Yes	Yes	--
G4ST01029113547	chr1:29113548-29113578	-	GGGCCGCGCCGGAGGGAAGGAGGGGGCCGGG<br>(((...))).	EPB41	NM_001166007	chr1:29086190-29319145	template	31	Intron_1.f	--	Yes	--	Yes	--	--	--	Yes	Yes	Yes	--
G4ST01029113547	chr1:29113548-29113578	-	GGGCCGCGCCGGAGGGAAGGAGGGGGCCGGG<br>(((...))).	EPB41	NM_203343	chr1:29086190-29319145	template	31	Intron_1.f	--	Yes	--	Yes	--	--	--	Yes	Yes	Yes	--
G4ST01029113547	chr1:29113548-29113578	-	GGGCCGCGCCGGAGGGAAGGAGGGGGCCGGG<br>(((...))).	EPB41	NM_203342	chr1:29086190-29319145	template	31	Intron_1.f	--	Yes	--	Yes	--	--	--	Yes	Yes	Yes	--
G4ST01029113547	chr1:29113548-29113578	-	GGGCCGCGCCGGAGGGAAGGAGGGGGCCGGG<br>(((...))).	EPB41	NM_001166005	chr1:29086190-29319145	template	31	Intron_1.f	--	Yes	--	Yes	--	--	--	Yes	Yes	Yes	--
G4ST01029113547	chr1:29113548-29113578	-	GGGCCGCGCCGGAGGGAAGGAGGGGGCCGGG<br>(((...))).	EPB41	NM_004437	chr1:29086190-29319145	template	31	Intron_1.f	--	Yes	--	Yes	--	--	--	Yes	Yes	Yes	--
G4ST01029315693	chr1:29315694-29315726	+	GGGAGCAGGGAAGCTGCTTTTTGGGAACCAGGG<br>(((...)))...	EPB41	NM_001166007	chr1:29086190-29319145	non-template	33	Intron_17.l	--	Yes	--	Yes	--	--	--	--	--	--	--
G4ST01029315693	chr1:29315694-29315726	+	GGGAGCAGGGAAGCTGCTTTTTGGGAACCAGGG<br>(((...)))...	EPB41	NM_203343	chr1:29086190-29319145	non-template	33	Intron_16.l	--	Yes	--	Yes	--	--	--	--	--	--	--
G4ST01029315693	chr1:29315694-29315726	+	GGGAGCAGGGAAGCTGCTTTTTGGGAACCAGGG<br>(((...)))...	EPB41	NM_203342	chr1:29086190-29319145	non-template	33	Intron_20.l	--	Yes	--	Yes	--	--	--	--	--	--	--
G4ST01029315693	chr1:29315694-29315726	+	GGGAGCAGGGAAGCTGCTTTTTGGGAACCAGGG<br>(((...)))...	EPB41	NM_001166005	chr1:29086190-29319145	non-template	33	Intron_20.l	--	Yes	--	Yes	--	--	--	--	--	--	--
G4ST01029315693	chr1:29315694-29315726	+	GGGAGCAGGGAAGCTGCTTTTTGGGAACCAGGG<br>(((...)))...	EPB41	NM_004437	chr1:29086190-29319145	non-template	33	Intron_18.l	--	Yes	--	Yes	--	--	--	--	--	--	--
G4ST01029317617	chr1:29317618-29317650	+	GGGTATCGGGCAGCAGTCACTGGGGCTCAAGGG<br>...(((...)))	EPB41	NM_001166007	chr1:29086190-29319145	non-template	33	Exon_18.l	--	Yes	Yes	--	--	--	Yes	--	--	--	--
G4ST01029317617	chr1:29317618-29317650	+	GGGTATCGGGCAGCAGTCACTGGGGCTCAAGGG<br>...(((...)))	EPB41	NM_203343	chr1:29086190-29319145	non-template	33	Exon_17.l	--	Yes	Yes	--	--	--	Yes	--	--	--	--
G4ST01029317617	chr1:29317618-29317650	+	GGGTATCGGGCAGCAGTCACTGGGGCTCAAGGG<br>...(((...)))	EPB41	NM_203342	chr1:29086190-29319145	non-template	33	Exon_21.l	--	Yes	Yes	--	--	--	Yes	--	--	--	--
G4ST01029317617	chr1:29317618-29317650	+	GGGTATCGGGCAGCAGTCACTGGGGCTCAAGGG<br>...(((...)))	EPB41	NM_001166005	chr1:29086190-29319145	non-template	33	Exon_21.l	--	Yes	Yes	--	--	--	Yes	--	--	--	--
G4ST01029317617	chr1:29317618-29317650	+	GGGTATCGGGCAGCAGTCACTGGGGCTCAAGGG<br>...(((...)))	EPB41	NM_004437	chr1:29086190-29319145	non-template	33	Exon_19.l	--	Yes	Yes	--	--	--	Yes	--	--	--	--
G4ST01029318687	chr1:29318688-29318715	-	GGGCCCAGCGGTGGAGGGGGGGGTGGGG<br>.(((....))).	TMEM200B	NM_001171868	chr1:29318524-29321600	non-template	28	Exon_2.l	--	Yes	Yes	--	--	--	Yes	--	--	--	Yes
G4ST01029318687	chr1:29318688-29318715	-	GGGCCCAGCGGTGGAGGGGGGGGTGGGG<br>.(((....))).	TMEM200B	NM_001003682	chr1:29318524-29323008	non-template	28	Exon_2.l	--	Yes	Yes	--	--	--	Yes	--	--	--	Yes
G4ST01029318687	chr1:29318688-29318715	-	GGGCCCAGCGGTGGAGGGGGGGGTGGGG<br>.(((....))).	EPB41	NM_001166007	chr1:29086190-29319145	template	28	Exon_18.l	--	Yes	Yes	--	--	--	Yes	--	--	--	Yes

G4ST01029318687	chr1:29318688-29318715	-	GGGCCCAGCGGTGGAGGGGGGGGTGGGG<br>.(((....))).	EPB41	NM_203343	chr1:29086190-29319145	template	28	Exon_17.l	--	Yes	Yes	--	--	--	Yes	--	--	--	Yes
G4ST01029318687	chr1:29318688-29318715	-	GGGCCCAGCGGTGGAGGGGGGGGTGGGG<br>.(((....))).	EPB41	NM_203342	chr1:29086190-29319145	template	28	Exon_21.l	--	Yes	Yes	--	--	--	Yes	--	--	--	Yes
G4ST01029318687	chr1:29318688-29318715	-	GGGCCCAGCGGTGGAGGGGGGGGTGGGG<br>.(((....))).	EPB41	NM_001166005	chr1:29086190-29319145	template	28	Exon_21.l	--	Yes	Yes	--	--	--	Yes	--	--	--	Yes
G4ST01029318687	chr1:29318688-29318715	-	GGGCCCAGCGGTGGAGGGGGGGGTGGGG<br>.(((....))).	EPB41	NM_004437	chr1:29086190-29319145	template	28	Exon_19.l	--	Yes	Yes	--	--	--	Yes	--	--	--	Yes
G4ST01029323892	chr1:29323893-29323923	-	GGGGGTCTCCACGGCCGGGGGGAGGGGCGGG<br>((((.....))))...	TMEM200B	NM_001003682	chr1:29318524-29323008	non-template	-884	upstream	Yes	--	--	--	--	--	--	Yes	Yes	Yes	--
G4ST01029402168	chr1:29402169-29402206	+	GGGATGGGAGGTGGGAAAGACCCAAGACATGGTCTGGG<br>..(((((........)))))	MECR	NM_016011	chr1:29391972-29430041	template	38	Intron_5	--	Yes	--	Yes	--	--	--	Yes	Yes	--	Yes
G4ST01029402168	chr1:29402169-29402206	+	GGGATGGGAGGTGGGAAAGACCCAAGACATGGTCTGGG<br>..(((((........)))))	MECR	NM_001024732	chr1:29391972-29430041	template	38	Intron_5	--	Yes	--	Yes	--	--	--	Yes	Yes	--	Yes
G4ST01029435441	chr1:29435442-29435476	+	GGGGCCTCATGGCGGGGAGGATGCGGGAGCGGGGG<br>(((....))).	PTPRU	NM_001195001	chr1:29435615-29525912	non-template	-138	upstream	Yes	--	--	--	--	--	--	Yes	Yes	Yes	Yes
G4ST01029435441	chr1:29435442-29435476	+	GGGGCCTCATGGCGGGGAGGATGCGGGAGCGGGGG<br>(((....))).	PTPRU	NM_133178	chr1:29435615-29525912	non-template	-138	upstream	Yes	--	--	--	--	--	--	Yes	Yes	Yes	Yes
G4ST01029435441	chr1:29435442-29435476	+	GGGGCCTCATGGCGGGGAGGATGCGGGAGCGGGGG<br>(((....))).	PTPRU	NM_133177	chr1:29435615-29525912	non-template	-138	upstream	Yes	--	--	--	--	--	--	Yes	Yes	Yes	Yes
G4ST01029435441	chr1:29435442-29435476	+	GGGGCCTCATGGCGGGGAGGATGCGGGAGCGGGGG<br>(((....))).	PTPRU	NM_005704	chr1:29435615-29525912	non-template	-138	upstream	Yes	--	--	--	--	--	--	Yes	Yes	Yes	Yes
G4ST01029435678	chr1:29435679-29435715	+	GGGCTCGGGCTCCGGGGGCGGCGTCCCCCGCGCCGGG<br>...(((((.....)))))	PTPRU	NM_001195001	chr1:29435615-29525912	non-template	37	Exon_1.f	--	Yes	Yes	--	--	Yes	--	Yes	Yes	Yes	Yes
G4ST01029435678	chr1:29435679-29435715	+	GGGCTCGGGCTCCGGGGGCGGCGTCCCCCGCGCCGGG<br>...(((((.....)))))	PTPRU	NM_133178	chr1:29435615-29525912	non-template	37	Exon_1.f	--	Yes	Yes	--	--	Yes	--	Yes	Yes	Yes	Yes
G4ST01029435678	chr1:29435679-29435715	+	GGGCTCGGGCTCCGGGGGCGGCGTCCCCCGCGCCGGG<br>...(((((.....)))))	PTPRU	NM_133177	chr1:29435615-29525912	non-template	37	Exon_1.f	--	Yes	Yes	--	--	Yes	--	Yes	Yes	Yes	Yes
G4ST01029435678	chr1:29435679-29435715	+	GGGCTCGGGCTCCGGGGGCGGCGTCCCCCGCGCCGGG<br>...(((((.....)))))	PTPRU	NM_005704	chr1:29435615-29525912	non-template	37	Exon_1.f	--	Yes	Yes	--	--	Yes	--	Yes	Yes	Yes	Yes
G4ST01029435684	chr1:29435685-29435722	+	GGGCTCCGGGGGCGGCGTCCCCCGCGCCGGGCCCCGGG<br>...(((((.....)))))	PTPRU	NM_001195001	chr1:29435615-29525912	non-template	38	Exon_1.f	--	Yes	Yes	--	--	Yes	--	Yes	Yes	Yes	Yes
G4ST01029435684	chr1:29435685-29435722	+	GGGCTCCGGGGGCGGCGTCCCCCGCGCCGGGCCCCGGG<br>...(((((.....)))))	PTPRU	NM_133178	chr1:29435615-29525912	non-template	38	Exon_1.f	--	Yes	Yes	--	--	Yes	--	Yes	Yes	Yes	Yes
G4ST01029435684	chr1:29435685-29435722	+	GGGCTCCGGGGGCGGCGTCCCCCGCGCCGGGCCCCGGG<br>...(((((.....)))))	PTPRU	NM_133177	chr1:29435615-29525912	non-template	38	Exon_1.f	--	Yes	Yes	--	--	Yes	--	Yes	Yes	Yes	Yes
G4ST01029435684	chr1:29435685-29435722	+	GGGCTCCGGGGGCGGCGTCCCCCGCGCCGGGCCCCGGG<br>...(((((.....)))))	PTPRU	NM_005704	chr1:29435615-29525912	non-template	38	Exon_1.f	--	Yes	Yes	--	--	Yes	--	Yes	Yes	Yes	Yes
G4ST01029435691	chr1:29435692-29435727	+	GGGGGCGGCGTCCCCCGCGCCGGGCCCCGGGACGGG<br>...(((((.....)))))	PTPRU	NM_001195001	chr1:29435615-29525912	non-template	36	Exon_1.f	--	Yes	Yes	--	--	Yes	--	Yes	Yes	Yes	Yes
G4ST01029435691	chr1:29435692-29435727	+	GGGGGCGGCGTCCCCCGCGCCGGGCCCCGGGACGGG<br>...(((((.....)))))	PTPRU	NM_133178	chr1:29435615-29525912	non-template	36	Exon_1.f	--	Yes	Yes	--	--	Yes	--	Yes	Yes	Yes	Yes
G4ST01029435691	chr1:29435692-29435727	+	GGGGGCGGCGTCCCCCGCGCCGGGCCCCGGGACGGG<br>...(((((.....)))))	PTPRU	NM_133177	chr1:29435615-29525912	non-template	36	Exon_1.f	--	Yes	Yes	--	--	Yes	--	Yes	Yes	Yes	Yes
G4ST01029435691	chr1:29435692-29435727	+	GGGGGCGGCGTCCCCCGCGCCGGGCCCCGGGACGGG<br>...(((((.....)))))	PTPRU	NM_005704	chr1:29435615-29525912	non-template	36	Exon_1.f	--	Yes	Yes	--	--	Yes	--	Yes	Yes	Yes	Yes
G4ST01029436908	chr1:29436909-29436934	+	GGGCAGGGGAGGGCCGGACCGGCGGG<br>(((...))).	PTPRU	NM_001195001	chr1:29435615-29525912	non-template	26	Intron_1.f	--	Yes	--	Yes	--	--	--	--	Yes	Yes	Yes
G4ST01029436908	chr1:29436909-29436934	+	GGGCAGGGGAGGGCCGGACCGGCGGG<br>(((...))).	PTPRU	NM_133178	chr1:29435615-29525912	non-template	26	Intron_1.f	--	Yes	--	Yes	--	--	--	--	Yes	Yes	Yes
G4ST01029436908	chr1:29436909-29436934	+	GGGCAGGGGAGGGCCGGACCGGCGGG<br>(((...))).	PTPRU	NM_133177	chr1:29435615-29525912	non-template	26	Intron_1.f	--	Yes	--	Yes	--	--	--	--	Yes	Yes	Yes
G4ST01029436908	chr1:29436909-29436934	+	GGGCAGGGGAGGGCCGGACCGGCGGG<br>(((...))).	PTPRU	NM_005704	chr1:29435615-29525912	non-template	26	Intron_1.f	--	Yes	--	Yes	--	--	--	--	Yes	Yes	Yes
G4ST01029437012	chr1:29437013-29437045	+	GGGCGCGGGCAGGGCCTGGCTCGCCGCCGGGGG<br>.(((((.....)))))	PTPRU	NM_001195001	chr1:29435615-29525912	non-template	33	Intron_1.f	--	Yes	--	Yes	--	--	--	Yes	Yes	Yes	--
G4ST01029437012	chr1:29437013-29437045	+	GGGCGCGGGCAGGGCCTGGCTCGCCGCCGGGGG<br>.(((((.....)))))	PTPRU	NM_133178	chr1:29435615-29525912	non-template	33	Intron_1.f	--	Yes	--	Yes	--	--	--	Yes	Yes	Yes	--
G4ST01029437012	chr1:29437013-29437045	+	GGGCGCGGGCAGGGCCTGGCTCGCCGCCGGGGG<br>.(((((.....)))))	PTPRU	NM_133177	chr1:29435615-29525912	non-template	33	Intron_1.f	--	Yes	--	Yes	--	--	--	Yes	Yes	Yes	--
G4ST01029437012	chr1:29437013-29437045	+	GGGCGCGGGCAGGGCCTGGCTCGCCGCCGGGGG<br>.(((((.....)))))	PTPRU	NM_005704	chr1:29435615-29525912	non-template	33	Intron_1.f	--	Yes	--	Yes	--	--	--	Yes	Yes	Yes	--
G4ST01029437761	chr1:29437762-29437791	-	GGGGAGGATGGGGTGGGAGGTCGCCTTGGG<br>(((...))).	PTPRU	NM_001195001	chr1:29435615-29525912	template	30	Intron_1.f	--	Yes	--	Yes	--	--	--	Yes	Yes	Yes	Yes
G4ST01029437761	chr1:29437762-29437791	-	GGGGAGGATGGGGTGGGAGGTCGCCTTGGG<br>(((...))).	PTPRU	NM_133178	chr1:29435615-29525912	template	30	Intron_1.f	--	Yes	--	Yes	--	--	--	Yes	Yes	Yes	Yes
G4ST01029437761	chr1:29437762-29437791	-	GGGGAGGATGGGGTGGGAGGTCGCCTTGGG<br>(((...))).	PTPRU	NM_133177	chr1:29435615-29525912	template	30	Intron_1.f	--	Yes	--	Yes	--	--	--	Yes	Yes	Yes	Yes
G4ST01029437761	chr1:29437762-29437791	-	GGGGAGGATGGGGTGGGAGGTCGCCTTGGG<br>(((...))).	PTPRU	NM_005704	chr1:29435615-29525912	template	30	Intron_1.f	--	Yes	--	Yes	--	--	--	Yes	Yes	Yes	Yes
G4ST01029439755	chr1:29439756-29439783	-	GGGGTGGGGCCAGAGCCTGGGGGAGGGG<br>.((((...))))	PTPRU	NM_001195001	chr1:29435615-29525912	template	28	Intron_1.f	--	Yes	--	Yes	--	--	--	Yes	--	--	--
G4ST01029439755	chr1:29439756-29439783	-	GGGGTGGGGCCAGAGCCTGGGGGAGGGG<br>.((((...))))	PTPRU	NM_133178	chr1:29435615-29525912	template	28	Intron_1.f	--	Yes	--	Yes	--	--	--	Yes	--	--	--
G4ST01029439755	chr1:29439756-29439783	-	GGGGTGGGGCCAGAGCCTGGGGGAGGGG<br>.((((...))))	PTPRU	NM_133177	chr1:29435615-29525912	template	28	Intron_1.f	--	Yes	--	Yes	--	--	--	Yes	--	--	--
G4ST01029439755	chr1:29439756-29439783	-	GGGGTGGGGCCAGAGCCTGGGGGAGGGG<br>.((((...))))	PTPRU	NM_005704	chr1:29435615-29525912	template	28	Intron_1.f	--	Yes	--	Yes	--	--	--	Yes	--	--	--
G4ST01029458221	chr1:29458222-29458247	+	GGGAGGGAGGGTCAGTGTGAGCTGGG<br>..(((....)))	PTPRU	NM_001195001	chr1:29435615-29525912	non-template	26	Intron_3	--	Yes	--	Yes	--	--	--	--	Yes	--	--
G4ST01029458221	chr1:29458222-29458247	+	GGGAGGGAGGGTCAGTGTGAGCTGGG<br>..(((....)))	PTPRU	NM_133178	chr1:29435615-29525912	non-template	26	Intron_3	--	Yes	--	Yes	--	--	--	--	Yes	--	--
G4ST01029458221	chr1:29458222-29458247	+	GGGAGGGAGGGTCAGTGTGAGCTGGG<br>..(((....)))	PTPRU	NM_133177	chr1:29435615-29525912	non-template	26	Intron_3	--	Yes	--	Yes	--	--	--	--	Yes	--	--
G4ST01029458221	chr1:29458222-29458247	+	GGGAGGGAGGGTCAGTGTGAGCTGGG<br>..(((....)))	PTPRU	NM_005704	chr1:29435615-29525912	non-template	26	Intron_3	--	Yes	--	Yes	--	--	--	--	Yes	--	--
G4ST01029459186	chr1:29459187-29459212	+	GGGGCGGGGCCGGCGACGGGGGCGGG<br>.(((....)))	PTPRU	NM_001195001	chr1:29435615-29525912	non-template	26	Intron_6	--	Yes	--	Yes	--	--	--	Yes	--	--	--
G4ST01029459186	chr1:29459187-29459212	+	GGGGCGGGGCCGGCGACGGGGGCGGG<br>.(((....)))	PTPRU	NM_133178	chr1:29435615-29525912	non-template	26	Intron_6	--	Yes	--	Yes	--	--	--	Yes	--	--	--
G4ST01029459186	chr1:29459187-29459212	+	GGGGCGGGGCCGGCGACGGGGGCGGG<br>.(((....)))	PTPRU	NM_133177	chr1:29435615-29525912	non-template	26	Intron_6	--	Yes	--	Yes	--	--	--	Yes	--	--	--
G4ST01029459186	chr1:29459187-29459212	+	GGGGCGGGGCCGGCGACGGGGGCGGG<br>.(((....)))	PTPRU	NM_005704	chr1:29435615-29525912	non-template	26	Intron_6	--	Yes	--	Yes	--	--	--	Yes	--	--	--
G4ST01029498159	chr1:29498160-29498196	+	GGGTGGTGGGAATCAGGGAAAGTGCCACCACTGAGGG<br>..((((....))))..	PTPRU	NM_001195001	chr1:29435615-29525912	non-template	37	Intron_15	--	Yes	--	Yes	--	--	--	--	--	--	--
G4ST01029498159	chr1:29498160-29498196	+	GGGTGGTGGGAATCAGGGAAAGTGCCACCACTGAGGG<br>..((((....))))..	PTPRU	NM_133178	chr1:29435615-29525912	non-template	37	Intron_15	--	Yes	--	Yes	--	--	--	--	--	--	--
G4ST01029498159	chr1:29498160-29498196	+	GGGTGGTGGGAATCAGGGAAAGTGCCACCACTGAGGG<br>..((((....))))..	PTPRU	NM_133177	chr1:29435615-29525912	non-template	37	Intron_15	--	Yes	--	Yes	--	--	--	--	--	--	--

G4ST01029498159	chr1:29498160-29498196	+	GGGTGGTGGGAATCAGGGAAAGTGCCACCACTGAGGG<br>..((((....))))..	PTPRU	NM_005704	chr1:29435615-29525912	non-template	37	Intron_16	--	Yes	--	Yes	--	--	--	--	--	--	--
G4ST01029498166	chr1:29498167-29498200	+	GGGAATCAGGGAAAGTGCCACCACTGAGGGAGGG<br>..((((....))))..	PTPRU	NM_001195001	chr1:29435615-29525912	non-template	34	Intron_15	--	Yes	--	Yes	--	--	--	--	--	--	--
G4ST01029498166	chr1:29498167-29498200	+	GGGAATCAGGGAAAGTGCCACCACTGAGGGAGGG<br>..((((....))))..	PTPRU	NM_133178	chr1:29435615-29525912	non-template	34	Intron_15	--	Yes	--	Yes	--	--	--	--	--	--	--
G4ST01029498166	chr1:29498167-29498200	+	GGGAATCAGGGAAAGTGCCACCACTGAGGGAGGG<br>..((((....))))..	PTPRU	NM_133177	chr1:29435615-29525912	non-template	34	Intron_15	--	Yes	--	Yes	--	--	--	--	--	--	--
G4ST01029498166	chr1:29498167-29498200	+	GGGAATCAGGGAAAGTGCCACCACTGAGGGAGGG<br>..((((....))))..	PTPRU	NM_005704	chr1:29435615-29525912	non-template	34	Intron_16	--	Yes	--	Yes	--	--	--	--	--	--	--
G4ST01029502772	chr1:29502773-29502806	+	GGGAGGGAATGTGTTCAGGACCACACAGGGTGGG<br>..(((((........)))))	PTPRU	NM_001195001	chr1:29435615-29525912	non-template	34	Intron_15	--	Yes	--	Yes	--	--	--	--	--	--	--
G4ST01029502772	chr1:29502773-29502806	+	GGGAGGGAATGTGTTCAGGACCACACAGGGTGGG<br>..(((((........)))))	PTPRU	NM_133178	chr1:29435615-29525912	non-template	34	Intron_15	--	Yes	--	Yes	--	--	--	--	--	--	--
G4ST01029502772	chr1:29502773-29502806	+	GGGAGGGAATGTGTTCAGGACCACACAGGGTGGG<br>..(((((........)))))	PTPRU	NM_133177	chr1:29435615-29525912	non-template	34	Intron_15	--	Yes	--	Yes	--	--	--	--	--	--	--
G4ST01029502772	chr1:29502773-29502806	+	GGGAGGGAATGTGTTCAGGACCACACAGGGTGGG<br>..(((((........)))))	PTPRU	NM_005704	chr1:29435615-29525912	non-template	34	Intron_16	--	Yes	--	Yes	--	--	--	--	--	--	--
G4ST01029506554	chr1:29506555-29506595	-	GGGAGGCTAGGGAGGACTCCTTGGGAATGGCGGAGCCAGGG<br>(((...))). ..((((...))))	PTPRU	NM_001195001	chr1:29435615-29525912	template	41	Intron_18	--	Yes	--	Yes	--	--	--	--	--	--	--
G4ST01029506554	chr1:29506555-29506595	-	GGGAGGCTAGGGAGGACTCCTTGGGAATGGCGGAGCCAGGG<br>(((...))). ..((((...))))	PTPRU	NM_133178	chr1:29435615-29525912	template	41	Intron_18	--	Yes	--	Yes	--	--	--	--	--	--	--
G4ST01029506554	chr1:29506555-29506595	-	GGGAGGCTAGGGAGGACTCCTTGGGAATGGCGGAGCCAGGG<br>(((...))). ..((((...))))	PTPRU	NM_133177	chr1:29435615-29525912	template	41	Intron_19	--	Yes	--	Yes	--	--	--	--	--	--	--
G4ST01029506554	chr1:29506555-29506595	-	GGGAGGCTAGGGAGGACTCCTTGGGAATGGCGGAGCCAGGG<br>(((...))). ..((((...))))	PTPRU	NM_005704	chr1:29435615-29525912	template	41	Intron_19	--	Yes	--	Yes	--	--	--	--	--	--	--
G4ST01029519580	chr1:29519581-29519611	-	GGGTTGACAGGGTGGGCCCAGGCCTCTGGGG<br>..(((....)))	PTPRU	NM_001195001	chr1:29435615-29525912	template	31	Intron_25	--	Yes	--	Yes	--	--	--	--	--	--	--
G4ST01029519580	chr1:29519581-29519611	-	GGGTTGACAGGGTGGGCCCAGGCCTCTGGGG<br>..(((....)))	PTPRU	NM_133178	chr1:29435615-29525912	template	31	Intron_25	--	Yes	--	Yes	--	--	--	--	--	--	--
G4ST01029519580	chr1:29519581-29519611	-	GGGTTGACAGGGTGGGCCCAGGCCTCTGGGG<br>..(((....)))	PTPRU	NM_133177	chr1:29435615-29525912	template	31	Intron_26	--	Yes	--	Yes	--	--	--	--	--	--	--
G4ST01029519580	chr1:29519581-29519611	-	GGGTTGACAGGGTGGGCCCAGGCCTCTGGGG<br>..(((....)))	PTPRU	NM_005704	chr1:29435615-29525912	template	31	Intron_26	--	Yes	--	Yes	--	--	--	--	--	--	--
G4ST01029521068	chr1:29521069-29521099	-	GGGCCCAGCAGAAAGCTGGGGTGGGAAGGGG<br>..((((.....))))	PTPRU	NM_001195001	chr1:29435615-29525912	template	31	Intron_26	--	Yes	--	Yes	--	--	--	--	--	--	--
G4ST01029521068	chr1:29521069-29521099	-	GGGCCCAGCAGAAAGCTGGGGTGGGAAGGGG<br>..((((.....))))	PTPRU	NM_133178	chr1:29435615-29525912	template	31	Intron_26	--	Yes	--	Yes	--	--	--	--	--	--	--
G4ST01029521068	chr1:29521069-29521099	-	GGGCCCAGCAGAAAGCTGGGGTGGGAAGGGG<br>..((((.....))))	PTPRU	NM_133177	chr1:29435615-29525912	template	31	Intron_27	--	Yes	--	Yes	--	--	--	--	--	--	--
G4ST01029521068	chr1:29521069-29521099	-	GGGCCCAGCAGAAAGCTGGGGTGGGAAGGGG<br>..((((.....))))	PTPRU	NM_005704	chr1:29435615-29525912	template	31	Intron_27	--	Yes	--	Yes	--	--	--	--	--	--	--
G4ST01030962766	chr1:30962767-30962803	-	GGGTTAGGGCAGGGACTTTACCCATTTGTAAAATGGG<br>..((((((......))))))..	LOC100129196	NR_034182	chr1:30964206-30972180	template	-1402	upstream	Yes	--	--	--	--	--	--	Yes	--	--	--
G4ST01030962766	chr1:30962767-30962803	-	GGGTTAGGGCAGGGACTTTACCCATTTGTAAAATGGG<br>..((((((......))))))..	MATN1	NM_002379	chr1:30956711-30969019	non-template	37	Intron_3	--	Yes	--	Yes	--	--	--	Yes	--	--	--
G4ST01030964121	chr1:30964122-30964154	-	GGGGGCGGAGCCAGGGCAGGGGCGGAGAAGGGG<br>(((...))).	LOC100129196	NR_034182	chr1:30964206-30972180	template	-51	upstream	Yes	--	--	--	--	--	--	Yes	Yes	Yes	--
G4ST01030964121	chr1:30964122-30964154	-	GGGGGCGGAGCCAGGGCAGGGGCGGAGAAGGGG<br>(((...))).	MATN1	NM_002379	chr1:30956711-30969019	non-template	33	Intron_3	--	Yes	--	Yes	--	--	--	Yes	Yes	Yes	--
G4ST01030964132	chr1:30964133-30964162	-	GGGCAGGCGGGGGCGGAGCCAGGGCAGGGG<br>(((...))).	LOC100129196	NR_034182	chr1:30964206-30972180	template	-43	upstream	Yes	--	--	--	--	--	--	Yes	Yes	Yes	--
G4ST01030964132	chr1:30964133-30964162	-	GGGCAGGCGGGGGCGGAGCCAGGGCAGGGG<br>(((...))).	MATN1	NM_002379	chr1:30956711-30969019	non-template	30	Intron_3	--	Yes	--	Yes	--	--	--	Yes	Yes	Yes	--
G4ST01030964760	chr1:30964761-30964789	+	GGGAGGCCCGGCCGGGGCTGGGTGGGGGG<br>.(((...))).	LOC100129196	NR_034182	chr1:30964206-30972180	non-template	29	Exon_1.f	--	Yes	Yes	--	--	--	--	Yes	Yes	Yes	--
G4ST01030964760	chr1:30964761-30964789	+	GGGAGGCCCGGCCGGGGCTGGGTGGGGGG<br>.(((...))).	MATN1	NM_002379	chr1:30956711-30969019	template	29	Intron_2	--	Yes	--	Yes	--	--	--	Yes	Yes	Yes	--
G4ST01030991470	chr1:30991471-30991497	-	GGGGCTTGGTTGCCAGGGAAGGGTGGG<br>...(((...)))	LAPTM5	NM_006762	chr1:30977902-31003270	non-template	27	Intron_1.f	--	Yes	--	Yes	--	--	--	Yes	Yes	Yes	--
G4ST01030991474	chr1:30991475-30991503	-	GGGGCTGGGGCTTGGTTGCCAGGGAAGGG<br>...(((...)))	LAPTM5	NM_006762	chr1:30977902-31003270	non-template	29	Intron_1.f	--	Yes	--	Yes	--	--	--	Yes	Yes	Yes	--
G4ST01031128136	chr1:31128137-31128165	-	GGGCTCTGAGAGAGGATGGGGTGGGAGGG<br>((((...))))....	SDC3	NM_014654	chr1:31114900-31154067	non-template	29	Intron_1.f	--	Yes	--	Yes	--	--	--	--	--	--	--
G4ST01031129517	chr1:31129518-31129545	-	GGGCAGCTGTGGCTGGGTGGGGGTGGGG<br>.(((....)))	SDC3	NM_014654	chr1:31114900-31154067	non-template	28	Intron_1.f	--	Yes	--	Yes	--	--	--	Yes	--	--	--
G4ST01031129522	chr1:31129523-31129567	-	GGGACTGCCGGCTTCCTGGCCAGGGCAGCTGTGGCTGGGTGGGGG<br>...(((((....))))).. .(((....)))	SDC3	NM_014654	chr1:31114900-31154067	non-template	45	Intron_1.f	--	Yes	--	Yes	--	--	--	Yes	--	--	--
G4ST01031129528	chr1:31129529-31129573	-	GGGTGTGGGACTGCCGGCTTCCTGGCCAGGGCAGCTGTGGCTGGG<br>...(((((....))))).. .(((....)))	SDC3	NM_014654	chr1:31114900-31154067	non-template	45	Intron_1.f	--	Yes	--	Yes	--	--	--	Yes	--	--	--
G4ST01031141054	chr1:31141055-31141083	+	GGGAACTGTGAGTTCTGGGGCCGGGAGGG<br>((((...))))....	SDC3	NM_014654	chr1:31114900-31154067	template	29	Intron_1.f	--	Yes	--	Yes	--	--	--	--	Yes	--	--
G4ST01031151715	chr1:31151716-31151755	-	GGGGGAGGGCAGGTACTGGGGCGCCTGGTGAGGTGGTGGG<br>(((...))) .(((((....)))))..	SDC3	NM_014654	chr1:31114900-31154067	non-template	40	Intron_1.f	--	Yes	--	Yes	--	--	--	--	--	--	Yes
G4ST01031151734	chr1:31151735-31151762	-	GGGGCCTGGGGGAGGGCAGGTACTGGGG<br>(((...)))	SDC3	NM_014654	chr1:31114900-31154067	non-template	28	Intron_1.f	--	Yes	--	Yes	--	--	--	--	--	--	Yes
G4ST01031153676	chr1:31153677-31153706	+	GGGGGAGATTCCGGAGGGGGGGGAGCAGGG<br>(((...)))...	SDC3	NM_014654	chr1:31114900-31154067	template	30	Intron_1.f	--	Yes	--	Yes	--	--	--	--	--	--	--
G4ST01031153801	chr1:31153802-31153837	+	GGGTCGCGGGCGGGGACCGGCACCCCGCGCGGGGGG<br>..(((((.....)).))).	SDC3	NM_014654	chr1:31114900-31154067	template	36	Intron_1.f	--	Yes	--	Yes	--	--	--	--	--	--	Yes
G4ST01031153808	chr1:31153809-31153848	+	GGGCGGGGACCGGCACCCCGCGCGGGGGGCGGCCCCGGGG<br>..(((((.....)).))).	SDC3	NM_014654	chr1:31114900-31154067	template	40	Intron_1.f	--	Yes	--	Yes	--	--	--	--	--	--	Yes
G4ST01031154287	chr1:31154288-31154328	-	GGGGTCAGAGGGCGCGGAGCCCCGCGGGGGGCGCACTGGGG<br>(((((....)))))..	SDC3	NM_014654	chr1:31114900-31154067	non-template	-220	upstream	Yes	--	--	--	--	--	--	Yes	--	Yes	Yes
G4ST01031154297	chr1:31154298-31154334	-	GGGACTGGGGTCAGAGGGCGCGGAGCCCCGCGGGGGG<br>(((((....)))))..	SDC3	NM_014654	chr1:31114900-31154067	non-template	-230	upstream	Yes	--	--	--	--	--	--	Yes	--	Yes	Yes
G4ST01031247150	chr1:31247151-31247190	-	GGGGAGGGCTGGTGAGGGCATTGTGAAAAAGGCAATGGGG<br>((((((.......))))))	PUM1	NM_001020658	chr1:31176940-31311151	non-template	40	Intron_5	--	Yes	--	Yes	--	--	--	--	--	--	--
G4ST01031247150	chr1:31247151-31247190	-	GGGGAGGGCTGGTGAGGGCATTGTGAAAAAGGCAATGGGG<br>((((((.......))))))	PUM1	NM_014676	chr1:31176940-31311151	non-template	40	Intron_5	--	Yes	--	Yes	--	--	--	--	--	--	--
G4ST01031268053	chr1:31268054-31268089	-	GGGACCCAAAATAATAATTTTGGGATTGGGATTGGG<br>....(((((....)))))	PUM1	NM_001020658	chr1:31176940-31311151	non-template	36	Intron_3	--	Yes	--	Yes	--	--	--	Yes	--	--	--
G4ST01031268053	chr1:31268054-31268089	-	GGGACCCAAAATAATAATTTTGGGATTGGGATTGGG<br>....(((((....)))))	PUM1	NM_014676	chr1:31176940-31311151	non-template	36	Intron_3	--	Yes	--	Yes	--	--	--	Yes	--	--	--
G4ST01031427115	chr1:31427116-31427153	+	GGGGTGGGCGCGGGGCAGAGGCTACCCCGACCTGCGGG<br>((((.((.....))...))))	NKAIN1	NM_024522	chr1:31425179-31485321	template	38	Intron_6.l-Exon_7.l	--	Yes	Yes	Yes	Yes	--	Yes	Yes	Yes	--	--
G4ST01031535029	chr1:31535030-31535081	-	GGGGAATTTTTTAAACTTCACAGGGGTAGATCTTTTATCTGGGCCATCTGGG<br>(((.((....)).))).... ..((((.....))))	SNRNP40	NM_004814	chr1:31505002-31542231	non-template	52	Intron_3	--	Yes	--	Yes	--	--	--	Yes	--	--	--
G4ST01031542598	chr1:31542599-31542628	+	GGGTGCTGACAGCAAGGGGTGGGAGGCGGG<br>((((...))))..	SNRNP40	NM_004814	chr1:31505002-31542231	template	-367	upstream	Yes	--	--	--	--	--	--	Yes	Yes	Yes	--

G4ST01031542598	chr1:31542599-31542628	+	GGGTGCTGACAGCAAGGGGTGGGAGGCGGG<br>((((...))))..	ZCCHC17	NM_016505	chr1:31542429-31610367	non-template	30	Intron_1.f	--	Yes	--	Yes	--	--	--	Yes	Yes	Yes	--
G4ST01031658953	chr1:31658954-31658996	+	GGGCAGCTGCGGTCCCGGCTCGGGTTTCCGCGGGCAGGAGGGG<br>.(((((......))))).	SERINC2	NM_001199039	chr1:31659247-31680114	non-template	-250	upstream	Yes	--	--	--	--	--	--	--	Yes	Yes	--
G4ST01031658953	chr1:31658954-31658996	+	GGGCAGCTGCGGTCCCGGCTCGGGTTTCCGCGGGCAGGAGGGG<br>.(((((......))))).	SERINC2	NM_001199038	chr1:31654999-31680114	non-template	43	Intron_2	--	Yes	--	Yes	--	--	--	--	Yes	Yes	--
G4ST01031658953	chr1:31658954-31658996	+	GGGCAGCTGCGGTCCCGGCTCGGGTTTCCGCGGGCAGGAGGGG<br>.(((((......))))).	SERINC2	NM_018565	chr1:31659247-31680114	non-template	-250	upstream	Yes	--	--	--	--	--	--	--	Yes	Yes	--
G4ST01031658953	chr1:31658954-31658996	+	GGGCAGCTGCGGTCCCGGCTCGGGTTTCCGCGGGCAGGAGGGG<br>.(((((......))))).	SERINC2	NM_001199037	chr1:31655608-31680114	non-template	43	Intron_1.f	--	Yes	--	Yes	--	--	--	--	Yes	Yes	--
G4ST01031658953	chr1:31658954-31658996	+	GGGCAGCTGCGGTCCCGGCTCGGGTTTCCGCGGGCAGGAGGGG<br>.(((((......))))).	SERINC2	NM_178865	chr1:31658550-31680114	non-template	43	Intron_1.f	--	Yes	--	Yes	--	--	--	--	Yes	Yes	--
G4ST01031679735	chr1:31679736-31679769	-	GGGGCAGGTCCTGGAGCTGGGGCAGGGGTGGGGG<br>.(((.((...)).)))	SERINC2	NM_001199039	chr1:31659247-31680114	template	34	Exon_11.l	--	Yes	Yes	--	--	--	Yes	Yes	Yes	--	--
G4ST01031679735	chr1:31679736-31679769	-	GGGGCAGGTCCTGGAGCTGGGGCAGGGGTGGGGG<br>.(((.((...)).)))	SERINC2	NM_001199038	chr1:31654999-31680114	template	34	Exon_11.l	--	Yes	Yes	--	--	--	Yes	Yes	Yes	--	--
G4ST01031679735	chr1:31679736-31679769	-	GGGGCAGGTCCTGGAGCTGGGGCAGGGGTGGGGG<br>.(((.((...)).)))	SERINC2	NM_018565	chr1:31659247-31680114	template	34	Exon_10.l	--	Yes	Yes	--	--	--	Yes	Yes	Yes	--	--
G4ST01031679735	chr1:31679736-31679769	-	GGGGCAGGTCCTGGAGCTGGGGCAGGGGTGGGGG<br>.(((.((...)).)))	SERINC2	NM_001199037	chr1:31655608-31680114	template	34	Exon_10.l	--	Yes	Yes	--	--	--	Yes	Yes	Yes	--	--
G4ST01031679735	chr1:31679736-31679769	-	GGGGCAGGTCCTGGAGCTGGGGCAGGGGTGGGGG<br>.(((.((...)).)))	SERINC2	NM_178865	chr1:31658550-31680114	template	34	Exon_10.l	--	Yes	Yes	--	--	--	Yes	Yes	Yes	--	--
G4ST01031755458	chr1:31755459-31755493	+	GGGCACAAGCCCTGCTTGGGGTGGAGCAGGGAGGG<br>..(((((...)))))	LOC284551	NR_027085	chr1:31756623-31762433	non-template	-1129	upstream	Yes	--	--	--	--	--	--	--	--	--	--
G4ST01031815133	chr1:31815134-31815164	-	GGGCCCTGGCTATAGGGGGATGGGGACTGGG<br>.((((....))))	TINAGL1	NM_022164	chr1:31814673-31825874	template	31	Intron_1.f	--	Yes	--	Yes	--	--	--	--	Yes	--	--
G4ST01031815133	chr1:31815134-31815164	-	GGGCCCTGGCTATAGGGGGATGGGGACTGGG<br>.((((....))))	TINAGL1	NM_001204414	chr1:31814673-31825874	template	31	Intron_1.f	--	Yes	--	Yes	--	--	--	--	Yes	--	--
G4ST01031815133	chr1:31815134-31815164	-	GGGCCCTGGCTATAGGGGGATGGGGACTGGG<br>.((((....))))	TINAGL1	NM_001204415	chr1:31816333-31825874	template	-1168	upstream	Yes	--	--	--	--	--	--	--	Yes	--	--
G4ST01031818604	chr1:31818605-31818632	+	GGGGGCGAGGGTGGGGGAGTGTCCAGGG<br>(((...))).	TINAGL1	NM_022164	chr1:31814673-31825874	non-template	28	Intron_3	--	Yes	--	Yes	--	--	--	Yes	--	--	--
G4ST01031818604	chr1:31818605-31818632	+	GGGGGCGAGGGTGGGGGAGTGTCCAGGG<br>(((...))).	TINAGL1	NM_001204414	chr1:31814673-31825874	non-template	28	Intron_3	--	Yes	--	Yes	--	--	--	Yes	--	--	--
G4ST01031818604	chr1:31818605-31818632	+	GGGGGCGAGGGTGGGGGAGTGTCCAGGG<br>(((...))).	TINAGL1	NM_001204415	chr1:31816333-31825874	non-template	28	Intron_2	--	Yes	--	Yes	--	--	--	Yes	--	--	--
G4ST01031818745	chr1:31818746-31818776	-	GGGGAGGGCCAGAGGAGTCTGCAGGGCCGGG<br>.((((....))))..	TINAGL1	NM_022164	chr1:31814673-31825874	template	31	Intron_3	--	Yes	--	Yes	--	--	--	Yes	--	--	--
G4ST01031818745	chr1:31818746-31818776	-	GGGGAGGGCCAGAGGAGTCTGCAGGGCCGGG<br>.((((....))))..	TINAGL1	NM_001204414	chr1:31814673-31825874	template	31	Intron_3	--	Yes	--	Yes	--	--	--	Yes	--	--	--
G4ST01031818745	chr1:31818746-31818776	-	GGGGAGGGCCAGAGGAGTCTGCAGGGCCGGG<br>.((((....))))..	TINAGL1	NM_001204415	chr1:31816333-31825874	template	31	Intron_2	--	Yes	--	Yes	--	--	--	Yes	--	--	--
G4ST01031818750	chr1:31818751-31818788	-	GGGGGCACTGCTGGGGAGGGCCAGAGGAGTCTGCAGGG<br>.((((....))))..	TINAGL1	NM_022164	chr1:31814673-31825874	template	38	Intron_3	--	Yes	--	Yes	--	--	--	Yes	--	--	--
G4ST01031818750	chr1:31818751-31818788	-	GGGGGCACTGCTGGGGAGGGCCAGAGGAGTCTGCAGGG<br>.((((....))))..	TINAGL1	NM_001204414	chr1:31814673-31825874	template	38	Intron_3	--	Yes	--	Yes	--	--	--	Yes	--	--	--
G4ST01031818750	chr1:31818751-31818788	-	GGGGGCACTGCTGGGGAGGGCCAGAGGAGTCTGCAGGG<br>.((((....))))..	TINAGL1	NM_001204415	chr1:31816333-31825874	template	38	Intron_2	--	Yes	--	Yes	--	--	--	Yes	--	--	--
G4ST01031825121	chr1:31825122-31825157	+	GGGCGTCTGGGGCCGCGTGGGCATGGAGGACATGGG<br>.(((.....)))	TINAGL1	NM_022164	chr1:31814673-31825874	non-template	36	Exon_12.l	--	Yes	Yes	--	Yes	--	--	--	--	--	--
G4ST01031825121	chr1:31825122-31825157	+	GGGCGTCTGGGGCCGCGTGGGCATGGAGGACATGGG<br>.(((.....)))	TINAGL1	NM_001204414	chr1:31814673-31825874	non-template	36	Exon_11.l	--	Yes	Yes	--	Yes	--	--	--	--	--	--
G4ST01031825121	chr1:31825122-31825157	+	GGGCGTCTGGGGCCGCGTGGGCATGGAGGACATGGG<br>.(((.....)))	TINAGL1	NM_001204415	chr1:31816333-31825874	non-template	36	Exon_11.l	--	Yes	Yes	--	Yes	--	--	--	--	--	--
G4ST01031855830	chr1:31855831-31855856	-	GGGCTGCGCGCTGCGGGGAGGGAGGG<br>...(((...)))	HCRTR1	NM_001525	chr1:31855888-31865506	template	-31	upstream	Yes	--	--	--	--	--	--	--	--	--	Yes
G4ST01031855834	chr1:31855835-31855866	-	GGGCTCCGCCGGGCTGCGCGCTGCGGGGAGGG<br>...(((...)))	HCRTR1	NM_001525	chr1:31855888-31865506	template	-21	upstream	Yes	--	--	--	--	--	--	--	--	--	Yes
G4ST01031903653	chr1:31903654-31903694	-	GGGGTAGGATGGTCCTGGGGAGAAGAGGGGAGAGGCAGGGG<br>..((((...)))).	COL16A1	NM_001856	chr1:31890435-31942355	non-template	41	Intron_55	--	Yes	--	Yes	--	--	--	--	Yes	--	--
G4ST01031910873	chr1:31910874-31910907	-	GGGACTGGGCTTGGGGCTGCCCTATGCAGGAGGG<br>.((((.....))))..	COL16A1	NM_001856	chr1:31890435-31942355	non-template	34	Intron_45	--	Yes	--	Yes	--	--	--	--	--	--	--
G4ST01031929998	chr1:31929999-31930031	+	GGGGCTCTGAGGCTGGGAGTGAGGGGGCCTGGG<br>(((....))).	COL16A1	NM_001856	chr1:31890435-31942355	template	33	Intron_17	--	Yes	--	Yes	--	--	--	--	--	--	--
G4ST01031930895	chr1:31930896-31930926	-	GGGCCAATATGGGGCCAGCCCCTGGGGAGGG<br>..(((...)))	COL16A1	NM_001856	chr1:31890435-31942355	non-template	31	Intron_14	--	Yes	--	Yes	--	--	--	--	--	--	--
G4ST01031934208	chr1:31934209-31934242	+	GGGGTTTACAGGGGCACAGAGGGCAGACTGAGGG<br>((...)).	COL16A1	NM_001856	chr1:31890435-31942355	template	34	Intron_9	--	Yes	--	Yes	--	--	--	--	--	--	--
G4ST01031943716	chr1:31943717-31943754	+	GGGTGCTGCCCCAGCAGTGGGCACTCGGGAGAGGTGGG<br>..((((....)))).	COL16A1	NM_001856	chr1:31890435-31942355	template	-1361	upstream	Yes	--	--	--	--	--	--	--	Yes	--	--
G4ST01031965324	chr1:31965325-31965352	-	GGGGAGGGGGGAGGGGAACTTTGTTGGG<br>.(((...)))	BAI2	NM_001703	chr1:31965305-32002235	non-template	28	Exon_33.l	--	Yes	Yes	--	--	--	Yes	--	--	--	Yes
G4ST01031966549	chr1:31966550-31966579	-	GGGGCTGGCAGCAGGGAGGGATGTGGGGGG<br>(((...))).	BAI2	NM_001703	chr1:31965305-32002235	non-template	30	Intron_30	--	Yes	--	Yes	--	--	--	--	--	--	Yes
G4ST01031970469	chr1:31970470-31970505	-	GGGGTAGGCCATGCCTGGGCTCTGCTGGGCCATGGG<br>..((((...))))	BAI2	NM_001703	chr1:31965305-32002235	non-template	36	Intron_27	--	Yes	--	Yes	--	--	--	--	--	--	Yes
G4ST01031970476	chr1:31970477-31970510	-	GGGCTGGGGTAGGCCATGCCTGGGCTCTGCTGGG<br>..((((...))))	BAI2	NM_001703	chr1:31965305-32002235	non-template	34	Intron_27	--	Yes	--	Yes	--	--	--	--	--	--	Yes
G4ST01031970664	chr1:31970665-31970689	-	GGGCAGGGCCTGGGCAGATTCTGGG<br>.((...))	BAI2	NM_001703	chr1:31965305-32002235	non-template	25	Intron_27	--	Yes	--	Yes	--	--	--	--	--	--	Yes
G4ST01031970920	chr1:31970921-31970956	+	GGGGAAGGTGATGACACCTGGGCAGGGTGCAACGGG<br>..(((((....)))))	BAI2	NM_001703	chr1:31965305-32002235	template	36	Intron_26	--	Yes	--	Yes	--	--	--	--	--	--	--
G4ST01031974112	chr1:31974113-31974135	-	GGGTGGGGAGGGAGGCTGCTGGG<br>((....))	BAI2	NM_001703	chr1:31965305-32002235	non-template	23	Intron_22	--	Yes	--	Yes	--	--	--	--	--	--	--
G4ST01031975909	chr1:31975910-31975949	+	GGGGGACCGAGGGGGAGCCCGCCAGCTCCAGGGTCTGGGG<br>(((((......))))).	BAI2	NM_001703	chr1:31965305-32002235	template	40	Intron_18-Exon_19	--	Yes	Yes	Yes	Yes	--	--	--	--	--	--
G4ST01031975919	chr1:31975920-31975957	+	GGGGGAGCCCGCCAGCTCCAGGGTCTGGGGAAGATGGG<br>(((((......))))).	BAI2	NM_001703	chr1:31965305-32002235	template	38	Intron_18-Exon_19	--	Yes	Yes	Yes	Yes	--	--	--	--	--	--
G4ST01031993328	chr1:31993329-31993366	-	GGGCCGGGGACTGACCAGGAGACCTGGAGGGAACAGGG<br>......(((((...))))).	BAI2	NM_001703	chr1:31965305-32002235	non-template	38	Intron_4	--	Yes	--	Yes	--	--	--	Yes	--	--	--
G4ST01031994492	chr1:31994493-31994529	+	GGGCAGCGGGCGCCAGCAGGCGCGGGGCCTCGGAGGG<br>((((....))))..	BAI2	NM_001703	chr1:31965305-32002235	template	37	Exon_4	--	Yes	Yes	--	Yes	--	--	Yes	--	--	--
G4ST01031998536	chr1:31998537-31998574	-	GGGCAGAGTGAGATTCTTGGGTTTGCTGGGGTCAGGGG<br>.((((.....)))).	MIR4254	NR_036216	chr1:31996848-31996923	non-template	-1613	upstream	Yes	--	--	--	--	--	--	--	--	--	--
G4ST01031998536	chr1:31998537-31998574	-	GGGCAGAGTGAGATTCTTGGGTTTGCTGGGGTCAGGGG<br>.((((.....)))).	BAI2	NM_001703	chr1:31965305-32002235	non-template	38	Intron_1.f	--	Yes	--	Yes	--	--	--	--	--	--	--
G4ST01031998869	chr1:31998870-31998906	-	GGGCAGGTCGGGCGGCGAGCGCCGGGGAGGCGGAGGG<br>((((....))))	MIR4254	NR_036216	chr1:31996848-31996923	non-template	-1946	upstream	Yes	--	--	--	--	--	--	Yes	Yes	--	--
G4ST01031998869	chr1:31998870-31998906	-	GGGCAGGTCGGGCGGCGAGCGCCGGGGAGGCGGAGGG<br>((((....))))	BAI2	NM_001703	chr1:31965305-32002235	non-template	37	Intron_1.f	--	Yes	--	Yes	--	--	--	Yes	Yes	--	--

G4ST01032002540	chr1:32002541-32002572	+	GGGACGCGGCGCCTGCGCGGGCCGGGCCCGGG<br>.((((.....)))).	BAI2	NM_001703	chr1:31965305-32002235	template	-305	upstream	Yes	--	--	--	--	--	--	Yes	Yes	Yes	Yes
G4ST01032002563	chr1:32002564-32002589	+	GGGCCCGGGAGGGGGCGCGCGCCGGG<br>(((....)))	BAI2	NM_001703	chr1:31965305-32002235	template	-328	upstream	Yes	--	--	--	--	--	--	Yes	Yes	Yes	Yes
G4ST01032002569	chr1:32002570-32002594	+	GGGAGGGGGCGCGCGCCGGGCCGGG<br>(((....)))	BAI2	NM_001703	chr1:31965305-32002235	template	-334	upstream	Yes	--	--	--	--	--	--	Yes	Yes	Yes	Yes
G4ST01032004228	chr1:32004229-32004258	+	GGGAGGGGGAATGGGGTTGCTAGACTGGGG<br>(((....)))..	BAI2	NM_001703	chr1:31965305-32002235	template	-1993	upstream	Yes	--	--	--	--	--	--	--	--	--	Yes
G4ST01032032264	chr1:32032265-32032297	-	GGGAGGGGTGCAGGGGTGGCAGCCAGGCTGGGG<br>....((((...))))	SPOCD1	NM_144569	chr1:32028612-32054167	non-template	33	Intron_11	--	Yes	--	Yes	--	--	--	--	--	--	Yes
G4ST01032036854	chr1:32036855-32036880	+	GGGAGGCAGCTGGGTGGGCGGCAGGG<br>((....))	SPOCD1	NM_144569	chr1:32028612-32054167	template	26	Intron_7	--	Yes	--	Yes	--	--	--	--	Yes	--	Yes
G4ST01032176116	chr1:32176117-32176157	-	GGGCGGCCCCCGCGCCGGCGCCGGGGGCCTCGGGGAAGGGG<br>((((..(((...))).)))).	PTP4A2	NM_080391	chr1:32144609-32176575	non-template	41	Intron_1.f	--	Yes	--	Yes	--	--	--	Yes	Yes	Yes	Yes
G4ST01032176934	chr1:32176935-32176976	+	GGGTCAGATCCTGATGGGACGGGGTGGCCGCCACGTCCTGGG<br>.(((...))).. ((((...))))....	PTP4A2	NM_080391	chr1:32144609-32176575	template	-359	upstream	Yes	--	--	--	--	--	--	Yes	Yes	Yes	--
G4ST01032176949	chr1:32176950-32176983	+	GGGACGGGGTGGCCGCCACGTCCTGGGCTTGGGG<br>((((...))))....	PTP4A2	NM_080391	chr1:32144609-32176575	template	-374	upstream	Yes	--	--	--	--	--	--	Yes	Yes	Yes	--
G4ST01032311497	chr1:32311498-32311531	+	GGGGCTAGAAAGGGGGGCGGGACTTACCAGTGGG<br>(((....)))	TMEM39B	NM_018056	chr1:32311090-32341054	non-template	34	Intron_1.f	--	Yes	--	Yes	--	--	--	Yes	Yes	Yes	--
G4ST01032418112	chr1:32418113-32418149	+	GGGCGCGCGGGGCGTGGGTGGCTGCGCGCGCGGGGGG<br>...((((....)))).	TXLNA	NM_175852	chr1:32417932-32436473	non-template	37	Intron_1.f	--	Yes	--	Yes	--	--	--	Yes	Yes	Yes	Yes
G4ST01032418120	chr1:32418121-32418160	+	GGGGCGTGGGTGGCTGCGCGCGCGGGGGGCGCACGTGGGG<br>...((((....)))).	TXLNA	NM_175852	chr1:32417932-32436473	non-template	40	Intron_1.f	--	Yes	--	Yes	--	--	--	Yes	Yes	Yes	Yes
G4ST01032418127	chr1:32418128-32418169	+	GGGTGGCTGCGCGCGCGGGGGGCGCACGTGGGGCCTGAGGGG<br>...((((....)))).	TXLNA	NM_175852	chr1:32417932-32436473	non-template	42	Intron_1.f	--	Yes	--	Yes	--	--	--	Yes	Yes	Yes	Yes
G4ST01032437916	chr1:32437917-32437955	+	GGGGAAGGTGGGCTGGGTGGCTGTGTGCACTGCCCTGGG<br>.(((.((....)).)))..	CCDC28B	NM_024296	chr1:32438789-32443578	non-template	-833	upstream	Yes	--	--	--	--	--	--	Yes	--	Yes	Yes
G4ST01032442026	chr1:32442027-32442064	-	GGGTGGGGTGGACATGACCATGATCACAGGGTAGGGGG<br>((((.(((....))).)))).	IQCC	NM_001160042	chr1:32443823-32446875	template	-1758	upstream	Yes	--	--	--	--	--	--	Yes	--	--	--
G4ST01032442026	chr1:32442027-32442064	-	GGGTGGGGTGGACATGACCATGATCACAGGGTAGGGGG<br>((((.(((....))).)))).	IQCC	NM_018134	chr1:32443823-32446875	template	-1758	upstream	Yes	--	--	--	--	--	--	Yes	--	--	--
G4ST01032442026	chr1:32442027-32442064	-	GGGTGGGGTGGACATGACCATGATCACAGGGTAGGGGG<br>((((.(((....))).)))).	CCDC28B	NM_024296	chr1:32438789-32443578	template	38	Intron_2	--	Yes	--	Yes	--	--	--	Yes	--	--	--
G4ST01032518409	chr1:32518410-32518439	+	GGGCAGGAACTGAAAGGGTGATGGGAAGGG<br>(((...)))...	LCK	NM_001042771	chr1:32512299-32524353	non-template	30	Intron_12.l	--	Yes	--	Yes	--	--	--	--	--	--	--
G4ST01032518409	chr1:32518410-32518439	+	GGGCAGGAACTGAAAGGGTGATGGGAAGGG<br>(((...)))...	LCK	NM_005356	chr1:32489427-32524353	non-template	30	Intron_12.l	--	Yes	--	Yes	--	--	--	--	--	--	--
G4ST01032530675	chr1:32530676-32530710	+	GGGACTCCTAGAGGGGAGGGTGCGTCGGAGCGGGG<br>.(((...))) ..(((....)))	HDAC1	NM_004964	chr1:32530295-32571811	non-template	35	Intron_1.f	--	Yes	--	Yes	--	--	--	--	Yes	Yes	--
G4ST01032570802	chr1:32570803-32570839	+	GGGGAAGGGGTCTCAGGGTAAATGAAGACCTCATGGG<br>...((((.....))))	HDAC1	NM_004964	chr1:32530295-32571811	non-template	37	Intron_12	--	Yes	--	Yes	--	--	--	Yes	Yes	Yes	--
G4ST01032573933	chr1:32573934-32573959	-	GGGGCGGGGAGGGGGCGCCGGCCGGG<br>(((....)))	MARCKSL1	NM_023009	chr1:32572027-32574421	non-template	26	Intron_1.u	--	Yes	--	Yes	--	--	--	Yes	Yes	Yes	Yes
G4ST01032576248	chr1:32576249-32576287	+	GGGCTCAGAGGGACGGAGGGTGTGTGAGTCTTGCACGGG<br>.(((((.....)))))	MARCKSL1	NM_023009	chr1:32572027-32574421	template	-1827	upstream	Yes	--	--	--	--	--	--	Yes	Yes	--	--
G4ST01032576257	chr1:32576258-32576293	+	GGGACGGAGGGTGTGTGAGTCTTGCACGGGTTGGGG<br>.(((((.....)))))	MARCKSL1	NM_023009	chr1:32572027-32574421	template	-1836	upstream	Yes	--	--	--	--	--	--	Yes	Yes	--	--
G4ST01032777907	chr1:32777908-32777937	+	GGGCCGGGAGCGCGGCGGGAGGCGCCCGGG<br>.((...)) .((...))	ZBTB8A	NM_001040441	chr1:32777359-32844129	non-template	30	Intron_1.f	--	Yes	--	Yes	--	--	--	--	Yes	Yes	--
G4ST01032777912	chr1:32777913-32777942	+	GGGAGCGCGGCGGGAGGCGCCCGGGAGGGG<br>.((...)) .((...))	ZBTB8A	NM_001040441	chr1:32777359-32844129	non-template	30	Intron_1.f	--	Yes	--	Yes	--	--	--	Yes	Yes	Yes	--
G4ST01032777923	chr1:32777924-32777949	+	GGGAGGCGCCCGGGAGGGGCTGCGGG<br>.((...))	ZBTB8A	NM_001040441	chr1:32777359-32844129	non-template	26	Intron_1.f	--	Yes	--	Yes	--	--	--	Yes	Yes	Yes	--
G4ST01032778133	chr1:32778134-32778164	+	GGGCGGCCGCCGCGGGGGCGCGCGGGGAGGG<br>...(((...)))	ZBTB8A	NM_001040441	chr1:32777359-32844129	non-template	31	Intron_1.f	--	Yes	--	Yes	--	--	--	Yes	Yes	Yes	Yes
G4ST01032778166	chr1:32778167-32778196	+	GGGCAGGGCCGGGGCCGGAGTCTCTGCGGG<br>..((((...)))).	ZBTB8A	NM_001040441	chr1:32777359-32844129	non-template	30	Intron_1.f	--	Yes	--	Yes	--	--	--	Yes	Yes	Yes	Yes
G4ST01032780500	chr1:32780501-32780530	+	GGGAGGCCAAGGCCTCGGGGGTCGGGGGGG<br>((((....))))...	ZBTB8A	NM_001040441	chr1:32777359-32844129	non-template	30	Intron_1.f	--	Yes	--	Yes	--	--	--	--	--	--	Yes
G4ST01032940892	chr1:32940893-32940924	-	GGGCGCGGGCGCCGGAGCGAGGGGCCCGCGGG<br>(((....)))..	SYNC	NM_001161708	chr1:32918094-32940948	non-template	32	Exon_1.f	--	Yes	Yes	--	--	Yes	--	Yes	Yes	Yes	--
G4ST01032940892	chr1:32940893-32940924	-	GGGCGCGGGCGCCGGAGCGAGGGGCCCGCGGG<br>(((....)))..	SYNC	NM_030786	chr1:32918094-32940948	non-template	32	Exon_1.f	--	Yes	Yes	--	--	Yes	--	Yes	Yes	Yes	--
G4ST01032979803	chr1:32979804-32979839	+	GGGCCCCAGCGGGGGGCACGACCCCGGGCTCCCGGG<br>(((.....))).	KIAA1522	NM_020888	chr1:32980099-33013158	non-template	-259	upstream	Yes	--	--	--	--	--	--	Yes	Yes	Yes	Yes
G4ST01032979967	chr1:32979968-32979996	-	GGGAGGGGCCGGCCGAGCGGCGGGCGGGG<br>((((......))))	KIAA1522	NM_020888	chr1:32980099-33013158	template	-102	upstream	Yes	--	--	--	--	--	--	Yes	Yes	Yes	--
G4ST01032979972	chr1:32979973-32980007	-	GGGAGCGGCGCGGGAGGGGCCGGCCGAGCGGCGGG<br>.((...)) ((((......))))	KIAA1522	NM_020888	chr1:32980099-33013158	template	-91	upstream	Yes	--	--	--	--	--	--	Yes	Yes	Yes	--
G4ST01032980114	chr1:32980115-32980152	-	GGGCCGCCATGGCAGGCGGGGGCGGAGCGGGCGGCGGG<br>(((((......)))))	KIAA1522	NM_020888	chr1:32980099-33013158	template	38	Exon_1.f	--	Yes	Yes	--	Yes	Yes	--	--	Yes	Yes	--
G4ST01032980121	chr1:32980122-32980163	-	GGGGGGCGCGCGGGCCGCCATGGCAGGCGGGGGCGGAGCGGG<br>(((((......)))))	KIAA1522	NM_020888	chr1:32980099-33013158	template	42	Exon_1.f	--	Yes	Yes	--	Yes	Yes	--	--	Yes	Yes	--
G4ST01032992168	chr1:32992169-32992203	-	GGGAGGCAGCGAGACTCGCGGGCGGGCACCGCGGG<br>.....((((...))))	KIAA1522	NM_020888	chr1:32980099-33013158	template	35	Intron_1.f	--	Yes	--	Yes	--	--	--	Yes	Yes	Yes	--
G4ST01032992177	chr1:32992178-32992207	-	GGGAGGGAGGCAGCGAGACTCGCGGGCGGG<br>.....((((...))))	KIAA1522	NM_020888	chr1:32980099-33013158	template	30	Intron_1.f	--	Yes	--	Yes	--	--	--	Yes	Yes	Yes	--
G4ST01032993119	chr1:32993120-32993162	-	GGGGGAGATGCGGCGGCGACCTCGGGGACCGTGGGGACGGGGG<br>.(((.(((....)))..))).	KIAA1522	NM_020888	chr1:32980099-33013158	template	43	Intron_1.f	--	Yes	--	Yes	--	--	--	Yes	Yes	--	Yes
G4ST01032998600	chr1:32998601-32998638	-	GGGGCTTTCCTAAGCCTGGGGAAAGGGAAGAATCGGGG<br>((((....))))...	KIAA1522	NM_020888	chr1:32980099-33013158	template	38	Intron_1.f	--	Yes	--	Yes	--	--	--	Yes	Yes	Yes	Yes
G4ST01033004946	chr1:33004947-33004973	+	GGGTTTCATTTAAAGGGGGGGCTGGGG<br>(((.....)))	KIAA1522	NM_001198972	chr1:33003822-33013158	non-template	27	Intron_1.f	--	Yes	--	Yes	--	--	--	Yes	Yes	--	--
G4ST01033004946	chr1:33004947-33004973	+	GGGTTTCATTTAAAGGGGGGGCTGGGG<br>(((.....)))	KIAA1522	NM_001198973	chr1:33003822-33013158	non-template	27	Intron_1.f	--	Yes	--	Yes	--	--	--	Yes	Yes	--	--
G4ST01033004946	chr1:33004947-33004973	+	GGGTTTCATTTAAAGGGGGGGCTGGGG<br>(((.....)))	KIAA1522	NM_020888	chr1:32980099-33013158	non-template	27	Intron_1.f	--	Yes	--	Yes	--	--	--	Yes	Yes	--	--
G4ST01033005090	chr1:33005091-33005121	+	GGGCCAGGGCCAGGGCCTCCAGGAGAGGGGG<br>((((.....))))	KIAA1522	NM_001198972	chr1:33003822-33013158	non-template	31	Intron_1.f	--	Yes	--	Yes	--	--	--	--	Yes	--	Yes
G4ST01033005090	chr1:33005091-33005121	+	GGGCCAGGGCCAGGGCCTCCAGGAGAGGGGG<br>((((.....))))	KIAA1522	NM_001198973	chr1:33003822-33013158	non-template	31	Intron_1.f	--	Yes	--	Yes	--	--	--	--	Yes	--	Yes
G4ST01033005090	chr1:33005091-33005121	+	GGGCCAGGGCCAGGGCCTCCAGGAGAGGGGG<br>((((.....))))	KIAA1522	NM_020888	chr1:32980099-33013158	non-template	31	Intron_1.f	--	Yes	--	Yes	--	--	--	--	Yes	--	Yes
G4ST01033005096	chr1:33005097-33005130	+	GGGCCAGGGCCTCCAGGAGAGGGGGATACCTGGG<br>((((.....))))	KIAA1522	NM_001198972	chr1:33003822-33013158	non-template	34	Intron_1.f	--	Yes	--	Yes	--	--	--	--	Yes	--	Yes
G4ST01033005096	chr1:33005097-33005130	+	GGGCCAGGGCCTCCAGGAGAGGGGGATACCTGGG<br>((((.....))))	KIAA1522	NM_001198973	chr1:33003822-33013158	non-template	34	Intron_1.f	--	Yes	--	Yes	--	--	--	--	Yes	--	Yes
G4ST01033005096	chr1:33005097-33005130	+	GGGCCAGGGCCTCCAGGAGAGGGGGATACCTGGG<br>((((.....))))	KIAA1522	NM_020888	chr1:32980099-33013158	non-template	34	Intron_1.f	--	Yes	--	Yes	--	--	--	--	Yes	--	Yes

G4ST01033009851	chr1:33009852-33009892	-	GGGGCTGGCGGAGCTGGAGGAGGGTCTGGGCTACCCGGGGG<br>.(((((...)))))....	KIAA1522	NM_001198972	chr1:33003822-33013158	template	41	Exon_6	--	Yes	Yes	--	Yes	--	--	Yes	--	Yes	--
G4ST01033009851	chr1:33009852-33009892	-	GGGGCTGGCGGAGCTGGAGGAGGGTCTGGGCTACCCGGGGG<br>.(((((...)))))....	KIAA1522	NM_001198973	chr1:33003822-33013158	template	41	Intron_4.l	--	Yes	--	Yes	--	--	--	Yes	--	Yes	--
G4ST01033009851	chr1:33009852-33009892	-	GGGGCTGGCGGAGCTGGAGGAGGGTCTGGGCTACCCGGGGG<br>.(((((...)))))....	KIAA1522	NM_020888	chr1:32980099-33013158	template	41	Exon_6	--	Yes	Yes	--	Yes	--	--	Yes	--	Yes	--
G4ST01033101833	chr1:33101834-33101867	-	GGGCACAGGGAGAGGGACAACTGCAGCAGTGGGG<br>...((((...)))).	FNDC5	NM_001171940	chr1:33100456-33109001	non-template	34	Exon_6.l	--	Yes	Yes	--	--	--	Yes	--	Yes	--	--
G4ST01033101833	chr1:33101834-33101867	-	GGGCACAGGGAGAGGGACAACTGCAGCAGTGGGG<br>...((((...)))).	FNDC5	NM_153756	chr1:33100456-33109001	non-template	34	Exon_6.l	--	Yes	Yes	--	--	--	Yes	--	Yes	--	--
G4ST01033101833	chr1:33101834-33101867	-	GGGCACAGGGAGAGGGACAACTGCAGCAGTGGGG<br>...((((...)))).	FNDC5	NM_001171941	chr1:33100456-33110669	non-template	34	Exon_5.l	--	Yes	Yes	--	--	--	Yes	--	Yes	--	--
G4ST01033108805	chr1:33108806-33108836	-	GGGGGCCCGGGGCGGGGGCCGGCGCCGGGG<br>.(((....))).	FNDC5	NM_001171940	chr1:33100456-33109001	non-template	31	Intron_1.f	--	Yes	--	Yes	--	--	--	Yes	Yes	--	Yes
G4ST01033108805	chr1:33108806-33108836	-	GGGGGCCCGGGGCGGGGGCCGGCGCCGGGG<br>.(((....))).	FNDC5	NM_153756	chr1:33100456-33109001	non-template	31	Intron_1.f	--	Yes	--	Yes	--	--	--	Yes	Yes	--	Yes
G4ST01033108805	chr1:33108806-33108836	-	GGGGGCCCGGGGCGGGGGCCGGCGCCGGGG<br>.(((....))).	FNDC5	NM_001171941	chr1:33100456-33110669	non-template	31	Intron_1.f	--	Yes	--	Yes	--	--	--	Yes	Yes	--	Yes
G4ST01033109018	chr1:33109019-33109054	-	GGGCGGCCGGAGCGGCCGGGCCGGCCGGGCCGGGGG<br>.(((((...)))))	FNDC5	NM_001171940	chr1:33100456-33109001	non-template	-17	upstream	Yes	--	--	--	--	--	--	--	Yes	--	--
G4ST01033109018	chr1:33109019-33109054	-	GGGCGGCCGGAGCGGCCGGGCCGGCCGGGCCGGGGG<br>.(((((...)))))	FNDC5	NM_153756	chr1:33100456-33109001	non-template	-17	upstream	Yes	--	--	--	--	--	--	--	Yes	--	--
G4ST01033109018	chr1:33109019-33109054	-	GGGCGGCCGGAGCGGCCGGGCCGGCCGGGCCGGGGG<br>.(((((...)))))	FNDC5	NM_001171941	chr1:33100456-33110669	non-template	36	Intron_1.f	--	Yes	--	Yes	--	--	--	--	Yes	--	--
G4ST01033109025	chr1:33109026-33109065	-	GGGGCGGCGCAGGGCGGCCGGAGCGGCCGGGCCGGCCGGG<br>.(((((...)))))	FNDC5	NM_001171940	chr1:33100456-33109001	non-template	-24	upstream	Yes	--	--	--	--	--	--	--	Yes	--	--
G4ST01033109025	chr1:33109026-33109065	-	GGGGCGGCGCAGGGCGGCCGGAGCGGCCGGGCCGGCCGGG<br>.(((((...)))))	FNDC5	NM_153756	chr1:33100456-33109001	non-template	-24	upstream	Yes	--	--	--	--	--	--	--	Yes	--	--
G4ST01033109025	chr1:33109026-33109065	-	GGGGCGGCGCAGGGCGGCCGGAGCGGCCGGGCCGGCCGGG<br>.(((((...)))))	FNDC5	NM_001171941	chr1:33100456-33110669	non-template	40	Intron_1.f	--	Yes	--	Yes	--	--	--	--	Yes	--	--
G4ST01033110714	chr1:33110715-33110745	-	GGGTGGGGTGGGGCAGGCAGCAGCTGCGGGG<br>....((((...)))).	FNDC5	NM_001171940	chr1:33100456-33109001	non-template	-1713	upstream	Yes	--	--	--	--	--	--	Yes	Yes	--	Yes
G4ST01033110714	chr1:33110715-33110745	-	GGGTGGGGTGGGGCAGGCAGCAGCTGCGGGG<br>....((((...)))).	FNDC5	NM_153756	chr1:33100456-33109001	non-template	-1713	upstream	Yes	--	--	--	--	--	--	Yes	Yes	--	Yes
G4ST01033110714	chr1:33110715-33110745	-	GGGTGGGGTGGGGCAGGCAGCAGCTGCGGGG<br>....((((...)))).	FNDC5	NM_001171941	chr1:33100456-33110669	non-template	-45	upstream	Yes	--	--	--	--	--	--	Yes	Yes	--	Yes
G4ST01033125983	chr1:33125984-33126023	+	GGGGTCGTGGGCTGGTGTGTCTTGATGCACAGGGTATGGG<br>...((((((....)))))).	HPCA	NM_002143	chr1:33124685-33132834	non-template	40	Intron_1.f	--	Yes	--	Yes	--	--	--	--	Yes	--	--
G4ST01033126518	chr1:33126519-33126548	+	GGGAGAACAGGGCAGTGTCTGGGCTGCGGG<br>.((...))	HPCA	NM_002143	chr1:33124685-33132834	non-template	30	Intron_1.f	--	Yes	--	Yes	--	--	--	--	--	--	--
G4ST01033130043	chr1:33130044-33130083	-	GGGATGCTTGGGCAGTGTGGGGTGGTGATAATCAGCAGGG<br>..(.(((...))).).	HPCA	NM_002143	chr1:33124685-33132834	template	40	Intron_2	--	Yes	--	Yes	--	--	--	--	--	--	--
G4ST01033131243	chr1:33131244-33131282	-	GGGGGCGCCGGGCCAAGGGGGCGGCACTGCGCTGCGGGG<br>.(((((.....))))).	HPCA	NM_002143	chr1:33124685-33132834	template	39	Intron_2	--	Yes	--	Yes	--	--	--	Yes	Yes	--	--
G4ST01033131555	chr1:33131556-33131593	-	GGGGAATCACGGAGGCGTGGGGAGCCGAGGGCTTCGGG<br>....((((....))))	HPCA	NM_002143	chr1:33124685-33132834	template	38	Intron_2	--	Yes	--	Yes	--	--	--	Yes	Yes	--	--
G4ST01033133251	chr1:33133252-33133285	-	GGGCCCAGGATGAGTCTTGAGGGAGGGGCAGGGG<br>..(((((....))))).	TMEM54	NM_033504	chr1:33132783-33139540	non-template	34	Intron_5.l	--	Yes	--	Yes	--	--	--	--	--	--	Yes
G4ST01033139324	chr1:33139325-33139364	+	GGGGGCTCGGGTTCCGGGCTCAGTGCTCCGCTCCTGTGGG<br>..(((.((...))..))).	TMEM54	NM_033504	chr1:33132783-33139540	template	40	Intron_1.f	--	Yes	--	Yes	--	--	--	--	Yes	Yes	Yes
G4ST01033139332	chr1:33139333-33139368	+	GGGTTCCGGGCTCAGTGCTCCGCTCCTGTGGGAGGG<br>..(((.((...))..))).	TMEM54	NM_033504	chr1:33132783-33139540	template	36	Intron_1.f	--	Yes	--	Yes	--	--	--	--	Yes	Yes	Yes
G4ST01033139339	chr1:33139340-33139374	+	GGGCTCAGTGCTCCGCTCCTGTGGGAGGGTTGGGG<br>..(((.((...))..))).	TMEM54	NM_033504	chr1:33132783-33139540	template	35	Intron_1.f	--	Yes	--	Yes	--	--	--	--	Yes	Yes	Yes
G4ST01033139512	chr1:33139513-33139549	-	GGGCGAGCGCGACGGTGCGGGGCGCGCGGGCGACGGG<br>....((((....))))	TMEM54	NM_033504	chr1:33132783-33139540	non-template	28	upstream-Exon_1.f	Yes	Yes	Yes	--	--	Yes	--	Yes	Yes	Yes	Yes
G4ST01033139519	chr1:33139520-33139556	-	GGGAAGTGGGCGAGCGCGACGGTGCGGGGCGCGCGGG<br>....((((....))))	TMEM54	NM_033504	chr1:33132783-33139540	non-template	21	upstream-Exon_1.f	Yes	Yes	Yes	--	--	Yes	--	Yes	Yes	Yes	Yes
G4ST01033139527	chr1:33139528-33139560	-	GGGCGGGAAGTGGGCGAGCGCGACGGTGCGGGG<br>....((((....))))	TMEM54	NM_033504	chr1:33132783-33139540	non-template	13	upstream-Exon_1.f	Yes	Yes	Yes	--	--	Yes	--	Yes	Yes	Yes	Yes
G4ST01033139713	chr1:33139714-33139740	-	GGGCGGCTCGGCGCCGGGCGGGCCGGG<br>.(((.....)))	TMEM54	NM_033504	chr1:33132783-33139540	non-template	-173	upstream	Yes	--	--	--	--	--	--	Yes	--	Yes	Yes
G4ST01033139718	chr1:33139719-33139744	-	GGGAGGGCGGCTCGGCGCCGGGCGGG<br>.(((.....)))	TMEM54	NM_033504	chr1:33132783-33139540	non-template	-178	upstream	Yes	--	--	--	--	--	--	Yes	--	Yes	Yes
G4ST01033139722	chr1:33139723-33139751	-	GGGCTGAGGGAGGGCGGCTCGGCGCCGGG<br>.(((.....)))	TMEM54	NM_033504	chr1:33132783-33139540	non-template	-182	upstream	Yes	--	--	--	--	--	--	Yes	--	Yes	Yes
G4ST01033139903	chr1:33139904-33139946	+	GGGACGGAGAGGGGCAGCAGGGGCAGTGAAAGAGCTGCTGGGG<br>(((((......)))))..	TMEM54	NM_033504	chr1:33132783-33139540	template	-363	upstream	Yes	--	--	--	--	--	--	--	--	--	--
G4ST01033139913	chr1:33139914-33139953	+	GGGGCAGCAGGGGCAGTGAAAGAGCTGCTGGGGATTGGGG<br>(((((......)))))..	TMEM54	NM_033504	chr1:33132783-33139540	template	-373	upstream	Yes	--	--	--	--	--	--	--	--	--	--
G4ST01033202204	chr1:33202205-33202240	-	GGGTGAGCGCGGGGGCGTGGCGCGGGTGGCGGCGGG<br>.(((...)))	RNF19B	NM_001127361	chr1:33174637-33202873	non-template	36	Exon_1.f-Intron_1.f	--	Yes	Yes	Yes	Yes	--	--	Yes	Yes	Yes	--
G4ST01033202204	chr1:33202205-33202240	-	GGGTGAGCGCGGGGGCGTGGCGCGGGTGGCGGCGGG<br>.(((...)))	RNF19B	NM_153341	chr1:33174637-33202873	non-template	36	Exon_1.f-Intron_1.f	--	Yes	Yes	Yes	Yes	--	--	Yes	Yes	Yes	--
G4ST01033202667	chr1:33202668-33202702	+	GGGGCCGGGGCGGGCGGCGGCTGCGCAGCCGGGGG<br>...(((((....))))).	RNF19B	NM_001127361	chr1:33174637-33202873	template	35	Exon_1.f	--	Yes	Yes	--	Yes	--	--	--	Yes	Yes	--
G4ST01033202667	chr1:33202668-33202702	+	GGGGCCGGGGCGGGCGGCGGCTGCGCAGCCGGGGG<br>...(((((....))))).	RNF19B	NM_153341	chr1:33174637-33202873	template	35	Exon_1.f	--	Yes	Yes	--	Yes	--	--	--	Yes	Yes	--
G4ST01033321014	chr1:33321015-33321048	-	GGGGAAAGCTGGGAATGGGGCTATGGAGGCAGGG<br>(((.....))).	ADC	NM_052998	chr1:33319301-33358582	template	34	Intron_4	--	Yes	--	Yes	--	--	--	Yes	--	--	--
G4ST01033330503	chr1:33330504-33330537	+	GGGCTGAGGGAAGGCTGGGCTTCAGAGTGAAGGG<br>.((((...))))	ADC	NM_052998	chr1:33319301-33358582	non-template	34	Intron_6	--	Yes	--	Yes	--	--	--	--	--	--	Yes
G4ST01033330510	chr1:33330511-33330542	+	GGGAAGGCTGGGCTTCAGAGTGAAGGGGGGGG<br>.((((...))))	ADC	NM_052998	chr1:33319301-33358582	non-template	32	Intron_6	--	Yes	--	Yes	--	--	--	--	--	--	Yes
G4ST01033330519	chr1:33330520-33330547	+	GGGCTTCAGAGTGAAGGGGGGGGTGGGG<br>.((((...))))	ADC	NM_052998	chr1:33319301-33358582	non-template	28	Intron_6	--	Yes	--	Yes	--	--	--	--	--	--	Yes
G4ST01033386403	chr1:33386404-33386444	-	GGGACACGTGAAAATCACTGGGAGTCGGGGAAGGTGTGGGG<br>....((((...)))).	TRIM62	NM_018207	chr1:33383590-33420258	non-template	41	Intron_4.l	--	Yes	--	Yes	--	--	--	--	Yes	--	--
G4ST01033392458	chr1:33392459-33392488	-	GGGCTGTCCTGGGTCAGTGACTGGGGAGGG<br>.(((...)))	TRIM62	NM_018207	chr1:33383590-33420258	non-template	30	Intron_4.l	--	Yes	--	Yes	--	--	--	--	--	--	--
G4ST01033398231	chr1:33398232-33398259	+	GGGTGGGAAAGGGCACGCGTGGTGGGGG<br>(((.....))).	TRIM62	NM_018207	chr1:33383590-33420258	template	28	Intron_2	--	Yes	--	Yes	--	--	--	--	--	--	--
G4ST01033401784	chr1:33401785-33401814	-	GGGTGTTCCAAGGGATGTGGTGGGTTGGGG<br>((...)).	TRIM62	NM_018207	chr1:33383590-33420258	non-template	30	Intron_2	--	Yes	--	Yes	--	--	--	--	Yes	--	Yes
G4ST01033402888	chr1:33402889-33402920	+	GGGCTGGGCCACCTCTGGAGGGCTTCGAGGGG<br>(((....))).	TRIM62	NM_018207	chr1:33383590-33420258	template	32	Intron_2	--	Yes	--	Yes	--	--	--	--	--	--	--
G4ST01033416425	chr1:33416426-33416470	+	GGGCCCCAGCTCCATCAGCTGCTGGGAGAGAAGGGGATGTGAGGG<br>...(((((.....)))))..	TRIM62	NM_018207	chr1:33383590-33420258	template	45	Intron_1.f	--	Yes	--	Yes	--	--	--	--	--	--	Yes
G4ST01033419008	chr1:33419009-33419042	-	GGGGCCGCCGCATGGCGGGGCCGTGGGGGCGGGG<br>..((((....))))	TRIM62	NM_018207	chr1:33383590-33420258	non-template	34	Intron_1.f	--	Yes	--	Yes	--	--	--	Yes	Yes	Yes	Yes

G4ST01033419013	chr1:33419014-33419047	-	GGGGCGGGGCCGCCGCATGGCGGGGCCGTGGGGG<br>..((((....))))	TRIM62	NM_018207	chr1:33383590-33420258	non-template	34	Intron_1.f	--	Yes	--	Yes	--	--	--	Yes	Yes	Yes	Yes
G4ST01033419022	chr1:33419023-33419051	-	GGGAGGGGCGGGGCCGCCGCATGGCGGGG<br>..((((....))))	TRIM62	NM_018207	chr1:33383590-33420258	non-template	29	Intron_1.f	--	Yes	--	Yes	--	--	--	Yes	Yes	Yes	Yes
G4ST01033494452	chr1:33494453-33494489	-	GGGCGCTGGCGACTGCGCGAGAGGGGAGGGGGCCGGG<br>..((.(((....))).))..	ZNF362	NM_152493	chr1:33494761-33538907	template	-271	upstream	Yes	--	--	--	--	--	--	Yes	--	Yes	Yes
G4ST01033494457	chr1:33494458-33494499	-	GGGCAGCGCCGGGCGCTGGCGACTGCGCGAGAGGGGAGGGGG<br>..((.(((....))).))..	ZNF362	NM_152493	chr1:33494761-33538907	template	-261	upstream	Yes	--	--	--	--	--	--	Yes	--	Yes	Yes
G4ST01033494933	chr1:33494934-33494963	+	GGGCCGGGGCGCGGGCGCTGTCAGCGCGGG<br>((((...)))).	ZNF362	NM_152493	chr1:33494761-33538907	non-template	30	Intron_1.f	--	Yes	--	Yes	--	--	--	--	Yes	Yes	Yes
G4ST01033494938	chr1:33494939-33494969	+	GGGGCGCGGGCGCTGTCAGCGCGGGCCGGGG<br>((((...)))).	ZNF362	NM_152493	chr1:33494761-33538907	non-template	31	Intron_1.f	--	Yes	--	Yes	--	--	--	--	Yes	Yes	Yes
G4ST01033507356	chr1:33507357-33507391	-	GGGGCAGGGGGAGGTCAGGGTCAGCTGCTGGTGGG<br>.(((...)))..	ZNF362	NM_152493	chr1:33494761-33538907	template	35	Intron_1.f	--	Yes	--	Yes	--	--	--	--	--	--	--
G4ST01033533225	chr1:33533226-33533255	-	GGGTGGGGGGGCACCAGAGCACCTGGTGGG<br>.(((((.....)))))	ZNF362	NM_152493	chr1:33494761-33538907	template	30	Intron_7	--	Yes	--	Yes	--	--	--	--	--	--	Yes
G4ST01033579480	chr1:33579481-33579507	+	GGGTGGGCCACACAGGTGGAGGGAGGG<br>((((....)))).	PHC2	NM_198040	chr1:33561811-33613781	template	27	Intron_8	--	Yes	--	Yes	--	--	--	--	--	--	--
G4ST01033579480	chr1:33579481-33579507	+	GGGTGGGCCACACAGGTGGAGGGAGGG<br>((((....)))).	PHC2	NM_004427	chr1:33561811-33588086	template	27	Intron_1.f	--	Yes	--	Yes	--	--	--	--	--	--	--
G4ST01033587984	chr1:33587985-33588011	+	GGGACGGGGGCGCGAGGCCGGGGCGGG<br>(((.....))).	PHC2	NM_198040	chr1:33561811-33613781	template	27	Intron_8	--	Yes	--	Yes	--	--	--	--	Yes	Yes	--
G4ST01033587984	chr1:33587985-33588011	+	GGGACGGGGGCGCGAGGCCGGGGCGGG<br>(((.....))).	PHC2	NM_004427	chr1:33561811-33588086	template	27	Exon_1.f	--	Yes	Yes	--	--	Yes	--	--	Yes	Yes	--
G4ST01033587989	chr1:33587990-33588019	+	GGGGGCGCGAGGCCGGGGCGGGAGCGCGGG<br>(((.....))).	PHC2	NM_198040	chr1:33561811-33613781	template	30	Intron_8	--	Yes	--	Yes	--	--	--	Yes	Yes	Yes	--
G4ST01033587989	chr1:33587990-33588019	+	GGGGGCGCGAGGCCGGGGCGGGAGCGCGGG<br>(((.....))).	PHC2	NM_004427	chr1:33561811-33588086	template	30	Exon_1.f	--	Yes	Yes	--	--	Yes	--	Yes	Yes	Yes	--
G4ST01033588003	chr1:33588004-33588031	+	GGGGCGGGAGCGCGGGCGGCGGCCGGGG<br>(((...)))	PHC2	NM_198040	chr1:33561811-33613781	template	28	Intron_8	--	Yes	--	Yes	--	--	--	Yes	Yes	Yes	--
G4ST01033588003	chr1:33588004-33588031	+	GGGGCGGGAGCGCGGGCGGCGGCCGGGG<br>(((...)))	PHC2	NM_004427	chr1:33561811-33588086	template	28	Exon_1.f	--	Yes	Yes	--	--	Yes	--	Yes	Yes	Yes	--
G4ST01033588008	chr1:33588009-33588053	+	GGGAGCGCGGGCGGCGGCCGGGGTTGCGCGCGCGCGCGCGGCGGG<br>(((...))) ....(((((....)))))..	PHC2	NM_198040	chr1:33561811-33613781	template	45	Intron_8	--	Yes	--	Yes	--	--	--	Yes	Yes	Yes	--
G4ST01033588008	chr1:33588009-33588053	+	GGGAGCGCGGGCGGCGGCCGGGGTTGCGCGCGCGCGCGCGGCGGG<br>(((...))) ....(((((....)))))..	PHC2	NM_004427	chr1:33561811-33588086	template	45	Exon_1.f	--	Yes	Yes	--	--	Yes	--	Yes	Yes	Yes	--
G4ST01033588016	chr1:33588017-33588063	+	GGGCGGCGGCCGGGGTTGCGCGCGCGCGCGCGGCGGGCGCTCGAGGG<br>(((...))) ....(((((....)))))..	PHC2	NM_198040	chr1:33561811-33613781	template	47	Intron_8	--	Yes	--	Yes	--	--	--	Yes	Yes	Yes	--
G4ST01033588016	chr1:33588017-33588063	+	GGGCGGCGGCCGGGGTTGCGCGCGCGCGCGCGGCGGGCGCTCGAGGG<br>(((...))) ....(((((....)))))..	PHC2	NM_004427	chr1:33561811-33588086	template	47	Exon_1.f	--	Yes	Yes	--	--	Yes	--	Yes	Yes	Yes	--
G4ST01033607063	chr1:33607064-33607091	+	GGGGTGGGTGGGGAGAGCCGCTCTGGGG<br>.((((...)))).	PHC2	NM_198040	chr1:33561811-33613781	template	28	Intron_4	--	Yes	--	Yes	--	--	--	--	--	--	Yes
G4ST01033612570	chr1:33612571-33612598	+	GGGCCAGGGCCAGGTGCTGGGGCTAGGG<br>.(((...)))	PHC2	NM_198040	chr1:33561811-33613781	template	28	Intron_1.f	--	Yes	--	Yes	--	--	--	--	Yes	--	Yes
G4ST01033612576	chr1:33612577-33612605	+	GGGCCAGGTGCTGGGGCTAGGGACAAGGG<br>.(((...)))	PHC2	NM_198040	chr1:33561811-33613781	template	29	Intron_1.f	--	Yes	--	Yes	--	--	--	--	Yes	--	Yes
G4ST01033709004	chr1:33709005-33709040	-	GGGGAGGGACAGTGCAACAGTGTGGGGTGCCAAGGG<br>(((.((...)).))).	ZSCAN20	NM_145238	chr1:33710819-33734582	template	-1778	upstream	Yes	--	--	--	--	--	--	Yes	Yes	--	--
G4ST01033727506	chr1:33727507-33727543	-	GGGGATGTTTGGGGCCCAGCAGGCTGGGGGTGAAGGG<br>..((((....))))	ZSCAN20	NM_145238	chr1:33710819-33734582	template	37	Intron_4	--	Yes	--	Yes	--	--	--	--	--	--	--
G4ST01033759023	chr1:33759024-33759062	-	GGGGGGAGGGGTCTGGTTTCTCAGAAGCAGGGGAGGGGG<br>..(((.((......)).)))	CSMD2	NM_052896	chr1:33752196-34404030	non-template	39	Intron_67	--	Yes	--	Yes	--	--	--	--	--	--	--
G4ST01033800393	chr1:33800394-33800439	-	GGGAATATGTGGGAAGCAGTGGGTAAGAGCCTGAACCAGGCAGGGG<br>.....((((((...))))))..	CSMD2	NM_052896	chr1:33752196-34404030	non-template	46	Intron_54	--	Yes	--	Yes	--	--	--	--	--	--	--
G4ST01033815150	chr1:33815151-33815182	-	GGGGAGGGCTCCCAGCAGAGGGGATGCTTGGG<br>(((......)))	CSMD2	NM_052896	chr1:33752196-34404030	non-template	32	Intron_50	--	Yes	--	Yes	--	--	--	Yes	--	--	--
G4ST01033871742	chr1:33871743-33871778	+	GGGGACTGGGCAGGCACGCTGGGGGCAGGAGAGGGG<br>(((.....))).	CSMD2	NM_052896	chr1:33752196-34404030	template	36	Intron_31	--	Yes	--	Yes	--	--	--	--	Yes	--	Yes
G4ST01033871749	chr1:33871750-33871787	+	GGGCAGGCACGCTGGGGGCAGGAGAGGGGATCTAGGGG<br>(((.....))).	CSMD2	NM_052896	chr1:33752196-34404030	template	38	Intron_31	--	Yes	--	Yes	--	--	--	--	Yes	--	Yes
G4ST01033883708	chr1:33883709-33883739	+	GGGAGCTGGGATTTAGATAATGGGATTTGGG<br>(((.....)))	CSMD2	NM_052896	chr1:33752196-34404030	template	31	Intron_29	--	Yes	--	Yes	--	--	--	--	--	--	--
G4ST01033883715	chr1:33883716-33883749	+	GGGATTTAGATAATGGGATTTGGGATAATTAGGG<br>(((.....)))	CSMD2	NM_052896	chr1:33752196-34404030	template	34	Intron_29	--	Yes	--	Yes	--	--	--	--	--	--	--
G4ST01033924977	chr1:33924978-33925011	-	GGGAGCATGTGTGCTAGGTGGGAAGGGACATGGG<br>((((....))))....	CSMD2	NM_052896	chr1:33752196-34404030	non-template	34	Intron_25	--	Yes	--	Yes	--	--	--	--	--	--	--
G4ST01033924984	chr1:33924985-33925015	-	GGGTGGGAGCATGTGTGCTAGGTGGGAAGGG<br>((((....))))....	CSMD2	NM_052896	chr1:33752196-34404030	non-template	31	Intron_25	--	Yes	--	Yes	--	--	--	--	--	--	--
G4ST01033934914	chr1:33934915-33934950	+	GGGGCCTGGCAGGTGGGCAGGAGGGGGCTGAGTGGG<br>.(((...))).	CSMD2	NM_052896	chr1:33752196-34404030	template	36	Intron_24	--	Yes	--	Yes	--	--	--	--	--	--	--
G4ST01034196559	chr1:34196560-34196590	-	GGGGATTGTGGAGATGGGACCAAGGGGTGGG<br>.(((.....)))	CSMD2	NM_052896	chr1:33752196-34404030	non-template	31	Intron_3	--	Yes	--	Yes	--	--	--	--	--	--	Yes
G4ST01034196563	chr1:34196564-34196594	-	GGGAGGGGATTGTGGAGATGGGACCAAGGGG<br>.(((.....)))	CSMD2	NM_052896	chr1:33752196-34404030	non-template	31	Intron_3	--	Yes	--	Yes	--	--	--	--	--	--	Yes
G4ST01034210316	chr1:34210317-34210358	+	GGGGACTTCTGGACAGCAGAGGGGGCAGTGGGATGAACAGGG<br>..((((((.....))))))	CSMD2	NM_052896	chr1:33752196-34404030	template	42	Intron_3	--	Yes	--	Yes	--	--	--	--	--	--	--
G4ST01034242111	chr1:34242112-34242141	-	GGGATGGGGGCAGGGCAGTTCTCCAATGGG<br>..(((....)))	CSMD2	NM_052896	chr1:33752196-34404030	non-template	30	Intron_3	--	Yes	--	Yes	--	--	--	--	--	--	--
G4ST01034266972	chr1:34266973-34267003	-	GGGCCGCAGCAGCTGGACGGGAGGGAAGGGG<br>((((....)).))..	CSMD2	NM_052896	chr1:33752196-34404030	non-template	31	Intron_3	--	Yes	--	Yes	--	--	--	--	--	--	--
G4ST01034296953	chr1:34296954-34296986	-	GGGGAGCAGGGTCCAGGCTTCTGGGGACCAGGG<br>..(((....)))	CSMD2	NM_052896	chr1:33752196-34404030	non-template	33	Intron_2	--	Yes	--	Yes	--	--	--	--	--	--	Yes
G4ST01034296960	chr1:34296961-34296993	-	GGGGTGTGGGGAGCAGGGTCCAGGCTTCTGGGG<br>..(((....)))	CSMD2	NM_052896	chr1:33752196-34404030	non-template	33	Intron_2	--	Yes	--	Yes	--	--	--	--	--	--	Yes
G4ST01034299237	chr1:34299238-34299273	-	GGGTTGGGGTGGAGCAAGCTTGCTGCAGGGGTTGGG<br>..(.((((....)))).)..	CSMD2	NM_052896	chr1:33752196-34404030	non-template	36	Intron_2	--	Yes	--	Yes	--	--	--	Yes	--	--	--
G4ST01034377405	chr1:34377406-34377442	+	GGGGTAATGGGTTAATGGATTAATGGGTTATCATGGG<br>((((....)))).	CSMD2	NM_052896	chr1:33752196-34404030	template	37	Intron_1.f	--	Yes	--	Yes	--	--	--	--	--	--	--
G4ST01034385553	chr1:34385554-34385589	+	GGGCAGTGTCAGACGCTGAAGAGGGGGAGGGGAGGG<br>((((((...)))))))......	CSMD2	NM_052896	chr1:33752196-34404030	template	36	Intron_1.f	--	Yes	--	Yes	--	--	--	--	--	--	--
G4ST01034391049	chr1:34391050-34391085	-	GGGGAGGGAGAGATGGGGAGTTAATCATTTAATGGG<br>..(((((....)))))	CSMD2	NM_052896	chr1:33752196-34404030	non-template	36	Intron_1.f	--	Yes	--	Yes	--	--	--	--	--	--	Yes
G4ST01034395757	chr1:34395758-34395791	-	GGGAATGTGTTAGAAGACACTGGGGTGGGATGGG<br>...(((((....)))))..	CSMD2	NM_052896	chr1:33752196-34404030	non-template	34	Intron_1.f	--	Yes	--	Yes	--	--	--	--	--	--	Yes
G4ST01034395762	chr1:34395763-34395796	-	GGGGAGGGAATGTGTTAGAAGACACTGGGGTGGG<br>...(((((....)))))..	CSMD2	NM_052896	chr1:33752196-34404030	non-template	34	Intron_1.f	--	Yes	--	Yes	--	--	--	--	--	--	Yes
G4ST01034395766	chr1:34395767-34395808	-	GGGGGGTGAGGTGGGGAGGGAATGTGTTAGAAGACACTGGGG<br>...(((((....)))))..	CSMD2	NM_052896	chr1:33752196-34404030	non-template	42	Intron_1.f	--	Yes	--	Yes	--	--	--	--	--	--	Yes
G4ST01034405380	chr1:34405381-34405413	+	GGGGGCTGTTAGCTGGGCTTGCCTGGGGGTGGG<br>.(((...))).	C1orf94	NM_032884	chr1:34405211-34457318	non-template	33	Intron_1.f	--	Yes	--	Yes	--	--	--	--	Yes	--	--

G4ST01034405380	chr1:34405381-34405413	+	GGGGGCTGTTAGCTGGGCTTGCCTGGGGGTGGG<br>.(((...))).	CSMD2	NM_052896	chr1:33752196-34404030	template	-1350	upstream	Yes	--	--	--	--	--	--	--	Yes	--	--
G4ST01034417542	chr1:34417543-34417574	+	GGGACAGGGCCTTGGCAGGAGGGCCCATGGGG<br>(((....))).	C1orf94	NM_032884	chr1:34405211-34457318	non-template	32	Intron_1.f	--	Yes	--	Yes	--	--	--	--	--	--	--
G4ST01034417542	chr1:34417543-34417574	+	GGGACAGGGCCTTGGCAGGAGGGCCCATGGGG<br>(((....))).	C1orf94	NM_001134734	chr1:34415140-34457318	non-template	32	Intron_1.f	--	Yes	--	Yes	--	--	--	--	--	--	--
G4ST01034450611	chr1:34450612-34450651	+	GGGAGCCTAAGGGTATCCAGAAAGCTGGTGGGAACGGGGG<br>...((((....)))).	C1orf94	NM_032884	chr1:34405211-34457318	non-template	40	Intron_6.l	--	Yes	--	Yes	--	--	--	--	--	--	Yes
G4ST01034450611	chr1:34450612-34450651	+	GGGAGCCTAAGGGTATCCAGAAAGCTGGTGGGAACGGGGG<br>...((((....)))).	C1orf94	NM_001134734	chr1:34415140-34457318	non-template	40	Intron_6.l	--	Yes	--	Yes	--	--	--	--	--	--	Yes
G4ST01034450621	chr1:34450622-34450660	+	GGGTATCCAGAAAGCTGGTGGGAACGGGGGCAAGTTGGG<br>...((((....)))).	C1orf94	NM_032884	chr1:34405211-34457318	non-template	39	Intron_6.l	--	Yes	--	Yes	--	--	--	--	--	--	Yes
G4ST01034450621	chr1:34450622-34450660	+	GGGTATCCAGAAAGCTGGTGGGAACGGGGGCAAGTTGGG<br>...((((....)))).	C1orf94	NM_001134734	chr1:34415140-34457318	non-template	39	Intron_6.l	--	Yes	--	Yes	--	--	--	--	--	--	Yes
G4ST01034451316	chr1:34451317-34451356	-	GGGTGTGCCCGGGAGACTGGGATTCCCAGGAATGACAGGG<br>((((....))))....	C1orf94	NM_032884	chr1:34405211-34457318	template	40	Intron_6.l	--	Yes	--	Yes	--	--	--	--	--	--	--
G4ST01034451316	chr1:34451317-34451356	-	GGGTGTGCCCGGGAGACTGGGATTCCCAGGAATGACAGGG<br>((((....))))....	C1orf94	NM_001134734	chr1:34415140-34457318	template	40	Intron_6.l	--	Yes	--	Yes	--	--	--	--	--	--	--
G4ST01034452637	chr1:34452638-34452673	+	GGGTGGGGTCTAGAGGTGGCTCTAGGGGCCTATGGG<br>..((((((....))))))	C1orf94	NM_032884	chr1:34405211-34457318	non-template	36	Intron_6.l	--	Yes	--	Yes	--	--	--	--	--	--	--
G4ST01034452637	chr1:34452638-34452673	+	GGGTGGGGTCTAGAGGTGGCTCTAGGGGCCTATGGG<br>..((((((....))))))	C1orf94	NM_001134734	chr1:34415140-34457318	non-template	36	Intron_6.l	--	Yes	--	Yes	--	--	--	--	--	--	--
G4ST01034994784	chr1:34994785-34994814	+	GGGACAGGGTTCCAGGGAACACTGTTTGGG<br>(((...))).	GJB5	NM_005268	chr1:34993308-34996699	non-template	30	Intron_1.u	--	Yes	--	Yes	--	--	--	--	--	--	--
G4ST01035001189	chr1:35001190-35001236	-	GGGGGCAGGTCTGGGTGAGCTGATGAGGTCAGCTTGGGAGTGGCGGG<br>..((((((.....)))))).	GJB4	NM_153212	chr1:34997929-35001912	template	47	Exon_2.l	--	Yes	Yes	--	--	--	Yes	Yes	Yes	--	--
G4ST01035020073	chr1:35020074-35020102	+	GGGATGGGGCGCGAAGCGGGGGTGCGGGG<br>.(((...))).	GJB3	NM_024009	chr1:35019377-35024552	non-template	29	Intron_1.u	--	Yes	--	Yes	--	--	--	Yes	Yes	Yes	--
G4ST01035020073	chr1:35020074-35020102	+	GGGATGGGGCGCGAAGCGGGGGTGCGGGG<br>.(((...))).	GJB3	NM_001005752	chr1:35020498-35024554	non-template	-395	upstream	Yes	--	--	--	--	--	--	Yes	Yes	Yes	--
G4ST01035023768	chr1:35023769-35023793	+	GGGCAGGGGTGGGGCAACATGCGGG<br>(((...)))	GJB3	NM_024009	chr1:35019377-35024552	non-template	25	Exon_2.l	--	Yes	Yes	--	--	--	Yes	--	--	--	--
G4ST01035023768	chr1:35023769-35023793	+	GGGCAGGGGTGGGGCAACATGCGGG<br>(((...)))	GJB3	NM_001005752	chr1:35020498-35024554	non-template	25	Exon_2.l	--	Yes	Yes	--	--	--	Yes	--	--	--	--
G4ST01035031001	chr1:35031002-35031039	-	GGGCCTGTGTGATTTGCGGTGGGGAGGGGCAAGAGGGG<br>.(((((.....))))))..	GJA4	NM_002060	chr1:35031186-35033935	template	-146	upstream	Yes	--	--	--	--	--	--	Yes	--	--	--
G4ST01035031010	chr1:35031011-35031047	-	GGGGAGGTGGGCCTGTGTGATTTGCGGTGGGGAGGGG<br>.(((((.....)))))..	GJA4	NM_002060	chr1:35031186-35033935	template	-138	upstream	Yes	--	--	--	--	--	--	Yes	--	--	--
G4ST01035108620	chr1:35108621-35108654	-	GGGGGTTGGGAGAGTCTCTTGGGGAGGTGGAGGG<br>(((...)))..	DLGAP3	NM_001080418	chr1:35103624-35143571	non-template	34	Intron_6	--	Yes	--	Yes	--	--	--	--	--	--	--
G4ST01035123602	chr1:35123603-35123640	-	GGGCTCTGACCCTGGTCGGTCGGGGCTGGGGTGAGGGG<br>..(((((....)))))...	DLGAP3	NM_001080418	chr1:35103624-35143571	non-template	38	Intron_5	--	Yes	--	Yes	--	--	--	--	--	--	--
G4ST01035123922	chr1:35123923-35123956	+	GGGGCGGGGCCTGGCTTCCCGGCGGGATGGGGGG<br>(((.((...)).)))	DLGAP3	NM_001080418	chr1:35103624-35143571	template	34	Exon_5	--	Yes	Yes	--	Yes	--	--	Yes	Yes	--	--
G4ST01035123927	chr1:35123928-35123961	+	GGGGCCTGGCTTCCCGGCGGGATGGGGGGCGGGG<br>(((.((...)).)))	DLGAP3	NM_001080418	chr1:35103624-35143571	template	34	Exon_5	--	Yes	Yes	--	Yes	--	--	Yes	Yes	--	--
G4ST01035137887	chr1:35137888-35137920	+	GGGATGGAGCTCCGGGGGTGGATCCGGGGTGGG<br>.(((....))).	DLGAP3	NM_001080418	chr1:35103624-35143571	template	33	Exon_3	--	Yes	Yes	--	Yes	--	--	--	--	--	--
G4ST01035141913	chr1:35141914-35141958	-	GGGGTACCGGTGGTGGGACAGCCGAAGCTGTGGAGGGGTCCTGGG<br>..(((...))) (((((....)))))....	DLGAP3	NM_001080418	chr1:35103624-35143571	non-template	45	Intron_1.f	--	Yes	--	Yes	--	--	--	--	--	--	--
G4ST01035142527	chr1:35142528-35142555	-	GGGCCGGGATGGATACCCGGGGGCCGGG<br>.(((....))).	DLGAP3	NM_001080418	chr1:35103624-35143571	non-template	28	Exon_1.f	--	Yes	Yes	--	Yes	--	--	--	--	--	--
G4ST01035142650	chr1:35142651-35142682	-	GGGACTTGAGCTTCAAGGGGCGCTCGGGCGGG<br>.(((((...)))))	DLGAP3	NM_001080418	chr1:35103624-35143571	non-template	32	Exon_1.f	--	Yes	Yes	--	Yes	--	--	--	--	--	--
G4ST01035142654	chr1:35142655-35142692	-	GGGTCCTACCGGGACTTGAGCTTCAAGGGGCGCTCGGG<br>.(((((...)))))	DLGAP3	NM_001080418	chr1:35103624-35143571	non-template	38	Exon_1.f	--	Yes	Yes	--	Yes	--	--	--	--	--	--
G4ST01035142662	chr1:35142663-35142702	-	GGGTCCAGATGGGTCCTACCGGGACTTGAGCTTCAAGGGG<br>.(((((...)))))	DLGAP3	NM_001080418	chr1:35103624-35143571	non-template	40	Exon_1.f	--	Yes	Yes	--	Yes	--	--	--	--	--	--
G4ST01035429310	chr1:35429311-35429345	-	GGGGTGGGGTGGGTAAACTAAGTGGTGTTAGTGGG<br>...(((((......)))))	SFPQ	NM_005066	chr1:35421788-35431330	non-template	35	Intron_2	--	Yes	--	Yes	--	--	--	--	Yes	Yes	--
G4ST01035430713	chr1:35430714-35430754	+	GGGACCCCGCTGCTTGGCGGGGTGGGTGGCGGCGCCCCGGG<br>....((((....)))) .(((...)))..	SFPQ	NM_005066	chr1:35421788-35431330	template	41	Exon_1.f	--	Yes	Yes	--	Yes	--	--	Yes	Yes	Yes	--
G4ST01035430731	chr1:35430732-35430759	+	GGGGTGGGTGGCGGCGCCCCGGGAGGGG<br>.(((...)))..	SFPQ	NM_005066	chr1:35421788-35431330	template	28	Exon_1.f	--	Yes	Yes	--	Yes	--	--	Yes	Yes	Yes	--
G4ST01035430736	chr1:35430737-35430783	+	GGGTGGCGGCGCCCCGGGAGGGGCCGAGGTGACTGCAGGCGGCGGGG<br>.(((...))).. ((((((....)).....)))).	SFPQ	NM_005066	chr1:35421788-35431330	template	47	Exon_1.f	--	Yes	Yes	--	Yes	--	--	Yes	Yes	Yes	--
G4ST01035657717	chr1:35657718-35657759	-	GGGTCTATCGGGAATGTGGAGTACCACATGGGGCTATTCGGG<br>.((((((....)))))).	ZMYM4	NM_005095	chr1:35507155-35660132	template	42	Exon_30.l	--	Yes	Yes	--	Yes	--	--	--	--	--	--
G4ST01035657726	chr1:35657727-35657767	-	GGGTTCCAGGGTCTATCGGGAATGTGGAGTACCACATGGGG<br>.((((((....)))))).	ZMYM4	NM_005095	chr1:35507155-35660132	template	41	Exon_30.l	--	Yes	Yes	--	Yes	--	--	--	--	--	--
G4ST01035677809	chr1:35677810-35677842	+	GGGAGGGATAAGAAAGAGCTTTCTAGGGCTGGG<br>...(((((....))))).	KIAA0319L	NM_024874	chr1:35671678-35795624	template	33	Intron_20.l	--	Yes	--	Yes	--	--	--	--	--	--	--
G4ST01035812251	chr1:35812252-35812282	-	GGGCAGCGGTGGCTGCGGGTAGGGCGGCGGG<br>((((....)))).	TFAP2E	NM_178548	chr1:35811558-35833514	template	31	Exon_2	--	Yes	Yes	--	Yes	--	--	--	--	--	Yes
G4ST01035815472	chr1:35815473-35815517	-	GGGCCAAGGTGAAAACTGCTGGGGGCGCCGAGGGCAGGTGTGGGG<br>(((.(((....)))..)))	TFAP2E	NM_178548	chr1:35811558-35833514	template	45	Intron_3	--	Yes	--	Yes	--	--	--	Yes	Yes	Yes	Yes
G4ST01035828606	chr1:35828607-35828640	-	GGGTTGGGCTCTGAGCTCAGAGATGGGAGCTGGG<br>(((((....)))))..	TFAP2E	NM_178548	chr1:35811558-35833514	template	34	Intron_5	--	Yes	--	Yes	--	--	--	--	Yes	--	--
G4ST01035828613	chr1:35828614-35828650	-	GGGGCCATTAGGGTTGGGCTCTGAGCTCAGAGATGGG<br>(((((....)))))..	TFAP2E	NM_178548	chr1:35811558-35833514	template	37	Intron_5	--	Yes	--	Yes	--	--	--	--	--	--	--
G4ST01035956145	chr1:35956146-35956182	-	GGGGAAGGCGGGGCTCCCTGGCTGGGGTAGGAAAGGG<br>(((....)))..	C1orf216	NM_152374	chr1:35952064-35957377	non-template	37	Intron_1.u	--	Yes	--	Yes	--	--	--	Yes	Yes	Yes	--
G4ST01035956852	chr1:35956853-35956880	-	GGGGCCCGAGGCGGGGTGGGGCTTGGGG<br>(((...))).	C1orf216	NM_152374	chr1:35952064-35957377	non-template	28	Intron_1.u	--	Yes	--	Yes	--	--	--	Yes	Yes	Yes	--
G4ST01036007577	chr1:36007578-36007605	-	GGGAGTTCCACACTGGGCCTGGGAGGGG<br>(((.....)))	CLSPN	NM_001190481	chr1:35970300-36008138	non-template	28	Intron_1.f	--	Yes	--	Yes	--	--	--	Yes	Yes	Yes	--
G4ST01036007577	chr1:36007578-36007605	-	GGGAGTTCCACACTGGGCCTGGGAGGGG<br>(((.....)))	CLSPN	NM_022111	chr1:35970300-36008138	non-template	28	Intron_1.f	--	Yes	--	Yes	--	--	--	Yes	Yes	Yes	--
G4ST01036007582	chr1:36007583-36007613	-	GGGTGTTAGGGAGTTCCACACTGGGCCTGGG<br>(((.....)))	CLSPN	NM_001190481	chr1:35970300-36008138	non-template	31	Intron_1.f	--	Yes	--	Yes	--	--	--	Yes	Yes	Yes	--
G4ST01036007582	chr1:36007583-36007613	-	GGGTGTTAGGGAGTTCCACACTGGGCCTGGG<br>(((.....)))	CLSPN	NM_022111	chr1:35970300-36008138	non-template	31	Intron_1.f	--	Yes	--	Yes	--	--	--	Yes	Yes	Yes	--
G4ST01036007588	chr1:36007589-36007623	-	GGGCGAGCGAGGGTGTTAGGGAGTTCCACACTGGG<br>(((.....)))	CLSPN	NM_001190481	chr1:35970300-36008138	non-template	35	Intron_1.f	--	Yes	--	Yes	--	--	--	Yes	Yes	Yes	--
G4ST01036007588	chr1:36007589-36007623	-	GGGCGAGCGAGGGTGTTAGGGAGTTCCACACTGGG<br>(((.....)))	CLSPN	NM_022111	chr1:35970300-36008138	non-template	35	Intron_1.f	--	Yes	--	Yes	--	--	--	Yes	Yes	Yes	--
G4ST01036045781	chr1:36045782-36045814	+	GGGACCAGGGAGGGAATGCATCATTCATCAGGG<br>((((...))))....	EIF2C4	NM_017629	chr1:36046415-36096077	non-template	-600	upstream	Yes	--	--	--	--	--	--	Yes	Yes	Yes	--
G4ST01036046306	chr1:36046307-36046343	-	GGGAGCCAGCGCGCGCGCCCGCCGGGAGGGAAGGGGG<br>.((..(((....)))..)).	EIF2C4	NM_017629	chr1:36046415-36096077	template	-71	upstream	Yes	--	--	--	--	--	--	Yes	Yes	Yes	Yes

G4ST01036046313	chr1:36046314-36046349	-	GGGAGCGGGAGCCAGCGCGCGCCCGCCGGGAGGG<br>.((..(((....)))..)).	EIF2C4	NM_017629	chr1:36046415-36096077	template	-65	upstream	Yes	--	--	--	--	--	--	Yes	Yes	Yes	Yes
G4ST01036046317	chr1:36046318-36046355	-	GGGAGCGGGAGCGGGAGCCAGCGCGCGCCCGCCGGG<br>.((..(((....)))..)).	EIF2C4	NM_017629	chr1:36046415-36096077	template	-59	upstream	Yes	--	--	--	--	--	--	Yes	Yes	Yes	Yes
G4ST01036046429	chr1:36046430-36046468	-	GGGCAGGGGGCGGCGGCGGCGCCCGGCCCGGGGACAGGG<br>(((((((....))))..)))..	EIF2C4	NM_017629	chr1:36046415-36096077	template	39	Exon_1.f	--	Yes	Yes	--	--	Yes	--	--	Yes	Yes	--
G4ST01036046435	chr1:36046436-36046475	-	GGGCGCTGGGCAGGGGGCGGCGGCGGCGCCCGGCCCGGGG<br>(((((((....))))..)))..	EIF2C4	NM_017629	chr1:36046415-36096077	template	40	Exon_1.f	--	Yes	Yes	--	--	Yes	--	--	Yes	Yes	--
G4ST01036121018	chr1:36121019-36121055	+	GGGAGGCAGGGCCCCTGGGCGCTGGAGTGCCAGGGGG<br>..((((....)))).	EIF2C1	NM_012199	chr1:36121397-36162486	non-template	-341	upstream	Yes	--	--	--	--	--	--	--	Yes	--	--
G4ST01036121026	chr1:36121027-36121061	+	GGGCCCCTGGGCGCTGGAGTGCCAGGGGGTCTGGG<br>..((((....)))).	EIF2C1	NM_012199	chr1:36121397-36162486	non-template	-335	upstream	Yes	--	--	--	--	--	--	--	Yes	--	--
G4ST01036121465	chr1:36121466-36121519	+	GGGGGCGGCGGCGCGAGCGGCCGGGCTTGGTAGGGGAGCCGAGCCCGGCCCGGG<br>...(((.((....)).))) ..((((....))))..	EIF2C1	NM_012199	chr1:36121397-36162486	non-template	54	Exon_1.f	--	Yes	Yes	--	--	Yes	--	--	Yes	Yes	--
G4ST01036122124	chr1:36122125-36122154	+	GGGGGCGGTGGGTGGGGACCCAGTCCCGGG<br>(((...)))..	EIF2C1	NM_012199	chr1:36121397-36162486	non-template	30	Intron_1.f	--	Yes	--	Yes	--	--	--	Yes	Yes	--	Yes
G4ST01036158201	chr1:36158202-36158233	+	GGGAAAGTGAGGGGCTTGAGAAAGTGGGTGGG<br>.(((....))).	EIF2C1	NM_012199	chr1:36121397-36162486	non-template	32	Exon_19.l	--	Yes	Yes	--	--	--	Yes	--	--	--	--
G4ST01036158211	chr1:36158212-36158240	+	GGGGCTTGAGAAAGTGGGTGGGAGGAGGG<br>.(((....))).	EIF2C1	NM_012199	chr1:36121397-36162486	non-template	29	Exon_19.l	--	Yes	Yes	--	--	--	Yes	--	--	--	--
G4ST01036322564	chr1:36322565-36322612	-	GGGACGCGTGCCTGTTGGCACTGGGGAGCTGGGGGGCGGCCCCCGGGG<br>....(((((....))))).. ...(((...)))	TEKT2	NM_014466	chr1:36322263-36326463	template	48	Intron_1.f	--	Yes	--	Yes	--	--	--	--	Yes	--	--
G4ST01036327305	chr1:36327306-36327340	+	GGGCGGGGCCGGGCGCAAGTCAGAGGCCGTGCGGG<br>.(((.((.....))..)))	ADPRHL2	NM_017825	chr1:36327040-36332120	non-template	35	Intron_1.f	--	Yes	--	Yes	--	--	--	Yes	Yes	Yes	--
G4ST01036327309	chr1:36327310-36327344	+	GGGGCCGGGCGCAAGTCAGAGGCCGTGCGGGAGGG<br>.(((.((.....))..)))	ADPRHL2	NM_017825	chr1:36327040-36332120	non-template	35	Intron_1.f	--	Yes	--	Yes	--	--	--	Yes	Yes	Yes	--
G4ST01036327352	chr1:36327353-36327387	+	GGGGCGCGGAGCCTCCGGGGGCGACGGGTCGGGGG<br>...((((...)))).	ADPRHL2	NM_017825	chr1:36327040-36332120	non-template	35	Intron_1.f	--	Yes	--	Yes	--	--	--	Yes	Yes	Yes	Yes
G4ST01036330520	chr1:36330521-36330557	-	GGGCAGGGGAGAGGGCTGCATGTGAGAACGAGCAGGG<br>..((.(((....))).)).	ADPRHL2	NM_017825	chr1:36327040-36332120	template	37	Intron_4	--	Yes	--	Yes	--	--	--	--	--	Yes	--
G4ST01036388949	chr1:36388950-36388980	+	GGGCCGAGGGCTCTGGGAAGGAACTTGAGGG<br>(((...)))..	TRAPPC3	NM_014408	chr1:36374760-36387654	template	-1295	upstream	Yes	--	--	--	--	--	--	Yes	Yes	Yes	Yes
G4ST01036388956	chr1:36388957-36388990	+	GGGCTCTGGGAAGGAACTTGAGGGTTGGTTGGGG<br>(((...)))..	TRAPPC3	NM_014408	chr1:36374760-36387654	template	-1302	upstream	Yes	--	--	--	--	--	--	Yes	Yes	Yes	Yes
G4ST01036388963	chr1:36388964-36388997	+	GGGAAGGAACTTGAGGGTTGGTTGGGGTTGGGGG<br>(((...)))..	TRAPPC3	NM_014408	chr1:36374760-36387654	template	-1309	upstream	Yes	--	--	--	--	--	--	Yes	Yes	Yes	Yes
G4ST01036396345	chr1:36396346-36396369	+	GGGCGGGGAGGGCTGCGTCAGGGG<br>(((...)))	MAP7D1	NM_018067	chr1:36394390-36419028	non-template	24	Intron_1.f	--	Yes	--	Yes	--	--	--	--	--	--	--
G4ST01036396349	chr1:36396350-36396377	+	GGGGAGGGCTGCGTCAGGGGCCGGTGGG<br>(((...)))	MAP7D1	NM_018067	chr1:36394390-36419028	non-template	28	Intron_1.f	--	Yes	--	Yes	--	--	--	--	--	--	--
G4ST01036399161	chr1:36399162-36399189	+	GGGCAGCCCTGCTGGGGCTGAGGGAGGG<br>((((...))))	MAP7D1	NM_018067	chr1:36394390-36419028	non-template	28	Intron_1.f	--	Yes	--	Yes	--	--	--	Yes	Yes	Yes	Yes
G4ST01036414542	chr1:36414543-36414574	+	GGGGCCGGGCAGGGGCCAGCCTGGCGCGCGGG<br>((((...))))....	MAP7D1	NM_018067	chr1:36394390-36419028	non-template	32	Exon_7	--	Yes	Yes	--	Yes	--	--	Yes	Yes	--	--
G4ST01036415940	chr1:36415941-36415980	+	GGGGGCGGGGGGCGGCGCTGATGTAGCCTGGGGGAGGGGG<br>.(((.(((....))).)))...	MAP7D1	NM_018067	chr1:36394390-36419028	non-template	40	Intron_8	--	Yes	--	Yes	--	--	--	--	--	--	--
G4ST01036417022	chr1:36417023-36417046	+	GGGCGGGAAGCGGCTGGGCGCGGG<br>.((...))	MAP7D1	NM_018067	chr1:36394390-36419028	non-template	24	Intron_11	--	Yes	--	Yes	--	--	--	Yes	Yes	--	Yes
G4ST01036417026	chr1:36417027-36417055	+	GGGAAGCGGCTGGGCGCGGGCGCCGCGGG<br>.((...))	MAP7D1	NM_018067	chr1:36394390-36419028	non-template	29	Intron_11	--	Yes	--	Yes	--	--	--	Yes	Yes	--	Yes
G4ST01036417601	chr1:36417602-36417633	+	GGGAGGCCGCTGCGGCCCGGGCAAGGGGAGGG<br>.(((((...))))).	MAP7D1	NM_018067	chr1:36394390-36419028	non-template	32	Intron_13	--	Yes	--	Yes	--	--	--	--	--	--	--
G4ST01036542017	chr1:36542018-36542057	+	GGGTGAGCCGGGGCCGGGGCCGAGGAGCCTTTCCTCGGGG<br>..((((((.....))))))	THRAP3	NM_005119	chr1:36462604-36543544	non-template	40	Exon_12.l	--	Yes	Yes	--	Yes	--	--	--	--	--	--
G4ST01036542026	chr1:36542027-36542063	+	GGGGCCGGGGCCGAGGAGCCTTTCCTCGGGGTCGGGG<br>..((((((.....))))))	THRAP3	NM_005119	chr1:36462604-36543544	non-template	37	Exon_12.l	--	Yes	Yes	--	Yes	--	--	--	--	--	--
G4ST01036556439	chr1:36556440-36556475	+	GGGGCATGGGTTTGGGAAGCAAGAGATTGGTTTGGG<br>((.(((....))).)).	SH3D21	NM_024676	chr1:36545295-36559535	non-template	36	Intron_7	--	Yes	--	Yes	--	--	--	--	--	--	--
G4ST01036556439	chr1:36556440-36556475	+	GGGGCATGGGTTTGGGAAGCAAGAGATTGGTTTGGG<br>((.(((....))).)).	SH3D21	NM_001162530	chr1:36544581-36559535	non-template	36	Intron_10	--	Yes	--	Yes	--	--	--	--	--	--	--
G4ST01036559319	chr1:36559320-36559356	+	GGGCCTGGGAAGGGACCGCGGCCTGACCTGGCTGGGG<br>....(((((......)))))	SH3D21	NM_024676	chr1:36545295-36559535	non-template	37	Exon_13.l	--	Yes	Yes	--	--	--	Yes	Yes	Yes	Yes	Yes
G4ST01036559319	chr1:36559320-36559356	+	GGGCCTGGGAAGGGACCGCGGCCTGACCTGGCTGGGG<br>....(((((......)))))	SH3D21	NM_001162530	chr1:36544581-36559535	non-template	37	Exon_16.l	--	Yes	Yes	--	--	--	Yes	Yes	Yes	Yes	Yes
G4ST01036560287	chr1:36560288-36560315	-	GGGGGCGGGGAGGGGGCAGCATGCAGGG<br>.(((...))).	FAM176B	NM_018166	chr1:36560219-36562342	non-template	28	Exon_3.l	--	Yes	Yes	--	--	--	Yes	--	Yes	Yes	--
G4ST01036562797	chr1:36562798-36562834	-	GGGGGAGTAGGGGTCTGCAGCCCGGACAGGGAGAGGG<br>(((((.....))))).	FAM176B	NM_018166	chr1:36560219-36562342	non-template	-455	upstream	Yes	--	--	--	--	--	--	Yes	--	--	--
G4ST01036562803	chr1:36562804-36562840	-	GGGACTGGGGGAGTAGGGGTCTGCAGCCCGGACAGGG<br>(((((.....))))).	FAM176B	NM_018166	chr1:36560219-36562342	non-template	-461	upstream	Yes	--	--	--	--	--	--	Yes	--	--	--
G4ST01036581478	chr1:36581479-36581511	-	GGGACAGGCTTCTGGGGGCAGGCAGGGCAAGGG<br>.(((....))).	STK40	NM_032017	chr1:36577812-36624072	non-template	33	Intron_11.l	--	Yes	--	Yes	--	--	--	--	Yes	--	--
G4ST01036592384	chr1:36592385-36592412	-	GGGATGGGATGAACATGGGAATTGGGGG<br>((....))	STK40	NM_032017	chr1:36577812-36624072	non-template	28	Intron_7	--	Yes	--	Yes	--	--	--	--	--	--	--
G4ST01036592393	chr1:36592394-36592418	-	GGGCTCGGGATGGGATGAACATGGG<br>((....))	STK40	NM_032017	chr1:36577812-36624072	non-template	25	Intron_7	--	Yes	--	Yes	--	--	--	--	--	--	--
G4ST01036593678	chr1:36593679-36593724	+	GGGCCTGGGACTCTCCTCTGGAGTGGGACACTCAAACCAGTGAGGG<br>...((((...)))). .((((......)))).	STK40	NM_032017	chr1:36577812-36624072	template	46	Intron_5	--	Yes	--	Yes	--	--	--	--	--	--	--
G4ST01036600501	chr1:36600502-36600543	+	GGGGAAGGTCAGGGTCACAGGATGCCCAGTGTGGGTGCAGGG<br>.(((.((....)).))).	STK40	NM_032017	chr1:36577812-36624072	template	42	Intron_2	--	Yes	--	Yes	--	--	--	--	--	--	--
G4ST01036600512	chr1:36600513-36600550	+	GGGTCACAGGATGCCCAGTGTGGGTGCAGGGCTCCGGG<br>.(((.((....)).))).	STK40	NM_032017	chr1:36577812-36624072	template	38	Intron_2	--	Yes	--	Yes	--	--	--	--	--	--	--
G4ST01036604707	chr1:36604708-36604737	-	GGGGCAGGGAAAGGGGGGTCTGTCCCTGGG<br>(((.....))).	STK40	NM_032017	chr1:36577812-36624072	non-template	30	Intron_2	--	Yes	--	Yes	--	--	--	Yes	Yes	--	--
G4ST01036623292	chr1:36623293-36623320	+	GGGGCCAAGTGGCGTGGGAATGGGCGGG<br>(((....)))..	STK40	NM_032017	chr1:36577812-36624072	template	28	Intron_1.f	--	Yes	--	Yes	--	--	--	Yes	Yes	Yes	--
G4ST01036699005	chr1:36699006-36699041	-	GGGCCCTTGGGGGGAGGAGGGCTAGTTCTTAGAGGG<br>(((.....))).	MRPS15	NM_031280	chr1:36693949-36702627	non-template	36	Intron_4	--	Yes	--	Yes	--	--	--	--	--	--	--
G4ST01036705560	chr1:36705561-36705594	-	GGGCAGCTGCACCTCTTCAGGTGGGGTGGGTGGG<br>......(((((....)))))	CSF3R	NM_000760	chr1:36704231-36721502	non-template	34	Intron_15	--	Yes	--	Yes	--	--	--	--	--	--	--
G4ST01036705560	chr1:36705561-36705594	-	GGGCAGCTGCACCTCTTCAGGTGGGGTGGGTGGG<br>......(((((....)))))	CSF3R	NM_156039	chr1:36704231-36721502	non-template	34	Intron_15	--	Yes	--	Yes	--	--	--	--	--	--	--
G4ST01036705560	chr1:36705561-36705594	-	GGGCAGCTGCACCTCTTCAGGTGGGGTGGGTGGG<br>......(((((....)))))	CSF3R	NM_172313	chr1:36704231-36721502	non-template	34	Intron_15	--	Yes	--	Yes	--	--	--	--	--	--	--
G4ST01037057006	chr1:37057007-37057038	-	GGGACCTGCTTAGGGGGCAGGCAGGGAAGGGG<br>.(((....)))	GRIK3	NM_000831	chr1:37033715-37272431	non-template	32	Intron_12	--	Yes	--	Yes	--	--	--	--	--	--	Yes
G4ST01037079881	chr1:37079882-37079911	-	GGGAGCATGGGAGGGCTCAGGACCCTGGGG<br>..(((....)))	GRIK3	NM_000831	chr1:37033715-37272431	non-template	30	Intron_10	--	Yes	--	Yes	--	--	--	--	--	--	--
G4ST01037088146	chr1:37088147-37088178	+	GGGAAAGGCTTTTGCCTGGGAACTGGGGTGGG<br>..((((....))))	GRIK3	NM_000831	chr1:37033715-37272431	template	32	Intron_9	--	Yes	--	Yes	--	--	--	--	--	--	--

G4ST01037097469	chr1:37097470-37097498	-	GGGTGGGCTGAGGACTGGAGGGCCTTGGG<br>(((.....))).	GRIK3	NM_000831	chr1:37033715-37272431	non-template	29	Intron_6	--	Yes	--	Yes	--	--	--	Yes	--	--	Yes
G4ST01037097476	chr1:37097477-37097503	-	GGGCAGGGTGGGCTGAGGACTGGAGGG<br>(((.....))).	GRIK3	NM_000831	chr1:37033715-37272431	non-template	27	Intron_6	--	Yes	--	Yes	--	--	--	Yes	--	--	Yes
G4ST01037100819	chr1:37100820-37100863	-	GGGAGCCGCCCTGGAGGAGCTGAGGGAGGGAGACTTCTTCTGGG<br>(((..((.....)).))).. (((.....)))	GRIK3	NM_000831	chr1:37033715-37272431	non-template	44	Intron_5	--	Yes	--	Yes	--	--	--	--	--	--	--
G4ST01037108730	chr1:37108731-37108764	-	GGGCTGTGACTCTTCAAAGGCGGGAGGGAATGGG<br>((.(((....))).))..	GRIK3	NM_000831	chr1:37033715-37272431	non-template	34	Intron_4	--	Yes	--	Yes	--	--	--	--	--	--	--
G4ST01037136310	chr1:37136311-37136340	+	GGGAAGGGTAGTGGTAGGCTGGGTGGGGGG<br>.(((.....)))	GRIK3	NM_000831	chr1:37033715-37272431	template	30	Intron_1.f	--	Yes	--	Yes	--	--	--	--	--	--	--
G4ST01037136315	chr1:37136316-37136349	+	GGGTAGTGGTAGGCTGGGTGGGGGGAGGTGGGGG<br>.(((.....)))	GRIK3	NM_000831	chr1:37033715-37272431	template	34	Intron_1.f	--	Yes	--	Yes	--	--	--	--	--	--	--
G4ST01037220209	chr1:37220210-37220243	-	GGGAGGAACTGGGAGCAGCGCTGGGTGCCTGGGG<br>(((...)))	GRIK3	NM_000831	chr1:37033715-37272431	non-template	34	Intron_1.f	--	Yes	--	Yes	--	--	--	--	--	--	--
G4ST01037220218	chr1:37220219-37220250	-	GGGAGTAGGGAGGAACTGGGAGCAGCGCTGGG<br>(((...)))	GRIK3	NM_000831	chr1:37033715-37272431	non-template	32	Intron_1.f	--	Yes	--	Yes	--	--	--	--	--	--	--
G4ST01037265990	chr1:37265991-37266028	-	GGGGCCAGGGGAGCCTGGGATAATGGTGGCTGCCAGGG<br>....((((....))))	GRIK3	NM_000831	chr1:37033715-37272431	non-template	38	Intron_1.f	--	Yes	--	Yes	--	--	--	--	--	--	--
G4ST01037272378	chr1:37272379-37272415	-	GGGAGCCGGGCGGCTGGGCCAGGCGGCGCCTTCGGGG<br>..((((...))))...	GRIK3	NM_000831	chr1:37033715-37272431	non-template	37	Exon_1.f	--	Yes	Yes	--	--	Yes	--	--	--	--	--
G4ST01037712649	chr1:37712650-37712683	-	GGGGCCGTCCCGGGAGACCGCCCCGGGGGCGGGG<br>...(((...)))	ZC3H12A	NM_025079	chr1:37712706-37722565	template	-22	upstream	Yes	--	--	--	--	--	--	Yes	Yes	Yes	Yes
G4ST01037712654	chr1:37712655-37712688	-	GGGGCGGGGCCGTCCCGGGAGACCGCCCCGGGGG<br>...(((...)))	ZC3H12A	NM_025079	chr1:37712706-37722565	template	-17	upstream	Yes	--	--	--	--	--	--	Yes	Yes	Yes	Yes
G4ST01037722161	chr1:37722162-37722195	-	GGGGTAGGGCGGAAGTGGGGTGGCGTGCACAGGG<br>(((.....))).	ZC3H12A	NM_025079	chr1:37712706-37722565	template	34	Exon_6.l	--	Yes	Yes	--	--	--	Yes	--	Yes	Yes	--
G4ST01037752775	chr1:37752776-37752802	+	GGGCTTGGGGCCTGGAGGCGGGGCGGG<br>((((....))).	MEAF6	NM_022756	chr1:37730763-37752951	template	27	Intron_1.f	--	Yes	--	Yes	--	--	--	Yes	Yes	Yes	--
G4ST01037852339	chr1:37852340-37852364	-	GGGTGGGCTGGGGCACTGTGCAGGG<br>(((...))).	RSPO1	NM_001038633	chr1:37849538-37873078	non-template	25	Intron_6	--	Yes	--	Yes	--	--	--	--	--	--	--
G4ST01037853252	chr1:37853253-37853290	+	GGGTGGGTCTTCCAGGGCTTGGCTGGAAGCAGGCAGGG<br>..((.(((....))).))	RSPO1	NM_001038633	chr1:37849538-37873078	template	38	Intron_5	--	Yes	--	Yes	--	--	--	--	--	--	Yes
G4ST01037859778	chr1:37859779-37859819	+	GGGGGTGAGGGGCAGGGGCAAGGTGACCTCGAGATGCTGGG<br>((((((...))).....))).	RSPO1	NM_001038633	chr1:37849538-37873078	template	41	Intron_4	--	Yes	--	Yes	--	--	--	--	--	--	--
G4ST01037863628	chr1:37863629-37863653	+	GGGACTCAGGTGGGATGGGTTAGGG<br>((....))	RSPO1	NM_001038633	chr1:37849538-37873078	template	25	Intron_4	--	Yes	--	Yes	--	--	--	--	--	--	--
G4ST01037863639	chr1:37863640-37863664	+	GGGATGGGTTAGGGACAGTGGTGGG<br>((....))	RSPO1	NM_001038633	chr1:37849538-37873078	template	25	Intron_4	--	Yes	--	Yes	--	--	--	--	--	--	--
G4ST01037871902	chr1:37871903-37871933	+	GGGGGCAGAAGAATTGCGGGCGGGGGGCGGG<br>.((((.....))))	RSPO1	NM_001038633	chr1:37849538-37873078	template	31	Intron_1.f	--	Yes	--	Yes	--	--	--	--	--	--	--
G4ST01037872420	chr1:37872421-37872456	+	GGGGTGGGGGTCACCAGAGACCGTGGGACTTGGGGG<br>((((......))))..	RSPO1	NM_001038633	chr1:37849538-37873078	template	36	Intron_1.f	--	Yes	--	Yes	--	--	--	--	--	--	--
G4ST01037955018	chr1:37955019-37955049	-	GGGAAAGGGAAGGTTCTTTCCTTGGGGTGGG<br>((((......)))).	EPHA10	NM_001099439	chr1:37954233-38003411	non-template	31	Exon_17.l	--	Yes	Yes	--	--	--	Yes	--	--	--	--
G4ST01037955022	chr1:37955023-37955062	-	GGGCCTTGCAGGTGGGAAAGGGAAGGTTCTTTCCTTGGGG<br>(((...))). ((((......)))).	EPHA10	NM_001099439	chr1:37954233-38003411	non-template	40	Exon_17.l	--	Yes	Yes	--	--	--	Yes	--	--	--	--
G4ST01037991875	chr1:37991876-37991909	+	GGGGTCGAGGGCGGATGAGCCCGGGGCAAGCGGG<br>(((......)))	EPHA10	NM_001099439	chr1:37954233-38003411	template	34	Intron_4	--	Yes	--	Yes	--	--	--	--	--	--	Yes
G4ST01037991883	chr1:37991884-37991917	+	GGGCGGATGAGCCCGGGGCAAGCGGGAGCAGGGG<br>(((......)))	EPHA10	NM_001099439	chr1:37954233-38003411	template	34	Intron_4	--	Yes	--	Yes	--	--	--	--	--	--	Yes
G4ST01038148845	chr1:38148846-38148883	-	GGGTAAGGGTGATTAGGTTTTAATGGGATGGTAAGGGG<br>..((((.....))))	INPP5B	NM_005540	chr1:38098956-38185316	non-template	38	Intron_7	--	Yes	--	Yes	--	--	--	--	--	--	--
G4ST01038169854	chr1:38169855-38169891	-	GGGGGCGGCGGTGCGCCCGGCCCTCGGGTGGGCAGGG<br>(((((((...)))))..)))...	INPP5B	NM_005540	chr1:38098956-38185316	non-template	37	Intron_7	--	Yes	--	Yes	--	--	--	Yes	Yes	Yes	Yes
G4ST01038169859	chr1:38169860-38169896	-	GGGCCGGGGGCGGCGGTGCGCCCGGCCCTCGGGTGGG<br>(((((((...)))))..)))...	INPP5B	NM_005540	chr1:38098956-38185316	non-template	37	Intron_7	--	Yes	--	Yes	--	--	--	Yes	Yes	Yes	Yes
G4ST01038169863	chr1:38169864-38169904	-	GGGGAGCTGGGCCGGGGGCGGCGGTGCGCCCGGCCCTCGGG<br>(((((((...)))))..)))...	INPP5B	NM_005540	chr1:38098956-38185316	non-template	41	Intron_7	--	Yes	--	Yes	--	--	--	Yes	Yes	Yes	Yes
G4ST01038170000	chr1:38170001-38170030	-	GGGGGCGGAGCGCGCGGGACGGGCGGCGGG<br>.(((.....)))	INPP5B	NM_005540	chr1:38098956-38185316	non-template	30	Intron_7	--	Yes	--	Yes	--	--	--	Yes	Yes	Yes	Yes
G4ST01038170007	chr1:38170008-38170040	-	GGGTTCGTCTGGGGGCGGAGCGCGCGGGACGGG<br>.(((.....)))	INPP5B	NM_005540	chr1:38098956-38185316	non-template	33	Intron_7	--	Yes	--	Yes	--	--	--	Yes	Yes	Yes	Yes
G4ST01038170012	chr1:38170013-38170046	-	GGGGGCGGGTTCGTCTGGGGGCGGAGCGCGCGGG<br>.(((.....)))	INPP5B	NM_005540	chr1:38098956-38185316	non-template	34	Intron_7	--	Yes	--	Yes	--	--	--	Yes	Yes	Yes	Yes
G4ST01038184136	chr1:38184137-38184172	-	GGGACAGCCTGGGTGGGGAGAGGCCTCGCCTCTGGG<br>.((((((...))))))	INPP5B	NM_005540	chr1:38098956-38185316	non-template	36	Intron_2	--	Yes	--	Yes	--	--	--	--	Yes	Yes	Yes
G4ST01038185155	chr1:38185156-38185180	-	GGGAGTGGGCAAGCTGAGGGCTGGG<br>((...)).	INPP5B	NM_005540	chr1:38098956-38185316	non-template	25	Intron_1.f	--	Yes	--	Yes	--	--	--	Yes	Yes	Yes	Yes
G4ST01038185160	chr1:38185161-38185188	-	GGGCAGGAGGGAGTGGGCAAGCTGAGGG<br>((...)).	INPP5B	NM_005540	chr1:38098956-38185316	non-template	28	Intron_1.f	--	Yes	--	Yes	--	--	--	Yes	Yes	Yes	Yes
G4ST01038243562	chr1:38243563-38243591	+	GGGCGCGGGGGCGGACCGCCAGGGCTGGG<br>((((...)))).	FHL3	NM_004468	chr1:38235029-38243821	template	29	Intron_1.f	--	Yes	--	Yes	--	--	--	Yes	Yes	Yes	Yes
G4ST01038243568	chr1:38243569-38243599	+	GGGGGCGGACCGCCAGGGCTGGGAGCCGGGG<br>((((...)))).	FHL3	NM_004468	chr1:38235029-38243821	template	31	Intron_1.f	--	Yes	--	Yes	--	--	--	Yes	Yes	Yes	Yes
G4ST01038282486	chr1:38282487-38282530	-	GGGGAGGCCGGGAGAGAAATTCCCCAAGTTTGGGGTGAGGTGGG<br>....(((((.....))))).	POU3F1	NM_002699	chr1:38282110-38285037	non-template	44	Exon_1.u	--	Yes	Yes	--	--	--	Yes	Yes	--	--	Yes
G4ST01038282495	chr1:38282496-38282539	-	GGGATGCCAGGGGAGGCCGGGAGAGAAATTCCCCAAGTTTGGGG<br>....(((((.....))))).	POU3F1	NM_002699	chr1:38282110-38285037	non-template	44	Exon_1.u	--	Yes	Yes	--	--	--	Yes	Yes	--	--	Yes
G4ST01039097581	chr1:39097582-39097610	-	GGGGACGCCCGGGAGGGCGGCGGCCGGGG<br>(((...)))	RRAGC	NM_022157	chr1:39077606-39097927	non-template	29	Intron_1.f	--	Yes	--	Yes	--	--	--	Yes	Yes	Yes	--
G4ST01039166174	chr1:39166175-39166201	+	GGGGAGGGCTGGGAGAAGAGTCTCGGG<br>(((....))).	RHBDL2	NM_017821	chr1:39124066-39180043	template	27	Intron_1.f	--	Yes	--	Yes	--	--	--	--	--	--	--
G4ST01039167880	chr1:39167881-39167917	+	GGGGAGCTCAGGAGGCTGGGCAGCGGGCTGTGCTGGG<br>.((((.....))))	RHBDL2	NM_017821	chr1:39124066-39180043	template	37	Intron_1.f	--	Yes	--	Yes	--	--	--	Yes	--	--	--
G4ST01039179885	chr1:39179886-39179924	-	GGGAGGGAGTGGAGGGAGTCCTATGTCAGGACAAGGGGG<br>.(((((.....)))))....	RHBDL2	NM_017821	chr1:39124066-39180043	non-template	39	Intron_1.f	--	Yes	--	Yes	--	--	--	--	Yes	Yes	Yes
G4ST01039229695	chr1:39229696-39229735	-	GGGCAGAGGGGCGCAGCGCCGCCGCTTCGGGGAGCCGGGG<br>.((.((((....)))).))	AKIRIN1	NM_001136275	chr1:39229503-39244324	template	40	Exon_1.f	--	Yes	Yes	--	Yes	--	--	--	Yes	Yes	--
G4ST01039229695	chr1:39229696-39229735	-	GGGCAGAGGGGCGCAGCGCCGCCGCTTCGGGGAGCCGGGG<br>.((.((((....)))).))	AKIRIN1	NM_024595	chr1:39229503-39244324	template	40	Exon_1.f	--	Yes	Yes	--	Yes	--	--	--	Yes	Yes	--
G4ST01039229703	chr1:39229704-39229741	-	GGGGCCGGGCAGAGGGGCGCAGCGCCGCCGCTTCGGGG<br>.((.((((....)))).))	AKIRIN1	NM_001136275	chr1:39229503-39244324	template	38	Exon_1.f	--	Yes	Yes	--	Yes	--	--	--	Yes	Yes	--
G4ST01039229703	chr1:39229704-39229741	-	GGGGCCGGGCAGAGGGGCGCAGCGCCGCCGCTTCGGGG<br>.((.((((....)))).))	AKIRIN1	NM_024595	chr1:39229503-39244324	template	38	Exon_1.f	--	Yes	Yes	--	Yes	--	--	--	Yes	Yes	--
G4ST01039229796	chr1:39229797-39229831	-	GGGCGGGGCGGGCTGCTGCAGACTCTGCGGTGGGG<br>...((((((...))))))..	AKIRIN1	NM_001136275	chr1:39229503-39244324	template	35	Exon_1.f	--	Yes	Yes	--	Yes	--	--	--	Yes	Yes	--
G4ST01039229796	chr1:39229797-39229831	-	GGGCGGGGCGGGCTGCTGCAGACTCTGCGGTGGGG<br>...((((((...))))))..	AKIRIN1	NM_024595	chr1:39229503-39244324	template	35	Exon_1.f	--	Yes	Yes	--	Yes	--	--	--	Yes	Yes	--
G4ST01039230023	chr1:39230024-39230050	+	GGGAGGCATCCGGGAGGGGTAATGGGG<br>.((...))	AKIRIN1	NM_001136275	chr1:39229503-39244324	non-template	27	Intron_1.f	--	Yes	--	Yes	--	--	--	Yes	Yes	Yes	Yes

G4ST01039230023	chr1:39230024-39230050	+	GGGAGGCATCCGGGAGGGGTAATGGGG<br>.((...))	AKIRIN1	NM_024595	chr1:39229503-39244324	non-template	27	Intron_1.f	--	Yes	--	Yes	--	--	--	Yes	Yes	Yes	Yes
G4ST01039319424	chr1:39319425-39319457	+	GGGAGGGCCTGGGGGCGGCCAGAGGACGCGGGG<br>.(((.((...)).))).	MACF1	NM_012090	chr1:39319676-39725397	non-template	-218	upstream	Yes	--	--	--	--	--	--	Yes	--	--	Yes
G4ST01039320159	chr1:39320160-39320195	-	GGGCGCTGGGGCCAGCGGCCGAGCCCTGGGGCCGGG<br>..(((.(((...))))))	MACF1	NM_012090	chr1:39319676-39725397	template	36	Intron_1.f	--	Yes	--	Yes	--	--	--	--	--	--	--
G4ST01039322738	chr1:39322739-39322774	+	GGGAGGAATGGGCCAGGGCACAGCAGCTGTGTGGGG<br>(((((...)))))..	MACF1	NM_012090	chr1:39319676-39725397	non-template	36	Intron_3	--	Yes	--	Yes	--	--	--	--	--	--	Yes
G4ST01039322747	chr1:39322748-39322784	+	GGGCCAGGGCACAGCAGCTGTGTGGGGAGCCGAGGGG<br>(((((...)))))..	MACF1	NM_012090	chr1:39319676-39725397	non-template	37	Intron_3	--	Yes	--	Yes	--	--	--	--	--	--	Yes
G4ST01039340693	chr1:39340694-39340746	+	GGGGGGGGGGTCAAGGAAGGCTTTCCCCTGGGGAGTTTCAGCAGAAGCATGGG<br>(((..(((.....)))..))).. ..(((((....)))))..	MACF1	NM_012090	chr1:39319676-39725397	non-template	53	Intron_3	--	Yes	--	Yes	--	--	--	--	Yes	--	--
G4ST01039433826	chr1:39433827-39433862	+	GGGAGGGAGAAGTTTACTTCTCACTGGGATGGAGGG<br>(((((....)))))....	MACF1	NM_012090	chr1:39319676-39725397	non-template	36	Intron_3	--	Yes	--	Yes	--	--	--	--	--	--	--
G4ST01039468085	chr1:39468086-39468115	-	GGGAGGGTGGGAGGATCACTTGAACCTGGG<br>(((.((....)).)))	MACF1	NM_012090	chr1:39319676-39725397	template	30	Intron_3	--	Yes	--	Yes	--	--	--	Yes	--	--	--
G4ST01039527156	chr1:39527157-39527188	-	GGGATCAGAGGGAGGTGGGGATGATTCATGGG<br>.(((...)))	MACF1	NM_012090	chr1:39319676-39725397	template	32	Intron_16	--	Yes	--	Yes	--	--	--	Yes	Yes	--	--
G4ST01039647262	chr1:39647263-39647304	+	GGGGGCTGCCCGGGCGGGGCGGCGCAGCGCGGCGGCGCGGGG<br>(((.(((......))).))).	KIAA0754	NM_015038	chr1:39647763-39654741	non-template	-458	upstream	Yes	--	--	--	--	--	--	Yes	Yes	Yes	--
G4ST01039647262	chr1:39647263-39647304	+	GGGGGCTGCCCGGGCGGGGCGGCGCAGCGCGGCGGCGCGGGG<br>(((.(((......))).))).	MACF1	NM_012090	chr1:39319676-39725397	non-template	42	Intron_54	--	Yes	--	Yes	--	--	--	Yes	Yes	Yes	--
G4ST01039647262	chr1:39647263-39647304	+	GGGGGCTGCCCGGGCGGGGCGGCGCAGCGCGGCGGCGCGGGG<br>(((.(((......))).))).	MACF1	NM_033044	chr1:39569397-39725397	non-template	42	Intron_22	--	Yes	--	Yes	--	--	--	Yes	Yes	Yes	--
G4ST01039647273	chr1:39647274-39647311	+	GGGCGGGGCGGCGCAGCGCGGCGGCGCGGGGAAGGGGG<br>(((.(((......))).))).	KIAA0754	NM_015038	chr1:39647763-39654741	non-template	-451	upstream	Yes	--	--	--	--	--	--	Yes	Yes	Yes	--
G4ST01039647273	chr1:39647274-39647311	+	GGGCGGGGCGGCGCAGCGCGGCGGCGCGGGGAAGGGGG<br>(((.(((......))).))).	MACF1	NM_012090	chr1:39319676-39725397	non-template	38	Intron_54	--	Yes	--	Yes	--	--	--	Yes	Yes	Yes	--
G4ST01039647273	chr1:39647274-39647311	+	GGGCGGGGCGGCGCAGCGCGGCGGCGCGGGGAAGGGGG<br>(((.(((......))).))).	MACF1	NM_033044	chr1:39569397-39725397	non-template	38	Intron_22	--	Yes	--	Yes	--	--	--	Yes	Yes	Yes	--
G4ST01039729840	chr1:39729841-39729876	+	GGGGGTGGGCGGGCGGCACAGACGGCTGAGCCGGGG<br>((((.(((....))).))))	BMP8A	NM_181809	chr1:39729905-39768128	non-template	-28	upstream	Yes	--	--	--	--	--	--	Yes	Yes	Yes	--
G4ST01039730315	chr1:39730316-39730347	-	GGGGACAGCCGGGCGGGGGTCGCAGGCCGGGG<br>((((...)))).	BMP8A	NM_181809	chr1:39729905-39768128	template	32	Exon_1.f	--	Yes	Yes	--	Yes	--	--	--	--	--	--
G4ST01039730631	chr1:39730632-39730664	+	GGGGAGGGGCGCGCATCCGCGGGCGGTGCCGGG<br>..(((....)))	BMP8A	NM_181809	chr1:39729905-39768128	non-template	33	Intron_1.f	--	Yes	--	Yes	--	--	--	--	--	--	--
G4ST01039730804	chr1:39730805-39730840	-	GGGTGTGTCTGGGGCAGGGTGAAGGCAGCGCCTGGG<br>...((((...))))	BMP8A	NM_181809	chr1:39729905-39768128	template	36	Intron_1.f	--	Yes	--	Yes	--	--	--	--	--	--	--
G4ST01039731460	chr1:39731461-39731493	+	GGGGGAGCTGTGGGGCTGAGAGCAGGGAGTGGG<br>(((...))).	BMP8A	NM_181809	chr1:39729905-39768128	non-template	33	Intron_1.f	--	Yes	--	Yes	--	--	--	--	--	--	--
G4ST01039737319	chr1:39737320-39737352	-	GGGCCGGGATGGAGCCATGTGGGTGGACATGGG<br>(((....)))..	BMP8A	NM_181809	chr1:39729905-39768128	template	33	Intron_1.f	--	Yes	--	Yes	--	--	--	--	--	--	--
G4ST01039739482	chr1:39739483-39739514	-	GGGACCTAGTGGGAGGTGTCTGGGTCATGGGG<br>((....))	BMP8A	NM_181809	chr1:39729905-39768128	template	32	Intron_1.f	--	Yes	--	Yes	--	--	--	--	--	--	--
G4ST01039748368	chr1:39748369-39748409	-	GGGCTGCGCCTCTGGCCAGTGGGAAGCACAGGGGACGGGGG<br>(((.(((...)))))).	BMP8A	NM_181809	chr1:39729905-39768128	template	41	Intron_1.f	--	Yes	--	Yes	--	--	--	--	--	--	Yes
G4ST01039756946	chr1:39756947-39756981	+	GGGAGGCTCTGGGCTCTGGAAGGAGAGGGCTGGGG<br>((((....)))).	BMP8A	NM_181809	chr1:39729905-39768128	non-template	35	Intron_3	--	Yes	--	Yes	--	--	--	--	--	--	--
G4ST01039756956	chr1:39756957-39756988	+	GGGCTCTGGAAGGAGAGGGCTGGGGATGGGGG<br>((((....)))).	BMP8A	NM_181809	chr1:39729905-39768128	non-template	32	Intron_3	--	Yes	--	Yes	--	--	--	--	--	--	--
G4ST01039757023	chr1:39757024-39757058	-	GGGGGGTGGTGAAGGGGACTAACAGTGGGTCTGGG<br>.(((...)))	BMP8A	NM_181809	chr1:39729905-39768128	template	35	Intron_3	--	Yes	--	Yes	--	--	--	--	--	--	--
G4ST01039757029	chr1:39757030-39757067	-	GGGCAAGATGGGGGGTGGTGAAGGGGACTAACAGTGGG<br>.(((...)))	BMP8A	NM_181809	chr1:39729905-39768128	template	38	Intron_3	--	Yes	--	Yes	--	--	--	--	--	--	--
G4ST01039760026	chr1:39760027-39760058	-	GGGGGAGAGAGGGAGACTTCTGGGCAGCAGGG<br>((....))	BMP8A	NM_181809	chr1:39729905-39768128	template	32	Intron_4	--	Yes	--	Yes	--	--	--	--	--	--	--
G4ST01039763468	chr1:39763469-39763503	+	GGGAGGCCAGGGTGTGGGGAGGCAGAGCCTCTGGG<br>(((((...))))).	PPIEL	NR_003929	chr1:39760539-39797957	template	35	Intron_8.l	--	Yes	--	Yes	--	--	--	--	--	--	Yes
G4ST01039763468	chr1:39763469-39763503	+	GGGAGGCCAGGGTGTGGGGAGGCAGAGCCTCTGGG<br>(((((...))))).	BMP8A	NM_181809	chr1:39729905-39768128	non-template	35	Intron_6.l	--	Yes	--	Yes	--	--	--	--	--	--	Yes
G4ST01039763477	chr1:39763478-39763507	+	GGGTGTGGGGAGGCAGAGCCTCTGGGGGGG<br>(((((...))))).	PPIEL	NR_003929	chr1:39760539-39797957	template	30	Intron_8.l	--	Yes	--	Yes	--	--	--	--	--	--	Yes
G4ST01039763477	chr1:39763478-39763507	+	GGGTGTGGGGAGGCAGAGCCTCTGGGGGGG<br>(((((...))))).	BMP8A	NM_181809	chr1:39729905-39768128	non-template	30	Intron_6.l	--	Yes	--	Yes	--	--	--	--	--	--	Yes
G4ST01039763483	chr1:39763484-39763516	+	GGGGAGGCAGAGCCTCTGGGGGGGTCTGAAGGG<br>(((((...))))).	PPIEL	NR_003929	chr1:39760539-39797957	template	33	Intron_8.l	--	Yes	--	Yes	--	--	--	--	--	--	Yes
G4ST01039763483	chr1:39763484-39763516	+	GGGGAGGCAGAGCCTCTGGGGGGGTCTGAAGGG<br>(((((...))))).	BMP8A	NM_181809	chr1:39729905-39768128	non-template	33	Intron_6.l	--	Yes	--	Yes	--	--	--	--	--	--	Yes
G4ST01039777588	chr1:39777589-39777621	+	GGGACTGGGCGGCGTGATTGCCCAGGGCATGGG<br>.((((....))))..	PPIEL	NR_003929	chr1:39760539-39797957	template	33	Intron_7	--	Yes	--	Yes	--	--	--	Yes	Yes	--	--
G4ST01039812426	chr1:39812427-39812463	+	GGGCCTCATCTGTGTGGGGGGTAGTTGGGAAAAGGGG<br>((.((....)).)).	PABPC4	NM_001135654	chr1:39799072-39815108	template	37	Intron_1.f	--	Yes	--	Yes	--	--	--	--	--	Yes	--
G4ST01039812426	chr1:39812427-39812463	+	GGGCCTCATCTGTGTGGGGGGTAGTTGGGAAAAGGGG<br>((.((....)).)).	PABPC4	NM_001135653	chr1:39799072-39815108	template	37	Intron_1.f	--	Yes	--	Yes	--	--	--	--	--	Yes	--
G4ST01039812426	chr1:39812427-39812463	+	GGGCCTCATCTGTGTGGGGGGTAGTTGGGAAAAGGGG<br>((.((....)).)).	PABPC4	NM_003819	chr1:39799072-39815108	template	37	Intron_1.f	--	Yes	--	Yes	--	--	--	--	--	Yes	--
G4ST01039814677	chr1:39814678-39814716	+	GGGCGCCGACTCGGCGAGCCCCGGGCGGGCGGCGAAGGG<br>(((((...)))))......	PABPC4	NM_001135654	chr1:39799072-39815108	template	39	Exon_1.f	--	Yes	Yes	--	--	Yes	--	Yes	Yes	Yes	--
G4ST01039814677	chr1:39814678-39814716	+	GGGCGCCGACTCGGCGAGCCCCGGGCGGGCGGCGAAGGG<br>(((((...)))))......	PABPC4	NM_001135653	chr1:39799072-39815108	template	39	Exon_1.f	--	Yes	Yes	--	--	Yes	--	Yes	Yes	Yes	--
G4ST01039814677	chr1:39814678-39814716	+	GGGCGCCGACTCGGCGAGCCCCGGGCGGGCGGCGAAGGG<br>(((((...)))))......	PABPC4	NM_003819	chr1:39799072-39815108	template	39	Exon_1.f	--	Yes	Yes	--	--	Yes	--	Yes	Yes	Yes	--
G4ST01039864116	chr1:39864117-39864146	+	GGGAGGAGGGTGGGAGCAGGCTCTGCTGGG<br>(((((...)))))	HEYL	NM_014571	chr1:39861690-39877935	template	30	Exon_5.l	--	Yes	Yes	--	--	--	Yes	Yes	Yes	--	--
G4ST01039864467	chr1:39864468-39864501	+	GGGCAGGAGAGGGGTCTGGGCGGATCTCTGAGGG<br>(((....))).	HEYL	NM_014571	chr1:39861690-39877935	template	34	Exon_5.l	--	Yes	Yes	--	--	--	Yes	--	--	--	--
G4ST01039874067	chr1:39874068-39874100	-	GGGCTGGAAGGGAGCTCTGGCTGGGCTCTTGGG<br>(((....)))	HEYL	NM_014571	chr1:39861690-39877935	non-template	33	Intron_1.f	--	Yes	--	Yes	--	--	--	--	--	--	--
G4ST01039877739	chr1:39877740-39877770	-	GGGCGAGCGGGGTGGGGGTCGGCCCGACGGG<br>.((((...))))	HEYL	NM_014571	chr1:39861690-39877935	non-template	31	Intron_1.f	--	Yes	--	Yes	--	--	--	--	Yes	Yes	--
G4ST01039920676	chr1:39920677-39920718	+	GGGTGGGCCAGAGAGGGGGGCTGGAGTGTCCCTCCTCCTGGG<br>.((..((((.....)))).)).	HPCAL4	NM_016257	chr1:39917232-39929676	template	42	Exon_4.l	--	Yes	Yes	--	--	--	Yes	--	--	--	--
G4ST01039922526	chr1:39922527-39922564	-	GGGGTGCGGGGGAGGTGAGGCACCCGGGGTGGCGGGGG<br>...((((...))))..	HPCAL4	NM_016257	chr1:39917232-39929676	non-template	38	Intron_2	--	Yes	--	Yes	--	--	--	--	--	--	--
G4ST01039928912	chr1:39928913-39928949	+	GGGGCCTCTGCCTAGCACTGGGGGCCACTGGGGAGGG<br>.((..(((...)))..))	HPCAL4	NM_016257	chr1:39917232-39929676	template	37	Intron_1.f	--	Yes	--	Yes	--	--	--	--	Yes	--	--
G4ST01039929028	chr1:39929029-39929057	+	GGGGTGGGATGTGGGGACAGTGTCTGGGG<br>..(((...)))	HPCAL4	NM_016257	chr1:39917232-39929676	template	29	Intron_1.f	--	Yes	--	Yes	--	--	--	--	--	--	--
G4ST01039929895	chr1:39929896-39929931	-	GGGGGCTGAGCCTCAGGGCAGGGGTGCAGATGGGGG<br>(((...)))...	HPCAL4	NM_016257	chr1:39917232-39929676	non-template	-219	upstream	Yes	--	--	--	--	--	--	Yes	Yes	Yes	--

G4ST01039999214	chr1:39999215-39999250	-	GGGTGTGGGAGGCAGAGCCTCTGGGGGTCCGAAGGG<br>((((...))))....	PPIE	NM_001195007	chr1:39977104-40002173	template	36	Intron_9	--	Yes	--	Yes	--	--	--	--	--	--	Yes
G4ST01039999214	chr1:39999215-39999250	-	GGGTGTGGGAGGCAGAGCCTCTGGGGGTCCGAAGGG<br>((((...))))....	PPIE	NM_203456	chr1:39977104-40002173	template	36	Intron_9.l	--	Yes	--	Yes	--	--	--	--	--	--	Yes
G4ST01039999214	chr1:39999215-39999250	-	GGGTGTGGGAGGCAGAGCCTCTGGGGGTCCGAAGGG<br>((((...))))....	BMP8B	NM_001720	chr1:39996490-40027120	non-template	36	Intron_6.l	--	Yes	--	Yes	--	--	--	--	--	--	Yes
G4ST01039999223	chr1:39999224-39999259	-	GGGAGGCCAGGGTGTGGGAGGCAGAGCCTCTGGGGG<br>((((...))))....	PPIE	NM_001195007	chr1:39977104-40002173	template	36	Intron_9	--	Yes	--	Yes	--	--	--	--	--	--	Yes
G4ST01039999223	chr1:39999224-39999259	-	GGGAGGCCAGGGTGTGGGAGGCAGAGCCTCTGGGGG<br>((((...))))....	PPIE	NM_203456	chr1:39977104-40002173	template	36	Intron_9.l	--	Yes	--	Yes	--	--	--	--	--	--	Yes
G4ST01039999223	chr1:39999224-39999259	-	GGGAGGCCAGGGTGTGGGAGGCAGAGCCTCTGGGGG<br>((((...))))....	BMP8B	NM_001720	chr1:39996490-40027120	non-template	36	Intron_6.l	--	Yes	--	Yes	--	--	--	--	--	--	Yes
G4ST01039999842	chr1:39999843-39999868	-	GGGAGGTGACACCAGGGTGGGCAGGG<br>.(((...))).	PPIE	NM_001195007	chr1:39977104-40002173	template	26	Intron_9	--	Yes	--	Yes	--	--	--	--	--	--	--
G4ST01039999842	chr1:39999843-39999868	-	GGGAGGTGACACCAGGGTGGGCAGGG<br>.(((...))).	PPIE	NM_203456	chr1:39977104-40002173	template	26	Intron_9.l	--	Yes	--	Yes	--	--	--	--	--	--	--
G4ST01039999842	chr1:39999843-39999868	-	GGGAGGTGACACCAGGGTGGGCAGGG<br>.(((...))).	BMP8B	NM_001720	chr1:39996490-40027120	non-template	26	Intron_6.l	--	Yes	--	Yes	--	--	--	--	--	--	--
G4ST01039999847	chr1:39999848-39999875	-	GGGCGGAGGGAGGTGACACCAGGGTGGG<br>.(((...))).	PPIE	NM_001195007	chr1:39977104-40002173	template	28	Intron_9	--	Yes	--	Yes	--	--	--	--	--	--	--
G4ST01039999847	chr1:39999848-39999875	-	GGGCGGAGGGAGGTGACACCAGGGTGGG<br>.(((...))).	PPIE	NM_203456	chr1:39977104-40002173	template	28	Intron_9.l	--	Yes	--	Yes	--	--	--	--	--	--	--
G4ST01039999847	chr1:39999848-39999875	-	GGGCGGAGGGAGGTGACACCAGGGTGGG<br>.(((...))).	BMP8B	NM_001720	chr1:39996490-40027120	non-template	28	Intron_6.l	--	Yes	--	Yes	--	--	--	--	--	--	--
G4ST01039999851	chr1:39999852-39999882	-	GGGAGGAGGGCGGAGGGAGGTGACACCAGGG<br>.(((...))).	PPIE	NM_001195007	chr1:39977104-40002173	template	31	Intron_9	--	Yes	--	Yes	--	--	--	--	--	--	--
G4ST01039999851	chr1:39999852-39999882	-	GGGAGGAGGGCGGAGGGAGGTGACACCAGGG<br>.(((...))).	PPIE	NM_203456	chr1:39977104-40002173	template	31	Intron_9.l	--	Yes	--	Yes	--	--	--	--	--	--	--
G4ST01039999851	chr1:39999852-39999882	-	GGGAGGAGGGCGGAGGGAGGTGACACCAGGG<br>.(((...))).	BMP8B	NM_001720	chr1:39996490-40027120	non-template	31	Intron_6.l	--	Yes	--	Yes	--	--	--	--	--	--	--
G4ST01040001319	chr1:40001320-40001355	+	GGGGGCTGGGCAGGATGGGCATGGTACTGACCAGGG<br>..((((....))))	PPIE	NM_001195007	chr1:39977104-40002173	non-template	36	Intron_10.l	--	Yes	--	Yes	--	--	--	--	--	--	--
G4ST01040001319	chr1:40001320-40001355	+	GGGGGCTGGGCAGGATGGGCATGGTACTGACCAGGG<br>..((((....))))	PPIE	NM_203456	chr1:39977104-40002173	non-template	36	Intron_9.l	--	Yes	--	Yes	--	--	--	--	--	--	--
G4ST01040001319	chr1:40001320-40001355	+	GGGGGCTGGGCAGGATGGGCATGGTACTGACCAGGG<br>..((((....))))	BMP8B	NM_001720	chr1:39996490-40027120	template	36	Exon_6-Intron_6.l	--	Yes	Yes	Yes	Yes	--	--	--	--	--	--
G4ST01040002652	chr1:40002653-40002684	+	GGGGGAGAGAGGGAGACTTCTGGGCAGCAGGG<br>((....))	BMP8B	NM_001720	chr1:39996490-40027120	template	32	Intron_4	--	Yes	--	Yes	--	--	--	--	--	--	--
G4ST01040005648	chr1:40005649-40005686	+	GGGCAAGATGGGGGGTGATGAAGGGGACTAACAGTGGG<br>.(((...)))	BMP8B	NM_001720	chr1:39996490-40027120	template	38	Intron_3	--	Yes	--	Yes	--	--	--	--	--	--	--
G4ST01040005657	chr1:40005658-40005692	+	GGGGGGTGATGAAGGGGACTAACAGTGGGTCTGGG<br>.(((...)))	BMP8B	NM_001720	chr1:39996490-40027120	template	35	Intron_3	--	Yes	--	Yes	--	--	--	--	--	--	--
G4ST01040005727	chr1:40005728-40005759	-	GGGCTCTGGAAGGAGAGGGCTGGGGATGGGGG<br>((((....)))).	BMP8B	NM_001720	chr1:39996490-40027120	non-template	32	Intron_3	--	Yes	--	Yes	--	--	--	--	--	--	--
G4ST01040005734	chr1:40005735-40005769	-	GGGAGGCTCTGGGCTCTGGAAGGAGAGGGCTGGGG<br>((((....)))).	BMP8B	NM_001720	chr1:39996490-40027120	non-template	35	Intron_3	--	Yes	--	Yes	--	--	--	--	--	--	--
G4ST01040014323	chr1:40014324-40014364	+	GGGCTGCGCCTCTGGCCAGTGGGAAGCACAGGGGACGGGGG<br>(((.(((...)))))).	BMP8B	NM_001720	chr1:39996490-40027120	template	41	Intron_1.f	--	Yes	--	Yes	--	--	--	--	--	--	Yes
G4ST01040016295	chr1:40016296-40016328	+	GGGCCGGGACGGAGCCACGTGGGTGGACATGGG<br>(((......)))	BMP8B	NM_001720	chr1:39996490-40027120	template	33	Intron_1.f	--	Yes	--	Yes	--	--	--	--	--	--	--
G4ST01040026154	chr1:40026155-40026190	+	GGGTGTGTTTGGGGCAGGGTGAAGGCAGCGCCTGGG<br>...((((...))))	BMP8B	NM_001720	chr1:39996490-40027120	template	36	Intron_1.f	--	Yes	--	Yes	--	--	--	--	--	--	--
G4ST01040026330	chr1:40026331-40026363	-	GGGGAGGGGCGCGCATCCGCGGGCTGTGCCGGG<br>..(((....)))	BMP8B	NM_001720	chr1:39996490-40027120	non-template	33	Intron_1.f	--	Yes	--	Yes	--	--	--	Yes	--	--	--
G4ST01040026647	chr1:40026648-40026679	+	GGGGACAGCCGGGCGGGGGTCGCAGGCCGGGG<br>((((...)))).	BMP8B	NM_001720	chr1:39996490-40027120	template	32	Exon_1.f	--	Yes	Yes	--	Yes	--	--	--	--	--	--
G4ST01040027193	chr1:40027194-40027228	+	GGGGCCTGGGAGCGGGGGCCACGCCTGCGAGGGGG<br>..((.((....)).))	BMP8B	NM_001720	chr1:39996490-40027120	template	-73	upstream	Yes	--	--	--	--	--	--	Yes	Yes	Yes	Yes
G4ST01040027200	chr1:40027201-40027235	+	GGGAGCGGGGGCCACGCCTGCGAGGGGGCCCGGGG<br>..((.((....)).))	BMP8B	NM_001720	chr1:39996490-40027120	template	-80	upstream	Yes	--	--	--	--	--	--	Yes	Yes	Yes	Yes
G4ST01040027206	chr1:40027207-40027254	+	GGGGGCCACGCCTGCGAGGGGGCCCGGGGCTGCGGCCGGTGTCGCGGG<br>..((.((....)).)) ...(((((....)))))	BMP8B	NM_001720	chr1:39996490-40027120	template	-86	upstream	Yes	--	--	--	--	--	--	Yes	Yes	Yes	Yes
G4ST01040027223	chr1:40027224-40027259	+	GGGGGCCCGGGGCTGCGGCCGGTGTCGCGGGTGGGG<br>...(((((....)))))	BMP8B	NM_001720	chr1:39996490-40027120	template	-103	upstream	Yes	--	--	--	--	--	--	Yes	Yes	Yes	--
G4ST01040399672	chr1:40399673-40399726	+	GGGAGACGCCGCCGCTGTCGCCGGGGCTGGGGCTGAGGCTCCGGCGGTAGCGGG<br>.(((((....)).))).... ((((..((....))..))))	RLF	NM_012421	chr1:40399628-40479180	non-template	54	Exon_1.f	--	Yes	Yes	--	Yes	--	--	Yes	Yes	Yes	--
G4ST01040549357	chr1:40549358-40549397	-	GGGCAAACGCGGGATTCTGGGTGATCCTGGCCACCAGGGG<br>.....((((...))))	COL9A2	NM_001852	chr1:40538750-40555526	non-template	40	Exon_12	--	Yes	Yes	--	Yes	--	--	--	Yes	--	Yes
G4ST01040554875	chr1:40554876-40554906	-	GGGCAGAGACTGGGCCCGGTCGGGTGCTGGG<br>.((...))	COL9A2	NM_001852	chr1:40538750-40555526	non-template	31	Intron_1.f	--	Yes	--	Yes	--	--	--	Yes	Yes	Yes	Yes
G4ST01040554882	chr1:40554883-40554911	-	GGGTAGGGCAGAGACTGGGCCCGGTCGGG<br>.((...))	COL9A2	NM_001852	chr1:40538750-40555526	non-template	29	Intron_1.f	--	Yes	--	Yes	--	--	--	Yes	Yes	Yes	Yes
G4ST01040554892	chr1:40554893-40554919	-	GGGCGCGCGGGTAGGGCAGAGACTGGG<br>.((...))	COL9A2	NM_001852	chr1:40538750-40555526	non-template	27	Intron_1.f	--	Yes	--	Yes	--	--	--	Yes	Yes	Yes	Yes
G4ST01040554951	chr1:40554952-40554986	+	GGGTAGGCGGGCAGGGCCGAGAGGCCGCCTCGGGG<br>.((((.......))))	COL9A2	NM_001852	chr1:40538750-40555526	template	35	Intron_1.f	--	Yes	--	Yes	--	--	--	Yes	Yes	Yes	Yes
G4ST01040556393	chr1:40556394-40556424	-	GGGAGGGAAGGGAAGGCTCTCTGGAGTCGGG<br>..(((((....)))))	COL9A2	NM_001852	chr1:40538750-40555526	non-template	-867	upstream	Yes	--	--	--	--	--	--	--	--	--	--
G4ST01040654386	chr1:40654387-40654430	-	GGGCAAAGAGGGCGGGGGGAGAATTGAAATGGTTCTCATGGGGG<br>..(((((((.....)))))))....	SMAP2	NM_001198980	chr1:40635059-40661585	template	44	Intron_7	--	Yes	--	Yes	--	--	--	--	--	--	--
G4ST01040654386	chr1:40654387-40654430	-	GGGCAAAGAGGGCGGGGGGAGAATTGAAATGGTTCTCATGGGGG<br>..(((((((.....)))))))....	SMAP2	NM_001198978	chr1:40612892-40661585	template	44	Intron_7	--	Yes	--	Yes	--	--	--	--	--	--	--
G4ST01040654386	chr1:40654387-40654430	-	GGGCAAAGAGGGCGGGGGGAGAATTGAAATGGTTCTCATGGGGG<br>..(((((((.....)))))))....	SMAP2	NM_001198979	chr1:40631604-40661585	template	44	Intron_7	--	Yes	--	Yes	--	--	--	--	--	--	--
G4ST01040654386	chr1:40654387-40654430	-	GGGCAAAGAGGGCGGGGGGAGAATTGAAATGGTTCTCATGGGGG<br>..(((((((.....)))))))....	SMAP2	NM_022733	chr1:40611965-40661585	template	44	Intron_7	--	Yes	--	Yes	--	--	--	--	--	--	--
G4ST01040878766	chr1:40878767-40878804	-	GGGATGGTGAGGGGGCGGGGGCTCCGGAGGATCCAGGG<br>((.(((...))).)).	RIMS3	NM_014747	chr1:40858939-40903911	non-template	38	Intron_3	--	Yes	--	Yes	--	--	--	--	--	--	--
G4ST01040903930	chr1:40903931-40903971	+	GGGTGCAAAGCCCAAGCTTGGGCCGGCGGGAGGCGAAGGGG<br>....((((....))))	RIMS3	NM_014747	chr1:40858939-40903911	template	-19	upstream	Yes	--	--	--	--	--	--	Yes	Yes	Yes	Yes
G4ST01040930482	chr1:40930483-40930517	+	GGGGATGCGCGCGTCTTGGGCTAGGGCGGCTGGGG<br>((((....))))..	LOC100130557	NR_024567	chr1:40927339-40930520	template	35	Exon_1.u	--	Yes	Yes	--	--	--	--	Yes	Yes	Yes	--
G4ST01040930482	chr1:40930483-40930517	+	GGGGATGCGCGCGTCTTGGGCTAGGGCGGCTGGGG<br>((((....))))..	NFYC	NM_001142590	chr1:40930267-41009862	non-template	35	Intron_1.f	--	Yes	--	Yes	--	--	--	Yes	Yes	Yes	--
G4ST01040930482	chr1:40930483-40930517	+	GGGGATGCGCGCGTCTTGGGCTAGGGCGGCTGGGG<br>((((....))))..	NFYC	NM_001142587	chr1:40929829-41009862	non-template	35	Intron_1.f	--	Yes	--	Yes	--	--	--	Yes	Yes	Yes	--
G4ST01040930482	chr1:40930483-40930517	+	GGGGATGCGCGCGTCTTGGGCTAGGGCGGCTGGGG<br>((((....))))..	NFYC	NM_014223	chr1:40929829-41009862	non-template	35	Intron_1.f	--	Yes	--	Yes	--	--	--	Yes	Yes	Yes	--
G4ST01040930482	chr1:40930483-40930517	+	GGGGATGCGCGCGTCTTGGGCTAGGGCGGCTGGGG<br>((((....))))..	NFYC	NM_001142589	chr1:40929829-41009862	non-template	35	Intron_1.f	--	Yes	--	Yes	--	--	--	Yes	Yes	Yes	--

G4ST01040930482	chr1:40930483-40930517	+	GGGGATGCGCGCGTCTTGGGCTAGGGCGGCTGGGG<br>((((....))))..	NFYC	NM_001142588	chr1:40929829-41009862	non-template	35	Intron_1.f	--	Yes	--	Yes	--	--	--	Yes	Yes	Yes	--
G4ST01040998600	chr1:40998601-40998637	+	GGGTCGCATGGGCCCGGGAGTTGGAGACCAGCCTGGG<br>.(((((...)))))..	NFYC	NM_001142590	chr1:40930267-41009862	non-template	37	Intron_11	--	Yes	--	Yes	--	--	--	--	--	--	--
G4ST01040998600	chr1:40998601-40998637	+	GGGTCGCATGGGCCCGGGAGTTGGAGACCAGCCTGGG<br>.(((((...)))))..	NFYC	NM_001142587	chr1:40929829-41009862	non-template	37	Intron_6	--	Yes	--	Yes	--	--	--	--	--	--	--
G4ST01040998600	chr1:40998601-40998637	+	GGGTCGCATGGGCCCGGGAGTTGGAGACCAGCCTGGG<br>.(((((...)))))..	NFYC	NM_014223	chr1:40929829-41009862	non-template	37	Intron_6	--	Yes	--	Yes	--	--	--	--	--	--	--
G4ST01040998600	chr1:40998601-40998637	+	GGGTCGCATGGGCCCGGGAGTTGGAGACCAGCCTGGG<br>.(((((...)))))..	NFYC	NM_001142589	chr1:40929829-41009862	non-template	37	Intron_5	--	Yes	--	Yes	--	--	--	--	--	--	--
G4ST01040998600	chr1:40998601-40998637	+	GGGTCGCATGGGCCCGGGAGTTGGAGACCAGCCTGGG<br>.(((((...)))))..	NFYC	NM_001142588	chr1:40929829-41009862	non-template	37	Intron_6	--	Yes	--	Yes	--	--	--	--	--	--	--
G4ST01041021913	chr1:41021914-41021952	-	GGGAACGCGCGGAGCAGGCGGCGGGGCAGGGCGCACGGG<br>..(((((...))..)))...	KCNQ4	NM_004700	chr1:41022271-41078711	template	-318	upstream	Yes	--	--	--	--	--	--	Yes	Yes	Yes	--
G4ST01041021913	chr1:41021914-41021952	-	GGGAACGCGCGGAGCAGGCGGCGGGGCAGGGCGCACGGG<br>..(((((...))..)))...	KCNQ4	NM_172163	chr1:41022271-41078711	template	-318	upstream	Yes	--	--	--	--	--	--	Yes	Yes	Yes	--
G4ST01041021921	chr1:41021922-41021956	-	GGGAGGGAACGCGCGGAGCAGGCGGCGGGGCAGGG<br>..(((((...))..)))...	KCNQ4	NM_004700	chr1:41022271-41078711	template	-314	upstream	Yes	--	--	--	--	--	--	Yes	Yes	Yes	--
G4ST01041021921	chr1:41021922-41021956	-	GGGAGGGAACGCGCGGAGCAGGCGGCGGGGCAGGG<br>..(((((...))..)))...	KCNQ4	NM_172163	chr1:41022271-41078711	template	-314	upstream	Yes	--	--	--	--	--	--	Yes	Yes	Yes	--
G4ST01041022816	chr1:41022817-41022843	-	GGGCTTAGGGTCGGGCTCAGCGAGGGG<br>(((...)))	KCNQ4	NM_004700	chr1:41022271-41078711	template	27	Intron_1.f	--	Yes	--	Yes	--	--	--	Yes	--	--	Yes
G4ST01041022816	chr1:41022817-41022843	-	GGGCTTAGGGTCGGGCTCAGCGAGGGG<br>(((...)))	KCNQ4	NM_172163	chr1:41022271-41078711	template	27	Intron_1.f	--	Yes	--	Yes	--	--	--	Yes	--	--	Yes
G4ST01041026285	chr1:41026286-41026315	+	GGGGTCAGAAGCTGTGGGAATGGGGAGGGG<br>..(((...))).	KCNQ4	NM_004700	chr1:41022271-41078711	non-template	30	Intron_1.f	--	Yes	--	Yes	--	--	--	--	--	--	--
G4ST01041026285	chr1:41026286-41026315	+	GGGGTCAGAAGCTGTGGGAATGGGGAGGGG<br>..(((...))).	KCNQ4	NM_172163	chr1:41022271-41078711	non-template	30	Intron_1.f	--	Yes	--	Yes	--	--	--	--	--	--	--
G4ST01041026300	chr1:41026301-41026339	+	GGGAATGGGGAGGGGAGGAGGTGGCAGCAGCTTCTGGGG<br>.(((((.((....)).))))).	KCNQ4	NM_004700	chr1:41022271-41078711	non-template	39	Intron_1.f	--	Yes	--	Yes	--	--	--	--	--	--	--
G4ST01041026300	chr1:41026301-41026339	+	GGGAATGGGGAGGGGAGGAGGTGGCAGCAGCTTCTGGGG<br>.(((((.((....)).))))).	KCNQ4	NM_172163	chr1:41022271-41078711	non-template	39	Intron_1.f	--	Yes	--	Yes	--	--	--	--	--	--	--
G4ST01041030906	chr1:41030907-41030947	+	GGGGATTGGTGGCAGTCTGGTGGGAGAGACGGGGGACGGGG<br>(((((....)))))....	KCNQ4	NM_004700	chr1:41022271-41078711	non-template	41	Intron_1.f	--	Yes	--	Yes	--	--	--	--	--	--	--
G4ST01041030906	chr1:41030907-41030947	+	GGGGATTGGTGGCAGTCTGGTGGGAGAGACGGGGGACGGGG<br>(((((....)))))....	KCNQ4	NM_172163	chr1:41022271-41078711	non-template	41	Intron_1.f	--	Yes	--	Yes	--	--	--	--	--	--	--
G4ST01041041832	chr1:41041833-41041866	-	GGGTGGTTGGGAGGGTCTTTAACTGGAAGCTGGG<br>.((((.....))))..	KCNQ4	NM_004700	chr1:41022271-41078711	template	34	Intron_1.f	--	Yes	--	Yes	--	--	--	Yes	--	--	--
G4ST01041041832	chr1:41041833-41041866	-	GGGTGGTTGGGAGGGTCTTTAACTGGAAGCTGGG<br>.((((.....))))..	KCNQ4	NM_172163	chr1:41022271-41078711	template	34	Intron_1.f	--	Yes	--	Yes	--	--	--	Yes	--	--	--
G4ST01041049888	chr1:41049889-41049917	+	GGGATAGTCATGGGATGGGGAGATGGGGG<br>((....))	KCNQ4	NM_004700	chr1:41022271-41078711	non-template	29	Intron_1.f	--	Yes	--	Yes	--	--	--	Yes	--	--	--
G4ST01041049888	chr1:41049889-41049917	+	GGGATAGTCATGGGATGGGGAGATGGGGG<br>((....))	KCNQ4	NM_172163	chr1:41022271-41078711	non-template	29	Intron_1.f	--	Yes	--	Yes	--	--	--	Yes	--	--	--
G4ST01041054903	chr1:41054904-41054933	-	GGGAGGGGAATTGGGCAGTGTGGAGCTGGG<br>.(((.....)))	KCNQ4	NM_004700	chr1:41022271-41078711	template	30	Intron_1.f	--	Yes	--	Yes	--	--	--	--	--	--	--
G4ST01041054903	chr1:41054904-41054933	-	GGGAGGGGAATTGGGCAGTGTGGAGCTGGG<br>.(((.....)))	KCNQ4	NM_172163	chr1:41022271-41078711	template	30	Intron_1.f	--	Yes	--	Yes	--	--	--	--	--	--	--
G4ST01041057157	chr1:41057158-41057189	+	GGGGGCGGGGCCGGAGAGGCGGGGCTTGAGGG<br>(((.....))).	KCNQ4	NM_004700	chr1:41022271-41078711	non-template	32	Intron_4	--	Yes	--	Yes	--	--	--	Yes	--	--	--
G4ST01041057157	chr1:41057158-41057189	+	GGGGGCGGGGCCGGAGAGGCGGGGCTTGAGGG<br>(((.....))).	KCNQ4	NM_172163	chr1:41022271-41078711	non-template	32	Intron_4	--	Yes	--	Yes	--	--	--	Yes	--	--	--
G4ST01041058004	chr1:41058005-41058043	-	GGGAAGGGGCCCCTGGTCACCCACGGGGGCTGGTTAGGG<br>..((.(((....))).))	KCNQ4	NM_004700	chr1:41022271-41078711	template	39	Intron_5	--	Yes	--	Yes	--	--	--	--	--	--	--
G4ST01041058004	chr1:41058005-41058043	-	GGGAAGGGGCCCCTGGTCACCCACGGGGGCTGGTTAGGG<br>..((.(((....))).))	KCNQ4	NM_172163	chr1:41022271-41078711	template	39	Intron_5	--	Yes	--	Yes	--	--	--	--	--	--	--
G4ST01041058786	chr1:41058787-41058823	+	GGGCCTGGGGAGGGGCGGAGCCCAGACAGGAGCTGGG<br>....(((((.....)).)))	KCNQ4	NM_004700	chr1:41022271-41078711	non-template	37	Intron_7	--	Yes	--	Yes	--	--	--	Yes	Yes	--	--
G4ST01041058786	chr1:41058787-41058823	+	GGGCCTGGGGAGGGGCGGAGCCCAGACAGGAGCTGGG<br>....(((((.....)).)))	KCNQ4	NM_172163	chr1:41022271-41078711	non-template	37	Intron_7	--	Yes	--	Yes	--	--	--	Yes	Yes	--	--
G4ST01041067483	chr1:41067484-41067513	+	GGGGTTGCACGGACCGGGCAGGGTGGAGGG<br>(((.....))).	KCNQ4	NM_004700	chr1:41022271-41078711	non-template	30	Intron_9	--	Yes	--	Yes	--	--	--	--	--	--	--
G4ST01041067483	chr1:41067484-41067513	+	GGGGTTGCACGGACCGGGCAGGGTGGAGGG<br>(((.....))).	KCNQ4	NM_172163	chr1:41022271-41078711	non-template	30	Intron_8	--	Yes	--	Yes	--	--	--	--	--	--	--
G4ST01041068972	chr1:41068973-41069007	-	GGGCCTGGCGCAGGTGAGTGGGCAGAGGGTGGGGG<br>((((...))))....	KCNQ4	NM_004700	chr1:41022271-41078711	template	35	Intron_9	--	Yes	--	Yes	--	--	--	--	--	--	--
G4ST01041068972	chr1:41068973-41069007	-	GGGCCTGGCGCAGGTGAGTGGGCAGAGGGTGGGGG<br>((((...))))....	KCNQ4	NM_172163	chr1:41022271-41078711	template	35	Intron_8	--	Yes	--	Yes	--	--	--	--	--	--	--
G4ST01041068978	chr1:41068979-41069014	-	GGGAGCTGGGCCTGGCGCAGGTGAGTGGGCAGAGGG<br>((((...))))....	KCNQ4	NM_004700	chr1:41022271-41078711	template	36	Intron_9	--	Yes	--	Yes	--	--	--	--	--	--	--
G4ST01041068978	chr1:41068979-41069014	-	GGGAGCTGGGCCTGGCGCAGGTGAGTGGGCAGAGGG<br>((((...))))....	KCNQ4	NM_172163	chr1:41022271-41078711	template	36	Intron_8	--	Yes	--	Yes	--	--	--	--	--	--	--
G4ST01041076172	chr1:41076173-41076199	-	GGGCGGGGGCTTCGGCCGGGGAGGGGG<br>(((....))).	KCNQ4	NM_004700	chr1:41022271-41078711	template	27	Intron_13.l	--	Yes	--	Yes	--	--	--	--	--	--	--
G4ST01041076172	chr1:41076173-41076199	-	GGGCGGGGGCTTCGGCCGGGGAGGGGG<br>(((....))).	KCNQ4	NM_172163	chr1:41022271-41078711	template	27	Intron_12.l	--	Yes	--	Yes	--	--	--	--	--	--	--
G4ST01041077017	chr1:41077018-41077050	-	GGGCAGGGCACTGGGGGGCCCAGAGGCCGCGGG<br>.((((....))))..	KCNQ4	NM_004700	chr1:41022271-41078711	template	33	Exon_14.l	--	Yes	Yes	--	--	--	Yes	--	--	--	--
G4ST01041077017	chr1:41077018-41077050	-	GGGCAGGGCACTGGGGGGCCCAGAGGCCGCGGG<br>.((((....))))..	KCNQ4	NM_172163	chr1:41022271-41078711	template	33	Exon_13.l	--	Yes	Yes	--	--	--	Yes	--	--	--	--
G4ST01041217879	chr1:41217880-41217914	-	GGGGCGGGGCGGGGCCTGGAGTGCGCAGGCGCGGG<br>(((((.......)))))..	CTPS	NM_001905	chr1:41217594-41250822	template	35	Exon_1.f	--	Yes	Yes	--	--	Yes	--	Yes	Yes	Yes	Yes
G4ST01041218334	chr1:41218335-41218375	+	GGGGCCAAGCGGGGCGCGGGGCGGCTTCAGAGGCTGCGGGG<br>((((((.....)))))).	CTPS	NM_001905	chr1:41217594-41250822	non-template	41	Intron_1.f	--	Yes	--	Yes	--	--	--	--	Yes	Yes	Yes
G4ST01041256707	chr1:41256708-41256733	+	GGGCTGGGTGCTGGGACATGTGTGGG<br>((....))	SLFNL1	NM_001168247	chr1:41253856-41259984	template	26	Intron_3	--	Yes	--	Yes	--	--	--	--	--	Yes	--
G4ST01041256707	chr1:41256708-41256733	+	GGGCTGGGTGCTGGGACATGTGTGGG<br>((....))	SLFNL1	NM_144990	chr1:41253856-41260014	template	26	Intron_2	--	Yes	--	Yes	--	--	--	--	--	Yes	--
G4ST01041256707	chr1:41256708-41256733	+	GGGCTGGGTGCTGGGACATGTGTGGG<br>((....))	LOC100507178	NR_037868	chr1:41252849-41282149	non-template	26	Intron_1.f	--	Yes	--	Yes	--	--	--	--	--	Yes	--
G4ST01041258910	chr1:41258911-41258943	+	GGGGGTCATGGGAAGGCTCTCCCTGGGAAGGGG<br>.(((.....)))	SLFNL1	NM_001168247	chr1:41253856-41259984	template	33	Exon_3	--	Yes	Yes	--	Yes	Yes	--	--	Yes	Yes	--
G4ST01041258910	chr1:41258911-41258943	+	GGGGGTCATGGGAAGGCTCTCCCTGGGAAGGGG<br>.(((.....)))	SLFNL1	NM_144990	chr1:41253856-41260014	template	33	Exon_2	--	Yes	Yes	--	Yes	Yes	--	--	Yes	Yes	--
G4ST01041258910	chr1:41258911-41258943	+	GGGGGTCATGGGAAGGCTCTCCCTGGGAAGGGG<br>.(((.....)))	LOC100507178	NR_037868	chr1:41252849-41282149	non-template	33	Intron_1.f	--	Yes	--	Yes	--	--	--	--	Yes	Yes	--
G4ST01041259255	chr1:41259256-41259286	-	GGGTTGGGGAGGGAAGACAGGACCCCTGGGG<br>....((((...))))	SLFNL1	NM_001168247	chr1:41253856-41259984	non-template	31	Intron_1.f	--	Yes	--	Yes	--	--	--	Yes	Yes	Yes	--
G4ST01041259255	chr1:41259256-41259286	-	GGGTTGGGGAGGGAAGACAGGACCCCTGGGG<br>....((((...))))	SLFNL1	NM_144990	chr1:41253856-41260014	non-template	31	Intron_1.f	--	Yes	--	Yes	--	--	--	Yes	Yes	Yes	--

G4ST01041259255	chr1:41259256-41259286	-	GGGTTGGGGAGGGAAGACAGGACCCCTGGGG<br>....((((...))))	LOC100507178	NR_037868	chr1:41252849-41282149	template	31	Intron_1.f	--	Yes	--	Yes	--	--	--	Yes	Yes	Yes	--
G4ST01041348687	chr1:41348688-41348721	-	GGGGAGGGAAAATGGGAAATGACTGCTATTGGGG<br>.((((.....)))).	SCMH1	NM_001172222	chr1:41265458-41398192	non-template	34	Intron_2	--	Yes	--	Yes	--	--	--	--	Yes	--	--
G4ST01041348687	chr1:41348688-41348721	-	GGGGAGGGAAAATGGGAAATGACTGCTATTGGGG<br>.((((.....)))).	SCMH1	NM_001031694	chr1:41265458-41399691	non-template	34	Intron_8	--	Yes	--	Yes	--	--	--	--	Yes	--	--
G4ST01041348687	chr1:41348688-41348721	-	GGGGAGGGAAAATGGGAAATGACTGCTATTGGGG<br>.((((.....)))).	SCMH1	NM_001172221	chr1:41265458-41401276	non-template	34	Intron_7	--	Yes	--	Yes	--	--	--	--	Yes	--	--
G4ST01041348687	chr1:41348688-41348721	-	GGGGAGGGAAAATGGGAAATGACTGCTATTGGGG<br>.((((.....)))).	SCMH1	NM_001172218	chr1:41265458-41480402	non-template	34	Intron_9	--	Yes	--	Yes	--	--	--	--	Yes	--	--
G4ST01041348687	chr1:41348688-41348721	-	GGGGAGGGAAAATGGGAAATGACTGCTATTGGGG<br>.((((.....)))).	SCMH1	NM_001172219	chr1:41265458-41480402	non-template	34	Intron_8	--	Yes	--	Yes	--	--	--	--	Yes	--	--
G4ST01041348687	chr1:41348688-41348721	-	GGGGAGGGAAAATGGGAAATGACTGCTATTGGGG<br>.((((.....)))).	SCMH1	NM_001172220	chr1:41265458-41480402	non-template	34	Intron_7	--	Yes	--	Yes	--	--	--	--	Yes	--	--
G4ST01041348687	chr1:41348688-41348721	-	GGGGAGGGAAAATGGGAAATGACTGCTATTGGGG<br>.((((.....)))).	SCMH1	NM_012236	chr1:41265458-41480402	non-template	34	Intron_8	--	Yes	--	Yes	--	--	--	--	Yes	--	--
G4ST01041400455	chr1:41400456-41400494	-	GGGGGAGCAGGCATTTCTGATCCTGGGAGGGAAGAGGGG<br>(((.(((......))).))).	SCMH1	NM_001031694	chr1:41265458-41399691	non-template	-764	upstream	Yes	--	--	--	--	--	--	--	--	--	--
G4ST01041400455	chr1:41400456-41400494	-	GGGGGAGCAGGCATTTCTGATCCTGGGAGGGAAGAGGGG<br>(((.(((......))).))).	SCMH1	NM_001172221	chr1:41265458-41401276	non-template	39	Intron_1.f	--	Yes	--	Yes	--	--	--	--	--	--	--
G4ST01041400455	chr1:41400456-41400494	-	GGGGGAGCAGGCATTTCTGATCCTGGGAGGGAAGAGGGG<br>(((.(((......))).))).	SCMH1	NM_001172218	chr1:41265458-41480402	non-template	39	Intron_2	--	Yes	--	Yes	--	--	--	--	--	--	--
G4ST01041400455	chr1:41400456-41400494	-	GGGGGAGCAGGCATTTCTGATCCTGGGAGGGAAGAGGGG<br>(((.(((......))).))).	SCMH1	NM_001172219	chr1:41265458-41480402	non-template	39	Intron_2	--	Yes	--	Yes	--	--	--	--	--	--	--
G4ST01041400455	chr1:41400456-41400494	-	GGGGGAGCAGGCATTTCTGATCCTGGGAGGGAAGAGGGG<br>(((.(((......))).))).	SCMH1	NM_001172220	chr1:41265458-41480402	non-template	39	Intron_2	--	Yes	--	Yes	--	--	--	--	--	--	--
G4ST01041400455	chr1:41400456-41400494	-	GGGGGAGCAGGCATTTCTGATCCTGGGAGGGAAGAGGGG<br>(((.(((......))).))).	SCMH1	NM_012236	chr1:41265458-41480402	non-template	39	Intron_2	--	Yes	--	Yes	--	--	--	--	--	--	--
G4ST01041480381	chr1:41480382-41480409	+	GGGAGGGAGCGGAGACGCGGGAAGGGGG<br>.(((....)))	SCMH1	NM_001172218	chr1:41265458-41480402	template	21	upstream-Exon_1.f	Yes	Yes	Yes	--	--	Yes	--	Yes	Yes	Yes	Yes
G4ST01041480381	chr1:41480382-41480409	+	GGGAGGGAGCGGAGACGCGGGAAGGGGG<br>.(((....)))	SCMH1	NM_001172219	chr1:41265458-41480402	template	21	upstream-Exon_1.f	Yes	Yes	Yes	--	--	Yes	--	Yes	Yes	Yes	Yes
G4ST01041480381	chr1:41480382-41480409	+	GGGAGGGAGCGGAGACGCGGGAAGGGGG<br>.(((....)))	SCMH1	NM_001172220	chr1:41265458-41480402	template	21	upstream-Exon_1.f	Yes	Yes	Yes	--	--	Yes	--	Yes	Yes	Yes	Yes
G4ST01041480381	chr1:41480382-41480409	+	GGGAGGGAGCGGAGACGCGGGAAGGGGG<br>.(((....)))	SCMH1	NM_012236	chr1:41265458-41480402	template	21	upstream-Exon_1.f	Yes	Yes	Yes	--	--	Yes	--	Yes	Yes	Yes	Yes
G4ST01041480385	chr1:41480386-41480413	+	GGGAGCGGAGACGCGGGAAGGGGGCGGG<br>.(((....)))	SCMH1	NM_001172218	chr1:41265458-41480402	template	17	upstream-Exon_1.f	Yes	Yes	Yes	--	--	Yes	--	Yes	Yes	Yes	Yes
G4ST01041480385	chr1:41480386-41480413	+	GGGAGCGGAGACGCGGGAAGGGGGCGGG<br>.(((....)))	SCMH1	NM_001172219	chr1:41265458-41480402	template	17	upstream-Exon_1.f	Yes	Yes	Yes	--	--	Yes	--	Yes	Yes	Yes	Yes
G4ST01041480385	chr1:41480386-41480413	+	GGGAGCGGAGACGCGGGAAGGGGGCGGG<br>.(((....)))	SCMH1	NM_001172220	chr1:41265458-41480402	template	17	upstream-Exon_1.f	Yes	Yes	Yes	--	--	Yes	--	Yes	Yes	Yes	Yes
G4ST01041480385	chr1:41480386-41480413	+	GGGAGCGGAGACGCGGGAAGGGGGCGGG<br>.(((....)))	SCMH1	NM_012236	chr1:41265458-41480402	template	17	upstream-Exon_1.f	Yes	Yes	Yes	--	--	Yes	--	Yes	Yes	Yes	Yes
G4ST01041722983	chr1:41722984-41723012	-	GGGGCAGGGCTGGACCAGAAGGGGTGGGG<br>(((...)))...	EDN2	NM_001956	chr1:41717033-41722931	non-template	-52	upstream	Yes	--	--	--	--	--	--	Yes	Yes	Yes	--
G4ST01041764340	chr1:41764341-41764365	+	GGGACAGCGTGTCGGGGCGGGGGGG<br>(((...)))..	HIVEP3	NM_001127714	chr1:41748271-42157083	template	25	Intron_4	--	Yes	--	Yes	--	--	--	--	Yes	--	--
G4ST01041764340	chr1:41764341-41764365	+	GGGACAGCGTGTCGGGGCGGGGGGG<br>(((...)))..	HIVEP3	NM_024503	chr1:41748271-42157083	template	25	Intron_5	--	Yes	--	Yes	--	--	--	--	Yes	--	--
G4ST01041769229	chr1:41769230-41769259	+	GGGGAACACGAGGGAGGAGCCTGGGTTGGG<br>((....))	HIVEP3	NM_001127714	chr1:41748271-42157083	template	30	Intron_4	--	Yes	--	Yes	--	--	--	--	--	--	--
G4ST01041769229	chr1:41769230-41769259	+	GGGGAACACGAGGGAGGAGCCTGGGTTGGG<br>((....))	HIVEP3	NM_024503	chr1:41748271-42157083	template	30	Intron_5	--	Yes	--	Yes	--	--	--	--	--	--	--
G4ST01041771687	chr1:41771688-41771723	-	GGGACAGCAGAGCTGGCCCGGGATTTGGGCCCAGGG<br>.((((...))))....	HIVEP3	NM_001127714	chr1:41748271-42157083	non-template	36	Intron_4	--	Yes	--	Yes	--	--	--	--	--	--	Yes
G4ST01041771687	chr1:41771688-41771723	-	GGGACAGCAGAGCTGGCCCGGGATTTGGGCCCAGGG<br>.((((...))))....	HIVEP3	NM_024503	chr1:41748271-42157083	non-template	36	Intron_5	--	Yes	--	Yes	--	--	--	--	--	--	Yes
G4ST01041804632	chr1:41804633-41804673	-	GGGGCAGGGGGCAGGAGCAAAGCCCTGGGGACCTGCAGGGG<br>..((((.((...))))))	HIVEP3	NM_001127714	chr1:41748271-42157083	non-template	41	Intron_4	--	Yes	--	Yes	--	--	--	--	--	--	--
G4ST01041804632	chr1:41804633-41804673	-	GGGGCAGGGGGCAGGAGCAAAGCCCTGGGGACCTGCAGGGG<br>..((((.((...))))))	HIVEP3	NM_024503	chr1:41748271-42157083	non-template	41	Intron_5	--	Yes	--	Yes	--	--	--	--	--	--	--
G4ST01041810693	chr1:41810694-41810720	-	GGGCTTCCCGGAGTGGGTGGGGCCGGG<br>(((....))).	HIVEP3	NM_001127714	chr1:41748271-42157083	non-template	27	Intron_4	--	Yes	--	Yes	--	--	--	--	--	--	Yes
G4ST01041810693	chr1:41810694-41810720	-	GGGCTTCCCGGAGTGGGTGGGGCCGGG<br>(((....))).	HIVEP3	NM_024503	chr1:41748271-42157083	non-template	27	Intron_5	--	Yes	--	Yes	--	--	--	--	--	--	Yes
G4ST01041820346	chr1:41820347-41820397	+	GGGTGGCAGCTTCTCAGCTGGGAGCTGGGCAAGTGTCTGGCTGCGCTGGGG<br>....((((....)))) ..(((((......))))).	HIVEP3	NM_001127714	chr1:41748271-42157083	template	51	Exon_3	--	Yes	Yes	--	Yes	--	--	--	--	--	--
G4ST01041820346	chr1:41820347-41820397	+	GGGTGGCAGCTTCTCAGCTGGGAGCTGGGCAAGTGTCTGGCTGCGCTGGGG<br>....((((....)))) ..(((((......))))).	HIVEP3	NM_024503	chr1:41748271-42157083	template	51	Exon_4	--	Yes	Yes	--	Yes	--	--	--	--	--	--
G4ST01041821708	chr1:41821709-41821746	+	GGGCTTGTCTTCGGTGGACAGGGGCAGGAGGGGCTGGG<br>..(((((.....))))).	HIVEP3	NM_001127714	chr1:41748271-42157083	template	38	Exon_3	--	Yes	Yes	--	Yes	--	--	--	--	--	--
G4ST01041821708	chr1:41821709-41821746	+	GGGCTTGTCTTCGGTGGACAGGGGCAGGAGGGGCTGGG<br>..(((((.....))))).	HIVEP3	NM_024503	chr1:41748271-42157083	template	38	Exon_4	--	Yes	Yes	--	Yes	--	--	--	--	--	--
G4ST01041822904	chr1:41822905-41822945	+	GGGCGGGGCTCTCTTGGGTGGCAGCTGTGCCGCTGCCTGGG<br>.((((((......)))))).	HIVEP3	NM_001127714	chr1:41748271-42157083	template	41	Exon_3	--	Yes	Yes	--	Yes	--	--	--	--	--	--
G4ST01041822904	chr1:41822905-41822945	+	GGGCGGGGCTCTCTTGGGTGGCAGCTGTGCCGCTGCCTGGG<br>.((((((......)))))).	HIVEP3	NM_024503	chr1:41748271-42157083	template	41	Exon_4	--	Yes	Yes	--	Yes	--	--	--	--	--	--
G4ST01041822908	chr1:41822909-41822951	+	GGGGCTCTCTTGGGTGGCAGCTGTGCCGCTGCCTGGGTATGGG<br>.((((((......)))))).	HIVEP3	NM_001127714	chr1:41748271-42157083	template	43	Exon_3	--	Yes	Yes	--	Yes	--	--	--	--	--	--
G4ST01041822908	chr1:41822909-41822951	+	GGGGCTCTCTTGGGTGGCAGCTGTGCCGCTGCCTGGGTATGGG<br>.((((((......)))))).	HIVEP3	NM_024503	chr1:41748271-42157083	template	43	Exon_4	--	Yes	Yes	--	Yes	--	--	--	--	--	--
G4ST01041900271	chr1:41900272-41900300	-	GGGAGCCGCGTCGGTGGGAGAGGGCTGGG<br>..(((...))).	HIVEP3	NM_001127714	chr1:41748271-42157083	non-template	29	Intron_1.f	--	Yes	--	Yes	--	--	--	--	Yes	--	Yes
G4ST01041900271	chr1:41900272-41900300	-	GGGAGCCGCGTCGGTGGGAGAGGGCTGGG<br>..(((...))).	HIVEP3	NM_024503	chr1:41748271-42157083	non-template	29	Intron_2	--	Yes	--	Yes	--	--	--	--	Yes	--	Yes
G4ST01041900276	chr1:41900277-41900309	-	GGGACCGGAGGGAGCCGCGTCGGTGGGAGAGGG<br>..(((...))).	HIVEP3	NM_001127714	chr1:41748271-42157083	non-template	33	Intron_1.f	--	Yes	--	Yes	--	--	--	--	Yes	--	Yes
G4ST01041900276	chr1:41900277-41900309	-	GGGACCGGAGGGAGCCGCGTCGGTGGGAGAGGG<br>..(((...))).	HIVEP3	NM_024503	chr1:41748271-42157083	non-template	33	Intron_2	--	Yes	--	Yes	--	--	--	--	Yes	--	Yes
G4ST01041900282	chr1:41900283-41900319	-	GGGCGGCAGAGGGACCGGAGGGAGCCGCGTCGGTGGG<br>..(((...))).	HIVEP3	NM_001127714	chr1:41748271-42157083	non-template	37	Intron_1.f	--	Yes	--	Yes	--	--	--	--	Yes	--	Yes
G4ST01041900282	chr1:41900283-41900319	-	GGGCGGCAGAGGGACCGGAGGGAGCCGCGTCGGTGGG<br>..(((...))).	HIVEP3	NM_024503	chr1:41748271-42157083	non-template	37	Intron_2	--	Yes	--	Yes	--	--	--	--	Yes	--	Yes
G4ST01041900581	chr1:41900582-41900609	+	GGGCTGGCGGGCGGGCGCCGGCGGCGGG<br>.(((...)))	HIVEP3	NM_001127714	chr1:41748271-42157083	template	28	Intron_1.f	--	Yes	--	Yes	--	--	--	Yes	Yes	Yes	Yes
G4ST01041900581	chr1:41900582-41900609	+	GGGCTGGCGGGCGGGCGCCGGCGGCGGG<br>.(((...)))	HIVEP3	NM_024503	chr1:41748271-42157083	template	28	Intron_2	--	Yes	--	Yes	--	--	--	Yes	Yes	Yes	Yes
G4ST01041900829	chr1:41900830-41900864	+	GGGCCCGGGCGCGGCTGGCGGCTGCCAGGGGAGGG<br>.....(((((....)))))	HIVEP3	NM_001127714	chr1:41748271-42157083	template	35	Intron_1.f	--	Yes	--	Yes	--	--	--	Yes	Yes	Yes	--

G4ST01041900829	chr1:41900830-41900864	+	GGGCCCGGGCGCGGCTGGCGGCTGCCAGGGGAGGG<br>.....(((((....)))))	HIVEP3	NM_024503	chr1:41748271-42157083	template	35	Intron_2	--	Yes	--	Yes	--	--	--	Yes	Yes	Yes	--
G4ST01041909357	chr1:41909358-41909389	-	GGGAGGCTTAGGAGAGGCAGGGTGGGGAGGGG<br>..((((.....)))).	HIVEP3	NM_001127714	chr1:41748271-42157083	non-template	32	Intron_1.f	--	Yes	--	Yes	--	--	--	--	--	--	--
G4ST01041909357	chr1:41909358-41909389	-	GGGAGGCTTAGGAGAGGCAGGGTGGGGAGGGG<br>..((((.....)))).	HIVEP3	NM_024503	chr1:41748271-42157083	non-template	32	Intron_2	--	Yes	--	Yes	--	--	--	--	--	--	--
G4ST01041954810	chr1:41954811-41954855	+	GGGTGTGGCCACAAAGGGCTTCTTGTAGGAGGAGCAGTGGGAGGG<br>(((....))).. (((((....)))))......	HIVEP3	NM_001127714	chr1:41748271-42157083	template	45	Intron_1.f	--	Yes	--	Yes	--	--	--	--	--	--	--
G4ST01041954810	chr1:41954811-41954855	+	GGGTGTGGCCACAAAGGGCTTCTTGTAGGAGGAGCAGTGGGAGGG<br>(((....))).. (((((....)))))......	HIVEP3	NM_024503	chr1:41748271-42157083	template	45	Intron_1.f	--	Yes	--	Yes	--	--	--	--	--	--	--
G4ST01041956498	chr1:41956499-41956536	+	GGGCTTCTGGGGCAGTGAGGCGACTGTGGGCAATGGGG<br>.((((......)))).	HIVEP3	NM_001127714	chr1:41748271-42157083	template	38	Intron_1.f	--	Yes	--	Yes	--	--	--	--	--	--	--
G4ST01041956498	chr1:41956499-41956536	+	GGGCTTCTGGGGCAGTGAGGCGACTGTGGGCAATGGGG<br>.((((......)))).	HIVEP3	NM_024503	chr1:41748271-42157083	template	38	Intron_1.f	--	Yes	--	Yes	--	--	--	--	--	--	--
G4ST01041959279	chr1:41959280-41959311	+	GGGTGCCAGGAGCTGGGGGAACGGGAAATGGG<br>..((((...))))	HIVEP3	NM_001127714	chr1:41748271-42157083	template	32	Intron_1.f	--	Yes	--	Yes	--	--	--	Yes	Yes	--	--
G4ST01041959279	chr1:41959280-41959311	+	GGGTGCCAGGAGCTGGGGGAACGGGAAATGGG<br>..((((...))))	HIVEP3	NM_024503	chr1:41748271-42157083	template	32	Intron_1.f	--	Yes	--	Yes	--	--	--	Yes	Yes	--	--
G4ST01041966674	chr1:41966675-41966706	+	GGGGCACTCAGTGCTGGGCAGGGGATGCGGGG<br>((((...)))).	HIVEP3	NM_001127714	chr1:41748271-42157083	template	32	Intron_1.f	--	Yes	--	Yes	--	--	--	--	Yes	Yes	--
G4ST01041966674	chr1:41966675-41966706	+	GGGGCACTCAGTGCTGGGCAGGGGATGCGGGG<br>((((...)))).	HIVEP3	NM_024503	chr1:41748271-42157083	template	32	Intron_1.f	--	Yes	--	Yes	--	--	--	--	Yes	Yes	--
G4ST01041972839	chr1:41972840-41972876	+	GGGCTGCCATGGCGGAGGGCAGTCTGGGAAGGAGGGG<br>((((....)))).	HIVEP3	NM_001127714	chr1:41748271-42157083	template	37	Intron_1.f	--	Yes	--	Yes	--	--	--	--	--	--	--
G4ST01041972839	chr1:41972840-41972876	+	GGGCTGCCATGGCGGAGGGCAGTCTGGGAAGGAGGGG<br>((((....)))).	HIVEP3	NM_024503	chr1:41748271-42157083	template	37	Intron_1.f	--	Yes	--	Yes	--	--	--	--	--	--	--
G4ST01041985467	chr1:41985468-41985498	+	GGGCAGTGAGGGTGAGGGGTGGTACGCTGGG<br>(((...))).	HIVEP3	NM_001127714	chr1:41748271-42157083	template	31	Intron_1.f	--	Yes	--	Yes	--	--	--	--	--	--	--
G4ST01041985467	chr1:41985468-41985498	+	GGGCAGTGAGGGTGAGGGGTGGTACGCTGGG<br>(((...))).	HIVEP3	NM_024503	chr1:41748271-42157083	template	31	Intron_1.f	--	Yes	--	Yes	--	--	--	--	--	--	--
G4ST01042001730	chr1:42001731-42001766	-	GGGGACATGAAGTCAGGGCTGTTCCCAGGGGTGGGG<br>(((.....))). (((....)))	HIVEP3	NM_001127714	chr1:41748271-42157083	non-template	36	Intron_1.f	--	Yes	--	Yes	--	--	--	--	--	--	--
G4ST01042001730	chr1:42001731-42001766	-	GGGGACATGAAGTCAGGGCTGTTCCCAGGGGTGGGG<br>(((.....))). (((....)))	HIVEP3	NM_024503	chr1:41748271-42157083	non-template	36	Intron_1.f	--	Yes	--	Yes	--	--	--	--	--	--	--
G4ST01042022357	chr1:42022358-42022399	+	GGGAGGCAGCTGTGCTTCCAGGGAGAGCAGGGTTCCTGGGGG<br>.((.(((...))).)).	HIVEP3	NM_001127714	chr1:41748271-42157083	template	42	Intron_1.f	--	Yes	--	Yes	--	--	--	--	--	--	Yes
G4ST01042022357	chr1:42022358-42022399	+	GGGAGGCAGCTGTGCTTCCAGGGAGAGCAGGGTTCCTGGGGG<br>.((.(((...))).)).	HIVEP3	NM_024503	chr1:41748271-42157083	template	42	Intron_1.f	--	Yes	--	Yes	--	--	--	--	--	--	Yes
G4ST01042039525	chr1:42039526-42039555	-	GGGAGGAATTACCTGGGGCAAGGGAGGGGG<br>(((.....))).	HIVEP3	NM_001127714	chr1:41748271-42157083	non-template	30	Intron_1.f	--	Yes	--	Yes	--	--	--	--	Yes	--	--
G4ST01042039525	chr1:42039526-42039555	-	GGGAGGAATTACCTGGGGCAAGGGAGGGGG<br>(((.....))).	HIVEP3	NM_024503	chr1:41748271-42157083	non-template	30	Intron_1.f	--	Yes	--	Yes	--	--	--	--	Yes	--	--
G4ST01042061915	chr1:42061916-42061952	+	GGGGAGGGGCAGGGTGCAGAGAGGAATTCTGCCTGGG<br>.((((((.....))))))..	HIVEP3	NM_001127714	chr1:41748271-42157083	template	37	Intron_1.f	--	Yes	--	Yes	--	--	--	Yes	Yes	--	--
G4ST01042061915	chr1:42061916-42061952	+	GGGGAGGGGCAGGGTGCAGAGAGGAATTCTGCCTGGG<br>.((((((.....))))))..	HIVEP3	NM_024503	chr1:41748271-42157083	template	37	Intron_1.f	--	Yes	--	Yes	--	--	--	Yes	Yes	--	--
G4ST01042079416	chr1:42079417-42079446	-	GGGCAGGTGTGCTGGGGGCACTGGGATGGG<br>(((.....))).	HIVEP3	NM_001127714	chr1:41748271-42157083	non-template	30	Intron_1.f	--	Yes	--	Yes	--	--	--	Yes	Yes	--	--
G4ST01042079416	chr1:42079417-42079446	-	GGGCAGGTGTGCTGGGGGCACTGGGATGGG<br>(((.....))).	HIVEP3	NM_024503	chr1:41748271-42157083	non-template	30	Intron_1.f	--	Yes	--	Yes	--	--	--	Yes	Yes	--	--
G4ST01042094915	chr1:42094916-42094948	+	GGGAGGAAGGGAGGGACAATTTAGCAAATTGGG<br>..((((.....))))	HIVEP3	NM_001127714	chr1:41748271-42157083	template	33	Intron_1.f	--	Yes	--	Yes	--	--	--	--	--	--	--
G4ST01042094915	chr1:42094916-42094948	+	GGGAGGAAGGGAGGGACAATTTAGCAAATTGGG<br>..((((.....))))	HIVEP3	NM_024503	chr1:41748271-42157083	template	33	Intron_1.f	--	Yes	--	Yes	--	--	--	--	--	--	--
G4ST01042098172	chr1:42098173-42098211	+	GGGAAGGGTTGAGCAGGGCCAGGTGGTTGCCCATCAGGG<br>...(((((....))))).	HIVEP3	NM_001127714	chr1:41748271-42157083	template	39	Intron_1.f	--	Yes	--	Yes	--	--	--	Yes	Yes	--	--
G4ST01042098172	chr1:42098173-42098211	+	GGGAAGGGTTGAGCAGGGCCAGGTGGTTGCCCATCAGGG<br>...(((((....))))).	HIVEP3	NM_024503	chr1:41748271-42157083	template	39	Intron_1.f	--	Yes	--	Yes	--	--	--	Yes	Yes	--	--
G4ST01042098177	chr1:42098178-42098218	+	GGGTTGAGCAGGGCCAGGTGGTTGCCCATCAGGGAACTGGG<br>...(((((....))))).	HIVEP3	NM_001127714	chr1:41748271-42157083	template	41	Intron_1.f	--	Yes	--	Yes	--	--	--	Yes	Yes	--	--
G4ST01042098177	chr1:42098178-42098218	+	GGGTTGAGCAGGGCCAGGTGGTTGCCCATCAGGGAACTGGG<br>...(((((....))))).	HIVEP3	NM_024503	chr1:41748271-42157083	template	41	Intron_1.f	--	Yes	--	Yes	--	--	--	Yes	Yes	--	--
G4ST01042106180	chr1:42106181-42106209	-	GGGAGCTTTTGGAAGGGGAGGGGGGAGGG<br>..(((....)))	HIVEP3	NM_001127714	chr1:41748271-42157083	non-template	29	Intron_1.f	--	Yes	--	Yes	--	--	--	--	--	--	--
G4ST01042106180	chr1:42106181-42106209	-	GGGAGCTTTTGGAAGGGGAGGGGGGAGGG<br>..(((....)))	HIVEP3	NM_024503	chr1:41748271-42157083	non-template	29	Intron_1.f	--	Yes	--	Yes	--	--	--	--	--	--	--
G4ST01042121469	chr1:42121470-42121506	+	GGGGTGGGACTGGCAGGAGCTGCTGGGAAGGCTGGGG<br>....((((...)))).	HIVEP3	NM_001127714	chr1:41748271-42157083	template	37	Intron_1.f	--	Yes	--	Yes	--	--	--	--	--	--	--
G4ST01042121469	chr1:42121470-42121506	+	GGGGTGGGACTGGCAGGAGCTGCTGGGAAGGCTGGGG<br>....((((...)))).	HIVEP3	NM_024503	chr1:41748271-42157083	template	37	Intron_1.f	--	Yes	--	Yes	--	--	--	--	--	--	--
G4ST01042129526	chr1:42129527-42129557	-	GGGAAATAAGTTTGGGGCTTTTGGGGTTGGG<br>(((....))).	HIVEP3	NM_001127714	chr1:41748271-42157083	non-template	31	Intron_1.f	--	Yes	--	Yes	--	--	--	--	Yes	--	--
G4ST01042129526	chr1:42129527-42129557	-	GGGAAATAAGTTTGGGGCTTTTGGGGTTGGG<br>(((....))).	HIVEP3	NM_024503	chr1:41748271-42157083	non-template	31	Intron_1.f	--	Yes	--	Yes	--	--	--	--	Yes	--	--
G4ST01042402843	chr1:42402844-42402871	+	GGGCACTAGGGGCAGGGAGCCAGCTGGG<br>((....))	GUCA2A	NM_033553	chr1:42400949-42402982	template	28	Intron_1.f	--	Yes	--	Yes	--	--	--	--	--	--	--
G4ST01042402851	chr1:42402852-42402877	+	GGGGCAGGGAGCCAGCTGGGCCGGGG<br>((....))	GUCA2A	NM_033553	chr1:42400949-42402982	template	26	Intron_1.f	--	Yes	--	Yes	--	--	--	--	--	--	--
G4ST01042402857	chr1:42402858-42402882	+	GGGAGCCAGCTGGGCCGGGGTAGGG<br>((....))	GUCA2A	NM_033553	chr1:42400949-42402982	template	25	Intron_1.f	--	Yes	--	Yes	--	--	--	--	--	--	--
G4ST01042403952	chr1:42403953-42403985	-	GGGGACTGTATCTGCAGGGGCAAGGGGGGCGGG<br>..((((....))))	GUCA2A	NM_033553	chr1:42400949-42402982	non-template	-970	upstream	Yes	--	--	--	--	--	--	Yes	Yes	--	Yes
G4ST01042404187	chr1:42404188-42404216	+	GGGCGGGAGGTTGGGCAGGAATTCCTGGG<br>.(((....)))	GUCA2A	NM_033553	chr1:42400949-42402982	template	-1205	upstream	Yes	--	--	--	--	--	--	Yes	Yes	--	--
G4ST01042404191	chr1:42404192-42404221	+	GGGAGGTTGGGCAGGAATTCCTGGGTGGGG<br>.(((....)))	GUCA2A	NM_033553	chr1:42400949-42402982	template	-1209	upstream	Yes	--	--	--	--	--	--	Yes	Yes	--	--
G4ST01042449188	chr1:42449189-42449221	+	GGGGGTGGGGCGGGGAGTGTACATCATTAAGGG<br>.((((.....))))..	FOXJ3	NM_001198851	chr1:42414797-42573223	template	33	Intron_7	--	Yes	--	Yes	--	--	--	--	Yes	--	Yes
G4ST01042449188	chr1:42449189-42449221	+	GGGGGTGGGGCGGGGAGTGTACATCATTAAGGG<br>.((((.....))))..	FOXJ3	NM_001198852	chr1:42414797-42573473	template	33	Intron_5	--	Yes	--	Yes	--	--	--	--	Yes	--	Yes
G4ST01042449188	chr1:42449189-42449221	+	GGGGGTGGGGCGGGGAGTGTACATCATTAAGGG<br>.((((.....))))..	FOXJ3	NM_014947	chr1:42414797-42573490	template	33	Intron_5	--	Yes	--	Yes	--	--	--	--	Yes	--	Yes
G4ST01042449188	chr1:42449189-42449221	+	GGGGGTGGGGCGGGGAGTGTACATCATTAAGGG<br>.((((.....))))..	FOXJ3	NM_001198850	chr1:42414797-42574135	template	33	Intron_5	--	Yes	--	Yes	--	--	--	--	Yes	--	Yes
G4ST01042500010	chr1:42500011-42500043	-	GGGGTGGGAGTAGATTTTGGTCTTGGGTATGGG<br>...((((....)))).	FOXJ3	NM_001198851	chr1:42414797-42573223	non-template	33	Intron_6	--	Yes	--	Yes	--	--	--	--	--	--	--
G4ST01042500010	chr1:42500011-42500043	-	GGGGTGGGAGTAGATTTTGGTCTTGGGTATGGG<br>...((((....)))).	FOXJ3	NM_001198852	chr1:42414797-42573473	non-template	33	Intron_4	--	Yes	--	Yes	--	--	--	--	--	--	--
G4ST01042500010	chr1:42500011-42500043	-	GGGGTGGGAGTAGATTTTGGTCTTGGGTATGGG<br>...((((....)))).	FOXJ3	NM_014947	chr1:42414797-42573490	non-template	33	Intron_4	--	Yes	--	Yes	--	--	--	--	--	--	--

G4ST01042500010	chr1:42500011-42500043	-	GGGGTGGGAGTAGATTTTGGTCTTGGGTATGGG<br>...((((....)))).	FOXJ3	NM_001198850	chr1:42414797-42574135	non-template	33	Intron_4	--	Yes	--	Yes	--	--	--	--	--	--	--
G4ST01042530269	chr1:42530270-42530302	+	GGGAGCCAGGGGATGGGACCCTGAGGAGGGGGG<br>.((((....))))	FOXJ3	NM_001198851	chr1:42414797-42573223	template	33	Intron_4	--	Yes	--	Yes	--	--	--	--	--	--	--
G4ST01042530269	chr1:42530270-42530302	+	GGGAGCCAGGGGATGGGACCCTGAGGAGGGGGG<br>.((((....))))	FOXJ3	NM_001198852	chr1:42414797-42573473	template	33	Intron_2	--	Yes	--	Yes	--	--	--	--	--	--	--
G4ST01042530269	chr1:42530270-42530302	+	GGGAGCCAGGGGATGGGACCCTGAGGAGGGGGG<br>.((((....))))	FOXJ3	NM_014947	chr1:42414797-42573490	template	33	Intron_2	--	Yes	--	Yes	--	--	--	--	--	--	--
G4ST01042530269	chr1:42530270-42530302	+	GGGAGCCAGGGGATGGGACCCTGAGGAGGGGGG<br>.((((....))))	FOXJ3	NM_001198850	chr1:42414797-42574135	template	33	Intron_2	--	Yes	--	Yes	--	--	--	--	--	--	--
G4ST01042551205	chr1:42551206-42551241	+	GGGAGGGGATCAGGGACTGTGACAGCAGTGCAAGGG<br>(((((....)))))....	FOXJ3	NM_001198851	chr1:42414797-42573223	template	36	Intron_3	--	Yes	--	Yes	--	--	--	Yes	Yes	--	--
G4ST01042551205	chr1:42551206-42551241	+	GGGAGGGGATCAGGGACTGTGACAGCAGTGCAAGGG<br>(((((....)))))....	FOXJ3	NM_001198852	chr1:42414797-42573473	template	36	Intron_1.f	--	Yes	--	Yes	--	--	--	Yes	Yes	--	--
G4ST01042551205	chr1:42551206-42551241	+	GGGAGGGGATCAGGGACTGTGACAGCAGTGCAAGGG<br>(((((....)))))....	FOXJ3	NM_014947	chr1:42414797-42573490	template	36	Intron_1.f	--	Yes	--	Yes	--	--	--	Yes	Yes	--	--
G4ST01042551205	chr1:42551206-42551241	+	GGGAGGGGATCAGGGACTGTGACAGCAGTGCAAGGG<br>(((((....)))))....	FOXJ3	NM_001198850	chr1:42414797-42574135	template	36	Intron_1.f	--	Yes	--	Yes	--	--	--	Yes	Yes	--	--
G4ST01042573340	chr1:42573341-42573382	-	GGGGGGGCGGAGACGCACGGAGCGGCCGGGGGCTCGGCCGGG<br>(((...(((.....))).))).	FOXJ3	NM_001198851	chr1:42414797-42573223	non-template	-117	upstream	Yes	--	--	--	--	--	--	Yes	Yes	Yes	--
G4ST01042573340	chr1:42573341-42573382	-	GGGGGGGCGGAGACGCACGGAGCGGCCGGGGGCTCGGCCGGG<br>(((...(((.....))).))).	FOXJ3	NM_001198852	chr1:42414797-42573473	non-template	42	Exon_1.f	--	Yes	Yes	--	--	Yes	--	Yes	Yes	Yes	--
G4ST01042573340	chr1:42573341-42573382	-	GGGGGGGCGGAGACGCACGGAGCGGCCGGGGGCTCGGCCGGG<br>(((...(((.....))).))).	FOXJ3	NM_014947	chr1:42414797-42573490	non-template	42	Exon_1.f	--	Yes	Yes	--	--	Yes	--	Yes	Yes	Yes	--
G4ST01042573340	chr1:42573341-42573382	-	GGGGGGGCGGAGACGCACGGAGCGGCCGGGGGCTCGGCCGGG<br>(((...(((.....))).))).	FOXJ3	NM_001198850	chr1:42414797-42574135	non-template	42	Intron_1.f	--	Yes	--	Yes	--	--	--	Yes	Yes	Yes	--
G4ST01042573820	chr1:42573821-42573859	-	GGGGGAGGGCAGAAGGCACCATTCCTTCGGGGCATGGGG<br>..(((((.......))))).	FOXJ3	NM_001198851	chr1:42414797-42573223	non-template	-597	upstream	Yes	--	--	--	--	--	--	Yes	Yes	Yes	--
G4ST01042573820	chr1:42573821-42573859	-	GGGGGAGGGCAGAAGGCACCATTCCTTCGGGGCATGGGG<br>..(((((.......))))).	FOXJ3	NM_001198852	chr1:42414797-42573473	non-template	-347	upstream	Yes	--	--	--	--	--	--	Yes	Yes	Yes	--
G4ST01042573820	chr1:42573821-42573859	-	GGGGGAGGGCAGAAGGCACCATTCCTTCGGGGCATGGGG<br>..(((((.......))))).	FOXJ3	NM_014947	chr1:42414797-42573490	non-template	-330	upstream	Yes	--	--	--	--	--	--	Yes	Yes	Yes	--
G4ST01042573820	chr1:42573821-42573859	-	GGGGGAGGGCAGAAGGCACCATTCCTTCGGGGCATGGGG<br>..(((((.......))))).	FOXJ3	NM_001198850	chr1:42414797-42574135	non-template	39	Intron_1.f	--	Yes	--	Yes	--	--	--	Yes	Yes	Yes	--
G4ST01042573827	chr1:42573828-42573866	-	GGGGGGCGGGGGAGGGCAGAAGGCACCATTCCTTCGGGG<br>..(((((.......))))).	FOXJ3	NM_001198851	chr1:42414797-42573223	non-template	-604	upstream	Yes	--	--	--	--	--	--	Yes	Yes	Yes	--
G4ST01042573827	chr1:42573828-42573866	-	GGGGGGCGGGGGAGGGCAGAAGGCACCATTCCTTCGGGG<br>..(((((.......))))).	FOXJ3	NM_001198852	chr1:42414797-42573473	non-template	-354	upstream	Yes	--	--	--	--	--	--	Yes	Yes	Yes	--
G4ST01042573827	chr1:42573828-42573866	-	GGGGGGCGGGGGAGGGCAGAAGGCACCATTCCTTCGGGG<br>..(((((.......))))).	FOXJ3	NM_014947	chr1:42414797-42573490	non-template	-337	upstream	Yes	--	--	--	--	--	--	Yes	Yes	Yes	--
G4ST01042573827	chr1:42573828-42573866	-	GGGGGGCGGGGGAGGGCAGAAGGCACCATTCCTTCGGGG<br>..(((((.......))))).	FOXJ3	NM_001198850	chr1:42414797-42574135	non-template	39	Intron_1.f	--	Yes	--	Yes	--	--	--	Yes	Yes	Yes	--
G4ST01042623130	chr1:42623131-42623162	-	GGGAGGGAACAGTGGGCATCTGAGGCAGAGGG<br>..((((...))))	RIMKLA	NM_173642	chr1:42619055-42662487	template	32	Intron_1.f	--	Yes	--	Yes	--	--	--	--	--	--	--
G4ST01042891145	chr1:42891146-42891185	+	GGGGAGTAGACTGCTCCTGGGTATGGGCTTTTTTGGGGGG<br>((((.....))))..	CCDC30	NM_001080850	chr1:42773147-42892922	non-template	40	Intron_14	--	Yes	--	Yes	--	--	--	--	--	--	--
G4ST01042921180	chr1:42921181-42921218	-	GGGGCGCGCGGCCGCGCGGGGTCCACAGGGGGCGCGGG<br>.(((((....)))))	YBX1	NM_004559	chr1:42920653-42940607	template	38	Intron_1.f	--	Yes	--	Yes	--	--	--	Yes	Yes	Yes	--
G4ST01042921186	chr1:42921187-42921224	-	GGGGGAGGGGCGCGCGGCCGCGCGGGGTCCACAGGGGG<br>.(((((....)))))	YBX1	NM_004559	chr1:42920653-42940607	template	38	Intron_1.f	--	Yes	--	Yes	--	--	--	Yes	Yes	Yes	--
G4ST01042972334	chr1:42972335-42972365	+	GGGAGACTGAGTCCCGGGAGAGGGTTTGGGG<br>.(((...)))..	CLDN19	NM_001123395	chr1:42971351-42978512	template	31	Exon_4.l	--	Yes	Yes	--	--	--	Yes	--	Yes	--	Yes
G4ST01042972334	chr1:42972335-42972365	+	GGGAGACTGAGTCCCGGGAGAGGGTTTGGGG<br>.(((...)))..	CLDN19	NM_001185117	chr1:42971351-42978512	template	31	Exon_3.l	--	Yes	Yes	--	--	--	Yes	--	Yes	--	Yes
G4ST01042972334	chr1:42972335-42972365	+	GGGAGACTGAGTCCCGGGAGAGGGTTTGGGG<br>.(((...)))..	CLDN19	NM_148960	chr1:42971351-42978512	template	31	Exon_5.l	--	Yes	Yes	--	--	--	Yes	--	Yes	--	Yes
G4ST01042977222	chr1:42977223-42977263	-	GGGGAGGGGTTGCTGCGGGGCTCAGGCTGAGTTGAGCTGGG<br>(((((.........))))).	CLDN19	NM_001123395	chr1:42971351-42978512	non-template	41	Intron_1.f	--	Yes	--	Yes	--	--	--	--	--	--	--
G4ST01042977222	chr1:42977223-42977263	-	GGGGAGGGGTTGCTGCGGGGCTCAGGCTGAGTTGAGCTGGG<br>(((((.........))))).	CLDN19	NM_001185117	chr1:42971351-42978512	non-template	41	Intron_1.f	--	Yes	--	Yes	--	--	--	--	--	--	--
G4ST01042977222	chr1:42977223-42977263	-	GGGGAGGGGTTGCTGCGGGGCTCAGGCTGAGTTGAGCTGGG<br>(((((.........))))).	CLDN19	NM_148960	chr1:42971351-42978512	non-template	41	Intron_1.f	--	Yes	--	Yes	--	--	--	--	--	--	--
G4ST01043063207	chr1:43063208-43063234	+	GGGTGGGCTGAGGGGACATGGTGTGGG<br>.(((...)))	ERMAP	NM_001017922	chr1:43055363-43083247	non-template	27	Intron_1.f	--	Yes	--	Yes	--	--	--	Yes	Yes	Yes	--
G4ST01043063207	chr1:43063208-43063234	+	GGGTGGGCTGAGGGGACATGGTGTGGG<br>.(((...)))	ERMAP	NM_018538	chr1:43063836-43083247	non-template	-601	upstream	Yes	--	--	--	--	--	--	Yes	Yes	Yes	--
G4ST01043084917	chr1:43084918-43084948	+	GGGTCCGGGTTGGGCCCGAGGTCGCGGGGGG<br>((((......))))	ZNF691	NM_015911	chr1:43084867-43090733	non-template	31	Intron_1.u	--	Yes	--	Yes	--	--	--	Yes	Yes	Yes	--
G4ST01043085081	chr1:43085082-43085119	-	GGGGACAGCCACCTGGCTGCGTGGGCCGGGGGCCAGGG<br>..(((((....)))))...	ZNF691	NM_015911	chr1:43084867-43090733	template	38	Intron_1.u	--	Yes	--	Yes	--	--	--	Yes	Yes	Yes	Yes
G4ST01043085087	chr1:43085088-43085124	-	GGGTTGGGGACAGCCACCTGGCTGCGTGGGCCGGGGG<br>..(((((....)))))...	ZNF691	NM_015911	chr1:43084867-43090733	template	37	Intron_1.u	--	Yes	--	Yes	--	--	--	Yes	Yes	Yes	Yes
G4ST01043085094	chr1:43085095-43085131	-	GGGGAATGGGTTGGGGACAGCCACCTGGCTGCGTGGG<br>..(((((....)))))...	ZNF691	NM_015911	chr1:43084867-43090733	template	37	Intron_1.u	--	Yes	--	Yes	--	--	--	Yes	Yes	Yes	Yes
G4ST01043164688	chr1:43164689-43164723	-	GGGAGCACTGGAGTGCAGGGAGGAGAGGGGAAGGG<br>.((((....)))).	SLC2A1	NM_006516	chr1:43163633-43197434	non-template	35	Exon_10.l	--	Yes	Yes	--	--	--	Yes	--	Yes	Yes	Yes
G4ST01043170166	chr1:43170167-43170204	-	GGGGGGAGGGCCTGATGGCAGGAGATGGGCCCCTGGGG<br>((((....))))....	SLC2A1	NM_006516	chr1:43163633-43197434	non-template	38	Intron_2	--	Yes	--	Yes	--	--	--	Yes	Yes	Yes	--
G4ST01043170175	chr1:43170176-43170210	-	GGGGTTGGGGGGAGGGCCTGATGGCAGGAGATGGG<br>((((....))))....	SLC2A1	NM_006516	chr1:43163633-43197434	non-template	35	Intron_2	--	Yes	--	Yes	--	--	--	Yes	Yes	Yes	--
G4ST01043197243	chr1:43197244-43197274	+	GGGCGGCGCTCTGCGGGGAGGGGGTGAGGGG<br>...(((...)))	FLJ32224	NR_033967	chr1:43197307-43221616	non-template	-32	upstream	Yes	--	--	--	--	--	--	Yes	Yes	Yes	Yes
G4ST01043197243	chr1:43197244-43197274	+	GGGCGGCGCTCTGCGGGGAGGGGGTGAGGGG<br>...(((...)))	SLC2A1	NM_006516	chr1:43163633-43197434	template	31	Exon_1.f	--	Yes	Yes	--	--	Yes	--	Yes	Yes	Yes	Yes
G4ST01043386378	chr1:43386379-43386411	+	GGGAGGGCGCCGGGGAAGGAGGAGCCCTCCGGG<br>...(((((...)))))	FAM183A	NM_001101376	chr1:43386181-43394654	non-template	33	Intron_1.f	--	Yes	--	Yes	--	--	--	--	--	--	Yes
G4ST01043386382	chr1:43386383-43386419	+	GGGCGCCGGGGAAGGAGGAGCCCTCCGGGTTGCGGGG<br>...(((((...)))))	FAM183A	NM_001101376	chr1:43386181-43394654	non-template	37	Intron_1.f	--	Yes	--	Yes	--	--	--	--	--	--	Yes
G4ST01043386389	chr1:43386390-43386423	+	GGGGAAGGAGGAGCCCTCCGGGTTGCGGGGTGGG<br>...(((((...)))))	FAM183A	NM_001101376	chr1:43386181-43394654	non-template	34	Intron_1.f	--	Yes	--	Yes	--	--	--	--	--	--	Yes
G4ST01043522584	chr1:43522585-43522619	+	GGGGAGTCCTTGGGGGCAGAGTAGCCGGGCTAGGG<br>(((......)))	C1orf210	NM_182517	chr1:43520144-43523837	template	35	Intron_1.f	--	Yes	--	Yes	--	--	--	--	--	--	--
G4ST01043522584	chr1:43522585-43522619	+	GGGGAGTCCTTGGGGGCAGAGTAGCCGGGCTAGGG<br>(((......)))	C1orf210	NM_001164829	chr1:43520144-43523875	template	35	Intron_1.f	--	Yes	--	Yes	--	--	--	--	--	--	--
G4ST01043524501	chr1:43524502-43524536	-	GGGCATGGAAGAGTGGGGACTTGCAGGGTAGAGGG<br>(((......)))	C1orf210	NM_182517	chr1:43520144-43523837	non-template	-664	upstream	Yes	--	--	--	--	--	--	--	--	--	--
G4ST01043524501	chr1:43524502-43524536	-	GGGCATGGAAGAGTGGGGACTTGCAGGGTAGAGGG<br>(((......)))	C1orf210	NM_001164829	chr1:43520144-43523875	non-template	-626	upstream	Yes	--	--	--	--	--	--	--	--	--	--
G4ST01043524508	chr1:43524509-43524545	-	GGGTGCTCTGGGCATGGAAGAGTGGGGACTTGCAGGG<br>(((......)))	C1orf210	NM_182517	chr1:43520144-43523837	non-template	-671	upstream	Yes	--	--	--	--	--	--	--	--	--	--

G4ST01043524508	chr1:43524509-43524545	-	GGGTGCTCTGGGCATGGAAGAGTGGGGACTTGCAGGG<br>(((......)))	C1orf210	NM_001164829	chr1:43520144-43523875	non-template	-633	upstream	Yes	--	--	--	--	--	--	--	--	--	--
G4ST01043538274	chr1:43538275-43538305	+	GGGTGCCAGGGCAGGGAGAGGTGAGTCTGGG<br>(((......)))	TIE1	NM_005424	chr1:43539251-43561366	non-template	-945	upstream	Yes	--	--	--	--	--	--	--	--	--	--
G4ST01043538282	chr1:43538283-43538309	+	GGGCAGGGAGAGGTGAGTCTGGGAGGG<br>(((......)))	TIE1	NM_005424	chr1:43539251-43561366	non-template	-941	upstream	Yes	--	--	--	--	--	--	--	--	--	--
G4ST01043587319	chr1:43587320-43587350	+	GGGTGCGAGTGGGGCGGGGCTCGGAGAGGGG<br>.(((...)))	MPL	NM_005373	chr1:43576062-43592722	non-template	31	Intron_9	--	Yes	--	Yes	--	--	--	Yes	Yes	Yes	Yes
G4ST01043587329	chr1:43587330-43587362	+	GGGGCGGGGCTCGGAGAGGGGCGAGGCGCGGGG<br>.(((...))) (((...))).	MPL	NM_005373	chr1:43576062-43592722	non-template	33	Intron_9	--	Yes	--	Yes	--	--	--	Yes	Yes	Yes	Yes
G4ST01043602765	chr1:43602766-43602801	-	GGGTGAGTGGTGAAGGCCACTATGGGAAAGGGTGGG<br>..((((((....))))))..	ELOVL1	NM_022821	chr1:43601659-43606286	non-template	36	Exon_6-Intron_6	--	Yes	Yes	Yes	Yes	--	--	--	--	Yes	--
G4ST01043602769	chr1:43602770-43602820	-	GGGGGGTAAAGATTGCCCCGGGTGAGTGGTGAAGGCCACTATGGGAAAGGG<br>(((((.....))))). ..((((((....))))))..	ELOVL1	NM_022821	chr1:43601659-43606286	non-template	51	Exon_6-Intron_6	--	Yes	Yes	Yes	Yes	--	--	--	--	Yes	--
G4ST01043604690	chr1:43604691-43604724	+	GGGTGGGTCCGGGCCACAGGTCAGTGCCTGGGGG<br>...(((((....))))).	ELOVL1	NM_022821	chr1:43601659-43606286	template	34	Intron_1.f	--	Yes	--	Yes	--	--	--	Yes	Yes	Yes	Yes
G4ST01043605286	chr1:43605287-43605321	-	GGGCCTCCTGGAGGTGAGGGGTGTGCGGGGCAGGG<br>((((...))))....	ELOVL1	NM_022821	chr1:43601659-43606286	non-template	35	Intron_1.f	--	Yes	--	Yes	--	--	--	Yes	Yes	Yes	--
G4ST01043606177	chr1:43606178-43606219	+	GGGGCCAGGCGGGCAACGGCGGAGCGCTCGGAGGGAGCAGGG<br>...(((((...))).))..	ELOVL1	NM_022821	chr1:43601659-43606286	template	42	Exon_1.f	--	Yes	Yes	--	--	Yes	--	Yes	Yes	Yes	--
G4ST01043606374	chr1:43606375-43606402	+	GGGGCGGGGGCAGGGGCGTGCCGCCGGG<br>(((...))).	ELOVL1	NM_022821	chr1:43601659-43606286	template	-88	upstream	Yes	--	--	--	--	--	--	Yes	Yes	Yes	--
G4ST01043670691	chr1:43670692-43670732	+	GGGGAGGTGAGTCTCACCTGGGAATGGAGGGGGGTGGTGGG<br>.((((((...))))))	KIAA0467	NM_015284	chr1:43661384-43690891	non-template	41	Exon_22-Intron_22	--	Yes	Yes	Yes	Yes	--	--	--	--	--	Yes
G4ST01043678769	chr1:43678770-43678808	-	GGGTGGAGTCACAGGCTCCCCAGGGACAGGGGCAGGGGG<br>.(((((.....)))))...	KIAA0467	NM_015284	chr1:43661384-43690891	template	39	Exon_37	--	Yes	Yes	--	Yes	--	--	--	--	Yes	--
G4ST01043687788	chr1:43687789-43687823	+	GGGTTTTGGGGAAGGGTGACAAGCCCAGCTTGGGG<br>...(((((...)))))	KIAA0467	NM_015284	chr1:43661384-43690891	non-template	35	Intron_55	--	Yes	--	Yes	--	--	--	--	--	--	--
G4ST01043688918	chr1:43688919-43688948	-	GGGTCAGTGGCTGTGGGTGTTTGGGGAGGG<br>.(((...))).	KIAA0467	NM_015284	chr1:43661384-43690891	template	30	Exon_57.l	--	Yes	Yes	--	--	--	Yes	--	--	--	--
G4ST01043691831	chr1:43691832-43691854	-	GGGTGCGTCGCGGGCCGGGAGGG<br>.((...))	HYI	NM_001190880	chr1:43689411-43692247	non-template	23	Exon_1.f-Intron_1.f	--	Yes	Yes	Yes	Yes	--	--	Yes	Yes	Yes	Yes
G4ST01043691831	chr1:43691832-43691854	-	GGGTGCGTCGCGGGCCGGGAGGG<br>.((...))	HYI	NM_031207	chr1:43689411-43692247	non-template	23	Exon_1.f-Intron_1.f	--	Yes	Yes	Yes	Yes	--	--	Yes	Yes	Yes	Yes
G4ST01043692055	chr1:43692056-43692085	+	GGGGAGGCCGGGCCGGGCGGAGTCCGCGGG<br>(((...))).	HYI	NM_001190880	chr1:43689411-43692247	template	30	Exon_1.f	--	Yes	Yes	--	--	Yes	--	Yes	Yes	Yes	--
G4ST01043692055	chr1:43692056-43692085	+	GGGGAGGCCGGGCCGGGCGGAGTCCGCGGG<br>(((...))).	HYI	NM_031207	chr1:43689411-43692247	template	30	Exon_1.f	--	Yes	Yes	--	--	Yes	--	Yes	Yes	Yes	--
G4ST01043769081	chr1:43769082-43769112	+	GGGGCAGGGGCGCGGGCGCGAGCGCGAGGGG<br>(((....)))..	PTPRF	NM_002840	chr1:43769134-43861930	non-template	-21	upstream	Yes	--	--	--	--	--	--	Yes	Yes	Yes	--
G4ST01043769081	chr1:43769082-43769112	+	GGGGCAGGGGCGCGGGCGCGAGCGCGAGGGG<br>(((....)))..	PTPRF	NM_130440	chr1:43769134-43861930	non-template	-21	upstream	Yes	--	--	--	--	--	--	Yes	Yes	Yes	--
G4ST01043769801	chr1:43769802-43769833	-	GGGAGGGGGCGCCTGTGCGGCGGGGGCGGGGG<br>..((((.....)))).	PTPRF	NM_002840	chr1:43769134-43861930	template	32	Intron_1.f	--	Yes	--	Yes	--	--	--	--	Yes	Yes	--
G4ST01043769801	chr1:43769802-43769833	-	GGGAGGGGGCGCCTGTGCGGCGGGGGCGGGGG<br>..((((.....)))).	PTPRF	NM_130440	chr1:43769134-43861930	template	32	Intron_1.f	--	Yes	--	Yes	--	--	--	--	Yes	Yes	--
G4ST01043775079	chr1:43775080-43775119	+	GGGTCGCCAAGCTGGCGAGGGCCATCTTGGGTCAGTTGGG<br>.(((((...))))).	PTPRF	NM_002840	chr1:43769134-43861930	non-template	40	Intron_1.f	--	Yes	--	Yes	--	--	--	--	--	--	--
G4ST01043775079	chr1:43775080-43775119	+	GGGTCGCCAAGCTGGCGAGGGCCATCTTGGGTCAGTTGGG<br>.(((((...))))).	PTPRF	NM_130440	chr1:43769134-43861930	non-template	40	Intron_1.f	--	Yes	--	Yes	--	--	--	--	--	--	--
G4ST01043783776	chr1:43783777-43783811	+	GGGGTCGGCATGGGAATGGGGGGTGGCACGCAGGG<br>..(((...))).	PTPRF	NM_002840	chr1:43769134-43861930	non-template	35	Intron_3	--	Yes	--	Yes	--	--	--	--	Yes	--	--
G4ST01043783776	chr1:43783777-43783811	+	GGGGTCGGCATGGGAATGGGGGGTGGCACGCAGGG<br>..(((...))).	PTPRF	NM_130440	chr1:43769134-43861930	non-template	35	Intron_3	--	Yes	--	Yes	--	--	--	--	Yes	--	--
G4ST01043796649	chr1:43796650-43796677	-	GGGCACGGCTGTGGGGGATTTGGGGGGG<br>(((....))).	PTPRF	NM_002840	chr1:43769134-43861930	template	28	Intron_5	--	Yes	--	Yes	--	--	--	--	--	--	--
G4ST01043796649	chr1:43796650-43796677	-	GGGCACGGCTGTGGGGGATTTGGGGGGG<br>(((....))).	PTPRF	NM_130440	chr1:43769134-43861930	template	28	Intron_5	--	Yes	--	Yes	--	--	--	--	--	--	--
G4ST01043822081	chr1:43822082-43822112	-	GGGAGCTAGCTGGGAAGCTTGGGCAGAGGGG<br>((....))	PTPRF	NM_002840	chr1:43769134-43861930	template	31	Intron_7	--	Yes	--	Yes	--	--	--	--	--	--	Yes
G4ST01043822081	chr1:43822082-43822112	-	GGGAGCTAGCTGGGAAGCTTGGGCAGAGGGG<br>((....))	PTPRF	NM_130440	chr1:43769134-43861930	template	31	Intron_7	--	Yes	--	Yes	--	--	--	--	--	--	Yes
G4ST01043826794	chr1:43826795-43826830	+	GGGGGCCACTTCTAGGCTGGAAGTGGGTGGGGTGGG<br>....((((((.....))))))	PTPRF	NM_002840	chr1:43769134-43861930	non-template	36	Intron_7	--	Yes	--	Yes	--	--	--	--	--	--	--
G4ST01043826794	chr1:43826795-43826830	+	GGGGGCCACTTCTAGGCTGGAAGTGGGTGGGGTGGG<br>....((((((.....))))))	PTPRF	NM_130440	chr1:43769134-43861930	non-template	36	Intron_7	--	Yes	--	Yes	--	--	--	--	--	--	--
G4ST01043853490	chr1:43853491-43853533	+	GGGCCCCCCTGGGGCCCTGCTCCACACAAGGCTGGGCTTTGGG<br>(((.((.......)).))).	PTPRF	NM_002840	chr1:43769134-43861930	non-template	43	Intron_23	--	Yes	--	Yes	--	--	--	--	--	--	--
G4ST01043853490	chr1:43853491-43853533	+	GGGCCCCCCTGGGGCCCTGCTCCACACAAGGCTGGGCTTTGGG<br>(((.((.......)).))).	PTPRF	NM_130440	chr1:43769134-43861930	non-template	43	Intron_22	--	Yes	--	Yes	--	--	--	--	--	--	--
G4ST01043889209	chr1:43889210-43889240	+	GGGGGAAGGGAATCCTAGGGTTGAAGAAGGG<br>((....))	KDM4A	NM_014663	chr1:43888384-43943776	non-template	31	Intron_1.f	--	Yes	--	Yes	--	--	--	--	Yes	Yes	--
G4ST01043911420	chr1:43911421-43911454	-	GGGGAGTCAGGGAAAGTCACTTGGGTAGTGGGGG<br>.(((...)))	KDM4A	NM_014663	chr1:43888384-43943776	template	34	Intron_11	--	Yes	--	Yes	--	--	--	--	--	--	--
G4ST01043934855	chr1:43934856-43934887	-	GGGCATGGTGAGGGCACCAGGGATGGCTAGGG<br>((...)).	KDM4A	NM_014663	chr1:43888384-43943776	template	32	Intron_18	--	Yes	--	Yes	--	--	--	--	--	--	Yes
G4ST01043943372	chr1:43943373-43943403	+	GGGTGTGGGTGGGGATTACCCTGAATCGGGG<br>((((......)))).	LOC100132774	NR_033827	chr1:43937996-43945599	template	31	Intron_1.f	--	Yes	--	Yes	--	--	--	--	Yes	Yes	--
G4ST01043943372	chr1:43943373-43943403	+	GGGTGTGGGTGGGGATTACCCTGAATCGGGG<br>((((......)))).	KDM4A	NM_014663	chr1:43888384-43943776	non-template	31	Exon_22.l	--	Yes	Yes	--	--	--	Yes	--	Yes	Yes	--
G4ST01043946015	chr1:43946016-43946046	-	GGGACCCGCGGCCGGCGGGGGAGGGGCGGGG<br>..((((.....))))	LOC100132774	NR_033827	chr1:43937996-43945599	non-template	-416	upstream	Yes	--	--	--	--	--	--	Yes	Yes	Yes	Yes
G4ST01043946015	chr1:43946016-43946046	-	GGGACCCGCGGCCGGCGGGGGAGGGGCGGGG<br>..((((.....))))	ST3GAL3	NM_174967	chr1:43945805-44169418	template	31	Intron_1.f	--	Yes	--	Yes	--	--	--	Yes	Yes	Yes	Yes
G4ST01043946015	chr1:43946016-43946046	-	GGGACCCGCGGCCGGCGGGGGAGGGGCGGGG<br>..((((.....))))	ST3GAL3	NM_174966	chr1:43945805-44169418	template	31	Intron_1.f	--	Yes	--	Yes	--	--	--	Yes	Yes	Yes	Yes
G4ST01043946015	chr1:43946016-43946046	-	GGGACCCGCGGCCGGCGGGGGAGGGGCGGGG<br>..((((.....))))	ST3GAL3	NM_174965	chr1:43945805-44169418	template	31	Intron_1.f	--	Yes	--	Yes	--	--	--	Yes	Yes	Yes	Yes
G4ST01043946015	chr1:43946016-43946046	-	GGGACCCGCGGCCGGCGGGGGAGGGGCGGGG<br>..((((.....))))	ST3GAL3	NM_174964	chr1:43945805-44169418	template	31	Intron_1.f	--	Yes	--	Yes	--	--	--	Yes	Yes	Yes	Yes
G4ST01043946015	chr1:43946016-43946046	-	GGGACCCGCGGCCGGCGGGGGAGGGGCGGGG<br>..((((.....))))	ST3GAL3	NM_174963	chr1:43945805-44169418	template	31	Intron_1.f	--	Yes	--	Yes	--	--	--	Yes	Yes	Yes	Yes
G4ST01043946015	chr1:43946016-43946046	-	GGGACCCGCGGCCGGCGGGGGAGGGGCGGGG<br>..((((.....))))	ST3GAL3	NM_174968	chr1:43945805-44169418	template	31	Intron_1.f	--	Yes	--	Yes	--	--	--	Yes	Yes	Yes	Yes
G4ST01043946015	chr1:43946016-43946046	-	GGGACCCGCGGCCGGCGGGGGAGGGGCGGGG<br>..((((.....))))	ST3GAL3	NM_174970	chr1:43945805-44169418	template	31	Intron_1.f	--	Yes	--	Yes	--	--	--	Yes	Yes	Yes	Yes
G4ST01043946015	chr1:43946016-43946046	-	GGGACCCGCGGCCGGCGGGGGAGGGGCGGGG<br>..((((.....))))	ST3GAL3	NM_174969	chr1:43945805-44169418	template	31	Intron_1.f	--	Yes	--	Yes	--	--	--	Yes	Yes	Yes	Yes
G4ST01043946015	chr1:43946016-43946046	-	GGGACCCGCGGCCGGCGGGGGAGGGGCGGGG<br>..((((.....))))	ST3GAL3	NM_006279	chr1:43945805-44169418	template	31	Intron_1.f	--	Yes	--	Yes	--	--	--	Yes	Yes	Yes	Yes

G4ST01043946015	chr1:43946016-43946046	-	GGGACCCGCGGCCGGCGGGGGAGGGGCGGGG<br>..((((.....))))	ST3GAL3	NM_174971	chr1:43945805-44169418	template	31	Intron_1.f	--	Yes	--	Yes	--	--	--	Yes	Yes	Yes	Yes
G4ST01043988000	chr1:43988001-43988031	+	GGGAGATGGGACCCTTTGGGAAGTGATTGGG<br>((....))	ST3GAL3	NM_174967	chr1:43945805-44169418	non-template	31	Intron_2	--	Yes	--	Yes	--	--	--	--	--	--	--
G4ST01043988000	chr1:43988001-43988031	+	GGGAGATGGGACCCTTTGGGAAGTGATTGGG<br>((....))	ST3GAL3	NM_174966	chr1:43945805-44169418	non-template	31	Intron_2	--	Yes	--	Yes	--	--	--	--	--	--	--
G4ST01043988000	chr1:43988001-43988031	+	GGGAGATGGGACCCTTTGGGAAGTGATTGGG<br>((....))	ST3GAL3	NM_174965	chr1:43945805-44169418	non-template	31	Intron_2	--	Yes	--	Yes	--	--	--	--	--	--	--
G4ST01043988000	chr1:43988001-43988031	+	GGGAGATGGGACCCTTTGGGAAGTGATTGGG<br>((....))	ST3GAL3	NM_174964	chr1:43945805-44169418	non-template	31	Intron_2	--	Yes	--	Yes	--	--	--	--	--	--	--
G4ST01043988000	chr1:43988001-43988031	+	GGGAGATGGGACCCTTTGGGAAGTGATTGGG<br>((....))	ST3GAL3	NM_174963	chr1:43945805-44169418	non-template	31	Intron_2	--	Yes	--	Yes	--	--	--	--	--	--	--
G4ST01043988000	chr1:43988001-43988031	+	GGGAGATGGGACCCTTTGGGAAGTGATTGGG<br>((....))	ST3GAL3	NM_174968	chr1:43945805-44169418	non-template	31	Intron_2	--	Yes	--	Yes	--	--	--	--	--	--	--
G4ST01043988000	chr1:43988001-43988031	+	GGGAGATGGGACCCTTTGGGAAGTGATTGGG<br>((....))	ST3GAL3	NM_174970	chr1:43945805-44169418	non-template	31	Intron_2	--	Yes	--	Yes	--	--	--	--	--	--	--
G4ST01043988000	chr1:43988001-43988031	+	GGGAGATGGGACCCTTTGGGAAGTGATTGGG<br>((....))	ST3GAL3	NM_174969	chr1:43945805-44169418	non-template	31	Intron_2	--	Yes	--	Yes	--	--	--	--	--	--	--
G4ST01043988000	chr1:43988001-43988031	+	GGGAGATGGGACCCTTTGGGAAGTGATTGGG<br>((....))	ST3GAL3	NM_006279	chr1:43945805-44169418	non-template	31	Intron_2	--	Yes	--	Yes	--	--	--	--	--	--	--
G4ST01043988000	chr1:43988001-43988031	+	GGGAGATGGGACCCTTTGGGAAGTGATTGGG<br>((....))	ST3GAL3	NM_174971	chr1:43945805-44169418	non-template	31	Intron_2	--	Yes	--	Yes	--	--	--	--	--	--	--
G4ST01044014941	chr1:44014942-44014982	-	GGGCACAGGGAGGAAGGGAACCCTCACACACTGCTGGTGGG<br>.(((..((.....))..)))	ST3GAL3	NM_174967	chr1:43945805-44169418	template	41	Intron_2	--	Yes	--	Yes	--	--	--	Yes	Yes	--	--
G4ST01044014941	chr1:44014942-44014982	-	GGGCACAGGGAGGAAGGGAACCCTCACACACTGCTGGTGGG<br>.(((..((.....))..)))	ST3GAL3	NM_174966	chr1:43945805-44169418	template	41	Intron_2	--	Yes	--	Yes	--	--	--	Yes	Yes	--	--
G4ST01044014941	chr1:44014942-44014982	-	GGGCACAGGGAGGAAGGGAACCCTCACACACTGCTGGTGGG<br>.(((..((.....))..)))	ST3GAL3	NM_174965	chr1:43945805-44169418	template	41	Intron_2	--	Yes	--	Yes	--	--	--	Yes	Yes	--	--
G4ST01044014941	chr1:44014942-44014982	-	GGGCACAGGGAGGAAGGGAACCCTCACACACTGCTGGTGGG<br>.(((..((.....))..)))	ST3GAL3	NM_174964	chr1:43945805-44169418	template	41	Intron_2	--	Yes	--	Yes	--	--	--	Yes	Yes	--	--
G4ST01044014941	chr1:44014942-44014982	-	GGGCACAGGGAGGAAGGGAACCCTCACACACTGCTGGTGGG<br>.(((..((.....))..)))	ST3GAL3	NM_174963	chr1:43945805-44169418	template	41	Intron_2	--	Yes	--	Yes	--	--	--	Yes	Yes	--	--
G4ST01044014941	chr1:44014942-44014982	-	GGGCACAGGGAGGAAGGGAACCCTCACACACTGCTGGTGGG<br>.(((..((.....))..)))	ST3GAL3	NM_174968	chr1:43945805-44169418	template	41	Intron_2	--	Yes	--	Yes	--	--	--	Yes	Yes	--	--
G4ST01044014941	chr1:44014942-44014982	-	GGGCACAGGGAGGAAGGGAACCCTCACACACTGCTGGTGGG<br>.(((..((.....))..)))	ST3GAL3	NM_174970	chr1:43945805-44169418	template	41	Intron_2	--	Yes	--	Yes	--	--	--	Yes	Yes	--	--
G4ST01044014941	chr1:44014942-44014982	-	GGGCACAGGGAGGAAGGGAACCCTCACACACTGCTGGTGGG<br>.(((..((.....))..)))	ST3GAL3	NM_174969	chr1:43945805-44169418	template	41	Intron_2	--	Yes	--	Yes	--	--	--	Yes	Yes	--	--
G4ST01044014941	chr1:44014942-44014982	-	GGGCACAGGGAGGAAGGGAACCCTCACACACTGCTGGTGGG<br>.(((..((.....))..)))	ST3GAL3	NM_006279	chr1:43945805-44169418	template	41	Intron_2	--	Yes	--	Yes	--	--	--	Yes	Yes	--	--
G4ST01044014941	chr1:44014942-44014982	-	GGGCACAGGGAGGAAGGGAACCCTCACACACTGCTGGTGGG<br>.(((..((.....))..)))	ST3GAL3	NM_174971	chr1:43945805-44169418	template	41	Intron_2	--	Yes	--	Yes	--	--	--	Yes	Yes	--	--
G4ST01044037809	chr1:44037810-44037842	-	GGGATTGATATTTTTATCAGGGAGGGCTGAGGG<br>..(((((....)))))	ST3GAL3	NM_174967	chr1:43945805-44169418	template	33	Intron_3	--	Yes	--	Yes	--	--	--	--	--	--	--
G4ST01044037809	chr1:44037810-44037842	-	GGGATTGATATTTTTATCAGGGAGGGCTGAGGG<br>..(((((....)))))	ST3GAL3	NM_174966	chr1:43945805-44169418	template	33	Intron_3	--	Yes	--	Yes	--	--	--	--	--	--	--
G4ST01044037809	chr1:44037810-44037842	-	GGGATTGATATTTTTATCAGGGAGGGCTGAGGG<br>..(((((....)))))	ST3GAL3	NM_174965	chr1:43945805-44169418	template	33	Intron_3	--	Yes	--	Yes	--	--	--	--	--	--	--
G4ST01044037809	chr1:44037810-44037842	-	GGGATTGATATTTTTATCAGGGAGGGCTGAGGG<br>..(((((....)))))	ST3GAL3	NM_174964	chr1:43945805-44169418	template	33	Intron_3	--	Yes	--	Yes	--	--	--	--	--	--	--
G4ST01044037809	chr1:44037810-44037842	-	GGGATTGATATTTTTATCAGGGAGGGCTGAGGG<br>..(((((....)))))	ST3GAL3	NM_174963	chr1:43945805-44169418	template	33	Intron_3	--	Yes	--	Yes	--	--	--	--	--	--	--
G4ST01044037809	chr1:44037810-44037842	-	GGGATTGATATTTTTATCAGGGAGGGCTGAGGG<br>..(((((....)))))	ST3GAL3	NM_174968	chr1:43945805-44169418	template	33	Intron_3	--	Yes	--	Yes	--	--	--	--	--	--	--
G4ST01044037809	chr1:44037810-44037842	-	GGGATTGATATTTTTATCAGGGAGGGCTGAGGG<br>..(((((....)))))	ST3GAL3	NM_174970	chr1:43945805-44169418	template	33	Intron_2	--	Yes	--	Yes	--	--	--	--	--	--	--
G4ST01044037809	chr1:44037810-44037842	-	GGGATTGATATTTTTATCAGGGAGGGCTGAGGG<br>..(((((....)))))	ST3GAL3	NM_174969	chr1:43945805-44169418	template	33	Intron_2	--	Yes	--	Yes	--	--	--	--	--	--	--
G4ST01044037809	chr1:44037810-44037842	-	GGGATTGATATTTTTATCAGGGAGGGCTGAGGG<br>..(((((....)))))	ST3GAL3	NM_006279	chr1:43945805-44169418	template	33	Intron_3	--	Yes	--	Yes	--	--	--	--	--	--	--
G4ST01044037809	chr1:44037810-44037842	-	GGGATTGATATTTTTATCAGGGAGGGCTGAGGG<br>..(((((....)))))	ST3GAL3	NM_174971	chr1:43945805-44169418	template	33	Intron_2	--	Yes	--	Yes	--	--	--	--	--	--	--
G4ST01044065761	chr1:44065762-44065798	+	GGGTTTTGGGGGTTTTGGGGTTTTTTTTGAAACAGGG<br>(((((....))))).	ST3GAL3	NM_174967	chr1:43945805-44169418	non-template	37	Intron_4	--	Yes	--	Yes	--	--	--	--	--	--	--
G4ST01044065761	chr1:44065762-44065798	+	GGGTTTTGGGGGTTTTGGGGTTTTTTTTGAAACAGGG<br>(((((....))))).	ST3GAL3	NM_174966	chr1:43945805-44169418	non-template	37	Intron_4	--	Yes	--	Yes	--	--	--	--	--	--	--
G4ST01044065761	chr1:44065762-44065798	+	GGGTTTTGGGGGTTTTGGGGTTTTTTTTGAAACAGGG<br>(((((....))))).	ST3GAL3	NM_174965	chr1:43945805-44169418	non-template	37	Intron_4	--	Yes	--	Yes	--	--	--	--	--	--	--
G4ST01044065761	chr1:44065762-44065798	+	GGGTTTTGGGGGTTTTGGGGTTTTTTTTGAAACAGGG<br>(((((....))))).	ST3GAL3	NM_174964	chr1:43945805-44169418	non-template	37	Intron_4	--	Yes	--	Yes	--	--	--	--	--	--	--
G4ST01044065761	chr1:44065762-44065798	+	GGGTTTTGGGGGTTTTGGGGTTTTTTTTGAAACAGGG<br>(((((....))))).	ST3GAL3	NM_174963	chr1:43945805-44169418	non-template	37	Intron_5	--	Yes	--	Yes	--	--	--	--	--	--	--
G4ST01044065761	chr1:44065762-44065798	+	GGGTTTTGGGGGTTTTGGGGTTTTTTTTGAAACAGGG<br>(((((....))))).	ST3GAL3	NM_174968	chr1:43945805-44169418	non-template	37	Intron_5	--	Yes	--	Yes	--	--	--	--	--	--	--
G4ST01044065761	chr1:44065762-44065798	+	GGGTTTTGGGGGTTTTGGGGTTTTTTTTGAAACAGGG<br>(((((....))))).	ST3GAL3	NM_174970	chr1:43945805-44169418	non-template	37	Intron_3	--	Yes	--	Yes	--	--	--	--	--	--	--
G4ST01044065761	chr1:44065762-44065798	+	GGGTTTTGGGGGTTTTGGGGTTTTTTTTGAAACAGGG<br>(((((....))))).	ST3GAL3	NM_174969	chr1:43945805-44169418	non-template	37	Intron_3	--	Yes	--	Yes	--	--	--	--	--	--	--
G4ST01044065761	chr1:44065762-44065798	+	GGGTTTTGGGGGTTTTGGGGTTTTTTTTGAAACAGGG<br>(((((....))))).	ST3GAL3	NM_006279	chr1:43945805-44169418	non-template	37	Intron_4	--	Yes	--	Yes	--	--	--	--	--	--	--
G4ST01044065761	chr1:44065762-44065798	+	GGGTTTTGGGGGTTTTGGGGTTTTTTTTGAAACAGGG<br>(((((....))))).	ST3GAL3	NM_174971	chr1:43945805-44169418	non-template	37	Intron_4	--	Yes	--	Yes	--	--	--	--	--	--	--
G4ST01044090290	chr1:44090291-44090323	-	GGGATGGGAGTCAATGACTAGGTGGGCTCAGGG<br>((((...))))....	ST3GAL3	NM_174967	chr1:43945805-44169418	template	33	Intron_5	--	Yes	--	Yes	--	--	--	--	--	--	--
G4ST01044090290	chr1:44090291-44090323	-	GGGATGGGAGTCAATGACTAGGTGGGCTCAGGG<br>((((...))))....	ST3GAL3	NM_174966	chr1:43945805-44169418	template	33	Intron_5	--	Yes	--	Yes	--	--	--	--	--	--	--
G4ST01044090290	chr1:44090291-44090323	-	GGGATGGGAGTCAATGACTAGGTGGGCTCAGGG<br>((((...))))....	ST3GAL3	NM_174965	chr1:43945805-44169418	template	33	Intron_5	--	Yes	--	Yes	--	--	--	--	--	--	--
G4ST01044090290	chr1:44090291-44090323	-	GGGATGGGAGTCAATGACTAGGTGGGCTCAGGG<br>((((...))))....	ST3GAL3	NM_174964	chr1:43945805-44169418	template	33	Intron_5	--	Yes	--	Yes	--	--	--	--	--	--	--
G4ST01044090290	chr1:44090291-44090323	-	GGGATGGGAGTCAATGACTAGGTGGGCTCAGGG<br>((((...))))....	ST3GAL3	NM_174963	chr1:43945805-44169418	template	33	Intron_6	--	Yes	--	Yes	--	--	--	--	--	--	--
G4ST01044090290	chr1:44090291-44090323	-	GGGATGGGAGTCAATGACTAGGTGGGCTCAGGG<br>((((...))))....	ST3GAL3	NM_174968	chr1:43945805-44169418	template	33	Intron_6	--	Yes	--	Yes	--	--	--	--	--	--	--
G4ST01044090290	chr1:44090291-44090323	-	GGGATGGGAGTCAATGACTAGGTGGGCTCAGGG<br>((((...))))....	ST3GAL3	NM_174970	chr1:43945805-44169418	template	33	Intron_4	--	Yes	--	Yes	--	--	--	--	--	--	--
G4ST01044090290	chr1:44090291-44090323	-	GGGATGGGAGTCAATGACTAGGTGGGCTCAGGG<br>((((...))))....	ST3GAL3	NM_174969	chr1:43945805-44169418	template	33	Intron_4	--	Yes	--	Yes	--	--	--	--	--	--	--
G4ST01044090290	chr1:44090291-44090323	-	GGGATGGGAGTCAATGACTAGGTGGGCTCAGGG<br>((((...))))....	ST3GAL3	NM_006279	chr1:43945805-44169418	template	33	Intron_5	--	Yes	--	Yes	--	--	--	--	--	--	--

G4ST01044090290	chr1:44090291-44090323	-	GGGATGGGAGTCAATGACTAGGTGGGCTCAGGG<br>((((...))))....	ST3GAL3	NM_174971	chr1:43945805-44169418	template	33	Intron_5	--	Yes	--	Yes	--	--	--	--	--	--	--
G4ST01044133428	chr1:44133429-44133462	-	GGGCTGATAGAGGGACGTGGGGCACTCCTGGGGG<br>((...)).	ST3GAL3	NM_174967	chr1:43945805-44169418	template	34	Intron_6.l	--	Yes	--	Yes	--	--	--	--	--	--	--
G4ST01044133428	chr1:44133429-44133462	-	GGGCTGATAGAGGGACGTGGGGCACTCCTGGGGG<br>((...)).	ST3GAL3	NM_174966	chr1:43945805-44169418	template	34	Intron_6	--	Yes	--	Yes	--	--	--	--	--	--	--
G4ST01044133428	chr1:44133429-44133462	-	GGGCTGATAGAGGGACGTGGGGCACTCCTGGGGG<br>((...)).	ST3GAL3	NM_174965	chr1:43945805-44169418	template	34	Intron_6.l	--	Yes	--	Yes	--	--	--	--	--	--	--
G4ST01044133428	chr1:44133429-44133462	-	GGGCTGATAGAGGGACGTGGGGCACTCCTGGGGG<br>((...)).	ST3GAL3	NM_174964	chr1:43945805-44169418	template	34	Intron_6	--	Yes	--	Yes	--	--	--	--	--	--	--
G4ST01044133428	chr1:44133429-44133462	-	GGGCTGATAGAGGGACGTGGGGCACTCCTGGGGG<br>((...)).	ST3GAL3	NM_174963	chr1:43945805-44169418	template	34	Intron_7	--	Yes	--	Yes	--	--	--	--	--	--	--
G4ST01044133428	chr1:44133429-44133462	-	GGGCTGATAGAGGGACGTGGGGCACTCCTGGGGG<br>((...)).	ST3GAL3	NM_174968	chr1:43945805-44169418	template	34	Intron_7	--	Yes	--	Yes	--	--	--	--	--	--	--
G4ST01044133428	chr1:44133429-44133462	-	GGGCTGATAGAGGGACGTGGGGCACTCCTGGGGG<br>((...)).	ST3GAL3	NM_174970	chr1:43945805-44169418	template	34	Intron_5.l	--	Yes	--	Yes	--	--	--	--	--	--	--
G4ST01044133428	chr1:44133429-44133462	-	GGGCTGATAGAGGGACGTGGGGCACTCCTGGGGG<br>((...)).	ST3GAL3	NM_174969	chr1:43945805-44169418	template	34	Intron_5	--	Yes	--	Yes	--	--	--	--	--	--	--
G4ST01044133428	chr1:44133429-44133462	-	GGGCTGATAGAGGGACGTGGGGCACTCCTGGGGG<br>((...)).	ST3GAL3	NM_006279	chr1:43945805-44169418	template	34	Intron_6	--	Yes	--	Yes	--	--	--	--	--	--	--
G4ST01044133428	chr1:44133429-44133462	-	GGGCTGATAGAGGGACGTGGGGCACTCCTGGGGG<br>((...)).	ST3GAL3	NM_174971	chr1:43945805-44169418	template	34	Intron_6	--	Yes	--	Yes	--	--	--	--	--	--	--
G4ST01044169346	chr1:44169347-44169392	-	GGGGGTGGCCTGGGACGGCTGCCACAGCTTGGGTAAGCTCCTTGGG<br>...((((...)))).. .(((...)))	ST3GAL3	NM_174967	chr1:43945805-44169418	template	46	Exon_7.l	--	Yes	Yes	--	--	--	Yes	Yes	Yes	--	--
G4ST01044169346	chr1:44169347-44169392	-	GGGGGTGGCCTGGGACGGCTGCCACAGCTTGGGTAAGCTCCTTGGG<br>...((((...)))).. .(((...)))	ST3GAL3	NM_174966	chr1:43945805-44169418	template	46	Exon_10.l	--	Yes	Yes	--	--	--	Yes	Yes	Yes	--	--
G4ST01044169346	chr1:44169347-44169392	-	GGGGGTGGCCTGGGACGGCTGCCACAGCTTGGGTAAGCTCCTTGGG<br>...((((...)))).. .(((...)))	ST3GAL3	NM_174965	chr1:43945805-44169418	template	46	Exon_7.l	--	Yes	Yes	--	--	--	Yes	Yes	Yes	--	--
G4ST01044169346	chr1:44169347-44169392	-	GGGGGTGGCCTGGGACGGCTGCCACAGCTTGGGTAAGCTCCTTGGG<br>...((((...)))).. .(((...)))	ST3GAL3	NM_174964	chr1:43945805-44169418	template	46	Exon_12.l	--	Yes	Yes	--	--	--	Yes	Yes	Yes	--	--
G4ST01044169346	chr1:44169347-44169392	-	GGGGGTGGCCTGGGACGGCTGCCACAGCTTGGGTAAGCTCCTTGGG<br>...((((...)))).. .(((...)))	ST3GAL3	NM_174963	chr1:43945805-44169418	template	46	Exon_13.l	--	Yes	Yes	--	--	--	Yes	Yes	Yes	--	--
G4ST01044169346	chr1:44169347-44169392	-	GGGGGTGGCCTGGGACGGCTGCCACAGCTTGGGTAAGCTCCTTGGG<br>...((((...)))).. .(((...)))	ST3GAL3	NM_174968	chr1:43945805-44169418	template	46	Exon_13.l	--	Yes	Yes	--	--	--	Yes	Yes	Yes	--	--
G4ST01044169346	chr1:44169347-44169392	-	GGGGGTGGCCTGGGACGGCTGCCACAGCTTGGGTAAGCTCCTTGGG<br>...((((...)))).. .(((...)))	ST3GAL3	NM_174970	chr1:43945805-44169418	template	46	Exon_6.l	--	Yes	Yes	--	--	--	Yes	Yes	Yes	--	--
G4ST01044169346	chr1:44169347-44169392	-	GGGGGTGGCCTGGGACGGCTGCCACAGCTTGGGTAAGCTCCTTGGG<br>...((((...)))).. .(((...)))	ST3GAL3	NM_174969	chr1:43945805-44169418	template	46	Exon_11.l	--	Yes	Yes	--	--	--	Yes	Yes	Yes	--	--
G4ST01044169346	chr1:44169347-44169392	-	GGGGGTGGCCTGGGACGGCTGCCACAGCTTGGGTAAGCTCCTTGGG<br>...((((...)))).. .(((...)))	ST3GAL3	NM_006279	chr1:43945805-44169418	template	46	Exon_12.l	--	Yes	Yes	--	--	--	Yes	Yes	Yes	--	--
G4ST01044169346	chr1:44169347-44169392	-	GGGGGTGGCCTGGGACGGCTGCCACAGCTTGGGTAAGCTCCTTGGG<br>...((((...)))).. .(((...)))	ST3GAL3	NM_174971	chr1:43945805-44169418	template	46	Exon_12.l	--	Yes	Yes	--	--	--	Yes	Yes	Yes	--	--
G4ST01044174680	chr1:44174681-44174710	+	GGGCTGGGGGCCCGGGCAGCCGCGCTCGGG<br>.(((...))).	ARTN	NM_001136215	chr1:44171579-44175499	non-template	30	Exon_4.l	--	Yes	Yes	--	Yes	--	--	Yes	Yes	--	--
G4ST01044174680	chr1:44174681-44174710	+	GGGCTGGGGGCCCGGGCAGCCGCGCTCGGG<br>.(((...))).	ARTN	NM_057090	chr1:44171579-44175499	non-template	30	Exon_5.l	--	Yes	Yes	--	Yes	--	--	Yes	Yes	--	--
G4ST01044174680	chr1:44174681-44174710	+	GGGCTGGGGGCCCGGGCAGCCGCGCTCGGG<br>.(((...))).	ARTN	NM_057091	chr1:44171579-44175499	non-template	30	Exon_5.l	--	Yes	Yes	--	Yes	--	--	Yes	Yes	--	--
G4ST01044174685	chr1:44174686-44174719	+	GGGGGCCCGGGCAGCCGCGCTCGGGCAGCGGGGG<br>.(((...))).	ARTN	NM_001136215	chr1:44171579-44175499	non-template	34	Exon_4.l	--	Yes	Yes	--	Yes	--	--	Yes	Yes	--	--
G4ST01044174685	chr1:44174686-44174719	+	GGGGGCCCGGGCAGCCGCGCTCGGGCAGCGGGGG<br>.(((...))).	ARTN	NM_057090	chr1:44171579-44175499	non-template	34	Exon_5.l	--	Yes	Yes	--	Yes	--	--	Yes	Yes	--	--
G4ST01044174685	chr1:44174686-44174719	+	GGGGGCCCGGGCAGCCGCGCTCGGGCAGCGGGGG<br>.(((...))).	ARTN	NM_057091	chr1:44171579-44175499	non-template	34	Exon_5.l	--	Yes	Yes	--	Yes	--	--	Yes	Yes	--	--
G4ST01044174693	chr1:44174694-44174726	+	GGGCAGCCGCGCTCGGGCAGCGGGGGCGCGGGG<br>.(((...))).	ARTN	NM_001136215	chr1:44171579-44175499	non-template	33	Exon_4.l	--	Yes	Yes	--	Yes	--	--	Yes	Yes	--	--
G4ST01044174693	chr1:44174694-44174726	+	GGGCAGCCGCGCTCGGGCAGCGGGGGCGCGGGG<br>.(((...))).	ARTN	NM_057090	chr1:44171579-44175499	non-template	33	Exon_5.l	--	Yes	Yes	--	Yes	--	--	Yes	Yes	--	--
G4ST01044174693	chr1:44174694-44174726	+	GGGCAGCCGCGCTCGGGCAGCGGGGGCGCGGGG<br>.(((...))).	ARTN	NM_057091	chr1:44171579-44175499	non-template	33	Exon_5.l	--	Yes	Yes	--	Yes	--	--	Yes	Yes	--	--
G4ST01044199775	chr1:44199776-44199807	+	GGGGGTGGGAGGTGGGCAGAGGCTCTGAGGGG<br>((((...))))..	IPO13	NM_014652	chr1:44185065-44206281	non-template	32	Intron_14	--	Yes	--	Yes	--	--	--	--	--	--	--
G4ST01044199781	chr1:44199782-44199814	+	GGGAGGTGGGCAGAGGCTCTGAGGGGCTTGGGG<br>((((...))))..	IPO13	NM_014652	chr1:44185065-44206281	non-template	33	Intron_14	--	Yes	--	Yes	--	--	--	--	--	--	--
G4ST01044203697	chr1:44203698-44203728	+	GGGAGGAGGGAAGAATAGGGAGTGTTCTGGG<br>((....))	IPO13	NM_014652	chr1:44185065-44206281	non-template	31	Intron_15	--	Yes	--	Yes	--	--	--	--	--	--	Yes
G4ST01044205139	chr1:44205140-44205170	+	GGGAGAGCTGGGAAGGAGCTGGGCTGATGGG<br>.((...))	IPO13	NM_014652	chr1:44185065-44206281	non-template	31	Intron_17	--	Yes	--	Yes	--	--	--	--	--	--	--
G4ST01044206246	chr1:44206247-44206273	-	GGGATTAATTTAATGGGGATGGGGGGG<br>(((.....))).	DPH2	NM_001039589	chr1:44208240-44211630	template	-1966	upstream	Yes	--	--	--	--	--	--	--	Yes	--	--
G4ST01044206246	chr1:44206247-44206273	-	GGGATTAATTTAATGGGGATGGGGGGG<br>(((.....))).	DPH2	NM_001384	chr1:44208240-44211630	template	-1966	upstream	Yes	--	--	--	--	--	--	--	Yes	--	--
G4ST01044206246	chr1:44206247-44206273	-	GGGATTAATTTAATGGGGATGGGGGGG<br>(((.....))).	IPO13	NM_014652	chr1:44185065-44206281	template	27	Exon_20.l	--	Yes	Yes	--	--	--	Yes	--	Yes	--	--
G4ST01044217380	chr1:44217381-44217414	+	GGGCGGGCTCGCAGCGGCGCGCGGCGGGTGCGGG<br>...((.(((...))).))	B4GALT2	NM_003780	chr1:44217453-44229427	non-template	-38	upstream	Yes	--	--	--	--	--	--	Yes	Yes	Yes	--
G4ST01044217380	chr1:44217381-44217414	+	GGGCGGGCTCGCAGCGGCGCGCGGCGGGTGCGGG<br>...((.(((...))).))	B4GALT2	NM_001005417	chr1:44218770-44229427	non-template	-1355	upstream	Yes	--	--	--	--	--	--	Yes	Yes	Yes	--
G4ST01044217426	chr1:44217427-44217460	+	GGGGGCCGCGGGGGCGCGCGCGCGGGCGCGCGGG<br>.(((....)))	B4GALT2	NM_003780	chr1:44217453-44229427	non-template	8	upstream-Exon_1.f	Yes	Yes	Yes	--	--	Yes	--	Yes	Yes	Yes	--
G4ST01044217426	chr1:44217427-44217460	+	GGGGGCCGCGGGGGCGCGCGCGCGGGCGCGCGGG<br>.(((....)))	B4GALT2	NM_001005417	chr1:44218770-44229427	non-template	-1309	upstream	Yes	--	--	--	--	--	--	Yes	Yes	Yes	--
G4ST01044217435	chr1:44217436-44217465	+	GGGGGCGCGCGCGCGGGCGCGCGGGCCGGG<br>.(((....)))	B4GALT2	NM_003780	chr1:44217453-44229427	non-template	13	upstream-Exon_1.f	Yes	Yes	Yes	--	--	Yes	--	Yes	Yes	Yes	--
G4ST01044217435	chr1:44217436-44217465	+	GGGGGCGCGCGCGCGGGCGCGCGGGCCGGG<br>.(((....)))	B4GALT2	NM_001005417	chr1:44218770-44229427	non-template	-1304	upstream	Yes	--	--	--	--	--	--	Yes	Yes	Yes	--
G4ST01044233894	chr1:44233895-44233923	-	GGGTGGGCTCCAGGGCTGCCTCAGAGGGG<br>(((...)))..	CCDC24	NM_152499	chr1:44229867-44234785	template	29	Exon_7	--	Yes	Yes	--	Yes	--	--	--	--	--	--
G4ST01044233948	chr1:44233949-44233979	-	GGGGTGGGAGCAGGAGGCTGGGTGTTCTGGG<br>(((.....)))	CCDC24	NM_152499	chr1:44229867-44234785	template	31	Intron_7	--	Yes	--	Yes	--	--	--	--	--	--	--
G4ST01044235538	chr1:44235539-44235579	-	GGGTTGCTGGGAGGTCCAAAGCACTTTGGAGGGGTCTCGGG<br>....(((((....)))))..	SLC6A9	NM_006934	chr1:44234742-44255584	non-template	41	Exon_13.l	--	Yes	Yes	--	--	--	Yes	--	--	--	Yes
G4ST01044235538	chr1:44235539-44235579	-	GGGTTGCTGGGAGGTCCAAAGCACTTTGGAGGGGTCTCGGG<br>....(((((....)))))..	SLC6A9	NM_201649	chr1:44234742-44255584	non-template	41	Exon_14.l	--	Yes	Yes	--	--	--	Yes	--	--	--	Yes
G4ST01044235538	chr1:44235539-44235579	-	GGGTTGCTGGGAGGTCCAAAGCACTTTGGAGGGGTCTCGGG<br>....(((((....)))))..	SLC6A9	NM_001024845	chr1:44234742-44269721	non-template	41	Exon_14.l	--	Yes	Yes	--	--	--	Yes	--	--	--	Yes
G4ST01044235545	chr1:44235546-44235588	-	GGGAGTGGAGGGTTGCTGGGAGGTCCAAAGCACTTTGGAGGGG<br>....(((((....)))))..	SLC6A9	NM_006934	chr1:44234742-44255584	non-template	43	Exon_13.l	--	Yes	Yes	--	--	--	Yes	--	--	--	Yes
G4ST01044235545	chr1:44235546-44235588	-	GGGAGTGGAGGGTTGCTGGGAGGTCCAAAGCACTTTGGAGGGG<br>....(((((....)))))..	SLC6A9	NM_201649	chr1:44234742-44255584	non-template	43	Exon_14.l	--	Yes	Yes	--	--	--	Yes	--	--	--	Yes

G4ST01044235545	chr1:44235546-44235588	-	GGGAGTGGAGGGTTGCTGGGAGGTCCAAAGCACTTTGGAGGGG<br>....(((((....))))))..	SLC6A9	NM_001024845	chr1:44234742-44269721	non-template	43	Exon_14.l	--	Yes	Yes	--	--	--	Yes	--	--	--	Yes
G4ST01044236390	chr1:44236391-44236431	+	GGGCATTTGGGGCAGAACAGGGACAGCTGTCAACATCTGGG<br>..((.((....)).))	SLC6A9	NM_006934	chr1:44234742-44255584	template	41	Intron_11	--	Yes	--	Yes	--	--	--	--	--	--	--
G4ST01044236390	chr1:44236391-44236431	+	GGGCATTTGGGGCAGAACAGGGACAGCTGTCAACATCTGGG<br>..((.((....)).))	SLC6A9	NM_201649	chr1:44234742-44255584	template	41	Intron_12	--	Yes	--	Yes	--	--	--	--	--	--	--
G4ST01044236390	chr1:44236391-44236431	+	GGGCATTTGGGGCAGAACAGGGACAGCTGTCAACATCTGGG<br>..((.((....)).))	SLC6A9	NM_001024845	chr1:44234742-44269721	template	41	Intron_12	--	Yes	--	Yes	--	--	--	--	--	--	--
G4ST01044241394	chr1:44241395-44241432	-	GGGGCTGCAGCAGGTGGAGGGGACTGGGCCGGCCAGGG<br>.((.((.....)).))	SLC6A9	NM_006934	chr1:44234742-44255584	non-template	38	Intron_4	--	Yes	--	Yes	--	--	--	--	--	--	Yes
G4ST01044241394	chr1:44241395-44241432	-	GGGGCTGCAGCAGGTGGAGGGGACTGGGCCGGCCAGGG<br>.((.((.....)).))	SLC6A9	NM_201649	chr1:44234742-44255584	non-template	38	Intron_5	--	Yes	--	Yes	--	--	--	--	--	--	Yes
G4ST01044241394	chr1:44241395-44241432	-	GGGGCTGCAGCAGGTGGAGGGGACTGGGCCGGCCAGGG<br>.((.((.....)).))	SLC6A9	NM_001024845	chr1:44234742-44269721	non-template	38	Intron_5	--	Yes	--	Yes	--	--	--	--	--	--	Yes
G4ST01044241404	chr1:44241405-44241442	-	GGGCTCCCCAGGGGCTGCAGCAGGTGGAGGGGACTGGG<br>.((.((.....)).))	SLC6A9	NM_006934	chr1:44234742-44255584	non-template	38	Intron_4	--	Yes	--	Yes	--	--	--	--	--	--	Yes
G4ST01044241404	chr1:44241405-44241442	-	GGGCTCCCCAGGGGCTGCAGCAGGTGGAGGGGACTGGG<br>.((.((.....)).))	SLC6A9	NM_201649	chr1:44234742-44255584	non-template	38	Intron_5	--	Yes	--	Yes	--	--	--	--	--	--	Yes
G4ST01044241404	chr1:44241405-44241442	-	GGGCTCCCCAGGGGCTGCAGCAGGTGGAGGGGACTGGG<br>.((.((.....)).))	SLC6A9	NM_001024845	chr1:44234742-44269721	non-template	38	Intron_5	--	Yes	--	Yes	--	--	--	--	--	--	Yes
G4ST01044249341	chr1:44249342-44249383	-	GGGCTGTGCACCTGGCACTGGGTGCTGTGGGGCTGGTGAGGG<br>..((((.....)))).	SLC6A9	NM_006934	chr1:44234742-44255584	non-template	42	Intron_1.f	--	Yes	--	Yes	--	--	--	--	--	--	--
G4ST01044249341	chr1:44249342-44249383	-	GGGCTGTGCACCTGGCACTGGGTGCTGTGGGGCTGGTGAGGG<br>..((((.....)))).	SLC6A9	NM_201649	chr1:44234742-44255584	non-template	42	Intron_2	--	Yes	--	Yes	--	--	--	--	--	--	--
G4ST01044249341	chr1:44249342-44249383	-	GGGCTGTGCACCTGGCACTGGGTGCTGTGGGGCTGGTGAGGG<br>..((((.....)))).	SLC6A9	NM_001024845	chr1:44234742-44269721	non-template	42	Intron_2	--	Yes	--	Yes	--	--	--	--	--	--	--
G4ST01044259629	chr1:44259630-44259664	+	GGGAGGGACACGTGCAGCAGGCATGTGGGCCTGGG<br>..((((((.....))))))	SLC6A9	NM_001024845	chr1:44234742-44269721	template	35	Intron_2	--	Yes	--	Yes	--	--	--	--	--	--	--
G4ST01044260822	chr1:44260823-44260863	+	GGGGGCTAGACCAGAGCTGGCCTGAGGGTTGGGGCCAGGGG<br>((((((.......))))))...	SLC6A9	NM_001024845	chr1:44234742-44269721	template	41	Intron_2	--	Yes	--	Yes	--	--	--	--	--	--	--
G4ST01044268614	chr1:44268615-44268649	-	GGGGCCGGTGCCGGCCCGGGGGCGGGGCCGTGGGG<br>((((....))))....	SLC6A9	NM_001024845	chr1:44234742-44269721	non-template	35	Intron_1.f	--	Yes	--	Yes	--	--	--	Yes	Yes	Yes	Yes
G4ST01044268622	chr1:44268623-44268653	-	GGGAGGGGCCGGTGCCGGCCCGGGGGCGGGG<br>((((....))))....	SLC6A9	NM_001024845	chr1:44234742-44269721	non-template	31	Intron_1.f	--	Yes	--	Yes	--	--	--	Yes	Yes	Yes	Yes
G4ST01044268627	chr1:44268628-44268658	-	GGGCCGGGAGGGGCCGGTGCCGGCCCGGGGG<br>((((....))))....	SLC6A9	NM_001024845	chr1:44234742-44269721	non-template	31	Intron_1.f	--	Yes	--	Yes	--	--	--	Yes	Yes	Yes	Yes
G4ST01044357459	chr1:44357460-44357486	+	GGGAGACTGGGGAGGGATTGGAAAGGG<br>.((...))	KLF17	NM_173484	chr1:44357109-44373396	non-template	27	Intron_1.f	--	Yes	--	Yes	--	--	--	--	--	--	Yes
G4ST01044357467	chr1:44357468-44357490	+	GGGGAGGGATTGGAAAGGGAGGG<br>.((...))	KLF17	NM_173484	chr1:44357109-44373396	non-template	23	Intron_1.f	--	Yes	--	Yes	--	--	--	--	--	--	Yes
G4ST01044371947	chr1:44371948-44371988	-	GGGGTGAGAGGGCAGGGGCAGGCACAGCCAAGGCTGTGGGG<br>.....((((((....))))))	KLF17	NM_173484	chr1:44357109-44373396	template	41	Exon_4.l	--	Yes	Yes	--	--	--	Yes	--	--	--	Yes
G4ST01044467671	chr1:44467672-44467708	+	GGGCCCTGGTGGCAGGCTGGGTGGGGGCCAGGCTGGG<br>.((((....))))..	ERI3	NM_024066	chr1:44459329-44593526	template	37	Intron_8.l	--	Yes	--	Yes	--	--	--	--	--	--	--
G4ST01044479363	chr1:44479364-44479401	+	GGGATTGATTCTCAGGGTACCAGGAACTGGGGGAGGGG<br>..(((...))) ..((((...))))	ERI3	NM_024066	chr1:44459329-44593526	template	38	Intron_8.l	--	Yes	--	Yes	--	--	--	--	--	--	--
G4ST01044486437	chr1:44486438-44486470	-	GGGAGTTTCTGCTGGGTCTGGGGAGGACCTGGG<br>(((....)))	ERI3	NM_024066	chr1:44459329-44593526	non-template	33	Intron_7	--	Yes	--	Yes	--	--	--	--	--	--	Yes
G4ST01044502065	chr1:44502066-44502094	-	GGGAGGAAGGGCTCAGGGCTCAGAGAGGG<br>((...)).	ERI3	NM_024066	chr1:44459329-44593526	non-template	29	Intron_7	--	Yes	--	Yes	--	--	--	--	--	--	--
G4ST01044535506	chr1:44535507-44535532	-	GGGAAGGGCAACTATGAGTGGGTGGG<br>..(((...)))	ERI3	NM_024066	chr1:44459329-44593526	non-template	26	Intron_6	--	Yes	--	Yes	--	--	--	--	--	--	--
G4ST01044535510	chr1:44535511-44535539	-	GGGCTTTGGGAAGGGCAACTATGAGTGGG<br>..(((...)))	ERI3	NM_024066	chr1:44459329-44593526	non-template	29	Intron_6	--	Yes	--	Yes	--	--	--	--	--	--	--
G4ST01044551116	chr1:44551117-44551144	-	GGGGTAGCCTATGCTGGGTGGGCTGGGG<br>..(((....)))	ERI3	NM_024066	chr1:44459329-44593526	non-template	28	Intron_5	--	Yes	--	Yes	--	--	--	--	--	--	Yes
G4ST01044582799	chr1:44582800-44582832	-	GGGTGCAAGGGCAAGTGGGATGCTGCTGCAGGG<br>.(((....)))	ERI3	NM_024066	chr1:44459329-44593526	non-template	33	Intron_2	--	Yes	--	Yes	--	--	--	--	--	--	--
G4ST01044645342	chr1:44645343-44645375	+	GGGTGCATGGGGCCCGGGCCATAGGTGTGGGGG<br>((((....))))	RNF220	NM_018150	chr1:44643547-44889983	non-template	33	Intron_1.f	--	Yes	--	Yes	--	--	--	--	--	--	Yes
G4ST01044683641	chr1:44683642-44683678	-	GGGCAATGGAGAGTCATTGAAGGGTTTTAGGGACGGG<br>((((((....))))))..	RNF220	NM_018150	chr1:44643547-44889983	template	37	Intron_2	--	Yes	--	Yes	--	--	--	--	--	--	--
G4ST01044803857	chr1:44803858-44803892	+	GGGCAAGCATGCCCCGCAGCTGGGGAGGGTGGGGG<br>..(((.(((...)))))).	RNF220	NM_018150	chr1:44643547-44889983	non-template	35	Intron_2	--	Yes	--	Yes	--	--	--	--	--	--	--
G4ST01044831434	chr1:44831435-44831461	-	GGGCATGGGATGGAACAGGGAAAAGGG<br>.((...))	RNF220	NM_018150	chr1:44643547-44889983	template	27	Intron_2	--	Yes	--	Yes	--	--	--	--	--	--	--
G4ST01044831441	chr1:44831442-44831468	-	GGGTCTAGGGCATGGGATGGAACAGGG<br>.((...))	RNF220	NM_018150	chr1:44643547-44889983	template	27	Intron_2	--	Yes	--	Yes	--	--	--	--	--	--	--
G4ST01044842591	chr1:44842592-44842619	-	GGGCCAGTGGAGGGTGGGGCAGGCAGGG<br>((...)).	RNF220	NM_018150	chr1:44643547-44889983	template	28	Intron_2	--	Yes	--	Yes	--	--	--	Yes	--	--	--
G4ST01044842600	chr1:44842601-44842624	-	GGGCTGGGCCAGTGGAGGGTGGGG<br>((...)).	RNF220	NM_018150	chr1:44643547-44889983	template	24	Intron_2	--	Yes	--	Yes	--	--	--	Yes	--	--	--
G4ST01044842605	chr1:44842606-44842629	-	GGGTTGGGCTGGGCCAGTGGAGGG<br>((...)).	RNF220	NM_018150	chr1:44643547-44889983	template	24	Intron_2	--	Yes	--	Yes	--	--	--	Yes	--	--	--
G4ST01044855830	chr1:44855831-44855860	-	GGGGCGGGGCAAGGGCAGCCAAGGCTGGGG<br>((((....))))	RNF220	NM_018150	chr1:44643547-44889983	template	30	Intron_3	--	Yes	--	Yes	--	--	--	Yes	--	--	Yes
G4ST01044869859	chr1:44869860-44869893	+	GGGGTTGGGCGGGGATCGCCCACAGGCGATAGGG<br>.((((((....)))))).	RNF220	NM_018150	chr1:44643547-44889983	non-template	34	Intron_5	--	Yes	--	Yes	--	--	--	Yes	Yes	Yes	--
G4ST01044909196	chr1:44909197-44909227	+	GGGAGGAGGGGTGGCACGGCCTGGGCCTGGG<br>..(((...))).	TMEM53	NM_024587	chr1:44892088-44912686	template	31	Intron_1.f	--	Yes	--	Yes	--	--	--	--	--	--	--
G4ST01044920731	chr1:44920732-44920757	+	GGGTTGAGGCAAGGGGTGGGGTGGGG<br>(((...))).	C1orf228	NM_001145636	chr1:44912981-44963850	non-template	26	Intron_1.f	--	Yes	--	Yes	--	--	--	Yes	Yes	--	--
G4ST01044920743	chr1:44920744-44920776	+	GGGGTGGGGTGGGGTGTCAGGTTTCACCTGGGG<br>....(((((...)))))	C1orf228	NM_001145636	chr1:44912981-44963850	non-template	33	Intron_1.f	--	Yes	--	Yes	--	--	--	Yes	Yes	--	--
G4ST01044941584	chr1:44941585-44941613	-	GGGAGGGAGGGCAGTGGTGAGTCACTGGG<br>.(((((....)))))	C1orf228	NM_001145636	chr1:44912981-44963850	template	29	Intron_5	--	Yes	--	Yes	--	--	--	Yes	Yes	--	Yes
G4ST01044962707	chr1:44962708-44962739	+	GGGCCCCGGGAGCGCTCCGTGCGCCGGGTGGG<br>.((((.....)))).	C1orf228	NM_001145636	chr1:44912981-44963850	non-template	32	Intron_8	--	Yes	--	Yes	--	--	--	Yes	--	Yes	--
G4ST01044962714	chr1:44962715-44962745	+	GGGAGCGCTCCGTGCGCCGGGTGGGCGGGGG<br>.((((.....)))).	C1orf228	NM_001145636	chr1:44912981-44963850	non-template	31	Intron_8	--	Yes	--	Yes	--	--	--	Yes	--	Yes	--
G4ST01044962732	chr1:44962733-44962756	+	GGGTGGGCGGGGGTGTGCGCCGGG<br>(((....)))	C1orf228	NM_001145636	chr1:44912981-44963850	non-template	24	Intron_8	--	Yes	--	Yes	--	--	--	Yes	--	Yes	--
G4ST01045005470	chr1:45005471-45005495	-	GGGGAGGGCCAGGGAAGAGGCTGGG<br>.((...))	KIF2C	NM_006845	chr1:44978077-45006025	template	25	Exon_21.l	--	Yes	Yes	--	--	--	Yes	--	--	--	--
G4ST01045005583	chr1:45005584-45005616	+	GGGCAGCTGGGGAGGGGGTCAGAGTGACATGGG<br>.((((...))))..	KIF2C	NM_006845	chr1:44978077-45006025	non-template	33	Exon_21.l	--	Yes	Yes	--	--	--	Yes	--	--	--	Yes
G4ST01045013602	chr1:45013603-45013642	-	GGGCTGGGGGAAGGGGGTCTGCGGCGTCGGCAGATCTGGG<br>(((((((.......))))))).	RPS8	NM_001012	chr1:45013833-45016999	template	-190	upstream	Yes	--	--	--	--	--	--	Yes	Yes	Yes	--
G4ST01045013602	chr1:45013603-45013642	-	GGGCTGGGGGAAGGGGGTCTGCGGCGTCGGCAGATCTGGG<br>(((((((.......))))))).	SNORD55	NR_000015	chr1:45014124-45014197	template	-481	upstream	Yes	--	--	--	--	--	--	Yes	Yes	Yes	--

G4ST01045013602	chr1:45013603-45013642	-	GGGCTGGGGGAAGGGGGTCTGCGGCGTCGGCAGATCTGGG<br>(((((((.......))))))).	SNORD46	NR_000024	chr1:45014751-45014848	template	-1108	upstream	Yes	--	--	--	--	--	--	Yes	Yes	Yes	--
G4ST01045024630	chr1:45024631-45024661	-	GGGAGAGTCCTGGGAGCAGGGGCCTCCAGGG<br>((....))	BEST4	NM_153274	chr1:45021844-45026013	non-template	31	Intron_3	--	Yes	--	Yes	--	--	--	Yes	Yes	Yes	Yes
G4ST01045052235	chr1:45052236-45052263	+	GGGCTTGGGCTGGGCCTCCCTGAGGGGG<br>((((...))))	BTBD19	NM_001136537	chr1:45046741-45052388	non-template	28	Exon_8.l	--	Yes	Yes	--	--	--	Yes	Yes	Yes	Yes	Yes
G4ST01045064650	chr1:45064651-45064687	+	GGGAGACAGGGCTCACAGAGGGCTCCTGGCAGGAGGG<br>((...)). ..(((...))).	PTCH2	NM_001166292	chr1:45058103-45081203	template	37	Intron_18	--	Yes	--	Yes	--	--	--	--	Yes	--	--
G4ST01045064650	chr1:45064651-45064687	+	GGGAGACAGGGCTCACAGAGGGCTCCTGGCAGGAGGG<br>((...)). ..(((...))).	PTCH2	NM_003738	chr1:45060525-45081203	template	37	Intron_18	--	Yes	--	Yes	--	--	--	--	Yes	--	--
G4ST01045066040	chr1:45066041-45066083	-	GGGCAGAGACTTAGCATCTCTGGGCCCAGAAGGGCAGAGAGGG<br>.(((((.......)))))	PTCH2	NM_001166292	chr1:45058103-45081203	non-template	43	Intron_14	--	Yes	--	Yes	--	--	--	--	--	--	--
G4ST01045066040	chr1:45066041-45066083	-	GGGCAGAGACTTAGCATCTCTGGGCCCAGAAGGGCAGAGAGGG<br>.(((((.......)))))	PTCH2	NM_003738	chr1:45060525-45081203	non-template	43	Intron_14	--	Yes	--	Yes	--	--	--	--	--	--	--
G4ST01045066049	chr1:45066050-45066093	-	GGGCAGAGCAGGGCAGAGACTTAGCATCTCTGGGCCCAGAAGGG<br>.(((((.......)))))	PTCH2	NM_001166292	chr1:45058103-45081203	non-template	44	Intron_14	--	Yes	--	Yes	--	--	--	--	--	--	--
G4ST01045066049	chr1:45066050-45066093	-	GGGCAGAGCAGGGCAGAGACTTAGCATCTCTGGGCCCAGAAGGG<br>.(((((.......)))))	PTCH2	NM_003738	chr1:45060525-45081203	non-template	44	Intron_14	--	Yes	--	Yes	--	--	--	--	--	--	--
G4ST01045067498	chr1:45067499-45067540	-	GGGACTGCGCCCAGTCCCAGGGTTCCGTGGGCCTTGCCGGGG<br>((((....))))....	PTCH2	NM_001166292	chr1:45058103-45081203	non-template	42	Exon_10	--	Yes	Yes	--	Yes	--	--	--	--	--	--
G4ST01045067498	chr1:45067499-45067540	-	GGGACTGCGCCCAGTCCCAGGGTTCCGTGGGCCTTGCCGGGG<br>((((....))))....	PTCH2	NM_003738	chr1:45060525-45081203	non-template	42	Exon_10	--	Yes	Yes	--	Yes	--	--	--	--	--	--
G4ST01045081370	chr1:45081371-45081401	+	GGGAGCTGCGGGTCCGGGGCGCGGCGCCGGG<br>(((...))).	PTCH2	NM_001166292	chr1:45058103-45081203	template	-167	upstream	Yes	--	--	--	--	--	--	--	Yes	Yes	--
G4ST01045081370	chr1:45081371-45081401	+	GGGAGCTGCGGGTCCGGGGCGCGGCGCCGGG<br>(((...))).	PTCH2	NM_003738	chr1:45060525-45081203	template	-167	upstream	Yes	--	--	--	--	--	--	--	Yes	Yes	--
G4ST01045081659	chr1:45081660-45081690	-	GGGAGGGAGGCGCGGGGTGGCCCAGCCTGGG<br>..(((...))).	PTCH2	NM_001166292	chr1:45058103-45081203	non-template	-456	upstream	Yes	--	--	--	--	--	--	Yes	Yes	Yes	Yes
G4ST01045081659	chr1:45081660-45081690	-	GGGAGGGAGGCGCGGGGTGGCCCAGCCTGGG<br>..(((...))).	PTCH2	NM_003738	chr1:45060525-45081203	non-template	-456	upstream	Yes	--	--	--	--	--	--	Yes	Yes	Yes	Yes
G4ST01045114918	chr1:45114919-45114958	-	GGGTACACCTCCCAGAGGGGGTCGTGGCCGGGCAGAGGGG<br>....((((...)))) (((....)))	EIF2B3	NM_020365	chr1:45088781-45224948	non-template	40	Intron_8	--	Yes	--	Yes	--	--	--	--	--	--	--
G4ST01045114918	chr1:45114919-45114958	-	GGGTACACCTCCCAGAGGGGGTCGTGGCCGGGCAGAGGGG<br>....((((...)))) (((....)))	EIF2B3	NM_001166588	chr1:45112779-45224948	non-template	40	Intron_8	--	Yes	--	Yes	--	--	--	--	--	--	--
G4ST01045444771	chr1:45444772-45444805	+	GGGCCCGGGAGCGCGCCGCGAGGGCAGACGCGGG<br>..(((...))).	ZSWIM5	NM_020883	chr1:45254663-45444837	template	34	Exon_1.f	--	Yes	Yes	--	--	Yes	--	Yes	Yes	Yes	--
G4ST01045541887	chr1:45541888-45541918	+	GGGAGCTCGGAGCGGGGGCCGGGGCCGAGGG<br>.(((...))))..	LOC400752	NR_024270	chr1:45542169-45543878	non-template	-250	upstream	Yes	--	--	--	--	--	--	Yes	Yes	Yes	Yes
G4ST01045542005	chr1:45542006-45542039	+	GGGGGCGGGTCCAGAATGGCGGGAGCTTCCGGGG<br>.(((...))).	LOC400752	NR_024270	chr1:45542169-45543878	non-template	-129	upstream	Yes	--	--	--	--	--	--	Yes	Yes	Yes	--
G4ST01045651606	chr1:45651607-45651644	+	GGGAGATAAAGGGAAAAAGGGAAACTAGGAGGTTGGGG<br>.((((....)))).	TESK2	NM_007170	chr1:45582142-45729427	template	38	Intron_3	--	Yes	--	Yes	--	--	--	--	--	--	--
G4ST01045807856	chr1:45807857-45807888	+	GGGGTTGGGAGGGAAGTCGGCTGTACTTAGGG<br>((((.......)))).	AKR1A1	NM_001202413	chr1:45789042-45808310	non-template	32	Intron_9.l	--	Yes	--	Yes	--	--	--	--	--	--	Yes
G4ST01045807856	chr1:45807857-45807888	+	GGGGTTGGGAGGGAAGTCGGCTGTACTTAGGG<br>((((.......)))).	AKR1A1	NM_001202414	chr1:45789042-45808310	non-template	32	Intron_10.l	--	Yes	--	Yes	--	--	--	--	--	--	Yes
G4ST01045807856	chr1:45807857-45807888	+	GGGGTTGGGAGGGAAGTCGGCTGTACTTAGGG<br>((((.......)))).	AKR1A1	NM_006066	chr1:45789042-45808310	non-template	32	Intron_9.l	--	Yes	--	Yes	--	--	--	--	--	--	Yes
G4ST01045807856	chr1:45807857-45807888	+	GGGGTTGGGAGGGAAGTCGGCTGTACTTAGGG<br>((((.......)))).	AKR1A1	NM_153326	chr1:45789042-45808310	non-template	32	Intron_8.l	--	Yes	--	Yes	--	--	--	--	--	--	Yes
G4ST01045821647	chr1:45821648-45821683	+	GGGCAGGGCCTAAGAGGATGGGATCAGCGGATTGGG<br>(((...))).. (((....))).	NASP	NM_002482	chr1:45822247-45857165	non-template	-563	upstream	Yes	--	--	--	--	--	--	Yes	Yes	Yes	--
G4ST01045821647	chr1:45821648-45821683	+	GGGCAGGGCCTAAGAGGATGGGATCAGCGGATTGGG<br>(((...))).. (((....))).	NASP	NM_001195193	chr1:45822247-45857165	non-template	-563	upstream	Yes	--	--	--	--	--	--	Yes	Yes	Yes	--
G4ST01045821647	chr1:45821648-45821683	+	GGGCAGGGCCTAAGAGGATGGGATCAGCGGATTGGG<br>(((...))).. (((....))).	NASP	NM_152298	chr1:45822247-45857165	non-template	-563	upstream	Yes	--	--	--	--	--	--	Yes	Yes	Yes	--
G4ST01045822227	chr1:45822228-45822264	+	GGGGGAGGATCCGGAAGTGGATGGGCGGGGCGAAGGG<br>.....(((((....)))))	NASP	NM_002482	chr1:45822247-45857165	non-template	18	upstream-Exon_1.f	Yes	Yes	Yes	--	--	Yes	--	Yes	Yes	Yes	--
G4ST01045822227	chr1:45822228-45822264	+	GGGGGAGGATCCGGAAGTGGATGGGCGGGGCGAAGGG<br>.....(((((....)))))	NASP	NM_001195193	chr1:45822247-45857165	non-template	18	upstream-Exon_1.f	Yes	Yes	Yes	--	--	Yes	--	Yes	Yes	Yes	--
G4ST01045822227	chr1:45822228-45822264	+	GGGGGAGGATCCGGAAGTGGATGGGCGGGGCGAAGGG<br>.....(((((....)))))	NASP	NM_152298	chr1:45822247-45857165	non-template	18	upstream-Exon_1.f	Yes	Yes	Yes	--	--	Yes	--	Yes	Yes	Yes	--
G4ST01045823002	chr1:45823003-45823033	+	GGGGCAGGGGGCGGGGCCTTGAGGAGGCGGG<br>((((.....))))	NASP	NM_002482	chr1:45822247-45857165	non-template	31	Intron_1.f	--	Yes	--	Yes	--	--	--	Yes	Yes	Yes	--
G4ST01045823002	chr1:45823003-45823033	+	GGGGCAGGGGGCGGGGCCTTGAGGAGGCGGG<br>((((.....))))	NASP	NM_001195193	chr1:45822247-45857165	non-template	31	Intron_1.f	--	Yes	--	Yes	--	--	--	Yes	Yes	Yes	--
G4ST01045823002	chr1:45823003-45823033	+	GGGGCAGGGGGCGGGGCCTTGAGGAGGCGGG<br>((((.....))))	NASP	NM_152298	chr1:45822247-45857165	non-template	31	Intron_1.f	--	Yes	--	Yes	--	--	--	Yes	Yes	Yes	--
G4ST01045823008	chr1:45823009-45823052	+	GGGGGCGGGGCCTTGAGGAGGCGGGAAAACTCCATTGTTTGGGG<br>((((.....)))) .((((......)))).	NASP	NM_002482	chr1:45822247-45857165	non-template	44	Intron_1.f	--	Yes	--	Yes	--	--	--	Yes	Yes	Yes	--
G4ST01045823008	chr1:45823009-45823052	+	GGGGGCGGGGCCTTGAGGAGGCGGGAAAACTCCATTGTTTGGGG<br>((((.....)))) .((((......)))).	NASP	NM_001195193	chr1:45822247-45857165	non-template	44	Intron_1.f	--	Yes	--	Yes	--	--	--	Yes	Yes	Yes	--
G4ST01045823008	chr1:45823009-45823052	+	GGGGGCGGGGCCTTGAGGAGGCGGGAAAACTCCATTGTTTGGGG<br>((((.....)))) .((((......)))).	NASP	NM_152298	chr1:45822247-45857165	non-template	44	Intron_1.f	--	Yes	--	Yes	--	--	--	Yes	Yes	Yes	--
G4ST01046125807	chr1:46125808-46125836	+	GGGCAGGGAGGAGTTCCTGGGCCCCAGGG<br>(((....)))	MAST2	NM_015112	chr1:46041872-46274383	non-template	29	Intron_4	--	Yes	--	Yes	--	--	--	Yes	Yes	--	--
G4ST01046145960	chr1:46145961-46145995	-	GGGGTAAGGGAAGAAGGGTGTTACTGCTTAATGGG<br>.((((.....))))	MAST2	NM_015112	chr1:46041872-46274383	template	35	Intron_4	--	Yes	--	Yes	--	--	--	--	--	--	--
G4ST01046267073	chr1:46267074-46267109	+	GGGCTATGGGAAGCCAGTGGACTGGTGGGCCATGGG<br>...((((....)))).	MAST2	NM_015112	chr1:46041872-46274383	non-template	36	Exon_18	--	Yes	Yes	--	Yes	--	--	--	--	--	--
G4ST01046319847	chr1:46319848-46319886	-	GGGACCAGTTTCATTGCTGGGGTGGGGTTAGGCTGGGGG<br>..((((......))))	PIK3R3	NM_003629	chr1:46278399-46370967	non-template	39	Intron_1.f	--	Yes	--	Yes	--	--	--	--	--	--	--
G4ST01046319847	chr1:46319848-46319886	-	GGGACCAGTTTCATTGCTGGGGTGGGGTTAGGCTGGGGG<br>..((((......))))	PIK3R3	NM_001114172	chr1:46278399-46371295	non-template	39	Intron_2	--	Yes	--	Yes	--	--	--	--	--	--	--
G4ST01046412153	chr1:46412154-46412180	+	GGGGAGGGAGGCTTCTGGGTCCCAGGG<br>((....))	TSPAN1	NM_005727	chr1:46413336-46424221	non-template	-1155	upstream	Yes	--	--	--	--	--	--	--	Yes	--	Yes
G4ST01046412158	chr1:46412159-46412189	+	GGGAGGCTTCTGGGTCCCAGGGCCGCAGGGG<br>((....))	TSPAN1	NM_005727	chr1:46413336-46424221	non-template	-1146	upstream	Yes	--	--	--	--	--	--	--	Yes	--	Yes
G4ST01046435541	chr1:46435542-46435568	-	GGGTAGGGAGGGCCCTAATGGAGGGGG<br>.(((.....)))	POMGNT1	NM_017739	chr1:46426940-46436708	non-template	27	Intron_2	--	Yes	--	Yes	--	--	--	--	--	--	--
G4ST01046435541	chr1:46435542-46435568	-	GGGTAGGGAGGGCCCTAATGGAGGGGG<br>.(((.....)))	POMGNT1	NR_024332	chr1:46426940-46458564	non-template	27	Intron_2	--	Yes	--	Yes	--	--	--	--	--	--	--
G4ST01046441552	chr1:46441553-46441589	-	GGGTCCCCGCCCGCGCGGCGCGGGGGCGGGAGGAGGG<br>.......(((((...)))))	POMGNT1	NR_024332	chr1:46426940-46458564	non-template	37	Intron_1.f	--	Yes	--	Yes	--	--	--	Yes	Yes	Yes	--
G4ST01046441552	chr1:46441553-46441589	-	GGGTCCCCGCCCGCGCGGCGCGGGGGCGGGAGGAGGG<br>.......(((((...)))))	C1orf190	NM_001013615	chr1:46441593-46459515	template	-3	upstream	Yes	--	--	--	--	--	--	Yes	Yes	Yes	--
G4ST01046441722	chr1:46441723-46441763	+	GGGATGTGGAGGGTAAGGTGGGCAGGAAGACCCCTGAAGGG<br>((((......))))..	POMGNT1	NR_024332	chr1:46426940-46458564	template	41	Intron_1.f	--	Yes	--	Yes	--	--	--	--	Yes	Yes	--
G4ST01046441722	chr1:46441723-46441763	+	GGGATGTGGAGGGTAAGGTGGGCAGGAAGACCCCTGAAGGG<br>((((......))))..	C1orf190	NM_001013615	chr1:46441593-46459515	non-template	41	Exon_1.f	--	Yes	Yes	--	Yes	--	--	--	Yes	Yes	--

G4ST01046485713	chr1:46485714-46485750	-	GGGGGAGGGGCGGGGCCAACAGCGAGGAGCAGTTGGG<br>...(((.((.....)).)))	RAD54L	NM_003579	chr1:46485954-46516732	template	-203	upstream	Yes	--	--	--	--	--	--	Yes	Yes	Yes	Yes
G4ST01046485713	chr1:46485714-46485750	-	GGGGGAGGGGCGGGGCCAACAGCGAGGAGCAGTTGGG<br>...(((.((.....)).)))	RAD54L	NM_001142548	chr1:46485954-46516732	template	-203	upstream	Yes	--	--	--	--	--	--	Yes	Yes	Yes	Yes
G4ST01046540401	chr1:46540402-46540443	-	GGGGGCCCCAGGCCTGAAGGCGGCCGGGACCAAAGGGCCGGG<br>((((....(((....)))))))	UQCRH	NM_006004	chr1:46541967-46555034	template	-1523	upstream	Yes	--	--	--	--	--	--	Yes	Yes	Yes	--
G4ST01046540401	chr1:46540402-46540443	-	GGGGGCCCCAGGCCTGAAGGCGGCCGGGACCAAAGGGCCGGG<br>((((....(((....)))))))	LRRC41	NM_006369	chr1:46516659-46541625	non-template	42	Intron_1.f	--	Yes	--	Yes	--	--	--	Yes	Yes	Yes	--
G4ST01046540406	chr1:46540407-46540453	-	GGGAGTGAACGGGGGCCCCAGGCCTGAAGGCGGCCGGGACCAAAGGG<br>((((....(((....)))))))	UQCRH	NM_006004	chr1:46541967-46555034	template	-1513	upstream	Yes	--	--	--	--	--	--	Yes	Yes	Yes	--
G4ST01046540406	chr1:46540407-46540453	-	GGGAGTGAACGGGGGCCCCAGGCCTGAAGGCGGCCGGGACCAAAGGG<br>((((....(((....)))))))	LRRC41	NM_006369	chr1:46516659-46541625	non-template	47	Intron_1.f	--	Yes	--	Yes	--	--	--	Yes	Yes	Yes	--
G4ST01046540415	chr1:46540416-46540462	-	GGGCTGAATGGGAGTGAACGGGGGCCCCAGGCCTGAAGGCGGCCGGG<br>((((....(((....)))))))	UQCRH	NM_006004	chr1:46541967-46555034	template	-1504	upstream	Yes	--	--	--	--	--	--	Yes	Yes	Yes	--
G4ST01046540415	chr1:46540416-46540462	-	GGGCTGAATGGGAGTGAACGGGGGCCCCAGGCCTGAAGGCGGCCGGG<br>((((....(((....)))))))	LRRC41	NM_006369	chr1:46516659-46541625	non-template	47	Intron_1.f	--	Yes	--	Yes	--	--	--	Yes	Yes	Yes	--
G4ST01046541273	chr1:46541274-46541316	-	GGGGGGCGAGGCCGGGAGGCCCGGGGCAAGGCCCAGCCTGGGG<br>...((((...)))).	UQCRH	NM_006004	chr1:46541967-46555034	template	-650	upstream	Yes	--	--	--	--	--	--	--	Yes	Yes	Yes
G4ST01046541273	chr1:46541274-46541316	-	GGGGGGCGAGGCCGGGAGGCCCGGGGCAAGGCCCAGCCTGGGG<br>...((((...)))).	LRRC41	NM_006369	chr1:46516659-46541625	non-template	43	Intron_1.f	--	Yes	--	Yes	--	--	--	--	Yes	Yes	Yes
G4ST01046541300	chr1:46541301-46541342	-	GGGGCGGCCCAGCGGCCGGGGGGCGCGGGGGGCGAGGCCGGG<br>.(((((....)))))..	UQCRH	NM_006004	chr1:46541967-46555034	template	-624	upstream	Yes	--	--	--	--	--	--	--	Yes	Yes	Yes
G4ST01046541300	chr1:46541301-46541342	-	GGGGCGGCCCAGCGGCCGGGGGGCGCGGGGGGCGAGGCCGGG<br>.(((((....)))))..	LRRC41	NM_006369	chr1:46516659-46541625	non-template	42	Intron_1.f	--	Yes	--	Yes	--	--	--	--	Yes	Yes	Yes
G4ST01046638444	chr1:46638445-46638474	+	GGGGGGTGGGAGGGGGCAGTGGTGCCTGGG<br>((((....)))).	FAAH	NM_001441	chr1:46632526-46652107	non-template	30	Intron_1.f	--	Yes	--	Yes	--	--	--	--	--	--	Yes
G4ST01046638451	chr1:46638452-46638479	+	GGGAGGGGGCAGTGGTGCCTGGGTGGGG<br>((((....)))).	FAAH	NM_001441	chr1:46632526-46652107	non-template	28	Intron_1.f	--	Yes	--	Yes	--	--	--	--	--	--	Yes
G4ST01046648776	chr1:46648777-46648807	+	GGGGCTAGGATGGCTGGGGGGGAACCTAGGG<br>(((.....))).	FAAH	NM_001441	chr1:46632526-46652107	non-template	31	Intron_10	--	Yes	--	Yes	--	--	--	--	--	--	--
G4ST01046800915	chr1:46800916-46800955	-	GGGGCCGACTGTGGCTGGGACCGGGGCGAGGTCTGCAGGG<br>((((....)))). (((.....))).	MKNK1	NR_024176	chr1:46795677-46842553	non-template	40	Exon_10	--	Yes	Yes	--	--	--	--	--	--	--	--
G4ST01046800915	chr1:46800916-46800955	-	GGGGCCGACTGTGGCTGGGACCGGGGCGAGGTCTGCAGGG<br>((((....)))). (((.....))).	MKNK1	NR_024174	chr1:46795677-46842553	non-template	40	Exon_9	--	Yes	Yes	--	--	--	--	--	--	--	--
G4ST01046800915	chr1:46800916-46800955	-	GGGGCCGACTGTGGCTGGGACCGGGGCGAGGTCTGCAGGG<br>((((....)))). (((.....))).	MKNK1	NM_001135553	chr1:46795677-46842553	non-template	40	Exon_10	--	Yes	Yes	--	Yes	--	--	--	--	--	--
G4ST01046800915	chr1:46800916-46800955	-	GGGGCCGACTGTGGCTGGGACCGGGGCGAGGTCTGCAGGG<br>((((....)))). (((.....))).	MKNK1	NM_003684	chr1:46795677-46842553	non-template	40	Exon_11	--	Yes	Yes	--	Yes	--	--	--	--	--	--
G4ST01046800915	chr1:46800916-46800955	-	GGGGCCGACTGTGGCTGGGACCGGGGCGAGGTCTGCAGGG<br>((((....)))). (((.....))).	MKNK1	NM_198973	chr1:46795677-46842553	non-template	40	Exon_10	--	Yes	Yes	--	Yes	--	--	--	--	--	--
G4ST01046800929	chr1:46800930-46800965	-	GGGTCACTGCGGGGCCGACTGTGGCTGGGACCGGGG<br>((((....)))).	MKNK1	NR_024176	chr1:46795677-46842553	non-template	36	Exon_10	--	Yes	Yes	--	--	--	--	--	--	--	--
G4ST01046800929	chr1:46800930-46800965	-	GGGTCACTGCGGGGCCGACTGTGGCTGGGACCGGGG<br>((((....)))).	MKNK1	NR_024174	chr1:46795677-46842553	non-template	36	Exon_9	--	Yes	Yes	--	--	--	--	--	--	--	--
G4ST01046800929	chr1:46800930-46800965	-	GGGTCACTGCGGGGCCGACTGTGGCTGGGACCGGGG<br>((((....)))).	MKNK1	NM_001135553	chr1:46795677-46842553	non-template	36	Exon_10	--	Yes	Yes	--	Yes	--	--	--	--	--	--
G4ST01046800929	chr1:46800930-46800965	-	GGGTCACTGCGGGGCCGACTGTGGCTGGGACCGGGG<br>((((....)))).	MKNK1	NM_003684	chr1:46795677-46842553	non-template	36	Exon_11	--	Yes	Yes	--	Yes	--	--	--	--	--	--
G4ST01046800929	chr1:46800930-46800965	-	GGGTCACTGCGGGGCCGACTGTGGCTGGGACCGGGG<br>((((....)))).	MKNK1	NM_198973	chr1:46795677-46842553	non-template	36	Exon_10	--	Yes	Yes	--	Yes	--	--	--	--	--	--
G4ST01046833645	chr1:46833646-46833677	-	GGGTGGGGGTTGGGCACAAGTCCCACTTGGGG<br>..(((((...)))))	MKNK1	NR_024176	chr1:46795677-46842553	non-template	32	Intron_1.f	--	Yes	--	Yes	--	--	--	Yes	Yes	--	--
G4ST01046833645	chr1:46833646-46833677	-	GGGTGGGGGTTGGGCACAAGTCCCACTTGGGG<br>..(((((...)))))	MKNK1	NR_024174	chr1:46795677-46842553	non-template	32	Intron_1.f	--	Yes	--	Yes	--	--	--	Yes	Yes	--	--
G4ST01046833645	chr1:46833646-46833677	-	GGGTGGGGGTTGGGCACAAGTCCCACTTGGGG<br>..(((((...)))))	MKNK1	NM_001135553	chr1:46795677-46842553	non-template	32	Intron_1.f	--	Yes	--	Yes	--	--	--	Yes	Yes	--	--
G4ST01046833645	chr1:46833646-46833677	-	GGGTGGGGGTTGGGCACAAGTCCCACTTGGGG<br>..(((((...)))))	MKNK1	NM_003684	chr1:46795677-46842553	non-template	32	Intron_1.f	--	Yes	--	Yes	--	--	--	Yes	Yes	--	--
G4ST01046833645	chr1:46833646-46833677	-	GGGTGGGGGTTGGGCACAAGTCCCACTTGGGG<br>..(((((...)))))	MKNK1	NM_198973	chr1:46795677-46842553	non-template	32	Intron_1.f	--	Yes	--	Yes	--	--	--	Yes	Yes	--	--
G4ST01046852051	chr1:46852052-46852092	+	GGGTGGAGGGGGGTCGGGGGTGGCTTTCGGCTTCCTCTGGG<br>((.(((.....))).))...	MOBKL2C	NM_145279	chr1:46845974-46853392	template	41	Intron_1.f	--	Yes	--	Yes	--	--	--	--	Yes	--	Yes
G4ST01046852051	chr1:46852052-46852092	+	GGGTGGAGGGGGGTCGGGGGTGGCTTTCGGCTTCCTCTGGG<br>((.(((.....))).))...	MOBKL2C	NM_201403	chr1:46845974-46855150	template	41	Intron_1.f	--	Yes	--	Yes	--	--	--	--	Yes	--	Yes
G4ST01046852995	chr1:46852996-46853024	+	GGGAGGGTGGGAACCAGGTGACACCTGGG<br>....((((...))))	MOBKL2C	NM_145279	chr1:46845974-46853392	template	29	Intron_1.f	--	Yes	--	Yes	--	--	--	--	--	--	Yes
G4ST01046852995	chr1:46852996-46853024	+	GGGAGGGTGGGAACCAGGTGACACCTGGG<br>....((((...))))	MOBKL2C	NM_201403	chr1:46845974-46855150	template	29	Intron_1.f	--	Yes	--	Yes	--	--	--	--	--	--	Yes
G4ST01046854316	chr1:46854317-46854357	-	GGGAAATGGGGGCTAGGACGGGGAACGGCAGTGGTTCAGGG<br>((((.......)))).	MOBKL2C	NM_145279	chr1:46845974-46853392	non-template	-924	upstream	Yes	--	--	--	--	--	--	--	Yes	--	--
G4ST01046854316	chr1:46854317-46854357	-	GGGAAATGGGGGCTAGGACGGGGAACGGCAGTGGTTCAGGG<br>((((.......)))).	MOBKL2C	NM_201403	chr1:46845974-46855150	non-template	41	Intron_1.f	--	Yes	--	Yes	--	--	--	--	Yes	--	--
G4ST01046885088	chr1:46885089-46885126	-	GGGACAGTAAGGGACAGGGAAGTATGAACTTCAAAGGG<br>((((....))))....	ATPAF1	NM_001042546	chr1:46870998-46906686	non-template	38	Intron_6.l	--	Yes	--	Yes	--	--	--	--	Yes	--	--
G4ST01046885088	chr1:46885089-46885126	-	GGGACAGTAAGGGACAGGGAAGTATGAACTTCAAAGGG<br>((((....))))....	ATPAF1	NM_022745	chr1:46870998-46906686	non-template	38	Intron_6	--	Yes	--	Yes	--	--	--	--	Yes	--	--
G4ST01046952939	chr1:46952940-46952969	+	GGGATTGGGGGTGTGGGGTGCTGACACGGG<br>(((....)))	KIAA0494	NM_014774	chr1:46913418-46957323	template	30	Intron_2	--	Yes	--	Yes	--	--	--	--	--	--	--
G4ST01047425990	chr1:47425991-47426034	-	GGGGCTGCATGTGACCCACCTGCGGGAGGGAAAGAGGCTCTGGG<br>...(((.(((...))).))) ..(((...)))	PDZK1IP1	NM_005764	chr1:47421848-47428358	non-template	44	Intron_1.f	--	Yes	--	Yes	--	--	--	Yes	Yes	--	--
G4ST01047427822	chr1:47427823-47427858	-	GGGATGGCTGGGTCCTGAGTACAGGGGACCCGGGGG<br>..(((....)))	PDZK1IP1	NM_005764	chr1:47421848-47428358	non-template	36	Intron_1.f	--	Yes	--	Yes	--	--	--	--	--	--	--
G4ST01047428834	chr1:47428835-47428871	-	GGGGGTGGATTTAGGAAGCTACTGGGCTGGGCCAGGG<br>.((((.((....)).)))).	PDZK1IP1	NM_005764	chr1:47421848-47428358	non-template	-476	upstream	Yes	--	--	--	--	--	--	--	Yes	--	--
G4ST01047457703	chr1:47457704-47457732	+	GGGAAGGATTTGGGACTGAGGGAAGAGGG<br>((...)).	TAL1	NM_003189	chr1:47454550-47468030	template	29	Exon_4.l	--	Yes	Yes	--	--	--	Yes	--	--	--	--
G4ST01047572655	chr1:47572656-47572693	+	GGGCTGGGAGAGGGATCCCGGAGGCCAACCTTTGGGGG<br>...(((((((....)))))))	CMPK1	NM_001136140	chr1:47572056-47617098	non-template	38	Intron_1.f	--	Yes	--	Yes	--	--	--	Yes	Yes	Yes	--
G4ST01047572655	chr1:47572656-47572693	+	GGGCTGGGAGAGGGATCCCGGAGGCCAACCTTTGGGGG<br>...(((((((....)))))))	CMPK1	NM_016308	chr1:47572056-47617098	non-template	38	Intron_1.f	--	Yes	--	Yes	--	--	--	Yes	Yes	Yes	--
G4ST01047655387	chr1:47655388-47655413	-	GGGCCGGGGCCGGGGCGCGTCGCGGG<br>(((...)))	FOXE3	NM_012186	chr1:47654331-47656311	template	26	Exon_1.u	--	Yes	Yes	--	Yes	--	--	--	--	--	Yes
G4ST01047655571	chr1:47655572-47655617	-	GGGGGGTAGAGATGGGGCGCTCGAGGGCGGCGCCGCAGGTCCGGGG<br>(((..((...)).)))	FOXE3	NM_012186	chr1:47654331-47656311	template	46	Exon_1.u	--	Yes	Yes	--	--	--	Yes	--	--	--	Yes
G4ST01047675496	chr1:47675497-47675531	+	GGGGTGGCGGGCCCGACAGCGGAGGGAGGAGAGGG<br>.(((....))).	FOXD2	NM_004474	chr1:47674276-47678950	non-template	35	Exon_1.u	--	Yes	Yes	--	--	Yes	--	Yes	Yes	Yes	--
G4ST01047676284	chr1:47676285-47676320	-	GGGGCGAGTGCGTTTTGCGCGCGGGCGGGGAGGGGG<br>....(((((.....)))))	FOXD2	NM_004474	chr1:47674276-47678950	template	36	Exon_1.u	--	Yes	Yes	--	--	Yes	--	Yes	Yes	Yes	Yes
G4ST01047676717	chr1:47676718-47676747	+	GGGGCCCGGGCCGGGACCGCCGTCGGGGGG<br>.(((....))).	FOXD2	NM_004474	chr1:47674276-47678950	non-template	30	Exon_1.u	--	Yes	Yes	--	Yes	--	--	Yes	Yes	--	--

G4ST01047678715	chr1:47678716-47678754	+	GGGGGTGGAGTGGGCAAGGGCACACCCTGTGGCAGGGGG<br>.....((((...))))	FOXD2	NM_004474	chr1:47674276-47678950	non-template	39	Exon_1.u	--	Yes	Yes	--	--	--	Yes	--	--	--	--
G4ST01048003272	chr1:48003273-48003303	-	GGGCCGAGGGACTGGAGTGGGCACAGGAGGG<br>((....))	LOC388630	NM_001194986	chr1:47998787-48235149	non-template	31	Exon_7.l	--	Yes	Yes	--	--	--	Yes	--	--	--	--
G4ST01048004298	chr1:48004299-48004340	+	GGGGGGCTTGGCGGTCCCCGAGCTGTGGGTGGGCTGCAGGGG<br>..((((((......))))))...	LOC388630	NM_001194986	chr1:47998787-48235149	template	42	Exon_7.l	--	Yes	Yes	--	Yes	--	--	--	--	--	Yes
G4ST01048008442	chr1:48008443-48008485	+	GGGGCACACAGGGCCTGGGTCACAGGTGGCCCTCTGCCTGGGG<br>...(((((((....)))))))	LOC388630	NM_001194986	chr1:47998787-48235149	template	43	Intron_6.l	--	Yes	--	Yes	--	--	--	--	--	--	--
G4ST01048013595	chr1:48013596-48013636	+	GGGGGTCACAGAGGGTGCTTCGGGGACAGCGGCAGCTGGGG<br>..((((....))))	LOC388630	NM_001194986	chr1:47998787-48235149	template	41	Exon_6	--	Yes	Yes	--	Yes	--	--	--	--	--	Yes
G4ST01048013607	chr1:48013608-48013645	+	GGGTGCTTCGGGGACAGCGGCAGCTGGGGTCACTGGGG<br>..((((....))))	LOC388630	NM_001194986	chr1:47998787-48235149	template	38	Exon_6	--	Yes	Yes	--	Yes	--	--	--	--	--	Yes
G4ST01048013616	chr1:48013617-48013650	+	GGGGACAGCGGCAGCTGGGGTCACTGGGGCCGGG<br>..((((....))))	LOC388630	NM_001194986	chr1:47998787-48235149	template	34	Exon_6	--	Yes	Yes	--	Yes	--	--	--	--	--	Yes
G4ST01048015443	chr1:48015444-48015475	+	GGGGAAGGTCCTTGGGGCTGCTGGGGTGAGGG<br>.(((...))).	LOC388630	NM_001194986	chr1:47998787-48235149	template	32	Intron_5	--	Yes	--	Yes	--	--	--	--	--	--	Yes
G4ST01048015537	chr1:48015538-48015573	-	GGGGGCCAGGGGCAAGCATGTGTTTGAGGGTTGGGG<br>.(((((....))))).	LOC388630	NM_001194986	chr1:47998787-48235149	non-template	36	Intron_5	--	Yes	--	Yes	--	--	--	--	--	--	--
G4ST01048047264	chr1:48047265-48047313	-	GGGCAGGGAGCAAGGTTTCGTCAGCCTGGGCAGTGAAAGCAGACTGGGG<br>....(((((.....))))) ((((........))))	LOC388630	NM_001194986	chr1:47998787-48235149	non-template	49	Intron_2	--	Yes	--	Yes	--	--	--	--	--	--	--
G4ST01048047283	chr1:48047284-48047322	-	GGGGATCCTGGGCAGGGAGCAAGGTTTCGTCAGCCTGGG<br>....(((((.....)))))	LOC388630	NM_001194986	chr1:47998787-48235149	non-template	39	Intron_2	--	Yes	--	Yes	--	--	--	--	--	--	--
G4ST01048068406	chr1:48068407-48068440	-	GGGAGGTCCCATCTGGGAGGGGGACATCCTGGGG<br>(((.....)))	LOC388630	NM_001194986	chr1:47998787-48235149	non-template	34	Intron_2	--	Yes	--	Yes	--	--	--	--	--	--	--
G4ST01048069277	chr1:48069278-48069314	-	GGGCACTCAAGGGCTGGGGGAATGTGGCTCAGTCGGG<br>.((.((.....)).))	LOC388630	NM_001194986	chr1:47998787-48235149	non-template	37	Intron_2	--	Yes	--	Yes	--	--	--	Yes	--	--	--
G4ST01048071066	chr1:48071067-48071100	+	GGGGCTCTCAGAGTGGGGCAGGGGGAGGCACGGG<br>.(((...)))..	LOC388630	NM_001194986	chr1:47998787-48235149	template	34	Intron_2	--	Yes	--	Yes	--	--	--	--	--	--	--
G4ST01048089506	chr1:48089507-48089533	-	GGGGTGGGCCATGGAGCATGGGGTGGG<br>.((((...))))	LOC388630	NM_001194986	chr1:47998787-48235149	non-template	27	Intron_2	--	Yes	--	Yes	--	--	--	--	--	--	--
G4ST01048089510	chr1:48089511-48089538	-	GGGCTGGGGTGGGCCATGGAGCATGGGG<br>.((((...))))	LOC388630	NM_001194986	chr1:47998787-48235149	non-template	28	Intron_2	--	Yes	--	Yes	--	--	--	--	--	--	--
G4ST01048150918	chr1:48150919-48150952	+	GGGAGGGCTTAGAGAGGGCAGCCCAGTGCTGGGG<br>((...)). ((((.....))))	LOC388630	NM_001194986	chr1:47998787-48235149	template	34	Intron_2	--	Yes	--	Yes	--	--	--	--	Yes	--	--
G4ST01048150922	chr1:48150923-48150962	+	GGGCTTAGAGAGGGCAGCCCAGTGCTGGGGAGACACTGGG<br>((...)). ((((.....))))	LOC388630	NM_001194986	chr1:47998787-48235149	template	40	Intron_2	--	Yes	--	Yes	--	--	--	--	Yes	--	--
G4ST01048154107	chr1:48154108-48154144	-	GGGGTCACCGCCTGGTGGGGGTAGGGGAGCGTGGGGG<br>..((((....)))).	LOC388630	NM_001194986	chr1:47998787-48235149	non-template	37	Intron_2	--	Yes	--	Yes	--	--	--	Yes	--	--	Yes
G4ST01048210078	chr1:48210079-48210111	-	GGGAGGTTTCATCTGGGGTGAGAGTGGGAGGGG<br>(((.....))).	LOC388630	NM_001194986	chr1:47998787-48235149	non-template	33	Intron_2	--	Yes	--	Yes	--	--	--	Yes	--	--	Yes
G4ST01048235055	chr1:48235056-48235086	+	GGGCCCGCGGGGCGCGGGGGACCCTCCTGGG<br>(((...))).	LOC388630	NM_001194986	chr1:47998974-48235149	template	31	Exon_1.f	--	Yes	Yes	--	--	Yes	--	--	Yes	--	--
G4ST01048341639	chr1:48341640-48341682	+	GGGGATACAGGGATGAAATAGGGACAGTGCCCATGCCTGAGGG<br>((...)). .(((.((....))))).	SKINTL	NR_026749	chr1:48339974-48420687	template	43	Intron_9.l	--	Yes	--	Yes	--	--	--	--	--	--	--
G4ST01048388532	chr1:48388533-48388586	+	GGGTGTGCGCACCGTGCGCGAGGGGAAGCAGGGCGAGGCATTGCCTCACCTGGG<br>..((((((...)))))).. .(((((...)))))....	SKINTL	NR_026749	chr1:48339974-48420687	template	54	Intron_7	--	Yes	--	Yes	--	--	--	--	--	--	--
G4ST01048459766	chr1:48459767-48459797	+	GGGTGTGGGACAGGGCCAGTGAGAGCTGGGG<br>.((((....))))	SLC5A9	NM_001135181	chr1:48460944-48486903	non-template	-1146	upstream	Yes	--	--	--	--	--	--	--	--	--	Yes
G4ST01048459766	chr1:48459767-48459797	+	GGGTGTGGGACAGGGCCAGTGAGAGCTGGGG<br>.((((....))))	SLC5A9	NM_001011547	chr1:48460944-48486903	non-template	-1146	upstream	Yes	--	--	--	--	--	--	--	--	--	Yes
G4ST01048459772	chr1:48459773-48459803	+	GGGACAGGGCCAGTGAGAGCTGGGGATGGGG<br>.((((....))))	SLC5A9	NM_001135181	chr1:48460944-48486903	non-template	-1140	upstream	Yes	--	--	--	--	--	--	--	--	--	Yes
G4ST01048459772	chr1:48459773-48459803	+	GGGACAGGGCCAGTGAGAGCTGGGGATGGGG<br>.((((....))))	SLC5A9	NM_001011547	chr1:48460944-48486903	non-template	-1140	upstream	Yes	--	--	--	--	--	--	--	--	--	Yes
G4ST01048459778	chr1:48459779-48459809	+	GGGCCAGTGAGAGCTGGGGATGGGGTGTGGG<br>.((((....))))	SLC5A9	NM_001135181	chr1:48460944-48486903	non-template	-1134	upstream	Yes	--	--	--	--	--	--	--	--	--	Yes
G4ST01048459778	chr1:48459779-48459809	+	GGGCCAGTGAGAGCTGGGGATGGGGTGTGGG<br>.((((....))))	SLC5A9	NM_001011547	chr1:48460944-48486903	non-template	-1134	upstream	Yes	--	--	--	--	--	--	--	--	--	Yes
G4ST01048461213	chr1:48461214-48461240	+	GGGTGGGGCTGTGGAGCTGGGGCAGGG<br>(((....)))..	SLC5A9	NM_001135181	chr1:48460944-48486903	non-template	27	Intron_1.f	--	Yes	--	Yes	--	--	--	--	Yes	Yes	--
G4ST01048461213	chr1:48461214-48461240	+	GGGTGGGGCTGTGGAGCTGGGGCAGGG<br>(((....)))..	SLC5A9	NM_001011547	chr1:48460944-48486903	non-template	27	Intron_1.f	--	Yes	--	Yes	--	--	--	--	Yes	Yes	--
G4ST01048462575	chr1:48462576-48462602	+	GGGAGCTCAGCTGGGAGCAGGGAGGGG<br>(((...)))	SLC5A9	NM_001135181	chr1:48460944-48486903	non-template	27	Intron_1.f	--	Yes	--	Yes	--	--	--	--	--	--	--
G4ST01048462575	chr1:48462576-48462602	+	GGGAGCTCAGCTGGGAGCAGGGAGGGG<br>(((...)))	SLC5A9	NM_001011547	chr1:48460944-48486903	non-template	27	Intron_1.f	--	Yes	--	Yes	--	--	--	--	--	--	--
G4ST01048466211	chr1:48466212-48466251	-	GGGAGGAGGCTTAAGGAGACCTCGGGGAGAGGGCTGGGGG<br>..((((((.....)).)))).	SLC5A9	NM_001135181	chr1:48460944-48486903	template	40	Intron_2	--	Yes	--	Yes	--	--	--	--	Yes	--	Yes
G4ST01048466211	chr1:48466212-48466251	-	GGGAGGAGGCTTAAGGAGACCTCGGGGAGAGGGCTGGGGG<br>..((((((.....)).)))).	SLC5A9	NM_001011547	chr1:48460944-48486903	template	40	Intron_2	--	Yes	--	Yes	--	--	--	--	Yes	--	Yes
G4ST01048466218	chr1:48466219-48466266	-	GGGGCTCTGGAGCCTGGGAGGAGGCTTAAGGAGACCTCGGGGAGAGGG<br>(((....))).. ..((((((.....)).)))).	SLC5A9	NM_001135181	chr1:48460944-48486903	template	48	Intron_2	--	Yes	--	Yes	--	--	--	--	Yes	--	Yes
G4ST01048466218	chr1:48466219-48466266	-	GGGGCTCTGGAGCCTGGGAGGAGGCTTAAGGAGACCTCGGGGAGAGGG<br>(((....))).. ..((((((.....)).)))).	SLC5A9	NM_001011547	chr1:48460944-48486903	template	48	Intron_2	--	Yes	--	Yes	--	--	--	--	Yes	--	Yes
G4ST01048466224	chr1:48466225-48466278	-	GGGGGAGAGGAAGGGGCTCTGGAGCCTGGGAGGAGGCTTAAGGAGACCTCGGGG<br>(((....))).. ..((((((.....)).)))).	SLC5A9	NM_001135181	chr1:48460944-48486903	template	54	Intron_2	--	Yes	--	Yes	--	--	--	--	Yes	--	Yes
G4ST01048466224	chr1:48466225-48466278	-	GGGGGAGAGGAAGGGGCTCTGGAGCCTGGGAGGAGGCTTAAGGAGACCTCGGGG<br>(((....))).. ..((((((.....)).)))).	SLC5A9	NM_001011547	chr1:48460944-48486903	template	54	Intron_2	--	Yes	--	Yes	--	--	--	--	Yes	--	Yes
G4ST01048466248	chr1:48466249-48466286	-	GGGGAGAAGGGGGAGAGGAAGGGGCTCTGGAGCCTGGG<br>(((....)))..	SLC5A9	NM_001135181	chr1:48460944-48486903	template	38	Intron_2	--	Yes	--	Yes	--	--	--	--	--	--	Yes
G4ST01048466248	chr1:48466249-48466286	-	GGGGAGAAGGGGGAGAGGAAGGGGCTCTGGAGCCTGGG<br>(((....)))..	SLC5A9	NM_001011547	chr1:48460944-48486903	template	38	Intron_2	--	Yes	--	Yes	--	--	--	--	--	--	Yes
G4ST01048647107	chr1:48647108-48647144	-	GGGACCTGGTGGGAGATGATTGAATCATGGGGATGGG<br>..((((.....)))).	SPATA6	NM_019073	chr1:48533631-48710463	non-template	37	Intron_5	--	Yes	--	Yes	--	--	--	--	--	--	--
G4ST01048647112	chr1:48647113-48647148	-	GGGAGGGACCTGGTGGGAGATGATTGAATCATGGGG<br>..((((.....)))).	SPATA6	NM_019073	chr1:48533631-48710463	non-template	36	Intron_5	--	Yes	--	Yes	--	--	--	--	--	--	--
G4ST01048860587	chr1:48860588-48860618	-	GGGCTCCGGGTACCATGTGGGTAAAGTAGGG<br>.((...))	AGBL4	NM_032785	chr1:48771114-50262213	non-template	31	Intron_9	--	Yes	--	Yes	--	--	--	--	--	--	--
G4ST01048913504	chr1:48913505-48913529	-	GGGAAGAGATTGGGCTCAGGGTGGG<br>((....))	AGBL4	NM_032785	chr1:48771114-50262213	non-template	25	Intron_6	--	Yes	--	Yes	--	--	--	--	--	--	Yes
G4ST01048913508	chr1:48913509-48913538	-	GGGGAGAGAGGGAAGAGATTGGGCTCAGGG<br>((....))	AGBL4	NM_032785	chr1:48771114-50262213	non-template	30	Intron_6	--	Yes	--	Yes	--	--	--	--	--	--	Yes
G4ST01048913515	chr1:48913516-48913542	-	GGGAGGGGAGAGAGGGAAGAGATTGGG<br>((....))	AGBL4	NM_032785	chr1:48771114-50262213	non-template	27	Intron_6	--	Yes	--	Yes	--	--	--	--	--	--	Yes
G4ST01048946572	chr1:48946573-48946605	+	GGGGCTGGAAGGGTTGTGTCAGGGACTGCAGGG<br>.((...))	AGBL4	NM_032785	chr1:48771114-50262213	template	33	Intron_6	--	Yes	--	Yes	--	--	--	--	--	--	--
G4ST01048950380	chr1:48950381-48950412	-	GGGAATGGGCCCCTTCCTGGGGGGAGAAGGGG<br>.(((.....)))	AGBL4	NM_032785	chr1:48771114-50262213	non-template	32	Intron_6	--	Yes	--	Yes	--	--	--	--	--	--	--

G4ST01048960393	chr1:48960394-48960419	+	GGGTGGGTGGGCATTGGTGAATAGGG<br>.(((....))).	AGBL4	NM_032785	chr1:48771114-50262213	template	26	Intron_6	--	Yes	--	Yes	--	--	--	--	--	--	Yes
G4ST01048960397	chr1:48960398-48960423	+	GGGTGGGCATTGGTGAATAGGGAGGG<br>.(((....))).	AGBL4	NM_032785	chr1:48771114-50262213	template	26	Intron_6	--	Yes	--	Yes	--	--	--	--	--	--	Yes
G4ST01048960401	chr1:48960402-48960432	+	GGGCATTGGTGAATAGGGAGGGACTGGGGGG<br>.(((....))).	AGBL4	NM_032785	chr1:48771114-50262213	template	31	Intron_6	--	Yes	--	Yes	--	--	--	--	--	--	Yes
G4ST01049010521	chr1:49010522-49010560	+	GGGAGAGAGTCTCTCTTGGGCGCAGAGGGAGGAATAGGG<br>(((((...))))).	BEND5	NM_024603	chr1:48966127-49015134	template	39	Intron_1.f	--	Yes	--	Yes	--	--	--	--	--	--	--
G4ST01049010521	chr1:49010522-49010560	+	GGGAGAGAGTCTCTCTTGGGCGCAGAGGGAGGAATAGGG<br>(((((...))))).	AGBL4	NM_032785	chr1:48771114-50262213	template	39	Intron_6	--	Yes	--	Yes	--	--	--	--	--	--	--
G4ST01049137448	chr1:49137449-49137481	+	GGGCAGGCGGCTCAGTGCCGCGGGGCGGGCGGG<br>...(((((.....))))).	AGBL4	NM_032785	chr1:48771114-50262213	template	33	Intron_5	--	Yes	--	Yes	--	--	--	--	--	--	--
G4ST01049454634	chr1:49454635-49454667	-	GGGTAGGGAAAGGGTTGAAAACTAACTTTTGGG<br>...((((.....))))	AGBL4	NM_032785	chr1:48771114-50262213	non-template	33	Intron_4	--	Yes	--	Yes	--	--	--	--	--	--	Yes
G4ST01049795324	chr1:49795325-49795350	-	GGGAGAGGCTCGGGTGGGAACCAGGG<br>.((...))	AGBL4	NM_032785	chr1:48771114-50262213	non-template	26	Intron_3	--	Yes	--	Yes	--	--	--	--	--	--	--
G4ST01049968677	chr1:49968678-49968729	-	GGGTGAATGGGGGCATGTCATAGTTCTGGCTTGGGGAGTCCCGAGGTCTGGG<br>..((.((((......)))).)) .((.((...)).))	AGBL4	NM_032785	chr1:48771114-50262213	non-template	52	Intron_2	--	Yes	--	Yes	--	--	--	--	--	--	--
G4ST01050047555	chr1:50047556-50047583	-	GGGCCTGTCGTGGGGGGAGGGGGGAGGG<br>(((.....)))	AGBL4	NM_032785	chr1:48771114-50262213	non-template	28	Intron_2	--	Yes	--	Yes	--	--	--	--	--	--	--
G4ST01050068020	chr1:50068021-50068050	-	GGGCCTGTTGAGGGGGTGTGGGCCTAGGGG<br>(((....)))	AGBL4	NM_032785	chr1:48771114-50262213	non-template	30	Intron_2	--	Yes	--	Yes	--	--	--	--	--	--	Yes
G4ST01050288574	chr1:50288575-50288609	+	GGGGAGGGAGATTTGGCTACAAAGGGACAGGAGGG<br>...((((....))))	ELAVL4	NM_001144777	chr1:50286273-50440127	non-template	35	Intron_1.f	--	Yes	--	Yes	--	--	--	--	--	--	--
G4ST01050393583	chr1:50393584-50393610	-	GGGCCAGTGGTGGGAGAGGGTGTGGGG<br>((...)).	ELAVL4	NM_001144775	chr1:50344551-50440127	template	27	Intron_2	--	Yes	--	Yes	--	--	--	--	--	--	--
G4ST01050393583	chr1:50393584-50393610	-	GGGCCAGTGGTGGGAGAGGGTGTGGGG<br>((...)).	ELAVL4	NM_001144774	chr1:50347181-50440127	template	27	Intron_2	--	Yes	--	Yes	--	--	--	--	--	--	--
G4ST01050393583	chr1:50393584-50393610	-	GGGCCAGTGGTGGGAGAGGGTGTGGGG<br>((...)).	ELAVL4	NM_021952	chr1:50347181-50440127	template	27	Intron_2	--	Yes	--	Yes	--	--	--	--	--	--	--
G4ST01050393583	chr1:50393584-50393610	-	GGGCCAGTGGTGGGAGAGGGTGTGGGG<br>((...)).	ELAVL4	NM_001144776	chr1:50342169-50440127	template	27	Intron_2	--	Yes	--	Yes	--	--	--	--	--	--	--
G4ST01050393583	chr1:50393584-50393610	-	GGGCCAGTGGTGGGAGAGGGTGTGGGG<br>((...)).	ELAVL4	NM_001144777	chr1:50286273-50440127	template	27	Intron_2	--	Yes	--	Yes	--	--	--	--	--	--	--
G4ST01050393590	chr1:50393591-50393618	-	GGGCTACAGGGCCAGTGGTGGGAGAGGG<br>((...)).	ELAVL4	NM_001144775	chr1:50344551-50440127	template	28	Intron_2	--	Yes	--	Yes	--	--	--	--	--	--	--
G4ST01050393590	chr1:50393591-50393618	-	GGGCTACAGGGCCAGTGGTGGGAGAGGG<br>((...)).	ELAVL4	NM_001144774	chr1:50347181-50440127	template	28	Intron_2	--	Yes	--	Yes	--	--	--	--	--	--	--
G4ST01050393590	chr1:50393591-50393618	-	GGGCTACAGGGCCAGTGGTGGGAGAGGG<br>((...)).	ELAVL4	NM_021952	chr1:50347181-50440127	template	28	Intron_2	--	Yes	--	Yes	--	--	--	--	--	--	--
G4ST01050393590	chr1:50393591-50393618	-	GGGCTACAGGGCCAGTGGTGGGAGAGGG<br>((...)).	ELAVL4	NM_001144776	chr1:50342169-50440127	template	28	Intron_2	--	Yes	--	Yes	--	--	--	--	--	--	--
G4ST01050393590	chr1:50393591-50393618	-	GGGCTACAGGGCCAGTGGTGGGAGAGGG<br>((...)).	ELAVL4	NM_001144777	chr1:50286273-50440127	template	28	Intron_2	--	Yes	--	Yes	--	--	--	--	--	--	--
G4ST01050703924	chr1:50703925-50703959	+	GGGGTTGTCAGTCTGGCAGGGTGGGGAAGGTAGGG<br>..(((((...)))))	FAF1	NM_007051	chr1:50679523-51198524	template	35	Intron_18.l	--	Yes	--	Yes	--	--	--	--	--	--	Yes
G4ST01051489679	chr1:51489680-51489709	-	GGGGGGGGGCCCGGAGGCCCTGGGATGGGG<br>((((....))))..	RNF11	NM_014372	chr1:51474533-51511707	template	30	Intron_1.f	--	Yes	--	Yes	--	--	--	--	--	--	--
G4ST01051535169	chr1:51535170-51535201	-	GGGGCGTGGTGTGCAGGGAGGACTGGGTGGGG<br>(((.....))).	TTC39A	NM_001080494	chr1:51525518-51560526	non-template	32	Intron_12	--	Yes	--	Yes	--	--	--	Yes	Yes	--	Yes
G4ST01051535169	chr1:51535170-51535201	-	GGGGCGTGGTGTGCAGGGAGGACTGGGTGGGG<br>(((.....))).	TTC39A	NM_001144832	chr1:51525518-51583373	non-template	32	Intron_12	--	Yes	--	Yes	--	--	--	Yes	Yes	--	Yes
G4ST01051535818	chr1:51535819-51535856	-	GGGACTTGGTGTCTCCACGTCAGGGGCAGGGGAAAGGG<br>((.(((.....))).))...	TTC39A	NM_001080494	chr1:51525518-51560526	non-template	38	Intron_12	--	Yes	--	Yes	--	--	--	Yes	Yes	--	--
G4ST01051535818	chr1:51535819-51535856	-	GGGACTTGGTGTCTCCACGTCAGGGGCAGGGGAAAGGG<br>((.(((.....))).))...	TTC39A	NM_001144832	chr1:51525518-51583373	non-template	38	Intron_12	--	Yes	--	Yes	--	--	--	Yes	Yes	--	--
G4ST01051540900	chr1:51540901-51540935	-	GGGGGAGGGGGAGACCCTGGGAAGCCCCTGCTGGG<br>.(((....)))	TTC39A	NM_001080494	chr1:51525518-51560526	non-template	35	Intron_9	--	Yes	--	Yes	--	--	--	--	--	--	--
G4ST01051540900	chr1:51540901-51540935	-	GGGGGAGGGGGAGACCCTGGGAAGCCCCTGCTGGG<br>.(((....)))	TTC39A	NM_001144832	chr1:51525518-51583373	non-template	35	Intron_9	--	Yes	--	Yes	--	--	--	--	--	--	--
G4ST01051541488	chr1:51541489-51541526	+	GGGCAGGGGCCTGGGAGGCAGCTGGACAGCTGTGTGGG<br>...(((((....)))))...	TTC39A	NM_001080494	chr1:51525518-51560526	template	38	Intron_8	--	Yes	--	Yes	--	--	--	--	--	--	--
G4ST01051541488	chr1:51541489-51541526	+	GGGCAGGGGCCTGGGAGGCAGCTGGACAGCTGTGTGGG<br>...(((((....)))))...	TTC39A	NM_001144832	chr1:51525518-51583373	template	38	Intron_8	--	Yes	--	Yes	--	--	--	--	--	--	--
G4ST01051541824	chr1:51541825-51541859	+	GGGCATAGGTGGGGCCTGGGAAGGCTTCCTGGGGG<br>.(((...)))..	TTC39A	NM_001080494	chr1:51525518-51560526	template	35	Intron_8	--	Yes	--	Yes	--	--	--	--	--	--	Yes
G4ST01051541824	chr1:51541825-51541859	+	GGGCATAGGTGGGGCCTGGGAAGGCTTCCTGGGGG<br>.(((...)))..	TTC39A	NM_001144832	chr1:51525518-51583373	template	35	Intron_8	--	Yes	--	Yes	--	--	--	--	--	--	Yes
G4ST01051556753	chr1:51556754-51556786	-	GGGTCAGTGGCCCAGCTTCTGGGGTGGGGTGGG<br>.(((.(((...))).)))	TTC39A	NM_001080494	chr1:51525518-51560526	non-template	33	Intron_2	--	Yes	--	Yes	--	--	--	--	--	--	Yes
G4ST01051556753	chr1:51556754-51556786	-	GGGTCAGTGGCCCAGCTTCTGGGGTGGGGTGGG<br>.(((.(((...))).)))	TTC39A	NM_001144832	chr1:51525518-51583373	non-template	33	Intron_2	--	Yes	--	Yes	--	--	--	--	--	--	Yes
G4ST01051567716	chr1:51567717-51567750	-	GGGTTGCTGGGGGACACCCTGGCCTGGGAGAGGG<br>((.((...)).)).	TTC39A	NM_001144832	chr1:51525518-51583373	non-template	34	Intron_1.f	--	Yes	--	Yes	--	--	--	Yes	--	--	--
G4ST01051573152	chr1:51573153-51573192	-	GGGCCAGAGCTCCTGGGGGCCAGGAACGGCCTGGGTGGGG<br>((((.....)))) ((((......)))).	TTC39A	NM_001144832	chr1:51525518-51583373	non-template	40	Intron_1.f	--	Yes	--	Yes	--	--	--	Yes	Yes	--	--
G4ST01051573157	chr1:51573158-51573198	-	GGGGCTGGGCCAGAGCTCCTGGGGGCCAGGAACGGCCTGGG<br>((((.....)))) ((((......)))).	TTC39A	NM_001144832	chr1:51525518-51583373	non-template	41	Intron_1.f	--	Yes	--	Yes	--	--	--	Yes	Yes	--	--
G4ST01051757561	chr1:51757562-51757595	+	GGGAGCGGCCGCAGGCGAGGCCGGGGCGGGCGGG<br>..(((((........)))))	EPS15	NM_001981	chr1:51592523-51757583	template	22	upstream-Exon_1.f	Yes	Yes	Yes	--	--	Yes	--	Yes	Yes	Yes	Yes
G4ST01051757604	chr1:51757605-51757637	-	GGGCGTGGGGAGGGAAGTTGTGGAAGTTAGGGG<br>((.((....)).))..	EPS15	NM_001981	chr1:51592523-51757583	non-template	-21	upstream	Yes	--	--	--	--	--	--	Yes	Yes	Yes	Yes
G4ST01051967864	chr1:51967865-51967890	-	GGGAGGGGTGCGCAGCGGCGGGAGGG<br>.(((...)))..	OSBPL9	NM_148905	chr1:51968074-52027479	template	-183	upstream	Yes	--	--	--	--	--	--	Yes	Yes	Yes	--
G4ST01051967864	chr1:51967865-51967890	-	GGGAGGGGTGCGCAGCGGCGGGAGGG<br>.(((...)))..	OSBPL9	NM_148907	chr1:51968074-52027479	template	-183	upstream	Yes	--	--	--	--	--	--	Yes	Yes	Yes	--
G4ST01051967864	chr1:51967865-51967890	-	GGGAGGGGTGCGCAGCGGCGGGAGGG<br>.(((...)))..	OSBPL9	NM_148904	chr1:51968074-52027479	template	-183	upstream	Yes	--	--	--	--	--	--	Yes	Yes	Yes	--
G4ST01051967864	chr1:51967865-51967890	-	GGGAGGGGTGCGCAGCGGCGGGAGGG<br>.(((...)))..	OSBPL9	NR_036662	chr1:51855134-52027479	template	26	Intron_3	--	Yes	--	Yes	--	--	--	Yes	Yes	Yes	--
G4ST01051967864	chr1:51967865-51967890	-	GGGAGGGGTGCGCAGCGGCGGGAGGG<br>.(((...)))..	OSBPL9	NM_024586	chr1:51855134-52027479	template	26	Intron_4	--	Yes	--	Yes	--	--	--	Yes	Yes	Yes	--
G4ST01051967864	chr1:51967865-51967890	-	GGGAGGGGTGCGCAGCGGCGGGAGGG<br>.(((...)))..	OSBPL9	NM_148909	chr1:51855134-52027479	template	26	Intron_4	--	Yes	--	Yes	--	--	--	Yes	Yes	Yes	--
G4ST01051967864	chr1:51967865-51967890	-	GGGAGGGGTGCGCAGCGGCGGGAGGG<br>.(((...)))..	OSBPL9	NM_148906	chr1:51855134-52027479	template	26	Intron_3	--	Yes	--	Yes	--	--	--	Yes	Yes	Yes	--
G4ST01051967864	chr1:51967865-51967890	-	GGGAGGGGTGCGCAGCGGCGGGAGGG<br>.(((...)))..	OSBPL9	NM_148908	chr1:51855134-52027479	template	26	Intron_4	--	Yes	--	Yes	--	--	--	Yes	Yes	Yes	--
G4ST01051967868	chr1:51967869-51967907	-	GGGAGCTGGCAGCTCGCGGGAGGGGTGCGCAGCGGCGGG<br>((((...))))... .(((...)))..	OSBPL9	NM_148905	chr1:51968074-52027479	template	-166	upstream	Yes	--	--	--	--	--	--	Yes	Yes	Yes	--

G4ST01051967868	chr1:51967869-51967907	-	GGGAGCTGGCAGCTCGCGGGAGGGGTGCGCAGCGGCGGG<br>((((...))))... .(((...)))..	OSBPL9	NM_148907	chr1:51968074-52027479	template	-166	upstream	Yes	--	--	--	--	--	--	Yes	Yes	Yes	--
G4ST01051967868	chr1:51967869-51967907	-	GGGAGCTGGCAGCTCGCGGGAGGGGTGCGCAGCGGCGGG<br>((((...))))... .(((...)))..	OSBPL9	NM_148904	chr1:51968074-52027479	template	-166	upstream	Yes	--	--	--	--	--	--	Yes	Yes	Yes	--
G4ST01051967868	chr1:51967869-51967907	-	GGGAGCTGGCAGCTCGCGGGAGGGGTGCGCAGCGGCGGG<br>((((...))))... .(((...)))..	OSBPL9	NR_036662	chr1:51855134-52027479	template	39	Intron_3	--	Yes	--	Yes	--	--	--	Yes	Yes	Yes	--
G4ST01051967868	chr1:51967869-51967907	-	GGGAGCTGGCAGCTCGCGGGAGGGGTGCGCAGCGGCGGG<br>((((...))))... .(((...)))..	OSBPL9	NM_024586	chr1:51855134-52027479	template	39	Intron_4	--	Yes	--	Yes	--	--	--	Yes	Yes	Yes	--
G4ST01051967868	chr1:51967869-51967907	-	GGGAGCTGGCAGCTCGCGGGAGGGGTGCGCAGCGGCGGG<br>((((...))))... .(((...)))..	OSBPL9	NM_148909	chr1:51855134-52027479	template	39	Intron_4	--	Yes	--	Yes	--	--	--	Yes	Yes	Yes	--
G4ST01051967868	chr1:51967869-51967907	-	GGGAGCTGGCAGCTCGCGGGAGGGGTGCGCAGCGGCGGG<br>((((...))))... .(((...)))..	OSBPL9	NM_148906	chr1:51855134-52027479	template	39	Intron_3	--	Yes	--	Yes	--	--	--	Yes	Yes	Yes	--
G4ST01051967868	chr1:51967869-51967907	-	GGGAGCTGGCAGCTCGCGGGAGGGGTGCGCAGCGGCGGG<br>((((...))))... .(((...)))..	OSBPL9	NM_148908	chr1:51855134-52027479	template	39	Intron_4	--	Yes	--	Yes	--	--	--	Yes	Yes	Yes	--
G4ST01052158407	chr1:52158408-52158439	-	GGGCACAGAAGGGTTCCAGCAGAAGGGATGGG<br>(((.....)))	RAB3B	NM_002867	chr1:52146216-52229024	non-template	32	Intron_4.l	--	Yes	--	Yes	--	--	--	--	--	--	--
G4ST01052158412	chr1:52158413-52158446	-	GGGCTGAGGGCACAGAAGGGTTCCAGCAGAAGGG<br>(((.....)))	RAB3B	NM_002867	chr1:52146216-52229024	non-template	34	Intron_4.l	--	Yes	--	Yes	--	--	--	--	--	--	--
G4ST01052228858	chr1:52228859-52228897	+	GGGACGGCAGGACCTGGCGTCGGGAGGGCGCGGAGGGGG<br>(((.(((...))).))).	RAB3B	NM_002867	chr1:52146216-52229024	template	39	Exon_1.f	--	Yes	Yes	--	--	Yes	--	Yes	Yes	Yes	Yes
G4ST01052380073	chr1:52380074-52380114	+	GGGAGAGAACGAAAAGGTGCTCGGGTCTGGCGGGGGCGGGG<br>.(((.((......)).)))	ZFYVE9	NM_007324	chr1:52380634-52584946	non-template	-519	upstream	Yes	--	--	--	--	--	--	--	Yes	Yes	Yes
G4ST01052380073	chr1:52380074-52380114	+	GGGAGAGAACGAAAAGGTGCTCGGGTCTGGCGGGGGCGGGG<br>.(((.((......)).)))	ZFYVE9	NM_004799	chr1:52380634-52584946	non-template	-519	upstream	Yes	--	--	--	--	--	--	--	Yes	Yes	Yes
G4ST01052380073	chr1:52380074-52380114	+	GGGAGAGAACGAAAAGGTGCTCGGGTCTGGCGGGGGCGGGG<br>.(((.((......)).)))	ZFYVE9	NM_007323	chr1:52380634-52502307	non-template	-519	upstream	Yes	--	--	--	--	--	--	--	Yes	Yes	Yes
G4ST01052380471	chr1:52380472-52380501	+	GGGCGCCGATGCGGCTGGGCGGGTGCCGGG<br>.((((...)))).	ZFYVE9	NM_007324	chr1:52380634-52584946	non-template	-132	upstream	Yes	--	--	--	--	--	--	Yes	Yes	Yes	--
G4ST01052380471	chr1:52380472-52380501	+	GGGCGCCGATGCGGCTGGGCGGGTGCCGGG<br>.((((...)))).	ZFYVE9	NM_004799	chr1:52380634-52584946	non-template	-132	upstream	Yes	--	--	--	--	--	--	Yes	Yes	Yes	--
G4ST01052380471	chr1:52380472-52380501	+	GGGCGCCGATGCGGCTGGGCGGGTGCCGGG<br>.((((...)))).	ZFYVE9	NM_007323	chr1:52380634-52502307	non-template	-132	upstream	Yes	--	--	--	--	--	--	Yes	Yes	Yes	--
G4ST01052594479	chr1:52594480-52594509	+	GGGTGGGAGGGTCGAGGCCCTGAGCCTGGG<br>...((((.....))))	CC2D1B	NM_032449	chr1:52588853-52604465	template	30	Exon_18	--	Yes	Yes	--	Yes	--	--	--	Yes	--	--
G4ST01052602501	chr1:52602502-52602535	-	GGGATGCCAGGGCATGAGGGCTGTAGTCAGTGGG<br>(((....))).	CC2D1B	NM_032449	chr1:52588853-52604465	non-template	34	Intron_2	--	Yes	--	Yes	--	--	--	--	--	--	--
G4ST01052784784	chr1:52784785-52784819	+	GGGAAGAAGGGAACGGGAAATTATCATTTAATGGG<br>((((....))))...	ZCCHC11	NM_015269	chr1:52661536-52791350	template	35	Intron_1.f	--	Yes	--	Yes	--	--	--	--	--	--	Yes
G4ST01052784784	chr1:52784785-52784819	+	GGGAAGAAGGGAACGGGAAATTATCATTTAATGGG<br>((((....))))...	ZCCHC11	NM_001009881	chr1:52661536-52791350	template	35	Intron_1.f	--	Yes	--	Yes	--	--	--	--	--	--	Yes
G4ST01052871506	chr1:52871507-52871534	-	GGGGCGGGCGGCGGGGGCCGGCGGCGGG<br>.(((...)))	FAM159A	NM_001042693	chr1:52871654-52895325	template	-119	upstream	Yes	--	--	--	--	--	--	Yes	Yes	--	Yes
G4ST01052931162	chr1:52931163-52931200	+	GGGGCTGCTGCTCGCAGTGGGGACTTGGGGGTAGGGGG<br>.((((.....))))..	C1orf163	NM_023077	chr1:52924602-52936626	template	38	Intron_1.f	--	Yes	--	Yes	--	--	--	--	--	--	--
G4ST01053144745	chr1:53144746-53144773	-	GGGCCAGGACTGGGGGCAGGGGGTGGGG<br>(((....)))	ECHDC2	NM_018281	chr1:53134170-53160034	non-template	28	Intron_4	--	Yes	--	Yes	--	--	--	Yes	Yes	--	Yes
G4ST01053144745	chr1:53144746-53144773	-	GGGCCAGGACTGGGGGCAGGGGGTGGGG<br>(((....)))	ECHDC2	NM_001198961	chr1:53134170-53160034	non-template	28	Intron_5	--	Yes	--	Yes	--	--	--	Yes	Yes	--	Yes
G4ST01053144745	chr1:53144746-53144773	-	GGGCCAGGACTGGGGGCAGGGGGTGGGG<br>(((....)))	ECHDC2	NM_001198962	chr1:53134170-53160034	non-template	28	Intron_4	--	Yes	--	Yes	--	--	--	Yes	Yes	--	Yes
G4ST01053159532	chr1:53159533-53159576	-	GGGGTCTTCCGACTGTGTGGAAGGGGGAGCGGGGTCCGCACGGG<br>..((((((......)))))).	ECHDC2	NM_018281	chr1:53134170-53160034	non-template	44	Intron_1.f	--	Yes	--	Yes	--	--	--	--	Yes	--	Yes
G4ST01053159532	chr1:53159533-53159576	-	GGGGTCTTCCGACTGTGTGGAAGGGGGAGCGGGGTCCGCACGGG<br>..((((((......)))))).	ECHDC2	NM_001198961	chr1:53134170-53160034	non-template	44	Intron_1.f	--	Yes	--	Yes	--	--	--	--	Yes	--	Yes
G4ST01053159532	chr1:53159533-53159576	-	GGGGTCTTCCGACTGTGTGGAAGGGGGAGCGGGGTCCGCACGGG<br>..((((((......)))))).	ECHDC2	NM_001198962	chr1:53134170-53160034	non-template	44	Intron_1.f	--	Yes	--	Yes	--	--	--	--	Yes	--	Yes
G4ST01053165472	chr1:53165473-53165500	-	GGGGTGGGGCGCGTCCTGTGGGGCGGGG<br>.(((.....)))	SCP2	NM_001193617	chr1:53165489-53289877	template	12	upstream-Exon_1.f	Yes	Yes	Yes	--	--	Yes	--	Yes	Yes	Yes	--
G4ST01053165472	chr1:53165473-53165500	-	GGGGTGGGGCGCGTCCTGTGGGGCGGGG<br>.(((.....)))	SCP2	NM_002979	chr1:53165489-53289877	template	12	upstream-Exon_1.f	Yes	Yes	Yes	--	--	Yes	--	Yes	Yes	Yes	--
G4ST01053165472	chr1:53165473-53165500	-	GGGGTGGGGCGCGTCCTGTGGGGCGGGG<br>.(((.....)))	SCP2	NM_001193599	chr1:53165489-53289877	template	12	upstream-Exon_1.f	Yes	Yes	Yes	--	--	Yes	--	Yes	Yes	Yes	--
G4ST01053165472	chr1:53165473-53165500	-	GGGGTGGGGCGCGTCCTGTGGGGCGGGG<br>.(((.....)))	SCP2	NM_001193600	chr1:53165489-53289877	template	12	upstream-Exon_1.f	Yes	Yes	Yes	--	--	Yes	--	Yes	Yes	Yes	--
G4ST01053165472	chr1:53165473-53165500	-	GGGGTGGGGCGCGTCCTGTGGGGCGGGG<br>.(((.....)))	SCP2	NM_001007098	chr1:53165489-53232492	template	12	upstream-Exon_1.f	Yes	Yes	Yes	--	--	Yes	--	Yes	Yes	Yes	--
G4ST01053165477	chr1:53165478-53165506	-	GGGGGCGGGGTGGGGCGCGTCCTGTGGGG<br>.(((.....)))	SCP2	NM_001193617	chr1:53165489-53289877	template	18	upstream-Exon_1.f	Yes	Yes	Yes	--	--	Yes	--	Yes	Yes	Yes	--
G4ST01053165477	chr1:53165478-53165506	-	GGGGGCGGGGTGGGGCGCGTCCTGTGGGG<br>.(((.....)))	SCP2	NM_002979	chr1:53165489-53289877	template	18	upstream-Exon_1.f	Yes	Yes	Yes	--	--	Yes	--	Yes	Yes	Yes	--
G4ST01053165477	chr1:53165478-53165506	-	GGGGGCGGGGTGGGGCGCGTCCTGTGGGG<br>.(((.....)))	SCP2	NM_001193599	chr1:53165489-53289877	template	18	upstream-Exon_1.f	Yes	Yes	Yes	--	--	Yes	--	Yes	Yes	Yes	--
G4ST01053165477	chr1:53165478-53165506	-	GGGGGCGGGGTGGGGCGCGTCCTGTGGGG<br>.(((.....)))	SCP2	NM_001193600	chr1:53165489-53289877	template	18	upstream-Exon_1.f	Yes	Yes	Yes	--	--	Yes	--	Yes	Yes	Yes	--
G4ST01053165477	chr1:53165478-53165506	-	GGGGGCGGGGTGGGGCGCGTCCTGTGGGG<br>.(((.....)))	SCP2	NM_001007098	chr1:53165489-53232492	template	18	upstream-Exon_1.f	Yes	Yes	Yes	--	--	Yes	--	Yes	Yes	Yes	--
G4ST01053300196	chr1:53300197-53300233	+	GGGTGGGGGTTCGGGGGGCGTGGTGCGGCGGCCGGGG<br>((.(((......))).)).	PODN	NM_001199082	chr1:53300312-53323762	non-template	-78	upstream	Yes	--	--	--	--	--	--	--	--	--	--
G4ST01053300196	chr1:53300197-53300233	+	GGGTGGGGGTTCGGGGGGCGTGGTGCGGCGGCCGGGG<br>((.(((......))).)).	PODN	NM_001199081	chr1:53300473-53323762	non-template	-239	upstream	Yes	--	--	--	--	--	--	--	--	--	--
G4ST01053300196	chr1:53300197-53300233	+	GGGTGGGGGTTCGGGGGGCGTGGTGCGGCGGCCGGGG<br>((.(((......))).)).	PODN	NM_001199080	chr1:53300312-53323762	non-template	-78	upstream	Yes	--	--	--	--	--	--	--	--	--	--
G4ST01053300196	chr1:53300197-53300233	+	GGGTGGGGGTTCGGGGGGCGTGGTGCGGCGGCCGGGG<br>((.(((......))).)).	PODN	NM_153703	chr1:53300312-53323762	non-template	-78	upstream	Yes	--	--	--	--	--	--	--	--	--	--
G4ST01053300200	chr1:53300201-53300243	+	GGGGGTTCGGGGGGCGTGGTGCGGCGGCCGGGGCTGAGAGGGG<br>((.(((......))).)).	PODN	NM_001199082	chr1:53300312-53323762	non-template	-68	upstream	Yes	--	--	--	--	--	--	--	--	--	--
G4ST01053300200	chr1:53300201-53300243	+	GGGGGTTCGGGGGGCGTGGTGCGGCGGCCGGGGCTGAGAGGGG<br>((.(((......))).)).	PODN	NM_001199081	chr1:53300473-53323762	non-template	-229	upstream	Yes	--	--	--	--	--	--	--	--	--	--
G4ST01053300200	chr1:53300201-53300243	+	GGGGGTTCGGGGGGCGTGGTGCGGCGGCCGGGGCTGAGAGGGG<br>((.(((......))).)).	PODN	NM_001199080	chr1:53300312-53323762	non-template	-68	upstream	Yes	--	--	--	--	--	--	--	--	--	--
G4ST01053300200	chr1:53300201-53300243	+	GGGGGTTCGGGGGGCGTGGTGCGGCGGCCGGGGCTGAGAGGGG<br>((.(((......))).)).	PODN	NM_153703	chr1:53300312-53323762	non-template	-68	upstream	Yes	--	--	--	--	--	--	--	--	--	--
G4ST01053300573	chr1:53300574-53300603	+	GGGCGCTGGCAGCCGGGGTGGGCCGAGGGG<br>.(((...)))..	PODN	NM_001199082	chr1:53300312-53323762	non-template	30	Intron_1.f	--	Yes	--	Yes	--	--	--	Yes	--	--	Yes
G4ST01053300573	chr1:53300574-53300603	+	GGGCGCTGGCAGCCGGGGTGGGCCGAGGGG<br>.(((...)))..	PODN	NM_001199081	chr1:53300473-53323762	non-template	30	Intron_1.f	--	Yes	--	Yes	--	--	--	Yes	--	--	Yes
G4ST01053300573	chr1:53300574-53300603	+	GGGCGCTGGCAGCCGGGGTGGGCCGAGGGG<br>.(((...)))..	PODN	NM_001199080	chr1:53300312-53323762	non-template	30	Intron_1.f	--	Yes	--	Yes	--	--	--	Yes	--	--	Yes
G4ST01053300573	chr1:53300574-53300603	+	GGGCGCTGGCAGCCGGGGTGGGCCGAGGGG<br>.(((...)))..	PODN	NM_153703	chr1:53300312-53323762	non-template	30	Intron_1.f	--	Yes	--	Yes	--	--	--	Yes	--	--	Yes

G4ST01053300592	chr1:53300593-53300621	+	GGGCCGAGGGGCCGGGGGCTGAGCCCGGG<br>(((...))).	PODN	NM_001199082	chr1:53300312-53323762	non-template	29	Intron_1.f	--	Yes	--	Yes	--	--	--	Yes	--	--	Yes
G4ST01053300592	chr1:53300593-53300621	+	GGGCCGAGGGGCCGGGGGCTGAGCCCGGG<br>(((...))).	PODN	NM_001199081	chr1:53300473-53323762	non-template	29	Intron_1.f	--	Yes	--	Yes	--	--	--	Yes	--	--	Yes
G4ST01053300592	chr1:53300593-53300621	+	GGGCCGAGGGGCCGGGGGCTGAGCCCGGG<br>(((...))).	PODN	NM_001199080	chr1:53300312-53323762	non-template	29	Intron_1.f	--	Yes	--	Yes	--	--	--	Yes	--	--	Yes
G4ST01053300592	chr1:53300593-53300621	+	GGGCCGAGGGGCCGGGGGCTGAGCCCGGG<br>(((...))).	PODN	NM_153703	chr1:53300312-53323762	non-template	29	Intron_1.f	--	Yes	--	Yes	--	--	--	Yes	--	--	Yes
G4ST01053309910	chr1:53309911-53309941	+	GGGTCAGGGACACCAAGTGGGGCCTTGGGGG<br>.(((...)))	PODN	NM_001199082	chr1:53300312-53323762	non-template	31	Intron_2	--	Yes	--	Yes	--	--	--	--	--	--	--
G4ST01053309910	chr1:53309911-53309941	+	GGGTCAGGGACACCAAGTGGGGCCTTGGGGG<br>.(((...)))	PODN	NM_001199081	chr1:53300473-53323762	non-template	31	Intron_4	--	Yes	--	Yes	--	--	--	--	--	--	--
G4ST01053309910	chr1:53309911-53309941	+	GGGTCAGGGACACCAAGTGGGGCCTTGGGGG<br>.(((...)))	PODN	NM_001199080	chr1:53300312-53323762	non-template	31	Intron_5	--	Yes	--	Yes	--	--	--	--	--	--	--
G4ST01053309910	chr1:53309911-53309941	+	GGGTCAGGGACACCAAGTGGGGCCTTGGGGG<br>.(((...)))	PODN	NM_153703	chr1:53300312-53323762	non-template	31	Intron_3	--	Yes	--	Yes	--	--	--	--	--	--	--
G4ST01053317761	chr1:53317762-53317797	+	GGGATGGGAGGCAGAGGGGACAGCCTGAGCTGTGGG<br>.(((((....)))))	PODN	NM_001199082	chr1:53300312-53323762	non-template	36	Intron_4	--	Yes	--	Yes	--	--	--	--	--	--	Yes
G4ST01053317761	chr1:53317762-53317797	+	GGGATGGGAGGCAGAGGGGACAGCCTGAGCTGTGGG<br>.(((((....)))))	PODN	NM_001199081	chr1:53300473-53323762	non-template	36	Intron_9	--	Yes	--	Yes	--	--	--	--	--	--	Yes
G4ST01053317761	chr1:53317762-53317797	+	GGGATGGGAGGCAGAGGGGACAGCCTGAGCTGTGGG<br>.(((((....)))))	PODN	NM_001199080	chr1:53300312-53323762	non-template	36	Intron_10	--	Yes	--	Yes	--	--	--	--	--	--	Yes
G4ST01053317761	chr1:53317762-53317797	+	GGGATGGGAGGCAGAGGGGACAGCCTGAGCTGTGGG<br>.(((((....)))))	PODN	NM_153703	chr1:53300312-53323762	non-template	36	Intron_8	--	Yes	--	Yes	--	--	--	--	--	--	Yes
G4ST01053322440	chr1:53322441-53322476	+	GGGGTGGGTGACAGGGTGACTGGCCATCCAGTGGGG<br>..(((((....))))).	PODN	NM_001199082	chr1:53300312-53323762	non-template	36	Intron_6.l	--	Yes	--	Yes	--	--	--	--	--	--	--
G4ST01053322440	chr1:53322441-53322476	+	GGGGTGGGTGACAGGGTGACTGGCCATCCAGTGGGG<br>..(((((....))))).	PODN	NM_001199081	chr1:53300473-53323762	non-template	36	Intron_11.l	--	Yes	--	Yes	--	--	--	--	--	--	--
G4ST01053322440	chr1:53322441-53322476	+	GGGGTGGGTGACAGGGTGACTGGCCATCCAGTGGGG<br>..(((((....))))).	PODN	NM_001199080	chr1:53300312-53323762	non-template	36	Intron_12.l	--	Yes	--	Yes	--	--	--	--	--	--	--
G4ST01053322440	chr1:53322441-53322476	+	GGGGTGGGTGACAGGGTGACTGGCCATCCAGTGGGG<br>..(((((....))))).	PODN	NM_153703	chr1:53300312-53323762	non-template	36	Intron_10.l	--	Yes	--	Yes	--	--	--	--	--	--	--
G4ST01053358717	chr1:53358718-53358745	-	GGGGACGCAGGGCAGGGAAGGAGCTGGG<br>.((...))	SLC1A7	NM_006671	chr1:53325443-53380877	non-template	28	Intron_2	--	Yes	--	Yes	--	--	--	Yes	--	--	--
G4ST01053361881	chr1:53361882-53361911	+	GGGCATCGGGCTGGGCCTCCCAGAGGAGGG<br>((((...)))).	SLC1A7	NM_006671	chr1:53325443-53380877	template	30	Intron_2	--	Yes	--	Yes	--	--	--	--	--	--	--
G4ST01053445247	chr1:53445248-53445283	+	GGGAGGGACCTAGTGGGAGGTGATTGGATCATGGGG<br>..((((.....)))).	CPT2	NM_000098	chr1:53434689-53452457	non-template	36	Intron_3	--	Yes	--	Yes	--	--	--	Yes	--	--	--
G4ST01053514678	chr1:53514679-53514707	+	GGGGGATGGGGGTGGGAAGTACGACTGGG<br>.(((...)))	LRP8	NM_017522	chr1:53483805-53566314	template	29	Intron_4	--	Yes	--	Yes	--	--	--	--	--	--	Yes
G4ST01053514678	chr1:53514679-53514707	+	GGGGGATGGGGGTGGGAAGTACGACTGGG<br>.(((...)))	LRP8	NM_004631	chr1:53483805-53566314	template	29	Intron_5	--	Yes	--	Yes	--	--	--	--	--	--	Yes
G4ST01053514678	chr1:53514679-53514707	+	GGGGGATGGGGGTGGGAAGTACGACTGGG<br>.(((...)))	LRP8	NM_033300	chr1:53483800-53566409	template	29	Intron_4	--	Yes	--	Yes	--	--	--	--	--	--	Yes
G4ST01053514678	chr1:53514679-53514707	+	GGGGGATGGGGGTGGGAAGTACGACTGGG<br>.(((...)))	LRP8	NM_001018054	chr1:53483805-53566314	template	29	Intron_5	--	Yes	--	Yes	--	--	--	--	--	--	Yes
G4ST01053514685	chr1:53514686-53514711	+	GGGGGTGGGAAGTACGACTGGGAGGG<br>.(((...)))	LRP8	NM_017522	chr1:53483805-53566314	template	26	Intron_4	--	Yes	--	Yes	--	--	--	--	--	--	Yes
G4ST01053514685	chr1:53514686-53514711	+	GGGGGTGGGAAGTACGACTGGGAGGG<br>.(((...)))	LRP8	NM_004631	chr1:53483805-53566314	template	26	Intron_5	--	Yes	--	Yes	--	--	--	--	--	--	Yes
G4ST01053514685	chr1:53514686-53514711	+	GGGGGTGGGAAGTACGACTGGGAGGG<br>.(((...)))	LRP8	NM_033300	chr1:53483800-53566409	template	26	Intron_4	--	Yes	--	Yes	--	--	--	--	--	--	Yes
G4ST01053514685	chr1:53514686-53514711	+	GGGGGTGGGAAGTACGACTGGGAGGG<br>.(((...)))	LRP8	NM_001018054	chr1:53483805-53566314	template	26	Intron_5	--	Yes	--	Yes	--	--	--	--	--	--	Yes
G4ST01053535031	chr1:53535032-53535065	-	GGGACGGCCGTCAGGCCAGGGCCCCCGGGTGGGG<br>..((((....)))).	LRP8	NM_017522	chr1:53483805-53566314	non-template	34	Intron_2	--	Yes	--	Yes	--	--	--	--	--	--	--
G4ST01053535031	chr1:53535032-53535065	-	GGGACGGCCGTCAGGCCAGGGCCCCCGGGTGGGG<br>..((((....)))).	LRP8	NM_004631	chr1:53483805-53566314	non-template	34	Intron_2	--	Yes	--	Yes	--	--	--	--	--	--	--
G4ST01053535031	chr1:53535032-53535065	-	GGGACGGCCGTCAGGCCAGGGCCCCCGGGTGGGG<br>..((((....)))).	LRP8	NM_033300	chr1:53483800-53566409	non-template	34	Intron_2	--	Yes	--	Yes	--	--	--	--	--	--	--
G4ST01053535031	chr1:53535032-53535065	-	GGGACGGCCGTCAGGCCAGGGCCCCCGGGTGGGG<br>..((((....)))).	LRP8	NM_001018054	chr1:53483805-53566314	non-template	34	Intron_2	--	Yes	--	Yes	--	--	--	--	--	--	--
G4ST01053539468	chr1:53539469-53539504	+	GGGGGCATTGGGGAACAAGGGAATTGTCTGATTGGG<br>((((....))))	LRP8	NM_017522	chr1:53483805-53566314	template	36	Intron_2	--	Yes	--	Yes	--	--	--	--	--	--	Yes
G4ST01053539468	chr1:53539469-53539504	+	GGGGGCATTGGGGAACAAGGGAATTGTCTGATTGGG<br>((((....))))	LRP8	NM_004631	chr1:53483805-53566314	template	36	Intron_2	--	Yes	--	Yes	--	--	--	--	--	--	Yes
G4ST01053539468	chr1:53539469-53539504	+	GGGGGCATTGGGGAACAAGGGAATTGTCTGATTGGG<br>((((....))))	LRP8	NM_033300	chr1:53483800-53566409	template	36	Intron_2	--	Yes	--	Yes	--	--	--	--	--	--	Yes
G4ST01053539468	chr1:53539469-53539504	+	GGGGGCATTGGGGAACAAGGGAATTGTCTGATTGGG<br>((((....))))	LRP8	NM_001018054	chr1:53483805-53566314	template	36	Intron_2	--	Yes	--	Yes	--	--	--	--	--	--	Yes
G4ST01053549386	chr1:53549387-53549417	-	GGGGGTGGACCAGGGCAGGGAGGCCCCCGGG<br>.((...))	LRP8	NM_017522	chr1:53483805-53566314	non-template	31	Intron_2	--	Yes	--	Yes	--	--	--	--	--	--	--
G4ST01053549386	chr1:53549387-53549417	-	GGGGGTGGACCAGGGCAGGGAGGCCCCCGGG<br>.((...))	LRP8	NM_004631	chr1:53483805-53566314	non-template	31	Intron_2	--	Yes	--	Yes	--	--	--	--	--	--	--
G4ST01053549386	chr1:53549387-53549417	-	GGGGGTGGACCAGGGCAGGGAGGCCCCCGGG<br>.((...))	LRP8	NM_033300	chr1:53483800-53566409	non-template	31	Intron_2	--	Yes	--	Yes	--	--	--	--	--	--	--
G4ST01053549386	chr1:53549387-53549417	-	GGGGGTGGACCAGGGCAGGGAGGCCCCCGGG<br>.((...))	LRP8	NM_001018054	chr1:53483805-53566314	non-template	31	Intron_2	--	Yes	--	Yes	--	--	--	--	--	--	--
G4ST01053555405	chr1:53555406-53555431	+	GGGTCCAGGAAGTGGGGGAGGGAGGG<br>.(((.....)))	LRP8	NM_017522	chr1:53483805-53566314	template	26	Intron_2	--	Yes	--	Yes	--	--	--	--	Yes	--	Yes
G4ST01053555405	chr1:53555406-53555431	+	GGGTCCAGGAAGTGGGGGAGGGAGGG<br>.(((.....)))	LRP8	NM_004631	chr1:53483805-53566314	template	26	Intron_2	--	Yes	--	Yes	--	--	--	--	Yes	--	Yes
G4ST01053555405	chr1:53555406-53555431	+	GGGTCCAGGAAGTGGGGGAGGGAGGG<br>.(((.....)))	LRP8	NM_033300	chr1:53483800-53566409	template	26	Intron_2	--	Yes	--	Yes	--	--	--	--	Yes	--	Yes
G4ST01053555405	chr1:53555406-53555431	+	GGGTCCAGGAAGTGGGGGAGGGAGGG<br>.(((.....)))	LRP8	NM_001018054	chr1:53483805-53566314	template	26	Intron_2	--	Yes	--	Yes	--	--	--	--	Yes	--	Yes
G4ST01053558029	chr1:53558030-53558070	+	GGGGGACCCAGCTCATGCTGGAGGGCGCCCAAGGGGCTGGG<br>....(((((....))))).	LRP8	NM_017522	chr1:53483805-53566314	template	41	Intron_2	--	Yes	--	Yes	--	--	--	Yes	--	Yes	--
G4ST01053558029	chr1:53558030-53558070	+	GGGGGACCCAGCTCATGCTGGAGGGCGCCCAAGGGGCTGGG<br>....(((((....))))).	LRP8	NM_004631	chr1:53483805-53566314	template	41	Intron_2	--	Yes	--	Yes	--	--	--	Yes	--	Yes	--
G4ST01053558029	chr1:53558030-53558070	+	GGGGGACCCAGCTCATGCTGGAGGGCGCCCAAGGGGCTGGG<br>....(((((....))))).	LRP8	NM_033300	chr1:53483800-53566409	template	41	Intron_2	--	Yes	--	Yes	--	--	--	Yes	--	Yes	--
G4ST01053558029	chr1:53558030-53558070	+	GGGGGACCCAGCTCATGCTGGAGGGCGCCCAAGGGGCTGGG<br>....(((((....))))).	LRP8	NM_001018054	chr1:53483805-53566314	template	41	Intron_2	--	Yes	--	Yes	--	--	--	Yes	--	Yes	--
G4ST01053559473	chr1:53559474-53559508	+	GGGAGCTCAGGGCCTGAGGGGCTGGAGGACAGGGG<br>.(((.....)))	LRP8	NM_017522	chr1:53483805-53566314	template	35	Intron_2	--	Yes	--	Yes	--	--	--	--	--	Yes	--
G4ST01053559473	chr1:53559474-53559508	+	GGGAGCTCAGGGCCTGAGGGGCTGGAGGACAGGGG<br>.(((.....)))	LRP8	NM_004631	chr1:53483805-53566314	template	35	Intron_2	--	Yes	--	Yes	--	--	--	--	--	Yes	--
G4ST01053559473	chr1:53559474-53559508	+	GGGAGCTCAGGGCCTGAGGGGCTGGAGGACAGGGG<br>.(((.....)))	LRP8	NM_033300	chr1:53483800-53566409	template	35	Intron_2	--	Yes	--	Yes	--	--	--	--	--	Yes	--

G4ST01053559473	chr1:53559474-53559508	+	GGGAGCTCAGGGCCTGAGGGGCTGGAGGACAGGGG<br>.(((.....)))	LRP8	NM_001018054	chr1:53483805-53566314	template	35	Intron_2	--	Yes	--	Yes	--	--	--	--	--	Yes	--
G4ST01053564547	chr1:53564548-53564581	+	GGGGCAGGGCAGGGCGGTGCCCCCGCGCCCAGGG<br>.(((((....)))))..	LRP8	NM_017522	chr1:53483805-53566314	template	34	Intron_2	--	Yes	--	Yes	--	--	--	Yes	Yes	Yes	Yes
G4ST01053564547	chr1:53564548-53564581	+	GGGGCAGGGCAGGGCGGTGCCCCCGCGCCCAGGG<br>.(((((....)))))..	LRP8	NM_004631	chr1:53483805-53566314	template	34	Intron_2	--	Yes	--	Yes	--	--	--	Yes	Yes	Yes	Yes
G4ST01053564547	chr1:53564548-53564581	+	GGGGCAGGGCAGGGCGGTGCCCCCGCGCCCAGGG<br>.(((((....)))))..	LRP8	NM_033300	chr1:53483800-53566409	template	34	Intron_2	--	Yes	--	Yes	--	--	--	Yes	Yes	Yes	Yes
G4ST01053564547	chr1:53564548-53564581	+	GGGGCAGGGCAGGGCGGTGCCCCCGCGCCCAGGG<br>.(((((....)))))..	LRP8	NM_001018054	chr1:53483805-53566314	template	34	Intron_2	--	Yes	--	Yes	--	--	--	Yes	Yes	Yes	Yes
G4ST01053698770	chr1:53698771-53698813	-	GGGGAAACGGAGGGAAGAGGTGCGCAGGCGCACTGGGCCAGGG<br>.....(((((....))))).	DMRTB1	NM_033067	chr1:53697660-53705746	template	43	Intron_1.f	--	Yes	--	Yes	--	--	--	Yes	Yes	--	--
G4ST01053705136	chr1:53705137-53705164	+	GGGGAGGGCCAGGGCTCTGAGGAGTGGG<br>(((....))).	DMRTB1	NM_033067	chr1:53697660-53705746	non-template	28	Exon_4.l	--	Yes	Yes	--	--	--	Yes	--	--	--	--
G4ST01053705141	chr1:53705142-53705171	+	GGGCCAGGGCTCTGAGGAGTGGGCGCTGGG<br>(((....))).	DMRTB1	NM_033067	chr1:53697660-53705746	non-template	30	Exon_4.l	--	Yes	Yes	--	--	--	Yes	--	--	--	--
G4ST01053745211	chr1:53745212-53745239	+	GGGGCGAGGGCTTGGGAGGCACCCTGGG<br>(((...)))	GLIS1	NM_147193	chr1:53744494-53972465	template	28	Intron_9.l	--	Yes	--	Yes	--	--	--	--	--	--	Yes
G4ST01053745218	chr1:53745219-53745257	+	GGGCTTGGGAGGCACCCTGGGCCTGGCACACCCAGGGGG<br>(((...))) (((((.....)))))	GLIS1	NM_147193	chr1:53744494-53972465	template	39	Intron_9.l	--	Yes	--	Yes	--	--	--	--	--	--	Yes
G4ST01053745224	chr1:53745225-53745264	+	GGGAGGCACCCTGGGCCTGGCACACCCAGGGGGCCCAGGG<br>(((...))) (((((.....)))))	GLIS1	NM_147193	chr1:53744494-53972465	template	40	Intron_9.l	--	Yes	--	Yes	--	--	--	--	--	--	Yes
G4ST01053745236	chr1:53745237-53745273	+	GGGCCTGGCACACCCAGGGGGCCCAGGGCAGCTGGGG<br>(((((.....)))))	GLIS1	NM_147193	chr1:53744494-53972465	template	37	Intron_9.l	--	Yes	--	Yes	--	--	--	--	--	--	--
G4ST01053746897	chr1:53746898-53746944	+	GGGGAGGCCCAGGGCCCTGCAGGGCAACACCAGGTACCTAAGTGGGG<br>...(((.((....))..)))	GLIS1	NM_147193	chr1:53744494-53972465	template	47	Intron_9.l	--	Yes	--	Yes	--	--	--	--	--	--	Yes
G4ST01053748320	chr1:53748321-53748351	+	GGGGTCTGGAGGGTGGGGGCCAGAGGCAGGG<br>.(((...))).	GLIS1	NM_147193	chr1:53744494-53972465	template	31	Intron_7	--	Yes	--	Yes	--	--	--	--	--	--	--
G4ST01053751376	chr1:53751377-53751418	-	GGGATGAGCTGGGGGTCCAGAGGGGCTGCTGACAGGCAGGGG<br>.(((((....)))))	GLIS1	NM_147193	chr1:53744494-53972465	non-template	42	Intron_7	--	Yes	--	Yes	--	--	--	--	--	--	--
G4ST01053751393	chr1:53751394-53751437	-	GGGAGAGGAGGAGGCCTCAGGGATGAGCTGGGGGTCCAGAGGGG<br>.((((......)))).	GLIS1	NM_147193	chr1:53744494-53972465	non-template	44	Intron_7	--	Yes	--	Yes	--	--	--	--	--	--	--
G4ST01053751403	chr1:53751404-53751442	-	GGGCAGGGAGAGGAGGAGGCCTCAGGGATGAGCTGGGGG<br>.((((......)))).	GLIS1	NM_147193	chr1:53744494-53972465	non-template	39	Intron_7	--	Yes	--	Yes	--	--	--	--	--	--	--
G4ST01053756041	chr1:53756042-53756069	-	GGGGCAAGGGCAGGGGACTGAGTCAGGG<br>(((...))).	GLIS1	NM_147193	chr1:53744494-53972465	non-template	28	Intron_6	--	Yes	--	Yes	--	--	--	--	--	--	Yes
G4ST01053758489	chr1:53758490-53758528	+	GGGCTGGCTGGGTGTGGACAGTGTCCGGGGGTCGGGGGG<br>..(((((...))))).	GLIS1	NM_147193	chr1:53744494-53972465	template	39	Intron_6	--	Yes	--	Yes	--	--	--	--	--	--	--
G4ST01053762798	chr1:53762799-53762833	+	GGGCAGTCTGTGGGTGGTAAAGGGCCAGCTGGGGG<br>((...)).	GLIS1	NM_147193	chr1:53744494-53972465	template	35	Intron_5	--	Yes	--	Yes	--	--	--	Yes	--	--	--
G4ST01053768279	chr1:53768280-53768315	-	GGGGCAGGGGTGCTGGGGCCAGGCAGGCCTGGGGGG<br>.(((((....))))).	GLIS1	NM_147193	chr1:53744494-53972465	non-template	36	Intron_3	--	Yes	--	Yes	--	--	--	--	--	--	Yes
G4ST01053775431	chr1:53775432-53775467	+	GGGACCCCTCAAGCTCTGGCTTGGGGAGGGGGAGGG<br>......(((((....)))))	GLIS1	NM_147193	chr1:53744494-53972465	template	36	Intron_3	--	Yes	--	Yes	--	--	--	--	--	--	--
G4ST01053782885	chr1:53782886-53782924	-	GGGACCAGGGAGCTTGGGGAGCCAGCTACAGCTGGAGGG<br>...(((((....))))).	GLIS1	NM_147193	chr1:53744494-53972465	non-template	39	Intron_3	--	Yes	--	Yes	--	--	--	--	--	--	--
G4ST01053793193	chr1:53793194-53793223	+	GGGCCTCACAGGTAGGGCAGGGCACTTGGG<br>(((...)))..	GLIS1	NM_147193	chr1:53744494-53972465	template	30	Intron_3	--	Yes	--	Yes	--	--	--	--	--	--	Yes
G4ST01053797689	chr1:53797690-53797725	-	GGGCACGAGGGCTGAGGGACTGACCACAGTGGAGGG<br>((((....))))....	GLIS1	NM_147193	chr1:53744494-53972465	non-template	36	Intron_3	--	Yes	--	Yes	--	--	--	--	--	--	--
G4ST01053797943	chr1:53797944-53797977	-	GGGCTGAGGCCAGGGGGTGCAGGGCGTGTGGGGG<br>(((....))).	GLIS1	NM_147193	chr1:53744494-53972465	non-template	34	Intron_3	--	Yes	--	Yes	--	--	--	Yes	--	--	--
G4ST01053800820	chr1:53800821-53800848	-	GGGCTGGGACTCAGCTGAGAGGGTGGGG<br>.(((....))).	GLIS1	NM_147193	chr1:53744494-53972465	non-template	28	Intron_3	--	Yes	--	Yes	--	--	--	Yes	--	--	--
G4ST01053818528	chr1:53818529-53818555	-	GGGAGGGCTTGGGGCAGCGGCCTGGGG<br>.(((....)))	GLIS1	NM_147193	chr1:53744494-53972465	non-template	27	Intron_3	--	Yes	--	Yes	--	--	--	--	--	--	Yes
G4ST01053828209	chr1:53828210-53828232	-	GGGTGGGAAGGGCAGTATGTGGG<br>((...)).	GLIS1	NM_147193	chr1:53744494-53972465	non-template	23	Intron_3	--	Yes	--	Yes	--	--	--	--	--	--	--
G4ST01053832565	chr1:53832566-53832612	-	GGGCCTGGCAGGCAGGGTGGTGGCCGGGCGGCAGGCGTGCCGCTGGG<br>(((...))).. .((...)) (((((....)))))..	GLIS1	NM_147193	chr1:53744494-53972465	non-template	47	Exon_3	--	Yes	Yes	--	Yes	--	--	Yes	Yes	--	--
G4ST01053832584	chr1:53832585-53832618	-	GGGGCTGGGCCTGGCAGGCAGGGTGGTGGCCGGG<br>(((...))).. .((...))	GLIS1	NM_147193	chr1:53744494-53972465	non-template	34	Exon_3	--	Yes	Yes	--	Yes	--	--	Yes	Yes	--	--
G4ST01053838594	chr1:53838595-53838623	+	GGGGTATGGGGAGAGGGCAGTCCCTGGGG<br>(((...)))	GLIS1	NM_147193	chr1:53744494-53972465	template	29	Intron_1.f	--	Yes	--	Yes	--	--	--	--	--	--	--
G4ST01053843589	chr1:53843590-53843630	-	GGGCCCAGAGGGGTTTGGAAGGGTCAGGCCACCCTGATGGG<br>.((((....))))..	GLIS1	NM_147193	chr1:53744494-53972465	non-template	41	Intron_1.f	--	Yes	--	Yes	--	--	--	--	--	--	Yes
G4ST01053867562	chr1:53867563-53867594	-	GGGCTGTGAAGCACAGAGGGGATTGGGGAGGG<br>(((((...)))))..	GLIS1	NM_147193	chr1:53744494-53972465	non-template	32	Intron_1.f	--	Yes	--	Yes	--	--	--	Yes	Yes	--	Yes
G4ST01053870007	chr1:53870008-53870065	+	GGGTAATGACCTGAAGCAGGTGAGGGATTGAGTCATATGAGTGTCTGGGTCTCGGGGG<br>.....(((((...))))).. ....((.(((....))).))	GLIS1	NM_147193	chr1:53744494-53972465	template	58	Intron_1.f	--	Yes	--	Yes	--	--	--	Yes	Yes	--	--
G4ST01053870030	chr1:53870031-53870069	+	GGGATTGAGTCATATGAGTGTCTGGGTCTCGGGGGAGGG<br>....((.(((....))).))	GLIS1	NM_147193	chr1:53744494-53972465	template	39	Intron_1.f	--	Yes	--	Yes	--	--	--	Yes	Yes	--	--
G4ST01053898882	chr1:53898883-53898916	+	GGGGAGAGTCAGGGTCAGCCTGACTGGGGCAGGG<br>.(((.....)))	GLIS1	NM_147193	chr1:53744494-53972465	template	34	Intron_1.f	--	Yes	--	Yes	--	--	--	--	--	--	--
G4ST01053898893	chr1:53898894-53898923	+	GGGTCAGCCTGACTGGGGCAGGGAGGAGGG<br>.(((.....)))	GLIS1	NM_147193	chr1:53744494-53972465	template	30	Intron_1.f	--	Yes	--	Yes	--	--	--	--	--	--	--
G4ST01053915330	chr1:53915331-53915361	-	GGGTAGCGGGACAGGGGAAGGAGAGCTTGGG<br>.(((.....)))	GLIS1	NM_147193	chr1:53744494-53972465	non-template	31	Intron_1.f	--	Yes	--	Yes	--	--	--	--	--	--	--
G4ST01053925681	chr1:53925682-53925717	-	GGGCTTGGTGAAGCGGGGCGGGCTGGTTGGACCGGG<br>(((....))).. ..(((...)))	GLIS1	NM_147193	chr1:53744494-53972465	non-template	36	Intron_1.f	--	Yes	--	Yes	--	--	--	--	--	--	--
G4ST01053931784	chr1:53931785-53931820	-	GGGCTGTGGGGGCAGCTCTGGCTGGGGTGCTGTGGG<br>..((((....))))	GLIS1	NM_147193	chr1:53744494-53972465	non-template	36	Intron_1.f	--	Yes	--	Yes	--	--	--	Yes	--	--	--
G4ST01054183864	chr1:54183865-54183895	-	GGGACGCTCTGCGTGGGGCGGGGCGCAAGGG<br>((((...)))).	LRRC42	NM_052940	chr1:54184625-54206427	template	-729	upstream	Yes	--	--	--	--	--	--	--	Yes	Yes	--
G4ST01054183864	chr1:54183865-54183895	-	GGGACGCTCTGCGTGGGGCGGGGCGCAAGGG<br>((((...)))).	HSPB11	NM_016126	chr1:54159822-54183876	non-template	12	upstream-Exon_1.f	Yes	Yes	Yes	--	--	Yes	--	--	Yes	Yes	--
G4ST01054183872	chr1:54183873-54183900	-	GGGTCGGGACGCTCTGCGTGGGGCGGGG<br>((((...)))).	LRRC42	NM_052940	chr1:54184625-54206427	template	-724	upstream	Yes	--	--	--	--	--	--	--	Yes	Yes	--
G4ST01054183872	chr1:54183873-54183900	-	GGGTCGGGACGCTCTGCGTGGGGCGGGG<br>((((...)))).	HSPB11	NM_016126	chr1:54159822-54183876	non-template	4	upstream-Exon_1.f	Yes	Yes	Yes	--	--	Yes	--	--	Yes	Yes	--
G4ST01054184584	chr1:54184585-54184622	-	GGGAGCAAGGCTGGGCCGGGGAGGCGCGCGTGCGCGGG<br>(((...))) ...((((....))))	LRRC42	NM_052940	chr1:54184625-54206427	template	-2	upstream	Yes	--	--	--	--	--	--	Yes	Yes	Yes	Yes
G4ST01054184584	chr1:54184585-54184622	-	GGGAGCAAGGCTGGGCCGGGGAGGCGCGCGTGCGCGGG<br>(((...))) ...((((....))))	HSPB11	NM_016126	chr1:54159822-54183876	non-template	-708	upstream	Yes	--	--	--	--	--	--	Yes	Yes	Yes	Yes
G4ST01054184601	chr1:54184602-54184628	-	GGGGGCGGGAGCAAGGCTGGGCCGGGG<br>(((...)))	LRRC42	NM_052940	chr1:54184625-54206427	template	4	upstream-Exon_1.f	Yes	Yes	Yes	--	--	Yes	--	Yes	Yes	Yes	Yes
G4ST01054184601	chr1:54184602-54184628	-	GGGGGCGGGAGCAAGGCTGGGCCGGGG<br>(((...)))	HSPB11	NM_016126	chr1:54159822-54183876	non-template	-725	upstream	Yes	--	--	--	--	--	--	Yes	Yes	Yes	Yes
G4ST01054184607	chr1:54184608-54184637	-	GGGACCGGCGGGGGCGGGAGCAAGGCTGGG<br>(((...)))	LRRC42	NM_052940	chr1:54184625-54206427	template	13	upstream-Exon_1.f	Yes	Yes	Yes	--	--	Yes	--	Yes	Yes	Yes	Yes

G4ST01054184607	chr1:54184608-54184637	-	GGGACCGGCGGGGGCGGGAGCAAGGCTGGG<br>(((...)))	HSPB11	NM_016126	chr1:54159822-54183876	non-template	-731	upstream	Yes	--	--	--	--	--	--	Yes	Yes	Yes	Yes
G4ST01054185505	chr1:54185506-54185534	+	GGGGCAGGAGAGCTGGGGTGGGCGAAGGG<br>.(((.....)))	LRRC42	NM_052940	chr1:54184625-54206427	non-template	29	Intron_1.f	--	Yes	--	Yes	--	--	--	--	Yes	--	Yes
G4ST01054185505	chr1:54185506-54185534	+	GGGGCAGGAGAGCTGGGGTGGGCGAAGGG<br>.(((.....)))	HSPB11	NM_016126	chr1:54159822-54183876	template	-1629	upstream	Yes	--	--	--	--	--	--	--	Yes	--	Yes
G4ST01054333694	chr1:54333695-54333725	+	GGGCCTGGAAAGGAGGGTAGGGGCTGGGGGG<br>(((....))).	TCEANC2	NM_153035	chr1:54291862-54338004	non-template	31	Intron_4.l	--	Yes	--	Yes	--	--	--	--	--	--	--
G4ST01054383134	chr1:54383135-54383170	+	GGGCAGTGGGCCCAGCTACAAAGCTGGGTTCTTGGG<br>...((((....))))	CDCP2	NM_201546	chr1:54377256-54392031	template	36	Intron_1.f	--	Yes	--	Yes	--	--	--	--	Yes	--	Yes
G4ST01054383141	chr1:54383142-54383175	+	GGGCCCAGCTACAAAGCTGGGTTCTTGGGATGGG<br>...((((....))))	CDCP2	NM_201546	chr1:54377256-54392031	template	34	Intron_1.f	--	Yes	--	Yes	--	--	--	--	Yes	--	Yes
G4ST01054416249	chr1:54416250-54416288	-	GGGCGCGAGGGATGGGCCACTGTTGCCCAGCAGCATGGG<br>.....(((((...)))))..	CYB5RL	NM_001031672	chr1:54410615-54438334	non-template	39	Intron_7.l	--	Yes	--	Yes	--	--	--	--	--	--	Yes
G4ST01054427713	chr1:54427714-54427737	+	GGGCAGGGGTGGGTTGGCAAAGGG<br>((....))	CYB5RL	NM_001031672	chr1:54410615-54438334	template	24	Intron_4	--	Yes	--	Yes	--	--	--	--	--	--	--
G4ST01054427878	chr1:54427879-54427916	+	GGGATGTCACCGCAGGGACAGAGAGGGTGAAGGAGGGG<br>.(((....)))	CYB5RL	NM_001031672	chr1:54410615-54438334	template	38	Intron_4	--	Yes	--	Yes	--	--	--	--	--	--	--
G4ST01054495678	chr1:54495679-54495701	+	GGGTGGGAAGGGCAGCGGCTGGG<br>.((...))	SSBP3	NM_018070	chr1:54464783-54644567	template	23	Intron_6	--	Yes	--	Yes	--	--	--	--	--	--	--
G4ST01054495678	chr1:54495679-54495701	+	GGGTGGGAAGGGCAGCGGCTGGG<br>.((...))	SSBP3	NM_145716	chr1:54464783-54644567	template	23	Intron_6	--	Yes	--	Yes	--	--	--	--	--	--	--
G4ST01054495678	chr1:54495679-54495701	+	GGGTGGGAAGGGCAGCGGCTGGG<br>.((...))	SSBP3	NM_001009955	chr1:54464783-54644680	template	23	Intron_5	--	Yes	--	Yes	--	--	--	--	--	--	--
G4ST01054510552	chr1:54510553-54510587	-	GGGCTGGGCTCTGGGCCCAGGCACTGCCCTGGGGG<br>.(((((......)))))	SSBP3	NM_018070	chr1:54464783-54644567	non-template	35	Intron_5	--	Yes	--	Yes	--	--	--	Yes	Yes	--	Yes
G4ST01054510552	chr1:54510553-54510587	-	GGGCTGGGCTCTGGGCCCAGGCACTGCCCTGGGGG<br>.(((((......)))))	SSBP3	NM_145716	chr1:54464783-54644567	non-template	35	Intron_5	--	Yes	--	Yes	--	--	--	Yes	Yes	--	Yes
G4ST01054510552	chr1:54510553-54510587	-	GGGCTGGGCTCTGGGCCCAGGCACTGCCCTGGGGG<br>.(((((......)))))	SSBP3	NM_001009955	chr1:54464783-54644680	non-template	35	Intron_5	--	Yes	--	Yes	--	--	--	Yes	Yes	--	Yes
G4ST01054513097	chr1:54513098-54513136	-	GGGGTGGCAGGGGCCTGGACTGCAGGCTGGGGTGTGGGG<br>(((((.....)))))..	SSBP3	NM_018070	chr1:54464783-54644567	non-template	39	Intron_5	--	Yes	--	Yes	--	--	--	--	--	--	--
G4ST01054513097	chr1:54513098-54513136	-	GGGGTGGCAGGGGCCTGGACTGCAGGCTGGGGTGTGGGG<br>(((((.....)))))..	SSBP3	NM_145716	chr1:54464783-54644567	non-template	39	Intron_5	--	Yes	--	Yes	--	--	--	--	--	--	--
G4ST01054513097	chr1:54513098-54513136	-	GGGGTGGCAGGGGCCTGGACTGCAGGCTGGGGTGTGGGG<br>(((((.....)))))..	SSBP3	NM_001009955	chr1:54464783-54644680	non-template	39	Intron_5	--	Yes	--	Yes	--	--	--	--	--	--	--
G4ST01054513104	chr1:54513105-54513147	-	GGGGCAAGTTTGGGGTGGCAGGGGCCTGGACTGCAGGCTGGGG<br>(((((.....)))))..	SSBP3	NM_018070	chr1:54464783-54644567	non-template	43	Intron_5	--	Yes	--	Yes	--	--	--	--	--	--	--
G4ST01054513104	chr1:54513105-54513147	-	GGGGCAAGTTTGGGGTGGCAGGGGCCTGGACTGCAGGCTGGGG<br>(((((.....)))))..	SSBP3	NM_145716	chr1:54464783-54644567	non-template	43	Intron_5	--	Yes	--	Yes	--	--	--	--	--	--	--
G4ST01054513104	chr1:54513105-54513147	-	GGGGCAAGTTTGGGGTGGCAGGGGCCTGGACTGCAGGCTGGGG<br>(((((.....)))))..	SSBP3	NM_001009955	chr1:54464783-54644680	non-template	43	Intron_5	--	Yes	--	Yes	--	--	--	--	--	--	--
G4ST01054514113	chr1:54514114-54514148	-	GGGGTTTCTCTGGGAGAAGGGCAGCCTCCTGAGGG<br>(((....))).	SSBP3	NM_018070	chr1:54464783-54644567	non-template	35	Intron_5	--	Yes	--	Yes	--	--	--	--	--	--	--
G4ST01054514113	chr1:54514114-54514148	-	GGGGTTTCTCTGGGAGAAGGGCAGCCTCCTGAGGG<br>(((....))).	SSBP3	NM_145716	chr1:54464783-54644567	non-template	35	Intron_5	--	Yes	--	Yes	--	--	--	--	--	--	--
G4ST01054514113	chr1:54514114-54514148	-	GGGGTTTCTCTGGGAGAAGGGCAGCCTCCTGAGGG<br>(((....))).	SSBP3	NM_001009955	chr1:54464783-54644680	non-template	35	Intron_5	--	Yes	--	Yes	--	--	--	--	--	--	--
G4ST01054527845	chr1:54527846-54527882	+	GGGCAGGCGCTGTGAGCGGGGATTAGGGGGATCAGGG<br>....((((...))))	SSBP3	NM_018070	chr1:54464783-54644567	template	37	Intron_4	--	Yes	--	Yes	--	--	--	Yes	--	--	--
G4ST01054527845	chr1:54527846-54527882	+	GGGCAGGCGCTGTGAGCGGGGATTAGGGGGATCAGGG<br>....((((...))))	SSBP3	NM_145716	chr1:54464783-54644567	template	37	Intron_4	--	Yes	--	Yes	--	--	--	Yes	--	--	--
G4ST01054527845	chr1:54527846-54527882	+	GGGCAGGCGCTGTGAGCGGGGATTAGGGGGATCAGGG<br>....((((...))))	SSBP3	NM_001009955	chr1:54464783-54644680	template	37	Intron_4	--	Yes	--	Yes	--	--	--	Yes	--	--	--
G4ST01054540078	chr1:54540079-54540113	-	GGGAGCAGGGGTGGCCCCGGGAGCATGTGGCTGGG<br>(((.....)))	SSBP3	NM_018070	chr1:54464783-54644567	non-template	35	Intron_4	--	Yes	--	Yes	--	--	--	--	Yes	--	Yes
G4ST01054540078	chr1:54540079-54540113	-	GGGAGCAGGGGTGGCCCCGGGAGCATGTGGCTGGG<br>(((.....)))	SSBP3	NM_145716	chr1:54464783-54644567	non-template	35	Intron_4	--	Yes	--	Yes	--	--	--	--	Yes	--	Yes
G4ST01054540078	chr1:54540079-54540113	-	GGGAGCAGGGGTGGCCCCGGGAGCATGTGGCTGGG<br>(((.....)))	SSBP3	NM_001009955	chr1:54464783-54644680	non-template	35	Intron_4	--	Yes	--	Yes	--	--	--	--	Yes	--	Yes
G4ST01054541131	chr1:54541132-54541172	-	GGGCATGGCTGGGTGTCAGGAGCCTGGCATCAGGGCCTGGG<br>.(((((....)))))....	SSBP3	NM_018070	chr1:54464783-54644567	non-template	41	Intron_4	--	Yes	--	Yes	--	--	--	--	--	--	--
G4ST01054541131	chr1:54541132-54541172	-	GGGCATGGCTGGGTGTCAGGAGCCTGGCATCAGGGCCTGGG<br>.(((((....)))))....	SSBP3	NM_145716	chr1:54464783-54644567	non-template	41	Intron_4	--	Yes	--	Yes	--	--	--	--	--	--	--
G4ST01054541131	chr1:54541132-54541172	-	GGGCATGGCTGGGTGTCAGGAGCCTGGCATCAGGGCCTGGG<br>.(((((....)))))....	SSBP3	NM_001009955	chr1:54464783-54644680	non-template	41	Intron_4	--	Yes	--	Yes	--	--	--	--	--	--	--
G4ST01054559453	chr1:54559454-54559484	+	GGGCCTTGGGGGCCCAGCCAGGGAACTCGGG<br>(((...))).	SSBP3	NM_018070	chr1:54464783-54644567	template	31	Intron_4	--	Yes	--	Yes	--	--	--	--	--	--	--
G4ST01054559453	chr1:54559454-54559484	+	GGGCCTTGGGGGCCCAGCCAGGGAACTCGGG<br>(((...))).	SSBP3	NM_145716	chr1:54464783-54644567	template	31	Intron_4	--	Yes	--	Yes	--	--	--	--	--	--	--
G4ST01054559453	chr1:54559454-54559484	+	GGGCCTTGGGGGCCCAGCCAGGGAACTCGGG<br>(((...))).	SSBP3	NM_001009955	chr1:54464783-54644680	template	31	Intron_4	--	Yes	--	Yes	--	--	--	--	--	--	--
G4ST01054575589	chr1:54575590-54575623	-	GGGCAGGCCAGGGTGGGTTATTGCAAAGCAAGGG<br>...((((...))))	SSBP3	NM_018070	chr1:54464783-54644567	non-template	34	Intron_4	--	Yes	--	Yes	--	--	--	--	--	--	--
G4ST01054575589	chr1:54575590-54575623	-	GGGCAGGCCAGGGTGGGTTATTGCAAAGCAAGGG<br>...((((...))))	SSBP3	NM_145716	chr1:54464783-54644567	non-template	34	Intron_4	--	Yes	--	Yes	--	--	--	--	--	--	--
G4ST01054575589	chr1:54575590-54575623	-	GGGCAGGCCAGGGTGGGTTATTGCAAAGCAAGGG<br>...((((...))))	SSBP3	NM_001009955	chr1:54464783-54644680	non-template	34	Intron_4	--	Yes	--	Yes	--	--	--	--	--	--	--
G4ST01054580783	chr1:54580784-54580812	-	GGGAGGCCTTGCCTGGGAATGGGAGGGGG<br>((((...))))	SSBP3	NM_018070	chr1:54464783-54644567	non-template	29	Intron_4	--	Yes	--	Yes	--	--	--	Yes	--	--	Yes
G4ST01054580783	chr1:54580784-54580812	-	GGGAGGCCTTGCCTGGGAATGGGAGGGGG<br>((((...))))	SSBP3	NM_145716	chr1:54464783-54644567	non-template	29	Intron_4	--	Yes	--	Yes	--	--	--	Yes	--	--	Yes
G4ST01054580783	chr1:54580784-54580812	-	GGGAGGCCTTGCCTGGGAATGGGAGGGGG<br>((((...))))	SSBP3	NM_001009955	chr1:54464783-54644680	non-template	29	Intron_4	--	Yes	--	Yes	--	--	--	Yes	--	--	Yes
G4ST01054580789	chr1:54580790-54580818	-	GGGTGTGGGAGGCCTTGCCTGGGAATGGG<br>((((...))))	SSBP3	NM_018070	chr1:54464783-54644567	non-template	29	Intron_4	--	Yes	--	Yes	--	--	--	Yes	--	--	Yes
G4ST01054580789	chr1:54580790-54580818	-	GGGTGTGGGAGGCCTTGCCTGGGAATGGG<br>((((...))))	SSBP3	NM_145716	chr1:54464783-54644567	non-template	29	Intron_4	--	Yes	--	Yes	--	--	--	Yes	--	--	Yes
G4ST01054580789	chr1:54580790-54580818	-	GGGTGTGGGAGGCCTTGCCTGGGAATGGG<br>((((...))))	SSBP3	NM_001009955	chr1:54464783-54644680	non-template	29	Intron_4	--	Yes	--	Yes	--	--	--	Yes	--	--	Yes
G4ST01054613181	chr1:54613182-54613220	+	GGGGCCAGAGAGGGGGAGGGGACGAGAGCATCCCGTGGG<br>.(((.((...)).)))	SSBP3	NM_018070	chr1:54464783-54644567	template	39	Intron_4	--	Yes	--	Yes	--	--	--	Yes	--	--	--
G4ST01054613181	chr1:54613182-54613220	+	GGGGCCAGAGAGGGGGAGGGGACGAGAGCATCCCGTGGG<br>.(((.((...)).)))	SSBP3	NM_145716	chr1:54464783-54644567	template	39	Intron_4	--	Yes	--	Yes	--	--	--	Yes	--	--	--
G4ST01054613181	chr1:54613182-54613220	+	GGGGCCAGAGAGGGGGAGGGGACGAGAGCATCCCGTGGG<br>.(((.((...)).)))	SSBP3	NM_001009955	chr1:54464783-54644680	template	39	Intron_4	--	Yes	--	Yes	--	--	--	Yes	--	--	--
G4ST01054618208	chr1:54618209-54618246	+	GGGGCTGGGAAAGGGGCACTCCTGGTCTCACAGTGGGG<br>.((((..((....)).))))	SSBP3	NM_018070	chr1:54464783-54644567	template	38	Intron_4	--	Yes	--	Yes	--	--	--	Yes	--	--	--
G4ST01054618208	chr1:54618209-54618246	+	GGGGCTGGGAAAGGGGCACTCCTGGTCTCACAGTGGGG<br>.((((..((....)).))))	SSBP3	NM_145716	chr1:54464783-54644567	template	38	Intron_4	--	Yes	--	Yes	--	--	--	Yes	--	--	--

G4ST01054618208	chr1:54618209-54618246	+	GGGGCTGGGAAAGGGGCACTCCTGGTCTCACAGTGGGG<br>.((((..((....)).))))	SSBP3	NM_001009955	chr1:54464783-54644680	template	38	Intron_4	--	Yes	--	Yes	--	--	--	Yes	--	--	--
G4ST01054618214	chr1:54618215-54618255	+	GGGAAAGGGGCACTCCTGGTCTCACAGTGGGGACAATGGGG<br>.((((..((....)).))))	SSBP3	NM_018070	chr1:54464783-54644567	template	41	Intron_4	--	Yes	--	Yes	--	--	--	Yes	--	--	--
G4ST01054618214	chr1:54618215-54618255	+	GGGAAAGGGGCACTCCTGGTCTCACAGTGGGGACAATGGGG<br>.((((..((....)).))))	SSBP3	NM_145716	chr1:54464783-54644567	template	41	Intron_4	--	Yes	--	Yes	--	--	--	Yes	--	--	--
G4ST01054618214	chr1:54618215-54618255	+	GGGAAAGGGGCACTCCTGGTCTCACAGTGGGGACAATGGGG<br>.((((..((....)).))))	SSBP3	NM_001009955	chr1:54464783-54644680	template	41	Intron_4	--	Yes	--	Yes	--	--	--	Yes	--	--	--
G4ST01054639166	chr1:54639167-54639203	-	GGGTAGGAATGATCCTGGGAAACCATGGGTCCTTGGG<br>.((((....))))	SSBP3	NM_018070	chr1:54464783-54644567	non-template	37	Intron_4	--	Yes	--	Yes	--	--	--	--	Yes	--	--
G4ST01054639166	chr1:54639167-54639203	-	GGGTAGGAATGATCCTGGGAAACCATGGGTCCTTGGG<br>.((((....))))	SSBP3	NM_145716	chr1:54464783-54644567	non-template	37	Intron_4	--	Yes	--	Yes	--	--	--	--	Yes	--	--
G4ST01054639166	chr1:54639167-54639203	-	GGGTAGGAATGATCCTGGGAAACCATGGGTCCTTGGG<br>.((((....))))	SSBP3	NM_001009955	chr1:54464783-54644680	non-template	37	Intron_4	--	Yes	--	Yes	--	--	--	--	Yes	--	--
G4ST01054643993	chr1:54643994-54644028	-	GGGAGCCGGCGGAGGTTGGGGGATCGGGGAAGGGG<br>..(((((....)))))	SSBP3	NM_018070	chr1:54464783-54644567	non-template	35	Intron_1.f	--	Yes	--	Yes	--	--	--	--	--	--	Yes
G4ST01054643993	chr1:54643994-54644028	-	GGGAGCCGGCGGAGGTTGGGGGATCGGGGAAGGGG<br>..(((((....)))))	SSBP3	NM_145716	chr1:54464783-54644567	non-template	35	Intron_1.f	--	Yes	--	Yes	--	--	--	--	--	--	Yes
G4ST01054643993	chr1:54643994-54644028	-	GGGAGCCGGCGGAGGTTGGGGGATCGGGGAAGGGG<br>..(((((....)))))	SSBP3	NM_001009955	chr1:54464783-54644680	non-template	35	Intron_1.f	--	Yes	--	Yes	--	--	--	--	--	--	Yes
G4ST01054644994	chr1:54644995-54645037	+	GGGGGGCTAGGGGGGCGCTGCGGCAGCGTCCGATGGGAGGGGG<br>((((((((...))))))))...	SSBP3	NM_018070	chr1:54464783-54644567	template	-427	upstream	Yes	--	--	--	--	--	--	Yes	Yes	Yes	--
G4ST01054644994	chr1:54644995-54645037	+	GGGGGGCTAGGGGGGCGCTGCGGCAGCGTCCGATGGGAGGGGG<br>((((((((...))))))))...	SSBP3	NM_145716	chr1:54464783-54644567	template	-427	upstream	Yes	--	--	--	--	--	--	Yes	Yes	Yes	--
G4ST01054644994	chr1:54644995-54645037	+	GGGGGGCTAGGGGGGCGCTGCGGCAGCGTCCGATGGGAGGGGG<br>((((((((...))))))))...	SSBP3	NM_001009955	chr1:54464783-54644680	template	-314	upstream	Yes	--	--	--	--	--	--	Yes	Yes	Yes	--
G4ST01054645043	chr1:54645044-54645078	-	GGGGCGGGAGGGAGCGAGCGGCGACCGCTTGGGGG<br>..(((((((...))))))).	SSBP3	NM_018070	chr1:54464783-54644567	non-template	-476	upstream	Yes	--	--	--	--	--	--	Yes	Yes	Yes	Yes
G4ST01054645043	chr1:54645044-54645078	-	GGGGCGGGAGGGAGCGAGCGGCGACCGCTTGGGGG<br>..(((((((...))))))).	SSBP3	NM_145716	chr1:54464783-54644567	non-template	-476	upstream	Yes	--	--	--	--	--	--	Yes	Yes	Yes	Yes
G4ST01054645043	chr1:54645044-54645078	-	GGGGCGGGAGGGAGCGAGCGGCGACCGCTTGGGGG<br>..(((((((...))))))).	SSBP3	NM_001009955	chr1:54464783-54644680	non-template	-363	upstream	Yes	--	--	--	--	--	--	Yes	Yes	Yes	Yes
G4ST01054817807	chr1:54817808-54817836	+	GGGAAGGGGCTGGGGCCACCTTTGTGGGG<br>..(((....)))	ACOT11	NM_147161	chr1:54786395-54848593	non-template	29	Intron_1.f	--	Yes	--	Yes	--	--	--	--	Yes	--	Yes
G4ST01054817807	chr1:54817808-54817836	+	GGGAAGGGGCTGGGGCCACCTTTGTGGGG<br>..(((....)))	ACOT11	NM_015547	chr1:54786395-54873005	non-template	29	Intron_1.f	--	Yes	--	Yes	--	--	--	--	Yes	--	Yes
G4ST01054850330	chr1:54850331-54850372	+	GGGGGAGGGGGCAGCTTTGGGAGTCCTATGGAAGGTAGGGGG<br>...(((((.....)))))	FAM151A	NM_176782	chr1:54847438-54861788	template	42	Intron_5	--	Yes	--	Yes	--	--	--	--	--	--	--
G4ST01054850330	chr1:54850331-54850372	+	GGGGGAGGGGGCAGCTTTGGGAGTCCTATGGAAGGTAGGGGG<br>...(((((.....)))))	ACOT11	NM_015547	chr1:54786395-54873005	non-template	42	Intron_15	--	Yes	--	Yes	--	--	--	--	--	--	--
G4ST01054850336	chr1:54850337-54850383	+	GGGGGCAGCTTTGGGAGTCCTATGGAAGGTAGGGGGTATAGTGGGGG<br>...(((((.....)))))	FAM151A	NM_176782	chr1:54847438-54861788	template	47	Intron_5	--	Yes	--	Yes	--	--	--	--	--	--	--
G4ST01054850336	chr1:54850337-54850383	+	GGGGGCAGCTTTGGGAGTCCTATGGAAGGTAGGGGGTATAGTGGGGG<br>...(((((.....)))))	ACOT11	NM_015547	chr1:54786395-54873005	non-template	47	Intron_15	--	Yes	--	Yes	--	--	--	--	--	--	--
G4ST01054850348	chr1:54850349-54850389	+	GGGAGTCCTATGGAAGGTAGGGGGTATAGTGGGGGCAGGGG<br>...(((((.....)))))	FAM151A	NM_176782	chr1:54847438-54861788	template	41	Intron_5	--	Yes	--	Yes	--	--	--	--	--	--	--
G4ST01054850348	chr1:54850349-54850389	+	GGGAGTCCTATGGAAGGTAGGGGGTATAGTGGGGGCAGGGG<br>...(((((.....)))))	ACOT11	NM_015547	chr1:54786395-54873005	non-template	41	Intron_15	--	Yes	--	Yes	--	--	--	--	--	--	--
G4ST01054858342	chr1:54858343-54858375	-	GGGGTACTGGACACAGTGGGAGCCCAGGGAGGG<br>..((((....))))	FAM151A	NM_176782	chr1:54847438-54861788	non-template	33	Intron_1.f	--	Yes	--	Yes	--	--	--	--	--	--	Yes
G4ST01054858342	chr1:54858343-54858375	-	GGGGTACTGGACACAGTGGGAGCCCAGGGAGGG<br>..((((....))))	ACOT11	NM_015547	chr1:54786395-54873005	template	33	Intron_15	--	Yes	--	Yes	--	--	--	--	--	--	Yes
G4ST01054911581	chr1:54911582-54911626	-	GGGGAGAGGCAGGAGTCTGGGACAGGGCCTGTGAGACAGACAGGG<br>...((((....)))) .((((...))))...	HEATR8	NR_026782	chr1:54880015-54948527	template	45	Intron_8	--	Yes	--	Yes	--	--	--	--	--	--	--
G4ST01054911581	chr1:54911582-54911626	-	GGGGAGAGGCAGGAGTCTGGGACAGGGCCTGTGAGACAGACAGGG<br>...((((....)))) .((((...))))...	HEATR8	NM_001039464	chr1:54880015-54948527	template	45	Intron_8	--	Yes	--	Yes	--	--	--	--	--	--	--
G4ST01054911581	chr1:54911582-54911626	-	GGGGAGAGGCAGGAGTCTGGGACAGGGCCTGTGAGACAGACAGGG<br>...((((....)))) .((((...))))...	HEATR8-TTC4	NR_037641	chr1:54880015-54980916	template	45	Intron_8	--	Yes	--	Yes	--	--	--	--	--	--	--
G4ST01054911581	chr1:54911582-54911626	-	GGGGAGAGGCAGGAGTCTGGGACAGGGCCTGTGAGACAGACAGGG<br>...((((....)))) .((((...))))...	HEATR8-TTC4	NR_037639	chr1:54880015-54980916	template	45	Intron_9	--	Yes	--	Yes	--	--	--	--	--	--	--
G4ST01054911581	chr1:54911582-54911626	-	GGGGAGAGGCAGGAGTCTGGGACAGGGCCTGTGAGACAGACAGGG<br>...((((....)))) .((((...))))...	HEATR8-TTC4	NR_037640	chr1:54880015-54980916	template	45	Intron_8	--	Yes	--	Yes	--	--	--	--	--	--	--
G4ST01054911599	chr1:54911600-54911637	-	GGGGAGAGAGTGGGGAGAGGCAGGAGTCTGGGACAGGG<br>...((((....))))	HEATR8	NR_026782	chr1:54880015-54948527	template	38	Intron_8	--	Yes	--	Yes	--	--	--	--	--	--	--
G4ST01054911599	chr1:54911600-54911637	-	GGGGAGAGAGTGGGGAGAGGCAGGAGTCTGGGACAGGG<br>...((((....))))	HEATR8	NM_001039464	chr1:54880015-54948527	template	38	Intron_8	--	Yes	--	Yes	--	--	--	--	--	--	--
G4ST01054911599	chr1:54911600-54911637	-	GGGGAGAGAGTGGGGAGAGGCAGGAGTCTGGGACAGGG<br>...((((....))))	HEATR8-TTC4	NR_037641	chr1:54880015-54980916	template	38	Intron_8	--	Yes	--	Yes	--	--	--	--	--	--	--
G4ST01054911599	chr1:54911600-54911637	-	GGGGAGAGAGTGGGGAGAGGCAGGAGTCTGGGACAGGG<br>...((((....))))	HEATR8-TTC4	NR_037639	chr1:54880015-54980916	template	38	Intron_9	--	Yes	--	Yes	--	--	--	--	--	--	--
G4ST01054911599	chr1:54911600-54911637	-	GGGGAGAGAGTGGGGAGAGGCAGGAGTCTGGGACAGGG<br>...((((....))))	HEATR8-TTC4	NR_037640	chr1:54880015-54980916	template	38	Intron_8	--	Yes	--	Yes	--	--	--	--	--	--	--
G4ST01054911605	chr1:54911606-54911645	-	GGGGGGATGGGGAGAGAGTGGGGAGAGGCAGGAGTCTGGG<br>...((((....))))	HEATR8	NR_026782	chr1:54880015-54948527	template	40	Intron_8	--	Yes	--	Yes	--	--	--	--	--	--	--
G4ST01054911605	chr1:54911606-54911645	-	GGGGGGATGGGGAGAGAGTGGGGAGAGGCAGGAGTCTGGG<br>...((((....))))	HEATR8	NM_001039464	chr1:54880015-54948527	template	40	Intron_8	--	Yes	--	Yes	--	--	--	--	--	--	--
G4ST01054911605	chr1:54911606-54911645	-	GGGGGGATGGGGAGAGAGTGGGGAGAGGCAGGAGTCTGGG<br>...((((....))))	HEATR8-TTC4	NR_037641	chr1:54880015-54980916	template	40	Intron_8	--	Yes	--	Yes	--	--	--	--	--	--	--
G4ST01054911605	chr1:54911606-54911645	-	GGGGGGATGGGGAGAGAGTGGGGAGAGGCAGGAGTCTGGG<br>...((((....))))	HEATR8-TTC4	NR_037639	chr1:54880015-54980916	template	40	Intron_9	--	Yes	--	Yes	--	--	--	--	--	--	--
G4ST01054911605	chr1:54911606-54911645	-	GGGGGGATGGGGAGAGAGTGGGGAGAGGCAGGAGTCTGGG<br>...((((....))))	HEATR8-TTC4	NR_037640	chr1:54880015-54980916	template	40	Intron_8	--	Yes	--	Yes	--	--	--	--	--	--	--
G4ST01054911622	chr1:54911623-54911664	-	GGGCAGCCATTTAGAGAGTGGGGGGATGGGGAGAGAGTGGGG<br>...(((((.....))))).	HEATR8	NR_026782	chr1:54880015-54948527	template	42	Intron_8	--	Yes	--	Yes	--	--	--	--	--	--	--
G4ST01054911622	chr1:54911623-54911664	-	GGGCAGCCATTTAGAGAGTGGGGGGATGGGGAGAGAGTGGGG<br>...(((((.....))))).	HEATR8	NM_001039464	chr1:54880015-54948527	template	42	Intron_8	--	Yes	--	Yes	--	--	--	--	--	--	--
G4ST01054911622	chr1:54911623-54911664	-	GGGCAGCCATTTAGAGAGTGGGGGGATGGGGAGAGAGTGGGG<br>...(((((.....))))).	HEATR8-TTC4	NR_037641	chr1:54880015-54980916	template	42	Intron_8	--	Yes	--	Yes	--	--	--	--	--	--	--
G4ST01054911622	chr1:54911623-54911664	-	GGGCAGCCATTTAGAGAGTGGGGGGATGGGGAGAGAGTGGGG<br>...(((((.....))))).	HEATR8-TTC4	NR_037639	chr1:54880015-54980916	template	42	Intron_9	--	Yes	--	Yes	--	--	--	--	--	--	--
G4ST01054911622	chr1:54911623-54911664	-	GGGCAGCCATTTAGAGAGTGGGGGGATGGGGAGAGAGTGGGG<br>...(((((.....))))).	HEATR8-TTC4	NR_037640	chr1:54880015-54980916	template	42	Intron_8	--	Yes	--	Yes	--	--	--	--	--	--	--
G4ST01054917712	chr1:54917713-54917746	+	GGGGGCAGGGAGTGAACTGTCACTGGGGCTCGGG<br>(((((....))))).	HEATR8	NR_026782	chr1:54880015-54948527	non-template	34	Intron_12	--	Yes	--	Yes	--	--	--	--	--	--	--
G4ST01054917712	chr1:54917713-54917746	+	GGGGGCAGGGAGTGAACTGTCACTGGGGCTCGGG<br>(((((....))))).	HEATR8	NM_001039464	chr1:54880015-54948527	non-template	34	Intron_12	--	Yes	--	Yes	--	--	--	--	--	--	--
G4ST01054917712	chr1:54917713-54917746	+	GGGGGCAGGGAGTGAACTGTCACTGGGGCTCGGG<br>(((((....))))).	HEATR8-TTC4	NR_037641	chr1:54880015-54980916	non-template	34	Intron_12	--	Yes	--	Yes	--	--	--	--	--	--	--
G4ST01054917712	chr1:54917713-54917746	+	GGGGGCAGGGAGTGAACTGTCACTGGGGCTCGGG<br>(((((....))))).	HEATR8-TTC4	NR_037639	chr1:54880015-54980916	non-template	34	Intron_13	--	Yes	--	Yes	--	--	--	--	--	--	--

G4ST01054917712	chr1:54917713-54917746	+	GGGGGCAGGGAGTGAACTGTCACTGGGGCTCGGG<br>(((((....))))).	HEATR8-TTC4	NR_037640	chr1:54880015-54980916	non-template	34	Intron_12	--	Yes	--	Yes	--	--	--	--	--	--	--
G4ST01054947408	chr1:54947409-54947441	+	GGGAAAGGGAAGGGATGTGTCCCAAGACAAGGG<br>...((((....)))).	HEATR8	NR_026782	chr1:54880015-54948527	non-template	33	Intron_23.l	--	Yes	--	Yes	--	--	--	--	--	--	Yes
G4ST01054947408	chr1:54947409-54947441	+	GGGAAAGGGAAGGGATGTGTCCCAAGACAAGGG<br>...((((....)))).	HEATR8	NM_001039464	chr1:54880015-54948527	non-template	33	Intron_23.l	--	Yes	--	Yes	--	--	--	--	--	--	Yes
G4ST01054947408	chr1:54947409-54947441	+	GGGAAAGGGAAGGGATGTGTCCCAAGACAAGGG<br>...((((....)))).	HEATR8-TTC4	NR_037641	chr1:54880015-54980916	non-template	33	Intron_22	--	Yes	--	Yes	--	--	--	--	--	--	Yes
G4ST01054947408	chr1:54947409-54947441	+	GGGAAAGGGAAGGGATGTGTCCCAAGACAAGGG<br>...((((....)))).	HEATR8-TTC4	NR_037639	chr1:54880015-54980916	non-template	33	Intron_23	--	Yes	--	Yes	--	--	--	--	--	--	Yes
G4ST01054947408	chr1:54947409-54947441	+	GGGAAAGGGAAGGGATGTGTCCCAAGACAAGGG<br>...((((....)))).	HEATR8-TTC4	NR_037640	chr1:54880015-54980916	non-template	33	Intron_23	--	Yes	--	Yes	--	--	--	--	--	--	Yes
G4ST01055019462	chr1:55019463-55019493	+	GGGCGGGGTCCCAGGGAGCCTCCGGCCTGGG<br>.(((...)))..	TTC22	NM_001114108	chr1:55019340-55039529	template	31	Exon_7.l	--	Yes	Yes	--	--	--	Yes	--	Yes	--	Yes
G4ST01055026622	chr1:55026623-55026651	-	GGGGTTGGGGGGCCTGTGGCGGGGTGGGG<br>..(((...))).	TTC22	NM_017904	chr1:55022812-55039529	non-template	29	Intron_1.f	--	Yes	--	Yes	--	--	--	--	--	--	--
G4ST01055026622	chr1:55026623-55026651	-	GGGGTTGGGGGGCCTGTGGCGGGGTGGGG<br>..(((...))).	TTC22	NM_001114108	chr1:55019340-55039529	non-template	29	Intron_1.f	--	Yes	--	Yes	--	--	--	--	--	--	--
G4ST01055038798	chr1:55038799-55038840	+	GGGACCATGGGAGGACCACAGTCCTCCCAGAGGGAGGTAGGG<br>(((((....)))))......	TTC22	NM_017904	chr1:55022812-55039529	template	42	Intron_1.f	--	Yes	--	Yes	--	--	--	--	--	--	--
G4ST01055038798	chr1:55038799-55038840	+	GGGACCATGGGAGGACCACAGTCCTCCCAGAGGGAGGTAGGG<br>(((((....)))))......	TTC22	NM_001114108	chr1:55019340-55039529	template	42	Intron_1.f	--	Yes	--	Yes	--	--	--	--	--	--	--
G4ST01055038806	chr1:55038807-55038849	+	GGGAGGACCACAGTCCTCCCAGAGGGAGGTAGGGAGACAGGGG<br>(((((....)))))......	TTC22	NM_017904	chr1:55022812-55039529	template	43	Intron_1.f	--	Yes	--	Yes	--	--	--	--	--	--	--
G4ST01055038806	chr1:55038807-55038849	+	GGGAGGACCACAGTCCTCCCAGAGGGAGGTAGGGAGACAGGGG<br>(((((....)))))......	TTC22	NM_001114108	chr1:55019340-55039529	template	43	Intron_1.f	--	Yes	--	Yes	--	--	--	--	--	--	--
G4ST01055064190	chr1:55064191-55064223	+	GGGCCCCTGGGAGATGCATGCATGGGCAGTGGG<br>..(((....)))	C1orf177	NM_001110533	chr1:55044324-55080525	non-template	33	Intron_9.l	--	Yes	--	Yes	--	--	--	--	--	--	--
G4ST01055064190	chr1:55064191-55064223	+	GGGCCCCTGGGAGATGCATGCATGGGCAGTGGG<br>..(((....)))	C1orf177	NM_152607	chr1:55044324-55080525	non-template	33	Intron_9	--	Yes	--	Yes	--	--	--	--	--	--	--
G4ST01055089556	chr1:55089557-55089594	-	GGGAGTGGGTCCGCGCTGCTCTGCAGCAGGGGCTGGGG<br>.....(((((...)))))..	DHCR24	NM_014762	chr1:55087888-55125509	non-template	38	Exon_9.l	--	Yes	Yes	--	--	--	Yes	--	--	Yes	--
G4ST01055219015	chr1:55219016-55219049	+	GGGGAGCGCGCAGCCCTCGGGGCGGGCGCCGGGG<br>(((.((...)).))).	TMEM61	NM_182532	chr1:55219053-55230554	non-template	-3	upstream	Yes	--	--	--	--	--	--	Yes	Yes	Yes	--
G4ST01055229284	chr1:55229285-55229311	-	GGGACACACAGTGGGGAGTGGGGTGGG<br>.(((...)))	TMEM61	NM_182532	chr1:55219053-55230554	template	27	Intron_2.l	--	Yes	--	Yes	--	--	--	--	--	--	--
G4ST01055236018	chr1:55236019-55236059	+	GGGGCAGCTGTGAGCACAGGTGTGGGGTTAGGGAACAAGGG<br>.((.((((....)))).))..	BSND	NM_057176	chr1:55237205-55247053	non-template	-1145	upstream	Yes	--	--	--	--	--	--	Yes	--	--	--
G4ST01055278447	chr1:55278448-55278489	+	GGGGACTGCAGGCAAGGCGGCGGGGGAGGACGGGTAGTGGGG<br>..((((..((...)).))))	PCSK9	NM_174936	chr1:55277737-55303114	non-template	42	Intron_1.f	--	Yes	--	Yes	--	--	--	--	Yes	Yes	--
G4ST01055284654	chr1:55284655-55284683	+	GGGACAAGGTGGGAGGCTGCTGGGCTGGG<br>((....))	PCSK9	NM_174936	chr1:55277737-55303114	non-template	29	Intron_2	--	Yes	--	Yes	--	--	--	--	Yes	Yes	--
G4ST01055284664	chr1:55284665-55284691	+	GGGAGGCTGCTGGGCTGGGATGTGGGG<br>((....))	PCSK9	NM_174936	chr1:55277737-55303114	non-template	27	Intron_2	--	Yes	--	Yes	--	--	--	--	Yes	Yes	--
G4ST01055294459	chr1:55294460-55294497	+	GGGGCTGCTGCCCCAAGGCGCGGGTAGGGGGCGGAGGG<br>...((.(((....)))))	PCSK9	NM_174936	chr1:55277737-55303114	non-template	38	Intron_6	--	Yes	--	Yes	--	--	--	--	--	--	Yes
G4ST01055294747	chr1:55294748-55294783	-	GGGGAGGGGGCGGGTACCAGAAGACCTTCAGGAGGG<br>..((.(((....))).)).	PCSK9	NM_174936	chr1:55277737-55303114	template	36	Intron_6	--	Yes	--	Yes	--	--	--	--	--	Yes	--
G4ST01055308424	chr1:55308425-55308456	+	GGGCCAGCTTTGGTGGGCGCAGGGAGGAGGGG<br>(((....))).	USP24	NM_015306	chr1:55304620-55453627	template	32	Intron_67.l	--	Yes	--	Yes	--	--	--	--	Yes	Yes	Yes
G4ST01055317857	chr1:55317858-55317894	+	GGGGCAACAGAGGGCTTGAGGGAGCAATGCTAATGGG<br>(((...)))...	USP24	NM_015306	chr1:55304620-55453627	template	37	Exon_60	--	Yes	Yes	--	Yes	--	--	--	Yes	--	--
G4ST01055454024	chr1:55454025-55454052	-	GGGTCGGGCGGCGCCGGCCAGGGAGGGG<br>.(((....))).	USP24	NM_015306	chr1:55304620-55453627	non-template	-397	upstream	Yes	--	--	--	--	--	--	Yes	Yes	Yes	--
G4ST01055454029	chr1:55454030-55454060	-	GGGGGAGCGGGTCGGGCGGCGCCGGCCAGGG<br>.(((....))).	USP24	NM_015306	chr1:55304620-55453627	non-template	-402	upstream	Yes	--	--	--	--	--	--	Yes	Yes	Yes	--
G4ST01056737677	chr1:56737678-56737720	-	GGGCTCAAAGCTAAGTGCTTTGGGTGATTCAGGGTGATTAGGG<br>...(((((.....)))))	PPAP2B	NM_003713	chr1:56733007-56817845	non-template	43	Intron_5.l	--	Yes	--	Yes	--	--	--	--	--	--	--
G4ST01056883797	chr1:56883798-56883830	+	GGGGAGAGGCGGGAGCCCCGGGCCTCCGGCGGG<br>((...)).	PRKAA2	NM_006252	chr1:56883578-56953596	non-template	33	Intron_1.f	--	Yes	--	Yes	--	--	--	Yes	Yes	Yes	Yes
G4ST01056883807	chr1:56883808-56883837	+	GGGAGCCCCGGGCCTCCGGCGGGCGCGGGG<br>((...)).	PRKAA2	NM_006252	chr1:56883578-56953596	non-template	30	Intron_1.f	--	Yes	--	Yes	--	--	--	Yes	Yes	Yes	Yes
G4ST01057057660	chr1:57057661-57057700	-	GGGGCAGGTGGGGAGCCTGGGCTCAGGTGTGTCCTGTGGG<br>..((((.....)))).	C1orf168	NM_001004303	chr1:56957065-57057957	non-template	40	Exon_1.f	--	Yes	Yes	--	--	Yes	--	Yes	Yes	--	--
G4ST01057102980	chr1:57102981-57103014	+	GGGCAATGAAGTTTTCTTTGAAGGGATGGGAGGG<br>(((.((.....)).)))..	C8A	NM_000562	chr1:57093031-57156482	non-template	34	Intron_1.f	--	Yes	--	Yes	--	--	--	--	--	--	--
G4ST01057338999	chr1:57339000-57339031	+	GGGAGCTCGCCAAGCTGGGGTGGGGGTGGGGG<br>((((.....)))).	DAB1	NM_021080	chr1:57236167-58488799	template	32	Intron_6	--	Yes	--	Yes	--	--	--	Yes	--	--	Yes
G4ST01057365043	chr1:57365044-57365079	-	GGGACTGGGTAACCAGGATTGAGCCTGGGGGCTGGG<br>...(((((......)))))	DAB1	NM_021080	chr1:57236167-58488799	non-template	36	Intron_6	--	Yes	--	Yes	--	--	--	--	--	--	--
G4ST01057451702	chr1:57451703-57451741	+	GGGTCAGGGTGGACAGAGTCCTGGCCTTTGGGCCCTGGG<br>.((.(((....))).))...	DAB1	NM_021080	chr1:57236167-58488799	template	39	Intron_4	--	Yes	--	Yes	--	--	--	--	--	--	--
G4ST01057606201	chr1:57606202-57606237	-	GGGCAGGGATGCCTGGGTCCACAGCTGTGGTTTGGG<br>.((((....)))).. .	DAB1	NM_021080	chr1:57236167-58488799	non-template	36	Intron_3	--	Yes	--	Yes	--	--	--	--	--	--	--
G4ST01057661743	chr1:57661744-57661772	+	GGGTCTGGCCACCAGCGAGGGAGGGAGGG<br>.((((...))))...	DAB1	NM_021080	chr1:57236167-58488799	template	29	Intron_3	--	Yes	--	Yes	--	--	--	Yes	--	--	Yes
G4ST01057700138	chr1:57700139-57700172	+	GGGCTCTTTGCTAAGGGGAGGTGGGAGTGGAGGG<br>..(((....)))	DAB1	NM_021080	chr1:57236167-58488799	template	34	Intron_3	--	Yes	--	Yes	--	--	--	--	--	--	--
G4ST01057775021	chr1:57775022-57775055	-	GGGGGTGGGAGTGGGGCTGCGGCAGCAGAAAGGG<br>.((((....))))...	DAB1	NM_021080	chr1:57236167-58488799	non-template	34	Intron_3	--	Yes	--	Yes	--	--	--	--	--	--	--
G4ST01058105509	chr1:58105510-58105548	+	GGGTTAAAGGGAAGGCCTCGCCTCTTAGGGAAATATGGG<br>.((((...))))....	DAB1	NM_021080	chr1:57236167-58488799	template	39	Intron_2	--	Yes	--	Yes	--	--	--	--	--	--	--
G4ST01058227765	chr1:58227766-58227792	-	GGGGTGAGGGCATCTTCATGGGGTGGG<br>(((....)))	DAB1	NM_021080	chr1:57236167-58488799	non-template	27	Intron_1.f	--	Yes	--	Yes	--	--	--	--	--	--	--
G4ST01058256671	chr1:58256672-58256701	-	GGGAGCCAAAGGCTGTGGGAGGGCCTAGGG<br>((((...))))..	DAB1	NM_021080	chr1:57236167-58488799	non-template	30	Intron_1.f	--	Yes	--	Yes	--	--	--	--	--	--	Yes
G4ST01058376826	chr1:58376827-58376859	+	GGGGCACTGGGCTTGAAAGAAGTGGGTTGGGGG<br>(((.....))).	DAB1	NM_021080	chr1:57236167-58488799	template	33	Intron_1.f	--	Yes	--	Yes	--	--	--	--	--	--	--
G4ST01058407507	chr1:58407508-58407536	+	GGGTCCAGGGACCATTGTGGGTTCTTGGG<br>((....))	DAB1	NM_021080	chr1:57236167-58488799	template	29	Intron_1.f	--	Yes	--	Yes	--	--	--	Yes	--	--	--
G4ST01058407587	chr1:58407588-58407616	+	GGGTCCAGGGACCATTGTGGGTTCTTGGG<br>((....))	DAB1	NM_021080	chr1:57236167-58488799	template	29	Intron_1.f	--	Yes	--	Yes	--	--	--	Yes	--	--	--
G4ST01058407594	chr1:58407595-58407623	+	GGGACCATTGTGGGTTCTTGGGCAGGGGG<br>((....))	DAB1	NM_021080	chr1:57236167-58488799	template	29	Intron_1.f	--	Yes	--	Yes	--	--	--	Yes	--	--	--
G4ST01058488278	chr1:58488279-58488314	-	GGGAGCCTCCTGGGTGGGAACTGGAGGCAGTGGGGG<br>((....)) .((((....))))..	DAB1	NM_021080	chr1:57236167-58488799	non-template	36	Intron_1.f	--	Yes	--	Yes	--	--	--	Yes	--	--	--
G4ST01058785063	chr1:58785064-58785106	-	GGGAGAACGACTCTGGGCGGCTGTATTGACTGCCGGGTGTGGG<br>(((.....))) .(((.((....)).)))	OMA1	NM_145243	chr1:58718979-58785034	non-template	-29	upstream	Yes	--	--	--	--	--	--	Yes	Yes	Yes	--
G4ST01058785069	chr1:58785070-58785115	-	GGGGCAGCTGGGAGAACGACTCTGGGCGGCTGTATTGACTGCCGGG<br>(((.....))) .(((.((....)).)))	OMA1	NM_145243	chr1:58718979-58785034	non-template	-35	upstream	Yes	--	--	--	--	--	--	Yes	Yes	Yes	--

G4ST01058785089	chr1:58785090-58785122	-	GGGGCCCGGGGCAGCTGGGAGAACGACTCTGGG<br>(((.....)))	OMA1	NM_145243	chr1:58718979-58785034	non-template	-55	upstream	Yes	--	--	--	--	--	--	Yes	Yes	Yes	--
G4ST01059052816	chr1:59052817-59052849	+	GGGGCTCTGGGTCGGTGGTCACCGGGCGTGGGG<br>..(((....)))	LOC100131060	NR_034014	chr1:59023411-59137972	non-template	33	Intron_1.f	--	Yes	--	Yes	--	--	--	--	Yes	Yes	--
G4ST01059052816	chr1:59052817-59052849	+	GGGGCTCTGGGTCGGTGGTCACCGGGCGTGGGG<br>..(((....)))	LOC100131060	NR_034015	chr1:59023411-59137972	non-template	33	Intron_1.f	--	Yes	--	Yes	--	--	--	--	Yes	Yes	--
G4ST01059063528	chr1:59063529-59063563	-	GGGTGAGGCAGGGAGGGGAGGCATTGCCCTCTGGG<br>((((......)))).	LOC100131060	NR_034014	chr1:59023411-59137972	template	35	Intron_1.f	--	Yes	--	Yes	--	--	--	Yes	Yes	--	Yes
G4ST01059063528	chr1:59063529-59063563	-	GGGTGAGGCAGGGAGGGGAGGCATTGCCCTCTGGG<br>((((......)))).	LOC100131060	NR_034015	chr1:59023411-59137972	template	35	Intron_1.f	--	Yes	--	Yes	--	--	--	Yes	Yes	--	Yes
G4ST01059099807	chr1:59099808-59099836	+	GGGTCAGAGGGCAGTGGGAAGAGCTTGGG<br>((....))	LOC100131060	NR_034014	chr1:59023411-59137972	non-template	29	Intron_1.f	--	Yes	--	Yes	--	--	--	--	--	--	--
G4ST01059099807	chr1:59099808-59099836	+	GGGTCAGAGGGCAGTGGGAAGAGCTTGGG<br>((....))	LOC100131060	NR_034015	chr1:59023411-59137972	non-template	29	Intron_1.f	--	Yes	--	Yes	--	--	--	--	--	--	--
G4ST01059131732	chr1:59131733-59131771	-	GGGGGCCATGGGGCCTCTGCTCAAGGCGGGAATCTCGGG<br>((((.......))))	LOC100131060	NR_034014	chr1:59023411-59137972	template	39	Intron_1.f	--	Yes	--	Yes	--	--	--	Yes	--	--	--
G4ST01059131732	chr1:59131733-59131771	-	GGGGGCCATGGGGCCTCTGCTCAAGGCGGGAATCTCGGG<br>((((.......))))	LOC100131060	NR_034015	chr1:59023411-59137972	template	39	Intron_1.f	--	Yes	--	Yes	--	--	--	Yes	--	--	--
G4ST01059377146	chr1:59377147-59377185	+	GGGATTGAGGGTCAGGGAGGTTAAGCAACTTGCTTTGGG<br>.....(((((...))))).	LOC729467	NR_027120	chr1:59370196-59385067	template	39	Intron_1.f	--	Yes	--	Yes	--	--	--	Yes	--	--	--
G4ST01059385161	chr1:59385162-59385194	+	GGGGGCGCTAGGCCAGGGACCGCCGGGCCAGGG<br>(((.....))).	LOC729467	NR_027120	chr1:59370196-59385067	template	-94	upstream	Yes	--	--	--	--	--	--	Yes	Yes	Yes	--
G4ST01059385269	chr1:59385270-59385313	+	GGGAGGCGGGCGTTCCGGCGGAGGGTCACGGCCTCGCCGCCGGG<br>(((....))).. ...((((...))))..	LOC729467	NR_027120	chr1:59370196-59385067	template	-202	upstream	Yes	--	--	--	--	--	--	Yes	Yes	Yes	--
G4ST01059817535	chr1:59817536-59817564	-	GGGGAAATGGGCAGGGAAGAGGTCTTGGG<br>(((....)))	FGGY	NM_018291	chr1:59535213-60000990	template	29	Intron_8	--	Yes	--	Yes	--	--	--	--	--	--	--
G4ST01059817535	chr1:59817536-59817564	-	GGGGAAATGGGCAGGGAAGAGGTCTTGGG<br>(((....)))	FGGY	NM_001113411	chr1:59535213-60000990	template	29	Intron_8	--	Yes	--	Yes	--	--	--	--	--	--	--
G4ST01060053055	chr1:60053056-60053085	+	GGGCGGCGGGGCGAGGTGTGCGGGAGTGGG<br>(((.....)))	HOOK1	NM_015888	chr1:60053121-60114638	non-template	-35	upstream	Yes	--	--	--	--	--	--	Yes	Yes	Yes	Yes
G4ST01060053062	chr1:60053063-60053094	+	GGGGCGAGGTGTGCGGGAGTGGGCAGGACGGG<br>(((.....)))	HOOK1	NM_015888	chr1:60053121-60114638	non-template	-26	upstream	Yes	--	--	--	--	--	--	Yes	Yes	Yes	Yes
G4ST01060053617	chr1:60053618-60053646	+	GGGCGCGGGTTGGTGTCCGGGGGTGGGGG<br>.(((...))).	HOOK1	NM_015888	chr1:60053121-60114638	non-template	29	Intron_1.f	--	Yes	--	Yes	--	--	--	--	Yes	--	--
G4ST01060164706	chr1:60164707-60164738	-	GGGACGTTCCGGCAACGTGGGTGGGGTGGGGG<br>(((((.....)))))	CYP2J2	NM_000775	chr1:60131568-60165011	non-template	32	Intron_1.f	--	Yes	--	Yes	--	--	--	Yes	Yes	Yes	Yes
G4ST01061321140	chr1:61321141-61321175	+	GGGTGGGGGGCACCGGGGCCGTCGCTCCGGCCGGG<br>((((......)))).	NFIA	NM_001145512	chr1:61320122-61701048	non-template	35	Intron_2	--	Yes	--	Yes	--	--	--	Yes	Yes	Yes	Yes
G4ST01061321140	chr1:61321141-61321175	+	GGGTGGGGGGCACCGGGGCCGTCGCTCCGGCCGGG<br>((((......)))).	NFIA	NM_001134673	chr1:61320568-61701048	non-template	35	Intron_1.f	--	Yes	--	Yes	--	--	--	Yes	Yes	Yes	Yes
G4ST01061321140	chr1:61321141-61321175	+	GGGTGGGGGGCACCGGGGCCGTCGCTCCGGCCGGG<br>((((......)))).	NFIA	NM_005595	chr1:61320568-61701048	non-template	35	Intron_1.f	--	Yes	--	Yes	--	--	--	Yes	Yes	Yes	Yes
G4ST01061321140	chr1:61321141-61321175	+	GGGTGGGGGGCACCGGGGCCGTCGCTCCGGCCGGG<br>((((......)))).	NFIA	NM_001145511	chr1:61315534-61701048	non-template	35	Intron_1.f	--	Yes	--	Yes	--	--	--	Yes	Yes	Yes	Yes
G4ST01061321144	chr1:61321145-61321182	+	GGGGGGCACCGGGGCCGTCGCTCCGGCCGGGCCCAGGG<br>((((......)))).	NFIA	NM_001145512	chr1:61320122-61701048	non-template	38	Intron_2	--	Yes	--	Yes	--	--	--	Yes	Yes	Yes	Yes
G4ST01061321144	chr1:61321145-61321182	+	GGGGGGCACCGGGGCCGTCGCTCCGGCCGGGCCCAGGG<br>((((......)))).	NFIA	NM_001134673	chr1:61320568-61701048	non-template	38	Intron_1.f	--	Yes	--	Yes	--	--	--	Yes	Yes	Yes	Yes
G4ST01061321144	chr1:61321145-61321182	+	GGGGGGCACCGGGGCCGTCGCTCCGGCCGGGCCCAGGG<br>((((......)))).	NFIA	NM_005595	chr1:61320568-61701048	non-template	38	Intron_1.f	--	Yes	--	Yes	--	--	--	Yes	Yes	Yes	Yes
G4ST01061321144	chr1:61321145-61321182	+	GGGGGGCACCGGGGCCGTCGCTCCGGCCGGGCCCAGGG<br>((((......)))).	NFIA	NM_001145511	chr1:61315534-61701048	non-template	38	Intron_1.f	--	Yes	--	Yes	--	--	--	Yes	Yes	Yes	Yes
G4ST01061321599	chr1:61321600-61321642	-	GGGGGGCTGCGGCAGCGCAGGCTGCGGGGAGCGGGCCGTTGGG<br>.(.(((((....))))).)....	NFIA	NM_001145512	chr1:61320122-61701048	template	43	Intron_2	--	Yes	--	Yes	--	--	--	Yes	Yes	Yes	--
G4ST01061321599	chr1:61321600-61321642	-	GGGGGGCTGCGGCAGCGCAGGCTGCGGGGAGCGGGCCGTTGGG<br>.(.(((((....))))).)....	NFIA	NM_001134673	chr1:61320568-61701048	template	43	Intron_1.f	--	Yes	--	Yes	--	--	--	Yes	Yes	Yes	--
G4ST01061321599	chr1:61321600-61321642	-	GGGGGGCTGCGGCAGCGCAGGCTGCGGGGAGCGGGCCGTTGGG<br>.(.(((((....))))).)....	NFIA	NM_005595	chr1:61320568-61701048	template	43	Intron_1.f	--	Yes	--	Yes	--	--	--	Yes	Yes	Yes	--
G4ST01061321599	chr1:61321600-61321642	-	GGGGGGCTGCGGCAGCGCAGGCTGCGGGGAGCGGGCCGTTGGG<br>.(.(((((....))))).)....	NFIA	NM_001145511	chr1:61315534-61701048	template	43	Intron_1.f	--	Yes	--	Yes	--	--	--	Yes	Yes	Yes	--
G4ST01061525889	chr1:61525890-61525924	-	GGGGGGGGTTGGGGACAGTCTTGCTCTGTCACGGG<br>(((((.......)))))..	NFIA	NM_001145512	chr1:61320122-61701048	template	35	Intron_4	--	Yes	--	Yes	--	--	--	--	--	--	--
G4ST01061525889	chr1:61525890-61525924	-	GGGGGGGGTTGGGGACAGTCTTGCTCTGTCACGGG<br>(((((.......)))))..	NFIA	NM_001134673	chr1:61320568-61701048	template	35	Intron_3	--	Yes	--	Yes	--	--	--	--	--	--	--
G4ST01061525889	chr1:61525890-61525924	-	GGGGGGGGTTGGGGACAGTCTTGCTCTGTCACGGG<br>(((((.......)))))..	NFIA	NM_005595	chr1:61320568-61701048	template	35	Intron_3	--	Yes	--	Yes	--	--	--	--	--	--	--
G4ST01061525889	chr1:61525890-61525924	-	GGGGGGGGTTGGGGACAGTCTTGCTCTGTCACGGG<br>(((((.......)))))..	NFIA	NM_001145511	chr1:61315534-61701048	template	35	Intron_3	--	Yes	--	Yes	--	--	--	--	--	--	--
G4ST01061625285	chr1:61625286-61625320	-	GGGCCTTTGGGAGGTGATTAGGTCACAGGGTGGGG<br>..((((.....)))).	NFIA	NM_001145512	chr1:61320122-61701048	template	35	Intron_8	--	Yes	--	Yes	--	--	--	Yes	--	--	--
G4ST01061625285	chr1:61625286-61625320	-	GGGCCTTTGGGAGGTGATTAGGTCACAGGGTGGGG<br>..((((.....)))).	NFIA	NM_001134673	chr1:61320568-61701048	template	35	Intron_7	--	Yes	--	Yes	--	--	--	Yes	--	--	--
G4ST01061625285	chr1:61625286-61625320	-	GGGCCTTTGGGAGGTGATTAGGTCACAGGGTGGGG<br>..((((.....)))).	NFIA	NM_005595	chr1:61320568-61701048	template	35	Intron_7	--	Yes	--	Yes	--	--	--	Yes	--	--	--
G4ST01061625285	chr1:61625286-61625320	-	GGGCCTTTGGGAGGTGATTAGGTCACAGGGTGGGG<br>..((((.....)))).	NFIA	NM_001145511	chr1:61315534-61701048	template	35	Intron_7	--	Yes	--	Yes	--	--	--	Yes	--	--	--
G4ST01061677389	chr1:61677390-61677429	-	GGGGGCTTGGGGGTGCACTCAGGTAACACAGGGCAAAGGG<br>.(((.((....))..))).	NFIA	NM_001145512	chr1:61320122-61701048	template	40	Intron_11.l	--	Yes	--	Yes	--	--	--	--	--	--	--
G4ST01061677389	chr1:61677390-61677429	-	GGGGGCTTGGGGGTGCACTCAGGTAACACAGGGCAAAGGG<br>.(((.((....))..))).	NFIA	NM_001134673	chr1:61320568-61701048	template	40	Intron_10.l	--	Yes	--	Yes	--	--	--	--	--	--	--
G4ST01061677389	chr1:61677390-61677429	-	GGGGGCTTGGGGGTGCACTCAGGTAACACAGGGCAAAGGG<br>.(((.((....))..))).	NFIA	NM_005595	chr1:61320568-61701048	template	40	Intron_9.l	--	Yes	--	Yes	--	--	--	--	--	--	--
G4ST01061677389	chr1:61677390-61677429	-	GGGGGCTTGGGGGTGCACTCAGGTAACACAGGGCAAAGGG<br>.(((.((....))..))).	NFIA	NM_001145511	chr1:61315534-61701048	template	40	Intron_10.l	--	Yes	--	Yes	--	--	--	--	--	--	--
G4ST01062479549	chr1:62479550-62479583	+	GGGGAGAGGATGGGCACTGTGTGCCAGGGCGGGG<br>(((...)))...	KANK4	NM_181712	chr1:62474425-62557671	template	34	Intron_9.l	--	Yes	--	Yes	--	--	--	--	Yes	--	--
G4ST01062674234	chr1:62674235-62674279	+	GGGCTCGCGCGGGAACCCTGGGAAGCTCCCGCGGCCGGAGCTGGG<br>.((((((.......))))))	USP1	NM_003368	chr1:62674971-62690063	non-template	-691	upstream	Yes	--	--	--	--	--	--	Yes	Yes	Yes	Yes
G4ST01062674234	chr1:62674235-62674279	+	GGGCTCGCGCGGGAACCCTGGGAAGCTCCCGCGGCCGGAGCTGGG<br>.((((((.......))))))	USP1	NM_001017416	chr1:62674563-62690063	non-template	-283	upstream	Yes	--	--	--	--	--	--	Yes	Yes	Yes	Yes
G4ST01062674234	chr1:62674235-62674279	+	GGGCTCGCGCGGGAACCCTGGGAAGCTCCCGCGGCCGGAGCTGGG<br>.((((((.......))))))	USP1	NM_001017415	chr1:62674563-62690063	non-template	-283	upstream	Yes	--	--	--	--	--	--	Yes	Yes	Yes	Yes
G4ST01063023070	chr1:63023071-63023102	-	GGGTAGGGTTGGGAGAGTTCACGGTGAGCGGG<br>...(((((...)))))	ATG4C	NM_032852	chr1:63022365-63103529	template	32	Intron_1.f	--	Yes	--	Yes	--	--	--	Yes	Yes	Yes	--
G4ST01063023070	chr1:63023071-63023102	-	GGGTAGGGTTGGGAGAGTTCACGGTGAGCGGG<br>...(((((...)))))	ATG4C	NM_178221	chr1:63022365-63103529	template	32	Intron_1.f	--	Yes	--	Yes	--	--	--	Yes	Yes	Yes	--
G4ST01063559764	chr1:63559765-63559804	-	GGGGGAACCTGGGCAGACTTGGGCATGCCAGCATGCTGGG<br>((((....))))..	FOXD3	NM_012183	chr1:63561318-63563385	template	-1513	upstream	Yes	--	--	--	--	--	--	Yes	--	--	--
G4ST01063561159	chr1:63561160-63561195	-	GGGGAGGGGGCCGCAGGTGGCTTGGGGCCCGCTGGG<br>.((((....))))...	FOXD3	NM_012183	chr1:63561318-63563385	template	-122	upstream	Yes	--	--	--	--	--	--	--	Yes	Yes	Yes

G4ST01063561168	chr1:63561169-63561201	-	GGGAGAGGGGAGGGGGCCGCAGGTGGCTTGGGG<br>.((((....))))...	FOXD3	NM_012183	chr1:63561318-63563385	template	-116	upstream	Yes	--	--	--	--	--	--	--	Yes	Yes	Yes
G4ST01063734164	chr1:63734165-63734197	+	GGGAGGGAATCTCTCAGGTAGATTAGGGCAGGG<br>(((((.......))))).	ITGB3BP	NM_014288	chr1:63679050-63761423	template	33	Intron_2	--	Yes	--	Yes	--	--	--	--	--	--	--
G4ST01063845732	chr1:63845733-63845773	+	GGGAGCTCAGGGCCAGCCTGGGATAGGCGAGTGTGCCTGGG<br>..(((((....)))))	PGM1	NM_001172819	chr1:63832068-63898505	non-template	41	Intron_1.f	--	Yes	--	Yes	--	--	--	--	--	--	--
G4ST01063845732	chr1:63845733-63845773	+	GGGAGCTCAGGGCCAGCCTGGGATAGGCGAGTGTGCCTGGG<br>..(((((....)))))	PGM1	NM_002633	chr1:63831535-63898505	non-template	41	Intron_1.f	--	Yes	--	Yes	--	--	--	--	--	--	--
G4ST01063845741	chr1:63845742-63845778	+	GGGCCAGCCTGGGATAGGCGAGTGTGCCTGGGATGGG<br>..(((((....)))))	PGM1	NM_001172819	chr1:63832068-63898505	non-template	37	Intron_1.f	--	Yes	--	Yes	--	--	--	--	--	--	--
G4ST01063845741	chr1:63845742-63845778	+	GGGCCAGCCTGGGATAGGCGAGTGTGCCTGGGATGGG<br>..(((((....)))))	PGM1	NM_002633	chr1:63831535-63898505	non-template	37	Intron_1.f	--	Yes	--	Yes	--	--	--	--	--	--	--
G4ST01064012103	chr1:64012104-64012135	+	GGGGAGGGGAGGGAGCGTGCCCGAGACGCGGG<br>.((((.......))))	ROR1	NM_005012	chr1:64012278-64417295	non-template	-142	upstream	Yes	--	--	--	--	--	--	Yes	Yes	Yes	Yes
G4ST01064012103	chr1:64012104-64012135	+	GGGGAGGGGAGGGAGCGTGCCCGAGACGCGGG<br>.((((.......))))	ROR1	NM_001083592	chr1:64012278-64381640	non-template	-142	upstream	Yes	--	--	--	--	--	--	Yes	Yes	Yes	Yes
G4ST01064013055	chr1:64013056-64013091	+	GGGTCCTGGGGGTGATCGGGGCACTTCTGTGCAGGG<br>((((....)))).	ROR1	NM_005012	chr1:64012278-64417295	non-template	36	Intron_1.f	--	Yes	--	Yes	--	--	--	Yes	--	--	Yes
G4ST01064013055	chr1:64013056-64013091	+	GGGTCCTGGGGGTGATCGGGGCACTTCTGTGCAGGG<br>((((....)))).	ROR1	NM_001083592	chr1:64012278-64381640	non-template	36	Intron_1.f	--	Yes	--	Yes	--	--	--	Yes	--	--	Yes
G4ST01064021225	chr1:64021226-64021254	+	GGGTGCGGGATTGAAGGATGGGAGATGGG<br>(((....)))	ROR1	NM_005012	chr1:64012278-64417295	non-template	29	Intron_1.f	--	Yes	--	Yes	--	--	--	--	--	--	--
G4ST01064021225	chr1:64021226-64021254	+	GGGTGCGGGATTGAAGGATGGGAGATGGG<br>(((....)))	ROR1	NM_001083592	chr1:64012278-64381640	non-template	29	Intron_1.f	--	Yes	--	Yes	--	--	--	--	--	--	--
G4ST01064162068	chr1:64162069-64162102	-	GGGGCATCCAGTGGAGGTGGGTAAGTGGGCAGGG<br>.(.(((...))).).	ROR1	NM_005012	chr1:64012278-64417295	template	34	Intron_1.f	--	Yes	--	Yes	--	--	--	--	--	--	--
G4ST01064162068	chr1:64162069-64162102	-	GGGGCATCCAGTGGAGGTGGGTAAGTGGGCAGGG<br>.(.(((...))).).	ROR1	NM_001083592	chr1:64012278-64381640	template	34	Intron_1.f	--	Yes	--	Yes	--	--	--	--	--	--	--
G4ST01064162073	chr1:64162074-64162111	-	GGGTATCGAGGGGCATCCAGTGGAGGTGGGTAAGTGGG<br>.(.(((...))).).	ROR1	NM_005012	chr1:64012278-64417295	template	38	Intron_1.f	--	Yes	--	Yes	--	--	--	--	--	--	--
G4ST01064162073	chr1:64162074-64162111	-	GGGTATCGAGGGGCATCCAGTGGAGGTGGGTAAGTGGG<br>.(.(((...))).).	ROR1	NM_001083592	chr1:64012278-64381640	template	38	Intron_1.f	--	Yes	--	Yes	--	--	--	--	--	--	--
G4ST01064195806	chr1:64195807-64195835	+	GGGAGTGGGAGAGTGGCTGCTGGGAAGGG<br>..(((....)))	ROR1	NM_005012	chr1:64012278-64417295	non-template	29	Intron_1.f	--	Yes	--	Yes	--	--	--	--	--	--	--
G4ST01064195806	chr1:64195807-64195835	+	GGGAGTGGGAGAGTGGCTGCTGGGAAGGG<br>..(((....)))	ROR1	NM_001083592	chr1:64012278-64381640	non-template	29	Intron_1.f	--	Yes	--	Yes	--	--	--	--	--	--	--
G4ST01064241134	chr1:64241135-64241170	+	GGGGACCTCTGGGGCTGAAGGGGGTTTAAGCCAGGG<br>(((....))).	ROR1	NM_005012	chr1:64012278-64417295	non-template	36	Intron_1.f	--	Yes	--	Yes	--	--	--	--	--	--	--
G4ST01064241134	chr1:64241135-64241170	+	GGGGACCTCTGGGGCTGAAGGGGGTTTAAGCCAGGG<br>(((....))).	ROR1	NM_001083592	chr1:64012278-64381640	non-template	36	Intron_1.f	--	Yes	--	Yes	--	--	--	--	--	--	--
G4ST01064464012	chr1:64464013-64464051	+	GGGACCAAGGGTAATTACTAGATTTGGGGTTGAACTGGG<br>.((((....))))..	UBE2U	NM_152489	chr1:64442078-64482615	non-template	39	Intron_6	--	Yes	--	Yes	--	--	--	--	--	--	--
G4ST01064464020	chr1:64464021-64464055	+	GGGTAATTACTAGATTTGGGGTTGAACTGGGTGGG<br>.((((....))))..	UBE2U	NM_152489	chr1:64442078-64482615	non-template	35	Intron_6	--	Yes	--	Yes	--	--	--	--	--	--	--
G4ST01064709408	chr1:64709409-64709439	-	GGGCAGGGGATTCTCCAGAGAAGGGCAGGGG<br>..((((....))))	CACHD1	NM_020925	chr1:64709064-64931329	template	31	Intron_1.f	--	Yes	--	Yes	--	--	--	--	--	--	--
G4ST01064744008	chr1:64744009-64744041	-	GGGGAAGGAGGGCGCCCGGAAGGCGGGGCGGGG<br>((((.....))))	CACHD1	NM_020925	chr1:64709064-64931329	template	33	Intron_1.f	--	Yes	--	Yes	--	--	--	Yes	--	--	--
G4ST01064744053	chr1:64744054-64744084	-	GGGGTCCTGGCGACCGGGAGGAGGGTCAGGG<br>(((.....))).	CACHD1	NM_020925	chr1:64709064-64931329	template	31	Intron_1.f	--	Yes	--	Yes	--	--	--	Yes	--	--	--
G4ST01064744059	chr1:64744060-64744091	-	GGGCCGCGGGGTCCTGGCGACCGGGAGGAGGG<br>(((.....))).	CACHD1	NM_020925	chr1:64709064-64931329	template	32	Intron_1.f	--	Yes	--	Yes	--	--	--	Yes	--	--	--
G4ST01064983157	chr1:64983158-64983191	+	GGGCGCGGGCGCGCCTGTGCGCCGGGGCCGGGGG<br>.((((....))))..	RAVER2	NM_018211	chr1:64983366-65071502	non-template	-174	upstream	Yes	--	--	--	--	--	--	Yes	Yes	Yes	--
G4ST01064983163	chr1:64983164-64983201	+	GGGCGCGCCTGTGCGCCGGGGCCGGGGGCCGGCGGGGG<br>.((((....))))..	RAVER2	NM_018211	chr1:64983366-65071502	non-template	-164	upstream	Yes	--	--	--	--	--	--	Yes	Yes	Yes	--
G4ST01065025840	chr1:65025841-65025876	+	GGGTGTGGGAGGACCTGAGAAGGTCGGGGAGGTGGG<br>..(((((....))))).	RAVER2	NM_018211	chr1:64983366-65071502	non-template	36	Intron_4	--	Yes	--	Yes	--	--	--	--	--	--	--
G4ST01065082200	chr1:65082201-65082236	-	GGGCATGGGTTTGAATCTCTTCACGGGCCTACTGGG<br>..((((....)))).	JAK1	NM_002227	chr1:65071494-65204775	non-template	36	Intron_17	--	Yes	--	Yes	--	--	--	--	--	--	--
G4ST01065082209	chr1:65082210-65082246	-	GGGAAGAGATGGGCATGGGTTTGAATCTCTTCACGGG<br>..((((....)))).	JAK1	NM_002227	chr1:65071494-65204775	non-template	37	Intron_17	--	Yes	--	Yes	--	--	--	--	--	--	--
G4ST01065204363	chr1:65204364-65204403	-	GGGCGGCGAGGAGGTCGCCGGGCGGGGGCCCCCGGCCGGG<br>.(((((.....))))) ((((...))))	JAK1	NM_002227	chr1:65071494-65204775	non-template	40	Intron_1.f	--	Yes	--	Yes	--	--	--	Yes	Yes	Yes	--
G4ST01065387218	chr1:65387219-65387255	-	GGGCGGCGCTCCGCCTCTGGGCTCCCGCGGGAGGGGG<br>.((((...))))...	AK4	NM_013410	chr1:65386438-65470416	template	37	Intron_1.f	--	Yes	--	Yes	--	--	--	Yes	Yes	Yes	--
G4ST01065387218	chr1:65387219-65387255	-	GGGCGGCGCTCCGCCTCTGGGCTCCCGCGGGAGGGGG<br>.((((...))))...	AK4	NM_203464	chr1:65385820-65470416	template	37	Intron_2	--	Yes	--	Yes	--	--	--	Yes	Yes	Yes	--
G4ST01065387218	chr1:65387219-65387255	-	GGGCGGCGCTCCGCCTCTGGGCTCCCGCGGGAGGGGG<br>.((((...))))...	AK4	NM_001005353	chr1:65386101-65470416	template	37	Intron_2	--	Yes	--	Yes	--	--	--	Yes	Yes	Yes	--
G4ST01065547795	chr1:65547796-65547829	-	GGGCTGAGCTCGGCTCGGGGCGCCGGGAGGAGGG<br>..((((...)))).	DNAJC6	NM_014787	chr1:65503018-65654140	template	34	Intron_1.f	--	Yes	--	Yes	--	--	--	Yes	Yes	--	Yes
G4ST01065658764	chr1:65658765-65658796	+	GGGGGCGGGGTTCTGCACCGGCGGGGGCGGGG<br>...((((....))))	LEPROT	NM_001198683	chr1:65658719-65666280	non-template	32	Exon_1.f	--	Yes	Yes	--	--	Yes	--	--	Yes	Yes	--
G4ST01065658764	chr1:65658765-65658796	+	GGGGGCGGGGTTCTGCACCGGCGGGGGCGGGG<br>...((((....))))	LEPROT	NM_017526	chr1:65658719-65674278	non-template	32	Exon_1.f	--	Yes	Yes	--	--	Yes	--	--	Yes	Yes	--
G4ST01065658764	chr1:65658765-65658796	+	GGGGGCGGGGTTCTGCACCGGCGGGGGCGGGG<br>...((((....))))	LEPR	NM_002303	chr1:65658923-65875764	non-template	-126	upstream	Yes	--	--	--	--	--	--	--	Yes	Yes	--
G4ST01065658764	chr1:65658765-65658796	+	GGGGGCGGGGTTCTGCACCGGCGGGGGCGGGG<br>...((((....))))	LEPROT	NM_001198681	chr1:65658987-65674278	non-template	-190	upstream	Yes	--	--	--	--	--	--	--	Yes	Yes	--
G4ST01065658764	chr1:65658765-65658796	+	GGGGGCGGGGTTCTGCACCGGCGGGGGCGGGG<br>...((((....))))	LEPR	NM_001003680	chr1:65658923-65868683	non-template	-126	upstream	Yes	--	--	--	--	--	--	--	Yes	Yes	--
G4ST01065658764	chr1:65658765-65658796	+	GGGGGCGGGGTTCTGCACCGGCGGGGGCGGGG<br>...((((....))))	LEPR	NM_001003679	chr1:65658923-65873699	non-template	-126	upstream	Yes	--	--	--	--	--	--	--	Yes	Yes	--
G4ST01065700418	chr1:65700419-65700446	-	GGGGCCTGTTGGCGGGTGGGGGGATGGG<br>(((....)))	LEPR	NM_002303	chr1:65658923-65875764	template	28	Intron_2	--	Yes	--	Yes	--	--	--	--	--	--	Yes
G4ST01065700418	chr1:65700419-65700446	-	GGGGCCTGTTGGCGGGTGGGGGGATGGG<br>(((....)))	LEPR	NM_001003680	chr1:65658923-65868683	template	28	Intron_2	--	Yes	--	Yes	--	--	--	--	--	--	Yes
G4ST01065700418	chr1:65700419-65700446	-	GGGGCCTGTTGGCGGGTGGGGGGATGGG<br>(((....)))	LEPR	NM_001003679	chr1:65658923-65873699	template	28	Intron_2	--	Yes	--	Yes	--	--	--	--	--	--	Yes
G4ST01065738244	chr1:65738245-65738273	+	GGGGAGGGAGCTTGTGGGAAGTGATTGGG<br>((....))	LEPR	NM_002303	chr1:65658923-65875764	non-template	29	Intron_2	--	Yes	--	Yes	--	--	--	--	--	--	--
G4ST01065738244	chr1:65738245-65738273	+	GGGGAGGGAGCTTGTGGGAAGTGATTGGG<br>((....))	LEPR	NM_001003680	chr1:65658923-65868683	non-template	29	Intron_2	--	Yes	--	Yes	--	--	--	--	--	--	--
G4ST01065738244	chr1:65738245-65738273	+	GGGGAGGGAGCTTGTGGGAAGTGATTGGG<br>((....))	LEPR	NM_001003679	chr1:65658923-65873699	non-template	29	Intron_2	--	Yes	--	Yes	--	--	--	--	--	--	--
G4ST01065738249	chr1:65738250-65738281	+	GGGAGCTTGTGGGAAGTGATTGGGTCATGGGG<br>((....))	LEPR	NM_002303	chr1:65658923-65875764	non-template	32	Intron_2	--	Yes	--	Yes	--	--	--	--	--	--	--
G4ST01065738249	chr1:65738250-65738281	+	GGGAGCTTGTGGGAAGTGATTGGGTCATGGGG<br>((....))	LEPR	NM_001003680	chr1:65658923-65868683	non-template	32	Intron_2	--	Yes	--	Yes	--	--	--	--	--	--	--

G4ST01065738249	chr1:65738250-65738281	+	GGGAGCTTGTGGGAAGTGATTGGGTCATGGGG<br>((....))	LEPR	NM_001003679	chr1:65658923-65873699	non-template	32	Intron_2	--	Yes	--	Yes	--	--	--	--	--	--	--
G4ST01065763872	chr1:65763873-65763909	-	GGGGCTGAGCAGGAGCAGGCTGGGGTGGGGAGAAGGG<br>(((..((....)).)))...	LEPR	NM_001198688	chr1:65763960-65864505	template	-50	upstream	Yes	--	--	--	--	--	--	Yes	Yes	Yes	Yes
G4ST01065763872	chr1:65763873-65763909	-	GGGGCTGAGCAGGAGCAGGCTGGGGTGGGGAGAAGGG<br>(((..((....)).)))...	LEPR	NM_001198687	chr1:65763960-65868683	template	-50	upstream	Yes	--	--	--	--	--	--	Yes	Yes	Yes	Yes
G4ST01065763872	chr1:65763873-65763909	-	GGGGCTGAGCAGGAGCAGGCTGGGGTGGGGAGAAGGG<br>(((..((....)).)))...	LEPR	NM_002303	chr1:65658923-65875764	template	37	Intron_2	--	Yes	--	Yes	--	--	--	Yes	Yes	Yes	Yes
G4ST01065763872	chr1:65763873-65763909	-	GGGGCTGAGCAGGAGCAGGCTGGGGTGGGGAGAAGGG<br>(((..((....)).)))...	LEPR	NM_001198689	chr1:65763960-65873699	template	-50	upstream	Yes	--	--	--	--	--	--	Yes	Yes	Yes	Yes
G4ST01065763872	chr1:65763873-65763909	-	GGGGCTGAGCAGGAGCAGGCTGGGGTGGGGAGAAGGG<br>(((..((....)).)))...	LEPR	NM_001003680	chr1:65658923-65868683	template	37	Intron_2	--	Yes	--	Yes	--	--	--	Yes	Yes	Yes	Yes
G4ST01065763872	chr1:65763873-65763909	-	GGGGCTGAGCAGGAGCAGGCTGGGGTGGGGAGAAGGG<br>(((..((....)).)))...	LEPR	NM_001003679	chr1:65658923-65873699	template	37	Intron_2	--	Yes	--	Yes	--	--	--	Yes	Yes	Yes	Yes
G4ST01065763879	chr1:65763880-65763917	-	GGGGAGGTGGGGCTGAGCAGGAGCAGGCTGGGGTGGGG<br>(((..((....)).)))...	LEPR	NM_001198688	chr1:65763960-65864505	template	-42	upstream	Yes	--	--	--	--	--	--	Yes	Yes	Yes	Yes
G4ST01065763879	chr1:65763880-65763917	-	GGGGAGGTGGGGCTGAGCAGGAGCAGGCTGGGGTGGGG<br>(((..((....)).)))...	LEPR	NM_001198687	chr1:65763960-65868683	template	-42	upstream	Yes	--	--	--	--	--	--	Yes	Yes	Yes	Yes
G4ST01065763879	chr1:65763880-65763917	-	GGGGAGGTGGGGCTGAGCAGGAGCAGGCTGGGGTGGGG<br>(((..((....)).)))...	LEPR	NM_002303	chr1:65658923-65875764	template	38	Intron_2	--	Yes	--	Yes	--	--	--	Yes	Yes	Yes	Yes
G4ST01065763879	chr1:65763880-65763917	-	GGGGAGGTGGGGCTGAGCAGGAGCAGGCTGGGGTGGGG<br>(((..((....)).)))...	LEPR	NM_001198689	chr1:65763960-65873699	template	-42	upstream	Yes	--	--	--	--	--	--	Yes	Yes	Yes	Yes
G4ST01065763879	chr1:65763880-65763917	-	GGGGAGGTGGGGCTGAGCAGGAGCAGGCTGGGGTGGGG<br>(((..((....)).)))...	LEPR	NM_001003680	chr1:65658923-65868683	template	38	Intron_2	--	Yes	--	Yes	--	--	--	Yes	Yes	Yes	Yes
G4ST01065763879	chr1:65763880-65763917	-	GGGGAGGTGGGGCTGAGCAGGAGCAGGCTGGGGTGGGG<br>(((..((....)).)))...	LEPR	NM_001003679	chr1:65658923-65873699	template	38	Intron_2	--	Yes	--	Yes	--	--	--	Yes	Yes	Yes	Yes
G4ST01066471366	chr1:66471367-66471395	-	GGGAGGGGTTGCAAAGGGAAACTTTTGGG<br>((....))	PDE4B	NM_001037340	chr1:66230978-66612850	template	29	Intron_1.f	--	Yes	--	Yes	--	--	--	--	--	--	Yes
G4ST01066471366	chr1:66471367-66471395	-	GGGAGGGGTTGCAAAGGGAAACTTTTGGG<br>((....))	PDE4B	NM_002600	chr1:66031444-66612850	template	29	Intron_3	--	Yes	--	Yes	--	--	--	--	--	--	Yes
G4ST01066471366	chr1:66471367-66471395	-	GGGAGGGGTTGCAAAGGGAAACTTTTGGG<br>((....))	PDE4B	NM_001037341	chr1:66030781-66612850	template	29	Intron_3	--	Yes	--	Yes	--	--	--	--	--	--	Yes
G4ST01067136719	chr1:67136720-67136751	+	GGGACTTTTGGGAGGTGACTGGGTCAAGAGGG<br>((....))	WDR78	NM_024763	chr1:67051160-67163158	template	32	Intron_2	--	Yes	--	Yes	--	--	--	--	--	--	--
G4ST01067136719	chr1:67136720-67136751	+	GGGACTTTTGGGAGGTGACTGGGTCAAGAGGG<br>((....))	WDR78	NM_207014	chr1:67075870-67163158	template	32	Intron_2	--	Yes	--	Yes	--	--	--	--	--	--	--
G4ST01067163457	chr1:67163458-67163493	-	GGGTCTTCCGGGCGGTACACTGCCGTGGGGGAGGGG<br>((((.....))))...	MIER1	NM_001077702	chr1:67163166-67226890	template	36	Intron_1.f	--	Yes	--	Yes	--	--	--	Yes	Yes	Yes	--
G4ST01067163457	chr1:67163458-67163493	-	GGGTCTTCCGGGCGGTACACTGCCGTGGGGGAGGGG<br>((((.....))))...	MIER1	NM_001146113	chr1:67163166-67226890	template	36	Intron_1.f	--	Yes	--	Yes	--	--	--	Yes	Yes	Yes	--
G4ST01067163457	chr1:67163458-67163493	-	GGGTCTTCCGGGCGGTACACTGCCGTGGGGGAGGGG<br>((((.....))))...	MIER1	NM_001146111	chr1:67163166-67226890	template	36	Intron_1.f	--	Yes	--	Yes	--	--	--	Yes	Yes	Yes	--
G4ST01067163457	chr1:67163458-67163493	-	GGGTCTTCCGGGCGGTACACTGCCGTGGGGGAGGGG<br>((((.....))))...	MIER1	NM_001077703	chr1:67163166-67226890	template	36	Intron_1.f	--	Yes	--	Yes	--	--	--	Yes	Yes	Yes	--
G4ST01067163457	chr1:67163458-67163493	-	GGGTCTTCCGGGCGGTACACTGCCGTGGGGGAGGGG<br>((((.....))))...	MIER1	NM_001146112	chr1:67163166-67226890	template	36	Intron_1.f	--	Yes	--	Yes	--	--	--	Yes	Yes	Yes	--
G4ST01067163457	chr1:67163458-67163493	-	GGGTCTTCCGGGCGGTACACTGCCGTGGGGGAGGGG<br>((((.....))))...	MIER1	NM_020948	chr1:67163166-67226890	template	36	Intron_1.f	--	Yes	--	Yes	--	--	--	Yes	Yes	Yes	--
G4ST01067163457	chr1:67163458-67163493	-	GGGTCTTCCGGGCGGTACACTGCCGTGGGGGAGGGG<br>((((.....))))...	MIER1	NM_001146110	chr1:67163166-67226890	template	36	Intron_1.f	--	Yes	--	Yes	--	--	--	Yes	Yes	Yes	--
G4ST01067163457	chr1:67163458-67163493	-	GGGTCTTCCGGGCGGTACACTGCCGTGGGGGAGGGG<br>((((.....))))...	MIER1	NM_001077700	chr1:67163166-67226890	template	36	Intron_1.f	--	Yes	--	Yes	--	--	--	Yes	Yes	Yes	--
G4ST01067163457	chr1:67163458-67163493	-	GGGTCTTCCGGGCGGTACACTGCCGTGGGGGAGGGG<br>((((.....))))...	WDR78	NM_024763	chr1:67051160-67163158	non-template	-299	upstream	Yes	--	--	--	--	--	--	Yes	Yes	Yes	--
G4ST01067163457	chr1:67163458-67163493	-	GGGTCTTCCGGGCGGTACACTGCCGTGGGGGAGGGG<br>((((.....))))...	WDR78	NM_207014	chr1:67075870-67163158	non-template	-299	upstream	Yes	--	--	--	--	--	--	Yes	Yes	Yes	--
G4ST01067168931	chr1:67168932-67168960	+	GGGGGAGGGGGCGCCCGCCGGGGAAGGGG<br>(((....))).	MIER1	NM_001077702	chr1:67163166-67226890	non-template	29	Intron_3	--	Yes	--	Yes	--	--	--	Yes	Yes	Yes	--
G4ST01067168931	chr1:67168932-67168960	+	GGGGGAGGGGGCGCCCGCCGGGGAAGGGG<br>(((....))).	MIER1	NM_001146113	chr1:67163166-67226890	non-template	29	Intron_2	--	Yes	--	Yes	--	--	--	Yes	Yes	Yes	--
G4ST01067168931	chr1:67168932-67168960	+	GGGGGAGGGGGCGCCCGCCGGGGAAGGGG<br>(((....))).	MIER1	NM_001146111	chr1:67163166-67226890	non-template	29	Intron_3	--	Yes	--	Yes	--	--	--	Yes	Yes	Yes	--
G4ST01067168931	chr1:67168932-67168960	+	GGGGGAGGGGGCGCCCGCCGGGGAAGGGG<br>(((....))).	MIER1	NM_001077704	chr1:67168514-67226890	non-template	29	Intron_1.f	--	Yes	--	Yes	--	--	--	Yes	Yes	Yes	--
G4ST01067168931	chr1:67168932-67168960	+	GGGGGAGGGGGCGCCCGCCGGGGAAGGGG<br>(((....))).	MIER1	NM_001077703	chr1:67163166-67226890	non-template	29	Intron_2	--	Yes	--	Yes	--	--	--	Yes	Yes	Yes	--
G4ST01067168931	chr1:67168932-67168960	+	GGGGGAGGGGGCGCCCGCCGGGGAAGGGG<br>(((....))).	MIER1	NM_001146112	chr1:67163166-67226890	non-template	29	Intron_3	--	Yes	--	Yes	--	--	--	Yes	Yes	Yes	--
G4ST01067168931	chr1:67168932-67168960	+	GGGGGAGGGGGCGCCCGCCGGGGAAGGGG<br>(((....))).	MIER1	NM_020948	chr1:67163166-67226890	non-template	29	Intron_3	--	Yes	--	Yes	--	--	--	Yes	Yes	Yes	--
G4ST01067168931	chr1:67168932-67168960	+	GGGGGAGGGGGCGCCCGCCGGGGAAGGGG<br>(((....))).	MIER1	NM_001077701	chr1:67168514-67226890	non-template	29	Intron_1.f	--	Yes	--	Yes	--	--	--	Yes	Yes	Yes	--
G4ST01067168931	chr1:67168932-67168960	+	GGGGGAGGGGGCGCCCGCCGGGGAAGGGG<br>(((....))).	MIER1	NM_001146110	chr1:67163166-67226890	non-template	29	Intron_3	--	Yes	--	Yes	--	--	--	Yes	Yes	Yes	--
G4ST01067168931	chr1:67168932-67168960	+	GGGGGAGGGGGCGCCCGCCGGGGAAGGGG<br>(((....))).	MIER1	NM_001077700	chr1:67163166-67226890	non-template	29	Intron_2	--	Yes	--	Yes	--	--	--	Yes	Yes	Yes	--
G4ST01067291800	chr1:67291801-67291831	+	GGGCAGGGTTTTCGGGGCGGTGCGCCCAGGG<br>(((...)))...	SLC35D1	NM_015139	chr1:67237603-67292668	template	31	Intron_1.f	--	Yes	--	Yes	--	--	--	Yes	Yes	Yes	--
G4ST01067291926	chr1:67291927-67291960	-	GGGGAGGGCGGGCGGCTGACGCGCGCGGCCCGGG<br>.(((((.......))))).	SLC35D1	NM_015139	chr1:67237603-67292668	non-template	34	Intron_1.f	--	Yes	--	Yes	--	--	--	Yes	Yes	Yes	Yes
G4ST01067545993	chr1:67545994-67546023	-	GGGGGTCGGGGCTGGGCGGTGGCCGCAGGG<br>(((...)))..	IL12RB2	NM_001559	chr1:67545635-67635171	template	30	Exon_1.f	--	Yes	Yes	--	--	Yes	--	Yes	Yes	--	Yes
G4ST01067556236	chr1:67556237-67556273	+	GGGGAAGACCACATTGGTCAGGGAGGGCGTCGGAGGG<br>...(((((...))))).	IL12RB2	NM_001559	chr1:67545635-67635171	non-template	37	Intron_1.f	--	Yes	--	Yes	--	--	--	--	Yes	--	--
G4ST01067580724	chr1:67580725-67580752	+	GGGTGCAGCCCCTGGGGGTTGGGGTGGG<br>..(((...))).	IL12RB2	NM_001559	chr1:67545635-67635171	non-template	28	Intron_8	--	Yes	--	Yes	--	--	--	Yes	Yes	--	--
G4ST01068071383	chr1:68071384-68071418	-	GGGCGCCGGAGACCGGGGGGAGGGGGAGGAAGGGG<br>..((((...)))).	GNG12	NM_018841	chr1:67939737-68071743	non-template	35	Intron_1.f	--	Yes	--	Yes	--	--	--	Yes	Yes	Yes	Yes
G4ST01068071392	chr1:68071393-68071425	-	GGGCGCTGGGCGCCGGAGACCGGGGGGAGGGGG<br>..((((...)))).	GNG12	NM_018841	chr1:67939737-68071743	non-template	33	Intron_1.f	--	Yes	--	Yes	--	--	--	Yes	Yes	Yes	Yes
G4ST01068071548	chr1:68071549-68071580	-	GGGTCCCCGCGGGGTCGGGGCGCGGCGCGGGG<br>.(((...)))	GNG12	NM_018841	chr1:67939737-68071743	non-template	32	Intron_1.f	--	Yes	--	Yes	--	--	--	Yes	Yes	Yes	--
G4ST01068071717	chr1:68071718-68071751	+	GGGCGCCGGCTCCGCCTCGCTGGGGTGGGCGGGG<br>.((.(((...)))..))..	GNG12	NM_018841	chr1:67939737-68071743	template	26	upstream-Exon_1.f	Yes	Yes	Yes	--	--	Yes	--	Yes	Yes	Yes	--
G4ST01068735477	chr1:68735478-68735510	+	GGGGCTCCAGTAGCTGGCGCGGGCTGGGGTGGG<br>((.((((...)))).))	DEPDC1	NM_017779	chr1:68712423-68735387	template	-90	upstream	Yes	--	--	--	--	--	--	Yes	Yes	Yes	Yes
G4ST01068735477	chr1:68735478-68735510	+	GGGGCTCCAGTAGCTGGCGCGGGCTGGGGTGGG<br>((.((((...)))).))	DEPDC1	NM_001114120	chr1:68712423-68735387	template	-90	upstream	Yes	--	--	--	--	--	--	Yes	Yes	Yes	Yes
G4ST01070277527	chr1:70277528-70277555	+	GGGGCCAGGGCGGGCTTCCTGAGAAGGG<br>.(((....)))	LRRC7	NM_020794	chr1:69998446-70361759	non-template	28	Exon_19	--	Yes	Yes	--	Yes	--	--	--	--	--	--

G4ST01070285190	chr1:70285191-70285229	-	GGGGAGAAGGGTTCAGGGAAATTGAACCTCAATCATGGG<br>..(((((...)))))...	LRRC7	NM_020794	chr1:69998446-70361759	template	39	Intron_20	--	Yes	--	Yes	--	--	--	--	--	--	--
G4ST01072451898	chr1:72451899-72451933	-	GGGAGGGTAACTAGCAATATTTTGGGACTTCTGGG<br>.((.((....)).)).	NEGR1	NM_173808	chr1:71641213-72520865	non-template	35	Intron_1.f	--	Yes	--	Yes	--	--	--	--	--	--	--
G4ST01074630478	chr1:74630479-74630505	+	GGGTCAGGGAGGGATTTGAAGGATGGG<br>(((.....)))	TNNI3K	NM_015978	chr1:74473659-74782704	non-template	27	Intron_17	--	Yes	--	Yes	--	--	--	--	--	--	Yes
G4ST01074630478	chr1:74630479-74630505	+	GGGTCAGGGAGGGATTTGAAGGATGGG<br>(((.....)))	FPGT-TNNI3K	NM_001199327	chr1:74436484-74703197	non-template	27	Intron_19	--	Yes	--	Yes	--	--	--	--	--	--	Yes
G4ST01074630478	chr1:74630479-74630505	+	GGGTCAGGGAGGGATTTGAAGGATGGG<br>(((.....)))	FPGT-TNNI3K	NM_001112808	chr1:74436484-74782704	non-template	27	Intron_19	--	Yes	--	Yes	--	--	--	--	--	--	Yes
G4ST01074898090	chr1:74898091-74898125	+	GGGAAGAAGTAAAACTTGGGTGGGTAATTGTGGGG<br>...((((...))))	C1orf173	NM_001002912	chr1:74806383-74912010	template	35	Intron_1.f	--	Yes	--	Yes	--	--	--	--	--	--	--
G4ST01075569976	chr1:75569977-75570003	-	GGGAGCGGGACTTAGGTAGTGGGTGGG<br>(((.....)))	SLC44A5	NM_152697	chr1:75444663-75849387	non-template	27	Intron_4	--	Yes	--	Yes	--	--	--	--	--	--	--
G4ST01075569976	chr1:75569977-75570003	-	GGGAGCGGGACTTAGGTAGTGGGTGGG<br>(((.....)))	SLC44A5	NM_001130058	chr1:75440404-75849387	non-template	27	Intron_4	--	Yes	--	Yes	--	--	--	--	--	--	--
G4ST01075653496	chr1:75653497-75653532	+	GGGAGGGACCTGGTGGGAGATGACTGAATCATGGGG<br>..((((.....)))).	SLC44A5	NM_152697	chr1:75444663-75849387	template	36	Intron_2	--	Yes	--	Yes	--	--	--	--	--	--	--
G4ST01075653496	chr1:75653497-75653532	+	GGGAGGGACCTGGTGGGAGATGACTGAATCATGGGG<br>..((((.....)))).	SLC44A5	NM_001130058	chr1:75440404-75849387	template	36	Intron_2	--	Yes	--	Yes	--	--	--	--	--	--	--
G4ST01075742571	chr1:75742572-75742608	+	GGGGAAGTGGTAGTAATTGGGAAGGGGCATTAAAGGG<br>.((.((....)).))	SLC44A5	NM_152697	chr1:75444663-75849387	template	37	Intron_2	--	Yes	--	Yes	--	--	--	--	--	--	Yes
G4ST01075742571	chr1:75742572-75742608	+	GGGGAAGTGGTAGTAATTGGGAAGGGGCATTAAAGGG<br>.((.((....)).))	SLC44A5	NM_001130058	chr1:75440404-75849387	template	37	Intron_2	--	Yes	--	Yes	--	--	--	--	--	--	Yes
G4ST01076035962	chr1:76035963-76035997	+	GGGAAACCAGGGTGGGCGGATCACTTGAGGCCGGG<br>.((.((....))..))	MSH4	NM_002440	chr1:76035218-76151511	non-template	35	Intron_1.f	--	Yes	--	Yes	--	--	--	--	--	Yes	--
G4ST01076313436	chr1:76313437-76313471	+	GGGGATGGTAGGGAGAGGAGGGGTGTTCCCGCGGG<br>(((....)))	ST6GALNAC3	NM_152996	chr1:76312977-76869257	non-template	35	Intron_1.f	--	Yes	--	Yes	--	--	--	Yes	Yes	Yes	--
G4ST01076313436	chr1:76313437-76313471	+	GGGGATGGTAGGGAGAGGAGGGGTGTTCCCGCGGG<br>(((....)))	ST6GALNAC3	NM_001160011	chr1:76312977-76815480	non-template	35	Intron_1.f	--	Yes	--	Yes	--	--	--	Yes	Yes	Yes	--
G4ST01076446300	chr1:76446301-76446325	-	GGGGGGGGGGCACTCTTGAGTGGG<br>.(((....)))	ST6GALNAC3	NM_152996	chr1:76312977-76869257	template	25	Intron_1.f	--	Yes	--	Yes	--	--	--	--	--	--	--
G4ST01076446300	chr1:76446301-76446325	-	GGGGGGGGGGCACTCTTGAGTGGG<br>.(((....)))	ST6GALNAC3	NM_001160011	chr1:76312977-76815480	template	25	Intron_1.f	--	Yes	--	Yes	--	--	--	--	--	--	--
G4ST01076528132	chr1:76528133-76528174	+	GGGTTCTTGAAGCTAGTCATGAAGGGAACAGAGGGAGGAGGG<br>(((.(((......))).)))	ST6GALNAC3	NM_152996	chr1:76312977-76869257	non-template	42	Intron_1.f	--	Yes	--	Yes	--	--	--	Yes	--	--	--
G4ST01076528132	chr1:76528133-76528174	+	GGGTTCTTGAAGCTAGTCATGAAGGGAACAGAGGGAGGAGGG<br>(((.(((......))).)))	ST6GALNAC3	NM_001160011	chr1:76312977-76815480	non-template	42	Intron_1.f	--	Yes	--	Yes	--	--	--	Yes	--	--	--
G4ST01076580122	chr1:76580123-76580155	-	GGGCCTGTTAGGAAGCAGGGGGATAGGGGAGGG<br>(((((......)))))	ST6GALNAC3	NM_152996	chr1:76312977-76869257	template	33	Intron_2	--	Yes	--	Yes	--	--	--	--	--	--	--
G4ST01076580122	chr1:76580123-76580155	-	GGGCCTGTTAGGAAGCAGGGGGATAGGGGAGGG<br>(((((......)))))	ST6GALNAC3	NM_001160011	chr1:76312977-76815480	template	33	Intron_2	--	Yes	--	Yes	--	--	--	--	--	--	--
G4ST01077107131	chr1:77107132-77107160	+	GGGGAGTGGACCCGCTGGGGTAGGGTGGG<br>.((((....)))).	ST6GALNAC5	NM_030965	chr1:77105774-77302325	non-template	29	Intron_2	--	Yes	--	Yes	--	--	--	--	--	--	--
G4ST01077121145	chr1:77121146-77121187	-	GGGTGCGTAGTGCCTAGGCTGAGGGCAGTGGTGGGGGTAGGG<br>.((.(((...))).))...	ST6GALNAC5	NM_030965	chr1:77105774-77302325	template	42	Intron_2	--	Yes	--	Yes	--	--	--	--	--	--	--
G4ST01077426916	chr1:77426917-77426941	-	GGGATTTTCAAAAAGGGAGGGAGGG<br>.(((....)))	PIGK	NM_005482	chr1:77327255-77457720	non-template	25	Intron_3	--	Yes	--	Yes	--	--	--	--	--	--	--
G4ST01077637806	chr1:77637807-77637836	+	GGGCTGTGAGGGAGGGGCAGCTAAGCTGGG<br>..(((...)))	AK5	NM_012093	chr1:77520875-77798242	non-template	30	Intron_6	--	Yes	--	Yes	--	--	--	--	--	--	--
G4ST01077637806	chr1:77637807-77637836	+	GGGCTGTGAGGGAGGGGCAGCTAAGCTGGG<br>..(((...)))	AK5	NM_174858	chr1:77520250-77798242	non-template	30	Intron_6	--	Yes	--	Yes	--	--	--	--	--	--	--
G4ST01077649745	chr1:77649746-77649776	-	GGGTCACACAGGAGCTGTGGGTTGGGATGGG<br>....((((...))))	AK5	NM_012093	chr1:77520875-77798242	template	31	Intron_7	--	Yes	--	Yes	--	--	--	--	--	--	--
G4ST01077649745	chr1:77649746-77649776	-	GGGTCACACAGGAGCTGTGGGTTGGGATGGG<br>....((((...))))	AK5	NM_174858	chr1:77520250-77798242	template	31	Intron_7	--	Yes	--	Yes	--	--	--	--	--	--	--
G4ST01077920730	chr1:77920731-77920764	+	GGGGGATGGGGAGGGCAGTGTTGCCTCAATGGGG<br>((.((......)).))	ZZZ3	NM_015534	chr1:77802778-77920931	template	34	Intron_1.f	--	Yes	--	Yes	--	--	--	Yes	Yes	Yes	--
G4ST01077920810	chr1:77920811-77920846	-	GGGAGGGGCCCGGGGCGAGTGCCTCTTGGTGCCGGG<br>....(((((....))))).	ZZZ3	NM_015534	chr1:77802778-77920931	non-template	36	Intron_1.f	--	Yes	--	Yes	--	--	--	Yes	Yes	Yes	--
G4ST01077921307	chr1:77921308-77921346	-	GGGGGTGCGCGCGCACGTGTGCGCGGGCGAAAGGGGGGG<br>...(((((((....)))))))	ZZZ3	NM_015534	chr1:77802778-77920931	non-template	-376	upstream	Yes	--	--	--	--	--	--	Yes	Yes	Yes	Yes
G4ST01077921311	chr1:77921312-77921351	-	GGGGCGGGGGTGCGCGCGCACGTGTGCGCGGGCGAAAGGG<br>...(((((((....)))))))	ZZZ3	NM_015534	chr1:77802778-77920931	non-template	-380	upstream	Yes	--	--	--	--	--	--	Yes	Yes	Yes	Yes
G4ST01078107978	chr1:78107979-78108017	-	GGGGGGTCGGCCAGCCGCCCTGTCCGGGAGGGAGGTGGG<br>(((.(((....)))))).....	FAM73A	NM_198549	chr1:78017897-78116669	template	39	Intron_14	--	Yes	--	Yes	--	--	--	--	--	--	Yes
G4ST01078107985	chr1:78107986-78108021	-	GGGGGGGGGGTCGGCCAGCCGCCCTGTCCGGGAGGG<br>(((.(((....)))))).....	FAM73A	NM_198549	chr1:78017897-78116669	template	36	Intron_14	--	Yes	--	Yes	--	--	--	--	--	--	Yes
G4ST01078293525	chr1:78293526-78293557	-	GGGGTCTTTGGGAGGTGACTGGGTCATGAGGG<br>((....))	GIPC2	NM_017655	chr1:78284177-78375700	template	32	Intron_1.f	--	Yes	--	Yes	--	--	--	--	--	--	--
G4ST01078748806	chr1:78748807-78748843	-	GGGGCCTGGTGGGAGGTGAATTCATCCTGGGAGTGGG<br>.((((....))))..	PTGFR	NM_000959	chr1:78729316-78778974	template	37	Intron_2.l	--	Yes	--	Yes	--	--	--	--	--	--	--
G4ST01078748806	chr1:78748807-78748843	-	GGGGCCTGGTGGGAGGTGAATTCATCCTGGGAGTGGG<br>.((((....))))..	PTGFR	NM_001039585	chr1:78729316-78778974	template	37	Intron_3.l	--	Yes	--	Yes	--	--	--	--	--	--	--
G4ST01082040057	chr1:82040058-82040088	-	GGGAGTCCCTTCGAAGCGACGGGCGGGAGGG<br>.(((.((....)).)))	LPHN2	NM_012302	chr1:82038670-82230695	template	31	Intron_1.f	--	Yes	--	Yes	--	--	--	Yes	Yes	Yes	Yes
G4ST01084158892	chr1:84158893-84158921	+	GGGGTGGGGGGGTTTGAATCTGTCAAGGG<br>.((((.....))))	TTLL7	NM_024686	chr1:84107645-84237421	template	29	Intron_12	--	Yes	--	Yes	--	--	--	--	--	--	Yes
G4ST01084325034	chr1:84325035-84325065	-	GGGAGGGGGCTCCCTGAGGGGTGAGGATGGG<br>..(((...)))	PRKACB	NM_002731	chr1:84316333-84476769	template	31	Intron_1.f	--	Yes	--	Yes	--	--	--	--	--	--	--
G4ST01084325034	chr1:84325035-84325065	-	GGGAGGGGGCTCCCTGAGGGGTGAGGATGGG<br>..(((...)))	PRKACB	NM_207578	chr1:84316333-84443567	template	31	Intron_1.f	--	Yes	--	Yes	--	--	--	--	--	--	--
G4ST01084609077	chr1:84609078-84609101	+	GGGAGGGACAGCTGTGGGAGTGGG<br>((....))	UOX	NR_003927	chr1:84603229-84636164	template	24	Intron_5	--	Yes	--	Yes	--	--	--	--	--	--	Yes
G4ST01084609081	chr1:84609082-84609110	+	GGGACAGCTGTGGGAGTGGGTGGTGGGGG<br>((....))	UOX	NR_003927	chr1:84603229-84636164	template	29	Intron_5	--	Yes	--	Yes	--	--	--	--	--	--	Yes
G4ST01084611184	chr1:84611185-84611225	+	GGGACGAACTTGGTTCATGGGTACCTGGGGGGAGAGAAGGG<br>..((((...))))..	UOX	NR_003927	chr1:84603229-84636164	template	41	Intron_4	--	Yes	--	Yes	--	--	--	--	--	--	--
G4ST01084744197	chr1:84744198-84744232	-	GGGATCCGGCGGCCCAGCTGGCGGGGGCGGGCGGG<br>..(((.((((...)))).)))	GNG5	NM_005274	chr1:84736594-84744850	non-template	35	Intron_2	--	Yes	--	Yes	--	--	--	Yes	Yes	Yes	--
G4ST01084744197	chr1:84744198-84744232	-	GGGATCCGGCGGCCCAGCTGGCGGGGGCGGGCGGG<br>..(((.((((...)))).)))	SPATA1	NR_036561	chr1:84744562-84804465	template	-329	upstream	Yes	--	--	--	--	--	--	Yes	Yes	Yes	--
G4ST01084744201	chr1:84744202-84744236	-	GGGTGGGATCCGGCGGCCCAGCTGGCGGGGGCGGG<br>..(((.((((...)))).)))	GNG5	NM_005274	chr1:84736594-84744850	non-template	35	Intron_2	--	Yes	--	Yes	--	--	--	Yes	Yes	Yes	--
G4ST01084744201	chr1:84744202-84744236	-	GGGTGGGATCCGGCGGCCCAGCTGGCGGGGGCGGG<br>..(((.((((...)))).)))	SPATA1	NR_036561	chr1:84744562-84804465	template	-325	upstream	Yes	--	--	--	--	--	--	Yes	Yes	Yes	--
G4ST01084891721	chr1:84891722-84891744	-	GGGCATAGTGAGGGATGGGAGGG<br>((...)).	SSX2IP	NM_001166295	chr1:84881978-84928739	non-template	23	Intron_10	--	Yes	--	Yes	--	--	--	Yes	--	--	--
G4ST01084891721	chr1:84891722-84891744	-	GGGCATAGTGAGGGATGGGAGGG<br>((...)).	SSX2IP	NM_001166417	chr1:84881978-84928739	non-template	23	Intron_12	--	Yes	--	Yes	--	--	--	Yes	--	--	--

G4ST01084891721 chr1:84891722-84891744	-	GGGCATAGTGAGGGATGGGAGGG<br>((...)).	SSX2IP	NM_001166293	chr1:84881978-84928828	non-template	23	Intron_11	--	Yes	--	Yes	--	--	--	Yes	--	--	--
G4ST01084891721 chr1:84891722-84891744	-	GGGCATAGTGAGGGATGGGAGGG<br>((...)).	SSX2IP	NM_001166294	chr1:84881978-84928828	non-template	23	Intron_10	--	Yes	--	Yes	--	--	--	Yes	--	--	--
G4ST01084891721 chr1:84891722-84891744	-	GGGCATAGTGAGGGATGGGAGGG<br>((...)).	SSX2IP	NM_014021	chr1:84881978-84928828	non-template	23	Intron_12	--	Yes	--	Yes	--	--	--	Yes	--	--	--
G4ST01084891725 chr1:84891726-84891752	-	GGGACAGTGGGCATAGTGAGGGATGGG<br>((...)).	SSX2IP	NM_001166295	chr1:84881978-84928739	non-template	27	Intron_10	--	Yes	--	Yes	--	--	--	Yes	--	--	--
G4ST01084891725 chr1:84891726-84891752	-	GGGACAGTGGGCATAGTGAGGGATGGG<br>((...)).	SSX2IP	NM_001166417	chr1:84881978-84928739	non-template	27	Intron_12	--	Yes	--	Yes	--	--	--	Yes	--	--	--
G4ST01084891725 chr1:84891726-84891752	-	GGGACAGTGGGCATAGTGAGGGATGGG<br>((...)).	SSX2IP	NM_001166293	chr1:84881978-84928828	non-template	27	Intron_11	--	Yes	--	Yes	--	--	--	Yes	--	--	--
G4ST01084891725 chr1:84891726-84891752	-	GGGACAGTGGGCATAGTGAGGGATGGG<br>((...)).	SSX2IP	NM_001166294	chr1:84881978-84928828	non-template	27	Intron_10	--	Yes	--	Yes	--	--	--	Yes	--	--	--
G4ST01084891725 chr1:84891726-84891752	-	GGGACAGTGGGCATAGTGAGGGATGGG<br>((...)).	SSX2IP	NM_014021	chr1:84881978-84928828	non-template	27	Intron_12	--	Yes	--	Yes	--	--	--	Yes	--	--	--
G4ST01085092453 chr1:85092454-85092487	-	GGGAGGCCATGCCAGGGCCGTGGGTAGAAAGGGG<br>.(((...))).	LPAR3	NM_012152	chr1:85051674-85131484	non-template	34	Intron_2.l	--	Yes	--	Yes	--	--	--	Yes	Yes	--	Yes
G4ST01085110062 chr1:85110063-85110096	-	GGGTATCTTGGGGAAAAGTCTTCTGGGCAGAGGG<br>(((.....))).	LPAR3	NM_012152	chr1:85051674-85131484	non-template	34	Intron_1.f	--	Yes	--	Yes	--	--	--	--	--	--	--
G4ST01085131350 chr1:85131351-85131384	+	GGGGTCGAGCGCGACGGGGCGACCGGGCGCCGGG<br>((((....)))).	LPAR3	NM_012152	chr1:85051674-85131484	template	34	Exon_1.f	--	Yes	Yes	--	--	Yes	--	--	Yes	Yes	--
G4ST01085286544 chr1:85286545-85286585	-	GGGAGGCAGCGAGCTGTCGGGGACGCCGCGGGAAGGCCGGG<br>.(((((....))))).	MCOLN3	NM_018298	chr1:85256353-85286757	non-template	41	Intron_1.f	--	Yes	--	Yes	--	--	--	--	Yes	Yes	Yes
G4ST01085286553 chr1:85286554-85286594	-	GGGGAGGCCGGGAGGCAGCGAGCTGTCGGGGACGCCGCGGG<br>.(((((....))))).	MCOLN3	NM_018298	chr1:85256353-85286757	non-template	41	Intron_1.f	--	Yes	--	Yes	--	--	--	--	Yes	Yes	Yes
G4ST01085286563 chr1:85286564-85286599	-	GGGCCGGGGAGGCCGGGAGGCAGCGAGCTGTCGGGG<br>.(((((....))))).	MCOLN3	NM_018298	chr1:85256353-85286757	non-template	36	Intron_1.f	--	Yes	--	Yes	--	--	--	--	Yes	Yes	Yes
G4ST01085286590 chr1:85286591-85286626	-	GGGAACGGCGCCCGCGGCGCACAGGGAGGGCCGGGG<br>....(((((...)))))...	MCOLN3	NM_018298	chr1:85256353-85286757	non-template	36	Intron_1.f	--	Yes	--	Yes	--	--	--	--	Yes	Yes	Yes
G4ST01085286596 chr1:85286597-85286635	-	GGGTGTCGCGGGAACGGCGCCCGCGGCGCACAGGGAGGG<br>....(((((...)))))...	MCOLN3	NM_018298	chr1:85256353-85286757	non-template	39	Intron_1.f	--	Yes	--	Yes	--	--	--	--	Yes	Yes	Yes
G4ST01085286600 chr1:85286601-85286643	-	GGGGCGTCGGGTGTCGCGGGAACGGCGCCCGCGGCGCACAGGG<br>....(((((...)))))...	MCOLN3	NM_018298	chr1:85256353-85286757	non-template	43	Intron_1.f	--	Yes	--	Yes	--	--	--	--	Yes	Yes	Yes
G4ST01085286643 chr1:85286644-85286682	+	GGGGCAGCGCCCCGCGGGCTCACCTGGGGGACAGCGGGG<br>...(((...)))	MCOLN3	NM_018298	chr1:85256353-85286757	template	39	Exon_1.f-Intron_1.f	--	Yes	Yes	Yes	--	Yes	--	Yes	Yes	Yes	--
G4ST01085514512 chr1:85514513-85514540	-	GGGCGGGGGCGGGCTGCAGCCCAGCGGG<br>(((.....))).	BCL10	NM_003921	chr1:85504048-85515175	non-template	28	Intron_1.f	--	Yes	--	Yes	--	--	--	Yes	Yes	Yes	Yes
G4ST01085570464 chr1:85570465-85570500	-	GGGATGTGAAGATACAGGGGTTGCTGGGTCCCAGGG<br>.((((....)))).	DDAH1	NM_001134445	chr1:85556756-85816634	non-template	36	Intron_5	--	Yes	--	Yes	--	--	--	--	--	--	--
G4ST01085570464 chr1:85570465-85570500	-	GGGATGTGAAGATACAGGGGTTGCTGGGTCCCAGGG<br>.((((....)))).	DDAH1	NM_012137	chr1:85556756-85703477	non-template	36	Intron_4	--	Yes	--	Yes	--	--	--	--	--	--	--
G4ST01085570472 chr1:85570473-85570509	-	GGGGCTCCAGGGATGTGAAGATACAGGGGTTGCTGGG<br>.((((....)))).	DDAH1	NM_001134445	chr1:85556756-85816634	non-template	37	Intron_5	--	Yes	--	Yes	--	--	--	--	--	--	--
G4ST01085570472 chr1:85570473-85570509	-	GGGGCTCCAGGGATGTGAAGATACAGGGGTTGCTGGG<br>.((((....)))).	DDAH1	NM_012137	chr1:85556756-85703477	non-template	37	Intron_4	--	Yes	--	Yes	--	--	--	--	--	--	--
G4ST01085572753 chr1:85572754-85572791	+	GGGGGAGGGACCCGGAGGGAGATGATTGGTCATGGGGG<br>..((((....))))..	DDAH1	NM_001134445	chr1:85556756-85816634	template	38	Intron_5	--	Yes	--	Yes	--	--	--	--	--	--	--
G4ST01085572753 chr1:85572754-85572791	+	GGGGGAGGGACCCGGAGGGAGATGATTGGTCATGGGGG<br>..((((....))))..	DDAH1	NM_012137	chr1:85556756-85703477	template	38	Intron_4	--	Yes	--	Yes	--	--	--	--	--	--	--
G4ST01085684934 chr1:85684935-85684969	-	GGGAGAGGAAGGGAGGTGGGATTGAAACAAGGGGG<br>.(((...)))..	DDAH1	NM_001134445	chr1:85556756-85816634	non-template	35	Intron_2	--	Yes	--	Yes	--	--	--	--	--	--	Yes
G4ST01085684934 chr1:85684935-85684969	-	GGGAGAGGAAGGGAGGTGGGATTGAAACAAGGGGG<br>.(((...)))..	DDAH1	NM_012137	chr1:85556756-85703477	non-template	35	Intron_1.f	--	Yes	--	Yes	--	--	--	--	--	--	Yes
G4ST01085704035 chr1:85704036-85704070	-	GGGCTCCAGGACTGGAGGGGGTTTAGGGTGGGGGG<br>(((((....))))).	DDAH1	NM_001134445	chr1:85556756-85816634	non-template	35	Intron_2	--	Yes	--	Yes	--	--	--	--	Yes	Yes	--
G4ST01085704035 chr1:85704036-85704070	-	GGGCTCCAGGACTGGAGGGGGTTTAGGGTGGGGGG<br>(((((....))))).	DDAH1	NM_012137	chr1:85556756-85703477	non-template	-558	upstream	Yes	--	--	--	--	--	--	--	Yes	Yes	--
G4ST01086114610 chr1:86114611-86114649	+	GGGTTATTGGGTATAAGGGATATCTTTCATTGAGATGGG<br>..(((((.....)))))	COL24A1	NM_152890	chr1:85967504-86394709	template	39	Intron_33	--	Yes	--	Yes	--	--	--	--	--	--	--
G4ST01086214722 chr1:86214723-86214757	-	GGGCAGGGCTTGCCACACTCCTGTGGGGGAGGGGG<br>....(((((....)))))	COL24A1	NM_152890	chr1:85967504-86394709	non-template	35	Intron_20	--	Yes	--	Yes	--	--	--	--	--	--	--
G4ST01086278730 chr1:86278731-86278771	-	GGGACAACTCAAAGAGCCTTGTAGGGGTGGGGGGAGGGGGG<br>(((((((...)))..))))..	COL24A1	NM_152890	chr1:85967504-86394709	non-template	41	Intron_12	--	Yes	--	Yes	--	--	--	--	--	--	Yes
G4ST01086306562 chr1:86306563-86306592	-	GGGCTGCTGCCAGGGGAGATGGGGGAGGGG<br>(((....)))	COL24A1	NM_152890	chr1:85967504-86394709	non-template	30	Intron_7	--	Yes	--	Yes	--	--	--	Yes	--	--	--
G4ST01086725748 chr1:86725749-86725784	+	GGGTGTGGGGTGGGAGAAGCTCATGTGGCTTCAGGG<br>.((((((.....)))))).	CLCA1	NM_001285	chr1:86707114-86738562	non-template	36	Intron_7	--	Yes	--	Yes	--	--	--	Yes	--	--	--
G4ST01087152990 chr1:87152991-87153024	+	GGGCGCGGGGGTCGGGGACTGAGGCAGTAGAGGG<br>.((((...))))...	SEP15	NM_203341	chr1:87100716-87152695	template	-295	upstream	Yes	--	--	--	--	--	--	--	Yes	Yes	--
G4ST01087152990 chr1:87152991-87153024	+	GGGCGCGGGGGTCGGGGACTGAGGCAGTAGAGGG<br>.((((...))))...	SEP15	NM_004261	chr1:87100716-87152695	template	-295	upstream	Yes	--	--	--	--	--	--	--	Yes	Yes	--
G4ST01087152990 chr1:87152991-87153024	+	GGGCGCGGGGGTCGGGGACTGAGGCAGTAGAGGG<br>.((((...))))...	HS2ST1	NM_012262	chr1:87152923-87348269	non-template	34	Exon_1.f	--	Yes	Yes	--	--	Yes	--	--	Yes	Yes	--
G4ST01087152990 chr1:87152991-87153024	+	GGGCGCGGGGGTCGGGGACTGAGGCAGTAGAGGG<br>.((((...))))...	HS2ST1	NM_001134492	chr1:87152923-87336713	non-template	34	Exon_1.f	--	Yes	Yes	--	--	Yes	--	--	Yes	Yes	--
G4ST01087201954 chr1:87201955-87201999	+	GGGGGTGACAGCCGGGCAGAGGGGCTTCTCACTTCCCAGAAGGGG<br>(((....))) .(((((........)))))	HS2ST1	NM_012262	chr1:87152923-87348269	non-template	45	Intron_1.f	--	Yes	--	Yes	--	--	--	--	--	--	--
G4ST01087201954 chr1:87201955-87201999	+	GGGGGTGACAGCCGGGCAGAGGGGCTTCTCACTTCCCAGAAGGGG<br>(((....))) .(((((........)))))	HS2ST1	NM_001134492	chr1:87152923-87336713	non-template	45	Intron_1.f	--	Yes	--	Yes	--	--	--	--	--	--	--
G4ST01087375481 chr1:87375482-87375525	+	GGGGGAGGCGGTGGGGATGGGGATGAGGTAGATCGCATTTAGGG<br>.(((.((....)).)))...	LOC339524	NR_026989	chr1:87368036-87407474	non-template	44	Intron_2.l	--	Yes	--	Yes	--	--	--	Yes	--	--	--
G4ST01087376225 chr1:87376226-87376250	+	GGGATGAGCATGGGCTTGGGTTGGG<br>((....))	LOC339524	NR_026989	chr1:87368036-87407474	non-template	25	Intron_2.l	--	Yes	--	Yes	--	--	--	--	--	--	--
G4ST01087567282 chr1:87567283-87567320	-	GGGTTGGGGGGCCCCCGCGTAAGTGCCGGGCGGCCGGG<br>.(((....((....)))))	LMO4	NM_006769	chr1:87566739-87587194	template	38	Exon_1.f	--	Yes	Yes	--	--	Yes	--	Yes	Yes	Yes	--
G4ST01087567290 chr1:87567291-87567325	-	GGGGCGGGTTGGGGGGCCCCCGCGTAAGTGCCGGG<br>.(((....((....)))))	LMO4	NM_006769	chr1:87566739-87587194	template	35	Exon_1.f	--	Yes	Yes	--	--	Yes	--	Yes	Yes	Yes	--
G4ST01087572950 chr1:87572951-87572991	+	GGGAGAGCTGGGAAAGGCCGGCCTGAGCGCGGGGGAGGGGG<br>.....(((((....)).)))	LMO4	NM_006769	chr1:87566739-87587194	non-template	41	Intron_2	--	Yes	--	Yes	--	--	--	Yes	Yes	Yes	--
G4ST01088923190 chr1:88923191-88923221	-	GGGAGGGGGCGACGGGCCGGCCTCTCCGGGG<br>.(((.....)))	PKN2	NM_006256	chr1:88922510-89074526	template	31	Intron_1.f	--	Yes	--	Yes	--	--	--	Yes	Yes	Yes	--
G4ST01090059927 chr1:90059928-90059955	+	GGGTGGGCTGCGCTGGGAGCCCGGCGGG<br>.((...))	LRRC8D	NM_018103	chr1:90060068-90174577	non-template	-112	upstream	Yes	--	--	--	--	--	--	Yes	Yes	Yes	--
G4ST01090059927 chr1:90059928-90059955	+	GGGTGGGCTGCGCTGGGAGCCCGGCGGG<br>.((...))	LRRC8D	NM_001134479	chr1:90059161-90174577	non-template	28	Intron_1.f	--	Yes	--	Yes	--	--	--	Yes	Yes	Yes	--
G4ST01090059931 chr1:90059932-90059961	+	GGGCTGCGCTGGGAGCCCGGCGGGCGGGGG<br>.((...))	LRRC8D	NM_018103	chr1:90060068-90174577	non-template	-106	upstream	Yes	--	--	--	--	--	--	Yes	Yes	Yes	--
G4ST01090059931 chr1:90059932-90059961	+	GGGCTGCGCTGGGAGCCCGGCGGGCGGGGG<br>.((...))	LRRC8D	NM_001134479	chr1:90059161-90174577	non-template	30	Intron_1.f	--	Yes	--	Yes	--	--	--	Yes	Yes	Yes	--

G4ST01090059941	chr1:90059942-90059966	+	GGGAGCCCGGCGGGCGGGGGCGGGG<br>.((...))	LRRC8D	NM_018103	chr1:90060068-90174577	non-template	-101	upstream	Yes	--	--	--	--	--	--	Yes	Yes	Yes	--
G4ST01090059941	chr1:90059942-90059966	+	GGGAGCCCGGCGGGCGGGGGCGGGG<br>.((...))	LRRC8D	NM_001134479	chr1:90059161-90174577	non-template	25	Intron_1.f	--	Yes	--	Yes	--	--	--	Yes	Yes	Yes	--
G4ST01090233161	chr1:90233162-90233187	-	GGGCGGGCCCTCGCGTGGGGGGCGGG<br>(((......)))	GEMIN8P4	NR_002830	chr1:90231412-90233113	non-template	-48	upstream	Yes	--	--	--	--	--	--	Yes	Yes	Yes	--
G4ST01090233161	chr1:90233162-90233187	-	GGGCGGGCCCTCGCGTGGGGGGCGGG<br>(((......)))	ZNF326	NM_182975	chr1:90233266-90244588	template	-78	upstream	Yes	--	--	--	--	--	--	Yes	Yes	Yes	--
G4ST01090233161	chr1:90233162-90233187	-	GGGCGGGCCCTCGCGTGGGGGGCGGG<br>(((......)))	ZNF326	NM_182976	chr1:90233266-90266682	template	-78	upstream	Yes	--	--	--	--	--	--	Yes	Yes	Yes	--
G4ST01090233165	chr1:90233166-90233195	-	GGGCTGGTGGGCGGGCCCTCGCGTGGGGGG<br>(((......)))	GEMIN8P4	NR_002830	chr1:90231412-90233113	non-template	-52	upstream	Yes	--	--	--	--	--	--	Yes	Yes	Yes	--
G4ST01090233165	chr1:90233166-90233195	-	GGGCTGGTGGGCGGGCCCTCGCGTGGGGGG<br>(((......)))	ZNF326	NM_182975	chr1:90233266-90244588	template	-70	upstream	Yes	--	--	--	--	--	--	Yes	Yes	Yes	--
G4ST01090233165	chr1:90233166-90233195	-	GGGCTGGTGGGCGGGCCCTCGCGTGGGGGG<br>(((......)))	ZNF326	NM_182976	chr1:90233266-90266682	template	-70	upstream	Yes	--	--	--	--	--	--	Yes	Yes	Yes	--
G4ST01090233527	chr1:90233528-90233550	-	GGGGCGGGCGGGCGGCCTCCGGG<br>.((...))	GEMIN8P4	NR_002830	chr1:90231412-90233113	non-template	-414	upstream	Yes	--	--	--	--	--	--	Yes	Yes	Yes	Yes
G4ST01090233527	chr1:90233528-90233550	-	GGGGCGGGCGGGCGGCCTCCGGG<br>.((...))	ZNF326	NM_182975	chr1:90233266-90244588	template	23	Intron_1.f	--	Yes	--	Yes	--	--	--	Yes	Yes	Yes	Yes
G4ST01090233527	chr1:90233528-90233550	-	GGGGCGGGCGGGCGGCCTCCGGG<br>.((...))	ZNF326	NM_182976	chr1:90233266-90266682	template	23	Intron_1.f	--	Yes	--	Yes	--	--	--	Yes	Yes	Yes	Yes
G4ST01090233583	chr1:90233584-90233616	-	GGGCGCCGAGCGGGCCGAATCGGGGCGCGTGGG<br>.((...))	GEMIN8P4	NR_002830	chr1:90231412-90233113	non-template	-470	upstream	Yes	--	--	--	--	--	--	Yes	Yes	Yes	--
G4ST01090233583	chr1:90233584-90233616	-	GGGCGCCGAGCGGGCCGAATCGGGGCGCGTGGG<br>.((...))	ZNF326	NM_182975	chr1:90233266-90244588	template	33	Intron_1.f	--	Yes	--	Yes	--	--	--	Yes	Yes	Yes	--
G4ST01090233583	chr1:90233584-90233616	-	GGGCGCCGAGCGGGCCGAATCGGGGCGCGTGGG<br>.((...))	ZNF326	NM_182976	chr1:90233266-90266682	template	33	Intron_1.f	--	Yes	--	Yes	--	--	--	Yes	Yes	Yes	--
G4ST01090950930	chr1:90950931-90950962	-	GGGTTTGGGTCTTTCTGGGAGCAAGGCTGGGG<br>(((...))).	BARHL2	NM_020063	chr1:90950167-90955382	non-template	32	Intron_2.l	--	Yes	--	Yes	--	--	--	--	--	--	--
G4ST01091260072	chr1:91260073-91260101	-	GGGACCCGAGTGGGTTTCGGGCCTCTGGG<br>((....))	ZNF644	NM_016620	chr1:91153445-91259618	non-template	-454	upstream	Yes	--	--	--	--	--	--	--	Yes	Yes	--
G4ST01091260072	chr1:91260073-91260101	-	GGGACCCGAGTGGGTTTCGGGCCTCTGGG<br>((....))	ZNF644	NM_032186	chr1:91153445-91260259	non-template	29	Intron_1.f	--	Yes	--	Yes	--	--	--	--	Yes	Yes	--
G4ST01091260072	chr1:91260073-91260101	-	GGGACCCGAGTGGGTTTCGGGCCTCTGGG<br>((....))	ZNF644	NM_201269	chr1:91153445-91260259	non-template	29	Intron_1.f	--	Yes	--	Yes	--	--	--	--	Yes	Yes	--
G4ST01091556360	chr1:91556361-91556400	-	GGGCTGACTAGGAAGTCATGGGAGCTGGGGTGAAGTGGGG<br>..((((....))))..	HFM1	NM_001017975	chr1:91498911-91643014	non-template	40	Intron_25	--	Yes	--	Yes	--	--	--	--	--	--	--
G4ST01091556370	chr1:91556371-91556411	-	GGGGTGGCAGAGGGCTGACTAGGAAGTCATGGGAGCTGGGG<br>..((((....))))..	HFM1	NM_001017975	chr1:91498911-91643014	non-template	41	Intron_25	--	Yes	--	Yes	--	--	--	--	--	--	--
G4ST01091575196	chr1:91575197-91575231	+	GGGGAACAACACACGTTGGGGTCTGTTGGGGAGGG<br>...((((....))))	HFM1	NM_001017975	chr1:91498911-91643014	template	35	Intron_20	--	Yes	--	Yes	--	--	--	--	--	--	Yes
G4ST01091739484	chr1:91739485-91739518	-	GGGACCGCGTGGGGGAGGGGCCGAGCGCCGGGGG<br>.(((.....)))	CDC7	NM_001134419	chr1:91738992-91763909	template	34	Intron_1.f	--	Yes	--	Yes	--	--	--	Yes	Yes	Yes	--
G4ST01091739484	chr1:91739485-91739518	-	GGGACCGCGTGGGGGAGGGGCCGAGCGCCGGGGG<br>.(((.....)))	CDC7	NM_003503	chr1:91738992-91763909	template	34	Intron_1.f	--	Yes	--	Yes	--	--	--	Yes	Yes	Yes	--
G4ST01091739484	chr1:91739485-91739518	-	GGGACCGCGTGGGGGAGGGGCCGAGCGCCGGGGG<br>.(((.....)))	CDC7	NM_001134420	chr1:91739253-91763909	template	34	Intron_1.f	--	Yes	--	Yes	--	--	--	Yes	Yes	Yes	--
G4ST01091941457	chr1:91941458-91941480	-	GGGAAGGGAGGGTTGAGGAAGGG<br>((....))	TGFBR3	NM_001195684	chr1:91918488-92144147	non-template	23	Intron_15	--	Yes	--	Yes	--	--	--	--	--	--	--
G4ST01091941457	chr1:91941458-91941480	-	GGGAAGGGAGGGTTGAGGAAGGG<br>((....))	TGFBR3	NM_001195683	chr1:91918488-92124424	non-template	23	Intron_14	--	Yes	--	Yes	--	--	--	--	--	--	--
G4ST01091941457	chr1:91941458-91941480	-	GGGAAGGGAGGGTTGAGGAAGGG<br>((....))	TGFBR3	NM_003243	chr1:91918488-92124424	non-template	23	Intron_14	--	Yes	--	Yes	--	--	--	--	--	--	--
G4ST01091941457	chr1:91941458-91941480	-	GGGAAGGGAGGGTTGAGGAAGGG<br>((....))	TGFBR3	NR_036634	chr1:91918488-92124424	non-template	23	Intron_15	--	Yes	--	Yes	--	--	--	--	--	--	--
G4ST01091957727	chr1:91957728-91957759	-	GGGCAGCTGGGAAGAGATACTTGGGGAAGGGG<br>(((.....))).	TGFBR3	NM_001195684	chr1:91918488-92144147	non-template	32	Intron_10	--	Yes	--	Yes	--	--	--	--	--	--	--
G4ST01091957727	chr1:91957728-91957759	-	GGGCAGCTGGGAAGAGATACTTGGGGAAGGGG<br>(((.....))).	TGFBR3	NM_001195683	chr1:91918488-92124424	non-template	32	Intron_9	--	Yes	--	Yes	--	--	--	--	--	--	--
G4ST01091957727	chr1:91957728-91957759	-	GGGCAGCTGGGAAGAGATACTTGGGGAAGGGG<br>(((.....))).	TGFBR3	NM_003243	chr1:91918488-92124424	non-template	32	Intron_9	--	Yes	--	Yes	--	--	--	--	--	--	--
G4ST01091957727	chr1:91957728-91957759	-	GGGCAGCTGGGAAGAGATACTTGGGGAAGGGG<br>(((.....))).	TGFBR3	NR_036634	chr1:91918488-92124424	non-template	32	Intron_10	--	Yes	--	Yes	--	--	--	--	--	--	--
G4ST01092057880	chr1:92057881-92057912	-	GGGCATCCTGTGGATTTAGGGGAGGGAGGGGG<br>.((((...))))....	TGFBR3	NM_001195684	chr1:91918488-92144147	non-template	32	Intron_3	--	Yes	--	Yes	--	--	--	Yes	Yes	--	--
G4ST01092057880	chr1:92057881-92057912	-	GGGCATCCTGTGGATTTAGGGGAGGGAGGGGG<br>.((((...))))....	TGFBR3	NM_001195683	chr1:91918488-92124424	non-template	32	Intron_2	--	Yes	--	Yes	--	--	--	Yes	Yes	--	--
G4ST01092057880	chr1:92057881-92057912	-	GGGCATCCTGTGGATTTAGGGGAGGGAGGGGG<br>.((((...))))....	TGFBR3	NM_003243	chr1:91918488-92124424	non-template	32	Intron_2	--	Yes	--	Yes	--	--	--	Yes	Yes	--	--
G4ST01092057880	chr1:92057881-92057912	-	GGGCATCCTGTGGATTTAGGGGAGGGAGGGGG<br>.((((...))))....	TGFBR3	NR_036634	chr1:91918488-92124424	non-template	32	Intron_2	--	Yes	--	Yes	--	--	--	Yes	Yes	--	--
G4ST01092123934	chr1:92123935-92123983	-	GGGCTGTGGGCTCTGCAGGCTGCGCGGGTGCCCGTCGGCGCGGCGCGGG<br>...((((...)))). .(((((....)).))))..	TGFBR3	NM_001195684	chr1:91918488-92144147	non-template	49	Intron_2	--	Yes	--	Yes	--	--	--	--	Yes	Yes	--
G4ST01092123934	chr1:92123935-92123983	-	GGGCTGTGGGCTCTGCAGGCTGCGCGGGTGCCCGTCGGCGCGGCGCGGG<br>...((((...)))). .(((((....)).))))..	TGFBR3	NM_001195683	chr1:91918488-92124424	non-template	49	Intron_1.f	--	Yes	--	Yes	--	--	--	--	Yes	Yes	--
G4ST01092123934	chr1:92123935-92123983	-	GGGCTGTGGGCTCTGCAGGCTGCGCGGGTGCCCGTCGGCGCGGCGCGGG<br>...((((...)))). .(((((....)).))))..	TGFBR3	NM_003243	chr1:91918488-92124424	non-template	49	Intron_1.f	--	Yes	--	Yes	--	--	--	--	Yes	Yes	--
G4ST01092123934	chr1:92123935-92123983	-	GGGCTGTGGGCTCTGCAGGCTGCGCGGGTGCCCGTCGGCGCGGCGCGGG<br>...((((...)))). .(((((....)).))))..	TGFBR3	NR_036634	chr1:91918488-92124424	non-template	49	Intron_1.f	--	Yes	--	Yes	--	--	--	--	Yes	Yes	--
G4ST01092188656	chr1:92188657-92188690	+	GGGTGGATGGGAGGGAATGAGAGAGGCTCTTGGG<br>....((((...)))).	BRDT	NM_207189	chr1:92187516-92252573	non-template	34	Intron_1.f	--	Yes	--	Yes	--	--	--	--	--	--	--
G4ST01092188656	chr1:92188657-92188690	+	GGGTGGATGGGAGGGAATGAGAGAGGCTCTTGGG<br>....((((...)))).	BRDT	NM_001726	chr1:92190297-92252573	non-template	-1606	upstream	Yes	--	--	--	--	--	--	--	--	--	--
G4ST01092718666	chr1:92718667-92718701	+	GGGGCCGCGGGGCGCAGGCGAGGCGCGGGTAGGGG<br>((((.......))))	GFI1	NM_005263	chr1:92712906-92725021	template	35	Intron_4	--	Yes	--	Yes	--	--	--	Yes	--	Yes	--
G4ST01092718666	chr1:92718667-92718701	+	GGGGCCGCGGGGCGCAGGCGAGGCGCGGGTAGGGG<br>((((.......))))	GFI1	NM_001127215	chr1:92712906-92721944	template	35	Intron_4	--	Yes	--	Yes	--	--	--	Yes	--	Yes	--
G4ST01092718666	chr1:92718667-92718701	+	GGGGCCGCGGGGCGCAGGCGAGGCGCGGGTAGGGG<br>((((.......))))	GFI1	NM_001127216	chr1:92712906-92724216	template	35	Intron_4	--	Yes	--	Yes	--	--	--	Yes	--	Yes	--
G4ST01092718674	chr1:92718675-92718708	+	GGGGCGCAGGCGAGGCGCGGGTAGGGGAAGCGGG<br>((((.......))))	GFI1	NM_005263	chr1:92712906-92725021	template	34	Intron_4	--	Yes	--	Yes	--	--	--	Yes	--	Yes	--
G4ST01092718674	chr1:92718675-92718708	+	GGGGCGCAGGCGAGGCGCGGGTAGGGGAAGCGGG<br>((((.......))))	GFI1	NM_001127215	chr1:92712906-92721944	template	34	Intron_4	--	Yes	--	Yes	--	--	--	Yes	--	Yes	--
G4ST01092718674	chr1:92718675-92718708	+	GGGGCGCAGGCGAGGCGCGGGTAGGGGAAGCGGG<br>((((.......))))	GFI1	NM_001127216	chr1:92712906-92724216	template	34	Intron_4	--	Yes	--	Yes	--	--	--	Yes	--	Yes	--
G4ST01092718966	chr1:92718967-92719001	-	GGGGGCCGGGGCGCCAGGGAGCTGCAGCGCAGGGG<br>..((((...))))	GFI1	NM_005263	chr1:92712906-92725021	non-template	35	Exon_4	--	Yes	Yes	--	Yes	--	--	--	--	Yes	--
G4ST01092718966	chr1:92718967-92719001	-	GGGGGCCGGGGCGCCAGGGAGCTGCAGCGCAGGGG<br>..((((...))))	GFI1	NM_001127215	chr1:92712906-92721944	non-template	35	Exon_4	--	Yes	Yes	--	Yes	--	--	--	--	Yes	--

G4ST01092718966	chr1:92718967-92719001	-	GGGGGCCGGGGCGCCAGGGAGCTGCAGCGCAGGGG<br>..((((...))))	GFI1	NM_001127216	chr1:92712906-92724216	non-template	35	Exon_4	--	Yes	Yes	--	Yes	--	--	--	--	Yes	--
G4ST01092720967	chr1:92720968-92720995	-	GGGAACCTCTTGGGCGGGAGCTGCAGGG<br>((....))	GFI1	NM_005263	chr1:92712906-92725021	non-template	28	Intron_3	--	Yes	--	Yes	--	--	--	Yes	--	Yes	--
G4ST01092720967	chr1:92720968-92720995	-	GGGAACCTCTTGGGCGGGAGCTGCAGGG<br>((....))	GFI1	NM_001127215	chr1:92712906-92721944	non-template	28	Intron_3	--	Yes	--	Yes	--	--	--	Yes	--	Yes	--
G4ST01092720967	chr1:92720968-92720995	-	GGGAACCTCTTGGGCGGGAGCTGCAGGG<br>((....))	GFI1	NM_001127216	chr1:92712906-92724216	non-template	28	Intron_3	--	Yes	--	Yes	--	--	--	Yes	--	Yes	--
G4ST01092853730	chr1:92853731-92853763	-	GGGGAGGGGTTGTTTCCACTACTGGGGATGGGG<br>((.((....)).))..	EVI5	NM_005665	chr1:92746841-93030549	non-template	33	Intron_14	--	Yes	--	Yes	--	--	--	--	--	--	--
G4ST01092853736	chr1:92853737-92853768	-	GGGTTGGGGAGGGGTTGTTTCCACTACTGGGG<br>((.((....)).))..	EVI5	NM_005665	chr1:92746841-93030549	non-template	32	Intron_14	--	Yes	--	Yes	--	--	--	--	--	--	--
G4ST01093069741	chr1:93069742-93069778	+	GGGGCTCTCAGGGAAGGCTTCCTGGGGGTGAGATGGG<br>.(((...)))..	RPL5	NM_000969	chr1:93070182-93080069	non-template	-403	upstream	Yes	--	--	--	--	--	--	Yes	Yes	--	Yes
G4ST01093069751	chr1:93069752-93069782	+	GGGAAGGCTTCCTGGGGGTGAGATGGGAGGG<br>.(((...)))..	RPL5	NM_000969	chr1:93070182-93080069	non-template	-399	upstream	Yes	--	--	--	--	--	--	Yes	Yes	--	Yes
G4ST01093199616	chr1:93199617-93199660	-	GGGGAGGCGGGGGGAGGCGGGGAGCCCGGCCGCCAGCGCTCGGG<br>((((...((.....))))))	FAM69A	NM_001006605	chr1:93080309-93199667	non-template	44	Exon_1.f	--	Yes	Yes	--	--	Yes	--	Yes	Yes	Yes	Yes
G4ST01093199682	chr1:93199683-93199723	-	GGGGAGCGCGGCGCGCGCCGCGGCGGGGGCGGGGGAGTGGG<br>...((((((....)))))))....	FAM69A	NM_001006605	chr1:93080309-93199667	non-template	-15	upstream	Yes	--	--	--	--	--	--	Yes	Yes	Yes	Yes
G4ST01093199688	chr1:93199689-93199730	-	GGGCCGAGGGGAGCGCGGCGCGCGCCGCGGCGGGGGCGGGGG<br>...((((((....))))))....	FAM69A	NM_001006605	chr1:93080309-93199667	non-template	-21	upstream	Yes	--	--	--	--	--	--	Yes	Yes	Yes	Yes
G4ST01093555784	chr1:93555785-93555811	-	GGGCTTGGGTGGGCATTCTGGAATGGG<br>.(((....)))	LOC100131564	NR_034089	chr1:93548254-93583956	non-template	27	Intron_8.l	--	Yes	--	Yes	--	--	--	--	--	--	--
G4ST01093583950	chr1:93583951-93583983	+	GGGCGGCTCCCGCCGGCGTGGGTGGGGGAAGGG<br>((((....))))....	LOC100131564	NR_034089	chr1:93548254-93583956	template	6	upstream-Exon_1.f	Yes	Yes	Yes	--	--	--	--	Yes	Yes	Yes	--
G4ST01093583950	chr1:93583951-93583983	+	GGGCGGCTCCCGCCGGCGTGGGTGGGGGAAGGG<br>((((....))))....	DR1	NM_001938	chr1:93584066-93600736	non-template	-82	upstream	Yes	--	--	--	--	--	--	Yes	Yes	Yes	--
G4ST01093596648	chr1:93596649-93596686	-	GGGATCCACACTGCCAGAGTGGGGGGATGGGGTGCGGG<br>..((((.((...)).)))).	DR1	NM_001938	chr1:93584066-93600736	template	38	Intron_2.l	--	Yes	--	Yes	--	--	--	Yes	--	--	--
G4ST01093596654	chr1:93596655-93596691	-	GGGTTGGGATCCACACTGCCAGAGTGGGGGGATGGGG<br>..((((.((...)).)))).	DR1	NM_001938	chr1:93584066-93600736	template	37	Intron_2.l	--	Yes	--	Yes	--	--	--	Yes	--	--	--
G4ST01093596660	chr1:93596661-93596700	-	GGGCCCCAAGGGTTGGGATCCACACTGCCAGAGTGGGGGG<br>..((((.((...)).)))).	DR1	NM_001938	chr1:93584066-93600736	template	40	Intron_2.l	--	Yes	--	Yes	--	--	--	Yes	--	--	--
G4ST01093685459	chr1:93685460-93685488	-	GGGCACCTCGTGGGTGAGGGTTGGATGGG<br>.((...))	FNBP1L	NM_017737	chr1:93686276-93792806	template	-787	upstream	Yes	--	--	--	--	--	--	Yes	Yes	--	--
G4ST01093685459	chr1:93685460-93685488	-	GGGCACCTCGTGGGTGAGGGTTGGATGGG<br>.((...))	FNBP1L	NM_001164473	chr1:93686276-93792806	template	-787	upstream	Yes	--	--	--	--	--	--	Yes	Yes	--	--
G4ST01093685459	chr1:93685460-93685488	-	GGGCACCTCGTGGGTGAGGGTTGGATGGG<br>.((...))	FNBP1L	NM_001024948	chr1:93686276-93792806	template	-787	upstream	Yes	--	--	--	--	--	--	Yes	Yes	--	--
G4ST01093686105	chr1:93686106-93686149	-	GGGATCGGGGAGGACCTGGGCTAGCCTGGCCACCGCCGGCGGGG<br>...(((.(((....)))))).	FNBP1L	NM_017737	chr1:93686276-93792806	template	-126	upstream	Yes	--	--	--	--	--	--	Yes	Yes	Yes	--
G4ST01093686105	chr1:93686106-93686149	-	GGGATCGGGGAGGACCTGGGCTAGCCTGGCCACCGCCGGCGGGG<br>...(((.(((....)))))).	FNBP1L	NM_001164473	chr1:93686276-93792806	template	-126	upstream	Yes	--	--	--	--	--	--	Yes	Yes	Yes	--
G4ST01093686105	chr1:93686106-93686149	-	GGGATCGGGGAGGACCTGGGCTAGCCTGGCCACCGCCGGCGGGG<br>...(((.(((....)))))).	FNBP1L	NM_001024948	chr1:93686276-93792806	template	-126	upstream	Yes	--	--	--	--	--	--	Yes	Yes	Yes	--
G4ST01093706126	chr1:93706127-93706169	-	GGGAAGAGGGGATGACTAGGGCAAGCACAAAGGAATTGTGGGG<br>....(((((.....)))))	FNBP1L	NM_017737	chr1:93686276-93792806	template	43	Intron_1.f	--	Yes	--	Yes	--	--	--	--	--	--	--
G4ST01093706126	chr1:93706127-93706169	-	GGGAAGAGGGGATGACTAGGGCAAGCACAAAGGAATTGTGGGG<br>....(((((.....)))))	FNBP1L	NM_001164473	chr1:93686276-93792806	template	43	Intron_1.f	--	Yes	--	Yes	--	--	--	--	--	--	--
G4ST01093706126	chr1:93706127-93706169	-	GGGAAGAGGGGATGACTAGGGCAAGCACAAAGGAATTGTGGGG<br>....(((((.....)))))	FNBP1L	NM_001024948	chr1:93686276-93792806	template	43	Intron_1.f	--	Yes	--	Yes	--	--	--	--	--	--	--
G4ST01093739720	chr1:93739721-93739752	-	GGGCCTAATGGGAGGTGTCTGGGTCATGAGGG<br>((....))	FNBP1L	NM_017737	chr1:93686276-93792806	template	32	Intron_2	--	Yes	--	Yes	--	--	--	Yes	Yes	--	--
G4ST01093739720	chr1:93739721-93739752	-	GGGCCTAATGGGAGGTGTCTGGGTCATGAGGG<br>((....))	FNBP1L	NM_001164473	chr1:93686276-93792806	template	32	Intron_2	--	Yes	--	Yes	--	--	--	Yes	Yes	--	--
G4ST01093739720	chr1:93739721-93739752	-	GGGCCTAATGGGAGGTGTCTGGGTCATGAGGG<br>((....))	FNBP1L	NM_001024948	chr1:93686276-93792806	template	32	Intron_2	--	Yes	--	Yes	--	--	--	Yes	Yes	--	--
G4ST01093739729	chr1:93739730-93739760	-	GGGAGATAGGGCCTAATGGGAGGTGTCTGGG<br>((....))	FNBP1L	NM_017737	chr1:93686276-93792806	template	31	Intron_2	--	Yes	--	Yes	--	--	--	Yes	Yes	--	--
G4ST01093739729	chr1:93739730-93739760	-	GGGAGATAGGGCCTAATGGGAGGTGTCTGGG<br>((....))	FNBP1L	NM_001164473	chr1:93686276-93792806	template	31	Intron_2	--	Yes	--	Yes	--	--	--	Yes	Yes	--	--
G4ST01093739729	chr1:93739730-93739760	-	GGGAGATAGGGCCTAATGGGAGGTGTCTGGG<br>((....))	FNBP1L	NM_001024948	chr1:93686276-93792806	template	31	Intron_2	--	Yes	--	Yes	--	--	--	Yes	Yes	--	--
G4ST01093816778	chr1:93816779-93816815	-	GGGGAAGGCACAGGAGCTGTGGGGCCTCTGGGCAGGG<br>.....(((((...)))))	BCAR3	NM_003567	chr1:93799937-93919973	non-template	37	Intron_7	--	Yes	--	Yes	--	--	--	--	--	--	--
G4ST01093919895	chr1:93919896-93919948	-	GGGGCCGGGACCGGAGCCGGAGCTGCGGGGCGCACCGGCTAGACGCGCGCGGG<br>..((.(((....))).)) ((((..((......)).))))	BCAR3	NM_003567	chr1:93799937-93919973	non-template	53	Exon_1.f	--	Yes	Yes	--	--	Yes	--	Yes	Yes	Yes	Yes
G4ST01093919918	chr1:93919919-93919955	-	GGGGGACGGGGCCGGGACCGGAGCCGGAGCTGCGGGG<br>..((.(((....))).))	BCAR3	NM_003567	chr1:93799937-93919973	non-template	37	Exon_1.f	--	Yes	Yes	--	--	Yes	--	Yes	Yes	Yes	Yes
G4ST01094147462	chr1:94147463-94147502	+	GGGCCCGAGGGAGGCCGGACGGCGGCTGGGCGGCGGCGGG<br>..((((.....)))).	GCLM	NM_002061	chr1:94125178-94147600	template	40	Exon_1.f	--	Yes	Yes	--	--	Yes	--	--	Yes	Yes	--
G4ST01094237572	chr1:94237573-94237606	+	GGGCTGATGGGCCAGGTGGGAGCAAAGGCTCGGG<br>(((....))).	ABCA4	NM_000350	chr1:94230982-94359293	template	34	Intron_47	--	Yes	--	Yes	--	--	--	--	--	--	--
G4ST01094239925	chr1:94239926-94239957	-	GGGTCTCTTGGGTTGGGGAAATAGGTTCTGGG<br>(((.....))).	ABCA4	NM_000350	chr1:94230982-94359293	non-template	32	Intron_45	--	Yes	--	Yes	--	--	--	Yes	--	--	--
G4ST01094295528	chr1:94295529-94295558	+	GGGACAAGGGAGGAAGACCTGGGTAGGGGG<br>(((....)))	ABCA4	NM_000350	chr1:94230982-94359293	template	30	Intron_14	--	Yes	--	Yes	--	--	--	--	--	--	--
G4ST01094295535	chr1:94295536-94295577	+	GGGAGGAAGACCTGGGTAGGGGGTCTTGGAGCATCTCCAGGG<br>(((....))) .....(((((...)))))	ABCA4	NM_000350	chr1:94230982-94359293	template	42	Intron_14	--	Yes	--	Yes	--	--	--	--	--	--	--
G4ST01095118872	chr1:95118873-95118908	+	GGGGGCTAGGGAGAGGGGAAATTAGTGTTTAGTGGG<br>.((((....))))...	SLC44A3	NM_001114106	chr1:95058489-95133390	non-template	36	Intron_12	--	Yes	--	Yes	--	--	--	--	--	--	Yes
G4ST01095118872	chr1:95118873-95118908	+	GGGGGCTAGGGAGAGGGGAAATTAGTGTTTAGTGGG<br>.((((....))))...	SLC44A3	NM_152369	chr1:95058688-95133390	non-template	36	Intron_11	--	Yes	--	Yes	--	--	--	--	--	--	Yes
G4ST01095118880	chr1:95118881-95118915	+	GGGAGAGGGGAAATTAGTGTTTAGTGGGTATAGGG<br>.((((....))))...	SLC44A3	NM_001114106	chr1:95058489-95133390	non-template	35	Intron_12	--	Yes	--	Yes	--	--	--	--	--	--	Yes
G4ST01095118880	chr1:95118881-95118915	+	GGGAGAGGGGAAATTAGTGTTTAGTGGGTATAGGG<br>.((((....))))...	SLC44A3	NM_152369	chr1:95058688-95133390	non-template	35	Intron_11	--	Yes	--	Yes	--	--	--	--	--	--	Yes
G4ST01095132736	chr1:95132737-95132776	-	GGGGTACAGATAATTCTGTTTTTGGGAAAGGGAGATGGGG<br>..(((((....)))))....	SLC44A3	NM_001114106	chr1:95058489-95133390	template	40	Intron_14.l	--	Yes	--	Yes	--	--	--	--	--	--	--
G4ST01095132736	chr1:95132737-95132776	-	GGGGTACAGATAATTCTGTTTTTGGGAAAGGGAGATGGGG<br>..(((((....)))))....	SLC44A3	NM_152369	chr1:95058688-95133390	template	40	Intron_13.l	--	Yes	--	Yes	--	--	--	--	--	--	--
G4ST01095238061	chr1:95238062-95238086	-	GGGAGGGTTGTGGGAACCAGGTGGG<br>.((...))	ALG14	NM_144988	chr1:95220867-95311095	non-template	25	Intron_3.l	--	Yes	--	Yes	--	--	--	--	--	--	Yes
G4ST01095240504	chr1:95240505-95240534	+	GGGGTGGGGCAAGGGACTGAGGACCAGGGG<br>.(((.....)))	ALG14	NM_144988	chr1:95220867-95311095	template	30	Intron_3.l	--	Yes	--	Yes	--	--	--	--	--	--	--
G4ST01095303628	chr1:95303629-95303663	+	GGGAGGCCACTGGGTGGAGATGGGGATTGTTAGGG<br>((....))	ALG14	NM_144988	chr1:95220867-95311095	template	35	Intron_1.f	--	Yes	--	Yes	--	--	--	--	--	--	--
G4ST01095304346	chr1:95304347-95304385	-	GGGGGGAGGTGAGGGGGTGGAGGGAATTTGTGTTGGGGG<br>(((....)))..	ALG14	NM_144988	chr1:95220867-95311095	non-template	39	Intron_1.f	--	Yes	--	Yes	--	--	--	--	--	--	--

G4ST01095355694	chr1:95355695-95355731	-	GGGCGGCGGCTGCGCCGAGCCGGGAGAGGGCGGCGGG<br>(((((....)))))....	TMEM56	NM_001199679	chr1:95330661-95435749	template	37	Intron_1.f	--	Yes	--	Yes	--	--	--	Yes	Yes	Yes	--
G4ST01095355694	chr1:95355695-95355731	-	GGGCGGCGGCTGCGCCGAGCCGGGAGAGGGCGGCGGG<br>(((((....)))))....	TMEM56	NM_152487	chr1:95355482-95435749	template	37	Exon_1.f	--	Yes	Yes	--	--	Yes	--	Yes	Yes	Yes	--
G4ST01095355694	chr1:95355695-95355731	-	GGGCGGCGGCTGCGCCGAGCCGGGAGAGGGCGGCGGG<br>(((((....)))))....	TMEM56-RWDD3	NM_001199691	chr1:95356067-95483097	template	-335	upstream	Yes	--	--	--	--	--	--	Yes	Yes	Yes	--
G4ST01095357667	chr1:95357668-95357693	+	GGGAAAGGGGCTGGGTTTCAAAAGGG<br>((....))	TMEM56	NM_001199679	chr1:95330661-95435749	non-template	26	Intron_1.f	--	Yes	--	Yes	--	--	--	--	Yes	--	--
G4ST01095357667	chr1:95357668-95357693	+	GGGAAAGGGGCTGGGTTTCAAAAGGG<br>((....))	TMEM56	NM_152487	chr1:95355482-95435749	non-template	26	Intron_1.f	--	Yes	--	Yes	--	--	--	--	Yes	--	--
G4ST01095357667	chr1:95357668-95357693	+	GGGAAAGGGGCTGGGTTTCAAAAGGG<br>((....))	TMEM56-RWDD3	NM_001199691	chr1:95356067-95483097	non-template	26	Intron_1.f	--	Yes	--	Yes	--	--	--	--	Yes	--	--
G4ST01095472833	chr1:95472834-95472860	-	GGGGAGTGCTCTCGGGGGGGCCTAGGG<br>(((....)))	RWDD3	NM_001128142	chr1:95472299-95485369	template	27	Intron_1.f	--	Yes	--	Yes	--	--	--	Yes	Yes	Yes	--
G4ST01095472833	chr1:95472834-95472860	-	GGGGAGTGCTCTCGGGGGGGCCTAGGG<br>(((....)))	RWDD3	NM_001199682	chr1:95472299-95485369	template	27	Intron_1.f	--	Yes	--	Yes	--	--	--	Yes	Yes	Yes	--
G4ST01095472833	chr1:95472834-95472860	-	GGGGAGTGCTCTCGGGGGGGCCTAGGG<br>(((....)))	RWDD3	NM_015485	chr1:95472299-95485369	template	27	Intron_1.f	--	Yes	--	Yes	--	--	--	Yes	Yes	Yes	--
G4ST01095472833	chr1:95472834-95472860	-	GGGGAGTGCTCTCGGGGGGGCCTAGGG<br>(((....)))	RWDD3	NR_037643	chr1:95472299-95485369	template	27	Intron_1.f	--	Yes	--	Yes	--	--	--	Yes	Yes	Yes	--
G4ST01095472833	chr1:95472834-95472860	-	GGGGAGTGCTCTCGGGGGGGCCTAGGG<br>(((....)))	TMEM56-RWDD3	NM_001199691	chr1:95356067-95483097	template	27	Intron_7.l	--	Yes	--	Yes	--	--	--	Yes	Yes	Yes	--
G4ST01097705072	chr1:97705073-97705101	+	GGGGCCTGCAGTGGGGGGTGGAGGGTGGG<br>.(((.....)))	DPYD	NM_000110	chr1:97315888-98159203	template	29	Intron_13	--	Yes	--	Yes	--	--	--	--	--	--	--
G4ST01098159179	chr1:98159180-98159213	+	GGGCCGGCGGCGCGGGGGCGGAGCGGGCGCGGGG<br>(((......)))	DPYD	NM_001160301	chr1:97957902-98159203	template	24	upstream-Exon_1.f	Yes	Yes	Yes	--	--	Yes	--	Yes	Yes	Yes	--
G4ST01098159179	chr1:98159180-98159213	+	GGGCCGGCGGCGCGGGGGCGGAGCGGGCGCGGGG<br>(((......)))	DPYD	NM_000110	chr1:97315888-98159203	template	24	upstream-Exon_1.f	Yes	Yes	Yes	--	--	Yes	--	Yes	Yes	Yes	--
G4ST01098159331	chr1:98159332-98159363	+	GGGAGGGAGGGAGTAGAGCACCCGCTCTGGGG<br>...(((((....))))).	DPYD	NM_001160301	chr1:97957902-98159203	template	-128	upstream	Yes	--	--	--	--	--	--	Yes	Yes	Yes	--
G4ST01098159331	chr1:98159332-98159363	+	GGGAGGGAGGGAGTAGAGCACCCGCTCTGGGG<br>...(((((....))))).	DPYD	NM_000110	chr1:97315888-98159203	template	-128	upstream	Yes	--	--	--	--	--	--	Yes	Yes	Yes	--
G4ST01098956798	chr1:98956799-98956829	-	GGGACTTGGGAGAATAGGATATTGGGATGGG<br>..((((...))))	SNX7	NM_015976	chr1:98899824-98998644	template	31	Intron_7	--	Yes	--	Yes	--	--	--	--	--	--	--
G4ST01098956798	chr1:98956799-98956829	-	GGGACTTGGGAGAATAGGATATTGGGATGGG<br>..((((...))))	SNX7	NM_152238	chr1:98899824-98998644	template	31	Intron_6	--	Yes	--	Yes	--	--	--	--	--	--	--
G4ST01098956798	chr1:98956799-98956829	-	GGGACTTGGGAGAATAGGATATTGGGATGGG<br>..((((...))))	SNX7	NR_033716	chr1:98899824-98998644	template	31	Intron_8.l	--	Yes	--	Yes	--	--	--	--	--	--	--
G4ST01098956803	chr1:98956804-98956836	-	GGGTAGTGGGACTTGGGAGAATAGGATATTGGG<br>..((((...))))	SNX7	NM_015976	chr1:98899824-98998644	template	33	Intron_7	--	Yes	--	Yes	--	--	--	--	--	--	--
G4ST01098956803	chr1:98956804-98956836	-	GGGTAGTGGGACTTGGGAGAATAGGATATTGGG<br>..((((...))))	SNX7	NM_152238	chr1:98899824-98998644	template	33	Intron_6	--	Yes	--	Yes	--	--	--	--	--	--	--
G4ST01098956803	chr1:98956804-98956836	-	GGGTAGTGGGACTTGGGAGAATAGGATATTGGG<br>..((((...))))	SNX7	NR_033716	chr1:98899824-98998644	template	33	Intron_8.l	--	Yes	--	Yes	--	--	--	--	--	--	--
G4ST01099129707	chr1:99129708-99129743	+	GGGCACTCTGCTGAGTGGGAGGCTGGGACCTGGGGG<br>.((((....))))	LPPR5	NM_001037317	chr1:99128389-99243037	template	36	Exon_6.l	--	Yes	Yes	--	--	--	Yes	--	--	--	Yes
G4ST01099129707	chr1:99129708-99129743	+	GGGCACTCTGCTGAGTGGGAGGCTGGGACCTGGGGG<br>.((((....))))	LPPR5	NM_001010861	chr1:99128389-99243037	template	36	Exon_6.l	--	Yes	Yes	--	--	--	Yes	--	--	--	Yes
G4ST01099181309	chr1:99181310-99181343	+	GGGAATGTATGACATGAGGGTGGGTGCCCCAGGG<br>.((((...))))..	LPPR5	NM_001037317	chr1:99128389-99243037	template	34	Intron_3	--	Yes	--	Yes	--	--	--	--	--	--	--
G4ST01099181309	chr1:99181310-99181343	+	GGGAATGTATGACATGAGGGTGGGTGCCCCAGGG<br>.((((...))))..	LPPR5	NM_001010861	chr1:99128389-99243037	template	34	Intron_3	--	Yes	--	Yes	--	--	--	--	--	--	--
G4ST01099350449	chr1:99350450-99350479	-	GGGGCCTGTCGGCGGGTGGAGGGCTAGGGG<br>(((....)))	LOC100129620	NR_033940	chr1:99242420-99386996	template	30	Intron_1.f	--	Yes	--	Yes	--	--	--	--	--	--	Yes
G4ST01100226307	chr1:100226308-100226338	-	GGGGCTGGGGTTGAGGGTTGACTGCAAAGGG<br>(((....))).	SLC35A3	NM_012243	chr1:100208128-100261594	template	31	Intron_1.f	--	Yes	--	Yes	--	--	--	--	--	--	--
G4ST01100276167	chr1:100276168-100276194	+	GGGCGGGGCGGTATCACGCGGGAGGGG<br>(((......)))	HIAT1	NM_033055	chr1:100276377-100321517	non-template	-182	upstream	Yes	--	--	--	--	--	--	Yes	Yes	Yes	--
G4ST01100276171	chr1:100276172-100276199	+	GGGGCGGTATCACGCGGGAGGGGCGGGG<br>(((......)))	HIAT1	NM_033055	chr1:100276377-100321517	non-template	-177	upstream	Yes	--	--	--	--	--	--	Yes	Yes	Yes	--
G4ST01100367514	chr1:100367515-100367540	+	GGGCTCCATGAGAGGGGAGGGAAGGG<br>(((.....)))	SASS6	NM_194292	chr1:100321690-100371099	template	26	Intron_1.f	--	Yes	--	Yes	--	--	--	--	--	--	--
G4ST01100451513	chr1:100451514-100451559	-	GGGCGGCTGCGGGGGCGCGGCGGGGATGGCGCCGTAGCCGTGCGGG<br>.((((((......))))))..	DBT	NM_001918	chr1:100425066-100487997	non-template	46	Intron_7	--	Yes	--	Yes	--	--	--	--	--	--	--
G4ST01100504886	chr1:100504887-100504917	+	GGGCGATCCAGGGCGGGGCACTCTGCCTGGG<br>(((...)))..	RTCD1	NM_001130841	chr1:100504302-100530914	non-template	31	Intron_2	--	Yes	--	Yes	--	--	--	Yes	Yes	Yes	--
G4ST01100504886	chr1:100504887-100504917	+	GGGCGATCCAGGGCGGGGCACTCTGCCTGGG<br>(((...)))..	RTCD1	NM_003729	chr1:100504302-100530914	non-template	31	Intron_2	--	Yes	--	Yes	--	--	--	Yes	Yes	Yes	--
G4ST01100687616	chr1:100687617-100687649	-	GGGTAAGCTGCGTGGATTTGGGTATGGGGAGGG<br>.((.((....)).)).	CDC14A	NM_003672	chr1:100590611-100758421	template	33	Intron_7	--	Yes	--	Yes	--	--	--	--	Yes	--	--
G4ST01100687616	chr1:100687617-100687649	-	GGGTAAGCTGCGTGGATTTGGGTATGGGGAGGG<br>.((.((....)).)).	CDC14A	NM_033313	chr1:100590611-100723514	template	33	Intron_7	--	Yes	--	Yes	--	--	--	--	Yes	--	--
G4ST01100687616	chr1:100687617-100687649	-	GGGTAAGCTGCGTGGATTTGGGTATGGGGAGGG<br>.((.((....)).)).	CDC14A	NM_033312	chr1:100590611-100737609	template	33	Intron_7	--	Yes	--	Yes	--	--	--	--	Yes	--	--
G4ST01100777903	chr1:100777904-100777931	-	GGGTGCAGCGCGGGCGGCAGGGGCTGGG<br>.((...))	GPR88	NM_022049	chr1:100776316-100780171	template	28	Exon_2.l	--	Yes	Yes	--	Yes	--	--	Yes	Yes	--	--
G4ST01101244040	chr1:101244041-101244071	+	GGGAGGGACCCGGTAGTGGGGGAGGTGAGGG<br>..(((....)))	DPH5	NM_001077394	chr1:101227769-101263950	template	31	Intron_4	--	Yes	--	Yes	--	--	--	--	--	--	--
G4ST01101244040	chr1:101244041-101244071	+	GGGAGGGACCCGGTAGTGGGGGAGGTGAGGG<br>..(((....)))	DPH5	NM_001077395	chr1:101227769-101263950	template	31	Intron_4	--	Yes	--	Yes	--	--	--	--	--	--	--
G4ST01101244040	chr1:101244041-101244071	+	GGGAGGGACCCGGTAGTGGGGGAGGTGAGGG<br>..(((....)))	DPH5	NM_015958	chr1:101227769-101263950	template	31	Intron_4	--	Yes	--	Yes	--	--	--	--	--	--	--
G4ST01101244044	chr1:101244045-101244075	+	GGGACCCGGTAGTGGGGGAGGTGAGGGGGGG<br>..(((....)))	DPH5	NM_001077394	chr1:101227769-101263950	template	31	Intron_4	--	Yes	--	Yes	--	--	--	--	--	--	--
G4ST01101244044	chr1:101244045-101244075	+	GGGACCCGGTAGTGGGGGAGGTGAGGGGGGG<br>..(((....)))	DPH5	NM_001077395	chr1:101227769-101263950	template	31	Intron_4	--	Yes	--	Yes	--	--	--	--	--	--	--
G4ST01101244044	chr1:101244045-101244075	+	GGGACCCGGTAGTGGGGGAGGTGAGGGGGGG<br>..(((....)))	DPH5	NM_015958	chr1:101227769-101263950	template	31	Intron_4	--	Yes	--	Yes	--	--	--	--	--	--	--
G4ST01103840717	chr1:103840718-103840743	-	GGGGCGGGGCGCACGGCGGGGCGGGG<br>.(((...)))	RNPC3	NM_017619	chr1:103841166-103899382	template	-422	upstream	Yes	--	--	--	--	--	--	Yes	--	Yes	--
G4ST01103840722	chr1:103840723-103840755	-	GGGGCGCACGGCGGGGCGGGGCGCACGGCGGGG<br>.(((...))) .(((...)))	RNPC3	NM_017619	chr1:103841166-103899382	template	-410	upstream	Yes	--	--	--	--	--	--	Yes	--	Yes	--
G4ST01103840734	chr1:103840735-103840760	-	GGGGCGGGGCGCACGGCGGGGCGGGG<br>.(((...)))	RNPC3	NM_017619	chr1:103841166-103899382	template	-405	upstream	Yes	--	--	--	--	--	--	--	--	Yes	--
G4ST01103840739	chr1:103840740-103840772	-	GGGGCGCACGGCGGGGCGGGGCGCACGGCGGGG<br>.(((...))) .(((...)))	RNPC3	NM_017619	chr1:103841166-103899382	template	-393	upstream	Yes	--	--	--	--	--	--	--	--	Yes	--
G4ST01103840751	chr1:103840752-103840777	-	GGGGCGGGGCGCACGGCGGGGCGGGG<br>.(((...)))	RNPC3	NM_017619	chr1:103841166-103899382	template	-388	upstream	Yes	--	--	--	--	--	--	--	--	Yes	--
G4ST01103840756	chr1:103840757-103840789	-	GGGGCGCACGGCGGGGCGGGGCGCACGGCGGGG<br>.(((...))) .(((...)))	RNPC3	NM_017619	chr1:103841166-103899382	template	-376	upstream	Yes	--	--	--	--	--	--	--	--	Yes	--

G4ST01103840768	chr1:103840769-103840794	-	GGGGCGGGGCGCACGGCGGGGCGGGG	.(((...)))	RNPC3	NM_017619	chr1:103841166-103899382	template	-371	upstream	Yes	--	--	--	--	--	--	--	--	Yes	--
G4ST01103840773	chr1:103840774-103840806	-	GGGGCGCACGGCGGGGCGGGGCGCACGGCGGGG	.(((...))) .(((...)))	RNPC3	NM_017619	chr1:103841166-103899382	template	-359	upstream	Yes	--	--	--	--	--	--	--	--	Yes	--
G4ST01103840785	chr1:103840786-103840811	-	GGGGCGGGGCGCACGGCGGGGCGGGG	.(((...)))	RNPC3	NM_017619	chr1:103841166-103899382	template	-354	upstream	Yes	--	--	--	--	--	--	--	--	Yes	--
G4ST01103840790	chr1:103840791-103840823	-	GGGGCGCACGGCGGGGCGGGGCGCACGGCGGGG	.(((...))) .(((...)))	RNPC3	NM_017619	chr1:103841166-103899382	template	-342	upstream	Yes	--	--	--	--	--	--	--	--	Yes	--
G4ST01103840802	chr1:103840803-103840828	-	GGGGCGGGGCGCACGGCGGGGCGGGG	.(((...)))	RNPC3	NM_017619	chr1:103841166-103899382	template	-337	upstream	Yes	--	--	--	--	--	--	--	--	Yes	--
G4ST01103840807	chr1:103840808-103840840	-	GGGGCGCACGGCGGGGCGGGGCGCACGGCGGGG	.(((...))) .(((...)))	RNPC3	NM_017619	chr1:103841166-103899382	template	-325	upstream	Yes	--	--	--	--	--	--	--	Yes	Yes	--
G4ST01103840819	chr1:103840820-103840845	-	GGGGCGGGGCGCACGGCGGGGCGGGG	.(((...)))	RNPC3	NM_017619	chr1:103841166-103899382	template	-320	upstream	Yes	--	--	--	--	--	--	--	Yes	Yes	--
G4ST01103840824	chr1:103840825-103840857	-	GGGGCGCACGGCGGGGCGGGGCGCACGGCGGGG	.(((...))) .(((...)))	RNPC3	NM_017619	chr1:103841166-103899382	template	-308	upstream	Yes	--	--	--	--	--	--	--	Yes	Yes	--
G4ST01103840836	chr1:103840837-103840862	-	GGGGCGGGGCGCACGGCGGGGCGGGG	.(((...)))	RNPC3	NM_017619	chr1:103841166-103899382	template	-303	upstream	Yes	--	--	--	--	--	--	Yes	Yes	Yes	--
G4ST01103840841	chr1:103840842-103840874	-	GGGGCGCACGGCGGGGCGGGGCGCACGGCGGGG	.(((...))) .(((...)))	RNPC3	NM_017619	chr1:103841166-103899382	template	-291	upstream	Yes	--	--	--	--	--	--	Yes	Yes	Yes	--
G4ST01103840853	chr1:103840854-103840879	-	GGGGCGGGGCGCACGGCGGGGCGGGG	.(((...)))	RNPC3	NM_017619	chr1:103841166-103899382	template	-286	upstream	Yes	--	--	--	--	--	--	Yes	Yes	Yes	--
G4ST01103840858	chr1:103840859-103840883	-	GGGCGGGGCGGGGCGCACGGCGGGG	.(((...)))	RNPC3	NM_017619	chr1:103841166-103899382	template	-282	upstream	Yes	--	--	--	--	--	--	Yes	Yes	Yes	--
G4ST01105203983	chr1:105203984-105204020	+	GGGCCCTTGGGCAGCAGGTTTGGTGTGGGTGATGGGG	((.((....)).)).	MIR548H3	NR_031679	chr1:105130114-105476802	non-template	37	Intron_3.l	--	Yes	--	Yes	--	--	--	--	--	--	--
G4ST01107484559	chr1:107484560-107484590	+	GGGAGCCGCTGGTGGGGGCGGGGAGTCCGGG	..(((....)))	NTNG1	NM_001113228	chr1:107484152-107825998	non-template	31	Intron_1.f	--	Yes	--	Yes	--	--	--	--	Yes	--	--
G4ST01107484559	chr1:107484560-107484590	+	GGGAGCCGCTGGTGGGGGCGGGGAGTCCGGG	..(((....)))	NTNG1	NM_001113226	chr1:107484152-107825998	non-template	31	Intron_1.f	--	Yes	--	Yes	--	--	--	--	Yes	--	--
G4ST01107484559	chr1:107484560-107484590	+	GGGAGCCGCTGGTGGGGGCGGGGAGTCCGGG	..(((....)))	NTNG1	NM_014917	chr1:107484268-107825998	non-template	31	Intron_1.f	--	Yes	--	Yes	--	--	--	--	Yes	--	--
G4ST01107485637	chr1:107485638-107485668	-	GGGGGAGAGGGGCACGGGCGAGAGGCTCGGG	.(((...)))	NTNG1	NM_001113228	chr1:107484152-107825998	template	31	Intron_1.f	--	Yes	--	Yes	--	--	--	Yes	Yes	--	Yes
G4ST01107485637	chr1:107485638-107485668	-	GGGGGAGAGGGGCACGGGCGAGAGGCTCGGG	.(((...)))	NTNG1	NM_001113226	chr1:107484152-107825998	template	31	Intron_1.f	--	Yes	--	Yes	--	--	--	Yes	Yes	--	Yes
G4ST01107485637	chr1:107485638-107485668	-	GGGGGAGAGGGGCACGGGCGAGAGGCTCGGG	.(((...)))	NTNG1	NM_014917	chr1:107484268-107825998	template	31	Intron_1.f	--	Yes	--	Yes	--	--	--	Yes	Yes	--	Yes
G4ST01108034060	chr1:108034061-108034100	-	GGGGAATAGGGATATTCTGGTGTATATGGGTCTTTCTGGG	((((........))))	VAV3	NM_006113	chr1:107915305-108309068	non-template	40	Intron_17	--	Yes	--	Yes	--	--	--	Yes	Yes	--	--
G4ST01108034060	chr1:108034061-108034100	-	GGGGAATAGGGATATTCTGGTGTATATGGGTCTTTCTGGG	((((........))))	VAV3	NM_001079874	chr1:107915305-108032649	non-template	-1411	upstream	Yes	--	--	--	--	--	--	Yes	Yes	--	--
G4ST01108132462	chr1:108132463-108132494	-	GGGTGGAAGGGGCAGGGCAGCAGCTCCTGGGG	...(((...)))	VAV3	NM_006113	chr1:107915305-108309068	non-template	32	Intron_2	--	Yes	--	Yes	--	--	--	Yes	--	--	--
G4ST01108132477	chr1:108132478-108132505	-	GGGCAGAGTGAGGGTGGAAGGGGCAGGG	((...)).	VAV3	NM_006113	chr1:107915305-108309068	non-template	28	Intron_2	--	Yes	--	Yes	--	--	--	Yes	--	--	--
G4ST01108290151	chr1:108290152-108290180	+	GGGAGTTGAGGCTGGGGAGGGCAGCAGGG	(((....))).	VAV3	NM_006113	chr1:107915305-108309068	template	29	Intron_1.f	--	Yes	--	Yes	--	--	--	--	--	--	--
G4ST01108290164	chr1:108290165-108290200	+	GGGGAGGGCAGCAGGGTCCATACTCCAAGCATGGGG	..(((.((...)).)))	VAV3	NM_006113	chr1:107915305-108309068	template	36	Intron_1.f	--	Yes	--	Yes	--	--	--	--	--	--	--
G4ST01108290169	chr1:108290170-108290214	+	GGGCAGCAGGGTCCATACTCCAAGCATGGGGAAGCATTCTAAGGG	..(((.((...)).))) (((...)))...	VAV3	NM_006113	chr1:107915305-108309068	template	45	Intron_1.f	--	Yes	--	Yes	--	--	--	--	--	--	--
G4ST01108290177	chr1:108290178-108290225	+	GGGTCCATACTCCAAGCATGGGGAAGCATTCTAAGGGTTACAAAGGGG	..(((.((...)).))) (((...)))...	VAV3	NM_006113	chr1:107915305-108309068	template	48	Intron_1.f	--	Yes	--	Yes	--	--	--	--	--	--	--
G4ST01108310205	chr1:108310206-108310239	-	GGGGCAGTGTGCAGTGGGAGTAAAAGGGGTAGGG	(((...)))...	VAV3	NM_006113	chr1:107915305-108309068	non-template	-1137	upstream	Yes	--	--	--	--	--	--	--	Yes	Yes	--
G4ST01108544399	chr1:108544400-108544442	+	GGGGCGCCGTCGGGGTTGCGGCTGCGGCGCGCAGGGCGCAGGG	..((((((....)).))))	SLC25A24	NM_013386	chr1:108478970-108544497	template	43	Exon_1.f	--	Yes	Yes	--	--	Yes	--	Yes	Yes	Yes	--
G4ST01108572119	chr1:108572120-108572149	-	GGGAACCCCTGTCCTCAGGGGGAGGGAGGG	....((((....))))	NBPF4	NM_001143989	chr1:108567486-108588226	non-template	30	Intron_13	--	Yes	--	Yes	--	--	--	--	--	--	--
G4ST01108810484	chr1:108810485-108810514	+	GGGAACCCCTGTCCTCAGGGGGAGGGAGGG	....((((....))))	NBPF6	NM_001143987	chr1:108794427-108814783	non-template	30	Intron_14	--	Yes	--	Yes	--	--	--	--	--	--	--
G4ST01108810484	chr1:108810485-108810514	+	GGGAACCCCTGTCCTCAGGGGGAGGGAGGG	....((((....))))	NBPF6	NM_001143988	chr1:108794427-108814783	non-template	30	Intron_13	--	Yes	--	Yes	--	--	--	--	--	--	--
G4ST01108904125	chr1:108904126-108904152	-	GGGGCGGAAGCCGGAGGGAGGGCGGGG	.(((...)))..	FAM102B	NM_001010883	chr1:108904494-108983472	template	-341	upstream	Yes	--	--	--	--	--	--	Yes	Yes	Yes	Yes
G4ST01108905331	chr1:108905332-108905364	-	GGGGCTCATTTTAAAATGAGGGCAGGGCCTGGG	...((((....)))).	FAM102B	NM_001010883	chr1:108904494-108983472	template	33	Intron_1.f	--	Yes	--	Yes	--	--	--	Yes	Yes	Yes	--
G4ST01109091197	chr1:109091198-109091228	-	GGGAAAGCCGGGCCGGGCGGCTCACCCGGGG	(((.....)))	STXBP3	NM_007269	chr1:109090808-109153671	template	31	Intron_1.f	--	Yes	--	Yes	--	--	--	Yes	Yes	Yes	Yes
G4ST01109178854	chr1:109178855-109178886	-	GGGCAGAGATCTGAGGGACGGGACAGCTGGGG	(((....))).	AKNAD1	NM_152763	chr1:109160048-109201239	non-template	32	Intron_8	--	Yes	--	Yes	--	--	--	--	--	--	Yes
G4ST01109307685	chr1:109307686-109307723	+	GGGCGGGGCTAGTAGCGGGACCGGCCTCTGCGCGCGGG	(((...))) ..((((....)).)).	CLCC1	NM_015127	chr1:109273653-109307634	template	-51	upstream	Yes	--	--	--	--	--	--	Yes	Yes	Yes	--
G4ST01109307685	chr1:109307686-109307723	+	GGGCGGGGCTAGTAGCGGGACCGGCCTCTGCGCGCGGG	(((...))) ..((((....)).)).	CLCC1	NM_001048210	chr1:109273653-109307634	template	-51	upstream	Yes	--	--	--	--	--	--	Yes	Yes	Yes	--
G4ST01109332720	chr1:109332721-109332754	+	GGGCGCTGGGGTGGGGGGATCACTTGAGCCTGGG	.((.((....)).)).	WDR47	NM_001142550	chr1:109314360-109386373	template	34	Intron_9	--	Yes	--	Yes	--	--	--	--	--	--	--
G4ST01109332720	chr1:109332721-109332754	+	GGGCGCTGGGGTGGGGGGATCACTTGAGCCTGGG	.((.((....)).)).	WDR47	NM_001142551	chr1:109314360-109386373	template	34	Intron_9	--	Yes	--	Yes	--	--	--	--	--	--	--
G4ST01109332720	chr1:109332721-109332754	+	GGGCGCTGGGGTGGGGGGATCACTTGAGCCTGGG	.((.((....)).)).	WDR47	NM_014969	chr1:109314360-109386373	template	34	Intron_9	--	Yes	--	Yes	--	--	--	--	--	--	--
G4ST01109385512	chr1:109385513-109385543	-	GGGAGCGGCGGGGCTGGGAGCCCCGCTGGGG	(((...))).	WDR47	NM_001142550	chr1:109314360-109386373	non-template	31	Intron_1.f	--	Yes	--	Yes	--	--	--	Yes	Yes	Yes	--
G4ST01109385512	chr1:109385513-109385543	-	GGGAGCGGCGGGGCTGGGAGCCCCGCTGGGG	(((...))).	WDR47	NM_001142551	chr1:109314360-109386373	non-template	31	Intron_1.f	--	Yes	--	Yes	--	--	--	Yes	Yes	Yes	--
G4ST01109385512	chr1:109385513-109385543	-	GGGAGCGGCGGGGCTGGGAGCCCCGCTGGGG	(((...))).	WDR47	NM_014969	chr1:109314360-109386373	non-template	31	Intron_1.f	--	Yes	--	Yes	--	--	--	Yes	Yes	Yes	--
G4ST01109454266	chr1:109454267-109454303	-	GGGTCTGGGGAGAAAGGGTTATTCTTGAGTAAAAGGG	(((((....)))))..	C1orf194	NM_001122961	chr1:109450096-109458002	non-template	37	Intron_1.f	--	Yes	--	Yes	--	--	--	Yes	Yes	--	Yes
G4ST01109458419	chr1:109458420-109458456	+	GGGCTGGGACCGCCTTCCAGGTGACCCAGGGAACGGG	..(((((...)))))....	C1orf194	NM_001122961	chr1:109450096-109458002	template	-417	upstream	Yes	--	--	--	--	--	--	--	Yes	--	--
G4ST01109458419	chr1:109458420-109458456	+	GGGCTGGGACCGCCTTCCAGGTGACCCAGGGAACGGG	..(((((...)))))....	KIAA1324	NM_020775	chr1:109458108-109550926	non-template	37	Exon_1.f	--	Yes	Yes	--	Yes	--	--	--	Yes	--	--
G4ST01109482239	chr1:109482240-109482267	-	GGGGCCACGCTCTGGGGGCAGGGTTGGG	.(((.....)))	KIAA1324	NM_020775	chr1:109458108-109550926	template	28	Intron_1.f	--	Yes	--	Yes	--	--	--	Yes	--	--	--
G4ST01109593501	chr1:109593502-109593543	+	GGGCATCTGGCGAGGCCGGCCTGGGGAAATTGGGATGGAGGG	...(((((...)))))....	CELSR2	NM_001408	chr1:109594164-109619901	non-template	-620	upstream	Yes	--	--	--	--	--	--	Yes	Yes	Yes	--
G4ST01109593996	chr1:109593997-109594028	+	GGGTGACCCGGGAGCGGGTCTGGCTCAGGGGG	.(((...))).	CELSR2	NM_001408	chr1:109594164-109619901	non-template	-135	upstream	Yes	--	--	--	--	--	--	--	Yes	--	--

G4ST01109594005	chr1:109594006-109594035	+	GGGAGCGGGTCTGGCTCAGGGGGCAGTGGG<br>.(((...))).	CELSR2	NM_001408	chr1:109594164-109619901	non-template	-128	upstream	Yes	--	--	--	--	--	--	--	Yes	--	--
G4ST01109594011	chr1:109594012-109594043	+	GGGTCTGGCTCAGGGGGCAGTGGGAGCCCGGG<br>.(((...))).	CELSR2	NM_001408	chr1:109594164-109619901	non-template	-120	upstream	Yes	--	--	--	--	--	--	--	Yes	--	--
G4ST01109600695	chr1:109600696-109600729	-	GGGGCAGGAGCCCATGTTTCTGGGGTGGGGTGGG<br>.(((((((....)))))))	CELSR2	NM_001408	chr1:109594164-109619901	template	34	Intron_1.f	--	Yes	--	Yes	--	--	--	--	--	--	--
G4ST01109603222	chr1:109603223-109603250	+	GGGTGAGCCAAGGGAGGGGACTCATGGG<br>((...)).	CELSR2	NM_001408	chr1:109594164-109619901	non-template	28	Exon_2-Intron_2	--	Yes	Yes	Yes	Yes	--	--	Yes	Yes	--	--
G4ST01109607490	chr1:109607491-109607520	+	GGGCAGGGCTCTGCTGAGCGGGGCTGTGGG<br>(((....)))..	CELSR2	NM_001408	chr1:109594164-109619901	non-template	30	Intron_8	--	Yes	--	Yes	--	--	--	Yes	Yes	--	--
G4ST01109608000	chr1:109608001-109608025	+	GGGCAGGTCCTGGGCAGGGGTGGGG<br>.((...))	CELSR2	NM_001408	chr1:109594164-109619901	non-template	25	Intron_9	--	Yes	--	Yes	--	--	--	Yes	Yes	--	--
G4ST01109609251	chr1:109609252-109609292	+	GGGTCAGAAGGGCCACATAGGGCTCACCTAGGTTAGGTGGG<br>...((((.....))))	CELSR2	NM_001408	chr1:109594164-109619901	non-template	41	Intron_12	--	Yes	--	Yes	--	--	--	--	--	--	--
G4ST01109609260	chr1:109609261-109609304	+	GGGCCACATAGGGCTCACCTAGGTTAGGTGGGAGTGGCAGGGGG<br>...((((.....))))	CELSR2	NM_001408	chr1:109594164-109619901	non-template	44	Intron_12	--	Yes	--	Yes	--	--	--	--	--	--	--
G4ST01109613244	chr1:109613245-109613277	-	GGGCCTGACCACGGGGGGCGTCTCTGGGAGGGG<br>((((....))))	CELSR2	NM_001408	chr1:109594164-109619901	template	33	Intron_19-Exon_20	--	Yes	Yes	Yes	Yes	--	--	--	--	--	--
G4ST01109613249	chr1:109613250-109613286	-	GGGGCCTGCGGGCCTGACCACGGGGGGCGTCTCTGGG<br>((((....))))	CELSR2	NM_001408	chr1:109594164-109619901	template	37	Intron_19-Exon_20	--	Yes	Yes	Yes	Yes	--	--	--	--	--	--
G4ST01109613259	chr1:109613260-109613296	-	GGGCCTCTCCGGGGCCTGCGGGCCTGACCACGGGGGG<br>((((....))))	CELSR2	NM_001408	chr1:109594164-109619901	template	37	Exon_20	--	Yes	Yes	--	Yes	--	--	--	--	--	--
G4ST01109618031	chr1:109618032-109618063	+	GGGCAGAGTGCGGGAGGATGGGGAGCTGGGGG<br>((...)).	CELSR2	NM_001408	chr1:109594164-109619901	non-template	32	Intron_33.l	--	Yes	--	Yes	--	--	--	--	Yes	Yes	--
G4ST01109627567	chr1:109627568-109627603	-	GGGAGCGGGGCGGGGCGCGAGTTCCATACTCGTGGG<br>...(((((.....))))).	PSRC1	NM_032636	chr1:109623699-109627313	non-template	-254	upstream	Yes	--	--	--	--	--	--	Yes	Yes	Yes	--
G4ST01109627567	chr1:109627568-109627603	-	GGGAGCGGGGCGGGGCGCGAGTTCCATACTCGTGGG<br>...(((((.....))))).	PSRC1	NM_001032291	chr1:109623699-109627313	non-template	-254	upstream	Yes	--	--	--	--	--	--	Yes	Yes	Yes	--
G4ST01109627567	chr1:109627568-109627603	-	GGGAGCGGGGCGGGGCGCGAGTTCCATACTCGTGGG<br>...(((((.....))))).	PSRC1	NM_001005290	chr1:109623699-109627313	non-template	-254	upstream	Yes	--	--	--	--	--	--	Yes	Yes	Yes	--
G4ST01109627588	chr1:109627589-109627625	-	GGGTGCTCGGCGGCCCAGCAGAGGGAGCGGGGCGGGG<br>.(((.((...)).)))...	PSRC1	NM_032636	chr1:109623699-109627313	non-template	-275	upstream	Yes	--	--	--	--	--	--	Yes	Yes	Yes	--
G4ST01109627588	chr1:109627589-109627625	-	GGGTGCTCGGCGGCCCAGCAGAGGGAGCGGGGCGGGG<br>.(((.((...)).)))...	PSRC1	NM_001032291	chr1:109623699-109627313	non-template	-275	upstream	Yes	--	--	--	--	--	--	Yes	Yes	Yes	--
G4ST01109627588	chr1:109627589-109627625	-	GGGTGCTCGGCGGCCCAGCAGAGGGAGCGGGGCGGGG<br>.(((.((...)).)))...	PSRC1	NM_001005290	chr1:109623699-109627313	non-template	-275	upstream	Yes	--	--	--	--	--	--	Yes	Yes	Yes	--
G4ST01109741698	chr1:109741699-109741725	-	GGGCGCCGGGCCCGGGAGCCGCTCGGG<br>((...)).	SORT1	NM_002959	chr1:109653715-109742086	non-template	27	Intron_1.f	--	Yes	--	Yes	--	--	--	--	Yes	--	--
G4ST01109741837	chr1:109741838-109741875	-	GGGGTGAGCTGGGGGCTGCGGGCGGCCGCAGCCGGGGG<br>((((....)))).	SORT1	NM_002959	chr1:109653715-109742086	non-template	38	Exon_1.f	--	Yes	Yes	--	Yes	--	--	Yes	Yes	Yes	--
G4ST01109828581	chr1:109828582-109828620	-	GGGGGGTGAGGAAGGGGGCACAGAAGTGGGGTGCGTGGG<br>..(((....)))	ATXN7L2	NM_153340	chr1:109828084-109836943	template	39	Intron_1.f	--	Yes	--	Yes	--	--	--	Yes	Yes	Yes	--
G4ST01109831606	chr1:109831607-109831634	-	GGGAGGGGAGGGGAGGCGAGGCCTGGGG<br>.((((...)))).	ATXN7L2	NM_153340	chr1:109828084-109836943	template	28	Intron_4	--	Yes	--	Yes	--	--	--	--	--	--	--
G4ST01109843212	chr1:109843213-109843246	-	GGGGGCTTGGGAGGGGTATCTGAACCCAGAAGGG<br>...((((....)))).	CYB561D1	NM_001134404	chr1:109838224-109844586	template	34	Exon_4.l	--	Yes	Yes	--	--	--	Yes	--	--	--	--
G4ST01109843212	chr1:109843213-109843246	-	GGGGGCTTGGGAGGGGTATCTGAACCCAGAAGGG<br>...((((....)))).	CYB561D1	NM_001134402	chr1:109838181-109844586	template	34	Exon_2.l	--	Yes	Yes	--	--	--	Yes	--	--	--	--
G4ST01109843212	chr1:109843213-109843246	-	GGGGGCTTGGGAGGGGTATCTGAACCCAGAAGGG<br>...((((....)))).	CYB561D1	NM_001134403	chr1:109838224-109844586	template	34	Exon_4.l	--	Yes	Yes	--	--	--	Yes	--	--	--	--
G4ST01109843212	chr1:109843213-109843246	-	GGGGGCTTGGGAGGGGTATCTGAACCCAGAAGGG<br>...((((....)))).	CYB561D1	NM_182580	chr1:109838224-109844586	template	34	Exon_3.l	--	Yes	Yes	--	--	--	Yes	--	--	--	--
G4ST01109843212	chr1:109843213-109843246	-	GGGGGCTTGGGAGGGGTATCTGAACCCAGAAGGG<br>...((((....)))).	CYB561D1	NM_001134400	chr1:109838224-109844586	template	34	Exon_3.l	--	Yes	Yes	--	--	--	Yes	--	--	--	--
G4ST01109969724	chr1:109969725-109969761	-	GGGCACTCTCCTACAGAGTCTGGGGATGGGGTGAGGG<br>.(((((.....)))))...	AMPD2	NM_203404	chr1:109969572-109976200	template	37	Exon_1.f	--	Yes	Yes	--	--	Yes	--	Yes	--	--	--
G4ST01109969724	chr1:109969725-109969761	-	GGGCACTCTCCTACAGAGTCTGGGGATGGGGTGAGGG<br>.(((((.....)))))...	AMPD2	NM_004037	chr1:109964799-109976200	template	37	Intron_2	--	Yes	--	Yes	--	--	--	Yes	--	--	--
G4ST01109969724	chr1:109969725-109969761	-	GGGCACTCTCCTACAGAGTCTGGGGATGGGGTGAGGG<br>.(((((.....)))))...	AMPD2	NM_139156	chr1:109963982-109976200	template	37	Intron_2	--	Yes	--	Yes	--	--	--	Yes	--	--	--
G4ST01109970943	chr1:109970944-109970976	-	GGGTGGTGGGGCAGAAGCTCTGCAGGGACAGGG<br>(((((...))))).	AMPD2	NM_203404	chr1:109969572-109976200	template	33	Exon_4	--	Yes	Yes	--	Yes	--	--	--	--	--	--
G4ST01109970943	chr1:109970944-109970976	-	GGGTGGTGGGGCAGAAGCTCTGCAGGGACAGGG<br>(((((...))))).	AMPD2	NM_004037	chr1:109964799-109976200	template	33	Exon_6	--	Yes	Yes	--	Yes	--	--	--	--	--	--
G4ST01109970943	chr1:109970944-109970976	-	GGGTGGTGGGGCAGAAGCTCTGCAGGGACAGGG<br>(((((...))))).	AMPD2	NM_139156	chr1:109963982-109976200	template	33	Exon_6	--	Yes	Yes	--	Yes	--	--	--	--	--	--
G4ST01109971387	chr1:109971388-109971422	+	GGGGACCTGGGCTTGGGTCTGCGCATGGTGCGGGG<br>....((((...))))	AMPD2	NM_203404	chr1:109969572-109976200	non-template	35	Exon_5	--	Yes	Yes	--	Yes	--	--	--	--	--	--
G4ST01109971387	chr1:109971388-109971422	+	GGGGACCTGGGCTTGGGTCTGCGCATGGTGCGGGG<br>....((((...))))	AMPD2	NM_004037	chr1:109964799-109976200	non-template	35	Exon_7	--	Yes	Yes	--	Yes	--	--	--	--	--	--
G4ST01109971387	chr1:109971388-109971422	+	GGGGACCTGGGCTTGGGTCTGCGCATGGTGCGGGG<br>....((((...))))	AMPD2	NM_139156	chr1:109963982-109976200	non-template	35	Exon_7	--	Yes	Yes	--	Yes	--	--	--	--	--	--
G4ST01109974576	chr1:109974577-109974610	+	GGGCGGGCGTCCCAGGACGCTGGGGTCTCCCGGG<br>((((....))))...	AMPD2	NM_203404	chr1:109969572-109976200	non-template	34	Intron_14	--	Yes	--	Yes	--	--	--	Yes	--	--	Yes
G4ST01109974576	chr1:109974577-109974610	+	GGGCGGGCGTCCCAGGACGCTGGGGTCTCCCGGG<br>((((....))))...	AMPD2	NM_004037	chr1:109964799-109976200	non-template	34	Intron_16	--	Yes	--	Yes	--	--	--	Yes	--	--	Yes
G4ST01109974576	chr1:109974577-109974610	+	GGGCGGGCGTCCCAGGACGCTGGGGTCTCCCGGG<br>((((....))))...	AMPD2	NM_139156	chr1:109963982-109976200	non-template	34	Intron_16	--	Yes	--	Yes	--	--	--	Yes	--	--	Yes
G4ST01109974580	chr1:109974581-109974615	+	GGGCGTCCCAGGACGCTGGGGTCTCCCGGGTTGGG<br>((((....))))...	AMPD2	NM_203404	chr1:109969572-109976200	non-template	35	Intron_14	--	Yes	--	Yes	--	--	--	Yes	--	--	Yes
G4ST01109974580	chr1:109974581-109974615	+	GGGCGTCCCAGGACGCTGGGGTCTCCCGGGTTGGG<br>((((....))))...	AMPD2	NM_004037	chr1:109964799-109976200	non-template	35	Intron_16	--	Yes	--	Yes	--	--	--	Yes	--	--	Yes
G4ST01109974580	chr1:109974581-109974615	+	GGGCGTCCCAGGACGCTGGGGTCTCCCGGGTTGGG<br>((((....))))...	AMPD2	NM_139156	chr1:109963982-109976200	non-template	35	Intron_16	--	Yes	--	Yes	--	--	--	Yes	--	--	Yes
G4ST01110099383	chr1:110099384-110099430	+	GGGTAATGGGGAATTATTGTTTAATGGGTACAGAATTTCTGTTTGGG<br>..((((.....)))) .(((((...))))))..	EPS8L3	NM_139053	chr1:110094225-110108167	template	47	Intron_12	--	Yes	--	Yes	--	--	--	--	--	--	--
G4ST01110099383	chr1:110099384-110099430	+	GGGTAATGGGGAATTATTGTTTAATGGGTACAGAATTTCTGTTTGGG<br>..((((.....)))) .(((((...))))))..	EPS8L3	NM_024526	chr1:110094225-110108167	template	47	Intron_12	--	Yes	--	Yes	--	--	--	--	--	--	--
G4ST01110099383	chr1:110099384-110099430	+	GGGTAATGGGGAATTATTGTTTAATGGGTACAGAATTTCTGTTTGGG<br>..((((.....)))) .(((((...))))))..	EPS8L3	NM_133181	chr1:110094225-110108167	template	47	Intron_12	--	Yes	--	Yes	--	--	--	--	--	--	--
G4ST01110102531	chr1:110102532-110102572	+	GGGCACTGGTGTGGCGTGGCAGGGGCCTTGGGGATGGTGGG<br>..(((.((.....)).)))	EPS8L3	NM_139053	chr1:110094225-110108167	template	41	Exon_8	--	Yes	Yes	--	Yes	--	--	--	--	--	--
G4ST01110102531	chr1:110102532-110102572	+	GGGCACTGGTGTGGCGTGGCAGGGGCCTTGGGGATGGTGGG<br>..(((.((.....)).)))	EPS8L3	NM_024526	chr1:110094225-110108167	template	41	Exon_8	--	Yes	Yes	--	Yes	--	--	--	--	--	--
G4ST01110102531	chr1:110102532-110102572	+	GGGCACTGGTGTGGCGTGGCAGGGGCCTTGGGGATGGTGGG<br>..(((.((.....)).)))	EPS8L3	NM_133181	chr1:110094225-110108167	template	41	Exon_8	--	Yes	Yes	--	Yes	--	--	--	--	--	--
G4ST01110104144	chr1:110104145-110104179	+	GGGACGGTGTCGAAGCACTGGGGCCAGGGATAGGG<br>.((((((....))))))	EPS8L3	NM_139053	chr1:110094225-110108167	template	35	Intron_3	--	Yes	--	Yes	--	--	--	Yes	Yes	--	--
G4ST01110104144	chr1:110104145-110104179	+	GGGACGGTGTCGAAGCACTGGGGCCAGGGATAGGG<br>.((((((....))))))	EPS8L3	NM_024526	chr1:110094225-110108167	template	35	Intron_3	--	Yes	--	Yes	--	--	--	Yes	Yes	--	--

G4ST01110104144	chr1:110104145-110104179	+	GGGACGGTGTCGAAGCACTGGGGCCAGGGATAGGG<br>.((((((....))))))	EPS8L3	NM_133181	chr1:110094225-110108167	template	35	Intron_3	--	Yes	--	Yes	--	--	--	Yes	Yes	--	--
G4ST01110106136	chr1:110106137-110106168	+	GGGAGGGTCAGGGAGGCCACAGCATGGCTGGG<br>..((((.....)))).	EPS8L3	NM_139053	chr1:110094225-110108167	template	32	Intron_1.f	--	Yes	--	Yes	--	--	--	--	Yes	--	Yes
G4ST01110106136	chr1:110106137-110106168	+	GGGAGGGTCAGGGAGGCCACAGCATGGCTGGG<br>..((((.....)))).	EPS8L3	NM_024526	chr1:110094225-110108167	template	32	Intron_1.f	--	Yes	--	Yes	--	--	--	--	Yes	--	Yes
G4ST01110106136	chr1:110106137-110106168	+	GGGAGGGTCAGGGAGGCCACAGCATGGCTGGG<br>..((((.....)))).	EPS8L3	NM_133181	chr1:110094225-110108167	template	32	Intron_1.f	--	Yes	--	Yes	--	--	--	--	Yes	--	Yes
G4ST01110106140	chr1:110106141-110106176	+	GGGTCAGGGAGGCCACAGCATGGCTGGGCCTCAGGG<br>..((((.....)))).	EPS8L3	NM_139053	chr1:110094225-110108167	template	36	Intron_1.f	--	Yes	--	Yes	--	--	--	--	Yes	--	Yes
G4ST01110106140	chr1:110106141-110106176	+	GGGTCAGGGAGGCCACAGCATGGCTGGGCCTCAGGG<br>..((((.....)))).	EPS8L3	NM_024526	chr1:110094225-110108167	template	36	Intron_1.f	--	Yes	--	Yes	--	--	--	--	Yes	--	Yes
G4ST01110106140	chr1:110106141-110106176	+	GGGTCAGGGAGGCCACAGCATGGCTGGGCCTCAGGG<br>..((((.....)))).	EPS8L3	NM_133181	chr1:110094225-110108167	template	36	Intron_1.f	--	Yes	--	Yes	--	--	--	--	Yes	--	Yes
G4ST01110106583	chr1:110106584-110106623	-	GGGTGGGGTTGGATGGTCCAGGAGGGCTTCACCAAGAGGG<br>..((((...))))... (((....))).	EPS8L3	NM_139053	chr1:110094225-110108167	non-template	40	Intron_1.f	--	Yes	--	Yes	--	--	--	--	--	--	Yes
G4ST01110106583	chr1:110106584-110106623	-	GGGTGGGGTTGGATGGTCCAGGAGGGCTTCACCAAGAGGG<br>..((((...))))... (((....))).	EPS8L3	NM_024526	chr1:110094225-110108167	non-template	40	Intron_1.f	--	Yes	--	Yes	--	--	--	--	--	--	Yes
G4ST01110106583	chr1:110106584-110106623	-	GGGTGGGGTTGGATGGTCCAGGAGGGCTTCACCAAGAGGG<br>..((((...))))... (((....))).	EPS8L3	NM_133181	chr1:110094225-110108167	non-template	40	Intron_1.f	--	Yes	--	Yes	--	--	--	--	--	--	Yes
G4ST01110106597	chr1:110106598-110106628	-	GGGCAGGGTGGGGTTGGATGGTCCAGGAGGG<br>..((((...))))...	EPS8L3	NM_139053	chr1:110094225-110108167	non-template	31	Intron_1.f	--	Yes	--	Yes	--	--	--	--	--	--	Yes
G4ST01110106597	chr1:110106598-110106628	-	GGGCAGGGTGGGGTTGGATGGTCCAGGAGGG<br>..((((...))))...	EPS8L3	NM_024526	chr1:110094225-110108167	non-template	31	Intron_1.f	--	Yes	--	Yes	--	--	--	--	--	--	Yes
G4ST01110106597	chr1:110106598-110106628	-	GGGCAGGGTGGGGTTGGATGGTCCAGGAGGG<br>..((((...))))...	EPS8L3	NM_133181	chr1:110094225-110108167	non-template	31	Intron_1.f	--	Yes	--	Yes	--	--	--	--	--	--	Yes
G4ST01110254799	chr1:110254800-110254830	+	GGGGCTGGCCGGCCGGCGGGTGGGGGAGGGG<br>(((((....)))))	CSF1	NM_000757	chr1:110254756-110273878	non-template	31	Exon_1.f	--	Yes	Yes	--	--	Yes	--	Yes	Yes	Yes	Yes
G4ST01110254799	chr1:110254800-110254830	+	GGGGCTGGCCGGCCGGCGGGTGGGGGAGGGG<br>(((((....)))))	CSF1	NM_172211	chr1:110254756-110273878	non-template	31	Exon_1.f	--	Yes	Yes	--	--	Yes	--	Yes	Yes	Yes	Yes
G4ST01110254799	chr1:110254800-110254830	+	GGGGCTGGCCGGCCGGCGGGTGGGGGAGGGG<br>(((((....)))))	CSF1	NM_172210	chr1:110254756-110269347	non-template	31	Exon_1.f	--	Yes	Yes	--	--	Yes	--	Yes	Yes	Yes	Yes
G4ST01110254799	chr1:110254800-110254830	+	GGGGCTGGCCGGCCGGCGGGTGGGGGAGGGG<br>(((((....)))))	CSF1	NM_172212	chr1:110254756-110270888	non-template	31	Exon_1.f	--	Yes	Yes	--	--	Yes	--	Yes	Yes	Yes	Yes
G4ST01110268536	chr1:110268537-110268565	+	GGGAAGGGACTGGAGCAGAAGGGAGCGGG<br>.(((...)))..	CSF1	NM_000757	chr1:110254756-110273878	non-template	29	Intron_6	--	Yes	--	Yes	--	--	--	Yes	--	--	--
G4ST01110268536	chr1:110268537-110268565	+	GGGAAGGGACTGGAGCAGAAGGGAGCGGG<br>.(((...)))..	CSF1	NM_172211	chr1:110254756-110273878	non-template	29	Intron_6	--	Yes	--	Yes	--	--	--	Yes	--	--	--
G4ST01110268536	chr1:110268537-110268565	+	GGGAAGGGACTGGAGCAGAAGGGAGCGGG<br>.(((...)))..	CSF1	NM_172210	chr1:110254756-110269347	non-template	29	Intron_7	--	Yes	--	Yes	--	--	--	Yes	--	--	--
G4ST01110268536	chr1:110268537-110268565	+	GGGAAGGGACTGGAGCAGAAGGGAGCGGG<br>.(((...)))..	CSF1	NM_172212	chr1:110254756-110270888	non-template	29	Intron_6	--	Yes	--	Yes	--	--	--	Yes	--	--	--
G4ST01110268967	chr1:110268968-110269011	+	GGGCAGGTGAGAGCTTGAGGTGGGGCTCTGGGAGGCACTGGGGG<br>(((((....)))))....	CSF1	NM_000757	chr1:110254756-110273878	non-template	44	Exon_7-Intron_7	--	Yes	Yes	Yes	Yes	--	--	--	--	--	--
G4ST01110268967	chr1:110268968-110269011	+	GGGCAGGTGAGAGCTTGAGGTGGGGCTCTGGGAGGCACTGGGGG<br>(((((....)))))....	CSF1	NM_172211	chr1:110254756-110273878	non-template	44	Exon_7-Intron_7	--	Yes	Yes	Yes	Yes	--	--	--	--	--	--
G4ST01110268967	chr1:110268968-110269011	+	GGGCAGGTGAGAGCTTGAGGTGGGGCTCTGGGAGGCACTGGGGG<br>(((((....)))))....	CSF1	NM_172210	chr1:110254756-110269347	non-template	44	Exon_8-Intron_8.l	--	Yes	Yes	Yes	Yes	--	--	--	--	--	--
G4ST01110268967	chr1:110268968-110269011	+	GGGCAGGTGAGAGCTTGAGGTGGGGCTCTGGGAGGCACTGGGGG<br>(((((....)))))....	CSF1	NM_172212	chr1:110254756-110270888	non-template	44	Exon_7-Intron_7	--	Yes	Yes	Yes	Yes	--	--	--	--	--	--
G4ST01110272709	chr1:110272710-110272733	-	GGGAGAGGTCTGGGCAGGGCTGGG<br>((....))	CSF1	NM_000757	chr1:110254756-110273878	template	24	Intron_8.l	--	Yes	--	Yes	--	--	--	--	--	Yes	Yes
G4ST01110272709	chr1:110272710-110272733	-	GGGAGAGGTCTGGGCAGGGCTGGG<br>((....))	CSF1	NM_172211	chr1:110254756-110273878	template	24	Intron_8.l	--	Yes	--	Yes	--	--	--	--	--	Yes	Yes
G4ST01110272714	chr1:110272715-110272740	-	GGGGCCCGGGAGAGGTCTGGGCAGGG<br>((....))	CSF1	NM_000757	chr1:110254756-110273878	template	26	Intron_8.l	--	Yes	--	Yes	--	--	--	--	--	Yes	Yes
G4ST01110272714	chr1:110272715-110272740	-	GGGGCCCGGGAGAGGTCTGGGCAGGG<br>((....))	CSF1	NM_172211	chr1:110254756-110273878	template	26	Intron_8.l	--	Yes	--	Yes	--	--	--	--	--	Yes	Yes
G4ST01110329090	chr1:110329091-110329126	+	GGGTGGGCGATCGCGTGTCGGAGGGCGCCGCGCGGG<br>((((.....)))).. .((...))	AHCYL1	NM_006621	chr1:110328831-110367887	non-template	36	Exon_1.f	--	Yes	Yes	--	--	Yes	--	Yes	Yes	Yes	Yes
G4ST01110329094	chr1:110329095-110329134	+	GGGCGATCGCGTGTCGGAGGGCGCCGCGCGGGCAGGCGGG<br>((((.....)))).. .((...))	AHCYL1	NM_006621	chr1:110328831-110367887	non-template	40	Exon_1.f	--	Yes	Yes	--	--	Yes	--	Yes	Yes	Yes	Yes
G4ST01110329112	chr1:110329113-110329138	+	GGGCGCCGCGCGGGCAGGCGGGCGGG<br>.((...))	AHCYL1	NM_006621	chr1:110328831-110367887	non-template	26	Exon_1.f	--	Yes	Yes	--	--	Yes	--	Yes	Yes	Yes	Yes
G4ST01110329400	chr1:110329401-110329445	+	GGGACTTCTGGGGGTGACTGGGGACGGCGGAGGTGATGTAGGGGG<br>.(((.((....)).)))...	AHCYL1	NM_006621	chr1:110328831-110367887	non-template	45	Intron_1.f	--	Yes	--	Yes	--	--	--	Yes	Yes	Yes	--
G4ST01110414553	chr1:110414554-110414592	+	GGGCGGCTTGGCCGGCTCTGGGAGGTAGGGCTCCAGGGG<br>((((...))))....	ALX3	NM_006492	chr1:110404520-110414845	template	39	Exon_1.f	--	Yes	Yes	--	Yes	--	--	--	--	--	--
G4ST01110414866	chr1:110414867-110414904	+	GGGAGCGGCGCGCGCCGCGGGGGGCGGGGGGCGGGGGG<br>.(((((....)))))...	ALX3	NM_006492	chr1:110404520-110414845	template	-21	upstream	Yes	--	--	--	--	--	--	--	--	--	Yes
G4ST01110536414	chr1:110536415-110536446	+	GGGCAGGGGTGGGGGCGCAGGTGTGCATGGGG<br>.((((....))))...	SLC6A17	NM_001010898	chr1:110494655-110546346	non-template	32	Intron_7	--	Yes	--	Yes	--	--	--	--	--	--	Yes
G4ST01110539678	chr1:110539679-110539705	-	GGGGGCAAAGGTGCAGGGTGGGCAGGG<br>.(((....))).	SLC6A17	NM_001010898	chr1:110494655-110546346	template	27	Intron_9	--	Yes	--	Yes	--	--	--	--	--	--	Yes
G4ST01110554376	chr1:110554377-110554406	-	GGGGTGGGCGGGGCGCGGCGCAGCGCGGGG<br>.((((......))))	KCNC4	NR_036437	chr1:110554859-110578197	template	-452	upstream	Yes	--	--	--	--	--	--	Yes	Yes	Yes	Yes
G4ST01110554376	chr1:110554377-110554406	-	GGGGTGGGCGGGGCGCGGCGCAGCGCGGGG<br>.((((......))))	KCNC4	NM_001039574	chr1:110554859-110578197	template	-452	upstream	Yes	--	--	--	--	--	--	Yes	Yes	Yes	Yes
G4ST01110554376	chr1:110554377-110554406	-	GGGGTGGGCGGGGCGCGGCGCAGCGCGGGG<br>.((((......))))	KCNC4	NM_004978	chr1:110554859-110578197	template	-452	upstream	Yes	--	--	--	--	--	--	Yes	Yes	Yes	Yes
G4ST01110554503	chr1:110554504-110554541	-	GGGGGCGCCTCGGCGCCGGTGGGCTGGGGCCCTAGGGG<br>(((((....)))))...	KCNC4	NR_036437	chr1:110554859-110578197	template	-317	upstream	Yes	--	--	--	--	--	--	--	Yes	Yes	--
G4ST01110554503	chr1:110554504-110554541	-	GGGGGCGCCTCGGCGCCGGTGGGCTGGGGCCCTAGGGG<br>(((((....)))))...	KCNC4	NM_001039574	chr1:110554859-110578197	template	-317	upstream	Yes	--	--	--	--	--	--	--	Yes	Yes	--
G4ST01110554503	chr1:110554504-110554541	-	GGGGGCGCCTCGGCGCCGGTGGGCTGGGGCCCTAGGGG<br>(((((....)))))...	KCNC4	NM_004978	chr1:110554859-110578197	template	-317	upstream	Yes	--	--	--	--	--	--	--	Yes	Yes	--
G4ST01110554512	chr1:110554513-110554546	-	GGGGAGGGGGCGCCTCGGCGCCGGTGGGCTGGGG<br>(((((....)))))...	KCNC4	NR_036437	chr1:110554859-110578197	template	-312	upstream	Yes	--	--	--	--	--	--	--	Yes	Yes	--
G4ST01110554512	chr1:110554513-110554546	-	GGGGAGGGGGCGCCTCGGCGCCGGTGGGCTGGGG<br>(((((....)))))...	KCNC4	NM_001039574	chr1:110554859-110578197	template	-312	upstream	Yes	--	--	--	--	--	--	--	Yes	Yes	--
G4ST01110554512	chr1:110554513-110554546	-	GGGGAGGGGGCGCCTCGGCGCCGGTGGGCTGGGG<br>(((((....)))))...	KCNC4	NM_004978	chr1:110554859-110578197	template	-312	upstream	Yes	--	--	--	--	--	--	--	Yes	Yes	--
G4ST01110554834	chr1:110554835-110554870	-	GGGCGCAGCGCTGCGGGGGCCGCGCTGGGGCGGGGG<br>((((....)))).	KCNC4	NR_036437	chr1:110554859-110578197	template	12	upstream-Exon_1.f	Yes	Yes	Yes	--	--	--	--	Yes	Yes	Yes	--
G4ST01110554834	chr1:110554835-110554870	-	GGGCGCAGCGCTGCGGGGGCCGCGCTGGGGCGGGGG<br>((((....)))).	KCNC4	NM_001039574	chr1:110554859-110578197	template	12	upstream-Exon_1.f	Yes	Yes	Yes	--	--	Yes	--	Yes	Yes	Yes	--
G4ST01110554834	chr1:110554835-110554870	-	GGGCGCAGCGCTGCGGGGGCCGCGCTGGGGCGGGGG<br>((((....)))).	KCNC4	NM_004978	chr1:110554859-110578197	template	12	upstream-Exon_1.f	Yes	Yes	Yes	--	--	Yes	--	Yes	Yes	Yes	--
G4ST01110559171	chr1:110559172-110559207	+	GGGCCCGCAGCACTGTGGGGTGGTGGGCGAAGAGGG<br>..((((....))))	KCNC4	NR_036437	chr1:110554859-110578197	non-template	36	Intron_1.f	--	Yes	--	Yes	--	--	--	Yes	Yes	--	--

G4ST01110559171	chr1:110559172-110559207	+	GGGCCCGCAGCACTGTGGGGTGGTGGGCGAAGAGGG<br>..((((....))))	KCNC4	NM_001039574	chr1:110554859-110578197	non-template	36	Intron_1.f	--	Yes	--	Yes	--	--	--	Yes	Yes	--	--
G4ST01110559171	chr1:110559172-110559207	+	GGGCCCGCAGCACTGTGGGGTGGTGGGCGAAGAGGG<br>..((((....))))	KCNC4	NM_004978	chr1:110554859-110578197	non-template	36	Intron_1.f	--	Yes	--	Yes	--	--	--	Yes	Yes	--	--
G4ST01110560561	chr1:110560562-110560593	-	GGGAGGATGCCCTGCGGGCAGGGCCTGCAGGG<br>(((....)))..	KCNC4	NR_036437	chr1:110554859-110578197	template	32	Intron_1.f	--	Yes	--	Yes	--	--	--	--	--	--	--
G4ST01110560561	chr1:110560562-110560593	-	GGGAGGATGCCCTGCGGGCAGGGCCTGCAGGG<br>(((....)))..	KCNC4	NM_001039574	chr1:110554859-110578197	template	32	Intron_1.f	--	Yes	--	Yes	--	--	--	--	--	--	--
G4ST01110560561	chr1:110560562-110560593	-	GGGAGGATGCCCTGCGGGCAGGGCCTGCAGGG<br>(((....)))..	KCNC4	NM_004978	chr1:110554859-110578197	template	32	Intron_1.f	--	Yes	--	Yes	--	--	--	--	--	--	--
G4ST01110565727	chr1:110565728-110565753	-	GGGAGTCTGCACTGGGATGGGGAGGG<br>(((....)))	KCNC4	NR_036437	chr1:110554859-110578197	template	26	Intron_1.f	--	Yes	--	Yes	--	--	--	--	--	--	--
G4ST01110565727	chr1:110565728-110565753	-	GGGAGTCTGCACTGGGATGGGGAGGG<br>(((....)))	KCNC4	NM_001039574	chr1:110554859-110578197	template	26	Intron_1.f	--	Yes	--	Yes	--	--	--	--	--	--	--
G4ST01110565727	chr1:110565728-110565753	-	GGGAGTCTGCACTGGGATGGGGAGGG<br>(((....)))	KCNC4	NM_004978	chr1:110554859-110578197	template	26	Intron_1.f	--	Yes	--	Yes	--	--	--	--	--	--	--
G4ST01110574125	chr1:110574126-110574155	+	GGGCAGCGTCGGGGCTGGGCAGAGGCTGGG<br>.((...))	KCNC4	NR_036437	chr1:110554859-110578197	non-template	30	Intron_3	--	Yes	--	Yes	--	--	--	Yes	--	--	--
G4ST01110574125	chr1:110574126-110574155	+	GGGCAGCGTCGGGGCTGGGCAGAGGCTGGG<br>.((...))	KCNC4	NM_001039574	chr1:110554859-110578197	non-template	30	Intron_3.l	--	Yes	--	Yes	--	--	--	Yes	--	--	--
G4ST01110574125	chr1:110574126-110574155	+	GGGCAGCGTCGGGGCTGGGCAGAGGCTGGG<br>.((...))	KCNC4	NM_004978	chr1:110554859-110578197	non-template	30	Intron_3.l	--	Yes	--	Yes	--	--	--	Yes	--	--	--
G4ST01110575054	chr1:110575055-110575100	-	GGGTGGGAGAGAGCCTTCTCTCAAGGAGGGAGGCTCAAGGCCTGGG<br>.(((((....)))))..... ((((.....))))	KCNC4	NR_036437	chr1:110554859-110578197	template	46	Intron_3	--	Yes	--	Yes	--	--	--	Yes	--	--	--
G4ST01110575054	chr1:110575055-110575100	-	GGGTGGGAGAGAGCCTTCTCTCAAGGAGGGAGGCTCAAGGCCTGGG<br>.(((((....)))))..... ((((.....))))	KCNC4	NM_001039574	chr1:110554859-110578197	template	46	Intron_3.l	--	Yes	--	Yes	--	--	--	Yes	--	--	--
G4ST01110575054	chr1:110575055-110575100	-	GGGTGGGAGAGAGCCTTCTCTCAAGGAGGGAGGCTCAAGGCCTGGG<br>.(((((....)))))..... ((((.....))))	KCNC4	NM_004978	chr1:110554859-110578197	template	46	Intron_3.l	--	Yes	--	Yes	--	--	--	Yes	--	--	--
G4ST01110643563	chr1:110643564-110643601	-	GGGGAGGAGGGAAAGAGGCCAGCTTCTGGGGCCTTGGG<br>..(((((....))))).	LOC440600	NR_036595	chr1:110630521-110683316	non-template	38	Intron_2	--	Yes	--	Yes	--	--	--	--	--	--	--
G4ST01110723751	chr1:110723752-110723781	+	GGGGGGTAGGGGGGGCAAGTTTTTGGAGGG<br>(((....)))..	SLC16A4	NM_001201549	chr1:110706996-110735227	template	30	Intron_5	--	Yes	--	Yes	--	--	--	--	--	--	--
G4ST01110723751	chr1:110723752-110723781	+	GGGGGGTAGGGGGGGCAAGTTTTTGGAGGG<br>(((....)))..	SLC16A4	NM_001201547	chr1:110706996-110735227	template	30	Intron_4	--	Yes	--	Yes	--	--	--	--	--	--	--
G4ST01110723751	chr1:110723752-110723781	+	GGGGGGTAGGGGGGGCAAGTTTTTGGAGGG<br>(((....)))..	SLC16A4	NM_001201546	chr1:110706996-110735227	template	30	Intron_4	--	Yes	--	Yes	--	--	--	--	--	--	--
G4ST01110723751	chr1:110723752-110723781	+	GGGGGGTAGGGGGGGCAAGTTTTTGGAGGG<br>(((....)))..	SLC16A4	NM_004696	chr1:110706996-110735227	template	30	Intron_5	--	Yes	--	Yes	--	--	--	--	--	--	--
G4ST01110723751	chr1:110723752-110723781	+	GGGGGGTAGGGGGGGCAAGTTTTTGGAGGG<br>(((....)))..	SLC16A4	NM_001201548	chr1:110706996-110735227	template	30	Intron_3	--	Yes	--	Yes	--	--	--	--	--	--	--
G4ST01110752209	chr1:110752210-110752249	+	GGGGCAAGTACGGGGAAGACACGGGACCTCTTGGAGGGGG<br>.((((...))))	HBXIP	NM_006402	chr1:110745400-110752069	template	-140	upstream	Yes	--	--	--	--	--	--	--	Yes	Yes	--
G4ST01110832063	chr1:110832064-110832100	+	GGGCAGCCAGGGCAGTCTTTGCTGAGGGAGGAGTGGG<br>((((....)))).	CYMP	NR_003599	chr1:110824911-110835414	non-template	37	Intron_5	--	Yes	--	Yes	--	--	--	Yes	Yes	--	--
G4ST01110948871	chr1:110948872-110948907	+	GGGTGCCCAGGGAGGGCAGCAGCCTCGTCTGCTGGG<br>.(((((......)))))	KCNA2	NM_004974	chr1:110947299-110950498	template	36	Exon_3.l	--	Yes	Yes	--	Yes	--	--	--	--	--	--
G4ST01110948871	chr1:110948872-110948907	+	GGGTGCCCAGGGAGGGCAGCAGCCTCGTCTGCTGGG<br>.(((((......)))))	KCNA2	NM_001204269	chr1:110937725-110975619	template	36	Exon_3	--	Yes	Yes	--	Yes	--	--	--	--	--	--
G4ST01110949712	chr1:110949713-110949751	+	GGGCTGGGCTGGAGGGGCGGTGAGGTGCCTGGCCAGGGG<br>..(((.((....)).)))..	KCNA2	NM_004974	chr1:110947299-110950498	template	39	Exon_2	--	Yes	Yes	--	--	Yes	--	--	--	--	--
G4ST01110949712	chr1:110949713-110949751	+	GGGCTGGGCTGGAGGGGCGGTGAGGTGCCTGGCCAGGGG<br>..(((.((....)).)))..	KCNA2	NM_001204269	chr1:110937725-110975619	template	39	Exon_2	--	Yes	Yes	--	--	Yes	--	--	--	--	--
G4ST01110949717	chr1:110949718-110949759	+	GGGCTGGAGGGGCGGTGAGGTGCCTGGCCAGGGGCATGCGGG<br>..(((.((....)).)))..	KCNA2	NM_004974	chr1:110947299-110950498	template	42	Exon_2	--	Yes	Yes	--	--	Yes	--	--	--	--	--
G4ST01110949717	chr1:110949718-110949759	+	GGGCTGGAGGGGCGGTGAGGTGCCTGGCCAGGGGCATGCGGG<br>..(((.((....)).)))..	KCNA2	NM_001204269	chr1:110937725-110975619	template	42	Exon_2	--	Yes	Yes	--	--	Yes	--	--	--	--	--
G4ST01110952724	chr1:110952725-110952763	+	GGGAGCCTGCCTAGAGCTGCAGGGGGTGGTCTGGGAGGG<br>..(((((........)))))	KCNA2	NM_001204269	chr1:110937725-110975619	template	39	Intron_1.f	--	Yes	--	Yes	--	--	--	--	--	--	--
G4ST01111538451	chr1:111538452-111538491	-	GGGGAAGGACAGGAAGTATTCCTGGGACAGCCTGGGTGGG<br>.......(((((...)))))	DENND2D	NM_024901	chr1:111531324-111544804	non-template	40	Intron_7	--	Yes	--	Yes	--	--	--	Yes	Yes	Yes	--
G4ST01111964115	chr1:111964116-111964149	+	GGGAGCGGGGGGCGCGGGTCGGCCGAGCCGAGGG<br>.((((...)))).	RAP1A	NM_002884	chr1:111963928-112057624	non-template	34	Intron_1.f	--	Yes	--	Yes	--	--	--	Yes	Yes	Yes	--
G4ST01111964115	chr1:111964116-111964149	+	GGGAGCGGGGGGCGCGGGTCGGCCGAGCCGAGGG<br>.((((...)))).	RAP1A	NM_001010935	chr1:111963928-112057624	non-template	34	Intron_1.f	--	Yes	--	Yes	--	--	--	Yes	Yes	Yes	--
G4ST01111964215	chr1:111964216-111964251	+	GGGGCGGCGGCCCGGGGGCCTGGCCGGCGGGAGGGG<br>.(((....))). .(((.....)))	RAP1A	NM_002884	chr1:111963928-112057624	non-template	36	Intron_1.f	--	Yes	--	Yes	--	--	--	Yes	Yes	Yes	--
G4ST01111964215	chr1:111964216-111964251	+	GGGGCGGCGGCCCGGGGGCCTGGCCGGCGGGAGGGG<br>.(((....))). .(((.....)))	RAP1A	NM_001010935	chr1:111963928-112057624	non-template	36	Intron_1.f	--	Yes	--	Yes	--	--	--	Yes	Yes	Yes	--
G4ST01112083614	chr1:112083615-112083651	+	GGGGGGCTGTCTTCAGCCAATGGGCAGGGCGGCCGGG<br>.(((((....)))))...	C1orf183	NM_019099	chr1:112066209-112083569	template	-45	upstream	Yes	--	--	--	--	--	--	Yes	Yes	Yes	Yes
G4ST01112083614	chr1:112083615-112083651	+	GGGGGGCTGTCTTCAGCCAATGGGCAGGGCGGCCGGG<br>.(((((....)))))...	C1orf183	NM_198926	chr1:112066209-112099942	template	37	Intron_1.u	--	Yes	--	Yes	--	--	--	Yes	Yes	Yes	Yes
G4ST01112084573	chr1:112084574-112084615	-	GGGCGCGGCGTCTCCGCGCTGGGAGGGAGCAGCGCTGGCGGG<br>(((((.....))))).. (((...)))...	C1orf183	NM_019099	chr1:112066209-112083569	non-template	-1004	upstream	Yes	--	--	--	--	--	--	Yes	Yes	--	--
G4ST01112084573	chr1:112084574-112084615	-	GGGCGCGGCGTCTCCGCGCTGGGAGGGAGCAGCGCTGGCGGG<br>(((((.....))))).. (((...)))...	C1orf183	NM_198926	chr1:112066209-112099942	non-template	42	Intron_1.u	--	Yes	--	Yes	--	--	--	Yes	Yes	--	--
G4ST01112122760	chr1:112122761-112122794	-	GGGAGGAAGGGGGCAGAATGCTGGGAATGAGGGG<br>.(((...))).	KCND3	NM_172198	chr1:112119977-112333300	non-template	34	Intron_5	--	Yes	--	Yes	--	--	--	--	Yes	--	--
G4ST01112122760	chr1:112122761-112122794	-	GGGAGGAAGGGGGCAGAATGCTGGGAATGAGGGG<br>.(((...))).	KCND3	NM_004980	chr1:112119977-112333300	non-template	34	Intron_5	--	Yes	--	Yes	--	--	--	--	Yes	--	--
G4ST01112137814	chr1:112137815-112137844	-	GGGCCCCAGGAGGGACCATGGGATAAAGGG<br>((...)).	KCND3	NM_172198	chr1:112119977-112333300	non-template	30	Intron_2	--	Yes	--	Yes	--	--	--	--	--	--	--
G4ST01112137814	chr1:112137815-112137844	-	GGGCCCCAGGAGGGACCATGGGATAAAGGG<br>((...)).	KCND3	NM_004980	chr1:112119977-112333300	non-template	30	Intron_2	--	Yes	--	Yes	--	--	--	--	--	--	--
G4ST01112165685	chr1:112165686-112165726	+	GGGTGGCCAGCTCTGGTCCTGGGTCACAGGGTTGCTCTGGG<br>.(((((....)))))..	KCND3	NM_172198	chr1:112119977-112333300	template	41	Intron_2	--	Yes	--	Yes	--	--	--	--	--	--	--
G4ST01112165685	chr1:112165686-112165726	+	GGGTGGCCAGCTCTGGTCCTGGGTCACAGGGTTGCTCTGGG<br>.(((((....)))))..	KCND3	NM_004980	chr1:112119977-112333300	template	41	Intron_2	--	Yes	--	Yes	--	--	--	--	--	--	--
G4ST01112225774	chr1:112225775-112225803	+	GGGAGTCCGGGGCAGCCCCTGGGGCAGGG<br>.(((...)))	KCND3	NM_172198	chr1:112119977-112333300	template	29	Intron_2	--	Yes	--	Yes	--	--	--	--	--	--	Yes
G4ST01112225774	chr1:112225775-112225803	+	GGGAGTCCGGGGCAGCCCCTGGGGCAGGG<br>.(((...)))	KCND3	NM_004980	chr1:112119977-112333300	template	29	Intron_2	--	Yes	--	Yes	--	--	--	--	--	--	Yes
G4ST01112225782	chr1:112225783-112225810	+	GGGGCAGCCCCTGGGGCAGGGCAAAGGG<br>.(((...)))	KCND3	NM_172198	chr1:112119977-112333300	template	28	Intron_2	--	Yes	--	Yes	--	--	--	--	--	--	Yes
G4ST01112225782	chr1:112225783-112225810	+	GGGGCAGCCCCTGGGGCAGGGCAAAGGG<br>.(((...)))	KCND3	NM_004980	chr1:112119977-112333300	template	28	Intron_2	--	Yes	--	Yes	--	--	--	--	--	--	Yes
G4ST01112226924	chr1:112226925-112226961	+	GGGTGAGGGTGGTGGAAGAGAGTCCAGCAGGGAGGGG<br>.(.((((......)))).).	KCND3	NM_172198	chr1:112119977-112333300	template	37	Intron_2	--	Yes	--	Yes	--	--	--	--	--	--	--
G4ST01112226924	chr1:112226925-112226961	+	GGGTGAGGGTGGTGGAAGAGAGTCCAGCAGGGAGGGG<br>.(.((((......)))).).	KCND3	NM_004980	chr1:112119977-112333300	template	37	Intron_2	--	Yes	--	Yes	--	--	--	--	--	--	--

G4ST01112264374	chr1:112264375-112264403	+	GGGGAGGGCAGGGCATGCAGCTGCATGGG<br>.((((....))))	KCND3	NM_172198	chr1:112119977-112333300	template	29	Intron_2	--	Yes	--	Yes	--	--	--	--	--	--	--
G4ST01112264374	chr1:112264375-112264403	+	GGGGAGGGCAGGGCATGCAGCTGCATGGG<br>.((((....))))	KCND3	NM_004980	chr1:112119977-112333300	template	29	Intron_2	--	Yes	--	Yes	--	--	--	--	--	--	--
G4ST01112272126	chr1:112272127-112272168	-	GGGGCCTGGTAGGTCCAGGCAGTGGGTAGGGGAGGCCCTGGG<br>((((((.....))))))...	KCND3	NM_172198	chr1:112119977-112333300	non-template	42	Intron_2	--	Yes	--	Yes	--	--	--	--	--	--	--
G4ST01112272126	chr1:112272127-112272168	-	GGGGCCTGGTAGGTCCAGGCAGTGGGTAGGGGAGGCCCTGGG<br>((((((.....))))))...	KCND3	NM_004980	chr1:112119977-112333300	non-template	42	Intron_2	--	Yes	--	Yes	--	--	--	--	--	--	--
G4ST01112281235	chr1:112281236-112281262	+	GGGTGTGGGGAGAGGGAGTTGTCTGGG<br>((....))	KCND3	NM_172198	chr1:112119977-112333300	template	27	Intron_2	--	Yes	--	Yes	--	--	--	Yes	--	--	--
G4ST01112281235	chr1:112281236-112281262	+	GGGTGTGGGGAGAGGGAGTTGTCTGGG<br>((....))	KCND3	NM_004980	chr1:112119977-112333300	template	27	Intron_2	--	Yes	--	Yes	--	--	--	Yes	--	--	--
G4ST01112333401	chr1:112333402-112333433	-	GGGCAGCCAGGCGCTGGGGTTCGGGGGTGGGG<br>((((.....))))	KCND3	NM_172198	chr1:112119977-112333300	non-template	-101	upstream	Yes	--	--	--	--	--	--	--	--	--	--
G4ST01112333401	chr1:112333402-112333433	-	GGGCAGCCAGGCGCTGGGGTTCGGGGGTGGGG<br>((((.....))))	KCND3	NM_004980	chr1:112119977-112333300	non-template	-101	upstream	Yes	--	--	--	--	--	--	--	--	--	--
G4ST01112333406	chr1:112333407-112333452	-	GGGCTGTGGAGCCACCTCCGGGCAGCCAGGCGCTGGGGTTCGGGGG<br>....((((....)))) ((((.....))))	KCND3	NM_172198	chr1:112119977-112333300	non-template	-106	upstream	Yes	--	--	--	--	--	--	--	--	--	--
G4ST01112333406	chr1:112333407-112333452	-	GGGCTGTGGAGCCACCTCCGGGCAGCCAGGCGCTGGGGTTCGGGGG<br>....((((....)))) ((((.....))))	KCND3	NM_004980	chr1:112119977-112333300	non-template	-106	upstream	Yes	--	--	--	--	--	--	--	--	--	--
G4ST01112740097	chr1:112740098-112740135	-	GGGCTTCAGGGTGGGCTCGCGCCCCTCCCGGCTCGGGG<br>..((.(((......))).))	CTTNBP2NL	NM_018704	chr1:112740323-112805309	template	-187	upstream	Yes	--	--	--	--	--	--	Yes	Yes	Yes	Yes
G4ST01112740124	chr1:112740125-112740160	-	GGGAGGAAGCGCCCTCCTGGGTCCTGGGCTTCAGGG<br>((((.......))))	CTTNBP2NL	NM_018704	chr1:112740323-112805309	template	-162	upstream	Yes	--	--	--	--	--	--	Yes	Yes	Yes	Yes
G4ST01112973988	chr1:112973989-112974019	-	GGGAGGAAAGGGAGGGCACTATGAGTGAGGG<br>((((...)))).	CAPZA1	NM_006135	chr1:112963598-113015764	template	31	Intron_1.f	--	Yes	--	Yes	--	--	--	Yes	--	--	Yes
G4ST01112984675	chr1:112984676-112984714	-	GGGCAGGGGGCTCCTCACTTCCCAGGAGGGGCGGCCGGG<br>..(((((........)))))	CAPZA1	NM_006135	chr1:112963598-113015764	template	39	Intron_1.f	--	Yes	--	Yes	--	--	--	--	--	--	--
G4ST01113019339	chr1:113019340-113019365	-	GGGGCGTGGGCGAGGCGCGGGAGGGG<br>((...)).	MOV10	NM_001130079	chr1:113018571-113044891	template	26	Intron_2	--	Yes	--	Yes	--	--	--	Yes	Yes	Yes	--
G4ST01113019339	chr1:113019340-113019365	-	GGGGCGTGGGCGAGGCGCGGGAGGGG<br>((...)).	MOV10	NM_020963	chr1:113018571-113044891	template	26	Intron_2	--	Yes	--	Yes	--	--	--	Yes	Yes	Yes	--
G4ST01113019435	chr1:113019436-113019469	-	GGGTTCCGGCCTCCGGGGGCGCGGGCAGCGCGGG<br>..((((...))))	MOV10	NM_001130079	chr1:113018571-113044891	template	34	Intron_2	--	Yes	--	Yes	--	--	--	Yes	Yes	Yes	--
G4ST01113019435	chr1:113019436-113019469	-	GGGTTCCGGCCTCCGGGGGCGCGGGCAGCGCGGG<br>..((((...))))	MOV10	NM_020963	chr1:113018571-113044891	template	34	Intron_2	--	Yes	--	Yes	--	--	--	Yes	Yes	Yes	--
G4ST01113019508	chr1:113019509-113019544	+	GGGGGCTGAGGGACAGCGCTCGCGGTGGGGGAGGGG<br>.((.((....)).)).	MOV10	NM_001130079	chr1:113018571-113044891	non-template	36	Intron_2	--	Yes	--	Yes	--	--	--	Yes	Yes	Yes	--
G4ST01113019508	chr1:113019509-113019544	+	GGGGGCTGAGGGACAGCGCTCGCGGTGGGGGAGGGG<br>.((.((....)).)).	MOV10	NM_020963	chr1:113018571-113044891	non-template	36	Intron_2	--	Yes	--	Yes	--	--	--	Yes	Yes	Yes	--
G4ST01113033506	chr1:113033507-113033535	-	GGGTTGGGGTGGGAGCGGTCTGACTGGGG<br>..((((...))))	MOV10	NM_001130079	chr1:113018571-113044891	template	29	Intron_3	--	Yes	--	Yes	--	--	--	--	--	--	Yes
G4ST01113033506	chr1:113033507-113033535	-	GGGTTGGGGTGGGAGCGGTCTGACTGGGG<br>..((((...))))	MOV10	NM_020963	chr1:113018571-113044891	template	29	Intron_3	--	Yes	--	Yes	--	--	--	--	--	--	Yes
G4ST01113033903	chr1:113033904-113033941	+	GGGGCTTCCTGGGGGAGTGTCCGGGATAAGGATATGGG<br>(((....)))	MOV10	NM_001130079	chr1:113018571-113044891	non-template	38	Intron_4	--	Yes	--	Yes	--	--	--	--	--	--	Yes
G4ST01113033903	chr1:113033904-113033941	+	GGGGCTTCCTGGGGGAGTGTCCGGGATAAGGATATGGG<br>(((....)))	MOV10	NM_020963	chr1:113018571-113044891	non-template	38	Intron_4	--	Yes	--	Yes	--	--	--	--	--	--	Yes
G4ST01113033913	chr1:113033914-113033945	+	GGGGGAGTGTCCGGGATAAGGATATGGGTGGG<br>(((....)))	MOV10	NM_001130079	chr1:113018571-113044891	non-template	32	Intron_4	--	Yes	--	Yes	--	--	--	--	--	--	Yes
G4ST01113033913	chr1:113033914-113033945	+	GGGGGAGTGTCCGGGATAAGGATATGGGTGGG<br>(((....)))	MOV10	NM_020963	chr1:113018571-113044891	non-template	32	Intron_4	--	Yes	--	Yes	--	--	--	--	--	--	Yes
G4ST01113033925	chr1:113033926-113033949	+	GGGATAAGGATATGGGTGGGAGGG<br>(((....)))	MOV10	NM_001130079	chr1:113018571-113044891	non-template	24	Intron_4	--	Yes	--	Yes	--	--	--	--	--	--	Yes
G4ST01113033925	chr1:113033926-113033949	+	GGGATAAGGATATGGGTGGGAGGG<br>(((....)))	MOV10	NM_020963	chr1:113018571-113044891	non-template	24	Intron_4	--	Yes	--	Yes	--	--	--	--	--	--	Yes
G4ST01113050757	chr1:113050758-113050800	-	GGGGCGGGATGTGACCATTGGGGCCTGCCACTGCTCGGCAGGG<br>(((....))).. (((.((....))..))).	RHOC	NM_001042678	chr1:113045272-113051548	non-template	43	Intron_1.f	--	Yes	--	Yes	--	--	--	Yes	Yes	Yes	--
G4ST01113050757	chr1:113050758-113050800	-	GGGGCGGGATGTGACCATTGGGGCCTGCCACTGCTCGGCAGGG<br>(((....))).. (((.((....))..))).	RHOC	NM_001042679	chr1:113045272-113051201	non-template	43	Intron_1.f	--	Yes	--	Yes	--	--	--	Yes	Yes	Yes	--
G4ST01113050757	chr1:113050758-113050800	-	GGGGCGGGATGTGACCATTGGGGCCTGCCACTGCTCGGCAGGG<br>(((....))).. (((.((....))..))).	RHOC	NM_175744	chr1:113045272-113051548	non-template	43	Intron_1.f	--	Yes	--	Yes	--	--	--	Yes	Yes	Yes	--
G4ST01113050777	chr1:113050778-113050807	-	GGGAATAGGGGCGGGATGTGACCATTGGGG<br>(((....)))..	RHOC	NM_001042678	chr1:113045272-113051548	non-template	30	Intron_1.f	--	Yes	--	Yes	--	--	--	Yes	Yes	Yes	--
G4ST01113050777	chr1:113050778-113050807	-	GGGAATAGGGGCGGGATGTGACCATTGGGG<br>(((....)))..	RHOC	NM_001042679	chr1:113045272-113051201	non-template	30	Intron_1.f	--	Yes	--	Yes	--	--	--	Yes	Yes	Yes	--
G4ST01113050777	chr1:113050778-113050807	-	GGGAATAGGGGCGGGATGTGACCATTGGGG<br>(((....)))..	RHOC	NM_175744	chr1:113045272-113051548	non-template	30	Intron_1.f	--	Yes	--	Yes	--	--	--	Yes	Yes	Yes	--
G4ST01113055058	chr1:113055059-113055097	-	GGGAGGTGGAGGTTCTACCTGAGGGTAGGGGTGGAAGGG<br>(((((((...)))))))..	PPM1J	NM_005167	chr1:113054139-113059473	non-template	39	Intron_7	--	Yes	--	Yes	--	--	--	--	--	--	--
G4ST01113059338	chr1:113059339-113059381	+	GGGCGGCGCCGAGGCGGCGTTGGGCAGGTCCGGGGATTTGGGG<br>((.(((....))).))..	PPM1J	NM_005167	chr1:113054139-113059473	template	43	Exon_1.f	--	Yes	Yes	--	Yes	--	--	--	Yes	Yes	--
G4ST01113067072	chr1:113067073-113067104	+	GGGAGGAGGGGCTGGAGGCCAGCCCGGGTGGG<br>(((((...)))))..	FAM19A3	NM_001004440	chr1:113064712-113071379	non-template	32	Intron_2	--	Yes	--	Yes	--	--	--	Yes	--	--	Yes
G4ST01113067072	chr1:113067073-113067104	+	GGGAGGAGGGGCTGGAGGCCAGCCCGGGTGGG<br>(((((...)))))..	FAM19A3	NM_182759	chr1:113064712-113071379	non-template	32	Intron_2	--	Yes	--	Yes	--	--	--	Yes	--	--	Yes
G4ST01113067079	chr1:113067080-113067108	+	GGGGCTGGAGGCCAGCCCGGGTGGGCGGG<br>(((((...)))))..	FAM19A3	NM_001004440	chr1:113064712-113071379	non-template	29	Intron_2	--	Yes	--	Yes	--	--	--	Yes	--	--	Yes
G4ST01113067079	chr1:113067080-113067108	+	GGGGCTGGAGGCCAGCCCGGGTGGGCGGG<br>(((((...)))))..	FAM19A3	NM_182759	chr1:113064712-113071379	non-template	29	Intron_2	--	Yes	--	Yes	--	--	--	Yes	--	--	Yes
G4ST01113300504	chr1:113300505-113300549	-	GGGACGCCGGTCACGTGGCGGGGAGGGGGCGCCGGCGCGCGCGGG<br>.(((((......))))) .((((....))))..	SLC16A1	NM_001166496	chr1:113255993-113300208	non-template	-296	upstream	Yes	--	--	--	--	--	--	Yes	Yes	Yes	--
G4ST01113300504	chr1:113300505-113300549	-	GGGACGCCGGTCACGTGGCGGGGAGGGGGCGCCGGCGCGCGCGGG<br>.(((((......))))) .((((....))))..	SLC16A1	NM_003051	chr1:113255993-113300498	non-template	-6	upstream	Yes	--	--	--	--	--	--	Yes	Yes	Yes	--
G4ST01113300520	chr1:113300521-113300559	-	GGGTCGGACTGGGACGCCGGTCACGTGGCGGGGAGGGGG<br>.(((((......)))))	SLC16A1	NM_001166496	chr1:113255993-113300208	non-template	-312	upstream	Yes	--	--	--	--	--	--	Yes	Yes	Yes	--
G4ST01113300520	chr1:113300521-113300559	-	GGGTCGGACTGGGACGCCGGTCACGTGGCGGGGAGGGGG<br>.(((((......)))))	SLC16A1	NM_003051	chr1:113255993-113300498	non-template	-22	upstream	Yes	--	--	--	--	--	--	Yes	Yes	Yes	--
G4ST01113300526	chr1:113300527-113300573	-	GGGGCGCAGCGCGCGGGTCGGACTGGGACGCCGGTCACGTGGCGGGG<br>((((...)))) .(((((......)))))	SLC16A1	NM_001166496	chr1:113255993-113300208	non-template	-318	upstream	Yes	--	--	--	--	--	--	Yes	Yes	Yes	--
G4ST01113300526	chr1:113300527-113300573	-	GGGGCGCAGCGCGCGGGTCGGACTGGGACGCCGGTCACGTGGCGGGG<br>((((...)))) .(((((......)))))	SLC16A1	NM_003051	chr1:113255993-113300498	non-template	-28	upstream	Yes	--	--	--	--	--	--	Yes	Yes	Yes	--
G4ST01113301107	chr1:113301108-113301137	+	GGGTGGGGAGACTGGCCCAGTGGGTTGGGG<br>...((((...))))	SLC16A1	NM_001166496	chr1:113255993-113300208	template	-899	upstream	Yes	--	--	--	--	--	--	Yes	Yes	Yes	--
G4ST01113301107	chr1:113301108-113301137	+	GGGTGGGGAGACTGGCCCAGTGGGTTGGGG<br>...((((...))))	SLC16A1	NM_003051	chr1:113255993-113300498	template	-609	upstream	Yes	--	--	--	--	--	--	Yes	Yes	Yes	--
G4ST01113734554	chr1:113734555-113734582	+	GGGCGCCGCGCGGCGGGAGGGGGCGGGG<br>.(((....)))	MAGI3	NM_001142782	chr1:113734998-114030068	non-template	-415	upstream	Yes	--	--	--	--	--	--	Yes	Yes	Yes	--
G4ST01113734554	chr1:113734555-113734582	+	GGGCGCCGCGCGGCGGGAGGGGGCGGGG<br>.(((....)))	MAGI3	NM_152900	chr1:113734998-114030068	non-template	-415	upstream	Yes	--	--	--	--	--	--	Yes	Yes	Yes	--

G4ST01114073421	chr1:114073422-114073457	+	GGGGGTGGAGTAGGGGATGTGGGGATGGTTCATGGG<br>.(((...)))	PHTF1	NM_006608	chr1:114041347-114103300	template	36	Intron_4	--	Yes	--	Yes	--	--	--	--	--	--	Yes
G4ST01114118896	chr1:114118897-114118936	+	GGGGCTGAGGTGGGAGGATCACTTGAGCCTGGGAGATGGG<br>(((.((....)).)))	RSBN1	NM_018364	chr1:114105977-114156593	template	40	Intron_4	--	Yes	--	Yes	--	--	--	--	--	--	--
G4ST01114248010	chr1:114248011-114248043	-	GGGACTGATTCTGATTTCAGTAGGGTGGGAGGG<br>(((((.........))))).	DCLRE1B	NM_022836	chr1:114249438-114258231	template	-1394	upstream	Yes	--	--	--	--	--	--	Yes	Yes	Yes	--
G4ST01114248010	chr1:114248011-114248043	-	GGGACTGATTCTGATTTCAGTAGGGTGGGAGGG<br>(((((.........))))).	AP4B1	NM_006594	chr1:114239200-114249264	template	33	Intron_2	--	Yes	--	Yes	--	--	--	Yes	Yes	Yes	--
G4ST01114273956	chr1:114273957-114273990	+	GGGGGCCGGGGCCCGGGCCGGCTCGCGCGGGGGG<br>((.((....)).))	HIPK1	NM_198268	chr1:114273519-114322014	non-template	34	Intron_1.f	--	Yes	--	Yes	--	--	--	--	Yes	Yes	Yes
G4ST01114273956	chr1:114273957-114273990	+	GGGGGCCGGGGCCCGGGCCGGCTCGCGCGGGGGG<br>((.((....)).))	HIPK1	NM_152696	chr1:114273519-114316218	non-template	34	Intron_1.f	--	Yes	--	Yes	--	--	--	--	Yes	Yes	Yes
G4ST01114438627	chr1:114438628-114438663	+	GGGCTGAGAGGGAAGGGGGCTGGTGCTGCAGCTGGG<br>.((((......)))).	SYT6	NM_205848	chr1:114433437-114497995	template	36	Intron_6	--	Yes	--	Yes	--	--	--	Yes	--	--	--
G4ST01114449131	chr1:114449132-114449158	-	GGGGACTACTAGAGTCGGGAGGGAGGG<br>((((.....))))	SYT6	NM_205848	chr1:114433437-114497995	non-template	27	Intron_3	--	Yes	--	Yes	--	--	--	--	--	--	Yes
G4ST01114451336	chr1:114451337-114451373	+	GGGAGGCTTGCCTGGGGATATGAGGGGTCCCTCGGGG<br>(((....))).	SYT6	NM_205848	chr1:114433437-114497995	template	37	Intron_3	--	Yes	--	Yes	--	--	--	--	--	--	--
G4ST01114456095	chr1:114456096-114456137	+	GGGCTCCTAGGGCTTCAGCAAGAATGCTGGGGGTGTCTGGGG<br>...(((((....))))).	SYT6	NM_205848	chr1:114433437-114497995	template	42	Intron_3	--	Yes	--	Yes	--	--	--	--	Yes	--	Yes
G4ST01114456104	chr1:114456105-114456145	+	GGGCTTCAGCAAGAATGCTGGGGGTGTCTGGGGTGGAAGGG<br>...(((((....))))).	SYT6	NM_205848	chr1:114433437-114497995	template	41	Intron_3	--	Yes	--	Yes	--	--	--	--	Yes	--	Yes
G4ST01114466683	chr1:114466684-114466718	+	GGGGCTGCCTGGGGTCTGGCCAGACTCAGGGTGGG<br>((((....))))....	SYT6	NM_205848	chr1:114433437-114497995	template	35	Intron_3	--	Yes	--	Yes	--	--	--	--	--	--	--
G4ST01114466693	chr1:114466694-114466728	+	GGGGTCTGGCCAGACTCAGGGTGGGTGGCAGAGGG<br>((((....))))....	SYT6	NM_205848	chr1:114433437-114497995	template	35	Intron_3	--	Yes	--	Yes	--	--	--	--	--	--	--
G4ST01114481470	chr1:114481471-114481504	+	GGGGCAGAGTGCAAGGGTAGACTGGGCCACTGGG<br>(((...)))..	SYT6	NM_205848	chr1:114433437-114497995	template	34	Intron_3	--	Yes	--	Yes	--	--	--	--	--	--	--
G4ST01114496424	chr1:114496425-114496456	-	GGGAAGGGGAGAAAGGGGCTGCCAGAGGCGGG<br>...(((...)))	SYT6	NM_205848	chr1:114433437-114497995	non-template	32	Intron_1.f	--	Yes	--	Yes	--	--	--	Yes	--	--	--
G4ST01114854468	chr1:114854469-114854504	-	GGGAGGGGTGCCGGCTGCGAGAGGCTGGGGGAGGGG<br>...((((((......))))))	TRIM33	NM_015906	chr1:114736922-114855304	non-template	36	Intron_1.f	--	Yes	--	Yes	--	--	--	Yes	Yes	Yes	--
G4ST01114854468	chr1:114854469-114854504	-	GGGAGGGGTGCCGGCTGCGAGAGGCTGGGGGAGGGG<br>...((((((......))))))	TRIM33	NM_033020	chr1:114736922-114855304	non-template	36	Intron_1.f	--	Yes	--	Yes	--	--	--	Yes	Yes	Yes	--
G4ST01115014195	chr1:115014196-115014225	-	GGGGCCTTCGGCAGGGCCTGCGGGGGCGGG<br>(((...))).	DENND2C	NM_198459	chr1:114928719-115014255	non-template	30	Exon_1.f	--	Yes	Yes	--	--	Yes	--	Yes	Yes	Yes	--
G4ST01115014199	chr1:115014200-115014230	-	GGGACGGGGCCTTCGGCAGGGCCTGCGGGGG<br>(((...))).	DENND2C	NM_198459	chr1:114928719-115014255	non-template	31	Exon_1.f	--	Yes	Yes	--	--	Yes	--	Yes	Yes	Yes	--
G4ST01115302442	chr1:115302443-115302472	+	GGGAGGGATGAAGAGGGATTGATTAATGGG<br>(((....)))	SYCP1	NM_003176	chr1:115198978-115339513	non-template	30	Intron_27	--	Yes	--	Yes	--	--	--	--	--	--	Yes
G4ST01115405089	chr1:115405090-115405128	+	GGGAGCATCACTGATGCGGCCTGGGCATGGGGAAGGGGG<br>.(((((...))))).....	TSPAN2	NM_005725	chr1:115392156-115433644	template	39	Intron_3	--	Yes	--	Yes	--	--	--	--	Yes	--	--
G4ST01115414710	chr1:115414711-115414749	-	GGGGGCTCTGTTGGGGTCTGAGGACTAGGGCATGGTGGG<br>(((....)))..	TSPAN2	NM_005725	chr1:115392156-115433644	non-template	39	Intron_2	--	Yes	--	Yes	--	--	--	--	--	--	Yes
G4ST01115414719	chr1:115414720-115414755	-	GGGGCTGGGGGCTCTGTTGGGGTCTGAGGACTAGGG<br>(((....)))..	TSPAN2	NM_005725	chr1:115392156-115433644	non-template	36	Intron_2	--	Yes	--	Yes	--	--	--	--	--	--	Yes
G4ST01115432866	chr1:115432867-115432903	-	GGGTTCTCTTCTGAGAAGGGGATGGGGAATGGAAGGG<br>(((((....))))).	TSPAN2	NM_005725	chr1:115392156-115433644	non-template	37	Intron_1.f	--	Yes	--	Yes	--	--	--	--	--	--	--
G4ST01115680092	chr1:115680093-115680119	-	GGGCAGGGCAGGGCTGGAGCCCGAGGG<br>.(((...))).	NGF	NM_002506	chr1:115630060-115682380	non-template	27	Intron_1.f	--	Yes	--	Yes	--	--	--	--	--	--	Yes
G4ST01115681759	chr1:115681760-115681791	-	GGGCTGGGAGTCGTGACTGAGGGCCCCGGGGG<br>(((....)))..	NGF	NM_002506	chr1:115630060-115682380	non-template	32	Intron_1.f	--	Yes	--	Yes	--	--	--	--	Yes	--	--
G4ST01115682288	chr1:115682289-115682315	-	GGGGCGCAGCGCGGGCGCGGGCAGGGG<br>(((...)))	NGF	NM_002506	chr1:115630060-115682380	non-template	27	Intron_1.f	--	Yes	--	Yes	--	--	--	Yes	Yes	--	Yes
G4ST01115986643	chr1:115986644-115986679	+	GGGCGGCTGCAGCAGCCGTGCAGGGCGGGAGCCGGG<br>((((((...))))))....	VANGL1	NM_138959	chr1:115986097-116042368	non-template	36	Intron_1.f	--	Yes	--	Yes	--	--	--	Yes	Yes	Yes	--
G4ST01115986643	chr1:115986644-115986679	+	GGGCGGCTGCAGCAGCCGTGCAGGGCGGGAGCCGGG<br>((((((...))))))....	VANGL1	NM_001172412	chr1:115986773-116042368	non-template	-93	upstream	Yes	--	--	--	--	--	--	Yes	Yes	Yes	--
G4ST01115986643	chr1:115986644-115986679	+	GGGCGGCTGCAGCAGCCGTGCAGGGCGGGAGCCGGG<br>((((((...))))))....	VANGL1	NM_001172411	chr1:115986097-116042368	non-template	36	Intron_1.f	--	Yes	--	Yes	--	--	--	Yes	Yes	Yes	--
G4ST01116036324	chr1:116036325-116036368	+	GGGAAGTGTGGGGTGCCCAGCAGGTAAAGCACAGGGAGAGAGGG<br>((((((....))....)))).	VANGL1	NM_138959	chr1:115986097-116042368	non-template	44	Exon_8.l	--	Yes	Yes	--	--	--	Yes	--	--	--	--
G4ST01116036324	chr1:116036325-116036368	+	GGGAAGTGTGGGGTGCCCAGCAGGTAAAGCACAGGGAGAGAGGG<br>((((((....))....)))).	VANGL1	NM_001172412	chr1:115986773-116042368	non-template	44	Exon_8.l	--	Yes	Yes	--	--	--	Yes	--	--	--	--
G4ST01116036324	chr1:116036325-116036368	+	GGGAAGTGTGGGGTGCCCAGCAGGTAAAGCACAGGGAGAGAGGG<br>((((((....))....)))).	VANGL1	NM_001172411	chr1:115986097-116042368	non-template	44	Exon_8.l	--	Yes	Yes	--	--	--	Yes	--	--	--	--
G4ST01116036333	chr1:116036334-116036375	+	GGGGTGCCCAGCAGGTAAAGCACAGGGAGAGAGGGCAGTGGG<br>((((((....))....)))).	VANGL1	NM_138959	chr1:115986097-116042368	non-template	42	Exon_8.l	--	Yes	Yes	--	--	--	Yes	--	--	--	--
G4ST01116036333	chr1:116036334-116036375	+	GGGGTGCCCAGCAGGTAAAGCACAGGGAGAGAGGGCAGTGGG<br>((((((....))....)))).	VANGL1	NM_001172412	chr1:115986773-116042368	non-template	42	Exon_8.l	--	Yes	Yes	--	--	--	Yes	--	--	--	--
G4ST01116036333	chr1:116036334-116036375	+	GGGGTGCCCAGCAGGTAAAGCACAGGGAGAGAGGGCAGTGGG<br>((((((....))....)))).	VANGL1	NM_001172411	chr1:115986097-116042368	non-template	42	Exon_8.l	--	Yes	Yes	--	--	--	Yes	--	--	--	--
G4ST01116187238	chr1:116187239-116187276	-	GGGAGGTCTTAGAAAAGGCAAAGGGGTGGGGGAGGGGG<br>..(((((....)))))....	NHLH2	NM_005599	chr1:116180522-116185270	non-template	-1968	upstream	Yes	--	--	--	--	--	--	--	--	--	--
G4ST01116320591	chr1:116320592-116320620	-	GGGGCGGGGCGCCTGCGCGGGCGGCGGGG<br>(((....)))	SLC22A15	NM_018420	chr1:116320642-116414198	template	-21	upstream	Yes	--	--	--	--	--	--	Yes	Yes	Yes	Yes
G4ST01116320599	chr1:116320600-116320625	-	GGGCCGGGGCGGGGCGCCTGCGCGGG<br>(((....)))	SLC22A15	NM_018420	chr1:116320642-116414198	template	-16	upstream	Yes	--	--	--	--	--	--	Yes	Yes	Yes	Yes
G4ST01116320710	chr1:116320711-116320736	+	GGGCGGGAGGGCAGCGCCTGAGAGGG<br>(((...)))...	SLC22A15	NM_018420	chr1:116320642-116414198	non-template	26	Exon_1.f	--	Yes	Yes	--	--	Yes	--	Yes	Yes	Yes	--
G4ST01116320714	chr1:116320715-116320744	+	GGGAGGGCAGCGCCTGAGAGGGCGGTGGGG<br>(((...)))...	SLC22A15	NM_018420	chr1:116320642-116414198	non-template	30	Exon_1.f	--	Yes	Yes	--	--	Yes	--	Yes	Yes	Yes	--
G4ST01116320718	chr1:116320719-116320752	+	GGGCAGCGCCTGAGAGGGCGGTGGGGTGGCGGGG<br>(((...)))...	SLC22A15	NM_018420	chr1:116320642-116414198	non-template	34	Exon_1.f	--	Yes	Yes	--	--	Yes	--	Yes	Yes	Yes	--
G4ST01116477672	chr1:116477673-116477707	-	GGGCTCCAGGGAGGCCTGGGCCAGACATCCTGGGG<br>.(((.....)))	MAB21L3	NM_152367	chr1:116455899-116479384	template	35	Exon_7.l	--	Yes	Yes	--	--	--	Yes	--	--	--	--
G4ST01116717054	chr1:116717055-116717092	+	GGGCCCTACATAGTAGGCGGGTCATGCAGGGCAGCGGG<br>.(((((...))))).	ATP1A1	NM_000701	chr1:116717318-116748919	non-template	-225	upstream	Yes	--	--	--	--	--	--	Yes	Yes	Yes	Yes
G4ST01116717054	chr1:116717055-116717092	+	GGGCCCTACATAGTAGGCGGGTCATGCAGGGCAGCGGG<br>.(((((...))))).	ATP1A1	NM_001160233	chr1:116718012-116748919	non-template	-919	upstream	Yes	--	--	--	--	--	--	Yes	Yes	Yes	Yes
G4ST01116717950	chr1:116717951-116717992	+	GGGCCGGGGGCTCCGAGAGGCGGTGCTTGGGAAGCCTCGGGG<br>.((.(((.....))).))..	ATP1A1	NM_000701	chr1:116717318-116748919	non-template	42	Intron_1.f	--	Yes	--	Yes	--	--	--	Yes	Yes	Yes	Yes
G4ST01116717950	chr1:116717951-116717992	+	GGGCCGGGGGCTCCGAGAGGCGGTGCTTGGGAAGCCTCGGGG<br>.((.(((.....))).))..	ATP1A1	NM_001160233	chr1:116718012-116748919	non-template	-19	upstream	Yes	--	--	--	--	--	--	Yes	Yes	Yes	Yes
G4ST01116717955	chr1:116717956-116717997	+	GGGGGCTCCGAGAGGCGGTGCTTGGGAAGCCTCGGGGCCGGG<br>.((.(((.....))).))..	ATP1A1	NM_000701	chr1:116717318-116748919	non-template	42	Intron_1.f	--	Yes	--	Yes	--	--	--	Yes	Yes	Yes	Yes
G4ST01116717955	chr1:116717956-116717997	+	GGGGGCTCCGAGAGGCGGTGCTTGGGAAGCCTCGGGGCCGGG<br>.((.(((.....))).))..	ATP1A1	NM_001160233	chr1:116718012-116748919	non-template	-14	upstream	Yes	--	--	--	--	--	--	Yes	Yes	Yes	Yes
G4ST01116718100	chr1:116718101-116718142	-	GGGGAGGAGCGCGCCTTTCCTCAGGGCACGGAGGGCGGAGGG<br>(((((.........))))).	ATP1A1	NM_000701	chr1:116717318-116748919	template	42	Intron_1.f	--	Yes	--	Yes	--	--	--	Yes	Yes	Yes	--

G4ST01116718100	chr1:116718101-116718142	-	GGGGAGGAGCGCGCCTTTCCTCAGGGCACGGAGGGCGGAGGG<br>(((((.........))))).	ATP1A1	NM_001160233	chr1:116718012-116748919	template	42	Exon_1.f	--	Yes	Yes	--	--	Yes	--	Yes	Yes	Yes	--
G4ST01116718107	chr1:116718108-116718148	-	GGGGAAGGGGAGGAGCGCGCCTTTCCTCAGGGCACGGAGGG<br>(((((.........))))).	ATP1A1	NM_000701	chr1:116717318-116748919	template	41	Intron_1.f	--	Yes	--	Yes	--	--	--	Yes	Yes	Yes	--
G4ST01116718107	chr1:116718108-116718148	-	GGGGAAGGGGAGGAGCGCGCCTTTCCTCAGGGCACGGAGGG<br>(((((.........))))).	ATP1A1	NM_001160233	chr1:116718012-116748919	template	41	Exon_1.f	--	Yes	Yes	--	--	Yes	--	Yes	Yes	Yes	--
G4ST01116878772	chr1:116878773-116878807	-	GGGCAGCGCCGCCGCGGCGGGCCTGGGGCTTGGGG<br>....((((...))))	CD58	NR_026665	chr1:116858679-116915238	non-template	35	Intron_3	--	Yes	--	Yes	--	--	--	--	--	Yes	--
G4ST01116878772	chr1:116878773-116878807	-	GGGCAGCGCCGCCGCGGCGGGCCTGGGGCTTGGGG<br>....((((...))))	CD58	NM_001779	chr1:116858679-116915238	non-template	35	Intron_3	--	Yes	--	Yes	--	--	--	--	--	Yes	--
G4ST01116878772	chr1:116878773-116878807	-	GGGCAGCGCCGCCGCGGCGGGCCTGGGGCTTGGGG<br>....((((...))))	CD58	NM_001144822	chr1:116862845-116915238	non-template	35	Intron_3	--	Yes	--	Yes	--	--	--	--	--	Yes	--
G4ST01116878779	chr1:116878780-116878811	-	GGGTGGGCAGCGCCGCCGCGGCGGGCCTGGGG<br>....((((...))))	CD58	NR_026665	chr1:116858679-116915238	non-template	32	Intron_3	--	Yes	--	Yes	--	--	--	--	--	Yes	--
G4ST01116878779	chr1:116878780-116878811	-	GGGTGGGCAGCGCCGCCGCGGCGGGCCTGGGG<br>....((((...))))	CD58	NM_001779	chr1:116858679-116915238	non-template	32	Intron_3	--	Yes	--	Yes	--	--	--	--	--	Yes	--
G4ST01116878779	chr1:116878780-116878811	-	GGGTGGGCAGCGCCGCCGCGGCGGGCCTGGGG<br>....((((...))))	CD58	NM_001144822	chr1:116862845-116915238	non-template	32	Intron_3	--	Yes	--	Yes	--	--	--	--	--	Yes	--
G4ST01116892053	chr1:116892054-116892095	-	GGGTGTGGTGGGAAAAGGGAACACCTGTACACTGCTGGTGGG<br>...(((.((.....)).)))	CD58	NR_026665	chr1:116858679-116915238	non-template	42	Intron_1.f	--	Yes	--	Yes	--	--	--	--	--	--	--
G4ST01116892053	chr1:116892054-116892095	-	GGGTGTGGTGGGAAAAGGGAACACCTGTACACTGCTGGTGGG<br>...(((.((.....)).)))	CD58	NM_001779	chr1:116858679-116915238	non-template	42	Intron_1.f	--	Yes	--	Yes	--	--	--	--	--	--	--
G4ST01116892053	chr1:116892054-116892095	-	GGGTGTGGTGGGAAAAGGGAACACCTGTACACTGCTGGTGGG<br>...(((.((.....)).)))	CD58	NM_001144822	chr1:116862845-116915238	non-template	42	Intron_1.f	--	Yes	--	Yes	--	--	--	--	--	--	--
G4ST01116955111	chr1:116955112-116955144	-	GGGGCTCTGGGGGTTCCTTGAACTGTGGGAGGG<br>.((((...))))....	IGSF3	NM_001007237	chr1:116918554-117011837	non-template	33	Intron_4	--	Yes	--	Yes	--	--	--	--	--	--	--
G4ST01116955111	chr1:116955112-116955144	-	GGGGCTCTGGGGGTTCCTTGAACTGTGGGAGGG<br>.((((...))))....	IGSF3	NM_001542	chr1:116918554-117011837	non-template	33	Intron_4	--	Yes	--	Yes	--	--	--	--	--	--	--
G4ST01116975143	chr1:116975144-116975178	+	GGGCAGGGATAGGGTGCTCTACTAAAGAGCATGGG<br>.(((((.....)))))..	IGSF3	NM_001007237	chr1:116918554-117011837	template	35	Intron_2	--	Yes	--	Yes	--	--	--	--	--	--	--
G4ST01116975143	chr1:116975144-116975178	+	GGGCAGGGATAGGGTGCTCTACTAAAGAGCATGGG<br>.(((((.....)))))..	IGSF3	NM_001542	chr1:116918554-117011837	template	35	Intron_2	--	Yes	--	Yes	--	--	--	--	--	--	--
G4ST01116975148	chr1:116975149-116975188	+	GGGATAGGGTGCTCTACTAAAGAGCATGGGCTTGGCCGGG<br>.(((((.....)))))..	IGSF3	NM_001007237	chr1:116918554-117011837	template	40	Intron_2	--	Yes	--	Yes	--	--	--	--	--	--	--
G4ST01116975148	chr1:116975149-116975188	+	GGGATAGGGTGCTCTACTAAAGAGCATGGGCTTGGCCGGG<br>.(((((.....)))))..	IGSF3	NM_001542	chr1:116918554-117011837	template	40	Intron_2	--	Yes	--	Yes	--	--	--	--	--	--	--
G4ST01117011887	chr1:117011888-117011917	-	GGGCTGGAGCTGGAGGGGCGGGGACTCGGG<br>(((....)))..	IGSF3	NM_001007237	chr1:116918554-117011837	non-template	-50	upstream	Yes	--	--	--	--	--	--	Yes	Yes	Yes	Yes
G4ST01117011887	chr1:117011888-117011917	-	GGGCTGGAGCTGGAGGGGCGGGGACTCGGG<br>(((....)))..	IGSF3	NM_001542	chr1:116918554-117011837	non-template	-50	upstream	Yes	--	--	--	--	--	--	Yes	Yes	Yes	Yes
G4ST01117011894	chr1:117011895-117011922	-	GGGCCGGGCTGGAGCTGGAGGGGCGGGG<br>(((....)))..	IGSF3	NM_001007237	chr1:116918554-117011837	non-template	-57	upstream	Yes	--	--	--	--	--	--	Yes	Yes	Yes	Yes
G4ST01117011894	chr1:117011895-117011922	-	GGGCCGGGCTGGAGCTGGAGGGGCGGGG<br>(((....)))..	IGSF3	NM_001542	chr1:116918554-117011837	non-template	-57	upstream	Yes	--	--	--	--	--	--	Yes	Yes	Yes	Yes
G4ST01117351415	chr1:117351416-117351439	-	GGGGCACACTGCGGGCTGGGTGGG<br>(((...)))	CD101	NM_004258	chr1:117345905-117380690	template	24	Intron_1.f	--	Yes	--	Yes	--	--	--	Yes	Yes	--	--
G4ST01117508898	chr1:117508899-117508929	+	GGGTGCGTGATAGCGCAGGGACTGGGCTGGG<br>.((((....)))).	VTCN1	NM_024626	chr1:117487732-117555072	template	31	Intron_2	--	Yes	--	Yes	--	--	--	--	--	--	--
G4ST01117710902	chr1:117710903-117710929	+	GGGCAGCCAGGCTGGGTGGGGGAGGGG<br>.(((...)))	MAN1A2	NM_006699	chr1:117711608-117869843	non-template	-678	upstream	Yes	--	--	--	--	--	--	Yes	Yes	Yes	--
G4ST01117711114	chr1:117711115-117711152	+	GGGTACGGGGACCGGGCCGCAGCCCCGAGCGGCGTGGG<br>.(((.((.....)).))).	MAN1A2	NM_006699	chr1:117711608-117869843	non-template	-455	upstream	Yes	--	--	--	--	--	--	Yes	Yes	Yes	Yes
G4ST01118266817	chr1:118266818-118266846	+	GGGGAGGGATTGTACGAATAGGGTGTGGG<br>(((.....))).	GDAP2	NM_017686	chr1:118207630-118273825	template	29	Intron_1.f	--	Yes	--	Yes	--	--	--	--	--	--	--
G4ST01118266817	chr1:118266818-118266846	+	GGGGAGGGATTGTACGAATAGGGTGTGGG<br>(((.....))).	GDAP2	NM_001135589	chr1:118221367-118273825	template	29	Intron_1.f	--	Yes	--	Yes	--	--	--	--	--	--	--
G4ST01118436436	chr1:118436437-118436475	-	GGGAGAGGGAGAATGGGATGACTCTGAGTCACCCAGGGG<br>.(((((...)))))....	SPAG17	NM_206996	chr1:118297811-118529371	non-template	39	Intron_8	--	Yes	--	Yes	--	--	--	--	--	--	--
G4ST01118511942	chr1:118511943-118511975	+	GGGGCCTGGTGGGAGGTGACTGGGTCATGGGGG<br>((....))	SPAG17	NM_206996	chr1:118297811-118529371	template	33	Intron_1.f	--	Yes	--	Yes	--	--	--	--	--	--	--
G4ST01118529154	chr1:118529155-118529191	+	GGGTCTGGCCGGGCCTCTGGCATTGCCGGGGGCAGGG<br>...(((((...)))))	SPAG17	NM_206996	chr1:118297811-118529371	template	37	Intron_1.f	--	Yes	--	Yes	--	--	--	--	Yes	Yes	Yes
G4ST01118529164	chr1:118529165-118529195	+	GGGCCTCTGGCATTGCCGGGGGCAGGGAGGG<br>...(((((...)))))	SPAG17	NM_206996	chr1:118297811-118529371	template	31	Intron_1.f	--	Yes	--	Yes	--	--	--	--	Yes	Yes	Yes
G4ST01119288288	chr1:119288289-119288326	-	GGGAGGAAGACTTTTCCTGGAGGGTCTGGGAAGTTGGG<br>(((((.....)))))...	TBX15	NM_152380	chr1:119227189-119333702	non-template	38	Intron_1.f	--	Yes	--	Yes	--	--	--	--	Yes	--	Yes
G4ST01119314077	chr1:119314078-119314115	+	GGGAGAGGGACCTGATGGGAGGTGATTGGATCATGGGG<br>..((((.....)))).	TBX15	NM_152380	chr1:119227189-119333702	template	38	Intron_1.f	--	Yes	--	Yes	--	--	--	--	--	--	--
G4ST01119333131	chr1:119333132-119333161	+	GGGGCGGGCGGCGGAGCGCGGGGTTTAGGG<br>..(((...))).	TBX15	NM_152380	chr1:119227189-119333702	template	30	Intron_1.f	--	Yes	--	Yes	--	--	--	Yes	--	--	--
G4ST01119484634	chr1:119484635-119484668	+	GGGCAGTGGGATGAGAAGAACCTCGCGGGATGGG<br>.((((......)))).	WARS2	NM_015836	chr1:119375362-119484818	template	34	Intron_1.f	--	Yes	--	Yes	--	--	--	Yes	Yes	Yes	--
G4ST01119484634	chr1:119484635-119484668	+	GGGCAGTGGGATGAGAAGAACCTCGCGGGATGGG<br>.((((......)))).	WARS2	NM_201263	chr1:119375362-119484818	template	34	Intron_1.f	--	Yes	--	Yes	--	--	--	Yes	Yes	Yes	--
G4ST01119738129	chr1:119738130-119738167	-	GGGGAACACAGGGGTTTGGAGGGCCTGAAGCACAGGGG<br>.(((.....)))	HAO2	NM_016527	chr1:119712925-119738274	template	38	Exon_8.l	--	Yes	Yes	--	--	--	Yes	--	Yes	--	--
G4ST01119738129	chr1:119738130-119738167	-	GGGGAACACAGGGGTTTGGAGGGCCTGAAGCACAGGGG<br>.(((.....)))	HAO2	NM_001005783	chr1:119712925-119738274	template	38	Exon_9.l	--	Yes	Yes	--	--	--	Yes	--	Yes	--	--
G4ST01119966805	chr1:119966806-119966845	-	GGGTCCGCGGGAGGCTGCCGTCTGGGGAGCCTGTGGGGGG<br>((((....)))).	ZNF697	NM_001080470	chr1:119963523-119991913	non-template	40	Exon_3.l	--	Yes	Yes	--	--	--	Yes	--	--	--	--
G4ST01119966818	chr1:119966819-119966851	-	GGGGCTGGGTCCGCGGGAGGCTGCCGTCTGGGG<br>((((....)))).	ZNF697	NM_001080470	chr1:119963523-119991913	non-template	33	Exon_3.l	--	Yes	Yes	--	Yes	--	Yes	--	--	--	--
G4ST01119969322	chr1:119969323-119969356	+	GGGCAGCAGGGAGTCTGGGACTGTGTCACAGGGG<br>.((((...))))	ZNF697	NM_001080470	chr1:119963523-119991913	template	34	Intron_2.l	--	Yes	--	Yes	--	--	--	--	--	--	--
G4ST01119992258	chr1:119992259-119992294	-	GGGCAGACGCGCTGCGGTGCGGGGCTCGGGCCGGGG<br>....(((((....)))))	ZNF697	NM_001080470	chr1:119963523-119991913	non-template	-345	upstream	Yes	--	--	--	--	--	--	Yes	Yes	Yes	--
G4ST01120147884	chr1:120147885-120147921	+	GGGAGGAGTCACTTTGGCTTGGGAGACAAGGGAAGGG<br>...(((((...))))).	REG4	NM_001159353	chr1:120146469-120155726	template	37	Intron_2.l	--	Yes	--	Yes	--	--	--	--	--	--	--
G4ST01120147884	chr1:120147885-120147921	+	GGGAGGAGTCACTTTGGCTTGGGAGACAAGGGAAGGG<br>...(((((...))))).	REG4	NM_001159352	chr1:120138164-120155726	template	37	Intron_3	--	Yes	--	Yes	--	--	--	--	--	--	--
G4ST01120147884	chr1:120147885-120147921	+	GGGAGGAGTCACTTTGGCTTGGGAGACAAGGGAAGGG<br>...(((((...))))).	REG4	NM_032044	chr1:120138164-120155726	template	37	Intron_2	--	Yes	--	Yes	--	--	--	--	--	--	--
G4ST01120639207	chr1:120639208-120639241	+	GGGATGGGAGTGGGCGAAGAGAAGTCCATCGGGG<br>(((.((....))..)))	FAM72B	NM_001100910	chr1:120640528-120657204	non-template	-1286	upstream	Yes	--	--	--	--	--	--	--	--	--	Yes
G4ST01120639212	chr1:120639213-120639245	+	GGGAGTGGGCGAAGAGAAGTCCATCGGGGCGGG<br>(((.((....))..)))	FAM72B	NM_001100910	chr1:120640528-120657204	non-template	-1282	upstream	Yes	--	--	--	--	--	--	--	--	--	Yes
G4ST01120639218	chr1:120639219-120639253	+	GGGCGAAGAGAAGTCCATCGGGGCGGGCCGGGGGG<br>(((.((....))..)))	FAM72B	NM_001100910	chr1:120640528-120657204	non-template	-1274	upstream	Yes	--	--	--	--	--	--	--	--	--	Yes
G4ST01120991065	chr1:120991066-120991094	-	GGGAGGGCAGGGGGTGCAGAGCATTAGGG<br>.((((...))))..	EMBP1	NR_003955	chr1:120962433-121015209	template	29	Intron_1.f	--	Yes	--	Yes	--	--	--	--	--	--	--

G4ST01142481007	chr1:142481008-142481046	+	GGGCACCTACTAGGTGCTAGGGTGGCTCTGGGCGAAGGG<br>(((((...)))))...	LOC100130000	NR_033189	chr1:142478653-142505703	non-template	39	Intron_1.f	--	Yes	--	Yes	--	--	--	--	--	--	--
G4ST01142534905	chr1:142534906-142534935	+	GGGGAGGCCTGGCCGGGCGGGAGGCGAGGG<br>..(((...)))	FLJ39739	NR_027468	chr1:142510036-142535980	template	30	Intron_1.f	--	Yes	--	Yes	--	--	--	Yes	Yes	Yes	Yes
G4ST01142534905	chr1:142534906-142534935	+	GGGGAGGCCTGGCCGGGCGGGAGGCGAGGG<br>..(((...)))	LOC100286793	NR_027469	chr1:142510036-142536110	template	30	Intron_1.f	--	Yes	--	Yes	--	--	--	Yes	Yes	Yes	Yes
G4ST01142625730	chr1:142625731-142625765	-	GGGCGAAGAGAAGTCCATCGGGGCGGGCCGGGGGG<br>(((.((....))..)))	SRGAP2P2	NR_034178	chr1:142627088-142805834	template	-1322	upstream	Yes	--	--	--	--	--	--	--	--	--	Yes
G4ST01142625738	chr1:142625739-142625771	-	GGGAGTGGGCGAAGAGAAGTCCATCGGGGCGGG<br>(((.((....))..)))	SRGAP2P2	NR_034178	chr1:142627088-142805834	template	-1316	upstream	Yes	--	--	--	--	--	--	--	--	--	Yes
G4ST01142625742	chr1:142625743-142625776	-	GGGATGGGAGTGGGCGAAGAGAAGTCCATCGGGG<br>(((.((....))..)))	SRGAP2P2	NR_034178	chr1:142627088-142805834	template	-1311	upstream	Yes	--	--	--	--	--	--	--	--	--	Yes
G4ST01142738409	chr1:142738410-142738450	-	GGGTAGGGGATGAATTTGTCATTCATGAGGGCCCACAGGGG<br>.((((((.....)))))))..	SRGAP2P2	NR_034178	chr1:142627088-142805834	template	41	Intron_2	--	Yes	--	Yes	--	--	--	--	--	--	--
G4ST01142804485	chr1:142804486-142804516	+	GGGAGCTAGCAGCTATGGTGGGAGGGATGGG<br>((((...)))).....	SRGAP2P2	NR_034178	chr1:142627088-142805834	non-template	31	Intron_3.l	--	Yes	--	Yes	--	--	--	--	--	--	--
G4ST01143020548	chr1:143020549-143020587	-	GGGCACCTACTAGGTGCTAGGGTGGCTCTGGGCGAAGGG<br>(((((...)))))...	LOC728855	NR_024510	chr1:143011869-143052130	non-template	39	Intron_1.u	--	Yes	--	Yes	--	--	--	--	--	--	--
G4ST01143020548	chr1:143020549-143020587	-	GGGCACCTACTAGGTGCTAGGGTGGCTCTGGGCGAAGGG<br>(((((...)))))...	LOC728855	NR_024511	chr1:143011869-143052130	non-template	39	Intron_1.f	--	Yes	--	Yes	--	--	--	--	--	--	--
G4ST01143020548	chr1:143020549-143020587	-	GGGCACCTACTAGGTGCTAGGGTGGCTCTGGGCGAAGGG<br>(((((...)))))...	LOC728875	NR_024584	chr1:143011874-143053112	non-template	39	Intron_2	--	Yes	--	Yes	--	--	--	--	--	--	--
G4ST01143051064	chr1:143051065-143051094	+	GGGGAGGCCTGGCCGGGCGGGTGGCGAGGG<br>..(((...)))	LOC728855	NR_024510	chr1:143011869-143052130	template	30	Intron_1.u	--	Yes	--	Yes	--	--	--	--	--	--	Yes
G4ST01143051064	chr1:143051065-143051094	+	GGGGAGGCCTGGCCGGGCGGGTGGCGAGGG<br>..(((...)))	LOC728855	NR_024511	chr1:143011869-143052130	template	30	Intron_1.f	--	Yes	--	Yes	--	--	--	--	--	--	Yes
G4ST01143051064	chr1:143051065-143051094	+	GGGGAGGCCTGGCCGGGCGGGTGGCGAGGG<br>..(((...)))	LOC728875	NR_024584	chr1:143011874-143053112	template	30	Intron_2	--	Yes	--	Yes	--	--	--	--	--	--	Yes
G4ST01143200777	chr1:143200778-143200816	-	GGGCACCTACTAGGTGCTAGGGTGGCTCTGGGCGAAGGG<br>(((((...)))))...	LOC728875	NR_024584	chr1:143192103-143233326	non-template	39	Intron_2	--	Yes	--	Yes	--	--	--	--	--	--	--
G4ST01143231313	chr1:143231314-143231343	+	GGGGAGGCCTGGCCGGGCGGGAGGCGAGGG<br>..(((...)))	LOC728875	NR_024584	chr1:143192103-143233326	template	30	Intron_2	--	Yes	--	Yes	--	--	--	--	Yes	--	Yes
G4ST01143574285	chr1:143574286-143574321	+	GGGTCAGGGTGGGAGCGGACTGCCTGCTGCATTGGG<br>(((((.....))))).....	PDE4DIP	NM_001198834	chr1:143562781-143706390	template	36	Intron_37	--	Yes	--	Yes	--	--	--	--	--	--	Yes
G4ST01143574285	chr1:143574286-143574321	+	GGGTCAGGGTGGGAGCGGACTGCCTGCTGCATTGGG<br>(((((.....))))).....	PDE4DIP	NM_001198832	chr1:143562781-143751349	template	36	Intron_39	--	Yes	--	Yes	--	--	--	--	--	--	Yes
G4ST01143574285	chr1:143574286-143574321	+	GGGTCAGGGTGGGAGCGGACTGCCTGCTGCATTGGG<br>(((((.....))))).....	PDE4DIP	NM_014644	chr1:143562784-143706379	template	36	Intron_37	--	Yes	--	Yes	--	--	--	--	--	--	Yes
G4ST01143574291	chr1:143574292-143574330	+	GGGTGGGAGCGGACTGCCTGCTGCATTGGGCTGAATGGG<br>(((((.....))))).....	PDE4DIP	NM_001198834	chr1:143562781-143706390	template	39	Intron_37	--	Yes	--	Yes	--	--	--	--	--	--	Yes
G4ST01143574291	chr1:143574292-143574330	+	GGGTGGGAGCGGACTGCCTGCTGCATTGGGCTGAATGGG<br>(((((.....))))).....	PDE4DIP	NM_001198832	chr1:143562781-143751349	template	39	Intron_39	--	Yes	--	Yes	--	--	--	--	--	--	Yes
G4ST01143574291	chr1:143574292-143574330	+	GGGTGGGAGCGGACTGCCTGCTGCATTGGGCTGAATGGG<br>(((((.....))))).....	PDE4DIP	NM_014644	chr1:143562784-143706379	template	39	Intron_37	--	Yes	--	Yes	--	--	--	--	--	--	Yes
G4ST01143637289	chr1:143637290-143637321	+	GGGCTAGAGCATAGTGGGCAAAGGGGACAGGG<br>(((.....))).	PDE4DIP	NM_001002811	chr1:143601948-143643389	template	32	Intron_1.f	--	Yes	--	Yes	--	--	--	Yes	--	--	--
G4ST01143637289	chr1:143637290-143637321	+	GGGCTAGAGCATAGTGGGCAAAGGGGACAGGG<br>(((.....))).	PDE4DIP	NM_001198834	chr1:143562781-143706390	template	32	Intron_5	--	Yes	--	Yes	--	--	--	Yes	--	--	--
G4ST01143637289	chr1:143637290-143637321	+	GGGCTAGAGCATAGTGGGCAAAGGGGACAGGG<br>(((.....))).	PDE4DIP	NM_001198832	chr1:143562781-143751349	template	32	Intron_8	--	Yes	--	Yes	--	--	--	Yes	--	--	--
G4ST01143637289	chr1:143637290-143637321	+	GGGCTAGAGCATAGTGGGCAAAGGGGACAGGG<br>(((.....))).	PDE4DIP	NM_014644	chr1:143562784-143706379	template	32	Intron_5	--	Yes	--	Yes	--	--	--	Yes	--	--	--
G4ST01143637289	chr1:143637290-143637321	+	GGGCTAGAGCATAGTGGGCAAAGGGGACAGGG<br>(((.....))).	PDE4DIP	NM_001002812	chr1:143601948-143706379	template	32	Intron_5	--	Yes	--	Yes	--	--	--	Yes	--	--	--
G4ST01143705901	chr1:143705902-143705938	-	GGGGCCCGCTTCTCCGAGGCAGGGGCAGGGGCAGGGG<br>(((((......)).)))..	PDE4DIP	NM_001002810	chr1:143663118-143706390	non-template	37	Intron_1.f	--	Yes	--	Yes	--	--	--	Yes	Yes	Yes	--
G4ST01143705901	chr1:143705902-143705938	-	GGGGCCCGCTTCTCCGAGGCAGGGGCAGGGGCAGGGG<br>(((((......)).)))..	PDE4DIP	NM_001195260	chr1:143663118-143708468	non-template	37	Intron_2	--	Yes	--	Yes	--	--	--	Yes	Yes	Yes	--
G4ST01143705901	chr1:143705902-143705938	-	GGGGCCCGCTTCTCCGAGGCAGGGGCAGGGGCAGGGG<br>(((((......)).)))..	PDE4DIP	NM_001195261	chr1:143663118-143706439	non-template	37	Intron_2	--	Yes	--	Yes	--	--	--	Yes	Yes	Yes	--
G4ST01143705901	chr1:143705902-143705938	-	GGGGCCCGCTTCTCCGAGGCAGGGGCAGGGGCAGGGG<br>(((((......)).)))..	PDE4DIP	NM_022359	chr1:143663118-143787543	non-template	37	Intron_4	--	Yes	--	Yes	--	--	--	Yes	Yes	Yes	--
G4ST01143705901	chr1:143705902-143705938	-	GGGGCCCGCTTCTCCGAGGCAGGGGCAGGGGCAGGGG<br>(((((......)).)))..	PDE4DIP	NM_001198834	chr1:143562781-143706390	non-template	37	Intron_1.f	--	Yes	--	Yes	--	--	--	Yes	Yes	Yes	--
G4ST01143705901	chr1:143705902-143705938	-	GGGGCCCGCTTCTCCGAGGCAGGGGCAGGGGCAGGGG<br>(((((......)).)))..	PDE4DIP	NM_001198832	chr1:143562781-143751349	non-template	37	Intron_4	--	Yes	--	Yes	--	--	--	Yes	Yes	Yes	--
G4ST01143705901	chr1:143705902-143705938	-	GGGGCCCGCTTCTCCGAGGCAGGGGCAGGGGCAGGGG<br>(((((......)).)))..	PDE4DIP	NM_014644	chr1:143562784-143706379	non-template	37	Intron_1.f	--	Yes	--	Yes	--	--	--	Yes	Yes	Yes	--
G4ST01143705901	chr1:143705902-143705938	-	GGGGCCCGCTTCTCCGAGGCAGGGGCAGGGGCAGGGG<br>(((((......)).)))..	PDE4DIP	NM_001002812	chr1:143601948-143706379	non-template	37	Intron_1.f	--	Yes	--	Yes	--	--	--	Yes	Yes	Yes	--
G4ST01143807514	chr1:143807515-143807540	-	GGGAGAGAGCTGGGAAGGAGGGTGGG<br>((....))	SEC22B	NM_004892	chr1:143807764-143828279	template	-223	upstream	Yes	--	--	--	--	--	--	--	Yes	Yes	Yes
G4ST01144185784	chr1:144185785-144185819	-	GGGAGCCGAGCCGGCTGGAGGGAGGGTCAACGGGG<br>(((((...)))))...	ANKRD34A	NM_001039888	chr1:144181865-144187004	template	35	Exon_4.l	--	Yes	Yes	--	Yes	--	--	Yes	Yes	Yes	--
G4ST01144186198	chr1:144186199-144186232	+	GGGTGGGGATTAGGACCTTAATGGGATAGGTGGG<br>.(((((....)))))	ANKRD34A	NM_001039888	chr1:144181865-144187004	non-template	34	Exon_4.l	--	Yes	Yes	--	--	--	Yes	--	Yes	Yes	--
G4ST01144188696	chr1:144188697-144188736	-	GGGCTGCCGGCGGCGCCGGGGGGCAGGGGTAGTCCTGGGG<br>...(((((...))))).	LIX1L	NM_153713	chr1:144188442-144210448	template	40	Exon_1.f	--	Yes	Yes	--	Yes	--	--	Yes	Yes	Yes	--
G4ST01144249223	chr1:144249224-144249253	+	GGGCAGAGGCTGGGATCTGAGGGTCCTGGG<br>.((...))	ITGA10	NM_003637	chr1:144236347-144255225	non-template	30	Intron_21	--	Yes	--	Yes	--	--	--	--	--	--	Yes
G4ST01144268193	chr1:144268194-144268232	-	GGGAGTGGGGGTGAGGTGTGCCCAGCTTACGGGGAGGGG<br>.(((((.((....)).)))))).	ANKRD35	NM_144698	chr1:144260566-144279883	template	39	Intron_3	--	Yes	--	Yes	--	--	--	Yes	Yes	--	--
G4ST01144289676	chr1:144289677-144289710	-	GGGTGCCAGGGGCCAACAGCGTTGGGGGAATGGG<br>.(((((...)))))	PIAS3	NM_006099	chr1:144287345-144297903	template	34	Exon_2	--	Yes	Yes	--	Yes	--	--	--	--	--	--
G4ST01144289682	chr1:144289683-144289721	-	GGGGCCCAGCAGGGTGCCAGGGGCCAACAGCGTTGGGGG<br>.(((((...)))))	PIAS3	NM_006099	chr1:144287345-144297903	template	39	Exon_2	--	Yes	Yes	--	Yes	--	--	--	--	--	--
G4ST01144424803	chr1:144424804-144424830	+	GGGACTGGTGCAGCGGGATGGGAGGGG<br>.(((...))).	CD160	NM_007053	chr1:144407155-144426922	template	27	Intron_1.f	--	Yes	--	Yes	--	--	--	--	Yes	--	Yes
G4ST01144460140	chr1:144460141-144460176	+	GGGGGAGGGACACTGGACAGCAGTGGGGGTGGGGGG<br>.(((((.....))))).	PDZK1	NM_002614	chr1:144439023-144475563	non-template	36	Intron_4	--	Yes	--	Yes	--	--	--	--	--	--	--
G4ST01144460140	chr1:144460141-144460176	+	GGGGGAGGGACACTGGACAGCAGTGGGGGTGGGGGG<br>.(((((.....))))).	PDZK1	NM_001201325	chr1:144439023-144475563	non-template	36	Intron_3	--	Yes	--	Yes	--	--	--	--	--	--	--
G4ST01144460140	chr1:144460141-144460176	+	GGGGGAGGGACACTGGACAGCAGTGGGGGTGGGGGG<br>.(((((.....))))).	PDZK1	NM_001201326	chr1:144439023-144475563	non-template	36	Intron_3	--	Yes	--	Yes	--	--	--	--	--	--	--
G4ST01144460146	chr1:144460147-144460183	+	GGGACACTGGACAGCAGTGGGGGTGGGGGGCACTGGG<br>.(((((.....))))).	PDZK1	NM_002614	chr1:144439023-144475563	non-template	37	Intron_4	--	Yes	--	Yes	--	--	--	--	--	--	--
G4ST01144460146	chr1:144460147-144460183	+	GGGACACTGGACAGCAGTGGGGGTGGGGGGCACTGGG<br>.(((((.....))))).	PDZK1	NM_001201325	chr1:144439023-144475563	non-template	37	Intron_3	--	Yes	--	Yes	--	--	--	--	--	--	--
G4ST01144460146	chr1:144460147-144460183	+	GGGACACTGGACAGCAGTGGGGGTGGGGGGCACTGGG<br>.(((((.....))))).	PDZK1	NM_001201326	chr1:144439023-144475563	non-template	37	Intron_3	--	Yes	--	Yes	--	--	--	--	--	--	--

G4ST01144538215	chr1:144538216-144538249	+	GGGTGTCGGGGCGGGAGGCGGTGACTCACCTGGG<br>....((((...)))).	GPR89A	NR_036541	chr1:144475952-144538460	template	34	Exon_1.f-Intron_1.f	--	Yes	Yes	Yes	--	--	--	Yes	Yes	Yes	--
G4ST01144538215	chr1:144538216-144538249	+	GGGTGTCGGGGCGGGAGGCGGTGACTCACCTGGG<br>....((((...)))).	GPR89A	NM_001097612	chr1:144475952-144538460	template	34	Exon_1.f-Intron_1.f	--	Yes	Yes	Yes	Yes	--	--	Yes	Yes	Yes	--
G4ST01144538215	chr1:144538216-144538249	+	GGGTGTCGGGGCGGGAGGCGGTGACTCACCTGGG<br>....((((...)))).	GPR89A	NM_001097613	chr1:144475952-144538460	template	34	Exon_1.f-Intron_1.f	--	Yes	Yes	Yes	--	Yes	--	Yes	Yes	Yes	--
G4ST01144650984	chr1:144650985-144651021	-	GGGACACTGGACAGCAGTGGGGGTGGGGGGCACTGGG<br>.(((((.....))))).	PDZK1P1	NR_003377	chr1:144635745-144653976	non-template	37	Intron_3	--	Yes	--	Yes	--	--	--	--	--	--	--
G4ST01144650991	chr1:144650992-144651027	-	GGGGGAGGGACACTGGACAGCAGTGGGGGTGGGGGG<br>.(((((.....))))).	PDZK1P1	NR_003377	chr1:144635745-144653976	non-template	36	Intron_3	--	Yes	--	Yes	--	--	--	--	--	--	--
G4ST01144788129	chr1:144788130-144788183	+	GGGCCAGGCCGGGCGGCGTTGTTGGCGGGGGCCCCGGTGGAGGCCCGGCCCGGG<br>((.((....)).)). ((((..((((....))).)))).	NBPF11	NM_183372	chr1:144743899-144793990	template	54	Intron_1.f	--	Yes	--	Yes	--	--	--	--	--	--	--
G4ST01144788927	chr1:144788928-144788968	-	GGGGTTCCCAGGGAGCCTGGCTCCACAGGCCAGGGTGTGGG<br>.(((((......)))))..	NBPF11	NM_183372	chr1:144743899-144793990	non-template	41	Intron_1.f	--	Yes	--	Yes	--	--	--	--	--	--	--
G4ST01144793405	chr1:144793406-144793442	-	GGGGCGGTTGCGCCGGGCACTGCCGGGCTCCGCAGGG<br>(((....))).	NBPF11	NM_183372	chr1:144743899-144793990	non-template	37	Intron_1.f	--	Yes	--	Yes	--	--	--	--	--	--	Yes
G4ST01145135688	chr1:145135689-145135724	+	GGGAGGGGCCTGGTGGGAGGTGATTTAATCATGGGG<br>..((((.....)))).	FMO5	NM_001144830	chr1:145124462-145163854	template	36	Intron_5	--	Yes	--	Yes	--	--	--	--	Yes	--	--
G4ST01145135688	chr1:145135689-145135724	+	GGGAGGGGCCTGGTGGGAGGTGATTTAATCATGGGG<br>..((((.....)))).	FMO5	NM_001461	chr1:145124462-145163854	template	36	Intron_7	--	Yes	--	Yes	--	--	--	--	Yes	--	--
G4ST01145135688	chr1:145135689-145135724	+	GGGAGGGGCCTGGTGGGAGGTGATTTAATCATGGGG<br>..((((.....)))).	FMO5	NM_001144829	chr1:145122508-145163854	template	36	Intron_7	--	Yes	--	Yes	--	--	--	--	Yes	--	--
G4ST01145483088	chr1:145483089-145483123	-	GGGAGGGGTAGGATATGTGTCCTTGCTGGGAGGGG<br>..(((((....)))))....	BCL9	NM_004326	chr1:145479806-145564639	template	35	Intron_1.f	--	Yes	--	Yes	--	--	--	--	--	--	--
G4ST01145867310	chr1:145867311-145867344	-	GGGTGTCGGGGCGGGAGGCGGTGACTCACCTGGG<br>....((((...)))).	GPR89B	NM_016334	chr1:145867130-145932377	template	34	Exon_1.f-Intron_1.f	--	Yes	Yes	Yes	Yes	--	--	Yes	Yes	Yes	--
G4ST01145947962	chr1:145947963-145947999	-	GGGACACTGGACAGCAGTGGGGGTGGGGGGCACTGGG<br>.(((((.....))))).	PDZK1P1	NR_003377	chr1:145932718-145950955	non-template	37	Intron_3	--	Yes	--	Yes	--	--	--	--	--	--	--
G4ST01145947969	chr1:145947970-145948005	-	GGGGGAGGGACACTGGACAGCAGTGGGGGTGGGGGG<br>.(((((.....))))).	PDZK1P1	NR_003377	chr1:145932718-145950955	non-template	36	Intron_3	--	Yes	--	Yes	--	--	--	--	--	--	--
G4ST01146085232	chr1:146085233-146085286	+	GGGCCAGGCCGGGCGGCGTTGTTGGCGGGGGCCCCGGTGGAGGCCCGGCCCGGG<br>((.((....)).)). ((((..((((....))).)))).	NBPF11	NM_183372	chr1:146040947-146091093	template	54	Intron_1.f	--	Yes	--	Yes	--	--	--	--	--	--	--
G4ST01146086030	chr1:146086031-146086071	-	GGGGTTCCCAGGGAGCCTGGCTCCACAGGCCAGGGTGTGGG<br>.(((((......)))))..	NBPF11	NM_183372	chr1:146040947-146091093	non-template	41	Intron_1.f	--	Yes	--	Yes	--	--	--	--	--	--	--
G4ST01146090508	chr1:146090509-146090545	-	GGGGCGGTTGCGCCGGGCACTGCCGGGCTCCGCAGGG<br>(((....))).	NBPF11	NM_183372	chr1:146040947-146091093	non-template	37	Intron_1.f	--	Yes	--	Yes	--	--	--	--	--	--	Yes
G4ST01146343617	chr1:146343618-146343656	+	GGGCACCTACTAGGTGCTAGGGTGGCTCTGGGCGAAGGG<br>(((((...)))))...	LOC100130000	NR_033189	chr1:146341263-146368313	non-template	39	Intron_1.f	--	Yes	--	Yes	--	--	--	--	--	--	--
G4ST01146397529	chr1:146397530-146397559	+	GGGGAGGCCTGGCCGGGCGGGTGGCGAGGG<br>..(((...)))	FLJ39739	NR_027468	chr1:146372646-146398604	template	30	Intron_1.f	--	Yes	--	Yes	--	--	--	--	--	--	Yes
G4ST01147197732	chr1:147197733-147197760	-	GGGGTGGGGTGGGGGCCTCTGGCCTGGG<br>((((...)))).	LOC645166	NR_027355	chr1:147194910-147218219	template	28	Intron_1.f	--	Yes	--	Yes	--	--	--	Yes	Yes	Yes	--
G4ST01147197732	chr1:147197733-147197760	-	GGGGTGGGGTGGGGGCCTCTGGCCTGGG<br>((((...)))).	LOC645166	NR_027356	chr1:147194910-147218219	template	28	Intron_1.f	--	Yes	--	Yes	--	--	--	Yes	Yes	Yes	--
G4ST01147197732	chr1:147197733-147197760	-	GGGGTGGGGTGGGGGCCTCTGGCCTGGG<br>((((...)))).	LOC645166	NR_027354	chr1:147197029-147219678	template	28	Intron_1.f	--	Yes	--	Yes	--	--	--	Yes	Yes	Yes	--
G4ST01147553116	chr1:147553117-147553148	-	GGGGGTGAGAGGGCGCGTGGTTGCGGGGCGGG<br>((((....))))	LOC388692	NR_027002	chr1:147546100-147558366	template	32	Exon_1.u	--	Yes	Yes	--	--	--	--	--	--	--	--
G4ST01147844116	chr1:147844117-147844142	-	GGGGAGGCCTGGCCGGGCGGGAGGGG<br>..(((...)))	LOC728855	NR_024510	chr1:147843077-147883410	template	26	Intron_1.u	--	Yes	--	Yes	--	--	--	Yes	Yes	--	Yes
G4ST01147874689	chr1:147874690-147874728	+	GGGCACCTACTAGGTGCTAGGGTGGCTCTGGGTGAAGGG<br>(((((...)))))...	LOC728855	NR_024510	chr1:147843077-147883410	non-template	39	Intron_1.u	--	Yes	--	Yes	--	--	--	--	--	--	--
G4ST01148127628	chr1:148127629-148127660	-	GGGAGGATCGCTTGAACCCGGGTGGGTGGGGG<br>.((.((....)).)).	HIST2H2AB	NM_175065	chr1:148125643-148126090	non-template	-1538	upstream	Yes	--	--	--	--	--	--	--	Yes	--	Yes
G4ST01148141633	chr1:148141634-148141670	+	GGGCTACGTCCAGCATGTGGGGAGTGAGGGACAAGGG<br>..((((.....)))).	SV2A	NM_014849	chr1:148141496-148156058	template	37	Exon_13.l	--	Yes	Yes	--	--	--	Yes	--	--	--	Yes
G4ST01148151155	chr1:148151156-148151182	-	GGGAGGGCAGCTGGACTGGGGCTTGGG<br>(((.....)))	SV2A	NM_014849	chr1:148141496-148156058	non-template	27	Intron_2	--	Yes	--	Yes	--	--	--	--	--	--	--
G4ST01148151161	chr1:148151162-148151191	-	GGGGCTTGCGGGAGGGCAGCTGGACTGGGG<br>(((.....)))	SV2A	NM_014849	chr1:148141496-148156058	non-template	30	Intron_2	--	Yes	--	Yes	--	--	--	--	--	--	--
G4ST01148151277	chr1:148151278-148151306	+	GGGAACATCTTGGGTGCAGGGTCCTAGGG<br>((....))	SV2A	NM_014849	chr1:148141496-148156058	template	29	Intron_2	--	Yes	--	Yes	--	--	--	--	--	--	--
G4ST01148154780	chr1:148154781-148154808	-	GGGAGGGTGGGGGGTTCTGGACCCAGGG<br>((((.....)))).	SV2A	NM_014849	chr1:148141496-148156058	non-template	28	Intron_1.f	--	Yes	--	Yes	--	--	--	--	--	--	--
G4ST01148175267	chr1:148175268-148175297	+	GGGAGAGGGGTAGGGGGTTGGACACAAGGG<br>..(((....)))	MTMR11	NM_001145862	chr1:148167167-148175415	template	30	Exon_1.f	--	Yes	Yes	--	--	Yes	--	Yes	Yes	--	Yes
G4ST01148175267	chr1:148175268-148175297	+	GGGAGAGGGGTAGGGGGTTGGACACAAGGG<br>..(((....)))	MTMR11	NM_181873	chr1:148167167-148174890	template	-377	upstream	Yes	--	--	--	--	--	--	Yes	Yes	--	Yes
G4ST01148188360	chr1:148188361-148188394	-	GGGAGGGACTCAGTTGGCAGAGTGGGATGTAGGG<br>((((........))))	OTUD7B	NM_020205	chr1:148178856-148249310	non-template	34	Intron_8	--	Yes	--	Yes	--	--	--	--	--	--	--
G4ST01148227143	chr1:148227144-148227175	+	GGGAAGTAATCTTTGGGAAAATGGGGGCAGGG<br>(((....))).	OTUD7B	NM_020205	chr1:148178856-148249310	template	32	Intron_1.f	--	Yes	--	Yes	--	--	--	Yes	--	--	--
G4ST01148249026	chr1:148249027-148249068	+	GGGCTCCCGGGGCAAGGCCCTAGGCCGGGGCGGAGCGCGGGG<br>....((((...)))). (((...))).	OTUD7B	NM_020205	chr1:148178856-148249310	template	42	Exon_1.f	--	Yes	Yes	--	--	Yes	--	--	Yes	Yes	--
G4ST01148388472	chr1:148388473-148388507	+	GGGCCCCGGGGCGGCCGGCGGCCGGGTCTCTCGGG<br>..((((...))))	PLEKHO1	NM_016274	chr1:148388794-148398449	non-template	-286	upstream	Yes	--	--	--	--	--	--	Yes	Yes	Yes	--
G4ST01148388739	chr1:148388740-148388769	+	GGGCCGGGGGAGCGGCCGCGCTGGGCCGGG<br>..((((....))))	PLEKHO1	NM_016274	chr1:148388794-148398449	non-template	-24	upstream	Yes	--	--	--	--	--	--	Yes	Yes	Yes	Yes
G4ST01148388744	chr1:148388745-148388773	+	GGGGGAGCGGCCGCGCTGGGCCGGGCGGG<br>..((((....))))	PLEKHO1	NM_016274	chr1:148388794-148398449	non-template	-20	upstream	Yes	--	--	--	--	--	--	Yes	Yes	Yes	Yes
G4ST01148388933	chr1:148388934-148388967	+	GGGAGGGCGCGAGGGAGGAGCGGCGGCGCCGGGG<br>.....((((...))))	PLEKHO1	NM_016274	chr1:148388794-148398449	non-template	34	Exon_1.f	--	Yes	Yes	--	--	Yes	--	Yes	Yes	Yes	Yes
G4ST01148389774	chr1:148389775-148389810	+	GGGTCCGGAAATTCTGCGGGAAAGGGATTTTCAGGG<br>..((((...)))).	PLEKHO1	NM_016274	chr1:148388794-148398449	non-template	36	Exon_2	--	Yes	Yes	--	Yes	--	--	Yes	Yes	Yes	--
G4ST01148474196	chr1:148474197-148474230	-	GGGCGTCCGACGTCGCGACGGGCGCGGGCTGGGG<br>.(((((....)).)))	ANP32E	NM_001136479	chr1:148457341-148473650	non-template	-546	upstream	Yes	--	--	--	--	--	--	Yes	Yes	Yes	--
G4ST01148474196	chr1:148474197-148474230	-	GGGCGTCCGACGTCGCGACGGGCGCGGGCTGGGG<br>.(((((....)).)))	ANP32E	NM_001136478	chr1:148457341-148475128	non-template	34	Intron_1.f	--	Yes	--	Yes	--	--	--	Yes	Yes	Yes	--
G4ST01148474196	chr1:148474197-148474230	-	GGGCGTCCGACGTCGCGACGGGCGCGGGCTGGGG<br>.(((((....)).)))	ANP32E	NM_030920	chr1:148457341-148475128	non-template	34	Intron_1.f	--	Yes	--	Yes	--	--	--	Yes	Yes	Yes	--
G4ST01148737573	chr1:148737574-148737615	-	GGGCGAACATCAGGCTGAGGTTGGGGTGCAGAGGGGTCAGGG<br>..(((.(((...))).))).	TARS2	NM_025150	chr1:148726544-148746373	template	42	Intron_10-Exon_11	--	Yes	Yes	Yes	Yes	--	--	--	--	--	Yes
G4ST01148737579	chr1:148737580-148737623	-	GGGCCGGTGGGCGAACATCAGGCTGAGGTTGGGGTGCAGAGGGG<br>..(((.(((...))).))).	TARS2	NM_025150	chr1:148726544-148746373	template	44	Intron_10-Exon_11	--	Yes	Yes	Yes	Yes	--	--	--	--	--	Yes
G4ST01148737589	chr1:148737590-148737645	-	GGGGCAGTTCCCGCCAGGATCTGGGCCGGTGGGCGAACATCAGGCTGAGGTTGGGG<br>..((.(((.....))).)) ..(((.(((...))).))).	TARS2	NM_025150	chr1:148726544-148746373	template	56	Intron_10-Exon_11	--	Yes	Yes	Yes	Yes	--	--	--	--	--	Yes

G4ST01148747405	chr1:148747406-148747437	+	GGGGGTCTGGGCTTGCATGGCAGGGGTCTGGG<br>..(((...))).	ECM1	NM_022664	chr1:148747111-148752889	non-template	32	Intron_1.f	--	Yes	--	Yes	--	--	--	--	Yes	Yes	--
G4ST01148747405	chr1:148747406-148747437	+	GGGGGTCTGGGCTTGCATGGCAGGGGTCTGGG<br>..(((...))).	ECM1	NM_004425	chr1:148747111-148752889	non-template	32	Intron_1.f	--	Yes	--	Yes	--	--	--	--	Yes	Yes	--
G4ST01148747405	chr1:148747406-148747437	+	GGGGGTCTGGGCTTGCATGGCAGGGGTCTGGG<br>..(((...))).	ECM1	NM_001202858	chr1:148747111-148752889	non-template	32	Intron_1.f	--	Yes	--	Yes	--	--	--	--	Yes	Yes	--
G4ST01148748348	chr1:148748349-148748377	-	GGGAGTATGGCATACGGGATTGGGGAGGG<br>.(((.....)))	ECM1	NM_022664	chr1:148747111-148752889	template	29	Intron_1.f	--	Yes	--	Yes	--	--	--	Yes	--	--	--
G4ST01148748348	chr1:148748349-148748377	-	GGGAGTATGGCATACGGGATTGGGGAGGG<br>.(((.....)))	ECM1	NM_004425	chr1:148747111-148752889	template	29	Intron_1.f	--	Yes	--	Yes	--	--	--	Yes	--	--	--
G4ST01148748348	chr1:148748349-148748377	-	GGGAGTATGGCATACGGGATTGGGGAGGG<br>.(((.....)))	ECM1	NM_001202858	chr1:148747111-148752889	template	29	Intron_1.f	--	Yes	--	Yes	--	--	--	Yes	--	--	--
G4ST01148748352	chr1:148748353-148748386	-	GGGCCTAAAGGGAGTATGGCATACGGGATTGGGG<br>.(((.....)))	ECM1	NM_022664	chr1:148747111-148752889	template	34	Intron_1.f	--	Yes	--	Yes	--	--	--	Yes	--	--	--
G4ST01148748352	chr1:148748353-148748386	-	GGGCCTAAAGGGAGTATGGCATACGGGATTGGGG<br>.(((.....)))	ECM1	NM_004425	chr1:148747111-148752889	template	34	Intron_1.f	--	Yes	--	Yes	--	--	--	Yes	--	--	--
G4ST01148748352	chr1:148748353-148748386	-	GGGCCTAAAGGGAGTATGGCATACGGGATTGGGG<br>.(((.....)))	ECM1	NM_001202858	chr1:148747111-148752889	template	34	Intron_1.f	--	Yes	--	Yes	--	--	--	Yes	--	--	--
G4ST01148792732	chr1:148792733-148792770	-	GGGGGGATGGGGTGTGGACAGACAGTTCTGTGGGAGGG<br>......(((((....)))))	ADAMTSL4	NM_025008	chr1:148788522-148797847	template	38	Exon_6	--	Yes	Yes	--	Yes	--	--	--	--	Yes	--
G4ST01148792732	chr1:148792733-148792770	-	GGGGGGATGGGGTGTGGACAGACAGTTCTGTGGGAGGG<br>......(((((....)))))	ADAMTSL4	NM_019032	chr1:148788522-148800036	template	38	Exon_6	--	Yes	Yes	--	Yes	--	--	--	--	Yes	--
G4ST01148792846	chr1:148792847-148792886	-	GGGTGAGGGGGGCTCTGTGCCAGAGGCCTGGGCTCTGGGG<br>((.(((((...))))).)).	ADAMTSL4	NM_025008	chr1:148788522-148797847	template	40	Exon_6	--	Yes	Yes	--	Yes	--	--	Yes	Yes	Yes	--
G4ST01148792846	chr1:148792847-148792886	-	GGGTGAGGGGGGCTCTGTGCCAGAGGCCTGGGCTCTGGGG<br>((.(((((...))))).)).	ADAMTSL4	NM_019032	chr1:148788522-148800036	template	40	Exon_6	--	Yes	Yes	--	Yes	--	--	Yes	Yes	Yes	--
G4ST01148795901	chr1:148795902-148795932	+	GGGGCCAGGGGCAGCTGAATGCTGGGGAGGG<br>.((((.....))))	ADAMTSL4	NM_025008	chr1:148788522-148797847	non-template	31	Intron_10	--	Yes	--	Yes	--	--	--	--	--	Yes	--
G4ST01148795901	chr1:148795902-148795932	+	GGGGCCAGGGGCAGCTGAATGCTGGGGAGGG<br>.((((.....))))	ADAMTSL4	NM_019032	chr1:148788522-148800036	non-template	31	Intron_10	--	Yes	--	Yes	--	--	--	--	--	Yes	--
G4ST01149213514	chr1:149213515-149213561	-	GGGGGGCGCTGCGGGCGCTCATGGCGGCGGCCGCCGGGTAAGGCGGG<br>.......(((((...)))))	LASS2	NM_181746	chr1:149204273-149214064	non-template	47	Intron_1.f	--	Yes	--	Yes	--	--	--	--	Yes	Yes	--
G4ST01149213514	chr1:149213515-149213561	-	GGGGGGCGCTGCGGGCGCTCATGGCGGCGGCCGCCGGGTAAGGCGGG<br>.......(((((...)))))	LASS2	NM_022075	chr1:149204273-149214064	non-template	47	Intron_1.f	--	Yes	--	Yes	--	--	--	--	Yes	Yes	--
G4ST01149224389	chr1:149224390-149224431	-	GGGATGGCAAGGGGCTGGGAAGGTGACATGAGTACCTTTGGG<br>(((((.........))))).	ANXA9	NM_003568	chr1:149221123-149234738	template	42	Intron_7	--	Yes	--	Yes	--	--	--	Yes	Yes	--	--
G4ST01149371849	chr1:149371850-149371879	+	GGGGAGCGGGGGCGGAGAGCGCGGGCCGGG<br>.(((.....)))	SEMA6C	NM_001178061	chr1:149370787-149385764	template	30	Exon_20.l	--	Yes	Yes	--	Yes	--	--	--	--	--	--
G4ST01149371849	chr1:149371850-149371879	+	GGGGAGCGGGGGCGGAGAGCGCGGGCCGGG<br>.(((.....)))	SEMA6C	NM_001178062	chr1:149370787-149385764	template	30	Exon_19.l	--	Yes	Yes	--	Yes	--	--	--	--	--	--
G4ST01149371849	chr1:149371850-149371879	+	GGGGAGCGGGGGCGGAGAGCGCGGGCCGGG<br>.(((.....)))	SEMA6C	NM_030913	chr1:149370787-149385764	template	30	Exon_19.l	--	Yes	Yes	--	Yes	--	--	--	--	--	--
G4ST01149371856	chr1:149371857-149371885	+	GGGGGCGGAGAGCGCGGGCCGGGCGGGGG<br>.(((.....)))	SEMA6C	NM_001178061	chr1:149370787-149385764	template	29	Exon_20.l	--	Yes	Yes	--	Yes	--	--	--	--	--	--
G4ST01149371856	chr1:149371857-149371885	+	GGGGGCGGAGAGCGCGGGCCGGGCGGGGG<br>.(((.....)))	SEMA6C	NM_001178062	chr1:149370787-149385764	template	29	Exon_19.l	--	Yes	Yes	--	Yes	--	--	--	--	--	--
G4ST01149371856	chr1:149371857-149371885	+	GGGGGCGGAGAGCGCGGGCCGGGCGGGGG<br>.(((.....)))	SEMA6C	NM_030913	chr1:149370787-149385764	template	29	Exon_19.l	--	Yes	Yes	--	Yes	--	--	--	--	--	--
G4ST01149384863	chr1:149384864-149384901	+	GGGCGGGCCTGAGGCCGGCGCTCAGTGGGGACCGTGGG<br>.(((((.......)))))..	SEMA6C	NM_001178061	chr1:149370787-149385764	template	38	Intron_1.f	--	Yes	--	Yes	--	--	--	Yes	--	--	--
G4ST01149384863	chr1:149384864-149384901	+	GGGCGGGCCTGAGGCCGGCGCTCAGTGGGGACCGTGGG<br>.(((((.......)))))..	SEMA6C	NM_001178062	chr1:149370787-149385764	template	38	Intron_1.f	--	Yes	--	Yes	--	--	--	Yes	--	--	--
G4ST01149384863	chr1:149384864-149384901	+	GGGCGGGCCTGAGGCCGGCGCTCAGTGGGGACCGTGGG<br>.(((((.......)))))..	SEMA6C	NM_030913	chr1:149370787-149385764	template	38	Intron_1.f	--	Yes	--	Yes	--	--	--	Yes	--	--	--
G4ST01149385663	chr1:149385664-149385695	+	GGGTTCCAGGGCGCTGTCGGGAGAGCCCTGGG<br>((....))	SEMA6C	NM_001178061	chr1:149370787-149385764	template	32	Exon_1.f	--	Yes	Yes	--	--	Yes	--	--	Yes	Yes	--
G4ST01149385663	chr1:149385664-149385695	+	GGGTTCCAGGGCGCTGTCGGGAGAGCCCTGGG<br>((....))	SEMA6C	NM_001178062	chr1:149370787-149385764	template	32	Exon_1.f	--	Yes	Yes	--	--	Yes	--	--	Yes	Yes	--
G4ST01149385663	chr1:149385664-149385695	+	GGGTTCCAGGGCGCTGTCGGGAGAGCCCTGGG<br>((....))	SEMA6C	NM_030913	chr1:149370787-149385764	template	32	Exon_1.f	--	Yes	Yes	--	--	Yes	--	--	Yes	Yes	--
G4ST01149415810	chr1:149415811-149415839	+	GGGCCGGGGCCCAGGGCTGAGGCAGTGGG<br>(((...))).	TMOD4	NM_013353	chr1:149409087-149415171	template	-639	upstream	Yes	--	--	--	--	--	--	--	--	--	--
G4ST01149415810	chr1:149415811-149415839	+	GGGCCGGGGCCCAGGGCTGAGGCAGTGGG<br>(((...))).	VPS72	NM_005997	chr1:149415558-149429264	template	29	Exon_6.l	--	Yes	Yes	--	Yes	--	--	--	--	--	--
G4ST01149503259	chr1:149503260-149503297	+	GGGGGATGAATCCCAGGGTAGTTGAGGGTTGTGAAGGG<br>(((....)))..	PSMD4	NM_002810	chr1:149493821-149506578	non-template	38	Intron_3	--	Yes	--	Yes	--	--	--	--	--	--	--
G4ST01149521273	chr1:149521274-149521308	+	GGGGCTGGAAGGGGCACAGGGCTGAGCGACGGGGG<br>(((.....)))	ZNF687	NM_020832	chr1:149521415-149531005	non-template	-106	upstream	Yes	--	--	--	--	--	--	Yes	Yes	Yes	--
G4ST01149521283	chr1:149521284-149521314	+	GGGGCACAGGGCTGAGCGACGGGGGCAAGGG<br>(((.....)))	ZNF687	NM_020832	chr1:149521415-149531005	non-template	-100	upstream	Yes	--	--	--	--	--	--	Yes	Yes	Yes	--
G4ST01149523424	chr1:149523425-149523457	+	GGGCTGGCTAGGGGAGGGCCAGGCACTCTGGGG<br>.(((.....)))	ZNF687	NM_020832	chr1:149521415-149531005	non-template	33	Intron_1.f	--	Yes	--	Yes	--	--	--	Yes	--	--	--
G4ST01149533903	chr1:149533904-149533946	-	GGGATAGTTTGGGTGCCAGAAGTGCAATTTCTGGGAAATTGGG<br>....((((((...))))))	PI4KB	NM_001198773	chr1:149530897-149566757	non-template	43	Intron_10	--	Yes	--	Yes	--	--	--	--	--	--	--
G4ST01149533903	chr1:149533904-149533946	-	GGGATAGTTTGGGTGCCAGAAGTGCAATTTCTGGGAAATTGGG<br>....((((((...))))))	PI4KB	NM_001198775	chr1:149530897-149566757	non-template	43	Intron_7	--	Yes	--	Yes	--	--	--	--	--	--	--
G4ST01149533903	chr1:149533904-149533946	-	GGGATAGTTTGGGTGCCAGAAGTGCAATTTCTGGGAAATTGGG<br>....((((((...))))))	PI4KB	NM_001198774	chr1:149530897-149566815	non-template	43	Intron_8	--	Yes	--	Yes	--	--	--	--	--	--	--
G4ST01149533903	chr1:149533904-149533946	-	GGGATAGTTTGGGTGCCAGAAGTGCAATTTCTGGGAAATTGGG<br>....((((((...))))))	PI4KB	NM_002651	chr1:149530897-149566757	non-template	43	Intron_10	--	Yes	--	Yes	--	--	--	--	--	--	--
G4ST01149586575	chr1:149586576-149586612	+	GGGGGAAGTCCGGGTTGGGTTTCGGTTAAAACTGGGG<br>...((((....))))	RFX5	NM_000449	chr1:149579740-149586393	template	-182	upstream	Yes	--	--	--	--	--	--	Yes	Yes	Yes	--
G4ST01149586575	chr1:149586576-149586612	+	GGGGGAAGTCCGGGTTGGGTTTCGGTTAAAACTGGGG<br>...((((....))))	RFX5	NM_001025603	chr1:149579740-149586393	template	-182	upstream	Yes	--	--	--	--	--	--	Yes	Yes	Yes	--
G4ST01149586586	chr1:149586587-149586621	+	GGGTTGGGTTTCGGTTAAAACTGGGGTCGGTTGGG<br>...((((....))))	RFX5	NM_000449	chr1:149579740-149586393	template	-193	upstream	Yes	--	--	--	--	--	--	Yes	Yes	Yes	--
G4ST01149586586	chr1:149586587-149586621	+	GGGTTGGGTTTCGGTTAAAACTGGGGTCGGTTGGG<br>...((((....))))	RFX5	NM_001025603	chr1:149579740-149586393	template	-193	upstream	Yes	--	--	--	--	--	--	Yes	Yes	Yes	--
G4ST01149603903	chr1:149603904-149603941	+	GGGTCTCAATGGGCCATCTGTGTGGTGGGGTACAGGGG<br>((((....))))..	SELENBP1	NM_003944	chr1:149603404-149611788	template	38	Intron_11.l	--	Yes	--	Yes	--	--	--	--	--	--	--
G4ST01149609114	chr1:149609115-149609143	+	GGGTGGGAGGAGGGCTGATGCAGCCGGGG<br>(((...)))...	SELENBP1	NM_003944	chr1:149603404-149611788	template	29	Intron_1.f	--	Yes	--	Yes	--	--	--	Yes	--	--	Yes
G4ST01149638916	chr1:149638917-149638956	+	GGGGACCCCGGGGGCGCGCGAACGGCAGGGGAGCTCAGGG<br>.((.((....))))..	PSMB4	NM_002796	chr1:149638665-149641036	non-template	40	Intron_1.f	--	Yes	--	Yes	--	--	--	Yes	Yes	Yes	--
G4ST01149667322	chr1:149667323-149667359	+	GGGAGTGGACTTGGTCTGCTGGGACTGGGACTGTGGG<br>(((((((...)))))))	POGZ	NM_207171	chr1:149641824-149681305	template	37	Exon_4	--	Yes	Yes	--	Yes	--	--	--	--	--	--
G4ST01149667322	chr1:149667323-149667359	+	GGGAGTGGACTTGGTCTGCTGGGACTGGGACTGTGGG<br>(((((((...)))))))	POGZ	NM_001194938	chr1:149641824-149698565	template	37	Exon_5	--	Yes	Yes	--	Yes	--	--	--	--	--	--
G4ST01149667322	chr1:149667323-149667359	+	GGGAGTGGACTTGGTCTGCTGGGACTGGGACTGTGGG<br>(((((((...)))))))	POGZ	NM_001194937	chr1:149641824-149698565	template	37	Exon_6	--	Yes	Yes	--	Yes	--	--	--	--	--	--

G4ST01149667322	chr1:149667323-149667359	+	GGGAGTGGACTTGGTCTGCTGGGACTGGGACTGTGGG<br>(((((((...)))))))	POGZ	NM_015100	chr1:149641824-149698565	template	37	Exon_6	--	Yes	Yes	--	Yes	--	--	--	--	--	--
G4ST01149667322	chr1:149667323-149667359	+	GGGAGTGGACTTGGTCTGCTGGGACTGGGACTGTGGG<br>(((((((...)))))))	POGZ	NM_145796	chr1:149641824-149698565	template	37	Exon_4	--	Yes	Yes	--	Yes	--	--	--	--	--	--
G4ST01149673466	chr1:149673467-149673511	+	GGGGGGCCGAGGTGGGAGGATCGTTTGAGCCTGGGAGGTGGAGGG<br>(((.((....)).)))	POGZ	NM_207171	chr1:149641824-149681305	template	45	Intron_1.f	--	Yes	--	Yes	--	--	--	--	--	--	--
G4ST01149673466	chr1:149673467-149673511	+	GGGGGGCCGAGGTGGGAGGATCGTTTGAGCCTGGGAGGTGGAGGG<br>(((.((....)).)))	POGZ	NM_001194938	chr1:149641824-149698565	template	45	Intron_2	--	Yes	--	Yes	--	--	--	--	--	--	--
G4ST01149673466	chr1:149673467-149673511	+	GGGGGGCCGAGGTGGGAGGATCGTTTGAGCCTGGGAGGTGGAGGG<br>(((.((....)).)))	POGZ	NM_001194937	chr1:149641824-149698565	template	45	Intron_3	--	Yes	--	Yes	--	--	--	--	--	--	--
G4ST01149673466	chr1:149673467-149673511	+	GGGGGGCCGAGGTGGGAGGATCGTTTGAGCCTGGGAGGTGGAGGG<br>(((.((....)).)))	POGZ	NM_015100	chr1:149641824-149698565	template	45	Intron_3	--	Yes	--	Yes	--	--	--	--	--	--	--
G4ST01149673466	chr1:149673467-149673511	+	GGGGGGCCGAGGTGGGAGGATCGTTTGAGCCTGGGAGGTGGAGGG<br>(((.((....)).)))	POGZ	NM_145796	chr1:149641824-149698565	template	45	Intron_3	--	Yes	--	Yes	--	--	--	--	--	--	--
G4ST01149759603	chr1:149759604-149759644	+	GGGAGCAAGGGTCAAGGTTAGAGCTTTGAGGGCCTCAGGGG<br>.((((((....)))))).	CGN	NM_020770	chr1:149750486-149777791	non-template	41	Intron_4	--	Yes	--	Yes	--	--	--	Yes	Yes	--	--
G4ST01149852699	chr1:149852700-149852727	-	GGGACTGCAGCAGGGGAGAGAGGGTGGG<br>.(((...)))	SNX27	NM_030918	chr1:149851286-149938183	template	28	Intron_1.f	--	Yes	--	Yes	--	--	--	Yes	--	--	--
G4ST01149852703	chr1:149852704-149852733	-	GGGGGAGGGACTGCAGCAGGGGAGAGAGGG<br>.(((...)))	SNX27	NM_030918	chr1:149851286-149938183	template	30	Intron_1.f	--	Yes	--	Yes	--	--	--	Yes	--	--	--
G4ST01149946779	chr1:149946780-149946808	-	GGGAGGGAGCCAGGCTTGGGCACAGGGGG<br>(((...))).	CELF3	NM_007185	chr1:149939158-149955914	non-template	29	Intron_6	--	Yes	--	Yes	--	--	--	--	--	--	--
G4ST01149946779	chr1:149946780-149946808	-	GGGAGGGAGCCAGGCTTGGGCACAGGGGG<br>(((...))).	CELF3	NM_001172649	chr1:149939158-149955506	non-template	29	Intron_6	--	Yes	--	Yes	--	--	--	--	--	--	--
G4ST01149946779	chr1:149946780-149946808	-	GGGAGGGAGCCAGGCTTGGGCACAGGGGG<br>(((...))).	CELF3	NM_001172648	chr1:149939158-149955416	non-template	29	Intron_6	--	Yes	--	Yes	--	--	--	--	--	--	--
G4ST01149946788	chr1:149946789-149946813	-	GGGCTGGGAGGGAGCCAGGCTTGGG<br>(((...))).	CELF3	NM_007185	chr1:149939158-149955914	non-template	25	Intron_6	--	Yes	--	Yes	--	--	--	--	--	--	--
G4ST01149946788	chr1:149946789-149946813	-	GGGCTGGGAGGGAGCCAGGCTTGGG<br>(((...))).	CELF3	NM_001172649	chr1:149939158-149955506	non-template	25	Intron_6	--	Yes	--	Yes	--	--	--	--	--	--	--
G4ST01149946788	chr1:149946789-149946813	-	GGGCTGGGAGGGAGCCAGGCTTGGG<br>(((...))).	CELF3	NM_001172648	chr1:149939158-149955416	non-template	25	Intron_6	--	Yes	--	Yes	--	--	--	--	--	--	--
G4ST01149948031	chr1:149948032-149948058	+	GGGCCAGGGCCTGGCAGGTGGGCAGGG<br>(((...))).	CELF3	NM_007185	chr1:149939158-149955914	template	27	Intron_5	--	Yes	--	Yes	--	--	--	--	--	--	--
G4ST01149948031	chr1:149948032-149948058	+	GGGCCAGGGCCTGGCAGGTGGGCAGGG<br>(((...))).	CELF3	NM_001172649	chr1:149939158-149955506	template	27	Intron_5	--	Yes	--	Yes	--	--	--	--	--	--	--
G4ST01149948031	chr1:149948032-149948058	+	GGGCCAGGGCCTGGCAGGTGGGCAGGG<br>(((...))).	CELF3	NM_001172648	chr1:149939158-149955416	template	27	Intron_5	--	Yes	--	Yes	--	--	--	--	--	--	--
G4ST01149948037	chr1:149948038-149948062	+	GGGCCTGGCAGGTGGGCAGGGTGGG<br>(((...))).	CELF3	NM_007185	chr1:149939158-149955914	template	25	Intron_5	--	Yes	--	Yes	--	--	--	--	--	--	--
G4ST01149948037	chr1:149948038-149948062	+	GGGCCTGGCAGGTGGGCAGGGTGGG<br>(((...))).	CELF3	NM_001172649	chr1:149939158-149955506	template	25	Intron_5	--	Yes	--	Yes	--	--	--	--	--	--	--
G4ST01149948037	chr1:149948038-149948062	+	GGGCCTGGCAGGTGGGCAGGGTGGG<br>(((...))).	CELF3	NM_001172648	chr1:149939158-149955416	template	25	Intron_5	--	Yes	--	Yes	--	--	--	--	--	--	--
G4ST01149948074	chr1:149948075-149948105	+	GGGGGCAGGGGAGTGGGGCTCACTGGCAGGG<br>(((....))).	CELF3	NM_007185	chr1:149939158-149955914	template	31	Exon_5-Intron_5	--	Yes	Yes	Yes	Yes	--	--	--	--	--	--
G4ST01149948074	chr1:149948075-149948105	+	GGGGGCAGGGGAGTGGGGCTCACTGGCAGGG<br>(((....))).	CELF3	NM_001172649	chr1:149939158-149955506	template	31	Exon_5-Intron_5	--	Yes	Yes	Yes	Yes	--	--	--	--	--	--
G4ST01149948074	chr1:149948075-149948105	+	GGGGGCAGGGGAGTGGGGCTCACTGGCAGGG<br>(((....))).	CELF3	NM_001172648	chr1:149939158-149955416	template	31	Exon_5-Intron_5	--	Yes	Yes	Yes	Yes	--	--	--	--	--	--
G4ST01149955504	chr1:149955505-149955533	+	GGGGAAAGGGCCTGAGGAGGGTGATGGGG<br>((...)).	CELF3	NM_007185	chr1:149939158-149955914	template	29	Exon_1.f	--	Yes	Yes	--	--	Yes	--	--	Yes	--	--
G4ST01149955504	chr1:149955505-149955533	+	GGGGAAAGGGCCTGAGGAGGGTGATGGGG<br>((...)).	CELF3	NM_001172649	chr1:149939158-149955506	template	2	upstream-Exon_1.f	Yes	Yes	Yes	--	--	Yes	--	--	Yes	--	--
G4ST01149955504	chr1:149955505-149955533	+	GGGGAAAGGGCCTGAGGAGGGTGATGGGG<br>((...)).	CELF3	NM_001172648	chr1:149939158-149955416	template	-88	upstream	Yes	--	--	--	--	--	--	--	Yes	--	--
G4ST01149967588	chr1:149967589-149967626	+	GGGGGGTGGGGGGTGGGGCCAGGCACAGCGTGGAGGGG<br>.(((.((...)).)))..	RIIAD1	NM_001144956	chr1:149960637-149968706	non-template	38	Intron_3	--	Yes	--	Yes	--	--	--	Yes	--	--	Yes
G4ST01149967595	chr1:149967596-149967644	+	GGGGGGTGGGGCCAGGCACAGCGTGGAGGGGAGGCAGCCAGCCTCTGGG<br>.(((.((...)).))).. (((((.....))))).	RIIAD1	NM_001144956	chr1:149960637-149968706	non-template	49	Intron_3	--	Yes	--	Yes	--	--	--	Yes	--	--	Yes
G4ST01150049234	chr1:150049235-150049267	-	GGGGATAATTGTTTTATGGGATTTGGGTGTGGG<br>.((((.....))))	RORC	NM_005060	chr1:150045171-150070972	non-template	33	Intron_10.l	--	Yes	--	Yes	--	--	--	--	--	--	Yes
G4ST01150049234	chr1:150049235-150049267	-	GGGGATAATTGTTTTATGGGATTTGGGTGTGGG<br>.((((.....))))	RORC	NM_001001523	chr1:150045171-150065177	non-template	33	Intron_9.l	--	Yes	--	Yes	--	--	--	--	--	--	Yes
G4ST01150066808	chr1:150066809-150066850	+	GGGCCTTCCTGGGGCTTCAGGAGCTGGGCCGTGGAGTGGGGG<br>(((.....))). (((.....)))	RORC	NM_005060	chr1:150045171-150070972	template	42	Intron_2	--	Yes	--	Yes	--	--	--	--	--	--	--
G4ST01150066808	chr1:150066809-150066850	+	GGGCCTTCCTGGGGCTTCAGGAGCTGGGCCGTGGAGTGGGGG<br>(((.....))). (((.....)))	RORC	NM_001001523	chr1:150045171-150065177	template	-1631	upstream	Yes	--	--	--	--	--	--	--	--	--	--
G4ST01150143823	chr1:150143824-150143856	+	GGGACTGAGGGATGTGGGGAGTTACTGACTGGG<br>.(((.....)))	THEM4	NM_053055	chr1:150112684-150148737	template	33	Intron_1.f	--	Yes	--	Yes	--	--	--	--	Yes	--	--
G4ST01150145514	chr1:150145515-150145554	-	GGGAGGCTCGGGCCGTGCAGGAGCCCACGGAGGGCGTGGG<br>(((((........))))).	THEM4	NM_053055	chr1:150112684-150148737	non-template	40	Intron_1.f	--	Yes	--	Yes	--	--	--	--	--	--	--
G4ST01150232633	chr1:150232634-150232664	+	GGGTCTGGGGGCGGCCGCGCCCGGGCCGGGG<br>((((....)))).	S100A10	NM_002966	chr1:150222010-150233338	template	31	Intron_1.f	--	Yes	--	Yes	--	--	--	Yes	Yes	Yes	--
G4ST01150232639	chr1:150232640-150232669	+	GGGGGCGGCCGCGCCCGGGCCGGGGAGGGG<br>((((....)))).	S100A10	NM_002966	chr1:150222010-150233338	template	30	Intron_1.f	--	Yes	--	Yes	--	--	--	Yes	Yes	Yes	--
G4ST01150233085	chr1:150233086-150233122	-	GGGCGAGGCCTCTGCGAGGCGGGTCCGGGAGCGAGGG<br>....(((((...)))))	S100A10	NM_002966	chr1:150222010-150233338	non-template	37	Exon_1.f	--	Yes	Yes	--	--	Yes	--	Yes	Yes	Yes	Yes
G4ST01150233093	chr1:150233094-150233127	-	GGGGCGGGCGAGGCCTCTGCGAGGCGGGTCCGGG<br>....(((((...)))))	S100A10	NM_002966	chr1:150222010-150233338	non-template	34	Exon_1.f	--	Yes	Yes	--	--	Yes	--	Yes	Yes	Yes	Yes
G4ST01150233460	chr1:150233461-150233493	-	GGGCCGGTGGGGCGGGAAGCCCGGCTTCTGGGG<br>((((...))))...	S100A10	NM_002966	chr1:150222010-150233338	non-template	-122	upstream	Yes	--	--	--	--	--	--	Yes	Yes	Yes	--
G4ST01150557196	chr1:150557197-150557229	-	GGGGGAGGGTGGAATAATGGTTCGAGGGAAGGG<br>..((((....))))..	FLG	NM_002016	chr1:150541275-150564303	non-template	33	Intron_1.f	--	Yes	--	Yes	--	--	--	--	--	--	--
G4ST01151035885	chr1:151035886-151035925	+	GGGATGCTCAGAACAGAGCAGGAGGGAAGGAGGGGCAGGG<br>.((((((......))))))...	LCE1D	NM_178352	chr1:151035851-151037281	non-template	40	Intron_1.u	--	Yes	--	Yes	--	--	--	--	--	--	Yes
G4ST01151050537	chr1:151050538-151050571	+	GGGCAGGAGGGGAGGGCAGGCAGAGCCTCCAGGG<br>.((((...))))...	LCE1B	NM_178349	chr1:151051071-151052209	non-template	-499	upstream	Yes	--	--	--	--	--	--	--	--	--	--
G4ST01151050545	chr1:151050546-151050577	+	GGGGAGGGCAGGCAGAGCCTCCAGGGCTGGGG<br>.((((...))))...	LCE1B	NM_178349	chr1:151051071-151052209	non-template	-493	upstream	Yes	--	--	--	--	--	--	--	--	--	--
G4ST01151066631	chr1:151066632-151066664	-	GGGGGGCACTTAGGAGTGGGGCACTTGGGAGGG<br>...((((....))))	LCE1A	NM_178348	chr1:151066573-151067197	template	33	Exon_1.u	--	Yes	Yes	--	Yes	--	--	--	--	--	Yes
G4ST01151066635	chr1:151066636-151066676	-	GGGGGACACTTTGGGGGGCACTTAGGAGTGGGGCACTTGGG<br>...((((....))))	LCE1A	NM_178348	chr1:151066573-151067197	template	41	Exon_1.u	--	Yes	Yes	--	Yes	--	--	--	--	--	Yes
G4ST01151066643	chr1:151066644-151066685	-	GGGCACTTAGGGGGACACTTTGGGGGGCACTTAGGAGTGGGG<br>...((((....))))	LCE1A	NM_178348	chr1:151066573-151067197	template	42	Exon_1.u	--	Yes	Yes	--	Yes	--	--	--	--	--	Yes
G4ST01151589293	chr1:151589294-151589322	-	GGGGAGCGAGGCTGGGCACTTGGGAAGGG<br>.(((...)))	PGLYRP4	NM_020393	chr1:151569221-151587646	non-template	-1647	upstream	Yes	--	--	--	--	--	--	--	--	--	--
G4ST01151589298	chr1:151589299-151589332	-	GGGCGCAGGTGGGGAGCGAGGCTGGGCACTTGGG<br>.(((...)))	PGLYRP4	NM_020393	chr1:151569221-151587646	non-template	-1652	upstream	Yes	--	--	--	--	--	--	--	--	--	--

G4ST01151595992	chr1:151595993-151596027	+	GGGAGGTGGGCCCTGTGTTCCCACAGTGGGCAGGG<br>..(((((....))))).	S100A9	NM_002965	chr1:151596954-151600127	non-template	-926	upstream	Yes	--	--	--	--	--	--	--	Yes	--	--
G4ST01151614911	chr1:151614912-151614943	-	GGGCACCCCTGTGCGGGTGGGTGGAGTGGGGG<br>(((....))).	S100A12	NM_005621	chr1:151612808-151614699	non-template	-212	upstream	Yes	--	--	--	--	--	--	--	Yes	--	--
G4ST01151658088	chr1:151658089-151658125	-	GGGGCTGAGAGCACAGGGTGAGTGGGTGAAGTTGGGG<br>(((...)))...	S100A7A	NM_176823	chr1:151655624-151662325	template	37	Intron_2.l	--	Yes	--	Yes	--	--	--	--	--	--	--
G4ST01151676124	chr1:151676125-151676156	+	GGGGGTCTCCAGAGGCCCCTGGGTAGGGTGGG<br>(((((....)))))...	S100A7L2	NM_001045479	chr1:151676095-151679127	template	32	Exon_3.l	--	Yes	Yes	--	--	--	Yes	--	--	--	--
G4ST01151697188	chr1:151697189-151697225	+	GGGGCTGAGAGCACAGGGTGAGTGGGTGAAGTTGGGG<br>(((...)))...	S100A7	NM_002963	chr1:151696844-151699761	template	37	Intron_2.l	--	Yes	--	Yes	--	--	--	--	--	--	--
G4ST01151776133	chr1:151776134-151776174	-	GGGTTGAGACAGCTTCTGCCTCTGGGGGTTAGGGTCCTGGG<br>..(((.(((...))).)))...	S100A6	NM_014624	chr1:151773700-151775341	non-template	-792	upstream	Yes	--	--	--	--	--	--	--	Yes	Yes	--
G4ST01151804736	chr1:151804737-151804776	-	GGGAATGAGTGGGAATGGCAAGAGGCCAGGGTTTGGTGGG<br>..((((.....))))	S100A2	NM_005978	chr1:151800209-151804930	non-template	40	Exon_1.f	--	Yes	Yes	--	--	Yes	--	Yes	Yes	Yes	Yes
G4ST01151804745	chr1:151804746-151804780	-	GGGAGGGAATGAGTGGGAATGGCAAGAGGCCAGGG<br>..((((.....))))	S100A2	NM_005978	chr1:151800209-151804930	non-template	35	Exon_1.f	--	Yes	Yes	--	--	Yes	--	Yes	Yes	Yes	Yes
G4ST01151850304	chr1:151850305-151850335	-	GGGTGGGGCTGGAGTGGCTGGGATCCAGGGG<br>(((.....))).	S100A16	NM_080388	chr1:151845991-151852138	non-template	31	Intron_1.f	--	Yes	--	Yes	--	--	--	Yes	Yes	Yes	Yes
G4ST01151850313	chr1:151850314-151850339	-	GGGAGGGTGGGGCTGGAGTGGCTGGG<br>(((.....))).	S100A16	NM_080388	chr1:151845991-151852138	non-template	26	Intron_1.f	--	Yes	--	Yes	--	--	--	Yes	Yes	Yes	Yes
G4ST01151856177	chr1:151856178-151856219	-	GGGGGGCTGTTGCTCTGCAGCTGGGTTAGGGAAGGCATGGGG<br>..(((((......))))).	S100A14	NM_020672	chr1:151853356-151855414	non-template	-763	upstream	Yes	--	--	--	--	--	--	--	Yes	Yes	Yes
G4ST01151898034	chr1:151898035-151898071	+	GGGGCGGGGCCAAGAAAGTGTTCTGGCTGGGAGTGGG<br>((((.(((....))))))).	SNAPIN	NM_012437	chr1:151897769-151900950	non-template	37	Intron_1.f	--	Yes	--	Yes	--	--	--	Yes	Yes	Yes	--
G4ST01151909716	chr1:151909717-151909751	+	GGGGGCGGGGGAAAGGTGGGGAGCTCCTCCAGGGG<br>(((...)))...	ILF2	NM_004515	chr1:151901138-151910103	template	35	Intron_1.f	--	Yes	--	Yes	--	--	--	Yes	Yes	Yes	--
G4ST01151917115	chr1:151917116-151917150	-	GGGTGTCTGGGAGGGAGGAGGTTCACACCTCAGGG<br>..(((((....))))).	NPR1	NM_000906	chr1:151917788-151933092	template	-637	upstream	Yes	--	--	--	--	--	--	Yes	Yes	--	Yes
G4ST01151917699	chr1:151917700-151917728	+	GGGAGGGAGGGTTCGGAGCTTGGCTCGGG<br>....((((...))))	NPR1	NM_000906	chr1:151917788-151933092	non-template	-59	upstream	Yes	--	--	--	--	--	--	Yes	Yes	Yes	--
G4ST01151919730	chr1:151919731-151919770	+	GGGCCCTGCTCCCCGCAGGCCCTGGGAGAGAGGGGATGGG<br>.(((((.....)))))....	NPR1	NM_000906	chr1:151917788-151933092	non-template	40	Exon_2	--	Yes	Yes	--	Yes	--	--	--	Yes	--	--
G4ST01152161617	chr1:152161618-152161650	+	GGGGGCGCGCCGTGCCGGGGTGGGGGGAGGGGG<br>((((.....)))).	GATAD2B	NM_020699	chr1:152043827-152162075	template	33	Intron_1.f	--	Yes	--	Yes	--	--	--	Yes	Yes	Yes	--
G4ST01152175104	chr1:152175105-152175141	-	GGGGTGGGATGGCAGCTGTGCCAGGGTGTGTGAGGGG<br>.(((((....)))))	DENND4B	NM_014856	chr1:152168601-152185778	non-template	37	Intron_17	--	Yes	--	Yes	--	--	--	--	Yes	--	--
G4ST01152175115	chr1:152175116-152175148	-	GGGGTGAGGGGTGGGATGGCAGCTGTGCCAGGG<br>.(((((....)))))	DENND4B	NM_014856	chr1:152168601-152185778	non-template	33	Intron_17	--	Yes	--	Yes	--	--	--	--	Yes	--	--
G4ST01152176617	chr1:152176618-152176652	+	GGGGGCGCTACGGGAAGGGCCTGGCACAGGCAGGG<br>((((...))))..	DENND4B	NM_014856	chr1:152168601-152185778	template	35	Exon_15	--	Yes	Yes	--	Yes	--	--	--	--	--	--
G4ST01152178600	chr1:152178601-152178637	-	GGGGCTGGGCTCTGTCAGAAAGGCGGAGGGGACTGGG<br>(((((((.....)))))))	DENND4B	NM_014856	chr1:152168601-152185778	non-template	37	Intron_12	--	Yes	--	Yes	--	--	--	--	--	--	--
G4ST01152179373	chr1:152179374-152179412	+	GGGGAAAGAGTGGCGAGGCTCTGGGATAGGGTGACGGGG<br>...(((((......)))))	DENND4B	NM_014856	chr1:152168601-152185778	template	39	Intron_10	--	Yes	--	Yes	--	--	--	--	--	--	--
G4ST01152184164	chr1:152184165-152184197	-	GGGCTCCATGGTGGAGGGTGTGGGATGGCCGGG<br>..(((...))).	DENND4B	NM_014856	chr1:152168601-152185778	non-template	33	Intron_1.f	--	Yes	--	Yes	--	--	--	Yes	Yes	Yes	--
G4ST01152192390	chr1:152192391-152192423	+	GGGAGGTGTGGGGCCTGGGTAGGTATCCTGGGG<br>.(((...))).	CRTC2	NM_181715	chr1:152186772-152197756	template	33	Exon_6	--	Yes	Yes	--	Yes	--	--	--	--	--	--
G4ST01152192399	chr1:152192400-152192430	+	GGGGCCTGGGTAGGTATCCTGGGGACTGGGG<br>.(((...))).	CRTC2	NM_181715	chr1:152186772-152197756	template	31	Exon_6	--	Yes	Yes	--	Yes	--	--	--	--	--	--
G4ST01152192406	chr1:152192407-152192452	+	GGGTAGGTATCCTGGGGACTGGGGTTCATCACACTTGTATGAAGGG<br>.(((...))). .(((((.........)))))	CRTC2	NM_181715	chr1:152186772-152197756	template	46	Exon_6	--	Yes	Yes	--	Yes	--	--	--	--	--	--
G4ST01152198479	chr1:152198480-152198511	-	GGGGTGGGGCAGAAATATTTTCTGGGCTGGGG<br>..((((.....))))	SLC39A1	NM_014437	chr1:152198212-152206812	non-template	32	Exon_5.l	--	Yes	Yes	--	--	--	Yes	--	--	--	--
G4ST01152198479	chr1:152198480-152198511	-	GGGGTGGGGCAGAAATATTTTCTGGGCTGGGG<br>..((((.....))))	CRTC2	NM_181715	chr1:152186772-152197756	non-template	-723	upstream	Yes	--	--	--	--	--	--	--	--	--	--
G4ST01152201935	chr1:152201936-152201971	-	GGGTAGGGGAAGGTCACAAAGTGGCTGGGGCCAGGG<br>....(((((...)))))..	SLC39A1	NM_014437	chr1:152198212-152206812	non-template	36	Intron_2	--	Yes	--	Yes	--	--	--	Yes	Yes	Yes	--
G4ST01152202660	chr1:152202661-152202698	-	GGGTACGCGGCTCCTGTGGGGGAGGGGGGAGCTATGGG<br>..((((.....)))).	SLC39A1	NM_014437	chr1:152198212-152206812	non-template	38	Exon_2-Intron_2	--	Yes	Yes	Yes	--	Yes	--	Yes	Yes	Yes	Yes
G4ST01152422486	chr1:152422487-152422523	+	GGGGTTGGGACGAGGGAGTGTTACCATGGTAACTGGG<br>...(((((....))))).	TPM3	NM_153649	chr1:152394404-152422349	template	-137	upstream	Yes	--	--	--	--	--	--	Yes	Yes	Yes	Yes
G4ST01152422486	chr1:152422487-152422523	+	GGGGTTGGGACGAGGGAGTGTTACCATGGTAACTGGG<br>...(((((....))))).	TPM3	NM_152263	chr1:152400914-152431233	template	37	Intron_2	--	Yes	--	Yes	--	--	--	Yes	Yes	Yes	Yes
G4ST01152422486	chr1:152422487-152422523	+	GGGGTTGGGACGAGGGAGTGTTACCATGGTAACTGGG<br>...(((((....))))).	TPM3	NM_001043353	chr1:152394404-152422349	template	-137	upstream	Yes	--	--	--	--	--	--	Yes	Yes	Yes	Yes
G4ST01152422486	chr1:152422487-152422523	+	GGGGTTGGGACGAGGGAGTGTTACCATGGTAACTGGG<br>...(((((....))))).	TPM3	NM_001043352	chr1:152394404-152422349	template	-137	upstream	Yes	--	--	--	--	--	--	Yes	Yes	Yes	Yes
G4ST01152422486	chr1:152422487-152422523	+	GGGGTTGGGACGAGGGAGTGTTACCATGGTAACTGGG<br>...(((((....))))).	TPM3	NM_001043351	chr1:152394404-152422349	template	-137	upstream	Yes	--	--	--	--	--	--	Yes	Yes	Yes	Yes
G4ST01152564842	chr1:152564843-152564884	+	GGGGCCAGGAGGGCAGAGGGAGATGCGGCCGCAGCCGCGGGG<br>....(((((....))))).	ATP8B2	NM_001005855	chr1:152564660-152576947	non-template	42	Intron_1.f	--	Yes	--	Yes	--	--	--	Yes	Yes	Yes	Yes
G4ST01152564852	chr1:152564853-152564893	+	GGGCAGAGGGAGATGCGGCCGCAGCCGCGGGGCTGGAGGGG<br>....(((((....))))).	ATP8B2	NM_001005855	chr1:152564660-152576947	non-template	41	Intron_1.f	--	Yes	--	Yes	--	--	--	Yes	Yes	Yes	Yes
G4ST01152564859	chr1:152564860-152564899	+	GGGAGATGCGGCCGCAGCCGCGGGGCTGGAGGGGCCGGGG<br>....(((((....))))).	ATP8B2	NM_001005855	chr1:152564660-152576947	non-template	40	Intron_1.f	--	Yes	--	Yes	--	--	--	Yes	Yes	Yes	Yes
G4ST01152567928	chr1:152567929-152567956	+	GGGAGGATGGCCTGGGCTGCGGGTGGGG<br>(((....)))	ATP8B2	NM_020452	chr1:152566900-152590404	non-template	28	Intron_2	--	Yes	--	Yes	--	--	--	--	Yes	--	--
G4ST01152567928	chr1:152567929-152567956	+	GGGAGGATGGCCTGGGCTGCGGGTGGGG<br>(((....)))	ATP8B2	NM_001005855	chr1:152564660-152576947	non-template	28	Intron_2	--	Yes	--	Yes	--	--	--	--	Yes	--	--
G4ST01152569626	chr1:152569627-152569661	+	GGGACTGAGGGCTGCCTGGGAAGAGTGCTTGGGGG<br>(((....)))..	ATP8B2	NM_020452	chr1:152566900-152590404	non-template	35	Intron_3	--	Yes	--	Yes	--	--	--	--	--	--	--
G4ST01152569626	chr1:152569627-152569661	+	GGGACTGAGGGCTGCCTGGGAAGAGTGCTTGGGGG<br>(((....)))..	ATP8B2	NM_001005855	chr1:152564660-152576947	non-template	35	Intron_3	--	Yes	--	Yes	--	--	--	--	--	--	--
G4ST01152569634	chr1:152569635-152569666	+	GGGCTGCCTGGGAAGAGTGCTTGGGGGAGGGG<br>(((....)))..	ATP8B2	NM_020452	chr1:152566900-152590404	non-template	32	Intron_3	--	Yes	--	Yes	--	--	--	--	--	--	--
G4ST01152569634	chr1:152569635-152569666	+	GGGCTGCCTGGGAAGAGTGCTTGGGGGAGGGG<br>(((....)))..	ATP8B2	NM_001005855	chr1:152564660-152576947	non-template	32	Intron_3	--	Yes	--	Yes	--	--	--	--	--	--	--
G4ST01152662257	chr1:152662258-152662298	-	GGGCACGGCAGAGCGCAGTGGGCACTGAGGGATGTCGGGGG<br>.((.((.....)).))	IL6R	NM_181359	chr1:152644293-152706812	template	41	Intron_1.f	--	Yes	--	Yes	--	--	--	--	--	--	Yes
G4ST01152662257	chr1:152662258-152662298	-	GGGCACGGCAGAGCGCAGTGGGCACTGAGGGATGTCGGGGG<br>.((.((.....)).))	IL6R	NM_000565	chr1:152644293-152706812	template	41	Intron_1.f	--	Yes	--	Yes	--	--	--	--	--	--	Yes
G4ST01152662267	chr1:152662268-152662304	-	GGGAGAGGGCACGGCAGAGCGCAGTGGGCACTGAGGG<br>.((.((.....)).))	IL6R	NM_181359	chr1:152644293-152706812	template	37	Intron_1.f	--	Yes	--	Yes	--	--	--	--	--	--	Yes
G4ST01152662267	chr1:152662268-152662304	-	GGGAGAGGGCACGGCAGAGCGCAGTGGGCACTGAGGG<br>.((.((.....)).))	IL6R	NM_000565	chr1:152644293-152706812	template	37	Intron_1.f	--	Yes	--	Yes	--	--	--	--	--	--	Yes
G4ST01152667920	chr1:152667921-152667962	+	GGGGAGTTGAGGGGAGGGCTCCACACCTTCTAAGGAGTGGGG<br>...(((.(((....))).)))	IL6R	NM_181359	chr1:152644293-152706812	non-template	42	Intron_1.f	--	Yes	--	Yes	--	--	--	--	--	--	--
G4ST01152667920	chr1:152667921-152667962	+	GGGGAGTTGAGGGGAGGGCTCCACACCTTCTAAGGAGTGGGG<br>...(((.(((....))).)))	IL6R	NM_000565	chr1:152644293-152706812	non-template	42	Intron_1.f	--	Yes	--	Yes	--	--	--	--	--	--	--

G4ST01152683009	chr1:152683010-152683042	-	GGGTGGGGTGTCCCCTGGACAAGCAGGGCAGGG<br>.(((((...)))))....	IL6R	NM_181359	chr1:152644293-152706812	template	33	Intron_6	--	Yes	--	Yes	--	--	--	--	--	--	Yes
G4ST01152683009	chr1:152683010-152683042	-	GGGTGGGGTGTCCCCTGGACAAGCAGGGCAGGG<br>.(((((...)))))....	IL6R	NM_000565	chr1:152644293-152706812	template	33	Intron_6	--	Yes	--	Yes	--	--	--	--	--	--	Yes
G4ST01152683014	chr1:152683015-152683046	-	GGGTGGGTGGGGTGTCCCCTGGACAAGCAGGG<br>.(((((...)))))....	IL6R	NM_181359	chr1:152644293-152706812	template	32	Intron_6	--	Yes	--	Yes	--	--	--	--	--	--	Yes
G4ST01152683014	chr1:152683015-152683046	-	GGGTGGGTGGGGTGTCCCCTGGACAAGCAGGG<br>.(((((...)))))....	IL6R	NM_000565	chr1:152644293-152706812	template	32	Intron_6	--	Yes	--	Yes	--	--	--	--	--	--	Yes
G4ST01152685492	chr1:152685493-152685528	-	GGGGAGAGGGTGACCCTGCTTCGGCGTCAGGGAGGG<br>.(((...((....))))).	IL6R	NM_181359	chr1:152644293-152706812	template	36	Intron_6	--	Yes	--	Yes	--	--	--	Yes	--	--	--
G4ST01152685492	chr1:152685493-152685528	-	GGGGAGAGGGTGACCCTGCTTCGGCGTCAGGGAGGG<br>.(((...((....))))).	IL6R	NM_000565	chr1:152644293-152706812	template	36	Intron_6	--	Yes	--	Yes	--	--	--	Yes	--	--	--
G4ST01152698214	chr1:152698215-152698246	-	GGGCCAGTGGTGGGCCAATGGGACCAGCTGGG<br>((...)).	IL6R	NM_181359	chr1:152644293-152706812	template	32	Intron_8.l	--	Yes	--	Yes	--	--	--	Yes	--	--	--
G4ST01152698214	chr1:152698215-152698246	-	GGGCCAGTGGTGGGCCAATGGGACCAGCTGGG<br>((...)).	IL6R	NM_000565	chr1:152644293-152706812	template	32	Intron_9.l	--	Yes	--	Yes	--	--	--	Yes	--	--	--
G4ST01152700612	chr1:152700613-152700645	-	GGGTATAGGGTCAGGTGCTGAAGGGACATTGGG<br>.(((...)))..	IL6R	NM_181359	chr1:152644293-152706812	template	33	Intron_8.l	--	Yes	--	Yes	--	--	--	--	--	--	--
G4ST01152700612	chr1:152700613-152700645	-	GGGTATAGGGTCAGGTGCTGAAGGGACATTGGG<br>.(((...)))..	IL6R	NM_000565	chr1:152644293-152706812	template	33	Intron_9.l	--	Yes	--	Yes	--	--	--	--	--	--	--
G4ST01152742154	chr1:152742155-152742180	+	GGGATGCCAGGCGAGGGTTGGGAGGG<br>.(((...))).	SHE	NM_001010846	chr1:152718578-152741150	template	-1004	upstream	Yes	--	--	--	--	--	--	Yes	Yes	--	Yes
G4ST01152742154	chr1:152742155-152742180	+	GGGATGCCAGGCGAGGGTTGGGAGGG<br>.(((...))).	TDRD10	NM_001098475	chr1:152741319-152787247	non-template	26	Intron_1.f	--	Yes	--	Yes	--	--	--	Yes	Yes	--	Yes
G4ST01152742154	chr1:152742155-152742180	+	GGGATGCCAGGCGAGGGTTGGGAGGG<br>.(((...))).	TDRD10	NM_182499	chr1:152741319-152787247	non-template	26	Intron_1.f	--	Yes	--	Yes	--	--	--	Yes	Yes	--	Yes
G4ST01152781741	chr1:152781742-152781773	+	GGGCAGGGCCAAGGCCTCTTGTGGGTTAGGGG<br>.((((....)))).	TDRD10	NM_001098475	chr1:152741319-152787247	non-template	32	Intron_7	--	Yes	--	Yes	--	--	--	--	--	--	--
G4ST01152781741	chr1:152781742-152781773	+	GGGCAGGGCCAAGGCCTCTTGTGGGTTAGGGG<br>.((((....)))).	TDRD10	NM_182499	chr1:152741319-152787247	non-template	32	Intron_7	--	Yes	--	Yes	--	--	--	--	--	--	--
G4ST01152811277	chr1:152811278-152811312	+	GGGACCCCGGGCGTGAGATATGGGGTCTGCCAGGG<br>(((.....)))	CHRNB2	NM_000748	chr1:152806881-152818977	non-template	35	Intron_5.l	--	Yes	--	Yes	--	--	--	--	--	--	--
G4ST01152811285	chr1:152811286-152811318	+	GGGCGTGAGATATGGGGTCTGCCAGGGCCCGGG<br>(((.....)))	CHRNB2	NM_000748	chr1:152806881-152818977	non-template	33	Intron_5.l	--	Yes	--	Yes	--	--	--	--	--	--	--
G4ST01152816749	chr1:152816750-152816784	-	GGGCCTGGGAGGGAGGCTGGCATGACAGTCAGGGG<br>.(((((......)))))..	CHRNB2	NM_000748	chr1:152806881-152818977	template	35	Exon_6.l	--	Yes	Yes	--	--	--	Yes	--	Yes	Yes	Yes
G4ST01152847150	chr1:152847151-152847193	-	GGGGGCCTCGAGGGGCTGGCGCGGCCCAGCGGTCGGGCCAGGG<br>(((((......)))))....	ADAR	NM_001193495	chr1:152821158-152845400	non-template	-1750	upstream	Yes	--	--	--	--	--	--	Yes	Yes	Yes	--
G4ST01152847150	chr1:152847151-152847193	-	GGGGGCCTCGAGGGGCTGGCGCGGCCCAGCGGTCGGGCCAGGG<br>(((((......)))))....	ADAR	NM_015840	chr1:152821158-152847348	non-template	43	Exon_1.f	--	Yes	Yes	--	--	Yes	--	Yes	Yes	Yes	--
G4ST01152847150	chr1:152847151-152847193	-	GGGGGCCTCGAGGGGCTGGCGCGGCCCAGCGGTCGGGCCAGGG<br>(((((......)))))....	ADAR	NM_015841	chr1:152821158-152847348	non-template	43	Exon_1.f	--	Yes	Yes	--	--	Yes	--	Yes	Yes	Yes	--
G4ST01152847150	chr1:152847151-152847193	-	GGGGGCCTCGAGGGGCTGGCGCGGCCCAGCGGTCGGGCCAGGG<br>(((((......)))))....	ADAR	NM_001111	chr1:152821158-152847348	non-template	43	Exon_1.f	--	Yes	Yes	--	--	Yes	--	Yes	Yes	Yes	--
G4ST01152847150	chr1:152847151-152847193	-	GGGGGCCTCGAGGGGCTGGCGCGGCCCAGCGGTCGGGCCAGGG<br>(((((......)))))....	ADAR	NM_001025107	chr1:152821158-152867080	non-template	43	Intron_1.f	--	Yes	--	Yes	--	--	--	Yes	Yes	Yes	--
G4ST01152999952	chr1:152999953-152999996	-	GGGCCGCGGGGCGCCTTTGCGGGGCCGGAGTGCTTCTTCGCGGG<br>.(((....))) ..(((((.....))))).	KCNN3	NM_170782	chr1:152936566-153098962	non-template	44	Intron_3	--	Yes	--	Yes	--	--	--	--	--	--	--
G4ST01152999952	chr1:152999953-152999996	-	GGGCCGCGGGGCGCCTTTGCGGGGCCGGAGTGCTTCTTCGCGGG<br>.(((....))) ..(((((.....))))).	KCNN3	NM_001204087	chr1:152936566-153109378	non-template	44	Intron_3	--	Yes	--	Yes	--	--	--	--	--	--	--
G4ST01152999952	chr1:152999953-152999996	-	GGGCCGCGGGGCGCCTTTGCGGGGCCGGAGTGCTTCTTCGCGGG<br>.(((....))) ..(((((.....))))).	KCNN3	NM_002249	chr1:152936566-153109378	non-template	44	Intron_3	--	Yes	--	Yes	--	--	--	--	--	--	--
G4ST01153034785	chr1:153034786-153034819	+	GGGGGCACTGAAAGTTGGTGGGGGTGGGTCAGGG<br>..(((((.....))))).	KCNN3	NM_170782	chr1:152936566-153098962	template	34	Intron_2	--	Yes	--	Yes	--	--	--	--	--	--	--
G4ST01153034785	chr1:153034786-153034819	+	GGGGGCACTGAAAGTTGGTGGGGGTGGGTCAGGG<br>..(((((.....))))).	KCNN3	NM_001204087	chr1:152936566-153109378	template	34	Intron_2	--	Yes	--	Yes	--	--	--	--	--	--	--
G4ST01153034785	chr1:153034786-153034819	+	GGGGGCACTGAAAGTTGGTGGGGGTGGGTCAGGG<br>..(((((.....))))).	KCNN3	NM_002249	chr1:152936566-153109378	template	34	Intron_2	--	Yes	--	Yes	--	--	--	--	--	--	--
G4ST01153045992	chr1:153045993-153046039	-	GGGACCACATTCTAATTTGGTGGGAGTCCTTGGGCACCCTGGTGGGG<br>((((.((....)).)))) (((....)))	KCNN3	NM_170782	chr1:152936566-153098962	non-template	47	Intron_2	--	Yes	--	Yes	--	--	--	--	Yes	--	--
G4ST01153045992	chr1:153045993-153046039	-	GGGACCACATTCTAATTTGGTGGGAGTCCTTGGGCACCCTGGTGGGG<br>((((.((....)).)))) (((....)))	KCNN3	NM_001204087	chr1:152936566-153109378	non-template	47	Intron_2	--	Yes	--	Yes	--	--	--	--	Yes	--	--
G4ST01153045992	chr1:153045993-153046039	-	GGGACCACATTCTAATTTGGTGGGAGTCCTTGGGCACCCTGGTGGGG<br>((((.((....)).)))) (((....)))	KCNN3	NM_002249	chr1:152936566-153109378	non-template	47	Intron_2	--	Yes	--	Yes	--	--	--	--	Yes	--	--
G4ST01153046005	chr1:153046006-153046045	-	GGGGCTGGGACCACATTCTAATTTGGTGGGAGTCCTTGGG<br>((((.((....)).))))	KCNN3	NM_170782	chr1:152936566-153098962	non-template	40	Intron_2	--	Yes	--	Yes	--	--	--	--	Yes	--	--
G4ST01153046005	chr1:153046006-153046045	-	GGGGCTGGGACCACATTCTAATTTGGTGGGAGTCCTTGGG<br>((((.((....)).))))	KCNN3	NM_001204087	chr1:152936566-153109378	non-template	40	Intron_2	--	Yes	--	Yes	--	--	--	--	Yes	--	--
G4ST01153046005	chr1:153046006-153046045	-	GGGGCTGGGACCACATTCTAATTTGGTGGGAGTCCTTGGG<br>((((.((....)).))))	KCNN3	NM_002249	chr1:152936566-153109378	non-template	40	Intron_2	--	Yes	--	Yes	--	--	--	--	Yes	--	--
G4ST01153052261	chr1:153052262-153052288	+	GGGTTGCTGGGGTGGGAGCCCCTTGGG<br>((...)).	KCNN3	NM_170782	chr1:152936566-153098962	template	27	Intron_2	--	Yes	--	Yes	--	--	--	--	--	--	Yes
G4ST01153052261	chr1:153052262-153052288	+	GGGTTGCTGGGGTGGGAGCCCCTTGGG<br>((...)).	KCNN3	NM_001204087	chr1:152936566-153109378	template	27	Intron_2	--	Yes	--	Yes	--	--	--	--	--	--	Yes
G4ST01153052261	chr1:153052262-153052288	+	GGGTTGCTGGGGTGGGAGCCCCTTGGG<br>((...)).	KCNN3	NM_002249	chr1:152936566-153109378	template	27	Intron_2	--	Yes	--	Yes	--	--	--	--	--	--	Yes
G4ST01153052269	chr1:153052270-153052293	+	GGGGTGGGAGCCCCTTGGGCAGGG<br>((...)).	KCNN3	NM_170782	chr1:152936566-153098962	template	24	Intron_2	--	Yes	--	Yes	--	--	--	--	--	--	Yes
G4ST01153052269	chr1:153052270-153052293	+	GGGGTGGGAGCCCCTTGGGCAGGG<br>((...)).	KCNN3	NM_001204087	chr1:152936566-153109378	template	24	Intron_2	--	Yes	--	Yes	--	--	--	--	--	--	Yes
G4ST01153052269	chr1:153052270-153052293	+	GGGGTGGGAGCCCCTTGGGCAGGG<br>((...)).	KCNN3	NM_002249	chr1:152936566-153109378	template	24	Intron_2	--	Yes	--	Yes	--	--	--	--	--	--	Yes
G4ST01153056026	chr1:153056027-153056062	-	GGGAATGTCTCAAGGACAGGGTCCTGGGTAGAGGGG<br>..((((.....))))	KCNN3	NM_170782	chr1:152936566-153098962	non-template	36	Intron_2	--	Yes	--	Yes	--	--	--	--	--	--	--
G4ST01153056026	chr1:153056027-153056062	-	GGGAATGTCTCAAGGACAGGGTCCTGGGTAGAGGGG<br>..((((.....))))	KCNN3	NM_001204087	chr1:152936566-153109378	non-template	36	Intron_2	--	Yes	--	Yes	--	--	--	--	--	--	--
G4ST01153056026	chr1:153056027-153056062	-	GGGAATGTCTCAAGGACAGGGTCCTGGGTAGAGGGG<br>..((((.....))))	KCNN3	NM_002249	chr1:152936566-153109378	non-template	36	Intron_2	--	Yes	--	Yes	--	--	--	--	--	--	--
G4ST01153068111	chr1:153068112-153068151	-	GGGATTCAGGCTCAGCAGGTTGGGGTTGGAGCGGGTGGGG<br>...(((.((....)).)))	KCNN3	NM_170782	chr1:152936566-153098962	non-template	40	Intron_1.f	--	Yes	--	Yes	--	--	--	--	--	--	--
G4ST01153068111	chr1:153068112-153068151	-	GGGATTCAGGCTCAGCAGGTTGGGGTTGGAGCGGGTGGGG<br>...(((.((....)).)))	KCNN3	NM_001204087	chr1:152936566-153109378	non-template	40	Intron_1.f	--	Yes	--	Yes	--	--	--	--	--	--	--
G4ST01153068111	chr1:153068112-153068151	-	GGGATTCAGGCTCAGCAGGTTGGGGTTGGAGCGGGTGGGG<br>...(((.((....)).)))	KCNN3	NM_002249	chr1:152936566-153109378	non-template	40	Intron_1.f	--	Yes	--	Yes	--	--	--	--	--	--	--
G4ST01153082817	chr1:153082818-153082856	-	GGGTTCTGTGGGAAGCAAGGGTGAGAAGGAGTCTCCGGG<br>.((((.....)))).	KCNN3	NM_170782	chr1:152936566-153098962	non-template	39	Intron_1.f	--	Yes	--	Yes	--	--	--	--	--	--	--
G4ST01153082817	chr1:153082818-153082856	-	GGGTTCTGTGGGAAGCAAGGGTGAGAAGGAGTCTCCGGG<br>.((((.....)))).	KCNN3	NM_001204087	chr1:152936566-153109378	non-template	39	Intron_1.f	--	Yes	--	Yes	--	--	--	--	--	--	--
G4ST01153082817	chr1:153082818-153082856	-	GGGTTCTGTGGGAAGCAAGGGTGAGAAGGAGTCTCCGGG<br>.((((.....)))).	KCNN3	NM_002249	chr1:152936566-153109378	non-template	39	Intron_1.f	--	Yes	--	Yes	--	--	--	--	--	--	--

G4ST01153091045	chr1:153091046-153091077	-	GGGCGGGGTTGTGAGGTCACAGGGGAGGCGGG<br>..((((....)))).	KCNN3	NM_170782	chr1:152936566-153098962	non-template	32	Intron_1.f	--	Yes	--	Yes	--	--	--	Yes	--	--	--
G4ST01153091045	chr1:153091046-153091077	-	GGGCGGGGTTGTGAGGTCACAGGGGAGGCGGG<br>..((((....)))).	KCNN3	NM_001204087	chr1:152936566-153109378	non-template	32	Intron_1.f	--	Yes	--	Yes	--	--	--	Yes	--	--	--
G4ST01153091045	chr1:153091046-153091077	-	GGGCGGGGTTGTGAGGTCACAGGGGAGGCGGG<br>..((((....)))).	KCNN3	NM_002249	chr1:152936566-153109378	non-template	32	Intron_1.f	--	Yes	--	Yes	--	--	--	Yes	--	--	--
G4ST01153091052	chr1:153091053-153091087	-	GGGATCATGTGGGCGGGGTTGTGAGGTCACAGGGG<br>..((((....)))).	KCNN3	NM_170782	chr1:152936566-153098962	non-template	35	Intron_1.f	--	Yes	--	Yes	--	--	--	Yes	--	--	--
G4ST01153091052	chr1:153091053-153091087	-	GGGATCATGTGGGCGGGGTTGTGAGGTCACAGGGG<br>..((((....)))).	KCNN3	NM_001204087	chr1:152936566-153109378	non-template	35	Intron_1.f	--	Yes	--	Yes	--	--	--	Yes	--	--	--
G4ST01153091052	chr1:153091053-153091087	-	GGGATCATGTGGGCGGGGTTGTGAGGTCACAGGGG<br>..((((....)))).	KCNN3	NM_002249	chr1:152936566-153109378	non-template	35	Intron_1.f	--	Yes	--	Yes	--	--	--	Yes	--	--	--
G4ST01153095098	chr1:153095099-153095127	-	GGGGAGCTTCCCTCAGGGCGGGGAGAGGG<br>(((.....))).	KCNN3	NM_170782	chr1:152936566-153098962	non-template	29	Intron_1.f	--	Yes	--	Yes	--	--	--	--	--	--	Yes
G4ST01153095098	chr1:153095099-153095127	-	GGGGAGCTTCCCTCAGGGCGGGGAGAGGG<br>(((.....))).	KCNN3	NM_001204087	chr1:152936566-153109378	non-template	29	Intron_1.f	--	Yes	--	Yes	--	--	--	--	--	--	Yes
G4ST01153095098	chr1:153095099-153095127	-	GGGGAGCTTCCCTCAGGGCGGGGAGAGGG<br>(((.....))).	KCNN3	NM_002249	chr1:152936566-153109378	non-template	29	Intron_1.f	--	Yes	--	Yes	--	--	--	--	--	--	Yes
G4ST01153095104	chr1:153095105-153095134	-	GGGCTCAGGGGAGCTTCCCTCAGGGCGGGG<br>(((.....))).	KCNN3	NM_170782	chr1:152936566-153098962	non-template	30	Intron_1.f	--	Yes	--	Yes	--	--	--	--	--	--	Yes
G4ST01153095104	chr1:153095105-153095134	-	GGGCTCAGGGGAGCTTCCCTCAGGGCGGGG<br>(((.....))).	KCNN3	NM_001204087	chr1:152936566-153109378	non-template	30	Intron_1.f	--	Yes	--	Yes	--	--	--	--	--	--	Yes
G4ST01153095104	chr1:153095105-153095134	-	GGGCTCAGGGGAGCTTCCCTCAGGGCGGGG<br>(((.....))).	KCNN3	NM_002249	chr1:152936566-153109378	non-template	30	Intron_1.f	--	Yes	--	Yes	--	--	--	--	--	--	Yes
G4ST01153107280	chr1:153107281-153107315	-	GGGGGAGCACCTCTCCTTGGGACGGGATGACAGGG<br>((((.....))))..	KCNN3	NM_001204087	chr1:152936566-153109378	non-template	35	Intron_1.f	--	Yes	--	Yes	--	--	--	--	--	--	--
G4ST01153107280	chr1:153107281-153107315	-	GGGGGAGCACCTCTCCTTGGGACGGGATGACAGGG<br>((((.....))))..	KCNN3	NM_002249	chr1:152936566-153109378	non-template	35	Intron_1.f	--	Yes	--	Yes	--	--	--	--	--	--	--
G4ST01153109684	chr1:153109685-153109718	+	GGGGAGGAGGGACAGAGGGCCTCCGAAGGAGGGG<br>.((((...))))	KCNN3	NM_001204087	chr1:152936566-153109378	template	-306	upstream	Yes	--	--	--	--	--	--	--	Yes	--	Yes
G4ST01153109684	chr1:153109685-153109718	+	GGGGAGGAGGGACAGAGGGCCTCCGAAGGAGGGG<br>.((((...))))	KCNN3	NM_002249	chr1:152936566-153109378	template	-306	upstream	Yes	--	--	--	--	--	--	--	Yes	--	Yes
G4ST01153169785	chr1:153169786-153169824	-	GGGAGCGATGCTGCAGGGTGGTGGGGTGGTGGTGGGGGG<br>.(((....))).	PMVK	NM_006556	chr1:153163832-153176108	non-template	39	Intron_2	--	Yes	--	Yes	--	--	--	--	--	--	Yes
G4ST01153201295	chr1:153201296-153201335	-	GGGGAGGCGGGAGGTCGCGGCCGCCCAGGGAAGCTGGGGG<br>.((.((....)).)).	PYGO2	NM_138300	chr1:153196126-153200882	non-template	-413	upstream	Yes	--	--	--	--	--	--	--	Yes	Yes	--
G4ST01153208585	chr1:153208586-153208615	-	GGGCCCTTTGTGAAGGGGCTGGGGTGGGGG<br>..(((....)))	SHC1	NM_003029	chr1:153201398-153213583	non-template	30	Intron_2	--	Yes	--	Yes	--	--	--	--	--	--	--
G4ST01153208585	chr1:153208586-153208615	-	GGGCCCTTTGTGAAGGGGCTGGGGTGGGGG<br>..(((....)))	SHC1	NM_183001	chr1:153201398-153209847	non-template	30	Intron_1.f	--	Yes	--	Yes	--	--	--	--	--	--	--
G4ST01153208585	chr1:153208586-153208615	-	GGGCCCTTTGTGAAGGGGCTGGGGTGGGGG<br>..(((....)))	SHC1	NM_001202859	chr1:153201398-153213583	non-template	30	Intron_2	--	Yes	--	Yes	--	--	--	--	--	--	--
G4ST01153208585	chr1:153208586-153208615	-	GGGCCCTTTGTGAAGGGGCTGGGGTGGGGG<br>..(((....)))	SHC1	NM_001130041	chr1:153201398-153213583	non-template	30	Intron_2	--	Yes	--	Yes	--	--	--	--	--	--	--
G4ST01153208585	chr1:153208586-153208615	-	GGGCCCTTTGTGAAGGGGCTGGGGTGGGGG<br>..(((....)))	SHC1	NM_001130040	chr1:153201398-153209847	non-template	30	Intron_1.f	--	Yes	--	Yes	--	--	--	--	--	--	--
G4ST01153209212	chr1:153209213-153209259	-	GGGTGGAAGGGGGCCAGCTTGGGGGCGAGGAGTGGACCCGCCACGGG<br>((((.((......))))))..	SHC1	NM_003029	chr1:153201398-153213583	non-template	47	Exon_2	--	Yes	Yes	--	Yes	--	--	Yes	Yes	--	--
G4ST01153209212	chr1:153209213-153209259	-	GGGTGGAAGGGGGCCAGCTTGGGGGCGAGGAGTGGACCCGCCACGGG<br>((((.((......))))))..	SHC1	NM_183001	chr1:153201398-153209847	non-template	47	Exon_1.f	--	Yes	Yes	--	Yes	--	--	Yes	Yes	--	--
G4ST01153209212	chr1:153209213-153209259	-	GGGTGGAAGGGGGCCAGCTTGGGGGCGAGGAGTGGACCCGCCACGGG<br>((((.((......))))))..	SHC1	NM_001202859	chr1:153201398-153213583	non-template	47	Exon_2	--	Yes	Yes	--	--	Yes	--	Yes	Yes	--	--
G4ST01153209212	chr1:153209213-153209259	-	GGGTGGAAGGGGGCCAGCTTGGGGGCGAGGAGTGGACCCGCCACGGG<br>((((.((......))))))..	SHC1	NM_001130041	chr1:153201398-153213583	non-template	47	Exon_2	--	Yes	Yes	--	Yes	--	--	Yes	Yes	--	--
G4ST01153209212	chr1:153209213-153209259	-	GGGTGGAAGGGGGCCAGCTTGGGGGCGAGGAGTGGACCCGCCACGGG<br>((((.((......))))))..	SHC1	NM_001130040	chr1:153201398-153209847	non-template	47	Exon_1.f	--	Yes	Yes	--	Yes	--	--	Yes	Yes	--	--
G4ST01153222209	chr1:153222210-153222251	-	GGGGGCCGGACCTTAGCCCACGGTCGGGGGCGGGACCTCGGG<br>..(((.(((.........))))))	FLAD1	NM_025207	chr1:153222394-153232211	template	-142	upstream	Yes	--	--	--	--	--	--	Yes	Yes	Yes	Yes
G4ST01153222209	chr1:153222210-153222251	-	GGGGGCCGGACCTTAGCCCACGGTCGGGGGCGGGACCTCGGG<br>..(((.(((.........))))))	FLAD1	NM_001184892	chr1:153222394-153228112	template	-142	upstream	Yes	--	--	--	--	--	--	Yes	Yes	Yes	Yes
G4ST01153222209	chr1:153222210-153222251	-	GGGGGCCGGACCTTAGCCCACGGTCGGGGGCGGGACCTCGGG<br>..(((.(((.........))))))	FLAD1	NM_001184891	chr1:153222394-153232211	template	-142	upstream	Yes	--	--	--	--	--	--	Yes	Yes	Yes	Yes
G4ST01153222209	chr1:153222210-153222251	-	GGGGGCCGGACCTTAGCCCACGGTCGGGGGCGGGACCTCGGG<br>..(((.(((.........))))))	FLAD1	NM_201398	chr1:153222394-153232211	template	-142	upstream	Yes	--	--	--	--	--	--	Yes	Yes	Yes	Yes
G4ST01153222951	chr1:153222952-153222984	-	GGGGGCCATAGCCTGGGAACAGGGGGTCCTGGG<br>(((....))).	FLAD1	NM_025207	chr1:153222394-153232211	template	33	Exon_1.f	--	Yes	Yes	--	Yes	--	--	--	Yes	--	Yes
G4ST01153222951	chr1:153222952-153222984	-	GGGGGCCATAGCCTGGGAACAGGGGGTCCTGGG<br>(((....))).	FLAD1	NM_001184892	chr1:153222394-153228112	template	33	Exon_2	--	Yes	Yes	--	--	Yes	--	--	Yes	--	Yes
G4ST01153222951	chr1:153222952-153222984	-	GGGGGCCATAGCCTGGGAACAGGGGGTCCTGGG<br>(((....))).	FLAD1	NM_001184891	chr1:153222394-153232211	template	33	Exon_2	--	Yes	Yes	--	--	Yes	--	--	Yes	--	Yes
G4ST01153222951	chr1:153222952-153222984	-	GGGGGCCATAGCCTGGGAACAGGGGGTCCTGGG<br>(((....))).	FLAD1	NM_201398	chr1:153222394-153232211	template	33	Exon_2	--	Yes	Yes	--	--	Yes	--	--	Yes	--	Yes
G4ST01153222958	chr1:153222959-153222991	-	GGGCACTGGGGGCCATAGCCTGGGAACAGGGGG<br>(((....))).	FLAD1	NM_025207	chr1:153222394-153232211	template	33	Exon_1.f	--	Yes	Yes	--	Yes	--	--	--	Yes	--	Yes
G4ST01153222958	chr1:153222959-153222991	-	GGGCACTGGGGGCCATAGCCTGGGAACAGGGGG<br>(((....))).	FLAD1	NM_001184892	chr1:153222394-153228112	template	33	Exon_2	--	Yes	Yes	--	--	Yes	--	--	Yes	--	Yes
G4ST01153222958	chr1:153222959-153222991	-	GGGCACTGGGGGCCATAGCCTGGGAACAGGGGG<br>(((....))).	FLAD1	NM_001184891	chr1:153222394-153232211	template	33	Exon_2	--	Yes	Yes	--	--	Yes	--	--	Yes	--	Yes
G4ST01153222958	chr1:153222959-153222991	-	GGGCACTGGGGGCCATAGCCTGGGAACAGGGGG<br>(((....))).	FLAD1	NM_201398	chr1:153222394-153232211	template	33	Exon_2	--	Yes	Yes	--	--	Yes	--	--	Yes	--	Yes
G4ST01153222967	chr1:153222968-153223012	-	GGGGGGCCTGCCAGGTCTACAGGGCACTGGGGGCCATAGCCTGGG<br>.(((((...))))).... (((....))).	FLAD1	NM_025207	chr1:153222394-153232211	template	45	Exon_1.f	--	Yes	Yes	--	Yes	--	--	--	Yes	--	Yes
G4ST01153222967	chr1:153222968-153223012	-	GGGGGGCCTGCCAGGTCTACAGGGCACTGGGGGCCATAGCCTGGG<br>.(((((...))))).... (((....))).	FLAD1	NM_001184892	chr1:153222394-153228112	template	45	Exon_2	--	Yes	Yes	--	--	Yes	--	--	Yes	--	Yes
G4ST01153222967	chr1:153222968-153223012	-	GGGGGGCCTGCCAGGTCTACAGGGCACTGGGGGCCATAGCCTGGG<br>.(((((...))))).... (((....))).	FLAD1	NM_001184891	chr1:153222394-153232211	template	45	Exon_2	--	Yes	Yes	--	--	Yes	--	--	Yes	--	Yes
G4ST01153222967	chr1:153222968-153223012	-	GGGGGGCCTGCCAGGTCTACAGGGCACTGGGGGCCATAGCCTGGG<br>.(((((...))))).... (((....))).	FLAD1	NM_201398	chr1:153222394-153232211	template	45	Exon_2	--	Yes	Yes	--	--	Yes	--	--	Yes	--	Yes
G4ST01153228487	chr1:153228488-153228522	+	GGGCAGGTCAGGGGCAGACTGCAGGGCCTAGCGGG<br>(((...))).	FLAD1	NM_025207	chr1:153222394-153232211	non-template	35	Intron_2	--	Yes	--	Yes	--	--	--	--	--	Yes	--
G4ST01153228487	chr1:153228488-153228522	+	GGGCAGGTCAGGGGCAGACTGCAGGGCCTAGCGGG<br>(((...))).	FLAD1	NM_001184891	chr1:153222394-153232211	non-template	35	Intron_3	--	Yes	--	Yes	--	--	--	--	--	Yes	--
G4ST01153228487	chr1:153228488-153228522	+	GGGCAGGTCAGGGGCAGACTGCAGGGCCTAGCGGG<br>(((...))).	FLAD1	NM_201398	chr1:153222394-153232211	non-template	35	Intron_3	--	Yes	--	Yes	--	--	--	--	--	Yes	--
G4ST01153243917	chr1:153243918-153243958	-	GGGGGTGTGTGGTACCCAAGGGAGGGGACAGTGTCTGGGGG<br>((((.....))))... ..(((...))).	ZBTB7B	NM_015872	chr1:153241736-153257623	template	41	Intron_2	--	Yes	--	Yes	--	--	--	--	Yes	Yes	--
G4ST01153243931	chr1:153243932-153243963	-	GGGCTGGGGGTGTGTGGTACCCAAGGGAGGGG<br>((((.....))))....	ZBTB7B	NM_015872	chr1:153241736-153257623	template	32	Intron_2	--	Yes	--	Yes	--	--	--	--	Yes	Yes	--
G4ST01153247991	chr1:153247992-153248022	-	GGGGGAGGGCCAGGCTTGGGGGGAGGTAGGG<br>(((....))).	ZBTB7B	NM_015872	chr1:153241736-153257623	template	31	Intron_2	--	Yes	--	Yes	--	--	--	Yes	--	--	--

G4ST01153247999	chr1:153248000-153248031	-	GGGACAGGTGGGGGAGGGCCAGGCTTGGGGGG<br>(((....))).	ZBTB7B	NM_015872	chr1:153241736-153257623	template	32	Intron_2	--	Yes	--	Yes	--	--	--	Yes	--	--	--
G4ST01153308103	chr1:153308104-153308143	-	GGGGATCTTGGGAGGACTCTGGCTCCTTGGGATGAGAGGG<br>((((.......)))).	EFNA4	NM_005227	chr1:153302837-153308653	template	40	Exon_4.l	--	Yes	Yes	--	--	--	Yes	--	--	--	--
G4ST01153308103	chr1:153308104-153308143	-	GGGGATCTTGGGAGGACTCTGGCTCCTTGGGATGAGAGGG<br>((((.......)))).	EFNA4	NM_182689	chr1:153302837-153308653	template	40	Intron_3.l-Exon_4.l	--	Yes	Yes	Yes	Yes	--	--	--	--	--	--
G4ST01153308103	chr1:153308104-153308143	-	GGGGATCTTGGGAGGACTCTGGCTCCTTGGGATGAGAGGG<br>((((.......)))).	EFNA4	NM_182690	chr1:153302837-153308653	template	40	Exon_4.l	--	Yes	Yes	--	Yes	--	--	--	--	--	--
G4ST01153319612	chr1:153319613-153319649	-	GGGGTGGGGGGCGGACGTGTGAGTCTGGGGGAGGGGG<br>...(((((......))))).	EFNA3	NM_004952	chr1:153317972-153326638	template	37	Intron_1.f	--	Yes	--	Yes	--	--	--	--	--	--	--
G4ST01153321826	chr1:153321827-153321859	-	GGGCCGCGCCTCCTGCGGAAGGGTGGGGGTGGG<br>(((((.....)))))..	EFNA3	NM_004952	chr1:153317972-153326638	template	33	Intron_1.f	--	Yes	--	Yes	--	--	--	Yes	--	--	Yes
G4ST01153323740	chr1:153323741-153323771	-	GGGATGGAAGCCAAGGGTGAACGTGGGAGGG<br>.(((...))).	EFNA3	NM_004952	chr1:153317972-153326638	template	31	Intron_1.f	--	Yes	--	Yes	--	--	--	Yes	Yes	--	Yes
G4ST01153323744	chr1:153323745-153323775	-	GGGAGGGATGGAAGCCAAGGGTGAACGTGGG<br>.(((...))).	EFNA3	NM_004952	chr1:153317972-153326638	template	31	Intron_1.f	--	Yes	--	Yes	--	--	--	Yes	Yes	--	Yes
G4ST01153323754	chr1:153323755-153323780	-	GGGATGGGAGGGATGGAAGCCAAGGG<br>.(((...))).	EFNA3	NM_004952	chr1:153317972-153326638	template	26	Intron_1.f	--	Yes	--	Yes	--	--	--	Yes	Yes	--	Yes
G4ST01153324281	chr1:153324282-153324309	+	GGGGCGGGACCGGGGCCCGGAGGCGGGG<br>(((....))).	EFNA3	NM_004952	chr1:153317972-153326638	non-template	28	Exon_2	--	Yes	Yes	--	Yes	--	--	Yes	Yes	Yes	Yes
G4ST01153324559	chr1:153324560-153324604	+	GGGGGGTGGAGCCCCTAGGCTCCGGGGCGGGGCCGGCGCGGCGGG<br>....((((((....)))))). ((((...))))	EFNA3	NM_004952	chr1:153317972-153326638	non-template	45	Intron_2	--	Yes	--	Yes	--	--	--	Yes	Yes	Yes	--
G4ST01153325663	chr1:153325664-153325698	+	GGGGAGAGATGGGGCGGGGCTTGGAAGGAGCAGGG<br>((((.....)))).	EFNA3	NM_004952	chr1:153317972-153326638	non-template	35	Exon_5.l	--	Yes	Yes	--	--	--	Yes	Yes	--	--	Yes
G4ST01153326477	chr1:153326478-153326515	-	GGGAGATGGTTCCCGGCTCAGGGCTGGAAGGGAGGGGG<br>((.(((...))).))..	EFNA3	NM_004952	chr1:153317972-153326638	template	38	Exon_5.l	--	Yes	Yes	--	--	--	Yes	Yes	--	--	--
G4ST01153365735	chr1:153365736-153365766	-	GGGTGTCAGCTCCTGGGGTGGAGAGGGCGGG<br>...(((...)))	EFNA1	NM_004428	chr1:153366973-153374010	template	-1206	upstream	Yes	--	--	--	--	--	--	Yes	Yes	Yes	--
G4ST01153365735	chr1:153365736-153365766	-	GGGTGTCAGCTCCTGGGGTGGAGAGGGCGGG<br>...(((...)))	EFNA1	NM_182685	chr1:153366973-153374010	template	-1206	upstream	Yes	--	--	--	--	--	--	Yes	Yes	Yes	--
G4ST01153365739	chr1:153365740-153365770	-	GGGCGGGTGTCAGCTCCTGGGGTGGAGAGGG<br>...(((...)))	EFNA1	NM_004428	chr1:153366973-153374010	template	-1202	upstream	Yes	--	--	--	--	--	--	Yes	Yes	Yes	--
G4ST01153365739	chr1:153365740-153365770	-	GGGCGGGTGTCAGCTCCTGGGGTGGAGAGGG<br>...(((...)))	EFNA1	NM_182685	chr1:153366973-153374010	template	-1202	upstream	Yes	--	--	--	--	--	--	Yes	Yes	Yes	--
G4ST01153365748	chr1:153365749-153365779	-	GGGCTTGGCGGGCGGGTGTCAGCTCCTGGGG<br>...(((...)))	EFNA1	NM_004428	chr1:153366973-153374010	template	-1193	upstream	Yes	--	--	--	--	--	--	Yes	Yes	Yes	--
G4ST01153365748	chr1:153365749-153365779	-	GGGCTTGGCGGGCGGGTGTCAGCTCCTGGGG<br>...(((...)))	EFNA1	NM_182685	chr1:153366973-153374010	template	-1193	upstream	Yes	--	--	--	--	--	--	Yes	Yes	Yes	--
G4ST01153365855	chr1:153365856-153365885	+	GGGGGAGGGGGCCTGAGCCGGGTACTGGGG<br>(((....)))	EFNA1	NM_004428	chr1:153366973-153374010	non-template	-1087	upstream	Yes	--	--	--	--	--	--	Yes	Yes	Yes	--
G4ST01153365855	chr1:153365856-153365885	+	GGGGGAGGGGGCCTGAGCCGGGTACTGGGG<br>(((....)))	EFNA1	NM_182685	chr1:153366973-153374010	non-template	-1087	upstream	Yes	--	--	--	--	--	--	Yes	Yes	Yes	--
G4ST01153413170	chr1:153413171-153413207	+	GGGTTCTGGGGCAAGCTGGGACCTGGAACAGGTGGGG<br>(((((...))))).	KRTCAP2	NM_173852	chr1:153408508-153412428	template	-742	upstream	Yes	--	--	--	--	--	--	--	--	Yes	Yes
G4ST01153413170	chr1:153413171-153413207	+	GGGTTCTGGGGCAAGCTGGGACCTGGAACAGGTGGGG<br>(((((...))))).	TRIM46	NM_025058	chr1:153412984-153424069	non-template	37	Intron_1.f	--	Yes	--	Yes	--	--	--	--	--	Yes	Yes
G4ST01153413915	chr1:153413916-153413952	+	GGGGGCGAAGGGGAATGGGTTGGTAAGACCGATGGGG<br>.((((...))))...	KRTCAP2	NM_173852	chr1:153408508-153412428	template	-1487	upstream	Yes	--	--	--	--	--	--	Yes	--	--	Yes
G4ST01153413915	chr1:153413916-153413952	+	GGGGGCGAAGGGGAATGGGTTGGTAAGACCGATGGGG<br>.((((...))))...	TRIM46	NM_025058	chr1:153412984-153424069	non-template	37	Intron_1.f	--	Yes	--	Yes	--	--	--	Yes	--	--	Yes
G4ST01153415354	chr1:153415355-153415385	+	GGGGAGGGAGGGACATGCTGCTGCAGGTGGG<br>((.(((....))).))	TRIM46	NM_025058	chr1:153412984-153424069	non-template	31	Intron_3	--	Yes	--	Yes	--	--	--	--	--	--	--
G4ST01153415359	chr1:153415360-153415391	+	GGGAGGGACATGCTGCTGCAGGTGGGTGGGGG<br>((.(((....))).))	TRIM46	NM_025058	chr1:153412984-153424069	non-template	32	Intron_3	--	Yes	--	Yes	--	--	--	--	--	--	--
G4ST01153415363	chr1:153415364-153415407	+	GGGACATGCTGCTGCAGGTGGGTGGGGGTGCCTGGCATTGTGGG<br>((.(((....))).)) .((((...))))...	TRIM46	NM_025058	chr1:153412984-153424069	non-template	44	Intron_3	--	Yes	--	Yes	--	--	--	--	--	--	--
G4ST01153415382	chr1:153415383-153415425	+	GGGTGGGGGTGCCTGGCATTGTGGGTTTCCAAAAGGAAGTGGG<br>.((((...))))... .((((....))))..	TRIM46	NM_025058	chr1:153412984-153424069	non-template	43	Intron_3	--	Yes	--	Yes	--	--	--	--	--	--	--
G4ST01153415386	chr1:153415387-153415430	+	GGGGGTGCCTGGCATTGTGGGTTTCCAAAAGGAAGTGGGAAGGG<br>.((((...))))... .((((....))))..	TRIM46	NM_025058	chr1:153412984-153424069	non-template	44	Intron_3	--	Yes	--	Yes	--	--	--	--	--	--	--
G4ST01153417963	chr1:153417964-153417994	-	GGGGGCCCGGCGGCGGGCAGGGCCCCCGGGG<br>.(((....)))	TRIM46	NM_025058	chr1:153412984-153424069	template	31	Intron_7	--	Yes	--	Yes	--	--	--	Yes	Yes	--	Yes
G4ST01153417972	chr1:153417973-153417999	-	GGGAAGGGGGCCCGGCGGCGGGCAGGG<br>.(((....)))	TRIM46	NM_025058	chr1:153412984-153424069	template	27	Intron_7	--	Yes	--	Yes	--	--	--	Yes	Yes	--	Yes
G4ST01153417977	chr1:153417978-153418004	-	GGGGTGGGAAGGGGGCCCGGCGGCGGG<br>.(((....)))	TRIM46	NM_025058	chr1:153412984-153424069	template	27	Intron_7	--	Yes	--	Yes	--	--	--	Yes	Yes	--	Yes
G4ST01153418191	chr1:153418192-153418231	-	GGGACAGCAAAAGCTGAGGGCAGGGGTTGGAGGTCAAGGG<br>.((((....)))). .(((.....)))	TRIM46	NM_025058	chr1:153412984-153424069	template	40	Intron_7	--	Yes	--	Yes	--	--	--	--	Yes	--	--
G4ST01153423330	chr1:153423331-153423370	-	GGGGGTAACAACCGGGTGCCAAGAGGTTTGGGGGCCAGGG<br>(((....))) ..((((.....))))	TRIM46	NM_025058	chr1:153412984-153424069	template	40	Exon_10.l	--	Yes	Yes	--	--	--	Yes	--	Yes	--	--
G4ST01153427684	chr1:153427685-153427711	+	GGGCTGGGGGGGCGGTGGAGCCCGGGG<br>(((......)))	MUC1	NM_001204289	chr1:153424924-153429330	template	27	Intron_2	--	Yes	--	Yes	--	--	--	--	Yes	--	--
G4ST01153427684	chr1:153427685-153427711	+	GGGCTGGGGGGGCGGTGGAGCCCGGGG<br>(((......)))	MUC1	NM_001204287	chr1:153424924-153429330	template	27	Intron_2	--	Yes	--	Yes	--	--	--	--	Yes	--	--
G4ST01153427684	chr1:153427685-153427711	+	GGGCTGGGGGGGCGGTGGAGCCCGGGG<br>(((......)))	MUC1	NM_001204288	chr1:153424924-153429330	template	27	Intron_2	--	Yes	--	Yes	--	--	--	--	Yes	--	--
G4ST01153427684	chr1:153427685-153427711	+	GGGCTGGGGGGGCGGTGGAGCCCGGGG<br>(((......)))	MUC1	NM_001044393	chr1:153424924-153429330	template	27	Intron_2	--	Yes	--	Yes	--	--	--	--	Yes	--	--
G4ST01153427684	chr1:153427685-153427711	+	GGGCTGGGGGGGCGGTGGAGCCCGGGG<br>(((......)))	MUC1	NM_002456	chr1:153424924-153429330	template	27	Intron_2	--	Yes	--	Yes	--	--	--	--	Yes	--	--
G4ST01153427684	chr1:153427685-153427711	+	GGGCTGGGGGGGCGGTGGAGCCCGGGG<br>(((......)))	MUC1	NM_001204295	chr1:153424924-153429330	template	27	Intron_2	--	Yes	--	Yes	--	--	--	--	Yes	--	--
G4ST01153427684	chr1:153427685-153427711	+	GGGCTGGGGGGGCGGTGGAGCCCGGGG<br>(((......)))	MUC1	NM_001204296	chr1:153424924-153429330	template	27	Intron_2	--	Yes	--	Yes	--	--	--	--	Yes	--	--
G4ST01153427684	chr1:153427685-153427711	+	GGGCTGGGGGGGCGGTGGAGCCCGGGG<br>(((......)))	MUC1	NM_001204292	chr1:153424924-153429330	template	27	Intron_2	--	Yes	--	Yes	--	--	--	--	Yes	--	--
G4ST01153427684	chr1:153427685-153427711	+	GGGCTGGGGGGGCGGTGGAGCCCGGGG<br>(((......)))	MUC1	NM_001204291	chr1:153424924-153429330	template	27	Intron_2	--	Yes	--	Yes	--	--	--	--	Yes	--	--
G4ST01153427684	chr1:153427685-153427711	+	GGGCTGGGGGGGCGGTGGAGCCCGGGG<br>(((......)))	MUC1	NM_001204290	chr1:153424924-153429330	template	27	Intron_2	--	Yes	--	Yes	--	--	--	--	Yes	--	--
G4ST01153427684	chr1:153427685-153427711	+	GGGCTGGGGGGGCGGTGGAGCCCGGGG<br>(((......)))	MUC1	NM_001044391	chr1:153424924-153429330	template	27	Intron_2	--	Yes	--	Yes	--	--	--	--	Yes	--	--
G4ST01153427684	chr1:153427685-153427711	+	GGGCTGGGGGGGCGGTGGAGCCCGGGG<br>(((......)))	MUC1	NM_001044392	chr1:153424924-153429330	template	27	Intron_2	--	Yes	--	Yes	--	--	--	--	Yes	--	--
G4ST01153427684	chr1:153427685-153427711	+	GGGCTGGGGGGGCGGTGGAGCCCGGGG<br>(((......)))	MUC1	NM_001204293	chr1:153424924-153429330	template	27	Intron_2	--	Yes	--	Yes	--	--	--	--	Yes	--	--
G4ST01153427684	chr1:153427685-153427711	+	GGGCTGGGGGGGCGGTGGAGCCCGGGG<br>(((......)))	MUC1	NM_001018016	chr1:153424924-153429330	template	27	Intron_2	--	Yes	--	Yes	--	--	--	--	Yes	--	--
G4ST01153427684	chr1:153427685-153427711	+	GGGCTGGGGGGGCGGTGGAGCCCGGGG<br>(((......)))	MUC1	NM_001044390	chr1:153424924-153429330	template	27	Intron_2	--	Yes	--	Yes	--	--	--	--	Yes	--	--

G4ST01153427684	chr1:153427685-153427711	+	GGGCTGGGGGGGCGGTGGAGCCCGGGG<br>(((.......)))	MUC1	NM_001204285	chr1:153424924-153429330	template	27	Intron_3-Exon_4	--	Yes	Yes	Yes	Yes	--	--	--	Yes	--	--
G4ST01153427684	chr1:153427685-153427711	+	GGGCTGGGGGGGCGGTGGAGCCCGGGG<br>(((.......)))	MUC1	NM_001204294	chr1:153424924-153429330	template	27	Intron_2	--	Yes	--	Yes	--	--	--	--	Yes	--	--
G4ST01153427684	chr1:153427685-153427711	+	GGGCTGGGGGGGCGGTGGAGCCCGGGG<br>(((.......)))	MUC1	NM_001204286	chr1:153424924-153429330	template	27	Intron_3-Exon_4	--	Yes	Yes	Yes	Yes	--	--	--	Yes	--	--
G4ST01153427684	chr1:153427685-153427711	+	GGGCTGGGGGGGCGGTGGAGCCCGGGG<br>(((.......)))	MUC1	NM_001018017	chr1:153424924-153429330	template	27	Intron_2	--	Yes	--	Yes	--	--	--	--	Yes	--	--
G4ST01153427684	chr1:153427685-153427711	+	GGGCTGGGGGGGCGGTGGAGCCCGGGG<br>(((.......)))	MUC1	NM_001204297	chr1:153424924-153429330	template	27	Intron_2	--	Yes	--	Yes	--	--	--	--	Yes	--	--
G4ST01153427744	chr1:153427745-153427771	+	GGGCTGGGGGGGCGGTGGAGCCCGGGG<br>(((.......)))	MUC1	NM_001204289	chr1:153424924-153429330	template	27	Intron_2	--	Yes	--	Yes	--	--	--	--	Yes	--	--
G4ST01153427744	chr1:153427745-153427771	+	GGGCTGGGGGGGCGGTGGAGCCCGGGG<br>(((.......)))	MUC1	NM_001204287	chr1:153424924-153429330	template	27	Intron_2	--	Yes	--	Yes	--	--	--	--	Yes	--	--
G4ST01153427744	chr1:153427745-153427771	+	GGGCTGGGGGGGCGGTGGAGCCCGGGG<br>(((.......)))	MUC1	NM_001204288	chr1:153424924-153429330	template	27	Intron_2	--	Yes	--	Yes	--	--	--	--	Yes	--	--
G4ST01153427744	chr1:153427745-153427771	+	GGGCTGGGGGGGCGGTGGAGCCCGGGG<br>(((.......)))	MUC1	NM_001044393	chr1:153424924-153429330	template	27	Intron_2	--	Yes	--	Yes	--	--	--	--	Yes	--	--
G4ST01153427744	chr1:153427745-153427771	+	GGGCTGGGGGGGCGGTGGAGCCCGGGG<br>(((.......)))	MUC1	NM_002456	chr1:153424924-153429330	template	27	Intron_2	--	Yes	--	Yes	--	--	--	--	Yes	--	--
G4ST01153427744	chr1:153427745-153427771	+	GGGCTGGGGGGGCGGTGGAGCCCGGGG<br>(((.......)))	MUC1	NM_001204295	chr1:153424924-153429330	template	27	Intron_2	--	Yes	--	Yes	--	--	--	--	Yes	--	--
G4ST01153427744	chr1:153427745-153427771	+	GGGCTGGGGGGGCGGTGGAGCCCGGGG<br>(((.......)))	MUC1	NM_001204296	chr1:153424924-153429330	template	27	Intron_2	--	Yes	--	Yes	--	--	--	--	Yes	--	--
G4ST01153427744	chr1:153427745-153427771	+	GGGCTGGGGGGGCGGTGGAGCCCGGGG<br>(((.......)))	MUC1	NM_001204292	chr1:153424924-153429330	template	27	Intron_2	--	Yes	--	Yes	--	--	--	--	Yes	--	--
G4ST01153427744	chr1:153427745-153427771	+	GGGCTGGGGGGGCGGTGGAGCCCGGGG<br>(((.......)))	MUC1	NM_001204291	chr1:153424924-153429330	template	27	Intron_2	--	Yes	--	Yes	--	--	--	--	Yes	--	--
G4ST01153427744	chr1:153427745-153427771	+	GGGCTGGGGGGGCGGTGGAGCCCGGGG<br>(((.......)))	MUC1	NM_001204290	chr1:153424924-153429330	template	27	Intron_2	--	Yes	--	Yes	--	--	--	--	Yes	--	--
G4ST01153427744	chr1:153427745-153427771	+	GGGCTGGGGGGGCGGTGGAGCCCGGGG<br>(((.......)))	MUC1	NM_001044391	chr1:153424924-153429330	template	27	Intron_2	--	Yes	--	Yes	--	--	--	--	Yes	--	--
G4ST01153427744	chr1:153427745-153427771	+	GGGCTGGGGGGGCGGTGGAGCCCGGGG<br>(((.......)))	MUC1	NM_001044392	chr1:153424924-153429330	template	27	Intron_2	--	Yes	--	Yes	--	--	--	--	Yes	--	--
G4ST01153427744	chr1:153427745-153427771	+	GGGCTGGGGGGGCGGTGGAGCCCGGGG<br>(((.......)))	MUC1	NM_001204293	chr1:153424924-153429330	template	27	Intron_2	--	Yes	--	Yes	--	--	--	--	Yes	--	--
G4ST01153427744	chr1:153427745-153427771	+	GGGCTGGGGGGGCGGTGGAGCCCGGGG<br>(((.......)))	MUC1	NM_001018016	chr1:153424924-153429330	template	27	Intron_2	--	Yes	--	Yes	--	--	--	--	Yes	--	--
G4ST01153427744	chr1:153427745-153427771	+	GGGCTGGGGGGGCGGTGGAGCCCGGGG<br>(((.......)))	MUC1	NM_001044390	chr1:153424924-153429330	template	27	Intron_2	--	Yes	--	Yes	--	--	--	--	Yes	--	--
G4ST01153427744	chr1:153427745-153427771	+	GGGCTGGGGGGGCGGTGGAGCCCGGGG<br>(((.......)))	MUC1	NM_001204285	chr1:153424924-153429330	template	27	Intron_3	--	Yes	--	Yes	--	--	--	--	Yes	--	--
G4ST01153427744	chr1:153427745-153427771	+	GGGCTGGGGGGGCGGTGGAGCCCGGGG<br>(((.......)))	MUC1	NM_001204294	chr1:153424924-153429330	template	27	Intron_2	--	Yes	--	Yes	--	--	--	--	Yes	--	--
G4ST01153427744	chr1:153427745-153427771	+	GGGCTGGGGGGGCGGTGGAGCCCGGGG<br>(((.......)))	MUC1	NM_001204286	chr1:153424924-153429330	template	27	Intron_3	--	Yes	--	Yes	--	--	--	--	Yes	--	--
G4ST01153427744	chr1:153427745-153427771	+	GGGCTGGGGGGGCGGTGGAGCCCGGGG<br>(((.......)))	MUC1	NM_001018017	chr1:153424924-153429330	template	27	Intron_2	--	Yes	--	Yes	--	--	--	--	Yes	--	--
G4ST01153427744	chr1:153427745-153427771	+	GGGCTGGGGGGGCGGTGGAGCCCGGGG<br>(((.......)))	MUC1	NM_001204297	chr1:153424924-153429330	template	27	Intron_2	--	Yes	--	Yes	--	--	--	--	Yes	--	--
G4ST01153427749	chr1:153427750-153427777	+	GGGGGGGCGGTGGAGCCCGGGGCCGGGG<br>(((.......)))	MUC1	NM_001204289	chr1:153424924-153429330	template	28	Intron_2	--	Yes	--	Yes	--	--	--	--	Yes	--	--
G4ST01153427749	chr1:153427750-153427777	+	GGGGGGGCGGTGGAGCCCGGGGCCGGGG<br>(((.......)))	MUC1	NM_001204287	chr1:153424924-153429330	template	28	Intron_2	--	Yes	--	Yes	--	--	--	--	Yes	--	--
G4ST01153427749	chr1:153427750-153427777	+	GGGGGGGCGGTGGAGCCCGGGGCCGGGG<br>(((.......)))	MUC1	NM_001204288	chr1:153424924-153429330	template	28	Intron_2	--	Yes	--	Yes	--	--	--	--	Yes	--	--
G4ST01153427749	chr1:153427750-153427777	+	GGGGGGGCGGTGGAGCCCGGGGCCGGGG<br>(((.......)))	MUC1	NM_001044393	chr1:153424924-153429330	template	28	Intron_2	--	Yes	--	Yes	--	--	--	--	Yes	--	--
G4ST01153427749	chr1:153427750-153427777	+	GGGGGGGCGGTGGAGCCCGGGGCCGGGG<br>(((.......)))	MUC1	NM_002456	chr1:153424924-153429330	template	28	Intron_2	--	Yes	--	Yes	--	--	--	--	Yes	--	--
G4ST01153427749	chr1:153427750-153427777	+	GGGGGGGCGGTGGAGCCCGGGGCCGGGG<br>(((.......)))	MUC1	NM_001204295	chr1:153424924-153429330	template	28	Intron_2	--	Yes	--	Yes	--	--	--	--	Yes	--	--
G4ST01153427749	chr1:153427750-153427777	+	GGGGGGGCGGTGGAGCCCGGGGCCGGGG<br>(((.......)))	MUC1	NM_001204296	chr1:153424924-153429330	template	28	Intron_2	--	Yes	--	Yes	--	--	--	--	Yes	--	--
G4ST01153427749	chr1:153427750-153427777	+	GGGGGGGCGGTGGAGCCCGGGGCCGGGG<br>(((.......)))	MUC1	NM_001204292	chr1:153424924-153429330	template	28	Intron_2	--	Yes	--	Yes	--	--	--	--	Yes	--	--
G4ST01153427749	chr1:153427750-153427777	+	GGGGGGGCGGTGGAGCCCGGGGCCGGGG<br>(((.......)))	MUC1	NM_001204291	chr1:153424924-153429330	template	28	Intron_2	--	Yes	--	Yes	--	--	--	--	Yes	--	--
G4ST01153427749	chr1:153427750-153427777	+	GGGGGGGCGGTGGAGCCCGGGGCCGGGG<br>(((.......)))	MUC1	NM_001204290	chr1:153424924-153429330	template	28	Intron_2	--	Yes	--	Yes	--	--	--	--	Yes	--	--
G4ST01153427749	chr1:153427750-153427777	+	GGGGGGGCGGTGGAGCCCGGGGCCGGGG<br>(((.......)))	MUC1	NM_001044391	chr1:153424924-153429330	template	28	Intron_2	--	Yes	--	Yes	--	--	--	--	Yes	--	--
G4ST01153427749	chr1:153427750-153427777	+	GGGGGGGCGGTGGAGCCCGGGGCCGGGG<br>(((.......)))	MUC1	NM_001044392	chr1:153424924-153429330	template	28	Intron_2	--	Yes	--	Yes	--	--	--	--	Yes	--	--
G4ST01153427749	chr1:153427750-153427777	+	GGGGGGGCGGTGGAGCCCGGGGCCGGGG<br>(((.......)))	MUC1	NM_001204293	chr1:153424924-153429330	template	28	Intron_2	--	Yes	--	Yes	--	--	--	--	Yes	--	--
G4ST01153427749	chr1:153427750-153427777	+	GGGGGGGCGGTGGAGCCCGGGGCCGGGG<br>(((.......)))	MUC1	NM_001018016	chr1:153424924-153429330	template	28	Intron_2	--	Yes	--	Yes	--	--	--	--	Yes	--	--
G4ST01153427749	chr1:153427750-153427777	+	GGGGGGGCGGTGGAGCCCGGGGCCGGGG<br>(((.......)))	MUC1	NM_001044390	chr1:153424924-153429330	template	28	Intron_2	--	Yes	--	Yes	--	--	--	--	Yes	--	--
G4ST01153427749	chr1:153427750-153427777	+	GGGGGGGCGGTGGAGCCCGGGGCCGGGG<br>(((.......)))	MUC1	NM_001204285	chr1:153424924-153429330	template	28	Intron_3	--	Yes	--	Yes	--	--	--	--	Yes	--	--
G4ST01153427749	chr1:153427750-153427777	+	GGGGGGGCGGTGGAGCCCGGGGCCGGGG<br>(((.......)))	MUC1	NM_001204294	chr1:153424924-153429330	template	28	Intron_2	--	Yes	--	Yes	--	--	--	--	Yes	--	--
G4ST01153427749	chr1:153427750-153427777	+	GGGGGGGCGGTGGAGCCCGGGGCCGGGG<br>(((.......)))	MUC1	NM_001204286	chr1:153424924-153429330	template	28	Intron_3	--	Yes	--	Yes	--	--	--	--	Yes	--	--
G4ST01153427749	chr1:153427750-153427777	+	GGGGGGGCGGTGGAGCCCGGGGCCGGGG<br>(((.......)))	MUC1	NM_001018017	chr1:153424924-153429330	template	28	Intron_2	--	Yes	--	Yes	--	--	--	--	Yes	--	--
G4ST01153427749	chr1:153427750-153427777	+	GGGGGGGCGGTGGAGCCCGGGGCCGGGG<br>(((.......)))	MUC1	NM_001204297	chr1:153424924-153429330	template	28	Intron_2	--	Yes	--	Yes	--	--	--	--	Yes	--	--
G4ST01153427882	chr1:153427883-153427909	+	GGGCTGGGGGGGCGGTGGAGCCCGGGG<br>(((.......)))	MUC1	NM_001204289	chr1:153424924-153429330	template	27	Intron_2	--	Yes	--	Yes	--	--	--	--	--	--	--
G4ST01153427882	chr1:153427883-153427909	+	GGGCTGGGGGGGCGGTGGAGCCCGGGG<br>(((.......)))	MUC1	NM_001204287	chr1:153424924-153429330	template	27	Intron_2	--	Yes	--	Yes	--	--	--	--	--	--	--
G4ST01153427882	chr1:153427883-153427909	+	GGGCTGGGGGGGCGGTGGAGCCCGGGG<br>(((.......)))	MUC1	NM_001204288	chr1:153424924-153429330	template	27	Intron_2	--	Yes	--	Yes	--	--	--	--	--	--	--
G4ST01153427882	chr1:153427883-153427909	+	GGGCTGGGGGGGCGGTGGAGCCCGGGG<br>(((.......)))	MUC1	NM_001044393	chr1:153424924-153429330	template	27	Intron_2	--	Yes	--	Yes	--	--	--	--	--	--	--
G4ST01153427882	chr1:153427883-153427909	+	GGGCTGGGGGGGCGGTGGAGCCCGGGG<br>(((.......)))	MUC1	NM_002456	chr1:153424924-153429330	template	27	Intron_2	--	Yes	--	Yes	--	--	--	--	--	--	--

G4ST01153427882	chr1:153427883-153427909	+	GGGCTGGGGGGGCGGTGGAGCCCGGGG<br>(((......)))	MUC1	NM_001204295	chr1:153424924-153429330	template	27	Intron_2	--	Yes	--	Yes	--	--	--	--	--	--	--
G4ST01153427882	chr1:153427883-153427909	+	GGGCTGGGGGGGCGGTGGAGCCCGGGG<br>(((......)))	MUC1	NM_001204296	chr1:153424924-153429330	template	27	Intron_2	--	Yes	--	Yes	--	--	--	--	--	--	--
G4ST01153427882	chr1:153427883-153427909	+	GGGCTGGGGGGGCGGTGGAGCCCGGGG<br>(((......)))	MUC1	NM_001204292	chr1:153424924-153429330	template	27	Intron_2	--	Yes	--	Yes	--	--	--	--	--	--	--
G4ST01153427882	chr1:153427883-153427909	+	GGGCTGGGGGGGCGGTGGAGCCCGGGG<br>(((......)))	MUC1	NM_001204291	chr1:153424924-153429330	template	27	Intron_2	--	Yes	--	Yes	--	--	--	--	--	--	--
G4ST01153427882	chr1:153427883-153427909	+	GGGCTGGGGGGGCGGTGGAGCCCGGGG<br>(((......)))	MUC1	NM_001204290	chr1:153424924-153429330	template	27	Intron_2	--	Yes	--	Yes	--	--	--	--	--	--	--
G4ST01153427882	chr1:153427883-153427909	+	GGGCTGGGGGGGCGGTGGAGCCCGGGG<br>(((......)))	MUC1	NM_001044391	chr1:153424924-153429330	template	27	Intron_2	--	Yes	--	Yes	--	--	--	--	--	--	--
G4ST01153427882	chr1:153427883-153427909	+	GGGCTGGGGGGGCGGTGGAGCCCGGGG<br>(((......)))	MUC1	NM_001044392	chr1:153424924-153429330	template	27	Intron_2	--	Yes	--	Yes	--	--	--	--	--	--	--
G4ST01153427882	chr1:153427883-153427909	+	GGGCTGGGGGGGCGGTGGAGCCCGGGG<br>(((......)))	MUC1	NM_001204293	chr1:153424924-153429330	template	27	Intron_2	--	Yes	--	Yes	--	--	--	--	--	--	--
G4ST01153427882	chr1:153427883-153427909	+	GGGCTGGGGGGGCGGTGGAGCCCGGGG<br>(((......)))	MUC1	NM_001018016	chr1:153424924-153429330	template	27	Intron_2	--	Yes	--	Yes	--	--	--	--	--	--	--
G4ST01153427882	chr1:153427883-153427909	+	GGGCTGGGGGGGCGGTGGAGCCCGGGG<br>(((......)))	MUC1	NM_001044390	chr1:153424924-153429330	template	27	Intron_2	--	Yes	--	Yes	--	--	--	--	--	--	--
G4ST01153427882	chr1:153427883-153427909	+	GGGCTGGGGGGGCGGTGGAGCCCGGGG<br>(((......)))	MUC1	NM_001204285	chr1:153424924-153429330	template	27	Intron_3	--	Yes	--	Yes	--	--	--	--	--	--	--
G4ST01153427882	chr1:153427883-153427909	+	GGGCTGGGGGGGCGGTGGAGCCCGGGG<br>(((......)))	MUC1	NM_001204294	chr1:153424924-153429330	template	27	Intron_2	--	Yes	--	Yes	--	--	--	--	--	--	--
G4ST01153427882	chr1:153427883-153427909	+	GGGCTGGGGGGGCGGTGGAGCCCGGGG<br>(((......)))	MUC1	NM_001204286	chr1:153424924-153429330	template	27	Intron_3	--	Yes	--	Yes	--	--	--	--	--	--	--
G4ST01153427882	chr1:153427883-153427909	+	GGGCTGGGGGGGCGGTGGAGCCCGGGG<br>(((......)))	MUC1	NM_001018017	chr1:153424924-153429330	template	27	Intron_2	--	Yes	--	Yes	--	--	--	--	--	--	--
G4ST01153427882	chr1:153427883-153427909	+	GGGCTGGGGGGGCGGTGGAGCCCGGGG<br>(((......)))	MUC1	NM_001204297	chr1:153424924-153429330	template	27	Intron_2	--	Yes	--	Yes	--	--	--	--	--	--	--
G4ST01153427942	chr1:153427943-153427969	+	GGGCTGGGGGGGCGGTGGAGCCCGGGG<br>(((......)))	MUC1	NM_001204289	chr1:153424924-153429330	template	27	Intron_2	--	Yes	--	Yes	--	--	--	--	--	--	--
G4ST01153427942	chr1:153427943-153427969	+	GGGCTGGGGGGGCGGTGGAGCCCGGGG<br>(((......)))	MUC1	NM_001204287	chr1:153424924-153429330	template	27	Intron_2	--	Yes	--	Yes	--	--	--	--	--	--	--
G4ST01153427942	chr1:153427943-153427969	+	GGGCTGGGGGGGCGGTGGAGCCCGGGG<br>(((......)))	MUC1	NM_001204288	chr1:153424924-153429330	template	27	Intron_2	--	Yes	--	Yes	--	--	--	--	--	--	--
G4ST01153427942	chr1:153427943-153427969	+	GGGCTGGGGGGGCGGTGGAGCCCGGGG<br>(((......)))	MUC1	NM_001044393	chr1:153424924-153429330	template	27	Intron_2	--	Yes	--	Yes	--	--	--	--	--	--	--
G4ST01153427942	chr1:153427943-153427969	+	GGGCTGGGGGGGCGGTGGAGCCCGGGG<br>(((......)))	MUC1	NM_002456	chr1:153424924-153429330	template	27	Intron_2	--	Yes	--	Yes	--	--	--	--	--	--	--
G4ST01153427942	chr1:153427943-153427969	+	GGGCTGGGGGGGCGGTGGAGCCCGGGG<br>(((......)))	MUC1	NM_001204295	chr1:153424924-153429330	template	27	Intron_2	--	Yes	--	Yes	--	--	--	--	--	--	--
G4ST01153427942	chr1:153427943-153427969	+	GGGCTGGGGGGGCGGTGGAGCCCGGGG<br>(((......)))	MUC1	NM_001204296	chr1:153424924-153429330	template	27	Intron_2	--	Yes	--	Yes	--	--	--	--	--	--	--
G4ST01153427942	chr1:153427943-153427969	+	GGGCTGGGGGGGCGGTGGAGCCCGGGG<br>(((......)))	MUC1	NM_001204292	chr1:153424924-153429330	template	27	Intron_2	--	Yes	--	Yes	--	--	--	--	--	--	--
G4ST01153427942	chr1:153427943-153427969	+	GGGCTGGGGGGGCGGTGGAGCCCGGGG<br>(((......)))	MUC1	NM_001204291	chr1:153424924-153429330	template	27	Intron_2	--	Yes	--	Yes	--	--	--	--	--	--	--
G4ST01153427942	chr1:153427943-153427969	+	GGGCTGGGGGGGCGGTGGAGCCCGGGG<br>(((......)))	MUC1	NM_001204290	chr1:153424924-153429330	template	27	Intron_2	--	Yes	--	Yes	--	--	--	--	--	--	--
G4ST01153427942	chr1:153427943-153427969	+	GGGCTGGGGGGGCGGTGGAGCCCGGGG<br>(((......)))	MUC1	NM_001044391	chr1:153424924-153429330	template	27	Intron_2	--	Yes	--	Yes	--	--	--	--	--	--	--
G4ST01153427942	chr1:153427943-153427969	+	GGGCTGGGGGGGCGGTGGAGCCCGGGG<br>(((......)))	MUC1	NM_001044392	chr1:153424924-153429330	template	27	Intron_2	--	Yes	--	Yes	--	--	--	--	--	--	--
G4ST01153427942	chr1:153427943-153427969	+	GGGCTGGGGGGGCGGTGGAGCCCGGGG<br>(((......)))	MUC1	NM_001204293	chr1:153424924-153429330	template	27	Intron_2	--	Yes	--	Yes	--	--	--	--	--	--	--
G4ST01153427942	chr1:153427943-153427969	+	GGGCTGGGGGGGCGGTGGAGCCCGGGG<br>(((......)))	MUC1	NM_001018016	chr1:153424924-153429330	template	27	Intron_2	--	Yes	--	Yes	--	--	--	--	--	--	--
G4ST01153427942	chr1:153427943-153427969	+	GGGCTGGGGGGGCGGTGGAGCCCGGGG<br>(((......)))	MUC1	NM_001044390	chr1:153424924-153429330	template	27	Intron_2	--	Yes	--	Yes	--	--	--	--	--	--	--
G4ST01153427942	chr1:153427943-153427969	+	GGGCTGGGGGGGCGGTGGAGCCCGGGG<br>(((......)))	MUC1	NM_001204285	chr1:153424924-153429330	template	27	Intron_3	--	Yes	--	Yes	--	--	--	--	--	--	--
G4ST01153427942	chr1:153427943-153427969	+	GGGCTGGGGGGGCGGTGGAGCCCGGGG<br>(((......)))	MUC1	NM_001204294	chr1:153424924-153429330	template	27	Intron_2	--	Yes	--	Yes	--	--	--	--	--	--	--
G4ST01153427942	chr1:153427943-153427969	+	GGGCTGGGGGGGCGGTGGAGCCCGGGG<br>(((......)))	MUC1	NM_001204286	chr1:153424924-153429330	template	27	Intron_3	--	Yes	--	Yes	--	--	--	--	--	--	--
G4ST01153427942	chr1:153427943-153427969	+	GGGCTGGGGGGGCGGTGGAGCCCGGGG<br>(((......)))	MUC1	NM_001018017	chr1:153424924-153429330	template	27	Intron_2	--	Yes	--	Yes	--	--	--	--	--	--	--
G4ST01153427942	chr1:153427943-153427969	+	GGGCTGGGGGGGCGGTGGAGCCCGGGG<br>(((......)))	MUC1	NM_001204297	chr1:153424924-153429330	template	27	Intron_2	--	Yes	--	Yes	--	--	--	--	--	--	--
G4ST01153428002	chr1:153428003-153428029	+	GGGCTGGGGGGGCGGTGGAGCCCGGGG<br>(((......)))	MUC1	NM_001204289	chr1:153424924-153429330	template	27	Intron_2	--	Yes	--	Yes	--	--	--	--	--	--	--
G4ST01153428002	chr1:153428003-153428029	+	GGGCTGGGGGGGCGGTGGAGCCCGGGG<br>(((......)))	MUC1	NM_001204287	chr1:153424924-153429330	template	27	Intron_2	--	Yes	--	Yes	--	--	--	--	--	--	--
G4ST01153428002	chr1:153428003-153428029	+	GGGCTGGGGGGGCGGTGGAGCCCGGGG<br>(((......)))	MUC1	NM_001204288	chr1:153424924-153429330	template	27	Intron_2	--	Yes	--	Yes	--	--	--	--	--	--	--
G4ST01153428002	chr1:153428003-153428029	+	GGGCTGGGGGGGCGGTGGAGCCCGGGG<br>(((......)))	MUC1	NM_001044393	chr1:153424924-153429330	template	27	Intron_2	--	Yes	--	Yes	--	--	--	--	--	--	--
G4ST01153428002	chr1:153428003-153428029	+	GGGCTGGGGGGGCGGTGGAGCCCGGGG<br>(((......)))	MUC1	NM_002456	chr1:153424924-153429330	template	27	Intron_2	--	Yes	--	Yes	--	--	--	--	--	--	--
G4ST01153428002	chr1:153428003-153428029	+	GGGCTGGGGGGGCGGTGGAGCCCGGGG<br>(((......)))	MUC1	NM_001204295	chr1:153424924-153429330	template	27	Intron_2	--	Yes	--	Yes	--	--	--	--	--	--	--
G4ST01153428002	chr1:153428003-153428029	+	GGGCTGGGGGGGCGGTGGAGCCCGGGG<br>(((......)))	MUC1	NM_001204296	chr1:153424924-153429330	template	27	Intron_2	--	Yes	--	Yes	--	--	--	--	--	--	--
G4ST01153428002	chr1:153428003-153428029	+	GGGCTGGGGGGGCGGTGGAGCCCGGGG<br>(((......)))	MUC1	NM_001204292	chr1:153424924-153429330	template	27	Intron_2	--	Yes	--	Yes	--	--	--	--	--	--	--
G4ST01153428002	chr1:153428003-153428029	+	GGGCTGGGGGGGCGGTGGAGCCCGGGG<br>(((......)))	MUC1	NM_001204291	chr1:153424924-153429330	template	27	Intron_2	--	Yes	--	Yes	--	--	--	--	--	--	--
G4ST01153428002	chr1:153428003-153428029	+	GGGCTGGGGGGGCGGTGGAGCCCGGGG<br>(((......)))	MUC1	NM_001204290	chr1:153424924-153429330	template	27	Intron_2	--	Yes	--	Yes	--	--	--	--	--	--	--
G4ST01153428002	chr1:153428003-153428029	+	GGGCTGGGGGGGCGGTGGAGCCCGGGG<br>(((......)))	MUC1	NM_001044391	chr1:153424924-153429330	template	27	Intron_2	--	Yes	--	Yes	--	--	--	--	--	--	--
G4ST01153428002	chr1:153428003-153428029	+	GGGCTGGGGGGGCGGTGGAGCCCGGGG<br>(((......)))	MUC1	NM_001044392	chr1:153424924-153429330	template	27	Intron_2	--	Yes	--	Yes	--	--	--	--	--	--	--
G4ST01153428002	chr1:153428003-153428029	+	GGGCTGGGGGGGCGGTGGAGCCCGGGG<br>(((......)))	MUC1	NM_001204293	chr1:153424924-153429330	template	27	Intron_2	--	Yes	--	Yes	--	--	--	--	--	--	--
G4ST01153428002	chr1:153428003-153428029	+	GGGCTGGGGGGGCGGTGGAGCCCGGGG<br>(((......)))	MUC1	NM_001018016	chr1:153424924-153429330	template	27	Intron_2	--	Yes	--	Yes	--	--	--	--	--	--	--
G4ST01153428002	chr1:153428003-153428029	+	GGGCTGGGGGGGCGGTGGAGCCCGGGG<br>(((......)))	MUC1	NM_001044390	chr1:153424924-153429330	template	27	Intron_2	--	Yes	--	Yes	--	--	--	--	--	--	--

G4ST01153428002	chr1:153428003-153428029	+	GGGCTGGGGGGGCGGTGGAGCCCGGGG<br>(((......)))	MUC1	NM_001204285	chr1:153424924-153429330	template	27	Intron_3	--	Yes	--	Yes	--	--	--	--	--	--	--
G4ST01153428002	chr1:153428003-153428029	+	GGGCTGGGGGGGCGGTGGAGCCCGGGG<br>(((......)))	MUC1	NM_001204294	chr1:153424924-153429330	template	27	Intron_2	--	Yes	--	Yes	--	--	--	--	--	--	--
G4ST01153428002	chr1:153428003-153428029	+	GGGCTGGGGGGGCGGTGGAGCCCGGGG<br>(((......)))	MUC1	NM_001204286	chr1:153424924-153429330	template	27	Intron_3	--	Yes	--	Yes	--	--	--	--	--	--	--
G4ST01153428002	chr1:153428003-153428029	+	GGGCTGGGGGGGCGGTGGAGCCCGGGG<br>(((......)))	MUC1	NM_001018017	chr1:153424924-153429330	template	27	Intron_2	--	Yes	--	Yes	--	--	--	--	--	--	--
G4ST01153428002	chr1:153428003-153428029	+	GGGCTGGGGGGGCGGTGGAGCCCGGGG<br>(((......)))	MUC1	NM_001204297	chr1:153424924-153429330	template	27	Intron_2	--	Yes	--	Yes	--	--	--	--	--	--	--
G4ST01153428062	chr1:153428063-153428089	+	GGGCTGGGGGGGCGGTGGAGCCCGGGG<br>(((......)))	MUC1	NM_001204289	chr1:153424924-153429330	template	27	Intron_2	--	Yes	--	Yes	--	--	--	--	--	--	--
G4ST01153428062	chr1:153428063-153428089	+	GGGCTGGGGGGGCGGTGGAGCCCGGGG<br>(((......)))	MUC1	NM_001204287	chr1:153424924-153429330	template	27	Intron_2	--	Yes	--	Yes	--	--	--	--	--	--	--
G4ST01153428062	chr1:153428063-153428089	+	GGGCTGGGGGGGCGGTGGAGCCCGGGG<br>(((......)))	MUC1	NM_001204288	chr1:153424924-153429330	template	27	Intron_2	--	Yes	--	Yes	--	--	--	--	--	--	--
G4ST01153428062	chr1:153428063-153428089	+	GGGCTGGGGGGGCGGTGGAGCCCGGGG<br>(((......)))	MUC1	NM_001044393	chr1:153424924-153429330	template	27	Intron_2	--	Yes	--	Yes	--	--	--	--	--	--	--
G4ST01153428062	chr1:153428063-153428089	+	GGGCTGGGGGGGCGGTGGAGCCCGGGG<br>(((......)))	MUC1	NM_002456	chr1:153424924-153429330	template	27	Intron_2	--	Yes	--	Yes	--	--	--	--	--	--	--
G4ST01153428062	chr1:153428063-153428089	+	GGGCTGGGGGGGCGGTGGAGCCCGGGG<br>(((......)))	MUC1	NM_001204295	chr1:153424924-153429330	template	27	Intron_2	--	Yes	--	Yes	--	--	--	--	--	--	--
G4ST01153428062	chr1:153428063-153428089	+	GGGCTGGGGGGGCGGTGGAGCCCGGGG<br>(((......)))	MUC1	NM_001204296	chr1:153424924-153429330	template	27	Intron_2	--	Yes	--	Yes	--	--	--	--	--	--	--
G4ST01153428062	chr1:153428063-153428089	+	GGGCTGGGGGGGCGGTGGAGCCCGGGG<br>(((......)))	MUC1	NM_001204292	chr1:153424924-153429330	template	27	Intron_2	--	Yes	--	Yes	--	--	--	--	--	--	--
G4ST01153428062	chr1:153428063-153428089	+	GGGCTGGGGGGGCGGTGGAGCCCGGGG<br>(((......)))	MUC1	NM_001204291	chr1:153424924-153429330	template	27	Intron_2	--	Yes	--	Yes	--	--	--	--	--	--	--
G4ST01153428062	chr1:153428063-153428089	+	GGGCTGGGGGGGCGGTGGAGCCCGGGG<br>(((......)))	MUC1	NM_001204290	chr1:153424924-153429330	template	27	Intron_2	--	Yes	--	Yes	--	--	--	--	--	--	--
G4ST01153428062	chr1:153428063-153428089	+	GGGCTGGGGGGGCGGTGGAGCCCGGGG<br>(((......)))	MUC1	NM_001044391	chr1:153424924-153429330	template	27	Intron_2	--	Yes	--	Yes	--	--	--	--	--	--	--
G4ST01153428062	chr1:153428063-153428089	+	GGGCTGGGGGGGCGGTGGAGCCCGGGG<br>(((......)))	MUC1	NM_001044392	chr1:153424924-153429330	template	27	Intron_2	--	Yes	--	Yes	--	--	--	--	--	--	--
G4ST01153428062	chr1:153428063-153428089	+	GGGCTGGGGGGGCGGTGGAGCCCGGGG<br>(((......)))	MUC1	NM_001204293	chr1:153424924-153429330	template	27	Intron_2	--	Yes	--	Yes	--	--	--	--	--	--	--
G4ST01153428062	chr1:153428063-153428089	+	GGGCTGGGGGGGCGGTGGAGCCCGGGG<br>(((......)))	MUC1	NM_001018016	chr1:153424924-153429330	template	27	Intron_2	--	Yes	--	Yes	--	--	--	--	--	--	--
G4ST01153428062	chr1:153428063-153428089	+	GGGCTGGGGGGGCGGTGGAGCCCGGGG<br>(((......)))	MUC1	NM_001044390	chr1:153424924-153429330	template	27	Intron_2	--	Yes	--	Yes	--	--	--	--	--	--	--
G4ST01153428062	chr1:153428063-153428089	+	GGGCTGGGGGGGCGGTGGAGCCCGGGG<br>(((......)))	MUC1	NM_001204285	chr1:153424924-153429330	template	27	Exon_3-Intron_3	--	Yes	Yes	Yes	Yes	--	--	--	--	--	--
G4ST01153428062	chr1:153428063-153428089	+	GGGCTGGGGGGGCGGTGGAGCCCGGGG<br>(((......)))	MUC1	NM_001204294	chr1:153424924-153429330	template	27	Intron_2	--	Yes	--	Yes	--	--	--	--	--	--	--
G4ST01153428062	chr1:153428063-153428089	+	GGGCTGGGGGGGCGGTGGAGCCCGGGG<br>(((......)))	MUC1	NM_001204286	chr1:153424924-153429330	template	27	Exon_3-Intron_3	--	Yes	Yes	Yes	Yes	--	--	--	--	--	--
G4ST01153428062	chr1:153428063-153428089	+	GGGCTGGGGGGGCGGTGGAGCCCGGGG<br>(((......)))	MUC1	NM_001018017	chr1:153424924-153429330	template	27	Intron_2	--	Yes	--	Yes	--	--	--	--	--	--	--
G4ST01153428062	chr1:153428063-153428089	+	GGGCTGGGGGGGCGGTGGAGCCCGGGG<br>(((......)))	MUC1	NM_001204297	chr1:153424924-153429330	template	27	Intron_2	--	Yes	--	Yes	--	--	--	--	--	--	--
G4ST01153428122	chr1:153428123-153428149	+	GGGCTGGGGGGGCGGTGGAGCCCGGGG<br>(((......)))	MUC1	NM_001204289	chr1:153424924-153429330	template	27	Intron_2	--	Yes	--	Yes	--	--	--	--	--	--	--
G4ST01153428122	chr1:153428123-153428149	+	GGGCTGGGGGGGCGGTGGAGCCCGGGG<br>(((......)))	MUC1	NM_001204287	chr1:153424924-153429330	template	27	Intron_2	--	Yes	--	Yes	--	--	--	--	--	--	--
G4ST01153428122	chr1:153428123-153428149	+	GGGCTGGGGGGGCGGTGGAGCCCGGGG<br>(((......)))	MUC1	NM_001204288	chr1:153424924-153429330	template	27	Intron_2	--	Yes	--	Yes	--	--	--	--	--	--	--
G4ST01153428122	chr1:153428123-153428149	+	GGGCTGGGGGGGCGGTGGAGCCCGGGG<br>(((......)))	MUC1	NM_001044393	chr1:153424924-153429330	template	27	Intron_2	--	Yes	--	Yes	--	--	--	--	--	--	--
G4ST01153428122	chr1:153428123-153428149	+	GGGCTGGGGGGGCGGTGGAGCCCGGGG<br>(((......)))	MUC1	NM_002456	chr1:153424924-153429330	template	27	Intron_2	--	Yes	--	Yes	--	--	--	--	--	--	--
G4ST01153428122	chr1:153428123-153428149	+	GGGCTGGGGGGGCGGTGGAGCCCGGGG<br>(((......)))	MUC1	NM_001204295	chr1:153424924-153429330	template	27	Intron_2	--	Yes	--	Yes	--	--	--	--	--	--	--
G4ST01153428122	chr1:153428123-153428149	+	GGGCTGGGGGGGCGGTGGAGCCCGGGG<br>(((......)))	MUC1	NM_001204296	chr1:153424924-153429330	template	27	Intron_2	--	Yes	--	Yes	--	--	--	--	--	--	--
G4ST01153428122	chr1:153428123-153428149	+	GGGCTGGGGGGGCGGTGGAGCCCGGGG<br>(((......)))	MUC1	NM_001204292	chr1:153424924-153429330	template	27	Intron_2	--	Yes	--	Yes	--	--	--	--	--	--	--
G4ST01153428122	chr1:153428123-153428149	+	GGGCTGGGGGGGCGGTGGAGCCCGGGG<br>(((......)))	MUC1	NM_001204291	chr1:153424924-153429330	template	27	Intron_2	--	Yes	--	Yes	--	--	--	--	--	--	--
G4ST01153428122	chr1:153428123-153428149	+	GGGCTGGGGGGGCGGTGGAGCCCGGGG<br>(((......)))	MUC1	NM_001204290	chr1:153424924-153429330	template	27	Intron_2	--	Yes	--	Yes	--	--	--	--	--	--	--
G4ST01153428122	chr1:153428123-153428149	+	GGGCTGGGGGGGCGGTGGAGCCCGGGG<br>(((......)))	MUC1	NM_001044391	chr1:153424924-153429330	template	27	Intron_2	--	Yes	--	Yes	--	--	--	--	--	--	--
G4ST01153428122	chr1:153428123-153428149	+	GGGCTGGGGGGGCGGTGGAGCCCGGGG<br>(((......)))	MUC1	NM_001044392	chr1:153424924-153429330	template	27	Intron_2	--	Yes	--	Yes	--	--	--	--	--	--	--
G4ST01153428122	chr1:153428123-153428149	+	GGGCTGGGGGGGCGGTGGAGCCCGGGG<br>(((......)))	MUC1	NM_001204293	chr1:153424924-153429330	template	27	Intron_2	--	Yes	--	Yes	--	--	--	--	--	--	--
G4ST01153428122	chr1:153428123-153428149	+	GGGCTGGGGGGGCGGTGGAGCCCGGGG<br>(((......)))	MUC1	NM_001018016	chr1:153424924-153429330	template	27	Intron_2	--	Yes	--	Yes	--	--	--	--	--	--	--
G4ST01153428122	chr1:153428123-153428149	+	GGGCTGGGGGGGCGGTGGAGCCCGGGG<br>(((......)))	MUC1	NM_001044390	chr1:153424924-153429330	template	27	Intron_2	--	Yes	--	Yes	--	--	--	--	--	--	--
G4ST01153428122	chr1:153428123-153428149	+	GGGCTGGGGGGGCGGTGGAGCCCGGGG<br>(((......)))	MUC1	NM_001204285	chr1:153424924-153429330	template	27	Intron_2	--	Yes	--	Yes	--	--	--	--	--	--	--
G4ST01153428122	chr1:153428123-153428149	+	GGGCTGGGGGGGCGGTGGAGCCCGGGG<br>(((......)))	MUC1	NM_001204294	chr1:153424924-153429330	template	27	Intron_2	--	Yes	--	Yes	--	--	--	--	--	--	--
G4ST01153428122	chr1:153428123-153428149	+	GGGCTGGGGGGGCGGTGGAGCCCGGGG<br>(((......)))	MUC1	NM_001204286	chr1:153424924-153429330	template	27	Intron_2	--	Yes	--	Yes	--	--	--	--	--	--	--
G4ST01153428122	chr1:153428123-153428149	+	GGGCTGGGGGGGCGGTGGAGCCCGGGG<br>(((......)))	MUC1	NM_001018017	chr1:153424924-153429330	template	27	Intron_2	--	Yes	--	Yes	--	--	--	--	--	--	--
G4ST01153428122	chr1:153428123-153428149	+	GGGCTGGGGGGGCGGTGGAGCCCGGGG<br>(((......)))	MUC1	NM_001204297	chr1:153424924-153429330	template	27	Intron_2	--	Yes	--	Yes	--	--	--	--	--	--	--
G4ST01153428182	chr1:153428183-153428209	+	GGGCTGGGGGGGCGGTGGAGCCCGGGG<br>(((......)))	MUC1	NM_001204289	chr1:153424924-153429330	template	27	Intron_2	--	Yes	--	Yes	--	--	--	--	--	--	--
G4ST01153428182	chr1:153428183-153428209	+	GGGCTGGGGGGGCGGTGGAGCCCGGGG<br>(((......)))	MUC1	NM_001204287	chr1:153424924-153429330	template	27	Intron_2	--	Yes	--	Yes	--	--	--	--	--	--	--
G4ST01153428182	chr1:153428183-153428209	+	GGGCTGGGGGGGCGGTGGAGCCCGGGG<br>(((......)))	MUC1	NM_001204288	chr1:153424924-153429330	template	27	Intron_2	--	Yes	--	Yes	--	--	--	--	--	--	--
G4ST01153428182	chr1:153428183-153428209	+	GGGCTGGGGGGGCGGTGGAGCCCGGGG<br>(((......)))	MUC1	NM_001044393	chr1:153424924-153429330	template	27	Intron_2	--	Yes	--	Yes	--	--	--	--	--	--	--
G4ST01153428182	chr1:153428183-153428209	+	GGGCTGGGGGGGCGGTGGAGCCCGGGG<br>(((......)))	MUC1	NM_002456	chr1:153424924-153429330	template	27	Intron_2	--	Yes	--	Yes	--	--	--	--	--	--	--

G4ST01153428182	chr1:153428183-153428209	+	GGGCTGGGGGGGCGGTGGAGCCCGGGG<br>(((......)))	MUC1	NM_001204295	chr1:153424924-153429330	template	27	Intron_2	--	Yes	--	Yes	--	--	--	--	--	--	--
G4ST01153428182	chr1:153428183-153428209	+	GGGCTGGGGGGGCGGTGGAGCCCGGGG<br>(((......)))	MUC1	NM_001204296	chr1:153424924-153429330	template	27	Intron_2	--	Yes	--	Yes	--	--	--	--	--	--	--
G4ST01153428182	chr1:153428183-153428209	+	GGGCTGGGGGGGCGGTGGAGCCCGGGG<br>(((......)))	MUC1	NM_001204292	chr1:153424924-153429330	template	27	Intron_2	--	Yes	--	Yes	--	--	--	--	--	--	--
G4ST01153428182	chr1:153428183-153428209	+	GGGCTGGGGGGGCGGTGGAGCCCGGGG<br>(((......)))	MUC1	NM_001204291	chr1:153424924-153429330	template	27	Intron_2	--	Yes	--	Yes	--	--	--	--	--	--	--
G4ST01153428182	chr1:153428183-153428209	+	GGGCTGGGGGGGCGGTGGAGCCCGGGG<br>(((......)))	MUC1	NM_001204290	chr1:153424924-153429330	template	27	Intron_2	--	Yes	--	Yes	--	--	--	--	--	--	--
G4ST01153428182	chr1:153428183-153428209	+	GGGCTGGGGGGGCGGTGGAGCCCGGGG<br>(((......)))	MUC1	NM_001044391	chr1:153424924-153429330	template	27	Intron_2	--	Yes	--	Yes	--	--	--	--	--	--	--
G4ST01153428182	chr1:153428183-153428209	+	GGGCTGGGGGGGCGGTGGAGCCCGGGG<br>(((......)))	MUC1	NM_001044392	chr1:153424924-153429330	template	27	Intron_2	--	Yes	--	Yes	--	--	--	--	--	--	--
G4ST01153428182	chr1:153428183-153428209	+	GGGCTGGGGGGGCGGTGGAGCCCGGGG<br>(((......)))	MUC1	NM_001204293	chr1:153424924-153429330	template	27	Intron_2	--	Yes	--	Yes	--	--	--	--	--	--	--
G4ST01153428182	chr1:153428183-153428209	+	GGGCTGGGGGGGCGGTGGAGCCCGGGG<br>(((......)))	MUC1	NM_001018016	chr1:153424924-153429330	template	27	Intron_2	--	Yes	--	Yes	--	--	--	--	--	--	--
G4ST01153428182	chr1:153428183-153428209	+	GGGCTGGGGGGGCGGTGGAGCCCGGGG<br>(((......)))	MUC1	NM_001044390	chr1:153424924-153429330	template	27	Intron_2	--	Yes	--	Yes	--	--	--	--	--	--	--
G4ST01153428182	chr1:153428183-153428209	+	GGGCTGGGGGGGCGGTGGAGCCCGGGG<br>(((......)))	MUC1	NM_001204285	chr1:153424924-153429330	template	27	Intron_2	--	Yes	--	Yes	--	--	--	--	--	--	--
G4ST01153428182	chr1:153428183-153428209	+	GGGCTGGGGGGGCGGTGGAGCCCGGGG<br>(((......)))	MUC1	NM_001204294	chr1:153424924-153429330	template	27	Intron_2	--	Yes	--	Yes	--	--	--	--	--	--	--
G4ST01153428182	chr1:153428183-153428209	+	GGGCTGGGGGGGCGGTGGAGCCCGGGG<br>(((......)))	MUC1	NM_001204286	chr1:153424924-153429330	template	27	Intron_2	--	Yes	--	Yes	--	--	--	--	--	--	--
G4ST01153428182	chr1:153428183-153428209	+	GGGCTGGGGGGGCGGTGGAGCCCGGGG<br>(((......)))	MUC1	NM_001018017	chr1:153424924-153429330	template	27	Intron_2	--	Yes	--	Yes	--	--	--	--	--	--	--
G4ST01153428182	chr1:153428183-153428209	+	GGGCTGGGGGGGCGGTGGAGCCCGGGG<br>(((......)))	MUC1	NM_001204297	chr1:153424924-153429330	template	27	Intron_2	--	Yes	--	Yes	--	--	--	--	--	--	--
G4ST01153428350	chr1:153428351-153428377	+	GGGCTGGGGGGGCGGTGGAGCCCGGGG<br>(((......)))	MUC1	NM_001204289	chr1:153424924-153429330	template	27	Intron_2	--	Yes	--	Yes	--	--	--	--	--	--	--
G4ST01153428350	chr1:153428351-153428377	+	GGGCTGGGGGGGCGGTGGAGCCCGGGG<br>(((......)))	MUC1	NM_001204287	chr1:153424924-153429330	template	27	Intron_2	--	Yes	--	Yes	--	--	--	--	--	--	--
G4ST01153428350	chr1:153428351-153428377	+	GGGCTGGGGGGGCGGTGGAGCCCGGGG<br>(((......)))	MUC1	NM_001204288	chr1:153424924-153429330	template	27	Intron_2	--	Yes	--	Yes	--	--	--	--	--	--	--
G4ST01153428350	chr1:153428351-153428377	+	GGGCTGGGGGGGCGGTGGAGCCCGGGG<br>(((......)))	MUC1	NM_001044393	chr1:153424924-153429330	template	27	Intron_2	--	Yes	--	Yes	--	--	--	--	--	--	--
G4ST01153428350	chr1:153428351-153428377	+	GGGCTGGGGGGGCGGTGGAGCCCGGGG<br>(((......)))	MUC1	NM_002456	chr1:153424924-153429330	template	27	Intron_2	--	Yes	--	Yes	--	--	--	--	--	--	--
G4ST01153428350	chr1:153428351-153428377	+	GGGCTGGGGGGGCGGTGGAGCCCGGGG<br>(((......)))	MUC1	NM_001204295	chr1:153424924-153429330	template	27	Intron_2	--	Yes	--	Yes	--	--	--	--	--	--	--
G4ST01153428350	chr1:153428351-153428377	+	GGGCTGGGGGGGCGGTGGAGCCCGGGG<br>(((......)))	MUC1	NM_001204296	chr1:153424924-153429330	template	27	Intron_2	--	Yes	--	Yes	--	--	--	--	--	--	--
G4ST01153428350	chr1:153428351-153428377	+	GGGCTGGGGGGGCGGTGGAGCCCGGGG<br>(((......)))	MUC1	NM_001204292	chr1:153424924-153429330	template	27	Intron_2	--	Yes	--	Yes	--	--	--	--	--	--	--
G4ST01153428350	chr1:153428351-153428377	+	GGGCTGGGGGGGCGGTGGAGCCCGGGG<br>(((......)))	MUC1	NM_001204291	chr1:153424924-153429330	template	27	Intron_2	--	Yes	--	Yes	--	--	--	--	--	--	--
G4ST01153428350	chr1:153428351-153428377	+	GGGCTGGGGGGGCGGTGGAGCCCGGGG<br>(((......)))	MUC1	NM_001204290	chr1:153424924-153429330	template	27	Intron_2	--	Yes	--	Yes	--	--	--	--	--	--	--
G4ST01153428350	chr1:153428351-153428377	+	GGGCTGGGGGGGCGGTGGAGCCCGGGG<br>(((......)))	MUC1	NM_001044391	chr1:153424924-153429330	template	27	Intron_2	--	Yes	--	Yes	--	--	--	--	--	--	--
G4ST01153428350	chr1:153428351-153428377	+	GGGCTGGGGGGGCGGTGGAGCCCGGGG<br>(((......)))	MUC1	NM_001044392	chr1:153424924-153429330	template	27	Intron_2	--	Yes	--	Yes	--	--	--	--	--	--	--
G4ST01153428350	chr1:153428351-153428377	+	GGGCTGGGGGGGCGGTGGAGCCCGGGG<br>(((......)))	MUC1	NM_001204293	chr1:153424924-153429330	template	27	Intron_2	--	Yes	--	Yes	--	--	--	--	--	--	--
G4ST01153428350	chr1:153428351-153428377	+	GGGCTGGGGGGGCGGTGGAGCCCGGGG<br>(((......)))	MUC1	NM_001018016	chr1:153424924-153429330	template	27	Intron_2	--	Yes	--	Yes	--	--	--	--	--	--	--
G4ST01153428350	chr1:153428351-153428377	+	GGGCTGGGGGGGCGGTGGAGCCCGGGG<br>(((......)))	MUC1	NM_001044390	chr1:153424924-153429330	template	27	Intron_2	--	Yes	--	Yes	--	--	--	--	--	--	--
G4ST01153428350	chr1:153428351-153428377	+	GGGCTGGGGGGGCGGTGGAGCCCGGGG<br>(((......)))	MUC1	NM_001204285	chr1:153424924-153429330	template	27	Exon_2	--	Yes	Yes	--	Yes	--	--	--	--	--	--
G4ST01153428350	chr1:153428351-153428377	+	GGGCTGGGGGGGCGGTGGAGCCCGGGG<br>(((......)))	MUC1	NM_001204294	chr1:153424924-153429330	template	27	Intron_2	--	Yes	--	Yes	--	--	--	--	--	--	--
G4ST01153428350	chr1:153428351-153428377	+	GGGCTGGGGGGGCGGTGGAGCCCGGGG<br>(((......)))	MUC1	NM_001204286	chr1:153424924-153429330	template	27	Exon_2	--	Yes	Yes	--	Yes	--	--	--	--	--	--
G4ST01153428350	chr1:153428351-153428377	+	GGGCTGGGGGGGCGGTGGAGCCCGGGG<br>(((......)))	MUC1	NM_001018017	chr1:153424924-153429330	template	27	Intron_2	--	Yes	--	Yes	--	--	--	--	--	--	--
G4ST01153428350	chr1:153428351-153428377	+	GGGCTGGGGGGGCGGTGGAGCCCGGGG<br>(((......)))	MUC1	NM_001204297	chr1:153424924-153429330	template	27	Intron_2	--	Yes	--	Yes	--	--	--	--	--	--	--
G4ST01153437004	chr1:153437005-153437042	+	GGGGAACAGCCGGCAGTTGTCCTGGGCAGAGGGGTGGG<br>..(((((.....)))))...	THBS3	NM_007112	chr1:153432003-153444314	template	38	Intron_13-Exon_14	--	Yes	Yes	Yes	Yes	--	--	--	--	--	Yes
G4ST01153437459	chr1:153437460-153437493	-	GGGCAAGGGGGCGTAGTGGGGAGCCCAAGCTGGG<br>.(((....)))	THBS3	NM_007112	chr1:153432003-153444314	non-template	34	Intron_12	--	Yes	--	Yes	--	--	--	--	--	--	Yes
G4ST01153443816	chr1:153443817-153443853	-	GGGCTCCTGGCTGCCCAAGGACAGGGCGGGGGTGGGG<br>..(((((....)).)))...	MTX1	NM_002455	chr1:153445114-153450238	template	-1260	upstream	Yes	--	--	--	--	--	--	Yes	Yes	--	--
G4ST01153443816	chr1:153443817-153443853	-	GGGCTCCTGGCTGCCCAAGGACAGGGCGGGGGTGGGG<br>..(((((....)).)))...	MTX1	NM_198883	chr1:153445114-153450248	template	-1260	upstream	Yes	--	--	--	--	--	--	Yes	Yes	--	--
G4ST01153443816	chr1:153443817-153443853	-	GGGCTCCTGGCTGCCCAAGGACAGGGCGGGGGTGGGG<br>..(((((....)).)))...	THBS3	NM_007112	chr1:153432003-153444314	non-template	37	Intron_1.f	--	Yes	--	Yes	--	--	--	Yes	Yes	--	--
G4ST01153445234	chr1:153445235-153445267	-	GGGGCTCGTCCCCGAGCGGGGACTGCGGGGGGG<br>(((((....))))).	MTX1	NM_002455	chr1:153445114-153450238	template	33	Exon_1.f	--	Yes	Yes	--	Yes	--	--	Yes	Yes	Yes	--
G4ST01153445234	chr1:153445235-153445267	-	GGGGCTCGTCCCCGAGCGGGGACTGCGGGGGGG<br>(((((....))))).	MTX1	NM_198883	chr1:153445114-153450248	template	33	Exon_1.f	--	Yes	Yes	--	Yes	--	--	Yes	Yes	Yes	--
G4ST01153445234	chr1:153445235-153445267	-	GGGGCTCGTCCCCGAGCGGGGACTGCGGGGGGG<br>(((((....))))).	THBS3	NM_007112	chr1:153432003-153444314	non-template	-920	upstream	Yes	--	--	--	--	--	--	Yes	Yes	Yes	--
G4ST01153445238	chr1:153445239-153445276	-	GGGCCCCTTGGGGCTCGTCCCCGAGCGGGGACTGCGGG<br>(((((....))))).	MTX1	NM_002455	chr1:153445114-153450238	template	38	Exon_1.f	--	Yes	Yes	--	Yes	--	--	Yes	Yes	Yes	--
G4ST01153445238	chr1:153445239-153445276	-	GGGCCCCTTGGGGCTCGTCCCCGAGCGGGGACTGCGGG<br>(((((....))))).	MTX1	NM_198883	chr1:153445114-153450248	template	38	Exon_1.f	--	Yes	Yes	--	Yes	--	--	Yes	Yes	Yes	--
G4ST01153445238	chr1:153445239-153445276	-	GGGCCCCTTGGGGCTCGTCCCCGAGCGGGGACTGCGGG<br>(((((....))))).	THBS3	NM_007112	chr1:153432003-153444314	non-template	-924	upstream	Yes	--	--	--	--	--	--	Yes	Yes	Yes	--
G4ST01153447955	chr1:153447956-153447991	+	GGGAAGGTAGGGCAGGCTGAGACCTGGGGGTATGGG<br>((((......)))).	MTX1	NM_002455	chr1:153445114-153450238	non-template	36	Intron_3	--	Yes	--	Yes	--	--	--	Yes	--	--	--
G4ST01153447955	chr1:153447956-153447991	+	GGGAAGGTAGGGCAGGCTGAGACCTGGGGGTATGGG<br>((((......)))).	MTX1	NM_198883	chr1:153445114-153450248	non-template	36	Intron_3	--	Yes	--	Yes	--	--	--	Yes	--	--	--
G4ST01153454155	chr1:153454156-153454188	+	GGGCAGGGGGTGACAGGGCAAGGATGTTGAGGG<br>(((.....))).	GBAP1	NR_002188	chr1:153450240-153463949	template	33	Intron_4	--	Yes	--	Yes	--	--	--	--	Yes	--	--
G4ST01153478205	chr1:153478206-153478236	+	GGGCACAAGAGGGTGGGACACTGGCAGTGGG<br>..(((...)))	GBA	NM_001171812	chr1:153470863-153477690	template	-515	upstream	Yes	--	--	--	--	--	--	--	Yes	Yes	--

G4ST01153478205	chr1:153478206-153478236	+	GGGCACAAGAGGGTGGGACACTGGCAGTGGG<br>..(((...)))	GBA	NM_001171811	chr1:153470863-153481277	template	31	Intron_1.f	--	Yes	--	Yes	--	--	--	--	Yes	Yes	--
G4ST01153478205	chr1:153478206-153478236	+	GGGCACAAGAGGGTGGGACACTGGCAGTGGG<br>..(((...)))	GBA	NM_000157	chr1:153470863-153477693	template	-512	upstream	Yes	--	--	--	--	--	--	--	Yes	Yes	--
G4ST01153478205	chr1:153478206-153478236	+	GGGCACAAGAGGGTGGGACACTGGCAGTGGG<br>..(((...)))	GBA	NM_001005742	chr1:153470863-153481277	template	31	Intron_1.f	--	Yes	--	Yes	--	--	--	--	Yes	Yes	--
G4ST01153478205	chr1:153478206-153478236	+	GGGCACAAGAGGGTGGGACACTGGCAGTGGG<br>..(((...)))	GBA	NM_001005741	chr1:153470863-153481277	template	31	Intron_1.f	--	Yes	--	Yes	--	--	--	--	Yes	Yes	--
G4ST01153490259	chr1:153490260-153490314	+	GGGTGCAAGCTCACCTTGAGGGCCCCTCGGGCTTCATCACAGGTGGAGTTAGGGG<br>..((((.....)))). ((((((.....))))))....	FAM189B	NM_006589	chr1:153483620-153491898	template	55	Exon_4-Intron_4	--	Yes	Yes	Yes	Yes	--	--	Yes	--	--	--
G4ST01153490259	chr1:153490260-153490314	+	GGGTGCAAGCTCACCTTGAGGGCCCCTCGGGCTTCATCACAGGTGGAGTTAGGGG<br>..((((.....)))). ((((((.....))))))....	FAM189B	NM_198264	chr1:153483620-153491898	template	55	Intron_1.f	--	Yes	--	Yes	--	--	--	Yes	--	--	--
G4ST01153493027	chr1:153493028-153493062	-	GGGATGGTGAGATCATGGGTCCCCAGGGGCGTGGG<br>((((.....))))	SCAMP3	NM_052837	chr1:153492396-153498819	non-template	35	Intron_7.l	--	Yes	--	Yes	--	--	--	Yes	--	--	--
G4ST01153493027	chr1:153493028-153493062	-	GGGATGGTGAGATCATGGGTCCCCAGGGGCGTGGG<br>((((.....))))	SCAMP3	NM_005698	chr1:153492394-153498819	non-template	35	Intron_8.l	--	Yes	--	Yes	--	--	--	Yes	--	--	--
G4ST01153493027	chr1:153493028-153493062	-	GGGATGGTGAGATCATGGGTCCCCAGGGGCGTGGG<br>((((.....))))	FAM189B	NM_006589	chr1:153483620-153491898	non-template	-1129	upstream	Yes	--	--	--	--	--	--	Yes	--	--	--
G4ST01153493027	chr1:153493028-153493062	-	GGGATGGTGAGATCATGGGTCCCCAGGGGCGTGGG<br>((((.....))))	FAM189B	NM_198264	chr1:153483620-153491898	non-template	-1129	upstream	Yes	--	--	--	--	--	--	Yes	--	--	--
G4ST01153493033	chr1:153493034-153493068	-	GGGGCCGGGATGGTGAGATCATGGGTCCCCAGGGG<br>((((.....))))	SCAMP3	NM_052837	chr1:153492396-153498819	non-template	35	Intron_7.l	--	Yes	--	Yes	--	--	--	Yes	--	--	--
G4ST01153493033	chr1:153493034-153493068	-	GGGGCCGGGATGGTGAGATCATGGGTCCCCAGGGG<br>((((.....))))	SCAMP3	NM_005698	chr1:153492394-153498819	non-template	35	Intron_8.l	--	Yes	--	Yes	--	--	--	Yes	--	--	--
G4ST01153493033	chr1:153493034-153493068	-	GGGGCCGGGATGGTGAGATCATGGGTCCCCAGGGG<br>((((.....))))	FAM189B	NM_006589	chr1:153483842-153491898	non-template	-1135	upstream	Yes	--	--	--	--	--	--	Yes	--	--	--
G4ST01153493033	chr1:153493034-153493068	-	GGGGCCGGGATGGTGAGATCATGGGTCCCCAGGGG<br>((((.....))))	FAM189B	NM_198264	chr1:153483620-153491898	non-template	-1135	upstream	Yes	--	--	--	--	--	--	Yes	--	--	--
G4ST01153522375	chr1:153522376-153522408	+	GGGGGTCAGCAGGCCTCAGGGAGGGTGGCAGGG<br>((((....))))...	HCN3	NM_020897	chr1:153513998-153526262	non-template	33	Exon_6-Intron_6	--	Yes	Yes	Yes	Yes	--	--	--	--	--	--
G4ST01153524439	chr1:153524440-153524474	-	GGGTGGCTGGAGAGTCAGGGGAGAGGGGCAGAGGG<br>...((((....))))	HCN3	NM_020897	chr1:153513998-153526262	template	35	Exon_8.l	--	Yes	Yes	--	Yes	--	--	--	Yes	Yes	--
G4ST01153526945	chr1:153526946-153526977	+	GGGGTACAAGGGTAGGCTGGGCCAGAGGAGGG<br>((...)).	PKLR	NM_181871	chr1:153525708-153537416	template	32	Exon_11.l	--	Yes	Yes	--	--	--	Yes	--	--	--	Yes
G4ST01153526945	chr1:153526946-153526977	+	GGGGTACAAGGGTAGGCTGGGCCAGAGGAGGG<br>((...)).	PKLR	NM_000298	chr1:153525708-153537849	template	32	Exon_11.l	--	Yes	Yes	--	--	--	Yes	--	--	--	Yes
G4ST01153526954	chr1:153526955-153526982	+	GGGTAGGCTGGGCCAGAGGAGGGAGGGG<br>((...)).	PKLR	NM_181871	chr1:153525708-153537416	template	28	Exon_11.l	--	Yes	Yes	--	--	--	Yes	--	--	--	Yes
G4ST01153526954	chr1:153526955-153526982	+	GGGTAGGCTGGGCCAGAGGAGGGAGGGG<br>((...)).	PKLR	NM_000298	chr1:153525708-153537849	template	28	Exon_11.l	--	Yes	Yes	--	--	--	Yes	--	--	--	Yes
G4ST01153531752	chr1:153531753-153531780	-	GGGCAGGGGCGGGTCCCGGACTCCGGGG<br>...(((...)))	PKLR	NM_181871	chr1:153525708-153537416	non-template	28	Intron_4	--	Yes	--	Yes	--	--	--	--	--	--	--
G4ST01153531752	chr1:153531753-153531780	-	GGGCAGGGGCGGGTCCCGGACTCCGGGG<br>...(((...)))	PKLR	NM_000298	chr1:153525708-153537849	non-template	28	Intron_4	--	Yes	--	Yes	--	--	--	--	--	--	--
G4ST01153537932	chr1:153537933-153537973	-	GGGCAGAGAGCAGGCCGTTCTGGGGGAGGGATTCTGTGGGG<br>...(((((.....)))))...	PKLR	NM_181871	chr1:153525708-153537416	non-template	-516	upstream	Yes	--	--	--	--	--	--	Yes	Yes	Yes	Yes
G4ST01153537932	chr1:153537933-153537973	-	GGGCAGAGAGCAGGCCGTTCTGGGGGAGGGATTCTGTGGGG<br>...(((((.....)))))...	PKLR	NM_000298	chr1:153525708-153537849	non-template	-83	upstream	Yes	--	--	--	--	--	--	Yes	Yes	Yes	Yes
G4ST01153548870	chr1:153548871-153548902	+	GGGGAGGGATGGGCCTGAAACTTATTTCTGGG<br>...((((....)))).	FDPS	NM_001135822	chr1:153545163-153557081	non-template	32	Intron_3	--	Yes	--	Yes	--	--	--	--	Yes	Yes	Yes
G4ST01153548870	chr1:153548871-153548902	+	GGGGAGGGATGGGCCTGAAACTTATTTCTGGG<br>...((((....)))).	FDPS	NM_001135821	chr1:153545329-153557081	non-template	32	Intron_4	--	Yes	--	Yes	--	--	--	--	Yes	Yes	Yes
G4ST01153548870	chr1:153548871-153548902	+	GGGGAGGGATGGGCCTGAAACTTATTTCTGGG<br>...((((....)))).	FDPS	NM_002004	chr1:153545163-153557081	non-template	32	Intron_4	--	Yes	--	Yes	--	--	--	--	Yes	Yes	Yes
G4ST01153559326	chr1:153559327-153559360	-	GGGGGGTCTCGAGGCGGGACTGGCGGGGCTGGGG<br>..(((....)))	C1orf104	NM_001039517	chr1:153556875-153560562	non-template	34	Intron_1.u	--	Yes	--	Yes	--	--	--	Yes	--	--	--
G4ST01153559326	chr1:153559327-153559360	-	GGGGGGTCTCGAGGCGGGACTGGCGGGGCTGGGG<br>..(((....)))	RUSC1	NM_001105203	chr1:153557264-153567533	template	34	Exon_2	--	Yes	Yes	--	Yes	--	--	Yes	--	--	--
G4ST01153559326	chr1:153559327-153559360	-	GGGGGGTCTCGAGGCGGGACTGGCGGGGCTGGGG<br>..(((....)))	RUSC1	NM_001105204	chr1:153557264-153567533	template	34	Exon_2	--	Yes	Yes	--	Yes	--	--	Yes	--	--	--
G4ST01153559326	chr1:153559327-153559360	-	GGGGGGTCTCGAGGCGGGACTGGCGGGGCTGGGG<br>..(((....)))	RUSC1	NM_001105205	chr1:153560352-153567533	template	-991	upstream	Yes	--	--	--	--	--	--	Yes	--	--	--
G4ST01153559326	chr1:153559327-153559360	-	GGGGGGTCTCGAGGCGGGACTGGCGGGGCTGGGG<br>..(((....)))	RUSC1	NM_014328	chr1:153560842-153567533	template	-1481	upstream	Yes	--	--	--	--	--	--	Yes	--	--	--
G4ST01153669655	chr1:153669656-153669683	+	GGGCCAGGGGGGCCGGAGCCGGGCTGGG<br>.(((....)))	POU5F1P4	NR_034180	chr1:153669595-153670677	non-template	28	Exon_1.u	--	Yes	Yes	--	--	--	--	--	--	--	--
G4ST01153669655	chr1:153669656-153669683	+	GGGCCAGGGGGGCCGGAGCCGGGCTGGG<br>.(((....)))	ASH1L	NM_018489	chr1:153571676-153798948	template	28	Intron_5	--	Yes	--	Yes	--	--	--	--	--	--	--
G4ST01153937528	chr1:153937529-153937568	-	GGGCAGAGGGGCTACTCACTTCCCAGTAGGGGCGGCCGGG<br>.(((((........)))))	DAP3	NM_001199851	chr1:153925506-153975424	template	40	Intron_1.f	--	Yes	--	Yes	--	--	--	--	--	--	--
G4ST01153937528	chr1:153937529-153937568	-	GGGCAGAGGGGCTACTCACTTCCCAGTAGGGGCGGCCGGG<br>.(((((........)))))	DAP3	NM_004632	chr1:153925509-153975424	template	40	Intron_1.f	--	Yes	--	Yes	--	--	--	--	--	--	--
G4ST01153937528	chr1:153937529-153937568	-	GGGCAGAGGGGCTACTCACTTCCCAGTAGGGGCGGCCGGG<br>.(((((........)))))	DAP3	NM_001199850	chr1:153925506-153975424	template	40	Intron_1.f	--	Yes	--	Yes	--	--	--	--	--	--	--
G4ST01153937528	chr1:153937529-153937568	-	GGGCAGAGGGGCTACTCACTTCCCAGTAGGGGCGGCCGGG<br>.(((((........)))))	DAP3	NM_001199849	chr1:153925506-153975424	template	40	Intron_1.f	--	Yes	--	Yes	--	--	--	--	--	--	--
G4ST01153937528	chr1:153937529-153937568	-	GGGCAGAGGGGCTACTCACTTCCCAGTAGGGGCGGCCGGG<br>.(((((........)))))	DAP3	NM_033657	chr1:153925509-153975424	template	40	Intron_1.f	--	Yes	--	Yes	--	--	--	--	--	--	--
G4ST01154077725	chr1:154077726-154077760	+	GGGCATGAGAGATTATAGGGGCTGGGTTTTGAGGG<br>.((((....))))..	GON4L	NM_032292	chr1:154001200-154093596	template	35	Intron_2	--	Yes	--	Yes	--	--	--	--	--	--	--
G4ST01154077725	chr1:154077726-154077760	+	GGGCATGAGAGATTATAGGGGCTGGGTTTTGAGGG<br>.((((....))))..	GON4L	NM_001037533	chr1:153986134-154093596	template	35	Intron_2	--	Yes	--	Yes	--	--	--	--	--	--	--
G4ST01154096696	chr1:154096697-154096734	+	GGGGAGGCGGCGGCCTCTGGGGAAAGAGGGGTGCGGGG<br>(((((....)))))..	SYT11	NM_152280	chr1:154095884-154121614	non-template	38	Intron_1.f	--	Yes	--	Yes	--	--	--	Yes	Yes	Yes	Yes
G4ST01154108159	chr1:154108160-154108201	-	GGGTTTAGGAAGCCTGGGAGGCTGGAAGGCTCTAGGGTGGGG<br>...(((...))) ((.((...)).))...	SYT11	NM_152280	chr1:154095884-154121614	template	42	Intron_2	--	Yes	--	Yes	--	--	--	--	Yes	--	--
G4ST01154108164	chr1:154108165-154108209	-	GGGAAAATGGGTTTAGGAAGCCTGGGAGGCTGGAAGGCTCTAGGG<br>...(((...))) ((.((...)).))...	SYT11	NM_152280	chr1:154095884-154121614	template	45	Intron_2	--	Yes	--	Yes	--	--	--	--	Yes	--	--
G4ST01154203081	chr1:154203082-154203112	+	GGGGCCCGAGGCCTGGGGTTGAGGGTCTGGG<br>(((...)))...	ARHGEF2	NM_004723	chr1:154183254-154214590	template	31	Intron_3	--	Yes	--	Yes	--	--	--	--	--	--	--
G4ST01154203081	chr1:154203082-154203112	+	GGGGCCCGAGGCCTGGGGTTGAGGGTCTGGG<br>(((...)))...	ARHGEF2	NM_001162384	chr1:154183254-154214960	template	31	Intron_3	--	Yes	--	Yes	--	--	--	--	--	--	--
G4ST01154203081	chr1:154203082-154203112	+	GGGGCCCGAGGCCTGGGGTTGAGGGTCTGGG<br>(((...)))...	ARHGEF2	NM_001162383	chr1:154183254-154214960	template	31	Intron_3	--	Yes	--	Yes	--	--	--	--	--	--	--
G4ST01154249399	chr1:154249400-154249435	+	GGGAGTTTATAATTAAATTGGGAGGTGGGGGTGGGG<br>.(((((....))))).	SSR2	NM_003145	chr1:154245463-154257382	template	36	Intron_4	--	Yes	--	Yes	--	--	--	--	--	--	Yes

G4ST01154278263	chr1:154278264-154278300	-	GGGCTGGTACCCAGGTGAGGGTAGGGGGGCAGGAGGG<br>((((...))))....	UBQLN4	NM_020131	chr1:154271716-154290140	non-template	37	Exon_9-Intron_9	--	Yes	Yes	Yes	Yes	--	--	--	--	--	--
G4ST01154291219	chr1:154291220-154291254	+	GGGACTACGGGAAGCAGCGGGCAGCGGCCCGCGGG<br>..(((...)))	LAMTOR2	NM_014017	chr1:154291141-154294925	non-template	35	Exon_1.f	--	Yes	Yes	--	--	Yes	--	Yes	Yes	Yes	--
G4ST01154291219	chr1:154291220-154291254	+	GGGACTACGGGAAGCAGCGGGCAGCGGCCCGCGGG<br>..(((...)))	LAMTOR2	NM_001145264	chr1:154291141-154294925	non-template	35	Exon_1.f	--	Yes	Yes	--	--	Yes	--	Yes	Yes	Yes	--
G4ST01154291219	chr1:154291220-154291254	+	GGGACTACGGGAAGCAGCGGGCAGCGGCCCGCGGG<br>..(((...)))	UBQLN4	NM_020131	chr1:154271716-154290140	template	-1079	upstream	Yes	--	--	--	--	--	--	Yes	Yes	Yes	--
G4ST01154294311	chr1:154294312-154294350	+	GGGGCCAGAGAAGCCTTTGGGGAAGGAGGGTGCTAAGGG<br>..(((((....)))))	LAMTOR2	NM_014017	chr1:154291141-154294925	non-template	39	Intron_2	--	Yes	--	Yes	--	--	--	--	--	--	--
G4ST01154294311	chr1:154294312-154294350	+	GGGGCCAGAGAAGCCTTTGGGGAAGGAGGGTGCTAAGGG<br>..(((((....)))))	LAMTOR2	NM_001145264	chr1:154291141-154294925	non-template	39	Intron_2.l	--	Yes	--	Yes	--	--	--	--	--	--	--
G4ST01154297631	chr1:154297632-154297670	-	GGGATTGGGGGCCAACTCAAGGTTAGGCAGGGCCTAGGG<br>.((((((.....))).))).	RAB25	NM_020387	chr1:154297590-154306919	template	39	Exon_1.f	--	Yes	Yes	--	--	Yes	--	--	Yes	Yes	--
G4ST01154297999	chr1:154298000-154298028	+	GGGAGTGGGGGAGGGTCTGGTTGCAGGGG<br>.(((....)))	RAB25	NM_020387	chr1:154297590-154306919	non-template	29	Intron_1.f	--	Yes	--	Yes	--	--	--	Yes	--	--	--
G4ST01154306741	chr1:154306742-154306781	-	GGGCAAGGAAAGGTCCTGGGGCTGAAGTGGGGACAAGGGG<br>..((((....)))).	RAB25	NM_020387	chr1:154297590-154306919	template	40	Exon_5.l	--	Yes	Yes	--	--	--	Yes	Yes	--	--	--
G4ST01154370945	chr1:154370946-154370983	+	GGGGACTGTGCTTTGCAAGCCAGAGGGCTGGGGCTGGG<br>..(((.(((.....)))))).	LMNA	NM_170708	chr1:154351085-154376502	non-template	38	Intron_3	--	Yes	--	Yes	--	--	--	--	--	Yes	--
G4ST01154370945	chr1:154370946-154370983	+	GGGGACTGTGCTTTGCAAGCCAGAGGGCTGGGGCTGGG<br>..(((.(((.....)))))).	LMNA	NM_170707	chr1:154351085-154376502	non-template	38	Intron_3	--	Yes	--	Yes	--	--	--	--	--	Yes	--
G4ST01154370945	chr1:154370946-154370983	+	GGGGACTGTGCTTTGCAAGCCAGAGGGCTGGGGCTGGG<br>..(((.(((.....)))))).	LMNA	NM_005572	chr1:154351085-154374281	non-template	38	Intron_3	--	Yes	--	Yes	--	--	--	--	--	Yes	--
G4ST01154372277	chr1:154372278-154372310	-	GGGGGGTGGGAGGGTTTGGTGAGCTCCCAAGGG<br>.((((.......))))	LMNA	NM_170708	chr1:154351085-154376502	template	33	Intron_5	--	Yes	--	Yes	--	--	--	--	Yes	Yes	--
G4ST01154372277	chr1:154372278-154372310	-	GGGGGGTGGGAGGGTTTGGTGAGCTCCCAAGGG<br>.((((.......))))	LMNA	NM_170707	chr1:154351085-154376502	template	33	Intron_5	--	Yes	--	Yes	--	--	--	--	Yes	Yes	--
G4ST01154372277	chr1:154372278-154372310	-	GGGGGGTGGGAGGGTTTGGTGAGCTCCCAAGGG<br>.((((.......))))	LMNA	NM_005572	chr1:154351085-154374281	template	33	Intron_5	--	Yes	--	Yes	--	--	--	--	Yes	Yes	--
G4ST01154372616	chr1:154372617-154372649	-	GGGCTGGGGGACAGGCGTAGCCTGGGGAGAGGG<br>...(((((...)))))	LMNA	NM_170708	chr1:154351085-154376502	template	33	Intron_6-Exon_7	--	Yes	Yes	Yes	Yes	--	--	--	--	--	--
G4ST01154372616	chr1:154372617-154372649	-	GGGCTGGGGGACAGGCGTAGCCTGGGGAGAGGG<br>...(((((...)))))	LMNA	NM_170707	chr1:154351085-154376502	template	33	Intron_6-Exon_7	--	Yes	Yes	Yes	Yes	--	--	--	--	--	--
G4ST01154372616	chr1:154372617-154372649	-	GGGCTGGGGGACAGGCGTAGCCTGGGGAGAGGG<br>...(((((...)))))	LMNA	NM_005572	chr1:154351085-154374281	template	33	Intron_6-Exon_7	--	Yes	Yes	Yes	Yes	--	--	--	--	--	--
G4ST01154374026	chr1:154374027-154374063	-	GGGGACATGGAACCAGGTCCAAGGGTCAGGGCCAGGG<br>(((.((....)).)))...	LMNA	NM_170708	chr1:154351085-154376502	template	37	Intron_9	--	Yes	--	Yes	--	--	--	--	Yes	--	Yes
G4ST01154374026	chr1:154374027-154374063	-	GGGGACATGGAACCAGGTCCAAGGGTCAGGGCCAGGG<br>(((.((....)).)))...	LMNA	NM_170707	chr1:154351085-154376502	template	37	Intron_9	--	Yes	--	Yes	--	--	--	--	Yes	--	Yes
G4ST01154374026	chr1:154374027-154374063	-	GGGGACATGGAACCAGGTCCAAGGGTCAGGGCCAGGG<br>(((.((....)).)))...	LMNA	NM_005572	chr1:154351085-154374281	template	37	Intron_9.l	--	Yes	--	Yes	--	--	--	--	Yes	--	Yes
G4ST01154375883	chr1:154375884-154375913	-	GGGTCCCCGGGGTGGGGGCAGCAGCCAGGG<br>(((....))).	LMNA	NM_170708	chr1:154351085-154376502	template	30	Exon_11.l	--	Yes	Yes	--	--	--	Yes	--	--	Yes	--
G4ST01154375883	chr1:154375884-154375913	-	GGGTCCCCGGGGTGGGGGCAGCAGCCAGGG<br>(((....))).	LMNA	NM_170707	chr1:154351085-154376502	template	30	Exon_12.l	--	Yes	Yes	--	--	--	Yes	--	--	Yes	--
G4ST01154386676	chr1:154386677-154386709	+	GGGTAAGGGGTTAGGAGGGACTGAGCTGGTGGG<br>(((.....)))	SEMA4A	NM_001193301	chr1:154386359-154414166	non-template	33	Intron_1.f	--	Yes	--	Yes	--	--	--	--	--	--	Yes
G4ST01154480526	chr1:154480527-154480563	+	GGGTCAGGGATGGGGCCTCACTGTTGTTTGGCCTGGG<br>(((..((....))..)))..	PAQR6	NM_024897	chr1:154479831-154484467	template	37	Exon_7.l	--	Yes	Yes	--	Yes	--	--	--	--	--	--
G4ST01154480526	chr1:154480527-154480563	+	GGGTCAGGGATGGGGCCTCACTGTTGTTTGGCCTGGG<br>(((..((....))..)))..	PAQR6	NM_198406	chr1:154479834-154484416	template	37	Exon_8.l	--	Yes	Yes	--	Yes	--	Yes	--	--	--	--
G4ST01154481468	chr1:154481469-154481497	+	GGGACTGAGGGGGCAGGGCTGGAAGTGGG<br>((...)).	PAQR6	NM_024897	chr1:154479831-154484467	template	29	Intron_5	--	Yes	--	Yes	--	--	--	--	--	--	--
G4ST01154481468	chr1:154481469-154481497	+	GGGACTGAGGGGGCAGGGCTGGAAGTGGG<br>((...)).	PAQR6	NM_198406	chr1:154479834-154484416	template	29	Intron_6	--	Yes	--	Yes	--	--	--	--	--	--	--
G4ST01154481476	chr1:154481477-154481508	+	GGGGGCAGGGCTGGAAGTGGGCACCTTGAGGG<br>((...)). ((...)).	PAQR6	NM_024897	chr1:154479831-154484467	template	32	Intron_5	--	Yes	--	Yes	--	--	--	--	--	--	--
G4ST01154481476	chr1:154481477-154481508	+	GGGGGCAGGGCTGGAAGTGGGCACCTTGAGGG<br>((...)). ((...)).	PAQR6	NM_198406	chr1:154479834-154484416	template	32	Intron_6	--	Yes	--	Yes	--	--	--	--	--	--	--
G4ST01154482862	chr1:154482863-154482896	+	GGGGTGGGATGGGGGTGGAGCGCAGCACACAGGG<br>.(((..((...)).))).	PAQR6	NM_024897	chr1:154479831-154484467	template	34	Intron_2	--	Yes	--	Yes	--	--	--	Yes	Yes	--	--
G4ST01154482862	chr1:154482863-154482896	+	GGGGTGGGATGGGGGTGGAGCGCAGCACACAGGG<br>.(((..((...)).))).	PAQR6	NM_198406	chr1:154479834-154484416	template	34	Intron_2	--	Yes	--	Yes	--	--	--	Yes	Yes	--	--
G4ST01154482867	chr1:154482868-154482906	+	GGGATGGGGGTGGAGCGCAGCACACAGGGCGCTCTGGGG<br>.(((..((...)).))).	PAQR6	NM_024897	chr1:154479831-154484467	template	39	Intron_2	--	Yes	--	Yes	--	--	--	Yes	Yes	--	--
G4ST01154482867	chr1:154482868-154482906	+	GGGATGGGGGTGGAGCGCAGCACACAGGGCGCTCTGGGG<br>.(((..((...)).))).	PAQR6	NM_198406	chr1:154479834-154484416	template	39	Intron_2	--	Yes	--	Yes	--	--	--	Yes	Yes	--	--
G4ST01154482872	chr1:154482873-154482912	+	GGGGGTGGAGCGCAGCACACAGGGCGCTCTGGGGAAGGGG<br>.(((..((...)).))).	PAQR6	NM_024897	chr1:154479831-154484467	template	40	Intron_2	--	Yes	--	Yes	--	--	--	Yes	Yes	--	--
G4ST01154482872	chr1:154482873-154482912	+	GGGGGTGGAGCGCAGCACACAGGGCGCTCTGGGGAAGGGG<br>.(((..((...)).))).	PAQR6	NM_198406	chr1:154479834-154484416	template	40	Intron_2	--	Yes	--	Yes	--	--	--	Yes	Yes	--	--
G4ST01154489405	chr1:154489406-154489433	+	GGGCCTGGGTGTCAGGGACGAGGTAGGG<br>((...)).	SMG5	NM_015327	chr1:154485639-154519244	template	28	Exon_18	--	Yes	Yes	--	Yes	--	--	Yes	--	--	--
G4ST01154518736	chr1:154518737-154518774	-	GGGCACTAAGCTTGGTGGAGTTGGGGTAATAGGGTGGG<br>(((((....))))))......	TMEM79	NR_026678	chr1:154519328-154528858	template	-553	upstream	Yes	--	--	--	--	--	--	Yes	Yes	Yes	--
G4ST01154518736	chr1:154518737-154518774	-	GGGCACTAAGCTTGGTGGAGTTGGGGTAATAGGGTGGG<br>(((((....))))))......	TMEM79	NM_032323	chr1:154520694-154528858	template	-1919	upstream	Yes	--	--	--	--	--	--	Yes	Yes	Yes	--
G4ST01154518736	chr1:154518737-154518774	-	GGGCACTAAGCTTGGTGGAGTTGGGGTAATAGGGTGGG<br>(((((....))))))......	SMG5	NM_015327	chr1:154485639-154519244	non-template	38	Intron_1.f	--	Yes	--	Yes	--	--	--	Yes	Yes	Yes	--
G4ST01154518740	chr1:154518741-154518779	-	GGGGCGGGCACTAAGCTTGGTGGAGTTGGGGTAATAGGG<br>(((((....))))))......	TMEM79	NR_026678	chr1:154519328-154528858	template	-548	upstream	Yes	--	--	--	--	--	--	Yes	Yes	Yes	--
G4ST01154518740	chr1:154518741-154518779	-	GGGGCGGGCACTAAGCTTGGTGGAGTTGGGGTAATAGGG<br>(((((....))))))......	TMEM79	NM_032323	chr1:154520694-154528858	template	-1914	upstream	Yes	--	--	--	--	--	--	Yes	Yes	Yes	--
G4ST01154518740	chr1:154518741-154518779	-	GGGGCGGGCACTAAGCTTGGTGGAGTTGGGGTAATAGGG<br>(((((....))))))......	SMG5	NM_015327	chr1:154485639-154519244	non-template	39	Intron_1.f	--	Yes	--	Yes	--	--	--	Yes	Yes	Yes	--
G4ST01154605525	chr1:154605526-154605558	+	GGGGCCCTGGGCCTGCGGCAGGTGAGGGCGGGG<br>((((...))))...	RHBG	NM_020407	chr1:154605627-154621635	non-template	-68	upstream	Yes	--	--	--	--	--	--	Yes	Yes	Yes	--
G4ST01154605525	chr1:154605526-154605558	+	GGGGCCCTGGGCCTGCGGCAGGTGAGGGCGGGG<br>((((...))))...	RHBG	NR_026549	chr1:154605627-154621635	non-template	-68	upstream	Yes	--	--	--	--	--	--	Yes	Yes	Yes	--
G4ST01154619592	chr1:154619593-154619631	+	GGGGATGGCTGCGCCATGTGGGGCAGGGACAAGGAGGGG<br>.(((((...)))))...	RHBG	NM_020407	chr1:154605627-154621635	non-template	39	Intron_8	--	Yes	--	Yes	--	--	--	Yes	Yes	Yes	--
G4ST01154619592	chr1:154619593-154619631	+	GGGGATGGCTGCGCCATGTGGGGCAGGGACAAGGAGGGG<br>.(((((...)))))...	RHBG	NR_026549	chr1:154605627-154621635	non-template	39	Intron_9	--	Yes	--	Yes	--	--	--	Yes	Yes	Yes	--
G4ST01154713490	chr1:154713491-154713526	+	GGGGGCTCCAAGCTGGGTGCTGTGGGTAGGTGGGGG<br>.(((...))).	MEF2D	NM_005920	chr1:154700143-154737153	template	36	Exon_7	--	Yes	Yes	--	Yes	--	--	Yes	Yes	--	--
G4ST01154719715	chr1:154719716-154719754	+	GGGGGTCCTCAGTGCTACGGAGGGGAGGGGCTCGCTGGG<br>....(((.((...)).)))	MEF2D	NM_005920	chr1:154700143-154737153	template	39	Exon_2	--	Yes	Yes	--	--	Yes	--	--	--	--	Yes
G4ST01154726875	chr1:154726876-154726905	+	GGGCTGGGCAGGGGCTATTTTTAGCCTGGG<br>((((....))))..	MEF2D	NM_005920	chr1:154700143-154737153	template	30	Intron_1.f	--	Yes	--	Yes	--	--	--	Yes	Yes	Yes	--

G4ST01154736054	chr1:154736055-154736085	-	GGGCTGCGAGCGGGGGTTTAGGGAGTTTGGG<br>(((....)))	MEF2D	NM_005920	chr1:154700143-154737153	non-template	31	Intron_1.f	--	Yes	--	Yes	--	--	--	Yes	Yes	--	Yes
G4ST01154777316	chr1:154777317-154777345	+	GGGTGGGGTGCATGCACTGGGAGGAAGGG<br>(((....))).	IQGAP3	NM_178229	chr1:154761821-154809020	template	29	Intron_22-Exon_23	--	Yes	Yes	Yes	Yes	--	--	--	--	--	--
G4ST01154794611	chr1:154794612-154794652	+	GGGAGGCGCGGGGGGGGGGCCGGCCAGCCGCCCTGTCCGGG<br>((((.((....))))))....	IQGAP3	NM_178229	chr1:154761821-154809020	template	41	Intron_11	--	Yes	--	Yes	--	--	--	--	--	--	Yes
G4ST01154794620	chr1:154794621-154794656	+	GGGGGGGGGGCCGGCCAGCCGCCCTGTCCGGGAGGG<br>((((.((....))))))....	IQGAP3	NM_178229	chr1:154761821-154809020	template	36	Intron_11	--	Yes	--	Yes	--	--	--	--	--	--	Yes
G4ST01154794624	chr1:154794625-154794663	+	GGGGGGCCGGCCAGCCGCCCTGTCCGGGAGGGAGGTGGG<br>((((.((....))))))....	IQGAP3	NM_178229	chr1:154761821-154809020	template	39	Intron_11	--	Yes	--	Yes	--	--	--	--	--	--	Yes
G4ST01154808770	chr1:154808771-154808798	-	GGGCGGTGGGACTGCTCAGTGGGACGGG<br>(((....)))	IQGAP3	NM_178229	chr1:154761821-154809020	non-template	28	Intron_1.f	--	Yes	--	Yes	--	--	--	--	Yes	Yes	--
G4ST01154808775	chr1:154808776-154808805	-	GGGGCGAGGGCGGTGGGACTGCTCAGTGGG<br>(((....)))	IQGAP3	NM_178229	chr1:154761821-154809020	non-template	30	Intron_1.f	--	Yes	--	Yes	--	--	--	--	Yes	Yes	--
G4ST01154818229	chr1:154818230-154818262	+	GGGTGACCAGGGAGAAGCCTGGGCAAAAATGGG<br>((....))	TTC24	NM_001105669	chr1:154816143-154823186	non-template	33	Exon_2	--	Yes	Yes	--	Yes	--	--	Yes	Yes	--	--
G4ST01154818238	chr1:154818239-154818283	+	GGGAGAAGCCTGGGCAAAAATGGGAGCCTGCTACCAGGCTCTGGG<br>((....)) ((((((....))))))..	TTC24	NM_001105669	chr1:154816143-154823186	non-template	45	Exon_2	--	Yes	Yes	--	Yes	--	--	Yes	Yes	--	--
G4ST01154819025	chr1:154819026-154819062	+	GGGTTGTGGGCCCAGAGGCCGAGGCCTCAGGGTTGGG<br>....(((((....))))).	TTC24	NM_001105669	chr1:154816143-154823186	non-template	37	Intron_3	--	Yes	--	Yes	--	--	--	Yes	--	--	--
G4ST01154859830	chr1:154859831-154859864	-	GGGCAGTTCTTAGGACTGGGGCAAAAGGGTGGGG<br>(((((.....)))))	HAPLN2	NM_021817	chr1:154855710-154862141	template	34	Intron_2	--	Yes	--	Yes	--	--	--	--	--	--	Yes
G4ST01154860678	chr1:154860679-154860709	-	GGGCGGCGCGGCCTGGGAGAGGGAGCTGGGG<br>.(((...))).	HAPLN2	NM_021817	chr1:154855710-154862141	template	31	Intron_4	--	Yes	--	Yes	--	--	--	--	Yes	--	Yes
G4ST01154860686	chr1:154860687-154860719	-	GGGGGAGGAAGGGCGGCGCGGCCTGGGAGAGGG<br>.(((...))).	HAPLN2	NM_021817	chr1:154855710-154862141	template	33	Intron_4	--	Yes	--	Yes	--	--	--	--	Yes	Yes	Yes
G4ST01154860692	chr1:154860693-154860728	-	GGGAGCGGAGGGGGAGGAAGGGCGGCGCGGCCTGGG<br>.(((...))).	HAPLN2	NM_021817	chr1:154855710-154862141	template	36	Intron_4	--	Yes	--	Yes	--	--	--	--	Yes	Yes	Yes
G4ST01154889513	chr1:154889514-154889552	-	GGGTCACAGATGGTCAGTGGCGGGGCTGGGATCACAGGG<br>.(((.((...)).)))...	BCAN	NM_198427	chr1:154878364-154889888	template	39	Exon_8.l	--	Yes	Yes	--	--	--	Yes	--	--	Yes	--
G4ST01154889513	chr1:154889514-154889552	-	GGGTCACAGATGGTCAGTGGCGGGGCTGGGATCACAGGG<br>.(((.((...)).)))...	BCAN	NM_021948	chr1:154878364-154895944	template	39	Intron_8	--	Yes	--	Yes	--	--	--	--	--	Yes	--
G4ST01154889522	chr1:154889523-154889557	-	GGGAAGGGTCACAGATGGTCAGTGGCGGGGCTGGG<br>.(((.((...)).)))...	BCAN	NM_198427	chr1:154878364-154889888	template	35	Exon_8.l	--	Yes	Yes	--	--	--	Yes	--	--	Yes	--
G4ST01154889522	chr1:154889523-154889557	-	GGGAAGGGTCACAGATGGTCAGTGGCGGGGCTGGG<br>.(((.((...)).)))...	BCAN	NM_021948	chr1:154878364-154895944	template	35	Intron_8	--	Yes	--	Yes	--	--	--	--	--	Yes	--
G4ST01154891031	chr1:154891032-154891060	+	GGGGGAGGGGCCTATGGCTTGGGTAGGGG<br>(((...)))..	BCAN	NM_021948	chr1:154878364-154895944	non-template	29	Intron_8	--	Yes	--	Yes	--	--	--	--	--	--	--
G4ST01154915279	chr1:154915280-154915316	-	GGGATGGGGTCCTTGAGAGACTCCTCAAGGGGAGGGG<br>...((((((......))))))	NES	NM_006617	chr1:154905180-154913813	non-template	-1466	upstream	Yes	--	--	--	--	--	--	--	--	--	--
G4ST01154915284	chr1:154915285-154915323	-	GGGGACAGGGATGGGGTCCTTGAGAGACTCCTCAAGGGG<br>...((((((......))))))	NES	NM_006617	chr1:154905180-154913813	non-template	-1471	upstream	Yes	--	--	--	--	--	--	--	--	--	--
G4ST01154943786	chr1:154943787-154943826	+	GGGGGTGTTTGGGAGGCCTGGGCTTCTGCAAGGAAGAGGG<br>(((((....))))).	CRABP2	NM_001878	chr1:154936024-154942083	template	-1703	upstream	Yes	--	--	--	--	--	--	--	--	--	--
G4ST01154943786	chr1:154943787-154943826	+	GGGGGTGTTTGGGAGGCCTGGGCTTCTGCAAGGAAGAGGG<br>(((((....))))).	CRABP2	NM_001199723	chr1:154936024-154942232	template	-1554	upstream	Yes	--	--	--	--	--	--	--	--	--	--
G4ST01154960624	chr1:154960625-154960661	+	GGGCAGTCAGCCTTCCGGTTGAGGGGGGGGATATGGG<br>....(((((....))))).	ISG20L2	NM_030980	chr1:154959037-154964329	template	37	Exon_2	--	Yes	Yes	--	Yes	--	--	--	--	--	--
G4ST01154965494	chr1:154965495-154965539	-	GGGCGGAGATGCCCGGCATCCCGGGGTCCAGCAGGGAGCGCGGGG<br>(((.(((((...))))))))	RRNAD1	NM_001142560	chr1:154964887-154973376	template	45	Exon_1.f	--	Yes	Yes	--	Yes	Yes	--	Yes	Yes	Yes	--
G4ST01154965494	chr1:154965495-154965539	-	GGGCGGAGATGCCCGGCATCCCGGGGTCCAGCAGGGAGCGCGGGG<br>(((.(((((...))))))))	RRNAD1	NM_015997	chr1:154964887-154973376	template	45	Exon_1.f	--	Yes	Yes	--	Yes	Yes	--	Yes	Yes	Yes	--
G4ST01154965494	chr1:154965495-154965539	-	GGGCGGAGATGCCCGGCATCCCGGGGTCCAGCAGGGAGCGCGGGG<br>(((.(((((...))))))))	ISG20L2	NM_030980	chr1:154959037-154964329	non-template	-1165	upstream	Yes	--	--	--	--	--	--	Yes	Yes	Yes	--
G4ST01154979295	chr1:154979296-154979325	-	GGGAAAGTGGGACAGCTTGTAGGGGAGGGG<br>(((...)))..	HDGF	NM_001126051	chr1:154978523-154988648	non-template	30	Exon_6.l	--	Yes	Yes	--	--	--	Yes	--	--	--	--
G4ST01154979295	chr1:154979296-154979325	-	GGGAAAGTGGGACAGCTTGTAGGGGAGGGG<br>(((...)))..	HDGF	NM_004494	chr1:154978523-154988167	non-template	30	Exon_6.l	--	Yes	Yes	--	--	--	Yes	--	--	--	--
G4ST01154979295	chr1:154979296-154979325	-	GGGAAAGTGGGACAGCTTGTAGGGGAGGGG<br>(((...)))..	HDGF	NM_001126050	chr1:154978523-154988864	non-template	30	Exon_6.l	--	Yes	Yes	--	--	--	Yes	--	--	--	--
G4ST01154979295	chr1:154979296-154979325	-	GGGAAAGTGGGACAGCTTGTAGGGGAGGGG<br>(((...)))..	MRPL24	NM_024540	chr1:154973718-154977547	non-template	-1748	upstream	Yes	--	--	--	--	--	--	--	--	--	--
G4ST01154979295	chr1:154979296-154979325	-	GGGAAAGTGGGACAGCTTGTAGGGGAGGGG<br>(((...)))..	MRPL24	NM_145729	chr1:154973718-154977547	non-template	-1748	upstream	Yes	--	--	--	--	--	--	--	--	--	--
G4ST01154980952	chr1:154980953-154980986	+	GGGCAGGCAGGGCTCTGTGCAGGAGGGGGGAGGG<br>((((.....))))..	HDGF	NM_001126051	chr1:154978523-154988648	template	34	Intron_3	--	Yes	--	Yes	--	--	--	--	--	--	--
G4ST01154980952	chr1:154980953-154980986	+	GGGCAGGCAGGGCTCTGTGCAGGAGGGGGGAGGG<br>((((.....))))..	HDGF	NM_004494	chr1:154978523-154988167	template	34	Intron_3	--	Yes	--	Yes	--	--	--	--	--	--	--
G4ST01154980952	chr1:154980953-154980986	+	GGGCAGGCAGGGCTCTGTGCAGGAGGGGGGAGGG<br>((((.....))))..	HDGF	NM_001126050	chr1:154978523-154988864	template	34	Intron_3	--	Yes	--	Yes	--	--	--	--	--	--	--
G4ST01155010500	chr1:155010501-155010543	+	GGGGCTGGGGGGAGGTGGGGATAAAGAGTTACTGTTTAATGGG<br>..((((.((...)).))))..	PRCC	NM_005973	chr1:155003898-155037233	non-template	43	Intron_1.f	--	Yes	--	Yes	--	--	--	--	Yes	--	--
G4ST01155078751	chr1:155078752-155078779	-	GGGTTGGGCACAGTGTGGGAGGTGGGGG<br>.((...))	INSRR	NM_014215	chr1:155077289-155095336	non-template	28	Intron_18	--	Yes	--	Yes	--	--	--	--	--	--	--
G4ST01155078751	chr1:155078752-155078779	-	GGGTTGGGCACAGTGTGGGAGGTGGGGG<br>.((...))	NTRK1	NM_001007792	chr1:155052166-155118266	template	28	Intron_2	--	Yes	--	Yes	--	--	--	--	--	--	--
G4ST01155081681	chr1:155081682-155081713	-	GGGACTCAGGGCGGCACCGCCGGGCAGCTGGG<br>.(((...)))	INSRR	NM_014215	chr1:155077289-155095336	non-template	32	Intron_11-Exon_12	--	Yes	Yes	Yes	Yes	--	--	--	Yes	--	--
G4ST01155081681	chr1:155081682-155081713	-	GGGACTCAGGGCGGCACCGCCGGGCAGCTGGG<br>.(((...)))	NTRK1	NM_001007792	chr1:155052166-155118266	template	32	Intron_2	--	Yes	--	Yes	--	--	--	--	Yes	--	--
G4ST01155085945	chr1:155085946-155085980	+	GGGTTCCTCCTGGAAGGGTTTTGCTGGGCCCGGGG<br>((((....))))	INSRR	NM_014215	chr1:155077289-155095336	template	35	Intron_5	--	Yes	--	Yes	--	--	--	--	--	--	Yes
G4ST01155085945	chr1:155085946-155085980	+	GGGTTCCTCCTGGAAGGGTTTTGCTGGGCCCGGGG<br>((((....))))	NTRK1	NM_001007792	chr1:155052166-155118266	non-template	35	Intron_2	--	Yes	--	Yes	--	--	--	--	--	--	Yes
G4ST01155095739	chr1:155095740-155095780	-	GGGGACAAAGGGTTGGCGGGAGCCGCTGCAGCCGCAGCGGG<br>....(((((....)))))	INSRR	NM_014215	chr1:155077289-155095336	non-template	-403	upstream	Yes	--	--	--	--	--	--	Yes	Yes	--	--
G4ST01155095739	chr1:155095740-155095780	-	GGGGACAAAGGGTTGGCGGGAGCCGCTGCAGCCGCAGCGGG<br>....(((((....)))))	NTRK1	NM_002529	chr1:155097295-155118266	template	-1514	upstream	Yes	--	--	--	--	--	--	Yes	Yes	--	--
G4ST01155095739	chr1:155095740-155095780	-	GGGGACAAAGGGTTGGCGGGAGCCGCTGCAGCCGCAGCGGG<br>....(((((....)))))	NTRK1	NM_001012331	chr1:155097295-155118266	template	-1514	upstream	Yes	--	--	--	--	--	--	Yes	Yes	--	--
G4ST01155095739	chr1:155095740-155095780	-	GGGGACAAAGGGTTGGCGGGAGCCGCTGCAGCCGCAGCGGG<br>....(((((....)))))	NTRK1	NM_001007792	chr1:155052166-155118266	template	41	Intron_2	--	Yes	--	Yes	--	--	--	Yes	Yes	--	--
G4ST01155099004	chr1:155099005-155099036	-	GGGAGGGGGCTGTGCAGGCAGTGGGGAGGGGG<br>..((((....))))..	NTRK1	NM_002529	chr1:155097295-155118266	template	32	Intron_1.f	--	Yes	--	Yes	--	--	--	--	Yes	--	--
G4ST01155099004	chr1:155099005-155099036	-	GGGAGGGGGCTGTGCAGGCAGTGGGGAGGGGG<br>..((((....))))..	NTRK1	NM_001012331	chr1:155097295-155118266	template	32	Intron_1.f	--	Yes	--	Yes	--	--	--	--	Yes	--	--
G4ST01155099004	chr1:155099005-155099036	-	GGGAGGGGGCTGTGCAGGCAGTGGGGAGGGGG<br>..((((....))))..	NTRK1	NM_001007792	chr1:155052166-155118266	template	32	Intron_2	--	Yes	--	Yes	--	--	--	--	Yes	--	--
G4ST01155100882	chr1:155100883-155100914	+	GGGCCTGGGGGAGACCAGAAGGTCAGGGAGGG<br>...((((....)))).	NTRK1	NM_002529	chr1:155097295-155118266	non-template	32	Intron_2	--	Yes	--	Yes	--	--	--	--	--	--	--

G4ST01155100882	chr1:155100883-155100914	+	GGGCCTGGGGGAGACCAGAAGGTCAGGGAGGG<br>...((((....)))).	NTRK1	NM_001012331	chr1:155097295-155118266	non-template	32	Intron_2	--	Yes	--	Yes	--	--	--	--	--	--	--
G4ST01155100882	chr1:155100883-155100914	+	GGGCCTGGGGGAGACCAGAAGGTCAGGGAGGG<br>...((((....)))).	NTRK1	NM_001007792	chr1:155052166-155118266	non-template	32	Intron_3	--	Yes	--	Yes	--	--	--	--	--	--	--
G4ST01155130337	chr1:155130338-155130381	-	GGGTCTGGGGCCTCAGACTCCAGTCGAGGAGGGGGACCTCAGGG<br>.((((.(((....)))))))...	PEAR1	NM_001080471	chr1:155130147-155152850	template	44	Intron_1.f	--	Yes	--	Yes	--	--	--	Yes	--	Yes	--
G4ST01155136549	chr1:155136550-155136582	+	GGGCTCGGAGGCTGAGAGGGCAGGGATGCTGGG<br>((((.....)))).	PEAR1	NM_001080471	chr1:155130147-155152850	non-template	33	Intron_1.f	--	Yes	--	Yes	--	--	--	Yes	--	--	--
G4ST01155137549	chr1:155137550-155137578	-	GGGGCTGGAAGCCAGGGTGGGGTCAGGGG<br>(((...)))..	PEAR1	NM_001080471	chr1:155130147-155152850	template	29	Intron_1.f	--	Yes	--	Yes	--	--	--	--	Yes	--	--
G4ST01155160735	chr1:155160736-155160773	-	GGGTGCCCGGGTGGGATTGGAGGACACCGCCAGAGGGG<br>..(((.((...)).)))...	LRRC71	NM_144702	chr1:155157048-155169504	template	38	Intron_2	--	Yes	--	Yes	--	--	--	--	--	--	--
G4ST01155161442	chr1:155161443-155161480	+	GGGAGGCCCCCTGTGCCTGGGAAGCCTGGCTGGGCGGG<br>((((.......)))) .(((...)))	LRRC71	NM_144702	chr1:155157048-155169504	non-template	38	Intron_5	--	Yes	--	Yes	--	--	--	--	Yes	--	--
G4ST01155162050	chr1:155162051-155162090	+	GGGGCAGGGTAACTACAGGTGTGGTTGGGAGTGTAAGGGG<br>.((((((....))))))	LRRC71	NM_144702	chr1:155157048-155169504	non-template	40	Intron_5	--	Yes	--	Yes	--	--	--	--	Yes	--	--
G4ST01155173530	chr1:155173531-155173556	+	GGGCGGGCTGGGGGAAGATCCCTGGG<br>(((...)))..	MIR765	NR_030527	chr1:155172547-155172660	template	-870	upstream	Yes	--	--	--	--	--	--	--	--	--	Yes
G4ST01155173530	chr1:155173531-155173556	+	GGGCGGGCTGGGGGAAGATCCCTGGG<br>(((...)))..	ARHGEF11	NM_014784	chr1:155171256-155281786	template	26	Intron_38	--	Yes	--	Yes	--	--	--	--	--	--	Yes
G4ST01155173530	chr1:155173531-155173556	+	GGGCGGGCTGGGGGAAGATCCCTGGG<br>(((...)))..	ARHGEF11	NM_198236	chr1:155171256-155281786	template	26	Intron_39	--	Yes	--	Yes	--	--	--	--	--	--	Yes
G4ST01155178475	chr1:155178476-155178528	-	GGGCACCTGCCAGGCAGAGGGTTCTTAGGGCCTGGAAGCCCTGTTCCAGTGGG<br>...((((...)))). .((((((......)))))).	ARHGEF11	NM_014784	chr1:155171256-155281786	non-template	53	Intron_32	--	Yes	--	Yes	--	--	--	--	--	--	--
G4ST01155178475	chr1:155178476-155178528	-	GGGCACCTGCCAGGCAGAGGGTTCTTAGGGCCTGGAAGCCCTGTTCCAGTGGG<br>...((((...)))). .((((((......)))))).	ARHGEF11	NM_198236	chr1:155171256-155281786	non-template	53	Intron_33	--	Yes	--	Yes	--	--	--	--	--	--	--
G4ST01155178498	chr1:155178499-155178535	-	GGGAGGTGGGCACCTGCCAGGCAGAGGGTTCTTAGGG<br>...((((...)))).	ARHGEF11	NM_014784	chr1:155171256-155281786	non-template	37	Intron_32	--	Yes	--	Yes	--	--	--	--	--	--	--
G4ST01155178498	chr1:155178499-155178535	-	GGGAGGTGGGCACCTGCCAGGCAGAGGGTTCTTAGGG<br>...((((...)))).	ARHGEF11	NM_198236	chr1:155171256-155281786	non-template	37	Intron_33	--	Yes	--	Yes	--	--	--	--	--	--	--
G4ST01155184916	chr1:155184917-155184949	+	GGGGGGAAGGGACAAAAGACTTTGGGACTTGGG<br>..(((....)))	ARHGEF11	NM_014784	chr1:155171256-155281786	template	33	Intron_21	--	Yes	--	Yes	--	--	--	--	Yes	--	--
G4ST01155184916	chr1:155184917-155184949	+	GGGGGGAAGGGACAAAAGACTTTGGGACTTGGG<br>..(((....)))	ARHGEF11	NM_198236	chr1:155171256-155281786	template	33	Intron_22	--	Yes	--	Yes	--	--	--	--	Yes	--	--
G4ST01155214682	chr1:155214683-155214725	+	GGGGATCACTGACGTGATTCGGGGAGCTCCTGCTGGGGATGGG<br>.(((((....)))))... .(((....))).	ARHGEF11	NM_014784	chr1:155171256-155281786	template	43	Exon_6	--	Yes	Yes	--	Yes	--	--	--	--	--	--
G4ST01155214682	chr1:155214683-155214725	+	GGGGATCACTGACGTGATTCGGGGAGCTCCTGCTGGGGATGGG<br>.(((((....)))))... .(((....))).	ARHGEF11	NM_198236	chr1:155171256-155281786	template	43	Exon_6	--	Yes	Yes	--	Yes	--	--	--	--	--	--
G4ST01155282230	chr1:155282231-155282257	-	GGGAGGGAAGGGGGGCGGCCGGCCGGG<br>.(((.....)))	ARHGEF11	NM_014784	chr1:155171256-155281786	non-template	-444	upstream	Yes	--	--	--	--	--	--	Yes	Yes	Yes	Yes
G4ST01155282230	chr1:155282231-155282257	-	GGGAGGGAAGGGGGGCGGCCGGCCGGG<br>.(((.....)))	ARHGEF11	NM_198236	chr1:155171256-155281786	non-template	-444	upstream	Yes	--	--	--	--	--	--	Yes	Yes	Yes	Yes
G4ST01155333389	chr1:155333390-155333427	-	GGGAGCAGGACTTTCTCCTGTGGGCAGGAGGGACTGGG<br>..(((((.....))))).	ETV3L	NM_001004341	chr1:155328459-155336224	non-template	38	Intron_4.l	--	Yes	--	Yes	--	--	--	--	--	--	Yes
G4ST01155335781	chr1:155335782-155335819	+	GGGAAGGCCAACCCTGGGTGGAGAGGGGAGAGTGAGGG<br>.(((.....)))	ETV3L	NM_001004341	chr1:155328459-155336224	template	38	Intron_1.f-Exon_2	--	Yes	Yes	Yes	Yes	--	--	--	--	--	--
G4ST01155337672	chr1:155337673-155337699	+	GGGCCAGGGCATTGGGAGGTTCCTGGG<br>((....))	ETV3L	NM_001004341	chr1:155328459-155336224	template	-1448	upstream	Yes	--	--	--	--	--	--	--	--	--	--
G4ST01155374984	chr1:155374985-155375012	-	GGGGCGGATGCGCGGGATGCGGGCCGGG<br>(((....)))	ETV3	NM_005240	chr1:155369600-155374801	non-template	-183	upstream	Yes	--	--	--	--	--	--	Yes	Yes	Yes	--
G4ST01155374984	chr1:155374985-155375012	-	GGGGCGGATGCGCGGGATGCGGGCCGGG<br>(((....)))	ETV3	NM_001145312	chr1:155361083-155375007	non-template	23	upstream-Exon_1.f	Yes	Yes	Yes	--	--	Yes	--	Yes	Yes	Yes	--
G4ST01155374989	chr1:155374990-155375022	-	GGGCCGTGCCGGGGCGGATGCGCGGGATGCGGG<br>(((....)))	ETV3	NM_005240	chr1:155369600-155374801	non-template	-188	upstream	Yes	--	--	--	--	--	--	Yes	Yes	Yes	--
G4ST01155374989	chr1:155374990-155375022	-	GGGCCGTGCCGGGGCGGATGCGCGGGATGCGGG<br>(((....)))	ETV3	NM_001145312	chr1:155361083-155375007	non-template	18	upstream-Exon_1.f	Yes	Yes	Yes	--	--	Yes	--	Yes	Yes	Yes	--
G4ST01155757231	chr1:155757232-155757261	-	GGGGCTGAGAGCGCGGGTTGGCGGGCTGGG<br>(((...)))..	FCRL5	NM_031281	chr1:155749791-155788934	non-template	30	Intron_11	--	Yes	--	Yes	--	--	--	--	--	--	--
G4ST01155757231	chr1:155757232-155757261	-	GGGGCTGAGAGCGCGGGTTGGCGGGCTGGG<br>(((...)))..	FCRL5	NM_001195388	chr1:155749791-155788934	non-template	30	Intron_11	--	Yes	--	Yes	--	--	--	--	--	--	--
G4ST01155826570	chr1:155826571-155826601	-	GGGAAAGGAGTTTTGGGCTTCTGGGTTGGGG<br>(((.....)))	FCRL4	NM_031282	chr1:155810163-155834494	non-template	31	Intron_2	--	Yes	--	Yes	--	--	--	--	--	--	--
G4ST01156071134	chr1:156071135-156071170	+	GGGACTGGGGAGAAAGAGCTCTCTGGGTCTGAGGGG<br>((((......)))).	CD5L	NM_005894	chr1:156067328-156078258	template	36	Intron_3-Exon_4	--	Yes	Yes	Yes	Yes	--	--	--	--	--	--
G4ST01156229630	chr1:156229631-156229670	+	GGGGGGTATGTGTGTGCGCGCGTTGTGGGGGGGCAGGGGG<br>....((((((....))))))...	KIRREL	NM_018240	chr1:156229687-156332468	non-template	-16	upstream	Yes	--	--	--	--	--	--	Yes	Yes	Yes	Yes
G4ST01156230149	chr1:156230150-156230184	-	GGGGGGAAGCCGAGCGTCCGGGGGTGGGCTGGGGG<br>.(((.((...)).)))..	KIRREL	NM_018240	chr1:156229687-156332468	template	35	Intron_1.f	--	Yes	--	Yes	--	--	--	--	Yes	Yes	--
G4ST01156279931	chr1:156279932-156279973	+	GGGAACACTGATAGTTCAGGGGAGTTTGAGGGACAGCTTGGG<br>....((((....))))	KIRREL	NM_018240	chr1:156229687-156332468	non-template	42	Intron_1.f	--	Yes	--	Yes	--	--	--	--	Yes	--	--
G4ST01156301097	chr1:156301098-156301133	+	GGGCTTGTATGGAAGGGGCAGTGATGGGGTCTGGGG<br>(((......)))	KIRREL	NM_018240	chr1:156229687-156332468	non-template	36	Intron_1.f	--	Yes	--	Yes	--	--	--	--	--	--	--
G4ST01156301111	chr1:156301112-156301151	+	GGGGCAGTGATGGGGTCTGGGGACACACCTGTGTTATGGG<br>.((((....))))...	KIRREL	NM_018240	chr1:156229687-156332468	non-template	40	Intron_1.f	--	Yes	--	Yes	--	--	--	--	--	--	--
G4ST01156323828	chr1:156323829-156323860	+	GGGAACCATGGGGGTGTGGGCAGCAACTGGGG<br>(((...)))	KIRREL	NM_018240	chr1:156229687-156332468	non-template	32	Intron_5	--	Yes	--	Yes	--	--	--	Yes	Yes	--	--
G4ST01156331893	chr1:156331894-156331929	+	GGGGGTACCCAGGGATGGGCAGGCTTTGGCCTAGGG<br>.((((....)))).	KIRREL	NM_018240	chr1:156229687-156332468	non-template	36	Exon_15.l	--	Yes	Yes	--	--	--	Yes	--	Yes	--	--
G4ST01156378559	chr1:156378560-156378589	+	GGGCTGCTATGGCAGGGGCGGGGGCAGGGG<br>((((....))))	LOC646268	NR_033946	chr1:156368458-156377054	template	-1505	upstream	Yes	--	--	--	--	--	--	--	--	--	Yes
G4ST01156590495	chr1:156590496-156590539	+	GGGCACCAGAGGGCTGAGAGGAAGCTCTGGGGAGGTCCTGGGGG<br>...((((....)))).	CD1E	NM_001042583	chr1:156590110-156593967	non-template	44	Intron_1.f	--	Yes	--	Yes	--	--	--	--	--	--	--
G4ST01156590495	chr1:156590496-156590539	+	GGGCACCAGAGGGCTGAGAGGAAGCTCTGGGGAGGTCCTGGGGG<br>...((((....)))).	CD1E	NM_001185112	chr1:156590110-156593967	non-template	44	Intron_1.f	--	Yes	--	Yes	--	--	--	--	--	--	--
G4ST01156590495	chr1:156590496-156590539	+	GGGCACCAGAGGGCTGAGAGGAAGCTCTGGGGAGGTCCTGGGGG<br>...((((....)))).	CD1E	NM_001185110	chr1:156590110-156593967	non-template	44	Intron_1.f	--	Yes	--	Yes	--	--	--	--	--	--	--
G4ST01156590495	chr1:156590496-156590539	+	GGGCACCAGAGGGCTGAGAGGAAGCTCTGGGGAGGTCCTGGGGG<br>...((((....)))).	CD1E	NM_001185114	chr1:156590110-156593967	non-template	44	Intron_1.f	--	Yes	--	Yes	--	--	--	--	--	--	--
G4ST01156590495	chr1:156590496-156590539	+	GGGCACCAGAGGGCTGAGAGGAAGCTCTGGGGAGGTCCTGGGGG<br>...((((....)))).	CD1E	NM_001185113	chr1:156590110-156593967	non-template	44	Intron_1.f	--	Yes	--	Yes	--	--	--	--	--	--	--
G4ST01156590495	chr1:156590496-156590539	+	GGGCACCAGAGGGCTGAGAGGAAGCTCTGGGGAGGTCCTGGGGG<br>...((((....)))).	CD1E	NM_001185108	chr1:156590110-156593967	non-template	44	Intron_1.f	--	Yes	--	Yes	--	--	--	--	--	--	--
G4ST01156590495	chr1:156590496-156590539	+	GGGCACCAGAGGGCTGAGAGGAAGCTCTGGGGAGGTCCTGGGGG<br>...((((....)))).	CD1E	NM_001185107	chr1:156590110-156593967	non-template	44	Intron_1.f	--	Yes	--	Yes	--	--	--	--	--	--	--
G4ST01156590495	chr1:156590496-156590539	+	GGGCACCAGAGGGCTGAGAGGAAGCTCTGGGGAGGTCCTGGGGG<br>...((((....)))).	CD1E	NM_001185115	chr1:156590110-156593967	non-template	44	Intron_1.f	--	Yes	--	Yes	--	--	--	--	--	--	--
G4ST01156590495	chr1:156590496-156590539	+	GGGCACCAGAGGGCTGAGAGGAAGCTCTGGGGAGGTCCTGGGGG<br>...((((....)))).	CD1E	NM_001042584	chr1:156590110-156593967	non-template	44	Intron_1.f	--	Yes	--	Yes	--	--	--	--	--	--	--

G4ST01156590495	chr1:156590496-156590539	+	GGGCACCAGAGGGCTGAGAGGAAGCTCTGGGGAGGTCCTGGGGG<br>...((((....)))).	CD1E	NM_001042585	chr1:156590110-156593967	non-template	44	Intron_1.f	--	Yes	--	Yes	--	--	--	--	--	--	--
G4ST01156590495	chr1:156590496-156590539	+	GGGCACCAGAGGGCTGAGAGGAAGCTCTGGGGAGGTCCTGGGGG<br>...((((....)))).	CD1E	NM_001042587	chr1:156590110-156593967	non-template	44	Intron_1.f	--	Yes	--	Yes	--	--	--	--	--	--	--
G4ST01156590495	chr1:156590496-156590539	+	GGGCACCAGAGGGCTGAGAGGAAGCTCTGGGGAGGTCCTGGGGG<br>...((((....)))).	CD1E	NM_001042586	chr1:156590110-156593967	non-template	44	Intron_1.f	--	Yes	--	Yes	--	--	--	--	--	--	--
G4ST01156590495	chr1:156590496-156590539	+	GGGCACCAGAGGGCTGAGAGGAAGCTCTGGGGAGGTCCTGGGGG<br>...((((....)))).	CD1E	NM_030893	chr1:156590110-156593967	non-template	44	Intron_1.f	--	Yes	--	Yes	--	--	--	--	--	--	--
G4ST01156590505	chr1:156590506-156590544	+	GGGCTGAGAGGAAGCTCTGGGGAGGTCCTGGGGGAAGGG<br>...((((....)))).	CD1E	NM_001042583	chr1:156590110-156593967	non-template	39	Intron_1.f	--	Yes	--	Yes	--	--	--	--	--	--	--
G4ST01156590505	chr1:156590506-156590544	+	GGGCTGAGAGGAAGCTCTGGGGAGGTCCTGGGGGAAGGG<br>...((((....)))).	CD1E	NM_001185112	chr1:156590110-156593967	non-template	39	Intron_1.f	--	Yes	--	Yes	--	--	--	--	--	--	--
G4ST01156590505	chr1:156590506-156590544	+	GGGCTGAGAGGAAGCTCTGGGGAGGTCCTGGGGGAAGGG<br>...((((....)))).	CD1E	NM_001185110	chr1:156590110-156593967	non-template	39	Intron_1.f	--	Yes	--	Yes	--	--	--	--	--	--	--
G4ST01156590505	chr1:156590506-156590544	+	GGGCTGAGAGGAAGCTCTGGGGAGGTCCTGGGGGAAGGG<br>...((((....)))).	CD1E	NM_001185114	chr1:156590110-156593967	non-template	39	Intron_1.f	--	Yes	--	Yes	--	--	--	--	--	--	--
G4ST01156590505	chr1:156590506-156590544	+	GGGCTGAGAGGAAGCTCTGGGGAGGTCCTGGGGGAAGGG<br>...((((....)))).	CD1E	NM_001185113	chr1:156590110-156593967	non-template	39	Intron_1.f	--	Yes	--	Yes	--	--	--	--	--	--	--
G4ST01156590505	chr1:156590506-156590544	+	GGGCTGAGAGGAAGCTCTGGGGAGGTCCTGGGGGAAGGG<br>...((((....)))).	CD1E	NM_001185108	chr1:156590110-156593967	non-template	39	Intron_1.f	--	Yes	--	Yes	--	--	--	--	--	--	--
G4ST01156590505	chr1:156590506-156590544	+	GGGCTGAGAGGAAGCTCTGGGGAGGTCCTGGGGGAAGGG<br>...((((....)))).	CD1E	NM_001185107	chr1:156590110-156593967	non-template	39	Intron_1.f	--	Yes	--	Yes	--	--	--	--	--	--	--
G4ST01156590505	chr1:156590506-156590544	+	GGGCTGAGAGGAAGCTCTGGGGAGGTCCTGGGGGAAGGG<br>...((((....)))).	CD1E	NM_001185115	chr1:156590110-156593967	non-template	39	Intron_1.f	--	Yes	--	Yes	--	--	--	--	--	--	--
G4ST01156590505	chr1:156590506-156590544	+	GGGCTGAGAGGAAGCTCTGGGGAGGTCCTGGGGGAAGGG<br>...((((....)))).	CD1E	NM_001042584	chr1:156590110-156593967	non-template	39	Intron_1.f	--	Yes	--	Yes	--	--	--	--	--	--	--
G4ST01156590505	chr1:156590506-156590544	+	GGGCTGAGAGGAAGCTCTGGGGAGGTCCTGGGGGAAGGG<br>...((((....)))).	CD1E	NM_001042585	chr1:156590110-156593967	non-template	39	Intron_1.f	--	Yes	--	Yes	--	--	--	--	--	--	--
G4ST01156590505	chr1:156590506-156590544	+	GGGCTGAGAGGAAGCTCTGGGGAGGTCCTGGGGGAAGGG<br>...((((....)))).	CD1E	NM_001042587	chr1:156590110-156593967	non-template	39	Intron_1.f	--	Yes	--	Yes	--	--	--	--	--	--	--
G4ST01156590505	chr1:156590506-156590544	+	GGGCTGAGAGGAAGCTCTGGGGAGGTCCTGGGGGAAGGG<br>...((((....)))).	CD1E	NM_001042586	chr1:156590110-156593967	non-template	39	Intron_1.f	--	Yes	--	Yes	--	--	--	--	--	--	--
G4ST01156590505	chr1:156590506-156590544	+	GGGCTGAGAGGAAGCTCTGGGGAGGTCCTGGGGGAAGGG<br>...((((....)))).	CD1E	NM_030893	chr1:156590110-156593967	non-template	39	Intron_1.f	--	Yes	--	Yes	--	--	--	--	--	--	--
G4ST01157313721	chr1:157313722-157313753	+	GGGAAATGGGCGGGCAATTCCCGGAATCGGGG<br>..((((...))))..	AIM2	NM_004833	chr1:157298899-157313271	template	-450	upstream	Yes	--	--	--	--	--	--	Yes	Yes	Yes	Yes
G4ST01157408275	chr1:157408276-157408304	+	GGGAGGGGGCGGCGCCTGGGGAGGTGGGG<br>(((...)))..	CADM3	NM_001127173	chr1:157408038-157439556	non-template	29	Intron_1.f	--	Yes	--	Yes	--	--	--	Yes	--	--	--
G4ST01157408275	chr1:157408276-157408304	+	GGGAGGGGGCGGCGCCTGGGGAGGTGGGG<br>(((...)))..	CADM3	NM_021189	chr1:157408001-157439556	non-template	29	Intron_1.f	--	Yes	--	Yes	--	--	--	Yes	--	--	--
G4ST01158091458	chr1:158091459-158091489	+	GGGCGGGTGAGCGGGCTGCTCCGCAGCGGGG<br>((((...))))..	VSIG8	NM_001013661	chr1:158090730-158099071	template	31	Intron_6.l	--	Yes	--	Yes	--	--	--	--	Yes	Yes	--
G4ST01158091458	chr1:158091459-158091489	+	GGGCGGGTGAGCGGGCTGCTCCGCAGCGGGG<br>((((...))))..	C1orf204	NM_001134233	chr1:158070888-158091761	template	31	Intron_1.f	--	Yes	--	Yes	--	--	--	--	Yes	Yes	--
G4ST01158091462	chr1:158091463-158091495	+	GGGTGAGCGGGCTGCTCCGCAGCGGGGCGTGGG<br>((((...))))..	VSIG8	NM_001013661	chr1:158090730-158099071	template	33	Intron_6.l	--	Yes	--	Yes	--	--	--	--	Yes	Yes	--
G4ST01158091462	chr1:158091463-158091495	+	GGGTGAGCGGGCTGCTCCGCAGCGGGGCGTGGG<br>((((...))))..	C1orf204	NM_001134233	chr1:158070888-158091761	template	33	Intron_1.f	--	Yes	--	Yes	--	--	--	--	Yes	Yes	--
G4ST01158099191	chr1:158099192-158099216	-	GGGAAGAGCTTGGGTGAGGGCTGGG<br>((....))	VSIG8	NM_001013661	chr1:158090730-158099071	non-template	-120	upstream	Yes	--	--	--	--	--	--	--	--	--	--
G4ST01158165011	chr1:158165012-158165062	+	GGGCAGGCTCAGGTACTGGAGCAGGGGTCCAGGACCTGGCAAGGGACTGGG<br>...((((((...)).)))).. ..((((...))))...	IGSF9	NM_020789	chr1:158163453-158182010	template	51	Exon_19	--	Yes	Yes	--	Yes	--	--	--	--	--	--
G4ST01158165011	chr1:158165012-158165062	+	GGGCAGGCTCAGGTACTGGAGCAGGGGTCCAGGACCTGGCAAGGGACTGGG<br>...((((((...)).)))).. ..((((...))))...	IGSF9	NM_001135050	chr1:158163453-158182010	template	51	Exon_19	--	Yes	Yes	--	Yes	--	--	--	--	--	--
G4ST01158165034	chr1:158165035-158165067	+	GGGGTCCAGGACCTGGCAAGGGACTGGGTGGGG<br>..((((...))))...	IGSF9	NM_020789	chr1:158163453-158182010	template	33	Exon_19	--	Yes	Yes	--	Yes	--	--	--	--	--	--
G4ST01158165034	chr1:158165035-158165067	+	GGGGTCCAGGACCTGGCAAGGGACTGGGTGGGG<br>..((((...))))...	IGSF9	NM_001135050	chr1:158163453-158182010	template	33	Exon_19	--	Yes	Yes	--	Yes	--	--	--	--	--	--
G4ST01158167759	chr1:158167760-158167788	-	GGGTGTGGGACCCCTGGGATGATGATGGG<br>((....))	IGSF9	NM_020789	chr1:158163453-158182010	non-template	29	Intron_12	--	Yes	--	Yes	--	--	--	--	--	--	--
G4ST01158167759	chr1:158167760-158167788	-	GGGTGTGGGACCCCTGGGATGATGATGGG<br>((....))	IGSF9	NM_001135050	chr1:158163453-158182010	non-template	29	Intron_12	--	Yes	--	Yes	--	--	--	--	--	--	--
G4ST01158168885	chr1:158168886-158168917	-	GGGGGTGGGCGGGGGCCTGTGGTGACAGTGGG<br>...((((....)))).	IGSF9	NM_020789	chr1:158163453-158182010	non-template	32	Intron_10	--	Yes	--	Yes	--	--	--	--	--	--	--
G4ST01158168885	chr1:158168886-158168917	-	GGGGGTGGGCGGGGGCCTGTGGTGACAGTGGG<br>...((((....)))).	IGSF9	NM_001135050	chr1:158163453-158182010	non-template	32	Intron_10	--	Yes	--	Yes	--	--	--	--	--	--	--
G4ST01158174989	chr1:158174990-158175025	-	GGGGTGCATAGTTGCATCCAGGGAGGGTGCTGAGGG<br>(((((....)))))...	IGSF9	NM_020789	chr1:158163453-158182010	non-template	36	Intron_3	--	Yes	--	Yes	--	--	--	Yes	Yes	--	Yes
G4ST01158174989	chr1:158174990-158175025	-	GGGGTGCATAGTTGCATCCAGGGAGGGTGCTGAGGG<br>(((((....)))))...	IGSF9	NM_001135050	chr1:158163453-158182010	non-template	36	Intron_3	--	Yes	--	Yes	--	--	--	Yes	Yes	--	Yes
G4ST01158181264	chr1:158181265-158181308	-	GGGAAGTGTGGGAGAGCTGCCTGGAGGCAGTGGGAAACAGGGGG<br>....(((((....))))).	IGSF9	NM_020789	chr1:158163453-158182010	non-template	44	Intron_1.f	--	Yes	--	Yes	--	--	--	--	--	--	--
G4ST01158181264	chr1:158181265-158181308	-	GGGAAGTGTGGGAGAGCTGCCTGGAGGCAGTGGGAAACAGGGGG<br>....(((((....))))).	IGSF9	NM_001135050	chr1:158163453-158182010	non-template	44	Intron_1.f	--	Yes	--	Yes	--	--	--	--	--	--	--
G4ST01158190860	chr1:158190861-158190894	-	GGGGCATGCAGTACGTGCGGGGACCTGGGGCGGG<br>(((((.....))))).	SLAMF9	NM_001146172	chr1:158187906-158190668	non-template	-192	upstream	Yes	--	--	--	--	--	--	--	Yes	--	--
G4ST01158190860	chr1:158190861-158190894	-	GGGGCATGCAGTACGTGCGGGGACCTGGGGCGGG<br>(((((.....))))).	SLAMF9	NM_033438	chr1:158187906-158190668	non-template	-192	upstream	Yes	--	--	--	--	--	--	--	Yes	--	--
G4ST01158190860	chr1:158190861-158190894	-	GGGGCATGCAGTACGTGCGGGGACCTGGGGCGGG<br>(((((.....))))).	SLAMF9	NM_001146173	chr1:158187906-158190668	non-template	-192	upstream	Yes	--	--	--	--	--	--	--	Yes	--	--
G4ST01158322507	chr1:158322508-158322539	-	GGGCTTCGAGGAGGAGAGGGCAGGGCTGGGGG<br>((((.....)))).	KCNJ9	NM_004983	chr1:158317984-158325836	template	32	Intron_2.l	--	Yes	--	Yes	--	--	--	--	--	--	--
G4ST01158322514	chr1:158322515-158322549	-	GGGGAGAGAAGGGCTTCGAGGAGGAGAGGGCAGGG<br>((((.....)))).	KCNJ9	NM_004983	chr1:158317984-158325836	template	35	Intron_2.l	--	Yes	--	Yes	--	--	--	--	--	--	--
G4ST01158322519	chr1:158322520-158322557	-	GGGACACTGGGGAGAGAAGGGCTTCGAGGAGGAGAGGG<br>((((.....)))).	KCNJ9	NM_004983	chr1:158317984-158325836	template	38	Intron_2.l	--	Yes	--	Yes	--	--	--	--	--	--	--
G4ST01158351916	chr1:158351917-158351947	-	GGGAAGGGGGCCTGGGTCCCAGGTTGGGGGG<br>.((((...))))	ATP1A2	NM_000702	chr1:158352144-158379998	template	-196	upstream	Yes	--	--	--	--	--	--	--	--	--	--
G4ST01158352052	chr1:158352053-158352079	+	GGGGCGGGAGGGGAGGAGTCCTCAGGG<br>((((...)))).	ATP1A2	NM_000702	chr1:158352144-158379998	non-template	-64	upstream	Yes	--	--	--	--	--	--	Yes	Yes	--	--
G4ST01158426759	chr1:158426760-158426791	+	GGGGGAGGGAAGGAGCCCCTGGGAGCCTGGGG<br>.(((....)))	CASQ1	NM_001231	chr1:158426909-158438300	non-template	-117	upstream	Yes	--	--	--	--	--	--	--	--	--	--
G4ST01158436048	chr1:158436049-158436075	-	GGGAGAGAATCGGGAGGGTCAGCTGGG<br>.((...))	CASQ1	NM_001231	chr1:158426909-158438300	template	27	Intron_9	--	Yes	--	Yes	--	--	--	--	--	--	--
G4ST01158461011	chr1:158461012-158461040	+	GGGACCAGGAGGTGGGGGTTGGGGTGGGG<br>.(((.....)))	DCAF8	NR_028103	chr1:158452129-158498942	template	29	Intron_9	--	Yes	--	Yes	--	--	--	--	--	--	--
G4ST01158461011	chr1:158461012-158461040	+	GGGACCAGGAGGTGGGGGTTGGGGTGGGG<br>.(((.....)))	DCAF8	NR_028104	chr1:158452129-158498942	template	29	Intron_8	--	Yes	--	Yes	--	--	--	--	--	--	--
G4ST01158461011	chr1:158461012-158461040	+	GGGACCAGGAGGTGGGGGTTGGGGTGGGG<br>.(((.....)))	DCAF8	NM_015726	chr1:158452129-158498974	template	29	Intron_9	--	Yes	--	Yes	--	--	--	--	--	--	--

G4ST01158603399	chr1:158603400-158603435	+	GGGGTGGGCCTGGGCCAGCCAATGGAGAGCTGGGGG<br>(((((.........)))))	NHLH1	NM_005598	chr1:158603485-158609262	non-template	-49	upstream	Yes	--	--	--	--	--	--	Yes	Yes	Yes	Yes
G4ST01158605089	chr1:158605090-158605123	+	GGGGAACTGGGAGGGGAGCTGTGCAGAGCTGGGG<br>.((((......)))).	NHLH1	NM_005598	chr1:158603485-158609262	non-template	34	Intron_1.u	--	Yes	--	Yes	--	--	--	Yes	Yes	--	Yes
G4ST01158636810	chr1:158636811-158636845	+	GGGCTGGGGGAGCGGCGACTCCGGGAAGAGCGGGG<br>((((......))))	VANGL2	NM_020335	chr1:158636988-158665092	non-template	-142	upstream	Yes	--	--	--	--	--	--	Yes	Yes	Yes	--
G4ST01158636907	chr1:158636908-158636944	-	GGGGGGTGCAGGGGGCGGGGGAGCGCGAGCTCCCGGG<br>(((((....))))).	VANGL2	NM_020335	chr1:158636988-158665092	template	-43	upstream	Yes	--	--	--	--	--	--	Yes	Yes	Yes	Yes
G4ST01158636957	chr1:158636958-158636994	-	GGGAGCCGGCGGCGCAGGGGGGAGGGGGCGGAGGGGG<br>..((.((...)).)).	VANGL2	NM_020335	chr1:158636988-158665092	template	7	upstream-Exon_1.f	Yes	Yes	Yes	--	--	Yes	--	Yes	Yes	Yes	Yes
G4ST01158646196	chr1:158646197-158646227	+	GGGATCCTGGGCTGGGAGGTTGGACCCTGGG<br>.(((...)))..	VANGL2	NM_020335	chr1:158636988-158665092	non-template	31	Intron_1.f	--	Yes	--	Yes	--	--	--	--	--	--	--
G4ST01158663737	chr1:158663738-158663765	+	GGGGTGGGGGAGAGGGAAAGGATTTGGG<br>(((...)))	VANGL2	NM_020335	chr1:158636988-158665092	non-template	28	Exon_8.l	--	Yes	Yes	--	--	--	Yes	--	--	--	--
G4ST01158789222	chr1:158789223-158789259	+	GGGAAAAGGAAGTTCTTGGGTGGACATGGGATGTGGG<br>..((((....))))	CD84	NM_001184881	chr1:158777508-158815930	template	37	Intron_4	--	Yes	--	Yes	--	--	--	--	Yes	--	--
G4ST01158789222	chr1:158789223-158789259	+	GGGAAAAGGAAGTTCTTGGGTGGACATGGGATGTGGG<br>..((((....))))	CD84	NM_001184879	chr1:158777508-158815930	template	37	Intron_4	--	Yes	--	Yes	--	--	--	--	Yes	--	--
G4ST01158789222	chr1:158789223-158789259	+	GGGAAAAGGAAGTTCTTGGGTGGACATGGGATGTGGG<br>..((((....))))	CD84	NM_001184882	chr1:158777508-158815930	template	37	Intron_3	--	Yes	--	Yes	--	--	--	--	Yes	--	--
G4ST01158789222	chr1:158789223-158789259	+	GGGAAAAGGAAGTTCTTGGGTGGACATGGGATGTGGG<br>..((((....))))	CD84	NM_003874	chr1:158777508-158815930	template	37	Intron_4	--	Yes	--	Yes	--	--	--	--	Yes	--	--
G4ST01158808076	chr1:158808077-158808101	+	GGGGGCTGGGGGGGAGGAGCTAGGG<br>((...)).	CD84	NM_001184881	chr1:158777508-158815930	template	25	Intron_1.f	--	Yes	--	Yes	--	--	--	--	--	--	--
G4ST01158808076	chr1:158808077-158808101	+	GGGGGCTGGGGGGGAGGAGCTAGGG<br>((...)).	CD84	NM_001184879	chr1:158777508-158815930	template	25	Intron_1.f	--	Yes	--	Yes	--	--	--	--	--	--	--
G4ST01158808076	chr1:158808077-158808101	+	GGGGGCTGGGGGGGAGGAGCTAGGG<br>((...)).	CD84	NM_001184882	chr1:158777508-158815930	template	25	Intron_1.f	--	Yes	--	Yes	--	--	--	--	--	--	--
G4ST01158808076	chr1:158808077-158808101	+	GGGGGCTGGGGGGGAGGAGCTAGGG<br>((...)).	CD84	NM_003874	chr1:158777508-158815930	template	25	Intron_1.f	--	Yes	--	Yes	--	--	--	--	--	--	--
G4ST01158808083	chr1:158808084-158808105	+	GGGGGGGAGGAGCTAGGGAGGG<br>((...)).	CD84	NM_001184881	chr1:158777508-158815930	template	22	Intron_1.f	--	Yes	--	Yes	--	--	--	--	--	--	--
G4ST01158808083	chr1:158808084-158808105	+	GGGGGGGAGGAGCTAGGGAGGG<br>((...)).	CD84	NM_001184879	chr1:158777508-158815930	template	22	Intron_1.f	--	Yes	--	Yes	--	--	--	--	--	--	--
G4ST01158808083	chr1:158808084-158808105	+	GGGGGGGAGGAGCTAGGGAGGG<br>((...)).	CD84	NM_001184882	chr1:158777508-158815930	template	22	Intron_1.f	--	Yes	--	Yes	--	--	--	--	--	--	--
G4ST01158808083	chr1:158808084-158808105	+	GGGGGGGAGGAGCTAGGGAGGG<br>((...)).	CD84	NM_003874	chr1:158777508-158815930	template	22	Intron_1.f	--	Yes	--	Yes	--	--	--	--	--	--	--
G4ST01158871152	chr1:158871153-158871182	+	GGGAGCTGTTGGCTGGGTTCAGTGGGTGGG<br>(((.....)))	SLAMF1	NM_003037	chr1:158846515-158883705	template	30	Exon_3	--	Yes	Yes	--	Yes	--	--	--	Yes	Yes	--
G4ST01159098971	chr1:159098972-159099008	-	GGGGCGAGGGAGGGACTAGGAGCTGTGCCCCTCAGGG<br>...(((.((...)).)))..	CD244	NM_001166664	chr1:159066574-159099316	non-template	37	Intron_1.f	--	Yes	--	Yes	--	--	--	--	Yes	Yes	--
G4ST01159098971	chr1:159098972-159099008	-	GGGGCGAGGGAGGGACTAGGAGCTGTGCCCCTCAGGG<br>...(((.((...)).)))..	CD244	NM_001166663	chr1:159066574-159099316	non-template	37	Intron_1.f	--	Yes	--	Yes	--	--	--	--	Yes	Yes	--
G4ST01159098971	chr1:159098972-159099008	-	GGGGCGAGGGAGGGACTAGGAGCTGTGCCCCTCAGGG<br>...(((.((...)).)))..	CD244	NM_016382	chr1:159066574-159099316	non-template	37	Intron_1.f	--	Yes	--	Yes	--	--	--	--	Yes	Yes	--
G4ST01159191289	chr1:159191290-159191326	-	GGGGCTAGCAGCCTAGGCTGCTCAGGGTTGGGCTGGG<br>...((((((....))))))..	ITLN2	NM_080878	chr1:159181440-159191213	non-template	-76	upstream	Yes	--	--	--	--	--	--	Yes	--	--	Yes
G4ST01159191294	chr1:159191295-159191330	-	GGGAGGGGCTAGCAGCCTAGGCTGCTCAGGGTTGGG<br>...((((((....))))))..	ITLN2	NM_080878	chr1:159181440-159191213	non-template	-81	upstream	Yes	--	--	--	--	--	--	Yes	--	--	Yes
G4ST01159191299	chr1:159191300-159191340	-	GGGCTTCAGAGGGAGGGGCTAGCAGCCTAGGCTGCTCAGGG<br>...((((((....))))))..	ITLN2	NM_080878	chr1:159181440-159191213	non-template	-86	upstream	Yes	--	--	--	--	--	--	Yes	--	--	Yes
G4ST01159285459	chr1:159285460-159285494	-	GGGTGGGGAAGAGCAGGCATGCTGGGAAGTTGGGG<br>....((((....))))	ARHGAP30	NM_181720	chr1:159283356-159306384	non-template	35	Exon_12	--	Yes	Yes	--	Yes	--	--	--	--	Yes	--
G4ST01159285459	chr1:159285460-159285494	-	GGGTGGGGAAGAGCAGGCATGCTGGGAAGTTGGGG<br>....((((....))))	ARHGAP30	NM_001025598	chr1:159283356-159306384	non-template	35	Exon_12.l	--	Yes	Yes	--	Yes	--	--	--	--	Yes	--
G4ST01159285468	chr1:159285469-159285500	-	GGGACAGGGTGGGGAAGAGCAGGCATGCTGGG<br>....((((....))))	ARHGAP30	NM_181720	chr1:159283356-159306384	non-template	32	Exon_12	--	Yes	Yes	--	Yes	--	--	--	--	Yes	--
G4ST01159285468	chr1:159285469-159285500	-	GGGACAGGGTGGGGAAGAGCAGGCATGCTGGG<br>....((((....))))	ARHGAP30	NM_001025598	chr1:159283356-159306384	non-template	32	Exon_12.l	--	Yes	Yes	--	Yes	--	--	--	--	Yes	--
G4ST01159285783	chr1:159285784-159285820	-	GGGTTTGGGGGTGGCATGGAGTCAAGGGTGGACAGGG<br>..((((.....)))).	ARHGAP30	NM_181720	chr1:159283356-159306384	non-template	37	Intron_11	--	Yes	--	Yes	--	--	--	--	--	Yes	--
G4ST01159285783	chr1:159285784-159285820	-	GGGTTTGGGGGTGGCATGGAGTCAAGGGTGGACAGGG<br>..((((.....)))).	ARHGAP30	NM_001025598	chr1:159283356-159306384	non-template	37	Intron_11.l	--	Yes	--	Yes	--	--	--	--	--	Yes	--
G4ST01159311274	chr1:159311275-159311310	-	GGGAGGGGCACCGTGGGAACAGCCTGCGGCTCTGGG<br>...((((...))))..	PVRL4	NM_030916	chr1:159307405-159326009	non-template	36	Intron_4	--	Yes	--	Yes	--	--	--	Yes	Yes	--	--
G4ST01159320148	chr1:159320149-159320180	-	GGGGGCCTGGCCAGGGTGGGGGAGGCCAAGGG<br>(((...))).	PVRL4	NM_030916	chr1:159307405-159326009	non-template	32	Intron_1.f	--	Yes	--	Yes	--	--	--	Yes	Yes	Yes	Yes
G4ST01159326090	chr1:159326091-159326116	+	GGGAGGGAAGGAGGGAAGGACCTGGG<br>.((...))	PVRL4	NM_030916	chr1:159307405-159326009	template	-81	upstream	Yes	--	--	--	--	--	--	--	--	--	Yes
G4ST01159326094	chr1:159326095-159326122	+	GGGAAGGAGGGAAGGACCTGGGTGGGGG<br>.((...))	PVRL4	NM_030916	chr1:159307405-159326009	template	-85	upstream	Yes	--	--	--	--	--	--	--	--	--	Yes
G4ST01159326102	chr1:159326103-159326130	+	GGGAAGGACCTGGGTGGGGGCGGTGGGG<br>.((...))	PVRL4	NM_030916	chr1:159307405-159326009	template	-93	upstream	Yes	--	--	--	--	--	--	--	--	--	Yes
G4ST01159368792	chr1:159368793-159368827	-	GGGAGGTGACGGGACGCTGAGAGGTGGGGGAGGGG<br>.((((....)))).	DEDD	NM_001039712	chr1:159357392-159368880	non-template	35	Exon_1.f-Intron_1.f	--	Yes	Yes	Yes	--	Yes	--	Yes	Yes	Yes	--
G4ST01159368792	chr1:159368793-159368827	-	GGGAGGTGACGGGACGCTGAGAGGTGGGGGAGGGG<br>.((((....)))).	DEDD	NM_001039711	chr1:159357392-159369102	non-template	35	Intron_1.f	--	Yes	--	Yes	--	--	--	Yes	Yes	Yes	--
G4ST01159368792	chr1:159368793-159368827	-	GGGAGGTGACGGGACGCTGAGAGGTGGGGGAGGGG<br>.((((....)))).	DEDD	NM_032998	chr1:159357392-159369102	non-template	35	Intron_1.f	--	Yes	--	Yes	--	--	--	Yes	Yes	Yes	--
G4ST01159429568	chr1:159429569-159429611	+	GGGAGCACTGCGGGTGTGTGTGACCATAGACAGGGCCTGGGGG<br>.((...)) .((.(((....))).)).	ADAMTS4	NM_005099	chr1:159426162-159435469	template	43	Intron_7	--	Yes	--	Yes	--	--	--	--	--	--	--
G4ST01159434872	chr1:159434873-159434903	+	GGGGGAGGGGGCTGGCCAGCCGGGCTGAGGG<br>((((....))))	ADAMTS4	NM_005099	chr1:159426162-159435469	template	31	Exon_1.f	--	Yes	Yes	--	Yes	--	--	Yes	Yes	Yes	--
G4ST01159434872	chr1:159434873-159434903	+	GGGGGAGGGGGCTGGCCAGCCGGGCTGAGGG<br>((((....))))	NDUFS2	NM_004550	chr1:159435729-159450808	non-template	-825	upstream	Yes	--	--	--	--	--	--	Yes	Yes	Yes	--
G4ST01159474739	chr1:159474740-159474762	+	GGGGAGGGACTCCAGTGGGTGGG<br>((....))	NR1I3	NM_005122	chr1:159466080-159474624	template	-115	upstream	Yes	--	--	--	--	--	--	Yes	Yes	--	--
G4ST01159474739	chr1:159474740-159474762	+	GGGGAGGGACTCCAGTGGGTGGG<br>((....))	NR1I3	NM_001077471	chr1:159466080-159474624	template	-115	upstream	Yes	--	--	--	--	--	--	Yes	Yes	--	--
G4ST01159474739	chr1:159474740-159474762	+	GGGGAGGGACTCCAGTGGGTGGG<br>((....))	NR1I3	NM_001077476	chr1:159466080-159474624	template	-115	upstream	Yes	--	--	--	--	--	--	Yes	Yes	--	--
G4ST01159474739	chr1:159474740-159474762	+	GGGGAGGGACTCCAGTGGGTGGG<br>((....))	NR1I3	NM_001077481	chr1:159466080-159474624	template	-115	upstream	Yes	--	--	--	--	--	--	Yes	Yes	--	--
G4ST01159474739	chr1:159474740-159474762	+	GGGGAGGGACTCCAGTGGGTGGG<br>((....))	NR1I3	NM_001077482	chr1:159466080-159474624	template	-115	upstream	Yes	--	--	--	--	--	--	Yes	Yes	--	--
G4ST01159474739	chr1:159474740-159474762	+	GGGGAGGGACTCCAGTGGGTGGG<br>((....))	NR1I3	NM_001077469	chr1:159466080-159474624	template	-115	upstream	Yes	--	--	--	--	--	--	Yes	Yes	--	--
G4ST01159474739	chr1:159474740-159474762	+	GGGGAGGGACTCCAGTGGGTGGG<br>((....))	NR1I3	NM_001077474	chr1:159466080-159474624	template	-115	upstream	Yes	--	--	--	--	--	--	Yes	Yes	--	--

G4ST01159474739	chr1:159474740-159474762	+	GGGGAGGGACTCCAGTGGGTGGG<br>((....))	NR1I3	NM_001077473	chr1:159466080-159474624	template	-115	upstream	Yes	--	--	--	--	--	--	Yes	Yes	--	--
G4ST01159474739	chr1:159474740-159474762	+	GGGGAGGGACTCCAGTGGGTGGG<br>((....))	NR1I3	NM_001077472	chr1:159466080-159474624	template	-115	upstream	Yes	--	--	--	--	--	--	Yes	Yes	--	--
G4ST01159474739	chr1:159474740-159474762	+	GGGGAGGGACTCCAGTGGGTGGG<br>((....))	NR1I3	NM_001077479	chr1:159466080-159474624	template	-115	upstream	Yes	--	--	--	--	--	--	Yes	Yes	--	--
G4ST01159474739	chr1:159474740-159474762	+	GGGGAGGGACTCCAGTGGGTGGG<br>((....))	NR1I3	NM_001077480	chr1:159466080-159474624	template	-115	upstream	Yes	--	--	--	--	--	--	Yes	Yes	--	--
G4ST01159474739	chr1:159474740-159474762	+	GGGGAGGGACTCCAGTGGGTGGG<br>((....))	NR1I3	NM_001077475	chr1:159466080-159474624	template	-115	upstream	Yes	--	--	--	--	--	--	Yes	Yes	--	--
G4ST01159474739	chr1:159474740-159474762	+	GGGGAGGGACTCCAGTGGGTGGG<br>((....))	NR1I3	NM_001077470	chr1:159466080-159474624	template	-115	upstream	Yes	--	--	--	--	--	--	Yes	Yes	--	--
G4ST01159474739	chr1:159474740-159474762	+	GGGGAGGGACTCCAGTGGGTGGG<br>((....))	NR1I3	NM_001077478	chr1:159466080-159474624	template	-115	upstream	Yes	--	--	--	--	--	--	Yes	Yes	--	--
G4ST01159474739	chr1:159474740-159474762	+	GGGGAGGGACTCCAGTGGGTGGG<br>((....))	NR1I3	NM_001077477	chr1:159466080-159474624	template	-115	upstream	Yes	--	--	--	--	--	--	Yes	Yes	--	--
G4ST01159495281	chr1:159495282-159495329	-	GGGGCTCCGCGGGCGACAGCTGAAAGTTGCCGGGCTCTGCGGCAGGGG<br>...(((((...))))).. ..(((...)))	PCP4L1	NM_001102566	chr1:159495141-159521864	template	48	Exon_1.f	--	Yes	Yes	--	--	Yes	--	Yes	Yes	--	--
G4ST01159542661	chr1:159542662-159542691	-	GGGCGGAGACTACTCCAGTGGGTGGGAGGG<br>.((((....))))....	MPZ	NM_000530	chr1:159541149-159546386	non-template	30	Intron_4	--	Yes	--	Yes	--	--	--	Yes	Yes	Yes	Yes
G4ST01159542824	chr1:159542825-159542864	-	GGGGTCGTTCTGGGAGCTGTGATCGGGGGTGTCCTCGGGG<br>..(((....)))	MPZ	NM_000530	chr1:159541149-159546386	non-template	40	Exon_4	--	Yes	Yes	--	Yes	--	--	Yes	Yes	Yes	--
G4ST01159546491	chr1:159546492-159546525	+	GGGGAGGGGGGCAGAGTGCAGGGAGCAGAAGGGG<br>..(((...))).	MPZ	NM_000530	chr1:159541149-159546386	template	-105	upstream	Yes	--	--	--	--	--	--	Yes	--	--	--
G4ST01159760825	chr1:159760826-159760855	+	GGGCGGGCGAGAGGCTCTCAACTGGGCGGG<br>.((((...))))....	HSPA6	NM_002155	chr1:159760660-159763311	non-template	30	Exon_1.u	--	Yes	Yes	--	--	Yes	--	Yes	Yes	Yes	--
G4ST01159760829	chr1:159760830-159760865	+	GGGCGAGAGGCTCTCAACTGGGCGGGAAGGTGCGGG<br>.((((...))))....	HSPA6	NM_002155	chr1:159760660-159763311	non-template	36	Exon_1.u	--	Yes	Yes	--	--	Yes	--	Yes	Yes	Yes	--
G4ST01159824741	chr1:159824742-159824770	+	GGGCGCTCTGGGCCTGGGCAGTCTCTGGG<br>.((...))	FCGR2C	NM_201563	chr1:159817753-159836656	non-template	29	Intron_1.f	--	Yes	--	Yes	--	--	--	--	--	--	--
G4ST01159824750	chr1:159824751-159824780	+	GGGCCTGGGCAGTCTCTGGGCCAAAGTGGG<br>.((...))	FCGR2C	NM_201563	chr1:159817753-159836656	non-template	30	Intron_1.f	--	Yes	--	Yes	--	--	--	--	--	--	--
G4ST01159906571	chr1:159906572-159906600	+	GGGCGCTCTGGGCCTGGGCAGTCTCTGGG<br>.((...))	FCGR2B	NM_004001	chr1:159899529-159915068	non-template	29	Intron_1.f	--	Yes	--	Yes	--	--	--	--	--	--	--
G4ST01159906571	chr1:159906572-159906600	+	GGGCGCTCTGGGCCTGGGCAGTCTCTGGG<br>.((...))	FCGR2B	NM_001190828	chr1:159899529-159915068	non-template	29	Intron_1.f	--	Yes	--	Yes	--	--	--	--	--	--	--
G4ST01159906571	chr1:159906572-159906600	+	GGGCGCTCTGGGCCTGGGCAGTCTCTGGG<br>.((...))	FCGR2B	NM_001002275	chr1:159899529-159915068	non-template	29	Intron_1.f	--	Yes	--	Yes	--	--	--	--	--	--	--
G4ST01159906571	chr1:159906572-159906600	+	GGGCGCTCTGGGCCTGGGCAGTCTCTGGG<br>.((...))	FCGR2B	NM_001002274	chr1:159899529-159915068	non-template	29	Intron_1.f	--	Yes	--	Yes	--	--	--	--	--	--	--
G4ST01159906571	chr1:159906572-159906600	+	GGGCGCTCTGGGCCTGGGCAGTCTCTGGG<br>.((...))	FCGR2B	NM_001002273	chr1:159899529-159915068	non-template	29	Intron_1.f	--	Yes	--	Yes	--	--	--	--	--	--	--
G4ST01159906580	chr1:159906581-159906610	+	GGGCCTGGGCAGTCTCTGGGCCAAAGTGGG<br>.((...))	FCGR2B	NM_004001	chr1:159899529-159915068	non-template	30	Intron_1.f	--	Yes	--	Yes	--	--	--	--	--	--	--
G4ST01159906580	chr1:159906581-159906610	+	GGGCCTGGGCAGTCTCTGGGCCAAAGTGGG<br>.((...))	FCGR2B	NM_001190828	chr1:159899529-159915068	non-template	30	Intron_1.f	--	Yes	--	Yes	--	--	--	--	--	--	--
G4ST01159906580	chr1:159906581-159906610	+	GGGCCTGGGCAGTCTCTGGGCCAAAGTGGG<br>.((...))	FCGR2B	NM_001002275	chr1:159899529-159915068	non-template	30	Intron_1.f	--	Yes	--	Yes	--	--	--	--	--	--	--
G4ST01159906580	chr1:159906581-159906610	+	GGGCCTGGGCAGTCTCTGGGCCAAAGTGGG<br>.((...))	FCGR2B	NM_001002274	chr1:159899529-159915068	non-template	30	Intron_1.f	--	Yes	--	Yes	--	--	--	--	--	--	--
G4ST01159906580	chr1:159906581-159906610	+	GGGCCTGGGCAGTCTCTGGGCCAAAGTGGG<br>.((...))	FCGR2B	NM_001002273	chr1:159899529-159915068	non-template	30	Intron_1.f	--	Yes	--	Yes	--	--	--	--	--	--	--
G4ST01159958684	chr1:159958685-159958719	+	GGGCTGGCTGGAAGGTGGAGAAGGGATGGGATGGG<br>(((.((...)).))))....	FCRLB	NM_001002901	chr1:159959081-159964557	non-template	-361	upstream	Yes	--	--	--	--	--	--	--	Yes	--	Yes
G4ST01159958711	chr1:159958712-159958736	+	GGGATGGGAGTGGGATGTCAATGGG<br>((....))	FCRLB	NM_001002901	chr1:159959081-159964557	non-template	-344	upstream	Yes	--	--	--	--	--	--	--	Yes	--	Yes
G4ST01159962922	chr1:159962923-159962955	-	GGGGCAGGGGGACCGAGTCCACGAGGGACGGGG<br>((((...))))....	FCRLB	NM_001002901	chr1:159959081-159964557	template	33	Intron_4	--	Yes	--	Yes	--	--	--	Yes	Yes	Yes	--
G4ST01159963140	chr1:159963141-159963180	+	GGGTGATGGGTCCGCGGGAGGCCCGCGGCGCGGCGCTGGG<br>..((((((...)))).)).	FCRLB	NM_001002901	chr1:159959081-159964557	non-template	40	Exon_5	--	Yes	Yes	--	Yes	--	--	Yes	Yes	Yes	--
G4ST01159963147	chr1:159963148-159963185	+	GGGTCCGCGGGAGGCCCGCGGCGCGGCGCTGGGTGGGG<br>..((((((...)))).)).	FCRLB	NM_001002901	chr1:159959081-159964557	non-template	38	Exon_5	--	Yes	Yes	--	Yes	--	--	Yes	Yes	Yes	--
G4ST01160018713	chr1:160018714-160018744	+	GGGCTTAGGGAATCAAAGGGAAGAGCCTGGG<br>.((...))	ATF6	NM_007348	chr1:160002708-160195476	non-template	31	Intron_3	--	Yes	--	Yes	--	--	--	--	Yes	--	--
G4ST01160306249	chr1:160306250-160306287	-	GGGGAGCGGTGGGGCTTGCGGCCCGAAGCGGGACTGGG<br>((((........))))	NOS1AP	NM_001164757	chr1:160306205-160606437	template	38	Exon_1.f	--	Yes	Yes	--	--	Yes	--	Yes	Yes	Yes	--
G4ST01160306249	chr1:160306250-160306287	-	GGGGAGCGGTGGGGCTTGCGGCCCGAAGCGGGACTGGG<br>((((........))))	NOS1AP	NM_014697	chr1:160306205-160606437	template	38	Exon_1.f	--	Yes	Yes	--	--	Yes	--	Yes	Yes	Yes	--
G4ST01160306255	chr1:160306256-160306292	-	GGGGAGGGGAGCGGTGGGGCTTGCGGCCCGAAGCGGG<br>((((........))))	NOS1AP	NM_001164757	chr1:160306205-160606437	template	37	Exon_1.f	--	Yes	Yes	--	--	Yes	--	Yes	Yes	Yes	--
G4ST01160306255	chr1:160306256-160306292	-	GGGGAGGGGAGCGGTGGGGCTTGCGGCCCGAAGCGGG<br>((((........))))	NOS1AP	NM_014697	chr1:160306205-160606437	template	37	Exon_1.f	--	Yes	Yes	--	--	Yes	--	Yes	Yes	Yes	--
G4ST01160306273	chr1:160306274-160306314	-	GGGCTGCGCGGCGCCCCTGCCCGGGGAGGGGAGCGGTGGGG<br>.....(((.((....)))))	NOS1AP	NM_001164757	chr1:160306205-160606437	template	41	Exon_1.f	--	Yes	Yes	--	--	Yes	--	Yes	Yes	Yes	--
G4ST01160306273	chr1:160306274-160306314	-	GGGCTGCGCGGCGCCCCTGCCCGGGGAGGGGAGCGGTGGGG<br>.....(((.((....)))))	NOS1AP	NM_014697	chr1:160306205-160606437	template	41	Exon_1.f	--	Yes	Yes	--	--	Yes	--	Yes	Yes	Yes	--
G4ST01160306283	chr1:160306284-160306320	-	GGGAGCGGGCTGCGCGGCGCCCCTGCCCGGGGAGGGG<br>.....(((.((....)))))	NOS1AP	NM_001164757	chr1:160306205-160606437	template	37	Exon_1.f	--	Yes	Yes	--	--	Yes	--	Yes	Yes	Yes	--
G4ST01160306283	chr1:160306284-160306320	-	GGGAGCGGGCTGCGCGGCGCCCCTGCCCGGGGAGGGG<br>.....(((.((....)))))	NOS1AP	NM_014697	chr1:160306205-160606437	template	37	Exon_1.f	--	Yes	Yes	--	--	Yes	--	Yes	Yes	Yes	--
G4ST01160306700	chr1:160306701-160306740	+	GGGGGCTCCGGGGAGGGTGCTACCTGTGGTGGCGGTTGGG<br>.((((((...))))))....	NOS1AP	NM_001164757	chr1:160306205-160606437	non-template	40	Intron_1.f	--	Yes	--	Yes	--	--	--	--	Yes	--	--
G4ST01160306700	chr1:160306701-160306740	+	GGGGGCTCCGGGGAGGGTGCTACCTGTGGTGGCGGTTGGG<br>.((((((...))))))....	NOS1AP	NM_014697	chr1:160306205-160606437	non-template	40	Intron_1.f	--	Yes	--	Yes	--	--	--	--	Yes	--	--
G4ST01160306709	chr1:160306710-160306744	+	GGGGAGGGTGCTACCTGTGGTGGCGGTTGGGGGGG<br>.((((((...))))))....	NOS1AP	NM_001164757	chr1:160306205-160606437	non-template	35	Intron_1.f	--	Yes	--	Yes	--	--	--	--	Yes	--	--
G4ST01160306709	chr1:160306710-160306744	+	GGGGAGGGTGCTACCTGTGGTGGCGGTTGGGGGGG<br>.((((((...))))))....	NOS1AP	NM_014697	chr1:160306205-160606437	non-template	35	Intron_1.f	--	Yes	--	Yes	--	--	--	--	Yes	--	--
G4ST01160556055	chr1:160556056-160556097	-	GGGCACTGAGGACAGTGGGGAGGGCTGCTGAGCAGATCTGGG<br>(((((....))))) ((((...))))....	NOS1AP	NM_001164757	chr1:160306205-160606437	template	42	Intron_4	--	Yes	--	Yes	--	--	--	--	--	--	--
G4ST01160556055	chr1:160556056-160556097	-	GGGCACTGAGGACAGTGGGGAGGGCTGCTGAGCAGATCTGGG<br>(((((....))))) ((((...))))....	NOS1AP	NM_014697	chr1:160306205-160606437	template	42	Intron_4	--	Yes	--	Yes	--	--	--	--	--	--	--
G4ST01160597136	chr1:160597137-160597175	-	GGGACTTGGGCAGTGCATGCCAGCGTGCTGGGGACTGGG<br>....(((((....)))))..	NOS1AP	NM_001164757	chr1:160306205-160606437	template	39	Intron_8	--	Yes	--	Yes	--	--	--	--	--	--	--
G4ST01160597136	chr1:160597137-160597175	-	GGGACTTGGGCAGTGCATGCCAGCGTGCTGGGGACTGGG<br>....(((((....)))))..	NOS1AP	NM_014697	chr1:160306205-160606437	template	39	Intron_8	--	Yes	--	Yes	--	--	--	--	--	--	--
G4ST01160616732	chr1:160616733-160616766	-	GGGCCTTTGGGTGGCAGGGAGGAAAACCTGTGGG<br>(((....)))..	C1orf226	NM_001135240	chr1:160615320-160620125	template	34	Intron_1.f	--	Yes	--	Yes	--	--	--	--	--	--	--
G4ST01160616732	chr1:160616733-160616766	-	GGGCCTTTGGGTGGCAGGGAGGAAAACCTGTGGG<br>(((....)))..	C1orf226	NM_001085375	chr1:160618144-160623232	template	-1377	upstream	Yes	--	--	--	--	--	--	--	--	--	--

G4ST01160798066	chr1:160798067-160798095	-	GGGGGGCCGCGGCGGCGGGGCGGGCCGGG<br>..((((...)))).	UAP1	NM_003115	chr1:160797920-160836257	template	29	Exon_1.f	--	Yes	Yes	--	--	Yes	--	--	Yes	Yes	Yes
G4ST01160969066	chr1:160969067-160969113	-	GGGCCTCTCTGGGCCCAGACAAGACTGACTGAGGGGCATTATTGGGG<br>..(((.((....)).)))...	DDR2	NM_006182	chr1:160868852-161016871	template	47	Intron_3	--	Yes	--	Yes	--	--	--	--	--	--	--
G4ST01160969066	chr1:160969067-160969113	-	GGGCCTCTCTGGGCCCAGACAAGACTGACTGAGGGGCATTATTGGGG<br>..(((.((....)).)))...	DDR2	NM_001014796	chr1:160868852-161016871	template	47	Intron_4	--	Yes	--	Yes	--	--	--	--	--	--	--
G4ST01161025153	chr1:161025154-161025187	+	GGGTGGGAGTGGGCCTGAGCAAGTGGCTCAAGGG<br>..(((((.....))))).	HSD17B7	NM_016371	chr1:161027120-161049232	non-template	-1932	upstream	Yes	--	--	--	--	--	--	--	--	--	--
G4ST01162835447	chr1:162835448-162835483	+	GGGGAGGGGCACAGGCCTGGCATGGGGATAACTGGG<br>...((.((...)).))	PBX1	NM_002585	chr1:162795221-163087684	non-template	36	Intron_2	--	Yes	--	Yes	--	--	--	--	Yes	--	--
G4ST01162835447	chr1:162835448-162835483	+	GGGGAGGGGCACAGGCCTGGCATGGGGATAACTGGG<br>...((.((...)).))	PBX1	NM_001204961	chr1:162795221-163087684	non-template	36	Intron_2	--	Yes	--	Yes	--	--	--	--	Yes	--	--
G4ST01162835447	chr1:162835448-162835483	+	GGGGAGGGGCACAGGCCTGGCATGGGGATAACTGGG<br>...((.((...)).))	PBX1	NM_001204963	chr1:162795221-163087684	non-template	36	Intron_2	--	Yes	--	Yes	--	--	--	--	Yes	--	--
G4ST01162835452	chr1:162835453-162835490	+	GGGGCACAGGCCTGGCATGGGGATAACTGGGAATGGGG<br>...((.((...)).))	PBX1	NM_002585	chr1:162795221-163087684	non-template	38	Intron_2	--	Yes	--	Yes	--	--	--	--	Yes	--	--
G4ST01162835452	chr1:162835453-162835490	+	GGGGCACAGGCCTGGCATGGGGATAACTGGGAATGGGG<br>...((.((...)).))	PBX1	NM_001204961	chr1:162795221-163087684	non-template	38	Intron_2	--	Yes	--	Yes	--	--	--	--	Yes	--	--
G4ST01162835452	chr1:162835453-162835490	+	GGGGCACAGGCCTGGCATGGGGATAACTGGGAATGGGG<br>...((.((...)).))	PBX1	NM_001204963	chr1:162795221-163087684	non-template	38	Intron_2	--	Yes	--	Yes	--	--	--	--	Yes	--	--
G4ST01163644630	chr1:163644631-163644664	+	GGGCTCTACATGGAACATGTGGGAAAGGGCAGGG<br>....(((((...)))))	RXRG	NM_006917	chr1:163636783-163681216	template	34	Intron_7	--	Yes	--	Yes	--	--	--	Yes	Yes	--	--
G4ST01163675242	chr1:163675243-163675275	-	GGGCTGATGAGCTGCTCAGGGTGGGAGCTGGGG<br>....((((...))))	RXRG	NM_006917	chr1:163636783-163681216	non-template	33	Intron_1.f	--	Yes	--	Yes	--	--	--	--	--	--	--
G4ST01163675242	chr1:163675243-163675275	-	GGGCTGATGAGCTGCTCAGGGTGGGAGCTGGGG<br>....((((...))))	RXRG	NR_033824	chr1:163672789-163681216	non-template	33	Intron_1.u	--	Yes	--	Yes	--	--	--	--	--	--	--
G4ST01163870926	chr1:163870927-163870963	-	GGGGTGGAGGGGGAGGGGAATGGTGAGAAGCCATGGG<br>..(((((.....)))))	MGST3	NM_004528	chr1:163867074-163891479	template	37	Intron_1.f	--	Yes	--	Yes	--	--	--	--	--	--	Yes
G4ST01163899173	chr1:163899174-163899213	+	GGGGAAAATGACCCACTGGCATTTGGGGATAGGGATAGGG<br>..(((((.((....))))))).	ALDH9A1	NM_000696	chr1:163898073-163934524	template	40	Intron_10.l	--	Yes	--	Yes	--	--	--	--	--	--	--
G4ST01164095439	chr1:164095440-164095475	+	GGGAGGCCAGGGTGGGTGGATCACTTTGAGCCCGGG<br>.((.(((...))).)).	UCK2	NM_012474	chr1:164063356-164147479	non-template	36	Intron_1.f	--	Yes	--	Yes	--	--	--	Yes	--	--	--
G4ST01164131954	chr1:164131955-164131987	-	GGGCCAGAACATCTGGCTGGGCTGGGTTCTGGG<br>(((((...)))))..	UCK2	NM_012474	chr1:164063356-164147479	template	33	Intron_3	--	Yes	--	Yes	--	--	--	--	--	--	--
G4ST01164322107	chr1:164322108-164322148	-	GGGGATGGTGGGGGAGGGAGAATCAGGTTAGAGGACCTGGG<br>......(((((....)))))	FAM78B	NM_001017961	chr1:164305880-164402582	non-template	41	Intron_1.u	--	Yes	--	Yes	--	--	--	Yes	Yes	--	--
G4ST01164328191	chr1:164328192-164328223	-	GGGAGGGTTGGGGGTGCAGGCTGCACATAGGG<br>.(((((...)))))...	FAM78B	NM_001017961	chr1:164305880-164402582	non-template	32	Intron_1.u	--	Yes	--	Yes	--	--	--	--	--	--	--
G4ST01164328495	chr1:164328496-164328530	-	GGGTCACAGGGGTCAGGACTGGAGTTCTGGGTGGG<br>...(((((....)))))	FAM78B	NM_001017961	chr1:164305880-164402582	non-template	35	Intron_1.u	--	Yes	--	Yes	--	--	--	--	--	--	--
G4ST01164353627	chr1:164353628-164353662	+	GGGACCTAGGGGCTGGACACAGCAGGGCTTCTGGG<br>((((....)))).	FAM78B	NM_001017961	chr1:164305880-164402582	template	35	Intron_1.u	--	Yes	--	Yes	--	--	--	--	--	--	--
G4ST01164353635	chr1:164353636-164353670	+	GGGGCTGGACACAGCAGGGCTTCTGGGTGTCTGGG<br>((((....)))).	FAM78B	NM_001017961	chr1:164305880-164402582	template	35	Intron_1.u	--	Yes	--	Yes	--	--	--	--	--	--	--
G4ST01164390219	chr1:164390220-164390248	-	GGGAGGGCTTGGCAGCCCAAGGGAAGGGG<br>.((((....))))	FAM78B	NM_001017961	chr1:164305880-164402582	non-template	29	Intron_1.u	--	Yes	--	Yes	--	--	--	--	--	--	--
G4ST01164402443	chr1:164402444-164402471	-	GGGGCGGGGGGCTCCGGCCCGGGGTGGG<br>((((....)))).	FAM78B	NM_001017961	chr1:164305880-164402582	non-template	28	Exon_1.f	--	Yes	Yes	--	--	Yes	--	--	Yes	--	--
G4ST01165075510	chr1:165075511-165075546	+	GGGTCCCGGGCGGCTGGGCCGCGGCAGAGGCGGGGG<br>((((.......))))	POGK	NM_017542	chr1:165075348-165090333	non-template	36	Intron_1.f	--	Yes	--	Yes	--	--	--	--	Yes	Yes	--
G4ST01165080822	chr1:165080823-165080858	+	GGGGAGAATATGGGCCAAGGTGTGGGGGCGTTGGGG<br>(((.....)))	POGK	NM_017542	chr1:165075348-165090333	non-template	36	Intron_2	--	Yes	--	Yes	--	--	--	Yes	Yes	--	--
G4ST01165080833	chr1:165080834-165080873	+	GGGCCAAGGTGTGGGGGCGTTGGGGAATGGTTGTTCAGGG<br>(((.....))) ((((....)))).	POGK	NM_017542	chr1:165075348-165090333	non-template	40	Intron_2	--	Yes	--	Yes	--	--	--	Yes	Yes	--	--
G4ST01165157275	chr1:165157276-165157314	-	GGGTGCTCTTGGGAGCCCTACCTGGAGCTCCAGGGAGGG<br>(((((.....)).)))...	ILDR2	NM_199351	chr1:165149065-165211185	non-template	39	Intron_8	--	Yes	--	Yes	--	--	--	--	--	--	--
G4ST01165192779	chr1:165192780-165192809	+	GGGGGTTGGGAAGGGAGAAACCTTCTGGGG<br>(((.....))).	ILDR2	NM_199351	chr1:165149065-165211185	template	30	Intron_2	--	Yes	--	Yes	--	--	--	--	--	--	--
G4ST01165209595	chr1:165209596-165209628	+	GGGTGGGAGTGGAAGGCACTGGGAGGCCAGGGG<br>((((.....))))	ILDR2	NM_199351	chr1:165149065-165211185	template	33	Intron_1.f	--	Yes	--	Yes	--	--	--	--	Yes	--	--
G4ST01165209599	chr1:165209600-165209635	+	GGGAGTGGAAGGCACTGGGAGGCCAGGGGAGCGGGG<br>((((.....))))	ILDR2	NM_199351	chr1:165149065-165211185	template	36	Intron_1.f	--	Yes	--	Yes	--	--	--	--	Yes	--	--
G4ST01165211213	chr1:165211214-165211251	+	GGGCGGCGGCGGAGCGGCGGGCACCGGGCGTCCCTGGG<br>..((.((...)).))	ILDR2	NM_199351	chr1:165149065-165211185	template	-28	upstream	Yes	--	--	--	--	--	--	--	Yes	Yes	--
G4ST01165325007	chr1:165325008-165325036	-	GGGAAGGGCAGCCTGCTGCTGGGGAAGGG<br>((((.....))))	GPA33	NM_005814	chr1:165288706-165326492	non-template	29	Intron_1.f	--	Yes	--	Yes	--	--	--	--	Yes	--	--
G4ST01165325012	chr1:165325013-165325047	-	GGGGCTCTCGAGGGAAGGGCAGCCTGCTGCTGGGG<br>((((.....))))	GPA33	NM_005814	chr1:165288706-165326492	non-template	35	Intron_1.f	--	Yes	--	Yes	--	--	--	--	Yes	--	--
G4ST01165326380	chr1:165326381-165326417	-	GGGGAACTTGAGGGAAGTAGGGAAGACTCCTCTTGGG<br>((((....))))	GPA33	NM_005814	chr1:165288706-165326492	non-template	37	Exon_1.f	--	Yes	Yes	--	--	Yes	--	Yes	--	--	--
G4ST01165362525	chr1:165362526-165362563	+	GGGAGCAGGGTGCGGGAGGATGATGAGGACAGCGTGGG<br>...(((.((....)).)))	DUSP27	NM_001080426	chr1:165330711-165365026	non-template	38	Exon_5.l	--	Yes	Yes	--	Yes	--	--	--	--	--	--
G4ST01165457439	chr1:165457440-165457478	+	GGGGGGCGGCCCTCAGGGCAACCGGGGGTGCGCGCGGGG<br>(((...)))...	POU2F1	NM_002697	chr1:165456690-165663206	non-template	39	Intron_1.f	--	Yes	--	Yes	--	--	--	Yes	Yes	Yes	--
G4ST01165457439	chr1:165457440-165457478	+	GGGGGGCGGCCCTCAGGGCAACCGGGGGTGCGCGCGGGG<br>(((...)))...	POU2F1	NM_001198786	chr1:165456690-165663206	non-template	39	Intron_1.f	--	Yes	--	Yes	--	--	--	Yes	Yes	Yes	--
G4ST01165457439	chr1:165457440-165457478	+	GGGGGGCGGCCCTCAGGGCAACCGGGGGTGCGCGCGGGG<br>(((...)))...	POU2F1	NR_037163	chr1:165456747-165663206	non-template	39	Intron_1.f	--	Yes	--	Yes	--	--	--	Yes	Yes	Yes	--
G4ST01165667712	chr1:165667713-165667748	-	GGGGGATTATTCCTGGGCCTCTGGAGGCTGGGTGGG<br>(((....))). (((....)))..	CD247	NM_000734	chr1:165666501-165754471	non-template	36	Intron_7.l	--	Yes	--	Yes	--	--	--	--	Yes	--	--
G4ST01165667712	chr1:165667713-165667748	-	GGGGGATTATTCCTGGGCCTCTGGAGGCTGGGTGGG<br>(((....))). (((....)))..	CD247	NM_198053	chr1:165666501-165754471	non-template	36	Intron_7.l	--	Yes	--	Yes	--	--	--	--	Yes	--	--
G4ST01165667716	chr1:165667717-165667755	-	GGGGCCAGGGGGATTATTCCTGGGCCTCTGGAGGCTGGG<br>(((....))). (((....)))..	CD247	NM_000734	chr1:165666501-165754471	non-template	39	Intron_7.l	--	Yes	--	Yes	--	--	--	--	Yes	--	--
G4ST01165667716	chr1:165667717-165667755	-	GGGGCCAGGGGGATTATTCCTGGGCCTCTGGAGGCTGGG<br>(((....))). (((....)))..	CD247	NM_198053	chr1:165666501-165754471	non-template	39	Intron_7.l	--	Yes	--	Yes	--	--	--	--	Yes	--	--
G4ST01165678474	chr1:165678475-165678510	-	GGGGTAGGCAGGGCTGGCCCAGGGGCAGGAAGGGGG<br>(((...)))	CD247	NM_000734	chr1:165666501-165754471	non-template	36	Intron_1.f	--	Yes	--	Yes	--	--	--	--	Yes	--	--
G4ST01165678474	chr1:165678475-165678510	-	GGGGTAGGCAGGGCTGGCCCAGGGGCAGGAAGGGGG<br>(((...)))	CD247	NM_198053	chr1:165666501-165754471	non-template	36	Intron_1.f	--	Yes	--	Yes	--	--	--	--	Yes	--	--
G4ST01165679207	chr1:165679208-165679239	-	GGGGCCAGAGGGCAGGCACACCTGGGGCTGGG<br>((((....))))	CD247	NM_000734	chr1:165666501-165754471	non-template	32	Intron_1.f	--	Yes	--	Yes	--	--	--	--	--	--	--
G4ST01165679207	chr1:165679208-165679239	-	GGGGCCAGAGGGCAGGCACACCTGGGGCTGGG<br>((((....))))	CD247	NM_198053	chr1:165666501-165754471	non-template	32	Intron_1.f	--	Yes	--	Yes	--	--	--	--	--	--	--
G4ST01165690846	chr1:165690847-165690876	-	GGGGAGCTCCGGGAGGAGCTTGGGCTTGGG<br>((...)).	CD247	NM_000734	chr1:165666501-165754471	non-template	30	Intron_1.f	--	Yes	--	Yes	--	--	--	Yes	Yes	Yes	--
G4ST01165690846	chr1:165690847-165690876	-	GGGGAGCTCCGGGAGGAGCTTGGGCTTGGG<br>((...)).	CD247	NM_198053	chr1:165666501-165754471	non-template	30	Intron_1.f	--	Yes	--	Yes	--	--	--	Yes	Yes	Yes	--
G4ST01165690852	chr1:165690853-165690900	-	GGGGCGGAGGCCTGGCCCCGGCTCGGGGAGCTCCGGGAGGAGCTTGGG<br>.(((.(((...)))))))..... ((...)).	CD247	NM_000734	chr1:165666501-165754471	non-template	48	Intron_1.f	--	Yes	--	Yes	--	--	--	Yes	Yes	Yes	--

G4ST01165690852	chr1:165690853-165690900	-	GGGGCGGAGGCCTGGCCCCGGCTCGGGGAGCTCCGGGAGGAGCTTGGG<br>.(((.(((...))))))..... ((...)).	CD247	NM_198053	chr1:165666501-165754471	non-template	48	Intron_1.f	--	Yes	--	Yes	--	--	--	Yes	Yes	Yes	--
G4ST01165690863	chr1:165690864-165690909	-	GGGCACCGAGGGGCGGAGGCCTGGCCCCGGCTCGGGGAGCTCCGGG<br>.(((.(((...))))))....	CD247	NM_000734	chr1:165666501-165754471	non-template	46	Intron_1.f	--	Yes	--	Yes	--	--	--	Yes	Yes	Yes	--
G4ST01165690863	chr1:165690864-165690909	-	GGGCACCGAGGGGCGGAGGCCTGGCCCCGGCTCGGGGAGCTCCGGG<br>.(((.(((...))))))....	CD247	NM_198053	chr1:165666501-165754471	non-template	46	Intron_1.f	--	Yes	--	Yes	--	--	--	Yes	Yes	Yes	--
G4ST01165691212	chr1:165691213-165691253	-	GGGACGGCGGTCCCGGCCGCCTGGGCTTTGGGCGTAGTGGG<br>..((((((....)))))).	CD247	NM_000734	chr1:165666501-165754471	non-template	41	Intron_1.f	--	Yes	--	Yes	--	--	--	Yes	Yes	Yes	--
G4ST01165691212	chr1:165691213-165691253	-	GGGACGGCGGTCCCGGCCGCCTGGGCTTTGGGCGTAGTGGG<br>..((((((....)))))).	CD247	NM_198053	chr1:165666501-165754471	non-template	41	Intron_1.f	--	Yes	--	Yes	--	--	--	Yes	Yes	Yes	--
G4ST01165691528	chr1:165691529-165691557	-	GGGGACAAGGGACCGCAAGTGGGGGCGGG<br>.(((....)))	CD247	NM_000734	chr1:165666501-165754471	non-template	29	Intron_1.f	--	Yes	--	Yes	--	--	--	Yes	Yes	Yes	--
G4ST01165691528	chr1:165691529-165691557	-	GGGGACAAGGGACCGCAAGTGGGGGCGGG<br>.(((....)))	CD247	NM_198053	chr1:165666501-165754471	non-template	29	Intron_1.f	--	Yes	--	Yes	--	--	--	Yes	Yes	Yes	--
G4ST01165691596	chr1:165691597-165691638	+	GGGCGTGGATCTGCGCCGGGCTCCGGCGCTGGGGGCCGAGGG<br>((((....)))).. ..(((....)))	CD247	NM_000734	chr1:165666501-165754471	template	42	Intron_1.f	--	Yes	--	Yes	--	--	--	Yes	Yes	Yes	--
G4ST01165691596	chr1:165691597-165691638	+	GGGCGTGGATCTGCGCCGGGCTCCGGCGCTGGGGGCCGAGGG<br>((((....)))).. ..(((....)))	CD247	NM_198053	chr1:165666501-165754471	template	42	Intron_1.f	--	Yes	--	Yes	--	--	--	Yes	Yes	Yes	--
G4ST01165691843	chr1:165691844-165691878	-	GGGGGCGGGGGGTGGGAAGGCCCAGCCTCTCAGGG<br>.((((...))))....	CD247	NM_000734	chr1:165666501-165754471	non-template	35	Intron_1.f	--	Yes	--	Yes	--	--	--	Yes	Yes	Yes	Yes
G4ST01165691843	chr1:165691844-165691878	-	GGGGGCGGGGGGTGGGAAGGCCCAGCCTCTCAGGG<br>.((((...))))....	CD247	NM_198053	chr1:165666501-165754471	non-template	35	Intron_1.f	--	Yes	--	Yes	--	--	--	Yes	Yes	Yes	Yes
G4ST01165724806	chr1:165724807-165724836	+	GGGCCGGGCCTCTTCTGAGAGGGCTGAGGG<br>.(((....))).	CD247	NM_000734	chr1:165666501-165754471	template	30	Intron_1.f	--	Yes	--	Yes	--	--	--	--	--	--	--
G4ST01165724806	chr1:165724807-165724836	+	GGGCCGGGCCTCTTCTGAGAGGGCTGAGGG<br>.(((....))).	CD247	NM_198053	chr1:165666501-165754471	template	30	Intron_1.f	--	Yes	--	Yes	--	--	--	--	--	--	--
G4ST01165745620	chr1:165745621-165745663	+	GGGTCTGGGAGATCCCCATACATCTGGGCTTAGAATTAAGGGG<br>((((........)))) ((((....))))	CD247	NM_000734	chr1:165666501-165754471	template	43	Intron_1.f	--	Yes	--	Yes	--	--	--	--	--	--	--
G4ST01165745620	chr1:165745621-165745663	+	GGGTCTGGGAGATCCCCATACATCTGGGCTTAGAATTAAGGGG<br>((((........)))) ((((....))))	CD247	NM_198053	chr1:165666501-165754471	template	43	Intron_1.f	--	Yes	--	Yes	--	--	--	--	--	--	--
G4ST01165745626	chr1:165745627-165745672	+	GGGAGATCCCCATACATCTGGGCTTAGAATTAAGGGGTGAACAGGG<br>((((........)))) ((((....))))	CD247	NM_000734	chr1:165666501-165754471	template	46	Intron_1.f	--	Yes	--	Yes	--	--	--	--	--	--	--
G4ST01165745626	chr1:165745627-165745672	+	GGGAGATCCCCATACATCTGGGCTTAGAATTAAGGGGTGAACAGGG<br>((((........)))) ((((....))))	CD247	NM_198053	chr1:165666501-165754471	template	46	Intron_1.f	--	Yes	--	Yes	--	--	--	--	--	--	--
G4ST01165871019	chr1:165871020-165871053	-	GGGTGGGTGGGTCAAAAAACAGCGAGGCTGTGGG<br>.......(((((...)))))	RCSD1	NM_052862	chr1:165866098-165942110	template	34	Intron_1.f	--	Yes	--	Yes	--	--	--	Yes	Yes	Yes	--
G4ST01165960036	chr1:165960037-165960069	+	GGGAGTTTAAACTGGGTAGGCATGGGGGAGGGG<br>(((....)))	MPZL1	NM_001146191	chr1:165957811-166027780	non-template	33	Intron_1.f	--	Yes	--	Yes	--	--	--	Yes	Yes	--	--
G4ST01165960036	chr1:165960037-165960069	+	GGGAGTTTAAACTGGGTAGGCATGGGGGAGGGG<br>(((....)))	MPZL1	NM_003953	chr1:165957811-166027780	non-template	33	Intron_1.f	--	Yes	--	Yes	--	--	--	Yes	Yes	--	--
G4ST01165960036	chr1:165960037-165960069	+	GGGAGTTTAAACTGGGTAGGCATGGGGGAGGGG<br>(((....)))	MPZL1	NM_024569	chr1:165957811-166027780	non-template	33	Intron_1.f	--	Yes	--	Yes	--	--	--	Yes	Yes	--	--
G4ST01166009227	chr1:166009228-166009266	+	GGGTAAGAAACACTGTTTTTTTAGGGCAGGGGTGGAGGG<br>.(((((((...)))))))..	MPZL1	NM_001146191	chr1:165957811-166027780	non-template	39	Intron_2.l	--	Yes	--	Yes	--	--	--	--	--	--	Yes
G4ST01166009227	chr1:166009228-166009266	+	GGGTAAGAAACACTGTTTTTTTAGGGCAGGGGTGGAGGG<br>.(((((((...)))))))..	MPZL1	NM_003953	chr1:165957811-166027780	non-template	39	Exon_4-Intron_4	--	Yes	Yes	Yes	Yes	--	--	--	--	--	Yes
G4ST01166009227	chr1:166009228-166009266	+	GGGTAAGAAACACTGTTTTTTTAGGGCAGGGGTGGAGGG<br>.(((((((...)))))))..	MPZL1	NM_024569	chr1:165957811-166027780	non-template	39	Exon_4-Intron_4.l	--	Yes	Yes	Yes	Yes	--	--	--	--	--	Yes
G4ST01166172715	chr1:166172716-166172750	-	GGGGAGACCACCGCGTGGGGGAGGGGAGGAGGGGG<br>....((((...))))	BRP44	NM_015415	chr1:166153934-166171857	non-template	-858	upstream	Yes	--	--	--	--	--	--	--	Yes	Yes	Yes
G4ST01166172715	chr1:166172716-166172750	-	GGGGAGACCACCGCGTGGGGGAGGGGAGGAGGGGG<br>....((((...))))	BRP44	NR_026550	chr1:166153934-166172004	non-template	-711	upstream	Yes	--	--	--	--	--	--	--	Yes	Yes	Yes
G4ST01166172715	chr1:166172716-166172750	-	GGGGAGACCACCGCGTGGGGGAGGGGAGGAGGGGG<br>....((((...))))	BRP44	NM_001143674	chr1:166153934-166172902	non-template	35	Intron_1.f	--	Yes	--	Yes	--	--	--	--	Yes	Yes	Yes
G4ST01166172715	chr1:166172716-166172750	-	GGGGAGACCACCGCGTGGGGGAGGGGAGGAGGGGG<br>....((((...))))	DCAF6	NM_001198956	chr1:166172421-166311705	template	35	Exon_1.f	--	Yes	Yes	--	--	Yes	--	--	Yes	Yes	Yes
G4ST01166172715	chr1:166172716-166172750	-	GGGGAGACCACCGCGTGGGGGAGGGGAGGAGGGGG<br>....((((...))))	DCAF6	NM_001198957	chr1:166172421-166311705	template	35	Exon_1.f	--	Yes	Yes	--	--	Yes	--	--	Yes	Yes	Yes
G4ST01166172715	chr1:166172716-166172750	-	GGGGAGACCACCGCGTGGGGGAGGGGAGGAGGGGG<br>....((((...))))	DCAF6	NM_018442	chr1:166172421-166311705	template	35	Exon_1.f	--	Yes	Yes	--	--	Yes	--	--	Yes	Yes	Yes
G4ST01166172715	chr1:166172716-166172750	-	GGGGAGACCACCGCGTGGGGGAGGGGAGGAGGGGG<br>....((((...))))	DCAF6	NM_001017977	chr1:166172421-166311705	template	35	Exon_1.f	--	Yes	Yes	--	--	Yes	--	--	Yes	Yes	Yes
G4ST01166172724	chr1:166172725-166172754	-	GGGAGGGGAGACCACCGCGTGGGGGAGGGG<br>....((((...))))	BRP44	NM_015415	chr1:166153934-166171857	non-template	-867	upstream	Yes	--	--	--	--	--	--	--	Yes	Yes	Yes
G4ST01166172724	chr1:166172725-166172754	-	GGGAGGGGAGACCACCGCGTGGGGGAGGGG<br>....((((...))))	BRP44	NR_026550	chr1:166153934-166172004	non-template	-720	upstream	Yes	--	--	--	--	--	--	--	Yes	Yes	Yes
G4ST01166172724	chr1:166172725-166172754	-	GGGAGGGGAGACCACCGCGTGGGGGAGGGG<br>....((((...))))	BRP44	NM_001143674	chr1:166153934-166172902	non-template	30	Intron_1.f	--	Yes	--	Yes	--	--	--	--	Yes	Yes	Yes
G4ST01166172724	chr1:166172725-166172754	-	GGGAGGGGAGACCACCGCGTGGGGGAGGGG<br>....((((...))))	DCAF6	NM_001198956	chr1:166172421-166311705	template	30	Exon_1.f	--	Yes	Yes	--	--	Yes	--	--	Yes	Yes	Yes
G4ST01166172724	chr1:166172725-166172754	-	GGGAGGGGAGACCACCGCGTGGGGGAGGGG<br>....((((...))))	DCAF6	NM_001198957	chr1:166172421-166311705	template	30	Exon_1.f	--	Yes	Yes	--	--	Yes	--	--	Yes	Yes	Yes
G4ST01166172724	chr1:166172725-166172754	-	GGGAGGGGAGACCACCGCGTGGGGGAGGGG<br>....((((...))))	DCAF6	NM_018442	chr1:166172421-166311705	template	30	Exon_1.f	--	Yes	Yes	--	--	Yes	--	--	Yes	Yes	Yes
G4ST01166172724	chr1:166172725-166172754	-	GGGAGGGGAGACCACCGCGTGGGGGAGGGG<br>....((((...))))	DCAF6	NM_001017977	chr1:166172421-166311705	template	30	Exon_1.f	--	Yes	Yes	--	--	Yes	--	--	Yes	Yes	Yes
G4ST01166172729	chr1:166172730-166172758	-	GGGTGGGAGGGGAGACCACCGCGTGGGGG<br>....((((...))))	BRP44	NM_015415	chr1:166153934-166171857	non-template	-872	upstream	Yes	--	--	--	--	--	--	--	Yes	Yes	Yes
G4ST01166172729	chr1:166172730-166172758	-	GGGTGGGAGGGGAGACCACCGCGTGGGGG<br>....((((...))))	BRP44	NR_026550	chr1:166153934-166172004	non-template	-725	upstream	Yes	--	--	--	--	--	--	--	Yes	Yes	Yes
G4ST01166172729	chr1:166172730-166172758	-	GGGTGGGAGGGGAGACCACCGCGTGGGGG<br>....((((...))))	BRP44	NM_001143674	chr1:166153934-166172902	non-template	29	Intron_1.f	--	Yes	--	Yes	--	--	--	--	Yes	Yes	Yes
G4ST01166172729	chr1:166172730-166172758	-	GGGTGGGAGGGGAGACCACCGCGTGGGGG<br>....((((...))))	DCAF6	NM_001198956	chr1:166172421-166311705	template	29	Exon_1.f	--	Yes	Yes	--	--	Yes	--	--	Yes	Yes	Yes
G4ST01166172729	chr1:166172730-166172758	-	GGGTGGGAGGGGAGACCACCGCGTGGGGG<br>....((((...))))	DCAF6	NM_001198957	chr1:166172421-166311705	template	29	Exon_1.f	--	Yes	Yes	--	--	Yes	--	--	Yes	Yes	Yes
G4ST01166172729	chr1:166172730-166172758	-	GGGTGGGAGGGGAGACCACCGCGTGGGGG<br>....((((...))))	DCAF6	NM_018442	chr1:166172421-166311705	template	29	Exon_1.f	--	Yes	Yes	--	--	Yes	--	--	Yes	Yes	Yes
G4ST01166172729	chr1:166172730-166172758	-	GGGTGGGAGGGGAGACCACCGCGTGGGGG<br>....((((...))))	DCAF6	NM_001017977	chr1:166172421-166311705	template	29	Exon_1.f	--	Yes	Yes	--	--	Yes	--	--	Yes	Yes	Yes
G4ST01166266811	chr1:166266812-166266842	+	GGGATGAAGCATGGGCGGGGACGTGTAGGGG<br>(((...)))	DCAF6	NM_001198956	chr1:166172421-166311705	non-template	31	Intron_12	--	Yes	--	Yes	--	--	--	--	--	--	--
G4ST01166266811	chr1:166266812-166266842	+	GGGATGAAGCATGGGCGGGGACGTGTAGGGG<br>(((...)))	DCAF6	NM_001198957	chr1:166172421-166311705	non-template	31	Intron_11	--	Yes	--	Yes	--	--	--	--	--	--	--
G4ST01166266811	chr1:166266812-166266842	+	GGGATGAAGCATGGGCGGGGACGTGTAGGGG<br>(((...)))	DCAF6	NM_018442	chr1:166172421-166311705	non-template	31	Intron_11	--	Yes	--	Yes	--	--	--	--	--	--	--
G4ST01166266811	chr1:166266812-166266842	+	GGGATGAAGCATGGGCGGGGACGTGTAGGGG<br>(((...)))	DCAF6	NM_001017977	chr1:166172421-166311705	non-template	31	Intron_10	--	Yes	--	Yes	--	--	--	--	--	--	--
G4ST01166372841	chr1:166372842-166372876	-	GGGGAGGCGGCTCGGGGCTCCGGGGGCCGGAGGGG<br>(((....))).	GPR161	NM_153832	chr1:166320621-166372248	non-template	-593	upstream	Yes	--	--	--	--	--	--	Yes	Yes	Yes	Yes

G4ST01166372850	chr1:166372851-166372883	-	GGGGCGCGGGGAGGCGGCTCGGGGCTCCGGGGG<br>(((....))).	GPR161	NM_153832	chr1:166320621-166372248	non-template	-602	upstream	Yes	--	--	--	--	--	--	Yes	Yes	Yes	Yes
G4ST01166372859	chr1:166372860-166372893	-	GGGGCAGTGCGGGGCGCGGGGAGGCGGCTCGGGG<br>(((....))).	GPR161	NM_153832	chr1:166320621-166372248	non-template	-611	upstream	Yes	--	--	--	--	--	--	Yes	Yes	Yes	Yes
G4ST01166461815	chr1:166461816-166461853	+	GGGCGGGGCGCGCCGCGGTTGGCGCCTGGGCGGCTGGG<br>..((((((....))))))..	SFT2D2	NM_199344	chr1:166461879-166478712	non-template	-25	upstream	Yes	--	--	--	--	--	--	Yes	Yes	Yes	--
G4ST01166611302	chr1:166611303-166611333	+	GGGGGCTTTGGGAGATTCTTGGGAGCCAGGG<br>((...)).	MIR557	NR_030284	chr1:166611386-166611483	non-template	-52	upstream	Yes	--	--	--	--	--	--	--	--	--	--
G4ST01166965950	chr1:166965951-166965990	-	GGGTGTTTGGGAAGATTTGACTGCAAAGGGATATAAGGGG<br>....((((....))))	DPT	NM_001937	chr1:166931319-166965066	non-template	-884	upstream	Yes	--	--	--	--	--	--	--	--	--	--
G4ST01167342058	chr1:167342059-167342088	-	GGGACTCGGCCCGAGAGGGCGGGTTCGGGG<br>.((((...)))).	ATP1B1	NM_001677	chr1:167342571-167368584	template	-482	upstream	Yes	--	--	--	--	--	--	Yes	Yes	Yes	--
G4ST01167342065	chr1:167342066-167342094	-	GGGGCAGGGACTCGGCCCGAGAGGGCGGG<br>.((((...)))).	ATP1B1	NM_001677	chr1:167342571-167368584	template	-476	upstream	Yes	--	--	--	--	--	--	Yes	Yes	Yes	--
G4ST01167441935	chr1:167441936-167441965	-	GGGAAATAGGGTTTTCCAGAAAAGGGAGGG<br>((((....))))	NME7	NM_013330	chr1:167368393-167603810	non-template	30	Intron_10	--	Yes	--	Yes	--	--	--	--	--	--	--
G4ST01167441935	chr1:167441936-167441965	-	GGGAAATAGGGTTTTCCAGAAAAGGGAGGG<br>((((....))))	NME7	NM_197972	chr1:167368397-167603810	non-template	30	Intron_10	--	Yes	--	Yes	--	--	--	--	--	--	--
G4ST01167441939	chr1:167441940-167441970	-	GGGGAGGGAAATAGGGTTTTCCAGAAAAGGG<br>((((....))))	NME7	NM_013330	chr1:167368393-167603810	non-template	31	Intron_10	--	Yes	--	Yes	--	--	--	--	--	--	--
G4ST01167441939	chr1:167441940-167441970	-	GGGGAGGGAAATAGGGTTTTCCAGAAAAGGG<br>((((....))))	NME7	NM_197972	chr1:167368397-167603810	non-template	31	Intron_10	--	Yes	--	Yes	--	--	--	--	--	--	--
G4ST01167538461	chr1:167538462-167538492	+	GGGGACTACTAGAGTGGGAGAAAGGGAAGGG<br>.(((.....)))	NME7	NM_013330	chr1:167368393-167603810	template	31	Intron_5	--	Yes	--	Yes	--	--	--	--	--	--	Yes
G4ST01167538461	chr1:167538462-167538492	+	GGGGACTACTAGAGTGGGAGAAAGGGAAGGG<br>.(((.....)))	NME7	NM_197972	chr1:167368397-167603810	template	31	Intron_5	--	Yes	--	Yes	--	--	--	--	--	--	Yes
G4ST01167653363	chr1:167653364-167653391	-	GGGTATGGGAAATGTGGTGTTGGGGGGG<br>.(((.....)))	C1orf114	NM_021179	chr1:167630738-167663294	non-template	28	Intron_4	--	Yes	--	Yes	--	--	--	--	--	--	--
G4ST01167845768	chr1:167845769-167845796	-	GGGAAGGGAGGGCTTCCTAAGGAAAGGG<br>.((((...)))).	SELP	NM_003005	chr1:167824712-167866001	non-template	28	Intron_7	--	Yes	--	Yes	--	--	--	--	--	--	--
G4ST01168243126	chr1:168243127-168243166	+	GGGAGAGAGGGCATATTAATATATATGAAAGGGAAGAGGG<br>(((((......)))))...	KIFAP3	NM_014970	chr1:168157094-168310503	template	40	Intron_10	--	Yes	--	Yes	--	--	--	--	--	--	--
G4ST01168243126	chr1:168243127-168243166	+	GGGAGAGAGGGCATATTAATATATATGAAAGGGAAGAGGG<br>(((((......)))))...	KIFAP3	NM_001204517	chr1:168157094-168304680	template	40	Intron_10	--	Yes	--	Yes	--	--	--	--	--	--	--
G4ST01168243126	chr1:168243127-168243166	+	GGGAGAGAGGGCATATTAATATATATGAAAGGGAAGAGGG<br>(((((......)))))...	KIFAP3	NM_001204514	chr1:168157094-168310503	template	40	Intron_11	--	Yes	--	Yes	--	--	--	--	--	--	--
G4ST01168243126	chr1:168243127-168243166	+	GGGAGAGAGGGCATATTAATATATATGAAAGGGAAGAGGG<br>(((((......)))))...	KIFAP3	NM_001204516	chr1:168157094-168310503	template	40	Intron_9	--	Yes	--	Yes	--	--	--	--	--	--	--
G4ST01168900247	chr1:168900248-168900277	+	GGGGGTGTGTGCCCGGGACAGAGGGCGGGG<br>(((....))).	PRRX1	NM_022716	chr1:168899937-168975165	non-template	30	Intron_1.f	--	Yes	--	Yes	--	--	--	Yes	--	--	--
G4ST01168900247	chr1:168900248-168900277	+	GGGGGTGTGTGCCCGGGACAGAGGGCGGGG<br>(((....))).	PRRX1	NM_006902	chr1:168899937-168975165	non-template	30	Intron_1.f	--	Yes	--	Yes	--	--	--	Yes	--	--	--
G4ST01169347796	chr1:169347797-169347828	+	GGGAATGAGCCATGAGGGTCTCAGGGGAAGGG<br>.(((...)))..	FMO3	NM_006894	chr1:169326642-169353583	non-template	32	Intron_6	--	Yes	--	Yes	--	--	--	--	--	--	--
G4ST01169347796	chr1:169347797-169347828	+	GGGAATGAGCCATGAGGGTCTCAGGGGAAGGG<br>.(((...)))..	FMO3	NM_001002294	chr1:169326642-169353583	non-template	32	Intron_6	--	Yes	--	Yes	--	--	--	--	--	--	--
G4ST01169874226	chr1:169874227-169874262	-	GGGGGAAATTAACTTCCCTGGGAGCAGAGGGAGGGG<br>((((......))))..	MYOC	NM_000261	chr1:169871180-169888396	non-template	36	Intron_2.l	--	Yes	--	Yes	--	--	--	--	--	--	--
G4ST01170020294	chr1:170020295-170020324	+	GGGGCTGGCTTACAGGGGCTGGGGCTTGGG<br>.(((.....)))	METTL13	NM_014955	chr1:170017384-170033479	non-template	30	Intron_2	--	Yes	--	Yes	--	--	--	--	--	--	Yes
G4ST01170020294	chr1:170020295-170020324	+	GGGGCTGGCTTACAGGGGCTGGGGCTTGGG<br>.(((.....)))	METTL13	NM_015935	chr1:170017384-170033479	non-template	30	Intron_2	--	Yes	--	Yes	--	--	--	--	--	--	Yes
G4ST01170020294	chr1:170020295-170020324	+	GGGGCTGGCTTACAGGGGCTGGGGCTTGGG<br>.(((.....)))	METTL13	NM_001007239	chr1:170017384-170033479	non-template	30	Intron_2	--	Yes	--	Yes	--	--	--	--	--	--	Yes
G4ST01171431581	chr1:171431582-171431612	+	GGGCAGCAGTGGGACGTGGGCTAGTAGAGGG<br>((...)).	TNFSF4	NM_003326	chr1:171419493-171443094	template	31	Intron_1.f	--	Yes	--	Yes	--	--	--	Yes	Yes	--	--
G4ST01171614120	chr1:171614121-171614149	+	GGGTCCAGGGACCGTTGTGGGTCCTTGGG<br>((....))	LOC100506023	NR_037845	chr1:171470822-171712917	template	29	Intron_2.l	--	Yes	--	Yes	--	--	--	--	--	--	--
G4ST01171614127	chr1:171614128-171614156	+	GGGACCGTTGTGGGTCCTTGGGCAGGGGG<br>((....))	LOC100506023	NR_037845	chr1:171470822-171712917	template	29	Intron_2.l	--	Yes	--	Yes	--	--	--	--	--	--	--
G4ST01171631679	chr1:171631680-171631729	-	GGGTTAATCCCTTTGATTGGGGTGGAGAGGCCACTCCCACTGGGGGTGGG<br>..((((.....)))). ((((.(((....)))))))...	LOC100506023	NR_037845	chr1:171470822-171712917	non-template	50	Intron_2.l	--	Yes	--	Yes	--	--	--	Yes	--	--	--
G4ST01171652256	chr1:171652257-171652285	-	GGGAGACCCTTGGGCCCTGTTGGGGAGGG<br>((...)).	LOC100506023	NR_037845	chr1:171470822-171712917	non-template	29	Intron_2.l	--	Yes	--	Yes	--	--	--	--	--	Yes	--
G4ST01171652260	chr1:171652261-171652290	-	GGGCTGGGAGACCCTTGGGCCCTGTTGGGG<br>((...)).	LOC100506023	NR_037845	chr1:171470822-171712917	non-template	30	Intron_2.l	--	Yes	--	Yes	--	--	--	--	--	Yes	--
G4ST01171713339	chr1:171713340-171713379	-	GGGGGAAGGCGAGGGCGGCGCAGGGGGCTGAGCGGCCGGG<br>((((....))))	PRDX6	NM_004905	chr1:171713109-171724569	template	40	Intron_1.f	--	Yes	--	Yes	--	--	--	Yes	Yes	Yes	--
G4ST01171713339	chr1:171713340-171713379	-	GGGGGAAGGCGAGGGCGGCGCAGGGGGCTGAGCGGCCGGG<br>((((....))))	LOC100506023	NR_037845	chr1:171470822-171712917	non-template	-422	upstream	Yes	--	--	--	--	--	--	Yes	Yes	Yes	--
G4ST01171713698	chr1:171713699-171713724	-	GGGTGGGAGGGCGGTGCGACCCTGGG<br>.(((...)))..	PRDX6	NM_004905	chr1:171713109-171724569	template	26	Intron_1.f	--	Yes	--	Yes	--	--	--	Yes	Yes	Yes	--
G4ST01171713698	chr1:171713699-171713724	-	GGGTGGGAGGGCGGTGCGACCCTGGG<br>.(((...)))..	LOC100506023	NR_037845	chr1:171470822-171712917	non-template	-781	upstream	Yes	--	--	--	--	--	--	Yes	Yes	Yes	--
G4ST01172104456	chr1:172104457-172104500	-	GGGGGCGGCGCCCAGGGACCGGCTCCAGGCGGCGGCGGGCGGGG<br>(((...))).. .(((((.....))..))).	GAS5	NR_002578	chr1:172099662-172103748	non-template	-708	upstream	Yes	--	--	--	--	--	--	Yes	Yes	Yes	--
G4ST01172104456	chr1:172104457-172104500	-	GGGGGCGGCGCCCAGGGACCGGCTCCAGGCGGCGGCGGGCGGGG<br>(((...))).. .(((((.....))..))).	ZBTB37	NM_032522	chr1:172104116-172109401	template	44	Intron_1.f	--	Yes	--	Yes	--	--	--	Yes	Yes	Yes	--
G4ST01172104456	chr1:172104457-172104500	-	GGGGGCGGCGCCCAGGGACCGGCTCCAGGCGGCGGCGGGCGGGG<br>(((...))).. .(((((.....))..))).	SNORD74	NR_002579	chr1:172103435-172103506	non-template	-950	upstream	Yes	--	--	--	--	--	--	Yes	Yes	Yes	--
G4ST01172104456	chr1:172104457-172104500	-	GGGGGCGGCGCCCAGGGACCGGCTCCAGGCGGCGGCGGGCGGGG<br>(((...))).. .(((((.....))..))).	SNORD76	NR_003942	chr1:172102396-172102476	non-template	-1980	upstream	Yes	--	--	--	--	--	--	Yes	Yes	Yes	--
G4ST01172104456	chr1:172104457-172104500	-	GGGGGCGGCGCCCAGGGACCGGCTCCAGGCGGCGGCGGGCGGGG<br>(((...))).. .(((((.....))..))).	SNORD75	NR_003941	chr1:172102640-172102699	non-template	-1757	upstream	Yes	--	--	--	--	--	--	Yes	Yes	Yes	--
G4ST01172104456	chr1:172104457-172104500	-	GGGGGCGGCGCCCAGGGACCGGCTCCAGGCGGCGGCGGGCGGGG<br>(((...))).. .(((((.....))..))).	ZBTB37	NM_001122770	chr1:172104116-172122397	template	44	Intron_1.f	--	Yes	--	Yes	--	--	--	Yes	Yes	Yes	--
G4ST01172553700	chr1:172553701-172553736	-	GGGCACAGGGGGAGGGAGAACACAGCAACTGTGGGG<br>....(((((...)))))	RABGAP1L	NM_014857	chr1:172395257-173193950	template	36	Intron_11	--	Yes	--	Yes	--	--	--	--	--	--	--
G4ST01172641631	chr1:172641632-172641666	+	GGGAGGCTGGGGTGGGAGGATCACTTGAGCCCGGG<br>.((.((....)).)).	RABGAP1L	NM_014857	chr1:172395257-173193950	non-template	35	Intron_13	--	Yes	--	Yes	--	--	--	--	--	--	--
G4ST01172872981	chr1:172872982-172873013	+	GGGTATAGGACTATGGGTCTGGGATAGAAGGG<br>.(((....)))	RABGAP1L	NM_014857	chr1:172395257-173193950	non-template	32	Intron_13	--	Yes	--	Yes	--	--	--	--	--	--	--
G4ST01173235776	chr1:173235777-173235814	+	GGGAGGCGGGCCGCGATCTTGCGCAGGGTCGGTGTGGG<br>.((((....))))..	CACYBP	NM_014412	chr1:173235515-173247786	non-template	38	Exon_1.f	--	Yes	Yes	--	--	Yes	--	Yes	Yes	Yes	--
G4ST01173235776	chr1:173235777-173235814	+	GGGAGGCGGGCCGCGATCTTGCGCAGGGTCGGTGTGGG<br>.((((....))))..	CACYBP	NM_001007214	chr1:173235194-173247786	non-template	38	Intron_1.f	--	Yes	--	Yes	--	--	--	Yes	Yes	Yes	--
G4ST01173311904	chr1:173311905-173311939	+	GGGCCGTCCTAGATGGGGGTTGGGGGCACAAGGGG<br>(((((...)))))	TNN	NM_022093	chr1:173303617-173383825	non-template	35	Intron_1.f	--	Yes	--	Yes	--	--	--	Yes	--	--	--
G4ST01173382401	chr1:173382402-173382433	-	GGGAGCAAGGGGGTGTGAGCCCGGGAGCTGGG<br>(((....))).	TNN	NM_022093	chr1:173303617-173383825	template	32	Intron_18.l	--	Yes	--	Yes	--	--	--	Yes	Yes	--	--

G4ST01173428852	chr1:173428853-173428882	-	GGGGAGCCACCGGCGGGAAGACGGGGAGGG ..(((...)))	KIAA0040	NM_001162895	chr1:173392746-173428572	non-template	-280	upstream	Yes	--	--	--	--	--	--	Yes	Yes	Yes	Yes
G4ST01173428852	chr1:173428853-173428882	-	GGGGAGCCACCGGCGGGAAGACGGGGAGGG ..(((...)))	KIAA0040	NM_014656	chr1:173392746-173428572	non-template	-280	upstream	Yes	--	--	--	--	--	--	Yes	Yes	Yes	Yes
G4ST01173428852	chr1:173428853-173428882	-	GGGGAGCCACCGGCGGGAAGACGGGGAGGG ..(((...)))	KIAA0040	NM_001162893	chr1:173392746-173428852	non-template	0	upstream	Yes	--	--	--	--	--	--	Yes	Yes	Yes	Yes
G4ST01173428852	chr1:173428853-173428882	-	GGGGAGCCACCGGCGGGAAGACGGGGAGGG ..(((...)))	KIAA0040	NM_001162894	chr1:173392746-173428852	non-template	0	upstream	Yes	--	--	--	--	--	--	Yes	Yes	Yes	Yes
G4ST01173428856	chr1:173428857-173428889	-	GGGGAGTGGGGAGCCACCGGCGGGAAGACGGGG ..(((...)))	KIAA0040	NM_001162895	chr1:173392746-173428572	non-template	-284	upstream	Yes	--	--	--	--	--	--	Yes	Yes	Yes	Yes
G4ST01173428856	chr1:173428857-173428889	-	GGGGAGTGGGGAGCCACCGGCGGGAAGACGGGG ..(((...)))	KIAA0040	NM_014656	chr1:173392746-173428572	non-template	-284	upstream	Yes	--	--	--	--	--	--	Yes	Yes	Yes	Yes
G4ST01173428856	chr1:173428857-173428889	-	GGGGAGTGGGGAGCCACCGGCGGGAAGACGGGG ..(((...)))	KIAA0040	NM_001162893	chr1:173392746-173428852	non-template	-4	upstream	Yes	--	--	--	--	--	--	Yes	Yes	Yes	Yes
G4ST01173428856	chr1:173428857-173428889	-	GGGGAGTGGGGAGCCACCGGCGGGAAGACGGGG ..(((...)))	KIAA0040	NM_001162894	chr1:173392746-173428852	non-template	-4	upstream	Yes	--	--	--	--	--	--	Yes	Yes	Yes	Yes
G4ST01173428865	chr1:173428866-173428896	-	GGGGGGCGGGGAGTGGGGAGCCACCGGCGGG ..(((...)))	KIAA0040	NM_001162895	chr1:173392746-173428572	non-template	-293	upstream	Yes	--	--	--	--	--	--	Yes	Yes	Yes	Yes
G4ST01173428865	chr1:173428866-173428896	-	GGGGGGCGGGGAGTGGGGAGCCACCGGCGGG ..(((...)))	KIAA0040	NM_014656	chr1:173392746-173428572	non-template	-293	upstream	Yes	--	--	--	--	--	--	Yes	Yes	Yes	Yes
G4ST01173428865	chr1:173428866-173428896	-	GGGGGGCGGGGAGTGGGGAGCCACCGGCGGG ..(((...)))	KIAA0040	NM_001162893	chr1:173392746-173428852	non-template	-13	upstream	Yes	--	--	--	--	--	--	Yes	Yes	Yes	Yes
G4ST01173428865	chr1:173428866-173428896	-	GGGGGGCGGGGAGTGGGGAGCCACCGGCGGG ..(((...)))	KIAA0040	NM_001162894	chr1:173392746-173428852	non-template	-13	upstream	Yes	--	--	--	--	--	--	Yes	Yes	Yes	Yes
G4ST01173598679	chr1:173598680-173598711	+	GGGGAGGGATGATACATGGTTCATGGGCTGGG ((((........))))	TNR	NM_003285	chr1:173558558-173979375	template	32	Intron_13	--	Yes	--	Yes	--	--	--	--	--	--	--
G4ST01173650752	chr1:173650753-173650787	+	GGGCAGGGAAGGAGAAGTGACCTCCTGGGGTTGGG .(((((.......))))).	TNR	NM_003285	chr1:173558558-173979375	template	35	Intron_2	--	Yes	--	Yes	--	--	--	--	--	--	--
G4ST01173813985	chr1:173813986-173814030	+	GGGGGAAACTGGATGCATTCAGGGGAGATCTGGGGACAGAGAGGG .....(((((....)))))	TNR	NM_003285	chr1:173558558-173979375	template	45	Intron_1.f	--	Yes	--	Yes	--	--	--	Yes	--	--	Yes
G4ST01173887098	chr1:173887099-173887129	+	GGGGAAAGGTTACCTTGAGGGGAGGGAAGGG ..((((...))))...	TNR	NM_003285	chr1:173558558-173979375	template	31	Intron_1.f	--	Yes	--	Yes	--	--	--	--	--	--	--
G4ST01173911589	chr1:173911590-173911618	+	GGGGAGGGGGCTTCTGGGAGGACGCTGGG ((....))	TNR	NM_003285	chr1:173558558-173979375	template	29	Intron_1.f	--	Yes	--	Yes	--	--	--	--	--	--	Yes
G4ST01173950210	chr1:173950211-173950241	-	GGGGCGCAGTGCCAGCTGGGGTGAGGGAGGG ...((((....))))	TNR	NM_003285	chr1:173558558-173979375	non-template	31	Intron_1.f	--	Yes	--	Yes	--	--	--	--	--	--	--
G4ST01173953987	chr1:173953988-173954024	-	GGGGGTGCTGGGGAAGGACTGGGGCAGAGTTCTGGGG .(((....)))	TNR	NM_003285	chr1:173558558-173979375	non-template	37	Intron_1.f	--	Yes	--	Yes	--	--	--	--	--	--	--
G4ST01173955001	chr1:173955002-173955033	+	GGGCTGCACACAGGGGTGTCGGGATATTGGGG (((....)))	TNR	NM_003285	chr1:173558558-173979375	template	32	Intron_1.f	--	Yes	--	Yes	--	--	--	--	--	--	--
G4ST01174784067	chr1:174784068-174784104	+	GGGGGAGCAAGGGTAGTGGGAACAAGAGCTCTTGGGG ..((((....))))	PAPPA2	NM_021936	chr1:174698930-174926964	non-template	37	Intron_1.f	--	Yes	--	Yes	--	--	--	--	--	--	--
G4ST01174784067	chr1:174784068-174784104	+	GGGGGAGCAAGGGTAGTGGGAACAAGAGCTCTTGGGG ..((((....))))	PAPPA2	NM_020318	chr1:174698930-175078593	non-template	37	Intron_1.f	--	Yes	--	Yes	--	--	--	--	--	--	--
G4ST01175313951	chr1:175313952-175313978	-	GGGGTAGGGCTTTGGGAGATTCCTGGG ((....))	ASTN1	NM_004319	chr1:175096826-175400647	non-template	27	Intron_1.f	--	Yes	--	Yes	--	--	--	--	--	--	Yes
G4ST01175313951	chr1:175313952-175313978	-	GGGGTAGGGCTTTGGGAGATTCCTGGG ((....))	ASTN1	NM_207108	chr1:175104577-175400647	non-template	27	Intron_1.f	--	Yes	--	Yes	--	--	--	--	--	--	Yes
G4ST01176173095	chr1:176173096-176173135	-	GGGCTCCTACCCAGCAGGAGGGGGTGCAGGGCAGACAGGG (((((.......))))).	SEC16B	NM_033127	chr1:176164865-176205673	non-template	40	Exon_19	--	Yes	Yes	--	Yes	--	--	--	--	--	--
G4ST01176329804	chr1:176329805-176329835	-	GGGCTGGGCTTGGGGGCGTCGCCGCCAGGGG ((((....))))..	LOC100302401	NR_027982	chr1:176327266-176329751	non-template	-53	upstream	Yes	--	--	--	--	--	--	Yes	Yes	Yes	Yes
G4ST01176329804	chr1:176329805-176329835	-	GGGCTGGGCTTGGGGGCGTCGCCGCCAGGGG ((((....))))..	RASAL2	NM_170692	chr1:176329487-176715271	template	31	Exon_1.f	--	Yes	Yes	--	--	Yes	--	Yes	Yes	Yes	Yes
G4ST01176330204	chr1:176330205-176330237	-	GGGAGGGGGACGAGGCGGCGGCTTCGGGGCGGG ...((((((....))))))	LOC100302401	NR_027982	chr1:176327266-176329751	non-template	-453	upstream	Yes	--	--	--	--	--	--	Yes	Yes	Yes	--
G4ST01176330204	chr1:176330205-176330237	-	GGGAGGGGGACGAGGCGGCGGCTTCGGGGCGGG ...((((((....))))))	RASAL2	NM_170692	chr1:176329487-176715271	template	33	Exon_1.f	--	Yes	Yes	--	--	Yes	--	Yes	Yes	Yes	--
G4ST01176418610	chr1:176418611-176418642	+	GGGGGTGGGTAGGTGGGGGAAATGGTTTCGGG .(((.....)))	RASAL2	NM_170692	chr1:176329487-176715271	non-template	32	Intron_1.f	--	Yes	--	Yes	--	--	--	--	--	--	Yes
G4ST01176961301	chr1:176961302-176961339	+	GGGCCCCGGGGCTGCTCGCCCTGCGCGCAGGGAGGGGG ...((.(((...))).)).	RALGPS2	NM_152663	chr1:176960923-177155860	non-template	38	Intron_1.f	--	Yes	--	Yes	--	--	--	Yes	Yes	Yes	--
G4ST01177261628	chr1:177261629-177261654	+	GGGGGCCTCGGAGGCGGGGGGGTGGG .((((...))))	FAM20B	NM_014864	chr1:177261697-177312325	non-template	-42	upstream	Yes	--	--	--	--	--	--	Yes	Yes	Yes	Yes
G4ST01177318560	chr1:177318561-177318600	+	GGGCCGAGTCGGGGCTGGGAGTTAGAATCTGAGTTCTGGG ....(((((....)))))	TOR3A	NM_022371	chr1:177317735-177331752	non-template	40	Intron_1.f	--	Yes	--	Yes	--	--	--	Yes	Yes	Yes	--
G4ST01177340931	chr1:177340932-177340965	-	GGGTAGGGGGTGGGGGCTGTGTCTTACTCAGGGG ..(((.((...)).)))	ABL2	NM_005158	chr1:177335085-177378847	non-template	34	Exon_12.l	--	Yes	Yes	--	--	--	Yes	--	Yes	Yes	--
G4ST01177340931	chr1:177340932-177340965	-	GGGTAGGGGGTGGGGGCTGTGTCTTACTCAGGGG ..(((.((...)).)))	ABL2	NM_001168237	chr1:177335085-177465442	non-template	34	Exon_13.l	--	Yes	Yes	--	--	--	Yes	--	Yes	Yes	--
G4ST01177340931	chr1:177340932-177340965	-	GGGTAGGGGGTGGGGGCTGTGTCTTACTCAGGGG ..(((.((...)).)))	ABL2	NM_007314	chr1:177335085-177465442	non-template	34	Exon_12.l	--	Yes	Yes	--	--	--	Yes	--	Yes	Yes	--
G4ST01177340931	chr1:177340932-177340965	-	GGGTAGGGGGTGGGGGCTGTGTCTTACTCAGGGG ..(((.((...)).)))	ABL2	NM_001168239	chr1:177335085-177378847	non-template	34	Exon_12.l	--	Yes	Yes	--	--	--	Yes	--	Yes	Yes	--
G4ST01177340931	chr1:177340932-177340965	-	GGGTAGGGGGTGGGGGCTGTGTCTTACTCAGGGG ..(((.((...)).)))	ABL2	NM_001168238	chr1:177335085-177465442	non-template	34	Exon_12.l	--	Yes	Yes	--	--	--	Yes	--	Yes	Yes	--
G4ST01177340931	chr1:177340932-177340965	-	GGGTAGGGGGTGGGGGCTGTGTCTTACTCAGGGG ..(((.((...)).)))	ABL2	NM_001168236	chr1:177335085-177465442	non-template	34	Exon_11.l	--	Yes	Yes	--	--	--	Yes	--	Yes	Yes	--
G4ST01177340931	chr1:177340932-177340965	-	GGGTAGGGGGTGGGGGCTGTGTCTTACTCAGGGG ..(((.((...)).)))	ABL2	NM_001136000	chr1:177335085-177378847	non-template	34	Exon_13.l	--	Yes	Yes	--	--	--	Yes	--	Yes	Yes	--
G4ST01177344792	chr1:177344793-177344825	-	GGGGGTGGGGGCAGTGGCACTGCTGGGGGTGGG .(((((...)))))...	ABL2	NM_005158	chr1:177335085-177378847	non-template	33	Exon_12.l	--	Yes	Yes	--	Yes	--	--	--	--	--	--
G4ST01177344792	chr1:177344793-177344825	-	GGGGGTGGGGGCAGTGGCACTGCTGGGGGTGGG .(((((...)))))...	ABL2	NM_001168237	chr1:177335085-177465442	non-template	33	Intron_12.l	--	Yes	--	Yes	--	--	--	--	--	--	--
G4ST01177344792	chr1:177344793-177344825	-	GGGGGTGGGGGCAGTGGCACTGCTGGGGGTGGG .(((((...)))))...	ABL2	NM_007314	chr1:177335085-177465442	non-template	33	Exon_12.l	--	Yes	Yes	--	Yes	--	--	--	--	--	--
G4ST01177344792	chr1:177344793-177344825	-	GGGGGTGGGGGCAGTGGCACTGCTGGGGGTGGG .(((((...)))))...	ABL2	NM_001168239	chr1:177335085-177378847	non-template	33	Intron_11.l	--	Yes	--	Yes	--	--	--	--	--	--	--
G4ST01177344792	chr1:177344793-177344825	-	GGGGGTGGGGGCAGTGGCACTGCTGGGGGTGGG .(((((...)))))...	ABL2	NM_001168238	chr1:177335085-177465442	non-template	33	Intron_11.l	--	Yes	--	Yes	--	--	--	--	--	--	--
G4ST01177344792	chr1:177344793-177344825	-	GGGGGTGGGGGCAGTGGCACTGCTGGGGGTGGG .(((((...)))))...	ABL2	NM_001168236	chr1:177335085-177465442	non-template	33	Exon_11.l	--	Yes	Yes	--	Yes	--	--	--	--	--	--
G4ST01177344792	chr1:177344793-177344825	-	GGGGGTGGGGGCAGTGGCACTGCTGGGGGTGGG .(((((...)))))...	ABL2	NM_001136000	chr1:177335085-177378847	non-template	33	Intron_12.l	--	Yes	--	Yes	--	--	--	--	--	--	--
G4ST01177979691	chr1:177979692-177979718	+	GGGTAGGGTGGGGGCCGACCGGCGGGG .(((....))).	FAM163A	NM_173509	chr1:177978921-178051957	non-template	27	Intron_1.f	--	Yes	--	Yes	--	--	--	Yes	--	--	Yes
G4ST01177979696	chr1:177979697-177979727	+	GGGTGGGGGCCGACCGGCGGGGTCTGCTGGG .(((....))).	FAM163A	NM_173509	chr1:177978921-178051957	non-template	31	Intron_1.f	--	Yes	--	Yes	--	--	--	Yes	--	--	Yes
G4ST01177989056	chr1:177989057-177989091	+	GGGCTAGGGAGAAGGGGGAGTTGGTAATCAATGGG ...((((.....))))	FAM163A	NM_173509	chr1:177978921-178051957	non-template	35	Intron_1.f	--	Yes	--	Yes	--	--	--	Yes	--	--	--

G4ST01178037294	chr1:178037295-178037327	-	GGGTGAGGATTACCTGGGAAAATGGGGGGAGGG<br>..(((....)))	FAM163A	NM_173509	chr1:177978921-178051957	template	33	Intron_1.f	--	Yes	--	Yes	--	--	--	--	--	--	--
G4ST01178049602	chr1:178049603-178049645	-	GGGAGCTGCAGGCATTGCAGGTGGGGCCTCTGGGATGCGTGGG<br>..(((((.....)))))...	FAM163A	NM_173509	chr1:177978921-178051957	template	43	Exon_5.l	--	Yes	Yes	--	Yes	--	--	--	--	--	--
G4ST01178049611	chr1:178049612-178049651	-	GGGCTTGGGAGCTGCAGGCATTGCAGGTGGGGCCTCTGGG<br>..(((((.....)))))...	FAM163A	NM_173509	chr1:177978921-178051957	template	40	Exon_5.l	--	Yes	Yes	--	Yes	--	--	--	--	--	--
G4ST01178190789	chr1:178190790-178190827	+	GGGGAGGCCAGGCCGGGCAGCCCTGGGGCCGGTCGGGG<br>..(((...)))	CEP350	NM_014810	chr1:178190531-178350638	non-template	38	Exon_1.f	--	Yes	Yes	--	--	Yes	--	Yes	Yes	Yes	--
G4ST01178337393	chr1:178337394-178337436	-	GGGGGCAGGGGCAGTCTTGGGGACTGAGCACTCACTCTGTGGG<br>.((.(((......))).))	CEP350	NM_014810	chr1:178190531-178350638	template	43	Intron_37.l	--	Yes	--	Yes	--	--	--	Yes	--	--	--
G4ST01178390504	chr1:178390505-178390534	+	GGGGCGGGGTGGCTGGGCCGGCCTCGGGGG<br>(((....)))	QSOX1	NM_001004128	chr1:178390591-178433792	non-template	-56	upstream	Yes	--	--	--	--	--	--	Yes	Yes	Yes	Yes
G4ST01178390504	chr1:178390505-178390534	+	GGGGCGGGGTGGCTGGGCCGGCCTCGGGGG<br>(((....)))	QSOX1	NM_002826	chr1:178390591-178433792	non-template	-56	upstream	Yes	--	--	--	--	--	--	Yes	Yes	Yes	Yes
G4ST01178390509	chr1:178390510-178390540	+	GGGGTGGCTGGGCCGGCCTCGGGGGCGGGGG<br>(((....)))	QSOX1	NM_001004128	chr1:178390591-178433792	non-template	-50	upstream	Yes	--	--	--	--	--	--	Yes	Yes	Yes	Yes
G4ST01178390509	chr1:178390510-178390540	+	GGGGTGGCTGGGCCGGCCTCGGGGGCGGGGG<br>(((....)))	QSOX1	NM_002826	chr1:178390591-178433792	non-template	-50	upstream	Yes	--	--	--	--	--	--	Yes	Yes	Yes	Yes
G4ST01178390518	chr1:178390519-178390548	+	GGGCCGGCCTCGGGGGCGGGGGCTCGGGGG<br>(((....)))	QSOX1	NM_001004128	chr1:178390591-178433792	non-template	-42	upstream	Yes	--	--	--	--	--	--	Yes	Yes	Yes	Yes
G4ST01178390518	chr1:178390519-178390548	+	GGGCCGGCCTCGGGGGCGGGGGCTCGGGGG<br>(((....)))	QSOX1	NM_002826	chr1:178390591-178433792	non-template	-42	upstream	Yes	--	--	--	--	--	--	Yes	Yes	Yes	Yes
G4ST01178391586	chr1:178391587-178391624	-	GGGTGCATGGGTTTAAAATATAAGGGTCTGAGCGGGGG<br>.(((.....)))	QSOX1	NM_001004128	chr1:178390591-178433792	template	38	Intron_1.f	--	Yes	--	Yes	--	--	--	--	Yes	--	--
G4ST01178391586	chr1:178391587-178391624	-	GGGTGCATGGGTTTAAAATATAAGGGTCTGAGCGGGGG<br>.(((.....)))	QSOX1	NM_002826	chr1:178390591-178433792	template	38	Intron_1.f	--	Yes	--	Yes	--	--	--	--	Yes	--	--
G4ST01178417876	chr1:178417877-178417909	-	GGGGGCCAGGCCACCGGGTGGAGGGAGCCAGGG<br>(((...)))...	QSOX1	NM_001004128	chr1:178390591-178433792	template	33	Intron_5	--	Yes	--	Yes	--	--	--	--	--	--	Yes
G4ST01178417876	chr1:178417877-178417909	-	GGGGGCCAGGCCACCGGGTGGAGGGAGCCAGGG<br>(((...)))...	QSOX1	NM_002826	chr1:178390591-178433792	template	33	Intron_5	--	Yes	--	Yes	--	--	--	--	--	--	Yes
G4ST01178417884	chr1:178417885-178417926	-	GGGACAGCAGAACATCTGGGGGCCAGGCCACCGGGTGGAGGG<br>....((((...)))). (((...)))...	QSOX1	NM_001004128	chr1:178390591-178433792	template	42	Intron_5	--	Yes	--	Yes	--	--	--	--	--	--	Yes
G4ST01178417884	chr1:178417885-178417926	-	GGGACAGCAGAACATCTGGGGGCCAGGCCACCGGGTGGAGGG<br>....((((...)))). (((...)))...	QSOX1	NM_002826	chr1:178390591-178433792	template	42	Intron_5	--	Yes	--	Yes	--	--	--	--	--	--	Yes
G4ST01178424064	chr1:178424065-178424094	-	GGGAGCACAGGCTGGGAGAGGCCGGGTGGG<br>(((....)))	QSOX1	NM_001004128	chr1:178390591-178433792	template	30	Intron_8	--	Yes	--	Yes	--	--	--	Yes	Yes	--	--
G4ST01178424064	chr1:178424065-178424094	-	GGGAGCACAGGCTGGGAGAGGCCGGGTGGG<br>(((....)))	QSOX1	NM_002826	chr1:178390591-178433792	template	30	Intron_8	--	Yes	--	Yes	--	--	--	Yes	Yes	--	--
G4ST01178434003	chr1:178434004-178434046	-	GGGCACTGAGGCAGGAGCTCTCAGGGAGGATTGGGGCAGGGGG<br>....(((((....)).))).	FLJ23867	NR_026900	chr1:178433767-178436482	template	43	Exon_1.u	--	Yes	Yes	--	--	--	--	Yes	--	--	--
G4ST01178465224	chr1:178465225-178465254	-	GGGCGGCGCCGGCCCGGGCTGGGGAAGGGG<br>.(((....))).	LHX4	NM_033343	chr1:178466056-178510811	template	-801	upstream	Yes	--	--	--	--	--	--	--	Yes	Yes	Yes
G4ST01178465481	chr1:178465482-178465522	-	GGGGCCGAGCGGGGCAGGCGGTCCGGAGGCCGGGGACGGGG<br>.....(((.((...)))))	LHX4	NM_033343	chr1:178466056-178510811	template	-533	upstream	Yes	--	--	--	--	--	--	Yes	Yes	Yes	--
G4ST01178484291	chr1:178484292-178484326	+	GGGTAGGCCAGGGAGGGTGGAGAGTCCACTCTGGG<br>...((((....))))	LHX4	NM_033343	chr1:178466056-178510811	non-template	35	Intron_2	--	Yes	--	Yes	--	--	--	--	Yes	--	--
G4ST01178484301	chr1:178484302-178484331	+	GGGAGGGTGGAGAGTCCACTCTGGGAGGGG<br>...((((....))))	LHX4	NM_033343	chr1:178466056-178510811	non-template	30	Intron_2	--	Yes	--	Yes	--	--	--	--	Yes	--	--
G4ST01178486878	chr1:178486879-178486911	-	GGGAAGGGGCAGGCCCTGGCCTGGGCTGTGGGG<br>..((((....))))	LHX4	NM_033343	chr1:178466056-178510811	template	33	Intron_2	--	Yes	--	Yes	--	--	--	--	--	--	--
G4ST01178489504	chr1:178489505-178489535	-	GGGCTGCGGGTCAAGGGGCCACACGGCAGGG<br>(((....))).	LHX4	NM_033343	chr1:178466056-178510811	template	31	Intron_2	--	Yes	--	Yes	--	--	--	Yes	Yes	--	--
G4ST01178491688	chr1:178491689-178491722	+	GGGATCTGGGTGACATGGGAGTGGAAGCTGTGGG<br>(((....)))..	LHX4	NM_033343	chr1:178466056-178510811	non-template	34	Intron_2	--	Yes	--	Yes	--	--	--	--	--	--	--
G4ST01178494820	chr1:178494821-178494859	+	GGGGTCTGGGCTGGAGGTGAGATCCCCAGGGGAGCGGGG<br>((((.((......))))))	LHX4	NM_033343	chr1:178466056-178510811	non-template	39	Intron_2	--	Yes	--	Yes	--	--	--	--	--	--	--
G4ST01178494827	chr1:178494828-178494863	+	GGGCTGGAGGTGAGATCCCCAGGGGAGCGGGGTGGG<br>((((.((......))))))	LHX4	NM_033343	chr1:178466056-178510811	non-template	36	Intron_2	--	Yes	--	Yes	--	--	--	--	--	--	--
G4ST01178494959	chr1:178494960-178494992	+	GGGAGGCCAGGGAGAGGGGAGCTGACCGCTGGG<br>.(((.....)))	LHX4	NM_033343	chr1:178466056-178510811	non-template	33	Intron_2	--	Yes	--	Yes	--	--	--	--	--	--	Yes
G4ST01178496954	chr1:178496955-178496987	+	GGGAGGCCGGGGAGAGGGGAGCTGACCGCTGGG<br>.(((.....)))	LHX4	NM_033343	chr1:178466056-178510811	non-template	33	Intron_2	--	Yes	--	Yes	--	--	--	--	--	--	Yes
G4ST01178506874	chr1:178506875-178506902	+	GGGCTGGCAGGGGAGGGTTGAGGCAGGG<br>.((...))	LOC100527964	NR_037642	chr1:178505421-178510439	template	28	Exon_5.l	--	Yes	Yes	--	--	--	--	--	Yes	--	--
G4ST01178506874	chr1:178506875-178506902	+	GGGCTGGCAGGGGAGGGTTGAGGCAGGG<br>.((...))	LHX4	NM_033343	chr1:178466056-178510811	non-template	28	Intron_3	--	Yes	--	Yes	--	--	--	--	Yes	--	--
G4ST01178738341	chr1:178738342-178738387	+	GGGATGCGTGCGAGCGCGGGGCGCGGCTGGCGCTGGGCCGTGGGGG<br>..((((....)))). ((((.....)))).	ACBD6	NM_032360	chr1:178523975-178738645	template	46	Exon_1.f	--	Yes	Yes	--	--	Yes	--	Yes	Yes	Yes	--
G4ST01178738358	chr1:178738359-178738392	+	GGGGCGCGGCTGGCGCTGGGCCGTGGGGGCGGGG<br>((((.....)))).	ACBD6	NM_032360	chr1:178523975-178738645	template	34	Exon_1.f	--	Yes	Yes	--	--	Yes	--	Yes	Yes	Yes	--
G4ST01178868382	chr1:178868383-178868417	-	GGGGACGAGAGGTCAGGGCGAGTGGGGAGAAGGGG<br>(((.....))).	XPR1	NM_004736	chr1:178867769-179126038	template	35	Intron_1.f	--	Yes	--	Yes	--	--	--	Yes	Yes	Yes	--
G4ST01178868382	chr1:178868383-178868417	-	GGGGACGAGAGGTCAGGGCGAGTGGGGAGAAGGGG<br>(((.....))).	XPR1	NM_001135669	chr1:178867769-179126038	template	35	Intron_1.f	--	Yes	--	Yes	--	--	--	Yes	Yes	Yes	--
G4ST01178868390	chr1:178868391-178868421	-	GGGTGGGGACGAGAGGTCAGGGCGAGTGGGG<br>(((.....))).	XPR1	NM_004736	chr1:178867769-179126038	template	31	Intron_1.f	--	Yes	--	Yes	--	--	--	Yes	Yes	Yes	--
G4ST01178868390	chr1:178868391-178868421	-	GGGTGGGGACGAGAGGTCAGGGCGAGTGGGG<br>(((.....))).	XPR1	NM_001135669	chr1:178867769-179126038	template	31	Intron_1.f	--	Yes	--	Yes	--	--	--	Yes	Yes	Yes	--
G4ST01178868399	chr1:178868400-178868436	-	GGGGGCGCTCGCCCAGGGTGGGGACGAGAGGTCAGGG<br>(((....))).. (((.....))).	XPR1	NM_004736	chr1:178867769-179126038	template	37	Intron_1.f	--	Yes	--	Yes	--	--	--	Yes	Yes	Yes	--
G4ST01178868399	chr1:178868400-178868436	-	GGGGGCGCTCGCCCAGGGTGGGGACGAGAGGTCAGGG<br>(((....))).. (((.....))).	XPR1	NM_001135669	chr1:178867769-179126038	template	37	Intron_1.f	--	Yes	--	Yes	--	--	--	Yes	Yes	Yes	--
G4ST01179045437	chr1:179045438-179045468	+	GGGAAGGTTTTGGGATCAGGGAGGAATAGGG<br>((....))	XPR1	NM_004736	chr1:178867769-179126038	non-template	31	Intron_6	--	Yes	--	Yes	--	--	--	--	Yes	--	--
G4ST01179045437	chr1:179045438-179045468	+	GGGAAGGTTTTGGGATCAGGGAGGAATAGGG<br>((....))	XPR1	NM_001135669	chr1:178867769-179126038	non-template	31	Intron_6	--	Yes	--	Yes	--	--	--	--	Yes	--	--
G4ST01179148362	chr1:179148363-179148417	-	GGGCGGTGGCCCCTTCCCCGCGGGAAAGCGGGCGCAAGAGCGGAGGCGCGCGGGG<br>(((.((......))))). (((....((((....))))))	KIAA1614	NM_020950	chr1:179148936-179181862	template	-518	upstream	Yes	--	--	--	--	--	--	Yes	Yes	Yes	--
G4ST01179148567	chr1:179148568-179148597	-	GGGGCCGCGGGAGGGGCTGGAGGAGCCGGG<br>(((.....))).	KIAA1614	NM_020950	chr1:179148936-179181862	template	-338	upstream	Yes	--	--	--	--	--	--	Yes	Yes	Yes	--
G4ST01179164011	chr1:179164012-179164043	-	GGGAACCCAGCCTGGGGGCCTGGGGCCTGGGG<br>...(((...)))	KIAA1614	NM_020950	chr1:179148936-179181862	template	32	Intron_3	--	Yes	--	Yes	--	--	--	--	--	--	Yes
G4ST01179164338	chr1:179164339-179164367	+	GGGACCTGGGCCAGGCTAGCCTGGGAGGG<br>..(((....)))	KIAA1614	NM_020950	chr1:179148936-179181862	non-template	29	Intron_4	--	Yes	--	Yes	--	--	--	--	--	--	--
G4ST01179253541	chr1:179253542-179253580	+	GGGAATTGGGTCCCTCAAACACTTTGAGGGGCAAGGGGG<br>...(((((.....)))))	STX6	NM_005819	chr1:179208799-179258669	template	39	Intron_1.f	--	Yes	--	Yes	--	--	--	--	--	--	--
G4ST01179258666	chr1:179258667-179258708	-	GGGGGTGGGGTCAGGCCCTCCGACGCTTCGGGAGGAGCCGGG<br>(((.((....)))))....	STX6	NM_005819	chr1:179208799-179258669	non-template	3	upstream-Exon_1.f	Yes	Yes	Yes	--	--	Yes	--	Yes	Yes	Yes	--

G4ST01179258676	chr1:179258677-179258715	-	GGGTCCCGGGGGTGGGGTCAGGCCCTCCGACGCTTCGGG<br>(((.((....))))).....	STX6	NM_005819	chr1:179208799-179258669	non-template	-7	upstream	Yes	--	--	--	--	--	--	Yes	Yes	Yes	--
G4ST01179278914	chr1:179278915-179278948	-	GGGTTGGGAGGGAAGACAGTGGAAGCCTCTGGGG<br>....(((.((...)).)))	MR1	NM_001195035	chr1:179269184-179297697	template	34	Intron_2	--	Yes	--	Yes	--	--	--	Yes	Yes	--	Yes
G4ST01179278914	chr1:179278915-179278948	-	GGGTTGGGAGGGAAGACAGTGGAAGCCTCTGGGG<br>....(((.((...)).)))	MR1	NM_001195000	chr1:179269184-179297697	template	34	Intron_2	--	Yes	--	Yes	--	--	--	Yes	Yes	--	Yes
G4ST01179278914	chr1:179278915-179278948	-	GGGTTGGGAGGGAAGACAGTGGAAGCCTCTGGGG<br>....(((.((...)).)))	MR1	NM_001531	chr1:179269184-179297697	template	34	Intron_2	--	Yes	--	Yes	--	--	--	Yes	Yes	--	Yes
G4ST01179278914	chr1:179278915-179278948	-	GGGTTGGGAGGGAAGACAGTGGAAGCCTCTGGGG<br>....(((.((...)).)))	MR1	NM_001194999	chr1:179269184-179297697	template	34	Intron_2	--	Yes	--	Yes	--	--	--	Yes	Yes	--	Yes
G4ST01179324810	chr1:179324811-179324852	-	GGGGTCCCAGCGGGACCGGCCAGGTAGAGGCCGGGGCACGGG<br>..(((((.......)))))	IER5	NM_016545	chr1:179324261-179326602	template	42	Exon_1.u	--	Yes	Yes	--	Yes	--	--	--	Yes	Yes	--
G4ST01179325353	chr1:179325354-179325388	-	GGGGCTTCTTGAGCGGGGTCGAGCCGGGCGCCGGG<br>(((.....))).	IER5	NM_016545	chr1:179324261-179326602	template	35	Exon_1.u	--	Yes	Yes	--	Yes	--	--	Yes	Yes	Yes	--
G4ST01179746991	chr1:179746992-179747032	-	GGGCCTGCCCATGTGTAGGTGGGAAGCAAGGGTGCTAAGGG<br>(((((......))))).	CACNA1E	NM_000721	chr1:179719339-180037338	template	41	Intron_2	--	Yes	--	Yes	--	--	--	--	--	--	--
G4ST01179788504	chr1:179788505-179788535	-	GGGGGCGAGGGGAGGGAATTTAGAGGATGGG<br>.(((.....)))	CACNA1E	NM_000721	chr1:179719339-180037338	template	31	Intron_3	--	Yes	--	Yes	--	--	--	--	--	--	Yes
G4ST01179792223	chr1:179792224-179792255	+	GGGACTCAGAGAGGGGCCCCTGTGGGAGGGGG<br>.(((...)))	CACNA1E	NM_000721	chr1:179719339-180037338	non-template	32	Intron_3	--	Yes	--	Yes	--	--	--	--	--	--	Yes
G4ST01179832738	chr1:179832739-179832773	+	GGGCAGGGAGAATGGGTGCAGATTGCAAGTGGGGG<br>((((...)))).....	CACNA1E	NM_000721	chr1:179719339-180037338	non-template	35	Intron_6	--	Yes	--	Yes	--	--	--	--	--	--	Yes
G4ST01179859973	chr1:179859974-179860004	-	GGGGCCTAATGGGAGGTGTCTGGGTCATGGG<br>((....))	CACNA1E	NM_000721	chr1:179719339-180037338	template	31	Intron_6	--	Yes	--	Yes	--	--	--	--	--	--	--
G4ST01179936000	chr1:179936001-179936036	-	GGGTCCAGGGTCTGTGGACTCAACAGGGGTTTGGGG<br>.((((.......))))	CACNA1E	NM_000721	chr1:179719339-180037338	template	36	Intron_7	--	Yes	--	Yes	--	--	--	--	--	--	--
G4ST01179968262	chr1:179968263-179968294	-	GGGAGCTGAGCGGGTTGAGGGGGTTGAGCGGG<br>.((...))	CACNA1E	NM_000721	chr1:179719339-180037338	template	32	Exon_20	--	Yes	Yes	--	Yes	--	--	--	--	--	--
G4ST01179968271	chr1:179968272-179968301	-	GGGTTGAGGGAGCTGAGCGGGTTGAGGGGG<br>.((...))	CACNA1E	NM_000721	chr1:179719339-180037338	template	30	Exon_20	--	Yes	Yes	--	Yes	--	--	--	--	--	--
G4ST01179989645	chr1:179989646-179989676	-	GGGAACCCACGCGGTGGGCCAGGGCCCTGGG<br>.(((.....)))	CACNA1E	NM_000721	chr1:179719339-180037338	template	31	Intron_27	--	Yes	--	Yes	--	--	--	--	--	--	--
G4ST01180628113	chr1:180628114-180628137	+	GGGCTAGGGCGGGTGCCGCGCGGG<br>.((...))	GLUL	NM_002065	chr1:180617462-180627964	template	-149	upstream	Yes	--	--	--	--	--	--	Yes	Yes	Yes	Yes
G4ST01180628113	chr1:180628114-180628137	+	GGGCTAGGGCGGGTGCCGCGCGGG<br>.((...))	GLUL	NM_001033056	chr1:180617462-180627162	template	-951	upstream	Yes	--	--	--	--	--	--	Yes	Yes	Yes	Yes
G4ST01180628113	chr1:180628114-180628137	+	GGGCTAGGGCGGGTGCCGCGCGGG<br>.((...))	GLUL	NM_001033044	chr1:180617462-180627964	template	-149	upstream	Yes	--	--	--	--	--	--	Yes	Yes	Yes	Yes
G4ST01180628119	chr1:180628120-180628144	+	GGGCGGGTGCCGCGCGGGCCTGGGG<br>.((...))	GLUL	NM_002065	chr1:180617462-180627964	template	-155	upstream	Yes	--	--	--	--	--	--	Yes	Yes	Yes	Yes
G4ST01180628119	chr1:180628120-180628144	+	GGGCGGGTGCCGCGCGGGCCTGGGG<br>.((...))	GLUL	NM_001033056	chr1:180617462-180627162	template	-957	upstream	Yes	--	--	--	--	--	--	Yes	Yes	Yes	Yes
G4ST01180628119	chr1:180628120-180628144	+	GGGCGGGTGCCGCGCGGGCCTGGGG<br>.((...))	GLUL	NM_001033044	chr1:180617462-180627964	template	-155	upstream	Yes	--	--	--	--	--	--	Yes	Yes	Yes	Yes
G4ST01180628123	chr1:180628124-180628155	+	GGGTGCCGCGCGGGCCTGGGGACAGGCCGGGG<br>.((...))	GLUL	NM_002065	chr1:180617462-180627964	template	-159	upstream	Yes	--	--	--	--	--	--	Yes	Yes	Yes	Yes
G4ST01180628123	chr1:180628124-180628155	+	GGGTGCCGCGCGGGCCTGGGGACAGGCCGGGG<br>.((...))	GLUL	NM_001033056	chr1:180617462-180627162	template	-961	upstream	Yes	--	--	--	--	--	--	Yes	Yes	Yes	Yes
G4ST01180628123	chr1:180628124-180628155	+	GGGTGCCGCGCGGGCCTGGGGACAGGCCGGGG<br>.((...))	GLUL	NM_001033044	chr1:180617462-180627964	template	-159	upstream	Yes	--	--	--	--	--	--	Yes	Yes	Yes	Yes
G4ST01180643814	chr1:180643815-180643849	-	GGGGCCCCTTGGGACTGTGGGATGGTGCATTGGGG<br>(((...)))..	NCRNA00272	NR_034131	chr1:180643379-180650571	template	35	Intron_1.f	--	Yes	--	Yes	--	--	--	--	Yes	--	--
G4ST01180706955	chr1:180706956-180706996	+	GGGGGTCTGGCAGTGTGCAAGGCTGGGAGAGGGGTCAGGGG<br>.((((.(((...))).)))).	RGSL1	NM_001137669	chr1:180685879-180796355	non-template	41	Intron_4	--	Yes	--	Yes	--	--	--	--	--	--	--
G4ST01180786772	chr1:180786773-180786811	-	GGGGGCTGGAGCACCGAGCACAGGGGAAACAGGGCCGGG<br>..(((..((.....)).)))	RGSL1	NM_001137669	chr1:180685879-180796355	template	39	Intron_17	--	Yes	--	Yes	--	--	--	--	--	--	--
G4ST01180824764	chr1:180824765-180824805	+	GGGCACTGGAGGAAGATCCTTCCGGGCAGCCAAGGGCTGGG<br>.......(((((...)))))	RNASEL	NM_021133	chr1:180809392-180825017	template	41	Intron_1.f	--	Yes	--	Yes	--	--	--	Yes	Yes	Yes	--
G4ST01180838939	chr1:180838940-180838964	-	GGGCTCCGAGAGGGAAGGGAGTGGG<br>((...)).	RGS16	NM_002928	chr1:180834381-180840171	non-template	25	Intron_2	--	Yes	--	Yes	--	--	--	Yes	Yes	Yes	Yes
G4ST01180890394	chr1:180890395-180890428	+	GGGCTATAGTGGGGAACTCTAAGAGTGGGAAGGG<br>..((((...))))	RGS8	NM_033345	chr1:180882415-180908690	template	34	Intron_4	--	Yes	--	Yes	--	--	--	--	--	--	--
G4ST01180890394	chr1:180890395-180890428	+	GGGCTATAGTGGGGAACTCTAAGAGTGGGAAGGG<br>..((((...))))	RGS8	NM_001102450	chr1:180882415-180908690	template	34	Intron_5	--	Yes	--	Yes	--	--	--	--	--	--	--
G4ST01181075309	chr1:181075310-181075342	+	GGGCGGCGGCGGCGGCTGGGGGTGGGGGAAGGG<br>...((((....)))).	DHX9	NR_033302	chr1:181075062-181123740	non-template	33	Intron_1.f	--	Yes	--	Yes	--	--	--	Yes	Yes	Yes	--
G4ST01181075309	chr1:181075310-181075342	+	GGGCGGCGGCGGCGGCTGGGGGTGGGGGAAGGG<br>...((((....)))).	DHX9	NM_001357	chr1:181075062-181123740	non-template	33	Intron_1.f	--	Yes	--	Yes	--	--	--	Yes	Yes	Yes	--
G4ST01181258813	chr1:181258814-181258839	-	GGGTGGTGCATGCCGGGAGGGAAGGG<br>.(((....)))	LAMC1	NM_002293	chr1:181259218-181381350	template	-378	upstream	Yes	--	--	--	--	--	--	Yes	Yes	Yes	Yes
G4ST01181258818	chr1:181258819-181258847	-	GGGCGGCTGGGTGGTGCATGCCGGGAGGG<br>.(((....)))	LAMC1	NM_002293	chr1:181259218-181381350	template	-370	upstream	Yes	--	--	--	--	--	--	Yes	Yes	Yes	Yes
G4ST01181258822	chr1:181258823-181258852	-	GGGGAGGGCGGCTGGGTGGTGCATGCCGGG<br>.(((....)))	LAMC1	NM_002293	chr1:181259218-181381350	template	-365	upstream	Yes	--	--	--	--	--	--	Yes	Yes	Yes	Yes
G4ST01181259906	chr1:181259907-181259945	-	GGGAGCTGGGCTGCTGGCGAGCAGTAGCAGGGGACGGGG<br>(((((....))))).......	LAMC1	NM_002293	chr1:181259218-181381350	template	39	Intron_1.f	--	Yes	--	Yes	--	--	--	Yes	Yes	Yes	--
G4ST01181370125	chr1:181370126-181370157	+	GGGTGGAAAGGGAGAAAAGGGAGCTTCTTGGG<br>((...)).	LAMC1	NM_002293	chr1:181259218-181381350	non-template	32	Intron_22	--	Yes	--	Yes	--	--	--	--	--	--	--
G4ST01181486929	chr1:181486930-181486958	-	GGGTTCCCAGAAGGGAGATCTGGGAGGGG<br>(((...)))	NMNAT2	NM_170706	chr1:181483995-181540632	non-template	29	Exon_11.l	--	Yes	Yes	--	--	--	Yes	--	--	--	--
G4ST01181486929	chr1:181486930-181486958	-	GGGTTCCCAGAAGGGAGATCTGGGAGGGG<br>(((...)))	NMNAT2	NM_015039	chr1:181483995-181654257	non-template	29	Exon_11.l	--	Yes	Yes	--	--	--	Yes	--	--	--	--
G4ST01181486934	chr1:181486935-181486965	-	GGGGAGTGGGTTCCCAGAAGGGAGATCTGGG<br>(((...)))	NMNAT2	NM_170706	chr1:181483995-181540632	non-template	31	Exon_11.l	--	Yes	Yes	--	--	--	Yes	Yes	Yes	--	--
G4ST01181486934	chr1:181486935-181486965	-	GGGGAGTGGGTTCCCAGAAGGGAGATCTGGG<br>(((...)))	NMNAT2	NM_015039	chr1:181483995-181654257	non-template	31	Exon_11.l	--	Yes	Yes	--	--	--	Yes	Yes	Yes	--	--
G4ST01181505334	chr1:181505335-181505366	+	GGGATGCACATGGGCCCATTGGGAAGGAGGGG<br>((....))	NMNAT2	NM_170706	chr1:181483995-181540632	template	32	Intron_8	--	Yes	--	Yes	--	--	--	--	--	--	--
G4ST01181505334	chr1:181505335-181505366	+	GGGATGCACATGGGCCCATTGGGAAGGAGGGG<br>((....))	NMNAT2	NM_015039	chr1:181483995-181654257	template	32	Intron_8	--	Yes	--	Yes	--	--	--	--	--	--	--
G4ST01181511959	chr1:181511960-181511995	+	GGGTTGGGGCTGGGACTTGCCAAGTAGTCAAGTGGG<br>(((((.........)))))	NMNAT2	NM_170706	chr1:181483995-181540632	template	36	Intron_8	--	Yes	--	Yes	--	--	--	Yes	Yes	--	Yes
G4ST01181511959	chr1:181511960-181511995	+	GGGTTGGGGCTGGGACTTGCCAAGTAGTCAAGTGGG<br>(((((.........)))))	NMNAT2	NM_015039	chr1:181483995-181654257	template	36	Intron_8	--	Yes	--	Yes	--	--	--	Yes	Yes	--	Yes
G4ST01181538106	chr1:181538107-181538134	+	GGGAGGGAGTCATCCCACTGGGACGGGG<br>(((......)))	NMNAT2	NM_170706	chr1:181483995-181540632	template	28	Intron_1.f	--	Yes	--	Yes	--	--	--	--	--	--	--
G4ST01181538106	chr1:181538107-181538134	+	GGGAGGGAGTCATCCCACTGGGACGGGG<br>(((......)))	NMNAT2	NM_015039	chr1:181483995-181654257	template	28	Intron_1.f	--	Yes	--	Yes	--	--	--	--	--	--	--
G4ST01181641047	chr1:181641048-181641074	+	GGGCAAGGATTGGGAAGTGGGTAGGGG<br>.((...))	NMNAT2	NM_015039	chr1:181483995-181654257	template	27	Intron_1.f	--	Yes	--	Yes	--	--	--	--	--	--	--

G4ST01181653693	chr1:181653694-181653721	-	GGGGGCCAAAGGTGGGGGATGGGAGGGG<br>..(((....)))	NMNAT2	NM_015039	chr1:181483995-181654257	non-template	28	Intron_1.f	--	Yes	--	Yes	--	--	--	--	Yes	--	--
G4ST01181708832	chr1:181708833-181708871	+	GGGGAGGAAGTCTGCACACCTGGGGTGTTGGGGCGGGGG<br>.(((..((....)).))).	SMG7	NM_001174061	chr1:181708129-181789951	non-template	39	Intron_1.f	--	Yes	--	Yes	--	--	--	Yes	Yes	Yes	--
G4ST01181708832	chr1:181708833-181708871	+	GGGGAGGAAGTCTGCACACCTGGGGTGTTGGGGCGGGGG<br>.(((..((....)).))).	SMG7	NM_173156	chr1:181708129-181789951	non-template	39	Intron_1.f	--	Yes	--	Yes	--	--	--	Yes	Yes	Yes	--
G4ST01181708832	chr1:181708833-181708871	+	GGGGAGGAAGTCTGCACACCTGGGGTGTTGGGGCGGGGG<br>.(((..((....)).))).	SMG7	NM_201569	chr1:181708129-181789951	non-template	39	Intron_1.f	--	Yes	--	Yes	--	--	--	Yes	Yes	Yes	--
G4ST01181708832	chr1:181708833-181708871	+	GGGGAGGAAGTCTGCACACCTGGGGTGTTGGGGCGGGGG<br>.(((..((....)).))).	SMG7	NM_201568	chr1:181708129-181789951	non-template	39	Intron_1.f	--	Yes	--	Yes	--	--	--	Yes	Yes	Yes	--
G4ST01181739375	chr1:181739376-181739407	+	GGGATCTCATGAAGCAGTGAGGGGAGGGAGGG<br>..(((((......)))))	SMG7	NM_001174061	chr1:181708129-181789951	non-template	32	Intron_1.f	--	Yes	--	Yes	--	--	--	--	--	--	--
G4ST01181739375	chr1:181739376-181739407	+	GGGATCTCATGAAGCAGTGAGGGGAGGGAGGG<br>..(((((......)))))	SMG7	NM_173156	chr1:181708129-181789951	non-template	32	Intron_1.f	--	Yes	--	Yes	--	--	--	--	--	--	--
G4ST01181739375	chr1:181739376-181739407	+	GGGATCTCATGAAGCAGTGAGGGGAGGGAGGG<br>..(((((......)))))	SMG7	NM_201569	chr1:181708129-181789951	non-template	32	Intron_1.f	--	Yes	--	Yes	--	--	--	--	--	--	--
G4ST01181739375	chr1:181739376-181739407	+	GGGATCTCATGAAGCAGTGAGGGGAGGGAGGG<br>..(((((......)))))	SMG7	NM_201568	chr1:181708129-181789951	non-template	32	Intron_1.f	--	Yes	--	Yes	--	--	--	--	--	--	--
G4ST01181814644	chr1:181814645-181814674	+	GGGAGGGAGCCATTGCTGGGCTATAGGGGG<br>(((....)))	NCF2	NM_001190794	chr1:181791320-181826362	template	30	Intron_2	--	Yes	--	Yes	--	--	--	--	--	Yes	--
G4ST01181814644	chr1:181814645-181814674	+	GGGAGGGAGCCATTGCTGGGCTATAGGGGG<br>(((....)))	NCF2	NM_001190789	chr1:181791320-181826362	template	30	Intron_2	--	Yes	--	Yes	--	--	--	--	--	Yes	--
G4ST01181814644	chr1:181814645-181814674	+	GGGAGGGAGCCATTGCTGGGCTATAGGGGG<br>(((....)))	NCF2	NM_001127651	chr1:181791320-181826679	template	30	Intron_3	--	Yes	--	Yes	--	--	--	--	--	Yes	--
G4ST01181814644	chr1:181814645-181814674	+	GGGAGGGAGCCATTGCTGGGCTATAGGGGG<br>(((....)))	NCF2	NM_000433	chr1:181791320-181826362	template	30	Intron_2	--	Yes	--	Yes	--	--	--	--	--	Yes	--
G4ST01181814648	chr1:181814649-181814678	+	GGGAGCCATTGCTGGGCTATAGGGGGTGGG<br>(((....)))	NCF2	NM_001190794	chr1:181791320-181826362	template	30	Intron_2	--	Yes	--	Yes	--	--	--	--	--	--	--
G4ST01181814648	chr1:181814649-181814678	+	GGGAGCCATTGCTGGGCTATAGGGGGTGGG<br>(((....)))	NCF2	NM_001190789	chr1:181791320-181826362	template	30	Intron_2	--	Yes	--	Yes	--	--	--	--	--	--	--
G4ST01181814648	chr1:181814649-181814678	+	GGGAGCCATTGCTGGGCTATAGGGGGTGGG<br>(((....)))	NCF2	NM_001127651	chr1:181791320-181826679	template	30	Intron_3	--	Yes	--	Yes	--	--	--	--	--	--	--
G4ST01181814648	chr1:181814649-181814678	+	GGGAGCCATTGCTGGGCTATAGGGGGTGGG<br>(((....)))	NCF2	NM_000433	chr1:181791320-181826362	template	30	Intron_2	--	Yes	--	Yes	--	--	--	--	--	--	--
G4ST01182151564	chr1:182151565-182151597	+	GGGAACTCGACAGTGGGAATCAGGGAGCTTGGG<br>.(((....)))	RGL1	NM_015149	chr1:181871831-182164289	non-template	33	Intron_16	--	Yes	--	Yes	--	--	--	Yes	Yes	--	--
G4ST01182265262	chr1:182265263-182265303	+	GGGTCTGATGGGGACTGGGCTGCCTGGACAAAATCCAGGGG<br>....(((((.....)))))	GLT25D2	NM_015101	chr1:182171589-182273486	template	41	Intron_1.f	--	Yes	--	Yes	--	--	--	--	--	--	--
G4ST01182272807	chr1:182272808-182272839	-	GGGCCCCGGGCCGCCGCGGCGGGACGCGGGGG<br>(((...))).	GLT25D2	NM_015101	chr1:182171589-182273486	non-template	32	Intron_1.f	--	Yes	--	Yes	--	--	--	Yes	Yes	Yes	--
G4ST01182273175	chr1:182273176-182273215	+	GGGCGGCGGCGGCTGCCGGGGAGCAAGGGGCTGCGAGGGG<br>...((((....))))	GLT25D2	NM_015101	chr1:182171589-182273486	template	40	Exon_1.f	--	Yes	Yes	--	--	Yes	--	Yes	Yes	Yes	Yes
G4ST01182622610	chr1:182622611-182622642	-	GGGGCGCAGCGCGCTGGGGAGCGGGTTGGGGG<br>((((...))))..	C1orf21	NM_030806	chr1:182622773-182864778	template	-130	upstream	Yes	--	--	--	--	--	--	Yes	Yes	Yes	Yes
G4ST01182622689	chr1:182622690-182622724	+	GGGAGCTAGGCTCGGGGAGGGGCCGGCGGTGGGGG<br>(((...))).. .(((....)))	C1orf21	NM_030806	chr1:182622773-182864778	non-template	-48	upstream	Yes	--	--	--	--	--	--	Yes	Yes	Yes	--
G4ST01182622771	chr1:182622772-182622812	-	GGGAGCGAGCGGGAGCTTGCGAGGAGCGAGCGAGGGAGGGG<br>.((((((.....))))))..	C1orf21	NM_030806	chr1:182622773-182864778	template	40	upstream-Exon_1.f	Yes	Yes	Yes	--	--	Yes	--	Yes	Yes	Yes	Yes
G4ST01182622776	chr1:182622777-182622817	-	GGGCAGGGAGCGAGCGGGAGCTTGCGAGGAGCGAGCGAGGG<br>.((((((.....))))))..	C1orf21	NM_030806	chr1:182622773-182864778	template	41	Exon_1.f	--	Yes	Yes	--	--	Yes	--	Yes	Yes	Yes	Yes
G4ST01182849402	chr1:182849403-182849435	-	GGGAAGCAGTGCTGGGGTGTGGGGGTAGCAGGG<br>.(((...))).	C1orf21	NM_030806	chr1:182622773-182864778	template	33	Intron_5.l	--	Yes	--	Yes	--	--	--	--	--	--	--
G4ST01182990567	chr1:182990568-182990610	-	GGGATACTGAGGGCGGAGGGCGGTGGCAGCGCTGGCGCTGGGG<br>......(((((....)))))	EDEM3	NM_025191	chr1:182926248-182990664	non-template	43	Exon_1.f	--	Yes	Yes	--	--	Yes	--	Yes	Yes	Yes	--
G4ST01182990590	chr1:182990591-182990624	-	GGGGGAGGCGCTCCGGGATACTGAGGGCGGAGGG<br>((((...))))	EDEM3	NM_025191	chr1:182926248-182990664	non-template	34	Exon_1.f	--	Yes	Yes	--	--	Yes	--	Yes	Yes	Yes	--
G4ST01183036602	chr1:183036603-183036641	+	GGGGCCACTTTGGGGGTTGGGAGACTCTGTAGTCAAGGG<br>.((((....))))..	FAM129A	NM_052966	chr1:183026789-183210305	template	39	Intron_12	--	Yes	--	Yes	--	--	--	--	--	--	--
G4ST01183185876	chr1:183185877-183185908	-	GGGTGAATGCATTCATTTGGGTTGGGGTTGGG<br>..((((....)))).	FAM129A	NM_052966	chr1:183026789-183210305	non-template	32	Intron_1.f	--	Yes	--	Yes	--	--	--	Yes	Yes	--	Yes
G4ST01183281505	chr1:183281506-183281538	+	GGGGGGGGTGTGTGTCCGCGGGGGTGTGAAGGG<br>.((((....)))).	RNF2	NM_007212	chr1:183281174-183338363	non-template	33	Intron_1.f	--	Yes	--	Yes	--	--	--	--	Yes	Yes	--
G4ST01184184594	chr1:184184595-184184628	+	GGGCTGCTTCAAGCGGGATTAGGGGCGGCGTGGG<br>..(((...)))	HMCN1	NM_031935	chr1:183970306-184426708	non-template	34	Intron_11	--	Yes	--	Yes	--	--	--	--	--	--	--
G4ST01184543349	chr1:184543350-184543384	-	GGGGTGGTGGGTGCGAGCTTCTCGGGGGTGGTGGG<br>..((((...)))).	PRG4	NM_001127708	chr1:184532028-184550311	template	35	Exon_6	--	Yes	Yes	--	Yes	--	--	--	--	--	Yes
G4ST01184543349	chr1:184543350-184543384	-	GGGGTGGTGGGTGCGAGCTTCTCGGGGGTGGTGGG<br>..((((...)))).	PRG4	NM_001127709	chr1:184532028-184550311	template	35	Exon_5	--	Yes	Yes	--	Yes	--	--	--	--	--	Yes
G4ST01184543349	chr1:184543350-184543384	-	GGGGTGGTGGGTGCGAGCTTCTCGGGGGTGGTGGG<br>..((((...)))).	PRG4	NM_001127710	chr1:184532028-184550311	template	35	Exon_4	--	Yes	Yes	--	Yes	--	--	--	--	--	Yes
G4ST01184543349	chr1:184543350-184543384	-	GGGGTGGTGGGTGCGAGCTTCTCGGGGGTGGTGGG<br>..((((...)))).	PRG4	NM_005807	chr1:184532028-184550311	template	35	Exon_7	--	Yes	Yes	--	Yes	--	--	--	--	--	Yes
G4ST01184543356	chr1:184543357-184543394	-	GGGCTTCTCAGGGGTGGTGGGTGCGAGCTTCTCGGGGG<br>..((((...)))).	PRG4	NM_001127708	chr1:184532028-184550311	template	38	Exon_6	--	Yes	Yes	--	Yes	--	--	--	--	--	Yes
G4ST01184543356	chr1:184543357-184543394	-	GGGCTTCTCAGGGGTGGTGGGTGCGAGCTTCTCGGGGG<br>..((((...)))).	PRG4	NM_001127709	chr1:184532028-184550311	template	38	Exon_5	--	Yes	Yes	--	Yes	--	--	--	--	--	Yes
G4ST01184543356	chr1:184543357-184543394	-	GGGCTTCTCAGGGGTGGTGGGTGCGAGCTTCTCGGGGG<br>..((((...)))).	PRG4	NM_001127710	chr1:184532028-184550311	template	38	Exon_4	--	Yes	Yes	--	Yes	--	--	--	--	--	Yes
G4ST01184543356	chr1:184543357-184543394	-	GGGCTTCTCAGGGGTGGTGGGTGCGAGCTTCTCGGGGG<br>..((((...)))).	PRG4	NM_005807	chr1:184532028-184550311	template	38	Exon_7	--	Yes	Yes	--	Yes	--	--	--	--	--	Yes
G4ST01185118344	chr1:185118345-185118375	+	GGGAGAACCTTGGGTTTGATGGGGGGTTGGG<br>((...)).	PLA2G4A	NM_024420	chr1:185064655-185224736	non-template	31	Intron_3	--	Yes	--	Yes	--	--	--	Yes	--	--	Yes
G4ST01188438362	chr1:188438363-188438398	+	GGGAGGGACCTCATGGGGGATGATTGAATCATTGGG<br>..((((.....)))).	FAM5C	NM_199051	chr1:188333420-188713382	template	36	Intron_6	--	Yes	--	Yes	--	--	--	--	--	--	--
G4ST01188864028	chr1:188864029-188864059	-	GGGGTCAGGGGATGTGGACATGGGTAAGGGG<br>.(((....)))	LOC440704	NR_033922	chr1:188860643-189037411	template	31	Intron_1.f	--	Yes	--	Yes	--	--	--	--	--	--	--
G4ST01195811673	chr1:195811674-195811706	+	GGGCCTGTTGGCAGGTAGGGGCCTGGGGGAGGG<br>((((....))))...	DENND1B	NM_001195215	chr1:195740502-196011246	template	33	Intron_15	--	Yes	--	Yes	--	--	--	--	--	--	--
G4ST01195811673	chr1:195811674-195811706	+	GGGCCTGTTGGCAGGTAGGGGCCTGGGGGAGGG<br>((((....))))...	DENND1B	NM_144977	chr1:195788008-196011246	template	33	Intron_15.l	--	Yes	--	Yes	--	--	--	--	--	--	--
G4ST01196010759	chr1:196010760-196010791	-	GGGCGGCCGAGGTTGGCCGGGCGGGCTGGGGG<br>.(((((....)))))	DENND1B	NM_001195216	chr1:195969762-196011246	non-template	32	Intron_1.f	--	Yes	--	Yes	--	--	--	Yes	Yes	Yes	--
G4ST01196010759	chr1:196010760-196010791	-	GGGCGGCCGAGGTTGGCCGGGCGGGCTGGGGG<br>.(((((....)))))	DENND1B	NM_001195215	chr1:195740502-196011246	non-template	32	Intron_1.f	--	Yes	--	Yes	--	--	--	Yes	Yes	Yes	--
G4ST01196010759	chr1:196010760-196010791	-	GGGCGGCCGAGGTTGGCCGGGCGGGCTGGGGG<br>.(((((....)))))	DENND1B	NM_144977	chr1:195788008-196011246	non-template	32	Intron_1.f	--	Yes	--	Yes	--	--	--	Yes	Yes	Yes	--
G4ST01196138332	chr1:196138333-196138365	-	GGGGCCCGGAGGGGCGACCAACGCGGGCCTGGG<br>(((.....)))	C1orf53	NM_001024594	chr1:196138305-196143120	template	33	Exon_1.f	--	Yes	Yes	--	--	Yes	--	Yes	Yes	Yes	--
G4ST01196154972	chr1:196154973-196155007	+	GGGTCAGTGGGACCCAGAGGGAAGCATAACTTGGG<br>(((.....)))	LHX9	NM_020204	chr1:196153140-196165896	non-template	35	Intron_1.f	--	Yes	--	Yes	--	--	--	--	--	--	--

G4ST01196154972	chr1:196154973-196155007	+	GGGTCAGTGGGACCCAGAGGGAAGCATAACTTGGG<br>(((.....)))	LHX9	NM_001014434	chr1:196148258-196165896	non-template	35	Intron_2	--	Yes	--	Yes	--	--	--	--	--	--	--
G4ST01196455317	chr1:196455318-196455353	+	GGGAGGGATGTGAGGGATGAAAAACTACTTTTTGGG<br>...(((((....)))))	NEK7	NM_133494	chr1:196392731-196558171	non-template	36	Intron_1.f	--	Yes	--	Yes	--	--	--	--	--	--	Yes
G4ST01198270714	chr1:198270715-198270750	+	GGGCGTGGGCGGGTAGCCCAGCCCTCTGAGCCAGGG<br>..((.((((....))).))..	NR5A2	NM_205860	chr1:198263393-198413173	non-template	36	Intron_1.f	--	Yes	--	Yes	--	--	--	Yes	--	--	--
G4ST01198270714	chr1:198270715-198270750	+	GGGCGTGGGCGGGTAGCCCAGCCCTCTGAGCCAGGG<br>..((.((((....))).))..	NR5A2	NM_003822	chr1:198263393-198413173	non-template	36	Intron_1.f	--	Yes	--	Yes	--	--	--	Yes	--	--	--
G4ST01198275585	chr1:198275586-198275623	-	GGGAGGCTGAAGCCTGCAGGGCGAGCCCGGGGGTGGGG<br>((((....))))....	NR5A2	NM_205860	chr1:198263393-198413173	template	38	Intron_2	--	Yes	--	Yes	--	--	--	Yes	--	Yes	Yes
G4ST01198275585	chr1:198275586-198275623	-	GGGAGGCTGAAGCCTGCAGGGCGAGCCCGGGGGTGGGG<br>((((....))))....	NR5A2	NM_003822	chr1:198263393-198413173	template	38	Intron_1.f	--	Yes	--	Yes	--	--	--	Yes	--	Yes	Yes
G4ST01198275590	chr1:198275591-198275628	-	GGGGCGGGAGGCTGAAGCCTGCAGGGCGAGCCCGGGGG<br>((((....))))....	NR5A2	NM_205860	chr1:198263393-198413173	template	38	Intron_2	--	Yes	--	Yes	--	--	--	Yes	--	Yes	Yes
G4ST01198275590	chr1:198275591-198275628	-	GGGGCGGGAGGCTGAAGCCTGCAGGGCGAGCCCGGGGG<br>((((....))))....	NR5A2	NM_003822	chr1:198263393-198413173	template	38	Intron_1.f	--	Yes	--	Yes	--	--	--	Yes	--	Yes	Yes
G4ST01198410441	chr1:198410442-198410480	-	GGGCAGAGGTGCTGGGGCCTTCAAAGGAAGAGGGAGGGG<br>(((.....))) ..((((....)))).	NR5A2	NM_205860	chr1:198263393-198413173	template	39	Exon_8.l	--	Yes	Yes	--	--	--	Yes	Yes	--	--	--
G4ST01198410441	chr1:198410442-198410480	-	GGGCAGAGGTGCTGGGGCCTTCAAAGGAAGAGGGAGGGG<br>(((.....))) ..((((....)))).	NR5A2	NM_003822	chr1:198263393-198413173	template	39	Exon_7.l	--	Yes	Yes	--	--	--	Yes	Yes	--	--	--
G4ST01198645235	chr1:198645236-198645285	+	GGGGGAGGGGCGGCCGACCCCGCCGGCCGGGTGAAGCTGGTGACCGGGGG<br>..(((((.......))))) .....((((...))))	ZNF281	NM_012482	chr1:198642043-198645789	template	50	Exon_2.l	--	Yes	Yes	--	Yes	--	--	Yes	Yes	Yes	--
G4ST01198905765	chr1:198905766-198905794	+	GGGAGCCGGCAGTGGCTTGGGAGGGAGGG<br>(((((....))))).	DDX59	NM_001031725	chr1:198879788-198905749	template	-16	upstream	Yes	--	--	--	--	--	--	Yes	Yes	Yes	Yes
G4ST01198974517	chr1:198974518-198974543	+	GGGCGCGGGCGGGGAGCGAGGCTGGG<br>.(((...)))	CAMSAP1L1	NM_203459	chr1:198975309-199096454	non-template	-765	upstream	Yes	--	--	--	--	--	--	Yes	Yes	Yes	--
G4ST01198975781	chr1:198975782-198975807	-	GGGGAGGGAGGGATTCGAGAATCGGG<br>(((....))).	CAMSAP1L1	NM_203459	chr1:198975309-199096454	template	26	Intron_1.f	--	Yes	--	Yes	--	--	--	Yes	Yes	Yes	--
G4ST01199127469	chr1:199127470-199127502	+	GGGGCACCCAGTGCGGGTGAACTGGGGCGGGGG<br>((((...))))	C1orf106	NM_018265	chr1:199127262-199151487	non-template	33	Intron_1.f	--	Yes	--	Yes	--	--	--	--	Yes	Yes	Yes
G4ST01199135726	chr1:199135727-199135761	+	GGGATGAGGTGGGGTGGAGGGTGGTCCCACCAGGG<br>.(((...))).	C1orf106	NM_018265	chr1:199127262-199151487	non-template	35	Intron_3	--	Yes	--	Yes	--	--	--	Yes	Yes	--	--
G4ST01199135726	chr1:199135727-199135761	+	GGGATGAGGTGGGGTGGAGGGTGGTCCCACCAGGG<br>.(((...))).	C1orf106	NM_001142569	chr1:199130622-199151487	non-template	35	Intron_3	--	Yes	--	Yes	--	--	--	Yes	Yes	--	--
G4ST01199144725	chr1:199144726-199144756	+	GGGCAGGGAGTCGGTGGGAGCTATGCTGGGG<br>(((...))).	C1orf106	NM_018265	chr1:199127262-199151487	non-template	31	Intron_7	--	Yes	--	Yes	--	--	--	--	--	Yes	Yes
G4ST01199144725	chr1:199144726-199144756	+	GGGCAGGGAGTCGGTGGGAGCTATGCTGGGG<br>(((...))).	C1orf106	NM_001142569	chr1:199130622-199151487	non-template	31	Intron_7	--	Yes	--	Yes	--	--	--	--	--	Yes	Yes
G4ST01199147202	chr1:199147203-199147233	+	GGGTGGATTCCTTCCGGGCGGGTCCTGAGGG<br>.(((.....)))	C1orf106	NM_018265	chr1:199127262-199151487	non-template	31	Intron_8-Exon_9	--	Yes	Yes	Yes	Yes	--	--	--	--	--	--
G4ST01199147202	chr1:199147203-199147233	+	GGGTGGATTCCTTCCGGGCGGGTCCTGAGGG<br>.(((.....)))	C1orf106	NM_001142569	chr1:199130622-199151487	non-template	31	Intron_8-Exon_9	--	Yes	Yes	Yes	Yes	--	--	--	--	--	--
G4ST01199147666	chr1:199147667-199147700	-	GGGAGGGCGTCCGCACGATGCGGCTGCGGGAGGG<br>...(((((...)))))....	C1orf106	NM_018265	chr1:199127262-199151487	template	34	Exon_9	--	Yes	Yes	--	Yes	--	--	--	--	--	--
G4ST01199147666	chr1:199147667-199147700	-	GGGAGGGCGTCCGCACGATGCGGCTGCGGGAGGG<br>...(((((...)))))....	C1orf106	NM_001142569	chr1:199130622-199151487	template	34	Exon_9	--	Yes	Yes	--	Yes	--	--	--	--	--	--
G4ST01199205365	chr1:199205366-199205397	+	GGGCAGGGCAGGGCCCTGATTCATCTCAGGGG<br>..((((......))))	KIF21B	NM_017596	chr1:199205143-199259451	template	32	Exon_34.l	--	Yes	Yes	--	--	--	Yes	--	--	--	--
G4ST01199206129	chr1:199206130-199206154	-	GGGAAGGGACAGCGGTGGGTCTGGG<br>((....))	KIF21B	NM_017596	chr1:199205143-199259451	non-template	25	Exon_34.l	--	Yes	Yes	--	--	--	Yes	--	Yes	--	--
G4ST01199209091	chr1:199209092-199209122	+	GGGGAGGGATGAGGGAACAGTGTCCTGTGGG<br>.((((....))))	KIF21B	NM_017596	chr1:199205143-199259451	template	31	Exon_34.l	--	Yes	Yes	--	--	--	Yes	--	--	--	--
G4ST01199210059	chr1:199210060-199210104	+	GGGACAGAGGGCCTGGCTGCTCCCCACACAGGGGGCCAGAGCGGG<br>((((..((.....))..))))	KIF21B	NM_017596	chr1:199205143-199259451	template	45	Intron_33.l	--	Yes	--	Yes	--	--	--	--	--	--	Yes
G4ST01199210067	chr1:199210068-199210108	+	GGGCCTGGCTGCTCCCCACACAGGGGGCCAGAGCGGGTGGG<br>((((..((.....))..))))	KIF21B	NM_017596	chr1:199205143-199259451	template	41	Intron_33.l	--	Yes	--	Yes	--	--	--	--	--	--	Yes
G4ST01199210192	chr1:199210193-199210225	+	GGGGAGGGTAGGAAGGGCTTGGAGACAAGCGGG<br>((((....)))).	KIF21B	NM_017596	chr1:199205143-199259451	template	33	Intron_33.l	--	Yes	--	Yes	--	--	--	Yes	--	--	Yes
G4ST01199219015	chr1:199219016-199219054	+	GGGCTGGTCTGGGAGCTGGGACTCTCCCCCAGGAGTGGG<br>...((((....)))).	KIF21B	NM_017596	chr1:199205143-199259451	template	39	Intron_27	--	Yes	--	Yes	--	--	--	--	--	--	Yes
G4ST01199219025	chr1:199219026-199219063	+	GGGAGCTGGGACTCTCCCCCAGGAGTGGGCTGCCAGGG<br>...((((....)))).	KIF21B	NM_017596	chr1:199205143-199259451	template	38	Intron_27	--	Yes	--	Yes	--	--	--	--	--	--	Yes
G4ST01199239613	chr1:199239614-199239644	-	GGGCATTAAGGTAGTGGGGGAATGGGATGGG<br>((((.....)))).	KIF21B	NM_017596	chr1:199205143-199259451	non-template	31	Intron_7	--	Yes	--	Yes	--	--	--	--	--	--	--
G4ST01199260395	chr1:199260396-199260425	-	GGGAAGAGGCGTCTGGGCTCAGAGGGAGGG<br>.(((....)))	KIF21B	NM_017596	chr1:199205143-199259451	non-template	-944	upstream	Yes	--	--	--	--	--	--	Yes	--	--	--
G4ST01199260399	chr1:199260400-199260432	-	GGGGGCAGGGAAGAGGCGTCTGGGCTCAGAGGG<br>.(((....)))	KIF21B	NM_017596	chr1:199205143-199259451	non-template	-948	upstream	Yes	--	--	--	--	--	--	Yes	--	--	--
G4ST01199260408	chr1:199260409-199260440	-	GGGAGGCAGGGGGCAGGGAAGAGGCGTCTGGG<br>.(((....)))	KIF21B	NM_017596	chr1:199205143-199259451	non-template	-957	upstream	Yes	--	--	--	--	--	--	Yes	--	--	--
G4ST01199289504	chr1:199289505-199289535	+	GGGCACAGGGCTCTGGGGCTGTTCAGCTGGG<br>(((....))).	CACNA1S	NM_000069	chr1:199275263-199348317	template	31	Intron_29	--	Yes	--	Yes	--	--	--	--	--	--	--
G4ST01199289511	chr1:199289512-199289543	+	GGGCTCTGGGGCTGTTCAGCTGGGAGGCAGGG<br>(((....))).	CACNA1S	NM_000069	chr1:199275263-199348317	template	32	Intron_29	--	Yes	--	Yes	--	--	--	--	--	--	--
G4ST01199301732	chr1:199301733-199301769	-	GGGGTTTCCTCAACTGGGCTAGCAGGGTTACTGTGGG<br>(((.....))).	CACNA1S	NM_000069	chr1:199275263-199348317	non-template	37	Intron_21	--	Yes	--	Yes	--	--	--	--	--	--	--
G4ST01199302488	chr1:199302489-199302530	-	GGGTCTCTCGGGGCCTCCGATGGAGGCAGGGGGTCCTGTGGG<br>((((((...))))))....	CACNA1S	NM_000069	chr1:199275263-199348317	non-template	42	Intron_20	--	Yes	--	Yes	--	--	--	--	--	--	--
G4ST01199303747	chr1:199303748-199303785	-	GGGGTTCCAGCCATCCTGGGGGTGGTGGGCTCTGGGGG<br>...((((.....))))	CACNA1S	NM_000069	chr1:199275263-199348317	non-template	38	Intron_19	--	Yes	--	Yes	--	--	--	--	--	--	--
G4ST01199336460	chr1:199336461-199336499	+	GGGGGCTGGGAGAGAAAGGGAGACCAGTGCTGTCTTGGG<br>((((.......)))).	CACNA1S	NM_000069	chr1:199275263-199348317	template	39	Intron_2	--	Yes	--	Yes	--	--	--	--	--	--	Yes
G4ST01199342454	chr1:199342455-199342488	-	GGGCACAGGATGTCGGGCTGTGGGATGGTGAGGG<br>.(((...))).	CACNA1S	NM_000069	chr1:199275263-199348317	non-template	34	Intron_2	--	Yes	--	Yes	--	--	--	--	Yes	--	Yes
G4ST01199346429	chr1:199346430-199346453	+	GGGAGGGTAAGGGTTGATGCAGGG<br>.((...))	CACNA1S	NM_000069	chr1:199275263-199348317	template	24	Intron_1.f	--	Yes	--	Yes	--	--	--	--	Yes	--	--
G4ST01199348346	chr1:199348347-199348380	-	GGGGACAGATTGGGAAGTCACTGGCTGGGCTGGG<br>.(((.....)))	CACNA1S	NM_000069	chr1:199275263-199348317	non-template	-29	upstream	Yes	--	--	--	--	--	--	--	--	--	--
G4ST01199350304	chr1:199350305-199350337	-	GGGGCGGAAGTGCAGGGCAGCGGGTACGATGGG<br>(((....))).	CACNA1S	NM_000069	chr1:199275263-199348317	non-template	-1987	upstream	Yes	--	--	--	--	--	--	Yes	Yes	Yes	--
G4ST01199448201	chr1:199448202-199448241	+	GGGATAATGGGGGCTTCTGGGTTTCTTGATGGCAAGGGGG<br>...((((....)))).	IGFN1	NM_001164586	chr1:199426576-199464703	non-template	40	Exon_12	--	Yes	Yes	--	Yes	--	--	--	Yes	--	--
G4ST01199452519	chr1:199452520-199452547	+	GGGGCTGGGGGTGGGGGACACTCCTGGG<br>(((...))).	IGFN1	NM_001164586	chr1:199426576-199464703	non-template	28	Intron_16	--	Yes	--	Yes	--	--	--	--	--	--	--
G4ST01199538503	chr1:199538504-199538528	-	GGGCACGCTGTGGGGGCGGGAGGGG<br>(((...))).	PKP1	NM_000299	chr1:199519203-199568744	template	25	Intron_2	--	Yes	--	Yes	--	--	--	Yes	Yes	--	Yes
G4ST01199538503	chr1:199538504-199538528	-	GGGCACGCTGTGGGGGCGGGAGGGG<br>(((...))).	PKP1	NM_001005337	chr1:199519203-199568744	template	25	Intron_2	--	Yes	--	Yes	--	--	--	Yes	Yes	--	Yes
G4ST01199540813	chr1:199540814-199540849	+	GGGCATGGGATGTGTGTCCCACGTGGGCAGGTGGGG<br>.((((.....)))).	PKP1	NM_000299	chr1:199519203-199568744	non-template	36	Intron_2	--	Yes	--	Yes	--	--	--	--	--	--	--

G4ST01199540813	chr1:199540814-199540849	+	GGGCATGGGATGTGTGTCCCACGTGGGCAGGTGGGG<br>.((((.....)))).	PKP1	NM_001005337	chr1:199519203-199568744	non-template	36	Intron_2	--	Yes	--	Yes	--	--	--	--	--	--	--
G4ST01199544598	chr1:199544599-199544628	+	GGGACCAGAGCCCTGGGGGCTGGGCAGGGG<br>.((((....))))	PKP1	NM_000299	chr1:199519203-199568744	non-template	30	Intron_2	--	Yes	--	Yes	--	--	--	--	Yes	--	Yes
G4ST01199544598	chr1:199544599-199544628	+	GGGACCAGAGCCCTGGGGGCTGGGCAGGGG<br>.((((....))))	PKP1	NM_001005337	chr1:199519203-199568744	non-template	30	Intron_2	--	Yes	--	Yes	--	--	--	--	Yes	--	Yes
G4ST01199559005	chr1:199559006-199559049	-	GGGAGGCTCCGAAGTCAGGGGACAGGCTGGGGGGAAAAGGAGGG<br>.((((....))))..	PKP1	NM_000299	chr1:199519203-199568744	template	44	Intron_10	--	Yes	--	Yes	--	--	--	--	--	--	--
G4ST01199559005	chr1:199559006-199559049	-	GGGAGGCTCCGAAGTCAGGGGACAGGCTGGGGGGAAAAGGAGGG<br>.((((....))))..	PKP1	NM_001005337	chr1:199519203-199568744	template	44	Intron_9	--	Yes	--	Yes	--	--	--	--	--	--	--
G4ST01199560630	chr1:199560631-199560665	-	GGGTCAGGATGACTGGGGAAGGAAGGGCTGAGGGG<br>.(((.....)))	PKP1	NM_000299	chr1:199519203-199568744	template	35	Intron_11	--	Yes	--	Yes	--	--	--	--	--	--	--
G4ST01199560630	chr1:199560631-199560665	-	GGGTCAGGATGACTGGGGAAGGAAGGGCTGAGGGG<br>.(((.....)))	PKP1	NM_001005337	chr1:199519203-199568744	template	35	Intron_10	--	Yes	--	Yes	--	--	--	--	--	--	--
G4ST01199560638	chr1:199560639-199560673	-	GGGGCACAGGGTCAGGATGACTGGGGAAGGAAGGG<br>.(((.....)))	PKP1	NM_000299	chr1:199519203-199568744	template	35	Intron_11	--	Yes	--	Yes	--	--	--	--	--	--	--
G4ST01199560638	chr1:199560639-199560673	-	GGGGCACAGGGTCAGGATGACTGGGGAAGGAAGGG<br>.(((.....)))	PKP1	NM_001005337	chr1:199519203-199568744	template	35	Intron_10	--	Yes	--	Yes	--	--	--	--	--	--	--
G4ST01199566909	chr1:199566910-199566954	+	GGGAGTGTTTGGGGTCCCAGGAGACTTGGACGGGGGGAGTTTGGG<br>((((.((....)).))))...	PKP1	NM_000299	chr1:199519203-199568744	non-template	45	Exon_15.l	--	Yes	Yes	--	--	--	Yes	--	--	--	--
G4ST01199566909	chr1:199566910-199566954	+	GGGAGTGTTTGGGGTCCCAGGAGACTTGGACGGGGGGAGTTTGGG<br>((((.((....)).))))...	PKP1	NM_001005337	chr1:199519203-199568744	non-template	45	Exon_14.l	--	Yes	Yes	--	--	--	Yes	--	--	--	--
G4ST01199568003	chr1:199568004-199568043	-	GGGGCTGGGCCCATGGGGCTCTTCATTCTTTGAAGGTGGG<br>...(((((.....)))))..	PKP1	NM_000299	chr1:199519203-199568744	template	40	Exon_15.l	--	Yes	Yes	--	--	--	Yes	--	--	--	Yes
G4ST01199568003	chr1:199568004-199568043	-	GGGGCTGGGCCCATGGGGCTCTTCATTCTTTGAAGGTGGG<br>...(((((.....)))))..	PKP1	NM_001005337	chr1:199519203-199568744	template	40	Exon_14.l	--	Yes	Yes	--	--	--	Yes	--	--	--	Yes
G4ST01199597603	chr1:199597604-199597639	-	GGGGCACTGCCCGCATCTGCTCTGGGAGGGGTCGGG<br>(((.((....))..)))...	TNNT2	NM_000364	chr1:199594765-199613428	non-template	36	Intron_13	--	Yes	--	Yes	--	--	--	Yes	Yes	--	Yes
G4ST01199597603	chr1:199597604-199597639	-	GGGGCACTGCCCGCATCTGCTCTGGGAGGGGTCGGG<br>(((.((....))..)))...	TNNT2	NM_001001432	chr1:199594765-199613428	non-template	36	Intron_11	--	Yes	--	Yes	--	--	--	Yes	Yes	--	Yes
G4ST01199597603	chr1:199597604-199597639	-	GGGGCACTGCCCGCATCTGCTCTGGGAGGGGTCGGG<br>(((.((....))..)))...	TNNT2	NM_001001431	chr1:199594765-199613428	non-template	36	Intron_12	--	Yes	--	Yes	--	--	--	Yes	Yes	--	Yes
G4ST01199597603	chr1:199597604-199597639	-	GGGGCACTGCCCGCATCTGCTCTGGGAGGGGTCGGG<br>(((.((....))..)))...	TNNT2	NM_001001430	chr1:199594765-199613428	non-template	36	Intron_13	--	Yes	--	Yes	--	--	--	Yes	Yes	--	Yes
G4ST01199597608	chr1:199597609-199597654	-	GGGAGGAATGGTCCTGGGGCACTGCCCGCATCTGCTCTGGGAGGGG<br>((((....)))). (((.((....))..)))...	TNNT2	NM_000364	chr1:199594765-199613428	non-template	46	Intron_13	--	Yes	--	Yes	--	--	--	Yes	Yes	--	Yes
G4ST01199597608	chr1:199597609-199597654	-	GGGAGGAATGGTCCTGGGGCACTGCCCGCATCTGCTCTGGGAGGGG<br>((((....)))). (((.((....))..)))...	TNNT2	NM_001001432	chr1:199594765-199613428	non-template	46	Intron_11	--	Yes	--	Yes	--	--	--	Yes	Yes	--	Yes
G4ST01199597608	chr1:199597609-199597654	-	GGGAGGAATGGTCCTGGGGCACTGCCCGCATCTGCTCTGGGAGGGG<br>((((....)))). (((.((....))..)))...	TNNT2	NM_001001431	chr1:199594765-199613428	non-template	46	Intron_12	--	Yes	--	Yes	--	--	--	Yes	Yes	--	Yes
G4ST01199597608	chr1:199597609-199597654	-	GGGAGGAATGGTCCTGGGGCACTGCCCGCATCTGCTCTGGGAGGGG<br>((((....)))). (((.((....))..)))...	TNNT2	NM_001001430	chr1:199594765-199613428	non-template	46	Intron_13	--	Yes	--	Yes	--	--	--	Yes	Yes	--	Yes
G4ST01199597613	chr1:199597614-199597661	-	GGGGGCTGGGAGGAATGGTCCTGGGGCACTGCCCGCATCTGCTCTGGG<br>((((....)))). (((.((....))..)))...	TNNT2	NM_000364	chr1:199594765-199613428	non-template	48	Intron_13	--	Yes	--	Yes	--	--	--	Yes	Yes	--	Yes
G4ST01199597613	chr1:199597614-199597661	-	GGGGGCTGGGAGGAATGGTCCTGGGGCACTGCCCGCATCTGCTCTGGG<br>((((....)))). (((.((....))..)))...	TNNT2	NM_001001432	chr1:199594765-199613428	non-template	48	Intron_11	--	Yes	--	Yes	--	--	--	Yes	Yes	--	Yes
G4ST01199597613	chr1:199597614-199597661	-	GGGGGCTGGGAGGAATGGTCCTGGGGCACTGCCCGCATCTGCTCTGGG<br>((((....)))). (((.((....))..)))...	TNNT2	NM_001001431	chr1:199594765-199613428	non-template	48	Intron_12	--	Yes	--	Yes	--	--	--	Yes	Yes	--	Yes
G4ST01199597613	chr1:199597614-199597661	-	GGGGGCTGGGAGGAATGGTCCTGGGGCACTGCCCGCATCTGCTCTGGG<br>((((....)))). (((.((....))..)))...	TNNT2	NM_001001430	chr1:199594765-199613428	non-template	48	Intron_13	--	Yes	--	Yes	--	--	--	Yes	Yes	--	Yes
G4ST01199598817	chr1:199598818-199598857	-	GGGGCTTGCTGGGGTAGGGGGCTACAGGCAGAGCCTGGGG<br>.....(((((...)))))	TNNT2	NM_000364	chr1:199594765-199613428	non-template	40	Intron_12	--	Yes	--	Yes	--	--	--	--	--	--	--
G4ST01199598817	chr1:199598818-199598857	-	GGGGCTTGCTGGGGTAGGGGGCTACAGGCAGAGCCTGGGG<br>.....(((((...)))))	TNNT2	NM_001001432	chr1:199594765-199613428	non-template	40	Intron_10	--	Yes	--	Yes	--	--	--	--	--	--	--
G4ST01199598817	chr1:199598818-199598857	-	GGGGCTTGCTGGGGTAGGGGGCTACAGGCAGAGCCTGGGG<br>.....(((((...)))))	TNNT2	NM_001001431	chr1:199594765-199613428	non-template	40	Intron_11	--	Yes	--	Yes	--	--	--	--	--	--	--
G4ST01199598817	chr1:199598818-199598857	-	GGGGCTTGCTGGGGTAGGGGGCTACAGGCAGAGCCTGGGG<br>.....(((((...)))))	TNNT2	NM_001001430	chr1:199594765-199613428	non-template	40	Intron_11	--	Yes	--	Yes	--	--	--	--	--	--	--
G4ST01199604167	chr1:199604168-199604201	-	GGGAGGTTGCCGGGATGAGTGGGGCTAAGCTGGG<br>.((...))	TNNT2	NM_000364	chr1:199594765-199613428	non-template	34	Intron_5	--	Yes	--	Yes	--	--	--	--	--	--	Yes
G4ST01199604167	chr1:199604168-199604201	-	GGGAGGTTGCCGGGATGAGTGGGGCTAAGCTGGG<br>.((...))	TNNT2	NM_001001432	chr1:199594765-199613428	non-template	34	Intron_3	--	Yes	--	Yes	--	--	--	--	--	--	Yes
G4ST01199604167	chr1:199604168-199604201	-	GGGAGGTTGCCGGGATGAGTGGGGCTAAGCTGGG<br>.((...))	TNNT2	NM_001001431	chr1:199594765-199613428	non-template	34	Intron_4	--	Yes	--	Yes	--	--	--	--	--	--	Yes
G4ST01199604167	chr1:199604168-199604201	-	GGGAGGTTGCCGGGATGAGTGGGGCTAAGCTGGG<br>.((...))	TNNT2	NM_001001430	chr1:199594765-199613428	non-template	34	Intron_4	--	Yes	--	Yes	--	--	--	--	--	--	Yes
G4ST01199604896	chr1:199604897-199604928	-	GGGATGTTGCATGGCCACTGGGTGGGCTGGGG<br>..((.((...)).)).	TNNT2	NM_000364	chr1:199594765-199613428	non-template	32	Intron_5	--	Yes	--	Yes	--	--	--	--	--	--	--
G4ST01199604896	chr1:199604897-199604928	-	GGGATGTTGCATGGCCACTGGGTGGGCTGGGG<br>..((.((...)).)).	TNNT2	NM_001001432	chr1:199594765-199613428	non-template	32	Intron_3	--	Yes	--	Yes	--	--	--	--	--	--	--
G4ST01199604896	chr1:199604897-199604928	-	GGGATGTTGCATGGCCACTGGGTGGGCTGGGG<br>..((.((...)).)).	TNNT2	NM_001001431	chr1:199594765-199613428	non-template	32	Intron_4	--	Yes	--	Yes	--	--	--	--	--	--	--
G4ST01199604896	chr1:199604897-199604928	-	GGGATGTTGCATGGCCACTGGGTGGGCTGGGG<br>..((.((...)).)).	TNNT2	NM_001001430	chr1:199594765-199613428	non-template	32	Intron_4	--	Yes	--	Yes	--	--	--	--	--	--	--
G4ST01199612704	chr1:199612705-199612736	+	GGGTCTGGGAGCTCAGCTCAGCTCTGGGAGGG<br>((((......))))..	TNNT2	NM_000364	chr1:199594765-199613428	template	32	Intron_1.f	--	Yes	--	Yes	--	--	--	Yes	Yes	--	--
G4ST01199612704	chr1:199612705-199612736	+	GGGTCTGGGAGCTCAGCTCAGCTCTGGGAGGG<br>((((......))))..	TNNT2	NM_001001432	chr1:199594765-199613428	template	32	Intron_1.f	--	Yes	--	Yes	--	--	--	Yes	Yes	--	--
G4ST01199612704	chr1:199612705-199612736	+	GGGTCTGGGAGCTCAGCTCAGCTCTGGGAGGG<br>((((......))))..	TNNT2	NM_001001431	chr1:199594765-199613428	template	32	Intron_1.f	--	Yes	--	Yes	--	--	--	Yes	Yes	--	--
G4ST01199612704	chr1:199612705-199612736	+	GGGTCTGGGAGCTCAGCTCAGCTCTGGGAGGG<br>((((......))))..	TNNT2	NM_001001430	chr1:199594765-199613428	template	32	Intron_1.f	--	Yes	--	Yes	--	--	--	Yes	Yes	--	--
G4ST01199625056	chr1:199625057-199625090	+	GGGGGAGGGGAGGAGAGGGTCAGCACAGCTGGGG<br>.((((...))))	LAD1	NM_005558	chr1:199616589-199635292	template	34	Intron_1.f	--	Yes	--	Yes	--	--	--	--	--	--	Yes
G4ST01199636019	chr1:199636020-199636058	+	GGGCTTGGGCTAAGAGAGGGTCAGGCGTTGAGCATGGGG<br>((...)). .((.((.....)).))	LAD1	NM_005558	chr1:199616589-199635292	template	-727	upstream	Yes	--	--	--	--	--	--	Yes	--	--	--
G4ST01199641474	chr1:199641475-199641505	+	GGGGACCGGGCACCCTGTGGGGAAAGGTGGG<br>(((...)))	TNNI1	NM_003281	chr1:199639518-199657497	template	31	Exon_8.l	--	Yes	Yes	--	--	--	Yes	Yes	Yes	Yes	--
G4ST01199650891	chr1:199650892-199650942	-	GGGGGGATGCCCAGTGGGAGGCAGAGGGGCAGCAGAGCAGACTCTGAAGGG<br>(((...)))... ....(((((....)))))..	TNNI1	NM_003281	chr1:199639518-199657497	non-template	51	Intron_3	--	Yes	--	Yes	--	--	--	--	--	--	--
G4ST01199703454	chr1:199703455-199703495	-	GGGGCCTCAGTGGGCCGCCTTGGCTGGACTGGGCCTGAGGG<br>((((....)).))...	PHLDA3	NM_012396	chr1:199701245-199704922	non-template	41	Intron_1.u	--	Yes	--	Yes	--	--	--	Yes	--	--	--
G4ST01199724716	chr1:199724717-199724757	+	GGGCCTGTGGCCAGGGGCTCTGCATGGAGAAGGGCATGGGG<br>.(((....))) .((((....))))..	CSRP1	NM_001193572	chr1:199719281-199732324	template	41	Intron_3-Exon_4	--	Yes	Yes	Yes	Yes	--	--	--	Yes	--	--
G4ST01199724716	chr1:199724717-199724757	+	GGGCCTGTGGCCAGGGGCTCTGCATGGAGAAGGGCATGGGG<br>.(((....))) .((((....))))..	CSRP1	NM_001193571	chr1:199719281-199742590	template	41	Intron_3-Exon_4	--	Yes	Yes	Yes	Yes	--	--	--	Yes	--	--
G4ST01199724716	chr1:199724717-199724757	+	GGGCCTGTGGCCAGGGGCTCTGCATGGAGAAGGGCATGGGG<br>.(((....))) .((((....))))..	CSRP1	NM_001193570	chr1:199719281-199743010	template	41	Intron_3-Exon_4	--	Yes	Yes	Yes	Yes	--	--	--	Yes	--	--
G4ST01199724716	chr1:199724717-199724757	+	GGGCCTGTGGCCAGGGGCTCTGCATGGAGAAGGGCATGGGG<br>.(((....))) .((((....))))..	CSRP1	NM_004078	chr1:199719281-199743010	template	41	Intron_3-Exon_4	--	Yes	Yes	Yes	Yes	--	--	--	Yes	--	--

G4ST01199883475	chr1:199883476-199883513	+	GGGGGAAAACCAAAGACGGTTTGGGGTTGGGGGAGGGG<br>...(((((......))))).	NAV1	NM_020443	chr1:199884073-200062725	non-template	-559	upstream	Yes	--	--	--	--	--	--	--	Yes	--	--
G4ST01199899544	chr1:199899545-199899572	+	GGGTGGGATGTTCCATGGGGGAGATGGG<br>(((...)))..	NAV1	NM_020443	chr1:199884073-200062725	non-template	28	Intron_1.f	--	Yes	--	Yes	--	--	--	--	--	--	--
G4ST01199905478	chr1:199905479-199905518	+	GGGTAGGGAAACATCATTGCAGATGTGAGGGTTGGAGGGG<br>..(((((......)))))..	NAV1	NM_020443	chr1:199884073-200062725	non-template	40	Intron_1.f	--	Yes	--	Yes	--	--	--	--	--	--	--
G4ST01199910844	chr1:199910845-199910875	-	GGGGCCTGATGGGAAGTGCTTGGGTCATGGG<br>((....))	NAV1	NM_020443	chr1:199884073-200062725	template	31	Intron_1.f	--	Yes	--	Yes	--	--	--	--	--	--	--
G4ST01199939860	chr1:199939861-199939897	-	GGGGAGCCCACCCGGTTCTTGGGGAGAGGGAGCAGGG<br>(((((.....)))))...	NAV1	NM_020443	chr1:199884073-200062725	template	37	Intron_1.f	--	Yes	--	Yes	--	--	--	--	Yes	--	--
G4ST01199956218	chr1:199956219-199956248	-	GGGTTGGTGGGGTGAAGGGCTCTCAGAGGG<br>((...)).	NAV1	NM_020443	chr1:199884073-200062725	template	30	Intron_3	--	Yes	--	Yes	--	--	--	--	--	--	--
G4ST01199975692	chr1:199975693-199975738	+	GGGTGTCCCCGGGTGTCAGGCGAGAGCGGTCCTGGGGGGCGCTGGG<br>...((((((....))..))))..	NAV1	NM_020443	chr1:199884073-200062725	non-template	46	Intron_3	--	Yes	--	Yes	--	--	--	Yes	Yes	Yes	--
G4ST01199975692	chr1:199975693-199975738	+	GGGTGTCCCCGGGTGTCAGGCGAGAGCGGTCCTGGGGGGCGCTGGG<br>...((((((....))..))))..	NAV1	NM_001167738	chr1:199975586-200062725	non-template	46	Exon_1.f	--	Yes	Yes	--	--	Yes	--	Yes	Yes	Yes	--
G4ST01200024587	chr1:200024588-200024626	+	GGGGCATTGGGATGGAGCATCCATCACTGGGGCTGGGGG<br>(((((...)))))).....	NAV1	NM_020443	chr1:199884073-200062725	non-template	39	Intron_10	--	Yes	--	Yes	--	--	--	--	--	--	--
G4ST01200024587	chr1:200024588-200024626	+	GGGGCATTGGGATGGAGCATCCATCACTGGGGCTGGGGG<br>(((((...)))))).....	NAV1	NM_001167738	chr1:199975586-200062725	non-template	39	Intron_8	--	Yes	--	Yes	--	--	--	--	--	--	--
G4ST01200024595	chr1:200024596-200024630	+	GGGATGGAGCATCCATCACTGGGGCTGGGGGAGGG<br>(((((...)))))).....	NAV1	NM_020443	chr1:199884073-200062725	non-template	35	Intron_10	--	Yes	--	Yes	--	--	--	--	--	--	--
G4ST01200024595	chr1:200024596-200024630	+	GGGATGGAGCATCCATCACTGGGGCTGGGGGAGGG<br>(((((...)))))).....	NAV1	NM_001167738	chr1:199975586-200062725	non-template	35	Intron_8	--	Yes	--	Yes	--	--	--	--	--	--	--
G4ST01200048159	chr1:200048160-200048201	-	GGGGAAAGTGGGGTCAGAGTGGCAATGGGGACATTGTCAGGG<br>((((...)))).... (((...))).	NAV1	NM_020443	chr1:199884073-200062725	template	42	Intron_26	--	Yes	--	Yes	--	--	--	--	--	--	--
G4ST01200048159	chr1:200048160-200048201	-	GGGGAAAGTGGGGTCAGAGTGGCAATGGGGACATTGTCAGGG<br>((((...)))).... (((...))).	NAV1	NM_001167738	chr1:199975586-200062725	template	42	Intron_23	--	Yes	--	Yes	--	--	--	--	--	--	--
G4ST01200122996	chr1:200122997-200123032	+	GGGGTCAAGGGTACACATGGGAGATTCAGCTCTGGG<br>(((......)))	SHISA4	NR_030775	chr1:200124420-200128338	non-template	-1387	upstream	Yes	--	--	--	--	--	--	Yes	Yes	--	--
G4ST01200122996	chr1:200122997-200123032	+	GGGGTCAAGGGTACACATGGGAGATTCAGCTCTGGG<br>(((......)))	SHISA4	NM_198149	chr1:200124428-200128338	non-template	-1395	upstream	Yes	--	--	--	--	--	--	Yes	Yes	--	--
G4ST01200124471	chr1:200124472-200124505	+	GGGGACAGGGGGAGCAGGGCAGTGCTCTGCTGGG<br>(((....)))..	SHISA4	NR_030775	chr1:200124420-200128338	non-template	34	Exon_1.f	--	Yes	Yes	--	--	--	--	Yes	Yes	Yes	--
G4ST01200124471	chr1:200124472-200124505	+	GGGGACAGGGGGAGCAGGGCAGTGCTCTGCTGGG<br>(((....)))..	SHISA4	NM_198149	chr1:200124428-200128338	non-template	34	Exon_1.f	--	Yes	Yes	--	--	Yes	--	Yes	Yes	Yes	--
G4ST01200124478	chr1:200124479-200124512	+	GGGGGAGCAGGGCAGTGCTCTGCTGGGTGAGGGG<br>(((....)))..	SHISA4	NR_030775	chr1:200124420-200128338	non-template	34	Exon_1.f	--	Yes	Yes	--	--	--	--	Yes	Yes	Yes	--
G4ST01200124478	chr1:200124479-200124512	+	GGGGGAGCAGGGCAGTGCTCTGCTGGGTGAGGGG<br>(((....)))..	SHISA4	NM_198149	chr1:200124428-200128338	non-template	34	Exon_1.f	--	Yes	Yes	--	--	Yes	--	Yes	Yes	Yes	--
G4ST01200124701	chr1:200124702-200124735	-	GGGCGCGGCCGGGGTCGGGCCTCCCAGAGAAGGG<br>.(((...)))..	SHISA4	NR_030775	chr1:200124420-200128338	template	34	Intron_1.f	--	Yes	--	Yes	--	--	--	Yes	--	Yes	Yes
G4ST01200124701	chr1:200124702-200124735	-	GGGCGCGGCCGGGGTCGGGCCTCCCAGAGAAGGG<br>.(((...)))..	SHISA4	NM_198149	chr1:200124428-200128338	template	34	Exon_1.f	--	Yes	Yes	--	--	Yes	--	Yes	--	Yes	Yes
G4ST01200125095	chr1:200125096-200125132	+	GGGAGCCGTCAGGCTGGGGACCGCCGGGGGAGTGGGG<br>((((....)))).	SHISA4	NR_030775	chr1:200124420-200128338	non-template	37	Intron_1.f	--	Yes	--	Yes	--	--	--	Yes	--	--	--
G4ST01200125095	chr1:200125096-200125132	+	GGGAGCCGTCAGGCTGGGGACCGCCGGGGGAGTGGGG<br>((((....)))).	SHISA4	NM_198149	chr1:200124428-200128338	non-template	37	Intron_1.f	--	Yes	--	Yes	--	--	--	Yes	--	--	--
G4ST01200125110	chr1:200125111-200125148	+	GGGGACCGCCGGGGGAGTGGGGCCGAGCGCGGCTGGGG<br>((((....))))..	SHISA4	NR_030775	chr1:200124420-200128338	non-template	38	Intron_1.f	--	Yes	--	Yes	--	--	--	Yes	--	--	--
G4ST01200125110	chr1:200125111-200125148	+	GGGGACCGCCGGGGGAGTGGGGCCGAGCGCGGCTGGGG<br>((((....))))..	SHISA4	NM_198149	chr1:200124428-200128338	non-template	38	Intron_1.f	--	Yes	--	Yes	--	--	--	Yes	--	--	--
G4ST01200134450	chr1:200134451-200134486	+	GGGGAGGCAGGGCTTGGAGAAGTAGGGTGTCCAGGG<br>(((....)))..	LMOD1	NM_012134	chr1:200132207-200182339	template	36	Intron_2.l	--	Yes	--	Yes	--	--	--	--	--	--	--
G4ST01200145925	chr1:200145926-200145960	+	GGGAGGAGCCCTTTTGGGCTGCTGGGAGCCAGGGG<br>(((...)))...	LMOD1	NM_012134	chr1:200132207-200182339	template	35	Intron_1.f	--	Yes	--	Yes	--	--	--	--	--	--	--
G4ST01200148847	chr1:200148848-200148881	+	GGGCCAGAGGTGGGAAGCTGGGACCAGGCGGGGG<br>((...)).	LMOD1	NM_012134	chr1:200132207-200182339	template	34	Intron_1.f	--	Yes	--	Yes	--	--	--	Yes	--	--	--
G4ST01200191466	chr1:200191467-200191510	-	GGGAGAGGGAGGCTCACGCGGCCGGGGAGGTACTAAGTACAGGG<br>.((((.....)))). ...((((...)))).	TIMM17A	NM_006335	chr1:200191242-200206412	template	44	Intron_1.f	--	Yes	--	Yes	--	--	--	Yes	Yes	Yes	Yes
G4ST01200191483	chr1:200191484-200191515	-	GGGTTGGGAGAGGGAGGCTCACGCGGCCGGGG<br>.((((.....)))).	TIMM17A	NM_006335	chr1:200191242-200206412	template	32	Intron_1.f	--	Yes	--	Yes	--	--	--	Yes	Yes	Yes	Yes
G4ST01200218320	chr1:200218321-200218349	+	GGGGGCGGGGCCCGCGCGGCGGGGGCGGG<br>..((((...))))	RNPEP	NM_020216	chr1:200218389-200241898	non-template	-39	upstream	Yes	--	--	--	--	--	--	Yes	Yes	Yes	--
G4ST01200248442	chr1:200248443-200248484	-	GGGGAGGGGGCTCCTGCGCCACAGCTGCCGGGGTGGTAGGGG<br>(((..(((.....)))..))).	ELF3	NM_001114309	chr1:200246313-200252938	template	42	Exon_5	--	Yes	Yes	--	Yes	--	--	--	Yes	--	--
G4ST01200248442	chr1:200248443-200248484	-	GGGGAGGGGGCTCCTGCGCCACAGCTGCCGGGGTGGTAGGGG<br>(((..(((.....)))..))).	ELF3	NM_004433	chr1:200246313-200252938	template	42	Exon_5	--	Yes	Yes	--	Yes	--	--	--	Yes	--	--
G4ST01200358764	chr1:200358765-200358806	+	GGGGCTAAGCAGGGTCTCTGGGGGTGCCCCCCTGCACCTGGG<br>(((((......))))).	GPR37L1	NM_004767	chr1:200358652-200365257	non-template	42	Exon_1.f	--	Yes	Yes	--	Yes	--	--	--	Yes	--	--
G4ST01200369929	chr1:200369930-200369960	+	GGGGCGGGGGCTGTGGCCCAGGGCTGCTGGG<br>(((....)))..	ARL8A	NM_138795	chr1:200368600-200380489	template	31	Exon_7.l	--	Yes	Yes	--	--	--	Yes	--	--	--	Yes
G4ST01200369934	chr1:200369935-200369964	+	GGGGGCTGTGGCCCAGGGCTGCTGGGAGGG<br>(((....)))..	ARL8A	NM_138795	chr1:200368600-200380489	template	30	Exon_7.l	--	Yes	Yes	--	--	--	Yes	--	--	--	Yes
G4ST01200371401	chr1:200371402-200371432	-	GGGGAGGGAGCTCGGCTGGGAAGTCACTGGG<br>(((...))) .((...))	ARL8A	NM_138795	chr1:200368600-200380489	non-template	31	Intron_4	--	Yes	--	Yes	--	--	--	Yes	Yes	--	Yes
G4ST01200371412	chr1:200371413-200371437	-	GGGATGGGGAGGGAGCTCGGCTGGG<br>(((...)))	ARL8A	NM_138795	chr1:200368600-200380489	non-template	25	Intron_4	--	Yes	--	Yes	--	--	--	Yes	Yes	--	Yes
G4ST01200379434	chr1:200379435-200379468	-	GGGGCCTTGGGAGAGTGGCTCTAAGGGCTTTGGG<br>((((...))))..	ARL8A	NM_138795	chr1:200368600-200380489	non-template	34	Intron_1.f	--	Yes	--	Yes	--	--	--	Yes	Yes	Yes	--
G4ST01200380356	chr1:200380357-200380391	-	GGGCCGCAGCGCGAGGGCAGCGAGGGGCGGCGGGG<br>.(((...))).	ARL8A	NM_138795	chr1:200368600-200380489	non-template	35	Exon_1.f	--	Yes	Yes	--	--	Yes	--	Yes	Yes	Yes	Yes
G4ST01200380364	chr1:200380365-200380402	-	GGGGCCGCTCCGGGCCGCAGCGCGAGGGCAGCGAGGGG<br>.(((...))).	ARL8A	NM_138795	chr1:200368600-200380489	non-template	38	Exon_1.f	--	Yes	Yes	--	--	Yes	--	Yes	Yes	Yes	Yes
G4ST01200380374	chr1:200380375-200380407	-	GGGGAGGGGCCGCTCCGGGCCGCAGCGCGAGGG<br>.(((...))).	ARL8A	NM_138795	chr1:200368600-200380489	non-template	33	Exon_1.f	--	Yes	Yes	--	--	Yes	--	Yes	Yes	Yes	Yes
G4ST01200380600	chr1:200380601-200380640	+	GGGGGGCCAGGGGCCGCACCCGCAGGTGGGGTGAGCAGGG<br>....((((....))))	ARL8A	NM_138795	chr1:200368600-200380489	template	-111	upstream	Yes	--	--	--	--	--	--	Yes	Yes	Yes	--
G4ST01200384318	chr1:200384319-200384356	+	GGGAGGTAGGCACCTGGGCCACCGGAGGGTGGCAGGGG<br>((((....)))) ((...)).	PTPN7	NR_037663	chr1:200382764-200397339	template	38	Exon_10.l	--	Yes	Yes	--	--	--	--	Yes	--	Yes	Yes
G4ST01200384318	chr1:200384319-200384356	+	GGGAGGTAGGCACCTGGGCCACCGGAGGGTGGCAGGGG<br>((((....)))) ((...)).	PTPN7	NR_037664	chr1:200382764-200397339	template	38	Exon_9.l	--	Yes	Yes	--	--	--	--	Yes	--	Yes	Yes
G4ST01200384318	chr1:200384319-200384356	+	GGGAGGTAGGCACCTGGGCCACCGGAGGGTGGCAGGGG<br>((((....)))) ((...)).	PTPN7	NM_001199797	chr1:200382764-200397339	template	38	Exon_10.l	--	Yes	Yes	--	--	--	Yes	Yes	--	Yes	Yes
G4ST01200384318	chr1:200384319-200384356	+	GGGAGGTAGGCACCTGGGCCACCGGAGGGTGGCAGGGG<br>((((....)))) ((...)).	PTPN7	NM_080588	chr1:200382764-200395742	template	38	Exon_9.l	--	Yes	Yes	--	--	--	Yes	Yes	--	Yes	Yes
G4ST01200384318	chr1:200384319-200384356	+	GGGAGGTAGGCACCTGGGCCACCGGAGGGTGGCAGGGG<br>((((....)))) ((...)).	PTPN7	NM_002832	chr1:200382764-200397339	template	38	Exon_10.l	--	Yes	Yes	--	--	--	Yes	Yes	--	Yes	Yes
G4ST01200384333	chr1:200384334-200384363	+	GGGCCACCGGAGGGTGGCAGGGGTCAGGGG<br>((...)).	PTPN7	NR_037663	chr1:200382764-200397339	template	30	Exon_10.l	--	Yes	Yes	--	--	--	--	Yes	--	Yes	Yes

G4ST01200384333	chr1:200384334-200384363	+	GGGCCACCGGAGGGTGGCAGGGGTCAGGGG<br>((...)).	PTPN7	NR_037664	chr1:200382764-200397339	template	30	Exon_9.l	--	Yes	Yes	--	--	--	--	Yes	--	Yes	Yes
G4ST01200384333	chr1:200384334-200384363	+	GGGCCACCGGAGGGTGGCAGGGGTCAGGGG<br>((...)).	PTPN7	NM_001199797	chr1:200382764-200397339	template	30	Exon_10.l	--	Yes	Yes	--	Yes	--	Yes	Yes	--	Yes	Yes
G4ST01200384333	chr1:200384334-200384363	+	GGGCCACCGGAGGGTGGCAGGGGTCAGGGG<br>((...)).	PTPN7	NM_080588	chr1:200382764-200395742	template	30	Exon_9.l	--	Yes	Yes	--	Yes	--	Yes	Yes	--	Yes	Yes
G4ST01200384333	chr1:200384334-200384363	+	GGGCCACCGGAGGGTGGCAGGGGTCAGGGG<br>((...)).	PTPN7	NM_002832	chr1:200382764-200397339	template	30	Exon_10.l	--	Yes	Yes	--	Yes	--	Yes	Yes	--	Yes	Yes
G4ST01200393442	chr1:200393443-200393474	-	GGGATGAAGGGAGTCGGGTACAGTCCCTGGGG<br>..(((...)))	PTPN7	NR_037663	chr1:200382764-200397339	non-template	32	Intron_4	--	Yes	--	Yes	--	--	--	Yes	Yes	Yes	--
G4ST01200393442	chr1:200393443-200393474	-	GGGATGAAGGGAGTCGGGTACAGTCCCTGGGG<br>..(((...)))	PTPN7	NR_037664	chr1:200382764-200397339	non-template	32	Intron_3	--	Yes	--	Yes	--	--	--	Yes	Yes	Yes	--
G4ST01200393442	chr1:200393443-200393474	-	GGGATGAAGGGAGTCGGGTACAGTCCCTGGGG<br>..(((...)))	PTPN7	NM_001199797	chr1:200382764-200397339	non-template	32	Intron_4	--	Yes	--	Yes	--	--	--	Yes	Yes	Yes	--
G4ST01200393442	chr1:200393443-200393474	-	GGGATGAAGGGAGTCGGGTACAGTCCCTGGGG<br>..(((...)))	PTPN7	NM_080588	chr1:200382764-200395742	non-template	32	Intron_3	--	Yes	--	Yes	--	--	--	Yes	Yes	Yes	--
G4ST01200393442	chr1:200393443-200393474	-	GGGATGAAGGGAGTCGGGTACAGTCCCTGGGG<br>..(((...)))	PTPN7	NM_002832	chr1:200382764-200397339	non-template	32	Intron_4	--	Yes	--	Yes	--	--	--	Yes	Yes	Yes	--
G4ST01200402668	chr1:200402669-200402695	+	GGGCTGGGAGTGGGCTACAGGTAGGGG<br>(((....)))	PTPRVP	NR_002930	chr1:200403802-200425200	non-template	-1106	upstream	Yes	--	--	--	--	--	--	--	Yes	--	--
G4ST01200407071	chr1:200407072-200407106	-	GGGGCCCTGGGAATGTGACATTGTGGGCCTCAGGG<br>((((...))))..	PTPRVP	NR_002930	chr1:200403802-200425200	template	35	Exon_5	--	Yes	Yes	--	--	--	--	--	--	--	--
G4ST01200407079	chr1:200407080-200407111	-	GGGCAGGGGCCCTGGGAATGTGACATTGTGGG<br>((((...))))..	PTPRVP	NR_002930	chr1:200403802-200425200	template	32	Exon_5	--	Yes	Yes	--	--	--	--	--	--	--	--
G4ST01200421155	chr1:200421156-200421185	+	GGGTGTCAGGGACCAGAAAGGTGGGCAGGG<br>(((.....)))	PTPRVP	NR_002930	chr1:200403802-200425200	non-template	30	Intron_15	--	Yes	--	Yes	--	--	--	--	--	--	Yes
G4ST01200421163	chr1:200421164-200421190	+	GGGACCAGAAAGGTGGGCAGGGAGGGG<br>(((.....)))	PTPRVP	NR_002930	chr1:200403802-200425200	non-template	27	Intron_15	--	Yes	--	Yes	--	--	--	--	--	--	Yes
G4ST01200422290	chr1:200422291-200422327	+	GGGGCTGTCCCCGCAGTGTGGGCATGGGCCAGACGGG<br>.((((....))))...	PTPRVP	NR_002930	chr1:200403802-200425200	non-template	37	Exon_17	--	Yes	Yes	--	--	--	--	--	--	Yes	--
G4ST01200422446	chr1:200422447-200422476	+	GGGCCTCGCAGGCTGGGCTGGGCAGCTGGG<br>(((...)))..	PTPRVP	NR_002930	chr1:200403802-200425200	non-template	30	Intron_17	--	Yes	--	Yes	--	--	--	--	--	Yes	--
G4ST01200422997	chr1:200422998-200423029	-	GGGCCAAGACCTGGTGGGGGGGTTGGAGAGGG<br>(((.....))).	PTPRVP	NR_002930	chr1:200403802-200425200	template	32	Intron_18-Exon_19	--	Yes	Yes	Yes	--	--	--	--	--	Yes	--
G4ST01200429584	chr1:200429585-200429626	-	GGGGCTCTATTGGGCGGCGGCGGCGGCGGTGGGAGCGCCGGG<br>((.((....)).))..	LGR6	NM_001017403	chr1:200429741-200555512	template	-114	upstream	Yes	--	--	--	--	--	--	Yes	--	--	--
G4ST01200435319	chr1:200435320-200435365	-	GGGTTGAGAGCCCATGGAATGGGGGGAAGGCGGGGTGGAGTGGGGG<br>.......(((((...)))))	LGR6	NM_001017403	chr1:200429741-200555512	template	46	Intron_1.f	--	Yes	--	Yes	--	--	--	--	Yes	Yes	Yes
G4ST01200435330	chr1:200435331-200435375	-	GGGGCCATCAGGGTTGAGAGCCCATGGAATGGGGGGAAGGCGGGG<br>.......(((((...)))))	LGR6	NM_001017403	chr1:200429741-200555512	template	45	Intron_1.f	--	Yes	--	Yes	--	--	--	--	Yes	Yes	Yes
G4ST01200435806	chr1:200435807-200435839	-	GGGCTTTGTTAGTAAAGGAGGAGGGTGGGAGGG<br>(((((....)))))....	LGR6	NM_001017403	chr1:200429741-200555512	template	33	Intron_1.f	--	Yes	--	Yes	--	--	--	Yes	Yes	--	Yes
G4ST01200435810	chr1:200435811-200435844	-	GGGACGGGCTTTGTTAGTAAAGGAGGAGGGTGGG<br>(((((....)))))....	LGR6	NM_001017403	chr1:200429741-200555512	template	34	Intron_1.f	--	Yes	--	Yes	--	--	--	Yes	Yes	--	Yes
G4ST01200436650	chr1:200436651-200436688	+	GGGCAGGCCGGCGGCTGGCGGGTTGGAGGGGAGCTGGG<br>...((((.....))))	LGR6	NM_001017403	chr1:200429741-200555512	non-template	38	Intron_1.f	--	Yes	--	Yes	--	--	--	--	Yes	--	--
G4ST01200449916	chr1:200449917-200449943	+	GGGAGAGGGCCGCTCAGCGAGGGCGGG<br>.(((...))).	LGR6	NM_021636	chr1:200439528-200555512	non-template	27	Intron_1.f	--	Yes	--	Yes	--	--	--	Yes	--	--	--
G4ST01200449916	chr1:200449917-200449943	+	GGGAGAGGGCCGCTCAGCGAGGGCGGG<br>.(((...))).	LGR6	NM_001017404	chr1:200449906-200555512	non-template	27	Exon_1.f	--	Yes	Yes	--	Yes	--	--	Yes	--	--	--
G4ST01200449916	chr1:200449917-200449943	+	GGGAGAGGGCCGCTCAGCGAGGGCGGG<br>.(((...))).	LGR6	NM_001017403	chr1:200429741-200555512	non-template	27	Intron_1.f	--	Yes	--	Yes	--	--	--	Yes	--	--	--
G4ST01200481029	chr1:200481030-200481062	+	GGGGCAGAGGCTGCTCTGAGGTGGGTGGGTGGG<br>.(((((....)))))....	LGR6	NM_021636	chr1:200439528-200555512	non-template	33	Intron_4	--	Yes	--	Yes	--	--	--	--	--	--	Yes
G4ST01200481029	chr1:200481030-200481062	+	GGGGCAGAGGCTGCTCTGAGGTGGGTGGGTGGG<br>.(((((....)))))....	LGR6	NM_001017404	chr1:200449906-200555512	non-template	33	Intron_4	--	Yes	--	Yes	--	--	--	--	--	--	Yes
G4ST01200481029	chr1:200481030-200481062	+	GGGGCAGAGGCTGCTCTGAGGTGGGTGGGTGGG<br>.(((((....)))))....	LGR6	NM_001017403	chr1:200429741-200555512	non-template	33	Intron_4	--	Yes	--	Yes	--	--	--	--	--	--	Yes
G4ST01200513583	chr1:200513584-200513617	+	GGGGAAGGAGGCTTCCTGGGGGAGGTGGGGTGGG<br>..((((....))))..	LGR6	NM_021636	chr1:200439528-200555512	non-template	34	Intron_5	--	Yes	--	Yes	--	--	--	--	--	--	Yes
G4ST01200513583	chr1:200513584-200513617	+	GGGGAAGGAGGCTTCCTGGGGGAGGTGGGGTGGG<br>..((((....))))..	LGR6	NM_001017404	chr1:200449906-200555512	non-template	34	Intron_4	--	Yes	--	Yes	--	--	--	--	--	--	Yes
G4ST01200513583	chr1:200513584-200513617	+	GGGGAAGGAGGCTTCCTGGGGGAGGTGGGGTGGG<br>..((((....))))..	LGR6	NM_001017403	chr1:200429741-200555512	non-template	34	Intron_5	--	Yes	--	Yes	--	--	--	--	--	--	Yes
G4ST01200542320	chr1:200542321-200542353	+	GGGAGGTGTGGGGGCCAGTTTGGCTGGGCTGGG<br>.((((...)))).	LGR6	NM_021636	chr1:200439528-200555512	non-template	33	Intron_12	--	Yes	--	Yes	--	--	--	--	--	--	--
G4ST01200542320	chr1:200542321-200542353	+	GGGAGGTGTGGGGGCCAGTTTGGCTGGGCTGGG<br>.((((...)))).	LGR6	NM_001017404	chr1:200449906-200555512	non-template	33	Intron_10	--	Yes	--	Yes	--	--	--	--	--	--	--
G4ST01200542320	chr1:200542321-200542353	+	GGGAGGTGTGGGGGCCAGTTTGGCTGGGCTGGG<br>.((((...)))).	LGR6	NM_001017403	chr1:200429741-200555512	non-template	33	Intron_12	--	Yes	--	Yes	--	--	--	--	--	--	--
G4ST01200552197	chr1:200552198-200552229	-	GGGGCCTAGTGGGAGGTGTCTGGGTCATGGGG<br>((....))	LGR6	NM_021636	chr1:200439528-200555512	template	32	Intron_17.l	--	Yes	--	Yes	--	--	--	--	--	--	--
G4ST01200552197	chr1:200552198-200552229	-	GGGGCCTAGTGGGAGGTGTCTGGGTCATGGGG<br>((....))	LGR6	NM_001017404	chr1:200449906-200555512	template	32	Intron_15.l	--	Yes	--	Yes	--	--	--	--	--	--	--
G4ST01200552197	chr1:200552198-200552229	-	GGGGCCTAGTGGGAGGTGTCTGGGTCATGGGG<br>((....))	LGR6	NM_001017403	chr1:200429741-200555512	template	32	Intron_17.l	--	Yes	--	Yes	--	--	--	--	--	--	--
G4ST01200628381	chr1:200628382-200628419	-	GGGCCTGTAGTCCCAGCTACTTGGGAGTCTGGGGTGGG<br>...(((((....)))))..	PPP1R12B	NM_001167857	chr1:200584453-200673993	template	38	Intron_1.f	--	Yes	--	Yes	--	--	--	--	--	--	--
G4ST01200628381	chr1:200628382-200628419	-	GGGCCTGTAGTCCCAGCTACTTGGGAGTCTGGGGTGGG<br>...(((((....)))))..	PPP1R12B	NM_001167858	chr1:200584453-200673993	template	38	Intron_1.f	--	Yes	--	Yes	--	--	--	--	--	--	--
G4ST01200628381	chr1:200628382-200628419	-	GGGCCTGTAGTCCCAGCTACTTGGGAGTCTGGGGTGGG<br>...(((((....)))))..	PPP1R12B	NM_002481	chr1:200584453-200824320	template	38	Intron_1.f	--	Yes	--	Yes	--	--	--	--	--	--	--
G4ST01200840169	chr1:200840170-200840214	-	GGGGGTCAGGTAGGGCACATTGGTTGTGAGGGGGACTGGAAGGGG<br>((((.....))))...	SYT2	NM_001136504	chr1:200826347-200879204	non-template	45	Exon_3-Intron_3	--	Yes	Yes	Yes	Yes	--	--	--	--	--	--
G4ST01200840169	chr1:200840170-200840214	-	GGGGGTCAGGTAGGGCACATTGGTTGTGAGGGGGACTGGAAGGGG<br>((((.....))))...	SYT2	NM_177402	chr1:200826347-200946174	non-template	45	Exon_3-Intron_3	--	Yes	Yes	Yes	Yes	--	--	--	--	--	--
G4ST01200869322	chr1:200869323-200869351	-	GGGAGCTAAGCTGGGATGGGTGCCCAGGG<br>(((...)))	SYT2	NM_001136504	chr1:200826347-200879204	non-template	29	Intron_1.f	--	Yes	--	Yes	--	--	--	--	--	--	--
G4ST01200869322	chr1:200869323-200869351	-	GGGAGCTAAGCTGGGATGGGTGCCCAGGG<br>(((...)))	SYT2	NM_177402	chr1:200826347-200946174	non-template	29	Intron_1.f	--	Yes	--	Yes	--	--	--	--	--	--	--
G4ST01200874515	chr1:200874516-200874547	-	GGGGAATAAGTCTGACTTGGGATGGGGCGGGG<br>....((((...))))	SYT2	NM_001136504	chr1:200826347-200879204	non-template	32	Intron_1.f	--	Yes	--	Yes	--	--	--	Yes	--	--	Yes
G4ST01200874515	chr1:200874516-200874547	-	GGGGAATAAGTCTGACTTGGGATGGGGCGGGG<br>....((((...))))	SYT2	NM_177402	chr1:200826347-200946174	non-template	32	Intron_1.f	--	Yes	--	Yes	--	--	--	Yes	--	--	Yes
G4ST01200880766	chr1:200880767-200880805	+	GGGGGAGTGTGCATCTGCATACCTGGGTGTTGGGAAGGG<br>...((((((....))))))..	SYT2	NM_001136504	chr1:200826347-200879204	template	-1562	upstream	Yes	--	--	--	--	--	--	--	--	--	--
G4ST01200880766	chr1:200880767-200880805	+	GGGGGAGTGTGCATCTGCATACCTGGGTGTTGGGAAGGG<br>...((((((....))))))..	SYT2	NM_177402	chr1:200826347-200946174	template	39	Intron_1.f	--	Yes	--	Yes	--	--	--	--	--	--	--
G4ST01200881551	chr1:200881552-200881585	+	GGGCCAAAATGGGGGAGGATGGGAACACAGTGGG<br>.((...))	SYT2	NM_177402	chr1:200826347-200946174	template	34	Intron_1.f	--	Yes	--	Yes	--	--	--	--	Yes	--	--

G4ST01200882094	chr1:200882095-200882130	-	GGGGTCAGGGAACTGGGACTGGCAGGATGGAGGGGG<br>.((..((...)).)).	SYT2	NM_177402	chr1:200826347-200946174	non-template	36	Intron_1.f	--	Yes	--	Yes	--	--	--	--	Yes	--	Yes
G4ST01200917822	chr1:200917823-200917857	-	GGGACCCAGGGAGCTACAGAGGTGGCTGGGCTGGG<br>((((((....))))))	SYT2	NM_177402	chr1:200826347-200946174	non-template	35	Intron_1.f	--	Yes	--	Yes	--	--	--	--	--	--	--
G4ST01200940344	chr1:200940345-200940383	-	GGGCCCCTTGCAAAGGCAGGGAGAATGGGAGTTAGTGGG<br>..((((...))))..	SYT2	NM_177402	chr1:200826347-200946174	non-template	39	Intron_1.f	--	Yes	--	Yes	--	--	--	--	--	--	Yes
G4ST01200940354	chr1:200940355-200940391	-	GGGGAGGAGGGCCCCTTGCAAAGGCAGGGAGAATGGG<br>..((((...))))..	SYT2	NM_177402	chr1:200826347-200946174	non-template	37	Intron_1.f	--	Yes	--	Yes	--	--	--	--	--	--	Yes
G4ST01200940362	chr1:200940363-200940396	-	GGGCTGGGGAGGAGGGCCCCTTGCAAAGGCAGGG<br>..((((...))))..	SYT2	NM_177402	chr1:200826347-200946174	non-template	34	Intron_1.f	--	Yes	--	Yes	--	--	--	--	--	--	Yes
G4ST01200944919	chr1:200944920-200944946	-	GGGGCCACTGCAGTGGGGAGGGAAGGG<br>..(((....)))	SYT2	NM_177402	chr1:200826347-200946174	non-template	27	Intron_1.f	--	Yes	--	Yes	--	--	--	--	--	--	--
G4ST01200945754	chr1:200945755-200945793	-	GGGGCGAGGAGCCGGCTCCTCTGAGGGACTGGGGCTGGG<br>..((((((....))))))...	SYT2	NM_177402	chr1:200826347-200946174	non-template	39	Intron_1.f	--	Yes	--	Yes	--	--	--	--	--	--	Yes
G4ST01200995935	chr1:200995936-200995975	+	GGGCCTGTAGTCCCAGCTACGTGGGGGGGGCTGAGGTGGG<br>..((((((....)))))).	KDM5B	NM_006618	chr1:200963155-201044172	template	40	Intron_8	--	Yes	--	Yes	--	--	--	--	--	--	--
G4ST01201043179	chr1:201043180-201043220	-	GGGGTCAAGAAGGGGGCGGCCAGCGCTGGCTGGGGGCGGGG<br>..((((((....)))))).	KDM5B	NM_006618	chr1:200963155-201044172	non-template	41	Intron_1.f	--	Yes	--	Yes	--	--	--	--	Yes	Yes	--
G4ST01201043184	chr1:201043185-201043225	-	GGGCCGGGGTCAAGAAGGGGGCGGCCAGCGCTGGCTGGGGG<br>..((((((....)))))).	KDM5B	NM_006618	chr1:200963155-201044172	non-template	41	Intron_1.f	--	Yes	--	Yes	--	--	--	--	Yes	Yes	--
G4ST01201043279	chr1:201043280-201043311	+	GGGTGGGGCGCCCAGGTGAGGGCCGCTCCGGG<br>.(((....))).	KDM5B	NM_006618	chr1:200963155-201044172	template	32	Intron_1.f	--	Yes	--	Yes	--	--	--	Yes	Yes	Yes	--
G4ST01201243220	chr1:201243221-201243255	-	GGGGCGAGGCCTGCCTAGCGGGCCGGGCCCCCGGG<br>((.(((....))).))	LOC401980	NR_036557	chr1:201222203-201243016	non-template	-204	upstream	Yes	--	--	--	--	--	--	Yes	Yes	Yes	--
G4ST01201243220	chr1:201243221-201243255	-	GGGGCGAGGCCTGCCTAGCGGGCCGGGCCCCCGGG<br>((.(((....))).))	TMEM183A	NM_138391	chr1:201243157-201259820	template	35	Exon_1.f	--	Yes	Yes	--	Yes	--	--	Yes	Yes	Yes	--
G4ST01201243220	chr1:201243221-201243255	-	GGGGCGAGGCCTGCCTAGCGGGCCGGGCCCCCGGG<br>((.(((....))).))	TMEM183B	NM_001079809	chr1:201243159-201259291	template	35	Exon_1.f	--	Yes	Yes	--	Yes	--	--	Yes	Yes	Yes	--
G4ST01201258769	chr1:201258770-201258808	-	GGGATGGGAAGCAGTAACTACGCTCTCAGGGAGAATGGG<br>.(((.((....)))))....	TMEM183A	NM_138391	chr1:201243157-201259820	template	39	Exon_8.l	--	Yes	Yes	--	Yes	--	Yes	--	--	--	--
G4ST01201258769	chr1:201258770-201258808	-	GGGATGGGAAGCAGTAACTACGCTCTCAGGGAGAATGGG<br>.(((.((....)))))....	TMEM183B	NM_001079809	chr1:201243159-201259291	template	39	Exon_8.l	--	Yes	Yes	--	Yes	--	Yes	--	--	--	--
G4ST01201295630	chr1:201295631-201295661	+	GGGGTCTGGAGGGAAGGATGCTTGGGGTGGG<br>(((....))).	PPFIA4	NM_015053	chr1:201286934-201314487	non-template	31	Intron_8	--	Yes	--	Yes	--	--	--	--	--	--	--
G4ST01201295640	chr1:201295641-201295669	+	GGGAAGGATGCTTGGGGTGGGCAGAGGGG<br>(((....))).	PPFIA4	NM_015053	chr1:201286934-201314487	non-template	29	Intron_8	--	Yes	--	Yes	--	--	--	--	--	--	--
G4ST01201310354	chr1:201310355-201310390	+	GGGCTGGGAGGGGTTGTTGAGGCAAGAACAGGTGGG<br>(((.(((...))).)))....	PPFIA4	NM_015053	chr1:201286934-201314487	non-template	36	Intron_15	--	Yes	--	Yes	--	--	--	--	--	--	Yes
G4ST01201321120	chr1:201321121-201321163	-	GGGGCTCAGAGGGTTGGTGCAGGTGCCAGACAGGGTCGAGGGG<br>.(((((....)))))....	MYOG	NM_002479	chr1:201318880-201322000	non-template	43	Intron_1.f	--	Yes	--	Yes	--	--	--	--	Yes	--	--
G4ST01201364090	chr1:201364091-201364118	-	GGGCCGCGCGCGGTAGGGCTGGGCTGGG<br>(((....)))..	ADORA1	NM_001048230	chr1:201363459-201403156	template	28	Intron_1.f	--	Yes	--	Yes	--	--	--	Yes	Yes	Yes	--
G4ST01201364090	chr1:201364091-201364118	-	GGGCCGCGCGCGGTAGGGCTGGGCTGGG<br>(((....)))..	ADORA1	NM_000674	chr1:201363459-201403156	template	28	Exon_2	--	Yes	Yes	--	--	Yes	--	Yes	Yes	Yes	--
G4ST01201364483	chr1:201364484-201364510	-	GGGGAGACCCGTCTGGGAGGGGCAGGG<br>.(((....)))	ADORA1	NM_001048230	chr1:201363459-201403156	template	27	Intron_1.f	--	Yes	--	Yes	--	--	--	--	--	--	--
G4ST01201364483	chr1:201364484-201364510	-	GGGGAGACCCGTCTGGGAGGGGCAGGG<br>.(((....)))	ADORA1	NM_000674	chr1:201363459-201403156	template	27	Intron_2	--	Yes	--	Yes	--	--	--	--	--	--	--
G4ST01201364488	chr1:201364489-201364515	-	GGGATGGGGAGACCCGTCTGGGAGGGG<br>.(((....)))	ADORA1	NM_001048230	chr1:201363459-201403156	template	27	Intron_1.f	--	Yes	--	Yes	--	--	--	--	--	--	--
G4ST01201364488	chr1:201364489-201364515	-	GGGATGGGGAGACCCGTCTGGGAGGGG<br>.(((....)))	ADORA1	NM_000674	chr1:201363459-201403156	template	27	Intron_2	--	Yes	--	Yes	--	--	--	--	--	--	--
G4ST01201364493	chr1:201364494-201364524	-	GGGAAGCCTGGGATGGGGAGACCCGTCTGGG<br>.(((....)))	ADORA1	NM_001048230	chr1:201363459-201403156	template	31	Intron_1.f	--	Yes	--	Yes	--	--	--	--	--	--	--
G4ST01201364493	chr1:201364494-201364524	-	GGGAAGCCTGGGATGGGGAGACCCGTCTGGG<br>.(((....)))	ADORA1	NM_000674	chr1:201363459-201403156	template	31	Intron_2	--	Yes	--	Yes	--	--	--	--	--	--	--
G4ST01201404928	chr1:201404929-201404968	-	GGGGGCAGGTGGGGACAGCAGGGCTGAGGAATCCTCCGGG<br>..((((...)))).	MYBPH	NM_004997	chr1:201403562-201411565	non-template	40	Intron_8	--	Yes	--	Yes	--	--	--	--	--	--	Yes
G4ST01201410793	chr1:201410794-201410831	-	GGGTAAGGGGACACTGGGTTCTCCTTTGGTGAGAAGGG<br>(((((.......)))))	MYBPH	NM_004997	chr1:201403562-201411565	non-template	38	Intron_2	--	Yes	--	Yes	--	--	--	--	--	--	Yes
G4ST01201411245	chr1:201411246-201411283	-	GGGGGAGGAGGGAGGCAGGCATGTCAGGGGTGGAGGGG<br>.((((....))))..	MYBPH	NM_004997	chr1:201403562-201411565	non-template	38	Intron_1.f	--	Yes	--	Yes	--	--	--	--	--	--	--
G4ST01201411253	chr1:201411254-201411289	-	GGGGCAGGGGGAGGAGGGAGGCAGGCATGTCAGGGG<br>.((((....))))..	MYBPH	NM_004997	chr1:201403562-201411565	non-template	36	Intron_1.f	--	Yes	--	Yes	--	--	--	--	--	--	--
G4ST01201411342	chr1:201411343-201411374	+	GGGGCCTGTGGGGCAGGGGCCTGCGGCTTGGG<br>(((...)))..	MYBPH	NM_004997	chr1:201403562-201411565	template	32	Exon_1.f	--	Yes	Yes	--	Yes	--	--	--	--	--	--
G4ST01201411780	chr1:201411781-201411817	+	GGGTGCTGGTGCCCAGCTGGGGGAGGGCGCAGATGGG<br>.(((((...)))))...	MYBPH	NM_004997	chr1:201403562-201411565	template	-215	upstream	Yes	--	--	--	--	--	--	Yes	Yes	--	Yes
G4ST01201462787	chr1:201462788-201462830	-	GGGCATTGGAGGGGGTACAGACAGACCCTGCTGGGGCAAAGGG<br>....(((.(((...))))))	CHIT1	NM_003465	chr1:201451830-201465483	non-template	43	Intron_2	--	Yes	--	Yes	--	--	--	--	--	--	--
G4ST01201539432	chr1:201539433-201539468	+	GGGCAGAGATGGGGATCATGGGCAAGCTGTTGAGGG<br>(((....))).	BTG2	NM_006763	chr1:201541287-201545352	non-template	-1818	upstream	Yes	--	--	--	--	--	--	--	Yes	Yes	--
G4ST01201539432	chr1:201539433-201539468	+	GGGCAGAGATGGGGATCATGGGCAAGCTGTTGAGGG<br>(((....))).	LOC730227	NR_034150	chr1:201534509-201541076	template	36	Intron_1.f	--	Yes	--	Yes	--	--	--	--	Yes	Yes	--
G4ST01201539432	chr1:201539433-201539468	+	GGGCAGAGATGGGGATCATGGGCAAGCTGTTGAGGG<br>(((....))).	LOC730227	NR_034151	chr1:201534509-201541076	template	36	Intron_2	--	Yes	--	Yes	--	--	--	--	Yes	Yes	--
G4ST01201586605	chr1:201586606-201586646	-	GGGATGCTGGAGAGTGGCAGAGAGGGTGTATAAGGGAGGGG<br>.(((((.....)))))....	FMOD	NM_002023	chr1:201576375-201586912	non-template	41	Intron_1.f	--	Yes	--	Yes	--	--	--	--	--	--	--
G4ST01201712793	chr1:201712794-201712834	+	GGGTCTGTCCCAAGGCAGGCGGGGTGTAGGGAGATCAGGGG<br>.(((((....)))))...	PRELP	NM_002725	chr1:201711506-201727102	non-template	41	Intron_1.f	--	Yes	--	Yes	--	--	--	--	--	--	--
G4ST01201712793	chr1:201712794-201712834	+	GGGTCTGTCCCAAGGCAGGCGGGGTGTAGGGAGATCAGGGG<br>.(((((....)))))...	PRELP	NM_201348	chr1:201711506-201727102	non-template	41	Intron_1.f	--	Yes	--	Yes	--	--	--	--	--	--	--
G4ST01201732933	chr1:201732934-201732977	+	GGGGGTACCAAAGTGGAACGTGGTTGGGGTTGGGGCATGGAGGG<br>...((((..((...)).))))..	OPTC	NM_014359	chr1:201729894-201744700	non-template	44	Intron_3	--	Yes	--	Yes	--	--	--	--	--	--	Yes
G4ST01201862660	chr1:201862661-201862687	+	GGGAGGGGGGGCACGAACTTCGTGGGG<br>(((((...)))))	ATP2B4	NM_001684	chr1:201862551-201979832	non-template	27	Exon_1.f	--	Yes	Yes	--	--	Yes	--	Yes	Yes	Yes	Yes
G4ST01201862660	chr1:201862661-201862687	+	GGGAGGGGGGGCACGAACTTCGTGGGG<br>(((((...)))))	ATP2B4	NM_001001396	chr1:201862551-201979832	non-template	27	Exon_1.f	--	Yes	Yes	--	--	Yes	--	Yes	Yes	Yes	Yes
G4ST01201864372	chr1:201864373-201864401	-	GGGGAGGGGCTTAAGGAGCAGGGCCAGGG<br>(((.....))).	ATP2B4	NM_001684	chr1:201862551-201979832	template	29	Intron_1.f	--	Yes	--	Yes	--	--	--	Yes	Yes	--	--
G4ST01201864372	chr1:201864373-201864401	-	GGGGAGGGGCTTAAGGAGCAGGGCCAGGG<br>(((.....))).	ATP2B4	NM_001001396	chr1:201862551-201979832	template	29	Intron_1.f	--	Yes	--	Yes	--	--	--	Yes	Yes	--	--
G4ST01201864378	chr1:201864379-201864411	-	GGGGCAGCTTGGGGAGGGGCTTAAGGAGCAGGG<br>(((.....))).	ATP2B4	NM_001684	chr1:201862551-201979832	template	33	Intron_1.f	--	Yes	--	Yes	--	--	--	Yes	Yes	--	--
G4ST01201864378	chr1:201864379-201864411	-	GGGGCAGCTTGGGGAGGGGCTTAAGGAGCAGGG<br>(((.....))).	ATP2B4	NM_001001396	chr1:201862551-201979832	template	33	Intron_1.f	--	Yes	--	Yes	--	--	--	Yes	Yes	--	--
G4ST01201898175	chr1:201898176-201898221	-	GGGGAATGGGCATTATTGTTTAATGGGTACAGAGTTTCAGTTTGGG<br>.((((.....)))) .((.((...)).))..	ATP2B4	NM_001684	chr1:201862551-201979832	template	46	Intron_1.f	--	Yes	--	Yes	--	--	--	--	--	--	--
G4ST01201898175	chr1:201898176-201898221	-	GGGGAATGGGCATTATTGTTTAATGGGTACAGAGTTTCAGTTTGGG<br>.((((.....)))) .((.((...)).))..	ATP2B4	NM_001001396	chr1:201862551-201979832	template	46	Intron_1.f	--	Yes	--	Yes	--	--	--	--	--	--	--

G4ST01201951662	chr1:201951663-201951700	-	GGGGTGGGGCCTGAGTGGGTGAATCTGCATTCTATGGG<br>.((((....))))...	ATP2B4	NM_001684	chr1:201862551-201979832	template	38	Intron_15	--	Yes	--	Yes	--	--	--	--	--	--	--
G4ST01201951662	chr1:201951663-201951700	-	GGGGTGGGGCCTGAGTGGGTGAATCTGCATTCTATGGG<br>.((((....))))...	ATP2B4	NM_001001396	chr1:201862551-201979832	template	38	Intron_15	--	Yes	--	Yes	--	--	--	--	--	--	--
G4ST01202308669	chr1:202308670-202308704	-	GGGGAAAGCTAAGGTTTACGGGCGGCGGGGAAGGG<br>..((((....))))..	SOX13	NM_005686	chr1:202308869-202363494	template	-164	upstream	Yes	--	--	--	--	--	--	--	Yes	Yes	Yes
G4ST01202310521	chr1:202310522-202310561	-	GGGCCCTCCAGGGCGGGAGCCGCCGCTCCAGCGCTCGGGG<br>...((.(((....)))..))	SOX13	NM_005686	chr1:202308869-202363494	template	40	Intron_1.f	--	Yes	--	Yes	--	--	--	Yes	Yes	Yes	--
G4ST01202317250	chr1:202317251-202317289	+	GGGCCCCACGGACCCCCTGGCGGGCTCTGGGGGTGGGGG<br>..(((.((....))))).	SOX13	NM_005686	chr1:202308869-202363494	non-template	39	Intron_1.f	--	Yes	--	Yes	--	--	--	Yes	--	--	Yes
G4ST01202320447	chr1:202320448-202320477	-	GGGCGGGGCCTGAGAGGAGGGAGGAAGGGG<br>.(((...))).	SOX13	NM_005686	chr1:202308869-202363494	template	30	Intron_1.f	--	Yes	--	Yes	--	--	--	Yes	Yes	--	--
G4ST01202328492	chr1:202328493-202328524	+	GGGTCATGGGCATGGCCTTTATGGGGTATGGG<br>((((.....))))	SOX13	NM_005686	chr1:202308869-202363494	non-template	32	Intron_1.f	--	Yes	--	Yes	--	--	--	--	Yes	--	--
G4ST01202328499	chr1:202328500-202328534	+	GGGCATGGCCTTTATGGGGTATGGGAAAGGAGGGG<br>((((.....))))	SOX13	NM_005686	chr1:202308869-202363494	non-template	35	Intron_1.f	--	Yes	--	Yes	--	--	--	--	Yes	--	--
G4ST01202336191	chr1:202336192-202336233	+	GGGGTGCTGGGCAGATTGGGGTGAAAGCTGGCTGCTTTGGGG<br>...(((((.....))))).	SOX13	NM_005686	chr1:202308869-202363494	non-template	42	Intron_1.f	--	Yes	--	Yes	--	--	--	--	--	--	--
G4ST01202352536	chr1:202352537-202352569	+	GGGGGCAGGCTGGGCAGGGTCTGTGGCCAGGGG<br>.(((....)))	SOX13	NM_005686	chr1:202308869-202363494	non-template	33	Intron_5	--	Yes	--	Yes	--	--	--	--	--	--	Yes
G4ST01202355227	chr1:202355228-202355259	-	GGGACCTTTGGGAGGTGCCTGGGTCATGAGGG<br>((....))	SOX13	NM_005686	chr1:202308869-202363494	template	32	Intron_7	--	Yes	--	Yes	--	--	--	--	--	--	--
G4ST01202357398	chr1:202357399-202357430	+	GGGAAACTCTATTTAGAGTGGGTTGGGAAGGG<br>..(((((....)))))	SOX13	NM_005686	chr1:202308869-202363494	non-template	32	Intron_7	--	Yes	--	Yes	--	--	--	--	--	--	Yes
G4ST01202358083	chr1:202358084-202358110	-	GGGGCCTCCATGGCTGGGGGGGCGGGG<br>(((.....))).	SOX13	NM_005686	chr1:202308869-202363494	template	27	Exon_9	--	Yes	Yes	--	Yes	--	--	--	--	--	--
G4ST01202358088	chr1:202358089-202358136	-	GGGGGGCTGGACTGCAGGTCCCGCGTGGGGCCTCCATGGCTGGGGGGG<br>..((.((((.....)))).))... (((.....))).	SOX13	NM_005686	chr1:202308869-202363494	template	48	Exon_9	--	Yes	Yes	--	Yes	--	--	--	--	--	--
G4ST01202387812	chr1:202387813-202387840	-	GGGGGCGGGCCGGGGGCGTTCCCGCGGG<br>.(((....)))	ETNK2	NM_018208	chr1:202366813-202387930	non-template	28	Exon_1.f	--	Yes	Yes	--	--	Yes	--	Yes	Yes	Yes	Yes
G4ST01202388243	chr1:202388244-202388270	-	GGGAGGTTGCCTGGGTGTGGGGGGGGG<br>(((...)))	ETNK2	NM_018208	chr1:202366813-202387930	non-template	-313	upstream	Yes	--	--	--	--	--	--	--	--	Yes	--
G4ST01202388249	chr1:202388250-202388276	-	GGGGATGGGAGGTTGCCTGGGTGTGGG<br>(((...)))	ETNK2	NM_018208	chr1:202366813-202387930	non-template	-319	upstream	Yes	--	--	--	--	--	--	--	--	Yes	--
G4ST01202388620	chr1:202388621-202388657	+	GGGAAGGAAGGGGAAGGGGTGACAAGGTGTCCCGGGG<br>..((((...)))))...	ETNK2	NM_018208	chr1:202366813-202387930	template	-690	upstream	Yes	--	--	--	--	--	--	--	--	--	Yes
G4ST01202390608	chr1:202390609-202390634	+	GGGAAGGGCTGGTGCAGGGGTCAGGG<br>(((...)))	REN	NM_000537	chr1:202390567-202402088	template	26	Exon_10.l	--	Yes	Yes	--	--	--	Yes	--	--	--	--
G4ST01202396236	chr1:202396237-202396276	+	GGGCCCTGGAGGGTCAGGAGAGGCCTGGGGACAGAAGGGG<br>.((((.....))))	REN	NM_000537	chr1:202390567-202402088	template	40	Intron_4	--	Yes	--	Yes	--	--	--	--	--	--	--
G4ST01202396246	chr1:202396247-202396283	+	GGGTCAGGAGAGGCCTGGGGACAGAAGGGGTCCGGGG<br>.((((.....))))	REN	NM_000537	chr1:202390567-202402088	template	37	Intron_4	--	Yes	--	Yes	--	--	--	--	--	--	--
G4ST01202426218	chr1:202426219-202426246	-	GGGCGGGGCTGAGGGCGCAGGTGCGGGG<br>(((....)))	KISS1	NM_002256	chr1:202426092-202432242	non-template	28	Exon_3.l	--	Yes	Yes	--	Yes	--	Yes	Yes	Yes	--	--
G4ST01202430640	chr1:202430641-202430678	+	GGGGGAAGGGGCCCTGCAGGGGCTTCATCCTGGAGGGG<br>.(((((...)))))	KISS1	NM_002256	chr1:202426092-202432242	template	38	Intron_1.f	--	Yes	--	Yes	--	--	--	Yes	--	--	--
G4ST01202447491	chr1:202447492-202447531	+	GGGGAAGCGGGGTGCGGGATGGAAATTTGTTTCCCAGGGG<br>..(((((....)))))...	GOLT1A	NM_198447	chr1:202433911-202449843	template	40	Intron_1.f	--	Yes	--	Yes	--	--	--	--	--	--	Yes
G4ST01202455090	chr1:202455091-202455133	-	GGGAGAGGCATGTTCTTGGGCAGGGCTGGCTACGGCAAGAGGG<br>((((.....)))). ((.((....)).)).	PLEKHA6	NM_014935	chr1:202454604-202595667	non-template	43	Exon_23.l	--	Yes	Yes	--	--	--	Yes	--	--	--	--
G4ST01202480740	chr1:202480741-202480777	+	GGGGCCAAGTCCTCCTTGGGGGAAACAAGGGGATGGG<br>.(((((.....)))))	PLEKHA6	NM_014935	chr1:202454604-202595667	template	37	Intron_14-Exon_15	--	Yes	Yes	Yes	Yes	--	--	--	--	--	--
G4ST01202512493	chr1:202512494-202512525	-	GGGGTTGCTGGGTAGGAAAGTCTGGGGCTGGG<br>.(((....))).	PLEKHA6	NM_014935	chr1:202454604-202595667	non-template	32	Intron_1.f	--	Yes	--	Yes	--	--	--	--	Yes	--	--
G4ST01202528311	chr1:202528312-202528346	-	GGGGGGCATCAGCCAGGGCGAGGGACACAGGTGGG<br>.(((....))). ((....))	PLEKHA6	NM_014935	chr1:202454604-202595667	non-template	35	Intron_1.f	--	Yes	--	Yes	--	--	--	--	--	--	--
G4ST01202528322	chr1:202528323-202528353	-	GGGAAATGGGGGGCATCAGCCAGGGCGAGGG<br>.(((....))).	PLEKHA6	NM_014935	chr1:202454604-202595667	non-template	31	Intron_1.f	--	Yes	--	Yes	--	--	--	--	--	--	--
G4ST01202528328	chr1:202528329-202528359	-	GGGTGAGGGAAATGGGGGGCATCAGCCAGGG<br>.(((....))).	PLEKHA6	NM_014935	chr1:202454604-202595667	non-template	31	Intron_1.f	--	Yes	--	Yes	--	--	--	--	--	--	--
G4ST01202561068	chr1:202561069-202561104	+	GGGTTTAGAGGGCTGCCAGGGGGAGAATCCTTAGGG<br>(((...)))...	PLEKHA6	NM_014935	chr1:202454604-202595667	template	36	Intron_1.f	--	Yes	--	Yes	--	--	--	--	--	--	--
G4ST01202571122	chr1:202571123-202571153	-	GGGGAAGGAAGGGAGGGACTTGGTCCAAGGG<br>..(((...)))	PLEKHA6	NM_014935	chr1:202454604-202595667	non-template	31	Intron_1.f	--	Yes	--	Yes	--	--	--	Yes	Yes	--	--
G4ST01202594558	chr1:202594559-202594587	-	GGGCAGGGAAGGGGGCTGTGGAGCCTGGG<br>((((....)))).	PLEKHA6	NM_014935	chr1:202454604-202595667	non-template	29	Intron_1.f	--	Yes	--	Yes	--	--	--	Yes	--	--	--
G4ST01202680029	chr1:202680030-202680064	+	GGGATACTGGGGATGGGACCAGCAAAACTGGAGGG<br>.((((.....)))).	PIK3C2B	NM_002646	chr1:202658381-202726097	template	35	Intron_18	--	Yes	--	Yes	--	--	--	Yes	--	--	--
G4ST01202704679	chr1:202704680-202704713	+	GGGAGGCATAGGTGCGGGGGGGCACCTGAGGGGG<br>..(((....)))	PIK3C2B	NM_002646	chr1:202658381-202726097	template	34	Exon_3	--	Yes	Yes	--	Yes	--	--	--	Yes	--	--
G4ST01202714004	chr1:202714005-202714044	-	GGGTCCCTGGCTGCAAAGGAGGTGGGATTGGGGGTATGGG<br>..(((..((....)).))).	PIK3C2B	NM_002646	chr1:202658381-202726097	non-template	40	Intron_2	--	Yes	--	Yes	--	--	--	Yes	--	--	Yes
G4ST01202716876	chr1:202716877-202716905	-	GGGTGTGAGGGGTATGGGATAAGCATGGG<br>((....))	PIK3C2B	NM_002646	chr1:202658381-202726097	non-template	29	Intron_2	--	Yes	--	Yes	--	--	--	Yes	Yes	--	Yes
G4ST01202717507	chr1:202717508-202717547	-	GGGGGGCCTGCCAGAAGGCCTGGGGGCATTTGGGAGAGGG<br>.(((((......)))))...	PIK3C2B	NM_002646	chr1:202658381-202726097	non-template	40	Intron_2	--	Yes	--	Yes	--	--	--	Yes	Yes	--	--
G4ST01202859095	chr1:202859096-202859129	-	GGGAAGGAAGGGGCAGCAGTCTGGGGTGAAGGGG<br>.(((....)))	LRRN2	NM_006338	chr1:202852926-202921104	non-template	34	Intron_2.l	--	Yes	--	Yes	--	--	--	Yes	Yes	--	--
G4ST01202859095	chr1:202859096-202859129	-	GGGAAGGAAGGGGCAGCAGTCTGGGGTGAAGGGG<br>.(((....)))	LRRN2	NM_201630	chr1:202852926-202921220	non-template	34	Intron_1.u	--	Yes	--	Yes	--	--	--	Yes	Yes	--	--
G4ST01202859103	chr1:202859104-202859134	-	GGGGAGGGAAGGAAGGGGCAGCAGTCTGGGG<br>.(((....)))	LRRN2	NM_006338	chr1:202852926-202921104	non-template	31	Intron_2.l	--	Yes	--	Yes	--	--	--	Yes	Yes	--	--
G4ST01202859103	chr1:202859104-202859134	-	GGGGAGGGAAGGAAGGGGCAGCAGTCTGGGG<br>.(((....)))	LRRN2	NM_201630	chr1:202852926-202921220	non-template	31	Intron_1.u	--	Yes	--	Yes	--	--	--	Yes	Yes	--	--
G4ST01202866444	chr1:202866445-202866477	-	GGGGAGGGGCACAGGAGAGGTCCTGGGGAAGGG<br>...(((((....)))))	LRRN2	NM_006338	chr1:202852926-202921104	non-template	33	Intron_2.l	--	Yes	--	Yes	--	--	--	--	Yes	--	--
G4ST01202866444	chr1:202866445-202866477	-	GGGGAGGGGCACAGGAGAGGTCCTGGGGAAGGG<br>...(((((....)))))	LRRN2	NM_201630	chr1:202852926-202921220	non-template	33	Intron_1.u	--	Yes	--	Yes	--	--	--	--	Yes	--	--
G4ST01202866449	chr1:202866450-202866483	-	GGGACTGGGGAGGGGCACAGGAGAGGTCCTGGGG<br>...(((((....)))))	LRRN2	NM_006338	chr1:202852926-202921104	non-template	34	Intron_2.l	--	Yes	--	Yes	--	--	--	--	Yes	--	--
G4ST01202866449	chr1:202866450-202866483	-	GGGACTGGGGAGGGGCACAGGAGAGGTCCTGGGG<br>...(((((....)))))	LRRN2	NM_201630	chr1:202852926-202921220	non-template	34	Intron_1.u	--	Yes	--	Yes	--	--	--	--	Yes	--	--
G4ST01202910399	chr1:202910400-202910430	+	GGGCAGGTGCAGCCTGGGCCAGTGGGCTGGG<br>.(((.....)))	LRRN2	NM_006338	chr1:202852926-202921104	template	31	Intron_2.l	--	Yes	--	Yes	--	--	--	--	--	--	--
G4ST01202910399	chr1:202910400-202910430	+	GGGCAGGTGCAGCCTGGGCCAGTGGGCTGGG<br>.(((.....)))	LRRN2	NM_201630	chr1:202852926-202921220	template	31	Intron_1.u	--	Yes	--	Yes	--	--	--	--	--	--	--
G4ST01203064344	chr1:203064345-203064373	+	GGGAGGGGCGCGGGGGCGCGCACGCAGGG<br>.(((....))).	NFASC	NM_015090	chr1:203064405-203258573	non-template	-31	upstream	Yes	--	--	--	--	--	--	Yes	Yes	--	--
G4ST01203064344	chr1:203064345-203064373	+	GGGAGGGGCGCGGGGGCGCGCACGCAGGG<br>.(((....))).	NFASC	NM_001160333	chr1:203064405-203212897	non-template	-31	upstream	Yes	--	--	--	--	--	--	Yes	Yes	--	--

G4ST01203064344	chr1:203064345-203064373	+	GGGAGGGGCGCGGGGGCGCGCACGCAGGG<br>.(((....))).	NFASC	NM_001160332	chr1:203064405-203258573	non-template	-31	upstream	Yes	--	--	--	--	--	--	Yes	Yes	--	--
G4ST01203064344	chr1:203064345-203064373	+	GGGAGGGGCGCGGGGGCGCGCACGCAGGG<br>.(((....))).	NFASC	NM_001005389	chr1:203064405-203212897	non-template	-31	upstream	Yes	--	--	--	--	--	--	Yes	Yes	--	--
G4ST01203064344	chr1:203064345-203064373	+	GGGAGGGGCGCGGGGGCGCGCACGCAGGG<br>.(((....))).	NFASC	NM_001005388	chr1:203064405-203258573	non-template	-31	upstream	Yes	--	--	--	--	--	--	Yes	Yes	--	--
G4ST01203064348	chr1:203064349-203064382	+	GGGGCGCGGGGGCGCGCACGCAGGGTGTCGGGGG<br>.(((....))).	NFASC	NM_015090	chr1:203064405-203258573	non-template	-22	upstream	Yes	--	--	--	--	--	--	Yes	Yes	--	--
G4ST01203064348	chr1:203064349-203064382	+	GGGGCGCGGGGGCGCGCACGCAGGGTGTCGGGGG<br>.(((....))).	NFASC	NM_001160333	chr1:203064405-203212897	non-template	-22	upstream	Yes	--	--	--	--	--	--	Yes	Yes	--	--
G4ST01203064348	chr1:203064349-203064382	+	GGGGCGCGGGGGCGCGCACGCAGGGTGTCGGGGG<br>.(((....))).	NFASC	NM_001160332	chr1:203064405-203258573	non-template	-22	upstream	Yes	--	--	--	--	--	--	Yes	Yes	--	--
G4ST01203064348	chr1:203064349-203064382	+	GGGGCGCGGGGGCGCGCACGCAGGGTGTCGGGGG<br>.(((....))).	NFASC	NM_001005389	chr1:203064405-203212897	non-template	-22	upstream	Yes	--	--	--	--	--	--	Yes	Yes	--	--
G4ST01203064348	chr1:203064349-203064382	+	GGGGCGCGGGGGCGCGCACGCAGGGTGTCGGGGG<br>.(((....))).	NFASC	NM_001005388	chr1:203064405-203258573	non-template	-22	upstream	Yes	--	--	--	--	--	--	Yes	Yes	--	--
G4ST01203067187	chr1:203067188-203067210	+	GGGGTGGGTGGGCCAGAGGAGGG<br>((...)).	NFASC	NM_015090	chr1:203064405-203258573	non-template	23	Intron_1.f	--	Yes	--	Yes	--	--	--	Yes	--	--	--
G4ST01203067187	chr1:203067188-203067210	+	GGGGTGGGTGGGCCAGAGGAGGG<br>((...)).	NFASC	NM_001160333	chr1:203064405-203212897	non-template	23	Intron_1.f	--	Yes	--	Yes	--	--	--	Yes	--	--	--
G4ST01203067187	chr1:203067188-203067210	+	GGGGTGGGTGGGCCAGAGGAGGG<br>((...)).	NFASC	NM_001160332	chr1:203064405-203258573	non-template	23	Intron_1.f	--	Yes	--	Yes	--	--	--	Yes	--	--	--
G4ST01203067187	chr1:203067188-203067210	+	GGGGTGGGTGGGCCAGAGGAGGG<br>((...)).	NFASC	NM_001005389	chr1:203064405-203212897	non-template	23	Intron_1.f	--	Yes	--	Yes	--	--	--	Yes	--	--	--
G4ST01203067187	chr1:203067188-203067210	+	GGGGTGGGTGGGCCAGAGGAGGG<br>((...)).	NFASC	NM_001005388	chr1:203064405-203258573	non-template	23	Intron_1.f	--	Yes	--	Yes	--	--	--	Yes	--	--	--
G4ST01203080803	chr1:203080804-203080838	-	GGGCACAGAGGGAAGTAGGGGGAAAGTCCCTTGGG<br>(((...)))...	NFASC	NM_015090	chr1:203064405-203258573	template	35	Intron_1.f	--	Yes	--	Yes	--	--	--	--	Yes	--	--
G4ST01203080803	chr1:203080804-203080838	-	GGGCACAGAGGGAAGTAGGGGGAAAGTCCCTTGGG<br>(((...)))...	NFASC	NM_001160333	chr1:203064405-203212897	template	35	Intron_1.f	--	Yes	--	Yes	--	--	--	--	Yes	--	--
G4ST01203080803	chr1:203080804-203080838	-	GGGCACAGAGGGAAGTAGGGGGAAAGTCCCTTGGG<br>(((...)))...	NFASC	NM_001160332	chr1:203064405-203258573	template	35	Intron_1.f	--	Yes	--	Yes	--	--	--	--	Yes	--	--
G4ST01203080803	chr1:203080804-203080838	-	GGGCACAGAGGGAAGTAGGGGGAAAGTCCCTTGGG<br>(((...)))...	NFASC	NM_001005389	chr1:203064405-203212897	template	35	Intron_1.f	--	Yes	--	Yes	--	--	--	--	Yes	--	--
G4ST01203080803	chr1:203080804-203080838	-	GGGCACAGAGGGAAGTAGGGGGAAAGTCCCTTGGG<br>(((...)))...	NFASC	NM_001005388	chr1:203064405-203258573	template	35	Intron_1.f	--	Yes	--	Yes	--	--	--	--	Yes	--	--
G4ST01203093273	chr1:203093274-203093311	-	GGGGGCTGCGACAGGGGAGACAGAGGGCACTCGAGGGG<br>..(((...)))	NFASC	NM_015090	chr1:203064405-203258573	template	38	Intron_1.f	--	Yes	--	Yes	--	--	--	--	--	--	--
G4ST01203093273	chr1:203093274-203093311	-	GGGGGCTGCGACAGGGGAGACAGAGGGCACTCGAGGGG<br>..(((...)))	NFASC	NM_001160333	chr1:203064405-203212897	template	38	Intron_1.f	--	Yes	--	Yes	--	--	--	--	--	--	--
G4ST01203093273	chr1:203093274-203093311	-	GGGGGCTGCGACAGGGGAGACAGAGGGCACTCGAGGGG<br>..(((...)))	NFASC	NM_001160332	chr1:203064405-203258573	template	38	Intron_1.f	--	Yes	--	Yes	--	--	--	--	--	--	--
G4ST01203093273	chr1:203093274-203093311	-	GGGGGCTGCGACAGGGGAGACAGAGGGCACTCGAGGGG<br>..(((...)))	NFASC	NM_001005389	chr1:203064405-203212897	template	38	Intron_1.f	--	Yes	--	Yes	--	--	--	--	--	--	--
G4ST01203093273	chr1:203093274-203093311	-	GGGGGCTGCGACAGGGGAGACAGAGGGCACTCGAGGGG<br>..(((...)))	NFASC	NM_001005388	chr1:203064405-203258573	template	38	Intron_1.f	--	Yes	--	Yes	--	--	--	--	--	--	--
G4ST01203093284	chr1:203093285-203093319	-	GGGATTCTGGGGGCTGCGACAGGGGAGACAGAGGG<br>..(((...)))	NFASC	NM_015090	chr1:203064405-203258573	template	35	Intron_1.f	--	Yes	--	Yes	--	--	--	--	--	--	--
G4ST01203093284	chr1:203093285-203093319	-	GGGATTCTGGGGGCTGCGACAGGGGAGACAGAGGG<br>..(((...)))	NFASC	NM_001160333	chr1:203064405-203212897	template	35	Intron_1.f	--	Yes	--	Yes	--	--	--	--	--	--	--
G4ST01203093284	chr1:203093285-203093319	-	GGGATTCTGGGGGCTGCGACAGGGGAGACAGAGGG<br>..(((...)))	NFASC	NM_001160332	chr1:203064405-203258573	template	35	Intron_1.f	--	Yes	--	Yes	--	--	--	--	--	--	--
G4ST01203093284	chr1:203093285-203093319	-	GGGATTCTGGGGGCTGCGACAGGGGAGACAGAGGG<br>..(((...)))	NFASC	NM_001005389	chr1:203064405-203212897	template	35	Intron_1.f	--	Yes	--	Yes	--	--	--	--	--	--	--
G4ST01203093284	chr1:203093285-203093319	-	GGGATTCTGGGGGCTGCGACAGGGGAGACAGAGGG<br>..(((...)))	NFASC	NM_001005388	chr1:203064405-203258573	template	35	Intron_1.f	--	Yes	--	Yes	--	--	--	--	--	--	--
G4ST01203094136	chr1:203094137-203094177	+	GGGGGAAGGGCATCTGGGAGAGGCCTTGTGGTTCCTCTGGG<br>(((((..........)))))	NFASC	NM_015090	chr1:203064405-203258573	non-template	41	Intron_1.f	--	Yes	--	Yes	--	--	--	Yes	--	--	Yes
G4ST01203094136	chr1:203094137-203094177	+	GGGGGAAGGGCATCTGGGAGAGGCCTTGTGGTTCCTCTGGG<br>(((((..........)))))	NFASC	NM_001160333	chr1:203064405-203212897	non-template	41	Intron_1.f	--	Yes	--	Yes	--	--	--	Yes	--	--	Yes
G4ST01203094136	chr1:203094137-203094177	+	GGGGGAAGGGCATCTGGGAGAGGCCTTGTGGTTCCTCTGGG<br>(((((..........)))))	NFASC	NM_001160332	chr1:203064405-203258573	non-template	41	Intron_1.f	--	Yes	--	Yes	--	--	--	Yes	--	--	Yes
G4ST01203094136	chr1:203094137-203094177	+	GGGGGAAGGGCATCTGGGAGAGGCCTTGTGGTTCCTCTGGG<br>(((((..........)))))	NFASC	NM_001005389	chr1:203064405-203212897	non-template	41	Intron_1.f	--	Yes	--	Yes	--	--	--	Yes	--	--	Yes
G4ST01203094136	chr1:203094137-203094177	+	GGGGGAAGGGCATCTGGGAGAGGCCTTGTGGTTCCTCTGGG<br>(((((..........)))))	NFASC	NM_001005388	chr1:203064405-203258573	non-template	41	Intron_1.f	--	Yes	--	Yes	--	--	--	Yes	--	--	Yes
G4ST01203174272	chr1:203174273-203174302	-	GGGAGCCGGCGGCAGGGTGGGGGGCTTGGG<br>.(((...))).	NFASC	NM_015090	chr1:203064405-203258573	template	30	Intron_2	--	Yes	--	Yes	--	--	--	--	--	--	--
G4ST01203174272	chr1:203174273-203174302	-	GGGAGCCGGCGGCAGGGTGGGGGGCTTGGG<br>.(((...))).	NFASC	NM_001160333	chr1:203064405-203212897	template	30	Intron_2	--	Yes	--	Yes	--	--	--	--	--	--	--
G4ST01203174272	chr1:203174273-203174302	-	GGGAGCCGGCGGCAGGGTGGGGGGCTTGGG<br>.(((...))).	NFASC	NM_001160332	chr1:203064405-203258573	template	30	Intron_2	--	Yes	--	Yes	--	--	--	--	--	--	--
G4ST01203174272	chr1:203174273-203174302	-	GGGAGCCGGCGGCAGGGTGGGGGGCTTGGG<br>.(((...))).	NFASC	NM_001005389	chr1:203064405-203212897	template	30	Intron_2	--	Yes	--	Yes	--	--	--	--	--	--	--
G4ST01203174272	chr1:203174273-203174302	-	GGGAGCCGGCGGCAGGGTGGGGGGCTTGGG<br>.(((...))).	NFASC	NM_001005388	chr1:203064405-203258573	template	30	Intron_2	--	Yes	--	Yes	--	--	--	--	--	--	--
G4ST01203243347	chr1:203243348-203243381	+	GGGAGTTGGGGAGAGGGGCTTTGTTTAAGCTGGG<br>((((.....)))).	NFASC	NM_001160331	chr1:203179977-203258573	non-template	34	Intron_24	--	Yes	--	Yes	--	--	--	--	--	--	Yes
G4ST01203243347	chr1:203243348-203243381	+	GGGAGTTGGGGAGAGGGGCTTTGTTTAAGCTGGG<br>((((.....)))).	NFASC	NM_015090	chr1:203064405-203258573	non-template	34	Intron_24	--	Yes	--	Yes	--	--	--	--	--	--	Yes
G4ST01203243347	chr1:203243348-203243381	+	GGGAGTTGGGGAGAGGGGCTTTGTTTAAGCTGGG<br>((((.....)))).	NFASC	NM_001160332	chr1:203064405-203258573	non-template	34	Intron_25	--	Yes	--	Yes	--	--	--	--	--	--	Yes
G4ST01203243347	chr1:203243348-203243381	+	GGGAGTTGGGGAGAGGGGCTTTGTTTAAGCTGGG<br>((((.....)))).	NFASC	NM_001005388	chr1:203064405-203258573	non-template	34	Intron_27	--	Yes	--	Yes	--	--	--	--	--	--	Yes
G4ST01203278647	chr1:203278648-203278675	+	GGGCAGGGGGACTAGAAGTGGGGCTGGG<br>..(((...))).	CNTN2	NM_005076	chr1:203278963-203313794	non-template	-287	upstream	Yes	--	--	--	--	--	--	--	--	--	Yes
G4ST01203278652	chr1:203278653-203278685	+	GGGGGACTAGAAGTGGGGCTGGGCAGGATCGGG<br>..(((...))).	CNTN2	NM_005076	chr1:203278963-203313794	non-template	-277	upstream	Yes	--	--	--	--	--	--	--	--	--	Yes
G4ST01203284164	chr1:203284165-203284204	-	GGGGGAGTGGACCACTGGGGGGTTCGGGGGCACCAGGGGG<br>..((((...))))...	CNTN2	NM_005076	chr1:203278963-203313794	template	40	Intron_1.f	--	Yes	--	Yes	--	--	--	--	--	--	--
G4ST01203284779	chr1:203284780-203284818	-	GGGGCTGGGATCCAGGAGCACCTGGGGTTGCCCTGGGGG<br>...((((....))))	CNTN2	NM_005076	chr1:203278963-203313794	template	39	Intron_1.f	--	Yes	--	Yes	--	--	--	--	--	--	--
G4ST01203284791	chr1:203284792-203284823	-	GGGGAGGGGCTGGGATCCAGGAGCACCTGGGG<br>...((((....))))	CNTN2	NM_005076	chr1:203278963-203313794	template	32	Intron_1.f	--	Yes	--	Yes	--	--	--	--	--	--	--
G4ST01203297033	chr1:203297034-203297069	-	GGGTGGGCTCAGCTGTGGTCCACTGCGGGGACAGGG<br>....((.(((...))).)).	CNTN2	NM_005076	chr1:203278963-203313794	template	36	Exon_8	--	Yes	Yes	--	Yes	--	--	--	--	--	--
G4ST01203297039	chr1:203297040-203297080	-	GGGGATCTGCAGGGTGGGCTCAGCTGTGGTCCACTGCGGGG<br>....((.(((...))).)).	CNTN2	NM_005076	chr1:203278963-203313794	template	41	Exon_8	--	Yes	Yes	--	Yes	--	--	--	--	--	--
G4ST01203307900	chr1:203307901-203307930	+	GGGGTGGGGGTAGGGCAATATTTTGGAGGG<br>(((....)))..	CNTN2	NM_005076	chr1:203278963-203313794	non-template	30	Intron_20	--	Yes	--	Yes	--	--	--	--	--	--	--

G4ST01203307905	chr1:203307906-203307934	+	GGGGGTAGGGCAATATTTTGGAGGGTGGG<br>(((....)))..	CNTN2	NM_005076	chr1:203278963-203313794	non-template	29	Intron_20	--	Yes	--	Yes	--	--	--	--	--	--	--
G4ST01203446749	chr1:203446750-203446787	-	GGGCCGGAGTTCCCGGAGCGGGACCCCGGGGACCGGGG<br>((((.....))))...	DSTYK	NM_199462	chr1:203378254-203447350	non-template	38	Intron_1.f	--	Yes	--	Yes	--	--	--	Yes	Yes	Yes	Yes
G4ST01203446749	chr1:203446750-203446787	-	GGGCCGGAGTTCCCGGAGCGGGACCCCGGGGACCGGGG<br>((((.....))))...	DSTYK	NM_015375	chr1:203378254-203447350	non-template	38	Intron_1.f	--	Yes	--	Yes	--	--	--	Yes	Yes	Yes	Yes
G4ST01203446756	chr1:203446757-203446798	-	GGGGCAACCGAGGGCCGGAGTTCCCGGAGCGGGACCCCGGGG<br>((((.....))))...	DSTYK	NM_199462	chr1:203378254-203447350	non-template	42	Intron_1.f	--	Yes	--	Yes	--	--	--	Yes	Yes	Yes	Yes
G4ST01203446756	chr1:203446757-203446798	-	GGGGCAACCGAGGGCCGGAGTTCCCGGAGCGGGACCCCGGGG<br>((((.....))))...	DSTYK	NM_015375	chr1:203378254-203447350	non-template	42	Intron_1.f	--	Yes	--	Yes	--	--	--	Yes	Yes	Yes	Yes
G4ST01203446765	chr1:203446766-203446806	-	GGGAGCCAGGGGCAACCGAGGGCCGGAGTTCCCGGAGCGGG<br>((((.....))))...	DSTYK	NM_199462	chr1:203378254-203447350	non-template	41	Intron_1.f	--	Yes	--	Yes	--	--	--	Yes	Yes	Yes	Yes
G4ST01203446765	chr1:203446766-203446806	-	GGGAGCCAGGGGCAACCGAGGGCCGGAGTTCCCGGAGCGGG<br>((((.....))))...	DSTYK	NM_015375	chr1:203378254-203447350	non-template	41	Intron_1.f	--	Yes	--	Yes	--	--	--	Yes	Yes	Yes	Yes
G4ST01203446809	chr1:203446810-203446850	-	GGGCATCCTTCTGAGGAGGGGAGGAAGGGGCCCGCTGGGGG<br>..((((....)))).	DSTYK	NM_199462	chr1:203378254-203447350	non-template	41	Intron_1.f	--	Yes	--	Yes	--	--	--	--	Yes	Yes	Yes
G4ST01203446809	chr1:203446810-203446850	-	GGGCATCCTTCTGAGGAGGGGAGGAAGGGGCCCGCTGGGGG<br>..((((....)))).	DSTYK	NM_015375	chr1:203378254-203447350	non-template	41	Intron_1.f	--	Yes	--	Yes	--	--	--	--	Yes	Yes	Yes
G4ST01203446820	chr1:203446821-203446854	-	GGGAGGGCATCCTTCTGAGGAGGGGAGGAAGGGG<br>..((((....)))).	DSTYK	NM_199462	chr1:203378254-203447350	non-template	34	Intron_1.f	--	Yes	--	Yes	--	--	--	--	Yes	Yes	Yes
G4ST01203446820	chr1:203446821-203446854	-	GGGAGGGCATCCTTCTGAGGAGGGGAGGAAGGGG<br>..((((....)))).	DSTYK	NM_015375	chr1:203378254-203447350	non-template	34	Intron_1.f	--	Yes	--	Yes	--	--	--	--	Yes	Yes	Yes
G4ST01203446829	chr1:203446830-203446864	-	GGGATACCTTGGGAGGGCATCCTTCTGAGGAGGGG<br>..((((....)))).	DSTYK	NM_199462	chr1:203378254-203447350	non-template	35	Intron_1.f	--	Yes	--	Yes	--	--	--	--	Yes	Yes	Yes
G4ST01203446829	chr1:203446830-203446864	-	GGGATACCTTGGGAGGGCATCCTTCTGAGGAGGGG<br>..((((....)))).	DSTYK	NM_015375	chr1:203378254-203447350	non-template	35	Intron_1.f	--	Yes	--	Yes	--	--	--	--	Yes	Yes	Yes
G4ST01203447532	chr1:203447533-203447564	+	GGGGTCTCCTGGGACTTGCAGTCCGGGCTGGG<br>(((...)))..	DSTYK	NM_199462	chr1:203378254-203447350	template	-182	upstream	Yes	--	--	--	--	--	--	Yes	Yes	Yes	--
G4ST01203447532	chr1:203447533-203447564	+	GGGGTCTCCTGGGACTTGCAGTCCGGGCTGGG<br>(((...)))..	DSTYK	NM_015375	chr1:203378254-203447350	template	-182	upstream	Yes	--	--	--	--	--	--	Yes	Yes	Yes	--
G4ST01203463468	chr1:203463469-203463498	-	GGGCTGCAGTTCAGGGGCGGGGGCGGCGGG<br>(((.....)))	TMCC2	NM_014858	chr1:203463661-203509094	template	-162	upstream	Yes	--	--	--	--	--	--	Yes	Yes	Yes	Yes
G4ST01203463776	chr1:203463777-203463802	+	GGGGAGGGGGCCGGGCGCGCTGCGGG<br>.((...))	TMCC2	NM_014858	chr1:203463661-203509094	non-template	26	Exon_1.f	--	Yes	Yes	--	--	Yes	--	--	Yes	Yes	Yes
G4ST01203470922	chr1:203470923-203470948	-	GGGGGTGGGGGGGAGGCAATCCTGGG<br>(((....)))	TMCC2	NM_014858	chr1:203463661-203509094	template	26	Intron_1.f	--	Yes	--	Yes	--	--	--	--	--	--	--
G4ST01203498795	chr1:203498796-203498834	-	GGGAGGCGGTGGGGGGTGCTTGAGGGAACACTCTGTGGG<br>.(((...)))	TMCC2	NM_014858	chr1:203463661-203509094	template	39	Intron_2	--	Yes	--	Yes	--	--	--	Yes	--	--	--
G4ST01203539755	chr1:203539756-203539792	+	GGGGAGCAGGGGGGCAGCCTGCCCTGGGCTCGCAGGG<br>(((((...))))).	NUAK2	NM_030952	chr1:203537814-203557506	template	37	Exon_7.l	--	Yes	Yes	--	Yes	--	--	--	--	--	--
G4ST01203539763	chr1:203539764-203539798	+	GGGGGGCAGCCTGCCCTGGGCTCGCAGGGATTGGG<br>(((((...))))).	NUAK2	NM_030952	chr1:203537814-203557506	template	35	Exon_7.l	--	Yes	Yes	--	Yes	--	--	--	--	--	--
G4ST01203539780	chr1:203539781-203539813	+	GGGCTCGCAGGGATTGGGCTGAGCTCCGGAGGG<br>(((.....))).	NUAK2	NM_030952	chr1:203537814-203557506	template	33	Exon_7.l	--	Yes	Yes	--	Yes	--	--	--	--	--	--
G4ST01203555271	chr1:203555272-203555304	-	GGGGGTGGAGACACTGGGTCGTGGGGACTGGGG<br>.(((....))).	NUAK2	NM_030952	chr1:203537814-203557506	non-template	33	Intron_1.f	--	Yes	--	Yes	--	--	--	--	Yes	--	--
G4ST01203748494	chr1:203748495-203748532	+	GGGGTTCTTGTCAGAATGTGGGCACTGGGCGGCGTGGG<br>(((((....)))))..	CDK18	NM_212502	chr1:203740307-203768544	non-template	38	Intron_1.f	--	Yes	--	Yes	--	--	--	--	--	--	--
G4ST01203748494	chr1:203748495-203748532	+	GGGGTTCTTGTCAGAATGTGGGCACTGGGCGGCGTGGG<br>(((((....)))))..	CDK18	NM_212503	chr1:203740307-203768544	non-template	38	Intron_1.f	--	Yes	--	Yes	--	--	--	--	--	--	--
G4ST01203748494	chr1:203748495-203748532	+	GGGGTTCTTGTCAGAATGTGGGCACTGGGCGGCGTGGG<br>(((((....)))))..	CDK18	NM_002596	chr1:203740307-203768544	non-template	38	Intron_1.f	--	Yes	--	Yes	--	--	--	--	--	--	--
G4ST01203748513	chr1:203748514-203748546	+	GGGCACTGGGCGGCGTGGGCCAGCTGGTGGGGG<br>(((.....)))	CDK18	NM_212502	chr1:203740307-203768544	non-template	33	Intron_1.f	--	Yes	--	Yes	--	--	--	--	--	--	--
G4ST01203748513	chr1:203748514-203748546	+	GGGCACTGGGCGGCGTGGGCCAGCTGGTGGGGG<br>(((.....)))	CDK18	NM_212503	chr1:203740307-203768544	non-template	33	Intron_1.f	--	Yes	--	Yes	--	--	--	--	--	--	--
G4ST01203748513	chr1:203748514-203748546	+	GGGCACTGGGCGGCGTGGGCCAGCTGGTGGGGG<br>(((.....)))	CDK18	NM_002596	chr1:203740307-203768544	non-template	33	Intron_1.f	--	Yes	--	Yes	--	--	--	--	--	--	--
G4ST01203759919	chr1:203759920-203759961	-	GGGGACACCACTGTTGGTTTCAGGGTAGCTAGGGCTGTGGGG<br>((.((((....)))).)).	CDK18	NM_212502	chr1:203740307-203768544	template	42	Intron_3	--	Yes	--	Yes	--	--	--	--	Yes	--	--
G4ST01203759919	chr1:203759920-203759961	-	GGGGACACCACTGTTGGTTTCAGGGTAGCTAGGGCTGTGGGG<br>((.((((....)))).)).	CDK18	NM_212503	chr1:203740307-203768544	template	42	Intron_3	--	Yes	--	Yes	--	--	--	--	Yes	--	--
G4ST01203759919	chr1:203759920-203759961	-	GGGGACACCACTGTTGGTTTCAGGGTAGCTAGGGCTGTGGGG<br>((.((((....)))).)).	CDK18	NM_002596	chr1:203740307-203768544	template	42	Intron_3	--	Yes	--	Yes	--	--	--	--	Yes	--	--
G4ST01203759927	chr1:203759928-203759971	-	GGGATGAGATGGGGACACCACTGTTGGTTTCAGGGTAGCTAGGG<br>((.((((....)))).)).	CDK18	NM_212502	chr1:203740307-203768544	template	44	Intron_3	--	Yes	--	Yes	--	--	--	--	Yes	--	--
G4ST01203759927	chr1:203759928-203759971	-	GGGATGAGATGGGGACACCACTGTTGGTTTCAGGGTAGCTAGGG<br>((.((((....)))).)).	CDK18	NM_212503	chr1:203740307-203768544	template	44	Intron_3	--	Yes	--	Yes	--	--	--	--	Yes	--	--
G4ST01203759927	chr1:203759928-203759971	-	GGGATGAGATGGGGACACCACTGTTGGTTTCAGGGTAGCTAGGG<br>((.((((....)))).)).	CDK18	NM_002596	chr1:203740307-203768544	template	44	Intron_3	--	Yes	--	Yes	--	--	--	--	Yes	--	--
G4ST01203763390	chr1:203763391-203763419	+	GGGTGGGGTCCAGGATGGGGGCTGCAGGG<br>..(((...)))	CDK18	NM_212502	chr1:203740307-203768544	non-template	29	Intron_8	--	Yes	--	Yes	--	--	--	--	--	--	--
G4ST01203763390	chr1:203763391-203763419	+	GGGTGGGGTCCAGGATGGGGGCTGCAGGG<br>..(((...)))	CDK18	NM_212503	chr1:203740307-203768544	non-template	29	Intron_8	--	Yes	--	Yes	--	--	--	--	--	--	--
G4ST01203763390	chr1:203763391-203763419	+	GGGTGGGGTCCAGGATGGGGGCTGCAGGG<br>..(((...)))	CDK18	NM_002596	chr1:203740307-203768544	non-template	29	Intron_8	--	Yes	--	Yes	--	--	--	--	--	--	--
G4ST01203766514	chr1:203766515-203766547	-	GGGGGGACCATGGGAGTCCCCACTGGGGAGGGG<br>(((....))).	CDK18	NM_212502	chr1:203740307-203768544	template	33	Intron_15.l	--	Yes	--	Yes	--	--	--	--	--	--	--
G4ST01203766514	chr1:203766515-203766547	-	GGGGGGACCATGGGAGTCCCCACTGGGGAGGGG<br>(((....))).	CDK18	NM_212503	chr1:203740307-203768544	template	33	Intron_15.l	--	Yes	--	Yes	--	--	--	--	--	--	--
G4ST01203766514	chr1:203766515-203766547	-	GGGGGGACCATGGGAGTCCCCACTGGGGAGGGG<br>(((....))).	CDK18	NM_002596	chr1:203740307-203768544	template	33	Intron_15.l	--	Yes	--	Yes	--	--	--	--	--	--	--
G4ST01203767043	chr1:203767044-203767074	-	GGGGCTGCAGGGAGGAGGCTGGGCTGACGGG<br>((....))	CDK18	NM_212502	chr1:203740307-203768544	template	31	Intron_15.l	--	Yes	--	Yes	--	--	--	--	--	--	--
G4ST01203767043	chr1:203767044-203767074	-	GGGGCTGCAGGGAGGAGGCTGGGCTGACGGG<br>((....))	CDK18	NM_212503	chr1:203740307-203768544	template	31	Intron_15.l	--	Yes	--	Yes	--	--	--	--	--	--	--
G4ST01203767043	chr1:203767044-203767074	-	GGGGCTGCAGGGAGGAGGCTGGGCTGACGGG<br>((....))	CDK18	NM_002596	chr1:203740307-203768544	template	31	Intron_15.l	--	Yes	--	Yes	--	--	--	--	--	--	--
G4ST01203804550	chr1:203804551-203804587	+	GGGGGAGGGCGAGCATGTGCGCTCGGGTAGGAGGGGG<br>.((((......))))	MFSD4	NM_181644	chr1:203804735-203838669	non-template	-147	upstream	Yes	--	--	--	--	--	--	--	--	--	--
G4ST01203805163	chr1:203805164-203805197	-	GGGGTGGTCGCGGCCGGGAGGCGGGGGCGACGGG<br>..(((....)))	MFSD4	NM_181644	chr1:203804735-203838669	template	34	Intron_1.f	--	Yes	--	Yes	--	--	--	--	--	--	--
G4ST01203805170	chr1:203805171-203805201	-	GGGAGGGGTGGTCGCGGCCGGGAGGCGGGGG<br>..(((....)))	MFSD4	NM_181644	chr1:203804735-203838669	template	31	Intron_1.f	--	Yes	--	Yes	--	--	--	--	--	--	--
G4ST01203811944	chr1:203811945-203811973	+	GGGGCAGGCCTGGGAGGGTAGAATCTGGG<br>.((...))	MFSD4	NM_181644	chr1:203804735-203838669	non-template	29	Intron_1.f	--	Yes	--	Yes	--	--	--	--	--	--	--
G4ST01203813752	chr1:203813753-203813787	-	GGGACCTGGGATCAGTAAACTGACAGGGGCAGGGG<br>.((((....))))...	MFSD4	NM_181644	chr1:203804735-203838669	template	35	Intron_1.f	--	Yes	--	Yes	--	--	--	--	--	--	--
G4ST01203813758	chr1:203813759-203813794	-	GGGGAACGGGACCTGGGATCAGTAAACTGACAGGGG<br>.((((....))))...	MFSD4	NM_181644	chr1:203804735-203838669	template	36	Intron_1.f	--	Yes	--	Yes	--	--	--	--	--	--	--

G4ST01203821690	chr1:203821691-203821721	-	GGGAGCCCAGGCTGGGCAGGTGTGGGGAGGG<br>(((....)))	MFSD4	NM_181644	chr1:203804735-203838669	template	31	Intron_5	--	Yes	--	Yes	--	--	--	--	--	--	Yes
G4ST01203822114	chr1:203822115-203822148	+	GGGGTGGGAGCCTGGGAGGTGGAGCCTACCTGGG<br>(((((.....)))))	MFSD4	NM_181644	chr1:203804735-203838669	non-template	34	Intron_6	--	Yes	--	Yes	--	--	--	--	--	--	--
G4ST01203905273	chr1:203905274-203905309	-	GGGGTGGGGGCTGCCCCCAGCCTGGGCTTCCACGGG<br>(((((....))))).	SLC45A3	NM_033102	chr1:203893604-203916253	non-template	36	Intron_1.f	--	Yes	--	Yes	--	--	--	--	Yes	Yes	--
G4ST01203906425	chr1:203906426-203906460	+	GGGTGAGGGGGCTGCAGGCCGGGACACAAAGGGGG<br>(((.....)))	SLC45A3	NM_033102	chr1:203893604-203916253	template	35	Intron_1.f	--	Yes	--	Yes	--	--	--	Yes	--	--	--
G4ST01203906431	chr1:203906432-203906467	+	GGGGGCTGCAGGCCGGGACACAAAGGGGGATTAGGG<br>(((.....)))	SLC45A3	NM_033102	chr1:203893604-203916253	template	36	Intron_1.f	--	Yes	--	Yes	--	--	--	Yes	--	--	--
G4ST01203915019	chr1:203915020-203915046	-	GGGGCCAGAGTTGGGGGTAGGGAGGGG<br>.(((....)))	SLC45A3	NM_033102	chr1:203893604-203916253	non-template	27	Intron_1.f	--	Yes	--	Yes	--	--	--	--	Yes	--	--
G4ST01203916337	chr1:203916338-203916371	+	GGGGGGATAGAGGGGTGGGGCTGGTACCCAGGGG<br>.((((...))))	SLC45A3	NM_033102	chr1:203893604-203916253	template	-84	upstream	Yes	--	--	--	--	--	--	Yes	Yes	Yes	--
G4ST01203956849	chr1:203956850-203956877	+	GGGGCCTGTGGCGAGGGAGGGGGCAGGG<br>(((...)))..	NUCKS1	NM_022731	chr1:203948570-203985995	template	28	Intron_4	--	Yes	--	Yes	--	--	--	--	--	--	Yes
G4ST01203985769	chr1:203985770-203985798	-	GGGGCGCCGAGCGCGCGGGGTGGGGGGGG<br>((((.....)))).	NUCKS1	NM_022731	chr1:203948570-203985995	non-template	29	Exon_1.f	--	Yes	Yes	--	--	Yes	--	Yes	Yes	Yes	--
G4ST01203985774	chr1:203985775-203985803	-	GGGGTGGGGCGCCGAGCGCGCGGGGTGGG<br>((((.....)))).	NUCKS1	NM_022731	chr1:203948570-203985995	non-template	29	Exon_1.f	--	Yes	Yes	--	--	Yes	--	--	Yes	Yes	--
G4ST01203985778	chr1:203985779-203985807	-	GGGGGGGGTGGGGCGCCGAGCGCGCGGGG<br>((((.....)))).	NUCKS1	NM_022731	chr1:203948570-203985995	non-template	29	Exon_1.f	--	Yes	Yes	--	--	Yes	--	--	Yes	Yes	--
G4ST01203987979	chr1:203987980-203988016	-	GGGGAAGGGGGAAAGTGGGGAGTTAGTGTTTAATGGG<br>..((((.....))))	NUCKS1	NM_022731	chr1:203948570-203985995	non-template	-1984	upstream	Yes	--	--	--	--	--	--	--	--	--	--
G4ST01204010555	chr1:204010556-204010588	-	GGGAGGTGAGACGTCTCGGGGGTGGGGAGGGGG<br>...((((....)))).	RAB7L1	NM_001135664	chr1:204003737-204011233	non-template	33	Intron_1.f	--	Yes	--	Yes	--	--	--	Yes	Yes	Yes	Yes
G4ST01204010555	chr1:204010556-204010588	-	GGGAGGTGAGACGTCTCGGGGGTGGGGAGGGGG<br>...((((....)))).	RAB7L1	NM_003929	chr1:204003737-204011233	non-template	33	Exon_2-Intron_2	--	Yes	Yes	Yes	Yes	--	--	Yes	Yes	Yes	Yes
G4ST01204010555	chr1:204010556-204010588	-	GGGAGGTGAGACGTCTCGGGGGTGGGGAGGGGG<br>...((((....)))).	RAB7L1	NM_001135663	chr1:204003737-204011233	non-template	33	Exon_1.f-Intron_1.f	--	Yes	Yes	Yes	Yes	--	--	Yes	Yes	Yes	Yes
G4ST01204010555	chr1:204010556-204010588	-	GGGAGGTGAGACGTCTCGGGGGTGGGGAGGGGG<br>...((((....)))).	RAB7L1	NM_001135662	chr1:204003737-204011233	non-template	33	Exon_2-Intron_2	--	Yes	Yes	Yes	Yes	--	--	Yes	Yes	Yes	Yes
G4ST01204029163	chr1:204029164-204029213	-	GGGTTCCAGTGCAGCACGTGGTGGGAAGATGGGGGTGGATATGCCAGGGG<br>..((((((...))).))). ((((....))))..	SLC41A1	NM_173854	chr1:204024844-204048784	non-template	50	Intron_10.l	--	Yes	--	Yes	--	--	--	--	--	--	--
G4ST01204048530	chr1:204048531-204048565	-	GGGGCAGGTCAGTCCTGTGGGGTCCTGGGGCGGGG<br>.((((.....))))..	SLC41A1	NM_173854	chr1:204024844-204048784	non-template	35	Exon_1.f-Intron_1.f	--	Yes	Yes	Yes	--	Yes	--	--	Yes	Yes	Yes
G4ST01204085489	chr1:204085490-204085520	+	GGGGCACGATGTGCTGGGAAGGGTGTTGGGG<br>((((...)))).	PM20D1	NM_152491	chr1:204063773-204085899	template	31	Intron_1.f	--	Yes	--	Yes	--	--	--	--	--	--	Yes
G4ST01204085599	chr1:204085600-204085634	+	GGGGAGGGAGGGACCCACATATGAGCATAGGTGGG<br>....((.(((....))).))	PM20D1	NM_152491	chr1:204063773-204085899	template	35	Intron_1.f	--	Yes	--	Yes	--	--	--	Yes	--	--	Yes
G4ST01204085604	chr1:204085605-204085642	+	GGGAGGGACCCACATATGAGCATAGGTGGGTGAAGGGG<br>....((.(((....))).))	PM20D1	NM_152491	chr1:204063773-204085899	template	38	Intron_1.f	--	Yes	--	Yes	--	--	--	Yes	--	--	Yes
G4ST01204085608	chr1:204085609-204085649	+	GGGACCCACATATGAGCATAGGTGGGTGAAGGGGACCCGGG<br>....((.(((....))).))	PM20D1	NM_152491	chr1:204063773-204085899	template	41	Intron_1.f	--	Yes	--	Yes	--	--	--	Yes	--	--	Yes
G4ST01204170598	chr1:204170599-204170635	+	GGGCTGGGCTGGGGCTTTTAGTACAAACTCAAGTGGG<br>.(((..(((....))).))).	SLC26A9	NM_052934	chr1:204148800-204179211	template	37	Intron_2	--	Yes	--	Yes	--	--	--	Yes	--	--	--
G4ST01204170598	chr1:204170599-204170635	+	GGGCTGGGCTGGGGCTTTTAGTACAAACTCAAGTGGG<br>.(((..(((....))).))).	SLC26A9	NM_134325	chr1:204148800-204179211	template	37	Intron_2	--	Yes	--	Yes	--	--	--	Yes	--	--	--
G4ST01204171911	chr1:204171912-204171947	-	GGGGAGGGGAGGGTCCACCTGCTGAATCAAGTGGGG<br>..(((.((......)).)))	SLC26A9	NM_052934	chr1:204148800-204179211	non-template	36	Intron_1.f	--	Yes	--	Yes	--	--	--	--	Yes	--	Yes
G4ST01204171911	chr1:204171912-204171947	-	GGGGAGGGGAGGGTCCACCTGCTGAATCAAGTGGGG<br>..(((.((......)).)))	SLC26A9	NM_134325	chr1:204148800-204179211	non-template	36	Intron_1.f	--	Yes	--	Yes	--	--	--	--	Yes	--	Yes
G4ST01204178932	chr1:204178933-204178958	+	GGGCTCCTCAGAGGGGAAGGGCTGGG<br>(((....)))	SLC26A9	NM_052934	chr1:204148800-204179211	template	26	Intron_1.f	--	Yes	--	Yes	--	--	--	--	--	--	--
G4ST01204178932	chr1:204178933-204178958	+	GGGCTCCTCAGAGGGGAAGGGCTGGG<br>(((....)))	SLC26A9	NM_134325	chr1:204148800-204179211	template	26	Intron_1.f	--	Yes	--	Yes	--	--	--	--	--	--	--
G4ST01204178944	chr1:204178945-204178981	+	GGGGAAGGGCTGGGAGGCTGCTCTCAGACTCCAGGGG<br>((.(((....))).))....	SLC26A9	NM_052934	chr1:204148800-204179211	template	37	Intron_1.f	--	Yes	--	Yes	--	--	--	--	--	--	--
G4ST01204178944	chr1:204178945-204178981	+	GGGGAAGGGCTGGGAGGCTGCTCTCAGACTCCAGGGG<br>((.(((....))).))....	SLC26A9	NM_134325	chr1:204148800-204179211	template	37	Intron_1.f	--	Yes	--	Yes	--	--	--	--	--	--	--
G4ST01204179137	chr1:204179138-204179169	+	GGGCAGGGCTGCAGGTGTGTCTGGGGAGCGGG<br>...((((....))))	SLC26A9	NM_052934	chr1:204148800-204179211	template	32	Exon_1.f	--	Yes	Yes	--	--	Yes	--	--	Yes	--	--
G4ST01204179137	chr1:204179138-204179169	+	GGGCAGGGCTGCAGGTGTGTCTGGGGAGCGGG<br>...((((....))))	SLC26A9	NM_134325	chr1:204148800-204179211	template	32	Exon_1.f	--	Yes	Yes	--	--	Yes	--	--	Yes	--	--
G4ST01204303788	chr1:204303789-204303822	+	GGGATGGGAGTGGGCGAAGAGAAGTCCATCGGGG<br>(((.((....))..)))	FAM72A	NM_001123168	chr1:204305534-204321697	non-template	-1711	upstream	Yes	--	--	--	--	--	--	--	--	--	Yes
G4ST01204303793	chr1:204303794-204303826	+	GGGAGTGGGCGAAGAGAAGTCCATCGGGGCGGG<br>(((.((....))..)))	FAM72A	NM_001123168	chr1:204305534-204321697	non-template	-1707	upstream	Yes	--	--	--	--	--	--	--	--	--	Yes
G4ST01204303799	chr1:204303800-204303834	+	GGGCGAAGAGAAGTCCATCGGGGCGGGCCGGGGGG<br>(((.((....))..)))	FAM72A	NM_001123168	chr1:204305534-204321697	non-template	-1699	upstream	Yes	--	--	--	--	--	--	--	--	--	Yes
G4ST01204440721	chr1:204440722-204440763	-	GGGAAACTTAAAGTTTTTCTGGGTGATCTGGGCCAAGAGGGG<br>(((((...)))))....	C1orf186	NM_001007544	chr1:204405495-204455270	non-template	42	Intron_1.f	--	Yes	--	Yes	--	--	--	--	--	Yes	--
G4ST01204442252	chr1:204442253-204442293	-	GGGACAGGAAAGTCCTGCCTGTGGGACAACAGGGATCAGGG<br>.(((((...)))))).....	C1orf186	NM_001007544	chr1:204405495-204455270	non-template	41	Intron_1.f	--	Yes	--	Yes	--	--	--	--	Yes	Yes	--
G4ST01204622758	chr1:204622759-204622789	+	GGGAGCTAGCAGCTATGGTGGGAGGGATGGG<br>((((...))))....	SRGAP2	NM_015326	chr1:204582823-204704406	non-template	31	Intron_1.f	--	Yes	--	Yes	--	--	--	--	--	--	--
G4ST01204622758	chr1:204622759-204622789	+	GGGAGCTAGCAGCTATGGTGGGAGGGATGGG<br>((((...))))....	SRGAP2	NM_001170637	chr1:204582823-204704406	non-template	31	Intron_1.f	--	Yes	--	Yes	--	--	--	--	--	--	--
G4ST01204622758	chr1:204622759-204622789	+	GGGAGCTAGCAGCTATGGTGGGAGGGATGGG<br>((((...))))....	SRGAP2	NM_001042758	chr1:204582823-204696128	non-template	31	Intron_1.f	--	Yes	--	Yes	--	--	--	--	--	--	--
G4ST01204730092	chr1:204730093-204730135	+	GGGCCCAGGAGGGGGACTCTGCTATGAGAGGAGTGGGATGGGG<br>..(((((.((...)).)))))	IKBKE	NM_014002	chr1:204710209-204736846	non-template	43	Intron_16	--	Yes	--	Yes	--	--	--	Yes	Yes	--	--
G4ST01204730092	chr1:204730093-204730135	+	GGGCCCAGGAGGGGGACTCTGCTATGAGAGGAGTGGGATGGGG<br>..(((((.((...)).)))))	IKBKE	NM_001193321	chr1:204710209-204736846	non-template	43	Intron_15	--	Yes	--	Yes	--	--	--	Yes	Yes	--	--
G4ST01204730092	chr1:204730093-204730135	+	GGGCCCAGGAGGGGGACTCTGCTATGAGAGGAGTGGGATGGGG<br>..(((((.((...)).)))))	IKBKE	NM_001193322	chr1:204710209-204736846	non-template	43	Intron_16	--	Yes	--	Yes	--	--	--	Yes	Yes	--	--
G4ST01204730938	chr1:204730939-204730971	+	GGGGATAACAGGTTATCTGGGGCCAGGGGAGGG<br>(((((....)))))..	IKBKE	NM_014002	chr1:204710209-204736846	non-template	33	Intron_17	--	Yes	--	Yes	--	--	--	Yes	--	--	--
G4ST01204730938	chr1:204730939-204730971	+	GGGGATAACAGGTTATCTGGGGCCAGGGGAGGG<br>(((((....)))))..	IKBKE	NM_001193321	chr1:204710209-204736846	non-template	33	Intron_16	--	Yes	--	Yes	--	--	--	Yes	--	--	--
G4ST01204730938	chr1:204730939-204730971	+	GGGGATAACAGGTTATCTGGGGCCAGGGGAGGG<br>(((((....)))))..	IKBKE	NM_001193322	chr1:204710209-204736846	non-template	33	Intron_17	--	Yes	--	Yes	--	--	--	Yes	--	--	--
G4ST01204748030	chr1:204748031-204748065	+	GGGGAGGGGCGTGCGGGGAGACCGCAGTCTGGGGG<br>.((((....))))..	RASSF5	NM_182663	chr1:204747502-204829239	non-template	35	Intron_1.f	--	Yes	--	Yes	--	--	--	Yes	--	--	--
G4ST01204748030	chr1:204748031-204748065	+	GGGGAGGGGCGTGCGGGGAGACCGCAGTCTGGGGG<br>.((((....))))..	RASSF5	NM_182664	chr1:204747502-204829239	non-template	35	Intron_1.f	--	Yes	--	Yes	--	--	--	Yes	--	--	--
G4ST01204797401	chr1:204797402-204797447	+	GGGGCTCAGAGCTAGGGGCTTACGCCAAGCGGAGCCCGGGGAGGGG<br>(((...)))... (((..(((...))).)))...	RASSF5	NM_182663	chr1:204747502-204829239	non-template	46	Intron_2	--	Yes	--	Yes	--	--	--	Yes	Yes	Yes	--
G4ST01204797401	chr1:204797402-204797447	+	GGGGCTCAGAGCTAGGGGCTTACGCCAAGCGGAGCCCGGGGAGGGG<br>(((...)))... (((..(((...))).)))...	RASSF5	NM_182665	chr1:204797115-204829239	non-template	46	Exon_1.f	--	Yes	Yes	--	--	Yes	--	Yes	Yes	Yes	--

G4ST01204797401	chr1:204797402-204797447	+	GGGGCTCAGAGCTAGGGGCTTACGCCAAGCGGAGCCCGGGGAGGGG<br>(((...)))... (((..(((...))).)))...	RASSF5	NM_182664	chr1:204747502-204829239	non-template	46	Intron_2	--	Yes	--	Yes	--	--	--	Yes	Yes	Yes	--
G4ST01204805312	chr1:204805313-204805341	-	GGGAACCTGGGAAAATTTTGGGATATGGG<br>((....))	RASSF5	NM_182663	chr1:204747502-204829239	template	29	Intron_2	--	Yes	--	Yes	--	--	--	--	--	--	--
G4ST01204805312	chr1:204805313-204805341	-	GGGAACCTGGGAAAATTTTGGGATATGGG<br>((....))	RASSF5	NM_182665	chr1:204797115-204829239	template	29	Intron_1.f	--	Yes	--	Yes	--	--	--	--	--	--	--
G4ST01204805312	chr1:204805313-204805341	-	GGGAACCTGGGAAAATTTTGGGATATGGG<br>((....))	RASSF5	NM_182664	chr1:204747502-204829239	template	29	Intron_2	--	Yes	--	Yes	--	--	--	--	--	--	--
G4ST01204924987	chr1:204924988-204925025	+	GGGCGCCGGGCCGGTGGGAGCCAGCGGCGCGCGGTGGG<br>..((.(((...))))).	MAPKAPK2	NM_004759	chr1:204924912-204974249	non-template	38	Exon_1.f	--	Yes	Yes	--	--	Yes	--	--	Yes	Yes	Yes
G4ST01204924987	chr1:204924988-204925025	+	GGGCGCCGGGCCGGTGGGAGCCAGCGGCGCGCGGTGGG<br>..((.(((...))))).	MAPKAPK2	NM_032960	chr1:204924912-204974249	non-template	38	Exon_1.f	--	Yes	Yes	--	--	Yes	--	--	Yes	Yes	Yes
G4ST01204925646	chr1:204925647-204925686	-	GGGAGCGGCCGGAAGCGGTCAGGGAGGCGAGGGCGCTGGG<br>...(((((....))))).	MAPKAPK2	NM_004759	chr1:204924912-204974249	template	40	Intron_1.f	--	Yes	--	Yes	--	--	--	Yes	Yes	Yes	--
G4ST01204925646	chr1:204925647-204925686	-	GGGAGCGGCCGGAAGCGGTCAGGGAGGCGAGGGCGCTGGG<br>...(((((....))))).	MAPKAPK2	NM_032960	chr1:204924912-204974249	template	40	Intron_1.f	--	Yes	--	Yes	--	--	--	Yes	Yes	Yes	--
G4ST01204942870	chr1:204942871-204942912	-	GGGAGGCAGTTCTGCCTGGAATTGGGCAGGGGAGTAAGGGGG<br>(((((....)))))......	MAPKAPK2	NM_004759	chr1:204924912-204974249	template	42	Intron_1.f	--	Yes	--	Yes	--	--	--	Yes	--	--	--
G4ST01204942870	chr1:204942871-204942912	-	GGGAGGCAGTTCTGCCTGGAATTGGGCAGGGGAGTAAGGGGG<br>(((((....)))))......	MAPKAPK2	NM_032960	chr1:204924912-204974249	template	42	Intron_1.f	--	Yes	--	Yes	--	--	--	Yes	--	--	--
G4ST01204942880	chr1:204942881-204942922	-	GGGCAGAGGAGGGAGGCAGTTCTGCCTGGAATTGGGCAGGGG<br>(((((....)))))......	MAPKAPK2	NM_004759	chr1:204924912-204974249	template	42	Intron_1.f	--	Yes	--	Yes	--	--	--	Yes	--	--	--
G4ST01204942880	chr1:204942881-204942922	-	GGGCAGAGGAGGGAGGCAGTTCTGCCTGGAATTGGGCAGGGG<br>(((((....)))))......	MAPKAPK2	NM_032960	chr1:204924912-204974249	template	42	Intron_1.f	--	Yes	--	Yes	--	--	--	Yes	--	--	--
G4ST01204973330	chr1:204973331-204973370	+	GGGGCAGGCAGGGCTGCAGAGGGAGACGGCCCTGCTGGGG<br>...((((...))))	MAPKAPK2	NM_004759	chr1:204924912-204974249	non-template	40	Exon_10.l	--	Yes	Yes	--	--	--	Yes	--	--	--	Yes
G4ST01204973330	chr1:204973331-204973370	+	GGGGCAGGCAGGGCTGCAGAGGGAGACGGCCCTGCTGGGG<br>...((((...))))	MAPKAPK2	NM_032960	chr1:204924912-204974249	non-template	40	Exon_10.l	--	Yes	Yes	--	--	--	Yes	--	--	--	Yes
G4ST01205050742	chr1:205050743-205050774	+	GGGGCCTACTGGGAAGTGTTTGGGTCAGGGGG<br>((....))	IL19	NM_153758	chr1:205038838-205082950	non-template	32	Intron_1.f	--	Yes	--	Yes	--	--	--	--	--	--	--
G4ST01205072023	chr1:205072024-205072060	-	GGGATGGAGGGAGGTGGGTGCTGTTTTCCCAGCAGGG<br>.((((......)))).	IL19	NM_013371	chr1:205068845-205082950	template	37	Intron_1.f	--	Yes	--	Yes	--	--	--	--	Yes	--	Yes
G4ST01205072023	chr1:205072024-205072060	-	GGGATGGAGGGAGGTGGGTGCTGTTTTCCCAGCAGGG<br>.((((......)))).	IL19	NM_153758	chr1:205038838-205082950	template	37	Intron_1.f	--	Yes	--	Yes	--	--	--	--	Yes	--	Yes
G4ST01205137360	chr1:205137361-205137392	-	GGGCATATATATGGGGGCTGATGGGGGAGGGG<br>(((....))).	IL24	NM_006850	chr1:205137411-205144107	template	-18	upstream	Yes	--	--	--	--	--	--	Yes	Yes	--	--
G4ST01205137360	chr1:205137361-205137392	-	GGGCATATATATGGGGGCTGATGGGGGAGGGG<br>(((....))).	IL24	NM_001185158	chr1:205137411-205144107	template	-18	upstream	Yes	--	--	--	--	--	--	Yes	Yes	--	--
G4ST01205137360	chr1:205137361-205137392	-	GGGCATATATATGGGGGCTGATGGGGGAGGGG<br>(((....))).	IL24	NM_001185157	chr1:205137411-205144107	template	-18	upstream	Yes	--	--	--	--	--	--	Yes	Yes	--	--
G4ST01205137360	chr1:205137361-205137392	-	GGGCATATATATGGGGGCTGATGGGGGAGGGG<br>(((....))).	IL24	NM_001185156	chr1:205137411-205144107	template	-18	upstream	Yes	--	--	--	--	--	--	Yes	Yes	--	--
G4ST01205149599	chr1:205149600-205149641	-	GGGAAAGCCGGGCGCAGCGGGGCGCGCTCCCAGGTGCCAGGG<br>..(((((....)))))..	FAIM3	NM_001193338	chr1:205143253-205161858	non-template	42	Intron_5	--	Yes	--	Yes	--	--	--	Yes	Yes	Yes	--
G4ST01205149599	chr1:205149600-205149641	-	GGGAAAGCCGGGCGCAGCGGGGCGCGCTCCCAGGTGCCAGGG<br>..(((((....)))))..	FAIM3	NM_001142473	chr1:205143253-205162001	non-template	42	Intron_5	--	Yes	--	Yes	--	--	--	Yes	Yes	Yes	--
G4ST01205149599	chr1:205149600-205149641	-	GGGAAAGCCGGGCGCAGCGGGGCGCGCTCCCAGGTGCCAGGG<br>..(((((....)))))..	FAIM3	NM_005449	chr1:205143253-205162001	non-template	42	Intron_6	--	Yes	--	Yes	--	--	--	Yes	Yes	Yes	--
G4ST01205162550	chr1:205162551-205162589	-	GGGCAGCACCTTGTGGGGACACTTTGGGGAGATTAAGGG<br>...(((...)))	FAIM3	NM_001193338	chr1:205143253-205161858	non-template	-692	upstream	Yes	--	--	--	--	--	--	--	--	Yes	--
G4ST01205162550	chr1:205162551-205162589	-	GGGCAGCACCTTGTGGGGACACTTTGGGGAGATTAAGGG<br>...(((...)))	FAIM3	NM_001142473	chr1:205143253-205162001	non-template	-549	upstream	Yes	--	--	--	--	--	--	--	--	Yes	--
G4ST01205162550	chr1:205162551-205162589	-	GGGCAGCACCTTGTGGGGACACTTTGGGGAGATTAAGGG<br>...(((...)))	FAIM3	NM_005449	chr1:205143253-205162001	non-template	-549	upstream	Yes	--	--	--	--	--	--	--	--	Yes	--
G4ST01205579610	chr1:205579611-205579653	-	GGGATTATCTTCCAAATAATCCAGGGAGTGTGGGTGAAGTGGG<br>(((((.......)))))...	CD55	NM_000574	chr1:205561440-205600934	template	43	Intron_9.l	--	Yes	--	Yes	--	--	--	--	--	--	--
G4ST01205579610	chr1:205579611-205579653	-	GGGATTATCTTCCAAATAATCCAGGGAGTGTGGGTGAAGTGGG<br>(((((.......)))))...	CD55	NM_001114752	chr1:205561440-205600934	template	43	Intron_9	--	Yes	--	Yes	--	--	--	--	--	--	--
G4ST01205884980	chr1:205884981-205885009	-	GGGGCCAATCGGCGGGCCGCGGGGCGGGG<br>(((....)))	CR1L	NM_175710	chr1:205885081-205963659	template	-71	upstream	Yes	--	--	--	--	--	--	Yes	Yes	Yes	--
G4ST01205884985	chr1:205884986-205885018	-	GGGGCGGCTGGGGCCAATCGGCGGGCCGCGGGG<br>(((....)))	CR1L	NM_175710	chr1:205885081-205963659	template	-62	upstream	Yes	--	--	--	--	--	--	Yes	Yes	Yes	--
G4ST01205885595	chr1:205885596-205885627	+	GGGGAGGGCTTACTGGGCGCCCCAGGTGAGGG<br>((((...)))).	CR1L	NM_175710	chr1:205885081-205963659	non-template	32	Intron_1.f	--	Yes	--	Yes	--	--	--	--	Yes	Yes	--
G4ST01205991960	chr1:205991961-205991992	-	GGGCGGCTGGGGCCAATCGGCGGGCCGCGGGG<br>(((....)))	CD46	NM_153826	chr1:205992006-206035484	template	-13	upstream	Yes	--	--	--	--	--	--	Yes	Yes	Yes	--
G4ST01205991960	chr1:205991961-205991992	-	GGGCGGCTGGGGCCAATCGGCGGGCCGCGGGG<br>(((....)))	CD46	NM_002389	chr1:205992006-206035484	template	-13	upstream	Yes	--	--	--	--	--	--	Yes	Yes	Yes	--
G4ST01205991960	chr1:205991961-205991992	-	GGGCGGCTGGGGCCAATCGGCGGGCCGCGGGG<br>(((....)))	CD46	NM_172359	chr1:205992006-206035484	template	-13	upstream	Yes	--	--	--	--	--	--	Yes	Yes	Yes	--
G4ST01205991960	chr1:205991961-205991992	-	GGGCGGCTGGGGCCAATCGGCGGGCCGCGGGG<br>(((....)))	CD46	NM_172353	chr1:205992006-206035484	template	-13	upstream	Yes	--	--	--	--	--	--	Yes	Yes	Yes	--
G4ST01205991960	chr1:205991961-205991992	-	GGGCGGCTGGGGCCAATCGGCGGGCCGCGGGG<br>(((....)))	CD46	NM_172352	chr1:205992006-206035484	template	-13	upstream	Yes	--	--	--	--	--	--	Yes	Yes	Yes	--
G4ST01205991960	chr1:205991961-205991992	-	GGGCGGCTGGGGCCAATCGGCGGGCCGCGGGG<br>(((....)))	CD46	NM_172351	chr1:205992006-206035484	template	-13	upstream	Yes	--	--	--	--	--	--	Yes	Yes	Yes	--
G4ST01205991960	chr1:205991961-205991992	-	GGGCGGCTGGGGCCAATCGGCGGGCCGCGGGG<br>(((....)))	CD46	NM_172350	chr1:205992006-206035484	template	-13	upstream	Yes	--	--	--	--	--	--	Yes	Yes	Yes	--
G4ST01205991960	chr1:205991961-205991992	-	GGGCGGCTGGGGCCAATCGGCGGGCCGCGGGG<br>(((....)))	CD46	NM_172361	chr1:205992006-206035484	template	-13	upstream	Yes	--	--	--	--	--	--	Yes	Yes	Yes	--
G4ST01206044268	chr1:206044269-206044297	-	GGGGCTGAGGGGTGGTAGCCAAGGGAGGG<br>.(((...))).	MIR29B2	NR_029518	chr1:206042411-206042491	non-template	-1777	upstream	Yes	--	--	--	--	--	--	--	--	Yes	Yes
G4ST01206044272	chr1:206044273-206044303	-	GGGGGCGGGGCTGAGGGGTGGTAGCCAAGGG<br>.(((...))).	MIR29B2	NR_029518	chr1:206042411-206042491	non-template	-1781	upstream	Yes	--	--	--	--	--	--	--	--	Yes	Yes
G4ST01206058772	chr1:206058773-206058810	+	GGGCTTTCTGGGCCCTGGGAAGATGTCTGTCATCTGGG<br>.(((((.....)))))	LOC148696	NR_026817	chr1:206058347-206062564	non-template	38	Exon_1.u	--	Yes	Yes	--	--	--	--	--	Yes	Yes	--
G4ST01206058781	chr1:206058782-206058819	+	GGGCCCTGGGAAGATGTCTGTCATCTGGGACTAGTGGG<br>.(((((.....)))))	LOC148696	NR_026817	chr1:206058347-206062564	non-template	38	Exon_1.u	--	Yes	Yes	--	--	--	--	--	Yes	Yes	--
G4ST01206269480	chr1:206269481-206269508	-	GGGAAGTAGCTTGGGCAGCCGGGCTGGG<br>(((...)))	PLXNA2	NM_025179	chr1:206262211-206484288	non-template	28	Intron_29	--	Yes	--	Yes	--	--	--	--	Yes	--	Yes
G4ST01206286692	chr1:206286693-206286732	+	GGGAACAGCACCAAAAGGCCTGGGGACAAGGGAGCATGGG<br>..(((..((....)).)))	PLXNA2	NM_025179	chr1:206262211-206484288	template	40	Intron_17	--	Yes	--	Yes	--	--	--	--	--	--	--
G4ST01206304839	chr1:206304840-206304881	-	GGGGACACAGTCTCTGTGTGTGTTGGGCGGGGCTGGTGGGGG<br>.((((((...))))))....	PLXNA2	NM_025179	chr1:206262211-206484288	non-template	42	Intron_12	--	Yes	--	Yes	--	--	--	--	--	--	Yes
G4ST01206308993	chr1:206308994-206309032	-	GGGCGTGGTCACTGACTGCTCCTGGGGCAGGGTTTAGGG<br>.((((((...))))))....	PLXNA2	NM_025179	chr1:206262211-206484288	non-template	39	Intron_12	--	Yes	--	Yes	--	--	--	--	--	--	--
G4ST01206424167	chr1:206424168-206424193	+	GGGGTGGGTTGAGACAGGGAGGAGGG<br>.((...))	PLXNA2	NM_025179	chr1:206262211-206484288	template	26	Intron_3	--	Yes	--	Yes	--	--	--	Yes	Yes	--	Yes
G4ST01206424172	chr1:206424173-206424198	+	GGGTTGAGACAGGGAGGAGGGAAGGG<br>.((...))	PLXNA2	NM_025179	chr1:206262211-206484288	template	26	Intron_3	--	Yes	--	Yes	--	--	--	Yes	Yes	--	Yes

G4ST01206484192	chr1:206484193-206484220	-	GGGAGGGCGGCGGAGGGCAGTTGCTGGG<br>.((...))	PLXNA2	NM_025179	chr1:206262211-206484288	non-template	28	Exon_1.f	--	Yes	Yes	--	--	Yes	--	Yes	Yes	Yes	--
G4ST01207835066	chr1:207835067-207835092	+	GGGTGGGCTGGGCTGCCTGCAGTGGG<br>(((....))).	CAMK1G	NM_020439	chr1:207823668-207853907	non-template	26	Intron_2	--	Yes	--	Yes	--	--	--	--	--	--	--
G4ST01207874705	chr1:207874706-207874744	+	GGGGGCCCAGTGGGAGCAAGTGGGCTCGGAGCATCCGGG<br>...(((...)))	LAMB3	NM_000228	chr1:207854841-207892443	template	39	Intron_5	--	Yes	--	Yes	--	--	--	--	--	--	Yes
G4ST01207874705	chr1:207874706-207874744	+	GGGGGCCCAGTGGGAGCAAGTGGGCTCGGAGCATCCGGG<br>...(((...)))	LAMB3	NM_001127641	chr1:207854841-207892297	template	39	Intron_5	--	Yes	--	Yes	--	--	--	--	--	--	Yes
G4ST01207874705	chr1:207874706-207874744	+	GGGGGCCCAGTGGGAGCAAGTGGGCTCGGAGCATCCGGG<br>...(((...)))	LAMB3	NM_001017402	chr1:207854841-207891302	template	39	Intron_4	--	Yes	--	Yes	--	--	--	--	--	--	Yes
G4ST01207891601	chr1:207891602-207891629	+	GGGCTGGGGCTCGACAAGAGGGGTGGGG<br>.(((.....)))	LAMB3	NM_000228	chr1:207854841-207892443	template	28	Intron_1.f	--	Yes	--	Yes	--	--	--	--	Yes	--	--
G4ST01207891601	chr1:207891602-207891629	+	GGGCTGGGGCTCGACAAGAGGGGTGGGG<br>.(((.....)))	LAMB3	NM_001127641	chr1:207854841-207892297	template	28	Intron_1.f	--	Yes	--	Yes	--	--	--	--	Yes	--	--
G4ST01207891601	chr1:207891602-207891629	+	GGGCTGGGGCTCGACAAGAGGGGTGGGG<br>.(((.....)))	LAMB3	NM_001017402	chr1:207854841-207891302	template	-299	upstream	Yes	--	--	--	--	--	--	--	Yes	--	--
G4ST01207891606	chr1:207891607-207891634	+	GGGGCTCGACAAGAGGGGTGGGGCTGGG<br>.(((.....)))	LAMB3	NM_000228	chr1:207854841-207892443	template	28	Intron_1.f	--	Yes	--	Yes	--	--	--	--	Yes	--	--
G4ST01207891606	chr1:207891607-207891634	+	GGGGCTCGACAAGAGGGGTGGGGCTGGG<br>.(((.....)))	LAMB3	NM_001127641	chr1:207854841-207892297	template	28	Intron_1.f	--	Yes	--	Yes	--	--	--	--	Yes	--	--
G4ST01207891606	chr1:207891607-207891634	+	GGGGCTCGACAAGAGGGGTGGGGCTGGG<br>.(((.....)))	LAMB3	NM_001017402	chr1:207854841-207891302	template	-304	upstream	Yes	--	--	--	--	--	--	--	Yes	--	--
G4ST01207974500	chr1:207974501-207974527	+	GGGCTGGGCATGCTGAGGGATTTTGGG<br>((...)).	HSD11B1	NM_005525	chr1:207944814-207974918	non-template	27	Exon_6.l	--	Yes	Yes	--	--	--	Yes	--	Yes	--	--
G4ST01207974500	chr1:207974501-207974527	+	GGGCTGGGCATGCTGAGGGATTTTGGG<br>((...)).	HSD11B1	NM_181755	chr1:207926173-207974918	non-template	27	Exon_7.l	--	Yes	Yes	--	--	--	Yes	--	Yes	--	--
G4ST01208028392	chr1:208028393-208028428	+	GGGGAGGCAGGGCAGGGGGCAGTTGCATGCTGGGGG<br>..((((.....)))).	IRF6	NM_006147	chr1:208027885-208046102	template	36	Exon_9.l	--	Yes	Yes	--	Yes	--	--	--	--	--	--
G4ST01208028401	chr1:208028402-208028432	+	GGGCAGGGGGCAGTTGCATGCTGGGGGTGGG<br>..((((.....)))).	IRF6	NM_006147	chr1:208027885-208046102	template	31	Exon_9.l	--	Yes	Yes	--	Yes	--	--	--	--	--	--
G4ST01208028406	chr1:208028407-208028441	+	GGGGGCAGTTGCATGCTGGGGGTGGGCTGCATGGG<br>..((((.....)))).	IRF6	NM_006147	chr1:208027885-208046102	template	35	Exon_9.l	--	Yes	Yes	--	Yes	--	--	--	--	--	--
G4ST01208045300	chr1:208045301-208045336	-	GGGGGTGGGCCCTCTTTGCCCGAAGGGGCTGGAGGG<br>....((((....))))	IRF6	NM_006147	chr1:208027885-208046102	non-template	36	Intron_1.f	--	Yes	--	Yes	--	--	--	--	--	--	--
G4ST01208178394	chr1:208178395-208178426	+	GGGAGGCGGGTGGGCGGAGCGGCGCCGCTGGG<br>...((((....))))	SYT14	NM_001146261	chr1:208178161-208404256	non-template	32	Intron_1.f	--	Yes	--	Yes	--	--	--	Yes	Yes	--	--
G4ST01208178394	chr1:208178395-208178426	+	GGGAGGCGGGTGGGCGGAGCGGCGCCGCTGGG<br>...((((....))))	SYT14	NM_001146262	chr1:208178161-208404256	non-template	32	Intron_1.f	--	Yes	--	Yes	--	--	--	Yes	Yes	--	--
G4ST01208178394	chr1:208178395-208178426	+	GGGAGGCGGGTGGGCGGAGCGGCGCCGCTGGG<br>...((((....))))	SYT14	NM_001146264	chr1:208178161-208404256	non-template	32	Intron_1.f	--	Yes	--	Yes	--	--	--	Yes	Yes	--	--
G4ST01208178394	chr1:208178395-208178426	+	GGGAGGCGGGTGGGCGGAGCGGCGCCGCTGGG<br>...((((....))))	SYT14	NM_153262	chr1:208178161-208404256	non-template	32	Intron_1.f	--	Yes	--	Yes	--	--	--	Yes	Yes	--	--
G4ST01208178394	chr1:208178395-208178426	+	GGGAGGCGGGTGGGCGGAGCGGCGCCGCTGGG<br>...((((....))))	SYT14	NR_027458	chr1:208178161-208404256	non-template	32	Intron_1.f	--	Yes	--	Yes	--	--	--	Yes	Yes	--	--
G4ST01208178394	chr1:208178395-208178426	+	GGGAGGCGGGTGGGCGGAGCGGCGCCGCTGGG<br>...((((....))))	SYT14	NR_027459	chr1:208178161-208404256	non-template	32	Intron_1.f	--	Yes	--	Yes	--	--	--	Yes	Yes	--	--
G4ST01208178401	chr1:208178402-208178434	+	GGGTGGGCGGAGCGGCGCCGCTGGGACGCCGGG<br>...((((....))))	SYT14	NM_001146261	chr1:208178161-208404256	non-template	33	Intron_1.f	--	Yes	--	Yes	--	--	--	Yes	Yes	--	--
G4ST01208178401	chr1:208178402-208178434	+	GGGTGGGCGGAGCGGCGCCGCTGGGACGCCGGG<br>...((((....))))	SYT14	NM_001146262	chr1:208178161-208404256	non-template	33	Intron_1.f	--	Yes	--	Yes	--	--	--	Yes	Yes	--	--
G4ST01208178401	chr1:208178402-208178434	+	GGGTGGGCGGAGCGGCGCCGCTGGGACGCCGGG<br>...((((....))))	SYT14	NM_001146264	chr1:208178161-208404256	non-template	33	Intron_1.f	--	Yes	--	Yes	--	--	--	Yes	Yes	--	--
G4ST01208178401	chr1:208178402-208178434	+	GGGTGGGCGGAGCGGCGCCGCTGGGACGCCGGG<br>...((((....))))	SYT14	NM_153262	chr1:208178161-208404256	non-template	33	Intron_1.f	--	Yes	--	Yes	--	--	--	Yes	Yes	--	--
G4ST01208178401	chr1:208178402-208178434	+	GGGTGGGCGGAGCGGCGCCGCTGGGACGCCGGG<br>...((((....))))	SYT14	NR_027458	chr1:208178161-208404256	non-template	33	Intron_1.f	--	Yes	--	Yes	--	--	--	Yes	Yes	--	--
G4ST01208178401	chr1:208178402-208178434	+	GGGTGGGCGGAGCGGCGCCGCTGGGACGCCGGG<br>...((((....))))	SYT14	NR_027459	chr1:208178161-208404256	non-template	33	Intron_1.f	--	Yes	--	Yes	--	--	--	Yes	Yes	--	--
G4ST01208473781	chr1:208473782-208473814	+	GGGCTTCCAGCGGGTTTGGCCGGCAAGGGAGGG<br>((...)). .(((.....)))	C1orf133	NR_024337	chr1:208471427-208474089	template	33	Exon_1.f	--	Yes	Yes	--	--	--	--	Yes	Yes	Yes	Yes
G4ST01208473781	chr1:208473782-208473814	+	GGGCTTCCAGCGGGTTTGGCCGGCAAGGGAGGG<br>((...)). .(((.....)))	SERTAD4	NM_019605	chr1:208472818-208483063	non-template	33	Intron_1.f	--	Yes	--	Yes	--	--	--	Yes	Yes	Yes	Yes
G4ST01208473792	chr1:208473793-208473818	+	GGGTTTGGCCGGCAAGGGAGGGAGGG<br>.(((.....)))	C1orf133	NR_024337	chr1:208471427-208474089	template	26	Exon_1.f	--	Yes	Yes	--	--	--	--	Yes	Yes	Yes	Yes
G4ST01208473792	chr1:208473793-208473818	+	GGGTTTGGCCGGCAAGGGAGGGAGGG<br>.(((.....)))	SERTAD4	NM_019605	chr1:208472818-208483063	non-template	26	Intron_1.f	--	Yes	--	Yes	--	--	--	Yes	Yes	Yes	Yes
G4ST01208616649	chr1:208616650-208616690	+	GGGGTGCGCTGGGTGGGGAGGTGTCTCTTGGACAGCTTGGG<br>.((.((((.....)))).)).	HHAT	NM_001170580	chr1:208569263-208916261	non-template	41	Intron_3	--	Yes	--	Yes	--	--	--	--	--	--	Yes
G4ST01208616649	chr1:208616650-208616690	+	GGGGTGCGCTGGGTGGGGAGGTGTCTCTTGGACAGCTTGGG<br>.((.((((.....)))).)).	HHAT	NM_001170587	chr1:208568873-208916261	non-template	41	Intron_2	--	Yes	--	Yes	--	--	--	--	--	--	Yes
G4ST01208616649	chr1:208616650-208616690	+	GGGGTGCGCTGGGTGGGGAGGTGTCTCTTGGACAGCTTGGG<br>.((.((((.....)))).)).	HHAT	NM_001122834	chr1:208568219-208916261	non-template	41	Intron_3	--	Yes	--	Yes	--	--	--	--	--	--	Yes
G4ST01208616649	chr1:208616650-208616690	+	GGGGTGCGCTGGGTGGGGAGGTGTCTCTTGGACAGCTTGGG<br>.((.((((.....)))).)).	HHAT	NM_001170564	chr1:208568219-208916261	non-template	41	Intron_3	--	Yes	--	Yes	--	--	--	--	--	--	Yes
G4ST01208616649	chr1:208616650-208616690	+	GGGGTGCGCTGGGTGGGGAGGTGTCTCTTGGACAGCTTGGG<br>.((.((((.....)))).)).	HHAT	NM_018194	chr1:208568873-208916261	non-template	41	Intron_3	--	Yes	--	Yes	--	--	--	--	--	--	Yes
G4ST01208616649	chr1:208616650-208616690	+	GGGGTGCGCTGGGTGGGGAGGTGTCTCTTGGACAGCTTGGG<br>.((.((((.....)))).)).	HHAT	NM_001170588	chr1:208568873-208916261	non-template	41	Intron_3	--	Yes	--	Yes	--	--	--	--	--	--	Yes
G4ST01208634894	chr1:208634895-208634928	+	GGGATGCAGTTGTTCAGACTGTGGGATGGGTGGG<br>...(((((.....))))).	HHAT	NM_001170580	chr1:208569263-208916261	non-template	34	Intron_4	--	Yes	--	Yes	--	--	--	--	--	--	--
G4ST01208634894	chr1:208634895-208634928	+	GGGATGCAGTTGTTCAGACTGTGGGATGGGTGGG<br>...(((((.....))))).	HHAT	NM_001170587	chr1:208568873-208916261	non-template	34	Intron_3	--	Yes	--	Yes	--	--	--	--	--	--	--
G4ST01208634894	chr1:208634895-208634928	+	GGGATGCAGTTGTTCAGACTGTGGGATGGGTGGG<br>...(((((.....))))).	HHAT	NM_001122834	chr1:208568219-208916261	non-template	34	Intron_4	--	Yes	--	Yes	--	--	--	--	--	--	--
G4ST01208634894	chr1:208634895-208634928	+	GGGATGCAGTTGTTCAGACTGTGGGATGGGTGGG<br>...(((((.....))))).	HHAT	NM_001170564	chr1:208568219-208916261	non-template	34	Intron_4	--	Yes	--	Yes	--	--	--	--	--	--	--
G4ST01208634894	chr1:208634895-208634928	+	GGGATGCAGTTGTTCAGACTGTGGGATGGGTGGG<br>...(((((.....))))).	HHAT	NM_018194	chr1:208568873-208916261	non-template	34	Intron_4	--	Yes	--	Yes	--	--	--	--	--	--	--
G4ST01208634894	chr1:208634895-208634928	+	GGGATGCAGTTGTTCAGACTGTGGGATGGGTGGG<br>...(((((.....))))).	HHAT	NM_001170588	chr1:208568873-208916261	non-template	34	Intron_4	--	Yes	--	Yes	--	--	--	--	--	--	--
G4ST01208764388	chr1:208764389-208764425	+	GGGAAAGGGCGAGGGCTGGACTGTTCAAGCCAGAGGG<br>((((.((.....)))))).	HHAT	NM_001170580	chr1:208569263-208916261	non-template	37	Intron_9	--	Yes	--	Yes	--	--	--	--	--	--	--
G4ST01208764388	chr1:208764389-208764425	+	GGGAAAGGGCGAGGGCTGGACTGTTCAAGCCAGAGGG<br>((((.((.....)))))).	HHAT	NM_001170587	chr1:208568873-208916261	non-template	37	Intron_8	--	Yes	--	Yes	--	--	--	--	--	--	--
G4ST01208764388	chr1:208764389-208764425	+	GGGAAAGGGCGAGGGCTGGACTGTTCAAGCCAGAGGG<br>((((.((.....)))))).	HHAT	NM_001122834	chr1:208568219-208916261	non-template	37	Intron_9	--	Yes	--	Yes	--	--	--	--	--	--	--
G4ST01208764388	chr1:208764389-208764425	+	GGGAAAGGGCGAGGGCTGGACTGTTCAAGCCAGAGGG<br>((((.((.....)))))).	HHAT	NM_001170564	chr1:208568219-208916261	non-template	37	Intron_7	--	Yes	--	Yes	--	--	--	--	--	--	--
G4ST01208764388	chr1:208764389-208764425	+	GGGAAAGGGCGAGGGCTGGACTGTTCAAGCCAGAGGG<br>((((.((.....)))))).	HHAT	NM_018194	chr1:208568873-208916261	non-template	37	Intron_9	--	Yes	--	Yes	--	--	--	--	--	--	--

G4ST01208764388	chr1:208764389-208764425	+	GGGAAAGGGCGAGGGCTGGACTGTTCAAGCCAGAGGG<br>((((.((.....)))))).	HHAT	NM_001170588	chr1:208568873-208916261	non-template	37	Intron_8	--	Yes	--	Yes	--	--	--	--	--	--	--
G4ST01208783551	chr1:208783552-208783575	-	GGGGTGGGGAGGGTTGAGGCAGGG<br>.((...))	HHAT	NM_001170580	chr1:208569263-208916261	template	24	Intron_9	--	Yes	--	Yes	--	--	--	--	--	--	--
G4ST01208783551	chr1:208783552-208783575	-	GGGGTGGGGAGGGTTGAGGCAGGG<br>.((...))	HHAT	NM_001170587	chr1:208568873-208916261	template	24	Intron_8	--	Yes	--	Yes	--	--	--	--	--	--	--
G4ST01208783551	chr1:208783552-208783575	-	GGGGTGGGGAGGGTTGAGGCAGGG<br>.((...))	HHAT	NM_001122834	chr1:208568219-208916261	template	24	Intron_9	--	Yes	--	Yes	--	--	--	--	--	--	--
G4ST01208783551	chr1:208783552-208783575	-	GGGGTGGGGAGGGTTGAGGCAGGG<br>.((...))	HHAT	NM_001170564	chr1:208568219-208916261	template	24	Intron_7	--	Yes	--	Yes	--	--	--	--	--	--	--
G4ST01208783551	chr1:208783552-208783575	-	GGGGTGGGGAGGGTTGAGGCAGGG<br>.((...))	HHAT	NM_018194	chr1:208568873-208916261	template	24	Intron_9	--	Yes	--	Yes	--	--	--	--	--	--	--
G4ST01208783551	chr1:208783552-208783575	-	GGGGTGGGGAGGGTTGAGGCAGGG<br>.((...))	HHAT	NM_001170588	chr1:208568873-208916261	template	24	Intron_8	--	Yes	--	Yes	--	--	--	--	--	--	--
G4ST01208796414	chr1:208796415-208796453	-	GGGGTTCAGGTGAGCAGCAGGGAGCAGTGGGAACCAGGG<br>((((....))))....	HHAT	NM_001170580	chr1:208569263-208916261	template	39	Intron_9	--	Yes	--	Yes	--	--	--	--	--	--	--
G4ST01208796414	chr1:208796415-208796453	-	GGGGTTCAGGTGAGCAGCAGGGAGCAGTGGGAACCAGGG<br>((((....))))....	HHAT	NM_001170587	chr1:208568873-208916261	template	39	Intron_8	--	Yes	--	Yes	--	--	--	--	--	--	--
G4ST01208796414	chr1:208796415-208796453	-	GGGGTTCAGGTGAGCAGCAGGGAGCAGTGGGAACCAGGG<br>((((....))))....	HHAT	NM_001122834	chr1:208568219-208916261	template	39	Intron_9	--	Yes	--	Yes	--	--	--	--	--	--	--
G4ST01208796414	chr1:208796415-208796453	-	GGGGTTCAGGTGAGCAGCAGGGAGCAGTGGGAACCAGGG<br>((((....))))....	HHAT	NM_001170564	chr1:208568219-208916261	template	39	Intron_7	--	Yes	--	Yes	--	--	--	--	--	--	--
G4ST01208796414	chr1:208796415-208796453	-	GGGGTTCAGGTGAGCAGCAGGGAGCAGTGGGAACCAGGG<br>((((....))))....	HHAT	NM_018194	chr1:208568873-208916261	template	39	Intron_9	--	Yes	--	Yes	--	--	--	--	--	--	--
G4ST01208796414	chr1:208796415-208796453	-	GGGGTTCAGGTGAGCAGCAGGGAGCAGTGGGAACCAGGG<br>((((....))))....	HHAT	NM_001170588	chr1:208568873-208916261	template	39	Intron_8	--	Yes	--	Yes	--	--	--	--	--	--	--
G4ST01208796422	chr1:208796423-208796465	-	GGGCAGCCCCCTGGGGTTCAGGTGAGCAGCAGGGAGCAGTGGG<br>(((....))) ((((....))))....	HHAT	NM_001170580	chr1:208569263-208916261	template	43	Intron_9	--	Yes	--	Yes	--	--	--	--	--	--	--
G4ST01208796422	chr1:208796423-208796465	-	GGGCAGCCCCCTGGGGTTCAGGTGAGCAGCAGGGAGCAGTGGG<br>(((....))) ((((....))))....	HHAT	NM_001170587	chr1:208568873-208916261	template	43	Intron_8	--	Yes	--	Yes	--	--	--	--	--	--	--
G4ST01208796422	chr1:208796423-208796465	-	GGGCAGCCCCCTGGGGTTCAGGTGAGCAGCAGGGAGCAGTGGG<br>(((....))) ((((....))))....	HHAT	NM_001122834	chr1:208568219-208916261	template	43	Intron_9	--	Yes	--	Yes	--	--	--	--	--	--	--
G4ST01208796422	chr1:208796423-208796465	-	GGGCAGCCCCCTGGGGTTCAGGTGAGCAGCAGGGAGCAGTGGG<br>(((....))) ((((....))))....	HHAT	NM_001170564	chr1:208568219-208916261	template	43	Intron_7	--	Yes	--	Yes	--	--	--	--	--	--	--
G4ST01208796422	chr1:208796423-208796465	-	GGGCAGCCCCCTGGGGTTCAGGTGAGCAGCAGGGAGCAGTGGG<br>(((....))) ((((....))))....	HHAT	NM_018194	chr1:208568873-208916261	template	43	Intron_9	--	Yes	--	Yes	--	--	--	--	--	--	--
G4ST01208796422	chr1:208796423-208796465	-	GGGCAGCCCCCTGGGGTTCAGGTGAGCAGCAGGGAGCAGTGGG<br>(((....))) ((((....))))....	HHAT	NM_001170588	chr1:208568873-208916261	template	43	Intron_8	--	Yes	--	Yes	--	--	--	--	--	--	--
G4ST01208796431	chr1:208796432-208796469	-	GGGAGGGCAGCCCCCTGGGGTTCAGGTGAGCAGCAGGG<br>(((....))) ((((....))))....	HHAT	NM_001170580	chr1:208569263-208916261	template	38	Intron_9	--	Yes	--	Yes	--	--	--	--	--	--	--
G4ST01208796431	chr1:208796432-208796469	-	GGGAGGGCAGCCCCCTGGGGTTCAGGTGAGCAGCAGGG<br>(((....))) ((((....))))....	HHAT	NM_001170587	chr1:208568873-208916261	template	38	Intron_8	--	Yes	--	Yes	--	--	--	--	--	--	--
G4ST01208796431	chr1:208796432-208796469	-	GGGAGGGCAGCCCCCTGGGGTTCAGGTGAGCAGCAGGG<br>(((....))) ((((....))))....	HHAT	NM_001122834	chr1:208568219-208916261	template	38	Intron_9	--	Yes	--	Yes	--	--	--	--	--	--	--
G4ST01208796431	chr1:208796432-208796469	-	GGGAGGGCAGCCCCCTGGGGTTCAGGTGAGCAGCAGGG<br>(((....))) ((((....))))....	HHAT	NM_001170564	chr1:208568219-208916261	template	38	Intron_7	--	Yes	--	Yes	--	--	--	--	--	--	--
G4ST01208796431	chr1:208796432-208796469	-	GGGAGGGCAGCCCCCTGGGGTTCAGGTGAGCAGCAGGG<br>(((....))) ((((....))))....	HHAT	NM_018194	chr1:208568873-208916261	template	38	Intron_9	--	Yes	--	Yes	--	--	--	--	--	--	--
G4ST01208796431	chr1:208796432-208796469	-	GGGAGGGCAGCCCCCTGGGGTTCAGGTGAGCAGCAGGG<br>(((....))) ((((....))))....	HHAT	NM_001170588	chr1:208568873-208916261	template	38	Intron_8	--	Yes	--	Yes	--	--	--	--	--	--	--
G4ST01208798769	chr1:208798770-208798800	+	GGGTTGGGGTCATGTGGCTGGGATGATGGGG<br>(((....))).	HHAT	NM_001170580	chr1:208569263-208916261	non-template	31	Intron_9	--	Yes	--	Yes	--	--	--	--	--	--	--
G4ST01208798769	chr1:208798770-208798800	+	GGGTTGGGGTCATGTGGCTGGGATGATGGGG<br>(((....))).	HHAT	NM_001170587	chr1:208568873-208916261	non-template	31	Intron_8	--	Yes	--	Yes	--	--	--	--	--	--	--
G4ST01208798769	chr1:208798770-208798800	+	GGGTTGGGGTCATGTGGCTGGGATGATGGGG<br>(((....))).	HHAT	NM_001122834	chr1:208568219-208916261	non-template	31	Intron_9	--	Yes	--	Yes	--	--	--	--	--	--	--
G4ST01208798769	chr1:208798770-208798800	+	GGGTTGGGGTCATGTGGCTGGGATGATGGGG<br>(((....))).	HHAT	NM_001170564	chr1:208568219-208916261	non-template	31	Intron_7	--	Yes	--	Yes	--	--	--	--	--	--	--
G4ST01208798769	chr1:208798770-208798800	+	GGGTTGGGGTCATGTGGCTGGGATGATGGGG<br>(((....))).	HHAT	NM_018194	chr1:208568873-208916261	non-template	31	Intron_9	--	Yes	--	Yes	--	--	--	--	--	--	--
G4ST01208798769	chr1:208798770-208798800	+	GGGTTGGGGTCATGTGGCTGGGATGATGGGG<br>(((....))).	HHAT	NM_001170588	chr1:208568873-208916261	non-template	31	Intron_8	--	Yes	--	Yes	--	--	--	--	--	--	--
G4ST01208798774	chr1:208798775-208798810	+	GGGGTCATGTGGCTGGGATGATGGGGCATCCTTGGG<br>(((....))).	HHAT	NM_001170580	chr1:208569263-208916261	non-template	36	Intron_9	--	Yes	--	Yes	--	--	--	--	--	--	--
G4ST01208798774	chr1:208798775-208798810	+	GGGGTCATGTGGCTGGGATGATGGGGCATCCTTGGG<br>(((....))).	HHAT	NM_001170587	chr1:208568873-208916261	non-template	36	Intron_8	--	Yes	--	Yes	--	--	--	--	--	--	--
G4ST01208798774	chr1:208798775-208798810	+	GGGGTCATGTGGCTGGGATGATGGGGCATCCTTGGG<br>(((....))).	HHAT	NM_001122834	chr1:208568219-208916261	non-template	36	Intron_9	--	Yes	--	Yes	--	--	--	--	--	--	--
G4ST01208798774	chr1:208798775-208798810	+	GGGGTCATGTGGCTGGGATGATGGGGCATCCTTGGG<br>(((....))).	HHAT	NM_001170564	chr1:208568219-208916261	non-template	36	Intron_7	--	Yes	--	Yes	--	--	--	--	--	--	--
G4ST01208798774	chr1:208798775-208798810	+	GGGGTCATGTGGCTGGGATGATGGGGCATCCTTGGG<br>(((....))).	HHAT	NM_018194	chr1:208568873-208916261	non-template	36	Intron_9	--	Yes	--	Yes	--	--	--	--	--	--	--
G4ST01208798774	chr1:208798775-208798810	+	GGGGTCATGTGGCTGGGATGATGGGGCATCCTTGGG<br>(((....))).	HHAT	NM_001170588	chr1:208568873-208916261	non-template	36	Intron_8	--	Yes	--	Yes	--	--	--	--	--	--	--
G4ST01208901795	chr1:208901796-208901828	+	GGGCCTGTTGGCAGGTGGGTGGCAGGGGGAGGG<br>((((....)))).	HHAT	NM_001170580	chr1:208569263-208916261	non-template	33	Intron_11.l	--	Yes	--	Yes	--	--	--	--	--	--	--
G4ST01208901795	chr1:208901796-208901828	+	GGGCCTGTTGGCAGGTGGGTGGCAGGGGGAGGG<br>((((....)))).	HHAT	NM_001170587	chr1:208568873-208916261	non-template	33	Intron_10.l	--	Yes	--	Yes	--	--	--	--	--	--	--
G4ST01208901795	chr1:208901796-208901828	+	GGGCCTGTTGGCAGGTGGGTGGCAGGGGGAGGG<br>((((....)))).	HHAT	NM_001122834	chr1:208568219-208916261	non-template	33	Intron_11.l	--	Yes	--	Yes	--	--	--	--	--	--	--
G4ST01208901795	chr1:208901796-208901828	+	GGGCCTGTTGGCAGGTGGGTGGCAGGGGGAGGG<br>((((....)))).	HHAT	NM_001170564	chr1:208568219-208916261	non-template	33	Intron_9.l	--	Yes	--	Yes	--	--	--	--	--	--	--
G4ST01208901795	chr1:208901796-208901828	+	GGGCCTGTTGGCAGGTGGGTGGCAGGGGGAGGG<br>((((....)))).	HHAT	NM_018194	chr1:208568873-208916261	non-template	33	Intron_11.l	--	Yes	--	Yes	--	--	--	--	--	--	--
G4ST01208901795	chr1:208901796-208901828	+	GGGCCTGTTGGCAGGTGGGTGGCAGGGGGAGGG<br>((((....)))).	HHAT	NM_001170588	chr1:208568873-208916261	non-template	33	Intron_10.l	--	Yes	--	Yes	--	--	--	--	--	--	--
G4ST01208934557	chr1:208934558-208934596	-	GGGACTAGGGCCTGCCCAGCTCCAGGGGGATTCCCTGGG<br>((((........))))	KCNH1	NM_002238	chr1:208918280-209374080	non-template	39	Intron_10.l	--	Yes	--	Yes	--	--	--	--	--	--	--
G4ST01208934557	chr1:208934558-208934596	-	GGGACTAGGGCCTGCCCAGCTCCAGGGGGATTCCCTGGG<br>((((........))))	KCNH1	NM_172362	chr1:208918280-209374080	non-template	39	Intron_10.l	--	Yes	--	Yes	--	--	--	--	--	--	--
G4ST01208934567	chr1:208934568-208934604	-	GGGGGCCTGGGACTAGGGCCTGCCCAGCTCCAGGGGG<br>((((........))))	KCNH1	NM_002238	chr1:208918280-209374080	non-template	37	Intron_10.l	--	Yes	--	Yes	--	--	--	--	--	--	--
G4ST01208934567	chr1:208934568-208934604	-	GGGGGCCTGGGACTAGGGCCTGCCCAGCTCCAGGGGG<br>((((........))))	KCNH1	NM_172362	chr1:208918280-209374080	non-template	37	Intron_10.l	--	Yes	--	Yes	--	--	--	--	--	--	--
G4ST01208985654	chr1:208985655-208985687	-	GGGCCAGCCCAGCACTGCTTGGGGGCGGGTGGG<br>....((.(((...))).))	KCNH1	NM_002238	chr1:208918280-209374080	non-template	33	Intron_10.l	--	Yes	--	Yes	--	--	--	--	--	--	Yes
G4ST01208985654	chr1:208985655-208985687	-	GGGCCAGCCCAGCACTGCTTGGGGGCGGGTGGG<br>....((.(((...))).))	KCNH1	NM_172362	chr1:208918280-209374080	non-template	33	Intron_10.l	--	Yes	--	Yes	--	--	--	--	--	--	Yes
G4ST01209144296	chr1:209144297-209144333	-	GGGGAAGCATGGGGGAAGGCTCTTGGGGTCAGTGGGG<br>..(((...))).	KCNH1	NM_002238	chr1:208918280-209374080	non-template	37	Intron_7	--	Yes	--	Yes	--	--	--	--	--	--	--

G4ST01209144296	chr1:209144297-209144333	-	GGGGAAGCATGGGGGAAGGCTCTTGGGGTCAGTGGGG<br>..(((...))).	KCNH1	NM_172362	chr1:208918280-209374080	non-template	37	Intron_7	--	Yes	--	Yes	--	--	--	--	--	--	--
G4ST01209144305	chr1:209144306-209144339	-	GGGGGTGGGGAAGCATGGGGGAAGGCTCTTGGGG<br>..(((...))).	KCNH1	NM_002238	chr1:208918280-209374080	non-template	34	Intron_7	--	Yes	--	Yes	--	--	--	--	--	--	--
G4ST01209144305	chr1:209144306-209144339	-	GGGGGTGGGGAAGCATGGGGGAAGGCTCTTGGGG<br>..(((...))).	KCNH1	NM_172362	chr1:208918280-209374080	non-template	34	Intron_7	--	Yes	--	Yes	--	--	--	--	--	--	--
G4ST01209324551	chr1:209324552-209324598	+	GGGCTCTTAAAAGAGTGGGTTCTCAGCTGGCTGAGCAAGGGCATGGG<br>((((....)))). ..(((((....)))))...	KCNH1	NM_002238	chr1:208918280-209374080	template	47	Intron_4	--	Yes	--	Yes	--	--	--	--	--	--	--
G4ST01209324551	chr1:209324552-209324598	+	GGGCTCTTAAAAGAGTGGGTTCTCAGCTGGCTGAGCAAGGGCATGGG<br>((((....)))). ..(((((....)))))...	KCNH1	NM_172362	chr1:208918280-209374080	template	47	Intron_4	--	Yes	--	Yes	--	--	--	--	--	--	--
G4ST01209500543	chr1:209500544-209500584	+	GGGCGGGTGGCAGAGGCCAGGGCGGCTGTCACCGGCCGGGG<br>.(((....))). ((((((....))))))	RCOR3	NM_001136225	chr1:209499331-209553278	non-template	41	Intron_2	--	Yes	--	Yes	--	--	--	Yes	Yes	Yes	--
G4ST01209500543	chr1:209500544-209500584	+	GGGCGGGTGGCAGAGGCCAGGGCGGCTGTCACCGGCCGGGG<br>.(((....))). ((((((....))))))	RCOR3	NM_001136223	chr1:209499331-209556348	non-template	41	Intron_2	--	Yes	--	Yes	--	--	--	Yes	Yes	Yes	--
G4ST01209500543	chr1:209500544-209500584	+	GGGCGGGTGGCAGAGGCCAGGGCGGCTGTCACCGGCCGGGG<br>.(((....))). ((((((....))))))	RCOR3	NM_001136224	chr1:209499331-209556348	non-template	41	Intron_2	--	Yes	--	Yes	--	--	--	Yes	Yes	Yes	--
G4ST01209500543	chr1:209500544-209500584	+	GGGCGGGTGGCAGAGGCCAGGGCGGCTGTCACCGGCCGGGG<br>.(((....))). ((((((....))))))	RCOR3	NM_018254	chr1:209499912-209556348	non-template	41	Intron_1.f	--	Yes	--	Yes	--	--	--	Yes	Yes	Yes	--
G4ST01209500547	chr1:209500548-209500594	+	GGGTGGCAGAGGCCAGGGCGGCTGTCACCGGCCGGGGAAGCCAAGGG<br>.(((....))). ((((((....))))))	RCOR3	NM_001136225	chr1:209499331-209553278	non-template	47	Intron_2	--	Yes	--	Yes	--	--	--	Yes	Yes	Yes	--
G4ST01209500547	chr1:209500548-209500594	+	GGGTGGCAGAGGCCAGGGCGGCTGTCACCGGCCGGGGAAGCCAAGGG<br>.(((....))). ((((((....))))))	RCOR3	NM_001136223	chr1:209499331-209556348	non-template	47	Intron_2	--	Yes	--	Yes	--	--	--	Yes	Yes	Yes	--
G4ST01209500547	chr1:209500548-209500594	+	GGGTGGCAGAGGCCAGGGCGGCTGTCACCGGCCGGGGAAGCCAAGGG<br>.(((....))). ((((((....))))))	RCOR3	NM_001136224	chr1:209499331-209556348	non-template	47	Intron_2	--	Yes	--	Yes	--	--	--	Yes	Yes	Yes	--
G4ST01209500547	chr1:209500548-209500594	+	GGGTGGCAGAGGCCAGGGCGGCTGTCACCGGCCGGGGAAGCCAAGGG<br>.(((....))). ((((((....))))))	RCOR3	NM_018254	chr1:209499912-209556348	non-template	47	Intron_1.f	--	Yes	--	Yes	--	--	--	Yes	Yes	Yes	--
G4ST01209567800	chr1:209567801-209567841	-	GGGTCAGAGCCCAAGGCTATGGAGGGTGGGGGCTGCAGGGG<br>.((.((((...)))).))..	TRAF5	NM_004619	chr1:209566580-209614909	template	41	Intron_1.f	--	Yes	--	Yes	--	--	--	Yes	Yes	Yes	--
G4ST01209567800	chr1:209567801-209567841	-	GGGTCAGAGCCCAAGGCTATGGAGGGTGGGGGCTGCAGGGG<br>.((.((((...)))).))..	TRAF5	NM_001033910	chr1:209566771-209614909	template	41	Intron_1.f	--	Yes	--	Yes	--	--	--	Yes	Yes	Yes	--
G4ST01209720343	chr1:209720344-209720368	+	GGGATGGGTGAGGGAAGATTTTGGG<br>((....))	RD3	NM_001164688	chr1:209716487-209732882	template	25	Intron_2.l	--	Yes	--	Yes	--	--	--	--	--	--	--
G4ST01209720343	chr1:209720344-209720368	+	GGGATGGGTGAGGGAAGATTTTGGG<br>((....))	RD3	NM_183059	chr1:209716487-209732882	template	25	Intron_2.l	--	Yes	--	Yes	--	--	--	--	--	--	--
G4ST01209721423	chr1:209721424-209721469	+	GGGCCCCTCTGTTAGTCAGAGTGGGGAACCTGGGAACCCAAGGGGG<br>...(((((.....)))))..	RD3	NM_001164688	chr1:209716487-209732882	template	46	Intron_1.f	--	Yes	--	Yes	--	--	--	--	--	--	--
G4ST01209721423	chr1:209721424-209721469	+	GGGCCCCTCTGTTAGTCAGAGTGGGGAACCTGGGAACCCAAGGGGG<br>...(((((.....)))))..	RD3	NM_183059	chr1:209716487-209732882	template	46	Intron_1.f	--	Yes	--	Yes	--	--	--	--	--	--	--
G4ST01209723627	chr1:209723628-209723666	+	GGGTAGTACTGTGTGTACTGGGGGGTGGGAGTGCAGGGG<br>.(((((.....)))))...	RD3	NM_001164688	chr1:209716487-209732882	template	39	Intron_1.f	--	Yes	--	Yes	--	--	--	--	--	--	Yes
G4ST01209723627	chr1:209723628-209723666	+	GGGTAGTACTGTGTGTACTGGGGGGTGGGAGTGCAGGGG<br>.(((((.....)))))...	RD3	NM_183059	chr1:209716487-209732882	template	39	Intron_1.f	--	Yes	--	Yes	--	--	--	--	--	--	Yes
G4ST01209819388	chr1:209819389-209819416	+	GGGCGTGGGCCGGGGGGCCGTGGCCGGG<br>.(((....)))	SLC30A1	NM_021194	chr1:209815004-209818722	template	-666	upstream	Yes	--	--	--	--	--	--	--	Yes	Yes	--
G4ST01210070433	chr1:210070434-210070473	-	GGGGGCGGGGAAGGGGGTGTCGTCGTCGCGTGGCCCGGGG<br>(((..(((....)))..)))..	LPGAT1	NM_014873	chr1:209983422-210070737	non-template	40	Intron_1.f	--	Yes	--	Yes	--	--	--	Yes	Yes	Yes	Yes
G4ST01210070635	chr1:210070636-210070671	-	GGGCGCGGGCCGCGCCACAGCCGGAGCGGGGCAGGG<br>.(((.((......)).)))	LPGAT1	NM_014873	chr1:209983422-210070737	non-template	36	Exon_1.f	--	Yes	Yes	--	--	Yes	--	Yes	Yes	Yes	--
G4ST01210070640	chr1:210070641-210070678	-	GGGGAACGGGCGCGGGCCGCGCCACAGCCGGAGCGGGG<br>.(((.((......)).)))	LPGAT1	NM_014873	chr1:209983422-210070737	non-template	38	Exon_1.f	--	Yes	Yes	--	--	Yes	--	Yes	Yes	Yes	--
G4ST01210255086	chr1:210255087-210255118	+	GGGAGGGAGGTGGGGGGCAGCCCCTGCCCGGG<br>((((((...))))))	INTS7	NM_001199809	chr1:210180364-210275625	template	32	Intron_3	--	Yes	--	Yes	--	--	--	--	--	--	--
G4ST01210255086	chr1:210255087-210255118	+	GGGAGGGAGGTGGGGGGCAGCCCCTGCCCGGG<br>((((((...))))))	INTS7	NM_001199811	chr1:210180364-210275625	template	32	Intron_4	--	Yes	--	Yes	--	--	--	--	--	--	--
G4ST01210255086	chr1:210255087-210255118	+	GGGAGGGAGGTGGGGGGCAGCCCCTGCCCGGG<br>((((((...))))))	INTS7	NM_001199812	chr1:210180364-210275625	template	32	Intron_4	--	Yes	--	Yes	--	--	--	--	--	--	--
G4ST01210255086	chr1:210255087-210255118	+	GGGAGGGAGGTGGGGGGCAGCCCCTGCCCGGG<br>((((((...))))))	INTS7	NM_015434	chr1:210180364-210275625	template	32	Intron_4	--	Yes	--	Yes	--	--	--	--	--	--	--
G4ST01210255086	chr1:210255087-210255118	+	GGGAGGGAGGTGGGGGGCAGCCCCTGCCCGGG<br>((((((...))))))	INTS7	NR_037667	chr1:210180364-210275625	template	32	Intron_5	--	Yes	--	Yes	--	--	--	--	--	--	--
G4ST01210255090	chr1:210255091-210255127	+	GGGAGGTGGGGGGCAGCCCCTGCCCGGGAAGTGTGGG<br>((((((...))))))	INTS7	NM_001199809	chr1:210180364-210275625	template	37	Intron_3	--	Yes	--	Yes	--	--	--	--	--	--	--
G4ST01210255090	chr1:210255091-210255127	+	GGGAGGTGGGGGGCAGCCCCTGCCCGGGAAGTGTGGG<br>((((((...))))))	INTS7	NM_001199811	chr1:210180364-210275625	template	37	Intron_4	--	Yes	--	Yes	--	--	--	--	--	--	--
G4ST01210255090	chr1:210255091-210255127	+	GGGAGGTGGGGGGCAGCCCCTGCCCGGGAAGTGTGGG<br>((((((...))))))	INTS7	NM_001199812	chr1:210180364-210275625	template	37	Intron_4	--	Yes	--	Yes	--	--	--	--	--	--	--
G4ST01210255090	chr1:210255091-210255127	+	GGGAGGTGGGGGGCAGCCCCTGCCCGGGAAGTGTGGG<br>((((((...))))))	INTS7	NM_015434	chr1:210180364-210275625	template	37	Intron_4	--	Yes	--	Yes	--	--	--	--	--	--	--
G4ST01210255090	chr1:210255091-210255127	+	GGGAGGTGGGGGGCAGCCCCTGCCCGGGAAGTGTGGG<br>((((((...))))))	INTS7	NR_037667	chr1:210180364-210275625	template	37	Intron_5	--	Yes	--	Yes	--	--	--	--	--	--	--
G4ST01210262860	chr1:210262861-210262892	-	GGGGACTTTTAAAAGTAGGGAGGAAGGGAGGG<br>.((((....)))).	INTS7	NM_001199809	chr1:210180364-210275625	non-template	32	Intron_1.f	--	Yes	--	Yes	--	--	--	--	--	--	Yes
G4ST01210262860	chr1:210262861-210262892	-	GGGGACTTTTAAAAGTAGGGAGGAAGGGAGGG<br>.((((....)))).	INTS7	NM_001199811	chr1:210180364-210275625	non-template	32	Intron_1.f	--	Yes	--	Yes	--	--	--	--	--	--	Yes
G4ST01210262860	chr1:210262861-210262892	-	GGGGACTTTTAAAAGTAGGGAGGAAGGGAGGG<br>.((((....)))).	INTS7	NM_001199812	chr1:210180364-210275625	non-template	32	Intron_1.f	--	Yes	--	Yes	--	--	--	--	--	--	Yes
G4ST01210262860	chr1:210262861-210262892	-	GGGGACTTTTAAAAGTAGGGAGGAAGGGAGGG<br>.((((....)))).	INTS7	NM_015434	chr1:210180364-210275625	non-template	32	Intron_1.f	--	Yes	--	Yes	--	--	--	--	--	--	Yes
G4ST01210262860	chr1:210262861-210262892	-	GGGGACTTTTAAAAGTAGGGAGGAAGGGAGGG<br>.((((....)))).	INTS7	NR_037667	chr1:210180364-210275625	non-template	32	Intron_1.f	--	Yes	--	Yes	--	--	--	--	--	--	Yes
G4ST01210654327	chr1:210654328-210654365	+	GGGTTCCTCTGGGCCATTGGGCTGCCGCTCCGGCGGGG<br>..((((...)))).	TMEM206	NM_001198862	chr1:210604439-210654866	template	38	Intron_1.f	--	Yes	--	Yes	--	--	--	Yes	Yes	Yes	--
G4ST01210654327	chr1:210654328-210654365	+	GGGTTCCTCTGGGCCATTGGGCTGCCGCTCCGGCGGGG<br>..((((...)))).	TMEM206	NM_018252	chr1:210604439-210654890	template	38	Intron_1.f	--	Yes	--	Yes	--	--	--	Yes	Yes	Yes	--
G4ST01210654581	chr1:210654582-210654615	-	GGGGCGGGGGGTGTCGGGGCTGCGCGGCAGAGGG<br>.((((...)))).	TMEM206	NM_001198862	chr1:210604439-210654866	non-template	34	Intron_1.f	--	Yes	--	Yes	--	--	--	Yes	Yes	Yes	Yes
G4ST01210654581	chr1:210654582-210654615	-	GGGGCGGGGGGTGTCGGGGCTGCGCGGCAGAGGG<br>.((((...)))).	TMEM206	NM_018252	chr1:210604439-210654890	non-template	34	Intron_1.f	--	Yes	--	Yes	--	--	--	Yes	Yes	Yes	Yes
G4ST01210673459	chr1:210673460-210673497	+	GGGGGACGGAGGGAGGGAAGCAGAGCCTGCTCCCTGGG<br>.(((((...)))))....	NENF	NR_026598	chr1:210672852-210686344	non-template	38	Intron_1.f	--	Yes	--	Yes	--	--	--	Yes	Yes	Yes	Yes
G4ST01210673459	chr1:210673460-210673497	+	GGGGGACGGAGGGAGGGAAGCAGAGCCTGCTCCCTGGG<br>.(((((...)))))....	NENF	NM_013349	chr1:210672852-210686344	non-template	38	Intron_1.f	--	Yes	--	Yes	--	--	--	Yes	Yes	Yes	Yes
G4ST01210673469	chr1:210673470-210673506	+	GGGAGGGAAGCAGAGCCTGCTCCCTGGGTTAGGTGGG<br>.(((((...)))))....	NENF	NR_026598	chr1:210672852-210686344	non-template	37	Intron_1.f	--	Yes	--	Yes	--	--	--	Yes	Yes	Yes	Yes
G4ST01210673469	chr1:210673470-210673506	+	GGGAGGGAAGCAGAGCCTGCTCCCTGGGTTAGGTGGG<br>.(((((...)))))....	NENF	NM_013349	chr1:210672852-210686344	non-template	37	Intron_1.f	--	Yes	--	Yes	--	--	--	Yes	Yes	Yes	Yes
G4ST01210846870	chr1:210846871-210846901	+	GGGGGTGAGGGCGTGGAAGCGGAGGGTGGGG<br>(((....)))..	ATF3	NM_001674	chr1:210848617-210860739	non-template	-1715	upstream	Yes	--	--	--	--	--	--	Yes	Yes	Yes	--
G4ST01210846870	chr1:210846871-210846901	+	GGGGGTGAGGGCGTGGAAGCGGAGGGTGGGG<br>(((....)))..	ATF3	NM_001040619	chr1:210848617-210860739	non-template	-1715	upstream	Yes	--	--	--	--	--	--	Yes	Yes	Yes	--

G4ST01210846870	chr1:210846871-210846901	+	GGGGGTGAGGGCGTGGAAGCGGAGGGTGGGG<br>(((....)))..	ATF3	NM_001030287	chr1:210805320-210860739	non-template	31	Intron_1.f	--	Yes	--	Yes	--	--	--	Yes	Yes	Yes	--
G4ST01210848549	chr1:210848550-210848588	-	GGGGGGTGGAGGAGGGGCCAAGGCGGGGCCGAAGGCGGG<br>(((...)))	ATF3	NM_001674	chr1:210848617-210860739	template	-28	upstream	Yes	--	--	--	--	--	--	Yes	Yes	Yes	--
G4ST01210848549	chr1:210848550-210848588	-	GGGGGGTGGAGGAGGGGCCAAGGCGGGGCCGAAGGCGGG<br>(((...)))	ATF3	NM_001040619	chr1:210848617-210860739	template	-28	upstream	Yes	--	--	--	--	--	--	Yes	Yes	Yes	--
G4ST01210848549	chr1:210848550-210848588	-	GGGGGGTGGAGGAGGGGCCAAGGCGGGGCCGAAGGCGGG<br>(((...)))	ATF3	NM_001030287	chr1:210805320-210860739	template	39	Intron_1.f	--	Yes	--	Yes	--	--	--	Yes	Yes	Yes	--
G4ST01210929576	chr1:210929577-210929609	+	GGGAGGATGGGATGGAAGCCAGGGCGTCAAGGG<br>.(((...)))	BATF3	NM_018664	chr1:210926382-210939950	template	33	Intron_2.l	--	Yes	--	Yes	--	--	--	Yes	Yes	--	--
G4ST01210939577	chr1:210939578-210939614	-	GGGCCGTGGGGTGCGGGGACCGGCGCGCCGCGGGGGG<br>..((.(((...))).))	BATF3	NM_018664	chr1:210926382-210939950	non-template	37	Intron_1.f	--	Yes	--	Yes	--	--	--	Yes	Yes	Yes	Yes
G4ST01210939603	chr1:210939604-210939632	-	GGGCGAGAGGCTCAGGGAGGGCCGTGGGG<br>.(((...))).	BATF3	NM_018664	chr1:210926382-210939950	non-template	29	Intron_1.f	--	Yes	--	Yes	--	--	--	Yes	Yes	Yes	Yes
G4ST01210940133	chr1:210940134-210940170	+	GGGTGGGGACCAGGGGGGCGCCATGCAGCCTTTGGGG<br>.(((((...)).))).....	BATF3	NM_018664	chr1:210926382-210939950	template	-183	upstream	Yes	--	--	--	--	--	--	Yes	Yes	Yes	Yes
G4ST01211014060	chr1:211014061-211014097	+	GGGGGTGGGGCAGGGGTTTAAAAAAGAATTATTTGGG<br>....(((.((...)).)))	NSL1	NM_001042549	chr1:210966118-211031762	template	37	Intron_4	--	Yes	--	Yes	--	--	--	--	--	--	--
G4ST01211014060	chr1:211014061-211014097	+	GGGGGTGGGGCAGGGGTTTAAAAAAGAATTATTTGGG<br>....(((.((...)).)))	NSL1	NM_015471	chr1:210966118-211031762	template	37	Intron_4	--	Yes	--	Yes	--	--	--	--	--	--	--
G4ST01211097980	chr1:211097981-211098006	-	GGGTGGGCGGGAGGCGGCCCCGCGGG<br>..(((....)))	NCRNA00292	NR_027285	chr1:211096569-211098103	non-template	26	Exon_1.f	--	Yes	Yes	--	--	--	--	--	Yes	Yes	--
G4ST01211097980	chr1:211097981-211098006	-	GGGTGGGCGGGAGGCGGCCCCGCGGG<br>..(((....)))	NCRNA00292	NR_027286	chr1:211096569-211098103	non-template	26	Exon_1.f	--	Yes	Yes	--	--	--	--	--	Yes	Yes	--
G4ST01211097980	chr1:211097981-211098006	-	GGGTGGGCGGGAGGCGGCCCCGCGGG<br>..(((....)))	FLVCR1	NM_014053	chr1:211098220-211139330	template	-213	upstream	Yes	--	--	--	--	--	--	--	Yes	Yes	--
G4ST01211190832	chr1:211190833-211190865	+	GGGGTCCGGGAGGGCTTCCTGGAAGAAAGCGGG<br>((((...))))....	VASH2	NM_001136475	chr1:211190510-211231550	non-template	33	Intron_1.f	--	Yes	--	Yes	--	--	--	Yes	Yes	Yes	--
G4ST01211190832	chr1:211190833-211190865	+	GGGGTCCGGGAGGGCTTCCTGGAAGAAAGCGGG<br>((((...))))....	VASH2	NM_001136474	chr1:211190510-211231550	non-template	33	Intron_1.f	--	Yes	--	Yes	--	--	--	Yes	Yes	Yes	--
G4ST01211190832	chr1:211190833-211190865	+	GGGGTCCGGGAGGGCTTCCTGGAAGAAAGCGGG<br>((((...))))....	VASH2	NM_024749	chr1:211190510-211231550	non-template	33	Intron_1.f	--	Yes	--	Yes	--	--	--	Yes	Yes	Yes	--
G4ST01211191057	chr1:211191058-211191095	-	GGGGGGTTGGGGATGGAGGGGCACAAGGAGCTTGGGGG<br>...((((...)))).	VASH2	NM_001136475	chr1:211190510-211231550	template	38	Intron_1.f	--	Yes	--	Yes	--	--	--	Yes	Yes	Yes	--
G4ST01211191057	chr1:211191058-211191095	-	GGGGGGTTGGGGATGGAGGGGCACAAGGAGCTTGGGGG<br>...((((...)))).	VASH2	NM_001136474	chr1:211190510-211231550	template	38	Intron_1.f	--	Yes	--	Yes	--	--	--	Yes	Yes	Yes	--
G4ST01211191057	chr1:211191058-211191095	-	GGGGGGTTGGGGATGGAGGGGCACAAGGAGCTTGGGGG<br>...((((...)))).	VASH2	NM_024749	chr1:211190510-211231550	template	38	Intron_1.f	--	Yes	--	Yes	--	--	--	Yes	Yes	Yes	--
G4ST01211418021	chr1:211418022-211418079	+	GGGCATGTAGTCCCAGCTGCTCGGGAGGCTGAGGGGAAAGGATCCCCTGAGCCTGGGG<br>...(((((....))))).. ...(((.((....)).))).	RPS6KC1	NM_001136138	chr1:211291211-211513431	non-template	58	Intron_7	--	Yes	--	Yes	--	--	--	--	--	--	--
G4ST01211418021	chr1:211418022-211418079	+	GGGCATGTAGTCCCAGCTGCTCGGGAGGCTGAGGGGAAAGGATCCCCTGAGCCTGGGG<br>...(((((....))))).. ...(((.((....)).))).	RPS6KC1	NM_012424	chr1:211291211-211513431	non-template	58	Intron_8	--	Yes	--	Yes	--	--	--	--	--	--	--
G4ST01212200798	chr1:212200799-212200834	-	GGGGAGTTTGTGAATTCATGTTGGGGCAGGGTGGGG<br>(((((....))))).......	LOC100505832	NR_037850	chr1:212057718-212206231	non-template	36	Intron_1.f	--	Yes	--	Yes	--	--	--	--	--	--	--
G4ST01212200803	chr1:212200804-212200840	-	GGGAATGGGGAGTTTGTGAATTCATGTTGGGGCAGGG<br>(((((....))))).......	LOC100505832	NR_037850	chr1:212057718-212206231	non-template	37	Intron_1.f	--	Yes	--	Yes	--	--	--	--	--	--	--
G4ST01212568377	chr1:212568378-212568413	-	GGGAAGGGAACAGGGAATCGCTGAGCAGGTGATGGG<br>.((((((.....))))))	SMYD2	NM_020197	chr1:212521188-212577100	template	36	Intron_7	--	Yes	--	Yes	--	--	--	--	Yes	--	--
G4ST01213306070	chr1:213306071-213306103	+	GGGCCTGTTGGGAGGTGCAGGGCTAGGAGAGGG<br>((...)).	MIR548F3	NR_031644	chr1:213146072-213334022	template	33	Intron_1.f	--	Yes	--	Yes	--	--	--	--	--	--	--
G4ST01213306070	chr1:213306071-213306103	+	GGGCCTGTTGGGAGGTGCAGGGCTAGGAGAGGG<br>((...)).	KCNK2	NM_001017424	chr1:213245508-213477059	non-template	33	Intron_1.f	--	Yes	--	Yes	--	--	--	--	--	--	--
G4ST01213807514	chr1:213807515-213807551	-	GGGGCGGCGGCGGCCGGAGGGCGGGGGGTGCACGGGG<br>.((((....))))..	KCTD3	NM_016121	chr1:213807358-213861772	template	37	Exon_1.f	--	Yes	Yes	--	--	Yes	--	--	Yes	Yes	--
G4ST01213807523	chr1:213807524-213807559	-	GGGCCAGCGGGGCGGCGGCGGCCGGAGGGCGGGGGG<br>.((((....))))..	KCTD3	NM_016121	chr1:213807358-213861772	template	36	Exon_1.f	--	Yes	Yes	--	--	Yes	--	--	Yes	Yes	--
G4ST01213807822	chr1:213807823-213807863	+	GGGCGAGCGTGGGAGGCCAAGCCTGGAGGGGAACGTGGGGG<br>((((...))))....	KCTD3	NM_016121	chr1:213807358-213861772	non-template	41	Intron_1.f	--	Yes	--	Yes	--	--	--	Yes	Yes	Yes	--
G4ST01213871655	chr1:213871656-213871691	-	GGGACTGGCTGGCTGCCAGGGGCTCTGGGCCCAGGG<br>.(((((.....)))))	USH2A	NM_206933	chr1:213862859-214663361	non-template	36	Intron_70	--	Yes	--	Yes	--	--	--	Yes	Yes	--	--
G4ST01213887637	chr1:213887638-213887668	+	GGGAGTACAGGGGAGGGCTGAGTCAGGAGGG<br>(((...)))..	USH2A	NM_206933	chr1:213862859-214663361	template	31	Exon_67	--	Yes	Yes	--	Yes	--	--	Yes	--	--	--
G4ST01214253280	chr1:214253281-214253314	-	GGGCTGCTTCGAGCGGGATTAGGGGCGGCGTGGG<br>..(((...)))	USH2A	NM_206933	chr1:213862859-214663361	non-template	34	Intron_32	--	Yes	--	Yes	--	--	--	--	--	--	--
G4ST01214653220	chr1:214653221-214653256	+	GGGTTGAGATTCTCTTAGAATGGGAGTGGGGCTGGG<br>.....(((((...)))))	USH2A	NM_007123	chr1:214413915-214663361	template	36	Intron_3	--	Yes	--	Yes	--	--	--	--	--	--	--
G4ST01214653220	chr1:214653221-214653256	+	GGGTTGAGATTCTCTTAGAATGGGAGTGGGGCTGGG<br>.....(((((...)))))	USH2A	NM_206933	chr1:213862859-214663361	template	36	Intron_3	--	Yes	--	Yes	--	--	--	--	--	--	--
G4ST01214841099	chr1:214841100-214841132	-	GGGAGCCCGGGGCAGCCTGCGGGTGGCAGTGGG<br>(((...)))	ESRRG	NR_024099	chr1:214743219-214963418	non-template	33	Intron_5	--	Yes	--	Yes	--	--	--	Yes	Yes	Yes	--
G4ST01214841099	chr1:214841100-214841132	-	GGGAGCCCGGGGCAGCCTGCGGGTGGCAGTGGG<br>(((...)))	ESRRG	NM_206594	chr1:214743211-215329599	non-template	33	Intron_5	--	Yes	--	Yes	--	--	--	Yes	Yes	Yes	--
G4ST01214841099	chr1:214841100-214841132	-	GGGAGCCCGGGGCAGCCTGCGGGTGGCAGTGGG<br>(((...)))	ESRRG	NM_206595	chr1:214743211-215329599	non-template	33	Intron_4	--	Yes	--	Yes	--	--	--	Yes	Yes	Yes	--
G4ST01214841099	chr1:214841100-214841132	-	GGGAGCCCGGGGCAGCCTGCGGGTGGCAGTGGG<br>(((...)))	ESRRG	NM_001438	chr1:214743211-214963430	non-template	33	Intron_3	--	Yes	--	Yes	--	--	--	Yes	Yes	Yes	--
G4ST01214841099	chr1:214841100-214841132	-	GGGAGCCCGGGGCAGCCTGCGGGTGGCAGTGGG<br>(((...)))	ESRRG	NM_001134285	chr1:214743219-215377720	non-template	33	Intron_5	--	Yes	--	Yes	--	--	--	Yes	Yes	Yes	--
G4ST01214841204	chr1:214841205-214841240	-	GGGAGGCCCAGGGGTGGGCGCGGTCCGACGGCCGGG<br>.((.((...)).)).	ESRRG	NR_024099	chr1:214743219-214963418	non-template	36	Intron_5	--	Yes	--	Yes	--	--	--	Yes	Yes	Yes	Yes
G4ST01214841204	chr1:214841205-214841240	-	GGGAGGCCCAGGGGTGGGCGCGGTCCGACGGCCGGG<br>.((.((...)).)).	ESRRG	NM_206594	chr1:214743211-215329599	non-template	36	Intron_5	--	Yes	--	Yes	--	--	--	Yes	Yes	Yes	Yes
G4ST01214841204	chr1:214841205-214841240	-	GGGAGGCCCAGGGGTGGGCGCGGTCCGACGGCCGGG<br>.((.((...)).)).	ESRRG	NM_206595	chr1:214743211-215329599	non-template	36	Intron_4	--	Yes	--	Yes	--	--	--	Yes	Yes	Yes	Yes
G4ST01214841204	chr1:214841205-214841240	-	GGGAGGCCCAGGGGTGGGCGCGGTCCGACGGCCGGG<br>.((.((...)).)).	ESRRG	NM_001438	chr1:214743211-214963430	non-template	36	Intron_3	--	Yes	--	Yes	--	--	--	Yes	Yes	Yes	Yes
G4ST01214841204	chr1:214841205-214841240	-	GGGAGGCCCAGGGGTGGGCGCGGTCCGACGGCCGGG<br>.((.((...)).)).	ESRRG	NM_001134285	chr1:214743219-215377720	non-template	36	Intron_5	--	Yes	--	Yes	--	--	--	Yes	Yes	Yes	Yes
G4ST01215193515	chr1:215193516-215193551	-	GGGCCAAGAGGGAGGATGAAGGTATCGGGGATAGGG<br>..((((....)))).	ESRRG	NM_206594	chr1:214743211-215329599	non-template	36	Intron_1.f	--	Yes	--	Yes	--	--	--	--	--	--	--
G4ST01215193515	chr1:215193516-215193551	-	GGGCCAAGAGGGAGGATGAAGGTATCGGGGATAGGG<br>..((((....)))).	ESRRG	NM_206595	chr1:214743211-215329599	non-template	36	Intron_1.f	--	Yes	--	Yes	--	--	--	--	--	--	--
G4ST01215193515	chr1:215193516-215193551	-	GGGCCAAGAGGGAGGATGAAGGTATCGGGGATAGGG<br>..((((....)))).	ESRRG	NM_001134285	chr1:214743219-215377720	non-template	36	Intron_2	--	Yes	--	Yes	--	--	--	--	--	--	--
G4ST01215330353	chr1:215330354-215330385	-	GGGAAAATGGTTTTTCAGGGTTGGGAGGTGGG<br>((((....))))..	ESRRG	NM_206594	chr1:214743211-215329599	non-template	-754	upstream	Yes	--	--	--	--	--	--	--	--	--	Yes

G4ST01215330353	chr1:215330354-215330385	-	GGGAAAATGGTTTTTCAGGGTTGGGAGGTGGG<br>((((....))))..	ESRRG	NM_206595	chr1:214743211-215329599	non-template	-754	upstream	Yes	--	--	--	--	--	--	--	--	--	Yes
G4ST01215330353	chr1:215330354-215330385	-	GGGAAAATGGTTTTTCAGGGTTGGGAGGTGGG<br>((((....))))..	ESRRG	NM_001134285	chr1:214743219-215377720	non-template	32	Intron_1.f	--	Yes	--	Yes	--	--	--	--	--	--	Yes
G4ST01215330360	chr1:215330361-215330393	-	GGGTAGAAGGGAAAATGGTTTTTCAGGGTTGGG<br>((((....))))..	ESRRG	NM_206594	chr1:214743211-215329599	non-template	-761	upstream	Yes	--	--	--	--	--	--	--	--	--	Yes
G4ST01215330360	chr1:215330361-215330393	-	GGGTAGAAGGGAAAATGGTTTTTCAGGGTTGGG<br>((((....))))..	ESRRG	NM_206595	chr1:214743211-215329599	non-template	-761	upstream	Yes	--	--	--	--	--	--	--	--	--	Yes
G4ST01215330360	chr1:215330361-215330393	-	GGGTAGAAGGGAAAATGGTTTTTCAGGGTTGGG<br>((((....))))..	ESRRG	NM_001134285	chr1:214743219-215377720	non-template	33	Intron_1.f	--	Yes	--	Yes	--	--	--	--	--	--	Yes
G4ST01215330365	chr1:215330366-215330402	-	GGGCCCGGTGGGTAGAAGGGAAAATGGTTTTTCAGGG<br>((((....))))..	ESRRG	NM_206594	chr1:214743211-215329599	non-template	-766	upstream	Yes	--	--	--	--	--	--	--	--	--	Yes
G4ST01215330365	chr1:215330366-215330402	-	GGGCCCGGTGGGTAGAAGGGAAAATGGTTTTTCAGGG<br>((((....))))..	ESRRG	NM_206595	chr1:214743211-215329599	non-template	-766	upstream	Yes	--	--	--	--	--	--	--	--	--	Yes
G4ST01215330365	chr1:215330366-215330402	-	GGGCCCGGTGGGTAGAAGGGAAAATGGTTTTTCAGGG<br>((((....))))..	ESRRG	NM_001134285	chr1:214743219-215377720	non-template	37	Intron_1.f	--	Yes	--	Yes	--	--	--	--	--	--	Yes
G4ST01216012915	chr1:216012916-216012942	+	GGGATGGGATGTTGCATTTGGGATGGG<br>(((...)))..	SPATA17	NM_138796	chr1:215871318-216107107	non-template	27	Intron_6	--	Yes	--	Yes	--	--	--	--	--	--	--
G4ST01216585960	chr1:216585961-216586007	-	GGGGGCGAGCCCAGCGCACCGCGGGGAGGAGGGTGCGCTTCGCAGGG<br>.(((.((.....))..))). .(((...))).	TGFB2	NM_003238	chr1:216585299-216684584	template	47	Exon_1.f	--	Yes	Yes	--	--	Yes	--	Yes	Yes	Yes	--
G4ST01216585960	chr1:216585961-216586007	-	GGGGGCGAGCCCAGCGCACCGCGGGGAGGAGGGTGCGCTTCGCAGGG<br>.(((.((.....))..))). .(((...))).	TGFB2	NM_001135599	chr1:216585299-216684584	template	47	Exon_1.f	--	Yes	Yes	--	--	Yes	--	Yes	Yes	Yes	--
G4ST01216605456	chr1:216605457-216605499	+	GGGAAAATCAGGAGTGTCTGAGGGTGTGGAAGGGGAGATGGGG<br>....(((((....)))))	TGFB2	NM_003238	chr1:216585299-216684584	non-template	43	Intron_1.f	--	Yes	--	Yes	--	--	--	--	--	--	--
G4ST01216605456	chr1:216605457-216605499	+	GGGAAAATCAGGAGTGTCTGAGGGTGTGGAAGGGGAGATGGGG<br>....(((((....)))))	TGFB2	NM_001135599	chr1:216585299-216684584	non-template	43	Intron_2	--	Yes	--	Yes	--	--	--	--	--	--	--
G4ST01218213081	chr1:218213082-218213120	+	GGGGGTTCAGAGGGAGGGAACAATTGCTTCCAATTGGGG<br>..((((((....))))))	EPRS	NM_004446	chr1:218208565-218286623	template	39	Intron_29	--	Yes	--	Yes	--	--	--	--	--	--	--
G4ST01218768269	chr1:218768270-218768302	+	GGGCCCCGGGCGCGTGGATGCGGCTGGGTCGGG<br>((((....))))....	MARK1	NM_018650	chr1:218768191-218904422	non-template	33	Exon_1.f	--	Yes	Yes	--	--	Yes	--	Yes	Yes	Yes	--
G4ST01218930702	chr1:218930703-218930738	+	GGGGGGCGCGGGTGTGGTGTCCCGCGGGGGAGCGGG<br>.((((....))))..	C1orf115	NM_024709	chr1:218930251-218939122	non-template	36	Intron_1.u	--	Yes	--	Yes	--	--	--	--	Yes	--	--
G4ST01218930711	chr1:218930712-218930745	+	GGGTGTGGTGTCCCGCGGGGGAGCGGGAGCCGGG<br>.((((....))))..	C1orf115	NM_024709	chr1:218930251-218939122	non-template	34	Intron_1.u	--	Yes	--	Yes	--	--	--	--	Yes	--	--
G4ST01218938678	chr1:218938679-218938709	-	GGGGGATGGGAGTGGGGAAGCTCTTCCTGGG<br>(((....)))..	C1orf115	NM_024709	chr1:218930251-218939122	template	31	Exon_2.l	--	Yes	Yes	--	--	--	Yes	Yes	Yes	--	--
G4ST01219982843	chr1:219982844-219982871	-	GGGGCAGCGACATCTGCGGGCGGGCGGG<br>((((......))))	DUSP10	NM_007207	chr1:219941387-219982139	non-template	-704	upstream	Yes	--	--	--	--	--	--	Yes	Yes	Yes	Yes
G4ST01219982843	chr1:219982844-219982871	-	GGGGCAGCGACATCTGCGGGCGGGCGGG<br>((((......))))	DUSP10	NM_144729	chr1:219941387-219982139	non-template	-704	upstream	Yes	--	--	--	--	--	--	Yes	Yes	Yes	Yes
G4ST01220857821	chr1:220857822-220857857	-	GGGCAGGGACCAGGCGGAGCGCCGGGGGCATGTGGG<br>.((.((((...))))))	MIA3	NM_198551	chr1:220858067-220907974	template	-209	upstream	Yes	--	--	--	--	--	--	Yes	Yes	Yes	Yes
G4ST01220857829	chr1:220857830-220857864	-	GGGACGCGGGCAGGGACCAGGCGGAGCGCCGGGGG<br>.((.((((...))))))	MIA3	NM_198551	chr1:220858067-220907974	template	-202	upstream	Yes	--	--	--	--	--	--	Yes	Yes	Yes	Yes
G4ST01220858337	chr1:220858338-220858371	+	GGGGGTCGCAGGCGGGATGGGCTGAACGCGGGGG<br>.(((...))) (((....)))	MIA3	NM_198551	chr1:220858067-220907974	non-template	34	Intron_1.f	--	Yes	--	Yes	--	--	--	Yes	Yes	Yes	--
G4ST01220858350	chr1:220858351-220858375	+	GGGATGGGCTGAACGCGGGGGTGGG<br>(((....)))	MIA3	NM_198551	chr1:220858067-220907974	non-template	25	Intron_1.f	--	Yes	--	Yes	--	--	--	Yes	Yes	Yes	--
G4ST01221054869	chr1:221054870-221054900	-	GGGAGAGGGATCGCCGGCGGCGGGCGACGGG<br>...(((...)))	DISP1	NM_032890	chr1:221055054-221245960	template	-153	upstream	Yes	--	--	--	--	--	--	--	Yes	Yes	--
G4ST01221179399	chr1:221179400-221179433	+	GGGTCTAGAGGGGATTTCAGGGTTCTTTGAAGGG<br>(((...)))	DISP1	NM_032890	chr1:221055054-221245960	non-template	34	Intron_3	--	Yes	--	Yes	--	--	--	--	--	--	--
G4ST01221182859	chr1:221182860-221182900	-	GGGCTGCATGGTCTCCATCACAGGGGGTGAGGGGACTCGGG<br>(((.((((...))))..))).	DISP1	NM_032890	chr1:221055054-221245960	template	41	Exon_4	--	Yes	Yes	--	Yes	--	--	--	--	--	--
G4ST01221235395	chr1:221235396-221235426	+	GGGAAATTTAGGGAGCAGGCTGGGAAAGGGG<br>((....))	DISP1	NM_032890	chr1:221055054-221245960	non-template	31	Intron_9.l	--	Yes	--	Yes	--	--	--	Yes	Yes	--	--
G4ST01221366306	chr1:221366307-221366340	+	GGGAGGGGAGGATGGGGAGTTAGCGTTTAATGGG<br>..((((.....))))	TLR5	NM_003268	chr1:221349371-221383247	template	34	Intron_5.l	--	Yes	--	Yes	--	--	--	--	--	--	Yes
G4ST01221368096	chr1:221368097-221368127	-	GGGAAGGGAGGGAGCTCCCAGCTGTGTGGGG<br>((((...)))).... .	TLR5	NM_003268	chr1:221349371-221383247	non-template	31	Intron_5.l	--	Yes	--	Yes	--	--	--	--	--	--	Yes
G4ST01221553879	chr1:221553880-221553911	-	GGGAAGGGGTGGGCAATTCCTGGAACTGAGGG<br>((.(((...))).)).	SUSD4	NM_017982	chr1:221460784-221604167	non-template	32	Intron_2	--	Yes	--	Yes	--	--	--	--	--	--	--
G4ST01221553879	chr1:221553880-221553911	-	GGGAAGGGGTGGGCAATTCCTGGAACTGAGGG<br>((.(((...))).)).	SUSD4	NM_001037175	chr1:221474875-221604167	non-template	32	Intron_2	--	Yes	--	Yes	--	--	--	--	--	--	--
G4ST01221567295	chr1:221567296-221567323	+	GGGAGGAGACTGGGAGTAGGGAATAGGG<br>((....))	SUSD4	NM_017982	chr1:221460784-221604167	template	28	Intron_2	--	Yes	--	Yes	--	--	--	--	--	--	--
G4ST01221567295	chr1:221567296-221567323	+	GGGAGGAGACTGGGAGTAGGGAATAGGG<br>((....))	SUSD4	NM_001037175	chr1:221474875-221604167	template	28	Intron_2	--	Yes	--	Yes	--	--	--	--	--	--	--
G4ST01221569396	chr1:221569397-221569431	-	GGGAAAGTTAGAACTTTGGGAAAAGGGACATTGGG<br>(((((....)))))	SUSD4	NM_017982	chr1:221460784-221604167	non-template	35	Intron_2	--	Yes	--	Yes	--	--	--	--	--	--	--
G4ST01221569396	chr1:221569397-221569431	-	GGGAAAGTTAGAACTTTGGGAAAAGGGACATTGGG<br>(((((....)))))	SUSD4	NM_001037175	chr1:221474875-221604167	non-template	35	Intron_2	--	Yes	--	Yes	--	--	--	--	--	--	--
G4ST01221573681	chr1:221573682-221573711	+	GGGGAGGGGTGCAGGGCTGAAAGCAGAGGG<br>(((....))).	SUSD4	NM_017982	chr1:221460784-221604167	template	30	Intron_2	--	Yes	--	Yes	--	--	--	--	--	--	--
G4ST01221573681	chr1:221573682-221573711	+	GGGGAGGGGTGCAGGGCTGAAAGCAGAGGG<br>(((....))).	SUSD4	NM_001037175	chr1:221474875-221604167	template	30	Intron_2	--	Yes	--	Yes	--	--	--	--	--	--	--
G4ST01221603520	chr1:221603521-221603564	-	GGGGGCGGGGGCCGCGCGGGGGAGGAACCAGGATGGAGCCTGGG<br>..(((..(((...)))..)))	SUSD4	NM_017982	chr1:221460784-221604167	non-template	44	Intron_1.f	--	Yes	--	Yes	--	--	--	Yes	--	--	Yes
G4ST01221603520	chr1:221603521-221603564	-	GGGGGCGGGGGCCGCGCGGGGGAGGAACCAGGATGGAGCCTGGG<br>..(((..(((...)))..)))	SUSD4	NM_001037175	chr1:221474875-221604167	non-template	44	Intron_1.f	--	Yes	--	Yes	--	--	--	Yes	--	--	Yes
G4ST01221604228	chr1:221604229-221604265	-	GGGCGCGCGGGCGCCTCCGCGGGGGCGCGCGGCCGGG<br>(((....))). (((.....)))	SUSD4	NM_017982	chr1:221460784-221604167	non-template	-61	upstream	Yes	--	--	--	--	--	--	Yes	--	--	--
G4ST01221604228	chr1:221604229-221604265	-	GGGCGCGCGGGCGCCTCCGCGGGGGCGCGCGGCCGGG<br>(((....))). (((.....)))	SUSD4	NM_001037175	chr1:221474875-221604167	non-template	-61	upstream	Yes	--	--	--	--	--	--	Yes	--	--	--
G4ST01221804855	chr1:221804856-221804885	-	GGGCAGCGCCAGCTGGGGCTGGGCTTGGGG<br>((((....))))	CAPN8	NM_001143962	chr1:221781595-221920059	non-template	30	Intron_10	--	Yes	--	Yes	--	--	--	--	--	--	Yes
G4ST01221804862	chr1:221804863-221804889	-	GGGTGGGCAGCGCCAGCTGGGGCTGGG<br>((((....))))	CAPN8	NM_001143962	chr1:221781595-221920059	non-template	27	Intron_10	--	Yes	--	Yes	--	--	--	--	--	--	Yes
G4ST01221805542	chr1:221805543-221805570	+	GGGGCCTGCCTCGCTGGGGGTGGGGGGG<br>.((.((...)).))	CAPN8	NM_001143962	chr1:221781595-221920059	template	28	Intron_10	--	Yes	--	Yes	--	--	--	--	--	--	--
G4ST01221808758	chr1:221808759-221808794	+	GGGGGTAGGGCATCTGCTGGATGCCGGGGATGAGGG<br>((((.....))))....	CAPN8	NM_001143962	chr1:221781595-221920059	template	36	Intron_10	--	Yes	--	Yes	--	--	--	--	--	--	--
G4ST01221808850	chr1:221808851-221808901	-	GGGGCCCCGGGACGGCGTGGCGGTGGCCGCGGGGGCTGCGGTGGCGGCGGG<br>....((((((....)))))). .(((((....)))))	CAPN8	NM_001143962	chr1:221781595-221920059	non-template	51	Intron_10	--	Yes	--	Yes	--	--	--	--	--	--	--
G4ST01221809968	chr1:221809969-221810003	+	GGGAGGGGCCGCGCTGGAGCAGCGGGGAAGGAGGG<br>....(((((...)))))	CAPN8	NM_001143962	chr1:221781595-221920059	template	35	Intron_10	--	Yes	--	Yes	--	--	--	--	--	--	Yes
G4ST01221809972	chr1:221809973-221810007	+	GGGGCCGCGCTGGAGCAGCGGGGAAGGAGGGAGGG<br>....(((((...)))))	CAPN8	NM_001143962	chr1:221781595-221920059	template	35	Intron_10	--	Yes	--	Yes	--	--	--	--	--	--	Yes
G4ST01221873121	chr1:221873122-221873151	-	GGGCTTGGAGGCCAGGGTTGTGAGGGTGGG<br>..(((...)))	CAPN8	NM_001143962	chr1:221781595-221920059	non-template	30	Intron_9	--	Yes	--	Yes	--	--	--	--	--	--	--

G4ST01221967846	chr1:221967847-221967882	-	GGGAGGTCAGAGACCCTGGGGGACGGGATAAGGGGG<br>.((((...))))....	CAPN2	NM_001748	chr1:221966742-222030343	template	36	Intron_1.f	--	Yes	--	Yes	--	--	--	Yes	Yes	Yes	Yes
G4ST01221967846	chr1:221967847-221967882	-	GGGAGGTCAGAGACCCTGGGGGACGGGATAAGGGGG<br>.((((...))))....	CAPN2	NM_001146068	chr1:221955918-222030343	template	36	Intron_1.f	--	Yes	--	Yes	--	--	--	Yes	Yes	Yes	Yes
G4ST01221967855	chr1:221967856-221967887	-	GGGCCGGGAGGTCAGAGACCCTGGGGGACGGG<br>.((((...))))....	CAPN2	NM_001748	chr1:221966742-222030343	template	32	Intron_1.f	--	Yes	--	Yes	--	--	--	Yes	Yes	Yes	Yes
G4ST01221967855	chr1:221967856-221967887	-	GGGCCGGGAGGTCAGAGACCCTGGGGGACGGG<br>.((((...))))....	CAPN2	NM_001146068	chr1:221955918-222030343	template	32	Intron_1.f	--	Yes	--	Yes	--	--	--	Yes	Yes	Yes	Yes
G4ST01222007338	chr1:222007339-222007368	-	GGGGTGTGGTACACAGGGTGGGGTAAGGGG<br>..(((...))).	CAPN2	NM_001748	chr1:221966742-222030343	template	30	Intron_9	--	Yes	--	Yes	--	--	--	Yes	--	--	--
G4ST01222007338	chr1:222007339-222007368	-	GGGGTGTGGTACACAGGGTGGGGTAAGGGG<br>..(((...))).	CAPN2	NM_001146068	chr1:221955918-222030343	template	30	Intron_9	--	Yes	--	Yes	--	--	--	Yes	--	--	--
G4ST01222007345	chr1:222007346-222007382	-	GGGGCTGCTAGGCTGGGGTGTGGTACACAGGGTGGGG<br>(((....))).. ..(((...))).	CAPN2	NM_001748	chr1:221966742-222030343	template	37	Intron_9	--	Yes	--	Yes	--	--	--	Yes	--	--	--
G4ST01222007345	chr1:222007346-222007382	-	GGGGCTGCTAGGCTGGGGTGTGGTACACAGGGTGGGG<br>(((....))).. ..(((...))).	CAPN2	NM_001146068	chr1:221955918-222030343	template	37	Intron_9	--	Yes	--	Yes	--	--	--	Yes	--	--	--
G4ST01222008654	chr1:222008655-222008687	-	GGGTAGGAGGGGTCCCTTGTGGGGGGCAGAGGG<br>..(((....)))	CAPN2	NM_001748	chr1:221966742-222030343	template	33	Intron_9	--	Yes	--	Yes	--	--	--	--	--	--	--
G4ST01222008654	chr1:222008655-222008687	-	GGGTAGGAGGGGTCCCTTGTGGGGGGCAGAGGG<br>..(((....)))	CAPN2	NM_001146068	chr1:221955918-222030343	template	33	Intron_9	--	Yes	--	Yes	--	--	--	--	--	--	--
G4ST01222100316	chr1:222100317-222100348	-	GGGCGCGCCGCGGGCGGGGTCGGCGCGGGGGG<br>.((...))	TP53BP2	NM_005426	chr1:222034218-222100297	non-template	-19	upstream	Yes	--	--	--	--	--	--	Yes	Yes	Yes	--
G4ST01222100316	chr1:222100317-222100348	-	GGGCGCGCCGCGGGCGGGGTCGGCGCGGGGGG<br>.((...))	TP53BP2	NM_001031685	chr1:222034218-222100297	non-template	-19	upstream	Yes	--	--	--	--	--	--	Yes	Yes	Yes	--
G4ST01222613497	chr1:222613498-222613523	+	GGGCTTGAATGTTAAGGGTGGGAGGG<br>.(((.....)))	CNIH4	NM_014184	chr1:222611218-222630318	non-template	26	Intron_1.f	--	Yes	--	Yes	--	--	--	--	--	--	--
G4ST01222915801	chr1:222915802-222915835	-	GGGGTCCCCAAGGGAGCAGAAGCTGAGGGATGGG<br>(((....)))..	CNIH3	NM_152495	chr1:222870802-222994872	template	34	Intron_1.f	--	Yes	--	Yes	--	--	--	--	--	--	--
G4ST01222915806	chr1:222915807-222915845	-	GGGAATCAAAGGGGTCCCCAAGGGAGCAGAAGCTGAGGG<br>(((....)))..	CNIH3	NM_152495	chr1:222870802-222994872	template	39	Intron_1.f	--	Yes	--	Yes	--	--	--	--	--	--	--
G4ST01222923128	chr1:222923129-222923162	+	GGGGGAACCTGGGGCTGGGGGTGATTACCTGGGG<br>(((....)))..	CNIH3	NM_152495	chr1:222870802-222994872	non-template	34	Intron_1.f	--	Yes	--	Yes	--	--	--	--	--	--	--
G4ST01222977102	chr1:222977103-222977140	+	GGGAGGCAGGGTGGGAACAGCCAGGCTGTAGAGCAGGG<br>.(((((...)))))......	CNIH3	NM_152495	chr1:222870802-222994872	non-template	38	Intron_3	--	Yes	--	Yes	--	--	--	--	--	--	Yes
G4ST01223320975	chr1:223320976-223320998	-	GGGACTCAGGTGGGAAGGGTGGG<br>((....))	DNAH14	NM_001373	chr1:223183979-223653619	template	23	Intron_17	--	Yes	--	Yes	--	--	--	--	--	--	Yes
G4ST01223682444	chr1:223682445-223682479	-	GGGCGGCCTCGCCGGTGGGGAGGGGAGGCGAGGGG<br>((((...))))...	LBR	NM_002296	chr1:223655827-223682407	non-template	-37	upstream	Yes	--	--	--	--	--	--	Yes	Yes	Yes	Yes
G4ST01223682444	chr1:223682445-223682479	-	GGGCGGCCTCGCCGGTGGGGAGGGGAGGCGAGGGG<br>((((...))))...	LBR	NM_194442	chr1:223655827-223683142	non-template	35	Intron_1.f	--	Yes	--	Yes	--	--	--	Yes	Yes	Yes	Yes
G4ST01223682454	chr1:223682455-223682488	-	GGGAGCCCCGGGCGGCCTCGCCGGTGGGGAGGGG<br>((((...))))...	LBR	NM_002296	chr1:223655827-223682407	non-template	-47	upstream	Yes	--	--	--	--	--	--	Yes	Yes	Yes	Yes
G4ST01223682454	chr1:223682455-223682488	-	GGGAGCCCCGGGCGGCCTCGCCGGTGGGGAGGGG<br>((((...))))...	LBR	NM_194442	chr1:223655827-223683142	non-template	34	Intron_1.f	--	Yes	--	Yes	--	--	--	Yes	Yes	Yes	Yes
G4ST01223786548	chr1:223786549-223786577	+	GGGGACACACAAGTGTGGGGAGGGGCGGG<br>.((((....)))).	ENAH	NM_018212	chr1:223741157-223907468	template	29	Intron_3	--	Yes	--	Yes	--	--	--	--	--	--	--
G4ST01223786548	chr1:223786549-223786577	+	GGGGACACACAAGTGTGGGGAGGGGCGGG<br>.((((....)))).	ENAH	NM_001008493	chr1:223741157-223907468	template	29	Intron_3	--	Yes	--	Yes	--	--	--	--	--	--	--
G4ST01223906377	chr1:223906378-223906405	-	GGGGTGTGTAAGCGGGGGCGGGGAAGGG<br>.(((....))).	ENAH	NM_018212	chr1:223741157-223907468	non-template	28	Intron_1.f	--	Yes	--	Yes	--	--	--	Yes	Yes	Yes	--
G4ST01223906377	chr1:223906378-223906405	-	GGGGTGTGTAAGCGGGGGCGGGGAAGGG<br>.(((....))).	ENAH	NM_001008493	chr1:223741157-223907468	non-template	28	Intron_1.f	--	Yes	--	Yes	--	--	--	Yes	Yes	Yes	--
G4ST01224064557	chr1:224064558-224064585	+	GGGCTCGGCCGCCGGGGTGGGCGGCGGG<br>..(((...)))	EPHX1	NM_001136018	chr1:224064420-224099885	non-template	28	Intron_1.f	--	Yes	--	Yes	--	--	--	Yes	Yes	Yes	Yes
G4ST01224064587	chr1:224064588-224064627	+	GGGGCGCCGAGGGGTCGGGGCTGCGGTCGGCCCGCAGGGG<br>.((((((.....))))))	EPHX1	NM_001136018	chr1:224064420-224099885	non-template	40	Intron_1.f	--	Yes	--	Yes	--	--	--	Yes	Yes	Yes	Yes
G4ST01224064597	chr1:224064598-224064636	+	GGGGTCGGGGCTGCGGTCGGCCCGCAGGGGTGCAGAGGG<br>.((((((.....))))))	EPHX1	NM_001136018	chr1:224064420-224099885	non-template	39	Intron_1.f	--	Yes	--	Yes	--	--	--	Yes	Yes	Yes	Yes
G4ST01224081100	chr1:224081101-224081133	+	GGGGTGATAAGGGCTCTGGTCCAGGGGAGGGGG<br>..(((...)))	EPHX1	NM_000120	chr1:224079625-224099885	non-template	33	Intron_1.f	--	Yes	--	Yes	--	--	--	Yes	Yes	--	--
G4ST01224081100	chr1:224081101-224081133	+	GGGGTGATAAGGGCTCTGGTCCAGGGGAGGGGG<br>..(((...)))	EPHX1	NM_001136018	chr1:224064420-224099885	non-template	33	Intron_1.f	--	Yes	--	Yes	--	--	--	Yes	Yes	--	--
G4ST01224081219	chr1:224081220-224081245	+	GGGTGGGAAGCTGGGAGTTCACTGGG<br>((....))	EPHX1	NM_000120	chr1:224079625-224099885	non-template	26	Intron_1.f	--	Yes	--	Yes	--	--	--	--	--	--	Yes
G4ST01224081219	chr1:224081220-224081245	+	GGGTGGGAAGCTGGGAGTTCACTGGG<br>((....))	EPHX1	NM_001136018	chr1:224064420-224099885	non-template	26	Intron_1.f	--	Yes	--	Yes	--	--	--	--	--	--	Yes
G4ST01224104189	chr1:224104190-224104227	+	GGGCTGGGAGGCCAAGGGCTGGCCCCAGGTCAGAAGGG<br>((((((...))))))..	TMEM63A	NM_014698	chr1:224099856-224137043	template	38	Intron_21	--	Yes	--	Yes	--	--	--	--	--	--	Yes
G4ST01224107216	chr1:224107217-224107244	-	GGGATGGGAAGGACCTTGGGAGGCGGGG<br>(((...)))	TMEM63A	NM_014698	chr1:224099856-224137043	non-template	28	Intron_19	--	Yes	--	Yes	--	--	--	--	--	--	Yes
G4ST01224107224	chr1:224107225-224107248	-	GGGAGGGATGGGAAGGACCTTGGG<br>(((...)))	TMEM63A	NM_014698	chr1:224099856-224137043	non-template	24	Intron_19	--	Yes	--	Yes	--	--	--	--	--	--	Yes
G4ST01224108162	chr1:224108163-224108209	+	GGGAGAGGAGGGGCCCCTAGGCTGGGCAGGAGCAGAAAGCCCCTGGG<br>(((....))). .(((.((.....)).)))	TMEM63A	NM_014698	chr1:224099856-224137043	template	47	Intron_18	--	Yes	--	Yes	--	--	--	--	--	--	--
G4ST01224108171	chr1:224108172-224108213	+	GGGGCCCCTAGGCTGGGCAGGAGCAGAAAGCCCCTGGGAGGG<br>(((....))). .(((.((.....)).)))	TMEM63A	NM_014698	chr1:224099856-224137043	template	42	Intron_18	--	Yes	--	Yes	--	--	--	--	--	--	--
G4ST01224124762	chr1:224124763-224124790	-	GGGAGCCAGGAGGCGGGGGGGGGCGGGG<br>.(((....)))	TMEM63A	NM_014698	chr1:224099856-224137043	non-template	28	Intron_6	--	Yes	--	Yes	--	--	--	--	--	--	--
G4ST01224129445	chr1:224129446-224129482	-	GGGGGATTGCAGGGCCTGCGGGGCCTGCCTTCAGGGG<br>..(((....)))	TMEM63A	NM_014698	chr1:224099856-224137043	non-template	37	Intron_3	--	Yes	--	Yes	--	--	--	--	--	--	--
G4ST01224136472	chr1:224136473-224136499	-	GGGGGTGACGACGCGGGATTGGGTGGG<br>.(((....)))	TMEM63A	NM_014698	chr1:224099856-224137043	non-template	27	Intron_1.f	--	Yes	--	Yes	--	--	--	--	Yes	Yes	Yes
G4ST01224137093	chr1:224137094-224137127	+	GGGAGAGTGGGGTTGCGGGGGTGGTAGCATGGGG<br>.(((....))).	TMEM63A	NM_014698	chr1:224099856-224137043	template	-50	upstream	Yes	--	--	--	--	--	--	Yes	Yes	Yes	--
G4ST01224137467	chr1:224137468-224137503	+	GGGACTGCTGGGCGCTGCGCGGGAAGCGAGGCTGGG<br>.((...)) .(((...)))	TMEM63A	NM_014698	chr1:224099856-224137043	template	-424	upstream	Yes	--	--	--	--	--	--	Yes	Yes	Yes	--
G4ST01224137476	chr1:224137477-224137510	+	GGGCGCTGCGCGGGAAGCGAGGCTGGGTTCGGGG<br>.((...)) .(((...)))	TMEM63A	NM_014698	chr1:224099856-224137043	template	-433	upstream	Yes	--	--	--	--	--	--	Yes	Yes	Yes	--
G4ST01224141449	chr1:224141450-224141478	+	GGGAGGGCTGAGGTCAGAGGGCGTCTGGG<br>(((....))).	LEFTY1	NM_020997	chr1:224140605-224143469	template	29	Intron_3.l	--	Yes	--	Yes	--	--	--	--	Yes	--	--
G4ST01224195558	chr1:224195559-224195602	+	GGGCCAGCTTTATAGCTGGCCCTGGGCTGGCCTGGGGTGAAGGG<br>((((((....)))))))....	LEFTY2	NM_001172425	chr1:224190921-224195706	template	44	Exon_1.f	--	Yes	Yes	--	--	Yes	--	Yes	--	--	Yes
G4ST01224195558	chr1:224195559-224195602	+	GGGCCAGCTTTATAGCTGGCCCTGGGCTGGCCTGGGGTGAAGGG<br>((((((....)))))))....	LEFTY2	NM_003240	chr1:224190921-224195706	template	44	Exon_1.f	--	Yes	Yes	--	--	Yes	--	Yes	--	--	Yes
G4ST01224316533	chr1:224316534-224316572	+	GGGTCTCCTCGGGGGCGCGCGCCGGGGCCCGCGCGGGGG<br>(((....))).	LOC440926	NR_002315	chr1:224317051-224326326	non-template	-478	upstream	Yes	--	--	--	--	--	--	Yes	Yes	Yes	--
G4ST01224316533	chr1:224316534-224316572	+	GGGTCTCCTCGGGGGCGCGCGCCGGGGCCCGCGCGGGGG<br>(((....))).	H3F3A	NM_002107	chr1:224317031-224326326	non-template	-458	upstream	Yes	--	--	--	--	--	--	Yes	Yes	Yes	--
G4ST01224441222	chr1:224441223-224441254	-	GGGATCCGAAAGGATGGGGTGCGGGGGAGGGG<br>((((....)))).	ACBD3	NM_022735	chr1:224399003-224441046	non-template	-176	upstream	Yes	--	--	--	--	--	--	Yes	Yes	Yes	Yes

G4ST01224563781	chr1:224563782-224563816	-	GGGCCGCGGAACATGCGCCGGGAGCGGGGCCCGGG<br>.((((.....))))..	LIN9	NM_173083	chr1:224485486-224563821	non-template	35	Exon_1.f	--	Yes	Yes	--	--	Yes	--	Yes	Yes	Yes	Yes
G4ST01224643207	chr1:224643208-224643234	-	GGGCACTGAGTGGGGCAGGGGTTAGGG<br>(((...)))	PARP1	NM_001618	chr1:224615015-224662424	non-template	27	Intron_4	--	Yes	--	Yes	--	--	--	Yes	--	--	--
G4ST01224643319	chr1:224643320-224643346	+	GGGAGAGGGGCTAGGGTTGTTCCAGGG<br>.((...))	PARP1	NM_001618	chr1:224615015-224662424	template	27	Intron_4	--	Yes	--	Yes	--	--	--	--	--	--	--
G4ST01224858027	chr1:224858028-224858057	-	GGGATCCATGATAAGGGGTGGGCAGCAGGG<br>(((...)))...	C1orf95	NM_001003665	chr1:224803124-224863538	template	30	Exon_4.l	--	Yes	Yes	--	--	--	Yes	--	--	--	--
G4ST01224860082	chr1:224860083-224860118	-	GGGAGCATGGGTGTGAGGGCTGCCCAGGCAGGTGGG<br>((((....))))..	C1orf95	NM_001003665	chr1:224803124-224863538	template	36	Exon_4.l	--	Yes	Yes	--	--	--	Yes	--	--	--	--
G4ST01224892304	chr1:224892305-224892337	-	GGGTTTATAGGGCTCCATGGAGAGGGCACTGGG<br>(((....))).	ITPKB	NM_002221	chr1:224886014-224993499	non-template	33	Intron_6	--	Yes	--	Yes	--	--	--	--	Yes	--	--
G4ST01224894778	chr1:224894779-224894813	+	GGGTGCAGGAGGGGCCGAGAGCCGGGGGCTGTGGG<br>.(((.....)))	ITPKB	NM_002221	chr1:224886014-224993499	template	35	Intron_5	--	Yes	--	Yes	--	--	--	--	--	--	--
G4ST01224898064	chr1:224898065-224898099	+	GGGCTGGAGGGGGAAGTCAGTAGCTTTGGGTTGGG<br>..((((.....)))).	ITPKB	NM_002221	chr1:224886014-224993499	template	35	Intron_4	--	Yes	--	Yes	--	--	--	Yes	Yes	--	--
G4ST01224905017	chr1:224905018-224905055	+	GGGGCTGACAGGCTTCAGGGGTGGTGTGGGAGGAGGGG<br>.((((......))))	ITPKB	NM_002221	chr1:224886014-224993499	template	38	Intron_2	--	Yes	--	Yes	--	--	--	Yes	Yes	--	--
G4ST01224977241	chr1:224977242-224977276	-	GGGTGGGGGGCAGGGGGAGCAGGTAGCCTGCAGGG<br>...(((((...))))).	ITPKB	NM_002221	chr1:224886014-224993499	non-template	35	Intron_2	--	Yes	--	Yes	--	--	--	Yes	--	--	--
G4ST01224991905	chr1:224991906-224991937	+	GGGGCGGCATGGCCTGGGCAGCGGGCTGGGGG<br>..(((...))).	ITPKB	NM_002221	chr1:224886014-224993499	template	32	Exon_2	--	Yes	Yes	--	--	Yes	--	--	Yes	Yes	--
G4ST01225146224	chr1:225146225-225146268	+	GGGTGGAGGCTCCCTGCAGCCTGGGTGGAGGAGGGCATGAGGGG<br>...(((((......)))))	PSEN2	NM_012486	chr1:225124896-225150427	non-template	44	Intron_11	--	Yes	--	Yes	--	--	--	--	--	--	Yes
G4ST01225146224	chr1:225146225-225146268	+	GGGTGGAGGCTCCCTGCAGCCTGGGTGGAGGAGGGCATGAGGGG<br>...(((((......)))))	PSEN2	NM_000447	chr1:225124896-225150427	non-template	44	Intron_11	--	Yes	--	Yes	--	--	--	--	--	--	Yes
G4ST01225194557	chr1:225194558-225194593	+	GGGAGCGGCCCGCGGGGCGGGGCTTGTGAGCTGGGG<br>.(((...))). (((....)))..	ADCK3	NM_020247	chr1:225194561-225241869	non-template	33	upstream-Exon_1.f	Yes	Yes	Yes	--	--	Yes	--	Yes	Yes	Yes	--
G4ST01225194570	chr1:225194571-225194608	+	GGGGCGGGGCTTGTGAGCTGGGGCTCCCCCGGAGCGGG<br>(((....))).. (((((...)))))	ADCK3	NM_020247	chr1:225194561-225241869	non-template	38	Exon_1.f	--	Yes	Yes	--	--	Yes	--	Yes	Yes	Yes	--
G4ST01225233882	chr1:225233883-225233921	-	GGGTGTCTCTGGGGGTCCCCATGGACGGGGGTGGAAGGG<br>.((((....))))..	ADCK3	NM_020247	chr1:225194561-225241869	template	39	Intron_5	--	Yes	--	Yes	--	--	--	--	--	Yes	--
G4ST01225233890	chr1:225233891-225233944	-	GGGCTGCAGTGTGGCCAGACACTGGGTGTCTCTGGGGGTCCCCATGGACGGGGG<br>....(((((......))))) .((((....))))..	ADCK3	NM_020247	chr1:225194561-225241869	template	54	Intron_5	--	Yes	--	Yes	--	--	--	--	--	Yes	--
G4ST01225233906	chr1:225233907-225233953	-	GGGCTGTGAGGGCTGCAGTGTGGCCAGACACTGGGTGTCTCTGGGGG<br>....(((((......)))))	ADCK3	NM_020247	chr1:225194561-225241869	template	47	Intron_5	--	Yes	--	Yes	--	--	--	--	--	Yes	--
G4ST01225236450	chr1:225236451-225236490	+	GGGCCAGATGCTGAGCATCCAGGGTGAGTGGGCGCGGGGG<br>...(((((...)))))..	ADCK3	NM_020247	chr1:225194561-225241869	non-template	40	Exon_6-Intron_6	--	Yes	Yes	Yes	Yes	--	--	--	--	--	--
G4ST01225236471	chr1:225236472-225236503	+	GGGTGAGTGGGCGCGGGGGCTGCTGCCCCGGG<br>(((....)))..	ADCK3	NM_020247	chr1:225194561-225241869	non-template	32	Exon_6-Intron_6	--	Yes	Yes	Yes	Yes	--	--	--	--	--	Yes
G4ST01225236479	chr1:225236480-225236513	+	GGGCGCGGGGGCTGCTGCCCCGGGACTGCGTGGG<br>(((....)))..	ADCK3	NM_020247	chr1:225194561-225241869	non-template	34	Intron_6	--	Yes	--	Yes	--	--	--	--	--	--	Yes
G4ST01225236485	chr1:225236486-225236521	+	GGGGGCTGCTGCCCCGGGACTGCGTGGGCTGCTGGG<br>(((....)))..	ADCK3	NM_020247	chr1:225194561-225241869	non-template	36	Intron_6	--	Yes	--	Yes	--	--	--	--	--	--	Yes
G4ST01225239311	chr1:225239312-225239342	+	GGGACAGGATGCTGGGGGACTCGGGGTAGGG<br>.(((....))).	ADCK3	NM_020247	chr1:225194561-225241869	non-template	31	Intron_13	--	Yes	--	Yes	--	--	--	--	Yes	Yes	--
G4ST01225239972	chr1:225239973-225240000	-	GGGAGGCTCGATGAGGGGAGGGCTGGGG<br>...(((...)))	ADCK3	NM_020247	chr1:225194561-225241869	template	28	Intron_14.l	--	Yes	--	Yes	--	--	--	--	Yes	Yes	--
G4ST01225244640	chr1:225244641-225244679	+	GGGGAGGGCCCAGCGTGCTGCCAGTGCTGGGGGGCAGGG<br>(((((((.........)))))))	CDC42BPA	NM_003607	chr1:225244189-225572449	template	39	Exon_36.l	--	Yes	Yes	--	--	--	Yes	Yes	Yes	--	--
G4ST01225244640	chr1:225244641-225244679	+	GGGGAGGGCCCAGCGTGCTGCCAGTGCTGGGGGGCAGGG<br>(((((((.........)))))))	CDC42BPA	NM_014826	chr1:225244189-225572449	template	39	Exon_35.l	--	Yes	Yes	--	--	--	Yes	Yes	Yes	--	--
G4ST01225497484	chr1:225497485-225497518	+	GGGACCTGTCAGAGGACAGAGGGTGGGAGGAGGG<br>..(((((....))))).	CDC42BPA	NM_003607	chr1:225244189-225572449	template	34	Intron_2	--	Yes	--	Yes	--	--	--	Yes	--	--	--
G4ST01225497484	chr1:225497485-225497518	+	GGGACCTGTCAGAGGACAGAGGGTGGGAGGAGGG<br>..(((((....))))).	CDC42BPA	NM_014826	chr1:225244189-225572449	template	34	Intron_2	--	Yes	--	Yes	--	--	--	Yes	--	--	--
G4ST01225522140	chr1:225522141-225522176	-	GGGAGGGACCTGGTGGGAGGTGATTGGATCATGGGG<br>..((((.....)))).	CDC42BPA	NM_003607	chr1:225244189-225572449	non-template	36	Intron_1.f	--	Yes	--	Yes	--	--	--	Yes	--	--	--
G4ST01225522140	chr1:225522141-225522176	-	GGGAGGGACCTGGTGGGAGGTGATTGGATCATGGGG<br>..((((.....)))).	CDC42BPA	NM_014826	chr1:225244189-225572449	non-template	36	Intron_1.f	--	Yes	--	Yes	--	--	--	Yes	--	--	--
G4ST01225869154	chr1:225869155-225869188	-	GGGTTGGTGGGACAGTCTGTGTGGGCATTCTGGG<br>(((.....)))	ZNF678	NM_178549	chr1:225817843-225916787	template	34	Intron_2	--	Yes	--	Yes	--	--	--	--	--	--	--
G4ST01225869154	chr1:225869155-225869188	-	GGGTTGGTGGGACAGTCTGTGTGGGCATTCTGGG<br>(((.....)))	ZNF678	NR_033184	chr1:225817843-225916787	template	34	Intron_1.f	--	Yes	--	Yes	--	--	--	--	--	--	--
G4ST01225982640	chr1:225982641-225982680	+	GGGGCTGCAGGGCCGGTGCTTGCCACCGGAGCGGGGGGGG<br>((((((.....)))))).. .	LOC100130093	NR_024485	chr1:225982863-225988678	non-template	-182	upstream	Yes	--	--	--	--	--	--	Yes	Yes	Yes	--
G4ST01225982640	chr1:225982641-225982680	+	GGGGCTGCAGGGCCGGTGCTTGCCACCGGAGCGGGGGGGG<br>((((((.....))))))...	LOC100130093	NR_024486	chr1:225982863-225988678	non-template	-182	upstream	Yes	--	--	--	--	--	--	Yes	Yes	Yes	--
G4ST01225982649	chr1:225982650-225982684	+	GGGCCGGTGCTTGCCACCGGAGCGGGGGGGGCGGG<br>((((((.....))))))...	LOC100130093	NR_024485	chr1:225982863-225988678	non-template	-178	upstream	Yes	--	--	--	--	--	--	Yes	Yes	Yes	--
G4ST01225982649	chr1:225982650-225982684	+	GGGCCGGTGCTTGCCACCGGAGCGGGGGGGGCGGG<br>((((((.....))))))...	LOC100130093	NR_024486	chr1:225982863-225988678	non-template	-178	upstream	Yes	--	--	--	--	--	--	Yes	Yes	Yes	--
G4ST01225982721	chr1:225982722-225982754	-	GGGCGGGCGGCGGGGCCCAGGACGACCCTGGGG<br>...((((.....))))	LOC100130093	NR_024485	chr1:225982863-225988678	template	-108	upstream	Yes	--	--	--	--	--	--	Yes	Yes	Yes	Yes
G4ST01225982721	chr1:225982722-225982754	-	GGGCGGGCGGCGGGGCCCAGGACGACCCTGGGG<br>...((((.....))))	LOC100130093	NR_024486	chr1:225982863-225988678	template	-108	upstream	Yes	--	--	--	--	--	--	Yes	Yes	Yes	Yes
G4ST01225987483	chr1:225987484-225987514	+	GGGCTTGCTCAGCAGGGAAGTGGGTGGCGGG<br>..(((...)))	JMJD4	NM_023007	chr1:225985513-225989735	template	31	Intron_4	--	Yes	--	Yes	--	--	--	--	--	Yes	--
G4ST01225987483	chr1:225987484-225987514	+	GGGCTTGCTCAGCAGGGAAGTGGGTGGCGGG<br>..(((...)))	JMJD4	NM_001161465	chr1:225985513-225989735	template	31	Intron_4	--	Yes	--	Yes	--	--	--	--	--	Yes	--
G4ST01225987483	chr1:225987484-225987514	+	GGGCTTGCTCAGCAGGGAAGTGGGTGGCGGG<br>..(((...)))	LOC100130093	NR_024485	chr1:225982863-225988678	non-template	31	Exon_3.l	--	Yes	Yes	--	--	--	--	--	--	Yes	--
G4ST01225987483	chr1:225987484-225987514	+	GGGCTTGCTCAGCAGGGAAGTGGGTGGCGGG<br>..(((...)))	LOC100130093	NR_024486	chr1:225982863-225988678	non-template	31	Exon_2.l	--	Yes	Yes	--	--	--	--	--	--	Yes	--
G4ST01225987483	chr1:225987484-225987514	+	GGGCTTGCTCAGCAGGGAAGTGGGTGGCGGG<br>..(((...)))	SNAP47	NM_053052	chr1:225989320-226035555	non-template	-1805	upstream	Yes	--	--	--	--	--	--	--	--	Yes	--
G4ST01225989996	chr1:225989997-225990032	+	GGGGGCGGGAGTGCGGCGGTGCGGGGGCGCCCAGGG<br>..((((....)))).	JMJD4	NM_023007	chr1:225985513-225989735	template	-261	upstream	Yes	--	--	--	--	--	--	Yes	Yes	Yes	--
G4ST01225989996	chr1:225989997-225990032	+	GGGGGCGGGAGTGCGGCGGTGCGGGGGCGCCCAGGG<br>..((((....)))).	JMJD4	NM_001161465	chr1:225985513-225989735	template	-261	upstream	Yes	--	--	--	--	--	--	Yes	Yes	Yes	--
G4ST01225989996	chr1:225989997-225990032	+	GGGGGCGGGAGTGCGGCGGTGCGGGGGCGCCCAGGG<br>..((((....)))).	SNAP47	NM_053052	chr1:225989320-226035555	non-template	36	Intron_1.f	--	Yes	--	Yes	--	--	--	Yes	Yes	Yes	--
G4ST01225990002	chr1:225990003-225990040	+	GGGAGTGCGGCGGTGCGGGGGCGCCCAGGGATGATGGG<br>..((((....)))).	JMJD4	NM_023007	chr1:225985513-225989735	template	-267	upstream	Yes	--	--	--	--	--	--	Yes	Yes	Yes	--
G4ST01225990002	chr1:225990003-225990040	+	GGGAGTGCGGCGGTGCGGGGGCGCCCAGGGATGATGGG<br>..((((....)))).	JMJD4	NM_001161465	chr1:225985513-225989735	template	-267	upstream	Yes	--	--	--	--	--	--	Yes	Yes	Yes	--
G4ST01225990002	chr1:225990003-225990040	+	GGGAGTGCGGCGGTGCGGGGGCGCCCAGGGATGATGGG<br>..((((....)))).	SNAP47	NM_053052	chr1:225989320-226035555	non-template	38	Intron_1.f	--	Yes	--	Yes	--	--	--	Yes	Yes	Yes	--
G4ST01225990624	chr1:225990625-225990660	+	GGGTAGGGATTTGGGGCTGTGTACACATGAGCAGGG<br>(((.((....))..))).	JMJD4	NM_023007	chr1:225985513-225989735	template	-889	upstream	Yes	--	--	--	--	--	--	--	Yes	--	Yes

G4ST01225990624	chr1:225990625-225990660	+	GGGTAGGGATTTGGGGCTGTGTACACATGAGCAGGG<br>(((.((....))..))).	JMJD4	NM_001161465	chr1:225985513-225989735	template	-889	upstream	Yes	--	--	--	--	--	--	--	Yes	--	Yes
G4ST01225990624	chr1:225990625-225990660	+	GGGTAGGGATTTGGGGCTGTGTACACATGAGCAGGG<br>(((.((....))..))).	SNAP47	NM_053052	chr1:225989320-226035555	non-template	36	Intron_1.f	--	Yes	--	Yes	--	--	--	--	Yes	--	Yes
G4ST01225990629	chr1:225990630-225990670	+	GGGATTTGGGGCTGTGTACACATGAGCAGGGCTGAAGTGGG<br>(((.((....))..))).	JMJD4	NM_023007	chr1:225985513-225989735	template	-894	upstream	Yes	--	--	--	--	--	--	--	Yes	--	Yes
G4ST01225990629	chr1:225990630-225990670	+	GGGATTTGGGGCTGTGTACACATGAGCAGGGCTGAAGTGGG<br>(((.((....))..))).	JMJD4	NM_001161465	chr1:225985513-225989735	template	-894	upstream	Yes	--	--	--	--	--	--	--	Yes	--	Yes
G4ST01225990629	chr1:225990630-225990670	+	GGGATTTGGGGCTGTGTACACATGAGCAGGGCTGAAGTGGG<br>(((.((....))..))).	SNAP47	NM_053052	chr1:225989320-226035555	non-template	41	Intron_1.f	--	Yes	--	Yes	--	--	--	--	Yes	--	Yes
G4ST01225991390	chr1:225991391-225991427	+	GGGGAAAACCTGTGGTTGGGGCACAGAGGGATCAGGG<br>...((((...)))).	JMJD4	NM_023007	chr1:225985513-225989735	template	-1655	upstream	Yes	--	--	--	--	--	--	--	--	--	--
G4ST01225991390	chr1:225991391-225991427	+	GGGGAAAACCTGTGGTTGGGGCACAGAGGGATCAGGG<br>...((((...)))).	JMJD4	NM_001161465	chr1:225985513-225989735	template	-1655	upstream	Yes	--	--	--	--	--	--	--	--	--	--
G4ST01225991390	chr1:225991391-225991427	+	GGGGAAAACCTGTGGTTGGGGCACAGAGGGATCAGGG<br>...((((...)))).	SNAP47	NM_053052	chr1:225989320-226035555	non-template	37	Intron_1.f	--	Yes	--	Yes	--	--	--	--	--	--	--
G4ST01225997075	chr1:225997076-225997111	+	GGGCTGAGCAGGGAGGGCCACAGCCCCTCCTGTGGG<br>..((((......))))	SNAP47	NM_053052	chr1:225989320-226035555	non-template	36	Intron_1.f	--	Yes	--	Yes	--	--	--	--	--	--	--
G4ST01226176339	chr1:226176340-226176361	+	GGGAGGGAGGGCAGTGTGAGGG<br>((...)).	WNT9A	NM_003395	chr1:226175788-226202299	template	22	Intron_3.l	--	Yes	--	Yes	--	--	--	--	--	--	Yes
G4ST01226179165	chr1:226179166-226179189	+	GGGAGGGGGCGGGAGGAGGCTGGG<br>((....))	WNT9A	NM_003395	chr1:226175788-226202299	template	24	Intron_2	--	Yes	--	Yes	--	--	--	--	--	--	--
G4ST01226179169	chr1:226179170-226179193	+	GGGGGCGGGAGGAGGCTGGGTGGG<br>((....))	WNT9A	NM_003395	chr1:226175788-226202299	template	24	Intron_2	--	Yes	--	Yes	--	--	--	--	--	--	--
G4ST01226179175	chr1:226179176-226179197	+	GGGAGGAGGCTGGGTGGGAGGG<br>((....))	WNT9A	NM_003395	chr1:226175788-226202299	template	22	Intron_2	--	Yes	--	Yes	--	--	--	--	--	--	--
G4ST01226180653	chr1:226180654-226180687	-	GGGGGGAGGGGGCACCCAGTGGGGTGGAGCCGGG<br>..(((...)))	WNT9A	NM_003395	chr1:226175788-226202299	non-template	34	Intron_1.f	--	Yes	--	Yes	--	--	--	--	--	--	--
G4ST01226181877	chr1:226181878-226181910	+	GGGCTAGGGATGGGATGTCCCAGGACATCTGGG<br>(((((....)))))..	WNT9A	NM_003395	chr1:226175788-226202299	template	33	Intron_1.f	--	Yes	--	Yes	--	--	--	--	--	--	--
G4ST01226195542	chr1:226195543-226195582	+	GGGAGAGCCTCAGGCTTGAGGGAGTCTTGGGATAGGAGGG<br>..((((...))))...	WNT9A	NM_003395	chr1:226175788-226202299	template	40	Intron_1.f	--	Yes	--	Yes	--	--	--	--	--	--	--
G4ST01226196440	chr1:226196441-226196481	+	GGGCAGGCACTGGGGCCCTCTGCAGCCTGCAGCAGGGCGGG<br>(((...))) .....(((((...)))))..	WNT9A	NM_003395	chr1:226175788-226202299	template	41	Intron_1.f	--	Yes	--	Yes	--	--	--	Yes	--	--	--
G4ST01226202547	chr1:226202548-226202578	+	GGGGGGCCGGAGGCGGGCATCGGGAAGCGGG<br>..(((...)))	WNT9A	NM_003395	chr1:226175788-226202299	template	-248	upstream	Yes	--	--	--	--	--	--	--	Yes	Yes	--
G4ST01226202601	chr1:226202602-226202629	+	GGGAGCTTGGGGCGGCCCCCGGGGCGGG<br>.(((...)))	WNT9A	NM_003395	chr1:226175788-226202299	template	-302	upstream	Yes	--	--	--	--	--	--	--	Yes	Yes	Yes
G4ST01226202609	chr1:226202610-226202639	+	GGGGCGGCCCCCGGGGCGGGATTGAGGGGG<br>.(((...)))	WNT9A	NM_003395	chr1:226175788-226202299	template	-310	upstream	Yes	--	--	--	--	--	--	--	Yes	Yes	Yes
G4ST01226202634	chr1:226202635-226202666	+	GGGGGAGGGGAGGGGAGGCGTTGGCCGCCGGG<br>..((((.....))))	WNT9A	NM_003395	chr1:226175788-226202299	template	-335	upstream	Yes	--	--	--	--	--	--	Yes	Yes	Yes	Yes
G4ST01226203819	chr1:226203820-226203843	+	GGGTGGGGAAGGGAGGAGACCGGG<br>.((...))	WNT9A	NM_003395	chr1:226175788-226202299	template	-1520	upstream	Yes	--	--	--	--	--	--	--	--	--	--
G4ST01226260695	chr1:226260696-226260726	-	GGGTCCGGGATGGGTGCAGCCTGCAATGGGG<br>((((...))))...	WNT3A	NM_033131	chr1:226261346-226315595	template	-619	upstream	Yes	--	--	--	--	--	--	Yes	--	--	--
G4ST01226261195	chr1:226261196-226261225	-	GGGGGGCGCCCGCCGGGGCCGGGGCCCGGG<br>.(((....))).	WNT3A	NM_033131	chr1:226261346-226315595	template	-120	upstream	Yes	--	--	--	--	--	--	--	--	--	Yes
G4ST01226261290	chr1:226261291-226261331	-	GGGTGTGCGCGGGCTCCGAGGGCGGCGCTGCGCCAGGTGGG<br>.((((...))))....	WNT3A	NM_033131	chr1:226261346-226315595	template	-14	upstream	Yes	--	--	--	--	--	--	--	--	--	Yes
G4ST01226261608	chr1:226261609-226261638	-	GGGGCTGGAGCAGAAGGGCGCGGGCTCGGG<br>.(((...)))..	WNT3A	NM_033131	chr1:226261346-226315595	template	30	Intron_1.f	--	Yes	--	Yes	--	--	--	Yes	--	--	Yes
G4ST01226271109	chr1:226271110-226271149	+	GGGGAGGTACGTGTGTCACACGCGGGAGAGGGGGCAGGGG<br>......(((((...))))).	WNT3A	NM_033131	chr1:226261346-226315595	non-template	40	Intron_1.f	--	Yes	--	Yes	--	--	--	--	--	--	Yes
G4ST01226272542	chr1:226272543-226272588	-	GGGCCAGGCTTGCCTGGGACAAGGGACAGACAGGCCTGTCTCAGGG<br>..(((....))) ..(((((....)))))..	WNT3A	NM_033131	chr1:226261346-226315595	template	46	Intron_1.f	--	Yes	--	Yes	--	--	--	--	--	--	--
G4ST01226277236	chr1:226277237-226277275	+	GGGGGTGGTCTGGGGGAGGGCAGATGAGTCTGGAGTGGG<br>((((....))))....	WNT3A	NM_033131	chr1:226261346-226315595	non-template	39	Intron_2	--	Yes	--	Yes	--	--	--	--	--	--	--
G4ST01226291434	chr1:226291435-226291467	-	GGGCTCTGGGAAGTGTCCACTGGGAGTTGTGGG<br>.(((....)))	WNT3A	NM_033131	chr1:226261346-226315595	template	33	Intron_2	--	Yes	--	Yes	--	--	--	--	--	--	--
G4ST01226291564	chr1:226291565-226291594	-	GGGGTTCACAGAATAGGGCAGGGGGTGGGG<br>((((...)))).	WNT3A	NM_033131	chr1:226261346-226315595	template	30	Intron_2	--	Yes	--	Yes	--	--	--	--	--	--	--
G4ST01226291569	chr1:226291570-226291605	-	GGGGATACCCTGGGGTTCACAGAATAGGGCAGGGGG<br>((((...)))).	WNT3A	NM_033131	chr1:226261346-226315595	template	36	Intron_2	--	Yes	--	Yes	--	--	--	--	--	--	--
G4ST01226300292	chr1:226300293-226300328	+	GGGAGGGAGTCAGTGGGCAAATCTTCCTGGTTGGGG<br>..((((.....)))).	WNT3A	NM_033131	chr1:226261346-226315595	non-template	36	Intron_2	--	Yes	--	Yes	--	--	--	Yes	--	--	--
G4ST01226300296	chr1:226300297-226300333	+	GGGAGTCAGTGGGCAAATCTTCCTGGTTGGGGAGGGG<br>..((((.....)))).	WNT3A	NM_033131	chr1:226261346-226315595	non-template	37	Intron_2	--	Yes	--	Yes	--	--	--	Yes	--	--	--
G4ST01226301371	chr1:226301372-226301411	+	GGGCCACCTGGGAAGGCCACAGCAGCCTGGGCGGCTGGGG<br>.((((.......))))	WNT3A	NM_033131	chr1:226261346-226315595	non-template	40	Intron_2	--	Yes	--	Yes	--	--	--	--	--	--	--
G4ST01226306471	chr1:226306472-226306501	+	GGGTAAAGATTTCTTTCTGGGGGGGTGGGG<br>.((((....))))..	WNT3A	NM_033131	chr1:226261346-226315595	non-template	30	Intron_3.l	--	Yes	--	Yes	--	--	--	--	--	--	Yes
G4ST01226336968	chr1:226336969-226337001	-	GGGGCACGCCGGGTAAGGGGCGGAGACGCCGGG<br>(((....))).	ARF1	NM_001658	chr1:226336984-226353536	template	18	upstream-Exon_1.f	Yes	Yes	Yes	--	--	Yes	--	Yes	Yes	Yes	Yes
G4ST01226336968	chr1:226336969-226337001	-	GGGGCACGCCGGGTAAGGGGCGGAGACGCCGGG<br>(((....))).	ARF1	NM_001024228	chr1:226337474-226353536	template	-472	upstream	Yes	--	--	--	--	--	--	Yes	Yes	Yes	Yes
G4ST01226336968	chr1:226336969-226337001	-	GGGGCACGCCGGGTAAGGGGCGGAGACGCCGGG<br>(((....))).	ARF1	NM_001024227	chr1:226337474-226353536	template	-472	upstream	Yes	--	--	--	--	--	--	Yes	Yes	Yes	Yes
G4ST01226336968	chr1:226336969-226337001	-	GGGGCACGCCGGGTAAGGGGCGGAGACGCCGGG<br>(((....))).	ARF1	NM_001024226	chr1:226336984-226353536	template	18	upstream-Exon_1.f	Yes	Yes	Yes	--	--	Yes	--	Yes	Yes	Yes	Yes
G4ST01226352122	chr1:226352123-226352162	-	GGGTTGGTGGGAAGGGCTGTCCACAGGACAGAGAAAGGGG<br>((((((...)))))))......	ARF1	NM_001658	chr1:226336984-226353536	template	40	Intron_4.l	--	Yes	--	Yes	--	--	--	--	Yes	Yes	Yes
G4ST01226352122	chr1:226352123-226352162	-	GGGTTGGTGGGAAGGGCTGTCCACAGGACAGAGAAAGGGG<br>((((((...)))))))......	ARF1	NM_001024228	chr1:226337474-226353536	template	40	Intron_4.l	--	Yes	--	Yes	--	--	--	--	Yes	Yes	Yes
G4ST01226352122	chr1:226352123-226352162	-	GGGTTGGTGGGAAGGGCTGTCCACAGGACAGAGAAAGGGG<br>((((((...)))))))......	ARF1	NM_001024227	chr1:226337474-226353536	template	40	Intron_4.l	--	Yes	--	Yes	--	--	--	--	Yes	Yes	Yes
G4ST01226352122	chr1:226352123-226352162	-	GGGTTGGTGGGAAGGGCTGTCCACAGGACAGAGAAAGGGG<br>((((((...)))))))......	ARF1	NM_001024226	chr1:226336984-226353536	template	40	Intron_4.l	--	Yes	--	Yes	--	--	--	--	Yes	Yes	Yes
G4ST01226357161	chr1:226357162-226357189	-	GGGACGGCGCTGCCGGGGCAGGGTGGGG<br>.((((...))))	C1orf35	NM_024319	chr1:226355051-226357645	non-template	28	Intron_2	--	Yes	--	Yes	--	--	--	Yes	Yes	Yes	Yes
G4ST01226394847	chr1:226394848-226394891	+	GGGCGGACGGGCTGGCAACGCAGCGGAGCCCGGGTCCCGGAGGG<br>..(((..((...))..))).	GUK1	NM_000858	chr1:226394552-226403278	non-template	44	Intron_1.f	--	Yes	--	Yes	--	--	--	Yes	Yes	Yes	--
G4ST01226394847	chr1:226394848-226394891	+	GGGCGGACGGGCTGGCAACGCAGCGGAGCCCGGGTCCCGGAGGG<br>..(((..((...))..))).	GUK1	NM_001159390	chr1:226394552-226403278	non-template	44	Intron_1.f	--	Yes	--	Yes	--	--	--	Yes	Yes	Yes	--
G4ST01226394855	chr1:226394856-226394896	+	GGGCTGGCAACGCAGCGGAGCCCGGGTCCCGGAGGGCGGGG<br>..(((..((...))..))).	GUK1	NM_000858	chr1:226394552-226403278	non-template	41	Intron_1.f	--	Yes	--	Yes	--	--	--	Yes	Yes	Yes	--
G4ST01226394855	chr1:226394856-226394896	+	GGGCTGGCAACGCAGCGGAGCCCGGGTCCCGGAGGGCGGGG<br>..(((..((...))..))).	GUK1	NM_001159390	chr1:226394552-226403278	non-template	41	Intron_1.f	--	Yes	--	Yes	--	--	--	Yes	Yes	Yes	--
G4ST01226394878	chr1:226394879-226394914	+	GGGTCCCGGAGGGCGGGGCGCTCCTCAGGAGCTGGG<br>..(((((...))))).	GUK1	NM_000858	chr1:226394552-226403278	non-template	36	Intron_1.f	--	Yes	--	Yes	--	--	--	Yes	Yes	Yes	--

G4ST01226394878	chr1:226394879-226394914	+	GGGTCCCGGAGGGCGGGGCGCTCCTCAGGAGCTGGG<br>..(((((...))))).	GUK1	NM_001159390	chr1:226394552-226403278	non-template	36	Intron_1.f	--	Yes	--	Yes	--	--	--	Yes	Yes	Yes	--
G4ST01226397120	chr1:226397121-226397155	+	GGGGTTTGGGGCCGAGGAGGGGATGGAGACCAGGG<br>..(((...)))	GUK1	NM_000858	chr1:226394552-226403278	non-template	35	Intron_2	--	Yes	--	Yes	--	--	--	--	--	--	Yes
G4ST01226397120	chr1:226397121-226397155	+	GGGGTTTGGGGCCGAGGAGGGGATGGAGACCAGGG<br>..(((...)))	GUK1	NM_001159390	chr1:226394552-226403278	non-template	35	Intron_1.f	--	Yes	--	Yes	--	--	--	--	--	--	Yes
G4ST01226397120	chr1:226397121-226397155	+	GGGGTTTGGGGCCGAGGAGGGGATGGAGACCAGGG<br>..(((...)))	GUK1	NM_001159391	chr1:226399027-226403278	non-template	-1871	upstream	Yes	--	--	--	--	--	--	--	--	--	Yes
G4ST01226400765	chr1:226400766-226400799	-	GGGTTGCACGGCAAGGGGCGGGGGTGCTGTTGGG<br>((((...)))).	GUK1	NM_000858	chr1:226394552-226403278	template	34	Intron_4	--	Yes	--	Yes	--	--	--	--	--	--	Yes
G4ST01226400765	chr1:226400766-226400799	-	GGGTTGCACGGCAAGGGGCGGGGGTGCTGTTGGG<br>((((...)))).	GUK1	NM_001159390	chr1:226394552-226403278	template	34	Intron_3	--	Yes	--	Yes	--	--	--	--	--	--	Yes
G4ST01226400765	chr1:226400766-226400799	-	GGGTTGCACGGCAAGGGGCGGGGGTGCTGTTGGG<br>((((...)))).	GUK1	NM_001159391	chr1:226399027-226403278	template	34	Intron_3	--	Yes	--	Yes	--	--	--	--	--	--	Yes
G4ST01226401281	chr1:226401282-226401315	+	GGGTGGGCTCCCAGGGTTGCTGTTGGCAACAGGG<br>((((.....))))..	GUK1	NM_000858	chr1:226394552-226403278	non-template	34	Intron_5	--	Yes	--	Yes	--	--	--	--	--	--	Yes
G4ST01226401281	chr1:226401282-226401315	+	GGGTGGGCTCCCAGGGTTGCTGTTGGCAACAGGG<br>((((.....))))..	GUK1	NM_001159390	chr1:226394552-226403278	non-template	34	Intron_4	--	Yes	--	Yes	--	--	--	--	--	--	Yes
G4ST01226401281	chr1:226401282-226401315	+	GGGTGGGCTCCCAGGGTTGCTGTTGGCAACAGGG<br>((((.....))))..	GUK1	NM_001159391	chr1:226399027-226403278	non-template	34	Intron_4	--	Yes	--	Yes	--	--	--	--	--	--	Yes
G4ST01226402934	chr1:226402935-226402963	-	GGGAGGGCTGGTATCCAGGAAGGGCTGGG<br>((((...))))....	GUK1	NM_000858	chr1:226394552-226403278	template	29	Intron_8.l	--	Yes	--	Yes	--	--	--	Yes	--	--	--
G4ST01226402934	chr1:226402935-226402963	-	GGGAGGGCTGGTATCCAGGAAGGGCTGGG<br>((((...))))....	GUK1	NM_001159390	chr1:226394552-226403278	template	29	Intron_7.l	--	Yes	--	Yes	--	--	--	Yes	--	--	--
G4ST01226402934	chr1:226402935-226402963	-	GGGAGGGCTGGTATCCAGGAAGGGCTGGG<br>((((...))))....	GUK1	NM_001159391	chr1:226399027-226403278	template	29	Intron_7.l	--	Yes	--	Yes	--	--	--	Yes	--	--	--
G4ST01226402934	chr1:226402935-226402963	-	GGGAGGGCTGGTATCCAGGAAGGGCTGGG<br>((((...))))....	GJC2	NM_020435	chr1:226404176-226414150	template	-1212	upstream	Yes	--	--	--	--	--	--	Yes	--	--	--
G4ST01226402939	chr1:226402940-226402970	-	GGGAGTTGGGAGGGCTGGTATCCAGGAAGGG<br>((((...))))....	GUK1	NM_000858	chr1:226394552-226403278	template	31	Intron_8.l	--	Yes	--	Yes	--	--	--	Yes	--	--	--
G4ST01226402939	chr1:226402940-226402970	-	GGGAGTTGGGAGGGCTGGTATCCAGGAAGGG<br>((((...))))....	GUK1	NM_001159390	chr1:226394552-226403278	template	31	Intron_7.l	--	Yes	--	Yes	--	--	--	Yes	--	--	--
G4ST01226402939	chr1:226402940-226402970	-	GGGAGTTGGGAGGGCTGGTATCCAGGAAGGG<br>((((...))))....	GUK1	NM_001159391	chr1:226399027-226403278	template	31	Intron_7.l	--	Yes	--	Yes	--	--	--	Yes	--	--	--
G4ST01226402939	chr1:226402940-226402970	-	GGGAGTTGGGAGGGCTGGTATCCAGGAAGGG<br>((((...))))....	GJC2	NM_020435	chr1:226404176-226414150	template	-1205	upstream	Yes	--	--	--	--	--	--	Yes	--	--	--
G4ST01226403529	chr1:226403530-226403565	+	GGGGCAGCCTGAGGCTGCAGGGGTGGGCCCTGAGGG<br>((((((...))))))..	GJC2	NM_020435	chr1:226404176-226414150	non-template	-610	upstream	Yes	--	--	--	--	--	--	--	Yes	Yes	--
G4ST01226405156	chr1:226405157-226405191	+	GGGTGGAGGGCAGGGACAGCCTTAGGAGGCTGGGG<br>.((((((....))))))	GJC2	NM_020435	chr1:226404176-226414150	non-template	35	Intron_1.u	--	Yes	--	Yes	--	--	--	Yes	Yes	Yes	--
G4ST01226413004	chr1:226413005-226413033	-	GGGCGGGGGCCGCGGCGCGGGGGCGGGGG<br>..((((...))))	GJC2	NM_020435	chr1:226404176-226414150	template	29	Exon_2.l	--	Yes	Yes	--	Yes	--	--	--	--	--	--
G4ST01226418266	chr1:226418267-226418303	-	GGGTGGGAATGACTAAGGAGACATCAGGGCTGAGGGG<br>.(((.((....)).)))..	IBA57	NM_001010867	chr1:226420052-226436581	template	-1748	upstream	Yes	--	--	--	--	--	--	--	--	Yes	--
G4ST01226418274	chr1:226418275-226418312	-	GGGGTCTGTGGGTGGGAATGACTAAGGAGACATCAGGG<br>.(((.((....)).)))..	IBA57	NM_001010867	chr1:226420052-226436581	template	-1739	upstream	Yes	--	--	--	--	--	--	--	--	Yes	--
G4ST01226419883	chr1:226419884-226419913	-	GGGGCGAGCGTGCCTGCTGGGTGGGTGGGG<br>...((((....))))	IBA57	NM_001010867	chr1:226420052-226436581	template	-138	upstream	Yes	--	--	--	--	--	--	Yes	Yes	Yes	--
G4ST01226419888	chr1:226419889-226419917	-	GGGCGGGGCGAGCGTGCCTGCTGGGTGGG<br>...((((....))))	IBA57	NM_001010867	chr1:226420052-226436581	template	-134	upstream	Yes	--	--	--	--	--	--	Yes	Yes	Yes	--
G4ST01226419892	chr1:226419893-226419922	-	GGGGCGGGCGGGGCGAGCGTGCCTGCTGGG<br>...((((....))))	IBA57	NM_001010867	chr1:226420052-226436581	template	-129	upstream	Yes	--	--	--	--	--	--	Yes	Yes	Yes	--
G4ST01226420479	chr1:226420480-226420511	+	GGGTGAGCGCGTGCTGGGAGGGCGCTCGGGGG<br>..(((....)))	IBA57	NM_001010867	chr1:226420052-226436581	non-template	32	Exon_1.f-Intron_1.f	--	Yes	Yes	Yes	Yes	--	--	Yes	Yes	Yes	Yes
G4ST01226426489	chr1:226426490-226426528	-	GGGGAACTGGGGAGGGGTCCGTGCGATCTCTCCCACGGG<br>....(((.((....)).)))	IBA57	NM_001010867	chr1:226420052-226436581	template	39	Intron_1.f	--	Yes	--	Yes	--	--	--	--	--	--	--
G4ST01226429969	chr1:226429970-226430001	-	GGGTGGGGCCAGGCAGGGCACCTACAAGGGGG<br>..(((...)))	IBA57	NM_001010867	chr1:226420052-226436581	template	32	Exon_3.l	--	Yes	Yes	--	--	--	Yes	--	--	--	--
G4ST01226462267	chr1:226462268-226462303	+	GGGGCTGGGCTGGGCCGGCCTGGCCGGCACAGGGGG<br>(((((...)))))......	OBSCN	NM_052843	chr1:226462484-226615574	non-template	-180	upstream	Yes	--	--	--	--	--	--	--	Yes	--	Yes
G4ST01226462267	chr1:226462268-226462303	+	GGGGCTGGGCTGGGCCGGCCTGGCCGGCACAGGGGG<br>(((((...)))))......	OBSCN	NM_001098623	chr1:226462484-226633198	non-template	-180	upstream	Yes	--	--	--	--	--	--	--	Yes	--	Yes
G4ST01226462273	chr1:226462274-226462308	+	GGGCTGGGCCGGCCTGGCCGGCACAGGGGGTGGGG<br>(((((...)))))......	OBSCN	NM_052843	chr1:226462484-226615574	non-template	-175	upstream	Yes	--	--	--	--	--	--	--	Yes	--	Yes
G4ST01226462273	chr1:226462274-226462308	+	GGGCTGGGCCGGCCTGGCCGGCACAGGGGGTGGGG<br>(((((...)))))......	OBSCN	NM_001098623	chr1:226462484-226633198	non-template	-175	upstream	Yes	--	--	--	--	--	--	--	Yes	--	Yes
G4ST01226462278	chr1:226462279-226462314	+	GGGCCGGCCTGGCCGGCACAGGGGGTGGGGTGGGGG<br>(((((...)))))......	OBSCN	NM_052843	chr1:226462484-226615574	non-template	-169	upstream	Yes	--	--	--	--	--	--	--	Yes	--	Yes
G4ST01226462278	chr1:226462279-226462314	+	GGGCCGGCCTGGCCGGCACAGGGGGTGGGGTGGGGG<br>(((((...)))))......	OBSCN	NM_001098623	chr1:226462484-226633198	non-template	-169	upstream	Yes	--	--	--	--	--	--	--	Yes	--	Yes
G4ST01226473463	chr1:226473464-226473500	+	GGGCAGGGGTTGGGCATCACAGCTGCCTGTGCATGGG<br>...(((((....)))))...	OBSCN	NM_052843	chr1:226462484-226615574	non-template	37	Intron_8	--	Yes	--	Yes	--	--	--	--	--	--	--
G4ST01226473463	chr1:226473464-226473500	+	GGGCAGGGGTTGGGCATCACAGCTGCCTGTGCATGGG<br>...(((((....)))))...	OBSCN	NM_001098623	chr1:226462484-226633198	non-template	37	Intron_8	--	Yes	--	Yes	--	--	--	--	--	--	--
G4ST01226524206	chr1:226524207-226524251	+	GGGAGGCCGGGGGGACCGACTGCTCTTGGCGCCCGGGGTCAGGGG<br>(((.(((........)))..))).	OBSCN	NM_052843	chr1:226462484-226615574	non-template	45	Intron_17	--	Yes	--	Yes	--	--	--	--	--	--	--
G4ST01226524206	chr1:226524207-226524251	+	GGGAGGCCGGGGGGACCGACTGCTCTTGGCGCCCGGGGTCAGGGG<br>(((.(((........)))..))).	OBSCN	NM_001098623	chr1:226462484-226633198	non-template	45	Intron_17	--	Yes	--	Yes	--	--	--	--	--	--	--
G4ST01226524214	chr1:226524215-226524259	+	GGGGGGACCGACTGCTCTTGGCGCCCGGGGTCAGGGGAGGCCGGG<br>(((.(((........)))..))).	OBSCN	NM_052843	chr1:226462484-226615574	non-template	45	Intron_17	--	Yes	--	Yes	--	--	--	--	--	--	--
G4ST01226524214	chr1:226524215-226524259	+	GGGGGGACCGACTGCTCTTGGCGCCCGGGGTCAGGGGAGGCCGGG<br>(((.(((........)))..))).	OBSCN	NM_001098623	chr1:226462484-226633198	non-template	45	Intron_17	--	Yes	--	Yes	--	--	--	--	--	--	--
G4ST01226527353	chr1:226527354-226527391	+	GGGCCCCAGGGCCCTGGAAGACCGAGGCAGGGAAGGGG<br>.(((((....)).)))..	OBSCN	NM_052843	chr1:226462484-226615574	non-template	38	Intron_17	--	Yes	--	Yes	--	--	--	--	--	--	--
G4ST01226527353	chr1:226527354-226527391	+	GGGCCCCAGGGCCCTGGAAGACCGAGGCAGGGAAGGGG<br>.(((((....)).)))..	OBSCN	NM_001098623	chr1:226462484-226633198	non-template	38	Intron_17	--	Yes	--	Yes	--	--	--	--	--	--	--
G4ST01226532709	chr1:226532710-226532736	+	GGGCCGGGACAGCGGGTAGGACCTGGG<br>.((...))	OBSCN	NM_052843	chr1:226462484-226615574	non-template	27	Intron_25	--	Yes	--	Yes	--	--	--	--	--	--	--
G4ST01226532709	chr1:226532710-226532736	+	GGGCCGGGACAGCGGGTAGGACCTGGG<br>.((...))	OBSCN	NM_001098623	chr1:226462484-226633198	non-template	27	Intron_25	--	Yes	--	Yes	--	--	--	--	--	--	--
G4ST01226570515	chr1:226570516-226570549	+	GGGGGCGGGGCTGCGGGCTGCAGCTGCAGAGGGG<br>((((....))))..	OBSCN	NM_052843	chr1:226462484-226615574	non-template	34	Intron_50	--	Yes	--	Yes	--	--	--	Yes	--	--	Yes
G4ST01226570515	chr1:226570516-226570549	+	GGGGGCGGGGCTGCGGGCTGCAGCTGCAGAGGGG<br>((((....))))..	OBSCN	NM_001098623	chr1:226462484-226633198	non-template	34	Intron_50	--	Yes	--	Yes	--	--	--	Yes	--	--	Yes
G4ST01226573217	chr1:226573218-226573260	-	GGGGGGCAAGCTGGGCTGAGGGGCCTCATCCACAGAGGCTGGG<br>(((((.......))))).	OBSCN	NM_052843	chr1:226462484-226615574	template	43	Exon_54	--	Yes	Yes	--	Yes	--	--	--	--	--	Yes
G4ST01226573217	chr1:226573218-226573260	-	GGGGGGCAAGCTGGGCTGAGGGGCCTCATCCACAGAGGCTGGG<br>(((((.......))))).	OBSCN	NM_001098623	chr1:226462484-226633198	template	43	Exon_54	--	Yes	Yes	--	Yes	--	--	--	--	--	Yes
G4ST01226588562	chr1:226588563-226588596	+	GGGGCCACAGAGTCTGTGGCAGGGCGAGGGTGGG<br>(((((((...))))))).	OBSCN	NM_052843	chr1:226462484-226615574	non-template	34	Intron_59	--	Yes	--	Yes	--	--	--	--	--	--	Yes

G4ST01226588562	chr1:226588563-226588596	+	GGGGCCACAGAGTCTGTGGCAGGGCGAGGGTGGG<br>(((((((...))))))).	OBSCN	NM_001098623	chr1:226462484-226633198	non-template	34	Intron_59	--	Yes	--	Yes	--	--	--	--	--	--	Yes
G4ST01226589617	chr1:226589618-226589648	+	GGGCCGTGAGTGGGGGTGGGGGGATGGGGGG<br>(((....)))	OBSCN	NM_052843	chr1:226462484-226615574	non-template	31	Exon_62-Intron_62	--	Yes	Yes	Yes	Yes	--	--	--	--	--	Yes
G4ST01226589617	chr1:226589618-226589648	+	GGGCCGTGAGTGGGGGTGGGGGGATGGGGGG<br>(((....)))	OBSCN	NM_001098623	chr1:226462484-226633198	non-template	31	Exon_62-Intron_62	--	Yes	Yes	Yes	Yes	--	--	--	--	--	Yes
G4ST01226590211	chr1:226590212-226590264	+	GGGCCAGGGAGGACGCTCTAGGAGTGGGGGACAGGACAGCGCTGCCCCTGGGG<br>....(((((...))))). ...((((.((....))..))))	OBSCN	NM_052843	chr1:226462484-226615574	non-template	53	Intron_63	--	Yes	--	Yes	--	--	--	--	--	--	--
G4ST01226590211	chr1:226590212-226590264	+	GGGCCAGGGAGGACGCTCTAGGAGTGGGGGACAGGACAGCGCTGCCCCTGGGG<br>....(((((...))))). ...((((.((....))..))))	OBSCN	NM_001098623	chr1:226462484-226633198	non-template	53	Intron_63	--	Yes	--	Yes	--	--	--	--	--	--	--
G4ST01226618808	chr1:226618809-226618847	+	GGGGCTGGCTGGGTGCAGGGCCTGGCCCAGGAGAAGGGG<br>((((...))))....	OBSCN	NM_001098623	chr1:226462484-226633198	non-template	39	Intron_80	--	Yes	--	Yes	--	--	--	--	--	--	--
G4ST01226619202	chr1:226619203-226619237	-	GGGACGGATGCCTGCCACAGGCAGGGTGGGTGGGG<br>.....((((((...))))))	OBSCN	NM_001098623	chr1:226462484-226633198	template	35	Intron_80	--	Yes	--	Yes	--	--	--	--	--	--	--
G4ST01226620542	chr1:226620543-226620581	+	GGGATGGCCCCAGGAGCTGGGGGGCCCACGGGAGGTGGG<br>.....(((((...)))))	OBSCN	NM_001098623	chr1:226462484-226633198	non-template	39	Intron_83	--	Yes	--	Yes	--	--	--	--	--	--	--
G4ST01226622055	chr1:226622056-226622092	-	GGGGATGATGCTCAGGGTTGAGGAGGGTGGAGCGGGG<br>..(((...)))	OBSCN	NM_001098623	chr1:226462484-226633198	template	37	Intron_86	--	Yes	--	Yes	--	--	--	--	Yes	Yes	--
G4ST01226626432	chr1:226626433-226626466	-	GGGAGGCTGACCCTGGGGAACTGCAGGGGCCGGG<br>(((.....))).	OBSCN	NM_001098623	chr1:226462484-226633198	template	34	Exon_94	--	Yes	Yes	--	Yes	--	--	--	--	--	--
G4ST01226626713	chr1:226626714-226626748	+	GGGGCAGGGGCCCATGTGGGCCAGGATAGCCTGGG<br>..(((....)))	OBSCN	NM_001098623	chr1:226462484-226633198	non-template	35	Exon_94	--	Yes	Yes	--	Yes	--	--	--	--	--	Yes
G4ST01226627855	chr1:226627856-226627894	-	GGGGTAAGGTAGGGTCTGGGGTCAGAGAGGACTATGGGG<br>..((.((....)).))	OBSCN	NM_001098623	chr1:226462484-226633198	template	39	Intron_94	--	Yes	--	Yes	--	--	--	--	--	--	Yes
G4ST01226627914	chr1:226627915-226627950	-	GGGCCTGAGGTCAGGAGGGTCTGGGGTCAGGTAGGG<br>((((....)))).	OBSCN	NM_001098623	chr1:226462484-226633198	template	36	Intron_94	--	Yes	--	Yes	--	--	--	--	--	--	Yes
G4ST01226627924	chr1:226627925-226627954	-	GGGAGGGCCTGAGGTCAGGAGGGTCTGGGG<br>((((....)))).	OBSCN	NM_001098623	chr1:226462484-226633198	template	30	Intron_94	--	Yes	--	Yes	--	--	--	--	--	--	Yes
G4ST01226627931	chr1:226627932-226627961	-	GGGGTCAGGGAGGGCCTGAGGTCAGGAGGG<br>((((....)))).	OBSCN	NM_001098623	chr1:226462484-226633198	template	30	Intron_94	--	Yes	--	Yes	--	--	--	--	--	--	Yes
G4ST01226660385	chr1:226660386-226660414	-	GGGTGGGAGGTTTGGGGGACTGCTCCGGG<br>(((....)))	TRIM11	NM_145214	chr1:226648000-226661140	non-template	29	Intron_1.f	--	Yes	--	Yes	--	--	--	--	Yes	Yes	Yes
G4ST01226741810	chr1:226741811-226741852	-	GGGCGCAGGCGGCCTCGGCGGGGCCCGCAGGGAGCGCCAGGG<br>(((.((.....)).)))	RNF187	NM_001010858	chr1:226741691-226750512	template	42	Exon_1.f	--	Yes	Yes	--	Yes	Yes	--	--	Yes	Yes	Yes
G4ST01226743202	chr1:226743203-226743238	+	GGGGTGATGTCCCTCAGTACTGGGCTGGGTTGGGGG<br>(((.((.....)).))).	RNF187	NM_001010858	chr1:226741691-226750512	non-template	36	Intron_1.f	--	Yes	--	Yes	--	--	--	--	--	Yes	--
G4ST01226747955	chr1:226747956-226747985	-	GGGCCATCTGGGTTGCTGCAGGGTCTGGGG<br>.((...))	RNF187	NM_001010858	chr1:226741691-226750512	template	30	Intron_3.l	--	Yes	--	Yes	--	--	--	--	--	--	--
G4ST01226749888	chr1:226749889-226749924	-	GGGCAAGCATCACAGGTGTGCAGGGCAGGGGCAGGG<br>...(((.((....))))).	RNF187	NM_001010858	chr1:226741691-226750512	template	36	Exon_4.l	--	Yes	Yes	--	--	--	Yes	--	Yes	Yes	Yes
G4ST01226879468	chr1:226879469-226879501	+	GGGCTGCTTCAAGCGGGATTGGGGTGGCGTGGG<br>..(((...)))	RHOU	NR_037962	chr1:226847017-226949039	non-template	33	Intron_1.f	--	Yes	--	Yes	--	--	--	--	--	--	--
G4ST01226882104	chr1:226882105-226882143	+	GGGGTAAGGGTGATTAGGTTTTAATGGGATGGTAAGGGG<br>..((((.....))))	RHOU	NR_037962	chr1:226847017-226949039	non-template	39	Intron_1.f	--	Yes	--	Yes	--	--	--	--	--	--	--
G4ST01226923199	chr1:226923200-226923237	-	GGGGCCAGGGGTGGGAGATGACTTCGTGGTCAGAGGGG<br>...(((((....)))))...	RHOU	NR_037962	chr1:226847017-226949039	template	38	Intron_1.f	--	Yes	--	Yes	--	--	--	--	--	--	Yes
G4ST01226938179	chr1:226938180-226938215	+	GGGAGCGCGGTGGACGCGGGGGACGCGGGCCTGGGG<br>..((((.....)))).	RHOU	NM_021205	chr1:226937447-226949039	non-template	36	Exon_1.f	--	Yes	Yes	--	Yes	--	--	--	Yes	--	Yes
G4ST01226938179	chr1:226938180-226938215	+	GGGAGCGCGGTGGACGCGGGGGACGCGGGCCTGGGG<br>..((((.....)))).	RHOU	NR_037962	chr1:226847017-226949039	non-template	36	Intron_1.f	--	Yes	--	Yes	--	--	--	--	Yes	--	Yes
G4ST01227473768	chr1:227473769-227473802	+	GGGTCGGGCCGCGGGGGGCGTCGGTGGCGCGGGG<br>..((((.....)))).	RAB4A	NM_004578	chr1:227473502-227507141	non-template	34	Intron_1.f	--	Yes	--	Yes	--	--	--	--	Yes	Yes	Yes
G4ST01227473773	chr1:227473774-227473807	+	GGGCCGCGGGGGGCGTCGGTGGCGCGGGGCCGGG<br>..((((.....)))).	RAB4A	NM_004578	chr1:227473502-227507141	non-template	34	Intron_1.f	--	Yes	--	Yes	--	--	--	--	Yes	Yes	Yes
G4ST01227473780	chr1:227473781-227473813	+	GGGGGGCGTCGGTGGCGCGGGGCCGGGCCTGGG<br>..((((.....)))).	RAB4A	NM_004578	chr1:227473502-227507141	non-template	33	Intron_1.f	--	Yes	--	Yes	--	--	--	--	Yes	Yes	Yes
G4ST01227494442	chr1:227494443-227494470	+	GGGGGGAGGGGGGAGGGATAGCAATGGG<br>((....))	RAB4A	NM_004578	chr1:227473502-227507141	non-template	28	Intron_3	--	Yes	--	Yes	--	--	--	--	--	--	Yes
G4ST01227528995	chr1:227528996-227529038	-	GGGCATCGTGTCCTTTGGCCACTGGGTGCTTTGGGCCCATGGG<br>....(((.((...)).))).	C1orf96	NM_145257	chr1:227523375-227545311	non-template	43	Intron_3.l	--	Yes	--	Yes	--	--	--	--	Yes	--	--
G4ST01227545007	chr1:227545008-227545044	+	GGGGGCGGGGCGGGGGCGGCCTCACCCGGCGGCCGGG<br>(((.(((......))).)))	C1orf96	NM_145257	chr1:227523375-227545311	template	37	Intron_1.f	--	Yes	--	Yes	--	--	--	Yes	Yes	Yes	Yes
G4ST01227545287	chr1:227545288-227545323	-	GGGCCTGGGCGCAGAGGATCGGCCGGGCGCGGCGGG<br>.((.((...)).)).	C1orf96	NM_145257	chr1:227523375-227545311	non-template	24	upstream-Exon_1.f	Yes	Yes	Yes	--	--	Yes	--	Yes	Yes	Yes	--
G4ST01227545599	chr1:227545600-227545632	-	GGGACAGCGTCTGCTGGGACCAGGGTGGACGGG<br>..(((....)))	C1orf96	NM_145257	chr1:227523375-227545311	non-template	-288	upstream	Yes	--	--	--	--	--	--	Yes	Yes	Yes	--
G4ST01227760768	chr1:227760769-227760800	-	GGGGGTCCGGGCGCCTCGCGGGGCGTCCTGGG<br>(((...)))	ABCB10	NM_012089	chr1:227718952-227761065	non-template	32	Exon_1.f	--	Yes	Yes	--	Yes	--	--	--	Yes	Yes	--
G4ST01227760777	chr1:227760778-227760805	-	GGGGCGGGGGTCCGGGCGCCTCGCGGGG<br>(((...)))	ABCB10	NM_012089	chr1:227718952-227761065	non-template	28	Exon_1.f	--	Yes	Yes	--	Yes	--	--	--	Yes	Yes	--
G4ST01227841057	chr1:227841058-227841091	-	GGGCATGAAGTCGTGGGTGCTGGGCCTAGAAGGG<br>.(((....)))	URB2	NM_014777	chr1:227828604-227862569	template	34	Intron_4	--	Yes	--	Yes	--	--	--	--	--	--	--
G4ST01227842968	chr1:227842969-227843005	+	GGGTGGGGCCACGGGCGTGCCTTTCGGTACGTGAGGG<br>((((((....))))))...	URB2	NM_014777	chr1:227828604-227862569	non-template	37	Intron_4	--	Yes	--	Yes	--	--	--	--	--	--	--
G4ST01227842972	chr1:227842973-227843014	+	GGGGCCACGGGCGTGCCTTTCGGTACGTGAGGGTTAAATGGG<br>((((((....))))))...	URB2	NM_014777	chr1:227828604-227862569	non-template	42	Intron_4	--	Yes	--	Yes	--	--	--	--	--	--	--
G4ST01228285707	chr1:228285708-228285739	+	GGGGAGCCGGGATGAGGGAGCAGGCAGCTGGG<br>(((.....)))	GALNT2	NM_004481	chr1:228269579-228484498	non-template	32	Intron_1.f	--	Yes	--	Yes	--	--	--	--	Yes	--	--
G4ST01228286967	chr1:228286968-228287008	+	GGGCTCGGGGGAGAATGGCTGAGAGCCAGGGTTGTGGAGGG<br>......(((((...)))))	GALNT2	NM_004481	chr1:228269579-228484498	non-template	41	Intron_1.f	--	Yes	--	Yes	--	--	--	Yes	Yes	--	--
G4ST01228322534	chr1:228322535-228322576	+	GGGGGTGGGGGTGAGTGGGCGGTGTTAAAGGCTACACCTGGG<br>.(((((........))))).	GALNT2	NM_004481	chr1:228269579-228484498	non-template	42	Intron_1.f	--	Yes	--	Yes	--	--	--	--	--	--	Yes
G4ST01228349491	chr1:228349492-228349524	+	GGGGATGAGGGGTGGGCAGTGCCAAGCTGGGGG<br>((((.....)))).	GALNT2	NM_004481	chr1:228269579-228484498	non-template	33	Intron_1.f	--	Yes	--	Yes	--	--	--	--	--	--	--
G4ST01228437835	chr1:228437836-228437868	+	GGGGTATTTGGGGATGGCCTTCCAGGGCAAGGG<br>..(((....)))	GALNT2	NM_004481	chr1:228269579-228484498	non-template	33	Intron_3	--	Yes	--	Yes	--	--	--	--	--	--	--
G4ST01228482186	chr1:228482187-228482219	+	GGGCGGGAGGAGGGGGCACACATGCCCCAGGGG<br>((((....))))....	GALNT2	NM_004481	chr1:228269579-228484498	non-template	33	Exon_16.l	--	Yes	Yes	--	--	--	Yes	--	--	--	--
G4ST01228525654	chr1:228525655-228525682	-	GGGAGGGATGCACAGGGCCTCTGGAGGG<br>((...)).	PGBD5	NM_024554	chr1:228524015-228580014	non-template	28	Exon_7.l	--	Yes	Yes	--	--	--	Yes	--	--	--	--
G4ST01228526478	chr1:228526479-228526504	-	GGGTCTGGGTGGGCTTCCCGGAGGGG<br>(((....)))	PGBD5	NM_024554	chr1:228524015-228580014	non-template	26	Intron_6.l	--	Yes	--	Yes	--	--	--	--	--	--	--
G4ST01228527417	chr1:228527418-228527457	+	GGGTGCCTCTGGGCACAGGGAATGTGAACCCAGCATTGGG<br>(((((.......)))))	PGBD5	NM_024554	chr1:228524015-228580014	template	40	Intron_6.l	--	Yes	--	Yes	--	--	--	--	--	--	--
G4ST01228535171	chr1:228535172-228535214	-	GGGTGAGTGCGGGTCCGCCATTGACACAGTGGGGCGCGCGGGG<br>.....(((((...)))))	PGBD5	NM_024554	chr1:228524015-228580014	non-template	43	Exon_5-Intron_5	--	Yes	Yes	Yes	Yes	--	--	Yes	Yes	Yes	--
G4ST01228556414	chr1:228556415-228556464	+	GGGGCATCCTCAGCAGATGGGGCAGGCTGCTGCTCCAGGGAATGGAGGGG<br>.((((.......)))) ((((....))))....	PGBD5	NM_024554	chr1:228524015-228580014	template	50	Intron_2	--	Yes	--	Yes	--	--	--	--	--	--	--

G4ST01228564402	chr1:228564403-228564435	+	GGGGAGTGGGTGGGGCTGGAGGCATCAGTGGGG<br>.((((.....))))..	PGBD5	NM_024554	chr1:228524015-228580014	template	33	Intron_1.f	--	Yes	--	Yes	--	--	--	Yes	Yes	--	--
G4ST01228564409	chr1:228564410-228564443	+	GGGTGGGGCTGGAGGCATCAGTGGGGCATGAGGG<br>.((((.....))))..	PGBD5	NM_024554	chr1:228524015-228580014	template	34	Intron_1.f	--	Yes	--	Yes	--	--	--	Yes	Yes	--	--
G4ST01228854545	chr1:228854546-228854584	-	GGGAGGGTGGTGGTGCTGAAGGTGCCGGGAGAGGAGGGG<br>....(((((.....)))))	COG2	NM_007357	chr1:228844825-228896354	template	39	Intron_1.f	--	Yes	--	Yes	--	--	--	--	--	--	Yes
G4ST01228854545	chr1:228854546-228854584	-	GGGAGGGTGGTGGTGCTGAAGGTGCCGGGAGAGGAGGGG<br>....(((((.....)))))	COG2	NM_001145036	chr1:228844825-228896354	template	39	Intron_1.f	--	Yes	--	Yes	--	--	--	--	--	--	Yes
G4ST01228854555	chr1:228854556-228854589	-	GGGATGGGAGGGTGGTGGTGCTGAAGGTGCCGGG<br>....(((((.....)))))	COG2	NM_007357	chr1:228844825-228896354	template	34	Intron_1.f	--	Yes	--	Yes	--	--	--	--	--	--	Yes
G4ST01228854555	chr1:228854556-228854589	-	GGGATGGGAGGGTGGTGGTGCTGAAGGTGCCGGG<br>....(((((.....)))))	COG2	NM_001145036	chr1:228844825-228896354	template	34	Intron_1.f	--	Yes	--	Yes	--	--	--	--	--	--	Yes
G4ST01228952586	chr1:228952587-228952612	-	GGGCTGGTACAGGGGCATGGGCTGGG<br>(((...)))	CAPN9	NM_006615	chr1:228949753-229004141	template	26	Intron_1.f	--	Yes	--	Yes	--	--	--	--	--	--	--
G4ST01228952586	chr1:228952587-228952612	-	GGGCTGGTACAGGGGCATGGGCTGGG<br>(((...)))	CAPN9	NM_016452	chr1:228949784-229004372	template	26	Intron_1.f	--	Yes	--	Yes	--	--	--	--	--	--	--
G4ST01228952591	chr1:228952592-228952620	-	GGGACAGAGGGCTGGTACAGGGGCATGGG<br>(((...)))	CAPN9	NM_006615	chr1:228949753-229004141	template	29	Intron_1.f	--	Yes	--	Yes	--	--	--	--	--	--	--
G4ST01228952591	chr1:228952592-228952620	-	GGGACAGAGGGCTGGTACAGGGGCATGGG<br>(((...)))	CAPN9	NM_016452	chr1:228949784-229004372	template	29	Intron_1.f	--	Yes	--	Yes	--	--	--	--	--	--	--
G4ST01228954937	chr1:228954938-228954974	+	GGGGCCAGGCCTGCAGAAGGCTGGGGGAGGGGAGGGG<br>.((((.(((.....)))))))	CAPN9	NM_006615	chr1:228949753-229004141	non-template	37	Intron_1.f	--	Yes	--	Yes	--	--	--	Yes	--	--	Yes
G4ST01228954937	chr1:228954938-228954974	+	GGGGCCAGGCCTGCAGAAGGCTGGGGGAGGGGAGGGG<br>.((((.(((.....)))))))	CAPN9	NM_016452	chr1:228949784-229004372	non-template	37	Intron_1.f	--	Yes	--	Yes	--	--	--	Yes	--	--	Yes
G4ST01228956201	chr1:228956202-228956223	-	GGGAGGGAGGGAGGAAGCCGGG<br>.((...))	CAPN9	NM_006615	chr1:228949753-229004141	template	22	Intron_1.f	--	Yes	--	Yes	--	--	--	Yes	Yes	Yes	Yes
G4ST01228956201	chr1:228956202-228956223	-	GGGAGGGAGGGAGGAAGCCGGG<br>.((...))	CAPN9	NM_016452	chr1:228949784-229004372	template	22	Intron_1.f	--	Yes	--	Yes	--	--	--	Yes	Yes	Yes	Yes
G4ST01228992905	chr1:228992906-228992939	-	GGGTACAGGGTTAACACTGTTAATGGGTAAAGGG<br>(((((...))))).	CAPN9	NM_006615	chr1:228949753-229004141	template	34	Intron_14	--	Yes	--	Yes	--	--	--	--	--	--	Yes
G4ST01228992905	chr1:228992906-228992939	-	GGGTACAGGGTTAACACTGTTAATGGGTAAAGGG<br>(((((...))))).	CAPN9	NM_016452	chr1:228949784-229004372	template	34	Intron_13	--	Yes	--	Yes	--	--	--	--	--	--	Yes
G4ST01228992912	chr1:228992913-228992948	-	GGGGGAAATGGGTACAGGGTTAACACTGTTAATGGG<br>(((((...))))).	CAPN9	NM_006615	chr1:228949753-229004141	template	36	Intron_14	--	Yes	--	Yes	--	--	--	--	--	--	Yes
G4ST01228992912	chr1:228992913-228992948	-	GGGGGAAATGGGTACAGGGTTAACACTGTTAATGGG<br>(((((...))))).	CAPN9	NM_016452	chr1:228949784-229004372	template	36	Intron_13	--	Yes	--	Yes	--	--	--	--	--	--	Yes
G4ST01229001027	chr1:229001028-229001066	+	GGGGCCATTGTAGCAATGACGGGGGTGGGAGACCATGGG<br>..(((((...)))))....	CAPN9	NM_006615	chr1:228949753-229004141	non-template	39	Intron_19.l	--	Yes	--	Yes	--	--	--	--	--	--	--
G4ST01229001027	chr1:229001028-229001066	+	GGGGCCATTGTAGCAATGACGGGGGTGGGAGACCATGGG<br>..(((((...)))))....	CAPN9	NM_016452	chr1:228949784-229004372	non-template	39	Intron_18.l	--	Yes	--	Yes	--	--	--	--	--	--	--
G4ST01229180577	chr1:229180578-229180611	+	GGGAGGCCGGCGGCGGGAACAGGGCTGCCCCGGG<br>..(((...)))	ARV1	NM_022786	chr1:229181446-229203102	non-template	-834	upstream	Yes	--	--	--	--	--	--	Yes	Yes	Yes	--
G4ST01229180577	chr1:229180578-229180611	+	GGGAGGCCGGCGGCGGGAACAGGGCTGCCCCGGG<br>..(((...)))	TTC13	NM_001122835	chr1:229108610-229181241	template	34	Intron_1.f	--	Yes	--	Yes	--	--	--	Yes	Yes	Yes	--
G4ST01229180577	chr1:229180578-229180611	+	GGGAGGCCGGCGGCGGGAACAGGGCTGCCCCGGG<br>..(((...)))	TTC13	NM_024525	chr1:229108610-229181241	template	34	Intron_1.f	--	Yes	--	Yes	--	--	--	Yes	Yes	Yes	--
G4ST01229234710	chr1:229234711-229234744	-	GGGGTGGGGTGGGGCGGTGTAAAGTGCTGCAGGG<br>((((((.....)))))).	FAM89A	NM_198552	chr1:229221327-229242618	non-template	34	Intron_1.u	--	Yes	--	Yes	--	--	--	--	--	--	Yes
G4ST01229236255	chr1:229236256-229236286	-	GGGGTGGGGGACAGTAAGGGCAGTGTGTGGG<br>((...)).	FAM89A	NM_198552	chr1:229221327-229242618	non-template	31	Intron_1.u	--	Yes	--	Yes	--	--	--	--	--	--	--
G4ST01229242545	chr1:229242546-229242574	-	GGGGCCCGGGCGGCGCCCGGGGCCGCGGG<br>(((...)))	FAM89A	NM_198552	chr1:229221327-229242618	non-template	29	Exon_1.f	--	Yes	Yes	--	Yes	--	--	Yes	Yes	Yes	--
G4ST01229242664	chr1:229242665-229242690	-	GGGCGTGGCGCGGGAGGGGAAGTGGG<br>((...)).	FAM89A	NM_198552	chr1:229221327-229242618	non-template	-46	upstream	Yes	--	--	--	--	--	--	Yes	Yes	Yes	Yes
G4ST01229242671	chr1:229242672-229242694	-	GGGGGGGCGTGGCGCGGGAGGGG<br>((...)).	FAM89A	NM_198552	chr1:229221327-229242618	non-template	-53	upstream	Yes	--	--	--	--	--	--	Yes	Yes	Yes	Yes
G4ST01229242676	chr1:229242677-229242700	-	GGGGCCGGGGGGGCGTGGCGCGGG<br>((...)).	FAM89A	NM_198552	chr1:229221327-229242618	non-template	-58	upstream	Yes	--	--	--	--	--	--	Yes	Yes	Yes	Yes
G4ST01229242691	chr1:229242692-229242721	-	GGGGCCTCGAGGAGAGGGGGCGGGGCCGGG<br>.((((.....))))	FAM89A	NM_198552	chr1:229221327-229242618	non-template	-73	upstream	Yes	--	--	--	--	--	--	Yes	Yes	Yes	Yes
G4ST01229393018	chr1:229393019-229393049	-	GGGATTGATGGTAAGGGCACAGGGGAGAGGG<br>.(((....)))	TRIM67	NM_001004342	chr1:229365297-229423937	template	31	Intron_1.f	--	Yes	--	Yes	--	--	--	--	--	--	--
G4ST01229393090	chr1:229393091-229393123	+	GGGCGGGGGACTGGATCCTGGGAGCGGACTGGG<br>(((.....))). ((....))	TRIM67	NM_001004342	chr1:229365297-229423937	non-template	33	Intron_1.f	--	Yes	--	Yes	--	--	--	--	--	--	--
G4ST01229393094	chr1:229393095-229393130	+	GGGGGACTGGATCCTGGGAGCGGACTGGGAGCGGGG<br>(((.....))). ((....))	TRIM67	NM_001004342	chr1:229365297-229423937	non-template	36	Intron_1.f	--	Yes	--	Yes	--	--	--	--	--	--	--
G4ST01229394335	chr1:229394336-229394373	+	GGGGCAGCAGGGCAGGGAGCTGATGCAGCTTTCCTGGG<br>(((((...)))))....	TRIM67	NM_001004342	chr1:229365297-229423937	non-template	38	Intron_1.f	--	Yes	--	Yes	--	--	--	--	--	--	--
G4ST01229406944	chr1:229406945-229406976	-	GGGCTGGGTTGGGGTGGAGTCCTGCTCCAGGG<br>..((((.....)))).	TRIM67	NM_001004342	chr1:229365297-229423937	template	32	Intron_6	--	Yes	--	Yes	--	--	--	--	--	--	--
G4ST01229409258	chr1:229409259-229409295	+	GGGGGGTGGTGCTGGGCACGGCTGGGTTCTCCAAGGG<br>((....))	TRIM67	NM_001004342	chr1:229365297-229423937	non-template	37	Intron_7	--	Yes	--	Yes	--	--	--	--	Yes	--	Yes
G4ST01229411960	chr1:229411961-229411995	+	GGGTTGCTGCAGGGTGTTAATGGGCACCACTGGGG<br>.((...))	TRIM67	NM_001004342	chr1:229365297-229423937	non-template	35	Intron_8	--	Yes	--	Yes	--	--	--	--	--	--	--
G4ST01229464489	chr1:229464490-229464519	-	GGGTGAATGGGTGTCATGTGGCTGGGAGGG<br>.(((....))).	GNPAT	NM_014236	chr1:229443542-229480342	template	30	Intron_3	--	Yes	--	Yes	--	--	--	--	--	--	--
G4ST01229828766	chr1:229828767-229828799	-	GGGGCTCATTTATACAAAGTGGGGGCGGGGGGG<br>...(((((.....))))).	DISC1	NM_001164545	chr1:229829184-230021613	template	-384	upstream	Yes	--	--	--	--	--	--	--	Yes	Yes	Yes
G4ST01229828766	chr1:229828767-229828799	-	GGGGCTCATTTATACAAAGTGGGGGCGGGGGGG<br>...(((((.....))))).	TSNAX-DISC1	NR_028396	chr1:229731022-230021613	template	33	Intron_5	--	Yes	--	Yes	--	--	--	--	Yes	Yes	Yes
G4ST01229828766	chr1:229828767-229828799	-	GGGGCTCATTTATACAAAGTGGGGGCGGGGGGG<br>...(((((.....))))).	TSNAX-DISC1	NR_028398	chr1:229731022-230021613	template	33	Intron_3	--	Yes	--	Yes	--	--	--	--	Yes	Yes	Yes
G4ST01229828766	chr1:229828767-229828799	-	GGGGCTCATTTATACAAAGTGGGGGCGGGGGGG<br>...(((((.....))))).	TSNAX-DISC1	NR_028397	chr1:229731022-230021613	template	33	Intron_5	--	Yes	--	Yes	--	--	--	--	Yes	Yes	Yes
G4ST01229828766	chr1:229828767-229828799	-	GGGGCTCATTTATACAAAGTGGGGGCGGGGGGG<br>...(((((.....))))).	TSNAX-DISC1	NR_028399	chr1:229731022-229904930	template	33	Intron_7	--	Yes	--	Yes	--	--	--	--	Yes	Yes	Yes
G4ST01229828766	chr1:229828767-229828799	-	GGGGCTCATTTATACAAAGTGGGGGCGGGGGGG<br>...(((((.....))))).	DISC1	NM_001164538	chr1:229829184-230212023	template	-384	upstream	Yes	--	--	--	--	--	--	--	Yes	Yes	Yes
G4ST01229828766	chr1:229828767-229828799	-	GGGGCTCATTTATACAAAGTGGGGGCGGGGGGG<br>...(((((.....))))).	DISC1	NM_001164548	chr1:229829184-230021613	template	-384	upstream	Yes	--	--	--	--	--	--	--	Yes	Yes	Yes
G4ST01229828766	chr1:229828767-229828799	-	GGGGCTCATTTATACAAAGTGGGGGCGGGGGGG<br>...(((((.....))))).	TSNAX-DISC1	NR_028394	chr1:229731022-230021613	template	33	Intron_6	--	Yes	--	Yes	--	--	--	--	Yes	Yes	Yes
G4ST01229828766	chr1:229828767-229828799	-	GGGGCTCATTTATACAAAGTGGGGGCGGGGGGG<br>...(((((.....))))).	TSNAX-DISC1	NR_028395	chr1:229731022-230021613	template	33	Intron_6	--	Yes	--	Yes	--	--	--	--	Yes	Yes	Yes
G4ST01229828766	chr1:229828767-229828799	-	GGGGCTCATTTATACAAAGTGGGGGCGGGGGGG<br>...(((((.....))))).	DISC1	NM_018662	chr1:229829184-230243641	template	-384	upstream	Yes	--	--	--	--	--	--	--	Yes	Yes	Yes
G4ST01229828766	chr1:229828767-229828799	-	GGGGCTCATTTATACAAAGTGGGGGCGGGGGGG<br>...(((((.....))))).	TSNAX-DISC1	NR_028400	chr1:229731022-229952988	template	33	Intron_6	--	Yes	--	Yes	--	--	--	--	Yes	Yes	Yes
G4ST01229828766	chr1:229828767-229828799	-	GGGGCTCATTTATACAAAGTGGGGGCGGGGGGG<br>...(((((.....))))).	TSNAX-DISC1	NR_028393	chr1:229731022-230243641	template	33	Intron_5	--	Yes	--	Yes	--	--	--	--	Yes	Yes	Yes

G4ST01229828766	chr1:229828767-229828799	-	GGGGCTCATTTATACAAAGTGGGGGCGGGGGGG<br>...(((((.....))))).	DISC1	NM_001164541	chr1:229829184-230212023	template	-384	upstream	Yes	--	--	--	--	--	--	--	Yes	Yes	Yes
G4ST01229828766	chr1:229828767-229828799	-	GGGGCTCATTTATACAAAGTGGGGGCGGGGGGG<br>...(((((.....))))).	DISC1	NM_001164539	chr1:229829184-230057748	template	-384	upstream	Yes	--	--	--	--	--	--	--	Yes	Yes	Yes
G4ST01229828766	chr1:229828767-229828799	-	GGGGCTCATTTATACAAAGTGGGGGCGGGGGGG<br>...(((((.....))))).	DISC1	NM_001164550	chr1:229829184-229926443	template	-384	upstream	Yes	--	--	--	--	--	--	--	Yes	Yes	Yes
G4ST01229828766	chr1:229828767-229828799	-	GGGGCTCATTTATACAAAGTGGGGGCGGGGGGG<br>...(((((.....))))).	DISC1	NM_001164547	chr1:229829184-230212023	template	-384	upstream	Yes	--	--	--	--	--	--	--	Yes	Yes	Yes
G4ST01229828766	chr1:229828767-229828799	-	GGGGCTCATTTATACAAAGTGGGGGCGGGGGGG<br>...(((((.....))))).	DISC1	NM_001164546	chr1:229829184-230161872	template	-384	upstream	Yes	--	--	--	--	--	--	--	Yes	Yes	Yes
G4ST01229828766	chr1:229828767-229828799	-	GGGGCTCATTTATACAAAGTGGGGGCGGGGGGG<br>...(((((.....))))).	DISC1	NM_001164544	chr1:229829184-230021613	template	-384	upstream	Yes	--	--	--	--	--	--	--	Yes	Yes	Yes
G4ST01229828766	chr1:229828767-229828799	-	GGGGCTCATTTATACAAAGTGGGGGCGGGGGGG<br>...(((((.....))))).	DISC1	NM_001164554	chr1:229829184-229904930	template	-384	upstream	Yes	--	--	--	--	--	--	--	Yes	Yes	Yes
G4ST01229828766	chr1:229828767-229828799	-	GGGGCTCATTTATACAAAGTGGGGGCGGGGGGG<br>...(((((.....))))).	DISC1	NM_001164537	chr1:229829184-230243641	template	-384	upstream	Yes	--	--	--	--	--	--	--	Yes	Yes	Yes
G4ST01229828766	chr1:229828767-229828799	-	GGGGCTCATTTATACAAAGTGGGGGCGGGGGGG<br>...(((((.....))))).	DISC1	NM_001164540	chr1:229829184-230243641	template	-384	upstream	Yes	--	--	--	--	--	--	--	Yes	Yes	Yes
G4ST01229828766	chr1:229828767-229828799	-	GGGGCTCATTTATACAAAGTGGGGGCGGGGGGG<br>...(((((.....))))).	DISC1	NM_001164551	chr1:229829184-229952988	template	-384	upstream	Yes	--	--	--	--	--	--	--	Yes	Yes	Yes
G4ST01229828766	chr1:229828767-229828799	-	GGGGCTCATTTATACAAAGTGGGGGCGGGGGGG<br>...(((((.....))))).	DISC1	NM_001164556	chr1:229829184-230021613	template	-384	upstream	Yes	--	--	--	--	--	--	--	Yes	Yes	Yes
G4ST01229828766	chr1:229828767-229828799	-	GGGGCTCATTTATACAAAGTGGGGGCGGGGGGG<br>...(((((.....))))).	DISC1	NM_001164555	chr1:229829184-229952988	template	-384	upstream	Yes	--	--	--	--	--	--	--	Yes	Yes	Yes
G4ST01229828766	chr1:229828767-229828799	-	GGGGCTCATTTATACAAAGTGGGGGCGGGGGGG<br>...(((((.....))))).	DISC1	NM_001164553	chr1:229829184-229926443	template	-384	upstream	Yes	--	--	--	--	--	--	--	Yes	Yes	Yes
G4ST01229828766	chr1:229828767-229828799	-	GGGGCTCATTTATACAAAGTGGGGGCGGGGGGG<br>...(((((.....))))).	DISC1	NM_001164552	chr1:229829184-229926443	template	-384	upstream	Yes	--	--	--	--	--	--	--	Yes	Yes	Yes
G4ST01229828766	chr1:229828767-229828799	-	GGGGCTCATTTATACAAAGTGGGGGCGGGGGGG<br>...(((((.....))))).	DISC1	NM_001164542	chr1:229829184-230161872	template	-384	upstream	Yes	--	--	--	--	--	--	--	Yes	Yes	Yes
G4ST01229828766	chr1:229828767-229828799	-	GGGGCTCATTTATACAAAGTGGGGGCGGGGGGG<br>...(((((.....))))).	DISC1	NM_001164549	chr1:229829184-229974031	template	-384	upstream	Yes	--	--	--	--	--	--	--	Yes	Yes	Yes
G4ST01229828766	chr1:229828767-229828799	-	GGGGCTCATTTATACAAAGTGGGGGCGGGGGGG<br>...(((((.....))))).	DISC1	NM_001012957	chr1:229829184-230243641	template	-384	upstream	Yes	--	--	--	--	--	--	--	Yes	Yes	Yes
G4ST01229828766	chr1:229828767-229828799	-	GGGGCTCATTTATACAAAGTGGGGGCGGGGGGG<br>...(((((.....))))).	DISC1	NM_001012959	chr1:229829184-230069075	template	-384	upstream	Yes	--	--	--	--	--	--	--	Yes	Yes	Yes
G4ST01229828766	chr1:229828767-229828799	-	GGGGCTCATTTATACAAAGTGGGGGCGGGGGGG<br>...(((((.....))))).	DISC1	NM_001012958	chr1:229829184-229926443	template	-384	upstream	Yes	--	--	--	--	--	--	--	Yes	Yes	Yes
G4ST01229828770	chr1:229828771-229828808	-	GGGATGTGAGGGGCTCATTTATACAAAGTGGGGGCGGG<br>...(((((.....))))).	DISC1	NM_001164545	chr1:229829184-230021613	template	-375	upstream	Yes	--	--	--	--	--	--	--	Yes	Yes	Yes
G4ST01229828770	chr1:229828771-229828808	-	GGGATGTGAGGGGCTCATTTATACAAAGTGGGGGCGGG<br>...(((((.....))))).	TSNAX-DISC1	NR_028396	chr1:229731022-230021613	template	38	Intron_5	--	Yes	--	Yes	--	--	--	--	Yes	Yes	Yes
G4ST01229828770	chr1:229828771-229828808	-	GGGATGTGAGGGGCTCATTTATACAAAGTGGGGGCGGG<br>...(((((.....))))).	TSNAX-DISC1	NR_028398	chr1:229731022-230021613	template	38	Intron_3	--	Yes	--	Yes	--	--	--	--	Yes	Yes	Yes
G4ST01229828770	chr1:229828771-229828808	-	GGGATGTGAGGGGCTCATTTATACAAAGTGGGGGCGGG<br>...(((((.....))))).	TSNAX-DISC1	NR_028397	chr1:229731022-230021613	template	38	Intron_5	--	Yes	--	Yes	--	--	--	--	Yes	Yes	Yes
G4ST01229828770	chr1:229828771-229828808	-	GGGATGTGAGGGGCTCATTTATACAAAGTGGGGGCGGG<br>...(((((.....))))).	TSNAX-DISC1	NR_028399	chr1:229731022-229904930	template	38	Intron_7	--	Yes	--	Yes	--	--	--	--	Yes	Yes	Yes
G4ST01229828770	chr1:229828771-229828808	-	GGGATGTGAGGGGCTCATTTATACAAAGTGGGGGCGGG<br>...(((((.....))))).	DISC1	NM_001164538	chr1:229829184-230212023	template	-375	upstream	Yes	--	--	--	--	--	--	--	Yes	Yes	Yes
G4ST01229828770	chr1:229828771-229828808	-	GGGATGTGAGGGGCTCATTTATACAAAGTGGGGGCGGG<br>...(((((.....))))).	DISC1	NM_001164548	chr1:229829184-230021613	template	-375	upstream	Yes	--	--	--	--	--	--	--	Yes	Yes	Yes
G4ST01229828770	chr1:229828771-229828808	-	GGGATGTGAGGGGCTCATTTATACAAAGTGGGGGCGGG<br>...(((((.....))))).	TSNAX-DISC1	NR_028394	chr1:229731022-230021613	template	38	Intron_6	--	Yes	--	Yes	--	--	--	--	Yes	Yes	Yes
G4ST01229828770	chr1:229828771-229828808	-	GGGATGTGAGGGGCTCATTTATACAAAGTGGGGGCGGG<br>...(((((.....))))).	TSNAX-DISC1	NR_028395	chr1:229731022-230021613	template	38	Intron_6	--	Yes	--	Yes	--	--	--	--	Yes	Yes	Yes
G4ST01229828770	chr1:229828771-229828808	-	GGGATGTGAGGGGCTCATTTATACAAAGTGGGGGCGGG<br>...(((((.....))))).	DISC1	NM_018662	chr1:229829184-230243641	template	-375	upstream	Yes	--	--	--	--	--	--	--	Yes	Yes	Yes
G4ST01229828770	chr1:229828771-229828808	-	GGGATGTGAGGGGCTCATTTATACAAAGTGGGGGCGGG<br>...(((((.....))))).	TSNAX-DISC1	NR_028400	chr1:229731022-229952988	template	38	Intron_6	--	Yes	--	Yes	--	--	--	--	Yes	Yes	Yes
G4ST01229828770	chr1:229828771-229828808	-	GGGATGTGAGGGGCTCATTTATACAAAGTGGGGGCGGG<br>...(((((.....))))).	TSNAX-DISC1	NR_028393	chr1:229731022-230243641	template	38	Intron_5	--	Yes	--	Yes	--	--	--	--	Yes	Yes	Yes
G4ST01229828770	chr1:229828771-229828808	-	GGGATGTGAGGGGCTCATTTATACAAAGTGGGGGCGGG<br>...(((((.....))))).	DISC1	NM_001164541	chr1:229829184-230212023	template	-375	upstream	Yes	--	--	--	--	--	--	--	Yes	Yes	Yes
G4ST01229828770	chr1:229828771-229828808	-	GGGATGTGAGGGGCTCATTTATACAAAGTGGGGGCGGG<br>...(((((.....))))).	DISC1	NM_001164539	chr1:229829184-230057748	template	-375	upstream	Yes	--	--	--	--	--	--	--	Yes	Yes	Yes
G4ST01229828770	chr1:229828771-229828808	-	GGGATGTGAGGGGCTCATTTATACAAAGTGGGGGCGGG<br>...(((((.....))))).	DISC1	NM_001164550	chr1:229829184-229926443	template	-375	upstream	Yes	--	--	--	--	--	--	--	Yes	Yes	Yes
G4ST01229828770	chr1:229828771-229828808	-	GGGATGTGAGGGGCTCATTTATACAAAGTGGGGGCGGG<br>...(((((.....))))).	DISC1	NM_001164547	chr1:229829184-230212023	template	-375	upstream	Yes	--	--	--	--	--	--	--	Yes	Yes	Yes
G4ST01229828770	chr1:229828771-229828808	-	GGGATGTGAGGGGCTCATTTATACAAAGTGGGGGCGGG<br>...(((((.....))))).	DISC1	NM_001164546	chr1:229829184-230161872	template	-375	upstream	Yes	--	--	--	--	--	--	--	Yes	Yes	Yes
G4ST01229828770	chr1:229828771-229828808	-	GGGATGTGAGGGGCTCATTTATACAAAGTGGGGGCGGG<br>...(((((.....))))).	DISC1	NM_001164544	chr1:229829184-230021613	template	-375	upstream	Yes	--	--	--	--	--	--	--	Yes	Yes	Yes
G4ST01229828770	chr1:229828771-229828808	-	GGGATGTGAGGGGCTCATTTATACAAAGTGGGGGCGGG<br>...(((((.....))))).	DISC1	NM_001164554	chr1:229829184-229904930	template	-375	upstream	Yes	--	--	--	--	--	--	--	Yes	Yes	Yes
G4ST01229828770	chr1:229828771-229828808	-	GGGATGTGAGGGGCTCATTTATACAAAGTGGGGGCGGG<br>...(((((.....))))).	DISC1	NM_001164537	chr1:229829184-230243641	template	-375	upstream	Yes	--	--	--	--	--	--	--	Yes	Yes	Yes
G4ST01229828770	chr1:229828771-229828808	-	GGGATGTGAGGGGCTCATTTATACAAAGTGGGGGCGGG<br>...(((((.....))))).	DISC1	NM_001164540	chr1:229829184-230243641	template	-375	upstream	Yes	--	--	--	--	--	--	--	Yes	Yes	Yes
G4ST01229828770	chr1:229828771-229828808	-	GGGATGTGAGGGGCTCATTTATACAAAGTGGGGGCGGG<br>...(((((.....))))).	DISC1	NM_001164551	chr1:229829184-229952988	template	-375	upstream	Yes	--	--	--	--	--	--	--	Yes	Yes	Yes
G4ST01229828770	chr1:229828771-229828808	-	GGGATGTGAGGGGCTCATTTATACAAAGTGGGGGCGGG<br>...(((((.....))))).	DISC1	NM_001164556	chr1:229829184-230021613	template	-375	upstream	Yes	--	--	--	--	--	--	--	Yes	Yes	Yes
G4ST01229828770	chr1:229828771-229828808	-	GGGATGTGAGGGGCTCATTTATACAAAGTGGGGGCGGG<br>...(((((.....))))).	DISC1	NM_001164555	chr1:229829184-229952988	template	-375	upstream	Yes	--	--	--	--	--	--	--	Yes	Yes	Yes
G4ST01229828770	chr1:229828771-229828808	-	GGGATGTGAGGGGCTCATTTATACAAAGTGGGGGCGGG<br>...(((((.....))))).	DISC1	NM_001164553	chr1:229829184-229926443	template	-375	upstream	Yes	--	--	--	--	--	--	--	Yes	Yes	Yes
G4ST01229828770	chr1:229828771-229828808	-	GGGATGTGAGGGGCTCATTTATACAAAGTGGGGGCGGG<br>...(((((.....))))).	DISC1	NM_001164552	chr1:229829184-229926443	template	-375	upstream	Yes	--	--	--	--	--	--	--	Yes	Yes	Yes
G4ST01229828770	chr1:229828771-229828808	-	GGGATGTGAGGGGCTCATTTATACAAAGTGGGGGCGGG<br>...(((((.....))))).	DISC1	NM_001164542	chr1:229829184-230161872	template	-375	upstream	Yes	--	--	--	--	--	--	--	Yes	Yes	Yes
G4ST01229828770	chr1:229828771-229828808	-	GGGATGTGAGGGGCTCATTTATACAAAGTGGGGGCGGG<br>...(((((.....))))).	DISC1	NM_001164549	chr1:229829184-229974031	template	-375	upstream	Yes	--	--	--	--	--	--	--	Yes	Yes	Yes
G4ST01229828770	chr1:229828771-229828808	-	GGGATGTGAGGGGCTCATTTATACAAAGTGGGGGCGGG<br>...(((((.....))))).	DISC1	NM_001012957	chr1:229829184-230243641	template	-375	upstream	Yes	--	--	--	--	--	--	--	Yes	Yes	Yes
G4ST01229828770	chr1:229828771-229828808	-	GGGATGTGAGGGGCTCATTTATACAAAGTGGGGGCGGG<br>...(((((.....))))).	DISC1	NM_001012959	chr1:229829184-230069075	template	-375	upstream	Yes	--	--	--	--	--	--	--	Yes	Yes	Yes
G4ST01229828770	chr1:229828771-229828808	-	GGGATGTGAGGGGCTCATTTATACAAAGTGGGGGCGGG<br>...(((((.....))))).	DISC1	NM_001012958	chr1:229829184-229926443	template	-375	upstream	Yes	--	--	--	--	--	--	--	Yes	Yes	Yes

G4ST01229829781	chr1:229829782-229829812	+	GGGGACCTGGAGGGAGGAGGCTGGGAGCGGG<br>((....))	DISC1	NM_001164545	chr1:229829184-230021613	non-template	31	Intron_1.f	--	Yes	--	Yes	--	--	--	Yes	Yes	Yes	--
G4ST01229829781	chr1:229829782-229829812	+	GGGGACCTGGAGGGAGGAGGCTGGGAGCGGG<br>((....))	TSNAX-DISC1	NR_028396	chr1:229731022-230021613	non-template	31	Intron_5	--	Yes	--	Yes	--	--	--	Yes	Yes	Yes	--
G4ST01229829781	chr1:229829782-229829812	+	GGGGACCTGGAGGGAGGAGGCTGGGAGCGGG<br>((....))	TSNAX-DISC1	NR_028398	chr1:229731022-230021613	non-template	31	Intron_3	--	Yes	--	Yes	--	--	--	Yes	Yes	Yes	--
G4ST01229829781	chr1:229829782-229829812	+	GGGGACCTGGAGGGAGGAGGCTGGGAGCGGG<br>((....))	TSNAX-DISC1	NR_028397	chr1:229731022-230021613	non-template	31	Intron_5	--	Yes	--	Yes	--	--	--	Yes	Yes	Yes	--
G4ST01229829781	chr1:229829782-229829812	+	GGGGACCTGGAGGGAGGAGGCTGGGAGCGGG<br>((....))	TSNAX-DISC1	NR_028399	chr1:229731022-229904930	non-template	31	Intron_7	--	Yes	--	Yes	--	--	--	Yes	Yes	Yes	--
G4ST01229829781	chr1:229829782-229829812	+	GGGGACCTGGAGGGAGGAGGCTGGGAGCGGG<br>((....))	DISC1	NM_001164538	chr1:229829184-230212023	non-template	31	Intron_1.f	--	Yes	--	Yes	--	--	--	Yes	Yes	Yes	--
G4ST01229829781	chr1:229829782-229829812	+	GGGGACCTGGAGGGAGGAGGCTGGGAGCGGG<br>((....))	DISC1	NM_001164548	chr1:229829184-230021613	non-template	31	Intron_1.f	--	Yes	--	Yes	--	--	--	Yes	Yes	Yes	--
G4ST01229829781	chr1:229829782-229829812	+	GGGGACCTGGAGGGAGGAGGCTGGGAGCGGG<br>((....))	TSNAX-DISC1	NR_028394	chr1:229731022-230021613	non-template	31	Intron_6	--	Yes	--	Yes	--	--	--	Yes	Yes	Yes	--
G4ST01229829781	chr1:229829782-229829812	+	GGGGACCTGGAGGGAGGAGGCTGGGAGCGGG<br>((....))	TSNAX-DISC1	NR_028395	chr1:229731022-230021613	non-template	31	Intron_6	--	Yes	--	Yes	--	--	--	Yes	Yes	Yes	--
G4ST01229829781	chr1:229829782-229829812	+	GGGGACCTGGAGGGAGGAGGCTGGGAGCGGG<br>((....))	DISC1	NM_018662	chr1:229829184-230243641	non-template	31	Intron_1.f	--	Yes	--	Yes	--	--	--	Yes	Yes	Yes	--
G4ST01229829781	chr1:229829782-229829812	+	GGGGACCTGGAGGGAGGAGGCTGGGAGCGGG<br>((....))	TSNAX-DISC1	NR_028400	chr1:229731022-229952988	non-template	31	Intron_6	--	Yes	--	Yes	--	--	--	Yes	Yes	Yes	--
G4ST01229829781	chr1:229829782-229829812	+	GGGGACCTGGAGGGAGGAGGCTGGGAGCGGG<br>((....))	TSNAX-DISC1	NR_028393	chr1:229731022-230243641	non-template	31	Intron_5	--	Yes	--	Yes	--	--	--	Yes	Yes	Yes	--
G4ST01229829781	chr1:229829782-229829812	+	GGGGACCTGGAGGGAGGAGGCTGGGAGCGGG<br>((....))	DISC1	NM_001164541	chr1:229829184-230212023	non-template	31	Intron_1.f	--	Yes	--	Yes	--	--	--	Yes	Yes	Yes	--
G4ST01229829781	chr1:229829782-229829812	+	GGGGACCTGGAGGGAGGAGGCTGGGAGCGGG<br>((....))	DISC1	NM_001164539	chr1:229829184-230057748	non-template	31	Intron_1.f	--	Yes	--	Yes	--	--	--	Yes	Yes	Yes	--
G4ST01229829781	chr1:229829782-229829812	+	GGGGACCTGGAGGGAGGAGGCTGGGAGCGGG<br>((....))	DISC1	NM_001164550	chr1:229829184-229926443	non-template	31	Intron_1.f	--	Yes	--	Yes	--	--	--	Yes	Yes	Yes	--
G4ST01229829781	chr1:229829782-229829812	+	GGGGACCTGGAGGGAGGAGGCTGGGAGCGGG<br>((....))	DISC1	NM_001164547	chr1:229829184-230212023	non-template	31	Intron_1.f	--	Yes	--	Yes	--	--	--	Yes	Yes	Yes	--
G4ST01229829781	chr1:229829782-229829812	+	GGGGACCTGGAGGGAGGAGGCTGGGAGCGGG<br>((....))	DISC1	NM_001164546	chr1:229829184-230161872	non-template	31	Intron_1.f	--	Yes	--	Yes	--	--	--	Yes	Yes	Yes	--
G4ST01229829781	chr1:229829782-229829812	+	GGGGACCTGGAGGGAGGAGGCTGGGAGCGGG<br>((....))	DISC1	NM_001164544	chr1:229829184-230021613	non-template	31	Intron_1.f	--	Yes	--	Yes	--	--	--	Yes	Yes	Yes	--
G4ST01229829781	chr1:229829782-229829812	+	GGGGACCTGGAGGGAGGAGGCTGGGAGCGGG<br>((....))	DISC1	NM_001164554	chr1:229829184-229904930	non-template	31	Intron_1.f	--	Yes	--	Yes	--	--	--	Yes	Yes	Yes	--
G4ST01229829781	chr1:229829782-229829812	+	GGGGACCTGGAGGGAGGAGGCTGGGAGCGGG<br>((....))	DISC1	NM_001164537	chr1:229829184-230243641	non-template	31	Intron_1.f	--	Yes	--	Yes	--	--	--	Yes	Yes	Yes	--
G4ST01229829781	chr1:229829782-229829812	+	GGGGACCTGGAGGGAGGAGGCTGGGAGCGGG<br>((....))	DISC1	NM_001164540	chr1:229829184-230243641	non-template	31	Intron_1.f	--	Yes	--	Yes	--	--	--	Yes	Yes	Yes	--
G4ST01229829781	chr1:229829782-229829812	+	GGGGACCTGGAGGGAGGAGGCTGGGAGCGGG<br>((....))	DISC1	NM_001164551	chr1:229829184-229952988	non-template	31	Intron_1.f	--	Yes	--	Yes	--	--	--	Yes	Yes	Yes	--
G4ST01229829781	chr1:229829782-229829812	+	GGGGACCTGGAGGGAGGAGGCTGGGAGCGGG<br>((....))	DISC1	NM_001164556	chr1:229829184-230021613	non-template	31	Intron_1.f	--	Yes	--	Yes	--	--	--	Yes	Yes	Yes	--
G4ST01229829781	chr1:229829782-229829812	+	GGGGACCTGGAGGGAGGAGGCTGGGAGCGGG<br>((....))	DISC1	NM_001164555	chr1:229829184-229952988	non-template	31	Intron_1.f	--	Yes	--	Yes	--	--	--	Yes	Yes	Yes	--
G4ST01229829781	chr1:229829782-229829812	+	GGGGACCTGGAGGGAGGAGGCTGGGAGCGGG<br>((....))	DISC1	NM_001164553	chr1:229829184-229926443	non-template	31	Intron_1.f	--	Yes	--	Yes	--	--	--	Yes	Yes	Yes	--
G4ST01229829781	chr1:229829782-229829812	+	GGGGACCTGGAGGGAGGAGGCTGGGAGCGGG<br>((....))	DISC1	NM_001164552	chr1:229829184-229926443	non-template	31	Intron_1.f	--	Yes	--	Yes	--	--	--	Yes	Yes	Yes	--
G4ST01229829781	chr1:229829782-229829812	+	GGGGACCTGGAGGGAGGAGGCTGGGAGCGGG<br>((....))	DISC1	NM_001164542	chr1:229829184-230161872	non-template	31	Intron_1.f	--	Yes	--	Yes	--	--	--	Yes	Yes	Yes	--
G4ST01229829781	chr1:229829782-229829812	+	GGGGACCTGGAGGGAGGAGGCTGGGAGCGGG<br>((....))	DISC1	NM_001164549	chr1:229829184-229974031	non-template	31	Intron_1.f	--	Yes	--	Yes	--	--	--	Yes	Yes	Yes	--
G4ST01229829781	chr1:229829782-229829812	+	GGGGACCTGGAGGGAGGAGGCTGGGAGCGGG<br>((....))	DISC1	NM_001012957	chr1:229829184-230243641	non-template	31	Intron_1.f	--	Yes	--	Yes	--	--	--	Yes	Yes	Yes	--
G4ST01229829781	chr1:229829782-229829812	+	GGGGACCTGGAGGGAGGAGGCTGGGAGCGGG<br>((....))	DISC1	NM_001012959	chr1:229829184-230069075	non-template	31	Intron_1.f	--	Yes	--	Yes	--	--	--	Yes	Yes	Yes	--
G4ST01229829781	chr1:229829782-229829812	+	GGGGACCTGGAGGGAGGAGGCTGGGAGCGGG<br>((....))	DISC1	NM_001012958	chr1:229829184-229926443	non-template	31	Intron_1.f	--	Yes	--	Yes	--	--	--	Yes	Yes	Yes	--
G4ST01229844611	chr1:229844612-229844647	-	GGGCGGGAACCGGGGGCTGCGCGCGGTGCTTGCGGG<br>.((.(((.....)))..))	DISC1	NM_001164545	chr1:229829184-230021613	template	36	Intron_1.f	--	Yes	--	Yes	--	--	--	--	--	--	--
G4ST01229844611	chr1:229844612-229844647	-	GGGCGGGAACCGGGGGCTGCGCGCGGTGCTTGCGGG<br>.((.(((.....)))..))	TSNAX-DISC1	NR_028396	chr1:229731022-230021613	template	36	Intron_5	--	Yes	--	Yes	--	--	--	--	--	--	--
G4ST01229844611	chr1:229844612-229844647	-	GGGCGGGAACCGGGGGCTGCGCGCGGTGCTTGCGGG<br>.((.(((.....)))..))	TSNAX-DISC1	NR_028398	chr1:229731022-230021613	template	36	Intron_3	--	Yes	--	Yes	--	--	--	--	--	--	--
G4ST01229844611	chr1:229844612-229844647	-	GGGCGGGAACCGGGGGCTGCGCGCGGTGCTTGCGGG<br>.((.(((.....)))..))	TSNAX-DISC1	NR_028397	chr1:229731022-230021613	template	36	Intron_5	--	Yes	--	Yes	--	--	--	--	--	--	--
G4ST01229844611	chr1:229844612-229844647	-	GGGCGGGAACCGGGGGCTGCGCGCGGTGCTTGCGGG<br>.((.(((.....)))..))	TSNAX-DISC1	NR_028399	chr1:229731022-229904930	template	36	Intron_7	--	Yes	--	Yes	--	--	--	--	--	--	--
G4ST01229844611	chr1:229844612-229844647	-	GGGCGGGAACCGGGGGCTGCGCGCGGTGCTTGCGGG<br>.((.(((.....)))..))	DISC1	NM_001164538	chr1:229829184-230212023	template	36	Intron_1.f	--	Yes	--	Yes	--	--	--	--	--	--	--
G4ST01229844611	chr1:229844612-229844647	-	GGGCGGGAACCGGGGGCTGCGCGCGGTGCTTGCGGG<br>.((.(((.....)))..))	DISC1	NM_001164548	chr1:229829184-230021613	template	36	Intron_1.f	--	Yes	--	Yes	--	--	--	--	--	--	--
G4ST01229844611	chr1:229844612-229844647	-	GGGCGGGAACCGGGGGCTGCGCGCGGTGCTTGCGGG<br>.((.(((.....)))..))	TSNAX-DISC1	NR_028394	chr1:229731022-230021613	template	36	Intron_6	--	Yes	--	Yes	--	--	--	--	--	--	--
G4ST01229844611	chr1:229844612-229844647	-	GGGCGGGAACCGGGGGCTGCGCGCGGTGCTTGCGGG<br>.((.(((.....)))..))	TSNAX-DISC1	NR_028395	chr1:229731022-230021613	template	36	Intron_6	--	Yes	--	Yes	--	--	--	--	--	--	--
G4ST01229844611	chr1:229844612-229844647	-	GGGCGGGAACCGGGGGCTGCGCGCGGTGCTTGCGGG<br>.((.(((.....)))..))	DISC1	NM_018662	chr1:229829184-230243641	template	36	Intron_1.f	--	Yes	--	Yes	--	--	--	--	--	--	--
G4ST01229844611	chr1:229844612-229844647	-	GGGCGGGAACCGGGGGCTGCGCGCGGTGCTTGCGGG<br>.((.(((.....)))..))	TSNAX-DISC1	NR_028400	chr1:229731022-229952988	template	36	Intron_6	--	Yes	--	Yes	--	--	--	--	--	--	--
G4ST01229844611	chr1:229844612-229844647	-	GGGCGGGAACCGGGGGCTGCGCGCGGTGCTTGCGGG<br>.((.(((.....)))..))	TSNAX-DISC1	NR_028393	chr1:229731022-230243641	template	36	Intron_5	--	Yes	--	Yes	--	--	--	--	--	--	--
G4ST01229844611	chr1:229844612-229844647	-	GGGCGGGAACCGGGGGCTGCGCGCGGTGCTTGCGGG<br>.((.(((.....)))..))	DISC1	NM_001164541	chr1:229829184-230212023	template	36	Intron_1.f	--	Yes	--	Yes	--	--	--	--	--	--	--
G4ST01229844611	chr1:229844612-229844647	-	GGGCGGGAACCGGGGGCTGCGCGCGGTGCTTGCGGG<br>.((.(((.....)))..))	DISC1	NM_001164539	chr1:229829184-230057748	template	36	Intron_1.f	--	Yes	--	Yes	--	--	--	--	--	--	--
G4ST01229844611	chr1:229844612-229844647	-	GGGCGGGAACCGGGGGCTGCGCGCGGTGCTTGCGGG<br>.((.(((.....)))..))	DISC1	NM_001164550	chr1:229829184-229926443	template	36	Intron_1.f	--	Yes	--	Yes	--	--	--	--	--	--	--
G4ST01229844611	chr1:229844612-229844647	-	GGGCGGGAACCGGGGGCTGCGCGCGGTGCTTGCGGG<br>.((.(((.....)))..))	DISC1	NM_001164547	chr1:229829184-230212023	template	36	Intron_1.f	--	Yes	--	Yes	--	--	--	--	--	--	--
G4ST01229844611	chr1:229844612-229844647	-	GGGCGGGAACCGGGGGCTGCGCGCGGTGCTTGCGGG<br>.((.(((.....)))..))	DISC1	NM_001164546	chr1:229829184-230161872	template	36	Intron_1.f	--	Yes	--	Yes	--	--	--	--	--	--	--
G4ST01229844611	chr1:229844612-229844647	-	GGGCGGGAACCGGGGGCTGCGCGCGGTGCTTGCGGG<br>.((.(((.....)))..))	DISC1	NM_001164544	chr1:229829184-230021613	template	36	Intron_1.f	--	Yes	--	Yes	--	--	--	--	--	--	--
G4ST01229844611	chr1:229844612-229844647	-	GGGCGGGAACCGGGGGCTGCGCGCGGTGCTTGCGGG<br>.((.(((.....)))..))	DISC1	NM_001164554	chr1:229829184-229904930	template	36	Intron_1.f	--	Yes	--	Yes	--	--	--	--	--	--	--

G4ST01229844611	chr1:229844612-229844647	-	GGGCGGGAACCGGGGGCTGCGCGGTGCTTGCGGG<br>.((.(((.....)))..))	DISC1	NM_001164537	chr1:229829184-230243641	template	36	Intron_1.f	--	Yes	--	Yes	--	--	--	--	--	--	--
G4ST01229844611	chr1:229844612-229844647	-	GGGCGGGAACCGGGGGCTGCGCGGTGCTTGCGGG<br>.((.(((.....)))..))	DISC1	NM_001164540	chr1:229829184-230243641	template	36	Intron_1.f	--	Yes	--	Yes	--	--	--	--	--	--	--
G4ST01229844611	chr1:229844612-229844647	-	GGGCGGGAACCGGGGGCTGCGCGGTGCTTGCGGG<br>.((.(((.....)))..))	DISC1	NM_001164551	chr1:229829184-229952988	template	36	Intron_1.f	--	Yes	--	Yes	--	--	--	--	--	--	--
G4ST01229844611	chr1:229844612-229844647	-	GGGCGGGAACCGGGGGCTGCGCGGTGCTTGCGGG<br>.((.(((.....)))..))	DISC1	NM_001164556	chr1:229829184-230021613	template	36	Intron_1.f	--	Yes	--	Yes	--	--	--	--	--	--	--
G4ST01229844611	chr1:229844612-229844647	-	GGGCGGGAACCGGGGGCTGCGCGGTGCTTGCGGG<br>.((.(((.....)))..))	DISC1	NM_001164555	chr1:229829184-229952988	template	36	Intron_1.f	--	Yes	--	Yes	--	--	--	--	--	--	--
G4ST01229844611	chr1:229844612-229844647	-	GGGCGGGAACCGGGGGCTGCGCGGTGCTTGCGGG<br>.((.(((.....)))..))	DISC1	NM_001164553	chr1:229829184-229926443	template	36	Intron_1.f	--	Yes	--	Yes	--	--	--	--	--	--	--
G4ST01229844611	chr1:229844612-229844647	-	GGGCGGGAACCGGGGGCTGCGCGGTGCTTGCGGG<br>.((.(((.....)))..))	DISC1	NM_001164552	chr1:229829184-229926443	template	36	Intron_1.f	--	Yes	--	Yes	--	--	--	--	--	--	--
G4ST01229844611	chr1:229844612-229844647	-	GGGCGGGAACCGGGGGCTGCGCGGTGCTTGCGGG<br>.((.(((.....)))..))	DISC1	NM_001164542	chr1:229829184-230161872	template	36	Intron_1.f	--	Yes	--	Yes	--	--	--	--	--	--	--
G4ST01229844611	chr1:229844612-229844647	-	GGGCGGGAACCGGGGGCTGCGCGGTGCTTGCGGG<br>.((.(((.....)))..))	DISC1	NM_001164549	chr1:229829184-229974031	template	36	Intron_1.f	--	Yes	--	Yes	--	--	--	--	--	--	--
G4ST01229844611	chr1:229844612-229844647	-	GGGCGGGAACCGGGGGCTGCGCGGTGCTTGCGGG<br>.((.(((.....)))..))	DISC1	NM_001012957	chr1:229829184-230243641	template	36	Intron_1.f	--	Yes	--	Yes	--	--	--	--	--	--	--
G4ST01229844611	chr1:229844612-229844647	-	GGGCGGGAACCGGGGGCTGCGCGGTGCTTGCGGG<br>.((.(((.....)))..))	DISC1	NM_001012959	chr1:229829184-230069075	template	36	Intron_1.f	--	Yes	--	Yes	--	--	--	--	--	--	--
G4ST01229844611	chr1:229844612-229844647	-	GGGCGGGAACCGGGGGCTGCGCGGTGCTTGCGGG<br>.((.(((.....)))..))	DISC1	NM_001012958	chr1:229829184-229926443	template	36	Intron_1.f	--	Yes	--	Yes	--	--	--	--	--	--	--
G4ST01229859147	chr1:229859148-229859174	-	GGGAACTATCAGTGGGCAGTGGGTGGG<br>.(((...)))	DISC1	NM_001164545	chr1:229829184-230021613	template	27	Intron_1.f	--	Yes	--	Yes	--	--	--	--	--	--	Yes
G4ST01229859147	chr1:229859148-229859174	-	GGGAACTATCAGTGGGCAGTGGGTGGG<br>.(((...)))	TSNAX-DISC1	NR_028396	chr1:229731022-230021613	template	27	Intron_5	--	Yes	--	Yes	--	--	--	--	--	--	Yes
G4ST01229859147	chr1:229859148-229859174	-	GGGAACTATCAGTGGGCAGTGGGTGGG<br>.(((...)))	TSNAX-DISC1	NR_028398	chr1:229731022-230021613	template	27	Intron_3	--	Yes	--	Yes	--	--	--	--	--	--	Yes
G4ST01229859147	chr1:229859148-229859174	-	GGGAACTATCAGTGGGCAGTGGGTGGG<br>.(((...)))	TSNAX-DISC1	NR_028397	chr1:229731022-230021613	template	27	Intron_5	--	Yes	--	Yes	--	--	--	--	--	--	Yes
G4ST01229859147	chr1:229859148-229859174	-	GGGAACTATCAGTGGGCAGTGGGTGGG<br>.(((...)))	TSNAX-DISC1	NR_028399	chr1:229731022-229904930	template	27	Intron_7	--	Yes	--	Yes	--	--	--	--	--	--	Yes
G4ST01229859147	chr1:229859148-229859174	-	GGGAACTATCAGTGGGCAGTGGGTGGG<br>.(((...)))	DISC1	NM_001164538	chr1:229829184-230212023	template	27	Intron_1.f	--	Yes	--	Yes	--	--	--	--	--	--	Yes
G4ST01229859147	chr1:229859148-229859174	-	GGGAACTATCAGTGGGCAGTGGGTGGG<br>.(((...)))	DISC1	NM_001164548	chr1:229829184-230021613	template	27	Intron_1.f	--	Yes	--	Yes	--	--	--	--	--	--	Yes
G4ST01229859147	chr1:229859148-229859174	-	GGGAACTATCAGTGGGCAGTGGGTGGG<br>.(((...)))	TSNAX-DISC1	NR_028394	chr1:229731022-230021613	template	27	Intron_6	--	Yes	--	Yes	--	--	--	--	--	--	Yes
G4ST01229859147	chr1:229859148-229859174	-	GGGAACTATCAGTGGGCAGTGGGTGGG<br>.(((...)))	TSNAX-DISC1	NR_028395	chr1:229731022-230021613	template	27	Intron_6	--	Yes	--	Yes	--	--	--	--	--	--	Yes
G4ST01229859147	chr1:229859148-229859174	-	GGGAACTATCAGTGGGCAGTGGGTGGG<br>.(((...)))	DISC1	NM_018662	chr1:229829184-230243641	template	27	Intron_1.f	--	Yes	--	Yes	--	--	--	--	--	--	Yes
G4ST01229859147	chr1:229859148-229859174	-	GGGAACTATCAGTGGGCAGTGGGTGGG<br>.(((...)))	TSNAX-DISC1	NR_028400	chr1:229731022-229952988	template	27	Intron_6	--	Yes	--	Yes	--	--	--	--	--	--	Yes
G4ST01229859147	chr1:229859148-229859174	-	GGGAACTATCAGTGGGCAGTGGGTGGG<br>.(((...)))	TSNAX-DISC1	NR_028393	chr1:229731022-230243641	template	27	Intron_5	--	Yes	--	Yes	--	--	--	--	--	--	Yes
G4ST01229859147	chr1:229859148-229859174	-	GGGAACTATCAGTGGGCAGTGGGTGGG<br>.(((...)))	DISC1	NM_001164541	chr1:229829184-230212023	template	27	Intron_1.f	--	Yes	--	Yes	--	--	--	--	--	--	Yes
G4ST01229859147	chr1:229859148-229859174	-	GGGAACTATCAGTGGGCAGTGGGTGGG<br>.(((...)))	DISC1	NM_001164539	chr1:229829184-230057748	template	27	Intron_1.f	--	Yes	--	Yes	--	--	--	--	--	--	Yes
G4ST01229859147	chr1:229859148-229859174	-	GGGAACTATCAGTGGGCAGTGGGTGGG<br>.(((...)))	DISC1	NM_001164550	chr1:229829184-229926443	template	27	Intron_1.f	--	Yes	--	Yes	--	--	--	--	--	--	Yes
G4ST01229859147	chr1:229859148-229859174	-	GGGAACTATCAGTGGGCAGTGGGTGGG<br>.(((...)))	DISC1	NM_001164547	chr1:229829184-230212023	template	27	Intron_1.f	--	Yes	--	Yes	--	--	--	--	--	--	Yes
G4ST01229859147	chr1:229859148-229859174	-	GGGAACTATCAGTGGGCAGTGGGTGGG<br>.(((...)))	DISC1	NM_001164546	chr1:229829184-230161872	template	27	Intron_1.f	--	Yes	--	Yes	--	--	--	--	--	--	Yes
G4ST01229859147	chr1:229859148-229859174	-	GGGAACTATCAGTGGGCAGTGGGTGGG<br>.(((...)))	DISC1	NM_001164544	chr1:229829184-230021613	template	27	Intron_1.f	--	Yes	--	Yes	--	--	--	--	--	--	Yes
G4ST01229859147	chr1:229859148-229859174	-	GGGAACTATCAGTGGGCAGTGGGTGGG<br>.(((...)))	DISC1	NM_001164554	chr1:229829184-229904930	template	27	Intron_1.f	--	Yes	--	Yes	--	--	--	--	--	--	Yes
G4ST01229859147	chr1:229859148-229859174	-	GGGAACTATCAGTGGGCAGTGGGTGGG<br>.(((...)))	DISC1	NM_001164537	chr1:229829184-230243641	template	27	Intron_1.f	--	Yes	--	Yes	--	--	--	--	--	--	Yes
G4ST01229859147	chr1:229859148-229859174	-	GGGAACTATCAGTGGGCAGTGGGTGGG<br>.(((...)))	DISC1	NM_001164540	chr1:229829184-230243641	template	27	Intron_1.f	--	Yes	--	Yes	--	--	--	--	--	--	Yes
G4ST01229859147	chr1:229859148-229859174	-	GGGAACTATCAGTGGGCAGTGGGTGGG<br>.(((...)))	DISC1	NM_001164551	chr1:229829184-229952988	template	27	Intron_1.f	--	Yes	--	Yes	--	--	--	--	--	--	Yes
G4ST01229859147	chr1:229859148-229859174	-	GGGAACTATCAGTGGGCAGTGGGTGGG<br>.(((...)))	DISC1	NM_001164556	chr1:229829184-230021613	template	27	Intron_1.f	--	Yes	--	Yes	--	--	--	--	--	--	Yes
G4ST01229859147	chr1:229859148-229859174	-	GGGAACTATCAGTGGGCAGTGGGTGGG<br>.(((...)))	DISC1	NM_001164555	chr1:229829184-229952988	template	27	Intron_1.f	--	Yes	--	Yes	--	--	--	--	--	--	Yes
G4ST01229859147	chr1:229859148-229859174	-	GGGAACTATCAGTGGGCAGTGGGTGGG<br>.(((...)))	DISC1	NM_001164553	chr1:229829184-229926443	template	27	Intron_1.f	--	Yes	--	Yes	--	--	--	--	--	--	Yes
G4ST01229859147	chr1:229859148-229859174	-	GGGAACTATCAGTGGGCAGTGGGTGGG<br>.(((...)))	DISC1	NM_001164552	chr1:229829184-229926443	template	27	Intron_1.f	--	Yes	--	Yes	--	--	--	--	--	--	Yes
G4ST01229859147	chr1:229859148-229859174	-	GGGAACTATCAGTGGGCAGTGGGTGGG<br>.(((...)))	DISC1	NM_001164542	chr1:229829184-230161872	template	27	Intron_1.f	--	Yes	--	Yes	--	--	--	--	--	--	Yes
G4ST01229859147	chr1:229859148-229859174	-	GGGAACTATCAGTGGGCAGTGGGTGGG<br>.(((...)))	DISC1	NM_001164549	chr1:229829184-229974031	template	27	Intron_1.f	--	Yes	--	Yes	--	--	--	--	--	--	Yes
G4ST01229859147	chr1:229859148-229859174	-	GGGAACTATCAGTGGGCAGTGGGTGGG<br>.(((...)))	DISC1	NM_001012957	chr1:229829184-230243641	template	27	Intron_1.f	--	Yes	--	Yes	--	--	--	--	--	--	Yes
G4ST01229859147	chr1:229859148-229859174	-	GGGAACTATCAGTGGGCAGTGGGTGGG<br>.(((...)))	DISC1	NM_001012959	chr1:229829184-230069075	template	27	Intron_1.f	--	Yes	--	Yes	--	--	--	--	--	--	Yes
G4ST01229859147	chr1:229859148-229859174	-	GGGAACTATCAGTGGGCAGTGGGTGGG<br>.(((...)))	DISC1	NM_001012958	chr1:229829184-229926443	template	27	Intron_1.f	--	Yes	--	Yes	--	--	--	--	--	--	Yes
G4ST01229888900	chr1:229888901-229888937	-	GGGCCGGGCCCAGGGTCCCCATGCTGTGTGCATGGGG<br>....(((((.....)))))	DISC1	NM_001164545	chr1:229829184-230021613	template	37	Intron_1.f	--	Yes	--	Yes	--	--	--	--	--	--	--
G4ST01229888900	chr1:229888901-229888937	-	GGGCCGGGCCCAGGGTCCCCATGCTGTGTGCATGGGG<br>....(((((.....)))))	TSNAX-DISC1	NR_028396	chr1:229731022-230021613	template	37	Intron_5	--	Yes	--	Yes	--	--	--	--	--	--	--
G4ST01229888900	chr1:229888901-229888937	-	GGGCCGGGCCCAGGGTCCCCATGCTGTGTGCATGGGG<br>....(((((.....)))))	TSNAX-DISC1	NR_028398	chr1:229731022-230021613	template	37	Intron_3	--	Yes	--	Yes	--	--	--	--	--	--	--
G4ST01229888900	chr1:229888901-229888937	-	GGGCCGGGCCCAGGGTCCCCATGCTGTGTGCATGGGG<br>....(((((.....)))))	TSNAX-DISC1	NR_028397	chr1:229731022-230021613	template	37	Intron_5	--	Yes	--	Yes	--	--	--	--	--	--	--
G4ST01229888900	chr1:229888901-229888937	-	GGGCCGGGCCCAGGGTCCCCATGCTGTGTGCATGGGG<br>....(((((.....)))))	TSNAX-DISC1	NR_028399	chr1:229731022-229904930	template	37	Intron_7	--	Yes	--	Yes	--	--	--	--	--	--	--
G4ST01229888900	chr1:229888901-229888937	-	GGGCCGGGCCCAGGGTCCCCATGCTGTGTGCATGGGG<br>....(((((.....)))))	DISC1	NM_001164538	chr1:229829184-230212023	template	37	Intron_1.f	--	Yes	--	Yes	--	--	--	--	--	--	--
G4ST01229888900	chr1:229888901-229888937	-	GGGCCGGGCCCAGGGTCCCCATGCTGTGTGCATGGGG<br>....(((((.....)))))	DISC1	NM_001164548	chr1:229829184-230021613	template	37	Intron_1.f	--	Yes	--	Yes	--	--	--	--	--	--	--

G4ST01229888900	chr1:229888901-229888937	-	GGGCCGGGCCCAGGGTCCCCATGCTGTGTGCATGGGG<br>....(((((.....)))))	TSNAX-DISC1	NR_028394	chr1:229731022-230021613	template	37	Intron_6	--	Yes	--	Yes	--	--	--	--	--	--	--
G4ST01229888900	chr1:229888901-229888937	-	GGGCCGGGCCCAGGGTCCCCATGCTGTGTGCATGGGG<br>....(((((.....)))))	TSNAX-DISC1	NR_028395	chr1:229731022-230021613	template	37	Intron_6	--	Yes	--	Yes	--	--	--	--	--	--	--
G4ST01229888900	chr1:229888901-229888937	-	GGGCCGGGCCCAGGGTCCCCATGCTGTGTGCATGGGG<br>....(((((.....)))))	DISC1	NM_018662	chr1:229829184-230243641	template	37	Intron_1.f	--	Yes	--	Yes	--	--	--	--	--	--	--
G4ST01229888900	chr1:229888901-229888937	-	GGGCCGGGCCCAGGGTCCCCATGCTGTGTGCATGGGG<br>....(((((.....)))))	TSNAX-DISC1	NR_028400	chr1:229731022-229952988	template	37	Intron_6	--	Yes	--	Yes	--	--	--	--	--	--	--
G4ST01229888900	chr1:229888901-229888937	-	GGGCCGGGCCCAGGGTCCCCATGCTGTGTGCATGGGG<br>....(((((.....)))))	TSNAX-DISC1	NR_028393	chr1:229731022-230243641	template	37	Intron_5	--	Yes	--	Yes	--	--	--	--	--	--	--
G4ST01229888900	chr1:229888901-229888937	-	GGGCCGGGCCCAGGGTCCCCATGCTGTGTGCATGGGG<br>....(((((.....)))))	DISC1	NM_001164541	chr1:229829184-230212023	template	37	Intron_1.f	--	Yes	--	Yes	--	--	--	--	--	--	--
G4ST01229888900	chr1:229888901-229888937	-	GGGCCGGGCCCAGGGTCCCCATGCTGTGTGCATGGGG<br>....(((((.....)))))	DISC1	NM_001164539	chr1:229829184-230057748	template	37	Intron_1.f	--	Yes	--	Yes	--	--	--	--	--	--	--
G4ST01229888900	chr1:229888901-229888937	-	GGGCCGGGCCCAGGGTCCCCATGCTGTGTGCATGGGG<br>....(((((.....)))))	DISC1	NM_001164550	chr1:229829184-229926443	template	37	Intron_1.f	--	Yes	--	Yes	--	--	--	--	--	--	--
G4ST01229888900	chr1:229888901-229888937	-	GGGCCGGGCCCAGGGTCCCCATGCTGTGTGCATGGGG<br>....(((((.....)))))	DISC1	NM_001164547	chr1:229829184-230212023	template	37	Intron_1.f	--	Yes	--	Yes	--	--	--	--	--	--	--
G4ST01229888900	chr1:229888901-229888937	-	GGGCCGGGCCCAGGGTCCCCATGCTGTGTGCATGGGG<br>....(((((.....)))))	DISC1	NM_001164546	chr1:229829184-230161872	template	37	Intron_1.f	--	Yes	--	Yes	--	--	--	--	--	--	--
G4ST01229888900	chr1:229888901-229888937	-	GGGCCGGGCCCAGGGTCCCCATGCTGTGTGCATGGGG<br>....(((((.....)))))	DISC1	NM_001164544	chr1:229829184-230021613	template	37	Intron_1.f	--	Yes	--	Yes	--	--	--	--	--	--	--
G4ST01229888900	chr1:229888901-229888937	-	GGGCCGGGCCCAGGGTCCCCATGCTGTGTGCATGGGG<br>....(((((.....)))))	DISC1	NM_001164554	chr1:229829184-229904930	template	37	Intron_1.f	--	Yes	--	Yes	--	--	--	--	--	--	--
G4ST01229888900	chr1:229888901-229888937	-	GGGCCGGGCCCAGGGTCCCCATGCTGTGTGCATGGGG<br>....(((((.....)))))	DISC1	NM_001164537	chr1:229829184-230243641	template	37	Intron_1.f	--	Yes	--	Yes	--	--	--	--	--	--	--
G4ST01229888900	chr1:229888901-229888937	-	GGGCCGGGCCCAGGGTCCCCATGCTGTGTGCATGGGG<br>....(((((.....)))))	DISC1	NM_001164540	chr1:229829184-230243641	template	37	Intron_1.f	--	Yes	--	Yes	--	--	--	--	--	--	--
G4ST01229888900	chr1:229888901-229888937	-	GGGCCGGGCCCAGGGTCCCCATGCTGTGTGCATGGGG<br>....(((((.....)))))	DISC1	NM_001164551	chr1:229829184-229952988	template	37	Intron_1.f	--	Yes	--	Yes	--	--	--	--	--	--	--
G4ST01229888900	chr1:229888901-229888937	-	GGGCCGGGCCCAGGGTCCCCATGCTGTGTGCATGGGG<br>....(((((.....)))))	DISC1	NM_001164556	chr1:229829184-230021613	template	37	Intron_1.f	--	Yes	--	Yes	--	--	--	--	--	--	--
G4ST01229888900	chr1:229888901-229888937	-	GGGCCGGGCCCAGGGTCCCCATGCTGTGTGCATGGGG<br>....(((((.....)))))	DISC1	NM_001164555	chr1:229829184-229952988	template	37	Intron_1.f	--	Yes	--	Yes	--	--	--	--	--	--	--
G4ST01229888900	chr1:229888901-229888937	-	GGGCCGGGCCCAGGGTCCCCATGCTGTGTGCATGGGG<br>....(((((.....)))))	DISC1	NM_001164553	chr1:229829184-229926443	template	37	Intron_1.f	--	Yes	--	Yes	--	--	--	--	--	--	--
G4ST01229888900	chr1:229888901-229888937	-	GGGCCGGGCCCAGGGTCCCCATGCTGTGTGCATGGGG<br>....(((((.....)))))	DISC1	NM_001164552	chr1:229829184-229926443	template	37	Intron_1.f	--	Yes	--	Yes	--	--	--	--	--	--	--
[truncated: 1,171,536 more chars]
